# Catalytic Deracemization of 1,2-Aminoalcohols through Enantioselective Hydrogen Atom Abstraction

**DOI:** 10.1021/jacs.5c20160

**Published:** 2025-12-23

**Authors:** Daniel J. Davies, Antti S. K. Lahdenperä, Robert J. Phipps

**Affiliations:** Yusuf Hamied Department of Chemistry, 150385 University of Cambridge, Lensfield Road, Cambridge, CB2 1EW, U.K.

## Abstract

Catalytic deracemization appears a superficially simple way to obtain enantioenriched chiral compounds but is deceptively challenging. The popularization of photochemical methods which utilize excited state pathways have permitted breakthroughs, but application to simple functional motifs that constitute common chiral building blocks is still rare. We report the catalytic deracemization of *N*-acyl-1,2-aminoalcohols using a cinchona alkaloid-derived catalyst that operates through enantioselective hydrogen atom abstraction, once oxidized by an excited photocatalyst. In combination with an achiral thiol to return the hydrogen atom, accumulation of the unreactive enantiomer occurs. Our catalyst exhibits extremely high chemoselectivity for abstraction adjacent to alcohols and permits a range of useful functionality to be incorporated into the substrates for deracemization. The method generates versatile small molecule building blocks with very high levels of enantioenrichment and demonstrates the synthetic potential of catalysts able to perform hydrogen atom abstraction in a stereoselective manner.

## Graphical abstract

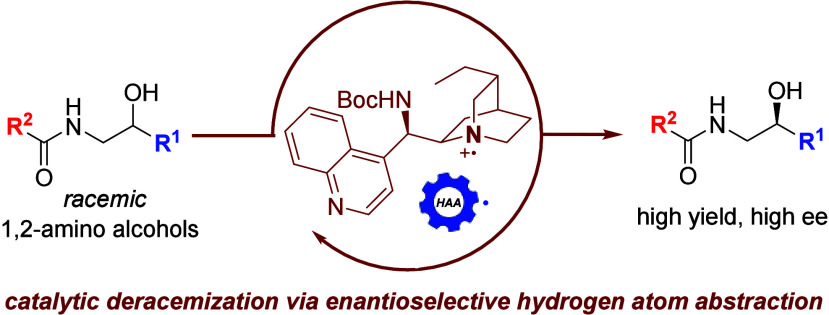

The development of methods to permit the transformation of a racemic mixture into a scalemic one has been a long-standing goal.^1^ Although important breakthroughs have periodically been made, it is particularly noticeable that the last five years or so have seen a step-change in the rate of advancement.^2,3^ A key factor has been the embrace of photocatalytic approaches to asymmetric catalysis for it is in the photochemical realm that excited state reaction pathways, distinct from the associated ground state ones, may provide an opportunity for the thermodynamic challenges of catalytic deracemization to be side-stepped.^2d^ There exist a variety of different mechanistic scenarios in which photochemical deracemization can be achieved and those involving Hydrogen Atom Transfer (HAT) are of particular pertinence when considering stereocenters that bear a hydrogen atom.^4^ Control over the final stereochemical outcome can, in principle, be achieved during either Hydrogen Atom Abstraction (HAA) or Hydrogen Atom Delivery (HAD) involving an appropriate chiral catalyst (Figure 1A). There have been several elegant reports of chiral thiol catalysts being used to enact HAD in deracemization processes in work from Knowles and Miller,^5^ Ye,^6^ and Dong (Figure 1A, upper).^7^ Recent breakthroughs in HAA-driven deracemization, where only one enantiomer of the starting material is reactive toward a chiral open-shell catalyst (Figure 1A, lower), have been achieved by Bach and co-workers using a bifunctional benzophenone catalyst which permits substrate binding through dual hydrogen bonding (Figure 1B).^8,9^ Upon photoexcitation, the catalyst abstracts a hydrogen atom selectively from only one enantiomer of bound substrate. Stereoablation of this reactive enantiomer permits its unreactive antipode to accumulate. Their catalyst has been applied to a number of cyclic substrate classes, a common requirement being a suitable lactam-type motif with which to hydrogen bond with the HAA catalyst. We recently developed a structurally distinct catalyst for enantioselective HAA inspired by MacMillan’s seminal disclosure of quinuclidine as a proficient achiral HAA catalyst.^10,11^ A modified cinchona alkaloid, upon oxidation, could desymmetrize *meso*-diols with very high levels of enantioselectivity through epimerization (in the presence of an achiral thiol), Giese addition or oxidation (Figure 1C, upper).^12^ We envisaged that our catalyst may potentially be able to achieve HAA-driven alcohol deracemization of substrates in which the catalyst can discriminate between the two enantiomers of the starting material. Here we report the successful application to the deracemization of acyclic 1,2-aminoalcohols, forming versatile small molecule chiral building blocks (Figure 1C, lower).^13^

**Figure 1:**
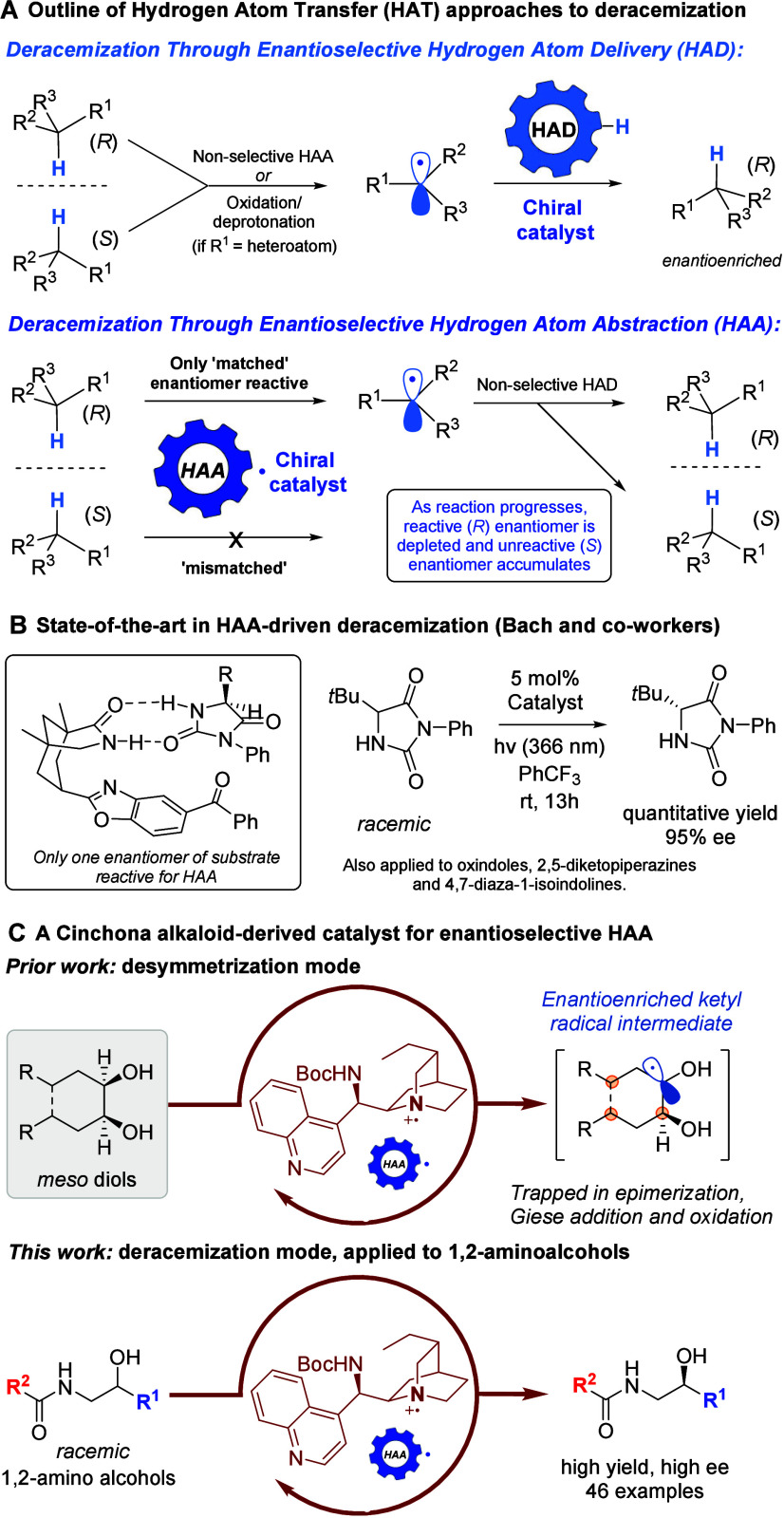
Outline of precedent and summary of this work.

Secondary alcohols were studied in early chemical deracemization approaches using sequential oxidation and reduction processes.^14,15^ Photocatalytic alcohol deracemization can, in principle, avoid the need for stoichiometric oxidant and reductant, but only a handful of reports exist. Zhang and Hu in 2021 developed a deracemization of secondary benzylic alcohols in which dehydrogenation and hydrogenation steps proceed via distinct catalytic pathways, the latter enantiocontrolled.^16^ Zuo and co-workers in 2023 reported a process in which a chiral titanium catalyst controls cleavage of the C–C bond adjacent to the alcohol, as well as its reformation, with the substrate requirement that a stabilized radical must be formed in the cleavage step.^17^ We envisaged that the excellent chemoselectivity demonstrated by our chiral HAA catalyst for alcohols may permit us to address the need for a wider range of secondary alcohol deracemization methods.

We formulated a tentative hypothesis that functionality on the carbon atom adjacent to the secondary alcohol may be needed to engage in productive interaction with the Cinchona alkaloid-derived chiral HAA catalyst. We therefore introduced an acetyl-protected amine with the intention that the NH may act as a hydrogen bond donor but that the neighboring hydrogen atoms should not be liable to abstraction by our catalyst. Using optimized conditions from our previous studies we were pleased to observe that after 24 h very high enantioenrichment of **1a** resulted (Table 1, entry 1). The reaction profile was clean with a high yield of the amino alcohol recovered, suggesting little decomposition of intermediates occurred. The *N*-acetyl group can be readily deprotected (see Supporting Information (SI)). Pleased with this outcome, we nevertheless examined cumulative modifications that might benefit a wider range of substrates, such as increased thiol loading (entry 2), HAA catalyst loading (entry 3) and time (entry 4). These delivered a minor increase in ee to 97% and an isolated yield of 79%.^18^ In parallel we evaluated a slower reacting substrate (**1v**, see later) and found the conditions in entry 4 gave the best outcome, so these were used in the scope exploration (see SI for further details). We omitted various reaction components to confirm their importance at 24h reaction time (compare entry 3). Without monobasic tetra-*n*-butylammonium phosphate, poorer ee was obtained (entry 5). This observation is in line with a role for the phosphate in activating the alcohol to HAA through hydrogen bonding, increasing conversion.^10^ Without the HAA catalyst or photocatalyst, racemic **1a** was obtained (entries 6 and 7). Without thiol, 5% ee was obtained, consistent with some initial reactivity of the HAA catalyst, but no turnover (entry 8). Finally, we evaluated an *N*-methylated derivative of (*rac*)-**1a**, which no longer possesses hydrogen bond donor functionality (entry 9). This gave only 19% ee, providing support for the importance of a hydrogen bond donor to obtain high selectivity, in line with our hypothesis.

**Table 1:**
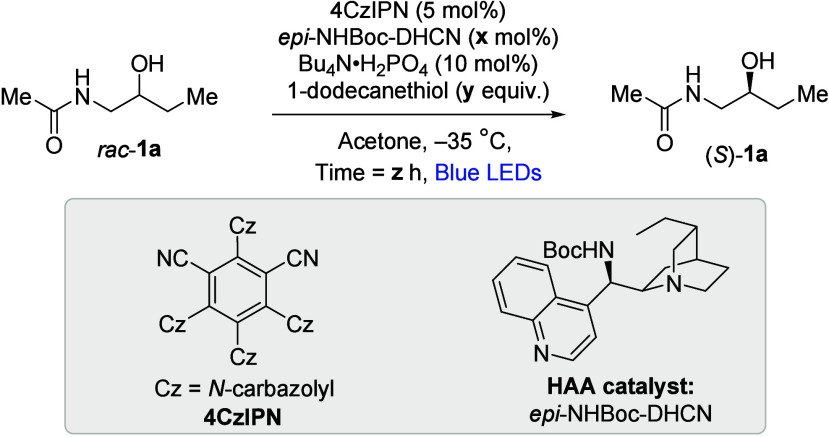
Selected Reaction Optimization and Control

Entry	**x** (mol % HAA catalyst)	**y** (equiv thiol)	**z** (hours)	Yield^a^	ee^b^
1	10	0.25	24	97	95
2	10	1	24	99	94
3	20	1	24	94	96
4^c^	20	1	48	99(79)	97

Entry	With **x** = 20 mol %, **y** = 1 equiv., **z** = 24 h. *Change from above*:	Yield^a^	ee^b^
5	No Bu_4_N·H_2_PO_4_	99	62
6	No HAA catalyst	85	rac
7	No 4CzIPN	82	rac
8	No thiol	93	5
9	Using *N*-methylated derivative of *rac*-**1a**	79 (59)	19

a Yields determined by ^1^H NMR using CH_2_Br_2_ as internal standard.

b ee determined by chiral SFC analysis of the dibenzoate ester of **1a**.

c Yield in parentheses refers to isolated yield.

We evaluated the scope first with respect to the alkyl chain (Scheme 1, blue). The ethyl group could be shortened (**1b**) and lengthened (**1c**) with no detriment. The chain could be terminated with *iso*-propyl (**1d**) and cycloalkyl (**1e**, **1f**) without issue, demonstrating that electron-rich tertiary C–H bonds are tolerated. We incorporated a phenyl group and found that these substrates worked extremely well, regardless of the chain length (**1g**–**1k**), demonstrating tolerance of benzylic positions. A Boc-protected amine (**1l**), ester (**1m**), silyl ether (**1n**), alkyl ether (**1o**), tetrahydropyran (**1p**) and aryl ether (**1q**) were all tolerated with no reduction of enantioselectivity. The latter are significant because the catalyst could feasibly abstract hydridic hydrogen atoms adjacent to the ether. If this does occur it is clearly not to the detriment of the desired process and is likely to be minimal. We next evaluated substrates featuring a substituted benzyl alcohol motif, incorporating electron donating groups (**1r**, **1s**) and halogens (**1t**, **1u**). In the cases of a 1-naphthyl (**1v**) and a 3-thiophene (**1w**), ee was reduced slightly.^19^ We did encounter some substrates that performed poorly; **1x**, which features a *tert-*butyl group close to the site of abstraction gave only 15% ee, potentially due to excessive steric hindrance. If a primary hydroxyl group is included elsewhere in the substrate the yield is significantly reduced (**1y**). In the crude reaction there is evidence of decomposition to a multitude of byproducts, and we envisage that the primary alcohol is reactive to HAA in a nonproductive manner, as supported by deuterium incorporation experiments (see SI). The radical intermediate involved may engage in side-reactions, reducing the yield. We next investigated the variation of the *N*-amide group (Scheme 1, red), including variation to *iso*-propyl (**1z**) and adamantyl (**1za**). Functional groups (nitrile, **1zb**; sulfone, **1zc**) could be included as could a phenyethyl group (**1zd**). Benzamides with various electronic characters could be incorporated (**1ze**–**1zi**), as could heterocyclic amides including furan (**1zj**), imidazole (**1zk**), pyridine (**1zl**), thiazole (**1zm**) and a saturated *N*-heterocycle (**1zn**). Furthermore, urea can be used instead of amide, with three diverse examples shown (**1zo**–**1zq**). Interestingly, an *N*-Boc protected amine gave very poor ee outcome (**1zr**), suggesting that productive interaction with the catalyst may be disrupted in this case. The various functionalities tolerated demonstrate the exceptionally high chemoselectivity of the catalyst, but there are limits. A substrate containing a benzyl ether gave low ee, potentially due to nonproductive HAA at the benzylic methylene, where the C–H bonds have a particularly low BDE of ∼86 kcal mol^–1^ vs 92 for an alcohol (**1zs**).^20^ A thioether-containing substrate (**1zt**) also performed poorly. Finally, we explored examples where both components are varied (**1zu** and **1zv**).

**Scheme 1:**
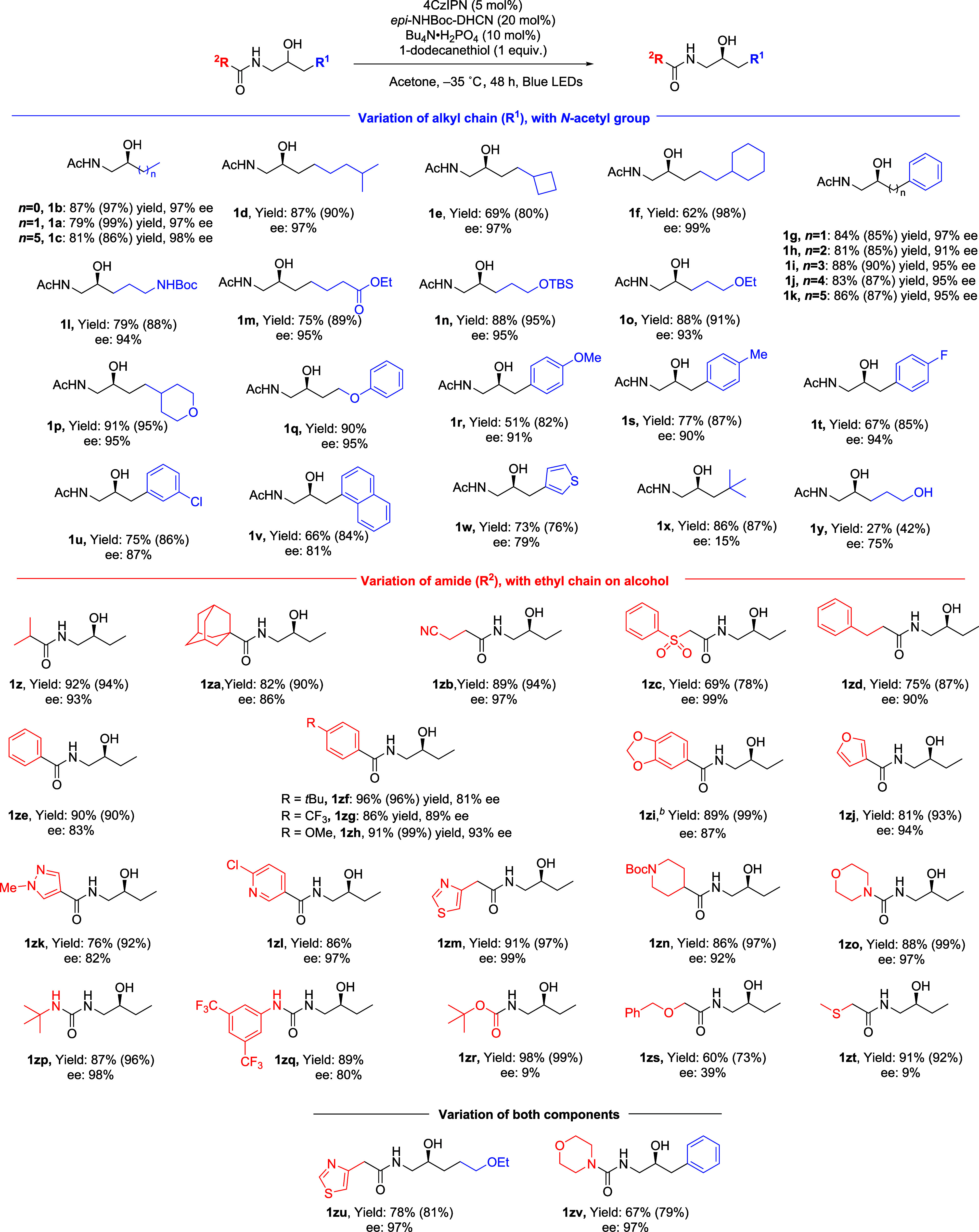
Substrate Scope of Aminoalcohol Deracemization^a^

a Yields refer to isolated product, with those in parentheses determined by NMR with reference to internal standard. %ee values determined by chiral SFC analysis, sometimes after derivatization as dibenzoyl ester (see SI).
b 70 h reaction time.

We were intrigued to evaluate substrates that already contain stereocenters (Scheme 2A). The starting materials for **2a** and **2b** comprised a 1:1 mixture of diastereomers, containing a defined stereocenter from an enantiopure amino acid and a nondefined stereocenter at the alcohol. With **2a** (incorporating l-phenylalanine), a 10:1 d.r. was obtained, in favor of the (*S*,*S*)-product. The corresponding d-phenylalanine-derived **2b** gave >20:1 d.r. of the (*R*,*S*)-product, suggesting that the latter is the perfectly matched case. We also investigated a substrate incorporating racemic ibuprofen (**2c**). Here the starting material is racemic and a 1:1 mixture of diastereomers. After submission to the deracemization reaction, a 1:1 mixture of diastereomers was obtained as expected, with each diastereomer obtained with 97% ee. Thus far, the Cinchonine-derived HAA catalyst *epi*-NHBoc-DHCN has been used. To demonstrate that the opposite product enantiomer can be accessed we evaluated several substrates using the pseudoenantiomeric, Cinchonidine-derived catalyst *epi*-NHBoc-DHCD (Scheme 2B). The ee values remained high, if a little reduced, demonstrating that the pseudoenantiomeric catalysts do not behave identically, but still give excellent outcomes.

**Scheme 2:**
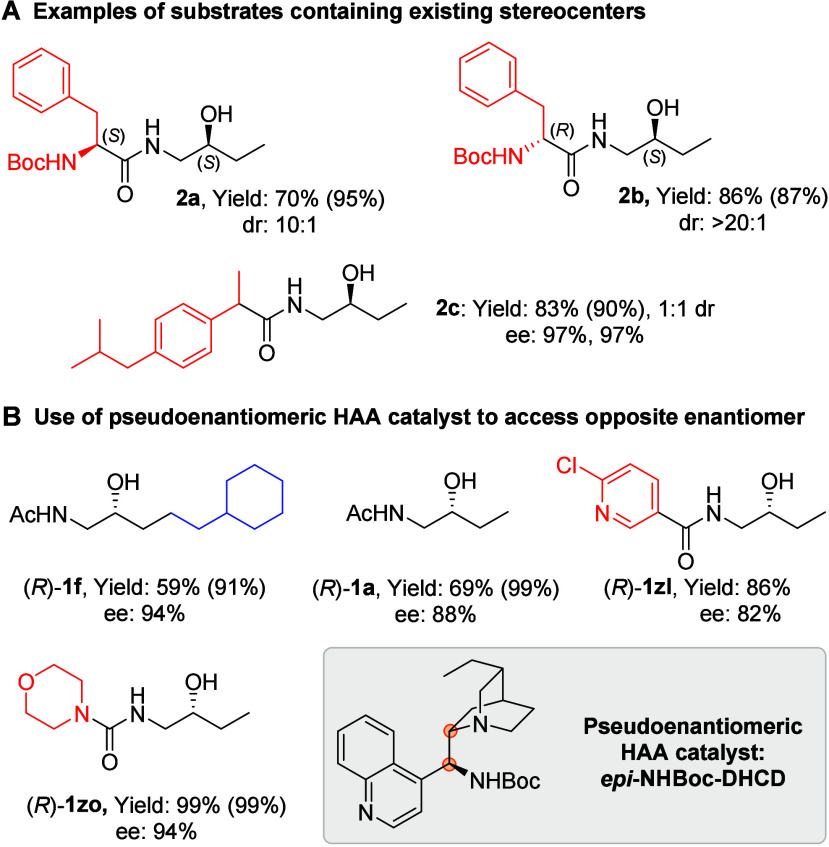
Diastereomeric Substrates and Pseudoenantiomeric Catalyst

We next carried out experiments to probe the hypothesis that one enantiomer of the starting material is unreactive and consequently accumulates, while the other undergoes HAA, followed by nonselective HAD (Figure 1A, lower). Exposing racemic **1b**-*d*, 96% deuterated, to the standard conditions resulted in 92% yield of product exhibiting 98% ee but only 42% deuteration (Scheme 3A). This level of deuterium erosion is consistent with our hypothesis – for the reactive enantiomer, hydrogen is introduced in the HAD step from the nondeuterated thiol. Our catalyst permits the trapping of the intermediate radical in other bond forming processes, not just HAD, as we have demonstrated in previous studies (Figure 1C).^12^ We replaced the thiol with DIAD as used previously for desymmetrizing diol oxidation (Scheme 3B).^12b^ This process now operates as a kinetic resolution and we observed unreacted starting material in 37% yield and 97% ee. Crucially, no deuterium loss had occurred in this material, demonstrating it remained untouched by the catalyst. The same was also true when the thiol was replaced by a Giese acceptor (Scheme 3C). Unreacted starting material displayed high ee and no deuterium loss. The Giese product was racemic, as expected given the ablation of stereochemistry in the HAA step and the inability of our catalyst to control enantioselectivity during Giese addition. For racemic cyclopropane-containing **2d**, it is apparent that the cyclopropane opening is far faster than the quenching of the ketyl radical intermediate by the thiol; fragmentation occurs, followed presumably by quenching of the primary radical by the thiol (Scheme 3D). The result is a kinetic resolution proceeding through intermediate fragmentation. Finally, we carried out a time-course study to visualize the complete inversion of (*R*)-**1b** to (*S*)-**1b** (Scheme 3E). The reaction is rapid, with racemic material obtained after 2 h and >90% ee of the (*S*) enantiomer after 10h. Scheme 3F depicts the envisaged mechanism of the transformation with a tentative working model for the interactions that could be involved. We anticipate that the phosphate additive may act as a hydrogen bond acceptor to promote abstraction, as originally proposed by MacMillan and co-workers.^10^

**Scheme 3:**
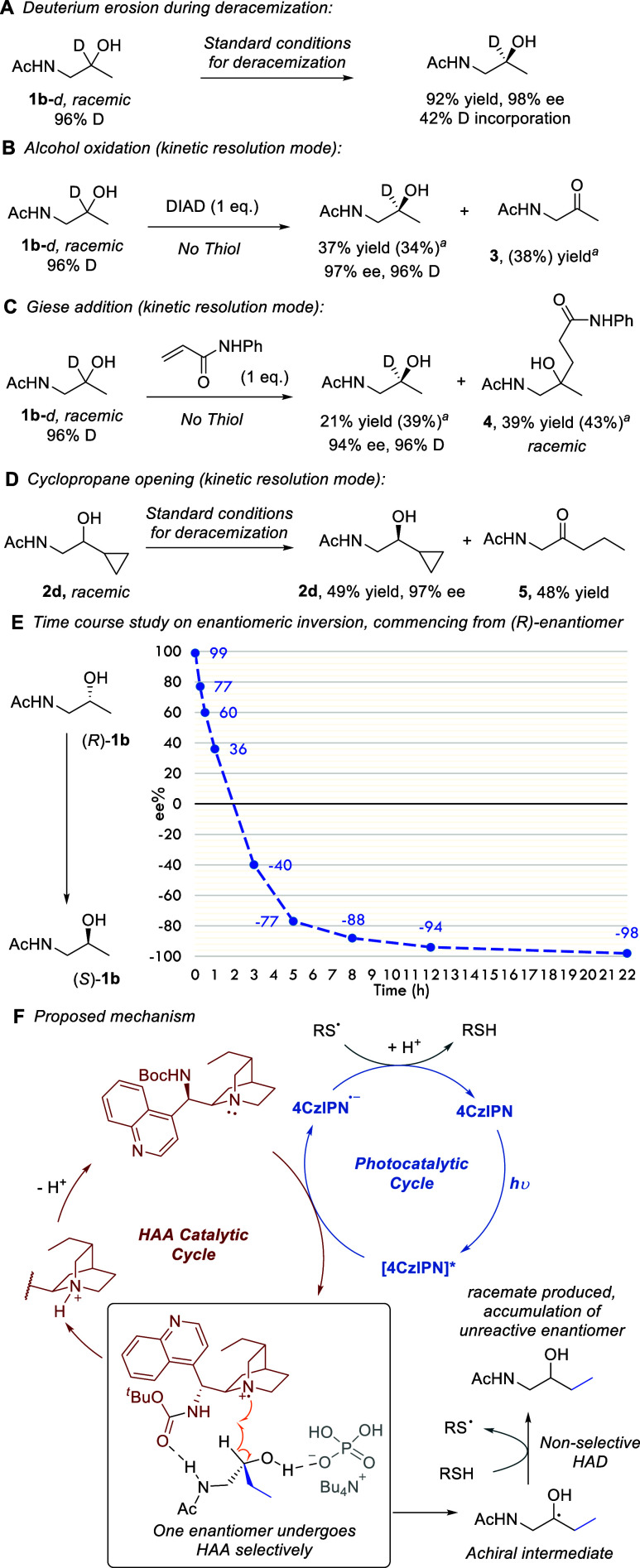
Mechanistic Experiments

a Yields in parentheses determined by NMR with internal standard.

In summary, we demonstrate that a catalyst derived from cinchona alkaloids permits deracemization of 1,2-amino alcohols via enantioselective hydrogen atom abstraction. The high enantioselectivity outcomes reflect excellent differentiation between the two enantiomers of the racemate. Use of an achiral thiol to quench nonselectively permits accumulation of the nonreactive enantiomer of starting material. The reaction is highly chemoselective for alcohols, tolerating much additional functionality in the substrates.

## Supplementary Material



## Notes

Additional reaction optimization, procedures and characterization data (PDF)

## References

[1] Faber K. (2001). Non-Sequential Processes for the Transformation of
a Racemate into a Single Stereoisomeric Product: Proposal for Stereochemical
Classification. Chem.—Eur. J..

[2] Shi Q., Ye J. (2020). Deracemization Enabled by Visible-Light
Photocatalysis. Angew. Chem., Int. Ed..

[3] Hölzl-Hobmeier A., Bauer A., Silva A. V., Huber S. M., Bannwarth C., Bach T. (2018). Catalytic deracemization
of chiral allenes by sensitized excitation with visible light. Nature.

[4] Cao H., Tang X., Tang H., Yuan Y., Wu J. (2021). Photoinduced intermolecular hydrogen
atom transfer reactions in organic
synthesis. Chem. Catal..

[5] Shin N. Y., Ryss J. M., Zhang X., Miller S. J., Knowles R. R. (2019). Light-driven
deracemization enabled by excited-state electron transfer. Science.

[6] Yan X., Pang Y., Zhou Y., Chang R., Ye J. (2025). Photochemical
Deracemization of Lactams with Deuteration Enabled by Dual Hydrogen
Atom Transfer. J. Am. Chem. Soc..

[7] Dai L., Shen C., Wang J., Li Y., Dong K. (2025). Visible Light-Driven
Deracemization of Cyclic Sulfonamides by Quinuclidine and Chiral Arylthiol
Catalysis. Angew. Chem., Int. Ed..

[8] Großkopf J., Plaza M., Seitz A., Breitenlechner S., Storch G., Bach T. (2021). Photochemical Deracemization
at sp3-Hybridized Carbon Centers via a Reversible Hydrogen Atom Transfer. J. Am. Chem. Soc..

[9] Burg F., Bach T. (2019). Lactam Hydrogen Bonds as Control
Elements in Enantioselective Transition-Metal-Catalyzed
and Photochemical Reactions. J. Org. Chem..

[10] Jeffrey J. L., Terrett J. A., MacMillan D. W. C. (2015). O-H
hydrogen bonding promotes H-atom
transfer from α-C-H bonds for C-alkylation of alcohols. Science.

[11] Wang Y., Carder H. M., Wendlandt A. E. (2020). Synthesis
of rare sugar isomers through site-selective epimerization. Nature.

[12] Lahdenperä A.
S. K., Dhankhar J., Davies D. J., Lam N. Y. S., Bacoş P. D., de la Vega-Hernández K., Phipps R. J. (2024). A chiral hydrogen
atom abstraction catalyst for the enantioselective epimerization of
meso-diols. Science.

[13] Ager D. J., Prakash I., Schaad D. R. (1996). 1,2-Amino
Alcohols and Their Heterocyclic Derivatives as Chiral Auxiliaries
in Asymmetric Synthesis. Chem. Rev..

[14] Adair G. R. A., Williams J. M. J. (2005). A novel ruthenium
catalysed deracemisation of alcohols. Chem.
Commun..

[15] Voss C. V., Gruber C. C., Faber K., Knaus T., Macheroux P., Kroutil W. (2008). Orchestration of Concurrent
Oxidation and Reduction Cycles for Stereoinversion and Deracemisation
of sec-Alcohols. J. Am. Chem. Soc..

[16] Zhang Z., Hu X. (2021). Visible-Light-Driven Catalytic Deracemization
of Secondary Alcohols. Angew. Chem., Int. Ed..

[17] Wen L., Ding J., Duan L., Wang S., An Q., Wang H., Zuo Z. (2023). Multiplicative
enhancement of stereoenrichment
by a single catalyst for deracemization of alcohols. Science.

[18] Analysis of the crude reaction mixture for entry 4 showed that 19 mol% of the HAA catalyst remained, suggesting that it is stable to decomposition under the optimized conditions.

[19] For further optimization of **1v**, see the Supporting Information.

[20] Ochiai M., Yamane S., Hoque M. M., Saito M., Miyamoto K. (2012). Metal-free α-CH amination of
ethers with hypervalent sulfonylimino-λ3-bromane that acts as
an active nitrenoid. Chem. Commun..

