# Supplementary material for: Catalytic Deracemization of 1,2-Aminoalcohols through Enantioselective Hydrogen Atom Abstraction
Source: J Am Chem Soc. 2025 Dec 23;148(1):141–7. doi: 10.1021/jacs.5c20160 (PMC12814349; doi:10.1021/jacs.5c20160)

Catalytic Deracemization of 1,2-Aminoalcohols Through Enantioselective Hydrogen Atom Abstraction

Daniel J. Davies, Antti S. K. Lahdenperä and Robert J. Phipps*

Yusuf Hamied Department of Chemistry, University of Cambridge, Lensfield Road, Cambridge, CB2 1EW, UK

*rjp71@cam.ac.uk

Supporting Information

Contents

General Information S2

Additional Optimization Data for Slower Reacting Substrate 1v S4

Substrate Syntheses S5

N-Acetyl Amino Alcohol Substrates S5

General Procedures S5

Characterization Data S7

N-Acyl Amino Alcohol and Urea Substrates S33

General Procedures S33

Characterization Data S34

Deracemization Reactions S48

General Procedures S48

Main Scope Product Characterization S50

Characterization of Products Containing Pre-Existing Stereocenters S88

Opposite Enantiomer Product Characterization S91

Assignment of Absolute Stereochemistry S95

Deacetylation with Retention of Stereochemistry S96

Evaluation of a 1,3-Amino Alcohol S98

D_2O Additive Experiments with Substrate 1y-rac S101

Analysis of HAA Catalyst and Photocatalyst Fate S103

Mechanistic Experiments S104

Scheme 3A - Deuterium erosion during deracemization: S104

Scheme 3C - Giese addition (kinetic resolution mode): S105

Scheme 3B - Alcohol oxidation (kinetic resolution mode): S106

Scheme 3D - Cyclopropane opening (kinetic resolution mode): S107

Scheme 3E - Time course study on enantiomeric inversion, commencing from (R)-enantiomer...... S109

References S111

General Information

Reagents: All reagents were used as supplied from commercial sources without further purification. Acetone used in the deracemization reaction was HPLC grade (≥ 99.8%) and degassed in 100 mL or 250 mL batches by purging with nitrogen for 2 – 3 hours followed by storing under a nitrogen atmosphere.

Reaction setup: Deracemization reactions were carried out in 4 mL, 15 × 45mm crimp-top vials. Reactions were performed in a custom-made cooling setup (Picnic Photo reactor) under a nitrogen atmosphere at –35 °C as detailed in previous work.[1,2] The synthesis of 4CzIPN was conducted as described in the literature.[3] The syntheses of HAA catalysts *epi*-NHBoc-dihydrocinchonine (*epi*-NHBoc-DHCN) and *epi*-NHBoc-dihydrocinchonidine (*epi*-NHBoc-DHCD) were conducted as previously reported.[1]

NMR spectra: ^{1}H NMR spectra were recorded on a 700 MHz Bruker TXO spectrometer, DRX-600 spectrometer, 500 MHz Bruker DCH Cryoprobe, 400 MHz Bruker QNP Cryoprobe or 400 MHz Bruker Avance NEO prodigy N2 Cryoprobe. Chemical shifts are reported in parts per million (ppm) and the spectra are calibrated to the resonance resulting from incomplete deuteration of the solvent ($CDCl_3$: 7.26 ppm, s; $(CD_3)_2CO$: 2.05 ppm, qn; $(CD_3)_2SO$: 2.50 ppm, qn; CD_3OD: 3.31 ppm, qn). ^{13}C NMR spectra were recorded on the same spectrometers with complete proton decoupling. Chemical shifts are reported in ppm with the solvent resonance as the internal standard ($CDCl_3$: 77.16 ppm, t; $(CD_3)_2CO$: 29.84 ppm, sept; $(CD_3)_2SO$: 39.52 ppm, sept; CD_3OD: 49.00 ppm, sept).[4] Data are reported as follows: chemical shift δ/ppm, integration (^{1}H only), multiplicity (s = singlet, d = doublet, t = triplet, q = quartet, quint = quintet, sept = septet, n = nonet, br. = broad, m = multiplet or combinations thereof; ^{13}C signals are singlets unless otherwise stated), coupling constants *J* in Hz. ^{1}H-COSY, HSQC, HMBC and NOESY were used where appropriate to facilitate structural determination.

High Resolution Mass Spectrometry (HRMS): Recorded on a Waters Xevo G2-S bench top QTOF using an electrospray ionization (ESI). Measured values are reported to four decimal places and are within ±5 ppm of the calculated value. The calculated values are based on the most abundant isotope.

Chromatography: Analytical and preparatory thin layer chromatography was performed using precoated Merck glass backed silica gel plates (Silicagel 60 F254). Visualisation was by ultraviolet fluorescence (λ = 254 or 365 nm) and/or staining with potassium permanganate ($KMnO_4$). Flash column chromatography was performed using silica gel 60 (0.040-0.063 µm).

Optical rotations: Measured in spectrophotometric grade $CHCl_3$ or MeOH or HPLC grade EtOH on a Perkin Elmer 43 Polarimeter using a sodium lamp (λ = 589 nm, D-line). α_D values are reported at a given temperature (°C) with concentration in g/100mL.

Chiral HPLC analysis: Performed on a Waters ARC-HPLC system with DAICEL CHIRALPAK IH column (4.6 × 250 mm, 3.0 µm) in a mixed solvent system of *n*-hexane and *i*PrOH.

Chiral SFC analysis: Performed on a Waters ACQUITY UPC2 system with DAICEL CHIRALPAK IA, IC, IE, IJ or IK columns (4.6 x 250 mm, 3 μm) in a mixed solvent system of supercritical CO_2 and MeOH. A system backpressure of 138 bar was used in all cases.

Chiral GC analysis: GC analyses were obtained on a Shimadzu GC-2010 Plus instrument equipped with a CP-Chirasil-Dex CB column (25 m x 0.25 mm ID x 0.25 μm film) for chiral analysis and an FID detector.

Additional Optimization Data for Slower Reacting Substrate 1v

In below table, for entries 1 – 3, enantiomeric excess (ee) values are quoted for samples of (*S*)-**1v** purified by preparative thin layer chromatography (elution with 5% MeOH in DCM). These were subjected to chiral SFC analysis.

For Entry 4, (*S*)-**1v** was purified by silica gel column chromatography (elution with 1% 7N ammonia in MeOH, 2% MeOH, 97% DCM) before subjecting to chiral SFC analysis.

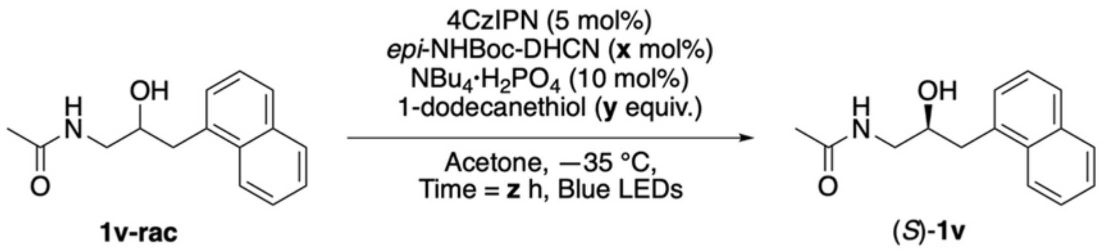

Entry	**x** (mol % HAA catalyst)	**y** (equiv. thiol)	**z** (hours)	Yield[a]	ee[b]
1	10	0.25	24	85	31
2	10	1	24	95	33
3	20	1	24	89	47
4[c]	20	1	48	84 (66)	81

[a]Yields determined by ^{1}H NMR using CH_2Br_2 as internal standard. [b]Enantiomeric excess (ee) determined by chiral SFC analysis. [c]Yield in parenthesis refers to isolated yield of (*S*)-**1v**.

An increase in thiol loading **y** to 1 equiv. resulted in a 10% increase in yield (entry 2 vs entry 1). An increase in HAA catalyst loading **x** to 20 mol% resulted in a 14% increase in ee (entry 3 vs entry 2). An increase in reaction time **z** to 48 h resulted in a further 34% increase in ee (entry 4 vs entry 3).

Despite the conditions in entry 4 resulting in only a minor improvement for faster reacting optimization substrate **1a** (see optimization table in main manuscript), a significant improvement is seen for the slower reacting substrate **1v**. The conditions in Entry 4 were therefore adopted for scope exploration.

Substrate Syntheses

N-Acetyl Amino Alcohol Substrates

General Procedures

A general pathway to *N*-acetylated amino alcohols is depicted below. Either amino alcohol, epoxide, or terminal alkene were employed as starting material depending on commercial availability. Alternatively, these intermediates were synthesized, including *via* **General Procedure A**.

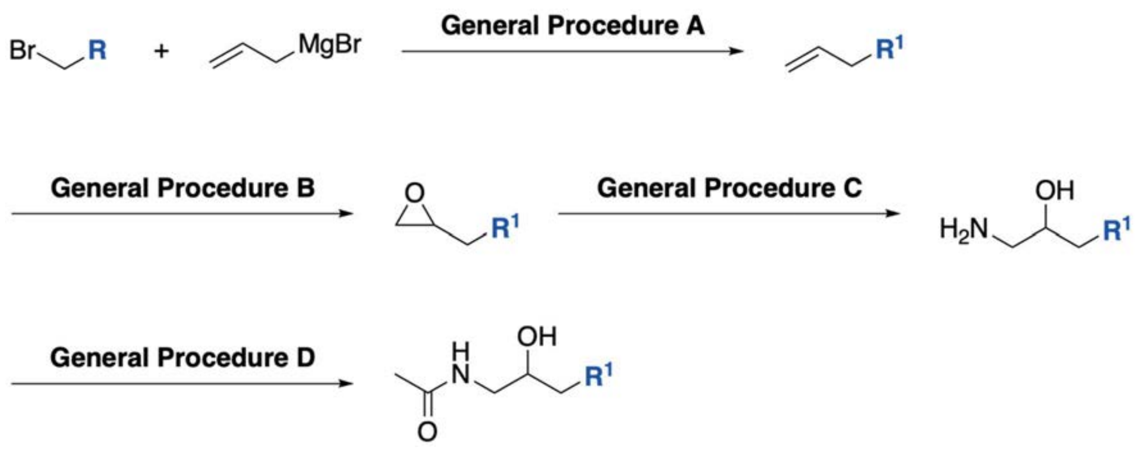

General Procedure A: terminal alkene synthesis *via* allyl Grignard addition into bromoalkanes

Preparation based on a modified literature procedure.[5] A solution of bromoalkane substrate (1.0 eq.) in dry THF (0.66 M) was stirred in an oven-dried two-necked RBF fitted with an oven-dried condenser under nitrogen atmosphere. Allyl Grignard solution (1 M in dry THF, 1.5 eq.) was added slowly at RT. The reaction mixture was then heated to reflux and stirred overnight. The mixture was cooled to 0 °C and quenched with saturated aqueous ammonium chloride solution and extracted with DCM. The combined organic extracts were dried over $MgSO_4$, filtered, and the solvent removed *in vacuo* to afford crude mixture. The crude mixture was either purified by silica gel column chromatography to afford pure terminal alkene product or used without additional purification.

General Procedure B: epoxide synthesis *via* Prilezhaev epoxidation

*m*CPBA (≤ 77%, 1.2 eq.) was added to a stirred solution of the alkene substrate (1.0 eq.) in dry DCM (0.2 M). The reaction mixture was stirred overnight at RT, quenched with saturated aqueous $NaHCO_3$ solution and extracted with DCM. The combined organic extracts were dried over $MgSO_4$, filtered, and the solvent removed *in vacuo* to afford crude mixture. The crude mixture was either purified by silica gel column chromatography to afford pure epoxide product or used without additional purification.

General Procedure C: amino alcohol synthesis *via* epoxide opening

Ethanol and 35% aqueous NH_4OH solution (1:1 v/v) was added to epoxide substrate (1.0 eq.) in a microwave vial. The microwave vial was sealed with a crimp-top cap and the reaction mixture stirred vigorously at 60 °C for 2 – 4 h. The crude material was concentrated *in vacuo* and purified by silica gel column chromatography to afford amino alcohol product.

General Procedure D: *N*-acetyl amino alcohol formation *via* acetyl chloride addition to amino alcohols

Triethylamine (2.0 eq.) was added to a stirred solution of amino alcohol substrate (1.0 eq.) in dry DCM (0.2 M). The solution was cooled to 0 °C and acetyl chloride (1.0 eq.) added slowly. The reaction mixture was stirred for 4 h at 0 °C, concentrated *in vacuo* and purified by silica gel column chromatography to afford acetylated amino alcohol product.

Characterization Data

N-(2-hydroxybutyl)acetamide (1a-rac)

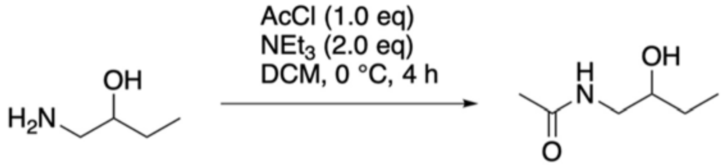

1a-rac

Prepared according to **General Procedure D** using amino alcohol substrate 1-amino-2-butanol (446 mg, 5.00 mmol), triethylamine (1.39 mL, 10.0 mmol) and acetyl chloride (0.36 mL, 5.06 mmol). Purification by silica gel column chromatography (elution with 7% MeOH in DCM) afforded **1a-rac** as a pale yellow oil (587 mg, 4.48 mmol, 90 % yield).

^{1}**H NMR** (700 MHz, $CDCl_3$) δ 6.03 (br. s, 1H), 3.66 – 3.60 (m, 1H), 3.48 (ddd, *J* = 14.0, 6.5, 2.9 Hz, 1H), 3.11 (ddd, *J* = 14.0, 7.9, 5.1 Hz, 1H), 2.72 – 2.64 (m, 1H), 2.01 (s, 3H), 1.54 – 1.44 (m, 2H), 0.96 (t, *J* = 7.5 Hz, 3H) ppm.
^{13}**C NMR** (176 MHz, $CDCl_3$) δ 171.3, 72.9, 45.6, 28.1, 23.4, 10.0 ppm.
HRMS $[M+H]^+$ m/z calc'd for $[C_6H_{13}NO_2H]^+$ expect 132.1025; found 132.1020.

N-(2-hydroxybutyl)-*N*-methylacetamide (*N*-Me-1a-rac)

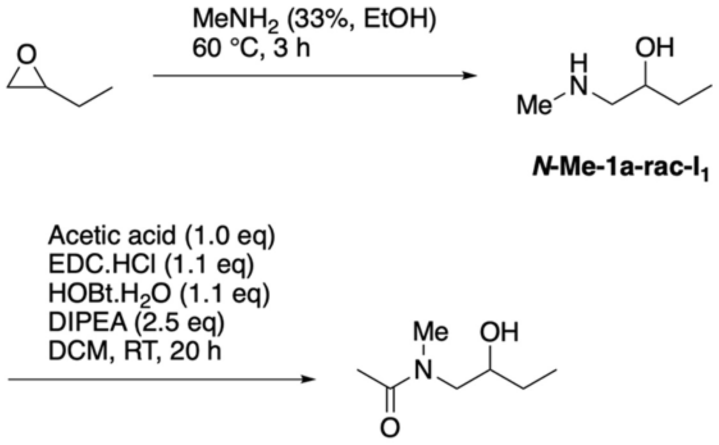

***N*-Me-1a-rac**

Methylamine (33% in EtOH, 15 mL) was added to 1,2-epoxybutane (0.26 mL, 2.99 mmol)) in a microwave vial. The microwave vial was sealed and the reaction mixture stirred vigorously at 60 °C for 3 h. The crude material was concentrated *in vacuo* to afford crude ***N*-Me-1a-rac-I$_1$**. DIPEA (1.31 mL, 7.52 mmol) was added to a stirred suspension of glacial acetic acid (0.17 mL, 2.94 mmol), crude ***N*-Me-1a-rac-I$_1$**, *N*-(3-Dimethylaminopropyl)-*N'*-ethylcarbodiimide hydrochloride (EDC.HCl) (633 mg, 3.30 mmol) and 1-hydroxybenzotriazole monohydrate (HOBt.H_2O) (≥20 wt% H_2O, 632 mg, 3.30 mmol) in DCM (8 mL). The reaction mixture was stirred for 20 h, concentrated *in vacuo*, water was added and the crude extracted with ethyl

acetate. The combined organic extracts were dried over $MgSO_4$, filtered, and the solvent removed *in vacuo* to afford crude mixture. The crude mixture was purified by silica gel column chromatography (elution with 3% MeOH in DCM) to afford ***N*-Me-1a-rac** as a pale yellow oil (198 mg, 1.36 mmol, 45% yield over two steps). Observed as two distinct rotamers (approx. 3:1). Combined signals for both rotamers are denoted *R*1+*R*2. Discernible signals are denoted *R*1 and *R*2.

^{1}H NMR (700 MHz, $CDCl_3$) δ 3.80 – 3.75 (m, 0.25H, *R*1), 3.75 – 3.69 (m, 0.75H, *R*2), 3.60 (dd, *J* = 14.2, 8.4 Hz, 0.75H, *R*2), 3.37 (dd, *J* = 14.0, 9.5 Hz, 0.25H, *R*1), 3.21 (dd, *J* = 14.2, 2.0 Hz, 0.75H, *R*2), 3.19 – 3.14 (m, 0.25H, *R*1), 3.08 (s, 2.25H, *R*2), 2.98 – 2.94 (m, 0.75H, *R*1), 2.70 (br. s, 1H, *R*1+*R*2), 2.16 – 2.13 (m, 0.75H, *R*1), 2.13 – 2.10 (m, 2.25H, *R*2), 1.55 – 1.42 (m, 2H, *R*1+*R*2), 1.01 (t, *J* = 7.4 Hz, 0.75H, *R*1), 0.97 (t, *J* = 7.4 Hz, 2.25H, *R*2) ppm.
^{13}C NMR (176 MHz, $CDCl_3$) δ 173.2 (*R*2), 171.9 (*R*1), 72.8 (*R*2), 70.7 (*R*1), 57.0 (*R*1), 55.2 (*R*2), 55.1 (*R*2), 38.4 (*R*2), 34.2 (*R*1), 28.5 (*R*2), 27.9 (*R*1), 21.9 (*R*2), 21.8 (*R*1), 10.1 (*R*1), 10.0 (*R*2) ppm.
HRMS $[M+H]^+$ m/z calc'd for $[C_7H_{15}NO_2H]^+$ expect 146.1181; found 146.1180.

***N*-(2-hydroxypropyl)acetamide (1b-rac)**

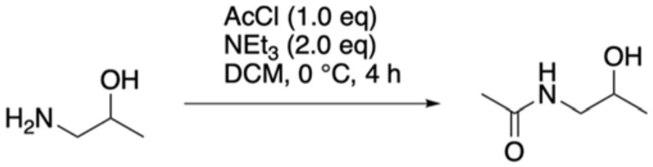

1b-rac

Prepared according to **General Procedure D** using amino alcohol substrate amino-2-propanol (751 mg, 10.0 mmol), triethylamine (2.79 mL, 20.0 mmol) and acetyl chloride (0.71 mL, 9.99 mmol). Purification by silica gel column chromatography (elution with 2.5% 7N ammonia in MeOH, 5% MeOH, 92.5% DCM followed by a second purification eluting with 10% MeOH in DCM) afforded **1b-rac** as a pale yellow oil (956 mg, 8.16 mmol, 82% yield).

^{1}H NMR (700 MHz, $CDCl_3$) δ 6.02 (br. s, 1H), 3.96 – 3.88 (m, 1H), 3.44 (ddd, *J* = 13.9, 6.6, 3.0 Hz, 1H), 3.10 (ddd, *J* = 13.9, 8.0, 5.4 Hz, 1H), 2.71 – 2.64 (m, 1H), 2.01 (s, 3H), 1.19 (d, *J* = 6.3 Hz, 3H) ppm.
^{13}C NMR (176 MHz, $CDCl_3$) δ 171.4, 67.6, 47.3, 23.3, 21.1 ppm.

The data is in good agreement with the literature (for reported (*R*)-enantiomer).[6]

N-(2-hydroxyoctyl)acetamide (1c-rac)

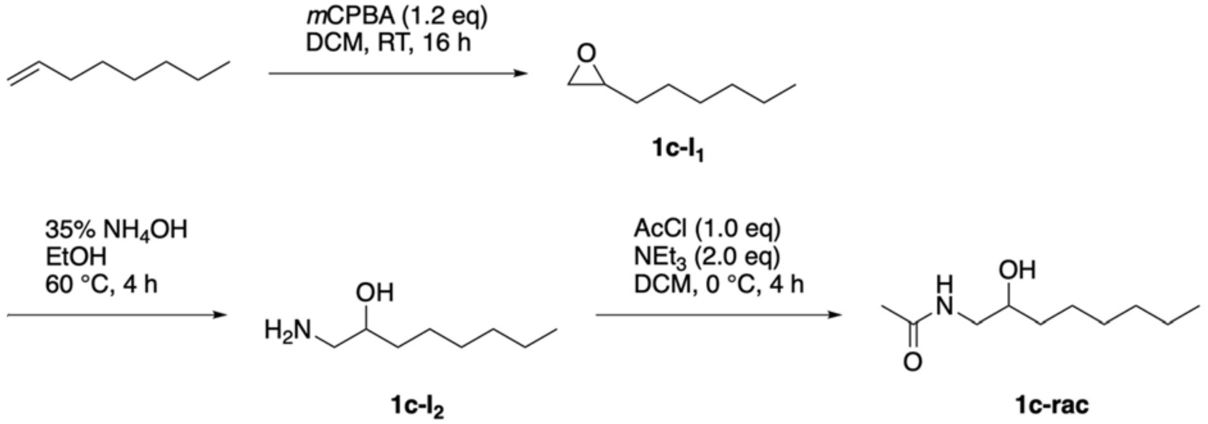

1c-I$_1$ was prepared according to **General Procedure B** using alkene substrate 1-octene (0.94 mL, 5.99 mmol) and *m*CPBA (1.61 g, 7.18 mmol) stirred overnight for 16 h. The crude material was brought forward without additional purification to **General Procedure C** using NH_4OH (11 mL) and ethanol (11 mL) stirred for 4 h. Purification by silica gel column chromatography (elution with 5% 7N ammonia in MeOH, 10% MeOH, 85% DCM) afforded **1c-I$_2$** as a pale yellow oil (622 mg, 4.28 mmol, 71% yield over two steps).

^{1}H NMR (500 MHz, $CDCl_3$) δ 3.54 – 3.46 (m, 1H), 2.83 (dd, *J* = 12.6, 3.2 Hz, 1H), 2.51 (dd, *J* = 12.6, 8.4 Hz, 1H), 2.08 (br. s, 3H), 1.49 – 1.37 (m, 3H), 1.36 – 1.21 (m, 7H), 0.92 – 0.84 (m, 3H) ppm.
^{13}C NMR (126 MHz, $CDCl_3$) δ 72.1, 47.5, 34.9, 31.9, 29.5, 25.8, 22.7, 14.2 ppm.

The data is in good agreement with the literature.[7]

1c-rac was prepared according to **General Procedure D** using amino alcohol substrate **1c-I$_2$** (291 mg, 2.00 mmol), triethylamine (0.56 mL, 4.02 mmol) and acetyl chloride (0.14 mL, 1.97 mmol). Purification by silica gel column chromatography (elution with 1.5% 7N ammonia in MeOH, 3% MeOH, 95.5% DCM) afforded **1c-rac** as a white solid (335 mg, 1.79 mmol, 89% yield).

^{1}H NMR (700 MHz, $CDCl_3$) δ 6.21 (br. s, 1H), 3.72 – 3.65 (m, 1H), 3.46 (ddd, *J* = 13.8, 6.5, 2.9 Hz, 1H), 3.08 (ddd, *J* = 13.8, 8.1, 5.2 Hz, 1H), 2.76 (br. s, 1H), 2.00 (s, 3H), 1.48 – 1.36 (m, 3H), 1.35 – 1.22 (m, 7H), 0.87 (t, *J* = 7.1 Hz, 3H) ppm.
^{13}C NMR (176 MHz, $CDCl_3$) δ 171.4, 71.4, 46.0, 35.2, 31.9, 29.4, 25.6, 23.3, 22.7, 14.2 ppm.
HRMS $[M+H]^+$ m/z calc'd for $[C_{10}H_{21}NO_2H]^+$ expect 188.1651; found 188.1657.

***N*-(2-hydroxy-7-methyloctyl)acetamide (1d-rac)**

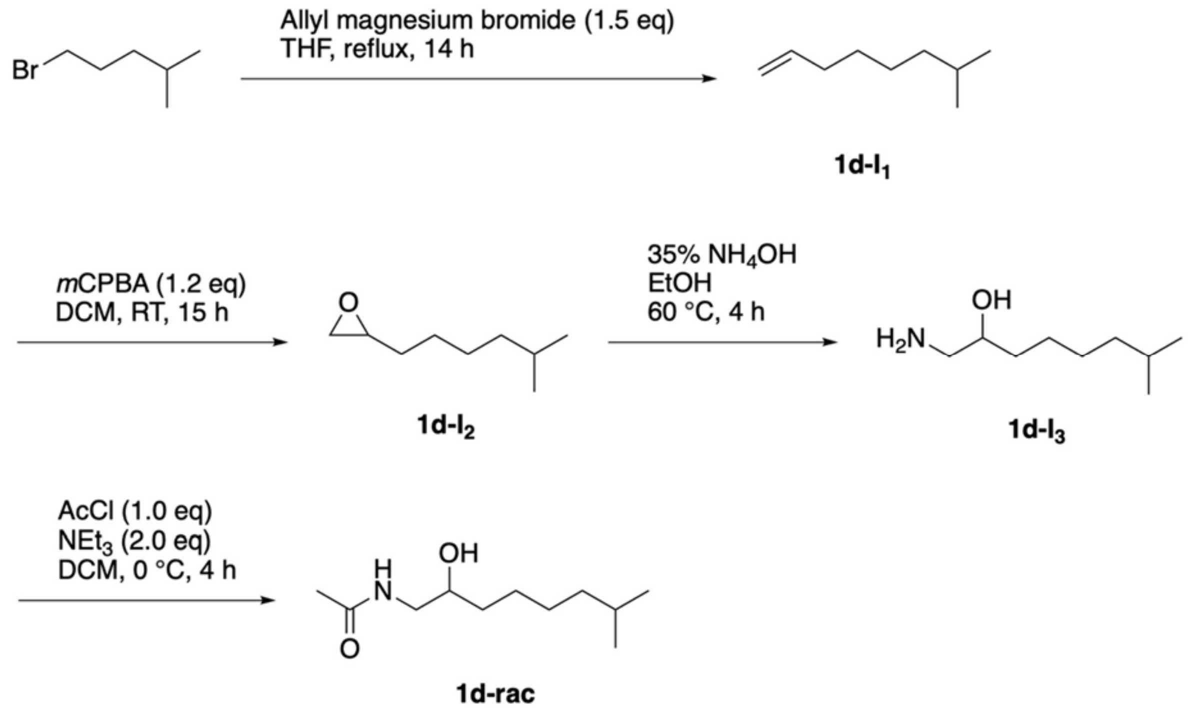

1d-I$_1$ was prepared according to **General Procedure A** using bromoalkane substrate 1-bromo-4-methylpentane (1.09 mL, 7.49 mmol) and allyl magnesium bromide (11.3 mL, 11.3 mmol) stirred overnight for 14 h. The crude material was brought forward without additional purification to **General Procedure B** using *m*CPBA (2.02 g, 9.01 mmol) stirred overnight for 15 h. The crude material was partially purified by silica gel column chromatography (elution with 5% acetone in petroleum ether). Impure **1d-I$_2$** was then brought forward to **General Procedure C** using 35% NH_4OH (11 mL) and ethanol (11 mL) stirred for 4 h. Purification by silica gel column chromatography (elution with 4% 7N ammonia in MeOH, 8% MeOH, 88% DCM) afforded **1d-I$_3$** as a viscous pale yellow oil (105 mg, 0.656 mmol, 9% yield over three steps).

^{1}H NMR (500 MHz, $CDCl_3$) δ 3.54 – 3.46 (m, 1H), 2.84 (app. br. d, *J* = 11.4 Hz, 1H), 2.51 (dd, *J* = 12.3, 8.5 Hz, 1H), 1.90 (br. s, 3H), 1.52 (hep, *J* = 6.6 Hz, 1H), 1.47 – 1.36 (m, 3H), 1.35 – 1.25 (m, 3H), 1.21 – 1.13 (m, 2H), 0.86 (d, *J* = 6.6 Hz, 6H) ppm.
^{13}C NMR (176 MHz, $CDCl_3$) δ 72.2, 47.5, 39.1, 35.0, 28.1, 27.6, 26.1, 22.8 (2C) ppm.
HRMS $[M+H]^+$ m/z calc'd for $[C_9H_{21}NOH]^+$ expect 160.1701; found 160.1694.

1d-rac was prepared according to **General Procedure D** using amino alcohol substrate **1d-I$_3$** (89.5 mg, 0.56 mmol), triethylamine (0.16 mL, 1.15 mmol) and acetyl chloride (0.04 mL, 0.56 mmol). Purification by silica gel column chromatography (elution with 2% 7N ammonia in MeOH, 4% MeOH, 94% DCM) afforded **1d-rac** as a pale yellow oil (85.5 mg, 0.42 mmol, 76% yield).

^{1}H NMR (700 MHz, $CDCl_3$) δ 6.25 – 5.96 (m, 1H), 3.68, (br. s, 1H), 3.50 – 3.43 (m, 1H), 3.12 – 3.05 (m, 1H), 2.92 – 2.59 (m, 1H), 2.01 – 1.99 (m, 3H), 1.55 – 1.47 (m, 1H), 1.47 – 1.35 (m, 3H), 1.34 – 1.23 (m, 3H), 1.19 – 1.12 (m, 2H), 0.87 – 0.83 (m, 6H) ppm.

^{13}C NMR (176 MHz, $CDCl_3$) δ 171.3, 71.5, 46.0, 39.0, 35.2, 28.0, 27.5, 25.9, 23.3, 22.7 (2C) ppm.
HRMS $[M+H]^+$ m/z calc'd for $[C_{11}H_{23}NO_2H]^+$ expect 202.1807; found 202.1809.

***N*-(4-cyclobutyl-2-hydroxybutyl)acetamide (1e-rac)**

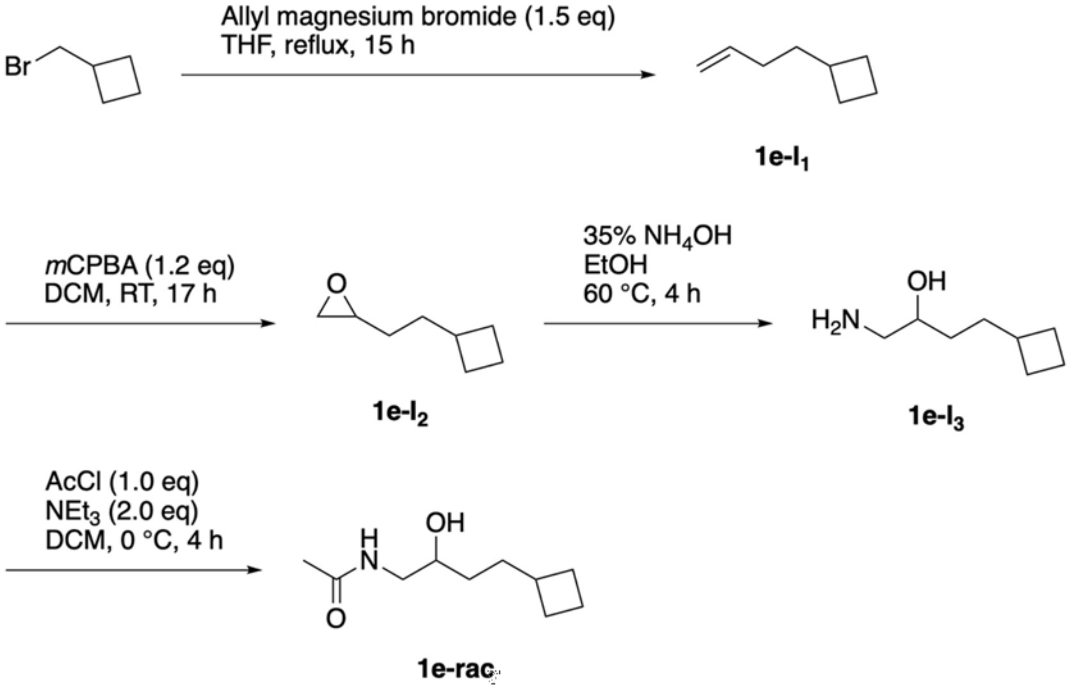

1e-I$_1$ was prepared according to **General Procedure A** using bromoalkane substrate (bromomethyl)cyclobutane (0.84 mL, 7.47 mmol) and allyl magnesium bromide (11.3 mL, 11.3 mmol) stirred overnight for 15 h. The crude material was brought forward without additional purification to **General Procedure B** using *m*CPBA (2.02 g, 9.01 mmol) stirred overnight for 17 h. Crude **Ie-I$_2$** was then brought forward without additional purification to **General Procedure C** using 35% NH_4OH (11 mL) and ethanol (11 mL) stirred for 4 h. The crude material was partially purified by silica gel column chromatography (elution with 5% 7N ammonia in MeOH, 10% MeOH, 85% DCM) to afford impure **1e-I$_3$** (104 mg). Impure **1e-I$_3$** (90 mg) was then brought forward to **General Procedure D** using triethylamine (0.18 mL, 1.29 mmol) and acetyl chloride (0.045 mL, 0.633 mmol). Purification by silica gel column chromatography (elution with 1.5% 7N ammonia in MeOH, 3% MeOH, 95.5% DCM) afforded **1e-rac** as a pale yellow oil (42 mg, approx. 0.23 mmol, approx. 4% corrected yield over four steps accounting for only 90 mg out of 104 mg of impure **1e-I$_3$** being brought forward). **1e-rac** contains unknown impurity, and so approximate yield is given. It is unclear whether this impurity is due to degradation of **1e-rac** during purification by silica gel column chromatography or due to impurity being inseparable from **1e-rac** in the eluent used.

^{1}H NMR (700 MHz, $CDCl_3$) δ 6.17 (br. s, 1H), 3.69 – 3.62 (m, 1H), 3.45 (ddd, *J* = 14.0, 6.5, 2.9 Hz, 1H), 3.07 (ddd, *J* = 14.0, 8.0, 5.1 Hz, 1H), 2.89 (br. s, 1H), 2.22 (app. hept, *J* = 7.7 Hz, 1H), 2.04 – 1.97 (m, 2H), 1.99 (s, 3H), 1.87 – 1.80 (m, 1H), 1.80 – 1.74 (m, 1H), 1.60 – 1.53 (m, 2H), 1.51 – 1.46 (m, 1H), 1.43 – 1.37 (m, 1H), 1.37 – 1.31 (m, 2H) ppm.
^{13}C NMR (176 MHz, $CDCl_3$) δ 171.3, 71.4, 45.9, 36.0, 32.8, 32.7, 28.4, 28.3, 23.3, 18.5 ppm.

HRMS [M+H]$^+$ m/z calc'd for $[C_{10}H_{19}NO_2H]^+$ expect 186.1494; found 186.1491.

***N*-(5-cyclohexyl-2-hydroxypentyl)acetamide (1f-rac)**

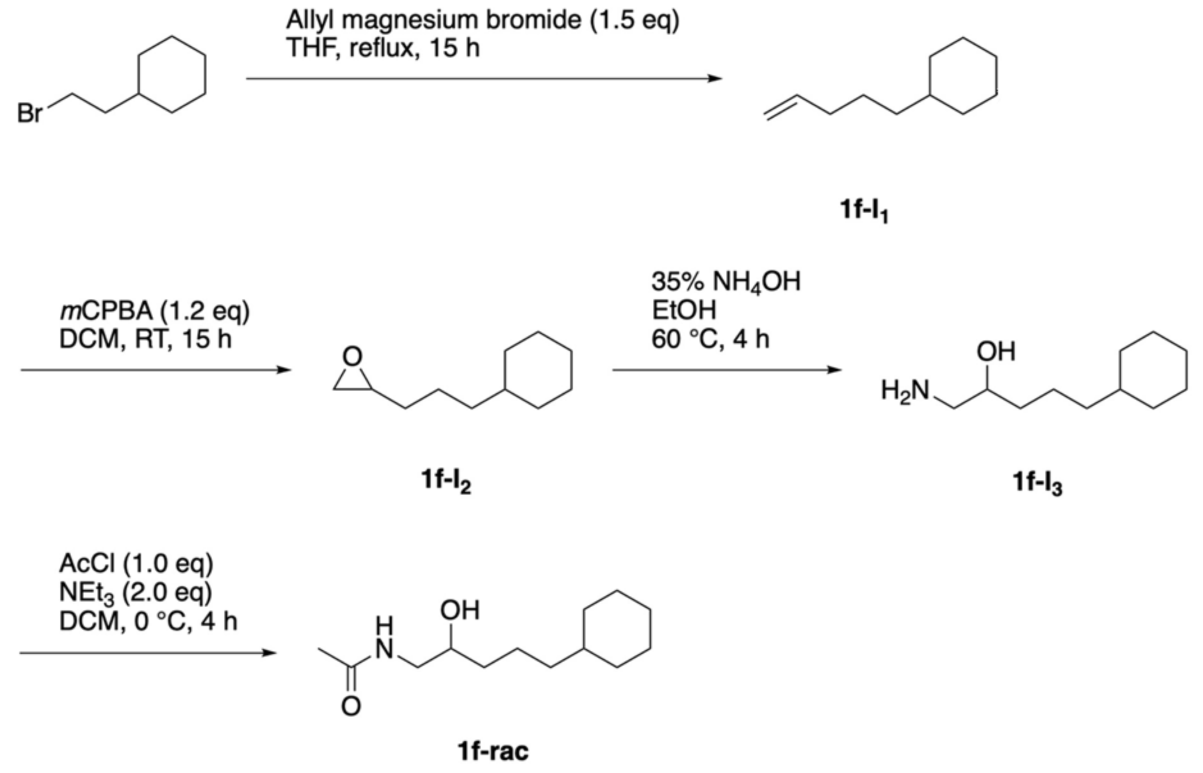

1f-I$_1$ was prepared according to **General Procedure A** using bromoalkane substrate 2-cyclohexylethyl bromide (1.17 mL, 7.48 mmol) and allyl magnesium bromide (11.3 mL, 11.3 mmol) stirred overnight for 15 h. The crude material was brought forward without additional purification to **General Procedure B** using *m*CPBA (2.02 g, 9.01 mmol) stirred overnight for 15 h to afford crude **1f-I$_2$**. Crude **1f-I$_2$** was brought forward without additional purification to **General Procedure C** using 35% NH_4OH (11 mL) and ethanol (11 mL) stirred for 4 h. Partial purification by silica gel column chromatography (elution with 5% 7N ammonia in MeOH, 10% MeOH, 85% DCM) afforded impure **1f-I$_3$**, which was brought forward to **General Procedure D** using triethylamine (0.75 mL, 5.36 mmol) and acetyl chloride (0.19 mL, 2.68 mmol). Purification by silica gel column chromatography (elution with 1.5% 7N ammonia in MeOH, 3% MeOH, 95.5% DCM) afforded **1f-rac** as a pale yellow oil that slowly forms a white solid (358 mg, 1.57 mmol, 21% yield over four steps).

^{1}H NMR (700 MHz, $CDCl_3$) δ 5.89 (br. s, 1H), 3.74 – 3.67 (m, 1H), 3.49 (ddd, *J* = 13.9, 6.6, 2.9 Hz, 1H), 3.10 (ddd, *J* = 13.9, 8.2, 5.3 Hz, 1H), 2.37 – 2.33 (m, 1H), 2.01 (s, 3H), 1.71 – 1.65 (m, 4H), 1.65 – 1.61 (m, 1H), 1.47 – 1.39 (m, 3H), 1.38 – 1.28 (m, 1H), 1.24 – 1.10 (m, 6H), 0.91 – 0.81 (m, 2H) ppm.
^{13}C NMR (176 MHz, $CDCl_3$) δ 171.2, 71.7, 46.0, 37.7, 37.5, 35.5, 33.5, 26.8, 26.5, 23.4, 22.9 ppm.
HRMS [M+H]$^+$ m/z calc'd for $[C_{13}H_{25}NO_2H]^+$ expect 228.1964; found 228.1968.

***N*-(2-hydroxy-3-phenylpropyl)acetamide (1g-rac)**

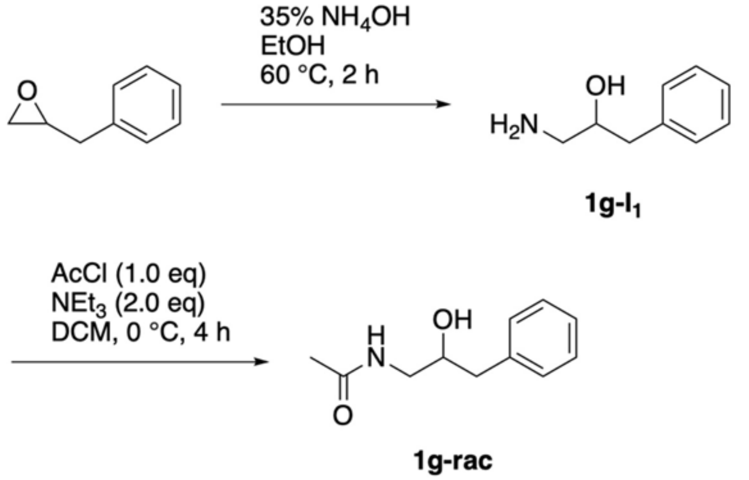

1g-I$_1$ was prepared according to **General Procedure C** using epoxide substrate (2,3-epoxypropane) benzene (0.39 mL, 2.96 mmol), NH_4OH (11 mL) and ethanol (11 mL) stirred for 2 h. Purification by silica gel column chromatography (elution with 5% 7N ammonia in MeOH, 10% MeOH, 85% DCM) afforded **1g-I$_1$** as an off-white solid (406 mg, 2.69 mmol, 91% yield).

^{1}H NMR (700 MHz, $CDCl_3$) δ 7.32 – 7.27 (m, 2H), 7.24 – 7.19 (m, 3H), 3.78 – 3.70 (m, 1H), 2.85 – 2.77 (m, 1H), 2.77 – 2.69 (m, 2H), 2.60 – 2.54 (m, 1H), 1.89 (br. s, 3H) ppm.
^{13}C NMR (176 MHz, $CDCl_3$) δ 138.6, 129.5, 128.6, 126.5, 73.2, 47.0, 41.6 ppm.

The data is in good agreement with the literature.[8]

1g-rac was prepared according to **General Procedure D** using amino alcohol substrate **1g-I$_1$** (302 mg, 2.00 mmol), triethylamine (0.56 mL, 4.02 mmol) and acetyl chloride (0.14 mL, 1.97 mmol). Purification by silica gel column chromatography (elution with 2% 7N ammonia in MeOH, 4% MeOH, 94% DCM) afforded **1g-rac** as a colorless, waxy solid (377 mg, 1.95 mmol, 97% yield).

^{1}H NMR (500 MHz, $CDCl_3$) δ 7.32 (t, J = 7.4 Hz, 2H), 7.24 (t, J = 7.4 Hz, 1H), 7.21 (d, J = 7.4 Hz, 2H), 5.93 (br. s, 1H), 3.98 – 3.91 (m, 1H), 3.55 (ddd, J = 14.0, 6.7, 2.9 Hz, 1H), 3.16 (ddd, J = 14.0, 7.8, 5.1 Hz, 1H), 2.81 (dd, J = 13.7, 5.3 Hz, 1H), 2.73 (dd, J = 13.7, 8.0 Hz, 1H), 2.66 (d, J = 3.8 Hz, 1H), 2.00 (s, 3H) ppm.
^{13}C NMR (176 MHz, $CDCl_3$) δ 171.3, 137.6, 129.5, 128.9, 126.9, 72.4, 45.3, 41.7, 23.4 ppm.

The data is in good agreement with the literature.[9]

***N*-(2-hydroxy-4-phenylbutyl)acetamide (1h-rac)**

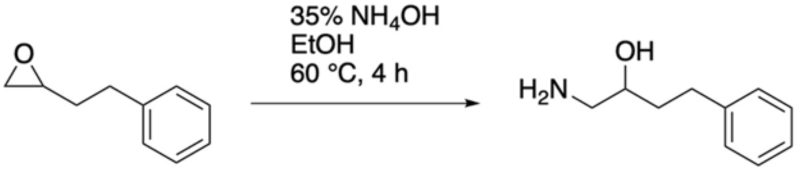

S-1h **1h-I$_1$**

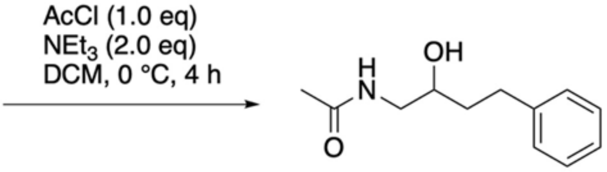

1h-rac

1h-I$_1$ was prepared according to **General Procedure C** using epoxide substrate **S-1h** (445 mg, 3.00 mmol; prepared according to literature procedure[10]), NH_4OH (11 mL) and ethanol (11 mL) stirred for 2 h. Purification by silica gel column chromatography (elution with 4% 7N ammonia in MeOH, 8% MeOH, 88% DCM) afforded **1h-I$_1$** as a pale yellow oil that slowly forms a colorless solid (426 mg, 2.58 mmol, 86% yield).

^{1}H NMR (500 MHz, $CDCl_3$) δ 7.31 – 7.26 (m, 2H), 7.23 – 7.16 (m, 3H), 3.56 – 3.49 (m, 1H), 2.90 – 2.78 (m, 2H), 2.69 (ddd, J = 13.8, 9.3, 7.2 Hz, 1H), 2.54 (dd, J = 12.5, 8.4 Hz, 1H), 1.90 (br. s, 3H), 1.80 – 1.66 (m, 2H) ppm.
^{13}C NMR (101 MHz, $CDCl_3$) δ 142.2, 128.6, 128.5, 126.0, 71.3, 47.5, 36.5, 32.1 ppm.

The data is in good agreement with the literature.[8]

1h-rac was prepared according to **General Procedure D** using amino alcohol substrate **1h-I$_1$** (331 mg, 2.00 mmol), triethylamine (0.56 mL, 4.02 mmol) and acetyl chloride (0.14 mL, 1.97 mmol). Purification by silica gel column chromatography (elution with 2% 7N ammonia in MeOH, 4% MeOH, 94% DCM) afforded **1h-rac** as a colorless oil that slowly forms a white solid (383 mg, 1.85 mmol, 92% yield).

^{1}H NMR (700 MHz, $CDCl_3$) δ 7.30 – 7.26 (m, 2H), 7.21 – 7.17 (m, 3H), 6.08 (br. s, 1H), 3.74 – 3.68 (m, 1H), 3.45 (ddd, J = 14.0, 6.4, 2.9 Hz, 1H), 3.17 (ddd, J = 14.0, 7.7, 5.4 Hz, 1H), 3.00 (br. s, 1H), 2.79 (ddd, J = 14.0, 8.8, 5.7 Hz, 1H), 2.68 (ddd, J = 14.0, 9.0, 7.2 Hz, 1H), 1.99 (s, 3H), 1.81 – 1.72 (m, 2H) ppm.
^{13}C NMR (176 MHz, $CDCl_3$) δ 171.5, 141.7, 128.6, 128.5, 126.1, 70.8, 46.1, 36.7, 31.9, 23.3 ppm.
HRMS $[M+H]^+$ m/z calc'd for $[C_{12}H_{17}NO_2H]^+$ expect 208.1338; found 208.1337.

***N*-(2-hydroxy-5-phenylpentyl)acetamide (1i-rac)**

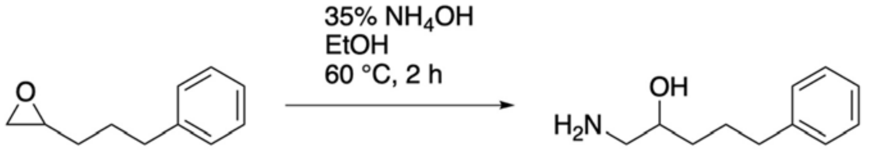

S-1i **1i-I1**

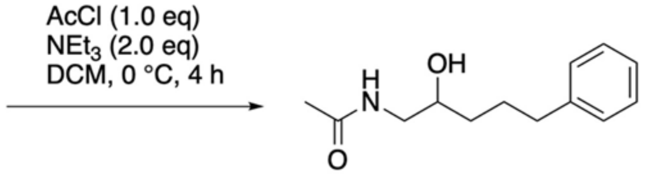

1i-rac

1i-I₁ was prepared according to **General Procedure C** using epoxide substrate **S-1i** (487 mg, 3.00 mmol; prepared in two steps according to literature procedures[11,12]), 35% NH_4OH (11 mL) and ethanol (11 mL) stirred for 2 h. Purification by silica gel column chromatography (elution with 4% 7N ammonia in MeOH, 8% MeOH, 88% DCM) afforded **1i-I₁** as a yellow oil (466 mg, 2.60 mmol, 87% yield).

^{1}H NMR (500 MHz, $CDCl_3$) δ 7.30 – 7.25 (m, 2H) (overlaps with $CHCl_3$ peak), 7.20 – 7.15 (m, 3H), 3.55 – 3.48 (m, 1H), 2.82 (app. d, *J* = 13.1 Hz, 1H), 2.64 (t, *J* = 7.7 Hz, 2H), 2.49 (dd, *J* = 12.6, 8.4 Hz, 1H), 1.97 – 1.75 (m, 4H), 1.73 – 1.62 (m, 1H), 1.52 – 1.39 (m, 2H) ppm.
^{13}C NMR (101 MHz, $CDCl_3$) δ 142.5, 128.5, 128.4, 125.9, 72.0, 47.5, 36.0, 34.4, 27.6 ppm.
HRMS $[M+H]^+$ m/z calc'd for $[C_{11}H_{17}NOH]^+$ expect 180.1388; found 180.1387.

1i-rac was prepared according to **General Procedure D** using amino alcohol substrate **1i-I₃** (338 mg, 1.89 mmol), triethylamine (0.53 mL, 3.80 mmol) and acetyl chloride (0.13 mL, 1.83 mmol). Purification by silica gel column chromatography (elution with 2% 7N ammonia in MeOH, 4% MeOH, 94% DCM) afforded **1i-rac** as a colorless oil (366 mg, 1.66 mmol, 88% yield).

^{1}H NMR (700 MHz, $CDCl_3$) δ 7.27 (t, *J* = 7.5 Hz, 2H), 7.21 – 7.14 (m, 3H), 5.91 (br. s, 1H), 3.75 – 3.69 (m, 1H), 3.45 (ddd, *J* = 14.0, 6.5, 2.9 Hz, 1H), 3.10 (ddd, *J* = 14.0, 7.8, 5.2 Hz, 1H), 2.64 (t, *J* = 7.6 Hz, 2H), 2.58 (br. d, *J* = 4.5 Hz, 1H), 1.99 (s, 3H), 1.83 – 1.75 (m, 1H), 1.71 – 1.63 (m, 1H) (overlaps with H_2O peak), 1.53 – 1.44 (m, 2H) ppm.
^{13}C NMR (176 MHz, $CDCl_3$) δ 171.4, 142.2, 128.6, 128.5, 126.0, 71.5, 46.0, 35.8, 34.6, 27.3, 23.3 ppm.
HRMS $[M+H]^+$ m/z calc'd for $[C_{13}H_{19}NO_2H]^+$ expect 222.1494; found 222.1485.

***N*-(2-hydroxy-6-phenylhexyl)acetamide (1j-rac)**

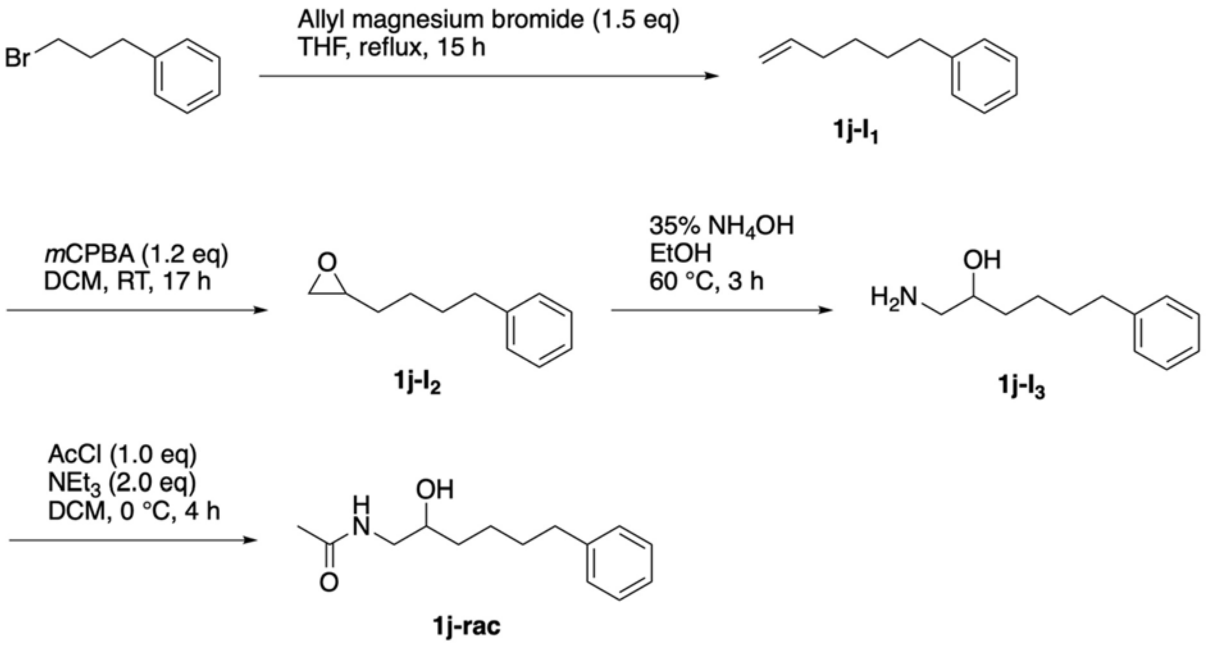

Impure **1j-I$_2$** was prepared in two steps according to modified literature procedures, performing **General Procedure A** using bromoalkane substrate 1-bromo-3-phenylpropane (1.14 mL, 7.50 mmol) and allyl magnesium bromide (11.3 mL, 11.3 mmol) stirred overnight for 15 h to afford crude **1j-I$_1$**. The crude material was brought forward without additional purification to **General Procedure B** using *m*CPBA (2.02 g, 9.01 mmol) stirred overnight for 17 h. Purification by silica gel column chromatography (elution with 5% acetone in petroleum ether) afforded **1j-I$_2$** as a pale yellow oil containing ≤ 24% impurity (806 mg).[5,13] **1j-I$_3$** was prepared according to **General Procedure C** using impure epoxide substrate **1j-I$_2$** (529 mg), 35% NH_4OH (11 mL) and ethanol (11 mL) stirred for 3 h. Purification by silica gel column chromatography (elution with 4% 7N ammonia in MeOH, 8% MeOH, 88% DCM) afforded **1j-I$_3$** as a white solid (138 mg, 0.715 mmol, 15% corrected yield over three steps).

^{1}H NMR (700 MHz, $CDCl_3$) δ 7.27 (t, *J* = 7.6 Hz, 2H) (overlaps with $CHCl_3$ peak), 7.19 – 7.15 (m, 3H), 3.54 – 3.48 (m, 1H), 2.83 (dd, *J* = 12.6, 2.8 Hz, 1H), 2.62 (t, *J* = 7.7 Hz, 2H), 2.51 (dd, *J* = 12.6, 8.5 Hz, 1H), 2.28 (br. s, 3H), 1.70 – 1.59 (m, 2H), 1.55 – 1.48 (m, 1H), 1.48 – 1.41 (m, 2H), 1.41 – 1.34 (m, 1H) ppm.
^{13}C NMR (176 MHz, $CDCl_3$) δ 142.7, 128.5, 128.4, 125.8, 71.8 (2C), 47.4, 36.0, 34.8, 31.7, 25.5 ppm.
HRMS $[M+H]^+$ m/z calc'd for $[C_{12}H_{19}NOH]^+$ expect 194.1545; found 194.1542.

1j-rac was prepared according to **General Procedure D** using amino alcohol substrate **1j-I$_3$** (123 mg, 0.64 mmol), triethylamine (0.18 mL, 1.29 mmol) and acetyl chloride (0.05 mL, 0.70 mmol). Purification by silica gel column chromatography (elution with 1.5% 7N ammonia in MeOH, 3% MeOH, 95.5% DCM) afforded **1j-rac** as a colorless oil that slowly forms a white solid (88.1 mg, 0.374 mmol, 58% yield).

^{1}H NMR (700 MHz, $CDCl_3$) δ 7.27 (t, *J* = 7.6 Hz, 2H), (overlaps with $CHCl_3$ peak), 7.19 – 7,14 (m, 3H), 6.07 (br. s, 1H), 3.72 – 3.64 (m, 1H), 3.45 (ddd, *J* = 13.9, 6.5, 2.9 Hz, 1H), 3.09 (ddd, *J* = 13.9, 8.1, 5.3 Hz, 1H), 2.61 (t, *J* = 7.7 Hz, 2H), 2.51 (br. s, 1H), 2.00 (s, 3H), 1.70 – 1.59 (m, 2H), 1.52 – 1.43 (m, 3H), 1.42 – 1.33 (m, 1H) ppm.
^{13}C NMR (176 MHz, $CDCl_3$) δ 171.4, 142.5, 128.5, 128.4, 125.8, 71.4, 46.0, 35.9, 35.0, 31.5, 25.3, 23.3 ppm.
HRMS $[M+H]^+$ m/z calc'd for $[C_{14}H_{21}NO_2H]^+$ expect 236.1651; found 236.1643.

***N*-(2-hydroxy-7-phenylheptyl)acetamide (1k-rac)**

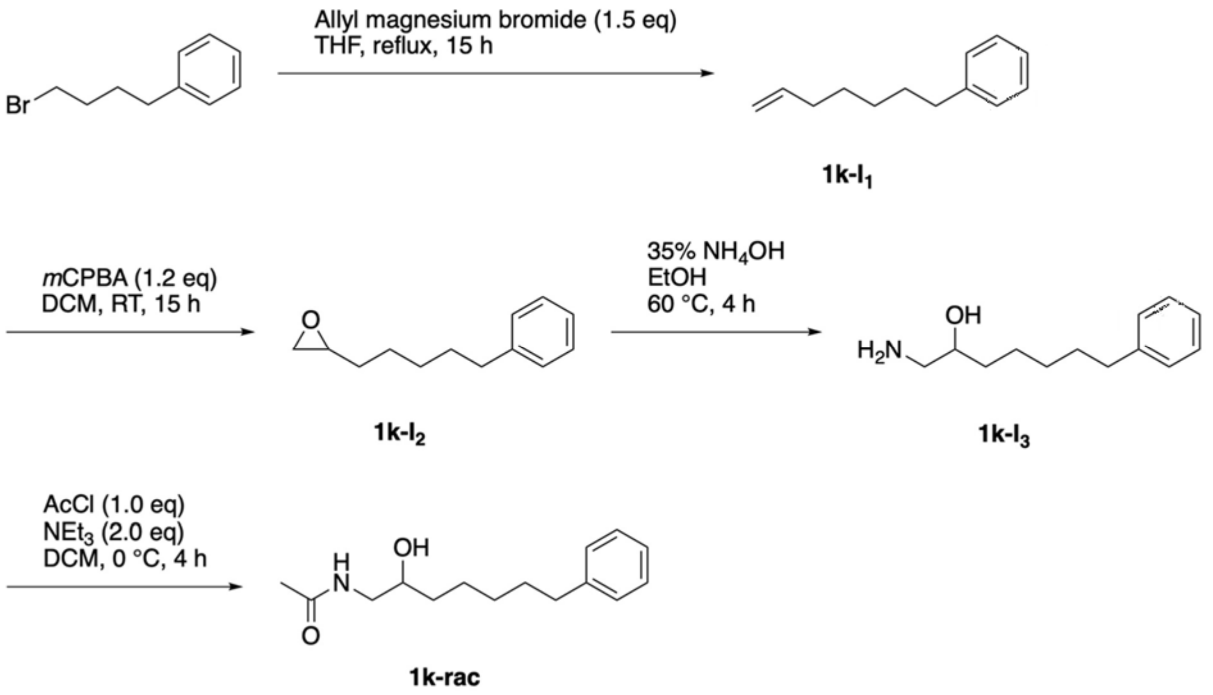

1k-I$_1$ was prepared according to modified literature procedure, performing **General Procedure A** using bromoalkane substrate 1-bromo-4-phenylbutane (1.32 mL, 7.50 mmol) and allyl magnesium bromide (11.3 mL, 11.3 mmol) stirred overnight for 15 h.[5] Purification by silica gel column chromatography (elution with hexane) afforded **1k-I$_1$** as a colorless oil (1.24g, 7.11 mmol, 95% yield).

^{1}H NMR (400 MHz, $CDCl_3$) δ 7.33 – 7.26 (m, 2H), 7.23 – 7.16 (m, 3H), 5.83 (ddt, *J* = 17.0, 10.3, 6.7 Hz, 1H), 5.01 (ddt, 17.0, 1.8, 1.6 Hz, 1H), 4.95 (ddt, 10.3, 2.2, 1.1 Hz, 1H), 2.62 (t, *J* = 7.8 Hz, 2H), 2.11 – 2.02 (m, 2H), 1.70 – 1.59 (m, 2H), 1.49 – 1.33 (m, 4H) ppm.
^{13}C NMR (101 MHz, $CDCl_3$) δ 143.0, 139.2, 128.5, 128.4, 125.7, 114.4, 36.1, 33.9, 31.5, 29.0, 28.9 ppm.

The data is in good agreement with the literature.[5]

1k-I$_2$ was prepared according to **General Procedure B** using alkene substrate **1k-I$_1$** (1.05 g, 6.00 mmol) and *m*CPBA (1.61 g, 7.18 mmol) stirred overnight for 15 h. Purification by silica gel column chromatography (elution with 5% acetone in petroleum ether) afforded **1k-I$_2$** as a colorless oil (1.00 g, 5.27 mmol, 88% yield).

^{1}H NMR (700 MHz, $CDCl_3$) δ 7.28 (t, *J* = 7.6 Hz, 2H), 7.20 – 7.16 (m, 3H), 2.93 – 2.88 (m, 1H), 2.74 (dd, *J* = 5.0, 4.1 Hz, 1H), 2.62 (t, *J* = 7.8 Hz, 2H), 2.46 (dd, *J* = 5.0, 2.7 Hz, 1H), 1.65 (app. quint, *J* = 7.7 Hz, 2H), 1.56 – 1.45 (m, 4H), 1.43 – 1.37 (m, 2H) ppm.
^{13}C NMR (176 MHz, $CDCl_3$) δ 142.8, 128.5, 128.4, 125.8, 52.5, 47.2, 36.0, 32.5, 31.5, 29.2, 26.0 ppm.
HRMS $[M+H]^+$ m/z calc'd for $[C_{13}H_{18}OH]^+$ expect 191.1436; found 191.1436.

1k-I$_3$ was prepared according to **General Procedure C** using epoxide substrate **1k-I$_2$** (571 mg, 3.00 mmol), 35% NH_4OH (11 mL) and ethanol (11 mL) stirred for 4 h. Purification by silica gel column chromatography (elution with 4% 7N ammonia in MeOH, 8% MeOH, 88% DCM) afforded **1k-I$_3$** as a yellow oil (458 mg, 2.21 mmol, 74% yield).

^{1}H NMR (700 MHz, $CDCl_3$) δ 7.29 – 7.25 (m, 2H) (overlaps with $CHCl_3$ peak), 7.19 – 7.15 (m, 3H), 3.53 – 3.45 (m, 1H), 2.83 (app. br. d, *J* = 11.8 Hz, 1H), 2.61 (t, *J* = 7.8 Hz, 2H), 2.50 (dd, *J* = 12.5, 8.4 Hz, 1H), 1.86 (br. s, 3H), 1.63 (app. quint, *J* = 7.6 Hz, 2H), 1.53 – 1.46 (m, 1H), 1.46 – 1.31 (m, 5H) ppm.
^{13}C NMR (176 MHz, $CDCl_3$) δ 142.9, 128.5, 128.4, 125.7, 72.1, 47.5, 36.0, 34.8, 31.5, 29.5, 25.7 ppm.
HRMS $[M+H]^+$ m/z calc'd for $[C_{13}H_{21}NOH]^+$ expect 208.1701; found 208.1704.

1k-rac was prepared according to **General Procedure D** using amino alcohol substrate **1k-I$_3$** (327 mg, 1.58 mmol), triethylamine (0.44 mL, 3.16 mmol) and acetyl chloride (0.11 mL, 1.55 mmol). Purification by silica gel column chromatography (elution with 2% 7N ammonia in MeOH, 4% MeOH, 94% DCM) afforded **1k-rac** as a white solid (357 mg, 1.43 mmol, 91% yield).

^{1}H NMR (700 MHz, $CDCl_3$) δ 7.29 – 7.24 (m, 2H) (overlaps with $CHCl_3$ peak), 7.19 – 7.14 (m, 3H), 6.05 (br. s, 1H), 3.71 – 3.64 (m, 1H), 3.45 (ddd, *J* = 14.0, 6.5, 2.9 Hz, 1H), 3.09 (ddd, *J* = 14.0, 8.0, 5.4 Hz, 1H), 2.79 – 2.69 (m, 1H), 2.60 (t, *J* = 7.7 Hz, 2H), 2.00 (s, 3H), 1.62 (app. quint, *J* = 7.6 Hz, 2H), 1.50 – 1.40 (m, 3H), 1.40 – 1.29 (m, 3H) ppm.
^{13}C NMR (176 MHz, $CDCl_3$) δ 171.4, 142.8, 128.5, 128.4, 125.8, 71.5, 46.0, 36.0, 35.1, 31.5, 29.3, 25.5, 23.3 ppm.
HRMS $[M+H]^+$ m/z calc'd for $[C_{15}H_{23}NO_2H]^+$ expect 250.1807; found 250.1799.

***tert*-butyl (5-acetamido-4-hydroxypentyl)carbamate (1l-rac)**

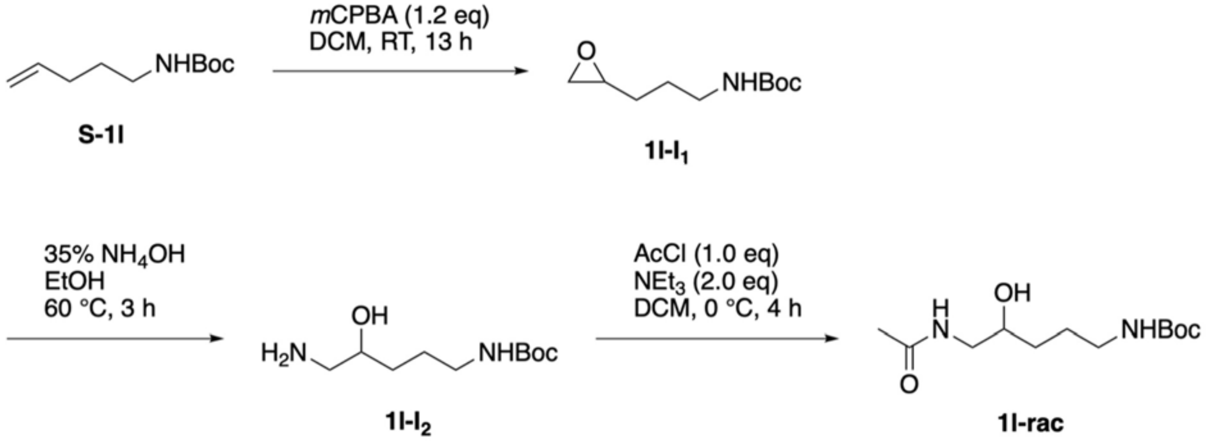

1l-I$_1$ was prepared according to **General Procedure B** using alkene substrate **S-1l** (556 mg, 3.00 mmol; prepared according to literature procedure[14]) and *m*CPBA (807 mg, 3.60 mmol) stirred overnight for 13 h. Crude **1l-I$_1$** was brought forward without additional purification to **General Procedure C** using 35% NH_4OH (11 mL) and ethanol (11 mL) stirred for 3 h. The crude material was partially purified by silica gel column chromatography (elution with 7% 7N ammonia in MeOH, 14% MeOH, 79% DCM). Impure **1l-I$_2$** (740 mg) was then brought forward to **General Procedure D** using triethylamine (0.84 mL, 6.03 mL) and acetyl chloride (0.21 mL, 2.95 mmol) to afford **1l-rac** as a colorless oil (489 mg, 1.88 mmol, 63% over three steps).

^{1}H NMR (700 MHz, $CDCl_3$) δ 6.13 (br. s, 1H), 4.68 (br. s, 1H), 3.77 – 3.70 (m, 1H), 3.46 (ddd, *J* = 14.0, 6.2, 2.8 Hz, 1H), 3.25 – 3.16 (m, 1H), 3.16 – 3.07 (m, 2H), 2.90 (br. s, 1H), 2.02 (s, 3H), 1.67 – 1.60 (m, 1H), 1.60 – 1.54 (m, 1H), 1.54 – 1.45 (m, 2H), 1.44 (s, 9H) ppm.
^{13}C NMR (176 MHz, $CDCl_3$) δ 171.6, 156.6, 79.6, 71.2, 46.2, 40.2, 31.6, 28.6, 26.5, 23.3 ppm.
HRMS $[M+H]^+$ m/z calc'd for $[C_{12}H_{24}N_2O_4H]^+$ expect 261.1814; found 261.1804.

ethyl 7-acetamido-6-hydroxyheptanoate (1m-rac)

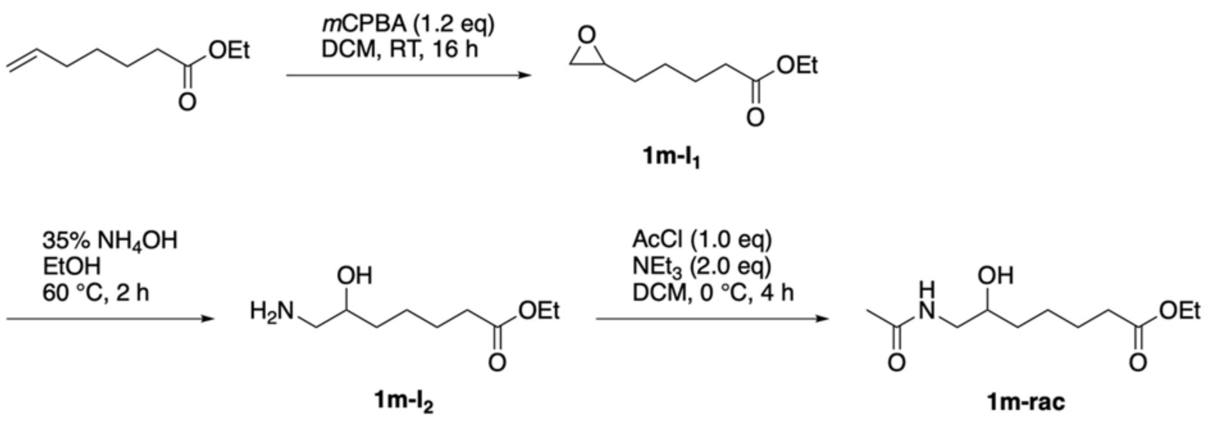

Crude **1m-I$_1$** was prepared according to modified literature procedure, performing **General Procedure B** using alkene substrate ethyl-6-heptenoate (0.88 mL, 5.01 mmol) and *m*CPBA

(1.34 g, 5.98 mmol) stirred overnight for 16 h without purification by silica gel column chromataography.[15] Crude **1m-I$_1$** was brought forward without additional purification to **General Procedure C** using NH_4OH (11 mL) and ethanol (11 mL) stirred for 2 h. The crude material was partially purified by silica gel column chromatography (elution with 5% 7N ammonia in MeOH, 10% MeOH, 85% DCM). Impure **1m-I$_2$** (607 mg) was then brought forward to **General Procedure D** using triethylamine (0.89 mL, 6.39 mmol) and acetyl chloride (0.23 mL, 3.23 mmol). Purification by silica gel column chromatography (elution with 1.5% 7N ammonia in MeOH, 3% MeOH, 95.5% DCM) afforded **1m-rac** as an off-white waxy solid (514 mg, 2.22 mmol, 44% yield over three steps).

^{1}H NMR (700 MHz, $CDCl_3$) δ 6.05 (br. s, 1H), 4.12 (q, *J* = 7.1 Hz, 2H), 3.74 – 3.67 (m, 1H), 3.46 (ddd, *J* = 13.9, 6.5, 2.9 Hz, 1H), 3.10 (ddd, *J* = 13.9, 7.9, 5.4 Hz, 1H), 2.89 – 2.82 (m, 1H), 2.30 (t, *J* = 7.4 Hz, 2H), 2.00 (s, 3H), 1.69 – 1.58 (m, 2H), 1.51 – 1.43 (m, 3H), 1.41 – 1.33 (m, 1H), 1.25 (t, *J* = 7.1 Hz, 3H) ppm.
^{13}C NMR (176 MHz, $CDCl_3$) δ 173.9, 171.4, 71.2, 60.5, 45.9, 34.6, 34.2, 25.0, 24.7, 23.3, 14.4 ppm.
HRMS $[M+H]^+$ m/z calc'd for $[C_{11}H_{21}NO_4H]^+$ expect 232.1549; found 232.1553.

***N*-(5-((*tert*-butyldimethylsilyl)oxy)-2-hydroxypentyl)acetamide (1n-rac)**

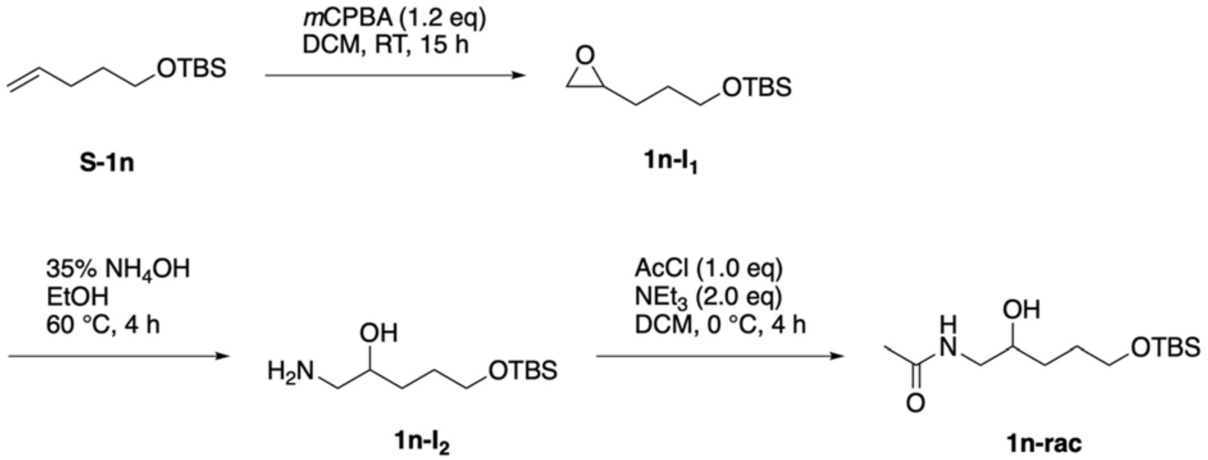

Crude **1n-I$_1$** was prepared according to modified literature procedure, performing **General Procedure B** using alkene substrate **S-1n** (3.63 g, 18.1 mmol; prepared according to literature procedure[16]) and *m*CPBA (4.87 g, 21.7 mmol) stirred overnight for 15 h.[17] A solution of crude **1n-I$_1$** in ethanol (44 mL) was partitioned equally into four microwave vials. 35% NH_4OH (11 mL) was added to each vial. The vials were sealed, stirred vigorously and heated to 60 °C for 4h. The reaction mixtures were combined, concentrated *in vacuo* and purified by silica gel column chromatography (elution with 4% 7N ammonia in MeOH, 8% MeOH, 88% DCM) to yield **1n-I$_2$** as a colorless oil (2.98 g, 12.8 mmol, 70% over two steps).

^{1}H NMR (700 MHz, $CDCl_3$) δ 3.69 – 3.62 (m, 2H), 3.54 (app. hept, *J* = 4.0 Hz, 1H), 2.82 (dd, *J* = 12.6, 3.3 Hz, 1H), 2.57 (dd, *J* = 12.6, 8.2 Hz, 1H), 2.42 (br. s, 3H), 1.70 – 1.60 (m, 2H), 1.59 – 1.53 (m, 1H), 1.48 – 1.42 (m, 1H), 0.89 (s, 9H), 0.06 (s, 6H) ppm.
^{13}C NMR (176 MHz, $CDCl_3$) δ 72.1, 63.6, 47.7, 32.1, 29.3, 26.1, 18.5, –5.23 ppm.
HRMS $[M+H]^+$ m/z calc'd for $[C_{11}H_{27}NO_2SiH]^+$ expect 234.1889; found 234.1881.

1n-rac was prepared according to **General Procedure D** using amino alcohol substrate **1n-I$_2$** (2.97 g, 12.7 mmol), triethylamine (3.54 mL, 25.4 mmol) and acetyl chloride (0.90 mL, 12.7 mmol). Purification by silica gel column chromatography (elution with 1.5% 7N ammonia in MeOH, 3% MeOH, 95.5% DCM) afforded **1n-rac** as a white solid (2.85 g, 10.3 mmol, 81% yield).

^{1}H NMR (700 MHz, $CDCl_3$) δ 6.05 (br. s, 1H), 3.99 – 3.93 (m, 1H), 3.73 – 3.66 (m, 2H), 3.66 – 3.61 (m, 1H), 3.50 (ddd, J = 13.6, 6.8, 3.2 Hz, 1H), 3.05 (ddd, J = 13.6, 8.3, 4.8 Hz, 1H), 1.90 (s, 3H), 1.74 – 1.59 (m, 3H), 1.51 – 1.44 (m, 1H), 0.89 (s, 9H), 0.07 (s, 6H) ppm.
^{13}C NMR (176 MHz, $CDCl_3$) δ 170.8, 70.8, 63.7, 45.7, 33.0, 29.2, 26.0, 23.4, 18.4, –5.29, –5.32 ppm.
HRMS $[M+H]^+$ m/z calc'd for $[C_{13}H_{29}NO_3SiH]^+$ expect 276.1995; found 276.1999.

***N*-(5-ethoxy-2-hydroxypentyl)acetamide (1o-rac)**

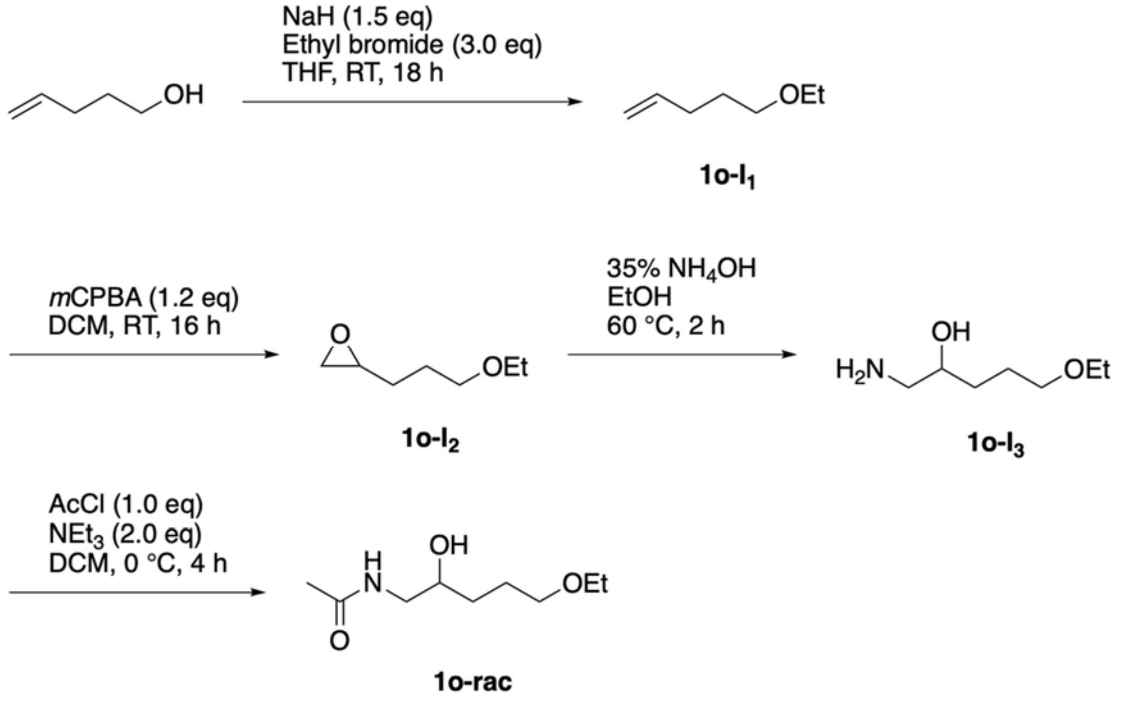

A solution of sodium hydride (60% in mineral oil, 288 mg, 7.20 mmol) in dry THF (20 mL) was stirred at 0 °C in a sealed oven-dried microwave vial under nitrogen atmosphere. 4-penten-1-ol (0.50 mL, 4.84 mmol) was added dropwise, and the reaction was stirred for 3 h at RT. Ethyl bromide (1.07 mL, 14.3 mmol) was then added dropwise at RT and the reaction mixture stirred overnight for 18 h. The reaction was quenched at 0 °C with saturated aqueous ammonium chloride solution, diluted with diethyl ether and extracted with diethyl ether. The combined organic extracts were dried over $MgSO_4$, filtered, and the solvent removed *in vacuo*. Crude **1o-I$_1$** was brought forward without additional purification to **General Procedure B** using *m*CPBA (1.29 g, 5.76 mmol) stirred overnight for 16 h. Crude **1o-I$_2$** was brought forward without additional purification to **General Procedure C** using 35% NH_4OH (11 mL) and ethanol (11 mL) stirred for 2 h. Purification by silica gel column chromatography (elution with 5% 7N

ammonia in MeOH, 10% MeOH, 85% DCM) afforded **1o-I$_3$** as a yellow oil (297 mg, 2.02 mmol, 42% yield over three steps).

^{1}H NMR (700 MHz, $CDCl_3$) δ 3.54 (app. hept, *J* = 4.0 Hz, 1H), 3.49 (q, *J* = 7.1 Hz, 2H), 3.46 (t, *J* = 6.0 Hz, 2H), 2.81 (dd, *J* = 12.7, 3.2 Hz, 1H), 2.58 (dd, *J* = 12.7, 8.2 Hz, 1H), 2.31 (br. s, 3H), 1.77 – 1.66 (m, 2H), 1.61 – 1.55 (m, 1H), 1.48 – 1.42 (m, 1H), 1.20 (t, *J* = 7.0 Hz, 3H) ppm.
^{13}C NMR (176 MHz, $CDCl_3$) δ 72.2, 70.9, 66.4, 47.7, 32.4, 26.5, 15.3 ppm.
HRMS $[M+Na]^+$ m/z calc'd for $[C_7H_{17}NO_2Na]^+$ expect 170.1157; found 170.1159.

1o-rac was prepared according to **General Procedure D** using amino alcohol substrate **1o-I$_3$** (147.2 mg, 1.0 mmol), triethylamine (0.28 mL, 2.01 mmol) and acetyl chloride (0.07 mL, 0.98 mmol). Purification by silica gel column chromatography (elution with 1.5% 7N ammonia in MeOH, 3% MeOH, 95.5% DCM) afforded **1o-rac** as a colorless oil (147 mg, 0.77 mmol, 77% yield).

^{1}H NMR (700 MHz, $CDCl_3$) δ 6.07 (br. s, 1H), 3.68 (app. tt, *J* = 8.5, 3.1 Hz, 1H), 3.54 – 3.47 (m, 4H), 3.44 (ddd, *J* = 9.4, 7.8, 4.0 Hz, 1H), 3.05 (ddd, *J* = 13.4, 8.4, 4.7 Hz, 1H), 2.00 (s, 3H), 1.79 – 1.64 (m, 3H), 1.51 – 1.44 (m, 1H), 1.21 (t, *J* = 7.0 Hz, 3H) ppm.
^{13}C NMR (176 MHz, $CDCl_3$) δ 170.8, 71.0, 70.8, 66.6, 45.8, 33.3, 26.6, 23.4, 15.1 ppm.
HRMS $[M+H]^+$ m/z calc'd for $[C_9H_{19}NO_3H]^+$ expect 190.1443; found 190.1442.

***N*-(2-hydroxy-4-(tetrahydro-2*H*-pyran-4-yl)butyl)acetamide (1p-rac)**

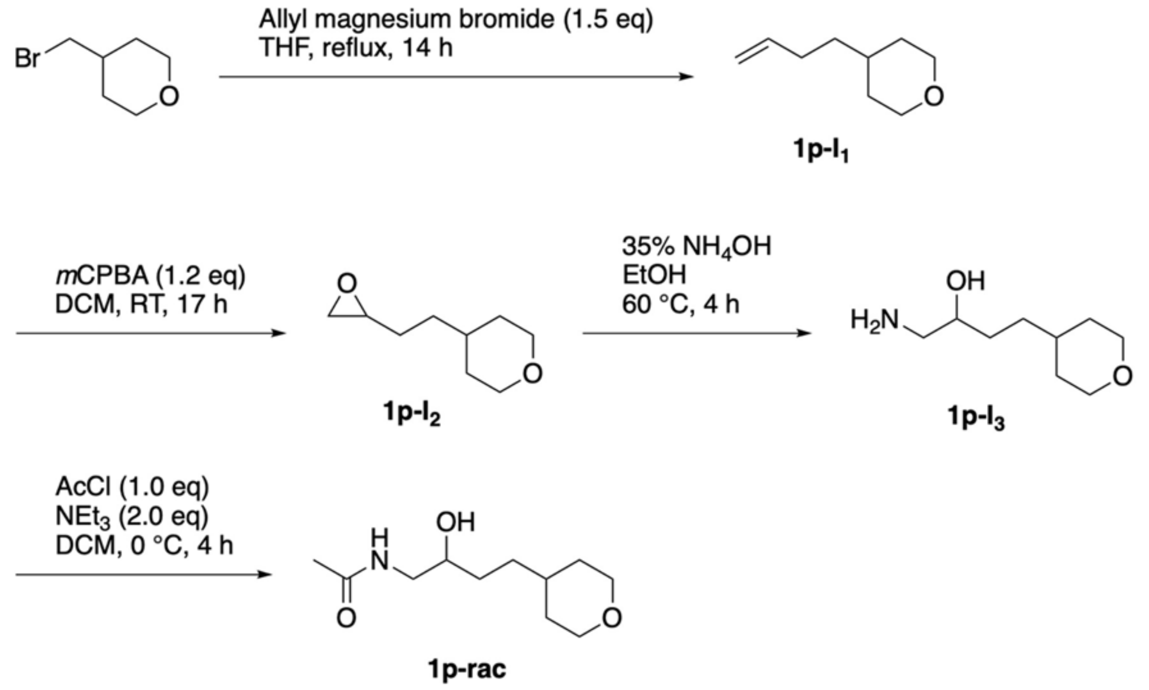

1p-I$_1$ was prepared according to **General Procedure A** using bromoalkane substrate 4-(bromomethyl)tetrahydropyran (0.54 mL, 4.10 mmol) and allyl magnesium bromide (6.15 mL, 6.15 mmol) stirred overnight for 14 h. The crude material was brought forward without

additional purification to **General Procedure B** using *m*CPBA (1.10 g, 4.91 mmol) stirred overnight for 17 h. Crude **1p-I$_2$** was then brought forward without additional purification to **General Procedure C** using 35% NH_4OH (11 mL) and ethanol (11 mL) stirred for 4 h. Purification by silica gel column chromatography (elution with 5% 7N ammonia in MeOH, 10% MeOH, 85% DCM) afforded **1p-I$_3$** as a yellow oil (121 mg, 0.696 mmol, 17% yield over three steps).

^{1}H NMR (700 MHz, $CDCl_3$) δ 3.94 (br. dd, *J* = 10.9, 3.9 Hz, 2H), 3.61 – 3.56 (m, 1H), 3.36 (br. t, *J* = 11.8 Hz, 2H), 2.95 – 2.85 (m, 4H), 2.60 (dd, *J* = 12.6, 8.9 Hz, 1H), 1.59 (app. br. d, *J* = 13.3 Hz, 2H), 1.51 – 1.40 (m, 4H), 1.31 – 1.23 (m, 3H) ppm.
^{13}C NMR (176 MHz, $CDCl_3$) δ 71.5, 68.2 (2C), 47.0, 35.2, 33.2 (2C), 33.0, 31.7 ppm.
HRMS $[M+H]^+$ m/z calc'd for $[C_9H_{19}NO_2H]^+$ expect 174.1494; found 174.1495.

1p-rac was prepared according to **General Procedure D** using amino alcohol substrate **1p-I$_3$** (114 mg, 0.66 mmol), triethylamine (0.18 mL, 1.29 mmol) and acetyl chloride (0.05 mL, 0.70 mmol). Purification by silica gel column chromatography (elution with 2% 7N ammonia in MeOH, 4% MeOH, 94% DCM) afforded **1p-rac** as an off-white solid (95.9 mg, 0.445 mmol, 68% yield).

^{1}H NMR (700 MHz, $CDCl_3$) δ 6.04 (br. s, 1H), 3.94 (br. dd, *J* = 11.0, 3.7 Hz, 2H), 3.70 – 3.65 (m, 1H), 3.46 (ddd, *J* = 14.0, 6.5, 2.9 Hz, 1H), 3.35 (br. t, *J* = 11.7 Hz, 2H), 3.11 (ddd, *J* = 14.0, 8.0, 5.4 Hz, 1H), 2.79 (br. s, 1H), 2.01 (s, 3H), 1.58 (app. br. d, *J* = 13.0 Hz, 2H), 1.50 – 1.43 (m, 3H), 1.43 – 1.36 (m, 1H), 1.31 – 1.22 (m, 3H) ppm.
^{13}C NMR (176 MHz, $CDCl_3$) δ 171.4, 71.7, 68.2 (2C), 46.1, 35.1, 33.2 (2C), 32.8, 32.0, 23.3 ppm.
HRMS $[M+H]^+$ m/z calc'd for $[C_{11}H_{21}NO_3H]^+$ expect 216.1600; found 216.1595.

***N*-(2-hydroxy-4-phenoxybutyl)acetamide (1q-rac)**

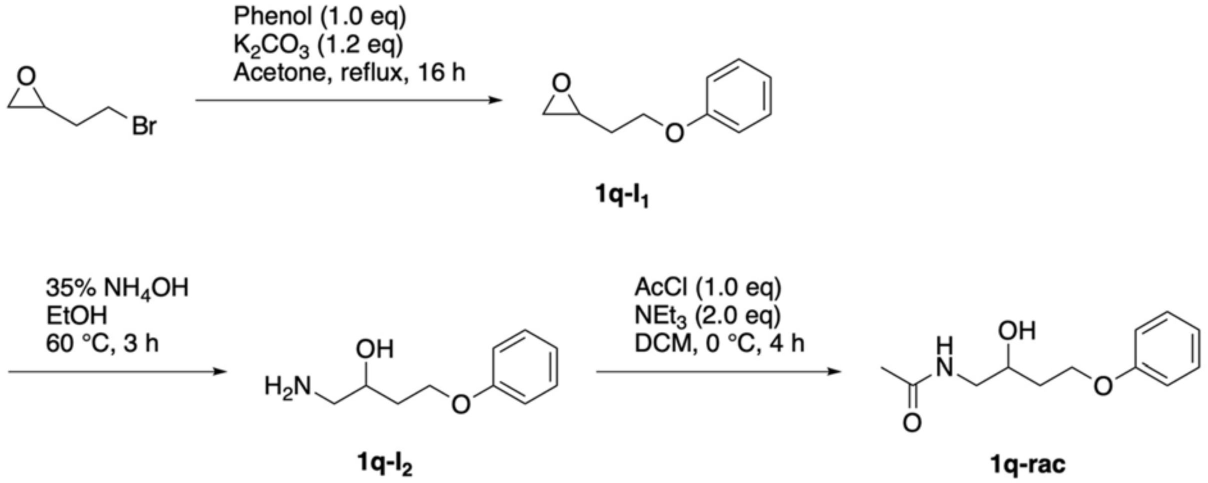

Crude **1q-I$_1$** was prepared according to modified literature procedure.[18] A suspension of phenol (565 mg, 6.00 mmol), 2-(2-bromoethyl)oxirane (0.60 mL, 6.00 mmol) and anhydrous potassium carbonate (995 mg, 7.20 mmol) in acetone (35 mL) was stirred vigorously in an

oven dried two-necked RBF attached to an oven-dried condenser. The reaction mixture was heated to reflux and stirred overnight for 16 h. The mixture was cooled to RT, filtered through a pad of Celite, the Celite washed with acetone, and the filtrate concentrated *in vacuo*. The crude mixture was redissolved in diethyl ether and sequentially washed with water, 5% aqueous sodium hydroxide and water. The organic phase was dried over $MgSO_4$, filtered, and the solvent removed *in vacuo*. The crude material was brought forward without additional purification to **General Procedure C** using 35% NH_4OH (11 mL) and ethanol (11 mL) stirred for 3 h. Purification by silica gel column chromatography (elution with 4% 7N ammonia in MeOH, 8% MeOH, 88% DCM) afforded **1q-I$_2$** as an orange solid (363 mg, 2.01 mmol, 33% yield over two steps).

^{1}H NMR (700 MHz, $CDCl_3$) δ 7.30 – 7.26 (m, 2H), 6.94 (t, *J* = 7.0 Hz, 1H), 6.90 (d, *J* = 7.2 Hz, 2H), 4.20 – 4.09 (m, 2H), 3.85 – 3.75 (m, 1H), 2.90 (app. br. s, 1H), 2.64 (app. br. s, 1H), 2.20 – 1.78 (m, 5H) ppm.
^{13}C NMR (176 MHz, $CDCl_3$) δ 158.9, 129.6, 120.9, 114.6, 69.9, 65.2, 47.6, 34.2 ppm.
HRMS $[M+H]^+$ m/z calc'd for $[C_{10}H_{15}NO_2H]^+$ expect 182.1181; found 182.1174.

1q-rac was prepared according to **General Procedure D** using amino alcohol substrate **1q-I$_2$** (256 mg, 1.41 mmol), triethylamine (0.39 mL, 2.80 mmol) and acetyl chloride (0.10 mL, 1.41 mmol). Purification by silica gel column chromatography (elution with 4% MeOH in DCM) afforded **1q-rac** as a white solid (283 mg, 1.27 mmol, 90% yield).

^{1}H NMR (500 MHz, $CDCl_3$) δ 7.31 – 7.26 (m, 2H), 6.96 (t, *J* = 7.4 Hz, 1H), 6.92 – 6.86 (m, 2H), 6.09 (br. s, 1H), 4.19 – 4.09 (m, 2H), 4.04 – 3.97 (m, 1H), 3.52 (ddd, *J* = 14.0, 6.4, 3.1 Hz, 1H), 3.39 (d, *J* = 3.9 Hz, 1H), 3.25 (ddd, *J* = 14.0, 7.3, 5.4 Hz, 1H), 2.01 (s, 3H), 1.97 – 1.89 (m, 2H) ppm.
^{13}C NMR (126 MHz, $CDCl_3$) δ 171.4, 158.6, 129.7, 121.2, 114.6, 69.7, 65.5, 45.9, 34.1, 23.3 ppm.
HRMS $[M+H]^+$ m/z calc'd for $[C_{12}H_{17}NO_3H]^+$ expect 224.1287; found 224.1280.

***N*-(2-hydroxy-3-(4-methoxyphenyl)propyl)acetamide (1r-rac)**

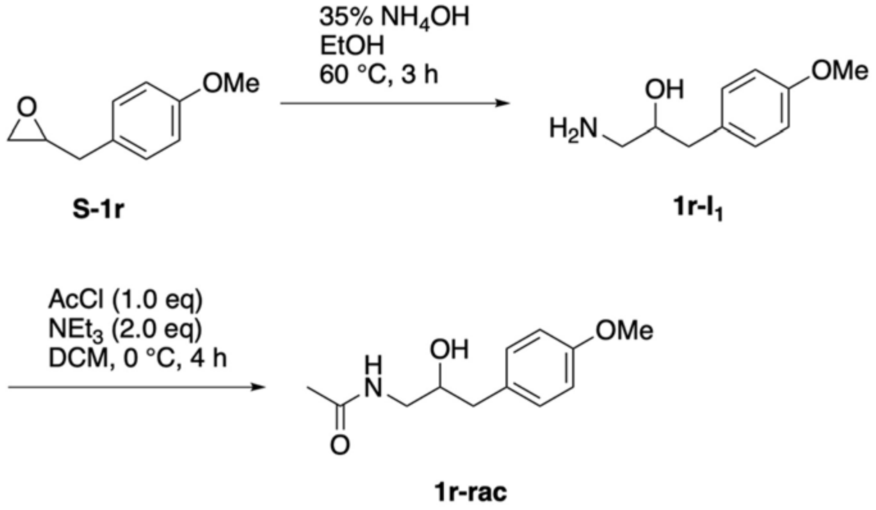

1r-I_1 was prepared according to **General Procedure C** using epoxide substrate **S-1r** (493 mg, 3.00 mmol; prepared according to literature procedure[10]), 35% NH_4OH (11 mL) and ethanol (11 mL) stirred for 3 h. Purification by silica gel column chromatography (elution with 5% 7N ammonia in MeOH, 10% MeOH, 85% DCM) afforded **1r-I_1** as a pale yellow solid (459 mg, 2.53 mmol, 84% yield).

^{1}H NMR (700 MHz, $CDCl_3$) δ 7.13 (d, *J* = 8.6 Hz, 2H), 6.84 (d, *J* = 8.6 Hz, 2H), 3.78 (s, 3H), 3.73 – 3.67 (m, 1H), 2.82 (dd, *J* = 12.7, 3.3 Hz, 1H), 2.68 (d, *J* = 6.5 Hz, 2H), 2.57 (dd, *J* = 12.7, 8.0 Hz, 1H), 1.80 (br. s, 3H) ppm.
^{13}C NMR (176 MHz, $CDCl_3$) δ 158.3, 130.43, 130.38, 114.1, 73.4, 55.4, 47.0, 40.6 ppm.
HRMS $[M+H]^+$ m/z calc'd for $[C_{10}H_{15}NO_2H]^+$ expect 182.1181; found 182.1174.

1r-rac was prepared according to **General Procedure D** using amino alcohol substrate **1r-I_1** (363 mg, 2.00 mmol), triethylamine (0.56 mL, 4.02 mmol) and acetyl chloride (0.14 mL, 1.97 mmol). Purification by silica gel column chromatography (elution with 1.5% 7N ammonia in MeOH, 3% MeOH, 95.5% DCM) afforded **1r-rac** as a pale yellow oil (387 mg, 1.73 mmol, 87% yield).

^{1}H NMR (700 MHz, $CDCl_3$) δ 7.12 (d, *J* = 8.6 Hz, 2H), 6.85 (d, *J* = 8.6 Hz, 2H), 5.91 (br. s, 1H), 3.92 – 3.86 (m, 1H), 3.79 (s, 3H), 3.54 (ddd, *J* = 14.0, 6.7, 2.9 Hz, 1H), 3.14 (ddd, *J* = 14.0, 7.9, 5.2 Hz, 1H), 2.75 (dd, *J* = 13.9, 5.2 Hz, 1H), 2.66 (dd, *J* = 13.9, 8.1 Hz, 1H), 2.59 – 2.52 (m, 1H), 2.00 (s, 3H) ppm.
^{13}C NMR (176 MHz, $CDCl_3$) δ 171.2, 158.6, 130.5, 129.5, 114.3, 72.4, 55.4, 45.2, 40.7, 23.4 ppm.
HRMS $[M+H]^+$ m/z calc'd for $[C_{12}H_{17}NO_3H]^+$ expect 224.1287; found 224.1280.

***N*-(2-hydroxy-3-(*p*-tolyl)propyl)acetamide (1s-rac)**

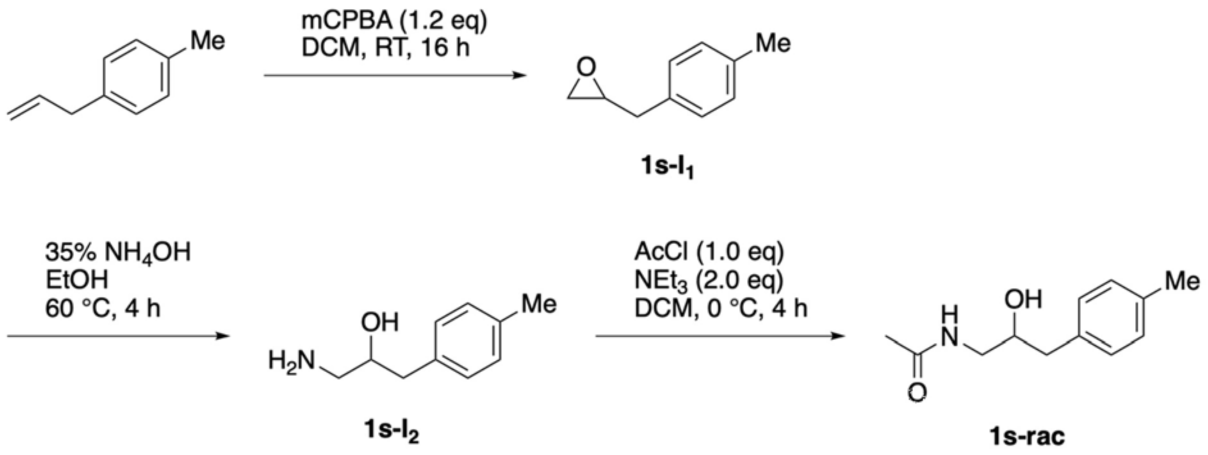

1s-I₁ was prepared according to **General Procedure B** using alkene substrate 1-allyl-4-methylbenzene (0.92 mL, 6.02 mmol) and *m*CPBA (1.61 g, 7.18 mmol) stirred overnight for 16 h. Crude **1s-I₁** was brought forward without additional purification to **General Procedure C** using NH_4OH (11 mL) and ethanol (11 mL) stirred for 4 h. Purification by silica gel column chromatography (elution with 4% 7N ammonia in MeOH, 8% MeOH, 88% DCM) afforded **1s-I₂** as a pale yellow solid (257 mg, 1.55 mmol, 26% yield over two steps).

^{1}H NMR (700 MHz, $CDCl_3$) δ 7.13 – 7.09 (m, 4H), 3.75 – 3.70 (m, 1H), 2.83 (dd, *J* = 12.7, 3.3 Hz, 1H), 2.73 – 2.67 (m, 2H), 2.58 (dd, *J* = 12.7, 8.1 Hz, 1H), 2.32 (s, 3H), 1.88 (br. s, 3H) ppm.
^{13}C NMR (176 MHz, $CDCl_3$) δ 136.1, 135.3, 129.34, 129.33, 73.3, 46.9, 41.1, 21.1 ppm.
HRMS $[M+H]^+$ m/z calc'd for $[C_{10}H_{15}NOH]^+$ expect 166.1232; found 166.1234.

1s-rac was prepared according to **General Procedure D** using amino alcohol substrate **1s-I₂** (247 mg, 1.50 mmol), triethylamine (0.42 mL, 3.01 mmol) and acetyl chloride (0.11 mL, 1.55 mmol). Purification by silica gel column chromatography (elution with 1.5% 7N ammonia in MeOH, 3% MeOH, 95.5% DCM) afforded **1s-rac** as a pale yellow oil that slowly forms a white solid (314 mg, 1.5 mmol, quantitative yield).

^{1}H NMR (700 MHz, $CDCl_3$) δ 7.12 (d, *J* = 7.9 Hz, 2H), 7.09 (d, *J* = 7.9 Hz, 2H), 5.90 (br. s, 1H), 3.94 – 3.89 (m, 1H), 3.55 (ddd, *J* = 14.0, 6.7, 2.9 Hz, 1H), 3.15 (ddd, *J* = 14.0, 7.8, 5.0 Hz, 1H), 2.77 (dd, *J* = 13.7, 5.2 Hz, 1H), 2.68 (dd, *J* = 13.7, 8.1 Hz), 2.57 – 2.50 (m, 1H), 2.32 (s, 3H), 2.00 (s, 3H) ppm.
^{13}C NMR (176 MHz, $CDCl_3$) δ 171.2, 136.5, 134.4, 129.6, 129.4, 72.4, 45.2, 41.2, 23.4, 21.2 ppm.
HRMS $[M+H]^+$ m/z calc'd for $[C_{12}H_{17}NO_2H]^+$ expect 208.1338; found 208.1331.

***N*-(3-(4-fluorophenyl)-2-hydroxypropyl)acetamide (1t-rac)**

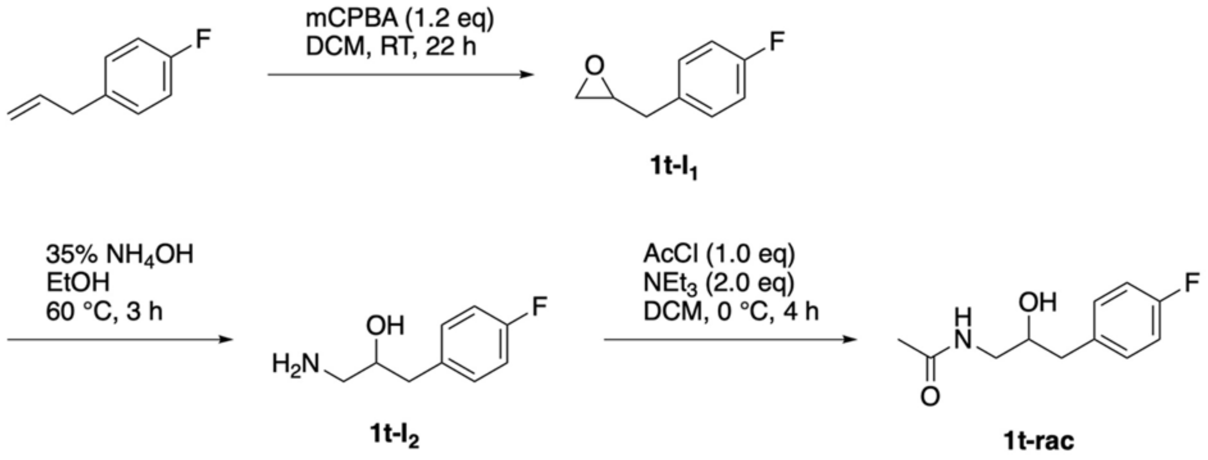

1t-I₁ was prepared according to **General Procedure B** using alkene substrate 1-allyl-4-fluorobenzene (0.81 mL, 6.00 mmol) and *m*CPBA (1.61 g, 7.18 mmol) stirred overnight for 22 h. Purification by silica gel column chromatography (5% acetone in petroleum ether) afforded **1t-I₁** as a colorless oil (836 mg, 5.49 mmol, 92% yield).

^{1}H NMR (400 MHz, $CDCl_3$) δ 7.24 – 7.18 (m, 2H), 7.04 – 6.96 (m, 2H), 3.16 – 3.09 (m, 1H), 2.85 (d, *J* = 5.4 Hz, 2H), 2.79 (dd, *J* = 4.9, 3.9 Hz, 1H), 2.52 (dd, *J* = 4.9, 2.7 Hz, 1H) ppm.
^{19}F NMR (376 MHz, $CDCl_3$) −116.5 ppm.
^{13}C NMR (101 MHz, $CDCl_3$) δ 161.9 (d, *J* = 244.5 Hz), 132.9 (d, *J* = 3.3 Hz), 130.6 (d, *J* = 7.9 Hz), 115.4 (d, *J* = 21.3 Hz), 52.48, 52.47, 46.9, 38.0 ppm.

The data is in good agreement with the literature.[19]

1t-I₂ was prepared according to **General Procedure C** using epoxide substrate **1t-I₁** (457 mg, 3.00 mmol), NH_4OH (11 mL) and ethanol (11 mL) stirred for 3 h. Purification by silica gel column chromatography (elution with 4% 7N ammonia in MeOH, 8% MeOH, 88% DCM) afforded **1t-I₂** as a viscous colorless oil (407 mg, 2.41 mmol, 80% yield).

^{1}H NMR (500 MHz, $CDCl_3$) δ 7.15 (dd, *J* = 8.6, 5.5 Hz, 2H), 6.96 (t, *J* = 8.6 Hz, 2H), 3.70 – 3.63 (m, 1H), 2.75 (dd, *J* = 12.7, 3.2 Hz, 1H), 2.66 (d, *J* = 6.5 Hz, 2H), 2.52 (dd, *J* = 12.7, 8.2 Hz, 1H), 2.12 (br. s, 3H) ppm.
^{19}F NMR (471 MHz, $CDCl_3$) −117.0 ppm.
^{13}C NMR (126 MHz, $CDCl_3$) δ 161.6 (d, *J* = 244.1 Hz), 134.3 (d, *J* = 3.2 Hz), 130.8 (d, *J* = 7.7 Hz), 115.2 (d, *J* = 21.1 Hz), 73.0, 46.9, 40.7 ppm.
HRMS $[M+H]^+$ m/z calc'd for $[C_9H_{12}FNOH]^+$ expect 170.0981; found 170.0974.

1t-rac was prepared according to **General Procedure D** using amino alcohol substrate **1t-I₂** (258 mg, 1.52 mmol), triethylamine (0.43 mL, 3.09 mmol) and acetyl chloride (0.11 mL, 1.55 mmol). Purification by silica gel column chromatography (elution with 1.5% 7N ammonia in MeOH, 3% MeOH, 95.5% DCM) afforded **1t-rac** as a pale yellow cloudy oil (316 mg, 1.50 mmol, 98% yield).

^{1}H NMR (700 MHz, $CDCl_3$) δ 7.17 (dd, *J* = 8.6, 5.4 Hz, 2H), 7.00 (t, *J* = 8.6 Hz, 2H), 5.92 (br. s, 1H), 3.94 – 3.88 (m, 1H), 3.52 (ddd, *J* = 14.0, 6.6, 2.9 Hz, 1H), 3.16 (ddd, *J* = 14.0, 8.0, 5.4 Hz, 1H), 2.77 (dd, *J* = 13.8, 5.1 Hz, 1H), 2.72 – 2.66 (m, 2H), 2.00 (s, 3H) ppm.
^{19}F NMR (376 MHz, $CDCl_3$) −116.3 ppm.
^{13}C NMR (176 MHz, $CDCl_3$) δ 171.4, 161.9 (d, *J* = 244.9 Hz), 133.3 (d, *J* = 3.3 Hz), 130.9 (d, *J* = 7.8 Hz), 115.7 (d, *J* = 21.2 Hz), 72.4 (2C), 45.3, 40.8, 23.4 ppm.
HRMS $[M+H]^+$ m/z calc'd for $[C_{11}H_{14}NO_2FH]^+$ expect 212.1087; found 212.1093.

***N*-(3-(3-chlorophenyl)-2-hydroxypropyl)acetamide (1u-rac)**

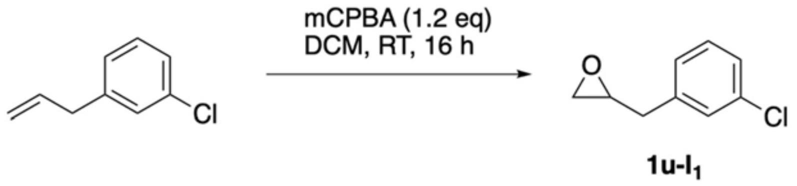

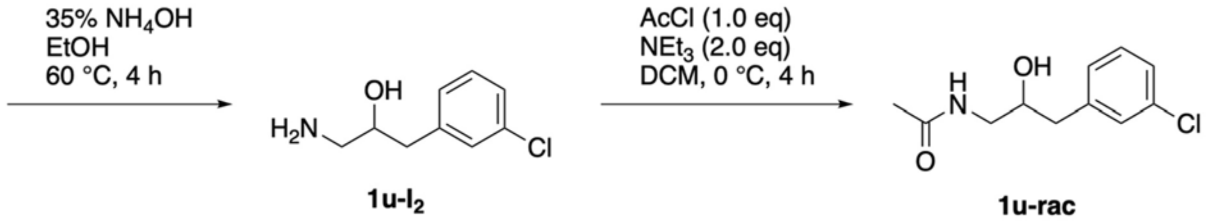

1u-I$_1$ was prepared according to modified literature procedure, performing **General Procedure B** using alkene substrate 1-allyl-3-chlorobenzene (1.09 g, 7.14 mmol) and *m*CPBA (1.92 g, 8.57 mmol) stirred overnight for 16 h.[20] A solution of crude **1u-I$_1$** in ethanol (22 mL) was partitioned equally into two microwave vials. 35% NH_4OH (11 mL) was added to each vial. The vials were sealed, stirred vigorously and heated to 60 °C for 4h. The reaction mixtures were combined, concentrated *in vacuo* and purified by silica gel column chromatography (elution with 4% 7N ammonia in MeOH, 8% MeOH, 88% DCM) to yield **1u-I$_2$** as a yellow oil (805 mg, 4.34 mmol, 61% over two steps).

^{1}H NMR (700 MHz, $CDCl_3$) δ 7.24 – 7.18 (m, 3H), 7.11 (d, *J* = 7.2 Hz, 1H), 3.76 – 3.70 (m, 1H), 2.84 (dd, *J* = 12.7, 3.4 Hz, 1H), 2.75 – 2.68 (m, 2H), 2.56 (dd, *J* = 12.7, 8.2 Hz, 1H), 1.82 (br. s, 3H) ppm.
^{13}C NMR (176 MHz, $CDCl_3$) δ 140.7, 134.3, 129.8, 129.5, 127.7, 126.7, 72.8, 46.9, 41.1 ppm.
HRMS $[M+H]^+$ m/z calc'd for $[C_9H_{12}ClNOH]^+$ expect 186.0686; found 186.0686.

1u-rac was prepared according to **General Procedure D** using amino alcohol substrate **1u-I$_2$** (371 mg, 2.00 mmol), triethylamine (0.56 mL, 4.02 mmol) and acetyl chloride (0.14 mL, 1.97 mmol). Purification by silica gel column chromatography (elution with 5% MeOH in DCM) afforded **1u-rac** as a pale yellow oil (460 mg, 2.0 mmol, quantitative yield).

^{1}H NMR (700 MHz, $CDCl_3$) δ 7.25 – 7.19 (m, 3H), 7.09 (d, *J* = 7.0 Hz, 1H), 6.02 (br. s, 1H), 3.97 – 3.90 (m, 1H), 3.50 (ddd, *J* = 14.0, 6.5, 2.8 Hz, 1H), 3.17 (ddd, *J* = 14.0, 7.9, 5.6 Hz, 1H), 2.76 (dd, *J* = 13.8, 5.1 Hz, 1H), 2.70 (dd, *J* = 13.8, 8.0 Hz, 1H), 2.49 (br. s, 1H), 2.01 (s, 3H) ppm.

^{13}C NMR** (176 MHz, $CDCl_3$) δ 171.6, 139.9, 134.5, 130.0, 129.6, 127.7, 127.0, 72.1, 45.4, 41.2, 23.3 ppm.
HRMS $[M+H]^+$ m/z calc'd for $[C_{11}H_{14}ClNO_2H]^+$ expect 228.0791; found 228.0802.

***N*-(2-hydroxy-3-(naphthalen-1-yl)propyl)acetamide (1v-rac)**

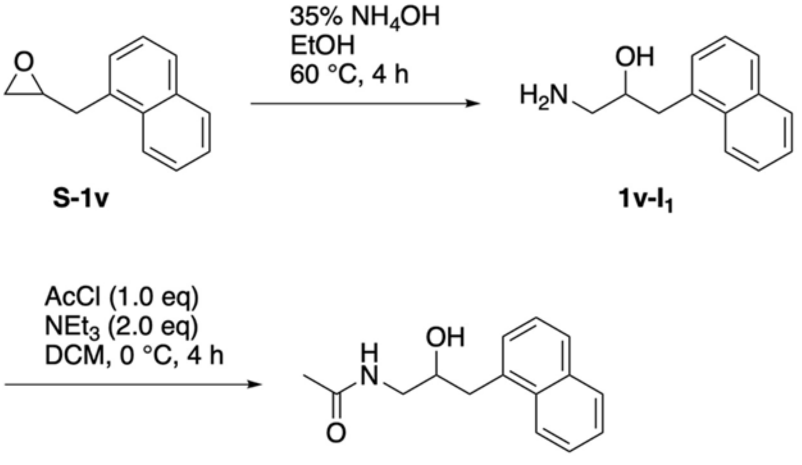

S-1v **1v-I$_1$** **1v-rac**

1v-I$_1$ was prepared according to **General Procedure C** using epoxide substrate **S-1v** (461 mg, 2.50 mmol; prepared according to literature procedure[21]), NH_4OH (9 mL) and ethanol (9 mL) stirred for 4 h. Purification by silica gel column chromatography (elution with 3% 7N ammonia in MeOH, 6% MeOH, 91% DCM) afforded **1v-I$_1$** as a white solid (405 mg, 2.01 mmol, 81% yield).

^{1}H NMR (400 MHz, $CDCl_3$) δ 8.05 (d, *J* = 8.1 Hz, 1H), 7.89 – 7.83 (m, 1H), 7.75 (d, *J* = 8.1 Hz, 1H), 7.56 – 7.45 (m, 2H), 7.40 (t, *J* = 7.5 Hz, 1H), 7.35 (d, *J* = 7.3 Hz, 1H), 3.97 – 3.84 (m, 1H), 3.18 (d, *J* = 6.5 Hz, 2H), 2.90 – 2.74 (m, 1H), 2.71 – 2.55 (m, 1H), 1.87 (br. s, 3H) ppm.
^{13}C NMR (101 MHz, $CDCl_3$) δ 134.6, 134.1, 132.3, 128.9, 127.7, 127.4, 126.1, 125.7, 125.6, 124.0, 72.5, 47.3, 38.6 ppm.
HRMS $[M+H]^+$ m/z calc'd for $[C_{13}H_{15}NOH]^+$ expect 202.1232; found 202.1237.

1v-rac was prepared according to **General Procedure D** using amino alcohol substrate **1v-I$_2$** (362 mg, 1.80 mmol), triethylamine (0.50 mL, 3.59 mmol) and acetyl chloride (0.13 mL, 1.83 mmol). Purification by silica gel column chromatography (elution with 1.5% 7N ammonia in MeOH, 3% MeOH, 95.5% DCM) afforded **1v-rac** as a white solid (402 mg, 1.65 mmol, 92% yield).

^{1}H NMR (500 MHz, $CDCl_3$) δ 8.02 (d, *J* = 8.3 Hz, 1H), 7.88 – 7.83 (m, 1H), 7.75 (d, *J* = 8.2 Hz, 1H), 7.55 – 7.46 (m, 2H), 7.40 (t, *J* = 7.6 Hz, 1H), 7.34 (d, *J* = 7.1 Hz, 1H), 6.05 (br. s, 1H), 4.13 – 4.06 (m, 1H), 3.55 (ddd, *J* = 14.0, 6.6, 2.9 Hz, 1H), 3.29 – 3.21 (m, 2H), 3.17 (dd, *J* = 14.0, 7.9 Hz, 1H), 2.59 (br. s, 1H), 1.96 (s, 3H) ppm.
^{13}C NMR (126 MHz, $CDCl_3$) δ 171.5, 134.1, 133.8, 132.2, 129.0, 127.8, 127.7, 126.3, 125.9, 125.6, 123.8, 71.6, 45.6, 38.7, 23.3 ppm.
HRMS $[M+H]^+$ m/z calc'd for $[C_{15}H_{17}NO_2H]^+$ expect 244.1338; found 244.1329.

***N*-(2-hydroxy-3-(thiophen-3-yl)propyl)acetamide (1w-rac)**

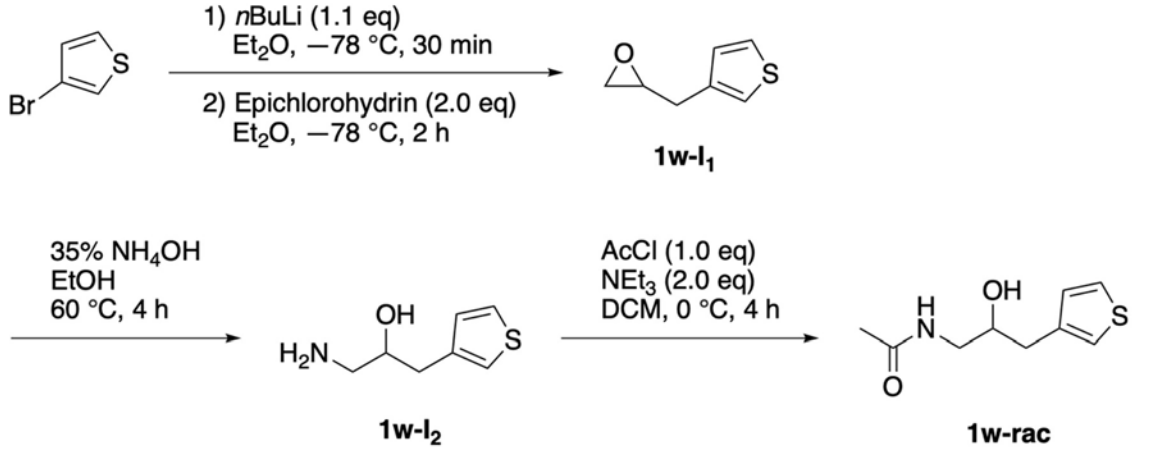

Crude **1w-I$_1$** was prepared according to modified literature procedure.[22] *n*BuLi (1.6 M in hexanes, 3.4 mL, 5.4 mmol) was added slowly to a solution of 3-bromothiophene (0.47 mL, 5.02 mmol) stirring in dry diethyl ether (20 mL) in an oven-dried two-neck flask under nitrogen atmosphere at –78 °C. The solution was stirred at –78 °C for 30 min. A solution of epichlorohydrin (0.78 mL, 9.96 mmol) in dry diethyl ether (10 mL) in a separate oven-dried 20 mL vial under nitrogen atmosphere was added slowly to the main reaction mixture, which was stirred at –78 °C for a further 1 h. A color change from light orange to colorless is observed. The reaction mixture is slowly warmed to RT (approx. 1 h), quenched with saturated aqueous ammonium chloride solution, diluted with H_2O and extracted with diethyl ether. The combined organic extracts were dried over $MgSO_4$, filtered, and the solvent removed *in vacuo* to afford crude mixture as an orange oil which was brought forward without further purification. **1w-I$_2$** was prepared according to **General Procedure C** using crude **1w-I$_1$**, NH_4OH (11 mL) and ethanol (11 mL) stirred for 4 h. Purification by silica gel column chromatography (elution with 3% 7N ammonia in MeOH, 6% MeOH, 91% DCM) afforded **1w-I$_2$** as an orange oil (106 mg, 0.672 mmol, 13% yield over two steps).

^{1}H NMR (700 MHz, $CDCl_3$) δ 7.27 (dd, *J* = 4.8, 2.6 Hz, 1H), 7.04 (d, *J* = 2.6 Hz, 1H), 6.98 (d, *J* = 4.8 Hz, 1H), 3.83 – 3.74 (m, 1H), 2.89 (dd, *J* = 12.7, 3.2 Hz, 1H), 2.78 (d, *J* = 6.5 Hz, 2H), 2.60 (dd, *J* = 12.7, 8.2 Hz, 1H), 2.23 (br. s, 3H) (overlaps with H_2O peak) ppm.
^{13}C NMR (176 MHz, $CDCl_3$) δ 138.6, 128.8, 125.9, 122.0, 72.3, 46.9, 35.8 ppm.
HRMS $[M+H]^+$ m/z calc'd for $[C_7H_{11}NOSH]^+$ expect 158.0640; found 158.0636.

1w-rac was prepared according to **General Procedure D** using amino alcohol substrate **1w-I$_2$** (99.0 mg, 0.63 mmol), triethylamine (0.18 mL, 1.29 mmol) and acetyl chloride (0.045 mL, 0.633 mmol). Purification by silica gel column chromatography (elution with 4% MeOH in DCM) afforded **1w-rac** as a viscous orange oil (104 mg, 0.523 mmol, 83% yield).

^{1}H NMR (700 MHz, $CDCl_3$) δ 7.30 (dd, *J* = 4.8, 2.7 Hz, 1H), 7.06 (d, *J* = 2.7 Hz, 1H), 6.98 (d, *J* = 4.8 Hz, 1H), 5.95 (br. s, 1H), 3.98 – 3.93 (m, 1H), 3.54 (ddd, *J* = 14.0, 6.6, 2.9 Hz, 1H), 3.17 (ddd, *J* = 14.0, 7.9, 5.4 Hz, 1H), 2.85 (dd, *J* = 14.3, 5.1 Hz, 1H), 2.78 (dd, *J* = 14.3, 7.9 Hz, 1H), 2.20 (br. s, 1H), 2.02 (s, 3H) ppm.
^{13}C NMR (176 MHz, $CDCl_3$) δ 171.4, 137.8, 128.6, 126.4, 122.5, 71.6, 45.3, 36.0, 23.3 ppm.

HRMS [M+H]+ m/z calc'd for $[C_9H_{13}NO_2SH]^+$ expect 200.0745; found 200.0739.

***N*-(2-hydroxy-4,4-dimethylpentyl)acetamide (1x-rac)**

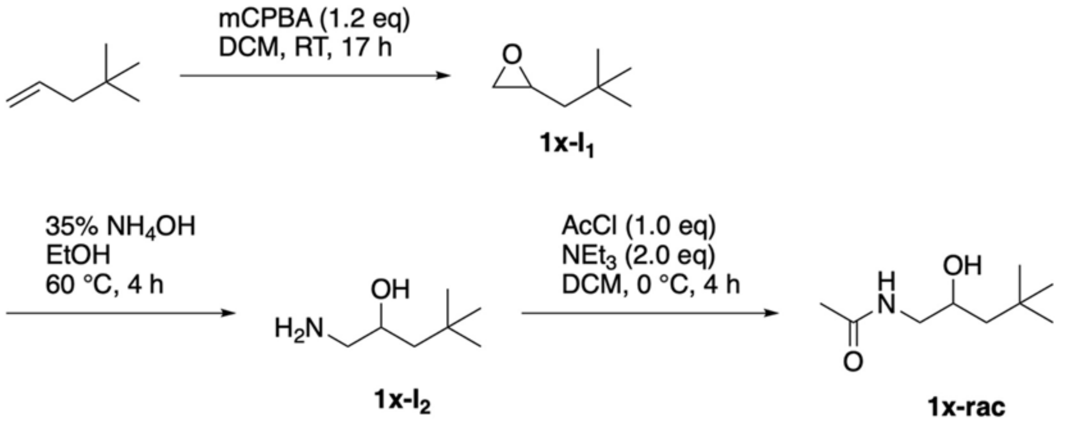

1x-I$_1$ was prepared according to **General Procedure B** using alkene substrate 4,4-dimethyl-1-pentene (0.86 mL, 5.98 mmol) and *m*CPBA (1.61 g, 7.18 mmol) stirred overnight for 17 h. Crude **1x-I$_1$** was brought forward without additional purification to **General Procedure C** using NH_4OH (11 mL) and ethanol (11 mL) stirred for 4 h. Purification by silica gel column chromatography (elution with 4% 7N ammonia in MeOH, 8% MeOH, 88% DCM) afforded **1x-I$_2$** as a yellow solid (289 mg, 2.20 mmol, 37% yield over two steps).

^{1}H NMR (700 MHz, $CDCl_3$) δ 3.65 – 3.59 (m, 1H), 2.76 (dd, *J* = 12.5, 3.1 Hz, 1H), 2.47 (dd, *J* = 12.5, 8.9 Hz, 1H), 2.11 (br. s, 3H), 1.35 (dd, *J* = 14.4, 8.1 Hz, 1H), 1.23 (dd, *J* = 14.4, 2.6 Hz, 1H), 0.96 (s, 9H) ppm.
^{13}C NMR (176 MHz, $CDCl_3$) δ 69.5, 49.0, 48.6, 30.3, 30.2 ppm.
HRMS [M+H]+ m/z calc'd for $[C_7H_{17}NOH]^+$ expect 132.1388; found 132.1384.

1x-rac was prepared according to **General Procedure D** using amino alcohol substrate **1x-I$_2$** (144 mg, 1.10 mmol), triethylamine (0.31 mL, 2.22 mmol) and acetyl chloride (0.08 mL, 1.13 mmol). Purification by silica gel column chromatography (elution with 1.5% 7N ammonia in MeOH, 3% MeOH, 95.5% DCM) afforded **1x-rac** as a white solid (159 mg, 0.92 mmol, 84% yield).

^{1}H NMR (700 MHz, $CDCl_3$) δ 6.06 (br. s, 1H), 3.87 – 3.81 (m, 1H), 3.41 (ddd, *J* = 13.8, 6.6, 3.2 Hz, 1H), 3.07 (ddd, *J* = 13.8, 8.3, 5.2 Hz, 1H), 2.40 (d, *J* = 4.7 Hz, 1H), 2.00 (s, 3H), 1.37 (dd, *J* = 14.5, 7.8 Hz, 1H), 1.33 (dd, *J* = 14.5, 3.0 Hz, 1H), 0.96 (s, 9H) ppm.
^{13}C NMR (176 MHz, $CDCl_3$) δ 171.2, 69.1, 48.8, 47.5, 30.3, 30.2, 23.4 ppm.
HRMS [M+H]+ m/z calc'd for $[C_9H_{19}NO_2H]^+$ expect 174.1494; found 174.1495.

***N*-(2,5-dihydroxypentyl)acetamide (1y-rac)**

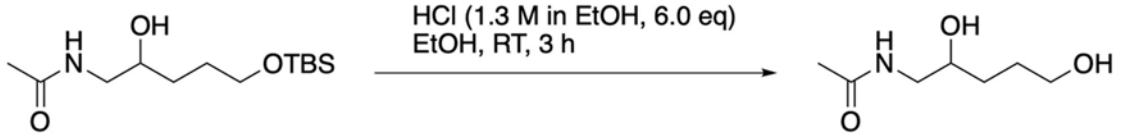

1n-rac **1y-rac**

Hydrochloric acid (1.3 M in ethanol, 4.62 mL, 6.01 mmol) was added slowly to a stirred solution of **1n-rac** (275 mg, 1.00 mmol) in ethanol (10 mL). The reaction mixture was stirred at RT for 3 h, concentrated *in vacuo* and purified by silica gel column chromatography (elution with 15% MeOH in DCM) to afford **1y-rac** as a pale yellow oil (143 mg, 0.884 mmol, 88% yield).

^{1}H NMR (700 MHz, CD_3OD) δ 3.67 – 3.61 (m, 1H), 3.57 (t, *J* = 6.4 Hz, 2H), 3.28 (dd, *J* = 13.6, 4.4 Hz, 1H), 3.09 (dd, *J* = 13.6, 7.2 Hz, 1H), 1.96 (s, 3H), 1.73 – 1.66 (m, 1H), 1.63 – 1.53 (m, 2H), 1.45 – 1.38 (m, 1H) ppm.
^{13}C NMR (176 MHz, CD_3OD) δ 173.6, 71.2, 62.9, 46.7, 32.2, 29.7, 22.5 ppm.
HRMS $[M+H]^+$ m/z calc'd for $[C_7H_{15}NO_3H]^+$ expect 162.1130; found 162.1124.

N-Acyl Amino Alcohol and Urea Substrates

General Procedures

Other *N*-acyl amino alcohols were synthesized according to General Procedure E and General Procedure F.

General Procedure E: *N*-acyl amino alcohol formation *via* acyl chloride addition to 1-amino-2-butanol

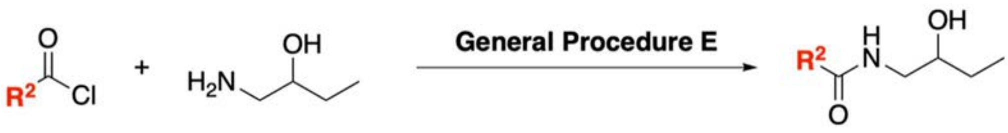

Triethylamine (2.0 eq.) was added to a stirred solution of 1-amino-2-butanol (1.0 eq.) in dry DCM (0.2 M). The solution was cooled to 0 °C and acyl chloride substrate (1.0 eq.) added slowly. The reaction mixture was stirred for 4 h at 0 °C, concentrated *in vacuo* and purified by silica gel column chromatography to afford the *N*-acyl amino alcohol product.

General Procedure F: amidated amino alcohol formation *via* amide coupling with 1-amino-2-butanol

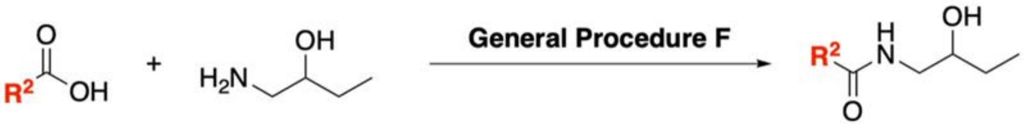

DIPEA (2.5 eq.) was added to a stirred suspension of substrate carboxylic acid (1.0 eq.), 1-amino-2-butanol (1.0 eq.), *N*-(3-Dimethylaminopropyl)-*N'*-ethylcarbodiimide hydrochloride (EDC.HCl) (1.1 eq.) and 1-hydroxybenzotriazole monohydrate (HOBt.H_2O) (≥20 wt% H_2O, 1.1 eq.) in DMF or DCM (0.4 M). The reaction mixture was stirred overnight. With DMF as solvent, water was added and the crude extracted with ethyl acetate. With DCM as solvent, the reaction mixture was concentrated *in vacuo*, water was added and the crude extracted with ethyl acetate. The combined organic extracts were dried over $MgSO_4$, filtered, and the solvent removed *in vacuo* to afford crude mixture. The crude mixture was purified by silica gel column chromatography to afford the *N*-acyl amino alcohol product.

Characterization Data

N-(2-hydroxybutyl)isobutyramide (1z-rac)

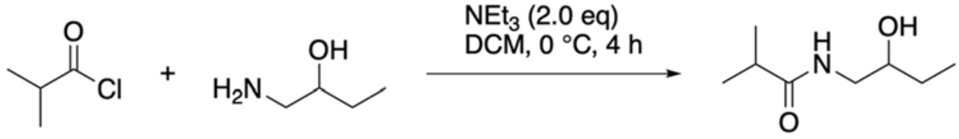

1z-rac

1z-rac was prepared according to **General Procedure E** using isobutyryl chloride (0.21 mL, 2.00 mmol), 1-amino-2-butanol (178 mg, 2.00 mmol) and triethylamine (0.56 mL, 4.02 mmol). Purification by silica gel column chromatography (elution with 2% 7N ammonia in MeOH, 4% MeOH, 94% DCM) afforded **1z-rac** as a white solid (269 mg, 1.69 mmol, 85% yield).

^{1}H NMR (700 MHz, $CDCl_3$) δ 6.04 (br. s, 1H), 3.65 – 3.58 (m, 1H), 3.47 (ddd, *J* = 14.0, 6.4, 2.9 Hz, 1H), 3.13 (ddd, *J* = 14.0, 7.7, 5.1 Hz, 1H), 2.77 (br. s, 1H), 2.38 (hept, *J* = 6.9 Hz, 1H), 1.54 – 1.42 (m, 2H), 1.17 (d, *J* = 6.9 Hz, 6H), 0.95 (t, *J* = 7.5 Hz, 3H) ppm.
^{13}C NMR (176 MHz, $CDCl_3$) δ 178.4, 73.1 (2C), 45.4, 35.7, 28.1, 19.8 (2C), 10.0 ppm.
HRMS $[M+H]^+$ m/z calc'd for $[C_8H_{17}NO_2H]^+$ expect 160.1338; found 160.1336.

(3,5,7)-*N*-(2-hydroxybutyl)adamantane-1-carboxamide (1za-rac)

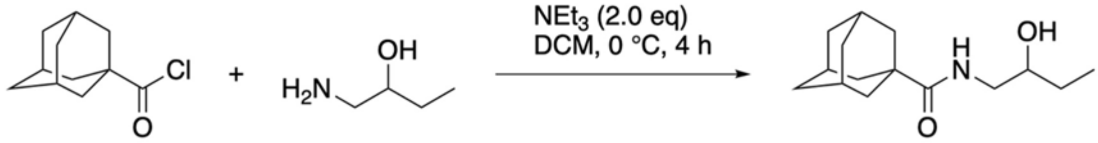

1za-rac

1za-rac was prepared according to **General Procedure E** using acyl chloride substrate 1-adematanecarbonyl chloride (397 mg, 2.00 mmol), 1-amino-2-butanol (178 mg, 2.00 mmol) and triethylamine (0.56 mL, 4.02 mmol). Purification by silica gel column chromatography (elution with 4% MeOH in DCM) afforded **1za-rac** as a white solid (490 mg, 1.95 mmol, 98% yield). Observed as two distinct rotamers.

^{1}H NMR (700 MHz, $CDCl_3$) δ 6.13 (br. s, 1H), 3.63 – 3.58 (m, 1H), 3.45 (ddd, *J* = 14.0, 6.3, 2.9 Hz, 1H), 3.14 (ddd, *J* = 14.0, 7.6, 5.2 Hz, 1H), 2.05 – 1.99 (m, 3.4 H), 1.91 – 1.89 (m, 0.7H), 1.87 – 1.82 (m, 6H), 1.76 – 1.71 (m, 3.4H), 1.71 – 1.66 (m, 3.5H), 1.50 – 1.42 (m, 2H), 0.94 (t, *J* = 7.5 Hz, 3H) ppm.
^{13}C NMR (176 MHz, $CDCl_3$) δ 181.4, 179.5, 73.1, 45.3, 40.8, 39.4, 38.8, 36.61, 36.59, 28.2, 28.04, 28.00, 10.0 ppm.
HRMS $[M+H]^+$ m/z calc'd for $[C_{15}H_{25}NO_2H]^+$ expect 252.1964; found 252.1962.

3-cyano-*N*-(2-hydroxybutyl)propanamide (1zb-rac)

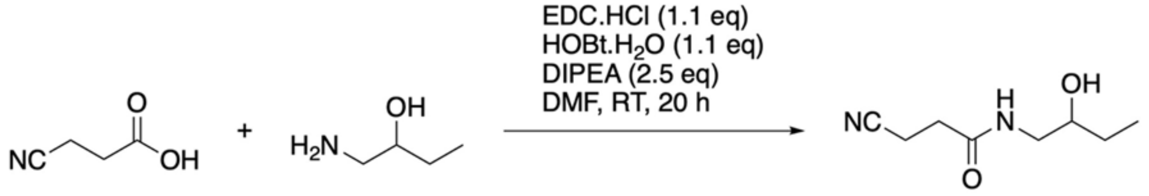

1zb-rac

1zb-rac was prepared according to **General Procedure F** using carboxylic acid substrate 3-cyanopropanoic acid (198 mg, 2.00 mmol), 1-amino-2-butanol (178 mg, 2.00 mmol), EDC.HCl (422 mg, 2.20 mmol), HOBt.H_2O (421 mg, 2.20 mmol) and DIPEA (0.86 mL, 4.94 mmol) stirred overnight for 20 h in DMF. Purification by silica gel column chromatography (elution with 5% MeOH in DCM) afforded **1zb-rac** as a pale yellow oil (72.4 mg, 0.43 mmol, 21% yield).

^{1}H NMR (700 MHz, $CDCl_3$) δ 6.09 (br. s, 1H), 3.68 – 3.62 (m, 1H), 3.55 (ddd, *J* = 13.9, 6.6, 3.0 Hz, 1H), 3.11 (ddd, *J* = 13.9, 8.1, 4.9 Hz, 1H), 2.74 – 2.66 (m, 2H), 2.57 (t, *J* = 7.2 Hz, 2H), 1.99 (br. s, 1H) (overlaps with H_2O peak), 1.56 – 1.44 (m, 2H), 0.97 (t, *J* = 7.5 Hz, 3H) ppm.
^{13}C NMR (176 MHz, $CDCl_3$) δ 169.5, 119.2, 72.6, 45.4, 31.8, 28.1, 13.5, 9.9 ppm.
HRMS $[M+H]^+$ m/z calc'd for $[C_8H_{14}N_2O_2H]^+$ expect 171.1134; found 171.1130.

***N*-(2-hydroxybutyl)-2-(phenylsulfonyl)acetamide (1zc-rac)**

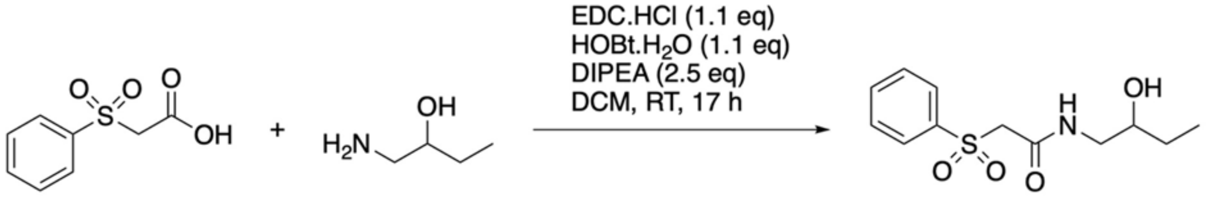

1zc-rac

1zc-rac was prepared according to **General Procedure F** using carboxylic acid substrate (phenyl sulfonyl) acetic acid (400 mg, 2.00 mmol), 1-amino-2-butanol (178 mg, 2.00 mmol), EDC.HCl (422 mg, 2.20 mmol), HOBt.H_2O (421 mg, 2.20 mmol) and DIPEA (0.86 mL, 4.94 mmol) stirred overnight for 17 h in DCM. Purification by silica gel column chromatography (elution with 4% MeOH in DCM) afforded **1zc-rac** as an off-white solid (367 mg, 1.35 mmol, 68% yield).

^{1}H NMR (700 MHz, $CDCl_3$) δ 7.94 (d, *J* = 8.4 Hz, 2H), 7.69 (t, *J* = 7.5 Hz, 1H), 7.59 (t, *J* = 7.9 Hz, 2H), 7.12 (br. s, 1H), 4.06 (s, 2H), 3.69 – 3.63 (m, 1H), 3.51 (ddd, *J* = 13.9, 6.5, 3.1 Hz, 1H), 3.14 (ddd, *J* = 13.9, 8.1, 5.4 Hz, 1H), 2.55 (br. d, *J* = 4.4 Hz, 1H), 1.55 – 1.44 (m, 2H), 0.96 (t, *J* = 7.5 Hz, 3H) ppm.
^{13}C NMR (176 MHz, $CDCl_3$) δ 161.4, 138.3, 134.6, 129.6, 128.4, 72.2, 62.2, 45.8, 27.9, 9.9 ppm.
HRMS $[M+H]^+$ m/z calc'd for $[C_{12}H_{17}NO_4SH]^+$ expect 272.0957; found 272.0951.

N-(2-hydroxybutyl)-3-phenylpropanamide (1zd-rac)

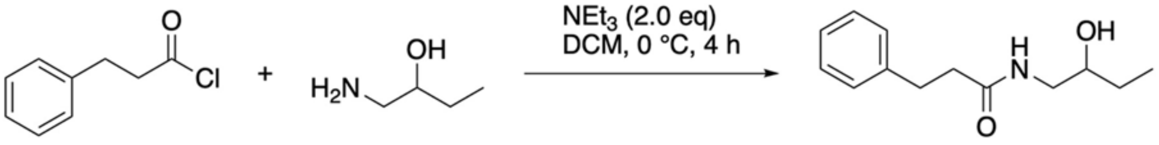

1zd-rac

1zd-rac was prepared according to **General Procedure E** using acyl chloride substrate hydrocinnamoyl chloride (0.30 mL, 2.02 mmol), 1-amino-2-butanol (178 mg, 2.00 mmol) and triethylamine (0.56 mL, 4.02 mmol). Purification by silica gel column chromatography (elution with 1.5% 7N ammonia in MeOH, 3% MeOH, 95.5% DCM) afforded **1zd-rac** as a white solid (379 mg, 1.71 mmol, 86% yield).

^{1}H NMR (700 MHz, $CDCl_3$) δ 7.28 (t, *J* = 7.6 Hz, 2H), 7.23 – 7.17 (m, 3H), 5.91 (br. s, 1H), 3.55 – 3.49 (m, 1H), 3.43 (ddd, *J* = 13.9, 6.5, 2.9 Hz, 1H), 3.04 (ddd, *J* = 13.9, 8.0, 5.4 Hz, 1H), 2.96 (t, *J* = 7.6 Hz, 2H), 2.55 – 2.43 (m, 3H), 1.45 – 1.35 (m, 2H), 0.92 (t, *J* = 7.5 Hz, 3H) ppm.
^{13}C NMR (176 MHz, $CDCl_3$) δ 173.3, 140.8, 128.7, 128.5, 126.4, 72.8, 45.4, 38.6, 31.9, 27.9, 9.9 ppm.
HRMS $[M+H]^+$ m/z calc'd for $[C_{13}H_{19}NO_2H]^+$ expect 222.1494; found 222.1497.

N-(2-hydroxybutyl)benzamide (1ze-rac)

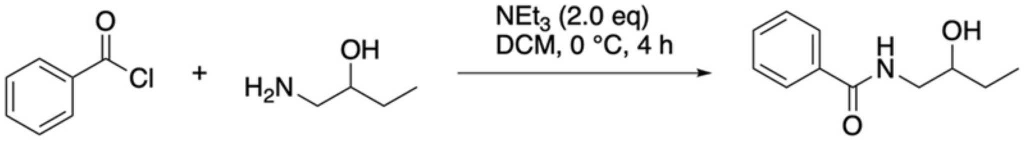

1ze-rac

1ze-rac was prepared according to **General Procedure E** using acyl chloride substrate benzoyl chloride (0.23 mL, 1.98 mmol), 1-amino-2-butanol (178 mg, 2.00 mmol) and triethylamine (0.56 mL, 4.02 mmol). Purification by silica gel column chromatography (elution with 3% MeOH in DCM) afforded **1ze-rac** as a white solid (366 mg, 1.89 mmol, 95% yield).

^{1}H NMR (700 MHz, $CDCl_3$) δ 7.77 (d, *J* = 8.5 Hz, 2H), 7.49 (t, *J* = 7.4 Hz, 1H), 7.41 (t, *J* = 7.7 Hz, 2H), 6.73 (br. s, 1H), 3.77 – 3.72 (m, 1H), 3.69 (ddd, *J* = 13.9, 6.5, 3.0 Hz, 1H), 3.31 (ddd, *J* = 13.9, 7.8, 5.1 Hz, 1H), 2.86 – 2.76 (m, 1H), 1.60 – 1.48 (m, 2H), 0.98 (t, *J* = 7.5 Hz, 3H) ppm.
^{13}C NMR (176 MHz, $CDCl_3$) δ 168.6, 134.4, 131.7, 128.7, 127.1, 72.8, 45.9, 28.1, 10.0 ppm.

The data is in good agreement with the literature.[23]

4-(*tert*-butyl)-*N*-(2-hydroxybutyl)benzamide (1zf-rac)

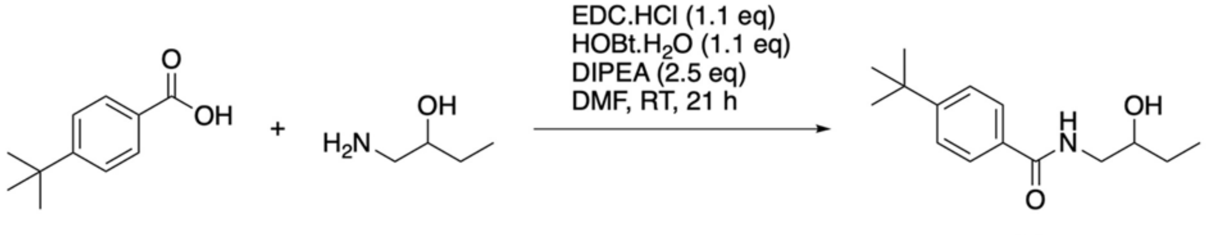

1zf-rac

1zf-rac was prepared according to **General Procedure F** using carboxylic acid substrate 4-*tert*-butylbenzoic acid (357 mg, 2.00 mmol), 1-amino-2-butanol (178 mg, 2.00 mmol), EDC.HCl (422 mg, 2.20 mmol), HOBt.H_2O (421 mg, 2.20 mmol) and DIPEA (0.86 mL, 4.94 mmol) stirred overnight for 21 h in DMF. Purification by silica gel column chromatography (elution with 2% MeOH in DCM) afforded **1zf-rac** as a white solid (321 mg, 1.29 mmol, 64% yield).

^{1}H NMR (700 MHz, $CDCl_3$) δ 7.72 (d, *J* = 8.3 Hz, 2H), 7.43 (d, *J* = 8.3 Hz, 2H), 6.67 (br. s, 1H), 3.77 – 3.71 (m, 1 H), 3.67 (ddd, *J* = 13.9, 6.5, 2.9 Hz, 1H), 3.32 (ddd, *J* = 13.9, 7.9, 5.3 Hz, 1H), 2.80 (br. s, 1H), 1.59 – 1.48 (m, 2H), 1.32 (s, 9H), 0.98 (t, *J* = 7.5 Hz, 3H) ppm.
^{13}C NMR (176 MHz, $CDCl_3$) δ 168.5, 155.3, 131.5, 127.0, 125.6, 73.0, 45.9, 35.1, 31.3, 28.2, 10.0 ppm.
HRMS $[M+H]^+$ m/z calc'd for $[C_{15}H_{23}NO_2H]^+$ expect 250.1807; found 250.1811.

***N*-(2-hydroxybutyl)-4-(trifluoromethyl)benzamide (1zg-rac)**

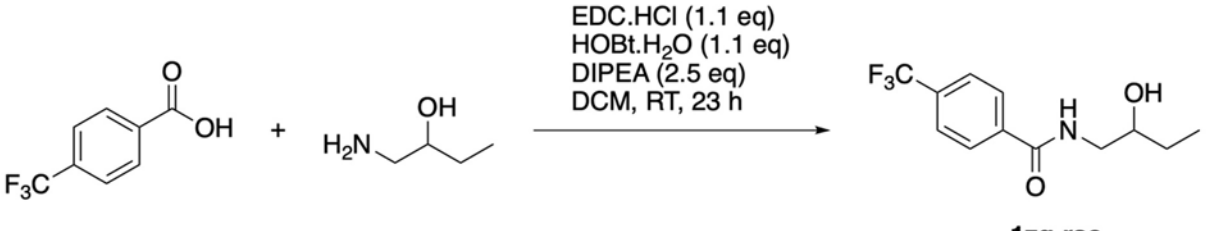

1zg-rac

1zg-rac was prepared according to **General Procedure F** using carboxylic acid substrate 4-(trifluoromethyl)benzoic acid (380 mg, 2.00 mmol), 1-amino-2-butanol (178 mg, 2.00 mmol), EDC.HCl (422 mg, 2.20 mmol), HOBt.H_2O (421 mg, 2.20 mmol) and DIPEA (0.86 mL, 4.94 mmol) stirred overnight for 23 h in DCM. Purification by silica gel column chromatography (elution with 4% MeOH in DCM) afforded **1zg-rac** as a white solid (349 mg, 1.34 mmol, 67% yield).

^{1}H NMR (700 MHz, $(CD_3)_2CO$) δ 8.10 (d, *J* = 8.1 Hz, 2H), 7.93 (br. s, 1H), 7.81 (d, *J* = 8.1 Hz, 2H), 4.06 (d, *J* = 5.0 Hz, 1H), 3.73 – 3.66 (m, 1H), 3.55 (ddd, *J* = 13.5, 6.1, 4.0 Hz, 1H), 3.32 (ddd, *J* = 13.5, 7.5, 5.7 Hz, 1H), 1.59 – 1.51 (m, 1H), 1.49 – 1.40 (m, 1H), 0.96 (t, *J* = 7.5 Hz, 3H) ppm.
^{19}F NMR (376 MHz, $(CD_3)_2CO$) –63.4 ppm.
^{13}C NMR (176 MHz, $(CD_3)_2CO$) δ 166.7, 139.6 (q, *J* = 1.2 Hz), 132.9 (q, *J* = 32.2 Hz), 128.9, 126.2 (q, *J* = 3.8 Hz), 125.1 (q, *J* = 271.6 Hz), 72.4, 46.9, 28.6, 10.3 ppm.

HRMS [M+H]+ m/z calc'd for $[C_{12}H_{14}F_3NO_2H]^+$ expect 262.1055; found 262.1068.

***N*-(2-hydroxybutyl)-4-methoxybenzamide (1zh-rac)**

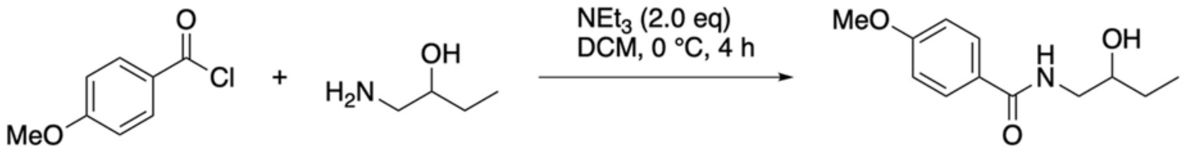

1zh-rac

1zh-rac was prepared according to **General Procedure E** using acyl chloride substrate *p*-anisoyl chloride (0.27 mL, 1.99 mmol), 1-amino-2-butanol (178 mg, 2.00 mmol) and triethylamine (0.56 mL, 4.02 mmol). Purification by silica gel column chromatography (elution with 3% MeOH in DCM) afforded **1zh-rac** as a white solid (376 mg, 1.68 mmol, 84% yield).

^{1}H NMR (700 MHz, $CDCl_3$) δ 7.74 (d, *J* = 8.9 Hz, 2H), 6.89 (d, *J* = 8.9 Hz, 2H), 6.70 (br. s, 1H), 3.83 (s, 3H), 3.75 – 3.70 (m, 1H), 3.66 (ddd, *J* = 13.9, 6.1, 2.9 Hz, 1H), 3.30, (ddd, *J* = 13.9, 7.9, 4.8 Hz, 1H), 2.77 (br. s, 1H), 1.58 – 1.48 (m, 2H), 0.97 (t, *J* = 7.5 Hz, 3H) ppm.
^{13}C NMR (176 MHz, $CDCl_3$) δ 168.2, 162.4, 129.0, 126.5, 113.9, 73.0, 55.5, 45.9, 28.1, 10.0 ppm.
HRMS [M+H]+ m/z calc'd for $[C_{12}H_{17}NO_3H]^+$ expect 224.1287; found 224.1283.

***N*-(2-hydroxybutyl)benzo[*d*][1,3]dioxole-5-carboxamide (1zi-rac)**

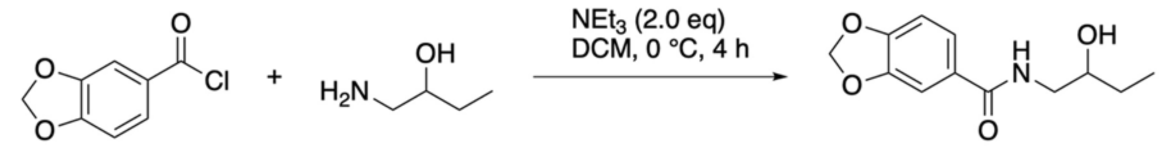

1zi-rac

1zi-rac was prepared according to **General Procedure E** using acyl chloride substrate piperonyloyl chloride (228 mg, 1.24 mmol), 1-amino-2-butanol (111 mg, 1.25 mmol) and triethylamine (0.35 mL, 2.51 mmol). Purification by silica gel column chromatography (elution with 1.5% 7N ammonia in MeOH, 3% MeOH, 95.5% DCM) afforded **1zi-rac** as a white solid (166 mg, 0.70 mmol, 56% yield).

^{1}H NMR (700 MHz, $CDCl_3$) δ 7.31 (dd, *J* = 8.1, 1.7 Hz, 1H), 7.27 (d, *J* = 1.7 Hz, 1H) (overlaps with $CHCl_3$ peak), 6.81 (d, *J* = 8.1 Hz, 1H), 6.58 (br. s, 1H), 6.01 (s, 2H), 3.76 – 3.70 (m, 1H), 3.67 (ddd, *J* = 13.8, 5.8, 2.7 Hz, 1H), 3.29 (ddd, *J* = 13.8, 8.1, 4.8 Hz, 1H), 2.37 (br. s, 1H), 1.59 – 1.48 (m, 2H), 0.99 (t, *J* = 7.5 Hz, 3H) ppm.
^{13}C NMR (176 MHz, $CDCl_3$) δ 167.8, 150.6, 148.1, 128.5, 121.8, 108.1, 107.8, 101.8, 73.0, 45.9, 28.2, 10.0 ppm.
HRMS [M+H]+ m/z calc'd for $[C_{12}H_{15}NO_4H]^+$ expect 238.1079; found 238.1083.

***N*-(2-hydroxybutyl)furan-3-carboxamide (1zj-rac)**

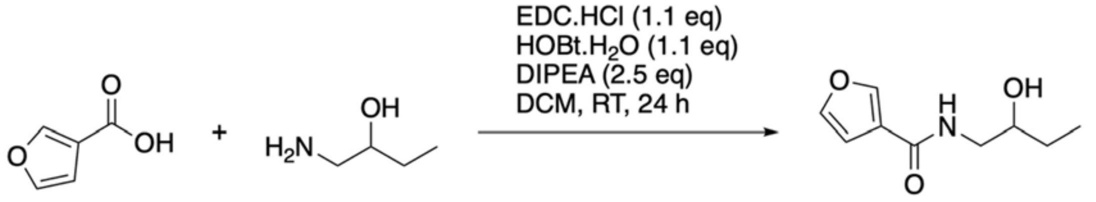

1zj-rac

1zj-rac was prepared according to **General Procedure F** using carboxylic acid substrate 3-furoic acid (224 mg, 2.00 mmol), 1-amino-2-butanol (178 mg, 2.00 mmol), EDC.HCl (422 mg, 2.20 mmol), HOBt.H_2O (421 mg, 2.20 mmol) and DIPEA (0.86 mL, 4.94 mmol) stirred overnight for 24 h in DCM. Purification by silica gel column chromatography (elution with 3% MeOH in DCM) afforded **1zj-rac** as a white solid (305 mg, 1.67 mmol, 83% yield).

^{1}H NMR (700 MHz, $CDCl_3$) δ 7.96 – 7.92 (m, 1H), 7.42 (t, *J* = 1.7 Hz, 1H), 6.64 – 6.62 (m, 1H), 6.60 (br. s, 1H), 3.73 – 3.66 (m, 1H), 3.61 (ddd, *J* = 14.0, 6.5, 2.9 Hz, 1H), 3.24 (ddd, *J* = 14.0, 7.9, 5.1 Hz, 1H), 3.13 (br. d, *J* = 3.8 Hz, 1H), 1.56 – 1.45 (m, 2H), 0.96 (t, *J* = 7.5 Hz, 3H) ppm.
^{13}C NMR (176 MHz, $CDCl_3$) δ 163.8, 145.1, 143.9, 122.5, 108.4, 72.8, 45.4, 28.1, 10.0 ppm.
HRMS $[M+H]^+$ m/z calc'd for $[C_9H_{13}NO_3H]^+$ expect 184.0974; found 184.0970.

***N*-(2-hydroxybutyl)-1-methyl-1*H*-pyrazole-4-carboxamide (1zk-rac)**

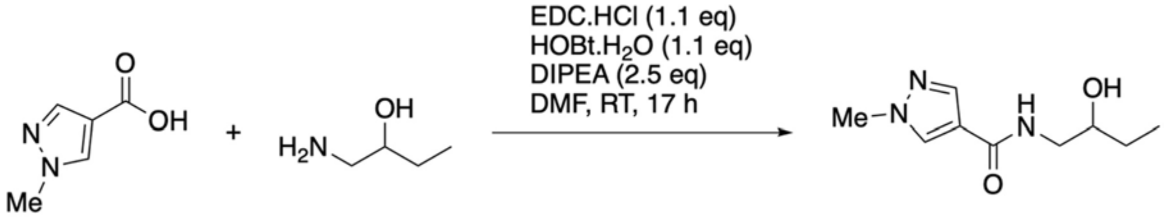

1zk-rac

1zk-rac was prepared according to **General Procedure F** using carboxylic acid substrate 1-methyl-1H-pyrazole-4-carboxylic acid (252 mg, 2.00 mmol), 1-amino-2-butanol (178 mg, 2.00 mmol), EDC.HCl (422 mg, 2.20 mmol), HOBt.H_2O (421 mg, 2.20 mmol) and DIPEA (0.86 mL, 4.94 mmol) stirred overnight for 17 h in DMF. Purification by silica gel column chromatography (elution with 7% MeOH in DCM) afforded **1zk-rac** as a white solid (101 mg, 0.51 mmol, 26% yield).

^{1}H NMR (700 MHz, $CDCl_3$) δ 7.82 (s, 1H), 7.74 (s, 1H), 6.43 (br. s, 1H), 3.90 (s, 3H), 3.73 – 3.68 (m, 1H), 3.62 (ddd, *J* = 14.0, 6.4, 2.8 Hz, 1H), 3.25 (ddd, *J* = 14.0, 8.0, 5.4 Hz, 1H), 2.36 (br. s, 1H) (overlaps with H_2O peak), 1.58 – 1.47 (m, 2H), 0.97 (t, *J* = 7.5 Hz, 3H) ppm.
^{13}C NMR (176 MHz, $CDCl_3$) δ 163.6, 138.1, 132.0, 118.6, 72.9, 45.4, 39.4, 28.1, 10.0 ppm.
HRMS $[M+H]^+$ m/z calc'd for $[C_9H_{15}N_3O_2H]^+$ expect 198.1243; found 198.1242.

6-chloro-*N*-(2-hydroxybutyl)nicotinamide (1zl-rac)

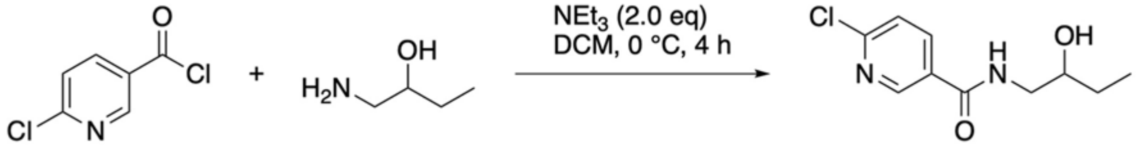

1zl-rac

1zl-rac was prepared according to **General Procedure E** using acyl chloride substrate 6-chloronicotinoyl chloride (352 mg, 2.00 mmol), 1-amino-2-butanol (178 mg, 2.00 mmol) and triethylamine (0.56 mL, 4.02 mmol). Purification by silica gel column chromatography (elution with 2% 7N ammonia in MeOH, 4% MeOH, 94% DCM) afforded **1zl-rac** as a white solid (299 mg, 1.31 mmol, 65% yield).

^{1}H NMR (700 MHz, $(CD_3)_2CO$) δ 8.88 (dd, *J* = 2.5, 0.6 Hz, 1H), 8.27 (dd, *J* = 8.3, 2.5 Hz, 1H), 7.97 (br. s, 1H), 7.55 (dd, *J* = 8.3, 0.6 Hz, 1H), 4.02 (d, *J* = 5.0 Hz, 1H), 3.72 – 3.65 (m, 1H), 3.53 (ddd, *J* = 13.5, 6.1, 4.0 Hz, 1H), 3.30 (ddd, *J* = 13.5, 7.5, 5.7 Hz, 1H), 1.58 – 1.50 (m, 1H), 1.47 – 1.39 (m, 1H), 0.96 (t, *J* = 7.5 Hz, 3H) ppm.
^{13}C NMR (176 MHz, $(CD_3)_2CO$) δ 165.3, 154.1, 149.8, 139.1, 130.7, 124.8, 72.3, 46.8, 28.6, 10.3 ppm.
HRMS $[M+H]^+$ m/z calc'd for $[C_{10}H_{13}ClN_2O_2H]^+$ expect 229.0744; found 229.0737.

***N*-(2-hydroxybutyl)-2-(thiazol-4-yl)acetamide (1zm-rac)**

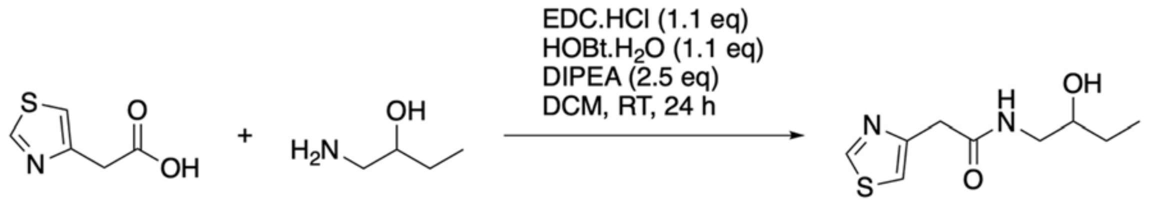

1zm-rac

1zm-rac was prepared according to **General Procedure F** using carboxylic acid substrate 2-(thiazol-4-yl) acetic acid (286 mg, 2.00 mmol), 1-amino-2-butanol (178 mg, 2.00 mmol), EDC.HCl (422 mg, 2.20 mmol), HOBt.H_2O (421 mg, 2.20 mmol) and DIPEA (0.86 mL, 4.94 mmol) stirred overnight for 24 h in DCM. Purification by silica gel column chromatography (elution with 4% MeOH in DCM) afforded **1zm-rac** as a pale yellow oil (171 mg, 0.80 mmol, 40% yield).

^{1}H NMR (700 MHz, $CDCl_3$) δ 8.81 (d, *J* = 2.0 Hz, 1H), 7.22 – 7.19 (m, 1H), 7.08 (br. s, 1H), 3.79 (s, 2H), 3.67 – 3.60 (m, 1H), 3.48 (ddd, *J* = 14.0, 6.5, 3.0 Hz, 1H), 3.14 (ddd, *J* = 14.0, 8.0, 5.5 Hz, 1H), 2.92 (br. s, 1H), 1.51 – 1.41 (m, 2H), 0.94 (t, *J* = 7.5 Hz, 3H) ppm.
^{13}C NMR (176 MHz, $CDCl_3$) δ 170.4, 153.7, 150.8, 116.3, 72.7, 45.8, 39.0, 27.9, 10.0 ppm.
HRMS $[M+H]^+$ m/z calc'd for $[C_9H_{14}N_2O_2SH]^+$ expect 215.0854; found 215.0858.

***tert*-butyl 4-((2-hydroxybutyl)carbamoyl)piperidine-1-carboxylate (1zn-rac)**

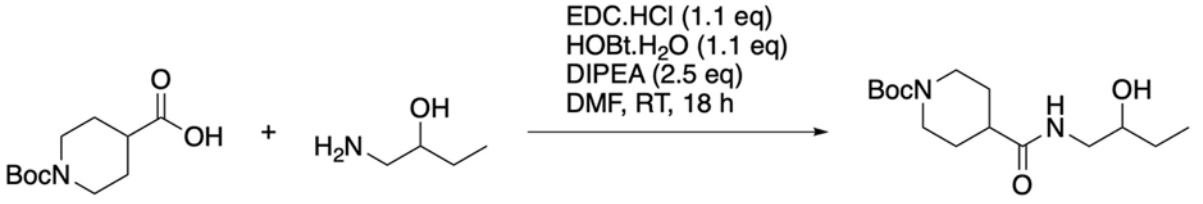

1zn-rac

1zn-rac was prepared according to **General Procedure F** using carboxylic acid substrate 1-(*tert*-butoxycarbonyl)piperidine-4-carboxylic acid (459 mg, 2.00 mmol), 1-amino-2-butanol (178 mg, 2.00 mmol), EDC.HCl (422 mg, 2.20 mmol), HOBt.H_2O (421 mg, 2.20 mmol) and DIPEA (0.86 mL, 4.94 mmol) stirred overnight for 18 h in DMF. Purification by silica gel column chromatography (elution with 4% MeOH in DCM) afforded **1zn-rac** as a white solid (524 mg, 1.75 mmol, 87% yield).

^{1}H NMR (500 MHz, $CDCl_3$) δ 6.18 – 5.99 (m, 1H), 4.28 – 3.97 (m, 2H), 3.66 – 3.55 (m, 1H), 3.48 (ddd, *J* = 13.9, 6.5, 3.0 Hz, 1H), 3.11 (ddd, *J* = 13.9, 7.7, 5.0 Hz, 1H), 2.80 – 2.64 (m, 2H), 2.47 (br. s, 1H), 2.25 (tt, *J* = 11.6, 3.7 Hz, 1H), 1.85 – 1.75 (m, 2H), 1.67 – 1.55 (m, 2H), 1.51 – 1.41 (m, 11H), 0.94 (t, *J* = 7.5 Hz, 3H) ppm.
^{13}C NMR (126 MHz, $CDCl_3$) δ 175.5, 154.8, 79.8, 72.8, 45.2, 43.4, 28.8, 28.7, 28.6, 28.1, 10.0 ppm.
HRMS $[M+H]^+$ m/z calc'd for $[C_{15}H_{28}N_2O_4Na]^+$ expect 323.1947; found 323.1963.

***N*-(2-hydroxybutyl)morpholine-4-carboxamide (1zo-rac)**

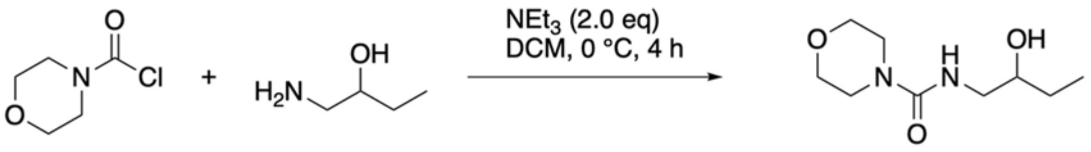

1zo-rac

1zo-rac was prepared according to **General Procedure E** using acyl chloride substrate 4-morpholinecarbonyl chloride (0.23 mL, 1.97 mmol), 1-amino-2-butanol (178 mg, 2.00 mmol) and triethylamine (0.56 mL, 4.02 mmol). Purification by silica gel column chromatography (elution with 5% MeOH in DCM) afforded **1zo-rac** as a white solid (366 mg, 1.81 mmol, 90% yield).

^{1}H NMR (500 MHz, $CDCl_3$) δ 5.04 (br. s, 1H), 3.68 (t, *J* = 4.9 Hz, 4H), 3.64 – 3.58 (m, 1H), 3.44 (dd, *J* = 14.1, 2.7 Hz, 1H), 3.35 (t, *J* = 4.9 Hz, 4H), 3.12 (dd, *J* = 14.1, 7.9 Hz, 1H), 2.68 (br. s, 1H), 1.52 – 1.43 (m, 2H), 0.95 (t, *J* = 7.5 Hz, 3H) ppm.
^{13}C NMR (126 MHz, $CDCl_3$) δ 158.9, 73.5, 66.6, 46.7, 44.2, 28.0, 10.0 ppm.
HRMS $[M+H]^+$ m/z calc'd for $[C_9H_{18}N_2O_3H]^+$ expect 203.1396; found 203.1388.

1-(*tert*-butyl)-3-(2-hydroxybutyl)urea (1zp-rac)

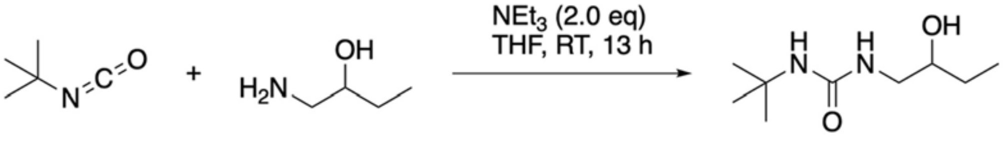

1zp-rac

A solution of *tert*-butyl isocyanate (0.23 mL, 2.01 mmol), 1-amino-2-butanol (178 mg, 2.00 mmol) and triethylamine (0.56 mL, 4.02 mmol) in dry THF (15 mL) was stirred at RT for 13 h. The crude mixture was concentrated *in vacuo* and purified by silica gel column chromatography (elution with 4% MeOH in DCM) to afford **1zp-rac** as a white solid (355 mg, 1.89 mmol, 94% yield).

^{1}H NMR (700 MHz, $CDCl_3$) δ 3.62 – 3.54 (m, 1H), 3.29 (dd, *J* = 14.1, 2.7 Hz, 1H), 3.02 (dd, *J* = 14.1, 7.7 Hz, 1H), 1.46 (app. quint, *J* = 7.2 Hz, 2H), 1.32 (s, 9H), 0.94 (t, *J* = 7.5 Hz, 3H) ppm.
^{13}C NMR (176 MHz, $CDCl_3$) δ 159.1, 73.8, 50.6, 46.3, 29.6, 27.9, 10.1 ppm.
HRMS $[M+H]^+$ m/z calc'd for $[C_9H_{20}N_2O_2H]^+$ expect 189.1603; found 189.1603.

1-(3,5-bis(trifluoromethyl)phenyl)-3-(2-hydroxybutyl)urea (1zq-rac)

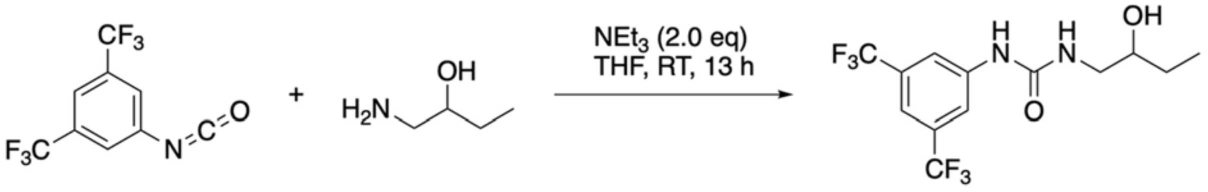

1zq-rac

A solution of 3,5-di(trifluoromethyl)phenyl isocyanate (0.35 mL, 2.02 mmol), 1-amino-2-butanol (178 mg, 2.00 mmol) and triethylamine (0.56 mL, 4.02 mmol) in dry THF (15 mL) was stirred at RT for 13 h. The crude mixture was concentrated *in vacuo* and purified by silica gel column chromatography (elution with 4% MeOH in DCM) to afford **1zq-rac** as a white solid (416 mg, 1.21 mmol, 60% yield).

^{1}H NMR (700 MHz, $(CD_3)_2CO$) δ 8.71 (br. s, 1H), 8.14 (s, 2H), 7.52 (s, 1H), 6.14 (br. s, 1H), 3.99 (br. s, 1H), 3.64 – 3.56 (m, 1H), 3.40 (ddd, *J* = 13.6, 6.3, 3.7 Hz, 1H), 3.11 (ddd, *J* = 13.6, 7.3, 5.1 Hz, 1H), 1.54 – 1.47 (m, 1H), 1.47 – 1.39 (m, 1H), 0.94 (t, *J* = 7.5 Hz, 3H) ppm.
^{19}F NMR (471 MHz, $(CD_3)_2CO$) –63.7 ppm.
^{13}C NMR (176 MHz, $(CD_3)_2CO$) δ 156.0 (2C), 143.7 (2C), 132.4 (q, *J* = 32.8 Hz), 124.5 (q, *J* = 271.9 Hz), 118.3 (2C, q, *J* = 3.7 Hz, m), 114.7 (app. quint, *J* = 4.0 Hz), 72.6 (2C), 46.3, 28.5 (2C), 10.3 ppm.
HRMS $[M+H]^+$ m/z calc'd for $[C_{13}H_{14}F_6N_2O_2H]^+$ expect 345.1038; found 345.1043.

***tert*-butyl (2-hydroxybutyl)carbamate (1zr-rac)**

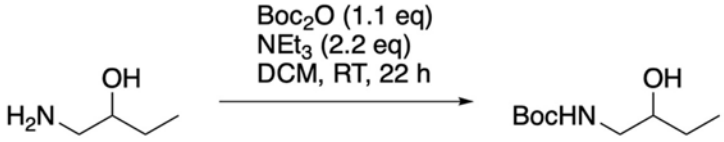

1zr-rac

Boc anhydride (0.51 mL, 2.22 mmol) was added slowly to a solution of 1-amino-2-butanol (178 mg, 2.00 mmol) and triethylamine (0.61 mL, 4.44 mmol) in DCM (7 mL) stirred at 0 °C. The reaction mixture was warmed to RT and stirred for 22 h. The crude mixture was concentrated *in vacuo* and purified by silica gel column chromatography (elution with 4% MeOH in DCM) to afford **1zr-rac** as a pale yellow oil (355 mg, 1.88 mmol, 94% yield).

^{1}H NMR (700 MHz, $CDCl_3$) δ 4.91 (br. s, 1H), 3.62 (app. br. s, 1H), 3.37 – 3.23 (m, 1H), 3.01 (ddd, *J* = 13.4, 7.1, 6.3 Hz, 1H), 2.30 (br. s, 1H), 1.53 – 1.40 (m, 11H), 0.96 (t, *J* = 7.5 Hz, 3H) ppm.
^{13}C NMR (176 MHz, $CDCl_3$) δ 157.0, 79.8, 73.2, 46.5, 28.5, 27.9, 10.0 ppm.

The data is in good agreement with the literature.[24]

2-(benzyloxy)-*N*-(2-hydroxybutyl)acetamide (1zs-rac)

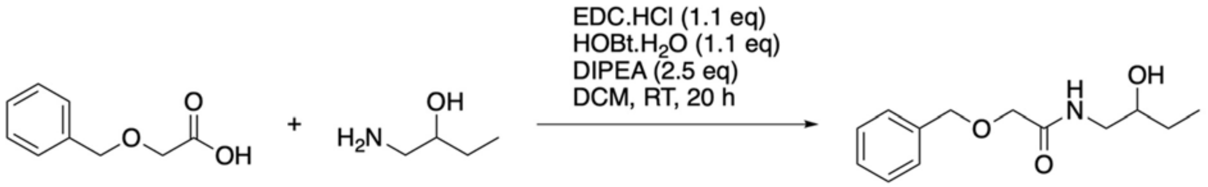

1zs-rac

1zs-rac was prepared according to **General Procedure F** using carboxylic acid substrate benzyloxyacetic acid (0.29 mL, 2.03 mmol), 1-amino-2-butanol (178 mg, 2.00 mmol), EDC.HCl (422 mg, 2.20 mmol), $HOBt.H_2O$ (421 mg, 2.20 mmol) and DIPEA (0.86 mL, 4.94 mmol) stirred overnight for 20 h in DCM. Purification by silica gel column chromatography (elution with 2% MeOH in DCM) afforded **1zs-rac** as a pale yellow oil that slowly forms a white solid (390 mg, 1.64 mmol, 82% yield).

^{1}H NMR (700 MHz, $CDCl_3$) δ 7.37 (t, *J* = 7.3 Hz, 2H), 7.35 – 7.31 (m, 3H), 6.98 (br. s, 1H), 4.58 (s, 2H), 4.01 (s, 2H), 3.67 – 3.61 (m, 1H), 3.49 (ddd, *J* = 13.9, 6.6, 3.0 Hz, 1H), 3.19 (ddd, *J* = 13.9, 7.9, 5.7 Hz, 1H), 2.26 (br. s, 1H), 1.54 – 1.44 (m, 2H), 0.96 (t, *J* = 7.5 Hz, 3H) ppm.
^{13}C NMR (176 MHz, $CDCl_3$) δ 170.8, 136.9, 128.8, 128.4, 128.1, 73.8, 72.9, 69.6, 45.0, 28.0, 10.0 ppm.
HRMS $[M+H]^+$ m/z calc'd for $[C_{13}H_{19}NO_3H]^+$ expect 238.1443; found 238.1439.

***N*-(2-hydroxybutyl)-2-(methylthio)acetamide (1zt-rac)**

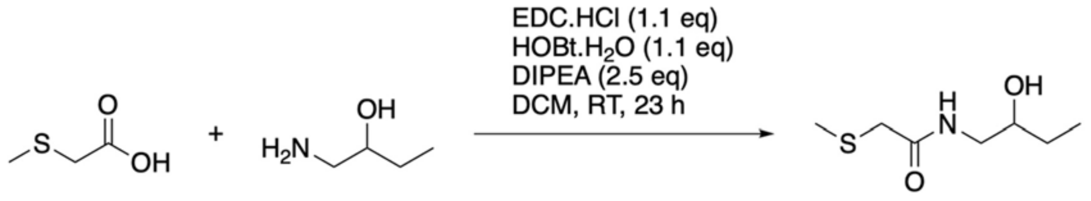

1zt-rac

1zt-rac was prepared according to **General Procedure F** using carboxylic acid substrate (methylthio)acetic acid (0.17 mL, 1.95 mmol), 1-amino-2-butanol (178 mg, 2.00 mmol), EDC.HCl (422 mg, 2.20 mmol), HOBt.H_2O (421 mg, 2.20 mmol) and DIPEA (0.86 mL, 4.94 mmol) stirred overnight for 23 h in DCM. Purification by silica gel column chromatography (elution with 4% MeOH in DCM) afforded **1zt-rac** as a pale yellow oil (221 mg, 1.25 mmol, 62% yield).

^{1}H NMR (700 MHz, $CDCl_3$) δ 7.24 (br. s, 1H) (overlaps with $CHCl_3$ peak), 3.70 – 3.63 (m, 1H), 3.52 (ddd, *J* = 14.0, 6.5, 3.0 Hz, 1H), 3.22 (s, 2H), 3.20 (ddd, *J* = 14.0, 7.8, 5.5 Hz, 1H), 2.42 (d, *J* = 4.7 Hz, 1H), 2.15 (s, 3H), 1.56 – 1.45 (m, 2H), 0.98 (t, *J* = 7.5 Hz, 3H) ppm.
^{13}C NMR (176 MHz, $CDCl_3$) δ 169.7, 72.9, 45.6, 38.3, 28.1, 16.5, 10.0 ppm.
HRMS $[M+H]^+$ m/z calc'd for $[C_7H_{15}NO_2SH]^+$ expect 178.0902; found 178.0908.

***N*-(5-ethoxy-2-hydroxypentyl)-2-(thiazol-4-yl)acetamide (1zu-rac)**

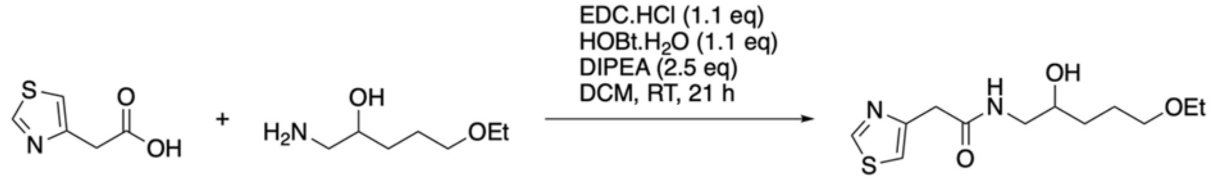

1o-I$_3$ **1zt-rac**

DIPEA (0.37 mL, 2.12 mmol) was added to a stirred suspension of 2-(thiazol-4-yl) acetic acid (125 mg, 0.87 mmol), **1o-I$_3$** (129 mg, 0.88 mmol), EDC.HCl (184 mg, 0.96 mmol) and HOBt.H_2O (≥20 wt% H_2O, 184 mg, 0.96 mmol) in DCM (3 mL). The reaction mixture was stirred for 21 h, concentrated *in vacuo*, water was added and the crude extracted with ethyl acetate. The combined organic extracts were dried over $MgSO_4$, filtered, and the solvent removed *in vacuo* to afford crude mixture. The crude mixture was purified by silica gel column chromatography (elution with 4% MeOH in DCM) to afford **1zt-rac** as a pale yellow oil (91.9 mg, 0.34 mmol, 39% yield).

^{1}H NMR (700 MHz, $CDCl_3$) δ 8.81 (d, *J* = 1.9 Hz, 1H), 7.22 – 7.19 (m, 1H), 7.00 (br. s, 1H), 3.85 (br. s, 1H), 3.79 (s, 2H), 3.72 – 3.66 (m, 1H), 3.52 – 3.44 (m, 4H), 3.44 – 3.39 (m, 1H), 3.11 (ddd, *J* = 13.5, 8.1, 5.1 Hz, 1H), 1.75 – 1.65 (m, 2H) (overlaps with H_2O peak), 1.65 – 1.59 (m, 1H), 1.48 – 1.41 (m, 1H), 1.20 (t, *J* = 7.0 Hz, 3H) ppm.
^{13}C NMR (176 MHz, $CDCl_3$) δ 170.0, 153.5, 150.9, 116.1, 70.9, 70.8, 66.5, 45.9, 39.1, 32.9, 26.5, 15.2 ppm.

HRMS $[M+H]^+$ m/z calc'd for $[C_{12}H_{20}N_2O_3SH]^+$ expect 273.1273; found 273.1286.

***N*-(2-hydroxy-3-phenylpropyl)morpholine-4-carboxamide (1zv-rac)**

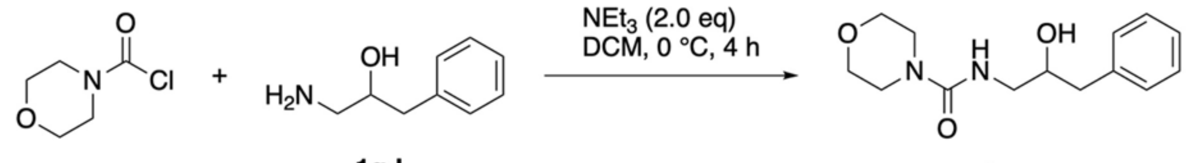

1g-I$_1$ **1zv-rac**

Triethylamine (0.17 mL, 1.22 mmol) was added to a stirred solution of **1g-I$_1$** (90.9 mg, 0.60 mmol) in dry DCM (5 mL). The solution was cooled to 0 °C and 4-morpholinecarbonyl chloride (0.070 mL, 0.60 mmol) added slowly. The reaction mixture was stirred for 4 h at 0 °C, concentrated *in vacuo* and purified by silica gel column chromatography (eluted with 3% MeOH in DCM) to afford **1zv-rac** as a viscous colorless oil that slowly forms a white solid (138 mg, 0.52 mmol, 87% yield).

^{1}H NMR (700 MHz, $CDCl_3$) δ 7.31 (t, *J* = 7.5 Hz, 2H), 7.25 – 7.19 (m, 3H), 4.82 (br. s, 1H), 3.99 – 3.93 (m, 1H), 3.67 (br. t, *J* = 4.9 Hz, 4H), 3.50 (ddd, *J* = 14.1, 6.5, 2.8 Hz, 1H), 3.33 (br. t, *J* = 4.9 Hz, 4H), 3.19 (ddd, *J* = 14.1, 7.7, 4.9 Hz, 1H), 3.11 (br. s, 1H), 2.83 – 2.75 (m, 2H) ppm.
^{13}C NMR (176 MHz, $CDCl_3$) δ 158.8, 138.0, 129.5, 128.8, 126.8, 73.0, 66.6, 46.4, 44.1, 41.7 ppm.
HRMS $[M+H]^+$ m/z calc'd for $[C_{14}H_{20}N_2O_3H]^+$ expect 265.1552; found 265.1556.

***tert*-butyl ((2*S*)-1-((2-hydroxybutyl)amino)-1-oxo-3-phenylpropan-2-yl)carbamate (2a (1:1 mix of (*S,S*) and (*S,R*))**

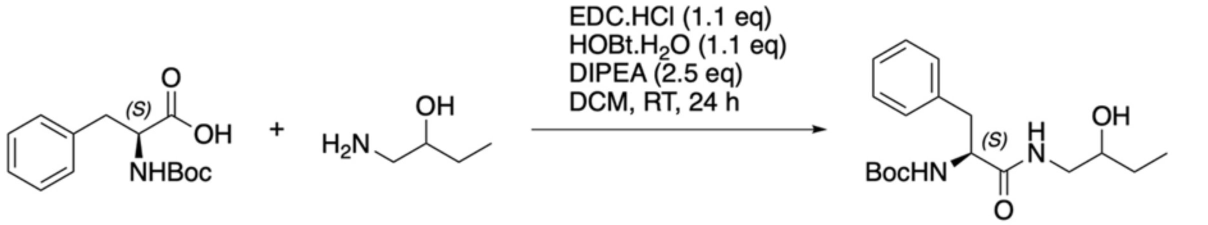

2a (1:1 mix of (*S,S*) and (*S,R*))

2a (1:1 mix of (*S,S*) and (*S,R*) was prepared according to **General Procedure F** using carboxylic acid substrate *N*-(*tert*-butoxycarbonyl)-L-phenylalanine (531 mg, 2.00 mmol), 1-amino-2-butanol (178 mg, 2.00 mmol), EDC.HCl (422 mg, 2.20 mmol), HOBt.H_2O (421 mg, 2.20 mmol) and DIPEA (0.86 mL, 4.94 mmol) stirred overnight for 24 h in DCM. Purification by silica gel column chromatography (elution with 3% MeOH in DCM) afforded **2a (1:1 mix of (*S,S*) and (*S,R*))** as a white solid, isolated as a mixture of diastereomers (618 mg, 1.84 mmol, 92% yield, 1:1 dr). Combined signals for both diastereomers are denoted *d*1+*d*2. Discernible signals are denoted *d*1 and *d*2.

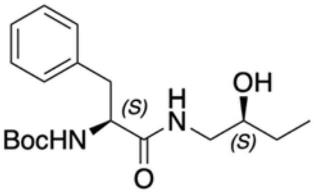

2a-(S,S) (d1)

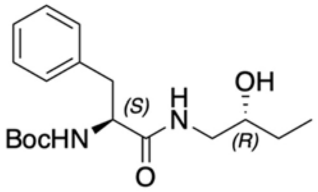

2a-(S,R) (d2)

^{1}H NMR (700 MHz, $CDCl_3$) δ 7.32 – 7.27 (m, 2H, *d*1+*d*2), 7.26 – 7.18 (m, 3H, *d*1+*d*2), 6.39 (br. s, 0.5H, *d*1), 6.29 (br. s, 0.5 H, *d*2), 5.19 (br.s, 1H, *d*1+*d*2), 4.38 – 4.20 (m, 1H, *d*1+*d*2), 3.57 – 3.49 (m, 0.5H, *d*1), 3.47 – 3.33 (m, 1.5H, *d1*+*d*2), 3.15 – 2.89 (m, 3H, *d*1+*d*2), 2.76 (br. s, 0.5H, *d*1), 2.45 (br. s, 0.5H, *d*2), 1.43 – 1.31 (m, 2H, *d1*+*d*2), 1.40 (s, 4.5H, *d*2), 1.39 (s, 4.5H, *d*1), 0.91 (t, *J* = 7.5 Hz, 1.5H, *d*1/*d*2), 0.90 (t, *J* = 7.4 Hz, 1.5H, *d*2/*d*1) ppm.
^{13}C NMR (176 MHz, $CDCl_3$) δ 172.2 (*d*1), 172.1 (*d*2), 155.7 (*d*1+*d*2), 136.9 (*d*2), 136.8 (*d*1), 129.5 (*d*2), 129.4 (*d*1), 128.83 (*d*2), 128.78 (*d*1), 127.11 (*d*2), 127.09 (*d*1), 80.4 (*d*1+*d*2), 72.3 (*d*2), 72.2 (*d*1), 56.4 (*d*2), 56.3 (*d*1), 45.45 (*d*1), 45.39 (*d*2), 38.9 (*d*2), 38.7 (*d*1), 28.41 (*d*2), 28.40 (*d*1), 27.69 (*d*1/*d*2), 27.66 (*d*2/*d*1), 10.0 (*d*1), 9.9 (*d*2) ppm.
HRMS $[M+H]^+$ m/z calc'd for $[C_{18}H_{28}N_2O_4H]^+$ expect 337.2127; found 337.2115.

***tert*-butyl ((2*R*)-1-((2-hydroxybutyl)amino)-1-oxo-3-phenylpropan-2-yl)carbamate (2b (1:1 mix of (*R,S) and (R,R)*))**

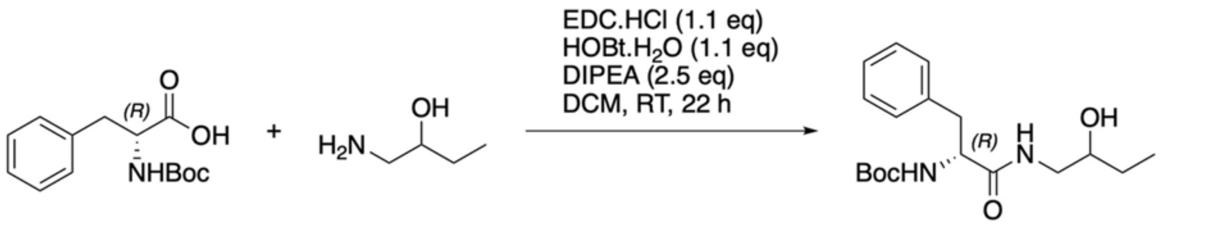

2b (1:1 mix of (*R*,*S*) and (*R*,*R*)) was prepared according to **General Procedure F** using carboxylic acid substrate *N*-(*tert*-butoxycarbonyl)-D-phenylalanine (531 mg, 2.00 mmol), 1-amino-2-butanol (178 mg, 2.00 mmol), EDC.HCl (422 mg, 2.20 mmol), HOBt.H_2O (421 mg, 2.20 mmol) and DIPEA (0.86 mL, 4.94 mmol) stirred overnight for 22 h in DCM. Purification by silica gel column chromatography (elution with 4% MeOH in DCM) afforded **2b (1:1 mix of (*R*,*S*) and (*R*,*R*))** as a white solid, isolated as a mixture of diastereomers (603 mg, 1.79 mmol, 90% yield, 1:1 dr). Combined signals for both diastereomers are denoted *d*1+*d*2. Discernible signals are denoted *d*1 and *d*2.

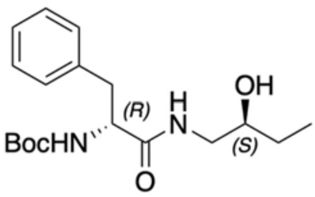

2b-(R,S) (d1)

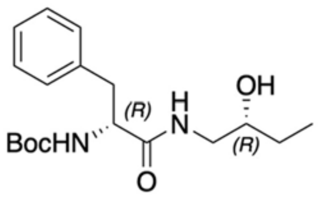

2b-(R,R) (d2)

^{1}H NMR (700 MHz, $CDCl_3$) δ 7.33 – 7.27 (m, 2H, *d*1+*d*2), 7.26 – 7.18 (m, 3H, *d*1+*d*2), 6.38 (br. s, 0.5H, *d*2), 6.28 (br. s, 0.5 H, *d*1), 5.19 (br.s, 1H, *d*1+*d*2), 4.38 – 4.19 (m, 1H, *d*1+*d*2), 3.58 – 3.49 (m, 0.5H, *d*2), 3.47 – 3.32 (m, 1.5H, *d1*+*d*2), 3.15 – 2.89 (m, 3H, *d*1+*d*2), 2.74 (br. s, 0.5H, *d*1/*d*2), 2.43 (br. s, 0.5H, *d*2/*d*1), 1.44 – 1.31 (m, 2H, *d1*+*d*2), 1.40 (s, 4.5H, *d*1), 1.39 (s, 4.5H, *d*2), 0.91 (t, *J* = 7.5 Hz, 1.5H, *d*1/*d*2), 0.90 (t, *J* = 7.4 Hz, 1.5H, *d*2/*d*1) ppm.
^{13}C NMR (176 MHz, $CDCl_3$) δ 172.2 (*d*2), 172.1 (*d*1), 155.7 (*d*1+*d*2), 136.9 (*d*1), 136.8 (*d*2), 129.5 (*d*1), 129.4 (*d*2), 128.82 (*d*1), 128.78 (*d*2), 127.11 (*d*1), 127.09 (*d*2), 80.4 (*d*1+*d*2), 72.3 (*d*1), 72.2 (*d*2), 56.35 (*d*1/*d*2), 56.28 (*d*2/*d*1), 45.44 (*d*1), 45.38 (*d*2), 38.9 (*d*1), 38.7 (*d*2), 28.4 (*d*1+*d*2), 27.7 (*d*1+*d*2), 10.0 (*d*2), 9.9 (*d*1) ppm.
HRMS $[M+H]^+$ m/z calc'd for $[C_{18}H_{28}N_2O_4H]^+$ expect 337.2127; found 337.2114.

***N*-(2-hydroxybutyl)-2-(4-isobutylphenyl)propanamide (2c-rac)**

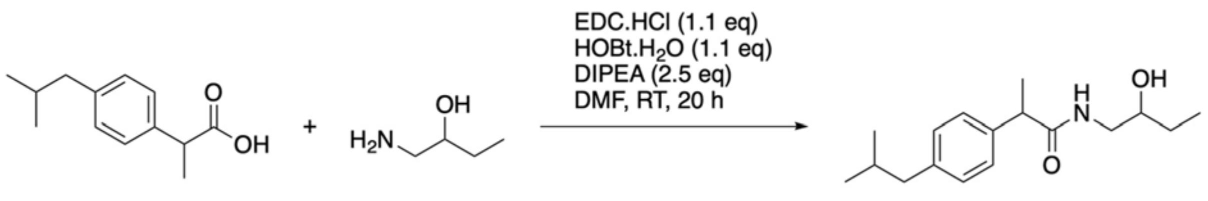

2c-rac

2c-rac was prepared according to **General Procedure F** using carboxylic acid substrate Ibuprofen (413 mg, 2.00 mmol), 1-amino-2-butanol (178 mg, 2.00 mmol), EDC.HCl (422 mg, 2.20 mmol), HOBt.H_2O (421 mg, 2.20 mmol) and DIPEA (0.86 mL, 4.94 mmol) stirred overnight for 20 h in DMF. Purification by silica gel column chromatography (elution with 5% MeOH in DCM) afforded **2c-rac** as an off-white solid, isolated as a mixture of diastereomers (481 mg, 1.73 mmol, 87% yield, 1:1 dr).

^{1}H NMR (700 MHz, $CDCl_3$) δ 7.19 (d, *J* = 7.6 Hz, 2H), 7.12 (d, *J* = 7.6 Hz, 2H), 5.76 (br. s, 1H), 3.59 – 3.52 (m, 2H), 3.43 – 3.35 (m, 1H), 3.13 – 3.04 (m, 1H), 2.45 (d, *J* = 7.2 Hz, 2H), 2.11 (br. s, 1H), 1.85 (app. n, *J* = 6.8 Hz, 1H), 1.52 (d, *J* = 7.2 Hz, 3H), 1.43 – 1.35 (m, 2H), 0.92 – 0.88 (m, 9H) ppm.
^{13}C NMR (176 MHz, $CDCl_3$) δ 176.0, 175.9, 140.99, 140.97, 138.5, 129.83, 129.82, 127.5, 73.1, 73.0, 46.85, 46.83, 45.7, 45.6, 45.1, 30.3, 27.96, 27.95, 22.5, 18.59, 18.58, 9.9 ppm.
^{13}C NMR (126 MHz, $(CD_3)_2SO$) δ 173.59, 173.55, 139.7, 139.6, 139.1, 128.69, 128.68, 126.98, 126.95, 70.5, 70.3, 44.63, 44.59, 44.58, 44.56, 44.249, 44.246, 29.641, 29.637, 27.22, 27.18, 22.18, 22.171, 22.167, 22.16, 18.7, 18.5, 9.83, 9.80 ppm.
^{13}C NMR (126 MHz, $(CD_3)_2SO$, 80 °C) δ 173.33, 173.28, 139.4, 139.3, 138.72, 138.71, 128.271, 128.269, 126.59, 126.58, 70.3, 70.2, 44.6, 44.5, 44.44, 44.42, 43.934, 43.930, 29.010, 29.006, 26.9, 26.8, 21.72, 21.714, 21.709, 21.70, 18.2, 18.1, 9.16, 9.15 ppm.
HRMS $[M+H]^+$ m/z calc'd for $[C_{17}H_{27}NO_2H]^+$ expect 278.2120; found 278.2122.

The two diastereomers of **2c-rac** could not be distinguished by ^{1}H NMR in $CDCl_3$. However, discernable signals consistent with two diastereomers are seen in the ^{13}C NMR spectrum. These are also observed in the ^{13}C NMR spectra in $(CD_3)_2SO$ at both RT and at 80°C. This provides strong evidence that the signals in the ^{13}C NMR spectra in $(CD_3)_2SO$ are due to a mixture of diastereomers rather than rotamers. These signals integrate 1:1, consistent with 1:1 dr for **2c-rac**.

Deracemization Reactions

General Procedures

General Procedure G: reaction procedure for the deracemization of *N*-acyl amino alcohols

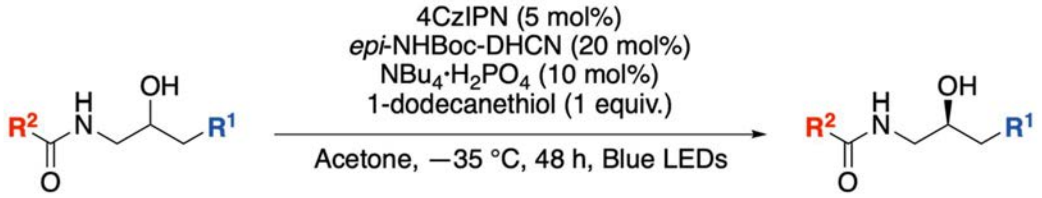

N-acyl amino alcohol substrate (0.10 mmol, 1 eq.), 4CzIPN (3.9 mg, 0.0050 mmol, 5 mol%), *epi*-NHBoc-DHCN (7.9 mg, 0.020 mmol, 20 mol%) and tetrabutylammonium phosphate monobasic ($NBu_4 \cdot H_2PO_4$) (3.4 mg, 0.010 mmol, 10 mol%) were added to a 4.0 mL crimp-top vial. The vial was sealed with a crimp-top cap and evacuated and backfilled with nitrogen 10 times. Freshly prepared 0.5 M stock solution of 1-dodecanethiol in degassed acetone (0.2 mL) followed by additional degassed acetone (3.8 mL) was added *via* syringe to the sealed vial (reaction molarity of 0.025 M). The top of the vial was further sealed with Teflon tape then electrical tape and placed in the custom made photoreactor with a positive flow of nitrogen through the apparatus. The reaction vial was cooled to –35 °C and the Kessil® A160WE Tuna blue, LED lamp (100% intensity, 100% ocean blue color) was switched on. After stirring for 48 h (unless otherwise specified) the crude mixture was concentrated *in vacuo*, analysed by ^{1}H NMR using internal standard (CH_2Br_2) and purified by silica gel column chromatography.

To generate the opposite enantiomer, *epi*-NHBoc-DHCD (7.9 mmol, 0.020 mmol, 20 mol%) was used in place of *epi*-NHBoc-DHCN and the procedure otherwise identically followed.

The custom made photoreactor (Picnic Photoreactor) used has been detailed in previous work.[1,2] The syntheses of HAA catalysts *epi*-NHBoc-dihydrocinchonine (*epi*-NHBoc-DHCN) and *epi*-NHBoc-dihydrocinchonidine (*epi*-NHBoc-DHCD) were conducted as previously reported.[1]

General Procedure H: benzoylation for chiral SFC or chiral HPLC analysis

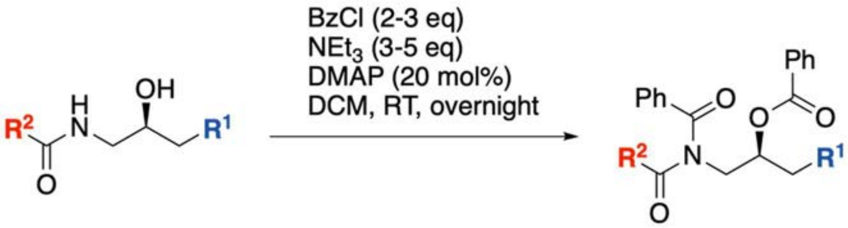

Derivatization *via* benzoylation was carried out for products which did not contain a chromophore. To purified *N*-acyl amino alcohol product (1.0 eq.), DMAP (20 mol%) and triethylamine (3-4 eq.) in DCM was added benzoyl chloride at RT (2-3 eq.). The reaction mixture was stirred at RT overnight, concentrated under nitrogen flow and purified by silica gel column chromatography to afford mono-, di- or tri-benzoylated product, dependant on substrate.

Racemic samples for chiral SFC or chiral HPLC analysis were obtained using this procedure with racemic *N*-acyl amino alcohol as substrate. The crude mixture was purified by preparative thin layer chromatography for analysis.

Main Scope Product Characterization

Yields are denoted as *isolated yield*% (*NMR yield*%) where NMR yield could be determined.

(*S*)-*N*-(2-hydroxybutyl)acetamide (1a)

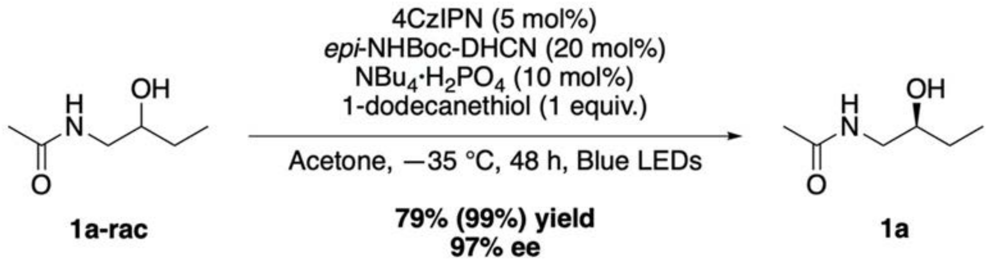

1a was prepared according to **General Procedure G** using amino alcohol-derived substrate **1a-rac** (13.1 mg, 0.10 mmol). Purification by silica gel column chromatography (elution with 2% 7N ammonia in MeOH, 4% MeOH, 94% DCM followed by a second purification eluting with 10% MeOH in DCM) afforded **1a** as a pale yellow oil (10.3 mg, 0.079 mmol, 79% yield).

^{1}H NMR (700 MHz, $CDCl_3$) δ 5.99 (br. s, 1H), 3.68 – 3.59 (m, 1H), 3.48 (ddd, *J* = 14.0, 6.5, 2.9 Hz, 1H), 3.11 (ddd, *J* = 14.0, 7.9, 5.1 Hz, 1H), 2.59 (br. s, 1H), 2.01 (s, 3H), 1.54 – 1.44 (m, 2H), 0.96 (t, *J* = 7.5 Hz, 3H) ppm.
^{13}C NMR (176 MHz, $CDCl_3$) δ 171.3, 73.0, 45.6, 28.1, 23.4, 10.0 ppm.
$[\alpha]_D^{25.0}$ = +27.8 (*c* 0.36, $CHCl_3$).

(*S*)-1-(*N*-acetylbenzamido)butan-2-yl benzoate (1a-Bz)

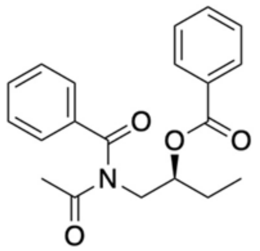

1a-Bz

1a-Bz was prepared according to **General Procedure H** using **1a** (5.2 mg, 0.040 mmol), DMAP (0.9 mg, 0.0074 mmol), triethylamine (0.02 mL, 0.14 mmol) and benzoyl chloride (0.01 mL, 0.086 mmol) in DCM (0.4 mL). Purification by silica gel column chromatography (elution with 0.5% MeOH in DCM followed by a second purification eluting with 20% acetone in petroleum ether) afforded **1a-Bz** (12.5 mg, 0.037 mmol, 93% yield, 97% ee).

^{1}H NMR (700 MHz, CD_3Cl) δ 7.91 – 7.86 (m, 2H), 7.53 – 7.46 (m, 4H), 7.40 – 7.33 (m, 4H), 5.41 – 5.39 (m, 1H), 4.17 (dd, *J* = 14.3, 8.7 Hz, 1H), 4.10 (dd, *J* = 14.3, 2.9 Hz, 1H), 2.05 (s, 3H), 1.78 – 1.66 (m, 2H), 0.97 (t, *J* = 7.5 Hz, 3H) ppm.
^{13}C NMR (176 MHz, CD_3Cl) δ 174.1, 173.3, 166.3, 135.3, 133.1, 132.7, 130.1, 129.7, 128.93, 128.86, 128.4, 74.7, 49.1, 26.3, 25.5, 9.6 ppm.

HRMS [M+H]$^+$ m/z calc'd for $[C_{20}H_{21}NO_4H]^+$ expect 340.1549; found 340.1548.
Chiral SFC Analysis: CHIRALPAK IK (CO_2:MeOH, 95:05, 2.5 mL min^{-1}, 40 °C, 228 nm) t_R = 5.4 (major), 6.2 (minor) minutes, 97% ee.

(*S*)-*N*-(2-hydroxybutyl)-*N*-methylacetamide (*N*-Me-1a)

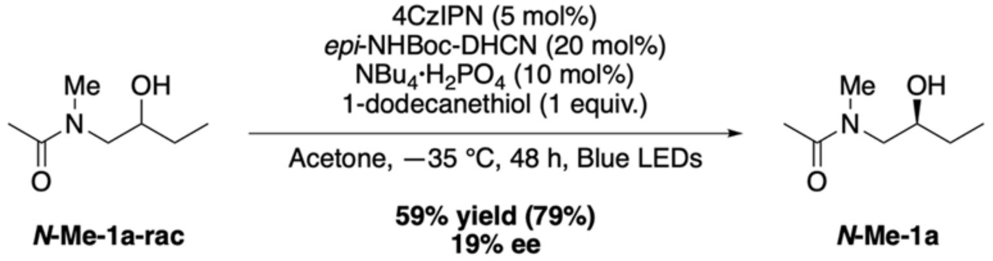

***N*-Me-1a** was prepared according to **General Procedure G** using amino alcohol-derived substrate ***N*-Me-1a-rac** (14.5 mg, 0.10 mmol). Purification by silica gel column chromatography (elution with 3% MeOH in DCM) afforded ***N*-Me-1a** as a pale yellow oil (8.6 mg, 0.059 mmol, 59% yield, 19% ee). Observed as two distinct rotamers (approx. 3:1). Combined signals for both rotamers are denoted *R*1+*R*2. Discernible signals are denoted *R*1 and *R*2.

^{1}H NMR (700 MHz, $CDCl_3$) δ 3.80 – 3.75 (m, 0.25H, *R*1), 3.75 – 3.70 (m, 0.75H, *R*2), 3.60 (dd, *J* = 14.2, 8.4 Hz, 0.75H, *R*2), 3.37 (dd, *J* = 14.4, 9.2 Hz, 0.25H, *R*1), 3.21 (dd, *J* = 14.2, 2.5 Hz, 0.75H, *R*2), 3.17 (dd, *J* = 14.4, 2.9 Hz, 0.25H, *R*1), 3.08 (s, 2.25H, *R*2), 2.96 (s, 0.75H, *R*1), 2.46 (br. s, 1H, *R*1+*R*2), 2.14 (br. s, 0.75H, *R*1), 2.12 (br. s, 2.25H, *R*2), 1.54 – 1.43 (m, 2H, *R*1+*R*2), 1.01 (t, *J* = 7.4 Hz, 0.75H, *R*1), 0.97 (t, *J* = 7.5 Hz, 2.25H, *R*2) ppm.
^{13}C NMR (176 MHz, $CDCl_3$) δ 173.2 (*R*2), 171.8 (*R*1), 72.8 (*R*2), 70.8 (*R*1), 56.9 (*R*1), 55.1 (*R*2), 38.4 (*R*2), 34.2 (*R*1), 28.5 (*R*2), 27.9 (*R*1), 21.9 (*R*2), 21.8 (*R*1), 10.1 (*R*1), 10.0 (*R*2) ppm.
Chiral GC Analysis: (CP-Chirasil-Dex CB Column 25 m × 0.25 mm × 0.25 μm, flow: 2.17 mL/min, 110 °C for 50 min; 110 °C to 200 °C at 11 °C/min): t_R = 27.3 (major), 30.4 (minor) minutes, 19% ee.

(*S*)-*N*-(2-hydroxypropyl)acetamide (1b)

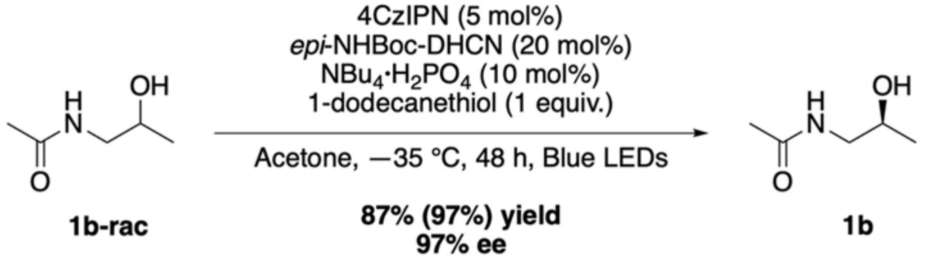

1b was prepared according to **General Procedure G** using amino alcohol-derived substrate **1b-rac** (11.7 mg, 0.10 mmol). Purification by silica gel column chromatography (elution with 7% MeOH in DCM) afforded **1b** as a pale yellow oil (10.2 mg, 0.087 mmol, 87% yield).

^{1}H NMR (700 MHz, $CDCl_3$) δ 6.35 – 6.03 (m, 1H), 3.96 – 3.86 (m, 1H), 3.47 – 3.38 (m, 1H), 3.14 – 3.04 (m, 1H), 2.60 (br. s, 1H) (overlaps with H_2O peak), 2.01 (s, 3H), 1.20 – 1.16 (m, 3H) ppm.
^{13}C NMR (176 MHz, $CDCl_3$) δ 171.5, 67.5, 47.3, 23.3, 21.1 (2C) ppm.
$[\alpha]_D^{25.0}$ = +28.8 (*c* 0.43, $CHCl_3$) (**$[\alpha]_D^{25.0}$** = –35.9 (*c* 1.0, $CHCl_3$) for (*R*)-enantiomer synthesized from commercially available (*R*)-1-aminopropan-2-ol).
$[\alpha]_D^{25.0}$ = +19.5 (*c* 0.44, EtOH) (lit. **$[\alpha]_D^{20}$** = –4.0 (*c* 0.4, EtOH) for (*R*)-enantiomer).[25]

(*S*)-1-(*N*-acetylbenzamido)propan-2-yl benzoate (1b-Bz)

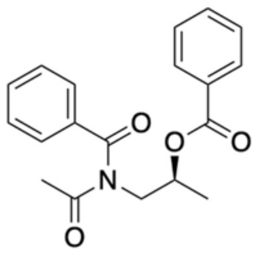

1b-Bz

1b-Bz was prepared according to **General Procedure H** using **1b** (5.1 mg, 0.044 mmol), DMAP (0.9 mg, 0.0074 mmol), triethylamine (0.02 mL, 0.14 mmol) and benzoyl chloride (0.01 mL, 0.086 mmol) in DCM (0.4 mL). Purification by silica gel column chromatography (elution with 0.5% MeOH in DCM followed by a second purification eluting with 20% acetone in petroleum ether) afforded **1b-Bz** (13.1 mg, 0.039 mmol, 89% yield, 97% ee).

^{1}H NMR (700 MHz, CD_3Cl) δ 7.89 – 7.83 (m, 2H), 7.57 – 7.52 (m, 2H), 7.51 – 7.47 (m, 2H), 7.38 – 7.32 (m, 4H), 5.49 – 5.42 (m, 1H), 4.19 (dd, *J* = 14.3, 8.8 Hz, 1H), 4.07 (dd, *J* = 14.3, 3.0 Hz, 1H), 2.04 (s, 3H), 1.36 (d, *J* = 6.4 Hz, 3H) ppm.
^{13}C NMR (176 MHz, CD_3Cl) δ 174.2, 173.3, 166.1, 135.3, 133.1, 132.9, 130.1, 129.7, 129.0, 128.9, 128.3, 70.5, 50.4, 26.3, 18.0 ppm.
HRMS $[M+H]^+$ m/z calc'd for $[C_{19}H_{19}NO_4H]^+$ expect 326.1392; found 326.1389.
Chiral SFC Analysis: CHIRALPAK IK (CO_2:MeOH, 95:05, 2.5 mL min^{-1}, 40 °C, 228 nm) t_R = 6.1 (major), 6.5 (minor) minutes, 97% ee.

(*S*)-*N*-(2-hydroxyoctyl)acetamide (1c)

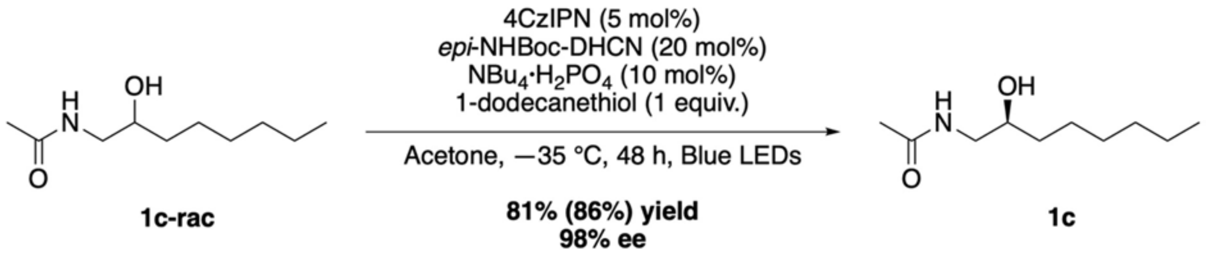

1c was prepared according to **General Procedure G** using amino alcohol-derived substrate **1c-rac** (18.7 mg, 0.10 mmol). Purification by silica gel column chromatography (elution with

1.5% 7N ammonia in MeOH, 3% MeOH, 95.5% DCM) afforded **1c** as a white solid (15.2 mg, 0.081 mmol, 81% yield).

^{1}H NMR (700 MHz, $CDCl_3$) δ 5.99 (br. s, 1H), 3.73 – 3.66 (m, 1H), 3.48 (ddd, *J* = 13.9, 6.6, 2.9 Hz, 1H), 3.09 (ddd, *J* = 13.9, 8.0, 5.2 Hz, 1H), 2.49 (br. s, 1H), 2.01 (s, 3H), 1.49 – 1.37 (m, 3H), 1.36 – 1.20 (m, 7H), 0.89 (t, *J* = 7.0 Hz, 3H) ppm.
^{13}C NMR (176 MHz, $CDCl_3$) δ 171.3, 71.6, 46.0, 35.2, 31.9, 29.4, 25.6, 23.4, 22.7, 14.2 ppm.
$[\alpha]_D^{25.0}$ = +18.0 (*c* 0.63, $CHCl_3$).

(*S*)-1-(*N*-acetylbenzamido)octan-2-yl benzoate (1c-Bz)

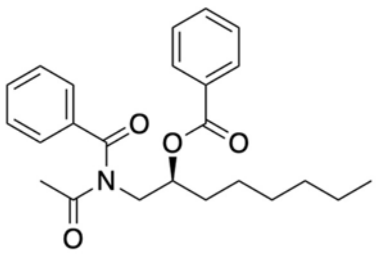

1c-Bz

1c-Bz was prepared according to **General Procedure H** using **1c** (6.9 mg, 0.037 mmol), DMAP (0.9 mg, 0.0074 mmol), triethylamine (0.02 mL, 0.14 mmol) and benzoyl chloride (0.01 mL, 0.086 mmol) in DCM (0.4 mL). Purification by silica gel column chromatography (elution with 0.5% MeOH in DCM followed by a second purification eluting with 10% acetone in petroleum ether) afforded **1c-Bz** (9.3 mg, 0.024 mmol, 64% yield, 98% ee).

^{1}H NMR (700 MHz, CD_3Cl) δ 7.91 – 7.86 (m, 2H), 7.53 – 7.46 (m, 4H), 7.40 – 7.32 (m, 4H), 5.46 – 5.40 (m, 1H), 4.16 (dd, *J* = 14.3, 8.7 Hz, 1H), 4.10 (dd, *J* = 14.3, 2.8 Hz, 1H), 2.05 (s, 3H), 1.71 – 1.62 (m, 2H), 1.40 – 1.33 (m, 2H), 1.33 – 1.27 (m, 2H), 1.27 – 1.21 (m, 4H), 0.85 (t, *J* = 7.1 Hz, 3H) ppm.
^{13}C NMR (176 MHz, CD_3Cl) δ 174.1, 173.3, 166.3, 135.3, 133.0, 132.7, 130.2, 129.8, 128.94, 128.85, 128.4, 73.6, 49.4, 32.5, 31.7, 29.2, 26.3, 25.2, 22.7, 14.2 ppm.
HRMS $[M+H]^+$ m/z calc'd for $[C_{24}H_{29}NO_4H]^+$ expect 396.2175; found 396.2161.
Chiral SFC Analysis: CHIRALPAK IK (CO_2:MeOH, 96:04, 2.5 mL min^{-1}, 40 °C, 228 nm) t_R = 6.3 (major), 8.2 (minor) minutes, 98% ee.

(*S*)-*N*-(2-hydroxy-7-methyloctyl)acetamide (1d)

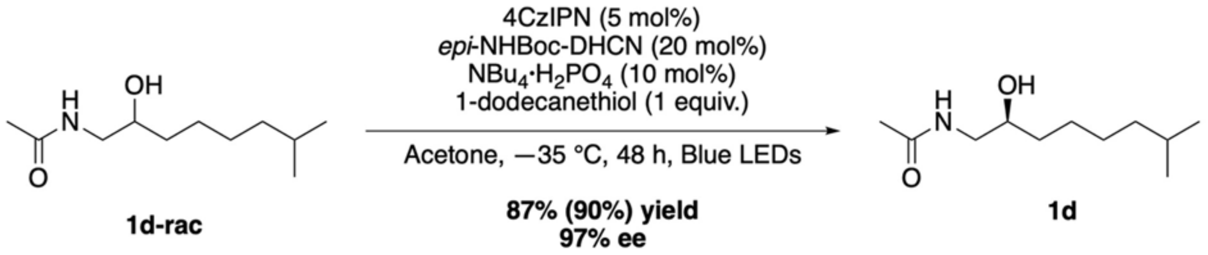

1d was prepared according to **General Procedure G** using amino alcohol-derived substrate **1d-rac** (20.1 mg, 0.10 mmol). Purification by silica gel column chromatography (elution with 1% 7N ammonia in MeOH, 2% MeOH, 97% DCM) afforded **1d** as a pale yellow oil (17.6 mg, 0.087 mmol, 87% yield).

^{1}H NMR (700 MHz, $CDCl_3$) δ 6.07 (br. s, 1H), 3.75 – 3.67 (m, 1H), 3.47 (ddd, *J* = 14.0, 6.4, 2.8 Hz, 1H), 3.09 (ddd, *J* = 14.0, 8.1, 5.2 Hz, 1H), 2.3 (br. s, 1H), 2.01 (s, 3H), 1.56 – 1.48 (m, 1H), 1.48 – 1.36 (m, 3H), 1.35 – 1.23 (m, 3H), 1.20 – 1.13 (m, 2H), 0.86 (d, *J* = 6.6 Hz, 6H) ppm.
^{13}C NMR (176 MHz, $CDCl_3$) δ 171.4, 71.5, 46.0, 39.0, 35.2, 28.0, 27.5, 25.9, 23.3, 22.7 (2C) ppm.
$[\alpha]_D^{25.0}$ = +30.4 (*c* 0.77, $CHCl_3$).

(*S*)-1-(*N*-acetylbenzamido)-7-methyloctan-2-yl benzoate (1d-Bz)

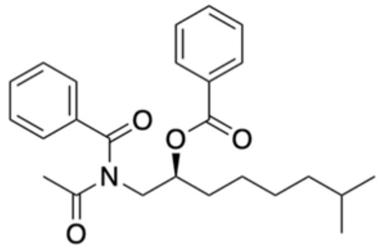

1d-Bz

1d-Bz was prepared according to **General Procedure H** using **1d** (8.8 mg, 0.044 mmol), DMAP (0.9 mg, 0.0074 mmol), triethylamine (0.02 mL, 0.14 mmol) and benzoyl chloride (0.01 mL, 0.086 mmol) in DCM (0.4 mL). Purification by silica gel column chromatography (elution with 0.5% MeOH in DCM followed by a second purification eluting with 5% acetone in petroleum ether) afforded **1d-Bz** (11.2 mg, 0.027 mmol, 63% yield, 97% ee).

^{1}H NMR (700 MHz, CD_3Cl) δ 7.91 – 7.86 (m, 2H), 7.53 – 7.45 (m, 4H), 7.40 – 7.32 (m, 4H), 5.46 – 5.39 (m, 1H), 4.16 (dd, *J* = 14.3, 8.7 Hz, 1H), 4.10 (dd, *J* = 14.3, 2.8 Hz, 1H), 2.05 (s, 3H), 1.72 – 1.62 (m, 2H), 1.48 (app. n, *J* = 6.7 Hz, 1H), 1.39 – 1.32 (m, 2H), 1.32 – 1.24 (m, 2H), 1.16 – 1.10 (m, 2H), 0.831 (d, *J* = 6.6 Hz, 3H), 0.829 (d, *J* = 6.6 Hz, 3H) ppm.
^{13}C NMR (176 MHz, CD_3Cl) δ 174.1, 173.4, 166.3, 135.3, 133.0, 132.7, 130.2, 129.8, 128.94, 128.85, 128.4, 73.6, 49.4, 38.9, 32.5, 28.0, 27.3, 26.3, 25.5, 22.73, 22.69 ppm.
HRMS $[M+H]^+$ m/z calc'd for $[C_{25}H_{31}NO_4H]^+$ expect 410.2331; found 410.2341.

Chiral SFC Analysis: CHIRALPAK IK (CO_2:MeOH, 96:04, 2.5 mL min^{-1}, 40 °C, 228 nm) t_R = 6.1 (major), 7.9 (minor) minutes, 97% ee.

(*S*)-*N*-(4-cyclobutyl-2-hydroxybutyl)acetamide (1e)

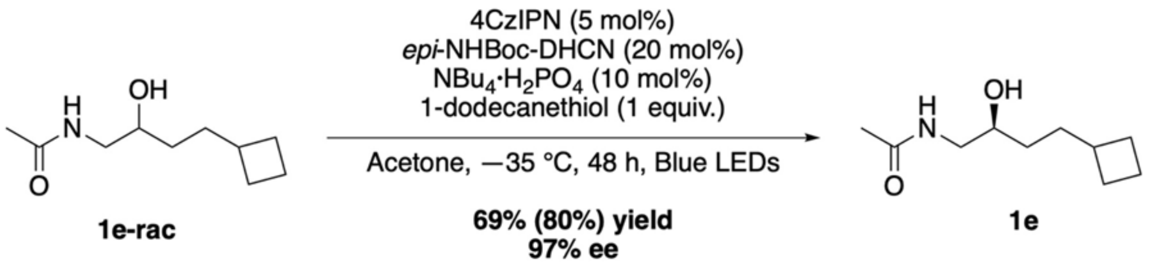

1e was prepared according to **General Procedure G** using amino alcohol-derived substrate **1e-rac** (18.5 mg, approx. 0.10 mmol). Purification by silica gel column chromatography (elution with 1% 7N ammonia in MeOH, 2% MeOH, 97% DCM) afforded **1e** as a pale yellow oil (12.8 mg, approx. 0.069 mmol, approx. 69% yield). Like **1e-rac**, **1e** contains unknown impurity, and so approximate yield is given. **1e** contains the same amount of impurity as **1e-rac**. It is unclear whether this impurity is due to degradation of **1e** during purification by silica gel column chromatography or due to impurity being inseparable from **1e** in the eluent used.

^{1}H NMR (700 MHz, $CDCl_3$) δ 5.97 (br. s, 1H), 3.70 – 3.63 (m, 1H), 3.47 (ddd, *J* = 14.0, 6.6, 2.9 Hz, 1H), 3.08 (ddd, *J* = 14.0, 8.1, 5.3 Hz, 1H), 2.50 (br. s, 1H), 2.24 (app. hept, *J* = 7.7 Hz, 1H), 2.06 – 1.97 (m, 2H), 2.01 (s, 3H), 1.88 – 1.81 (m, 1H), 1.81 – 1.76 (m, 1H), 1.61 – 1.54 (m, 2H), 1.53 – 1.46 (m, 1H), 1.44 – 1.38 (m, 1H), 1.38 – 1.32 (m, 2H) ppm.
^{13}C NMR (176 MHz, $CDCl_3$) δ 171.3, 71.6, 46.0, 36.0, 32.81, 32.79, 28.4, 28.3, 23.4, 18.5 ppm.
$[\alpha]_D^{25.0}$ = +15.1 (*c* 0.52, $CHCl_3$).

(*S*)-1-(*N*-acetylbenzamido)-4-cyclobutylbutan-2-yl benzoate (1e-Bz)

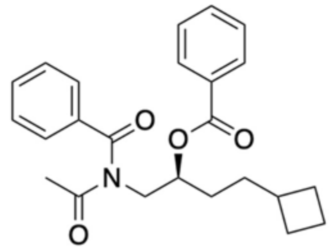

1e-Bz

1e-Bz was prepared according to **General Procedure H** using **1e** (6.4 mg, approx. 0.035 mmol), DMAP (0.8 mg, 0.0065 mmol), triethylamine (0.02 mL, 0.14 mmol) and benzoyl chloride (0.01 mL, 0.086 mmol) in DCM (0.4 mL). Purification by silica gel column chromatography (elution with 0.5% MeOH in DCM followed by a second purification eluting

with 10% acetone in petroleum ether) afforded **1e-Bz** (8.8 mg, 0.022 mmol, approx. 65% yield, 97% ee).

^{1}H NMR (700 MHz, CD_3Cl) δ 7.91 – 7.87 (2H, m), 7.54 – 7.46 (m, 4H), 7.39 – 7.32 (m, 4H), 5.46 – 5.37 (m, 1H), 4.15 (dd, *J* = 14.3, 8.7 Hz, 1H), 4.09 (dd, *J* = 14.3, 2.8 Hz, 1H), 2.23 (app. hept, *J* = 7.8 Hz, 1H), 2.04 (s, 3H), 2.03 – 1.97 (m, 2H), 1.86 – 1.79 (m, 1H), 1.79 – 1.72 (m, 1H), 1.60 – 1.51 (m, 4H) (overlaps with H_2O peak), 1.48 – 1.43 (m, 2H) ppm.
^{13}C NMR (176 MHz, CD_3Cl) δ 174.1, 173.3, 166.2, 135.3, 133.1, 132.7, 130.1, 129.8, 128.94, 128.86, 128.4, 73.5, 49.4, 35.8, 32.3, 30.1, 28.3, 28.2, 26.3, 18.5 ppm.
HRMS $[M+H]^+$ m/z calc'd for $[C_{24}H_{27}NO_4H]^+$ expect 394.2018; found 394.2002.
Chiral SFC Analysis: CHIRALPAK IK (CO_2:MeOH, 95:05, 2.5 mL min^{-1}, 40 °C, 228 nm) t_R = 6.7 (major), 8.6 (minor) minutes, 97% ee.

(*S*)-*N*-(5-cyclohexyl-2-hydroxypentyl)acetamide (1f)

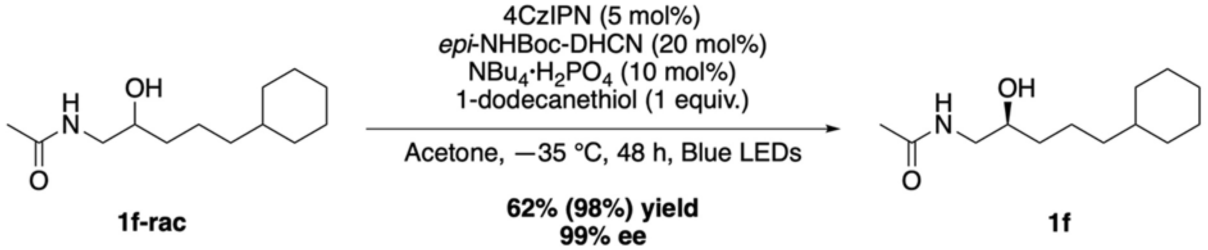

1f was prepared according to **General Procedure G** using amino alcohol-derived substrate **1f-rac** (22.7 mg, 0.10 mmol). Purification by silica gel column chromatography (elution with 1% 7N ammonia in MeOH, 2% MeOH, 97% DCM) afforded **1f** as a pale yellow oil that slowly forms a white solid (14.2 mg, 0.062 mmol, 62% yield).

^{1}H NMR (700 MHz, $CDCl_3$) δ 6.02 (br. s, 1H), 3.74 – 3.66 (m, 1H), 3.48 (ddd, *J* = 13.9, 6.4, 2.8 Hz, 1H), 3.09 (ddd, *J* = 13.9, 8.1, 5.2 Hz, 1H), 2.17 (br. s, 1H), 2.02 (s, 3H), 1.71 – 1.65 (m, 4H), 1.65 – 1.60 (m, 1H), 1.47 – 1.39 (m, 3H), 1.36 – 1.29 (m, 1H), 1.24 – 1.09 (m, 6H), 0.90 – 0.81 (m, 2H) ppm.
^{13}C NMR (176 MHz, $CDCl_3$) δ 171.3, 71.6, 46.0, 37.7, 37.5, 35.5, 33.5, 26.8, 26.5, 23.4, 22.9 ppm.
$[\alpha]_D^{25.0}$ = +15.7 (*c* 0.62, $CHCl_3$).

(*S*)-1-(*N*-acetylbenzamido)-5-cyclohexylpentan-2-yl benzoate (1f-Bz)

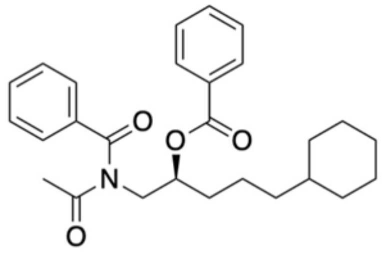

1f-Bz

1f-Bz was prepared according to **General Procedure H** using **1f** (7.1 mg, 0.031 mmol), DMAP (0.8 mg, 0.0065 mmol), triethylamine (0.02 mL, 0.14 mmol) and benzoyl chloride (0.01 mL, 0.086 mmol) in DCM (0.4 mL). Purification by silica gel column chromatography (elution with 0.5% MeOH in DCM followed by a second purification eluting with 5% acetone in petroleum ether) afforded **1f-Bz** (7.1 mg, 0.016 mmol, 53% yield, 99% ee).

^{1}H NMR (700 MHz, CD_3Cl) δ 7.91 – 7.86 (m, 2H), 7.54 – 7.46 (m, 4H), 7.40 – 7.32 (m, 4H), 5.47 – 5.38 (m, 1H), 4.16 (dd, *J* = 14.3, 8.7 Hz, 1H), 4.10 (dd, *J* = 14.3, 2.8 Hz, 1H), 2.04 (s, 3H), 1.69 – 1.60 (m, 7H), 1.42 – 1.33 (m, 2H), 1.22 – 1.07 (m, 6H), 0.86 – 0.78 (m, 2H) ppm.
^{13}C NMR (176 MHz, CD_3Cl) δ 174.1, 173.4, 166.3, 135.3, 133.0, 132.7, 130.2, 129.8, 128.94, 128.86, 128.4, 73.6, 49.4, 37.5, 37.3, 33.5, 33.4, 32.8, 26.8, 26.49, 26.48, 26.3, 22.5 ppm.
HRMS $[M+H]^+$ m/z calc'd for $[C_{27}H_{33}NO_4H]^+$ expect 436.2488; found 436.2489.
Chiral SFC Analysis: CHIRALPAK IK (CO_2:MeOH, 95:05, 2.5 mL min^{-1}, 40 °C, 228 nm) t_R = 8.5 (major), 11.1 (minor) minutes, 99% ee.

(*S*)-*N*-(2-hydroxy-3-phenylpropyl)acetamide (1g)

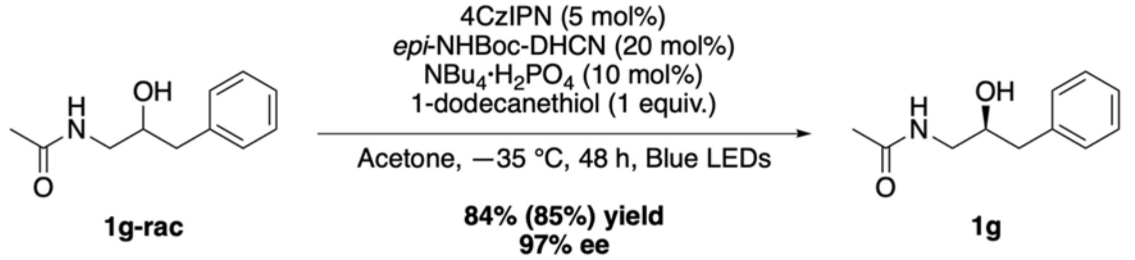

1g was prepared according to **General Procedure G** using amino alcohol-derived substrate **1g-rac** (19.3 mg, 0.10 mmol). Purification by silica gel column chromatography (elution with 1% 7N ammonia in MeOH, 2% MeOH, 97% DCM) afforded **1g** as a colorless waxy solid (16.2 mg, 0.084 mmol, 84% yield, 97% ee).

^{1}H NMR (500 MHz, $CDCl_3$) δ 7.31 (t, *J* = 7.4 Hz, 2H), 7.23 (t, *J* = 7.4 Hz, 1H), 7.20 (d, *J* = 7.4 Hz, 2H), 6.00 (br. s, 1H), 3.98 – 3.89 (m, 1H), 3.53 (ddd, *J* = 14.0, 6.6, 2.9 Hz, 1H), 3.15 (ddd, *J* = 14.0, 7.8, 5.1 Hz, 1H), 2.80 (dd, *J* = 13.7, 5.3 Hz, 1H), 2.78 (br. s, 1H), 2.72 (dd, *J* = 13.7, 8.0 Hz, 1H), 1.99 (s, 3H) ppm.
^{13}C NMR (126 MHz, $CDCl_3$) δ 171.3, 137.7, 129.5, 128.8, 126.9, 72.3, 45.3, 41.6, 23.3 ppm.
$[\alpha]_D^{25.0}$ = +20.5 (*c* 1.31, $CHCl_3$).

Chiral SFC Analysis: CHIRALPAK IJ (CO_2:MeOH, 95:05, 2.5 mL min^{-1}, 40 °C, 203 nm) t_R = 5.7 (minor), 7.3 (major) minutes, 97% ee.

(*S*)-*N*-(2-hydroxy-4-phenylbutyl)acetamide (1h)

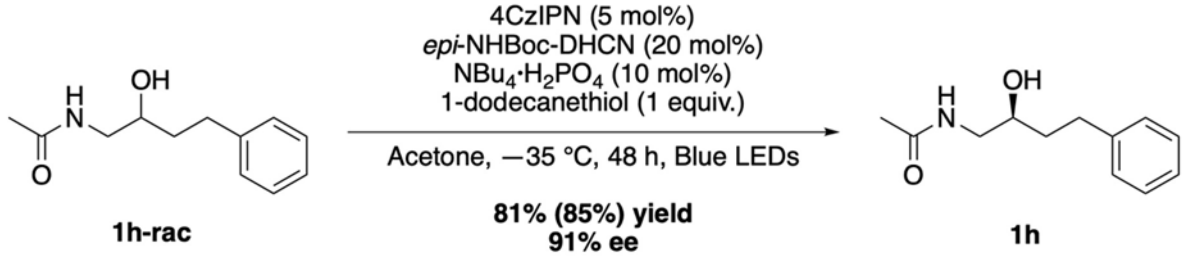

1h was prepared according to **General Procedure G** using amino alcohol-derived substrate **1h-rac** (20.7 mg, 0.10 mmol). Purification by silica gel column chromatography (elution with 1% 7N ammonia in MeOH, 2% MeOH, 97% DCM) afforded **1h** as a colorless oil that slowly forms a white solid (16.7 mg, 0.081 mmol, 81% yield, 91% ee).

^{1}H NMR (500 MHz, $CDCl_3$) δ 7.31 – 7.26 (m, 2H), 7.22 – 7.16 (m, 3H), 6.05 (br. s, 1H), 3.77 – 3.66 (m, 1H), 3.45 (ddd, *J* = 14.0, 6.4, 2.9 Hz, 1H), 3.17 (ddd, *J* = 14.0, 7.8, 5.5 Hz, 1H), 2.96 (br. s, 1H), 2.79 (ddd, *J* = 13.9, 8.7, 6.2 Hz, 1H), 2.69 (ddd, *J* = 13.9, 8.2, 7.9 Hz, 1H), 1.99 (s, 3H), 1.81 – 1.72 (m, 2H) ppm.
^{13}C NMR (126 MHz, $CDCl_3$) δ 171.5, 141.7, 128.6, 128.5, 126.1, 70.9, 46.1, 36.7, 31.9, 23.3 ppm.
$[\alpha]_D^{25.0}$ = +3.1 (*c* 1.03, $CHCl_3$).
Chiral SFC Analysis: CHIRALPAK IK (CO_2:MeOH, 93:07, 2.5 mL min^{-1}, 40 °C, 203 nm) t_R = 11.5 (major), 12.8 (minor) minutes, 91% ee.

(*S*)-*N*-(2-hydroxy-5-phenylpentyl)acetamide (1i)

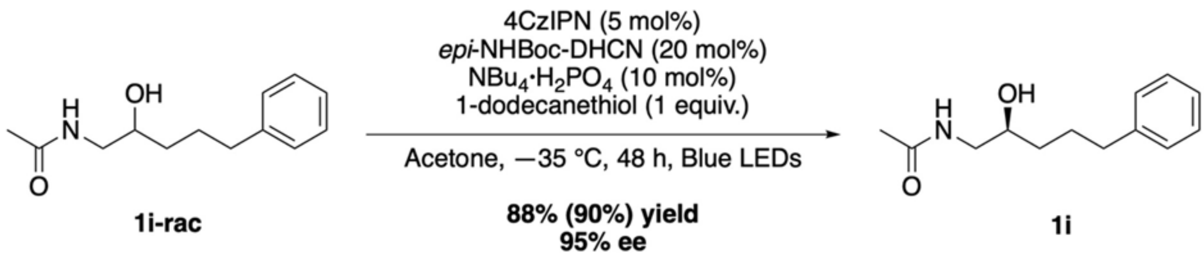

1i was prepared according to **General Procedure G** using amino alcohol-derived substrate **1i-rac** (22.1 mg, 0.10 mmol). Purification by silica gel column chromatography (elution with 1% 7N ammonia in MeOH, 2% MeOH, 97% DCM) afforded **1i** as a colorless oil (19.5 mg, 0.088 mmol, 88% yield, 95% ee).

^{1}H NMR (500 MHz, $CDCl_3$) δ 7.27 (t, *J* = 7.5 Hz, 2H), (overlaps with $CHCl_3$ peak), 7.21 – 7.14 (m, 3H), 5.98 (br. s, 1H), 3.76 – 3.65 (m, 1H), 3.44 (ddd, *J* = 14.0, 6.5, 2.9 Hz, 1H), 3.09 (ddd,

J = 14.0, 7.9, 5.2 Hz, 1H), 2.72 (d, *J* = 4.0 Hz, 1H), 2.63 (t, *J* = 7.6 Hz, 2H), 1.99 (s, 3H), 1.84 – 1.71 (m, 1H) (overlaps with H_2O peak), 1.71 – 1.61 (m, 1H), 1.53 – 1.43 (m, 2H) ppm.
^{13}C NMR (126 MHz, $CDCl_3$) δ 171.4, 142.2, 128.54, 128.48, 126.0, 71.4, 46.0, 35.8, 34.6, 27.3, 23.3 ppm.
$[\alpha]_D^{25.0}$ = +14.8 (*c* 1.64, $CHCl_3$).
Chiral SFC Analysis: CHIRALPAK IJ (CO_2:MeOH, 95:05, 2.5 mL min^{-1}, 40 °C, 204 nm) t_R = 7.9 (minor), 9.2 (major) minutes, 95% ee.

(*S*)-*N*-(2-hydroxy-6-phenylhexyl)acetamide (1j)

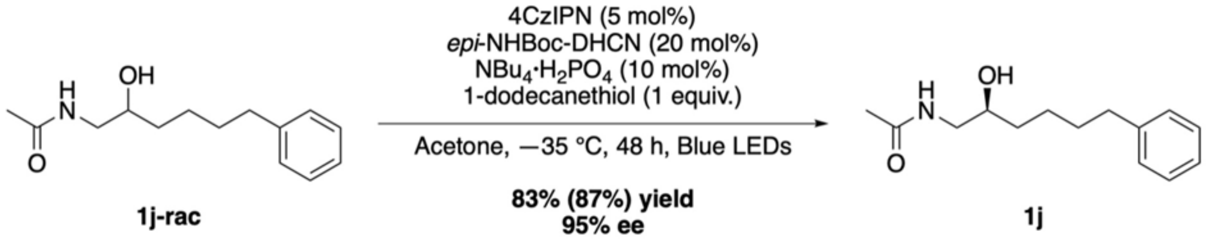

1j-rac **1j**

1j was prepared according to **General Procedure G** using amino alcohol-derived substrate **1j-rac** (23.5 mg, 0.10 mmol). Purification by silica gel column chromatography (elution with 1.5% 7N ammonia in MeOH, 3% MeOH, 95.5% DCM) afforded **1j** as a colorless oil that slowly forms a white solid (19.5 mg, 0.083 mmol, 83% yield, 95% ee).

^{1}H NMR (700 MHz, $CDCl_3$) δ 7.27 (t, *J* = 7.6 Hz, 2H) (overlaps with $CHCl_3$ peak), 7.21 – 7.13 (m, 3H), 6.05 (br. s, 1H), 3.73 – 3.63 (m, 1H), 3.45 (ddd, *J* = 13.9, 6.5, 2.9 Hz, 1H), 3.09 (ddd, *J* = 13.9, 7.9, 5.2 Hz, 1H), 2.77 (d, *J* = 3.4 Hz, 1H), 2.61 (t, *J* = 7.7 Hz, 2H), 2.00 (s, 3H), 1.69 – 1.59 (m, 2H), 1.52 – 1.44 (m, 3H), 1.41 – 1.33 (m, 1H) ppm.
^{13}C NMR (176 MHz, $CDCl_3$) δ 171.4, 142.5, 128.5, 128.4, 125.9, 71.4, 46.0, 36.0, 35.0, 31.5, 25.3, 23.3 ppm.
$[\alpha]_D^{25.0}$ = +13.0 (*c* 1.68, $CHCl_3$).
Chiral SFC Analysis: CHIRALPAK IK (CO_2:MeOH, 90:10, 2.5 mL min^{-1}, 40 °C, 203 nm) t_R = 7.5 (major), 8.2 (minor) minutes, 95% ee.

(*S*)-*N*-(2-hydroxy-7-phenylheptyl)acetamide (1k)

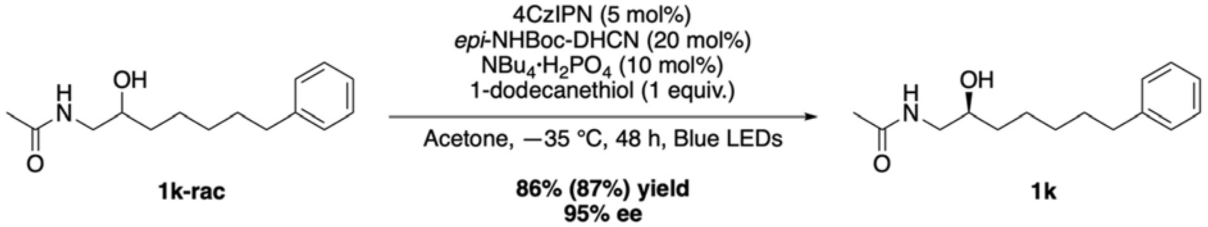

1k-rac **1k**

1k was prepared according to **General Procedure G** using amino alcohol-derived substrate **1k-rac** (24.9 mg, 0.10 mmol). Purification by silica gel column chromatography (elution with 1% 7N ammonia in MeOH, 2% MeOH, 97% DCM) afforded **1k** as a white solid (21.5 mg, 0.086 mmol, 86% yield, 95% ee).

^{1}H NMR (500 MHz, $CDCl_3$) δ 7.30 – 7.24 (m, 2H) (overlaps with $CHCl_3$ peak), 7.20 – 7.14 (m, 3H), 6.00 (br. s, 1H), 3.74 – 3.63 (m, 1H), 3.46 (ddd, *J* = 14.0, 6.5, 2.9 Hz, 1H), 3.09 (ddd, *J* = 14.0, 8.1, 5.3 Hz, 1H), 2.63 (d, *J* = 4.7 Hz, 1H), 2.60 (t, *J* = 7.7 Hz, 2H), 2.00 (s, 3H), 1.62 (app. quint, *J* = 7.6 Hz, 2H), 1.51 – 1.41 (m, 3H), 1.41 – 1.29 (m, 3H) ppm.
^{13}C NMR (126 MHz, $CDCl_3$) δ 171.3, 142.8, 128.5, 128.4, 125.8, 71.5, 46.0, 36.0, 35.1, 31.5, 29.3, 25.5, 23.4 ppm.
$[\alpha]_D^{25.0}$ = +11.8 (*c* 1.73, $CHCl_3$).
Chiral SFC Analysis: CHIRALPAK IK (CO_2:MeOH, 90:10, 2.5 mL min^{-1}, 40 °C, 214 nm) t_R = 8.0 (major), 8.8 (minor) minutes, 95% ee.

***tert*-butyl (*S*)-(5-acetamido-4-hydroxypentyl)carbamate (1l)**

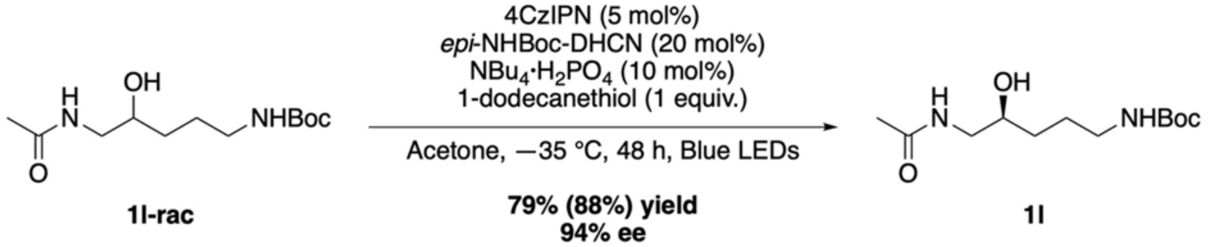

1l was prepared according to **General Procedure G** using amino alcohol-derived substrate **1l-rac** (26.0 mg, 0.10 mmol). Purification by silica gel column chromatography (elution with 5% MeOH in DCM) afforded **1l** as a colorless oil (20.6 mg, 0.079 mmol, 79% yield).

^{1}H NMR (700 MHz, $CDCl_3$) δ 6.24 (br. s, 1H), 4.74 (br. s, 1H), 3.76 – 3.68 (m, 1H), 3.44 (ddd, *J* = 14.0, 6.4, 3.0 Hz, 1H), 3.23 – 3.14 (m, 1H), 3.14 – 3.05 (m, 2H), 2.78 (br. s, 1H), 2.01 (s, 3H), 1.67 – 1.60 (m, 1H), 1.60 – 1.53 (m, 1H), 1.53 – 1.44 (m, 2H), 1.43 (s, 9H) ppm.
^{13}C NMR (176 MHz, $CDCl_3$) δ 171.5, 156.6, 79.5, 71.1, 46.1, 40.3, 31.7, 28.5, 26.5, 23.3 ppm.
$[\alpha]_D^{25.0}$ = +10.1 (*c* 1.05, $CHCl_3$).

(*S*)-1-(*N*-acetylbenzamido)-5-((*tert*-butoxycarbonyl)amino)pentan-2-yl benzoate (1l-Bz)

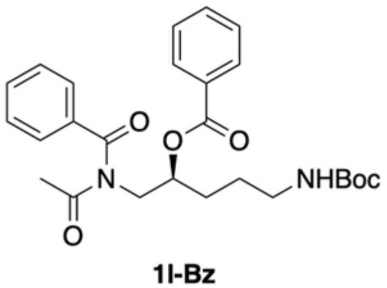

1l-Bz

1l-Bz was prepared according to **General Procedure H** using **1l** (10.3 mg, 0.040 mmol), DMAP (0.9 mg, 0.0074 mmol), triethylamine (0.02 mL, 0.14 mmol) and benzoyl chloride (0.01 mL, 0.086 mmol) in DCM (0.4 mL). Purification by silica gel column chromatography (elution with 1% MeOH in DCM followed by a second purification eluting with 15% acetone in petroleum ether) afforded **1l-Bz** (11.9 mg, 0.025 mmol, 63% yield, 94% ee).

^{1}H NMR (700 MHz, CD_3Cl) δ 7.89 – 7.85 (m, 2H), 7.54 – 7.46 (m, 4H), 7.40 – 7.32 (m, 4H), 5.46 – 5.38 (m, 1H), 4.56 (br. s, 1H), 4.17 (dd, *J* = 14.3, 8.6 Hz, 1H), 4.09 (dd, *J* = 14.3, 2.6 Hz, 1H), 3.20 – 3.02 (m, 2H), 2.04 (s, 3H), 1.75 – 1.65 (m, 2H), 1.64 – 1.53 (m, 2H) (overlaps with H_2O peak), 1.42 (s, 9H) ppm.
^{13}C NMR (176 MHz, CD_3Cl) δ 174.0, 173.4, 166.2, 156.1, 135.2, 133.2, 132.8, 129.9, 129.8, 128.94, 128.90, 128.4, 79.3, 73.1, 49.2, 40.3, 29.7, 28.5, 26.3, 26.0 ppm.
HRMS $[M+H]^+$ m/z calc'd for $[C_{26}H_{32}N_2O_6H]^+$ expect 469.2339; found 469.2345.
Chiral SFC Analysis: CHIRALPAK IK (CO_2:MeOH, 90:10, 2.5 mL min^{-1}, 40 °C, 229 nm) t_R = 8.3 (major), 10.1 (minor) minutes, 94% ee.

ethyl (*S*)-7-acetamido-6-hydroxyheptanoate (1m)

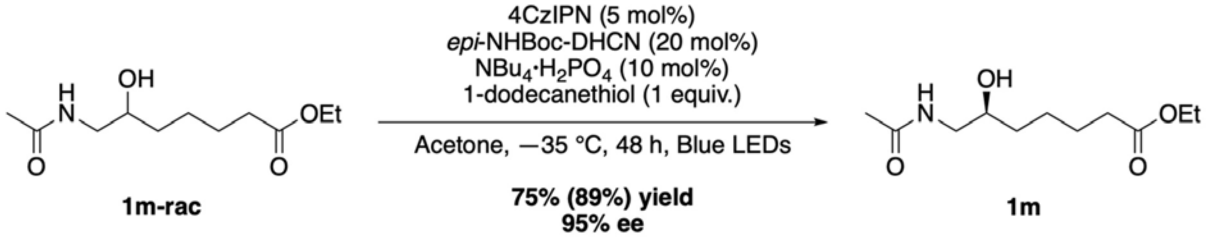

1m was prepared according to **General Procedure G** using amino alcohol-derived substrate **1m-rac** (23.1 mg, 0.10 mmol). Purification by silica gel column chromatography (elution with 6% MeOH in DCM) afforded **1m** as an off-white waxy solid (17.3 mg, 0.075 mmol, 75% yield).

^{1}H NMR (700 MHz, $CDCl_3$) δ 6.08 (br. s, 1H), 4.19 (q, *J* = 7.1 Hz, 2H), 3.75 – 3.69 (m, 1H), 3.46 (ddd, *J* = 13.9, 6.4, 2.8 Hz, 1H), 3.11 (ddd, *J* = 13.9, 8.0, 5.4 Hz, 1H), 2.52 (br. s, 1H), 2.31 (t, *J* = 7.4 Hz, 2H), 2.01 (s, 3H), 1.70 – 1.58 (m, 2H), 1.51 – 1.43 (m, 3H), 1.42 – 1.32 (m, 1H), 1.25 (t, *J* = 7.1 Hz, 3H) ppm.
^{13}C NMR (176 MHz, $CDCl_3$) δ 173.9, 171.4, 71.2, 60.5, 46.0, 34.6, 34.2, 25.0, 24.7, 23.3, 14.4 ppm.
$[\alpha]_D^{25.0}$ = +18.6 (*c* 0.79, $CHCl_3$).

(*S*)-1-(*N*-acetylbenzamido)-7-ethoxy-7-oxoheptan-2-yl benzoate (1m-Bz)

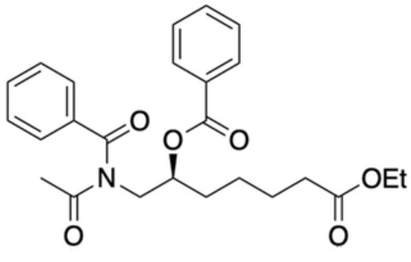

1m-Bz

1m-Bz was prepared according to **General Procedure H** using **1m** (8.6 mg, 0.037 mmol), DMAP (0.9 mg, 0.0074 mmol), triethylamine (0.02 mL, 0.14 mmol) and benzoyl chloride (0.01 mL, 0.086 mmol) in DCM (0.4 mL). Purification by silica gel column chromatography (elution

with 1% MeOH in DCM followed by a second purification eluting with 20% acetone in petroleum ether) afforded **1m-Bz** (12.2 mg, 0.028 mmol, 75% yield, 95% ee).

^{1}H NMR (700 MHz, CD_3Cl) δ 7.90 – 7.84 (m, 2H), 7.54 – 7.50 (m, 4H), 7.41 – 7.31 (m, 4H), 5.46 – 5.39 (m, 1H), 4.16 (dd, *J* = 14.3, 8.7 Hz, 1H), 4.09 (dd, *J* = 14.3, 3.1 Hz, 1H), 4.08 (q, *J* = 7.1 Hz, 2H), 2.27 (t, *J* = 7.5 Hz, 2H), 2.04 (s, 3H), 1.73 – 1.67 (m, 2H), 1.67 – 1.61 (m, 2H), 1.46 – 1.37 (m, 2H), 1.21 (t, *J* = 7.1 Hz, 3H) ppm.
^{13}C NMR (176 MHz, CD_3Cl) δ 174.1, 173.6, 173.3, 166.2, 135.3, 133.1, 132.8, 130.0, 129.8, 128.93, 128.87, 128.4, 73.3, 60.4, 49.3, 34.2, 32.2, 26.3, 24.9, 24.8, 14.3 ppm.
HRMS $[M+H]^+$ m/z calc'd for $[C_{25}H_{29}NO_6H]^+$ expect 440.2073; found 440.2064.
Chiral SFC Analysis: CHIRALPAK IK (CO_2:MeOH, 93:07, 2.5 mL min^{-1}, 40 °C, 228 nm) t_R = 7.7 (major), 9.3 (minor) minutes, 95% ee.

(*S*)-*N*-(5-((*tert*-butyldimethylsilyl)oxy)-2-hydroxypentyl)acetamide (1n)

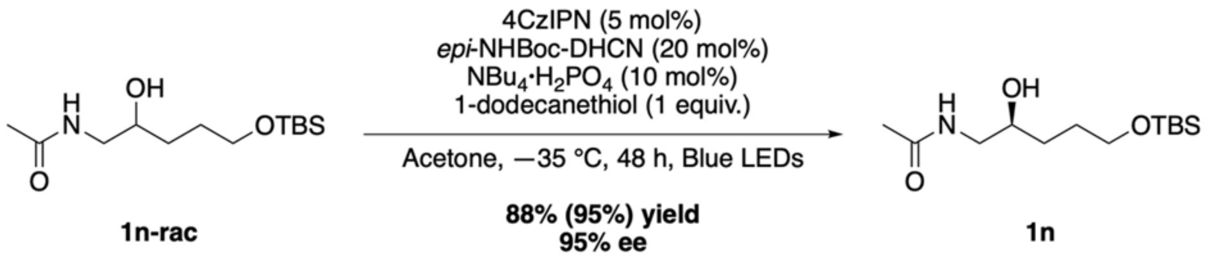

1n was prepared according to **General Procedure G** using amino alcohol-derived substrate **1n-rac** (27.5 mg, 0.10 mmol). Purification by silica gel column chromatography (elution with 1% 7N ammonia in MeOH, 2% MeOH, 97% DCM) afforded **1n** as a white solid (24.2 mg, 0.088 mmol, 88% yield).

^{1}H NMR (700 MHz, $CDCl_3$) δ 5.99 (br. s, 1H), 3.95 – 3.89 (m, 1H), 3.74 – 3.67 (m, 2H), 3.67 – 3.61 (m, 1H), 3.51 (ddd, *J* = 13.5, 6.9, 2.9 Hz, 1H), 3.06 (ddd, *J* = 13.5, 8.2, 4.8 Hz, 1H), 2.00 (s, 3H), 1.75 – 1.60 (m, 3H) (overlaps with H_2O peak), 1.53 – 1.44 (m, 1H), 0.90 (s, 9H), 0.08 (s, 6H) ppm.
^{13}C NMR (176 MHz, $CDCl_3$) δ 170.8, 70.8, 63.8, 45.7, 33.1, 29.3, 26.0, 23.4, 18.4, –5.28, –5.32 ppm.
$[\alpha]_D^{25.0}$ = +12.6 (*c* 0.94, $CHCl_3$).

(*S*)-1-(*N*-acetylbenzamido)-5-((*tert*-butyldimethylsilyl)oxy)pentan-2-yl benzoate (1n-Bz)

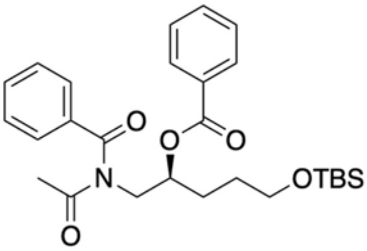

1n-Bz

1n-Bz was prepared according to **General Procedure H** using **1n** (12.1 mg, 0.044 mmol), DMAP (0.9 mg, 0.0074 mmol), triethylamine (0.02 mL, 0.14 mmol) and benzoyl chloride (0.01 mL, 0.086 mmol) in DCM (0.4 mL). Purification by silica gel column chromatography (elution with 0.5% MeOH in DCM followed by a second purification eluting with 10% acetone in petroleum ether) afforded **1n-Bz** (14.9 mg, 0.031 mmol, 70% yield, 95% ee).

^{1}H NMR (700 MHz, CD_3Cl) δ 7.92 – 7.85 (m, 2H), 7.55 – 7.44 (m, 4H), 7.41 – 7.31 (m, 4H), 5.51 – 5.40 (m, 1H), 4.18 (dd, *J* = 14.3, 8.7 Hz, 1H), 4.10 (dd, *J* = 14.3, 2.7 Hz, 1H), 3.64 – 3.56 (m, 2H), 2.04 (s, 3H), 1.81 – 1.74 (m, 1H), 1.74 – 1.67 (m, 1H), 1.65 – 1.54 (m, 2H) (overlaps with H_2O peak), 0.86 (s, 9H), 0.019 (s, 3H), 0.014 (s, 3H) ppm.
^{13}C NMR (176 MHz, CD_3Cl) δ 174.1, 173.3, 166.2, 135.3, 133.1, 132.7, 130.1, 129.8, 128.93, 128.86, 128.4, 73.3, 62.7, 49.4, 28.9, 28.5, 26.3, 26.1, 18.5, –5.20, –5.21 ppm.
HRMS $[M+H]^+$ m/z calc'd for $[C_{27}H_{37}NO_5SiH]^+$ expect 484.2519; found 484.2513.
Chiral SFC Analysis: CHIRALPAK IK (CO_2:MeOH, 97:03, 2.5 mL min^{-1}, 40 °C, 228 nm) t_R = 6.1 (major), 7.9 (minor) minutes, 95% ee.

(*S*)-*N*-(5-ethoxy-2-hydroxypentyl)acetamide (1o)

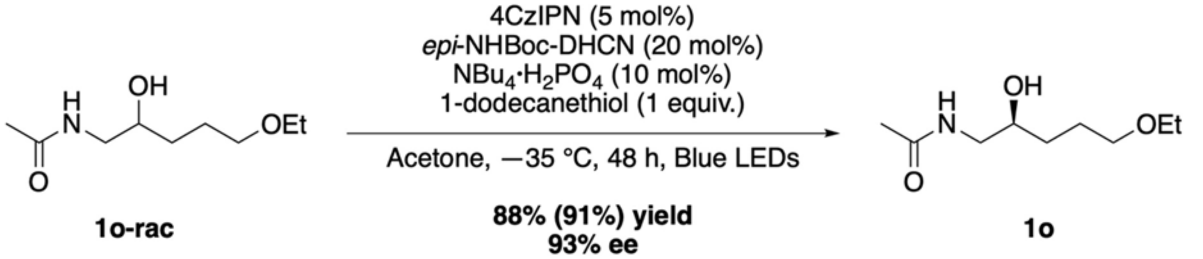

1o was prepared according to **General Procedure G** using amino alcohol-derived substrate **1o-rac** (18.9 mg, 0.10 mmol). Purification by silica gel column chromatography (elution with 5% MeOH in DCM) afforded **1o** as a colorless oil (16.6 mg, 0.088 mmol, 88% yield).

^{1}H NMR (700 MHz, $CDCl_3$) δ 6.19 (br. s, 1H), 3.67 (app. tt, *J* = 8.7, 2.8 Hz, 1H), 3.55 – 3.47 (m, 4H), 3.43 (ddd, *J* = 9.1, 8.2, 3.7 Hz, 1H), 3.02 (ddd, *J* = 13.4, 8.5, 4.6 Hz, 1H), 2.00 (s, 3H), 1.79 – 1.72 (m, 1H), 1.72 – 1.63 (m, 2H), 1.50 – 1.41 (m, 1H), 1.20 (t, *J* = 7.0 Hz, 3H) ppm.
^{13}C NMR (176 MHz, $CDCl_3$) δ 170.9, 71.0, 70.7, 66.6, 45.6, 33.3, 26.7, 23.4, 15.1 ppm.
$[\alpha]_D^{25.0}$ = +18.1 (*c* 1.02, $CHCl_3$).

(*S*)-1-(*N*-acetylbenzamido)-5-ethoxypentan-2-yl benzoate (1o-Bz)

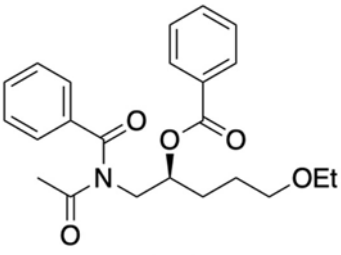

1o-Bz

1o-Bz was prepared according to **General Procedure H** using **1o** (5.4 mg, 0.029 mmol), DMAP (0.7 mg, 0.0057 mmol), triethylamine (0.02 mL, 0.14 mmol) and benzoyl chloride (0.01 mL, 0.086 mmol) in DCM (0.4 mL). Purification by silica gel column chromatography (elution with 1.5% MeOH in DCM followed by a second purification eluting with 20% acetone in petroleum ether) afforded **1o-Bz** (10.7 mg, 0.027 mmol, 94% yield, 93% ee).

^{1}H NMR (700 MHz, CD_3Cl) δ 7.91 – 7.86 (m, 2H), 7.54 – 7.45 (m, 4H), 7.40 – 7.32 (m, 4H), 5.51 – 5.43 (m, 1H), 4.17 (dd, *J* = 14.3, 8.7 Hz, 1H), 4.10 (dd, *J* = 14.3, 2.9 Hz, 1H), 3.44 – 3.38 (m, 2H), 3.43 (q, *J* = 7.0 Hz, 2H), 2.05 (s, 3H), 1.81 – 1.71 (m, 2H), 1.69 – 1.63 (m, 2H), 1.16 (t, *J* = 7.0 Hz, 3H) ppm.
^{13}C NMR (176 MHz, CD_3Cl) δ 174.1, 173.4, 166.2, 135.3, 133.1, 132.7, 130.0, 129.8, 128.93, 128.86, 128.4, 73.2, 70.0, 66.3, 49.4, 29.2, 26.3, 25.6, 15.3 ppm.
HRMS $[M+H]^+$ m/z calc'd for $[C_{23}H_{27}NO_5H]^+$ expect 398.1967; found 398.1958.
Chiral SFC Analysis: CHIRALPAK IK (CO_2:MeOH, 95:05, 2.5 mL min^{-1}, 40 °C, 228 nm) t_R = 7.5 (major), 8.9 (minor) minutes, 93% ee.

(*S*)-*N*-(2-hydroxy-4-(tetrahydro-2*H*-pyran-4-yl)butyl)acetamide (1p)

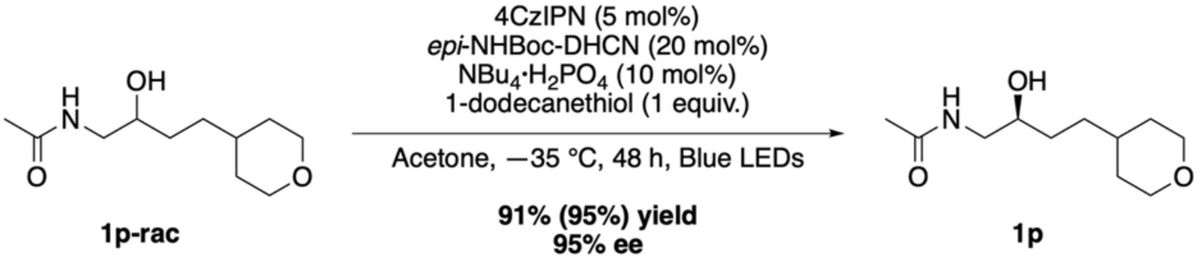

1p was prepared according to **General Procedure G** using amino alcohol-derived substrate **1p-rac** (21.5 mg, 0.10 mmol). Purification by silica gel column chromatography (elution with 1.5% 7N ammonia in MeOH, 3% MeOH, 95.5% DCM followed by a second purification eluting with 6% MeOH in DCM) afforded **1p** as an off-white solid (19.5 mg, 0.091 mmol, 91% yield).

^{1}H NMR (700 MHz, $CDCl_3$) δ 6.05 (br. s, 1H), 3.94 (br. dd, *J* = 10.8, 3.6 Hz, 2H), 3.72 – 3.66 (m, 1H), 3.46 (ddd, *J* = 14.0, 6.4, 2.8 Hz, 1H), 3.35 (br. t, *J* = 11.7 Hz, 2H), 3.12 (ddd, *J* = 14.0, 8.0, 5.4 Hz, 1H), 2.44 (br. s, 1H), 2.02 (s, 3H), 1.59 (app. br. d, *J* = 13.1 Hz, 2H), 1.51 – 1.43 (m, 3H), 1.43 – 1.37 (m, 1H), 1.31 – 1.22 (m, 3H) ppm.

^{13}**C NMR** (176 MHz, $CDCl_3$) δ 171.5, 71.8, 68.182, 68.178, 46.1, 35.1, 33.3, 33.2, 32.8, 32.0, 23.3 ppm.
[α]$_D$$^{25.0}$ = +10.0 (*c* 0.89, $CHCl_3$).

(*S*)-1-(*N*-acetylbenzamido)-4-(tetrahydro-2*H*-pyran-4-yl)butan-2-yl benzoate (1p-Bz)

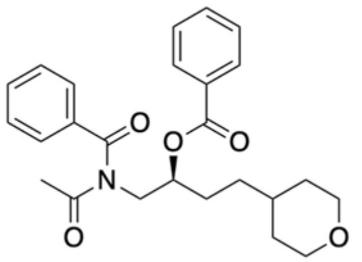

1p-Bz

1p-Bz was prepared according to **General Procedure H** using **1p** (9.7 mg, 0.045 mmol), DMAP (0.9 mg, 0.0074 mmol), triethylamine (0.02 mL, 0.14 mmol) and benzoyl chloride (0.01 mL, 0.086 mmol) in DCM (0.4 mL). Purification by silica gel column chromatography (elution with 1% MeOH in DCM followed by a second purification eluting with 20% acetone in petroleum ether) afforded **1p-Bz** (13.2 mg, 0.031 mmol, 69% yield, 95% ee).

^{1}**H NMR** (700 MHz, CD_3Cl) δ 7.91 – 7.85 (m, 2H), 7.54 – 7.46 (m, 4H), 7.40 – 7.32 (m, 4H), 5.45 – 5.38 (m, 1H), 4.18 (dd, *J* = 14.3, 8.7 Hz, 1H), 4.10 (dd, *J* = 14.3, 2.7 Hz, 1H), 3.92 (dd, *J* = 11.1, 3.8 Hz, 2H), 3.33 (t, *J* = 11.6 Hz, 2H), 2.04 (s, 3H), 1.73 – 1.67 (m, 2H), 1.62 – 1.56 (m, 2H), 1.51 – 1.43 (m, 1H), 1.37 – 1.31 (m, 2H), 1.27 – 1.20 (m, 2H) ppm.
^{13}**C NMR** (176 MHz, CD_3Cl) δ 174.1, 173.4, 166.3, 135.3, 133.2, 132.8, 130.0, 129.7, 129.0, 128.9, 128.4, 73.6, 68.14, 68.13, 49.3, 34.9, 33.1, 32.2, 29.3, 26.3 ppm.
HRMS $[M+H]^+$ m/z calc'd for $[C_{25}H_{29}NO_5H]^+$ expect 424.2124; found 424.2109.
Chiral SFC Analysis: CHIRALPAK IK (CO_2:MeOH, 90:10, 2.5 mL min^{-1}, 40 °C, 230 nm) t_R = 8.5 (major), 10.1 (minor) minutes, 95% ee.

(*S*)-*N*-(2-hydroxy-4-phenoxybutyl)acetamide (1q)

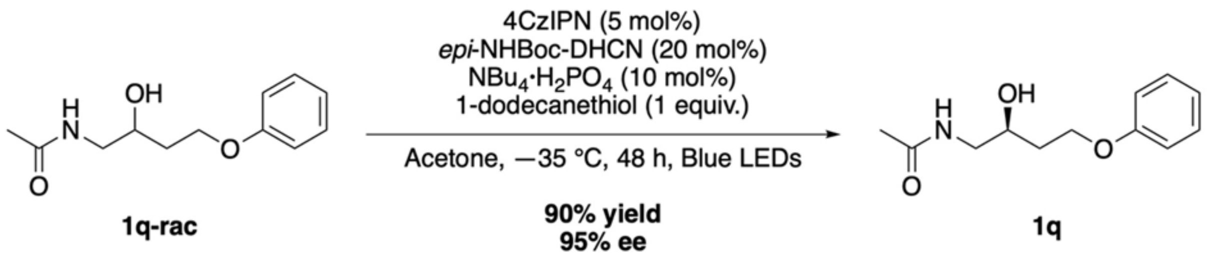

1q was prepared according to **General Procedure G** using amino alcohol-derived substrate **1q-rac** (22.3 mg, 0.10 mmol). Purification by silica gel column chromatography (elution with 3% MeOH in DCM) afforded **1q** as an orange solid (20.1 mg, 0.090 mmol, 90% yield, 95% ee).

^{1}**H NMR** (500 MHz, $CDCl_3$) δ 7.31 – 7.26 (m, 2H), 6.96 (t, *J* = 7.4 Hz, 1H), 6.92 – 6.86 (m, 2H), 6.10 (br. s, 1H), 4.20 – 4.08 (m, 2H), 4.05 – 3.96 (m, 1H), 3.52 (ddd, *J* = 14.0, 6.4, 3.1 Hz, 1H), 3.41 (br. s, 1H), 3.24 (ddd, *J* = 14.0, 7.3, 5.4 Hz, 1H), 2.01 (s, 3H), 1.97 – 1.89 (m, 2H) ppm.
^{13}**C NMR** (126 MHz, $CDCl_3$) δ 171.4, 158.6, 129.7, 121.2, 114.6, 69.7, 65.5, 45.9, 34.1, 23.3 ppm.
[α]$_D$$^{25.0}$ = +16.5 (*c* 1.81, $CHCl_3$).
Chiral SFC Analysis: CHIRALPAK IJ (CO_2:MeOH, 95:05, 2.5 mL min^{-1}, 40 °C, 217 nm) t_R = 9.4 (minor), 10.7 (major) minutes, 95% ee.

(*S*)-*N*-(2-hydroxy-3-(4-methoxyphenyl)propyl)acetamide (1r)

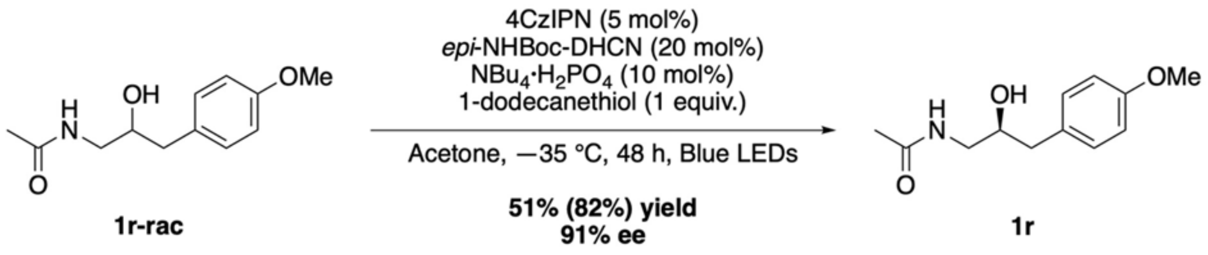

1r was prepared according to **General Procedure G** using amino alcohol-derived substrate **1r-rac** (22.3 mg, 0.10 mmol). Purification by silica gel column chromatography (elution with 1% 7N ammonia in MeOH, 2% MeOH, 97% DCM) afforded **1r** as a pale yellow oil (11.4 mg, 0.051 mmol, 51% yield, 91% ee).

^{1}**H NMR** (400 MHz, $CDCl_3$) δ 7.12 (d, *J* = 8.6 Hz, 2H), 6.85 (d, *J* = 8.6 Hz, 2H), 5.98 (br. s, 1H), 3.94 – 3.85 (m, 1H), 3.79 (s, 3H), 3.53 (ddd, *J* = 14.0, 6.6, 2.9 Hz, 1H), 3.14 (ddd, *J* = 14.0, 8.0, 5.2 Hz, 1H), 2.75 (dd, *J* = 13.8, 5.2 Hz, 1H), 2.66 (dd, *J* = 13.8, 8.0 Hz, 1H), 2.24 (br. s, 1H), 2.00 (s, 3H) ppm.
^{13}**C NMR** (176 MHz, $CDCl_3$) δ 171.3, 158.6, 130.4, 129.5, 114.3, 72.3, 55.4, 45.2, 40.7, 23.3 ppm.
[α]$_D$$^{25.0}$ = +18.7 (*c* 1.04, $CHCl_3$).
Chiral SFC Analysis: CHIRALPAK IJ (CO_2:MeOH, 93:07, 2.5 mL min^{-1}, 40 °C, 223 nm) t_R = 5.6 (minor), 7.3 (major) minutes, 91% ee.

(*S*)-*N*-(2-hydroxy-3-(*p*-tolyl)propyl)acetamide (1s)

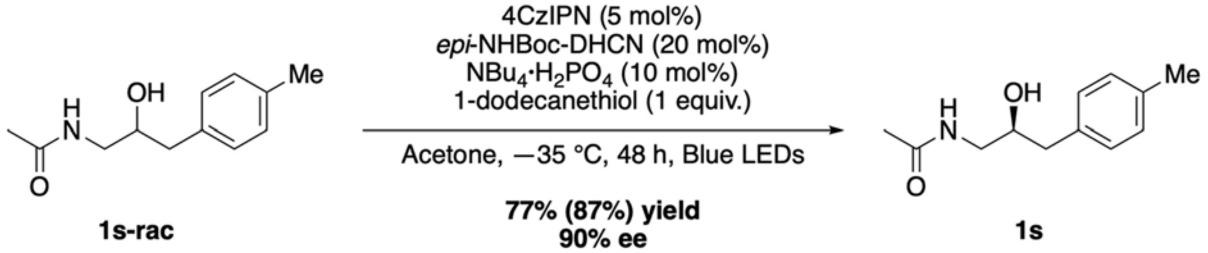

1s was prepared according to **General Procedure G** using amino alcohol-derived substrate **1s-rac** (20.7 mg, 0.10 mmol). Purification by silica gel column chromatography (elution with

1% 7N ammonia in MeOH, 2% MeOH, 97% DCM) afforded **1s** as a pale yellow oil that slowly forms a white solid (15.9 mg, 0.077 mmol, 77% yield, 90% ee).

^{1}H NMR (700 MHz, $CDCl_3$) δ 7.12 (d, *J* = 7.9 Hz, 2H), 7.09 (d, *J* = 7.9 Hz, 2H), 5.94 (br. s, 1H), 3.95 – 3.87 (m, 1H), 3.54 (ddd, *J* = 14.0, 6.6, 2.8 Hz, 1H), 3.15 (ddd, *J* = 14.0, 7.9, 5.2 Hz, 1H), 2.77 (dd, *J* = 13.7, 5.2 Hz, 1H), 2.68 (dd, *J* = 13.7, 8.1 Hz, 1H), 2.66 – 2.57 (m, 1H), 2.32 (s, 3H), 2.00 (s, 3H) ppm.
^{13}C NMR (176 MHz, $CDCl_3$) δ 171.3, 136.5, 134.4, 129.6, 129.3, 72.3, 45.2, 41.2, 23.4, 21.2 ppm.
$[\alpha]_D^{25.0}$ = +18.9 (*c* 1.43, $CHCl_3$).
Chiral SFC Analysis: CHIRALPAK IJ (CO_2:MeOH, 95:05, 2.5 mL min^{-1}, 40 °C, 216 nm) t_R = 6.3 (minor), 7.9 (major) minutes, 90% ee.

(*S*)-*N*-(3-(4-fluorophenyl)-2-hydroxypropyl)acetamide (1t)

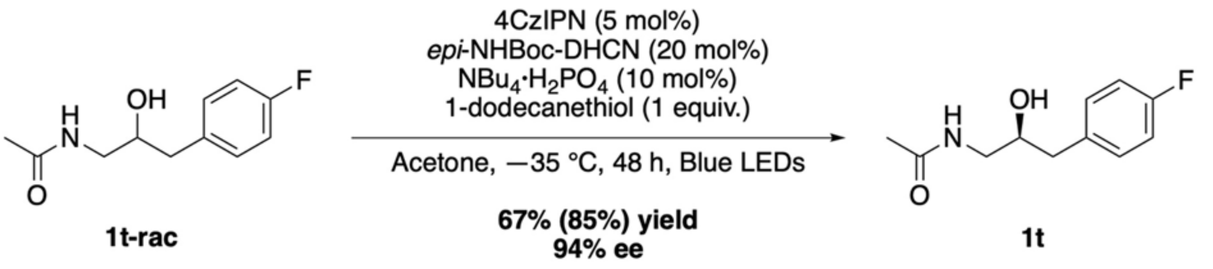

1t-rac **1t**

1t was prepared according to **General Procedure G** using amino alcohol-derived substrate **1t-rac** (21.1 mg, 0.10 mmol). Purification by silica gel column chromatography (elution with 1% 7N ammonia in MeOH, 2% MeOH, 97% DCM) afforded **1t** as a pale yellow cloudy oil (14.1 mg, 0.067 mmol, 67% yield, 94% ee).

^{1}H NMR (400 MHz, $CDCl_3$) δ 7.17 (dd, *J* = 8.6, 5.4 Hz, 2H), 6.99 (t, *J* = 8.6 Hz, 2H), 6.00 (br. s, 1H), 3.96 – 3.82 (m, 1H), 3.50 (ddd, 14.0, 6.6, 2.9 Hz, 1H), 3.15 (ddd, *J* = 14.0, 7.9, 5.3 Hz, 1H), 2.82 (br. s, 1H), 2.76 (dd, *J* = 13.8, 5.3 Hz, 1H), 2.69 (dd, *J* = 13.8, 7.9 Hz, 1H), 2.00 (s, 3H) ppm.
^{19}F NMR (376 MHz, $CDCl_3$) –116.3 ppm.
^{13}C NMR (176 MHz, $CDCl_3$) δ 171.5, 161.9 (d, *J* = 244.9 Hz), 133.4 (d, *J* = 3.3 Hz), 130.9 (d, *J* = 7.9 Hz), 115.6 (d, *J* = 21.3 Hz), 72.3 (2C), 45.3, 40.7, 23.3 ppm.
$[\alpha]_D^{25.0}$ = +15.8 (*c* 1.28, $CHCl_3$).
Chiral SFC Analysis: CHIRALPAK IJ (CO_2:MeOH, 95:05, 2.5 mL min^{-1}, 40 °C, 203 nm) t_R = 4.5 (minor), 5.2 (major) minutes, 94% ee.

(*S*)-*N*-(3-(3-chlorophenyl)-2-hydroxypropyl)acetamide (1u)

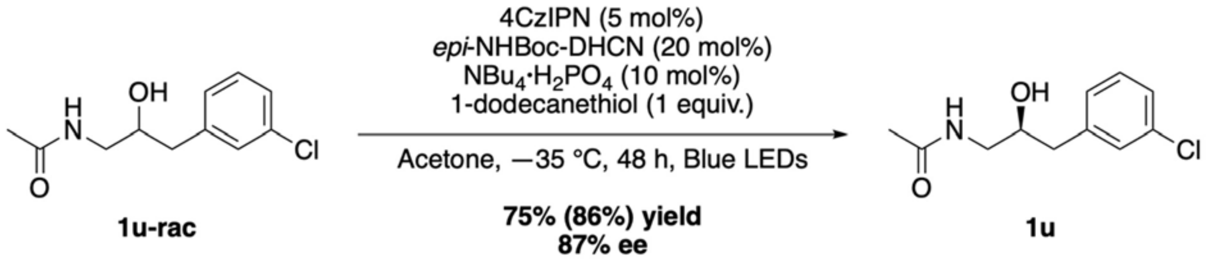

1u was prepared according to **General Procedure G** using amino alcohol-derived substrate **1u-rac** (22.8 mg, 0.10 mmol). Purification by silica gel column chromatography (elution with 4% MeOH in DCM) afforded **1u** as a pale yellow oil (17.1 mg, 0.075 mmol, 75% yield, 87% ee).

^{1}H NMR (500 MHz, $CDCl_3$) δ 7.26 – 7.19 (m, 3H), 7.09 (d, *J* = 7.0 Hz, 1H), 6.04 (br. s, 1H), 3.97 – 3.89 (m, 1H), 3.50 (ddd, *J* = 14.0, 6.5, 2.9 Hz, 1H), 3.16 (ddd, *J* = 14.0, 7.7, 5.3 Hz, 1H), 2.98 (br. s, 1H), 2.76 (dd, *J* = 13.8, 5.2 Hz, 1H), 2.70 (dd, *J* = 13.8, 8.0 Hz, 1H), 2.00 (s, 3H) ppm.
^{13}C NMR (126 MHz, $CDCl_3$) δ 171.5, 139.9, 134.5, 130.0, 129.6, 127.7, 127.0, 72.1, 45.4, 41.2, 23.3 ppm.
$[\alpha]_D^{25.0}$ = +11.6 (*c* 1.62, $CHCl_3$).
Chiral SFC Analysis: CHIRALPAK IJ (CO_2:MeOH, 95:05, 2.5 mL min^{-1}, 40 °C, 211 nm) t_R = 7.1 (minor), 8.8 (major) minutes, 87% ee.

(*S*)-*N*-(2-hydroxy-3-(naphthalen-1-yl)propyl)acetamide (1v)

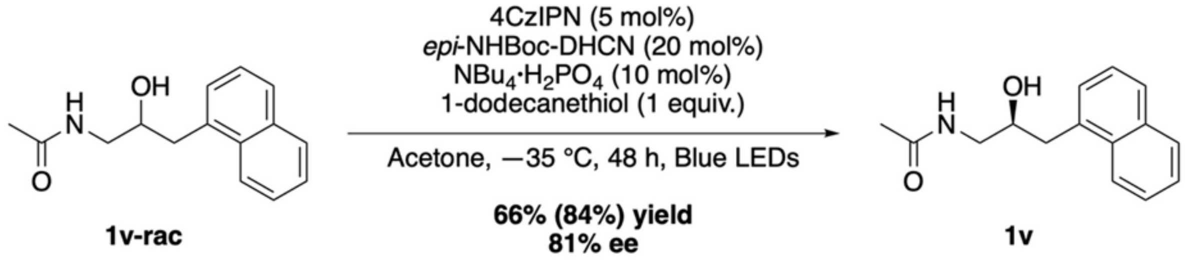

1v was prepared according to **General Procedure G** using amino alcohol-derived substrate **1v-rac** (24.3 mg, 0.10 mmol). Purification by silica gel column chromatography (elution with 1% 7N ammonia in MeOH, 2% MeOH, 97% DCM) afforded **1v** as a white solid (16.0 mg, 0.066 mmol, 66% yield, 81% ee).

^{1}H NMR (700 MHz, $CDCl_3$) δ 8.02 (d, *J* = 8.3 Hz, 1H), 7.86 (d, *J* = 7.9 Hz, 1H), 7.76 (d, *J* = 8.2 Hz, 1H), 7.55 – 7.46 (m, 2H), 7.41 (t, *J* = 7.6 Hz, 1H), 7.34 (d, *J* = 6.9 Hz, 1H), 5.99 (br. s, 1H), 4.14 – 4.07 (m, 1H), 3.57 (ddd, *J* = 14.0, 6.6, 2.8 Hz, 1H), 3.31 – 3.23 (m, 2H), 3.17 (dd, *J* = 14.0, 8.0 Hz, 1H), 2.40 (br. s, 1H), 1.98 (s, 3H) ppm.
^{13}C NMR (176 MHz, $CDCl_3$) δ 171.4, 134.2, 133.8, 132.2, 129.0, 127.79, 127.78, 126.4, 126.0, 125.6, 123.8, 71.6, 45.6, 38.7, 23.3 ppm.
$[\alpha]_D^{25.0}$ = +15.0 (*c* 1.08, $CHCl_3$).

Chiral SFC Analysis: CHIRALPAK IJ (CO_2:MeOH, 91:09, 2.5 mL min^{-1}, 40 °C, 280 nm) t_R = 5.8 (minor), 6.5 (major) minutes, 81% ee.

(*S*)-*N*-(2-hydroxy-3-(thiophen-3-yl)propyl)acetamide (1w)

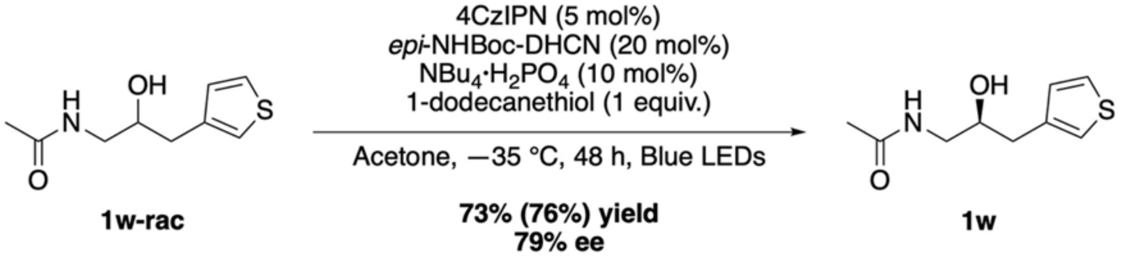

1w was prepared according to **General Procedure G** using amino alcohol-derived substrate **1w-rac** (19.9 mg, 0.10 mmol). Purification by silica gel column chromatography (elution with 3% MeOH in DCM) afforded **1w** as a pale yellow viscous oil (14.5 mg, 0.073 mmol, 73% yield, 79% ee).

^{1}H NMR (700 MHz, $CDCl_3$) δ 7.29 (dd, *J* = 4.8, 2.7 Hz, 1H), 7.05 (d, *J* = 2.7 Hz, 1H), 6.97 (d, *J* = 4.8 Hz, 1H), 6.01 (br. s, 1H), 3.97 – 3.90 (m, 1H), 3.52 (ddd, *J* = 14.0, 6.6, 3.0 Hz, 1H), 3.15 (ddd, *J* = 14.0, 7.9, 5.4 Hz, 1H), 2.84 (br. s, 1H), 2.83 (dd, *J* = 14.3, 5.2 Hz, 1H), 2.77 (dd, *J* = 14.3, 7.8 Hz, 1H), 2.00 (s, 3H) ppm.
^{13}C NMR (176 MHz, $CDCl_3$) δ 171.4, 137.8, 128.6, 126.3, 122.4, 71.5, 45.3, 36.0, 23.3 ppm.
$[\alpha]_D^{25.0}$ = +16.7 (*c* 1.32, $CHCl_3$).
Chiral SFC Analysis: CHIRALPAK IJ (CO_2:MeOH, 95:05, 2.5 mL min^{-1}, 40 °C, 232 nm) t_R = 6.6 (minor), 7.3 (major) minutes, 79% ee.

(*S*)-*N*-(2-hydroxy-4,4-dimethylpentyl)acetamide (1x)

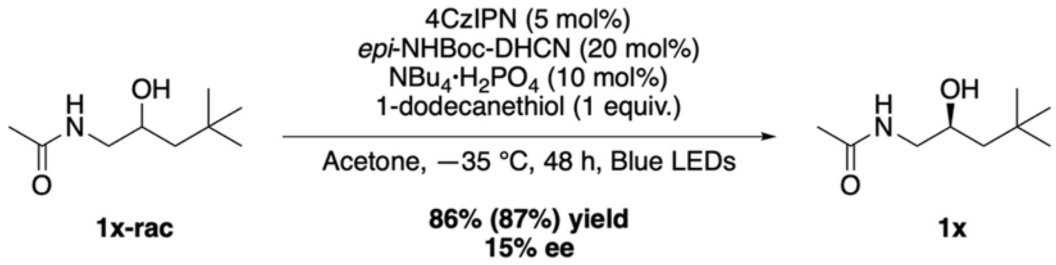

1x was prepared according to **General Procedure G** using amino alcohol-derived substrate **1x-rac** (17.3 mg, 0.10 mmol). Purification by silica gel column chromatography (elution with 1.5% 7N ammonia in MeOH, 3% MeOH, 95.5% DCM) afforded **1x** as a white solid (14.9 mg, 0.086 mmol, 86% yield).

^{1}H NMR (700 MHz, $CDCl_3$) δ 5.98 (br. s, 1H), 3.89 – 3.81 (m, 1H), 3.42 (ddd, *J* = 13.8, 6.6, 3.2 Hz, 1H), 3.07 (ddd, *J* = 13.8, 8.3, 5.2 Hz, 1H), 2.25 (br. s, 1H), 2.01 (s, 3H), 1.38 (dd, *J* = 14.5, 7.8 Hz, 1H), 1.34 (dd, *J* = 14.5, 3.1 Hz, 1H), 0.96 (s, 9H) ppm.
^{13}C NMR (176 MHz, $CDCl_3$) δ 171.1, 69.2, 48.9, 47.4, 30.3, 30.2, 23.4 ppm.

(*S*)-1-(*N*-acetylbenzamido)-4,4-dimethylpentan-2-yl benzoate (1x-Bz)

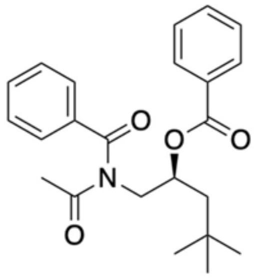

1x-Bz

1x-Bz was prepared according to **General Procedure H** using **1x** (6.4 mg, 0.037 mmol), DMAP (0.9 mg, 0.0074 mmol), triethylamine (0.02 mL, 0.14 mmol) and benzoyl chloride (0.01 mL, 0.086 mmol) in DCM (0.4 mL). Purification by silica gel column chromatography (elution with 0.5% MeOH in DCM followed by a second purification eluting with 10% acetone in petroleum ether) afforded **1x-Bz** (8.7 mg, 0.023 mmol, 62% yield, 15% ee).

^{1}H NMR (700 MHz, CD_3Cl) δ 7.91 (d, *J* = 8.3 Hz, 2H), 7.52 (t, *J* = 7.4 Hz, 1H), 7.50 – 7.44 (m, 3H), 7.39 (t, *J* = 7.8 Hz, 2H), 7.35 (t, *J* = 8.0 Hz, 2H), 5.63 – 5.57 (m, 1H), 4.10 (dd, *J* = 14.3, 8.7 Hz, 1H), 4.04 (dd, *J* = 14.3, 3.0 Hz, 1H), 2.07 (s, 3H), 1.67 (dd, *J* = 14.8, 7.8 Hz, 1H), 1.50 (dd, *J* = 14.8, 3.5 Hz, 1H), 0.94 (s, 9H) ppm.
^{13}C NMR (176 MHz, CD_3Cl) δ 173.9, 173.5, 166.1, 135.3, 133.1, 132.6, 130.3, 129.8, 128.9, 128.8, 128.4, 71.0, 50.9, 45.9, 30.3, 29.9, 26.3 ppm.
HRMS $[M+H]^+$ m/z calc'd for $[C_{23}H_{27}NO_4H]^+$ expect 382.2018; found 382.2016.
Chiral SFC Analysis: CHIRALPAK IK (CO_2:MeOH, 96:04, 2.5 mL min^{-1}, 40 °C, 227 nm) t_R = 4.6 (major), 5.4 (minor) minutes, 15% ee.

(*S*)-*N*-(2,5-dihydroxypentyl)acetamide (1y)

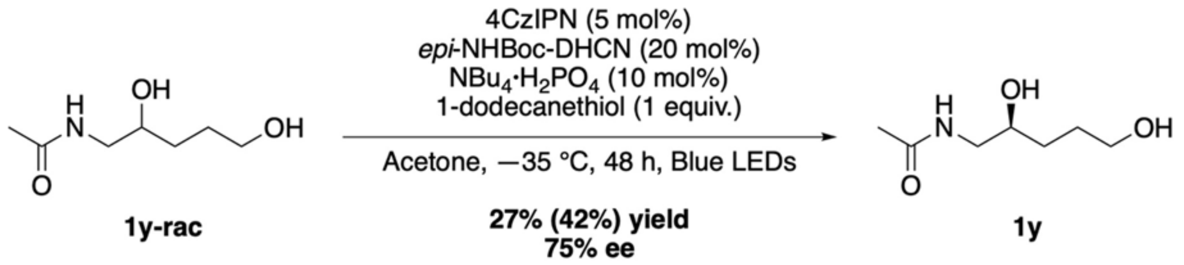

1y was prepared according to **General Procedure G** using amino alcohol-derived substrate **1y-rac** (16.1 mg, 0.10 mmol). Purification by silica gel column chromatography (elution with 15% MeOH in DCM followed by a second purification eluting with 5% ammonia in MeOH, 10% MeOH, 85% DCM) afforded **1y** as a pale yellow oil (4.4 mg, 0.027 mmol, 27% yield).

^{1}H NMR (700 MHz, CD_3OD) δ 3.66 – 3.61 (m, 1H), 3.57 (t, *J* = 6.4 Hz, 2H), 3.28 (dd, *J* = 13.6, 4.5 Hz, 1H), 3.09 (dd, *J* = 13.6, 7.2 Hz, 1H), 1.96 (s, 3H), 1.73 – 1.66 (m, 1H), 1.63 – 1.52 (m, 2H), 1.46 – 1.38 (m, 1H) ppm.
^{13}C NMR (176 MHz, CD_3OD) δ 173.6, 71.2, 62.9, 46.7, 32.2, 29.7, 22.5 ppm.

$[\alpha]_D^{25.0}$ = +8.6 (*c* 0.37, MeOH).

(*S*)-5-(*N*-acetylbenzamido)pentane-1,4-diyl dibenzoate (1y-Bz)

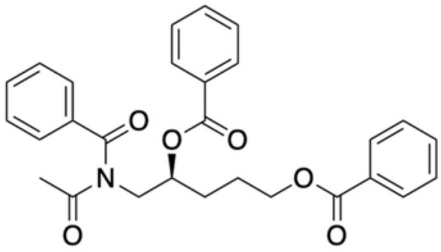

1y-Bz

1y-Bz was prepared according to **General Procedure H** using **1y** (4.4 mg, 0.027 mmol), DMAP (0.7 mg, 0.0057 mmol), triethylamine (0.02 mL, 0.14 mmol) and benzoyl chloride (0.01 mL, 0.086 mmol) in DCM (0.2 mL). Purification by silica gel column chromatography (elution with 0.5% MeOH in DCM followed by a second purification eluting with 20% acetone in petroleum ether) afforded **1y-Bz** (3.4 mg, 0.0072 mmol, 26% yield, 75% ee).

^{1}H NMR (700 MHz, CD_3Cl) δ 8.02 (d, *J* = 8.4 Hz, 2H), 7.89 (d, *J* = 8.3 Hz, 2H), 7.55 (t, *J* = 7.4 Hz, 1H), 7.53 – 7.46 (m, 4H), 7.43 (t, *J* = 7.8 Hz, 2H), 7.37 (t, *J* = 7.9 Hz, 2H), 7.34 (t, *J* = 7.8 Hz, 2H), 5.56 – 5.48 (m, 1H), 4.33 (t, *J* = 5.9 Hz, 2H), 4.22 (dd, *J* = 14.3, 8.6 Hz, 1H), 4.13 (*J* = 14.3, 2.9 Hz, 1H), 2.04 (s, 3H), 1.91 – 1.80 (m, 4H) ppm.
^{13}C NMR (176 MHz, CD_3Cl) δ 174.0, 173.4, 166.7, 166.2, 135.2, 133.2, 133.1, 132.8, 130.4, 129.9, 129.8, 129.7, 128.95, 128.92, 128.5, 128.4, 73.0, 64.5, 49.2, 29.1, 26.3, 24.8 ppm.
HRMS $[M+H]^+$ m/z calc'd for $[C_{28}H_{27}NO_6H]^+$ expect 474.1917; found 474.1921.
Chiral SFC Analysis: CHIRALPAK IK (CO_2:MeOH, 87:13, 2.5 mL min^{-1}, 40 °C, 226 nm) t_R = 8.4 (major), 9.8 (minor) minutes, 75% ee.

(*S*)-*N*-(2-hydroxybutyl)isobutyramide (1z)

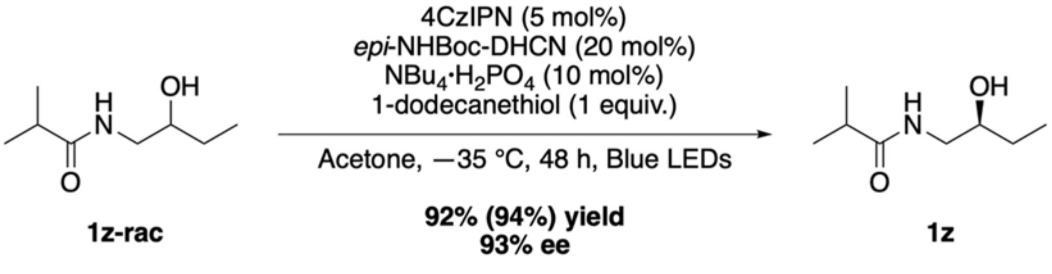

1z was prepared according to **General Procedure G** using amino alcohol-derived substrate **1z-rac** (15.9 mg, 0.10 mmol). Purification by silica gel column chromatography (elution with 4% MeOH in DCM) afforded **1z** as a white solid (14.7 mg, 0.092 mmol, 92% yield).

^{1}H NMR (700 MHz, $CDCl_3$) δ 5.99 (br. s, 1H), 3.67 – 3.59 (m, 1H), 3.47 (ddd, *J* = 14.0, 6.4, 2.8 Hz, 1H), 3.14 (ddd, *J* = 14.0, 7.7, 5.2 Hz, 1H), 2.43 (br. s, 1H), 2.39 (hept, *J* = 6.9 Hz, 1H), 1.53 – 1.44 (m, 2H), 1.16 (d, *J* = 6.9 Hz, 6H), 0.96 (t, *J* = 7.5 Hz, 3H) ppm.

^{13}**C NMR** (176 MHz, $CDCl_3$) δ 178.4, 73.2 (2C), 45.4, 35.8, 28.1, 19.8 (2C), 10.0 ppm.
$[\alpha]_D^{25.0}$ = +18.9 (*c* 0.62, $CHCl_3$).

(*S*)-1-(*N*-isobutyrylbenzamido)butan-2-yl benzoate (1z-Bz)

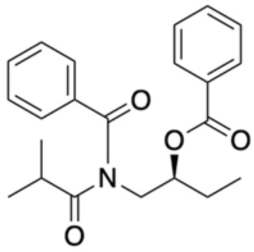

1z-Bz

1z-Bz was prepared according to **General Procedure H** using **1z** (7.3 mg, 0.046 mmol), DMAP (0.9 mg, 0.0074 mmol), triethylamine (0.02 mL, 0.14 mmol) and benzoyl chloride (0.01 mL, 0.086 mmol) in DCM (0.4 mL). Purification by silica gel column chromatography (elution with 0.5% MeOH in DCM followed by a second purification eluting with 10% acetone in petroleum ether) afforded **1z-Bz** (3.0 mg, 0.0082 mmol, 18% yield, 93% ee).

^{1}**H NMR** (700 MHz, CD_3Cl) δ 7.87 (d, *J* = 7.8 Hz, 2H), 7.52 (d, *J* = 7.3 Hz, 2H), 7.49 (t, *J* = 7.4 Hz, 1H), 7.47 (t, *J* = 7.5 Hz, 1H), 7.37 – 7.32 (m, 4H), 5.41 – 5.34 (m, 1H), 4.15 (dd, *J* = 14.3, 8.6 Hz, 1H), 4.07 (dd, *J* = 14.3, 2.8 Hz, 1H), 2.71 (hept, *J* = 6.6 Hz, 1H), 1.78 – 1.67 (m, 2H), 1.02 (d, *J* = 2.6 Hz, 3H), 1.01 (d, *J* = 2.8 Hz, 3H), 0.97 (t, *J* = 7.5 Hz, 3H) ppm.
^{13}**C NMR** (176 MHz, CD_3Cl) δ 181.4, 174.2, 166.2, 135.4, 133.0, 132.8, 130.2, 129.8, 129.0, 128.9, 128.3, 75.0, 49.5, 35.9, 25.6, 20.2, 19.4, 9.6 ppm.
HRMS $[M+H]^+$ m/z calc'd for $[C_{22}H_{25}NO_4H]^+$ expect 368.1862; found 368.1859.
Chiral SFC Analysis: CHIRALPAK IK (CO_2:MeOH, 98:02, 2.5 mL min^{-1}, 40 °C, 228 nm) t_R = 7.6 (major), 8.8 (minor) minutes, 93% ee.

(3,5,7)-*N*-((*S*)-2-hydroxybutyl)adamantane-1-carboxamide (1za)

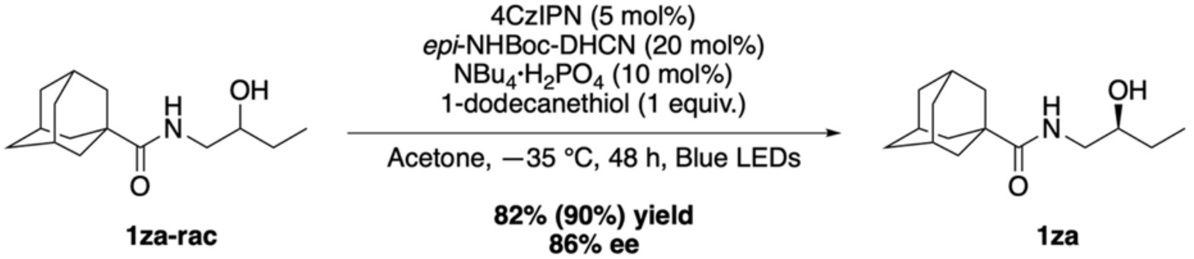

1za was prepared according to **General Procedure G** using amino alcohol-derived substrate **1za-rac** (25.1 mg, 0.10 mmol). Purification by silica gel column chromatography (elution with 2% MeOH in DCM) afforded **1za** as a white solid (20.6 mg, 0.082 mmol, 82% yield).

^{1}**H NMR** (700 MHz, $CDCl_3$) δ 6.15 (br. s, 1H), 3.64 – 3.58 (m, 1H), 3.46 (ddd, *J* = 14.0, 6.3, 2.5 Hz, 1H), 3.15 (ddd, *J* = 14.0, 7.7, 5.5 Hz, 1H), 2.53 (br. s, 1H), 2.06 – 2.01 (m, 3 H), 1.87 –

1.83 (m, 6H), 1.75 – 1.70 (m, 3H), 1.70 – 1.65 (m, 3H), 1.50 – 1.43 (m, 2H), 0.94 (t, *J* = 7.5 Hz, 3H) ppm.
^{13}C NMR (176 MHz, $CDCl_3$) δ 179.7, 73.33, 73.31, 45.4, 40.7, 39.2, 36.5, 28.1, 28.0, 10.1 ppm.
$[\alpha]_D^{25.0}$ = +9.3 (*c* 0.95, $CHCl_3$).

(*S*)-1-((3,5,7)-adamantane-1-carboxamido)butan-2-yl benzoate (1za-Bz)

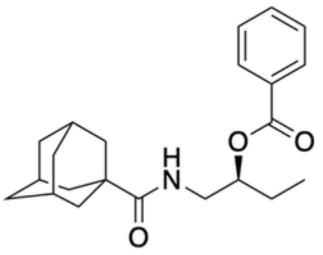

1za-Bz

1za-Bz was prepared according to **General Procedure H** using **1za** (10.3 mg, 0.041 mmol), DMAP (0.9 mg, 0.0074 mmol), triethylamine (0.02 mL, 0.14 mmol) and benzoyl chloride (0.01 mL, 0.086 mmol) in DCM (0.4 mL). Purification by silica gel column chromatography (elution with 0.5% MeOH in DCM) afforded **1za-Bz** (14.0 mg, 0.039 mmol, 96% yield, 86% ee).

^{1}H NMR (700 MHz, CD_3Cl) δ 8.05 (d, *J* = 8.4 Hz, 2H), 7.58 (t, *J* = 7.4 Hz, 1H), 7.46 (t, *J* = 7.8 Hz, 2H), 6.07 (br. s, 1H), 5.16 – 5.10 (m, 1H), 3.60 – 3.49 (m, 2H), 2.02 – 1.97 (m, 3H), 1.81 – 1.77 (m, 6H), 1.77 – 1.72 (m, 2H), 1.72 – 1.68 (m, 3H), 1.67 – 1.62 (m, 3H), 1.01 (t, *J* = 7.5 Hz, 3H) ppm.
^{13}C NMR (176 MHz, CD_3Cl) δ 178.3, 167.2, 133.3, 130.2, 129.8, 128.6, 75.6, 43.2, 40.7, 39.2, 36.6, 28.2, 25.5, 9.9 ppm.
HRMS $[M+H]^+$ m/z calc'd for $[C_{22}H_{29}NO_3H]^+$ expect 356.2226; found 356.2229.
Chiral SFC Analysis: CHIRALPAK IK (CO_2:MeOH, 92:08, 1.25 mL min^{-1}, 40 °C, 226 nm) t_R = 20.0 (minor), 20.8 (major) minutes, 86% ee.

(*S*)-3-cyano-*N*-(2-hydroxybutyl)propanamide (1zb)

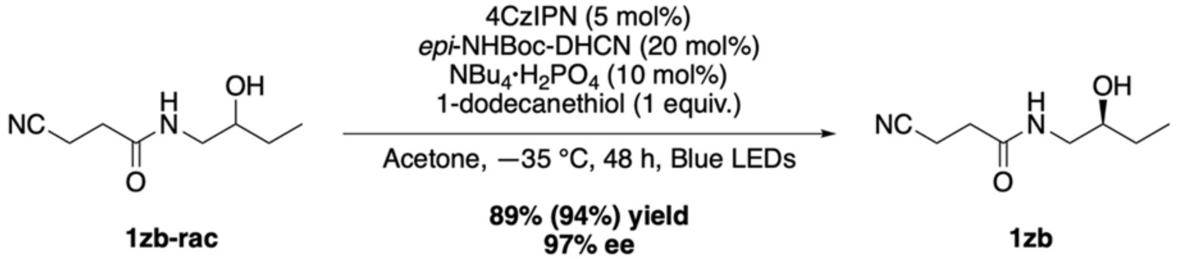

1zb was prepared according to **General Procedure G** using amino alcohol-derived substrate **1zb-rac** (17.0 mg, 0.10 mmol). Purification by silica gel column chromatography (elution with 5% MeOH in DCM) afforded **1zb** as a pale yellow oil (15.2 mg, 0.089 mmol, 89% yield).

^{1}H NMR (700 MHz, $CDCl_3$) δ 6.23 – 6.01 (m, 1H), 3.68 – 3.62 (m, 1H), 3.58 – 3.52 (m, 1H), 3.15 – 3.08 (m, 1H), 2.75 – 2.64 (m, 2H), 2.56 (br. t, *J* = 7.2 Hz, 2H), 1.96 (br. s, 1H) (overlaps with H_2O peak), 1.56 – 1.44 (m, 2H), 0.97 (br. t, *J* = 7.4 Hz, 3H) ppm.
^{13}C NMR (176 MHz, $CDCl_3$) δ 169.4, 119.1, 72.4, 45.3, 31.7 (2C), 28.0 (2C), 13.3, 9.8 ppm.
$[\alpha]_D^{25.0}$ = +30.7 (*c* 0.71, $CHCl_3$).

(*S*)-1-(*N*-(3-cyanopropanoyl)benzamido)butan-2-yl benzoate (1zb-Bz)

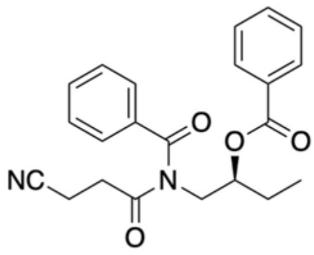

1zb-Bz

1zb-Bz was prepared according to **General Procedure H** using **1zb** (7.6 mg, 0.045 mmol), DMAP (0.9 mg, 0.0074 mmol), triethylamine (0.02 mL, 0.14 mmol) and benzoyl chloride (0.01 mL, 0.086 mmol) in DCM (0.4 mL). Purification by silica gel column chromatography (elution with 0.5% MeOH in DCM followed by a second purification eluting with 20% acetone in petroleum ether) afforded **1zb-Bz** (1.6 mg, 0.0042 mmol, 10% yield, 97% ee).

^{1}H NMR (700 MHz, CD_3Cl) δ 7.90 (d, *J* = 8.5 Hz, 2H), 7.57 (t, *J* = 7.4 Hz, 1H), 7.54 (t, *J* = 7.4 Hz, 1H), 7.47 – 7.38 (m, 6H), 5.36 – 5.30 (m, 1H), 4.15 (dd, *J* = 14.4, 2.6 Hz, 1H), 4.02 (dd, *J* = 14.4, 9.0 Hz, 1H), 2.97 (ddd, *J* = 17.4, 7.5, 6.2 Hz, 1H), 2.79 (dt, *J* = 17.4, 7.5 Hz, 1H), 2.68 (dt, *J* = 16.9, 7.5 Hz, 1H), 2.63 (ddd, *J* = 16.9, 7.5, 6.2 Hz, 1H), 1.71 – 1.59 (m, 2H), 0.93 (t, *J* = 7.5 Hz, 3H) ppm.
^{13}C NMR (176 MHz, CD_3Cl) δ 173.6, 172.8, 166.3, 134.3, 133.3, 133.0, 129.9, 129.8, 129.1, 128.9, 128.5, 119.0, 74.0, 50.1, 33.7, 25.5, 13.4, 9.6 ppm.
HRMS $[M+H]^+$ m/z calc'd for $[C_{22}H_{22}N_2O_4H]^+$ expect 379.1658; found 379.1651.
Chiral SFC Analysis: CHIRALPAK IK (CO_2:MeOH, 93:07, 2.5 mL min^{-1}, 40 °C, 228 nm) t_R = 6.9 (major), 8.3 (minor) minutes, 97% ee.

(*S*)-*N*-(2-hydroxybutyl)-2-(phenylsulfonyl)acetamide (1zc)

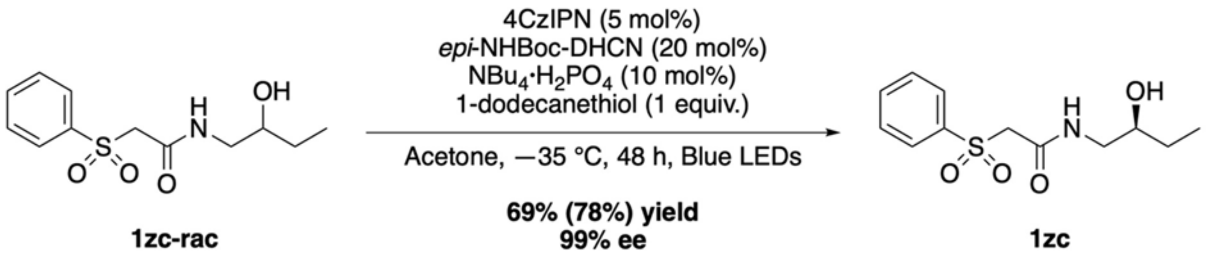

1zc-rac **1zc**

1zc was prepared according to **General Procedure G** using amino alcohol-derived substrate **1zc-rac** (27.1 mg, 0.10 mmol). Purification by silica gel column chromatography (elution with

4% MeOH in DCM) afforded **1zc** as an off-white solid (18.8 mg, 0.069 mmol, 69% yield, 99% ee).

^{1}H NMR (700 MHz, $CDCl_3$) δ 7.94 (d, *J* = 8.4 Hz, 2H), 7.69 (t, *J* = 7.5 Hz, 1H), 7.58 (t, *J* = 7.9 Hz, 2H), 7.15 (br. s, 1H), 4.07 (s, 2H), 3.69 – 3.63 (m, 1H), 3.50 (ddd, *J* = 13.9, 6.5, 3.1 Hz, 1H), 3.13 (ddd, *J* = 13.9, 8.2, 5.4 Hz, 1H), 2.64 (br. s, 1H), 1.55 – 1.44 (m, 2H), 0.96 (t, *J* = 7.5 Hz, 3H) ppm.
^{13}C NMR (176 MHz, $CDCl_3$) δ 161.4, 138.3, 134.6, 129.6, 128.4, 72.2, 62.2, 45.8, 27.9, 9.9 ppm.
$[\alpha]_D^{25.0}$ = +19.8 (*c* 1.71, $CHCl_3$).
Chiral SFC Analysis: CHIRALPAK IE (CO_2:MeOH, 85:15, 2.5 mL min^{-1}, 40 °C, 215 nm) t_R = 12.1 (major), 13.0 (minor) minutes, 99% ee.

(*S*)-*N*-(2-hydroxybutyl)-3-phenylpropanamide (1zd)

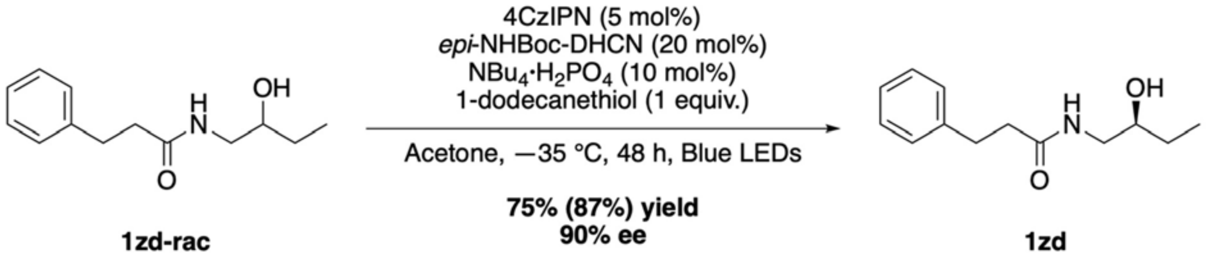

1zd was prepared according to **General Procedure G** using amino alcohol-derived substrate **1zd-rac** (22.1 mg, 0.10 mmol). Purification by silica gel column chromatography (elution with 3% MeOH in DCM) afforded **1zd** as a white solid (16.5 mg, 0.075 mmol, 75% yield, 90% ee).

^{1}H NMR (700 MHz, $CDCl_3$) δ 7.29 (t, *J* = 7.6 Hz, 2H), 7.23 – 7.18 (m, 3H), 5.82 (br. s, 1H), 3.55 – 3.50 (m, 1H), 3.44 (ddd, *J* = 13.9, 6.5, 2.9 Hz, 1H), 3.04 (ddd, *J* = 13.9, 8.0, 5.4 Hz, 1H), 2.97 (t, *J* = 7.6 Hz, 2H), 2.55 – 2.46 (m, 2H), 2.13 (br. s, 1H), 1.46 – 1.35 (m, 2H), 0.92 (t, *J* = 7.5 Hz, 3H) ppm.
^{13}C NMR (176 MHz, $CDCl_3$) δ 173.2, 140.9, 128.7, 128.5, 126.5, 72.8, 45.4, 38.6, 31.9, 27.9, 9.9 ppm.
$[\alpha]_D^{25.0}$ = +10.9 (*c* 1.30, $CHCl_3$).
Chiral SFC Analysis: CHIRALPAK IJ (CO_2:MeOH, 96:04, 2.5 mL min^{-1}, 40 °C, 203 nm) t_R = 8.2 (minor), 9.5 (major) minutes, 90% ee.

(*S*)-*N*-(2-hydroxybutyl)benzamide (1ze)

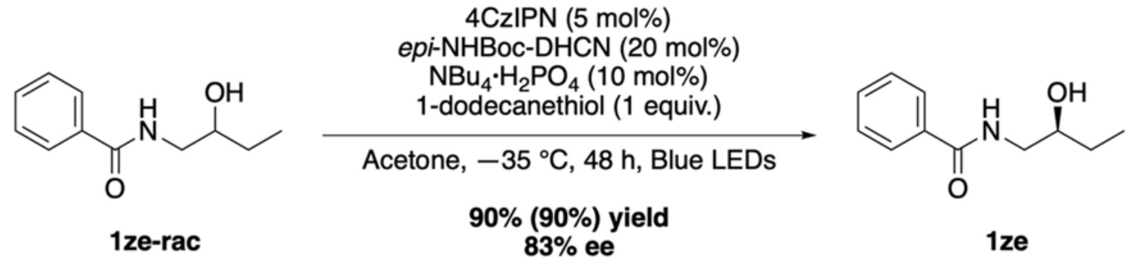

1ze was prepared according to **General Procedure G** using amino alcohol-derived substrate **1ze-rac** (19.3 mg, 0.10 mmol). Purification by silica gel column chromatography (elution with 3% MeOH in DCM) afforded **1ze** as a white solid (17.3 mg, 0.090 mmol, 90% yield, 83% ee).

^{1}H NMR (700 MHz, $CDCl_3$) δ 7.77 (d, *J* = 8.4 Hz, 2H), 7.49 (t, *J* = 7.4 Hz, 1H), 7.41 (t, *J* = 7.7 Hz, 2H), 6.76 (br. s, 1H), 3.77 – 3.72 (m, 1H), 3.69 (ddd, *J* = 13.9, 6.4, 3.0 Hz, 1H), 3.31 (ddd, *J* = 13.9, 7.8, 5.0 Hz, 1H), 2.60 (br. s, 1H), 1.60 – 1.48 (m, 2H), 0.98 (t, *J* = 7.5 Hz, 3H) ppm.
^{13}C NMR (176 MHz, $CDCl_3$) δ 168.6, 134.4, 131.7, 128.7, 127.1, 72.9, 45.9, 28.2, 10.0 ppm.
$[\alpha]_D^{25.0}$ = +19.6 (*c* 1.27, $CHCl_3$).
Chiral SFC Analysis: CHIRALPAK IJ (CO_2:MeOH, 95:05, 2.5 mL min^{-1}, 40 °C, 223 nm) t_R = 4.5 (minor), 5.2 (major) minutes, 83% ee.

(*S*)-4-(*tert*-butyl)-*N*-(2-hydroxybutyl)benzamide (1zf)

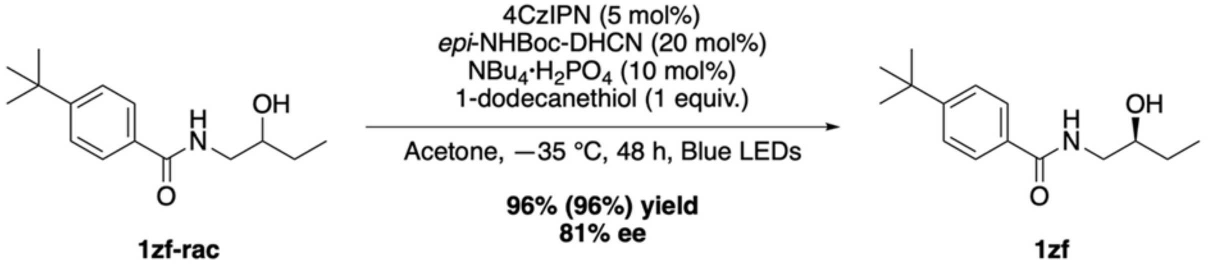

1zf was prepared according to **General Procedure G** using amino alcohol-derived substrate **1zf-rac** (24.9 mg, 0.10 mmol). Purification by silica gel column chromatography (elution with 3% MeOH in DCM) afforded **1zf** as a white solid (24.0 mg, 0.096 mmol, 96% yield, 81% ee).

^{1}H NMR (700 MHz, $CDCl_3$) δ 7.72 (d, *J* = 8.3 Hz, 2H), 7.42 (d, *J* = 8.3 Hz, 2H), 6.73 (br. s, 1H), 3.77 – 3.70 (m, 1 H), 3.68 (ddd, *J* = 13.9, 6.4, 2.9 Hz, 1H), 3.31 (ddd, *J* = 13.9, 7.9, 5.4 Hz, 1H), 2.93 (br. s, 1H), 1.59 – 1.48 (m, 2H), 1.32 (s, 9H), 0.97 (t, *J* = 7.5 Hz, 3H) ppm.
^{13}C NMR (176 MHz, $CDCl_3$) δ 168.5, 155.2, 131.5, 127.0, 125.6, 73.0, 45.9, 35.0, 31.3, 28.1, 10.0 ppm.
$[\alpha]_D^{25.0}$ = +11.1 (*c* 2.18, $CHCl_3$).
Chiral SFC Analysis: CHIRALPAK IJ (CO_2:MeOH, 95:05, 2.5 mL min^{-1}, 40 °C, 233 nm) t_R = 4.2 (minor), 4.7 (major) minutes, 81% ee.

(*S*)-*N*-(2-hydroxybutyl)-4-(trifluoromethyl)benzamide (1zg)

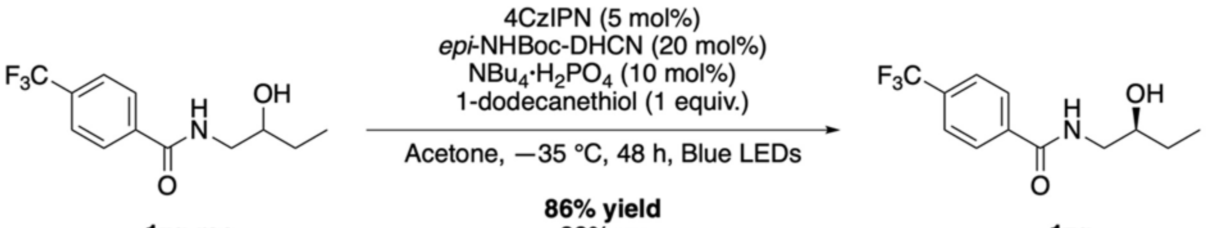

1zg was prepared according to **General Procedure G** using amino alcohol-derived substrate **1zg-rac** (26.1 mg, 0.10 mmol). Purification by silica gel column chromatography (elution with 3% MeOH in DCM) afforded **1zg** as a white solid (22.4 mg, 0.086 mmol, 86% yield, 89% ee).

^{1}H NMR (700 MHz, $(CD_3)_2CO$) δ 8.10 (d, *J* = 8.1 Hz, 2H), 7.94 (br. s, 1H), 7.81 (d, *J* = 8.1 Hz, 2H), 4.06 (d, *J* = 5.0 Hz, 1H), 3.73 – 3.67 (m, 1H), 3.55 (ddd, *J* = 13.5, 6.1, 4.0 Hz, 1H), 3.33 (ddd, *J* = 13.5, 7.5, 5.7 Hz, 1H), 1.58 – 1.51 (m, 1H), 1.48 – 1.40 (m, 1H), 0.96 (t, *J* = 7.5 Hz, 3H) ppm.
^{19}F NMR (376 MHz, $(CD_3)_2CO$) –63.4 ppm.
^{13}C NMR (176 MHz, $(CD_3)_2CO$) δ 166.7, 139.6 (q, *J* = 1.3 Hz), 132.9 (q, *J* = 32.3 Hz), 128.9, 126.1 (q, *J* = 3.8 Hz), 125.1 (q, *J* = 271.6 Hz), 72.4, 46.9, 28.6, 10.3 ppm.
$[\alpha]_D^{25.0}$ = +16.0 (*c* 1.96, MeOH).
Chiral SFC Analysis: CHIRALPAK IJ (CO_2:MeOH, 97:03, 2.5 mL min^{-1}, 40 °C, 220 nm) t_R = 4.7 (minor), 5.1 (major) minutes, 89% ee.

(*S*)-*N*-(2-hydroxybutyl)-4-methoxybenzamide (1zh)

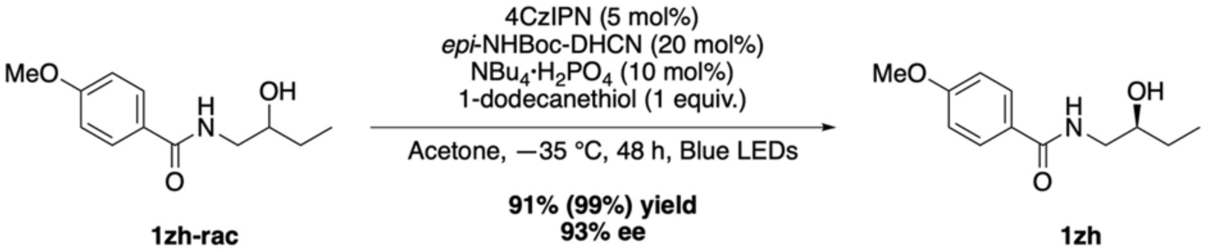

1zh was prepared according to **General Procedure G** using amino alcohol-derived substrate **1zh-rac** (22.3 mg, 0.10 mmol). Purification by silica gel column chromatography (elution with 3% MeOH in DCM) afforded **1zh** as a white solid (20.4 mg, 0.091 mmol, 91% yield, 93% ee).

^{1}H NMR (700 MHz, $CDCl_3$) δ 7.74 (d, *J* = 8.9 Hz, 2H), 6.89 (d, *J* = 8.9 Hz, 2H), 6.75 (br. s, 1H), 3.83 (s, 3H), 3.74 – 3.69 (m, 1H), 3.65 (ddd, *J* = 13.9, 6.0, 2.8 Hz, 1H), 3.29 (ddd, *J* = 13.9, 7.9, 4.7 Hz, 1H), 2.84 (br. s, 1H), 1.58 – 1.47 (m, 2H), 0.97 (t, *J* = 7.5 Hz, 3H) ppm.
^{13}C NMR (176 MHz, $CDCl_3$) δ 168.2, 162.4, 129.0, 126.5, 113.9, 73.0, 55.5, 45.9, 28.1, 10.0 ppm.
$[\alpha]_D^{25.0}$ = +14.2 (*c* 1.85, $CHCl_3$).
Chiral SFC Analysis: CHIRALPAK IJ (CO_2:MeOH, 95:05, 2.5 mL min^{-1}, 40 °C, 247 nm) t_R = 7.3 (minor), 8.5 (major) minutes, 93% ee.

(*S*)-*N*-(2-hydroxybutyl)benzo[*d*][1,3]dioxole-5-carboxamide (1zi)

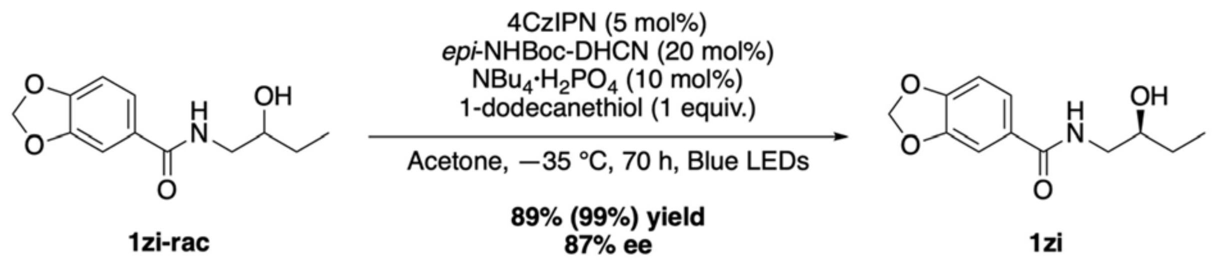

1zi was prepared according to **General Procedure G** using amino alcohol-derived substrate **1zi-rac** (23.7 mg, 0.10 mmol) stirred for 70 h. Purification by silica gel column chromatography (elution with 4% MeOH in DCM) afforded **1zi** as a white solid (21.1 mg, 0.089 mmol, 89% yield, 87% ee).

^{1}H NMR (700 MHz, $CDCl_3$) δ 7.30 (d, *J* = 8.0 Hz, 1H), 7.26 (s, 1H) (overlaps with $CHCl_3$ peak), 6.79 (d, *J* = 8.0 Hz, 1H), 6.67 (br. s, 1H), 6.00 (s, 2H), 3.76 – 3.68 (m, 1H), 3.68 – 3.61 (m, 1H), 3.33 – 3.23 (m, 1H), 2.57 (br. s, 1H), 1.58 – 1.47 (m, 2H), 0.97 (t, *J* = 7.4 Hz, 3H) ppm.
^{13}C NMR (176 MHz, $CDCl_3$) δ 167.9, 150.5, 148.1, 128.5, 121.8, 108.1, 107.8, 101.8, 72.9, 45.9, 28.1, 10.0 ppm.
$[\alpha]_D^{25.0}$ = +15.7 (*c* 1.93, $CHCl_3$).
Chiral SFC Analysis: CHIRALPAK IJ (CO_2:MeOH, 95:05, 2.5 mL min^{-1}, 40 °C, 255 nm) t_R = 7.5 (minor), 8.3 (major) minutes, 87% ee.

(*S*)-*N*-(2-hydroxybutyl)furan-3-carboxamide (1zj)

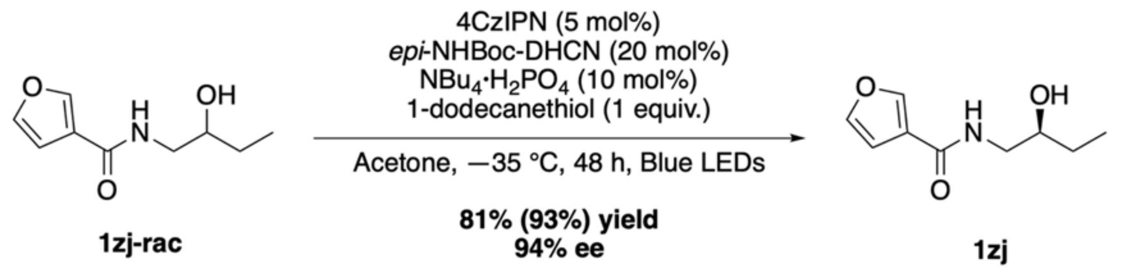

1zj was prepared according to **General Procedure G** using amino alcohol-derived substrate **1zj-rac** (18.3 mg, 0.10 mmol). Purification by silica gel column chromatography (elution with 2% MeOH in DCM) afforded **1zj** as a white solid (14.9 mg, 0.081 mmol, 81% yield, 94% ee).

^{1}H NMR (700 MHz, $CDCl_3$) δ 7.96 – 7.92 (m, 1H), 7.41 (t, *J* = 1.7 Hz, 1H), 6.65 – 6.61 (m, 1H), 6.49 (br. s, 1H), 3.74 – 3.67 (m, 1H), 3.62 (ddd, *J* = 14.0, 6.5, 2.9 Hz, 1H), 3.24 (ddd, *J* = 14.0, 7.9, 5.2 Hz, 1H), 2.92 (br. s, 1H), 1.58 – 1.46 (m, 2H), 0.97 (t, *J* = 7.5 Hz, 3H) ppm.
^{13}C NMR (176 MHz, $CDCl_3$) δ 163.7, 145.0, 143.9, 122.5, 108.4, 72.8, 45.4, 28.1, 10.0 ppm.
$[\alpha]_D^{25.0}$ = +22.2 (*c* 1.35, $CHCl_3$).
Chiral SFC Analysis: CHIRALPAK IJ (CO_2:MeOH, 95:05, 2.5 mL min^{-1}, 40 °C, 212 nm) t_R = 3.3 (minor), 3.6 (major) minutes, 94% ee.

(*S*)-*N*-(2-hydroxybutyl)-1-methyl-1*H*-pyrazole-4-carboxamide (1zk)

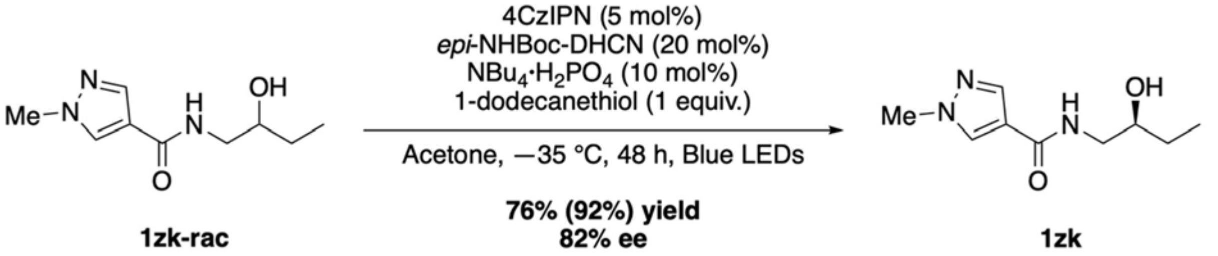

1zk was prepared according to **General Procedure G** using amino alcohol-derived substrate **1zk-rac** (19.7 mg, 0.10 mmol). Purification by silica gel column chromatography (elution with 6% MeOH in DCM) afforded **1zk** as a white solid (14.9 mg, 0.076 mmol, 76% yield, 82% ee).

^{1}H NMR (700 MHz, $CDCl_3$) δ 7.83 (s, 1H), 7.74 (s, 1H), 6.52 (br. s, 1H), 3.89 (s, 3H), 3.72 – 3.67 (m, 1H), 3.61 (ddd, *J* = 14.0, 6.3, 2.9 Hz, 1H), 3.25 (ddd, *J* = 14.0, 8.0, 5.2 Hz, 1H), 2.85 (br. s, 1H), 1.57 – 1.46 (m, 2H), 0.96 (t, *J* = 7.5 Hz, 3H) ppm.
^{13}C NMR (176 MHz, $CDCl_3$) δ 163.6, 138.1, 132.0, 118.6, 72.9, 45.4, 39.4, 28.1, 10.0 ppm.
$[\alpha]_D^{25.0}$ = +17.3 (*c* 1.39, $CHCl_3$).
Chiral SFC Analysis: CHIRALPAK IJ (CO_2:MeOH, 95:05, 2.5 mL min^{-1}, 40 °C, 224 nm) t_R = 4.3 (minor), 5.0 (major) minutes, 82% ee.

(*S*)-6-chloro-*N*-(2-hydroxybutyl)nicotinamide (1zl)

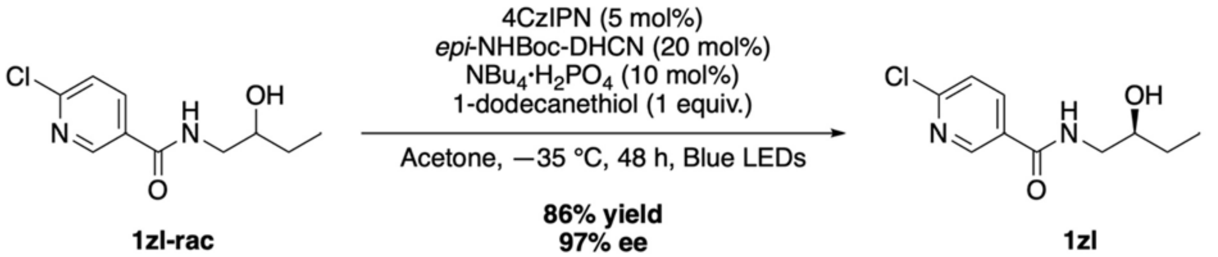

1zl was prepared according to **General Procedure G** using amino alcohol-derived substrate **1zl-rac** (22.9 mg, 0.10 mmol). Purification by silica gel column chromatography (elution with 4% MeOH in DCM) afforded **1zl** as a white solid (19.6 mg, 0.086 mmol, 86% yield, 97% ee).

^{1}H NMR (700 MHz, $(CD_3)_2CO$) δ 8.88 (d, *J* = 2.3 Hz, 1H), 8.27 (dd, *J* = 8.3, 2.3 Hz, 1H), 7.96 (br. s, 1H), 7.55 (d, *J* = 8.3 Hz, 1H), 4.01 (d, *J* = 4.9 Hz, 1H), 3.72 – 3.65 (m, 1H), 3.53 (ddd, *J* = 13.5, 6.0, 4.1 Hz, 1H), 3.30 (ddd, *J* = 13.5, 7.4, 5.9 Hz, 1H), 1.58 – 1.50 (m, 1H), 1.47 – 1.39 (m, 1H), 0.96 (t, *J* = 7.5 Hz, 3H) ppm.
^{13}C NMR (176 MHz, $(CD_3)_2CO$) δ 165.3, 154.1, 149.8, 139.1, 130.7, 124.8, 72.3, 46.8, 28.6, 10.3 ppm.
$[\alpha]_D^{25.0}$ = +18.1 (*c* 1.91, MeOH).
Chiral SFC Analysis: CHIRALPAK IJ (CO_2:MeOH, 94:06, 2.5 mL min^{-1}, 40 °C, 267 nm) t_R = 6.1 (minor), 7.6 (major) minutes, 97% ee.

(*S*)-*N*-(2-hydroxybutyl)-2-(thiazol-4-yl)acetamide (1zm)

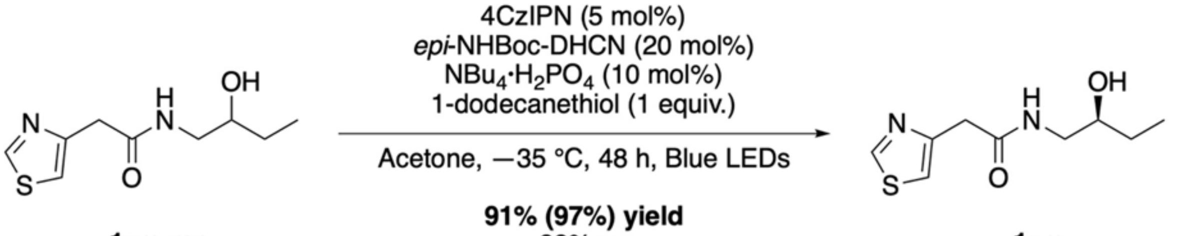

1zm was prepared according to **General Procedure G** using amino alcohol-derived substrate **1zm-rac** (21.4 mg, 0.10 mmol). Purification by silica gel column chromatography (elution with 5% MeOH in DCM) afforded **1zm** as a pale yellow oil (19.4 mg, 0.091 mmol, 91% yield, 99% ee).

^{1}H NMR (700 MHz, $CDCl_3$) δ 8.80 (d, *J* = 2.0 Hz, 1H), 7.22 – 7.19 (m, 1H), 7.12 (br. s, 1H), 3.78 (s, 2H), 3.66 – 3.60 (m, 1H), 3.47 (ddd, *J* = 14.0, 6.5, 3.0 Hz, 1H), 3.19 (br. s, 1H), 3.12 (ddd, *J* = 14.0, 8.0, 5.5 Hz, 1H), 1.51 – 1.40 (m, 2H), 0.94 (t, *J* = 7.5 Hz, 3H) ppm.
^{13}C NMR (176 MHz, $CDCl_3$) δ 170.3, 153.7, 150.8, 116.3, 72.6, 45.8, 38.9, 27.9, 10.0 ppm.
$[\alpha]_D^{25.0}$ = +23.1 (*c* 1.76, $CHCl_3$).
Chiral SFC Analysis: CHIRALPAK IC (CO_2:MeOH, 90:10, 2.5 mL min^{-1}, 40 °C, 239 nm) t_R = 11.3 minutes, 99% ee.

***tert*-butyl (*S*)-4-((2-hydroxybutyl)carbamoyl)piperidine-1-carboxylate (1zn)**

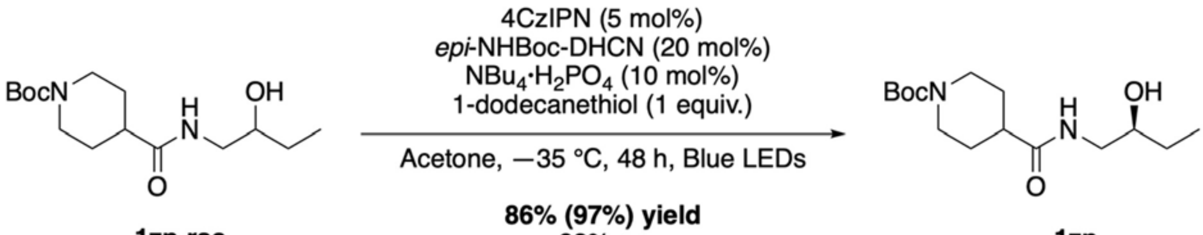

1zn was prepared according to **General Procedure G** using amino alcohol-derived substrate **1zn-rac** (30.0 mg, 0.10 mmol). Purification by silica gel column chromatography (elution with 3% MeOH in DCM) afforded **1zn** as a white solid (25.8 mg, 0.086 mmol, 86% yield).

^{1}H NMR (700 MHz, $CDCl_3$) δ 6.13 (br. s, 1H), 4.29 – 3.97 (m, 2H), 3.65 – 3.57 (m, 1H), 3.50 (ddd, *J* = 13.9, 6.5, 2.7 Hz, 1H), 3.10 (ddd, *J* = 13.9, 8.1, 5.2 Hz, 1H), 2.83 – 2.60 (m, 2H), 2.34 (br. s, 1H) (overlaps with H_2O peak), 2.25 (tt, *J* = 11.7, 3.5 Hz, 1H), 1.86 – 1.74 (m, 2H), 1.66 – 1.57 (m, 2H), 1.51 – 1.44 (m, 2H), 1.44 (s, 9H), 0.94 (t, *J* = 7.5 Hz, 3H) ppm.
^{13}C NMR (176 MHz, $CDCl_3$) δ 175.6, 154.8, 79.8, 72.9, 45.1, 43.4, 28.7, 28.6, 28.5, 28.0, 10.0 ppm.
$[\alpha]_D^{25.0}$ = +10.8 (*c* 1.10, $CHCl_3$).

***tert*-butyl (*S*)-4-((2-(benzoyloxy)butyl)carbamoyl)piperidine-1-carboxylate (1zn-Bz)**

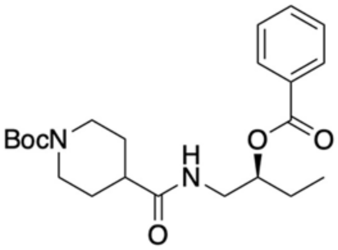

1zn-Bz

1zn-Bz was prepared according to **General Procedure H** using **1zn** (12.9 mg, 0.043 mmol), DMAP (0.9 mg, 0.0074 mmol), triethylamine (0.02 mL, 0.14 mmol) and benzoyl chloride (0.01 mL, 0.086 mmol) in DCM (0.4 mL). Purification by silica gel column chromatography (elution with 2.5% MeOH in DCM followed by a second purification eluting with 30% acetone in petroleum ether) afforded **1zn-Bz** (12.5 mg, 0.031 mmol, 72% yield, 92% ee).

^{1}H NMR (700 MHz, CD_3Cl) δ 8.04 (d, *J* = 8.4 Hz, 2H), 7.59 (t, *J* = 7.4 Hz, 1H), 7.46 (t, *J* = 7.8 Hz, 2H), 5.96 (br. s, 1H), 5.15 – 5.09 (m, 1H), 4.15 – 3.99 (m, 2H), 3.59 (ddd, *J* = 14.3, 5.2, 3.0 Hz, 1H), 3.52 (ddd, *J* = 14.3, 8.3, 5.8 Hz, 1H), 2.76 – 2.63 (m, 2H), 2.17 (tt, *J* = 11.5, 3.7 Hz, 1H), 1.80 – 1.70 (m, 4H), 1.61 – 1.50 (m, 2H), 1.44 (s, 9H), 1.00 (t, *J* = 7.5 Hz, 3H) ppm.
^{13}C NMR (176 MHz, CD_3Cl) δ 174.6, 167.2, 154.8, 133.5, 130.0, 129.8, 128.7, 79.7, 75.5, 43.4, 43.3, 28.7, 28.60, 28.56, 25.5, 9.9 ppm.
HRMS $[M+H]^+$ m/z calc'd for $[C_{22}H_{32}N_2O_5H]^+$ expect 405.2389; found 405.2379.
Chiral SFC Analysis: CHIRALPAK IJ (CO_2:MeOH, 96:04, 2.5 mL min^{-1}, 40 °C, 226 nm) t_R = 4.4 (major), 5.0 (minor) minutes, 92% ee.

(*S*)-*N*-(2-hydroxybutyl)morpholine-4-carboxamide (1zo)

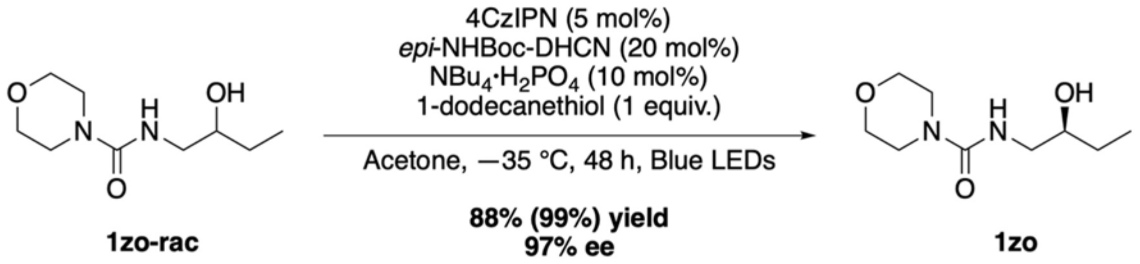

1zo was prepared according to **General Procedure G** using amino alcohol-derived substrate **1zo-rac** (20.2 mg, 0.10 mmol). Purification by silica gel column chromatography (elution with 4% MeOH in DCM) afforded **1zo** as a white solid (17.7 mg, 0.088 mmol, 88% yield).

^{1}H NMR (700 MHz, $CDCl_3$) δ 5.18 (br. s, 1H), 3.68 (t, *J* = 4.9 Hz, 4H), 3.64 – 3.59 (m, 1H), 3.43 (br. d, *J* = 14.1 Hz, 1H), 3.36 (t, *J* = 4.9 Hz, 4H), 3.24 (br. s, 1H), 3.12 (dd, *J* = 14.1, 7.9 Hz, 1H), 1.51 – 1.43 (m, 2H), 0.94 (t, *J* = 7.5 Hz, 3H) ppm.
^{13}C NMR (176 MHz, $CDCl_3$) δ 158.9, 73.6, 66.5, 46.7, 44.0, 27.9, 10.1 ppm.
$[\alpha]_D^{25.0}$ = +12.1 (*c* 0.81, $CHCl_3$).

(*S*)-1-(*N*-benzoylmorpholine-4-carboxamido)butan-2-yl benzoate (1zo-Bz)

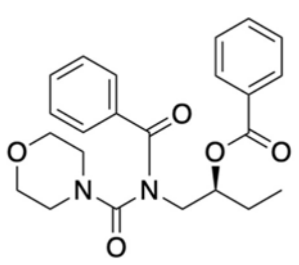

1zo-Bz

1zo-Bz was prepared according to **General Procedure H** using **1zo** (8.8 mg, 0.044 mmol), DMAP (0.9 mg, 0.0074 mmol), triethylamine (0.02 mL, 0.14 mmol) and benzoyl chloride (0.01 mL, 0.086 mmol) in DCM (0.4 mL). Purification by silica gel column chromatography (elution with 1% MeOH in DCM followed by a second purification eluting with 20% acetone in petroleum ether) afforded **1zo-Bz** (7.9 mg, 0.019 mmol, 44% yield, 97% ee).

^{1}H NMR (500 MHz, CD_3Cl) δ 8.04 (d, *J* = 8.1 Hz, 2H), 7.61 (d, *J* = 7.2 Hz, 2H), 7.57 (t, *J* = 7.4 Hz, 1H), 7.49 (t, *J* = 7.5 Hz, 1H), 7.45 (t, *J* = 7.7 Hz, 2H), 7.39 (t, *J* = 7.7 Hz, 2H), 5.39 (br. s, 1H), 4.23 (dd, *J* = 14.3, 9.5 Hz, 1H), 4.13 (dd, *J* = 14.3, 2.0 Hz, 1H), 2.96 (br. s, 8H), 1.86 – 1.72 (m, 2H), 1.03 (t, *J* = 7.5 Hz, 3H) ppm.
^{13}C NMR (126 MHz, CD_3Cl) δ 170.1, 166.2, 157.0, 134.9, 133.5, 132.1, 130.2, 129.9, 128.7, 128.6, 128.3, 75.8 (br.), 65.4, 49.5, 46.0 (br.), 44.6 (br.), 25.7, 9.5 ppm.
HRMS $[M+H]^+$ m/z calc'd for $[C_{23}H_{26}N_2O_5H]^+$ expect 411.1920; found 411.1926.
Chiral SFC Analysis: CHIRALPAK IK (CO_2:MeOH, 95:05, 2.5 mL min^{-1}, 40 °C, 225 nm) t_R = 9.8 (major), 11.0 (minor) minutes, 97% ee.

(*S*)-1-(*tert*-butyl)-3-(2-hydroxybutyl)urea (1zp)

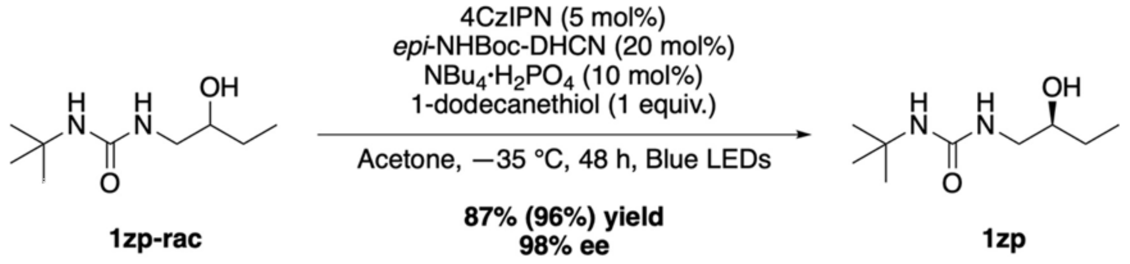

1zp was prepared according to **General Procedure G** using amino alcohol-derived substrate **1zp-rac** (18.8 mg, 0.10 mmol). Purification by silica gel column chromatography (elution with 3% MeOH in DCM) afforded **1zp** as a white solid (16.4 mg, 0.087 mmol, 87% yield).

^{1}H NMR (700 MHz, $CDCl_3$) δ 3.63 – 3.55 (m, 1H), 3.31 (br. d, *J* = 14.0 Hz, 1H), 3.04 (dd, *J* = 14.0, 7.7 Hz, 1H), 1.47 (app. quint, *J* = 7.1 Hz, 2H), 1.32 (s, 9H), 0.95 (t, *J* = 7.4 Hz, 3H) ppm.
^{13}C NMR (176 MHz, $CDCl_3$) δ 159.2 (br.), 73.8, 50.7, 46.4, 29.6, 27.9, 10.1 ppm.
$[\alpha]_D^{25.0}$ = +9.1 (*c* 0.76, $CHCl_3$).

(*S*)-1-(*N*-(*tert*-butylcarbamoyl)benzamido)butan-2-yl benzoate (1zp-Bz)

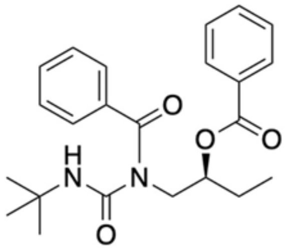

1zp-Bz

1zp-Bz was prepared according to **General Procedure H** using **1zp** (8.2 mg, 0.044 mmol), DMAP (0.9 mg, 0.0074 mmol), triethylamine (0.02 mL, 0.14 mmol) and benzoyl chloride (0.01 mL, 0.086 mmol) in DCM (0.4 mL). Purification by silica gel column chromatography (elution with 0.5% MeOH in DCM followed by a second purification eluting with 10% acetone in petroleum ether) afforded **1zp-Bz** (10.7 mg, 0.027 mmol, 62% yield, 98% ee).

^{1}H NMR (700 MHz, CD_3Cl) δ 8.25 (br. s, 1H), 7.99 (d, *J* = 7.6 Hz, 2H), 7.56 (t, *J* = 7.4 Hz, 1H), 7.47 – 7.41 (m, 3H), 7.40 – 7.35 (m, 4H), 5.27 – 5.17 (m, 1H), 4.22 – 4.11 (m, 1H), 3.98 (br. d, *J* = 14.5 Hz, 1H), 1.61 – 1.53 (m, 2H) (overlaps with H_2O peak), 1.22 (s, 9H), 0.83 (t, *J* = 7.4 Hz, 3H) ppm.
^{13}C NMR (176 MHz, CD_3Cl) δ 174.2, 166.4, 153.3, 136.4, 133.2, 130.7, 130.2, 130.0, 128.7, 128.4, 127.1, 74.6, 51.3, 49.2, 28.6, 25.4, 9.5 ppm.
HRMS $[M+H]^+$ m/z calc'd for $[C_{23}H_{28}N_2O_4H]^+$ expect 397.2127; found 397.2125.
Chiral SFC Analysis: CHIRALPAK IK (CO_2:MeOH, 97:03, 2.5 mL min^{-1}, 40 °C, 225 nm) t_R = 6.5 (major), 7.4 (minor) minutes, 98% ee.

(*S*)-1-(3,5-bis(trifluoromethyl)phenyl)-3-(2-hydroxybutyl)urea (1zq)

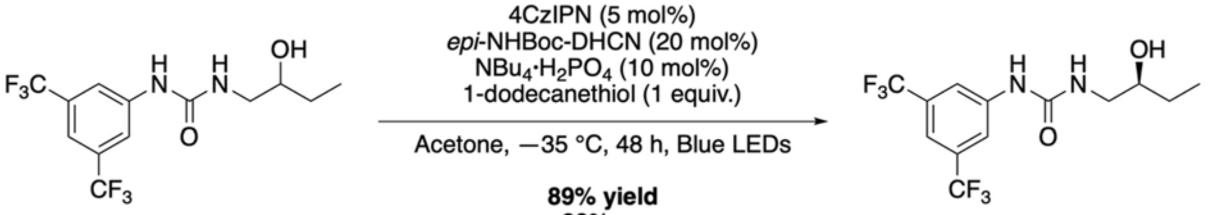

1zq-rac **1zq**

1zq was prepared according to **General Procedure G** using amino alcohol-derived substrate **1zq-rac** (34.4 mg, 0.10 mmol). Purification by silica gel column chromatography (elution with 3% MeOH in DCM) afforded **1zq** as a white solid (30.5 mg, 0.089 mmol, 89% yield, 80% ee).

^{1}H NMR (700 MHz, $(CD_3)_2CO$) δ 8.71 (br. s, 1H), 8.14 (s, 2H), 7.51 (s, 1H), 6.15 (br. s, 1H), 4.00 (br. s, 1H), 3.64 – 3.56 (m, 1H), 3.44 – 3.37 (m, 1H), 3.15 – 3.09 (m, 1H), 1.54 – 1.47 (m, 1H), 1.47 – 1.39 (m, 1H), 0.94 (t, *J* = 7.5 Hz, 3H) ppm.
^{19}F NMR (471 MHz, $(CD_3)_2CO$) –63.7 ppm.
^{13}C NMR (176 MHz, $(CD_3)_2CO$) δ 156.0, 143.7, 132.4 (q, *J* = 32.8 Hz), 124.5 (q, *J* = 271.9 Hz), 118.3 (q, *J* = 3.7 Hz), 114.7 (app. quint, *J* = 4.0 Hz), 72.6 (2C), 46.3, 28.51 (2C), 10.3 ppm.

$[\alpha]_D^{25.0}$ = +12.0 (*c* 2.55, MeOH).
Chiral HPLC Analysis: CHIRALPAK IH (Hexane:^{i}PrOH, 95:05, 1.25 mL min^{-1}, 40 °C, 245 nm) t_R = 7.3 (minor), 9.1 (major) minutes, 80% ee.

***tert*-butyl (*S*)-(2-hydroxybutyl)carbamate (1zr)**

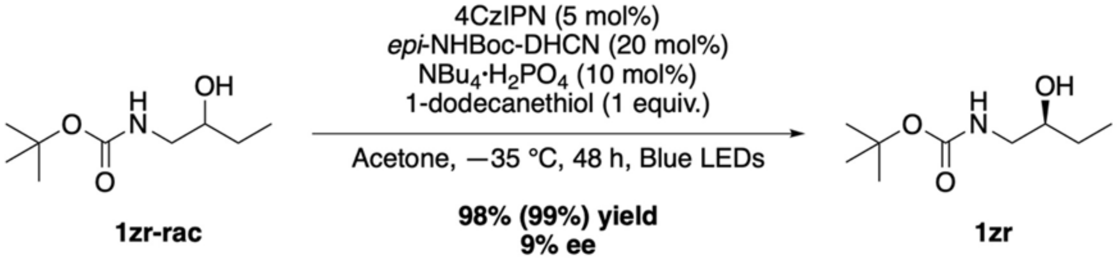

1zr was prepared according to **General Procedure G** using amino alcohol-derived substrate **1zr-rac** (18.9 mg, 0.10 mmol). Purification by silica gel column chromatography (elution with 2% MeOH in DCM) afforded **1zr** as a pale yellow oil (18.6 mg, 0.098 mmol, 98% yield).

^{1}H NMR (700 MHz, $CDCl_3$) δ 4.96 (br. s, 1H), 3.64 – 3.56 (m, 1H), 3.30 (br. d, *J* = 13.6 Hz, 1H), 3.00 (dd, *J* = 14.0, 7.7 Hz, 1H), 2.28 (br. s, 1H), 1.51 – 1.40 (m, 11H), 0.95 (t, *J* = 7.5 Hz, 3H) ppm.
^{13}C NMR (176 MHz, $CDCl_3$) δ 157.0, 79.7, 73.1, 46.4, 28.5, 27.8, 10.0 ppm.

(*S*)-1-(*N*-(*tert*-butoxycarbonyl)benzamido)butan-2-yl benzoate (1zr-Bz)

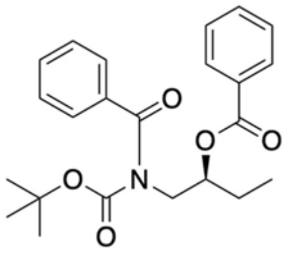

1zr-Bz

1zr-Bz was prepared according to **General Procedure H** using **1zr** (9.3 mg, 0.049 mmol), DMAP (1.1 mg, 0.0090 mmol), triethylamine (0.02 mL, 0.14 mmol) and benzoyl chloride (0.01 mL, 0.086 mmol) in DCM (0.4 mL). Purification by silica gel column chromatography (elution with DCM) afforded **1zr-Bz** (1.4 mg, 0.0035 mmol, 7% yield, 9% ee).

^{1}H NMR (700 MHz, CD_3Cl) δ 7.97 (d, *J* = 8.4 Hz, 2H), 7.47 (t, *J* = 7.4 Hz, 1H), 7.43 (d, *J* = 8.3 Hz, 2H), 7.38 (t, *J* = 7.5 Hz, 1H), 7.34 (t, *J* = 7.8 Hz, 2H), 7.28 (t, *J* = 7.8 Hz, 2H), 5.52 – 5.46 (m, 1H), 4.32 (dd, *J* = 14.2, 8.9 Hz, 1H), 3.97 (dd, *J* = 14.2, 3.0 Hz, 1H), 1.86 – 1.77 (m, 2H), 1.05 (s, 9H), 1.04 (t, *J* = 7.4 Hz, 3H) ppm.
^{13}C NMR (176 MHz, CD_3Cl) δ 173.4, 166.6, 153.5, 137.8, 132.9, 131.0, 130.4, 129.8, 128.3, 128.0, 127.6, 83.3, 74.7, 48.1, 27.4, 25.6, 9.7 ppm.
HRMS $[M+H]^+$ m/z calc'd for $[C_{23}H_{27}NO_5Na]^+$ expect 420.1787; found 420.1799.

Chiral SFC Analysis: CHIRALPAK IK (CO_2:MeOH, 95:05, 2.5 mL min^{-1}, 40 °C, 226 nm) t_R = 3.3 (major), 3.9 (minor) minutes, 9% ee.

(*S*)-2-(benzyloxy)-*N*-(2-hydroxybutyl)acetamide (1zs)

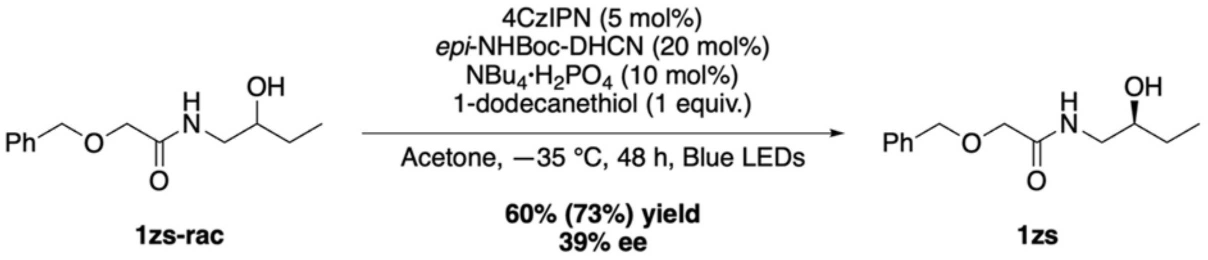

1zs was prepared according to **General Procedure G** using amino alcohol-derived substrate **1zs-rac** (23.7 mg, 0.10 mmol). Purification by silica gel column chromatography (elution with 2% MeOH in DCM) afforded **1zs** as a pale yellow oil that slowly forms a white solid (14.3 mg, 0.060 mmol, 60% yield, 39% ee).

^{1}H NMR (700 MHz, $CDCl_3$) δ 7.37 (t, *J* = 7.3 Hz, 2H), 7.35 – 7.31 (m, 3H), 6.98 (br. s, 1H), 4.58 (s, 2H), 4.01 (s, 2H), 3.67 – 3.61 (m, 1H), 3.49 (ddd, *J* = 13.9, 6.6, 3.0 Hz, 1H), 3.19 (ddd, *J* = 13.9, 7.9, 5.7 Hz, 1H), 2.06 (br. s, 1H) (overlaps with H_2O peak), 1.55 – 1.44 (m, 2H), 0.96 (t, *J* = 7.5 Hz, 3H) ppm.
^{13}C NMR (176 MHz, $CDCl_3$) δ 170.8, 136.9, 128.8, 128.4, 128.2, 73.8, 72.9, 69.6, 45.0, 28.1, 10.0 ppm.
Chiral SFC Analysis: CHIRALPAK IJ (CO_2:MeOH, 95:05, 2.5 mL min^{-1}, 40 °C, 205 nm) t_R = 4.0 (minor), 4.6 (major) minutes, 39% ee.

(*S*)-*N*-(2-hydroxybutyl)-2-(methylthio)acetamide (1zt)

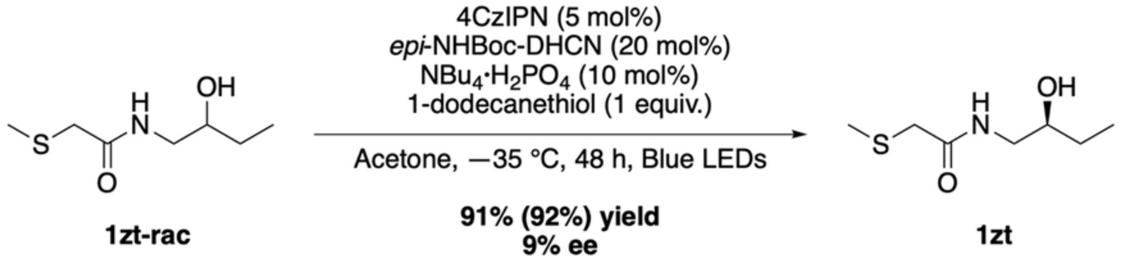

1zt was prepared according to **General Procedure G** using amino alcohol-derived substrate **1zt-rac** (17.7 mg, 0.10 mmol). Purification by silica gel column chromatography (elution with 3% MeOH in DCM) afforded **1zt** as a pale yellow oil (16.1 mg, 0.091 mmol, 91% yield, 9% ee).

^{1}H NMR (700 MHz, $CDCl_3$) δ 7.25 (br. s, 1H) (overlaps with $CHCl_3$ peak), 3.70 – 3.63 (m, 1H), 3.51 (ddd, *J* = 14.0, 6.5, 3.0 Hz, 1H), 3.22 (s, 2H), 3.20 (ddd, *J* = 14.0, 7.8, 5.4 Hz, 1H), 2.54 (br. s, 1H), 2.14 (s, 3H), 1.56 – 1.45 (m, 2H), 0.97 (t, *J* = 7.5 Hz, 3H) ppm.
^{13}C NMR (176 MHz, $CDCl_3$) δ 169.7, 72.9, 45.6, 38.3, 28.1, 16.5, 10.0 ppm.

Chiral SFC Analysis: CHIRALPAK IJ (CO_2:MeOH, 99:01, 2.5 mL min^{-1}, 40 °C, 212 nm) t_R = 7.5 (minor), 8.0 (major) minutes, 9% ee.

(*S*)-*N*-(5-ethoxy-2-hydroxypentyl)-2-(thiazol-4-yl)acetamide (1zu)

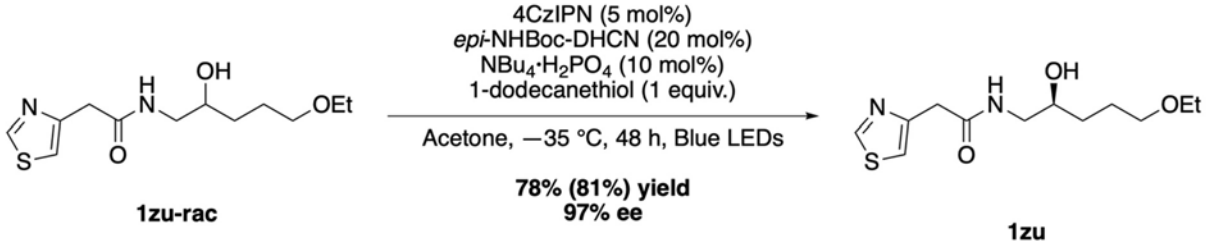

1zu was prepared according to **General Procedure G** using amino alcohol-derived substrate **1zu-rac** (27.2 mg, 0.10 mmol). Purification by silica gel column chromatography (elution with 4% MeOH in DCM) afforded **1zu** as a pale yellow oil (21.2 mg, 0.078 mmol, 78% yield, 97% ee).

^{1}H NMR (700 MHz, $CDCl_3$) δ 8.81 (d, *J* = 1.5 Hz, 1H), 7.22 (d, *J* = 1.5 Hz, 1H), 7.04 (br. s, 1H), 3.79 (s, 2H), 3.72 – 3.66 (m, 1H), 3.51 – 3.43 (m, 4H), 3.43 – 3.39 (m, 1H), 3.10 (ddd, *J* = 13.5, 8.1, 5.2 Hz, 1H), 3.08 (br. s, 1H), 1.74 – 1.65 (m, 2H), 1.64 – 1.57 (m, 1H), 1.48 – 1.40 (m, 1H), 1.19 (t, *J* = 7.0 Hz, 3H) ppm.
^{13}C NMR (176 MHz, $CDCl_3$) δ 169.9, 153.5, 150.8, 116.2, 70.8, 70.7, 66.4, 45.9, 39.0, 32.8, 26.4, 15.2 ppm.
$[\alpha]_D^{25.0}$ = +12.3 (*c* 1.93, $CHCl_3$).
Chiral SFC Analysis: CHIRALPAK IA (CO_2:MeOH, 90:10, 2.5 mL min^{-1}, 40 °C, 239 nm) t_R = 8.6 (minor), 9.0 (major) minutes, 97% ee.

(*S*)-*N*-(2-hydroxy-3-phenylpropyl)morpholine-4-carboxamide (1zv)

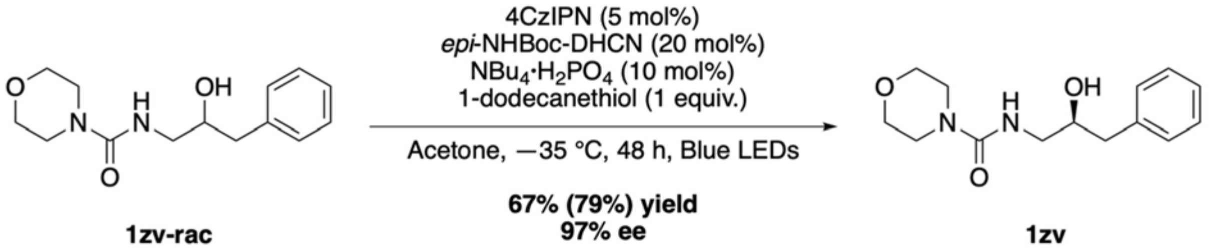

1zv was prepared according to **General Procedure G** using amino alcohol-derived substrate **1zv-rac** (26.4 mg, 0.10 mmol). Purification by silica gel column chromatography (elution with 3% MeOH in DCM followed by a second purification eluting with 40% acetone in petroleum ether) afforded **1zv** as a viscous colorless oil that slowly forms a white solid (17.8 mg, 0.067 mmol, 67% yield, 97% ee).

^{1}H NMR (700 MHz, $CDCl_3$) δ 7.30 (t, *J* = 7.5 Hz, 2H), 7.25 – 7.19 (m, 3H), 4.96 (br. s, 1H), 3.99 – 3.93 (m, 1H), 3.66 (br. t, *J* = 4.9 Hz, 4H), 3.49 (dd, *J* = 14.1, 2.7 Hz, 1H), 3.32 (br. t, *J* = 4.9 Hz, 4H), 3.18 (dd, *J* = 14.1, 7.7 Hz, 1H), 2.82 (br. s, 1H), 2.82 – 2.74 (m, 2H) ppm.

^{13}C NMR (176 MHz, $CDCl_3$) δ 158.8, 138.0, 129.5, 128.8, 126.7, 73.0, 66.5, 46.4, 44.2, 41.7 ppm.
$[\alpha]_D^{25.0}$ = +8.5 (*c* 1.58, $CHCl_3$).
Chiral SFC Analysis: CHIRALPAK IJ (CO_2:MeOH, 95:05, 2.5 mL min^{-1}, 40 °C, 202 nm) t_R = 8.4 (minor), 10.1 (major) minutes, 97% ee.

Characterization of Products Containing Pre-Existing Stereocenters

Yields are denoted as *isolated yield*% (*NMR yield*%) where NMR yield could be determined.

***tert*-butyl ((*S*)-1-(((*S*)-2-hydroxybutyl)amino)-1-oxo-3-phenylpropan-2-yl)carbamate (2a)**

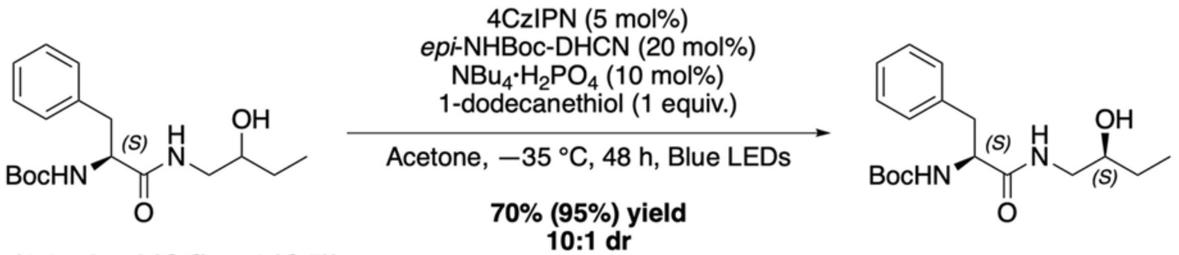

2a was prepared according to **General Procedure G** using amino alcohol-derived substrate **2a (1:1 mix of (*S,S*) and (*S,R*))** (33.6 mg, 0.10 mmol). Purification by silica gel column chromatography (elution with 2% MeOH in DCM) afforded **2a** as a white solid, isolated as a mixture of diastereomers (23.4 mg, 0.070 mmol, 70% yield, 10:1 dr). Combined signals for both diastereomers are denoted *d*1+*d*2. Discernible signals are denoted *d*1 and *d*2.

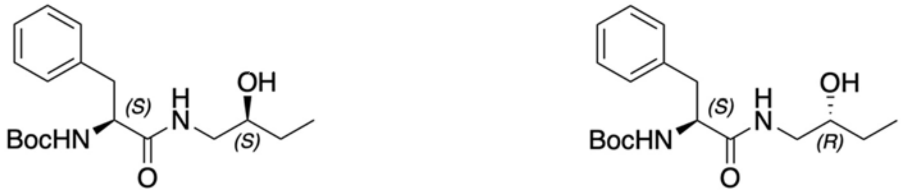

2a-(S,S) (d1) (major) *2a-(S,R) (d2) (minor)*

^{1}H NMR (700 MHz, $CDCl_3$) δ 7.32 – 7.27 (m, 2H, *d*1+*d*2), 7.26 – 7.17 (m, 3H, *d*1+*d*2), 6.36 (br. s, 0.9H, *d*1), 6.27 (br. s, 0.1 H, *d*2), 5.18 (br.s, 1H, *d*1+*d*2), 4.37 – 4.21 (m, 1H, *d*1+*d*2), 3.57 – 3.49 (m, 0.9H, *d*1), 3.45 – 3.34 (m, 1.1H, *d1*+*d*2), 3.12 – 2.92 (m, 3H, *d*1+*d*2), 2.72 (br. s, 0.9H, *d*1), 2.42 (br. s, 0.1H, *d*2), 1.43 – 1.33 (m, 2H, *d1*+*d*2), 1.40 (s, 0.9H, *d*2), 1.39 (s, 8.1H, *d*1), 0.91 (t, *J* = 7.4 Hz, 3H, *d*1+*d*2) ppm.
^{13}C NMR (176 MHz, $CDCl_3$) δ 172.2 (*d*1), 172.1 (*d*2), 155.7 (*d*1+*d*2), 136.9 (*d*2), 136.8 (*d*1), 129.5 (*d*2), 129.4 (*d*1), 128.84 (*d*2), 128.79 (*d*1), 127.1 (*d*1+*d*2), 80.5 (*d*1+*d*2), 72.4 (*d*2), 72.2 (*d*1), 56.3 (*d*1+*d*2), 45.45 (*d*1), 45.39 (*d*2), 38.9 (*d*2), 38.7 (*d*1), 28.4 (*d*1+*d*2), 27.7 (*d*1+*d*2), 10.0 (*d*1), 9.9 (*d*2) ppm.
$[\alpha]_D^{25.0}$ = +15.1 (*c* 2.00, $CHCl_3$).
Chiral SFC Analysis: CHIRALPAK IA (CO_2:MeOH, 95:05, 2.5 mL min^{-1}, 40 °C, 204 nm) t_R = 9.6 (major), 11.0 (minor) minutes, 10.6:1.0 dr.

dr could not be determined from crude ^{1}H NMR analysis. However, comparison of the peak integrals at 6.36 / 6.27 ppm and 2.72 / 2.42 ppm (the N-H and O-H protons respectively for each diastereomer) indicates approx. 10:1 dr *via* ^{1}H NMR analysis of the isolated product. Chiral SFC analysis of the isolated product indicates 10.6:1 dr. We therefore report **2a** as 10:1 dr.

tert-butyl ((_R_)-1-(((_S_)-2-hydroxybutyl)amino)-1-oxo-3-phenylpropan-2-yl)carbamate (2b)

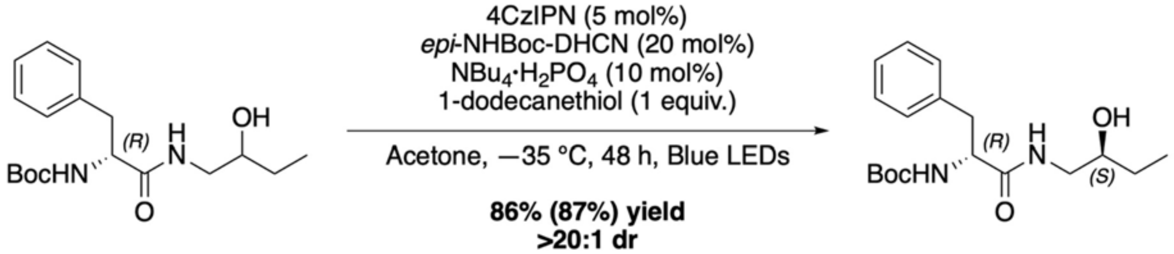

2b (1:1 mix of (_R_,_S_) and (_R_,_R_)) **2b**

2b was prepared according to **General Procedure G** using amino alcohol-derived substrate **2b (1:1 mix of (_R_,_S_) and (_R_,_R_))** (33.6 mg, 0.10 mmol). Purification by silica gel column chromatography (elution with 3% MeOH in DCM) afforded **2b** as a white solid, isolated as a mixture of diastereomers (28.8 mg, 0.086 mmol, 86% yield, >20:1 dr). Combined signals for both diastereomers are denoted _d_1+_d_2. Discernible signals are denoted _d_1 and _d_2. Approximately 24:1 dr is observed from ^{1}H NMR analysis of the isolated product. The majority of _d_2 ^{13}C NMR signals are too weak to be observed.

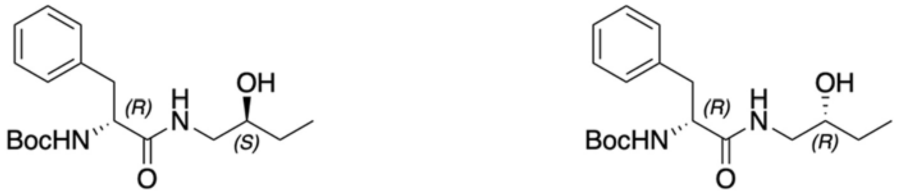

2b-(R,S) (d1) (major) *2b-(R,R) (d2) (minor)*

^{1}H NMR (700 MHz, $CDCl_3$) δ 7.33 – 7.27 (m, 2H, _d_1+_d_2), 7.26 – 7.19 (m, 3H, _d_1+_d_2), 6.42 (br. s, 0.04H, _d_2), 6.32 (br. s, 0.96 H, _d_1), 5.21 (br.s, 1H, _d_1+_d_2), 4.41 – 4.15 (m, 1H, _d_1+_d_2), 3.56 – 3.50 (m, 0.04H, _d_2), 3.50 – 3.31 (m, 1.96H, _d1_+_d_2), 3.16 – 2.85 (m, 3H, _d_1+_d_2), 2.35 (br. s, 1H, _d_1+_d_2), 1.46 – 1.31 (m, 2.36H, _d1_+_d_2), 1.40 (s, 8.64H, _d_1), 0.90 (t, _J_ = 7.4 Hz, 1.5H, _d_1+_d_2), ppm.
^{13}C NMR (176 MHz, $CDCl_3$) δ 172.1 (_d_1), 155.7 (_d_1+_d_2), 136.9 (_d_1), 129.5 (_d_1), 128.8 (_d_1), 127.1 (_d_1), 80.4 (_d_1+_d_2), 72.3 (_d_1), 56.3 (_d_1), 45.4 (_d_1), 38.9 (_d_1), 28.4 (_d_1+_d_2), 27.7 (_d_1+_d_2), 10.0 (_d_2), 9.9 (_d_1) ppm.
$[\alpha]_D^{25.0}$ = +11.5 (_c_ 2.59, $CHCl_3$).
Chiral SFC Analysis: CHIRALPAK IJ (CO_2:MeOH, 97:03, 2.5 mL min^{-1}, 40 °C, 216 nm) t_R = 4.6 (minor), 5.0 (major) minutes, 39:1 dr.

dr could not be determined from crude ^{1}H NMR analysis. However, comparison of the peak integrals at 6.42 / 6.32 ppm (the N-H protons for each diastereomer) indicates approx. 25:1 dr _via_ ^{1}H NMR analysis of the isolated product. Chiral SFC analysis of the isolated product indicates 39:1 dr. We therefore report **2b** as >20:1 dr.

***N*-((*S*)-2-hydroxybutyl)-2-(4-isobutylphenyl)propanamide (2c)**

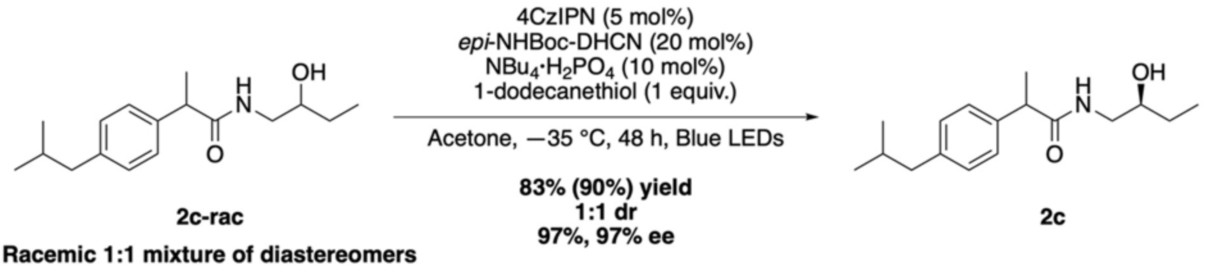

2c was prepared according to **General Procedure G** using amino alcohol-derived substrate **2c-rac** (27.7 mg, 0.10 mmol). Purification by silica gel column chromatography (elution with 3% MeOH in DCM) afforded **2c** as an off-white solid, isolated as a mixture of diastereomers (23.0 mg, 0.083 mmol, 83% yield, 1:1 dr, 97%, 97% ee).

^{1}H NMR (700 MHz, $CDCl_3$) δ 7.19 (d, *J* = 7.6 Hz, 2H), 7.11 (d, *J* = 7.6 Hz, 2H), 5.81 (br. s, 1H), 3.59 – 3.51 (m, 2H), 3.43 – 3.35 (m, 1H), 3.12 – 3.04 (m, 1H), 2.45 (d, *J* = 7.1 Hz, 2H), 2.17 (br. s, 1H) (overlaps with H_2O peak), 1.84 (app. n, *J* = 6.8 Hz, 1H), 1.51 (d, *J* = 7.1 Hz, 3H), 1.43 – 1.34 (m, 2H), 0.92 – 0.87 (m, 9H) ppm.
^{13}C NMR (176 MHz, $CDCl_3$) δ 176.0, 175.9, 140.97, 140.95, 138.5, 129.81, 129.80, 127.4, 73.01, 72.98, 46.82, 46.81, 45.7, 45.6, 45.1, 30.3, 27.94, 27.93, 22.5, 18.59, 18.58, 9.9 ppm.
^{13}C NMR (176 MHz, $(CD_3)_2SO$) δ 173.59, 173.55, 139.7, 139.6, 139.1, 128.69, 128.68, 126.98, 126.95, 70.5, 70.3, 44.63, 44.59, 44.56, 44.2, 29.64, 29.63, 27.22, 27.18, 22.18, 22.17, 22.16, 22.15, 18.7, 18.5, 9.82, 9.80 ppm.
$[\alpha]_D^{25.0}$ = +4.5 (*c* 2.09, $CHCl_3$) (note a 1:1 mixture of (*R*,*S*):(*S*,*S*) diastereomers).
Chiral HPLC Analysis: CHIRALPAK IH (Hexane:^{i}PrOH, 99:01, 1.25 mL min^{-1}, 40 °C, 220 nm) t_R = 68.5 (minor), 70.3 (minor), 74.9 (major), 82.3 (major) minutes, 97%, 97% ee.

Like with **2c-rac**, the two diastereomers of **2c** could not be distinguished by ^{1}H NMR in $CDCl_3$. However, discernable signals for each diastereomer are confirmed to be observed in the ^{13}C NMR spectrum in $(CD_3)_2SO$. For **2c** these ^{13}C NMR signals integrate 1:1, consistent with approximately 1:1 dr.

Opposite Enantiomer Product Characterization

Yields are denoted as *isolated yield*% (*NMR yield*%) where NMR yield could be determined.

(*R*)-*N*-(2-hydroxybutyl)acetamide ((*R*)-1a)

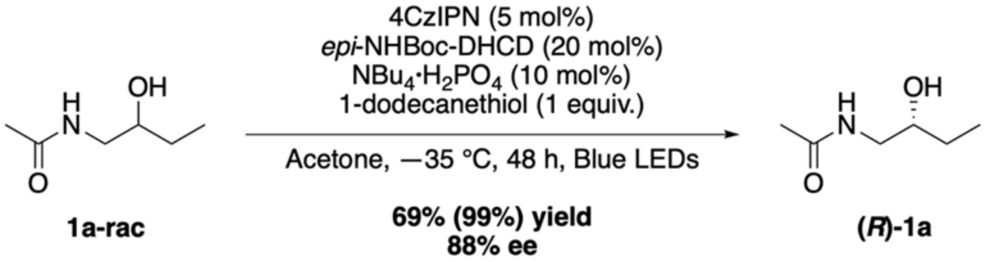

(*R*)-1a was prepared according to **General Procedure G** using amino alcohol-derived substrate **1a-rac** (13.1 mg, 0.10 mmol) and *epi*-NHBoc-DHCD (7.9 mg, 0.020 mmol). Purification by silica gel column chromatography (elution with 6% MeOH in DCM) afforded **(*R*)-1a** as a pale yellow oil (9.0 mg, 0.069 mmol, 69% yield).

^{1}H NMR (700 MHz, $CDCl_3$) δ 5.96 (br. s, 1H), 3.67 – 3.60 (m, 1H), 3.51 – 3.45 (m, 1H), 3.15 – 3.08 (m, 1H), 2.62 – 2.45 (m, 1H), 2.01 (s, 3H), 1.54 – 1.44 (m, 2H), 0.96 (t, *J* = 7.5 Hz, 3H) ppm.
^{13}C NMR (176 MHz, $CDCl_3$) δ 171.3 (2C), 73.0 (2C), 45.6, 28.1 (2C), 23.4, 9.9 ppm.
$[\alpha]_D^{25.0}$ = –18.4 (*c* 0.39, $CHCl_3$).

(*R*)-1-(*N*-acetylbenzamido)butan-2-yl benzoate ((*R*)-1a-Bz)

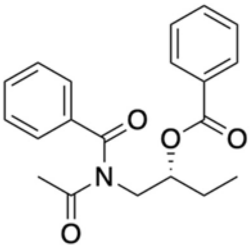

(*R*)-1a-Bz

(*R*)-1a-Bz was prepared according to **General Procedure H** using **(*R*)-1a** (4.5 mg, 0.034 mmol), DMAP (0.8 mg, 0.0065 mmol), triethylamine (0.02 mL, 0.14 mmol) and benzoyl chloride (0.01 mL, 0.086 mmol) in DCM (0.4 mL). Purification by silica gel column chromatography (elution with 0.5% MeOH in DCM followed by a second purification eluting with 20% acetone in petroleum ether) afforded **(*R*)-1a-Bz** (5.6 mg, 0.017 mmol, 48% yield, 88% ee).

^{1}H NMR (700 MHz, CD_3Cl) δ 7.91 – 7.87 (m, 2H), 7.53 – 7.46 (m, 4H), 7.40 – 7.33 (m, 4H), 5.41 – 5.34 (m, 1H), 4.17 (dd, *J* = 14.3, 8.7 Hz, 1H), 4.10 (dd, *J* = 14.3, 2.9 Hz, 1H), 2.05 (s, 3H), 1.78 – 1.66 (m, 2H), 0.97 (t, *J* = 7.5 Hz, 3H) ppm.

^{13}C NMR (176 MHz, CD_3Cl) δ 174.1, 173.4, 166.3, 135.3, 133.1, 132.7, 130.1, 129.7, 128.94, 128.87, 128.4, 74.7, 49.1, 26.3, 25.5, 9.6 ppm
Chiral SFC Analysis: CHIRALPAK IK (CO_2:MeOH, 95:05, 2.5 mL min^{-1}, 40 °C, 228 nm) t_R = 5.4 (minor), 6.2 (major) minutes, 88% ee.

(*R*)-*N*-(5-cyclohexyl-2-hydroxypentyl)acetamide ((*R*)-1f)

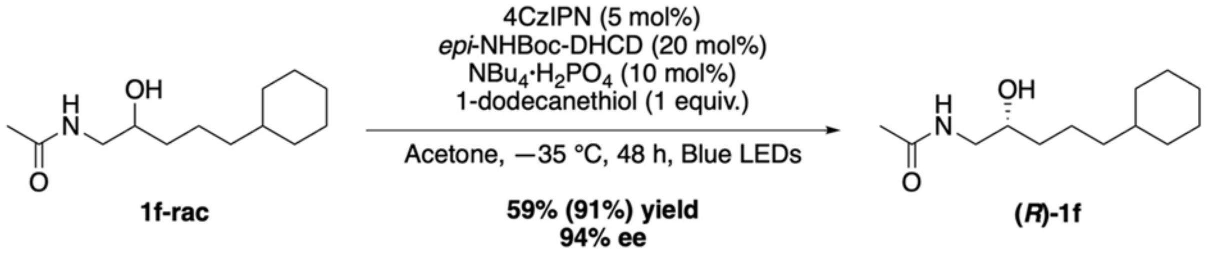

(*R*)-1f was prepared according to **General Procedure G** using amino alcohol-derived substrate **1f-rac** (22.7 mg, 0.10 mmol) and *epi*-NHBoc-DHCD (7.9 mg, 0.020 mmol). Purification by silica gel column chromatography (elution with 0.7% 7N ammonia in MeOH, 1.4% MeOH, 97.9% DCM) afforded **(*R*)-1f** as a pale yellow oil that slowly forms a white solid (13.4 mg, 0.059 mmol, 59% yield).

^{1}H NMR (700 MHz, $CDCl_3$) δ 5.94 (br. s, 1H), 3.74 – 3.66 (m, 1H), 3.48 (ddd, *J* = 13.9, 6.5, 2.7 Hz, 1H), 3.09 (ddd, *J* = 13.9, 8.1, 5.3 Hz, 1H), 2.50 – 2.36 (m, 1H), 2.01 (s, 3H), 1.71 – 1.65 (m, 4H), 1.65 – 1.58 (m, 1H), 1.47 – 1.39 (m, 3H), 1.36 – 1.29 (m, 1H), 1.24 – 1.09 (m, 6H), 0.90 – 0.81 (m, 2H) ppm.
^{13}C NMR (176 MHz, $CDCl_3$) δ 171.2, 71.6, 46.0, 37.7, 37.5, 35.5, 33.5, 26.8, 26.5, 23.4, 22.9 ppm.
$[\alpha]_D^{25.0}$ = –13.7 (*c* 0.59, $CHCl_3$).

(*R*)-1-(*N*-acetylbenzamido)-5-cyclohexylpentan-2-yl benzoate ((*R*)-1f-Bz)

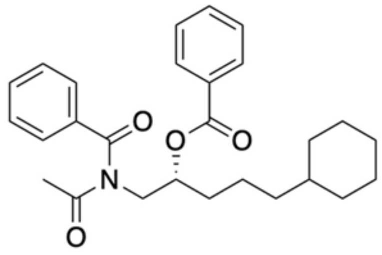

(*R*)-1f-Bz

(*R*)-1f-Bz was prepared according to **General Procedure H** using **(*R*)-1f** (6.7 mg, 0.029 mmol), DMAP (0.8 mg, 0.0065 mmol), triethylamine (0.02 mL, 0.14 mmol) and benzoyl chloride (0.01 mL, 0.086 mmol) in DCM (0.4 mL). Purification by silica gel column chromatography (elution with 0.5% MeOH in DCM followed by a second purification eluting with 5% acetone in petroleum ether) afforded **(*R*)-1f-Bz** (10.7 mg, 0.025 mmol, 83% yield, 94% ee).

^{1}H NMR (700 MHz, CD_3Cl) δ 7.91 – 7.86 (m, 2H), 7.53 – 7.46 (m, 4H), 7.39 – 7.32 (m, 4H), 5.46 – 5.38 (m, 1H), 4.16 (dd, *J* = 14.3, 8.7 Hz, 1H), 4.10 (14.3, 2.8 Hz, 1H), 2.04 (s, 3H), 1.69 – 1.58 (m, 7H), 1.42 – 1.33 (m, 2H), 1.22 – 1.06 (m, 6H), 0.86 – 0.77 (m, 2H) ppm.
^{13}C NMR (176 MHz, CD_3Cl) δ 174.1, 173.4, 166.3, 135.3, 133.0, 132.7, 130.2, 129.8, 128.94, 128.85, 128.4, 73.6, 49.4, 37.5, 37.3, 33.5, 33.4, 32.8, 26.8, 26.49, 26.48, 26.3, 22.5 ppm.
Chiral SFC Analysis: CHIRALPAK IK (CO_2:MeOH, 95:05, 2.5 mL min^{-1}, 40 °C, 228 nm) t_R = 8.7 (minor), 11.3 (major) minutes, 94% ee.

(*R*)-6-chloro-*N*-(2-hydroxybutyl)nicotinamide ((*R*)-1zl)

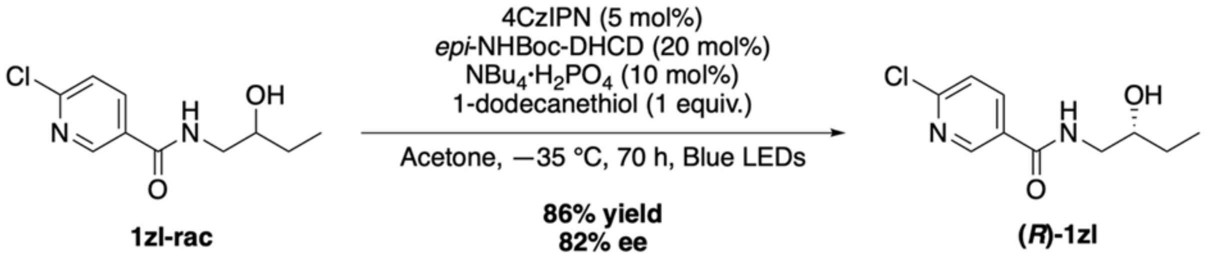

(*R*)-1zl was prepared according to **General Procedure G** using amino alcohol-derived substrate **1zl-rac** (22.9 mg, 0.10 mmol) and *epi*-NHBoc-DHCD (7.9 mg, 0.020 mmol) stirred for 70 h. Purification by silica gel column chromatography (elution with 4% MeOH in DCM) afforded **(*R*)-1zl** as a white solid (19.6 mg, 0.086 mmol, 86% yield, 82% ee).

^{1}H NMR (500 MHz, $(CD_3)_2CO$) δ 8.88 (dd, *J* = 2.5, 0.6 Hz, 1H), 8.27 (dd, *J* = 8.3, 2.5 Hz, 1H), 7.99 (br. s, 1H), 7.55 (dd, *J* = 8.3, 0.6 Hz, 1H), 4.05 (d, *J* = 5.0 Hz, 1H), 3.72 – 3.65 (m, 1H), 3.53 (ddd, *J* = 13.5, 6.1, 4.0 Hz, 1H), 3.30 (ddd, *J* = 13.5, 7.5, 5.7 Hz, 1H), 1.59 – 1.49 (m, 1H), 1.49 – 1.38 (m, 1H), 0.96 (t, *J* = 7.5 Hz, 3H) ppm.
^{13}C NMR (126 MHz, $(CD_3)_2CO$) δ 165.3, 154.0, 149.8, 139.1, 130.7, 124.8, 72.2 (2C), 46.8 (2C), 28.6, 10.3 ppm.
$[\alpha]_D^{25.0}$ = –18.6 (*c* 1.75, MeOH).
Chiral SFC Analysis: CHIRALPAK IJ (CO_2:MeOH, 94:06, 2.5 mL min^{-1}, 40 °C, 267 nm) t_R = 6.1 (major), 7.5 (minor) minutes, 82% ee.

(*R*)-*N*-(2-hydroxybutyl)morpholine-4-carboxamide ((*R*)-1zo)

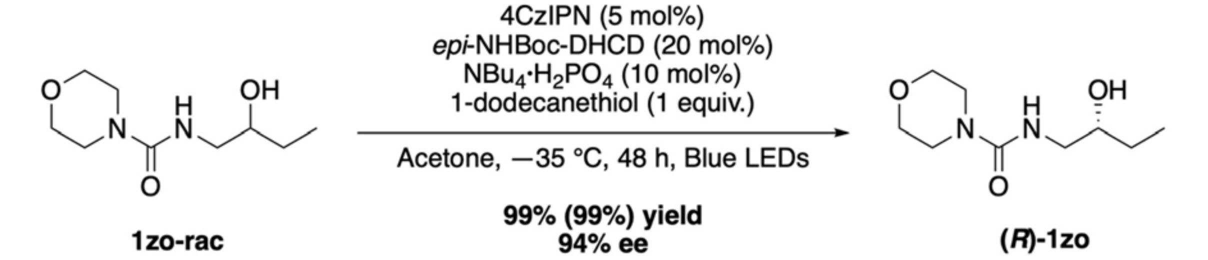

(*R*)-1zo was prepared according to **General Procedure G** using amino alcohol-derived substrate **1zo-rac** (20.2 mg, 0.10 mmol) and *epi*-NHBoc-DHCD (7.9 mg, 0.020 mmol).

Purification by silica gel column chromatography (elution with 4% MeOH in DCM) afforded **(*R*)-1zo** as a white solid (20.1 mg, 0.099 mmol, 99% yield).

^{1}H NMR (700 MHz, $CDCl_3$) δ 5.06 (br. s, 1H), 3.68 (br. t, *J* = 4.9 Hz, 4H), 3.65 – 3.59 (m, 1H), 3.44 (dd, *J* = 14.1, 2.6 Hz, 1H), 3.36 (br. t, *J* = 4.9 Hz, 4H), 3.13 (dd, *J* = 14.1, 7.9 Hz, 1H), 2.64 (br. s, 1H), 1.53 – 1.43 (m, 2H), 0.96 (t, *J* = 7.5 Hz, 3H) ppm.
^{13}C NMR (176 MHz, $CDCl_3$) δ 158.9, 73.6, 66.6, 46.8, 44.2, 28.0, 10.0 ppm.
$[\alpha]_D^{25.0}$ = −9.4 (*c* 0.88, $CHCl_3$).

(*R*)-1-(*N*-benzoylmorpholine-4-carboxamido)butan-2-yl benzoate ((*R*)-1zo-Bz)

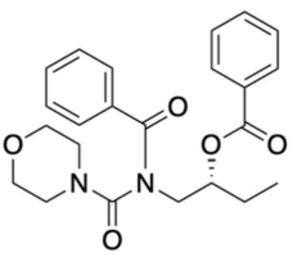

(*R*)-1zo-Bz

(*R*)-1zo-Bz was prepared according to **General Procedure H** using **(*R*)-1zo** (10.0 mg, 0.049 mmol), DMAP (1.1 mg, 0.0090 mmol), triethylamine (0.02 mL, 0.14 mmol) and benzoyl chloride (0.01 mL, 0.086 mmol) in DCM (0.4 mL). Purification by silica gel column chromatography (elution with 1% MeOH in DCM followed by a second purification eluting with 20% acetone in petroleum ether) afforded **(*R*)-1zo-Bz** (10.2 mg, 0.025 mmol, 50% yield, 94% ee).

^{1}H NMR (500 MHz, CD_3Cl) δ 8.05 (d, *J* = 7.9 Hz, 2H), 7.61 (d, *J* = 7.3 Hz, 2H), 7.57 (t, *J* = 7.4 Hz, 1H), 7.49 (t, *J* = 7.5 Hz, 1H), 7.45 (t, *J* = 7.7 Hz, 2H), 7.40 (t, *J* = 7.6 Hz, 2H), 5.38 (br. s, 1H), 4.23 (dd, *J* = 14.3, 9.5 Hz, 1H), 4.14 (dd, *J* = 14.3, 1.9 Hz, 1H), 2.97 (br. s, 8H), 1.87 – 1.73 (m, 2H), 1.03 (t, *J* = 7.5 Hz, 3H) ppm.
^{13}C NMR (126 MHz, CD_3Cl) δ 170.1, 166.2, 157.1, 135.0, 133.5, 132.2, 130.2, 129.9, 128.7, 128.6, 128.3, 75.9 (br.), 65.4, 49.6, 46.0 (br.), 44.7 (br.), 25.7, 9.5 ppm.
Chiral SFC Analysis: CHIRALPAK IK (CO_2:MeOH, 95:05, 2.5 mL min^{-1}, 40 °C, 225 nm) t_R = 9.7 (minor), 10.8 (major) minutes, 94% ee.

Assignment of Absolute Stereochemistry

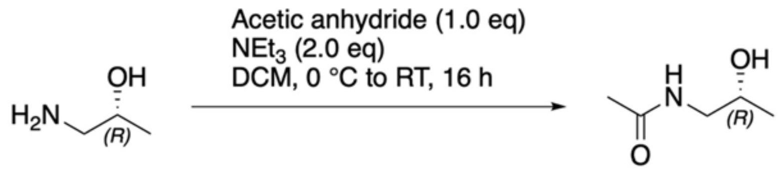

(*R*)-1b

[α]$_D^{25}$ = −22.4 (*c* 1.9, MeOH)
(lit. **[α]**$_D^{20}$ = −23.5 (*c* 1.0, MeOH))[26]

[α]$_D^{25}$ = −35.9 (*c* 1.0, $CHCl_3$)

(*R*)-1b was synthesized by acetylating commercially available (*R*)-1-aminopropan-2-ol (see Scheme 3E for more details). **(*R*)-1b**: **[α]**$_D^{25.0}$ = –35.9 (*c* 1.0, $CHCl_3$).

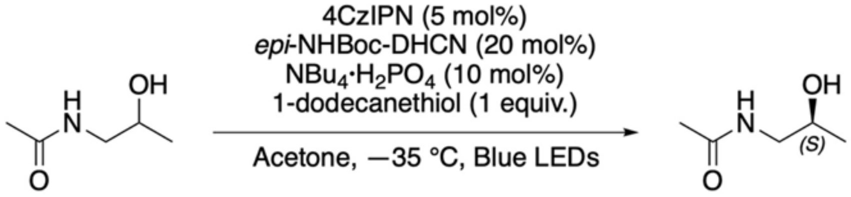

1b-rac

1b

[α]$_D^{25}$ = +28.8 (*c* 0.43, $CHCl_3$)

[α]$_D^{25}$ = +19.5 (*c* 0.44, EtOH)
(lit. **[α]**$_D^{20}$ = −4.0 (*c* 0.4, EtOH) for **(*R*)-1b**)[25]

In the deracemization of **1b-rac**, for observed product **1b**: **[α]**$_D^{25.0}$ = +28.8 (*c* 0.43, $CHCl_3$). This is the opposite sign compared to **(*R*)-1b**, confirming formation of the (*S*)-enantiomer. Further, for **1b** in EtOH: **[α]**$_D^{25.0}$ = +19.5 (*c* 0.44, EtOH). This is the opposite sign to **(*R*)-1b** as reported in the literature in EtOH: lit. **[α]**$_D^{20}$ = –4.0 (*c* 0.4, EtOH).[25] The absolute stereochemistry of **1b** is therefore determined to be the (*S*)-enantiomer.

By tentative analogy, we therefore report that using *epi*-NHBoc-DHCN as HAA catalyst produces the (*S*)-enantiomer as the product of the deracemization reaction. Using *epi*-NHBoc-DHCD as HAA catalyst produces the (*R*)-enantiomer.

Deacetylation with Retention of Stereochemistry

The deacetylation of *N*-acetyl amino alcohol deracemization products can be performed without loss of enantioselectivity. This was demonstrated in the deacetylation of **1s**. **(*S*)-HCl-1s-I_2** was Boc-protected *in-situ* to generate **Boc-1s** for facile SFC analysis.

***tert*-butyl (*S*)-(2-hydroxy-3-(*p*-tolyl)propyl)carbamate (Boc-1s)**

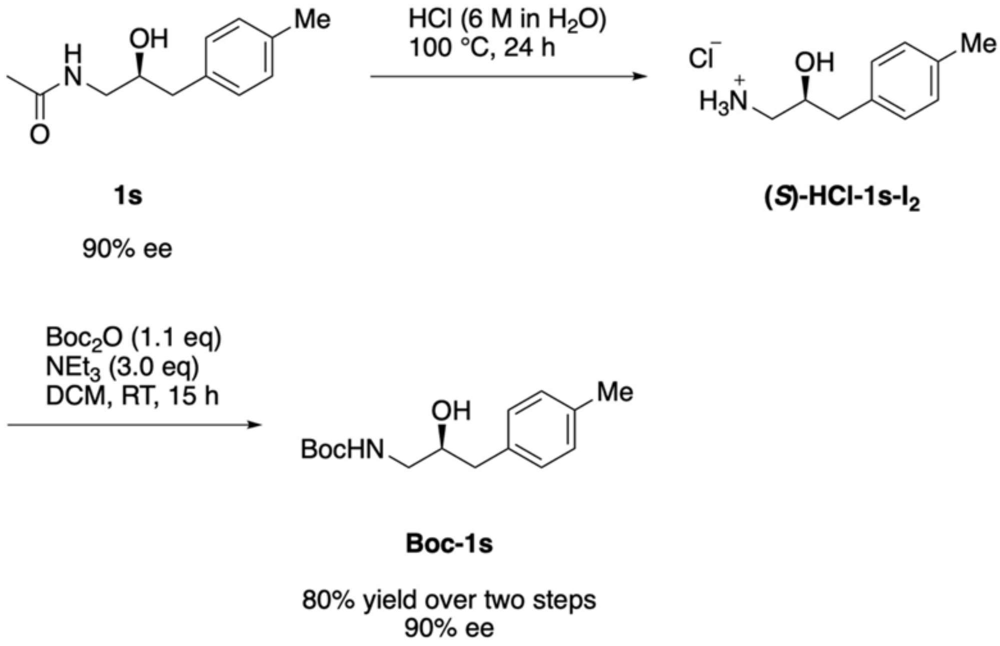

HCl (6 M in H_2O, 3 mL) was added to *N*-acetyl amino alcohol substrate **1s** (15.7 mg, 0.076 mmol) in a microwave vial. The microwave vial was sealed with a crimp-top cap and the reaction mixture stirred vigorously at 100 °C for 24 h. The crude material was concentrated *in vacuo* to afford crude **(*S*)-HCl-1s-I_2** as an off-white solid. DCM (2 mL) and triethylamine (0.03 mL, 0.22 mmol) were added sequentially to crude **(*S*)-HCl-1s-I_2** and the mixture stirred for 5 min to form a homogenous solution. Boc anhydride (0.02 mL, 0.087 mmol) was added slowly and the reaction mixture stirred overnight for 15 h at RT. The crude mixture was concentrated *in vacuo* and purified by silica gel column chromatography (elution with 15% acetone in petroleum ether) to afford **Boc-1s** as a colorless solid (16.1 mg, 0.061 mmol, 80% yield over two steps, 90% ee).

^{1}H NMR (700 MHz, $CDCl_3$) δ 7.12 (d, *J* = 8.0 Hz, 2H), 7.10 (d, *J* = 8.0 Hz, 2H), 4.94 (br. s, 1H), 3.95 – 3.82 (m, 1H), 3.42 – 3.31 (m, 1H), 3.06 (ddd, *J* = 13.4, 7.1, 6.2 Hz, 1H), 2.76 (dd, *J* = 13.7, 5.1 Hz, 1H), 2.67 (dd, *J* = 13.7, 8.1 Hz, 1H), 2.32 (s, 3H), 1.45 (s, 9H) ppm.
^{13}C NMR (176 MHz, $CDCl_3$) δ 156.9, 136.3, 134.6, 129.5, 129.4, 79.8, 72.6, 46.1, 41.0, 28.5, 21.2 ppm.
HRMS $[M+Na]^+$ m/z calc'd for $[C_{15}H_{23}NO_3Na]^+$ expect 288.1570; found 288.1564.
$[\alpha]_D^{25.0}$ = +11.0 (*c* 1.43, $CHCl_3$).
Chiral SFC Analysis: CHIRALPAK IK (CO_2:MeOH, 95:05, 2.5 mL min^{-1}, 40 °C, 212 nm) t_R = 7.4 (minor), 7.8 (major) minutes, 90% ee.

Racemic sample for SFC analysis was prepared by analogous Boc protection of racemic **1s-I_2**. An analytical sample was obtained by purification by preparative thin layer chromatography (elution with 5% MeOH in DCM).

Evaluation of a 1,3-Amino Alcohol

***N*-(3-hydroxypentyl)acetamide (6-rac)**

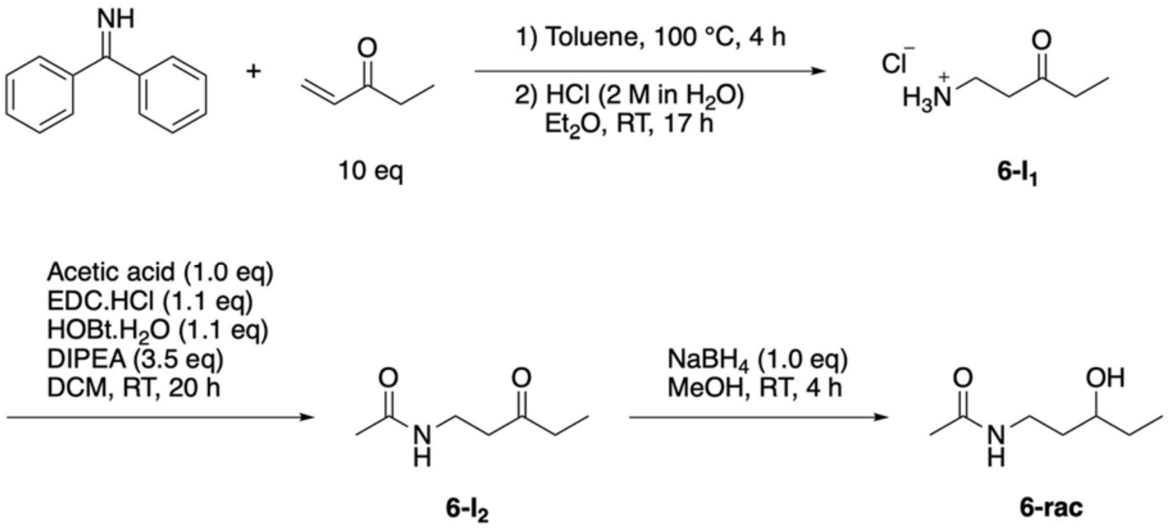

A solution of benzophenone imine (1.34 mL, 8.00 mmol) and 1-penten-3-one (7.96 mL, 80.0 mmol) in dry toluene (16 mL) was stirred at 100 °C for 4 h in an oven-dried Schlenk flask under N_2 atmosphere. The crude mixture was concentrated *in vacuo* and redissolved in diethyl ether (60 mL) and HCl (2 M in H_2O, 21 mL) and stirred overnight for 17 h at RT. The aqueous phase was washed with diethyl ether and extensively concentrated *in vacuo* to afford crude **6-I$_1$** as an off-white solid (1.06 g). DIPEA (4.59 mL, 26.8 mmol) was added to a stirred suspension of crude **6-I$_1$**, acetic acid (0.44 mL, 7.69 mmol), EDC.HCl (1.61 g, 8.40 mmol) and HOBt.H_2O (≥20 wt% H_2O, 1.61 g, 8.41 mmol) in DCM (40 mL) and the reaction mixture was stirred overnight for 20 h. The reaction mixture was concentrated *in vacuo*, water was added and the crude extracted with ethyl acetate. The combined organic extracts were dried over $MgSO_4$, filtered, and the solvent removed *in vacuo* to afford crude mixture. The crude mixture was purified by silica gel column chromatography (elution with 3% MeOH in DCM) to afford **6-I$_2$** as a white solid (654 mg). A solution of **6-I$_2$** in dry MeOH (15 mL) was immediately formed, and sodium borohydride (170 mg, 4.49 mmol) was added slowly at 0 °C. The reaction mixture was warmed to RT and stirred for 4 h. The reaction mixture was diluted with H_2O, further stirred for 1 h, concentrated extensively *in vacuo* and purified by silica gel column chromatography (elution with 7% MeOH in DCM) to afford **6-rac** as a pale yellow oil (645 mg, 4.44 mmol, 56% yield over three steps).

^{1}H NMR (700 MHz, $CDCl_3$) δ 6.09 (br. s, 1H), 3.68 (dddd, *J* = 14.0, 9.7, 7.2, 4.3 Hz, 1H), 3.53 (dddd, *J* = 9.8, 7.4, 5.1, 2.5 Hz, 1H), 3.14 (app. dq, *J* = 14.5, 4.8 Hz, 1H), 2.89 (br. s, 1H), 2.00 (s, 3H), 1.64 (dddd, *J* = 14.3, 9.7, 4.9, 2.8 Hz, 1H), 1.54 – 1.44 (m, 3H), 0.94 (t, *J* = 7.5 Hz, 3H) ppm.
^{13}C NMR (176 MHz, $CDCl_3$) δ 171.3, 70.7, 37.0, 36.7, 30.3, 23.3, 10.3 ppm.
HRMS $[M+H]^+$ m/z calc'd for $[C_7H_{15}NO_2H]^+$ expect 146.1176; found 146.1171.

(*S*)-*N*-(3-hydroxypentyl)acetamide (6)

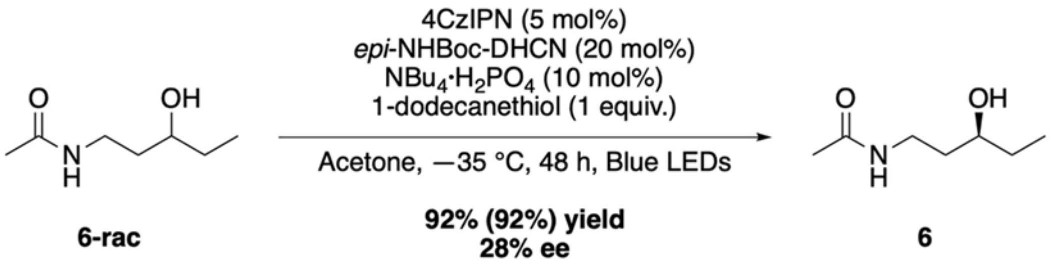

6 was prepared according to **General Procedure G** using amino alcohol-derived substrate **6-rac** (14.5 mg, 0.10 mmol). Purification by silica gel column chromatography (elution with 6% MeOH in DCM) afforded **6** as a pale yellow oil (13.4 mg, 0.092 mmol, 92% yield).

^{1}H NMR (700 MHz, $CDCl_3$) δ 6.11 (br. s, 1H), 3.67 (dddd, *J* = 14.0, 9.7, 7.2, 4.4 Hz, 1H), 3.53 (dddd, *J* = 9.8, 7.4, 5.1, 2.5 Hz, 1H), 3.14 (app. dq, *J* = 14.6, 4.9 Hz, 1H), 2.62 (br. s, 1H), 1.99 (s, 3H), 1.64 (dddd, *J* = 14.3, 9.7, 4.9, 2.8 Hz, 1H), 1.54 – 1.44 (m, 3H), 0.94 (t, *J* = 7.5 Hz, 3H) ppm.
^{13}C NMR (176 MHz, $CDCl_3$) δ 171.3, 70.7, 37.0, 36.7, 30.3, 23.3, 10.3 ppm.

(*S*)-1-(*N*-acetylbenzamido)pentan-3-yl benzoate (6-Bz)

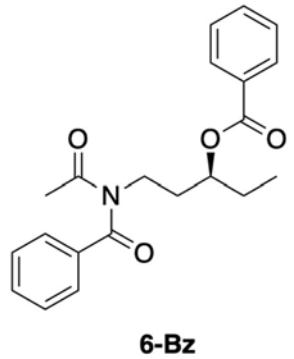

6-Bz

6-Bz was prepared according to **General Procedure H** using **6** (6.7 mg, 0.046 mmol), DMAP (0.9 mg, 0.0074 mmol), triethylamine (0.02 mL, 0.14 mmol) and benzoyl chloride (0.01 mL, 0.086 mmol) in DCM (0.4 mL). Purification by silica gel column chromatography (elution with 10% acetone in petroleum ether followed by a second purification eluting with 0.5% MeOH in DCM) afforded **6-Bz** (6.8 mg, 0.019 mmol, 42% yield, 28% ee).

^{1}H NMR (700 MHz, CD_3Cl) δ 7.92 (d, *J* = 8.1 Hz, 2H), 7.58 (d, *J* = 8.4 Hz, 2H), 7.54 (t, *J* = 7.4 Hz, 1H), 7.46 (t, *J* = 7.4 Hz, 1H), 7.42 – 7.37 (m, 4H), 5.05 – 4.99 (m, 1H), 3.88 (t, *J* = 7.7 Hz, 2H), 2.15 (s, 3H), 2.06 – 1.95 (m, 2H), 1.74 – 1.66 (m, 2H), 0.92 (t, *J* = 7.5 Hz, 3H) ppm.
^{13}C NMR (176 MHz, CD_3Cl) δ 174.5, 173.4, 166.3, 135.6, 132.9, 132.5, 130.5, 129.7, 128.9, 128.43, 128.40, 73.9, 43.4, 32.8, 27.1, 26.5, 9.6 ppm.
HRMS $[M+H]^+$ m/z calc'd for $[C_{21}H_{23}NO_4H]^+$ expect 354.1700; found 354.1711.
Chiral SFC Analysis: CHIRALPAK IE (CO_2:MeOH, 90:10, 2.5 mL min^{-1}, 40 °C, 227 nm) t_R = 7.2 (minor), 8.0 (major) minutes, 28% ee.

Poor enantioselectivity is observed for **6**. It is therefore broadly concluded that 1,3-*N*-acetyl amino alcohol substrates are less effective than 1,2-*N*-acetyl amino alcohol substrates with the current reaction conditions.

D_2O Additive Experiments with Substrate 1y-rac

To probe whether or not HAA is occurring at the primary alcohol during deracemization of substrate **1y-rac**, the reaction with D_2O as an additive was performed.

Products were prepared according to **General Procedure G** for 24 h, with H_2O added from a freshly prepared 0.1 M stock solution of H_2O in degassed acetone and D_2O added from a freshly prepared 0.25 M stock solution of D_2O in degassed acetone. A reaction molarity of 0.025 M was used throughout. Deuterium incorporation was determined from the ^1H NMR of the isolated material, with the assumption that minimal deuteration would occur at the acetyl CH_3. Silica gel column chromatography (elution with 4% 7N ammonia in MeOH, 8% MeOH, 88% DCM) afforded the product when necessary. Enantioselectivity was determined by derivatization to the dibenzoylated product **1y-Bz** (**General Procedure H**) either directly on the crude material or the isolated product, with an analytical sample obtained by purification by preparative thin layer chromatography (elution with 25% acetone in petroleum ether).

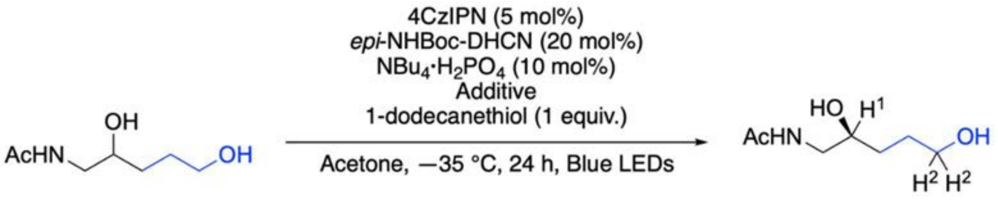

1y-rac **1y-*d***

Entry	Additive	%D at H^1	%D at H^2	Yield[a]	ee[b]
1[c]	-	-	-	43% (27%)	74%
2	-	-	-	69%	50%
3	H_2O (1 eq)	-	-	78%	52%
4	D_2O (1 eq)	10%	30%	(69%)	63%
5	D_2O (5 eq)	23%	61%	(90%)	48%

[a]Yield determined by ^1H NMR using CH_2Br_2 as internal standard, except that in parenthesis which refers to isolated yield. [b]Enantiomeric excess (ee) determined by chiral SFC analysis on the dibenzoylated derivative. [c]Reaction time of 48 h (See Main Scope Product Characterization, substrate **1y** for more details).

Entry 1 is the 48h reaction taken from Scheme 1, main manuscript. Entry 2 is the same but run for 24h, with which the entries 3-5 can be compared. Entry 3 is the comparison with 1 equiv. of H_2O. With 1 equiv. D_2O (entry 4), 30% deuterium incorporation *α* to the primary alcohol was observed, confirming that the primary alcohol is reactive to HAA from the catalyst. 10% deuterium incorporation was observed at the position *α* to the secondary alcohol, significantly less than at the primary alcohol. On increasing to 5 equiv. of D_2O, increased deuterium incorporation was observed at both the primary and secondary alcohols, consistent with a higher level of deuterated thiol being present in the reaction mixture on exchange with D_2O.

Entry 4:

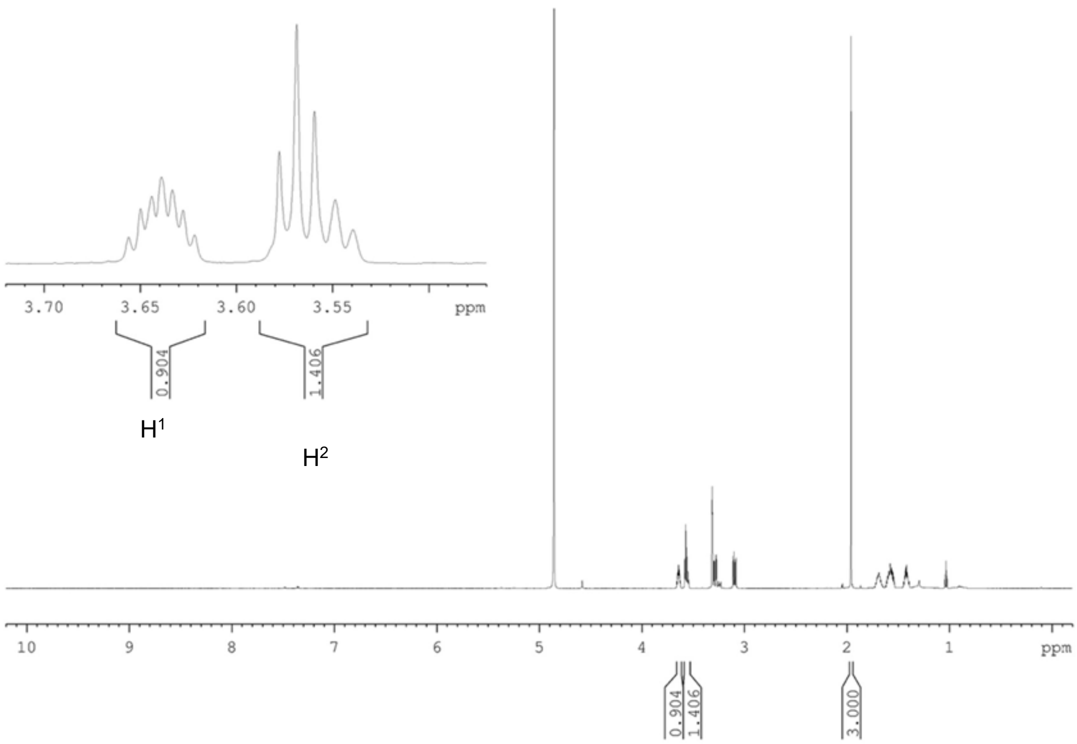

Entry 5:

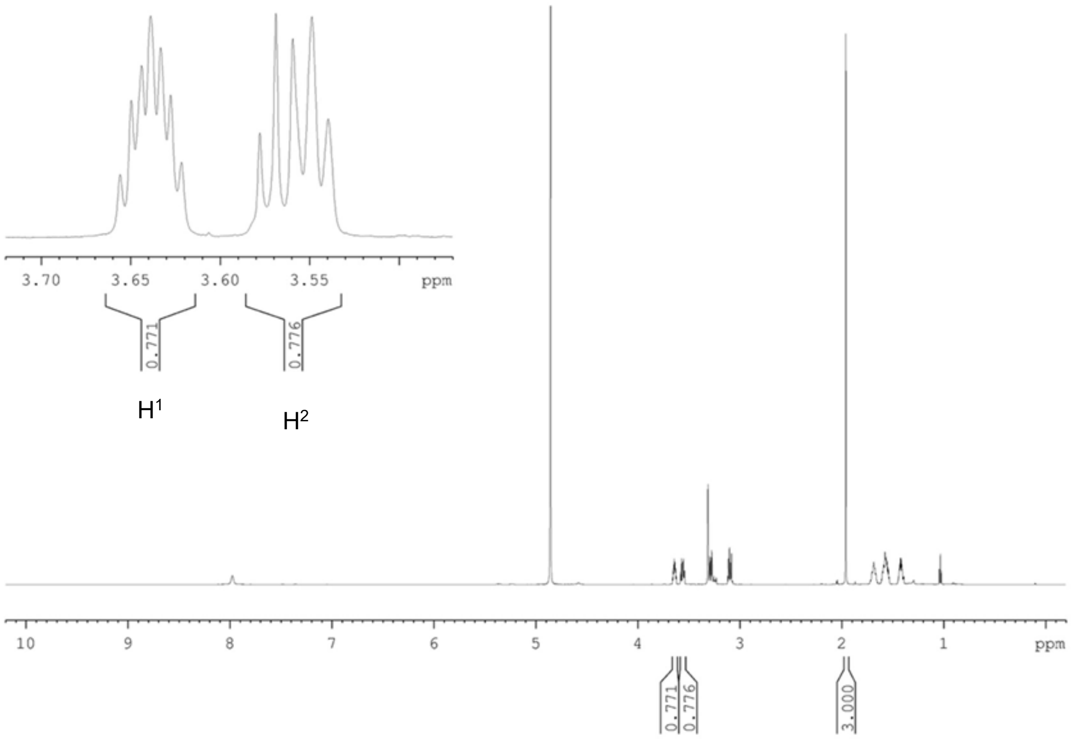

Analysis of HAA Catalyst and Photocatalyst Fate

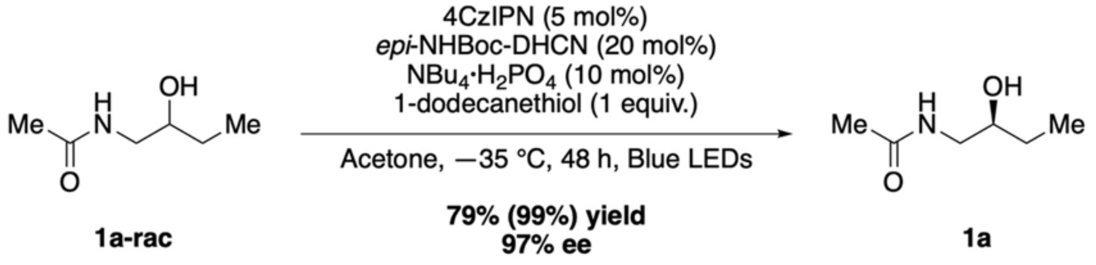

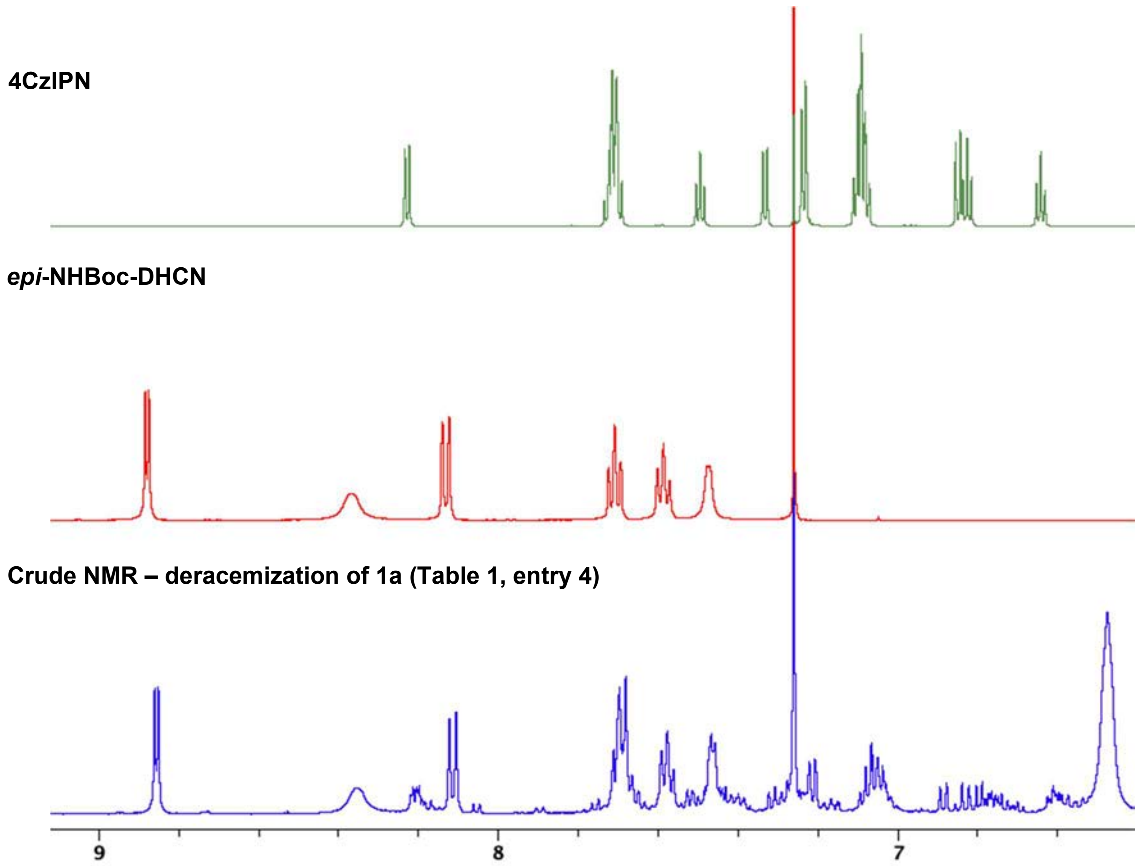

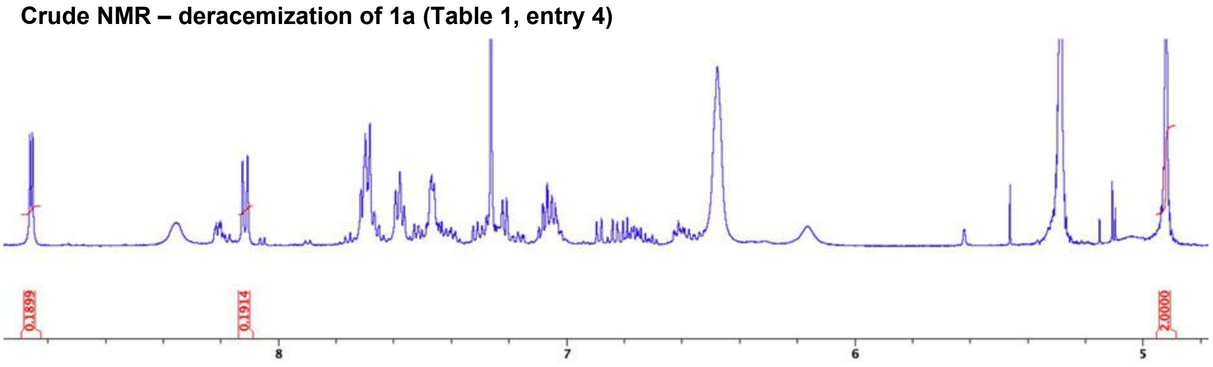

19 mol% of *epi*-NHBoc-DHCN was observed in the crude NMR, evidencing good recovery yield of the HAA catalyst. Evidence of 4CzIPN photocatalyst degradation was observed in the crude NMR.

Mechanistic Experiments

Scheme 3A - Deuterium erosion during deracemization:

***N*-(2-hydroxypropyl-2-d)acetamide (1b-*d*)**

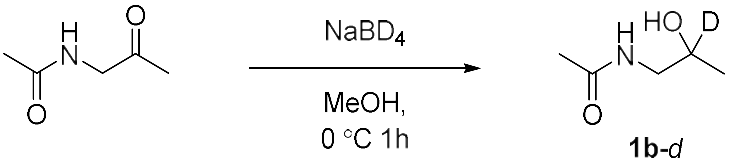

1b-*d*

Sodium borodeuteride (0.4 g, 9.6 mmol) was added portion wise to a solution of *N*-(2-oxopropyl)acetamide (1.0 g, 8.7 mmol) in MeOH (10 mL) at 0 °C. The mixture was stirred for 1 hour and quenched by addition of aqueous saturated NH_4Cl (40 mL). The mixture was extracted with EtOAc, dried over $MgSO_4$, filtered and concentrated *in vacuo*. The crude mixture was purified by silica gel column chromatography (eluting with 7% MeOH in DCM) to afford the title compound **1b-*d*** as a colourless, viscous liquid (0.45 g, 3.8 mmol, 44% yield). Deuterium incorporation (96% D) was determined by ^{1}H NMR of the purified product.

^{1}H NMR (700 MHz, $CDCl_3$) δ 6.02 (br. s, 1H), 3.90 (dqd, *J* = 7.7, 6.3, 3.0 Hz, 1H), 3.41 (dd, *J* = 13.9, 6.5 Hz, 1H), 3.08 (dd, *J* = 13.9, 5.0 Hz, 1H), 2.81 (br. s, 1H), 2.00 (s, 3H), 1.17 (s, 3H) ppm.
^{2}H NMR (700 MHz, $CDCl_3$) δ 3.91 (s, 1H) ppm.
^{13}C NMR (176 MHz, $CDCl_3$) δ 171.5 (CH, CD), 67.4 (CH), 67.0 (t, *J* = 22.0 Hz, CD), 47.3 (CH), 47.2 (CD), 23.3 (CH, CD), 21.0 (CH), 20.9 (CD) ppm.
HRMS $[M+H]^+$ m/z calc'd for $[C_5H_{10}DNO_2H]^+$ expect 119.0925; found 119.0925.

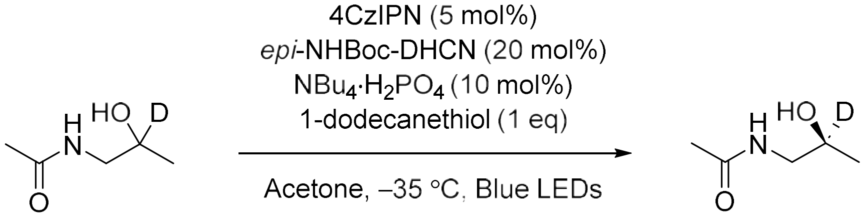

1b-*d*-*rac*
(96% D)

92% yield
98% ee
(42% D)

(*S*)-1b-*d*
(42% D)

1b-*d* was prepared according to **General Procedure G** using amino alcohol-derived substrate **1b-*d*-rac** (11.8 mg, 0.10 mmol). Purification by silica gel column chromatography (elution with 7% MeOH in DCM) afforded **1b-*d*** as a colorless film (10.9 mg, 0.092 mmol, 92% yield). Deuterium incorporation (42% D) was determined by ^{1}H NMR of the purified product (3.94 – 3.86 ppm).

^{1}H NMR (700 MHz, $CDCl_3$) δ 6.25 (br. s, 1H), 3.94 – 3.86 (m, 0.58 H), 3.45 – 3.37 (m, 1H), 3.12 – 3.04 (m, 1H), 2.64 (br. s, 1H), 2.00 (s, 3H), 1.19 – 1.16 (m, 3H) ppm.
^{13}C NMR (176 MHz, $CDCl_3$) δ 171.5 (CH, CD), 67.4 (CH), 67.0 (t, *J* = 22.1 Hz, CD), 47.3 (CH), 47.2 (CD), 23.3 (CH, CD), 21.0 (CH), 20.9 (CD) ppm.

(*S*)-1-(N-acetylbenzamido)propan-2-yl-2-d benzoate (S)-(1b-*d*-Bz)

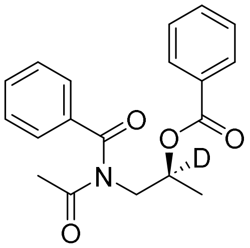

(S)-1b-*d*-Bz
42% D

1b-d-**Bz** was prepared according to **General Procedure H** using **2b-*d*** (8.0 mg, 0.067 mmol), DMAP (0.8 mg (0.007 mmol)), triethylamine (0.02 mL, 0.14 mmol) and benzoyl chloride (0.01 mL, 0.086 mmol) in DCM (0.4 mL). Purification by silica gel column chromatography (elution with 1% MeOH in DCM followed by a second purification eluting with 20% acetone in hexane) afforded **1d-*d*-Bz** (4.6 mg, 0.014 mmol, 21% yield). Deuterium incorporation (42% D) was determined by ^{1}H NMR of the purified product.

^{1}H NMR (700 MHz, $CDCl_3$) δ 7.86 (d, *J* = 8.1 Hz, 2H), 7.54 (d, *J* = 7.1 Hz, 2H), 7.15 – 7.47 (m, 2H), 7.35 (q, *J* = 7.69 Hz, 4H), 5.46 (dqd, *J* = 9.6, 6.1, 3.0 Hz, 0.58 H), 4.18 (dd, *J* = 14.5, 8.5 Hz, <1H), 4.18 (d, *J* = 14.3 Hz, <1H(D)), 4.06 (dd, *J* = 14.3, 3.0 Hz, <1H), 4.06 (d, *J* = 14.3 Hz, <1H(D)), 2.04 (s, 3H), 1.36 (s, <3H(D)), 1.35 (d, *J* = 1.5 Hz, <3H).
^{13}C NMR (176 MHz, $CDCl_3$) δ 174.2, 174.2, 173.3, 173.3, 166.1, 135.3, 133.1, 132.9, 130.1, 129.7, 129.0, 128.9, 128.3, 70.5, 50.4, 50.3, 26.3, 18.0, 17.9.
Chiral SFC Analysis: CHIRALPAK IK (CO_2:MeOH, 95:05, 2.5 mL min^{-1}, 40 °C, 228 nm) t_R = 6.1 (major), 6.5 (minor) minutes, 98% ee.

Scheme 3C - Giese addition (kinetic resolution mode):

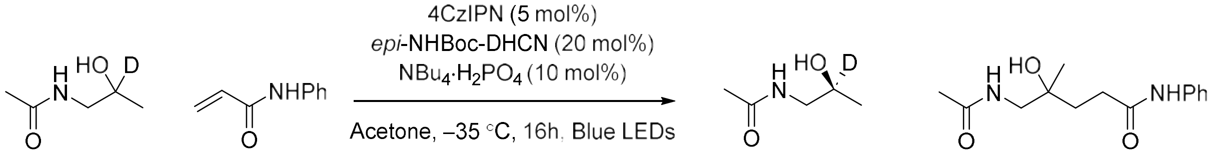

1b-*d*-*rac*
(96% D)

(*S*)-1b-*d*
(96% D)
20% yield (39% NMR)
94% ee
(96% D)

4-*rac*
39% yield (43% NMR)

N-(2-hydroxypropyl-2-d)acetamide **1b-*d*-*rac*** (11.8 mg, 0.10 mmol, 1 eq), *N*-phenylacrylamide (14.7 mg, 0.10 mmol, 1 eq), *epi*-NHBoc-DHCN (7.9 mg, 0.020 mmol, 20 mol%), 4CzIPN (0.005 mmol, 5 mol%) and $TBAH_2PO_4$ (0.010 mmol, 10 mol%) were measured into a 4.0 mL, crimp-top vial. The vial was sealed, evacuated and backfilled with nitrogen 5 to 10 times. 4 mL of degassed acetone was added *via* syringe to the sealed vial. The vial was further sealed with teflon and electrical tape and was placed in the custom made photoreactor. The reaction vial was cooled to -35 °C (5-10 minutes) and the Kessil® A160WE Tuna blue lamp (100% intensity, 100% ocean blue colour) was switched on. Reaction time was 16h. The crude mixture was evaporated *in vacuo* and the NMR-yield was analysed by ^{1}H NMR using internal standard (CH_2Br_2). The material was subjected to purification by silica gel column chromatography (eluting with 5% 7N NH_3 in MeOH in DCM). The fractions containing the Giese product **4-*rac*** were repurified by using preparatory thin layer chromatography (eluting with 10% MeOH in

DCM) to afford the **4**-*rac* as transparent film (10.2 mg, 0.039 mmol, 39%, 43% NMR-yield) which upon chiral SFC analysis was found to be racemic. No deuteration was observed in the Giese product **4**-*rac*.

Fractions containing the recovered starting material **1b**-*d*-rac were repurified by column chromatography (eluting with 10% MeOH in DCM) to afford the starting material **1b**-*d*-rac (2.4 mg, 0.020 mmol, 20% yield, 39% NMR-yield). This material was further subjected to benzoylation conditions according to **General Procedure H** using **(*S*)-1b-*d*** (2.4 mg, 0.020 mmol), few crystals of DMAP, triethylamine (0.02 mL, 0.14 mmol) and benzoyl chloride (0.01 mL, 0.086 mmol) in DCM (0.4 mL). The crude benzoylation material was purified by silica gel column chromatography (elution with 1% MeOH in DCM followed by a second purification eluting with 20% acetone in hexane) which afforded **1b-Bz-*d*** (3.1 mg, 0.014 mmol, 69% yield, 94% ee). Level of deuteration (96% D) was obtained from the purified **(*S*)-1b-*d*** and **1b-Bz-*d*.** Data of for **(*S*)-1b-*d*** and **1b-Bz-*d*** are in accordance with the data reported above.

5-acetamido-4-hydroxy-4-methyl-*N*-phenylpentanamide (4-*rac*)

^{1}H NMR (700 MHz, $CDCl_3$) δ 8.31 (br. s, 1H), 7.52 (d, *J* = 7.7 Hz, 2H), 7.29 (7, *J* = 7.9 Hz, 2H), 7.09 (t, *J* = 7.4 Hz, 1H), 6.39 (t, *J* = 5.6 Hz, 1H), 3.38 (dd, *J* = 13.9, 6.7 Hz, 1H), 3.18 (dd, *J* = 13.9, 5.7 Hz, 1H), 2.53 (td, *J* = 7.3, 1.4 Hz, 2H), 2.02 (s, 3H), 1.91 (dt, *J* = 14.4, 7.5 Hz, 1H), 1.79 (dt, *J* = 14.4, 7.0 Hz, 1H), 1.17 (s, 3H) ppm.
^{13}C NMR (176 MHz, $CDCl_3$) δ 172.6, 172.0, 138.2, 129.1, 124.4, 120.1, 72.4, 49.1, 34.8, 32.6, 25.1, 23.4 ppm.
HRMS $[M+H]^+$ m/z calc'd for $[C_{12}H_{14}DNO_2H]^+$ expect 265.1552; found 265.1556.
Chiral SFC Analysis: CHIRALPAK IG (CO_2:MeOH, 80:20, 2.5 mL min^{-1}, 40 °C, 228 nm) t_R = 5.8, 10.7 minutes, racemic.

Scheme 3B - Alcohol oxidation (kinetic resolution mode):

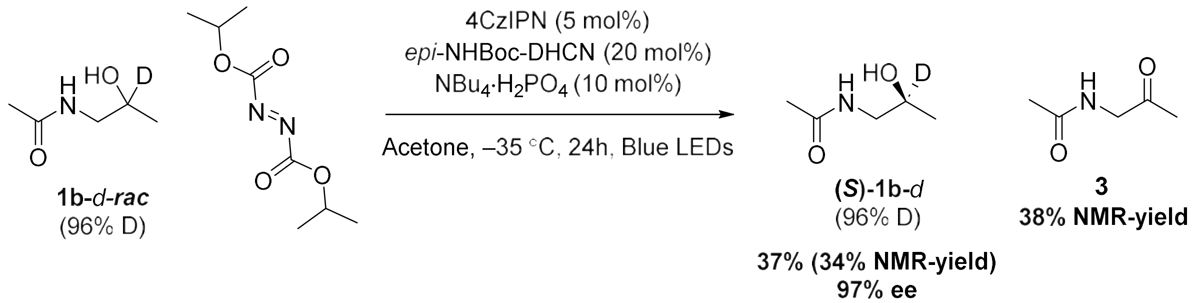

1b-*d*-*rac* (96% D) · **(*S*)-1b-*d*** (96% D), **37% (34% NMR-yield) 97% ee** · **3**, **38% NMR-yield**

N-(2-hydroxypropyl-2-d)acetamide (11.8 mg, 0.10 mmol, 1 eq), DIAD (20.2 mg, 0.10 mmol, 1 eq), *epi*-NHBoc-DHCN (7.9 mg, 0.020 mmol, 20 mol%), 4CzIPN (0.005 mmol, 5 mol%) and $TBAH_2PO_4$ (0.010 mmol, 10 mol%) were measured into a 4.0 mL, crimp-top vial. The vial was sealed, and 4 mL of acetone was added *via* syringe to the vial. The vial was sealed with electrical tape and was placed in the custom made photoreactor. The reaction vial was cooled to -35 °C (5-10 minutes) and the Kessil® A160WE Tuna blue lamp (100% intensity, 100% ocean blue colour) was switched on. Reaction time was 24h. The crude mixture was evaporated *in vacuo* and the NMR-yield was analysed by ^{1}H NMR which gave 38% NMR-yield for the oxidised product **3**. The material was subjected to purification by silica gel column chromatography (eluting with 10% MeOH in DCM) to afford the **(S)-1b-*d*** as transparent film

(4.4 mg, 0.037 mmol, 37% yield, 34% NMR-yield). Deuterium incorporation (96% D) was determined by ^{1}H NMR of the purified product.

The purified **(S)-1b-*d*** was further subjected to benzoylation conditions according to **General Procedure H** using **(*S*)-1b-*d*** (2.3 mg, 0.020 mmol), few crystals of DMAP, triethylamine (0.02 mL, 0.14 mmol) and benzoyl chloride (0.01 mL, 0.086 mmol) in DCM (0.4 mL). The crude benzoylation material was purified by silica gel column chromatography (elution with 1% MeOH in DCM followed by a second purification eluting with 20% acetone in hexane) which afforded **1b-Bz-*d*** (4.6 mg, 0.014 mmol, 72% yield, 97% ee). Deuterium incorporation (97% D) was determined by ^{1}H NMR of the purified product.

Data of for **(*S*)-1b-*d*** and **1b-Bz-*d*** are in accordance with the data reported above

Scheme 3D - Cyclopropane opening (kinetic resolution mode):

***N*-(2-cyclopropyl-2-hydroxyethyl)acetamide (2d-rac)**

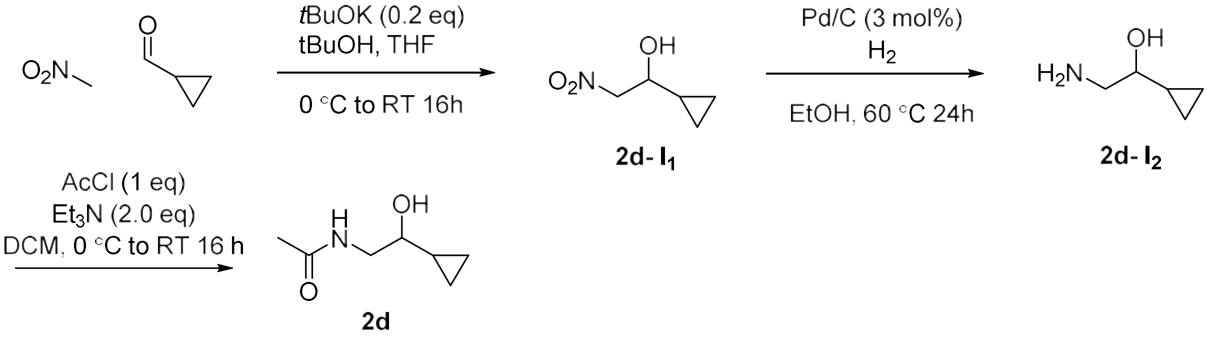

Cyclopropanecarbaldehyde (2.55 g, 36.4 mmol), nitromethane (2.43 mL, 45.5 mmol), THF (15 mL) and tBuOH (15 mL) were added into an oven-dried flask. The mixture was cooled to 0 °C and KOtBu (0.81 g, 7.28 mmol) was added into the stirred mixture. The reaction was allowed to warm up to ambient temperature and was stirred for 16 hours. The reaction was quenched by addition of aqueous saturated NH_4Cl was added and the mixture was extracted with DCM. The combined organic phases were dried over $MgSO_4$, filtered and evaporated *in vacuo*. The crude mixture containing 1-cyclopropyl-2-nitroethan-1-ol **2d-I₁** (3.7 g, 28.2 mmol, 78% yield) together with a minor impurity was used without further purification in the next step.

^{1}H NMR (700 MHz, $CDCl_3$) δ 4.57 – 4.50 (m, 2H), 3.69 – 3.64 (m, 1H), 2.19 (br. s, 1H), 0.99 – 0.91 (m, 1H), 0.68 – 0.57 (m, 2H), 0.50 – 0.44 (m, 1H), 0.38 – 0.31 (m, 1H) ppm.
^{13}C NMR (176 MHz, $CDCl_3$) δ 80.4, 73.1, 14.5, 3.2, 2.4 ppm.

The data is in good agreement with the literature.[27]

1-cyclopropyl-2-nitroethan-1-ol **2d-I₁** (1.0 g, 7.63 mmol) and EtOH (50 mL) were added into a round bottom flask and nitrogen was purged through the mixture for 15 minutes. Pd/C 10% w/w was added to the mixture (0.228 mmol), the flask was sealed and connected to Schlenk line, evacuated and refilled with nitrogen atmosphere two times followed by evacuation and filling with H_2 atmosphere twice. The sealed reaction mixture was equipped with hydrogen balloon, warmed to 60 °C and the mixture was vigorously stirred for 24 hours. The hydrogen balloon was removed, the flask was briefly flushed with flow of compressed air, and the mixture was filtered through small pad of celite. The celite layer was further eluted with methanol. Evaporation of the filtrate *in vacuo* provided the 2-amino-1-cyclopropylethan-1-ol **2d-I₂** (0.55

g, 4.19 mmol, 55% yield) with small impurities. The material was used in the next step without further purification.

^{1}H NMR (700 MHz, $CDCl_3$) δ 2.90 (dd, *J* = 12.8, 3.4 Hz, 1H), 2.84 (td, *J* = 8.1, 3.4 Hz, 1H), 2.71 (dd, *J* = 12.4, 8.1 Hz, 1H), 2.52 (br. s, 3H), 0.86 – 0.79 (m, 1H), 0.52 – 0.45 (m, 2H), 0.34 – 0.30 (m, 1H), 0.21 – 0.17 (m, 1H) ppm.
^{13}C NMR (176 MHz, $CDCl_3$) δ 76.9, 47.4, 15.1, 2.5, 2.0 ppm.

The data is in good agreement with the literature.[28]

Prepared according to **General Procedure D** using amino alcohol substrate 2-amino-1-cyclopropylethan-1-ol (200 mg, 1.98 mmol), triethylamine (0.55 mL, 3.95 mmol) and acetyl chloride (0.14 mL, 1.98 mmol). Purification by silica gel column chromatography (elution with 7% MeOH in DCM) afforded **2d-rac** as a colorless oil (127 mg, 0.89 mmol, 45% yield).

^{1}H NMR (700 MHz, $CDCl_3$) δ 6.15 (br. s, 1H), 3.61 (ddd, *J* = 13.9, 6.7, 3.1 Hz, 1H), 3.24 (ddd, *J* = 13.6, 8.2, 5.1 Hz, 1H), 3.01 (td, *J* = 8.2, 2.8 Hz, 1H), 2.85 (br. s., 1H), 2.00 (s, 3H), 1.90 (br. s., 1H) 0.92 – 0.86 (m, 1H), 0.56 – 0.5 (m, 2H), 0.37 – 0.33 (m, 1H), 0.28 – 0.24 (m, 1H) ppm.
^{13}C NMR (176 MHz, $CDCl_3$) δ 171.2, 75.9, 45.7, 23.3, 15.5, 2.8, 2.3 ppm.
HRMS $[M+H]^+$ m/z calc'd for $[C_7H_{13}NO_2H]^+$ expect 144.1020; found 144.1019.

(*S*)-N-(2-cyclopropyl-2-hydroxyethyl)acetamide ((S)-2d) and ***N*-(2-oxopentyl)acetamide (5)**

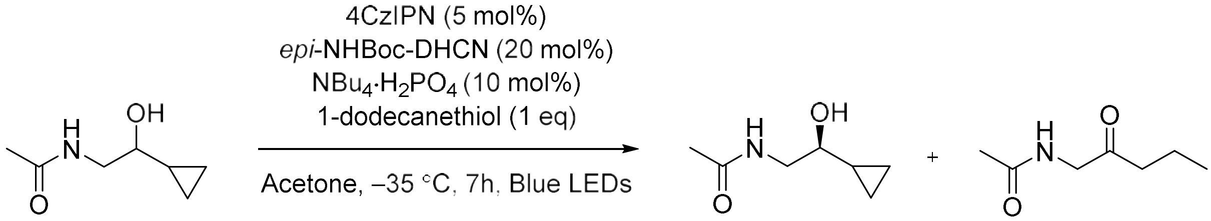

2d-rac **(*S*)-2d** **5**

49% (48%) yield 97% ee **48% (50%) yield**

(*S*)-2d was prepared according to **General Procedure G** using amino alcohol-derived substrate **2d-rac** (14.3 mg, 0.10 mmol) and *epi*-NHBoc-DHCN (7.9 mg, 0.020 mmol). Reaction time was 7h. After removal of the solvents *in vacuo* the crude material was analysed by ^{1}H NMR using internal standard (CH_2Br_2) to obtain NMR-yields for the reaction. Purification by silica gel column chromatography (elution with 5% MeOH in DCM) separated the title compounds. Fractions containing the *N*-(2-oxopentyl)acetamide were further purified (eluting with 20 to 30% acetone in hexane) to afford ***(5)*** as a colorless oil (6.9 mg, 0.048 mmol, 48% yield). The fractions containing **(*S*)-2d** were repurified by eluting with 2% to 5% MeOH in DCM to afford **(S)-2d** (7.0 mg, 0.049 mmol, 49% yield).

***N*-(2-oxopentyl)acetamide (5)**

^{1}H NMR (700 MHz, $CDCl_3$) δ 6.23 (br. s, 1H), 4.14 (d, *J* = 4.4 Hz, 2H), 2.42 (t, *J* = 7.4 Hz, 1H), 2.04 (s, 3H), 1.65 (sext., *J* = 1.66 Hz, 1H), 1.67 – 1.59 (br. s, 1H), 0.94 (t, *J* = 7.4 Hz, 3H) ppm.
^{13}C NMR (176 MHz, $CDCl_3$) δ 205.6, 170.2, 49.5, 42.4, 23.1, 17.4, 13.8 ppm.
HRMS $[M+H]^+$ m/z calc'd for $[C_7H_{13}NO_2H]^+$ expect 144.1017; found 144.1017.

(*S*)-N-(2-cyclopropyl-2-hydroxyethyl)acetamide ((*S*)-2d)

^{1}H NMR (700 MHz, $CDCl_3$) δ 6.06 (br. s, 1H), 3.60 (ddd, *J* = 13.9, 6.7, 3.1 Hz, 1H), 3.24 (ddd, *J* = 13.9, 8.0, 4.9 Hz, 1H), 3.00 (td, *J* = 8.2, 3.0 Hz, 1H), 2.69 (br. s, 1H), 2.00 (br. s, 1H), 0.89 (tdt, *J* = 8.2, 8.2, 4.9 Hz, 1H), 0.56 – 0.50 (m, 2H), 0.36 – 0.31 (m, 1H), 0.28 – 0.24 (m, 1H) ppm.
^{13}C NMR (176 MHz, $CDCl_3$) δ 171.2, 75.9, 45.7, 23.4, 15.5, 2.8, 2.3 ppm
HRMS $[M+H]^+$ m/z calc'd for $[C_7H_{13}NO_2H]^+$ expect 144.1020; found 144.1019.
$[\alpha]_D^{25.0}$ = +30.3 (*c* 0.55, $CHCl_3$).

(*S*)-2-(N-acetylbenzamido)-1-cyclopropylethyl benzoate (2d-Bz)

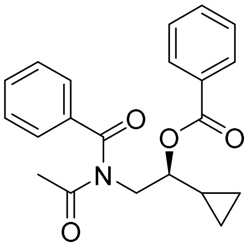

2d-Bz

2d-Bz was prepared according to **General Procedure H** using **(S)-2d** (3.5 mg, 0.024 mmol), few crystals of DMAP, triethylamine (0.02 mL, 0.14 mmol) and benzoyl chloride (0.01 mL, 0.086 mmol) in DCM (0.4 mL). Purification by silica gel column chromatography (elution with 1% MeOH in DCM followed by a second purification eluting with 20% acetone in hexane) afforded **2d-Bz** (1.5 mg, 0.004 mmol, 17% yield, 97% ee).

^{1}H NMR (700 MHz, $CDCl_3$) δ 7.93 - 7.90 (m, 2H), 7.53 – 7.48 (m, 4H), 7.40 – 7.35 (m, 2H), 4.85 (td, *J* = 8.9, 3.2 Hz, 1H), 4.30 (dd, *J* = 14.3, 8.9 Hz, 1H), 4.23 (dd, *J* = 14.3, 3.2 Hz, 1H), 2.06 (s, 3H), 1.04 – 0.98 (m, 1H), 0.64 – 0.59 (m, 1H), 0.58 – 0.50 (m, 2H), 0.48 – 0.43 (m, 1H) ppm.
^{13}C NMR (176 MHz, $CDCl_3$) δ 174.1, 173.4, 166.2, 135.3, 133.1, 132.7, 130.2, 129.8, 128.9, 128.9, 128.4, 77.4, 49.6, 26.3, 13.3, 3.7, 2.9 ppm
Chiral SFC Analysis: CHIRALPAK IK (CO_2:MeOH, 95:05, 2.5 mL min^{-1}, 40 °C, 227 nm) t_R = 6.35 (major), 7.49 (minor) minutes, 97% ee.

Scheme 3E - Time course study on enantiomeric inversion, commencing from (R)-enantiomer

(*R*)-N-(2-hydroxypropyl)acetamide ((*R*)-1b)

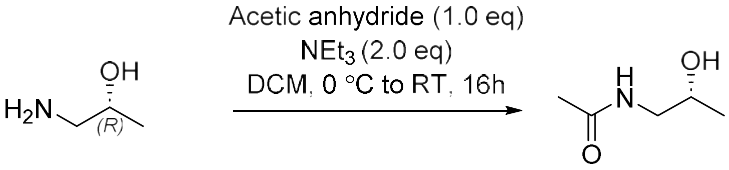

(*R*)-1b

Commercially available (*R*)-1-aminopropan-2-ol (375 mg, 5.0 mmol), triethylamine (1.39 mL, 10.0 mmol) and DCM (20 mL) were added to an oven-dried round bottom flask. The flask was cooled to 0 °C and acetic anhydride (0.472 mL, 5.0 mmol) was added dropwise to the mixture. The reaction was allowed to slowly warm up to ambient temperature and was

further stirred overnight. The mixture was concentrated *in vacuo* and purified twice by silica gel column chromatography (elution with 10% MeOH in DCM and 7% MeOH in DCM) which afforded **(*R*)-1b** as a colorless oil (78 mg, 0.67 mmol, 13% yield).

^{1}H NMR (700 MHz, $CDCl_3$) δ 6.17 (br. s, 1H), 3.94 – 3.86 (m, 1H), 3.42 (ddd, *J* = 14.0, 6.5, 3.0 Hz, 1H), 3.09 (ddd, *J* = 14.0, 7.7, 5.1 Hz, 1H), 2.50 (br. s, 1H), 2.01 (s, 3H), 1.18 (d, *J* = 6.3 Hz, 3H) ppm.
^{13}C NMR (176 MHz, $CDCl_3$) δ 171.5, 67.5, 47.3, 23.3, 21.1 ppm.
$[\alpha]_D^{25.0}$ = -35.9 (*c* 1.0, $CHCl_3$).

Data is in accordance with the data reported for **1b** above.

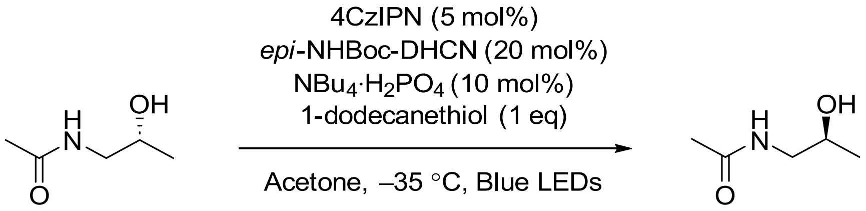

(*R*)-1b **(*S*)-1b**

Time course study was performed according to **General Procedure G** using enantiopure amino alcohol **(*R*)-1b** (11.7 mg, 0.10 mmol). Each data point represents an individual reaction. After the reaction, solvents were removed *in vacuo* and the crude mixture was benzoylated according to the **General Procedure H**. The benzoylated product was purified by preparatory thin layer chromatography (Eluting with 1% MeOH in DCM) to obtain analytical samples for SFC.

Chiral SFC Analysis: CHIRALPAK IK (CO_2:MeOH, 95:05, 2.5 mL min^{-1}, 40 °C, 228 nm) t_R = 6.1 and 6.5 minutes.

Time (h)	ee%
0	99 (*R*)-1b
0.25	77
0.5	60
1	36
3	-40
5	-77
8	-88
12	-94
22	-98

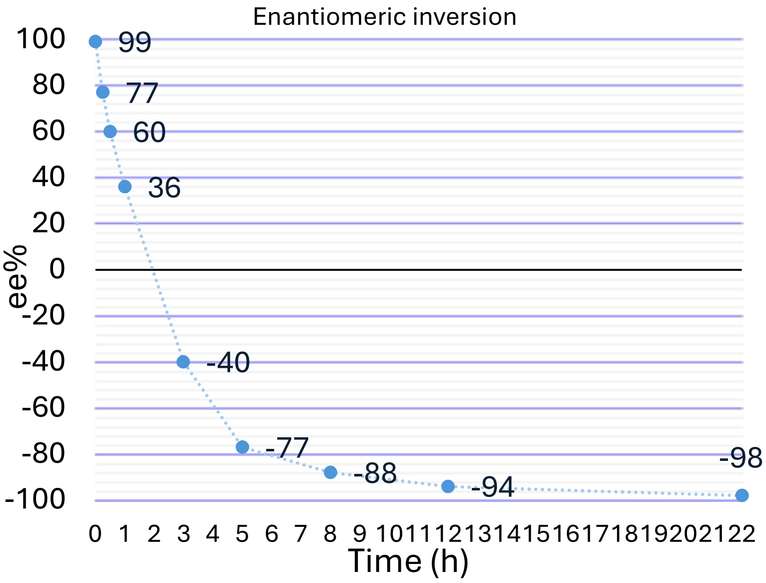

References

1. Lahdenpera, A. S. K.; Dhankhar, J.; Davies, D. J.; Lam, N. Y. S.; Bacos, P. D.; de la Vega-Hernandez, K.; Phipps, R. J., A chiral hydrogen atom abstraction catalyst for the enantioselective epimerization of meso-diols. *Science* **2024,** *386*, 42-49.
2. Lam, N. Y. S.; Dhankhar, J.; Lahdenpera, A. S. K.; Phipps, R. J., Catalytic Enantioselective Hydrogen Atom Abstraction Enables the Asymmetric Oxidation of Meso Diols. *J. Am. Chem. Soc.* **2024,** *146*, 33302-33308.
3. Luo, J.; Zhang, J., Donor–Acceptor Fluorophores for Visible-Light-Promoted Organic Synthesis: Photoredox/Ni Dual Catalytic C(sp3)–C(sp2) Cross-Coupling. *ACS Catal.* **2016,** *6*, 873-877.
4. Fulmer, G. R.; Miller, A. J. M.; Sherden, N. H.; Gottlieb, H. E.; Nudelman, A.; Stoltz, B. M.; Bercaw, J. E.; Goldberg, K. I., NMR Chemical Shifts of Trace Impurities: Common Laboratory Solvents, Organics, and Gases in Deuterated Solvents Relevant to the Organometallic Chemist. *Organometallics* **2010,** *29*, 2176-2179.
5. Du, Y. M.; Lin, J. N.; Li, Y. L.; Yu, Q.; Shu, W., Nickel-Catalyzed Adaptive Migration-Enabled Asymmetric Cross-Hydrocarbonylation of Unactivated Alkenes. *J. Am. Chem. Soc.* **2025,** *147*, 18944-18952.
6. Cotman, A. E.; Lozinsek, M.; Wang, B.; Stephan, M.; Mohar, B., trans-Diastereoselective Ru(II)-Catalyzed Asymmetric Transfer Hydrogenation of alpha-Acetamido Benzocyclic Ketones via Dynamic Kinetic Resolution. *Org. Lett.* **2019,** *21*, 3644-3648.
7. You, Q.; Collum, D. B., Carbon-Nitrogen Bond Formation Using Sodium Hexamethyldisilazide: Solvent-Dependent Reactivities and Mechanisms. *J. Am. Chem. Soc.* **2023,** *145*, 23568-23584.
8. Tu, W.; Farndon, J. J.; Robertson, C. M.; Bower, J. F., An Aza-Prilezhaev-Based Method for Inversion of Regioselectivity in Stereospecific Alkene 1,2-Aminohydroxylations. *Angew. Chem.* **2024,** *63*, e202409836.
9. Ojima, I.; Zhao, M.; Yamato, T.; Nakahashi, K.; Yamashita, M.; Abe, R., Azetidines and bisazetidines. Their synthesis and use as the key intermediates to enantiomerically pure diamines, amino alcohols, and polyamines. *J. Org. Chem.* **2002,** *56*, 5263-5277.
10. Yao, Y. X.; Zhang, H. W.; Lu, C. B.; Shang, H. Y.; Tian, Y. Y., Highly Selective and Practical Iron-Catalyzed Formal Hydrogenation of Epoxides to Primary Alcohols Using Formic Acid. *Eur. J. Org. Chem.* **2023,** *26*.
11. Meng, Q. Y.; Schirmer, T. E.; Katou, K.; Konig, B., Controllable Isomerization of Alkenes by Dual Visible-Light-Cobalt Catalysis. *Angew. Chem.* **2019,** *58*, 5723-5728.
12. Adamczyk, M.; Johnson, D. D.; Reddy, R. E., Collagen cross-links: Synthesis of pyridinoline, deoxypyridinoline and their analogues. *Tetrahedron* **1999,** *55*, 63-88.
13. Radha Krishna, P.; Srinivas, R., First stereoselective total synthesis of (6R)-6-[(4R,6R)-4,6-dihydroxy-10-phenyldec-1-enyl]-5,6-dihydro-2H-pyran-2-one. *Tetrahedron Lett.* **2007,** *48*, 2013-2015.

14. Pavlyuk, O.; Teller, H.; McMills, M. C., An efficient synthesis of nitrogen-containing heterocycles via a tandem carbenoid N–H insertion/ring-closing metathesis sequence. *Tetrahedron Lett.* **2009,** *50*, 2716-2718.
15. Neufeld, K.; Henssen, B.; Pietruszka, J., Enantioselective allylic hydroxylation of omega-alkenoic acids and esters by P450 BM3 monooxygenase. *Angew. Chem.* **2014,** *53*, 13253-7.
16. Stang, E. M.; White, M. C., Molecular complexity via C-H activation: a dehydrogenative Diels-Alder reaction. *J. Am. Chem. Soc.* **2011,** *133*, 14892-5.
17. Xie, C.; Nowak, P.; Kishi, Y., Synthesis of the C20-C26 building block of halichondrins via a regiospecific and stereoselective S(N)2' reaction. *Org. Lett.* **2002,** *4*, 4427-9.
18. Xu, Y.; Chen, S.; Cao, Y.; Zhou, P.; Chen, Z.; Cheng, K., Discovery of novel small molecule TLR4 inhibitors as potent anti-inflammatory agents. *Eur. J. Med. Chem.* **2018,** *154*, 253-266.
19. Kon, Y.; Nakashima, T.; Makino, Y.; Nagashima, H.; Onozawa, S. y.; Kobayashi, S.; Sato, K., Continuous Synthesis of Epoxides from Alkenes by Hydrogen Peroxide with Titanium Silicalite-1 Catalyst Using Flow Reactors. *Adv. Synth. Catal.* **2023,** *365*, 3227-3233.
20. Damalanka, V. C.; Kim, Y.; Galasiti Kankanamalage, A. C.; Rathnayake, A. D.; Mehzabeen, N.; Battaile, K. P.; Lovell, S.; Nguyen, H. N.; Lushington, G. H.; Chang, K. O.; Groutas, W. C., Structure-guided design, synthesis and evaluation of oxazolidinone-based inhibitors of norovirus 3CL protease. *Eur. J. Med. Chem.* **2018,** *143*, 881-890.
21. Hothker, S.; Goli, H.; Klare, S.; Krebs, T.; Schacht, J. H.; Gansauer, A., Attenuating Nucleophilicity of Titanocene Hydrides Beyond Steric Effects en Route to Fatty Alcohols. *Chem. Eur. J.* **2024,** *30*, e202402694.
22. Rudolph, A.; Rackelmann, N.; Turcotte-Savard, M. O.; Lautens, M., Application of secondary alkyl halides to a domino aryl alkylation reaction for the synthesis of aromatic heterocycles. *J. Org. Chem.* **2009,** *74*, 289-97.
23. Lim, J. J.; Leitch, D. C., Lewis Acid-Catalyzed Addition of Benzophenone Imine to Epoxides Enables the Selective Synthesis and Derivatization of Primary 1,2-Amino Alcohols. *Org. Process Res. Dev.* **2018,** *22*, 641-649.
24. Enders, D.; Haertwig, A.; Raabe, G.; Runsink, J., Diastereo- and Enantioselective Synthesis of Vicinal Amino Alcohols by Oxa Michael Addition ofN-Formylnorephedrine to Nitro Alkenes. *Eur. J. Org. Chem.* **1998,** 1771-1792.
25. Gotor, V.; Brieva, R.; Rebolledo, F., Enantioselective acylation of amino alcohols by porcine pancreatic lipase. *Chem. Commun.* **1988,** 957-958.
26. Abadji, V.; Lin, S.; Taha, G.; Griffin, G.; Stevenson, L.; Pertwee, R.; Makriyannis, A., (*R*)-Methanandamide: A Chiral Novel Anandamide Possessing Higher Potency and Metabolic Stability. *J. Med. Chem.* **1994**, *37*, 1889-1893.
27. Sharma, S.; Kumar, M.; Bhatt, V.; Nayal, O. S.; Thakur, M. S.; Kumar, N.; Singh, B.; Sharma, U. Vasicine from *Adhatoda vasica* as an organocatalyst for metal-free Henry

reaction and reductive heterocyclization of *o*-nitroacylbenzenes. *Tetrahedron Lett.* 2016, *57*, 5003-5008.
28. Zhang, R.; McIntyre, P. J.; Collins, P. M.; Foley, D. J.; Arter, C.; von Delft, F.; Bayliss, R.; Warriner, S.; Nelson, A. Discovery of inhibitors of the mitotic kinesin Eg5 using fragment-based screening. *Chem. Eur. J.* 2019, *25* (27), 6831-6839.

(*S*)-1-(*N*-acetylbenzamido)butan-2-yl benzoate (1a-Bz), (*R*)-1-(*N*-acetylbenzamido)butan-2-yl benzoate ((*R*)-1a-Bz)

Chiral SFC Analysis: CHIRALPAK IK (CO_2:MeOH, 95:05, 2.5 mL min^{-1}, 40 °C, 228 nm) t_R = 5.4 (major), 6.2 (minor) minutes, 97% ee; t_R = 5.4 (minor), 6.2 (major) minutes, −88% ee

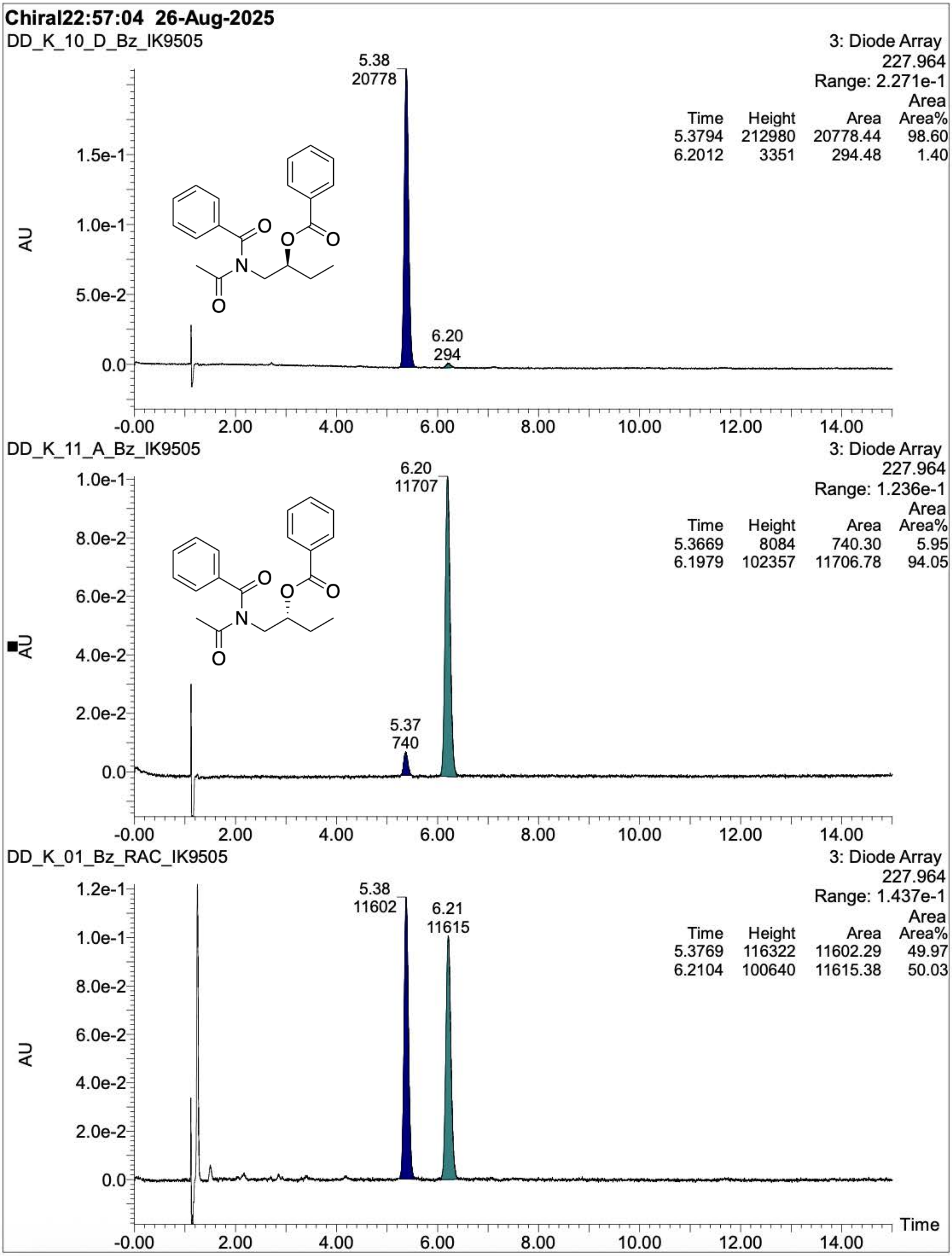

(*S*)-*N*-(2-hydroxybutyl)-*N*-methylacetamide (*N*-Me-1a)

Chiral GC Analysis: CP-Chirasil-Dex CB Column 25 m × 0.25 mm × 0.25 µm, flow: 2.17 mL/min, 110 °C for 50 min; 110 °C to 200 °C at 11 °C/min: t_R = 27.3 (major), 30.4 (minor) minutes, 19% ee

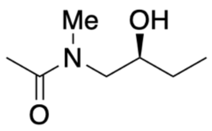

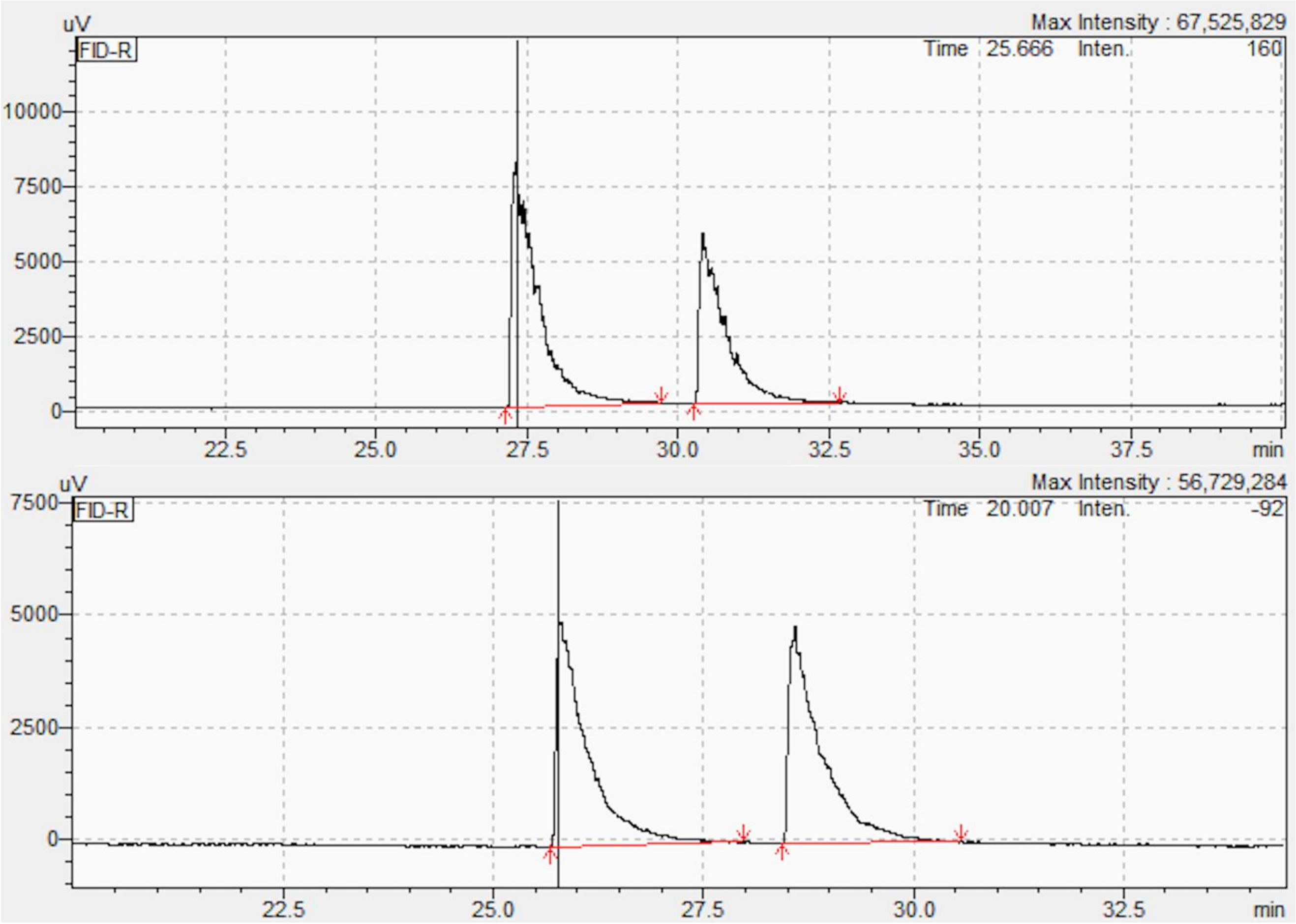

Peak#	Ret. Time	Area	Conc.	Area%
1	27.340	239329	59.455	59.455
2	30.412	163208	40.545	40.545
Total		402537	100.000	100.000

Peak#	Ret. Time	Area	Conc.	Area%
1	25.784	127181	50.043	50.043
2	28.588	126964	49.957	49.957
Total		254144	100.000	100.000

(*S*)-1-(*N*-acetylbenzamido)propan-2-yl benzoate (1b-Bz)

Chiral SFC Analysis: CHIRALPAK IK (CO_2:MeOH, 95:05, 2.5 mL min^{-1}, 40 °C, 228 nm) t_R = 6.1 (major), 6.5 (minor) minutes, 97% ee

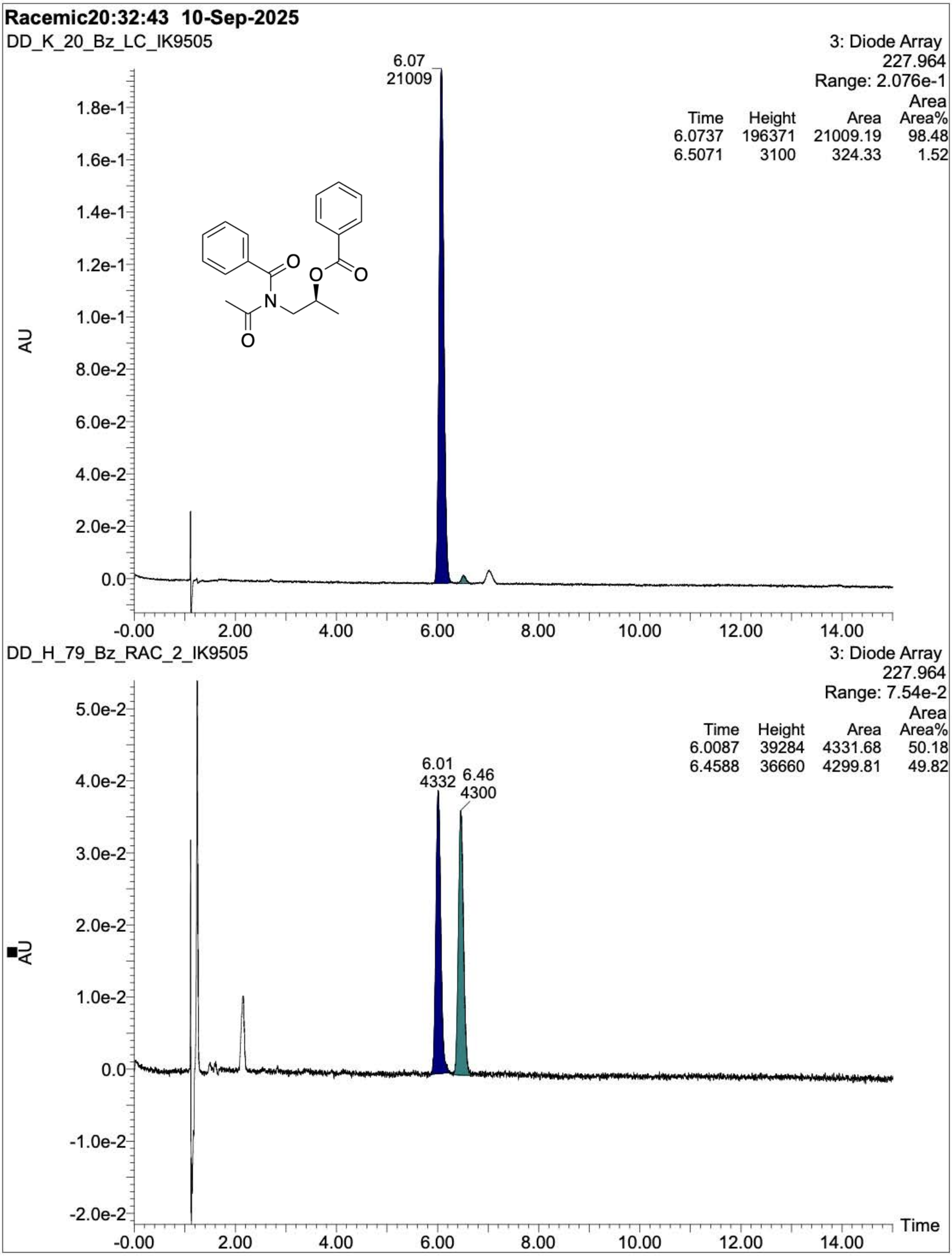

(*S*)-1-(*N*-acetylbenzamido)octan-2-yl benzoate (1c-Bz)

Chiral SFC Analysis: CHIRALPAK IK (CO_2:MeOH, 96:04, 2.5 mL min^{-1}, 40 °C, 228 nm) t_R = 6.3 (major), 8.2 (minor) minutes, 98% ee

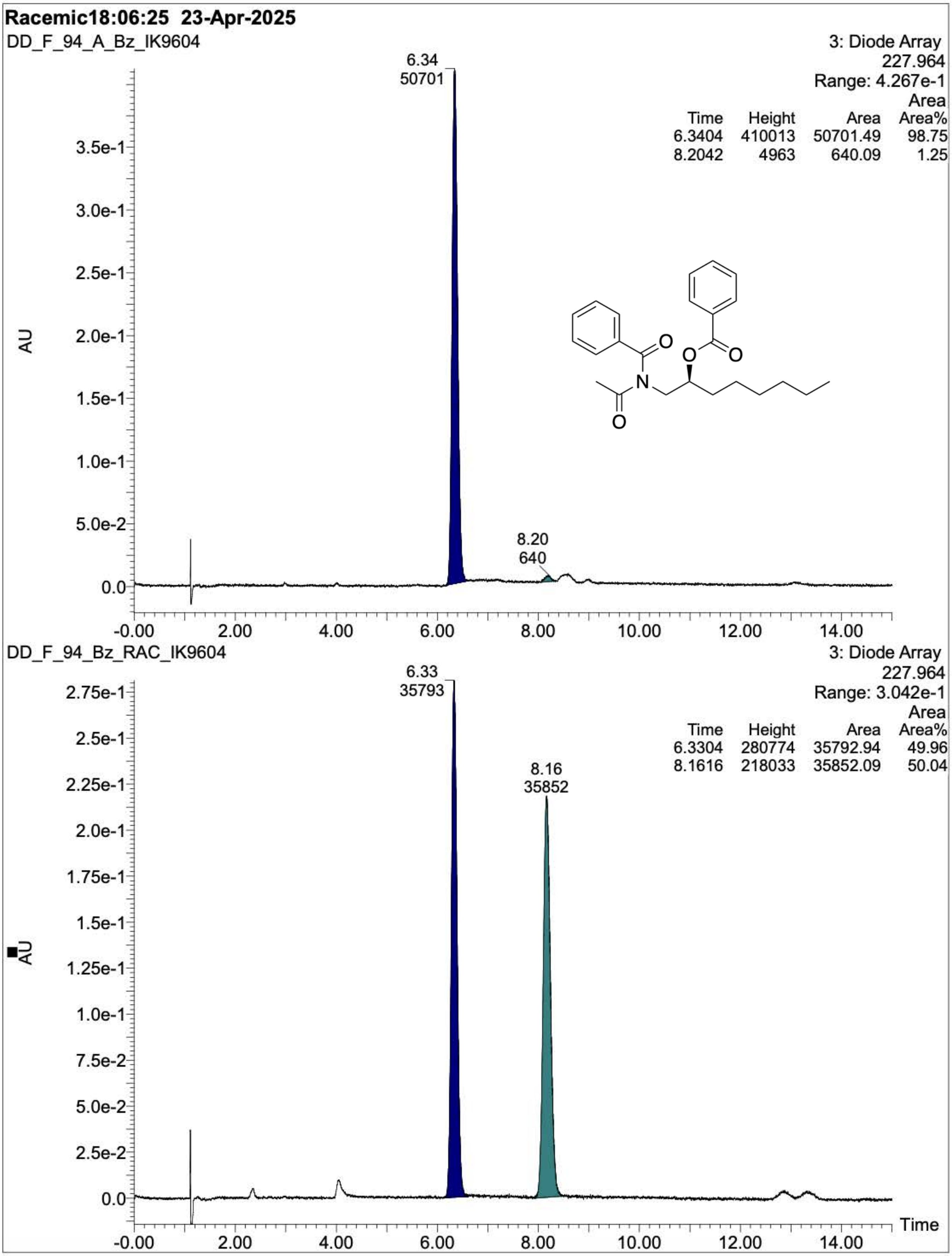

(*S*)-1-(*N*-acetylbenzamido)-7-methyloctan-2-yl benzoate (1d-Bz)

Chiral SFC Analysis: CHIRALPAK IK (CO_2:MeOH, 96:04, 2.5 mL min^{-1}, 40 °C, 228 nm) t_R = 6.1 (major), 7.9 (minor) minutes, 97% ee

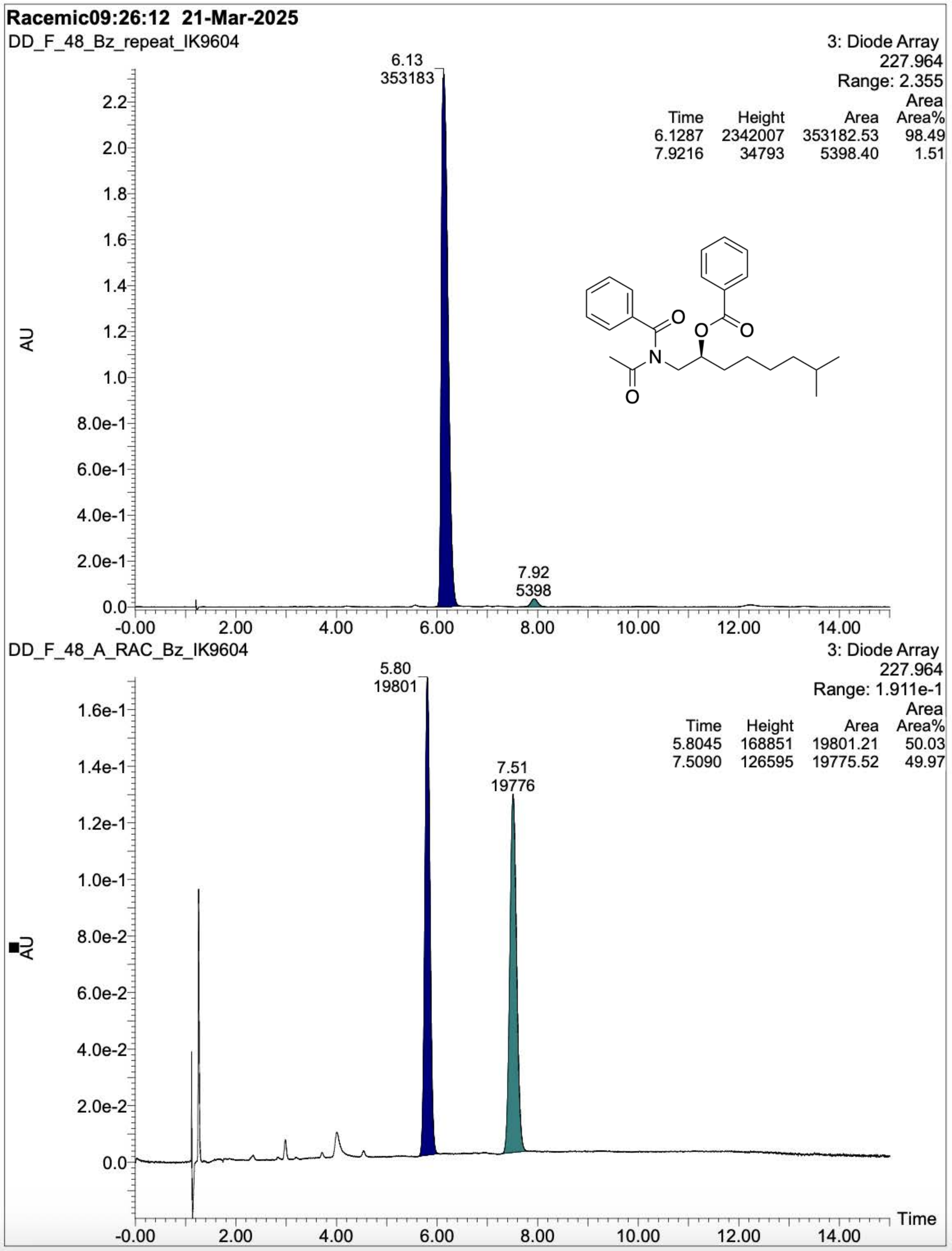

(*S*)-1-(*N*-acetylbenzamido)-4-cyclobutylbutan-2-yl benzoate (1e-Bz)

Chiral SFC Analysis: CHIRALPAK IK (CO_2:MeOH, 95:05, 2.5 mL min^{-1}, 40 °C, 228 nm) t_R = 6.7 (major), 8.6 (minor) minutes, 97% ee

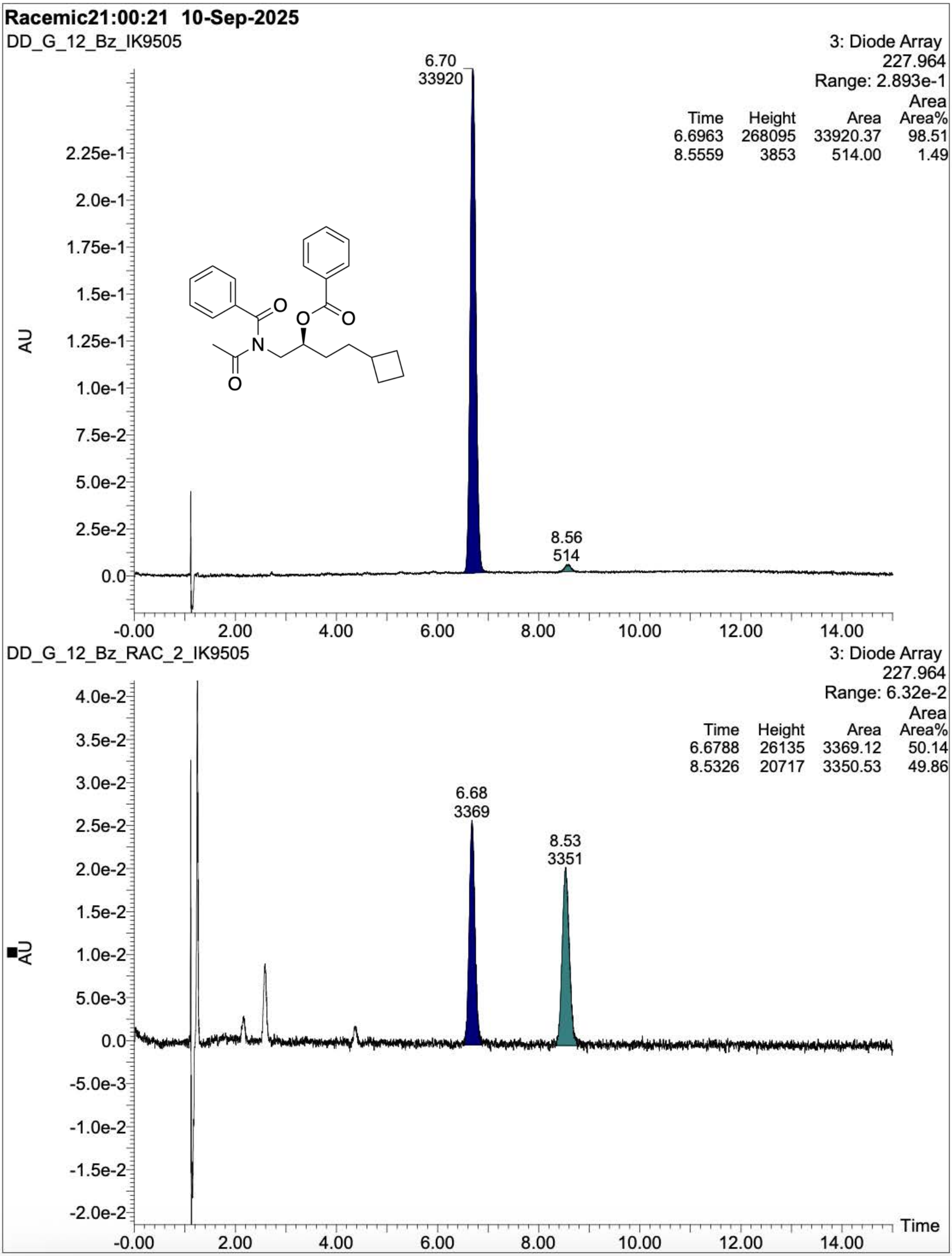

(*S*)-1-(*N*-acetylbenzamido)-5-cyclohexylpentan-2-yl benzoate (1f-Bz), (*R*)-1-(*N*-acetylbenzamido)-5-cyclohexylpentan-2-yl benzoate ((*R*)-1f-Bz)

Chiral SFC Analysis: CHIRALPAK IK (CO_2:MeOH, 95:05, 2.5 mL min^{-1}, 40 °C, 228 nm) t_R = 8.5 (major), 11.1 (minor) minutes, 99% ee; t_R = 8.7 (minor), 11.3 (major) minutes, −94% ee

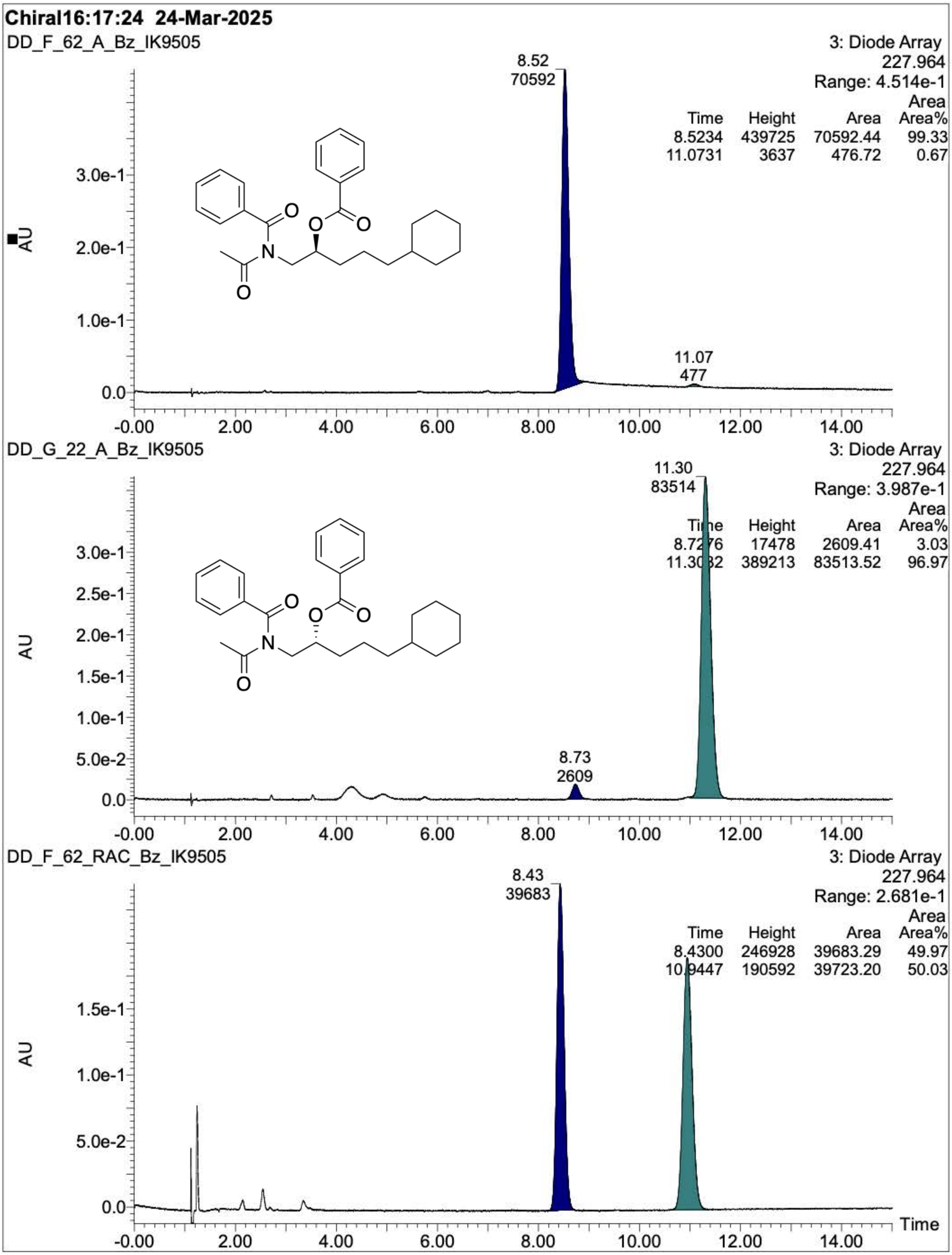

(*S*)-*N*-(2-hydroxy-3-phenylpropyl)acetamide (1g)

Chiral SFC Analysis: CHIRALPAK IJ (CO_2:MeOH, 95:05, 2.5 mL min^{-1}, 40 °C, 203 nm) t_R = 5.7 (minor), 7.3 (major) minutes, 97% ee

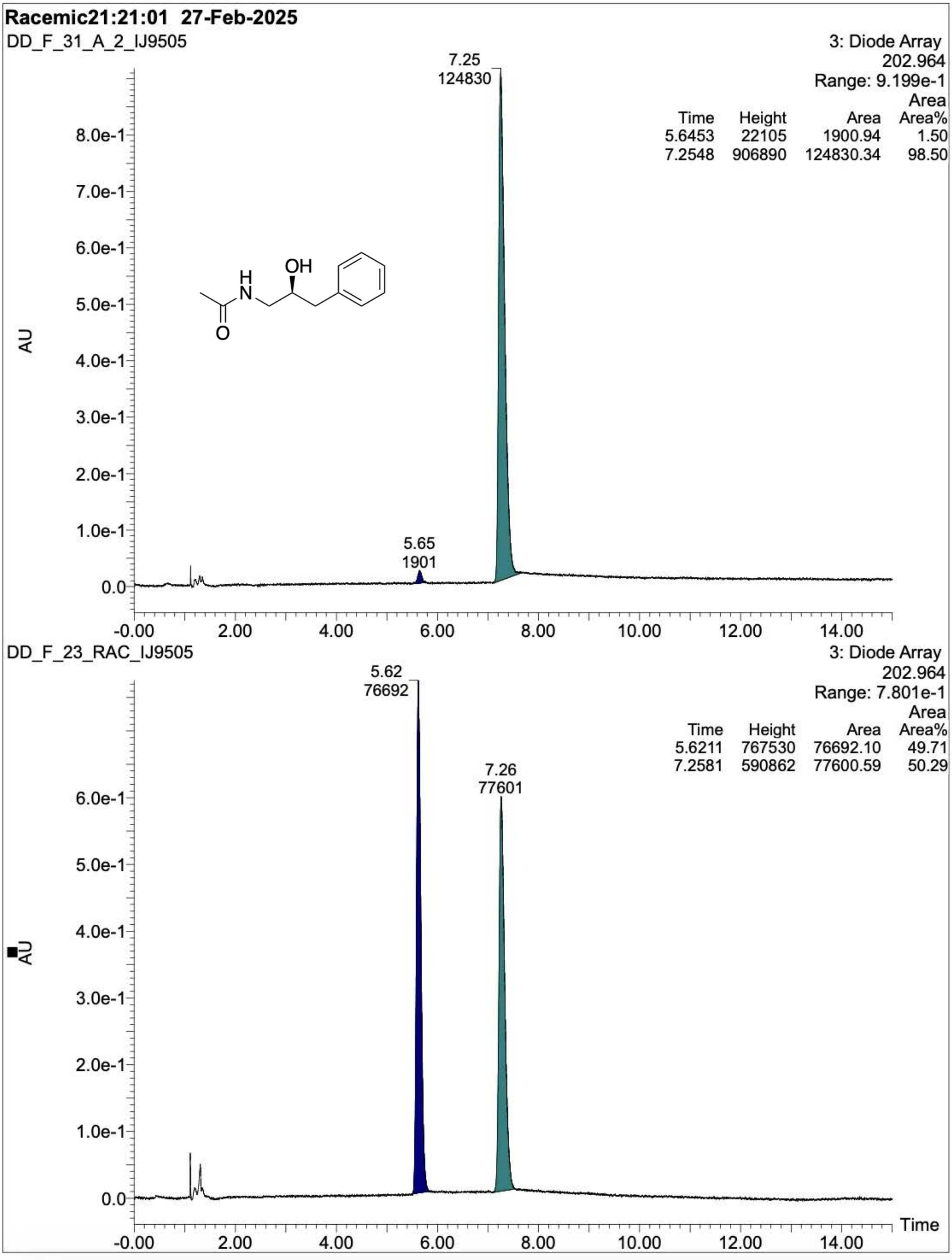

(*S*)-*N*-(2-hydroxy-4-phenylbutyl)acetamide (1h)

Chiral SFC Analysis: CHIRALPAK IK (CO_2:MeOH, 93:07, 2.5 mL min^{-1}, 40 °C, 203 nm) t_R = 11.5 (major), 12.8 (minor) minutes, 91% ee

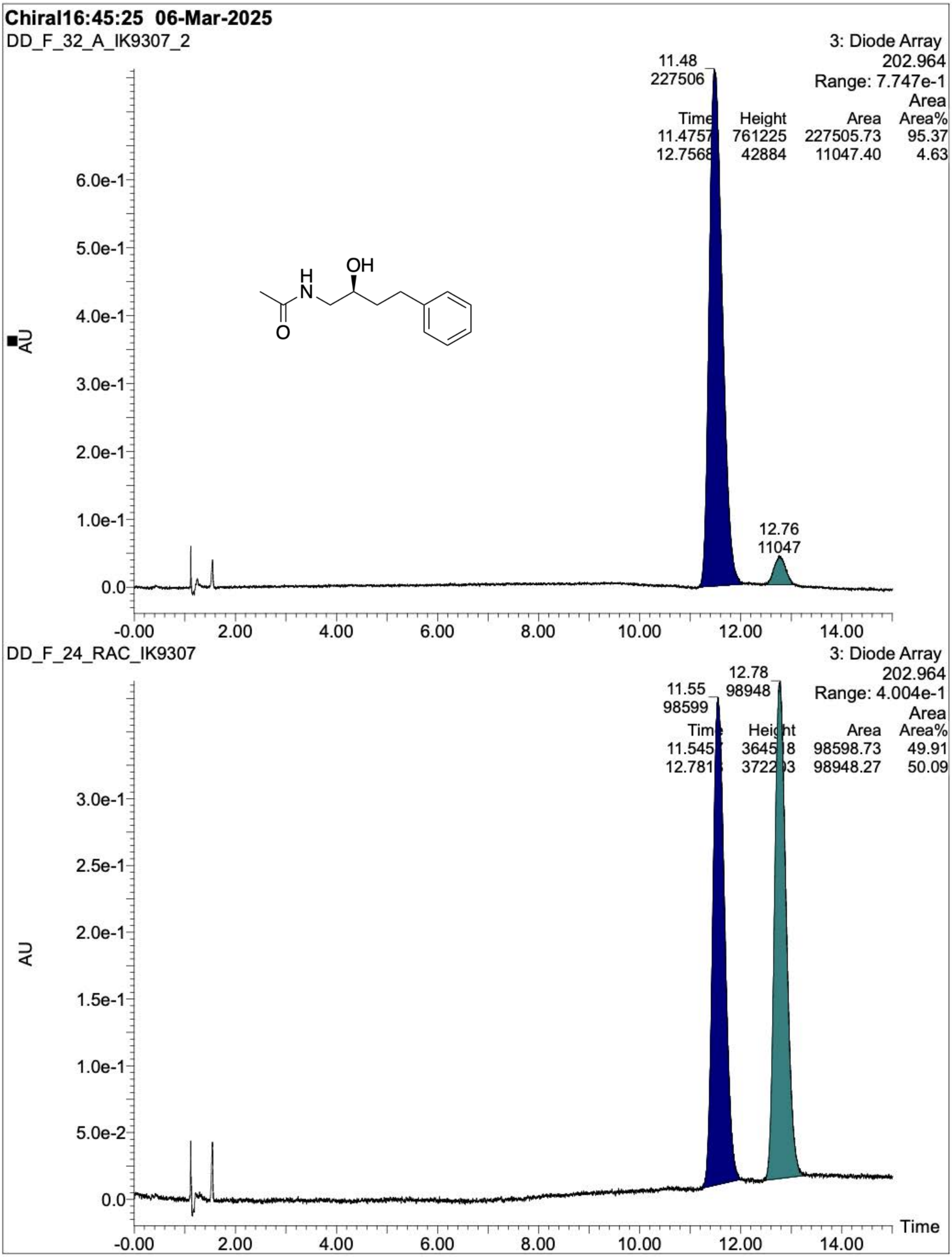

(*S*)-*N*-(2-hydroxy-5-phenylpentyl)acetamide (1i)

Chiral SFC Analysis: CHIRALPAK IJ (CO_2:MeOH, 95:05, 2.5 mL min^{-1}, 40 °C, 204 nm) t_R = 7.9 (minor), 9.2 (major) minutes, 95% ee

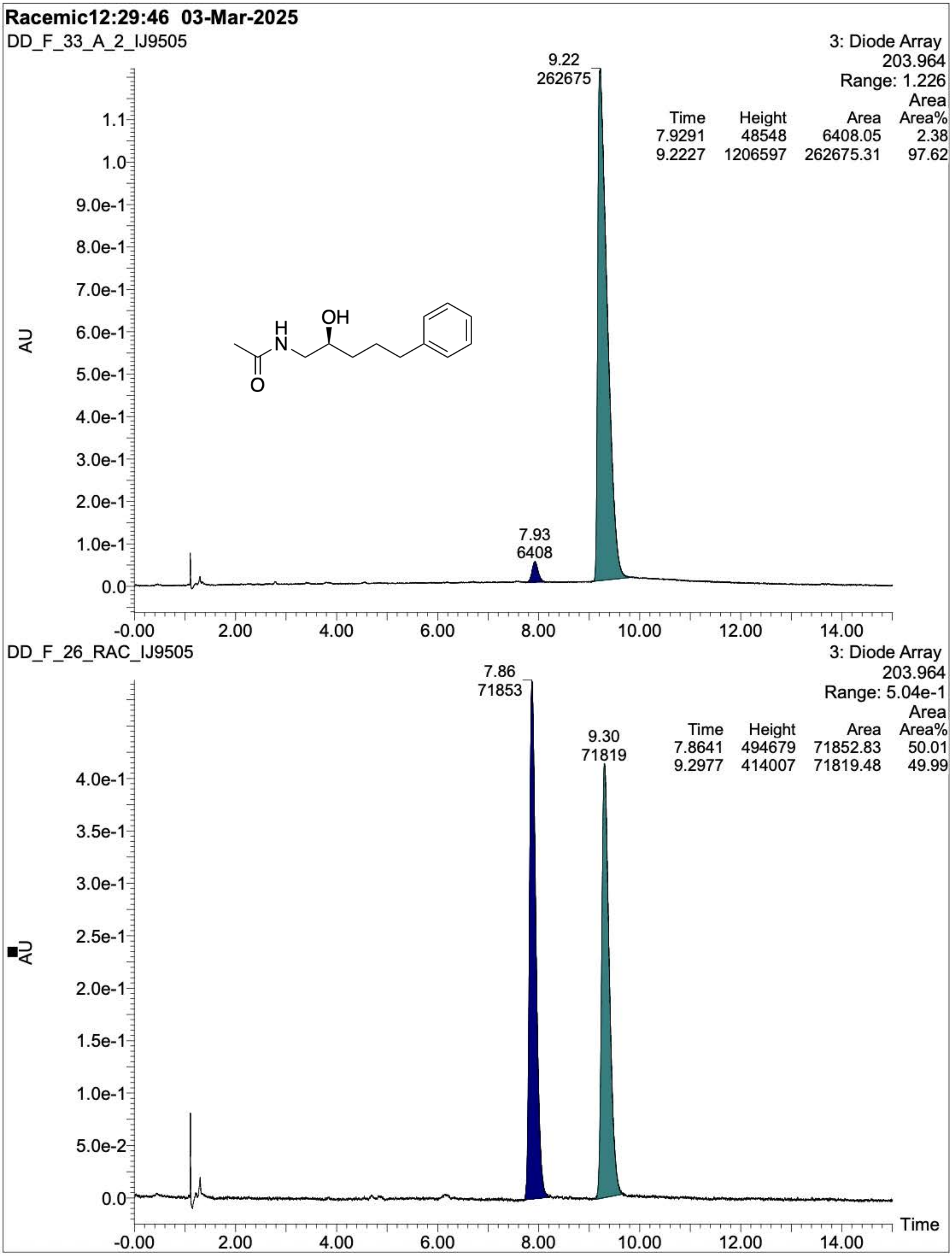

(*S*)-*N*-(2-hydroxy-6-phenylhexyl)acetamide (1j)

Chiral SFC Analysis: CHIRALPAK IK (CO_2:MeOH, 90:10, 2.5 mL min^{-1}, 40 °C, 203 nm) t_R = 7.5 (major), 8.2 (minor) minutes, 95% ee

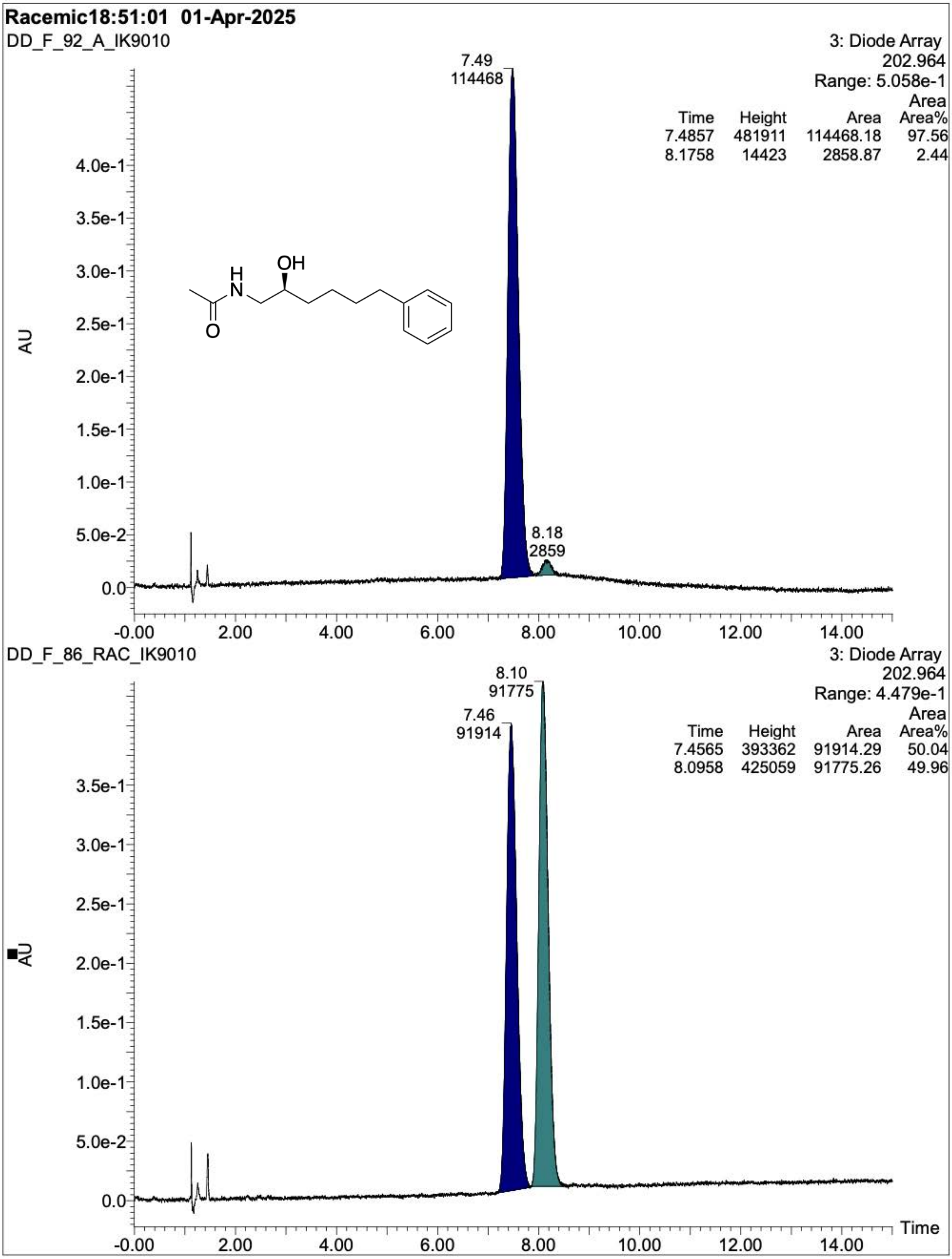

Time	Height	Area	Area%
7.4857	481911	114468.18	97.56
8.1758	14423	2858.87	2.44

Time	Height	Area	Area%
7.4565	393362	91914.29	50.04
8.0958	425059	91775.26	49.96

(*S*)-*N*-(2-hydroxy-3-phenylpropyl)acetamide (1k)

Chiral SFC Analysis: CHIRALPAK IK (CO_2:MeOH, 90:10, 2.5 mL min^{-1}, 40 °C, 214 nm) t_R = 8.0 (major), 8.8 (minor) minutes, 95% ee

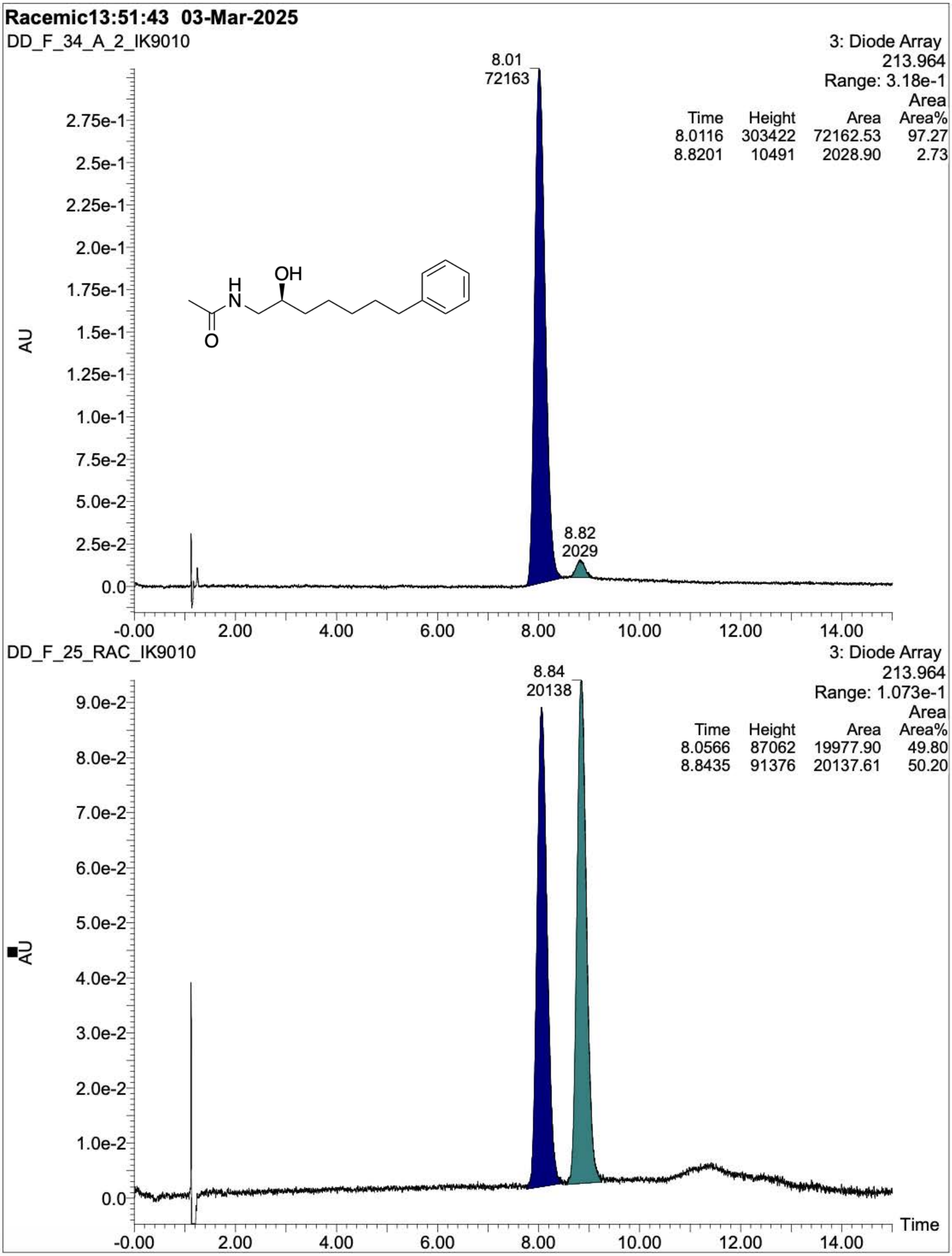

Time	Height	Area	Area%
8.0116	303422	72162.53	97.27
8.8201	10491	2028.90	2.73

Time	Height	Area	Area%
8.0566	87062	19977.90	49.80
8.8435	91376	20137.61	50.20

(*S*)-1-(*N*-acetylbenzamido)-5-((*tert*-butoxycarbonyl)amino)pentan-2-yl benzoate (1l-Bz)

Chiral SFC Analysis: CHIRALPAK IK (CO_2:MeOH, 90:10, 2.5 mL min^{-1}, 40 °C, 229 nm) t_R = 8.3 (major), 10.1 (minor) minutes, 94% ee

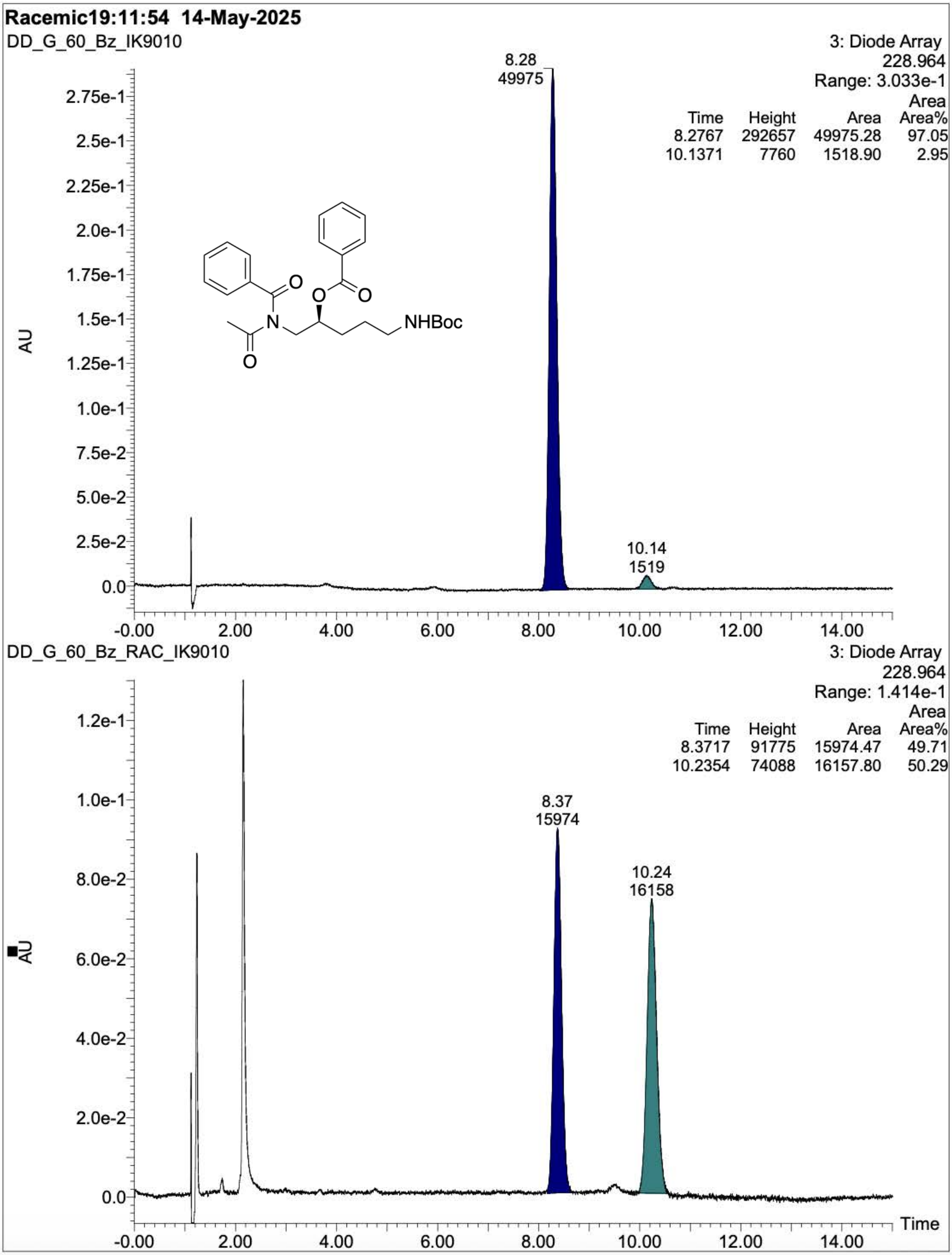

(*S*)-1-(*N*-acetylbenzamido)-7-ethoxy-7-oxoheptan-2-yl benzoate (1m-Bz)

Chiral SFC Analysis: CHIRALPAK IK (CO_2:MeOH, 93:07, 2.5 mL min^{-1}, 40 °C, 228 nm) t_R = 7.7 (major), 9.3 (minor) minutes, 95% ee

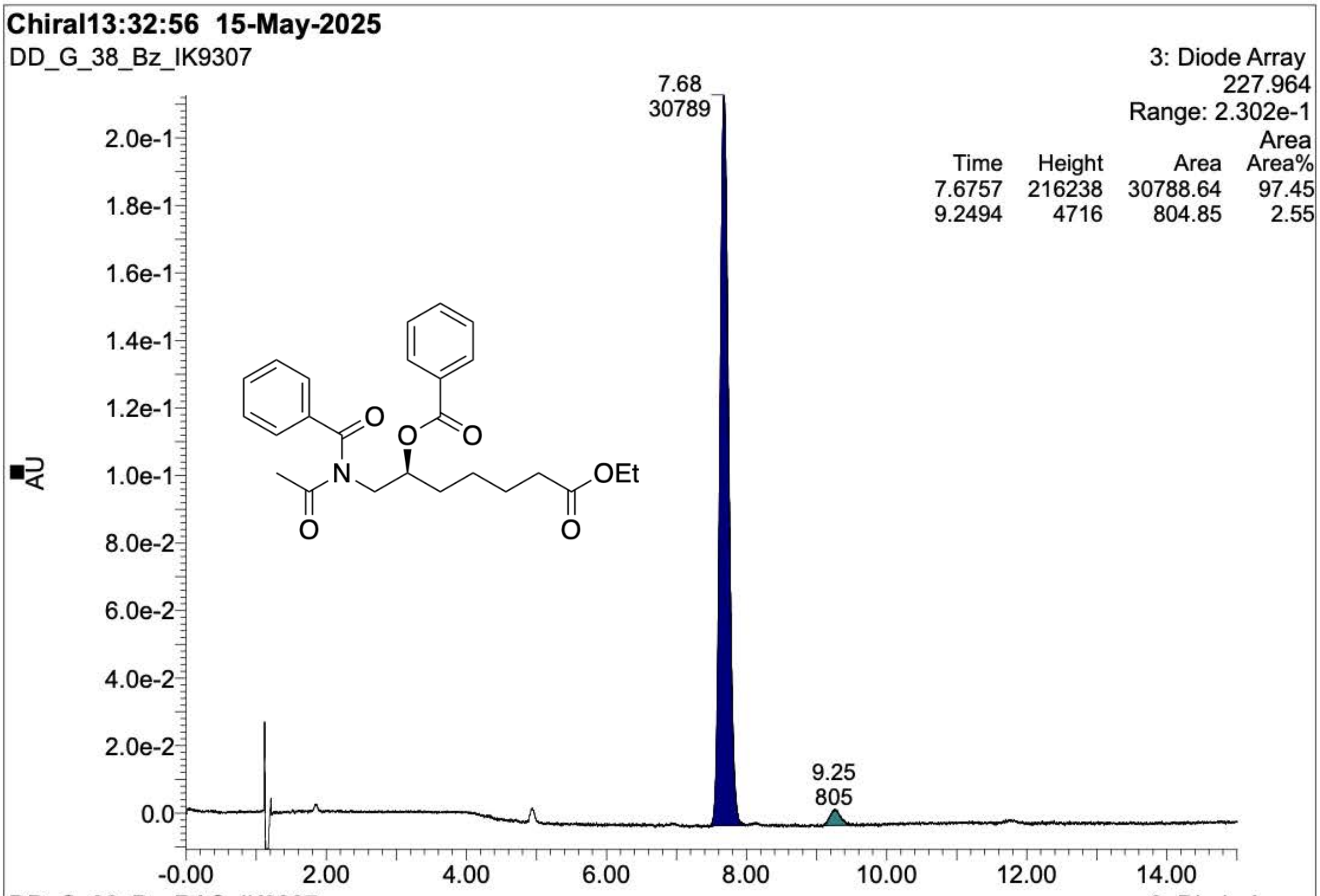

Time	Height	Area	Area Area%
7.6757	216238	30788.64	97.45
9.2494	4716	804.85	2.55

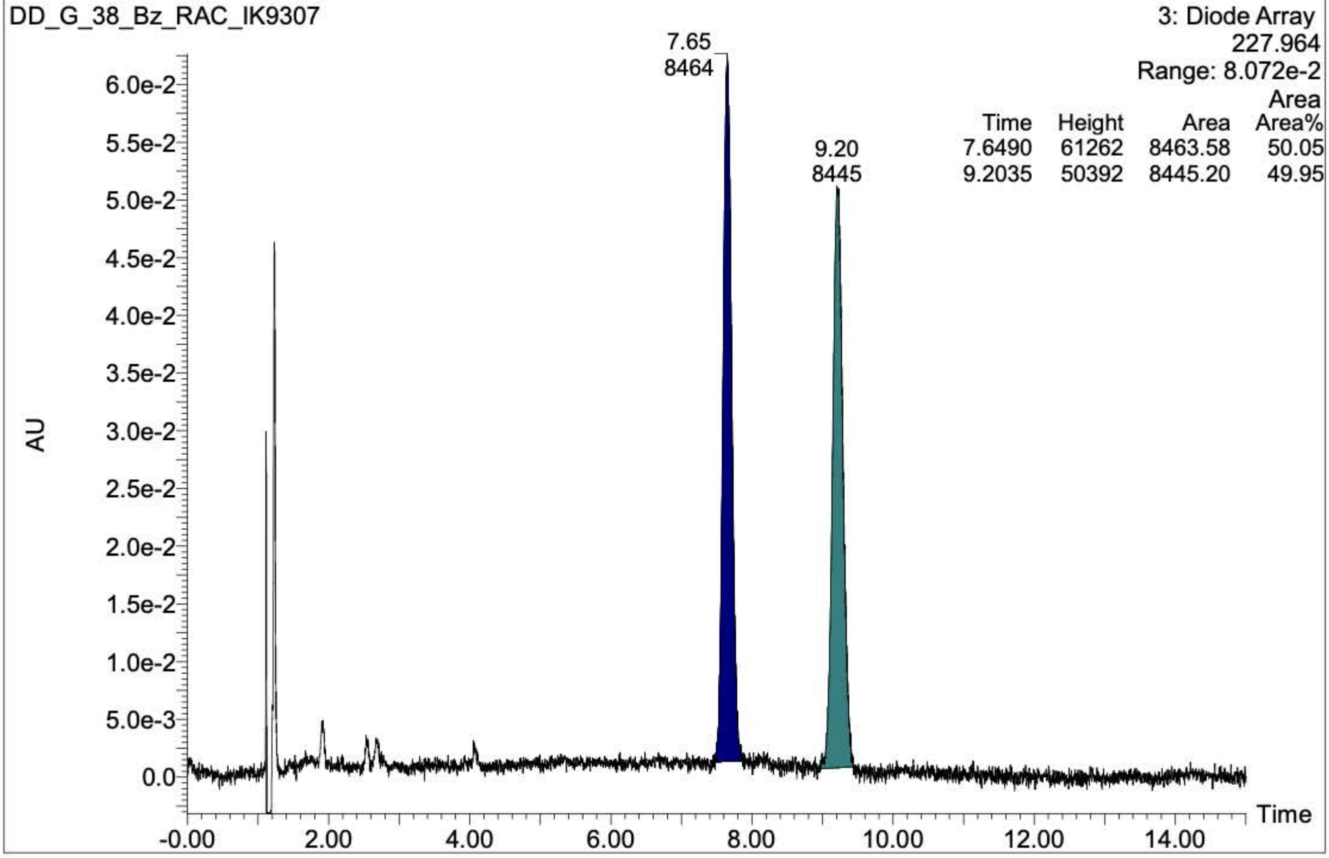

Time	Height	Area	Area Area%
7.6490	61262	8463.58	50.05
9.2035	50392	8445.20	49.95

(*S*)-1-(*N*-acetylbenzamido)-5-((*tert*-butyldimethylsilyl)oxy)pentan-2-yl benzoate (1n-Bz)

Chiral SFC Analysis: CHIRALPAK IK (CO_2:MeOH, 97:03, 2.5 mL min^{-1}, 40 °C, 228 nm) t_R = 6.1 (major), 7.9 (minor) minutes, 95% ee

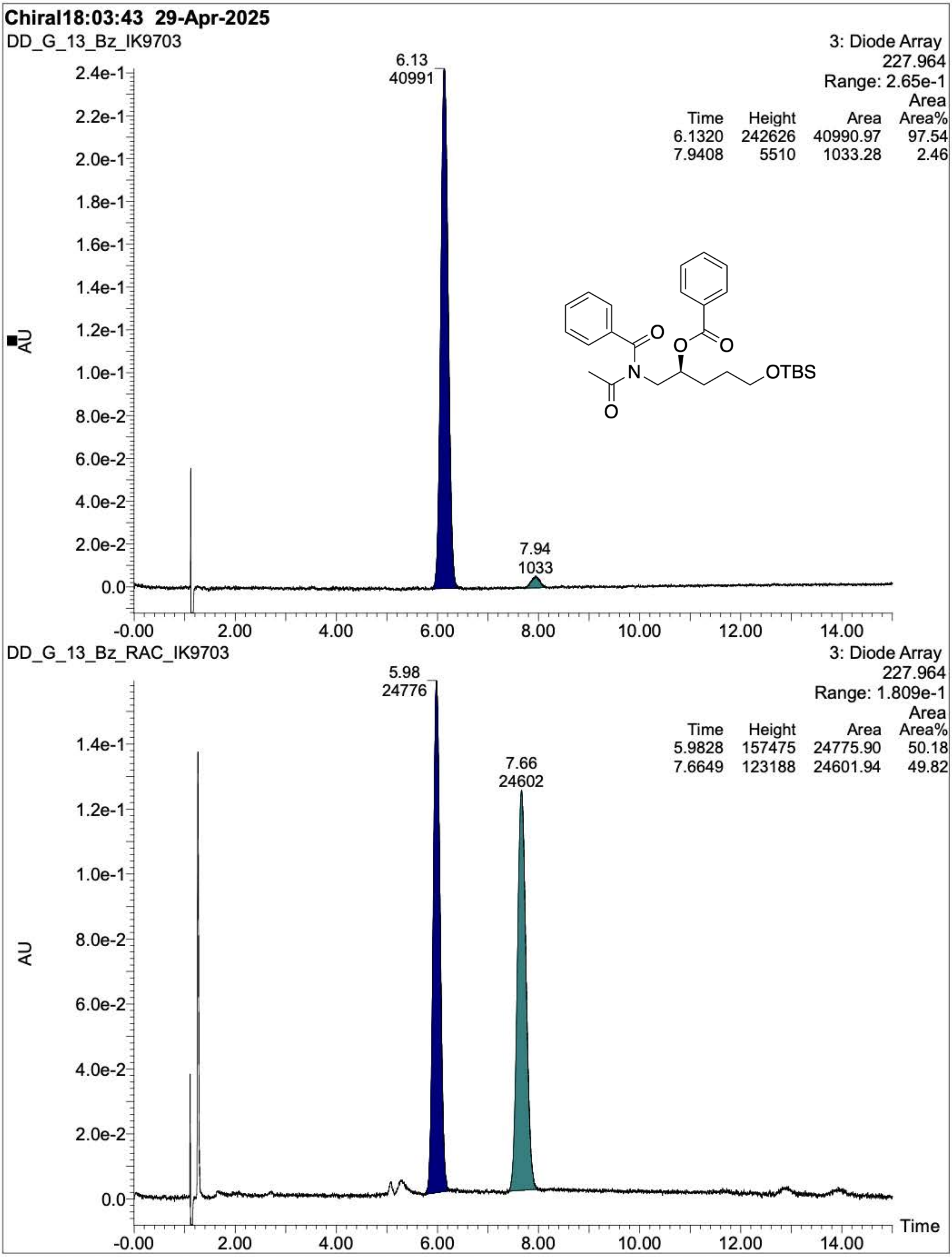

(*S*)-1-(*N*-acetylbenzamido)-5-ethoxypentan-2-yl benzoate (1o-Bz)

Chiral SFC Analysis: CHIRALPAK IK (CO_2:MeOH, 95:05, 2.5 mL min^{-1}, 40 °C, 228 nm) t_R = 7.5 (major), 8.9 (minor) minutes, 93% ee

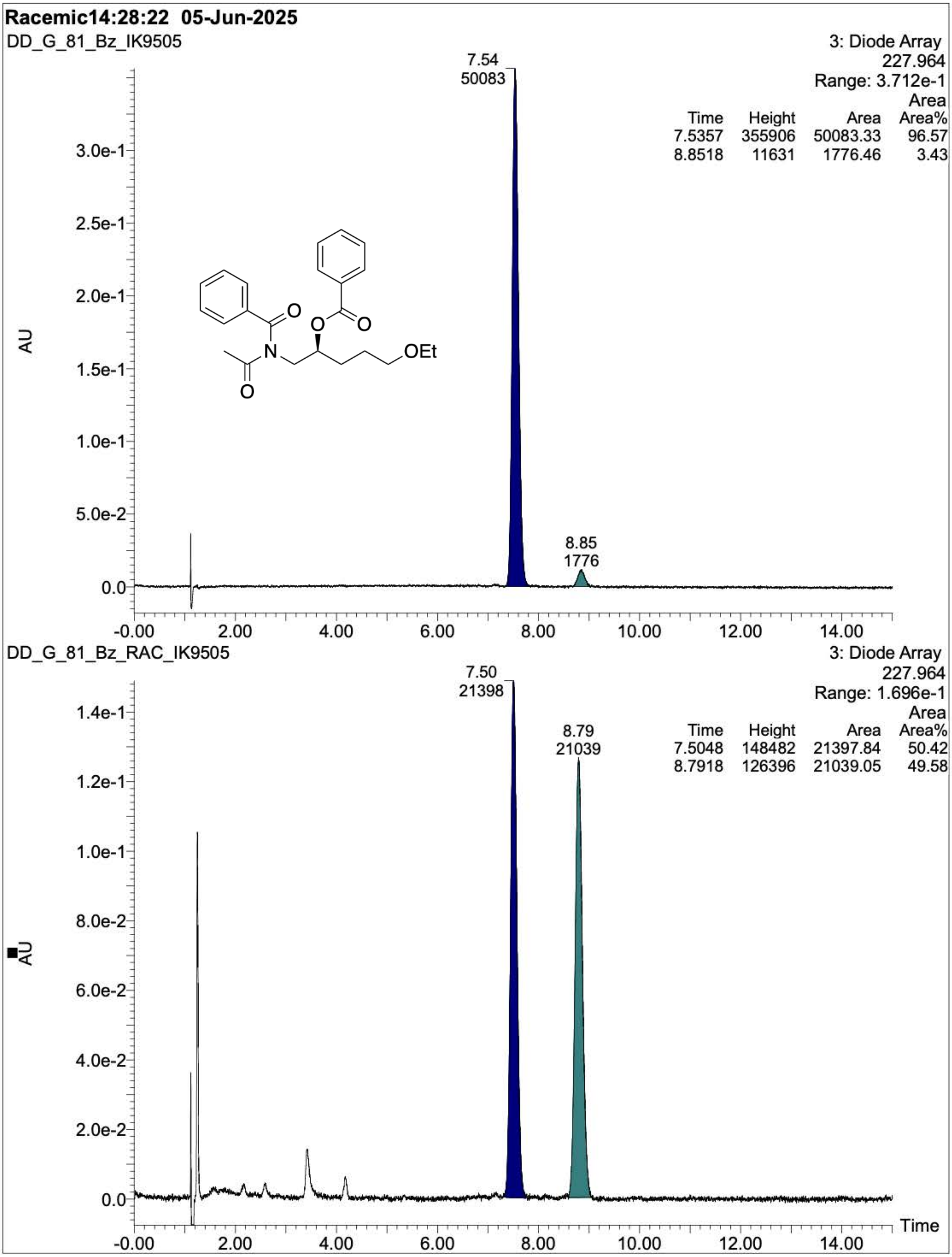

(*S*)-1-(*N*-acetylbenzamido)-4-(tetrahydro-2*H*-pyran-4-yl)butan-2-yl benzoate (1p-Bz)

Chiral SFC Analysis: CHIRALPAK IK (CO_2:MeOH, 90:10, 2.5 mL min^{-1}, 40 °C, 230 nm) t_R = 8.5 (major), 10.1 (minor) minutes, 95% ee

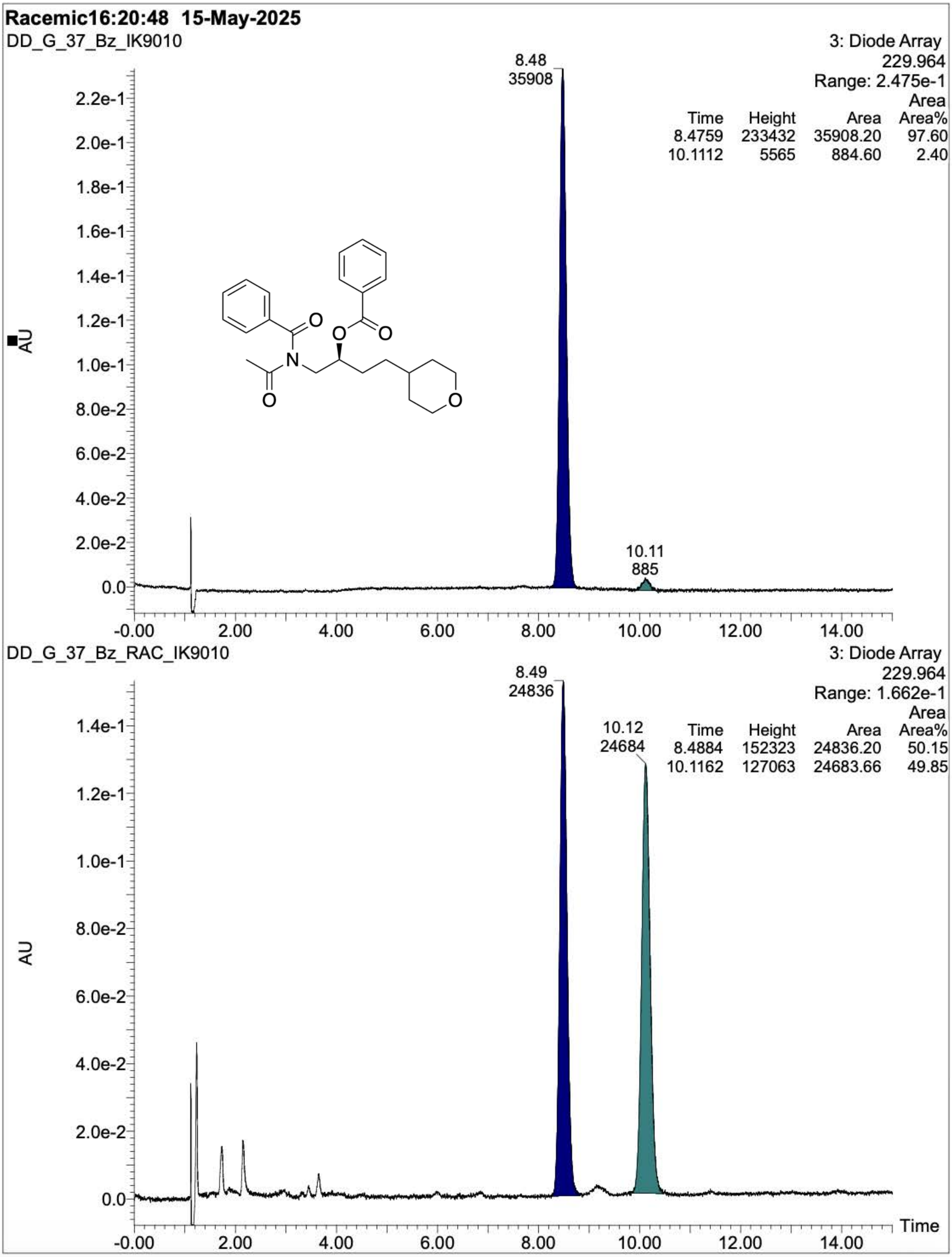

(S)-N-(2-hydroxy-4-phenoxybutyl)acetamide (1q)

Chiral SFC Analysis: CHIRALPAK IJ (CO_2:MeOH, 95:05, 2.5 mL min^{-1}, 40 °C, 217 nm) t_R = 9.4 (minor), 10.7 (major) minutes, 95% ee

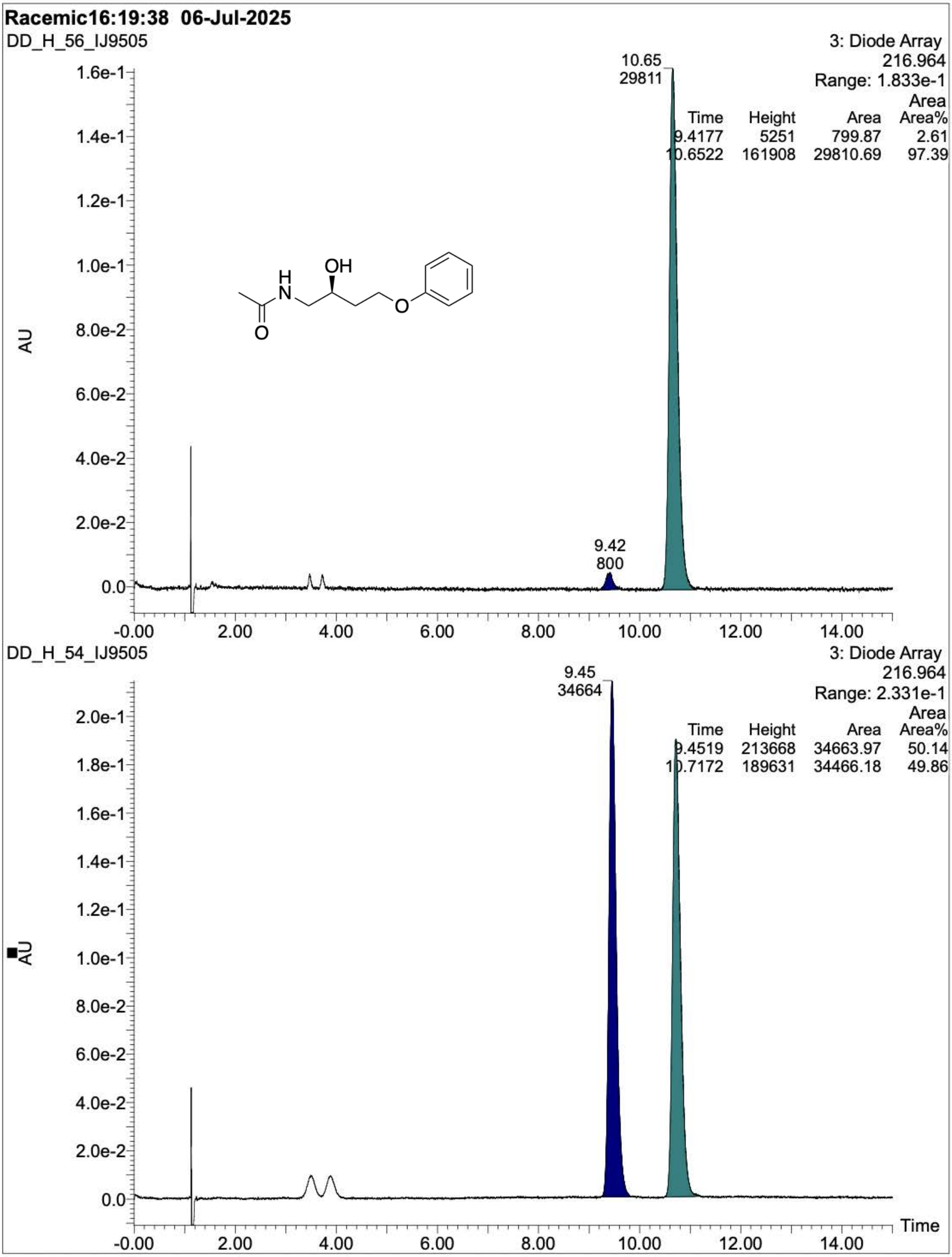

(*S*)-*N*-(2-hydroxy-3-(4-methoxyphenyl)propyl)acetamide (1r)

Chiral SFC Analysis: CHIRALPAK IJ (CO_2:MeOH, 93:07, 2.5 mL min^{-1}, 40 °C, 223 nm) t_R = 5.6 (minor), 7.3 (major) minutes, 91% ee

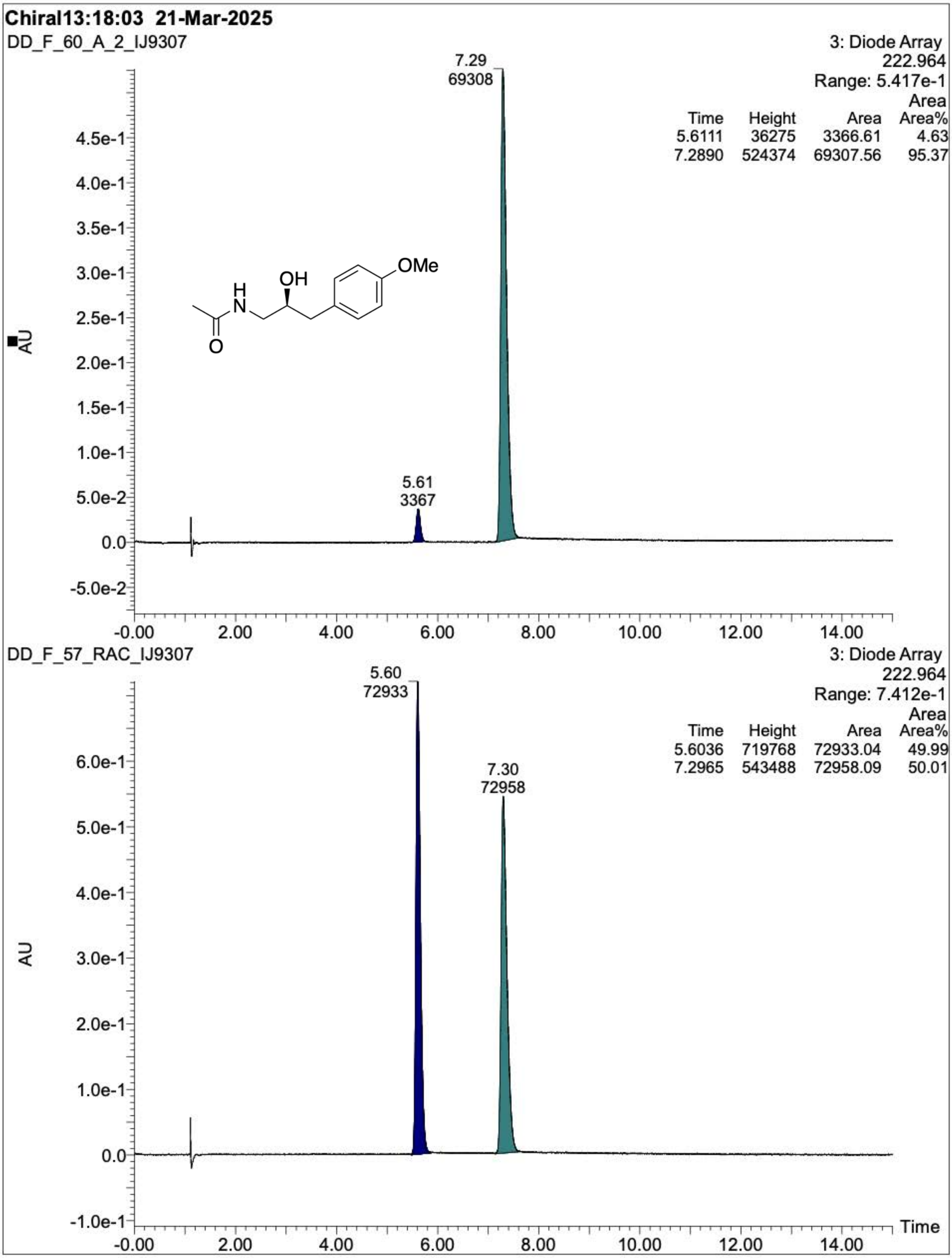

(*S*)-*N*-(2-hydroxy-3-(*p*-tolyl)propyl)acetamide (1s)

Chiral SFC Analysis: CHIRALPAK IJ (CO_2:MeOH, 95:05, 2.5 mL min^{-1}, 40 °C, 216 nm) t_R = 6.3 (minor), 7.9 (major) minutes, 90% ee

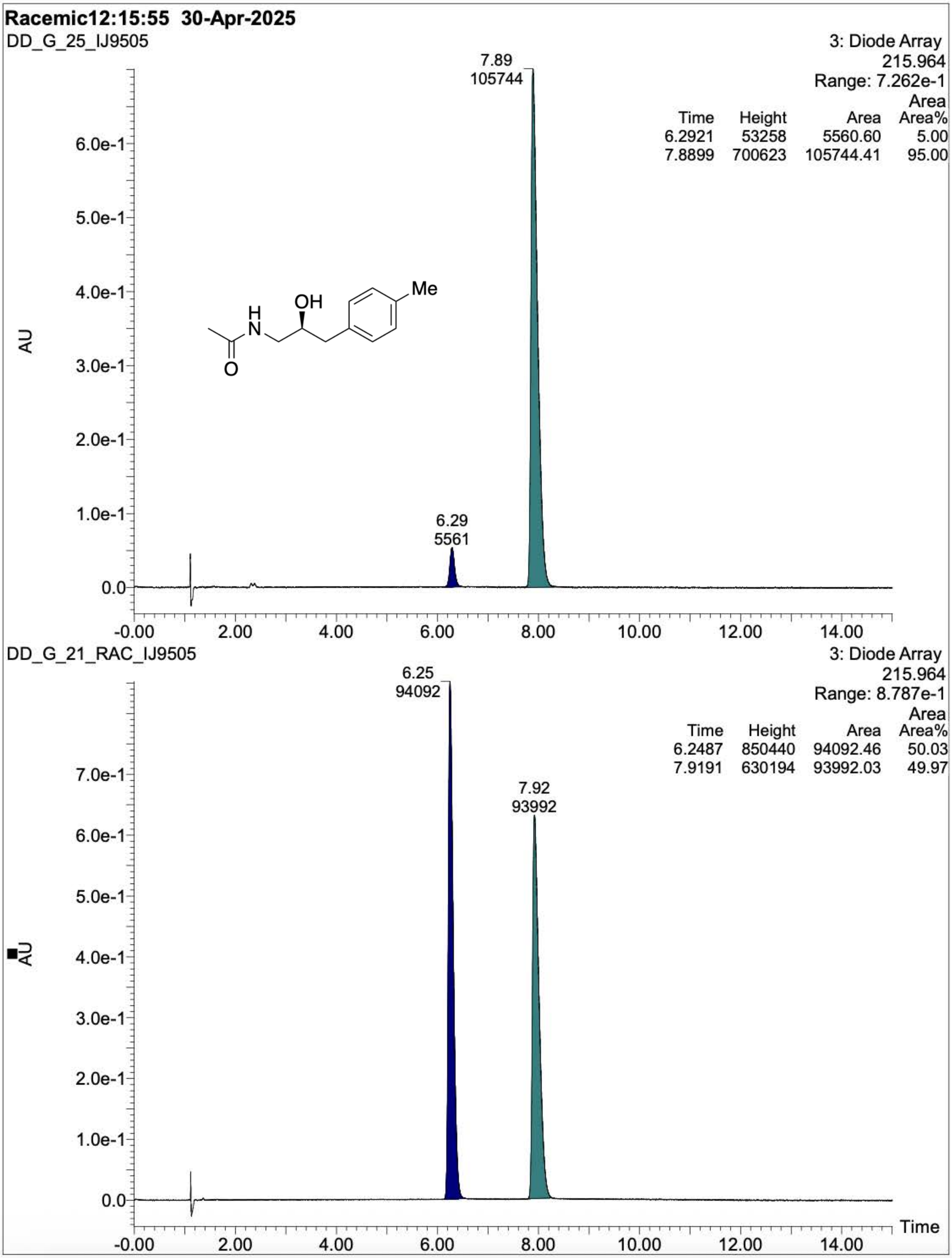

***tert*-butyl (*S*)-(2-hydroxy-3-(*p*-tolyl)propyl)carbamate (Boc-1s)**

Chiral SFC Analysis: CHIRALPAK IK (CO_2:MeOH, 95:05, 2.5 mL min^{-1}, 40 °C, 212 nm) t_R = 7.4 (minor), 7.8 (major) minutes, 90% ee

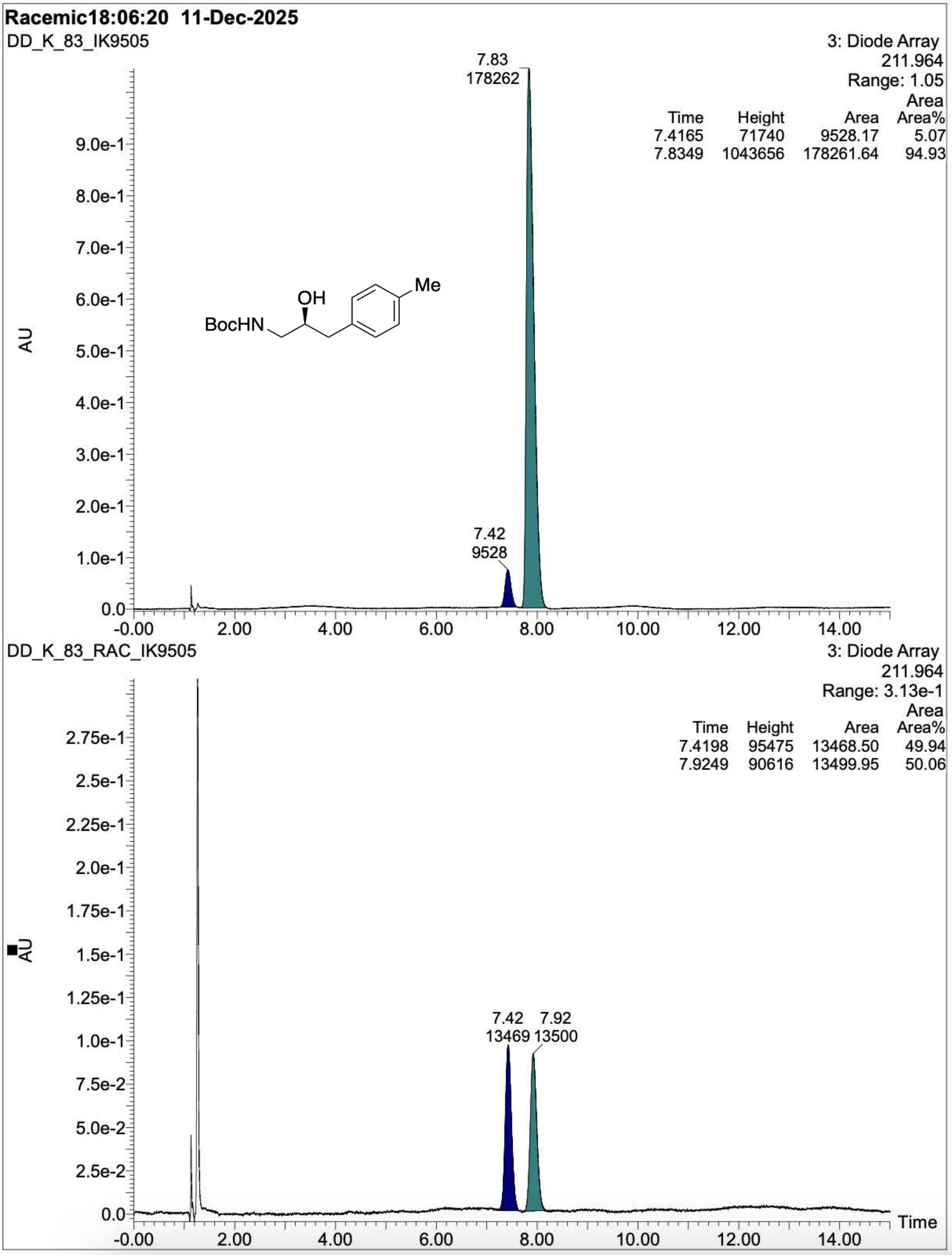

(*S*)-*N*-(3-(4-fluorophenyl)-2-hydroxypropyl)acetamide (1t)

Chiral SFC Analysis: CHIRALPAK IJ (CO_2:MeOH, 95:05, 2.5 mL min^{-1}, 40 °C, 203 nm) t_R = 4.5 (minor), 5.2 (major) minutes, 94% ee

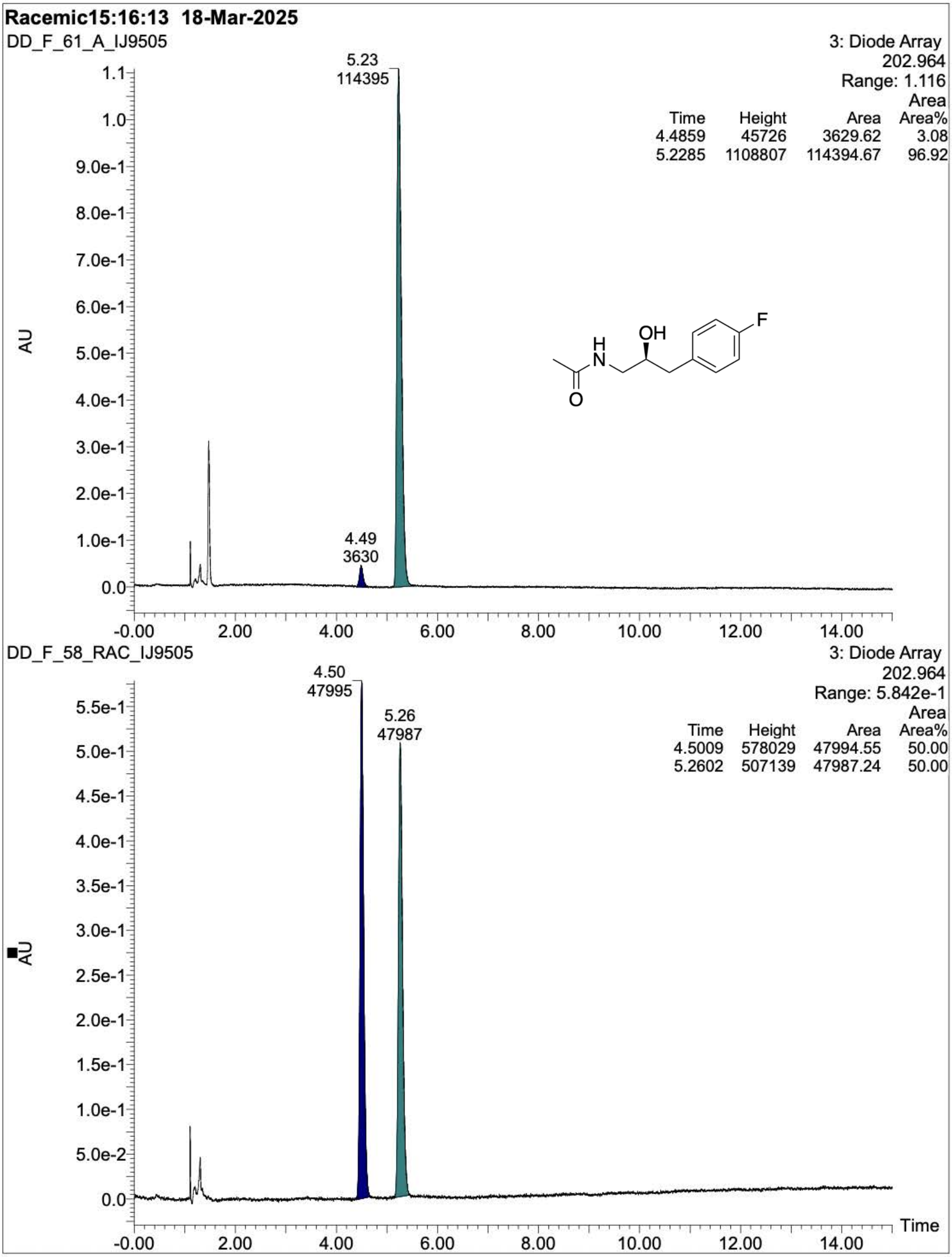

(*S*)-*N*-(3-(3-chlorophenyl)-2-hydroxypropyl)acetamide (1u)

Chiral SFC Analysis: CHIRALPAK IJ (CO_2:MeOH, 95:05, 2.5 mL min^{-1}, 40 °C, 211 nm) t_R = 7.1 (minor), 8.8 (major) minutes, 87% ee

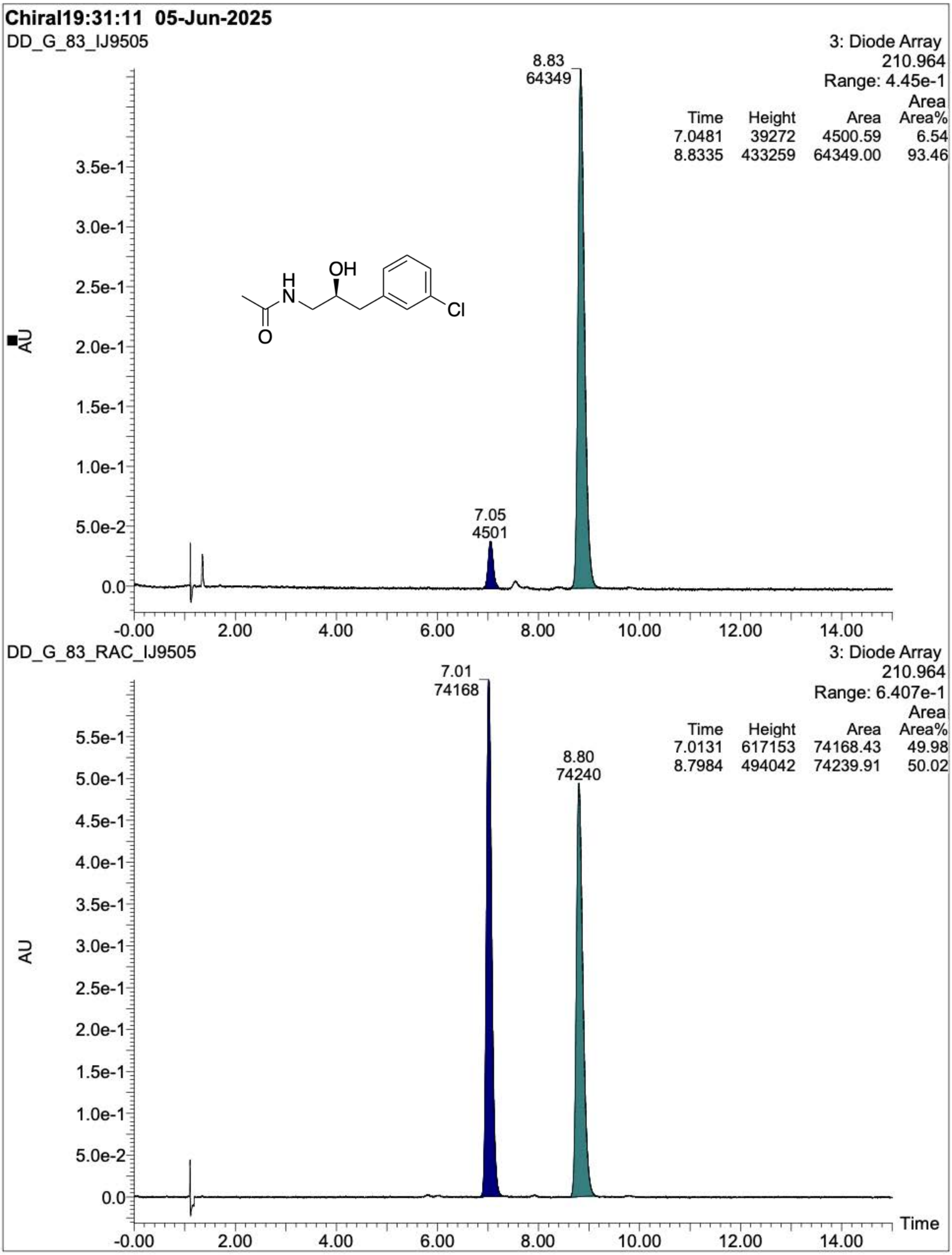

(*S*)-*N*-(2-hydroxy-3-(naphthalen-1-yl)propyl)acetamide (1v)

Chiral SFC Analysis: CHIRALPAK IJ (CO_2:MeOH, 91:09, 2.5 mL min^{-1}, 40 °C, 280 nm) t_R = 5.8 (minor), 6.5 (major) minutes, 81% ee

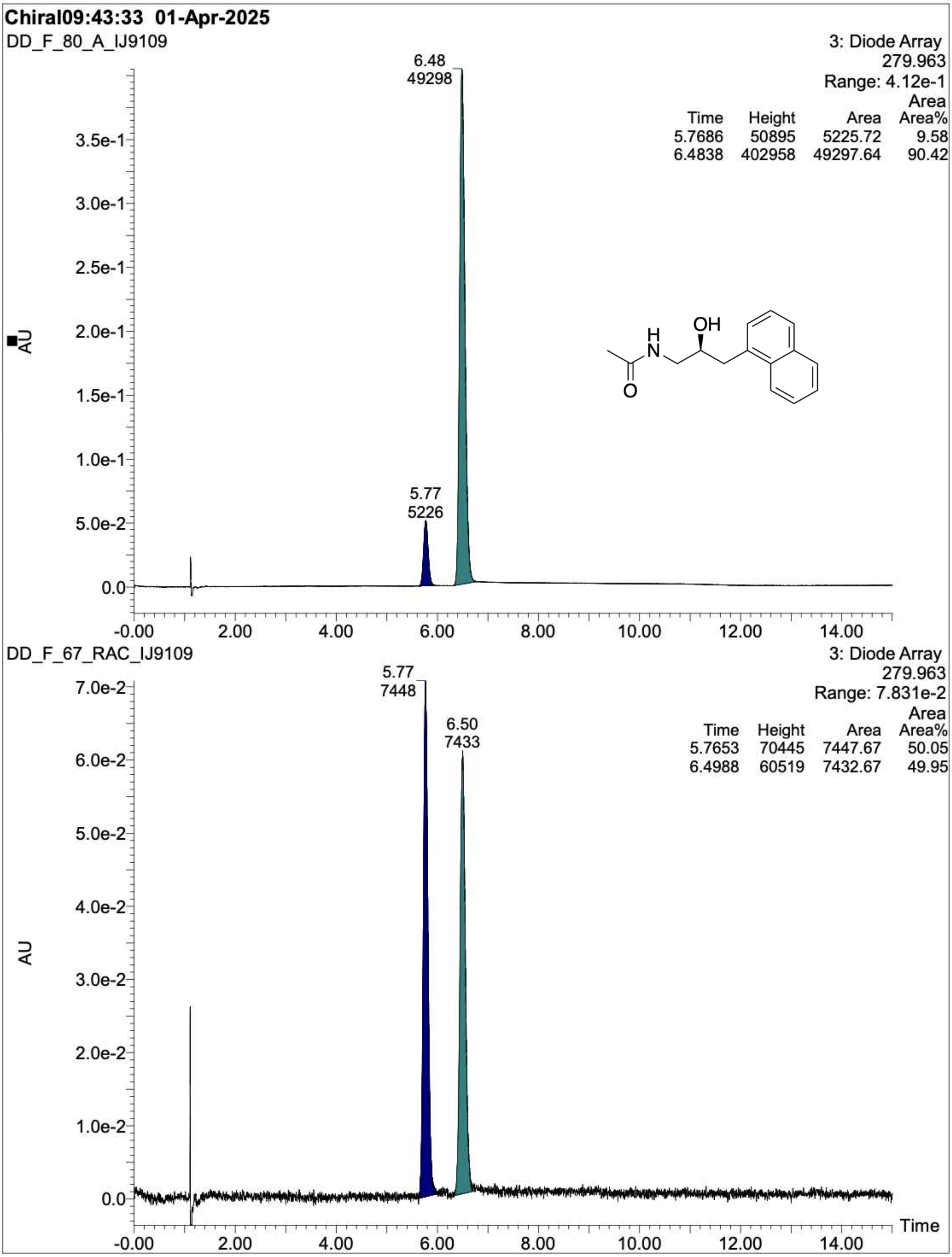

(*S*)-*N*-(2-hydroxy-3-(thiophen-3-yl)propyl)acetamide (1w)

Chiral SFC Analysis: CHIRALPAK IJ (CO_2:MeOH, 95:05, 2.5 mL min^{-1}, 40 °C, 232 nm) t_R = 6.6 (minor), 7.3 (major) minutes, 79% ee

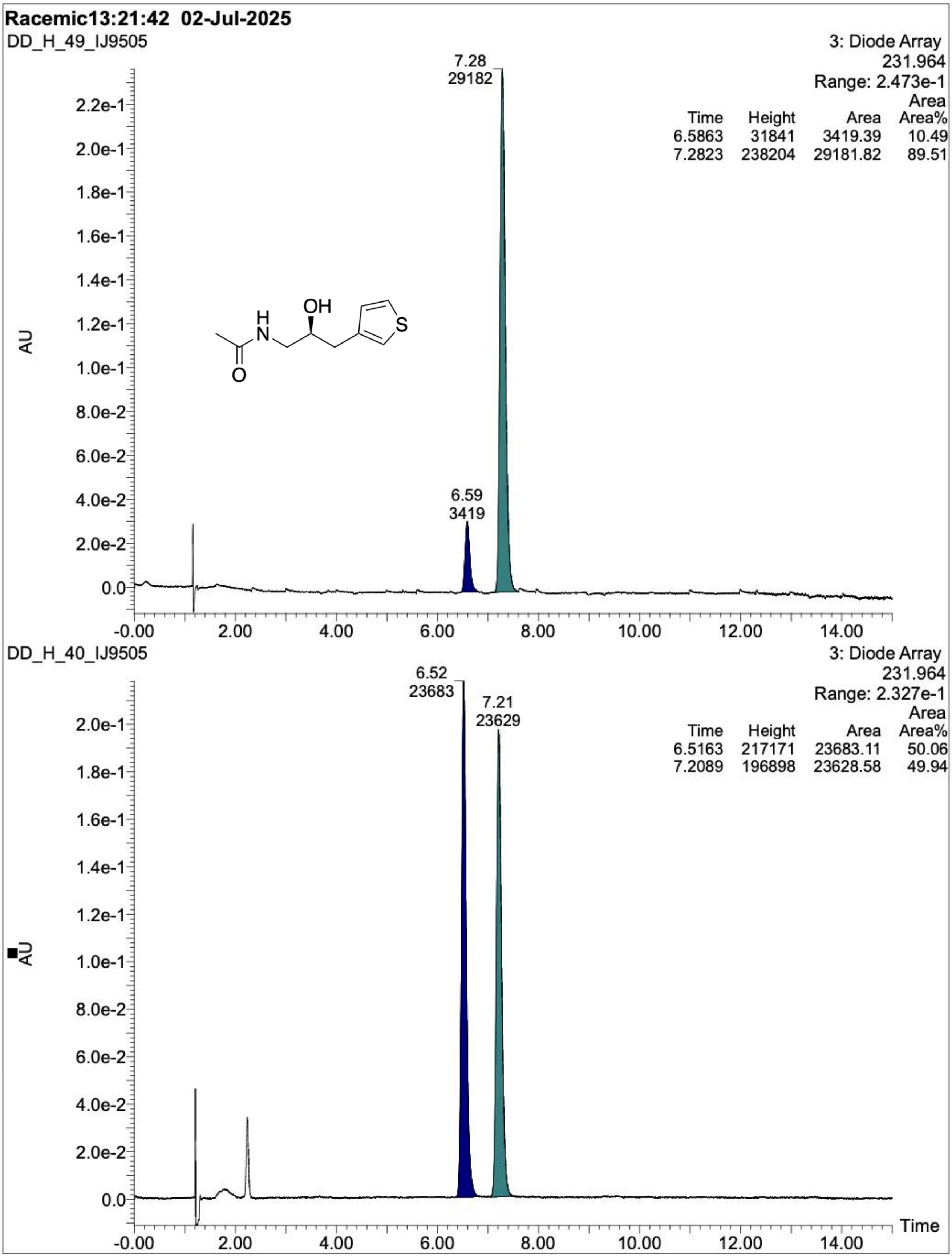

(*S*)-1-(*N*-acetylbenzamido)-4,4-dimethylpentan-2-yl benzoate (1x-Bz)

Chiral SFC Analysis: CHIRALPAK IK (CO_2:MeOH, 96:04, 2.5 mL min^{-1}, 40 °C, 227 nm) t_R = 4.6 (major), 5.4 (minor) minutes, 15% ee

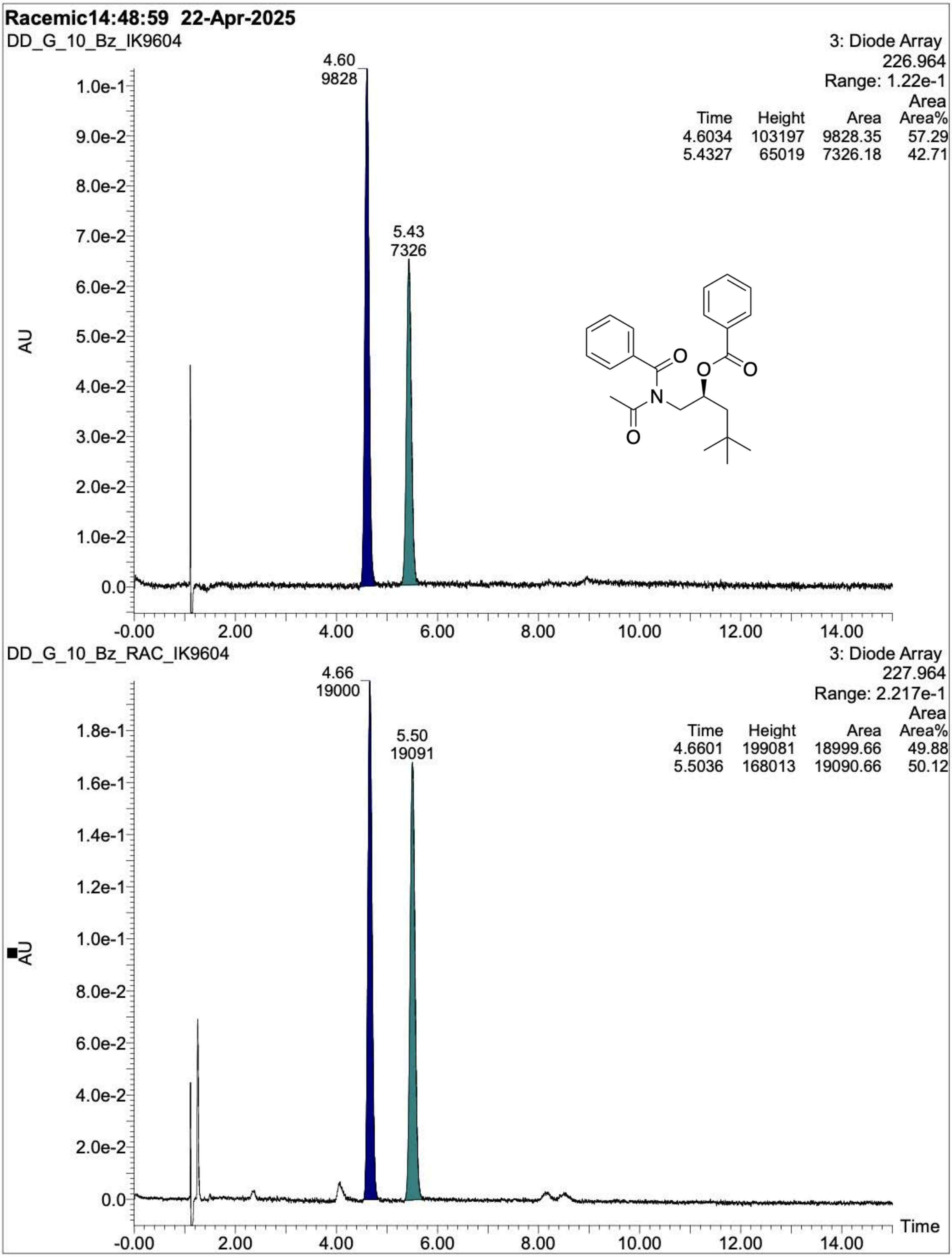

(*S*)-5-(*N*-acetylbenzamido)pentane-1,4-diyl dibenzoate (1y)

Chiral SFC Analysis: CHIRALPAK IK (CO_2:MeOH, 87:13, 2.5 mL min^{-1}, 40 °C, 226 nm) t_R = 8.4 (major), 9.8 (minor) minutes, 75% ee

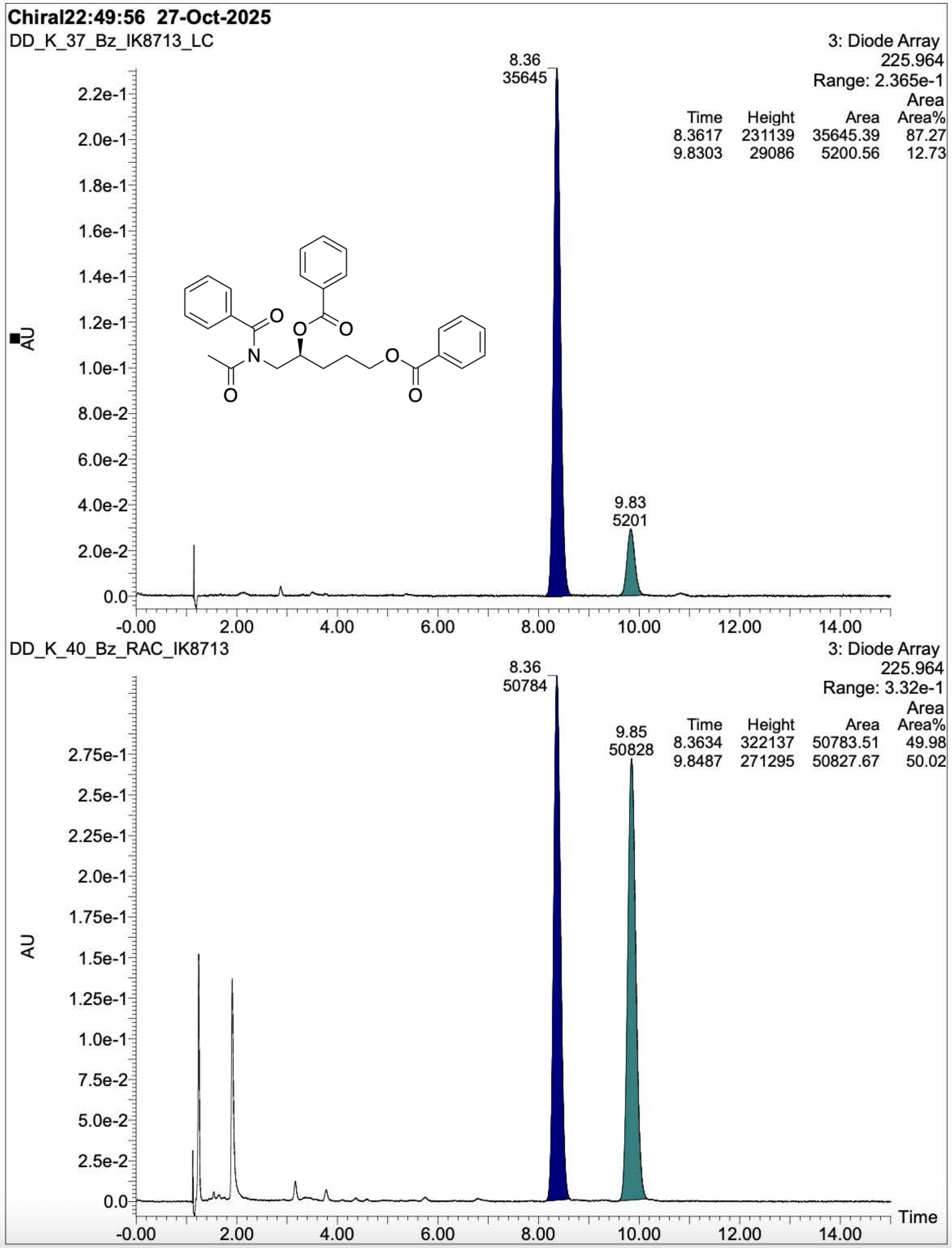

(*S*)-1-(*N*-isobutyrylbenzamido)butan-2-yl benzoate (1z-Bz)

Chiral SFC Analysis: CHIRALPAK IK (CO_2:MeOH, 98:02, 2.5 mL min^{-1}, 40 °C, 228 nm) t_R = 7.6 (major), 8.8 (minor) minutes, 93% ee

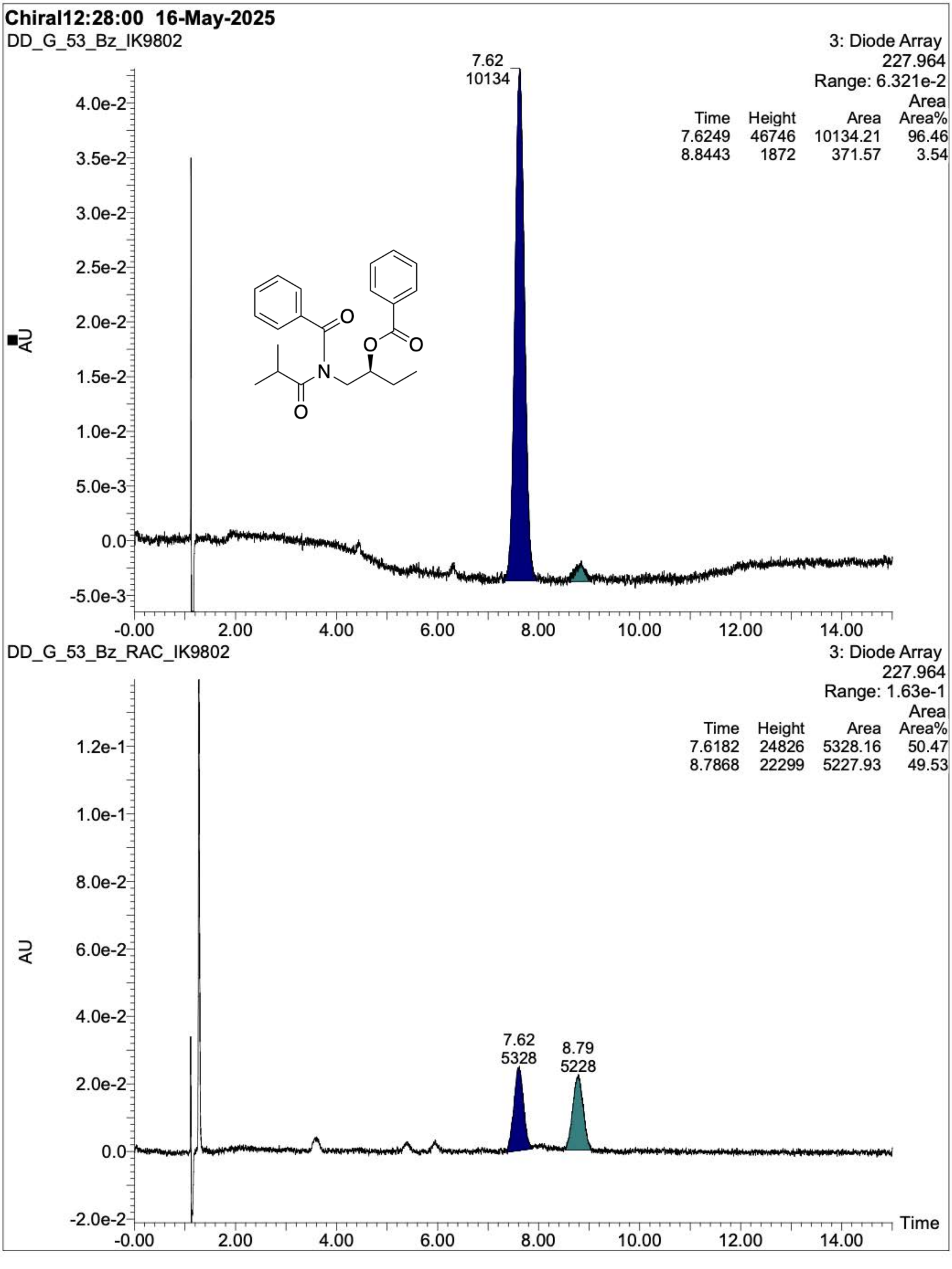

(*S*)-1-((3,5,7)-adamantane-1-carboxamido)butan-2-yl benzoate (1za-Bz)

Chiral SFC Analysis: CHIRALPAK IK (CO_2:MeOH, 92:08, 1.25 mL min^{-1}, 40 °C, 226 nm) t_R = 20.0 (minor), 20.8 (major) minutes, 86% ee

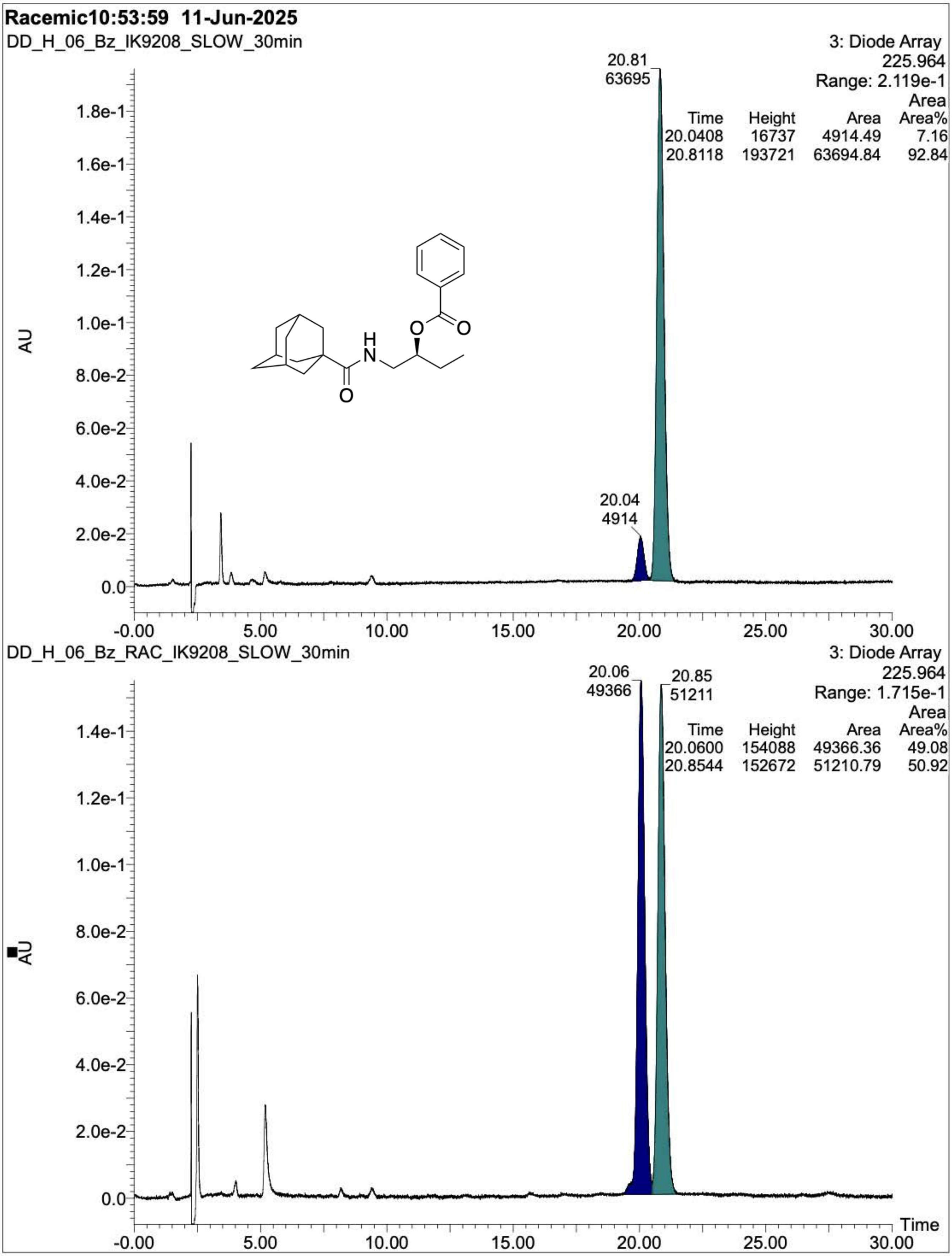

(*S*)-1-(*N*-(3-cyanopropanoyl)benzamido)butan-2-yl benzoate (1zb-Bz)

Chiral SFC Analysis: CHIRALPAK IK (CO_2:MeOH, 93:07, 2.5 mL min^{-1}, 40 °C, 228 nm) t_R = 6.9 (major), 8.3 (minor) minutes, 97% ee

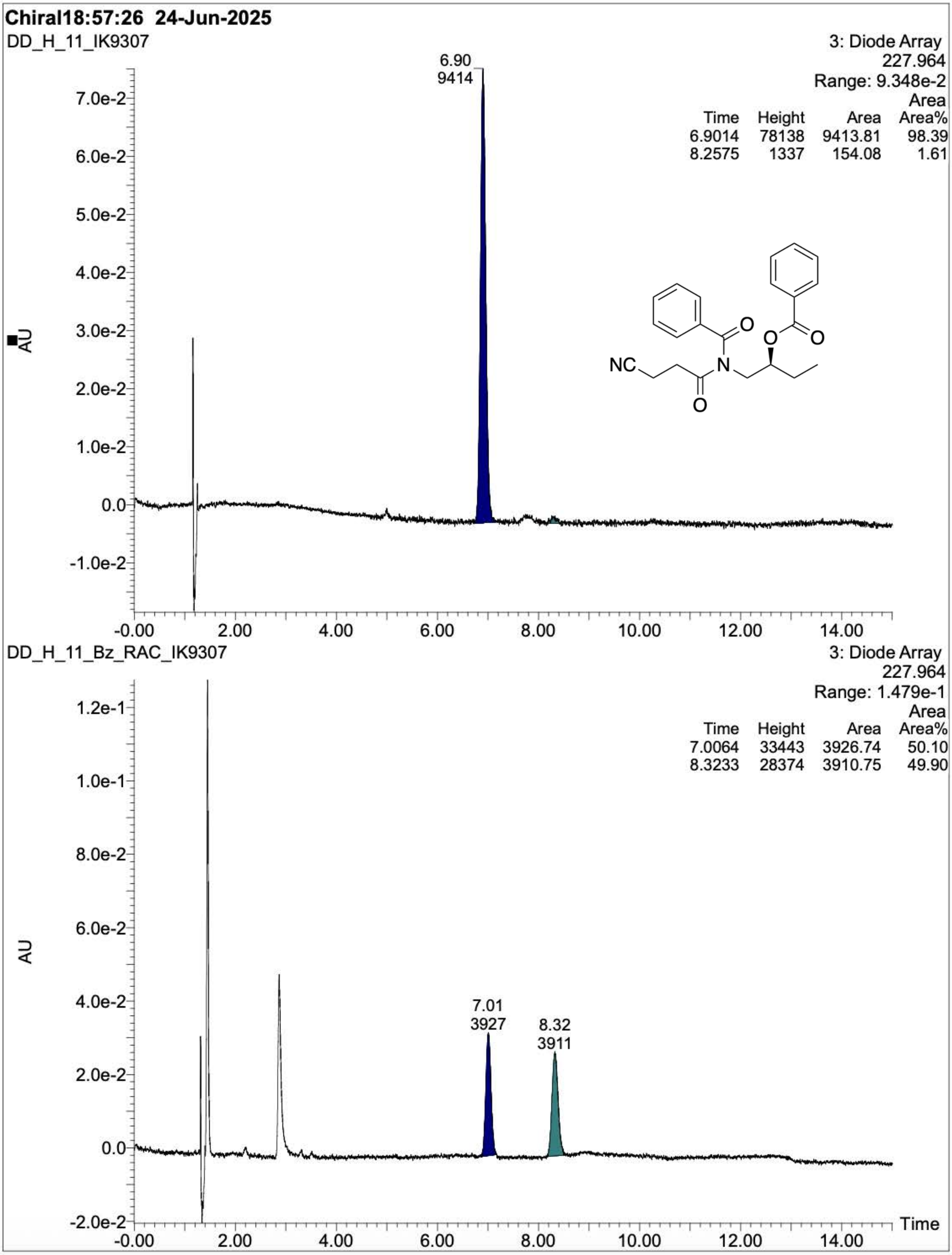

(*S*)-*N*-(2-hydroxybutyl)-2-(phenylsulfonyl)acetamide (1zc)

Chiral SFC Analysis: CHIRALPAK IE (CO_2:MeOH, 85:15, 2.5 mL min^{-1}, 40 °C, 215 nm) t_R = 12.1 (major), 13.0 (minor) minutes, 99% ee

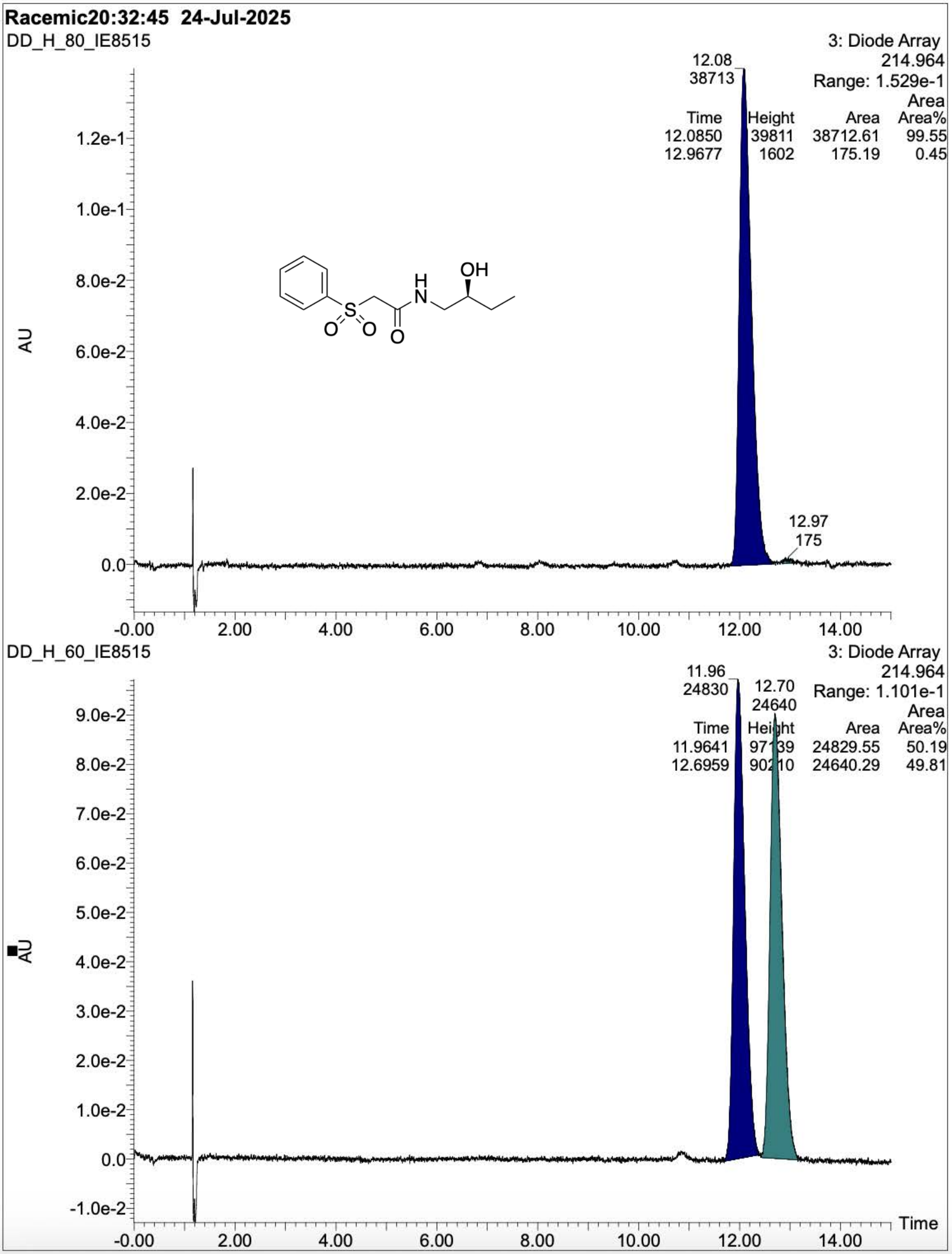

(*S*)-*N*-(2-hydroxybutyl)-3-phenylpropanamide (1zd)

Chiral SFC Analysis: CHIRALPAK IJ (CO_2:MeOH, 96:04, 2.5 mL min^{-1}, 40 °C, 203 nm) t_R = 8.2 (minor), 9.5 (major) minutes, 90% ee

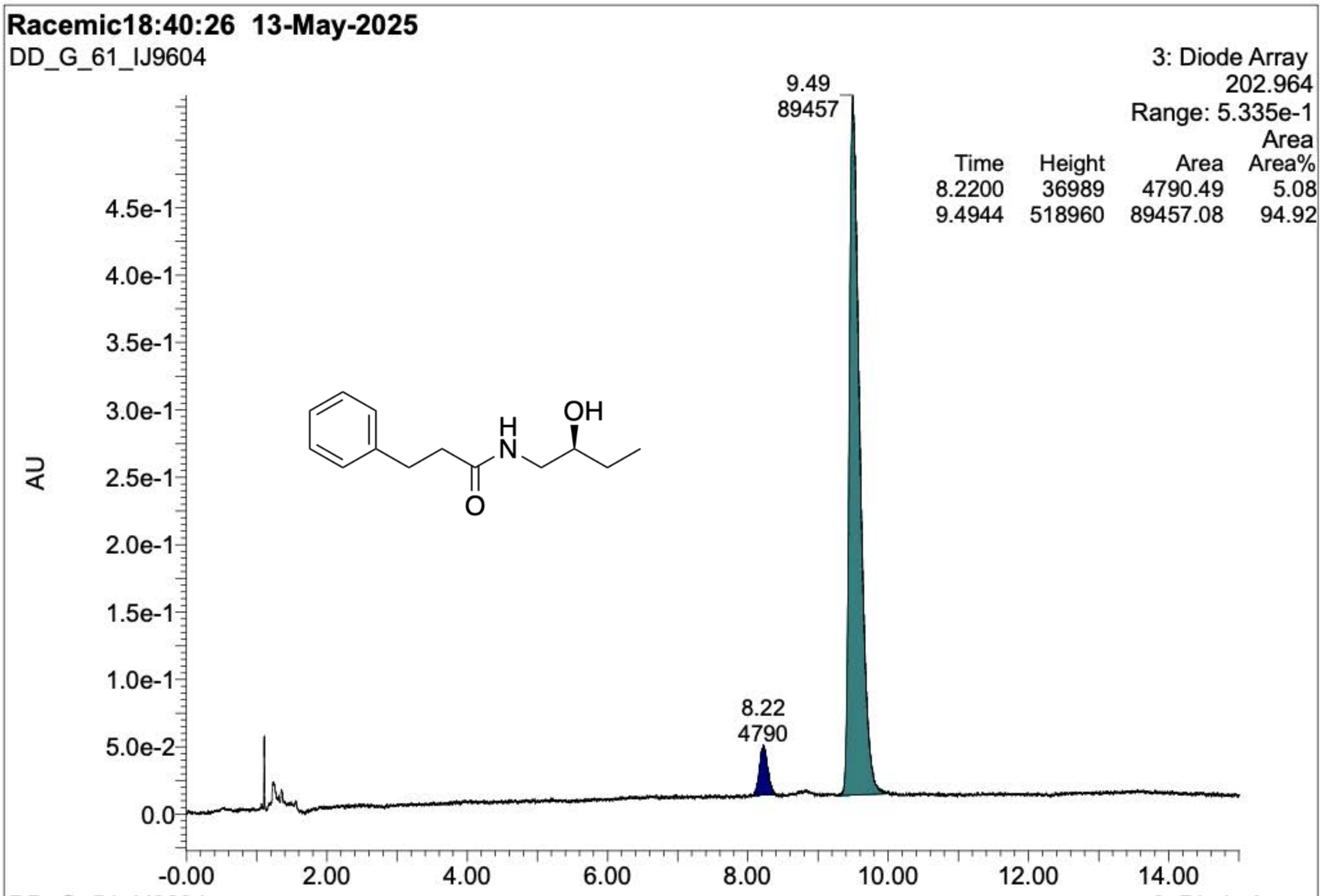

Time	Height	Area	Area Area%
8.2200	36989	4790.49	5.08
9.4944	518960	89457.08	94.92

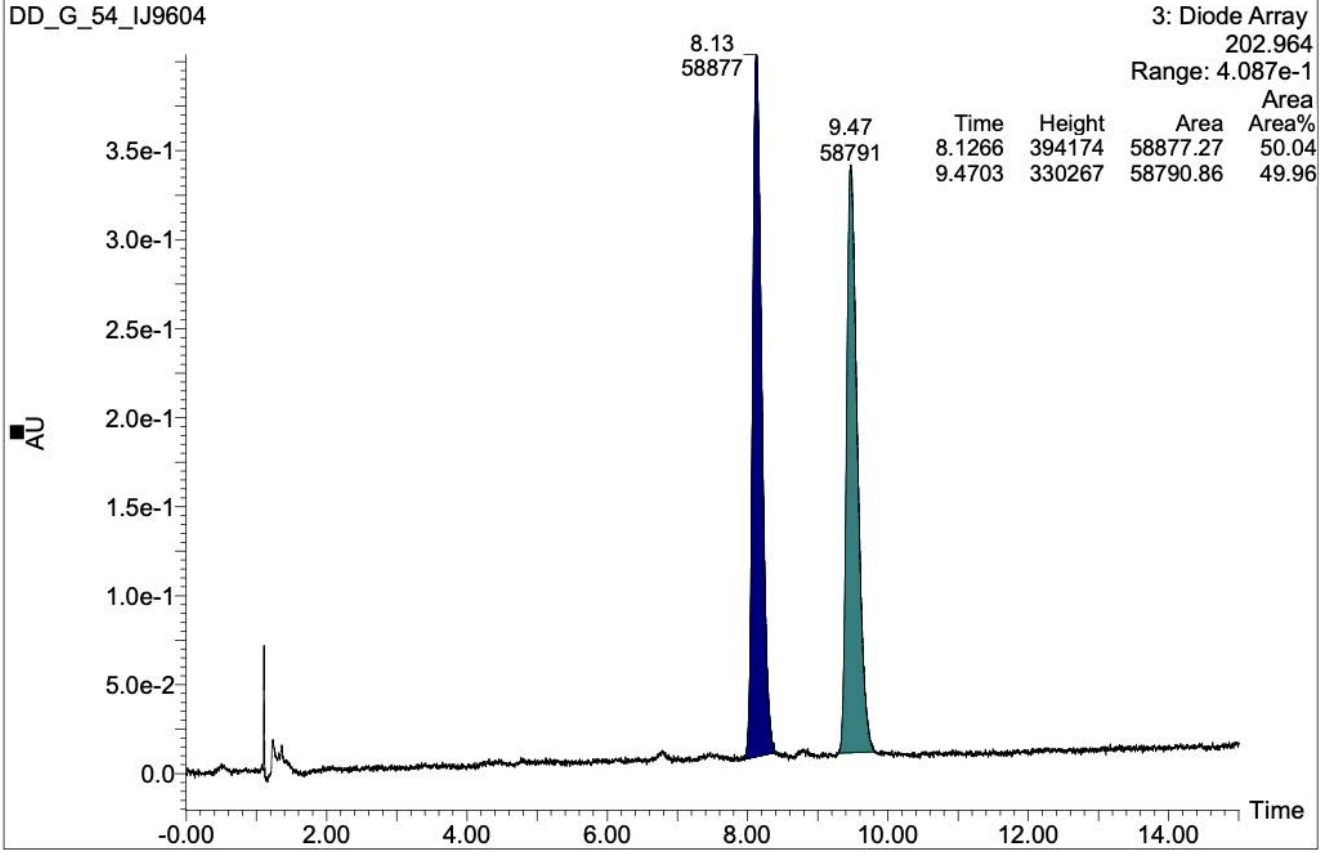

Time	Height	Area	Area Area%
8.1266	394174	58877.27	50.04
9.4703	330267	58790.86	49.96

(*S*)-*N*-(2-hydroxybutyl)benzamide (1ze)

Chiral SFC Analysis: CHIRALPAK IJ (CO_2:MeOH, 95:05, 2.5 mL min^{-1}, 40 °C, 223 nm) t_R = 4.5 (minor), 5.2 (major) minutes, 83% ee

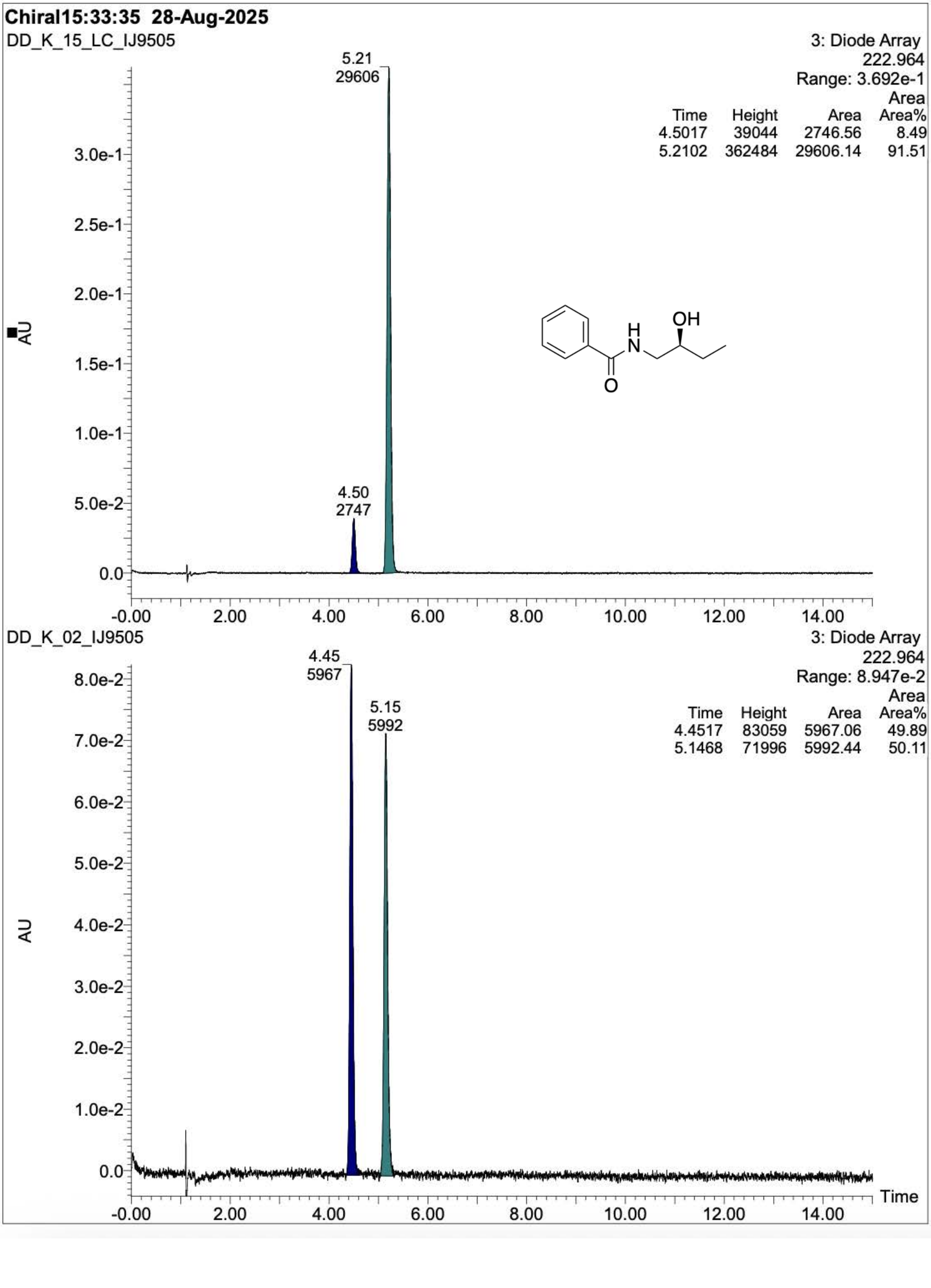

(*S*)-4-(*tert*-butyl)-*N*-(2-hydroxybutyl)benzamide (1zf)

Chiral SFC Analysis: CHIRALPAK IJ (CO_2:MeOH, 95:05, 2.5 mL min^{-1}, 40 °C, 233 nm) t_R = 4.2 (minor), 4.7 (major) minutes, 81% ee

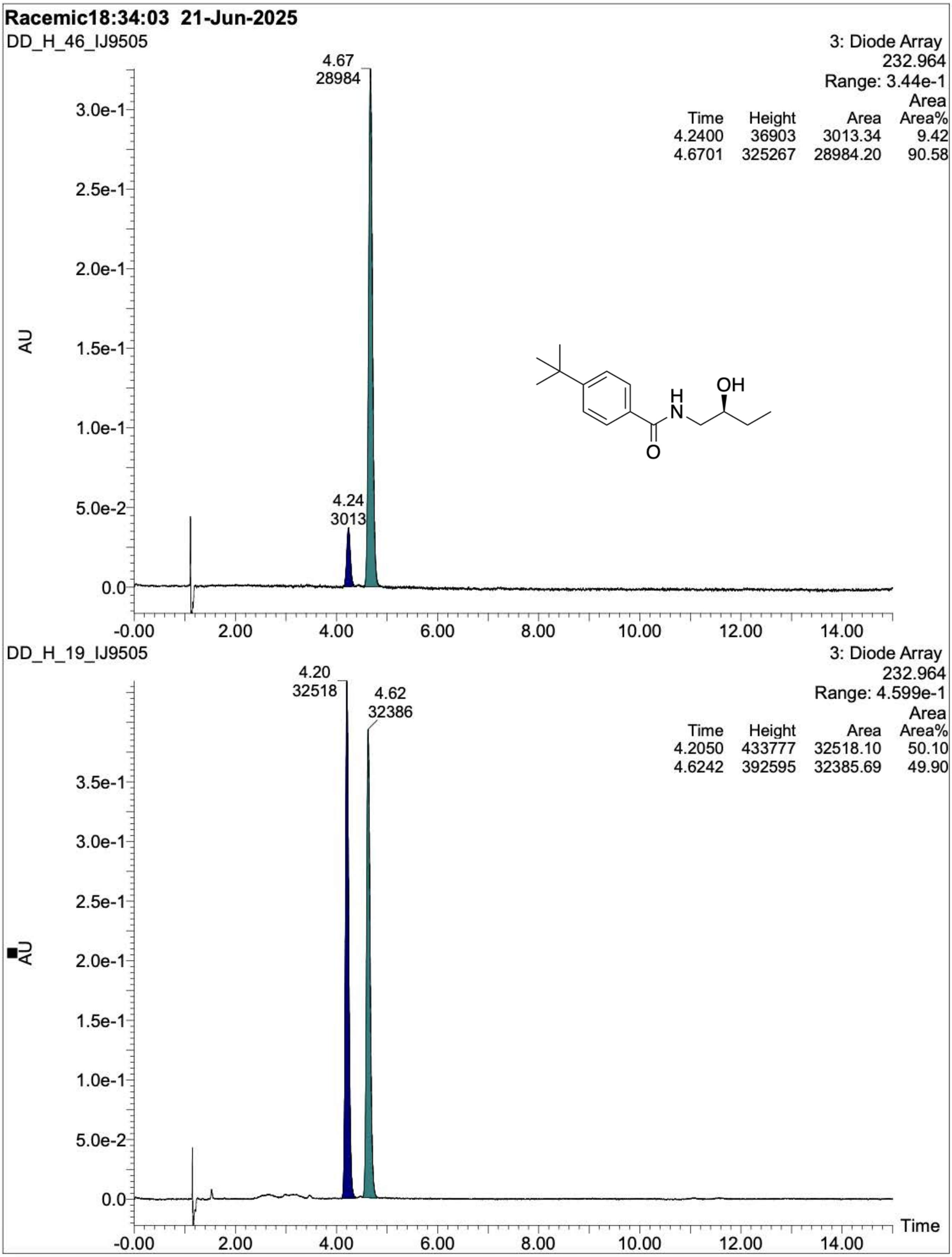

(*S*)-*N*-(2-hydroxybutyl)-4-(trifluoromethyl)benzamide (1zg)

Chiral SFC Analysis: CHIRALPAK IJ (CO_2:MeOH, 97:03, 2.5 mL min^{-1}, 40 °C, 220 nm) t_R = 4.7 (minor), 5.1 (major) minutes, 89% ee

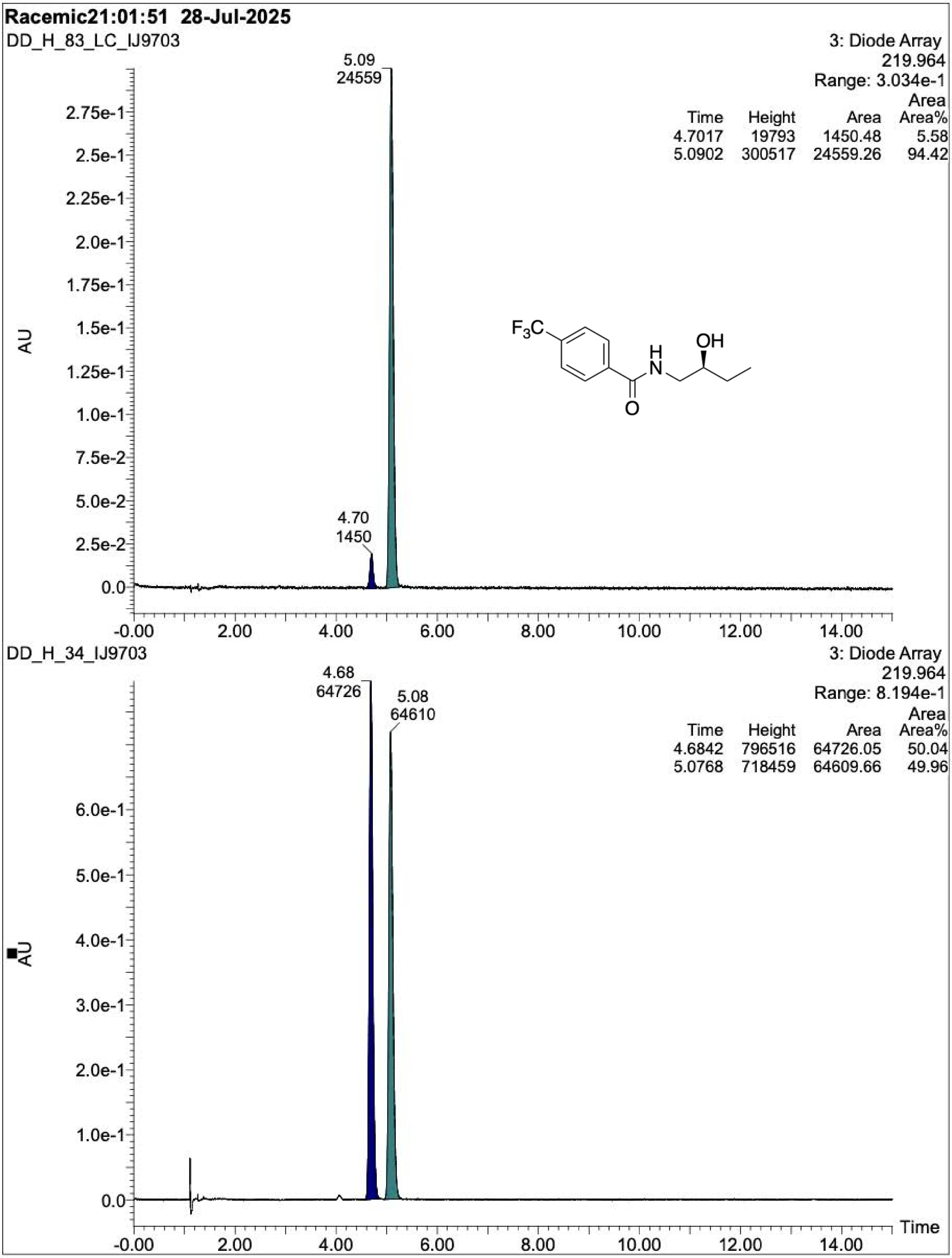

(*S*)-*N*-(2-hydroxybutyl)-4-methoxybenzamide (1zh)

Chiral SFC Analysis: CHIRALPAK IJ (CO_2:MeOH, 95:05, 2.5 mL min^{-1}, 40 °C, 247 nm) t_R = 7.3 (minor), 8.5 (major) minutes, 93% ee

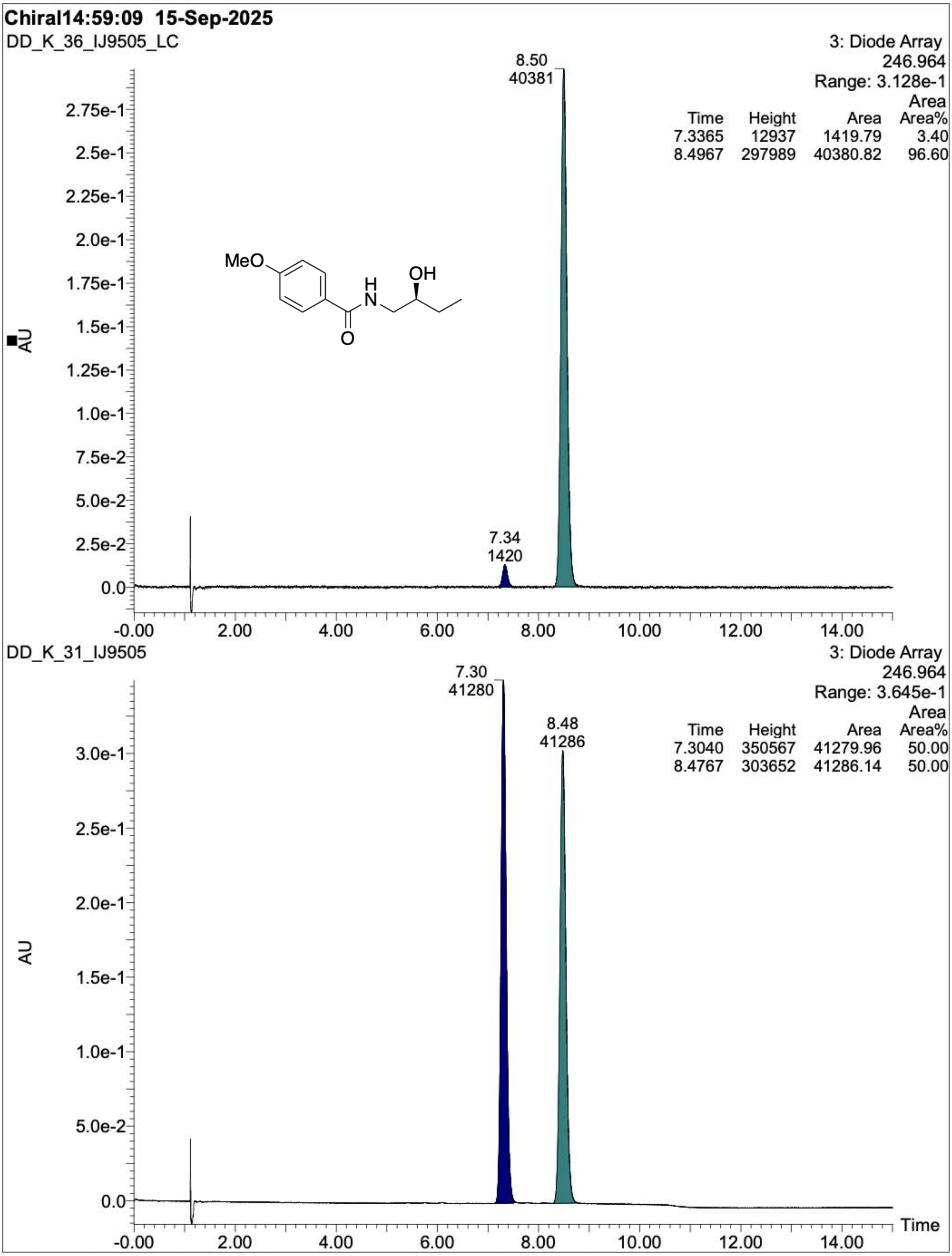

(*S*)-*N*-(2-hydroxybutyl)benzo[*d*][1,3]dioxole-5-carboxamide (1zi)

Chiral SFC Analysis: CHIRALPAK IJ (CO_2:MeOH, 95:05, 2.5 mL min^{-1}, 40 °C, 255 nm) t_R = 7.5 (minor), 8.3 (major) minutes, 87% ee

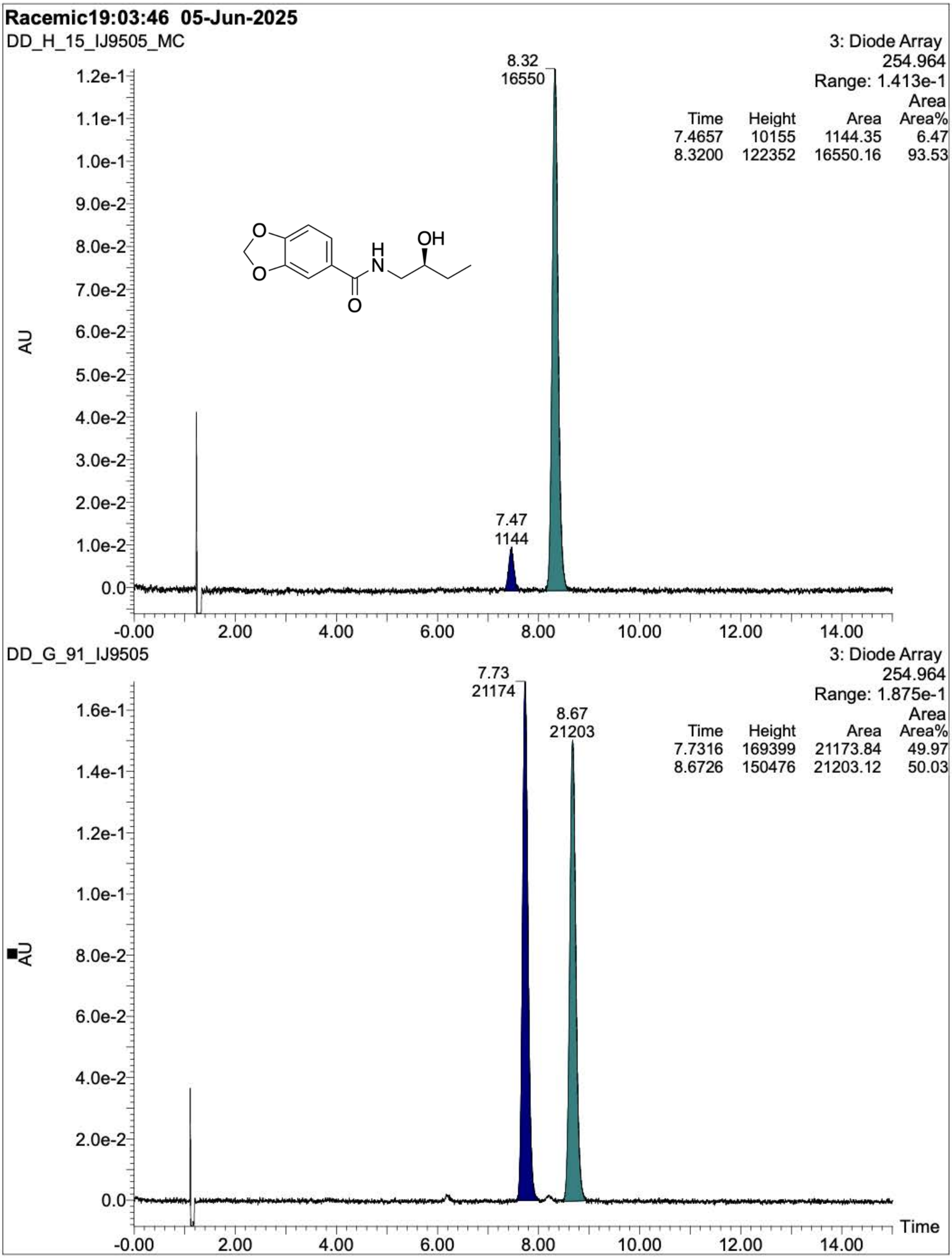

(*S*)-*N*-(2-hydroxybutyl)furan-3-carboxamide (1zj)

Chiral SFC Analysis: CHIRALPAK IJ (CO_2:MeOH, 95:05, 2.5 mL min^{-1}, 40 °C, 212 nm) t_R = 3.3 (minor), 3.6 (major) minutes, 94% ee

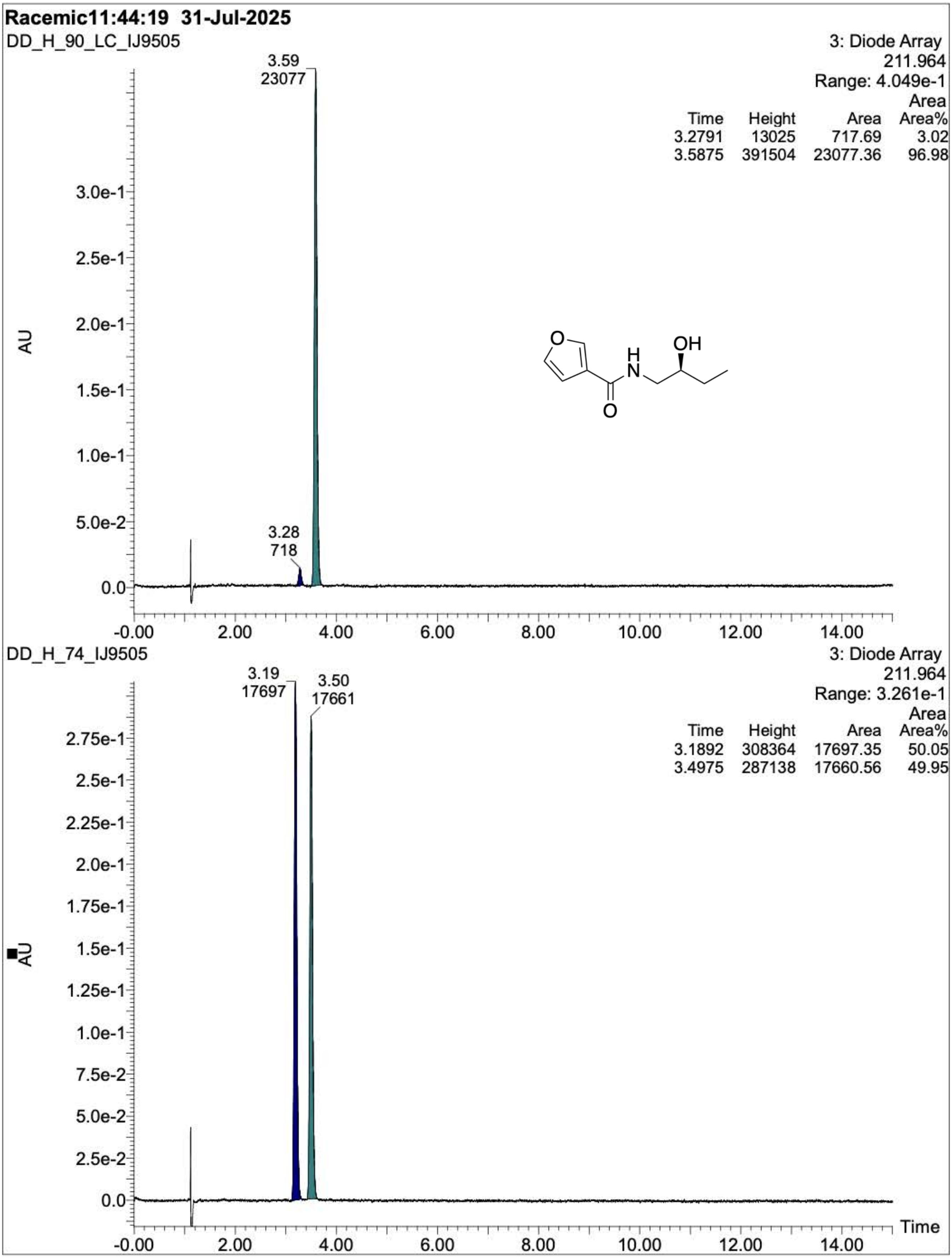

(*S*)-*N*-(2-hydroxybutyl)-1-methyl-1*H*-pyrazole-4-carboxamide (1zk)

Chiral SFC Analysis: CHIRALPAK IJ (CO_2:MeOH, 95:05, 2.5 mL min^{-1}, 40 °C, 224 nm) t_R = 4.3 (minor), 5.0 (major) minutes, 82% ee

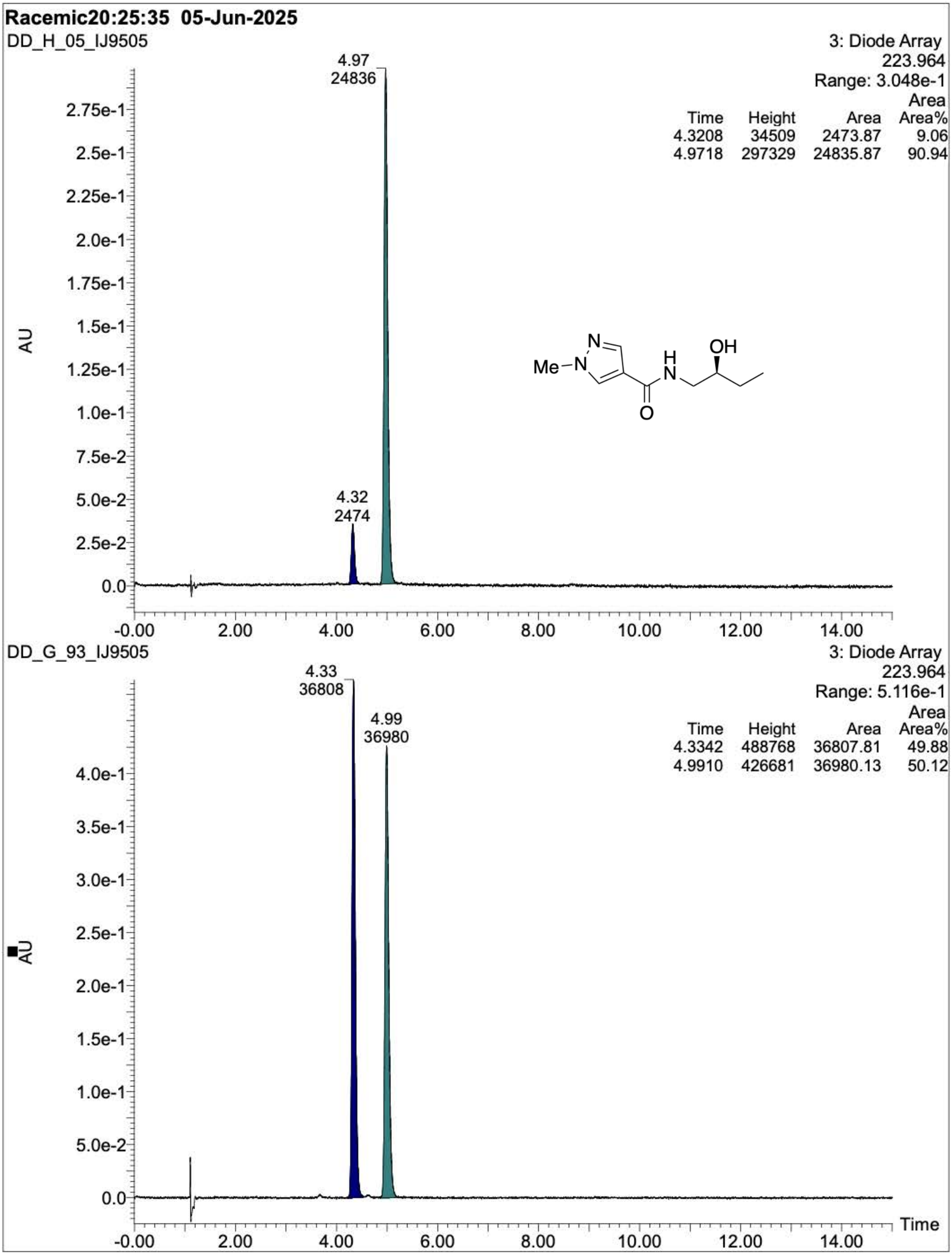

(*S*)-6-chloro-*N*-(2-hydroxybutyl)nicotinamide (1zl), (*R*)-6-chloro-*N*-(2-hydroxybutyl)nicotinamide ((*R*)-1zl)

Chiral SFC Analysis: CHIRALPAK IJ (CO_2:MeOH, 94:06, 2.5 mL min^{-1}, 40 °C, 267 nm) t_R = 6.1 (minor), 7.6 (major) minutes, 97% ee; t_R = 6.1 (major), 7.5 (minor) minutes, −82% ee

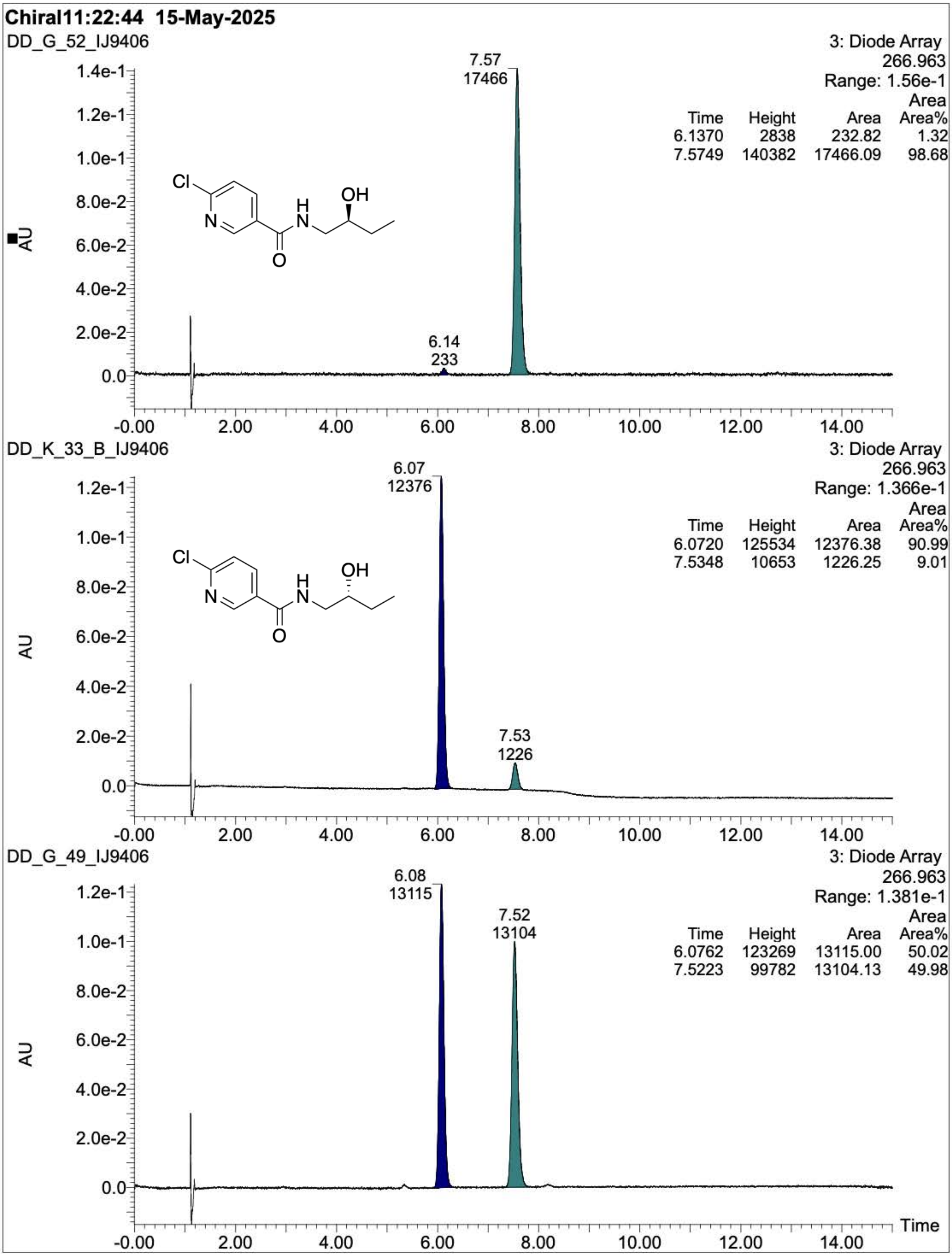

(*S*)-*N*-(2-hydroxybutyl)-2-(thiazol-4-yl)acetamide (1zm)

Chiral SFC Analysis: CHIRALPAK IC (CO_2:MeOH, 90:10, 2.5 mL min^{-1}, 40 °C, 239 nm) t_R = 11.3 minutes, > 99% ee

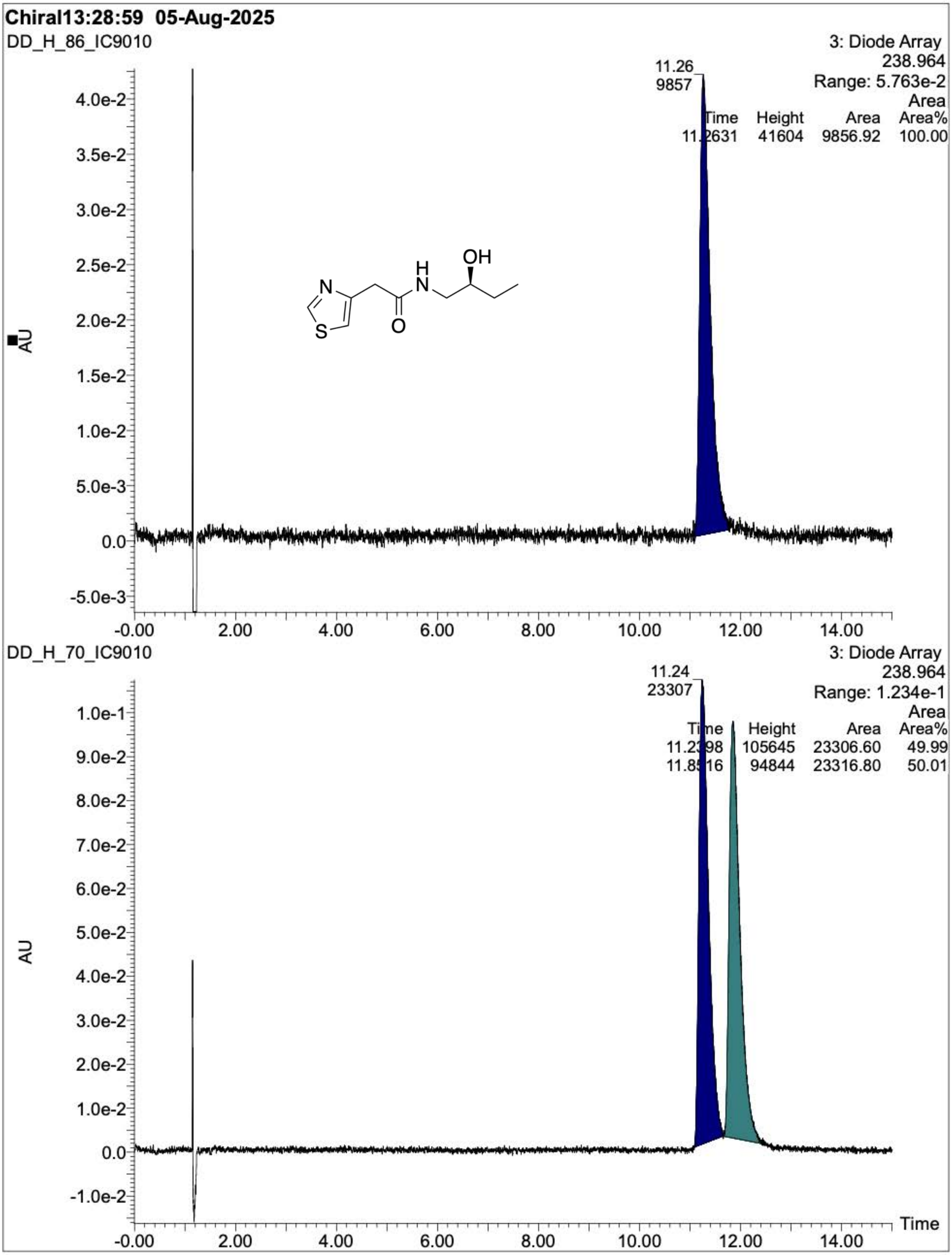

tert-butyl (_S_)-4-(benzoyl(2-(benzoyloxy)butyl)carbamoyl)piperidine-1-carboxylate (1zn-Bz)

Chiral SFC Analysis: CHIRALPAK IJ (CO_2:MeOH, 96:04, 2.5 mL min^{-1}, 40 °C, 226 nm) t_R = 4.4 (major), 5.0 (minor) minutes, 92% ee

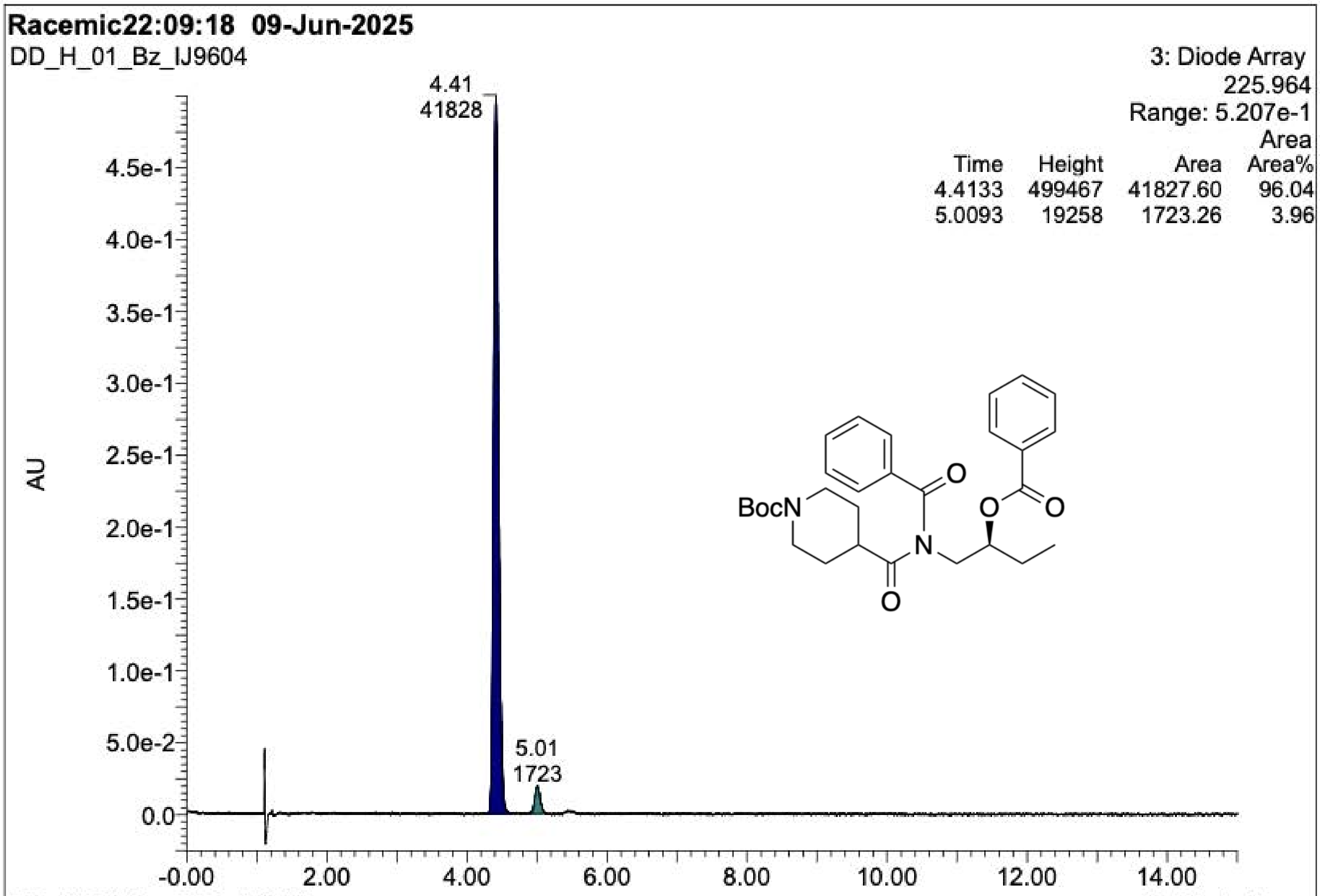

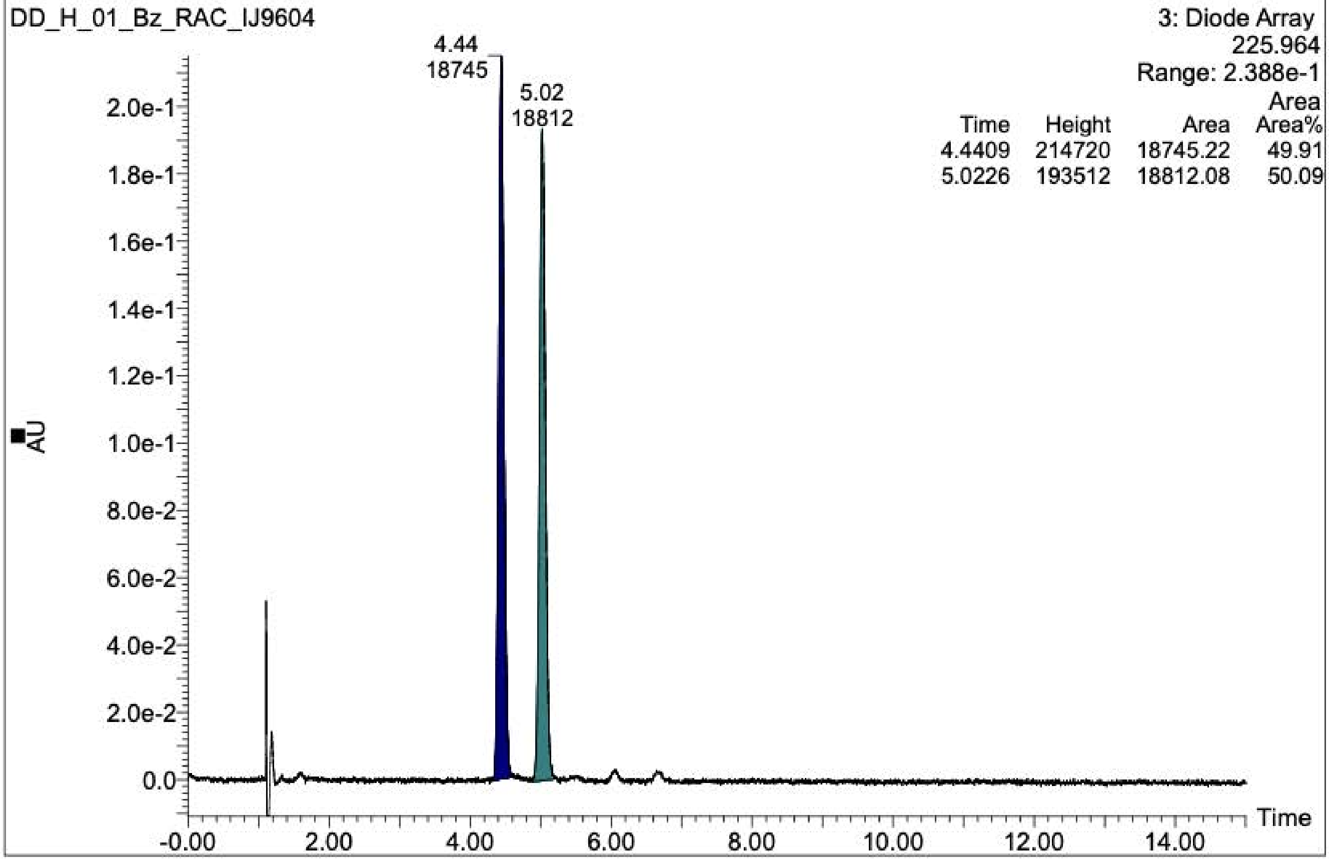

(*S*)-1-(*N*-benzoylmorpholine-4-carboxamido)butan-2-yl benzoate (1zo-Bz), (*R*)-1-(*N*-benzoylmorpholine-4-carboxamido)butan-2-yl benzoate (1zo-(*R*)-Bz)

Chiral SFC Analysis: CHIRALPAK IK (CO_2:MeOH, 95:05, 2.5 mL min^{-1}, 40 °C, 225 nm) t_R = 9.8 (major), 11.0 (minor) minutes, 97% ee; t_R = 9.7 (minor), 10.8 (major) minutes, 94% ee

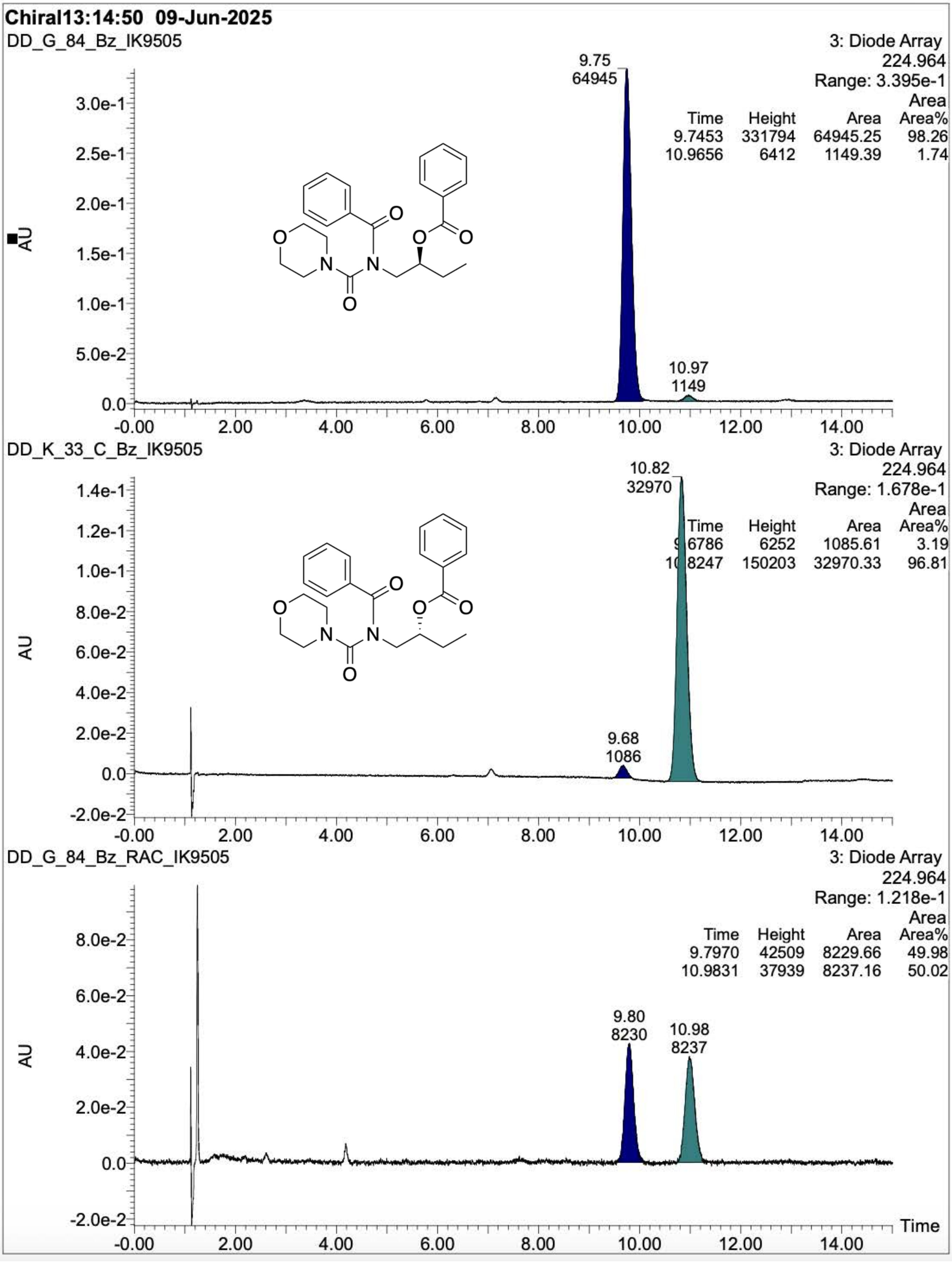

(*S*)-1-(*N*-(*tert*-butylcarbamoyl)benzamido)butan-2-yl benzoate (1zp-Bz)

Chiral SFC Analysis: CHIRALPAK IK (CO_2:MeOH, 97:03, 2.5 mL min^{-1}, 40 °C, 225 nm) t_R = 6.5 (major), 7.4 (minor) minutes, 98% ee

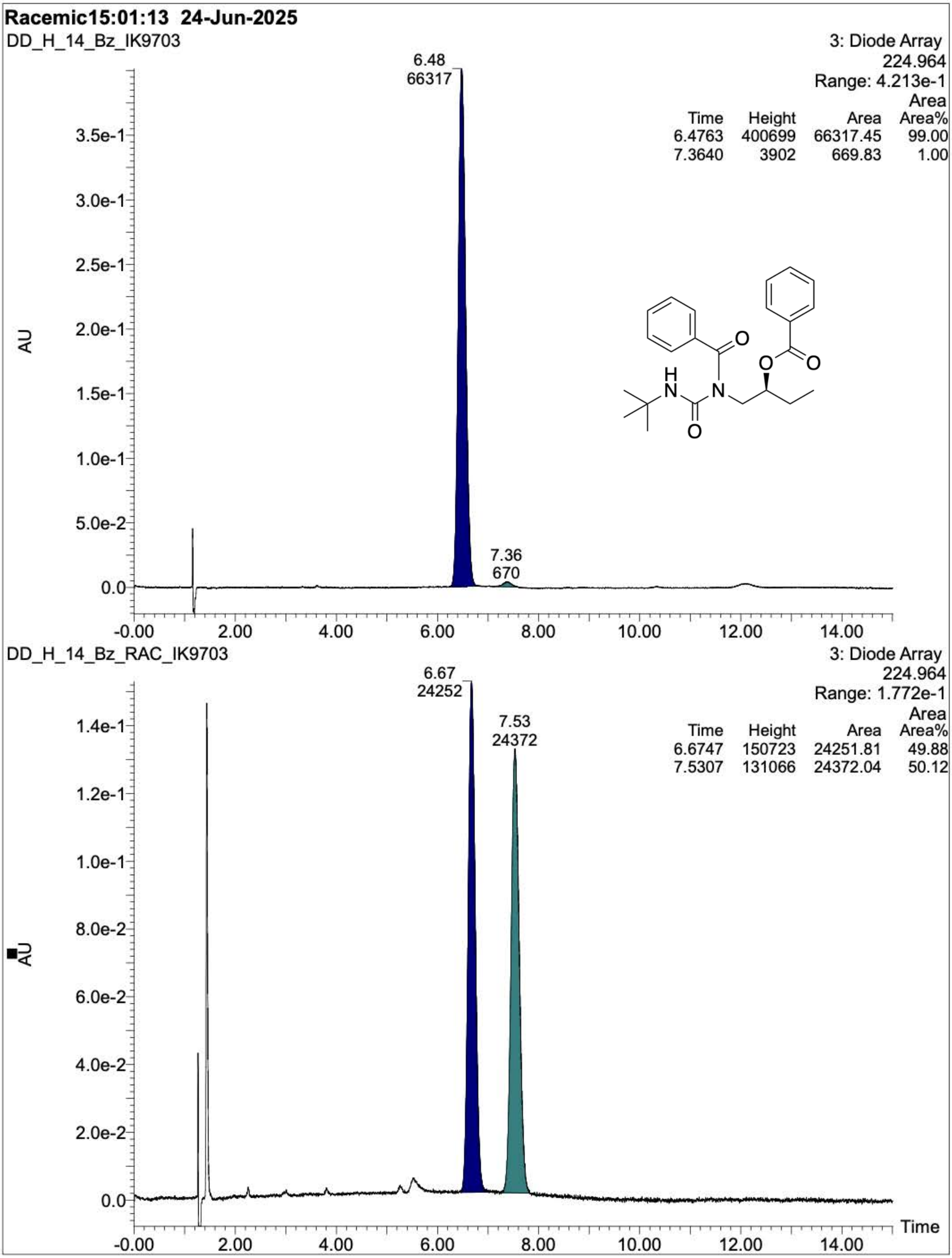

(*S*)-1-(3,5-bis(trifluoromethyl)phenyl)-3-(2-hydroxybutyl)urea (1zq)

Chiral HPLC Analysis: CHIRALPAK IH (Hexane:^{i}PrOH, 95:05, 1.25 mL min^{-1}, 40 °C, 245 nm) t_R = 7.3 (minor), 9.1 (major) minutes, 80% ee

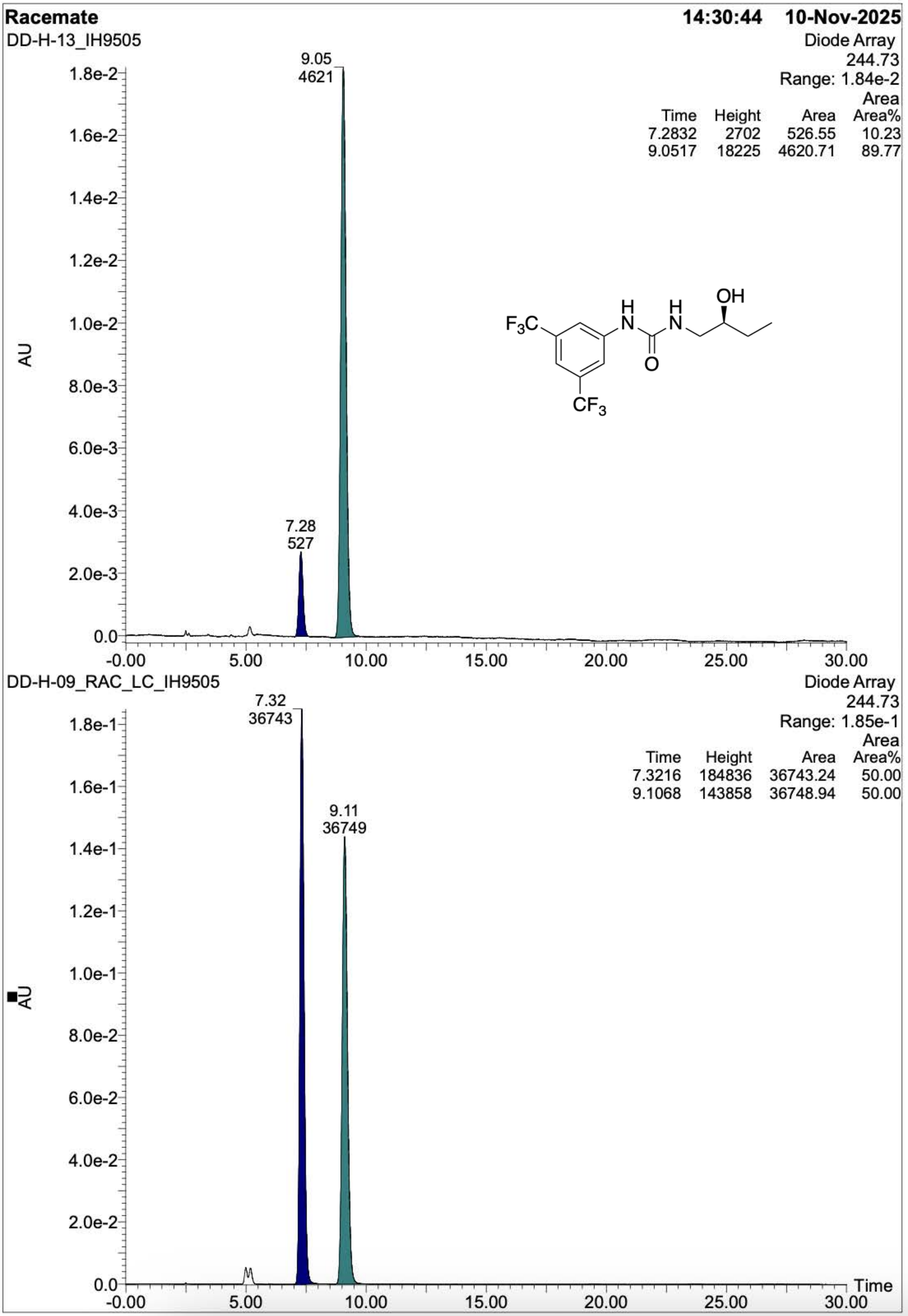

(*S*)-1-(*N*-(*tert*-butoxycarbonyl)benzamido)butan-2-yl benzoate (1zr-Bz)

Chiral SFC Analysis: CHIRALPAK IK (CO_2:MeOH, 95:05, 2.5 mL min^{-1}, 40 °C, 226 nm) t_R = 3.3 (major), 3.9 (minor) minutes, 9% ee

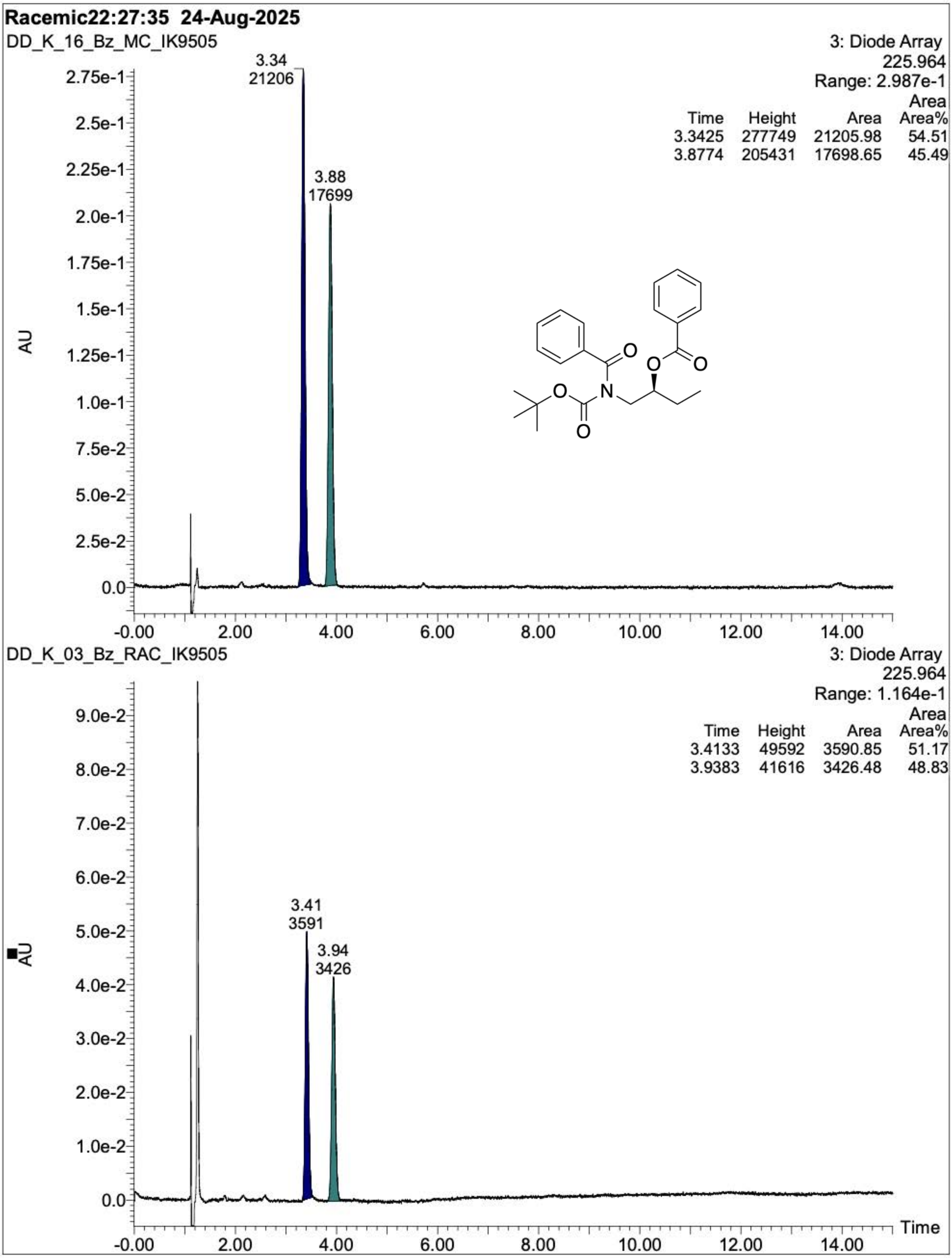

(*S*)-2-(benzyloxy)-*N*-(2-hydroxybutyl)acetamide (1zs)

Chiral SFC Analysis: CHIRALPAK IJ (CO_2:MeOH, 95:05, 2.5 mL min^{-1}, 40 °C, 205 nm) t_R = 4.0 (minor), 4.6 (major) minutes, 39% ee

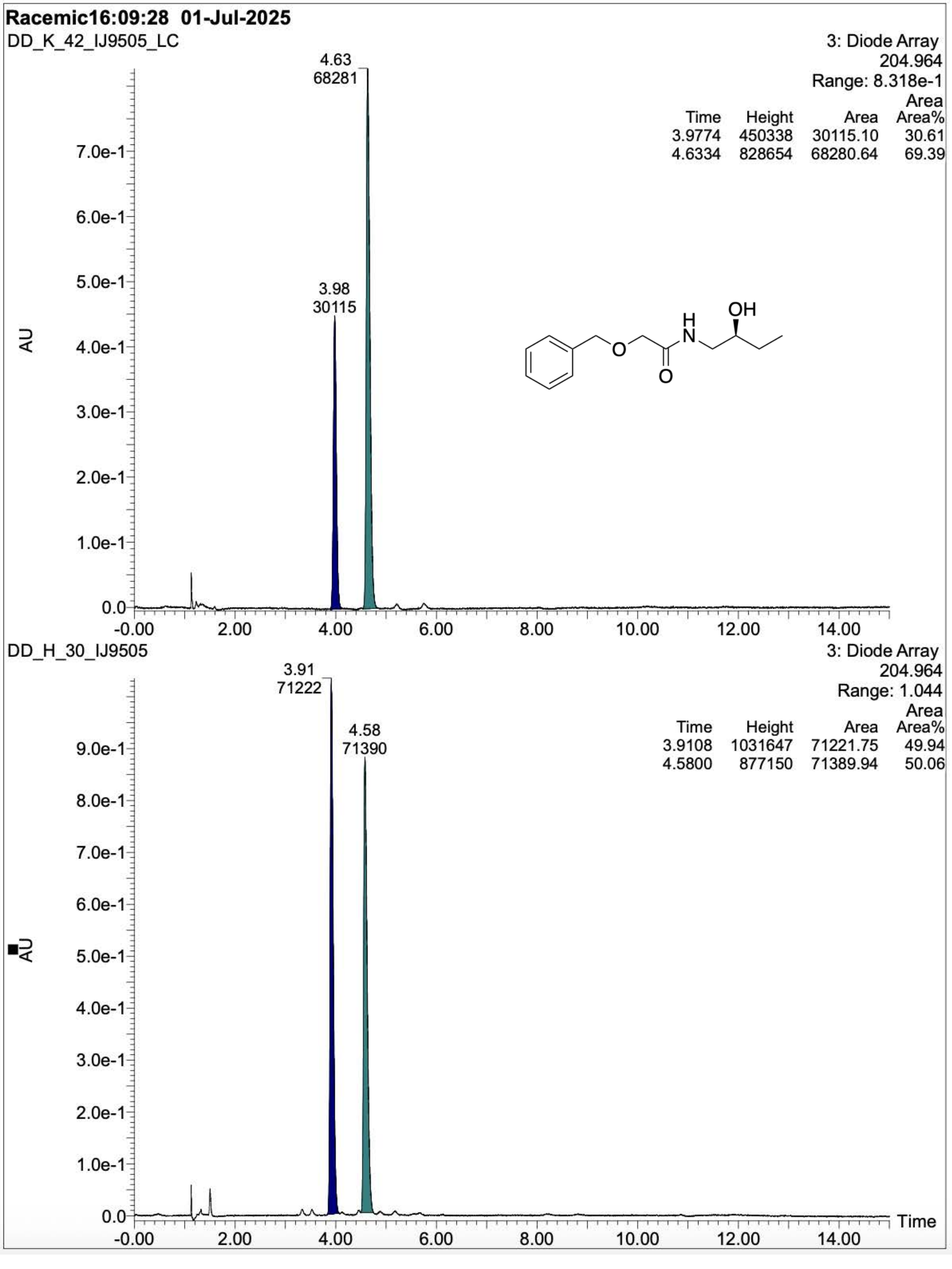

(*S*)-*N*-(2-hydroxybutyl)-2-(methylthio)acetamide (1zt)

Chiral SFC Analysis: CHIRALPAK IJ (CO_2:MeOH, 99:01, 2.5 mL min^{-1}, 40 °C, 212 nm) t_R = 7.5 (minor), 8.0 (major) minutes, 9% ee

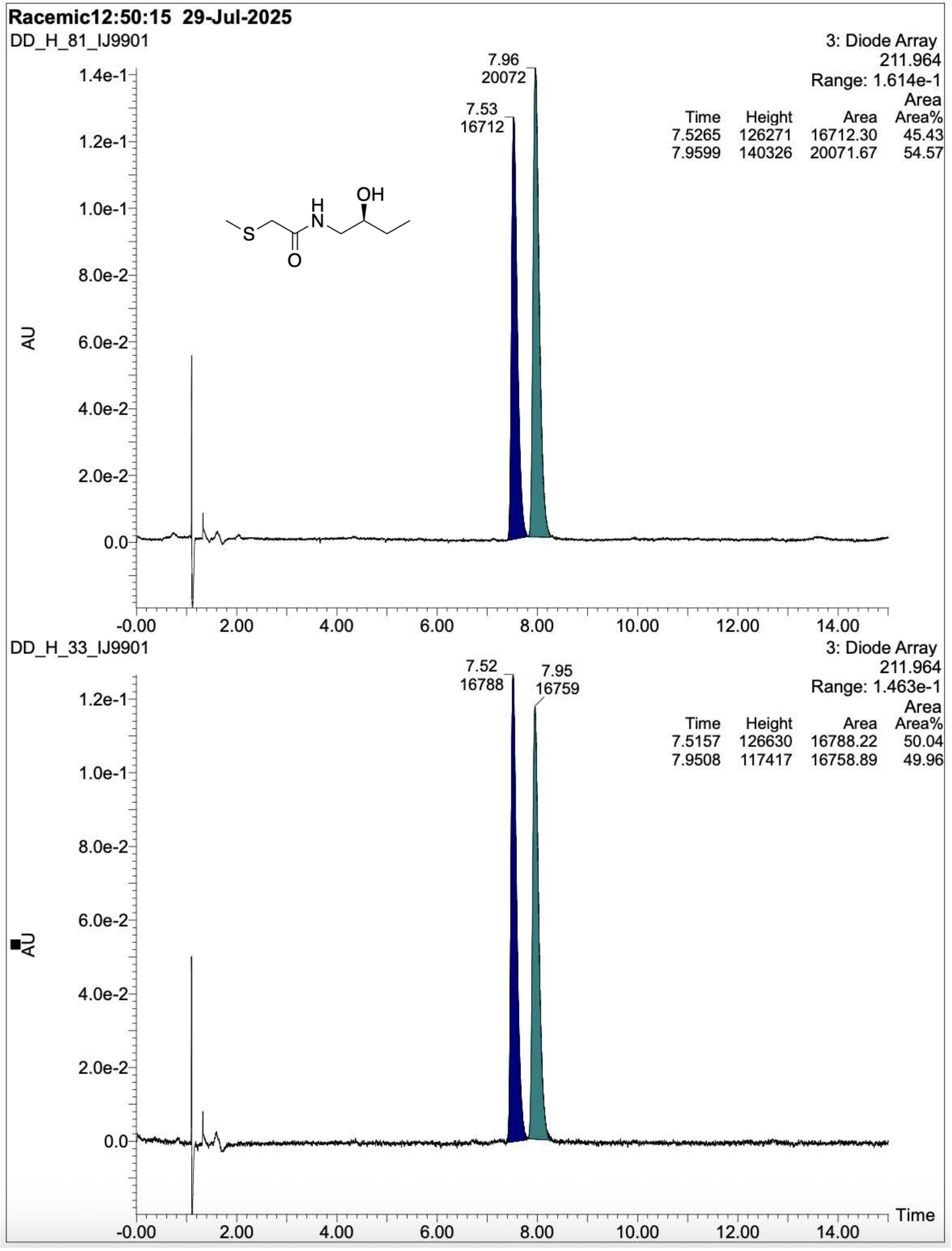

(*S*)-*N*-(5-ethoxy-2-hydroxypentyl)-2-(thiazol-4-yl)acetamide (1zu)

Chiral SFC Analysis: CHIRALPAK IA (CO_2:MeOH, 90:10, 2.5 mL min^{-1}, 40 °C, 239 nm) t_R = 8.6 (minor), 9.0 (major) minutes, 97% ee

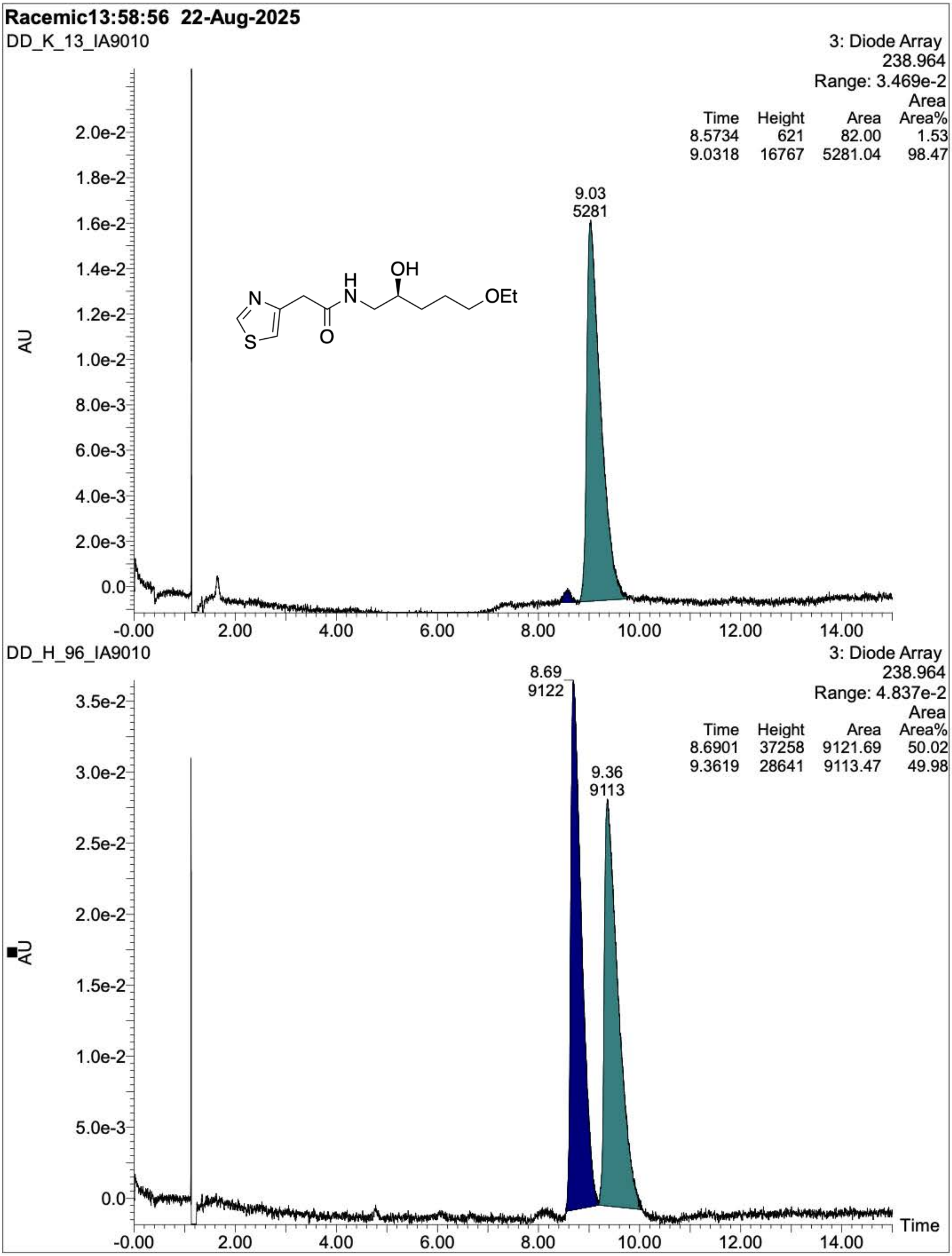

(*S*)-*N*-(2-hydroxy-3-phenylpropyl)morpholine-4-carboxamide (1zv)

Chiral SFC Analysis: CHIRALPAK IJ (CO_2:MeOH, 95:05, 2.5 mL min^{-1}, 40 °C, 202 nm) t_R = 8.4 (minor), 10.1 (major) minutes, 97% ee

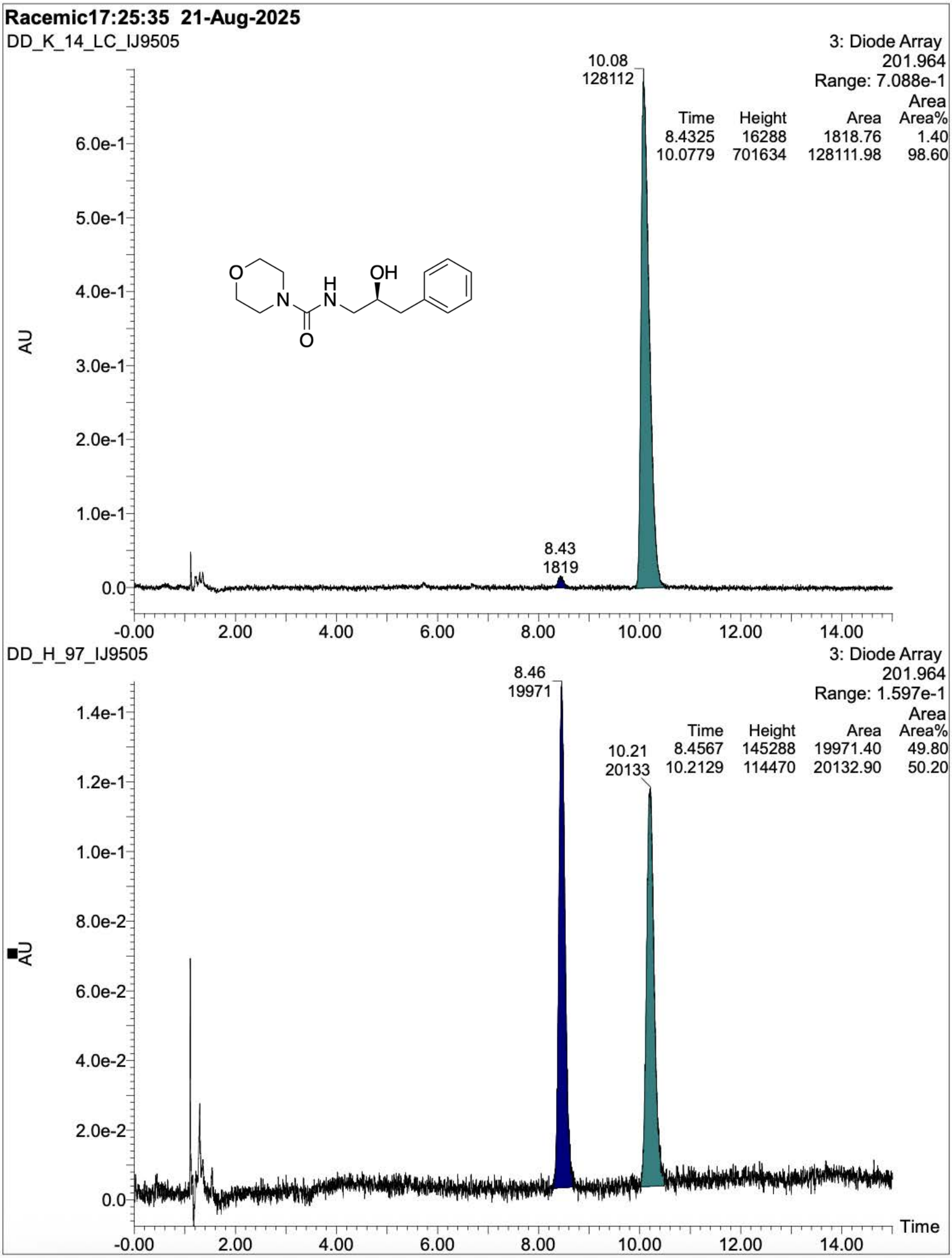

***tert*-butyl ((*S*)-1-(((*S*)-2-hydroxybutyl)amino)-1-oxo-3-phenylpropan-2-yl)carbamate (2a)**

Chiral SFC Analysis: CHIRALPAK IA (CO_2:MeOH, 95:05, 2.5 mL min^{-1}, 40 °C, 204 nm) t_R = 9.6 (major), 11.0 (minor) minutes, 10.6 : 1.0 dr

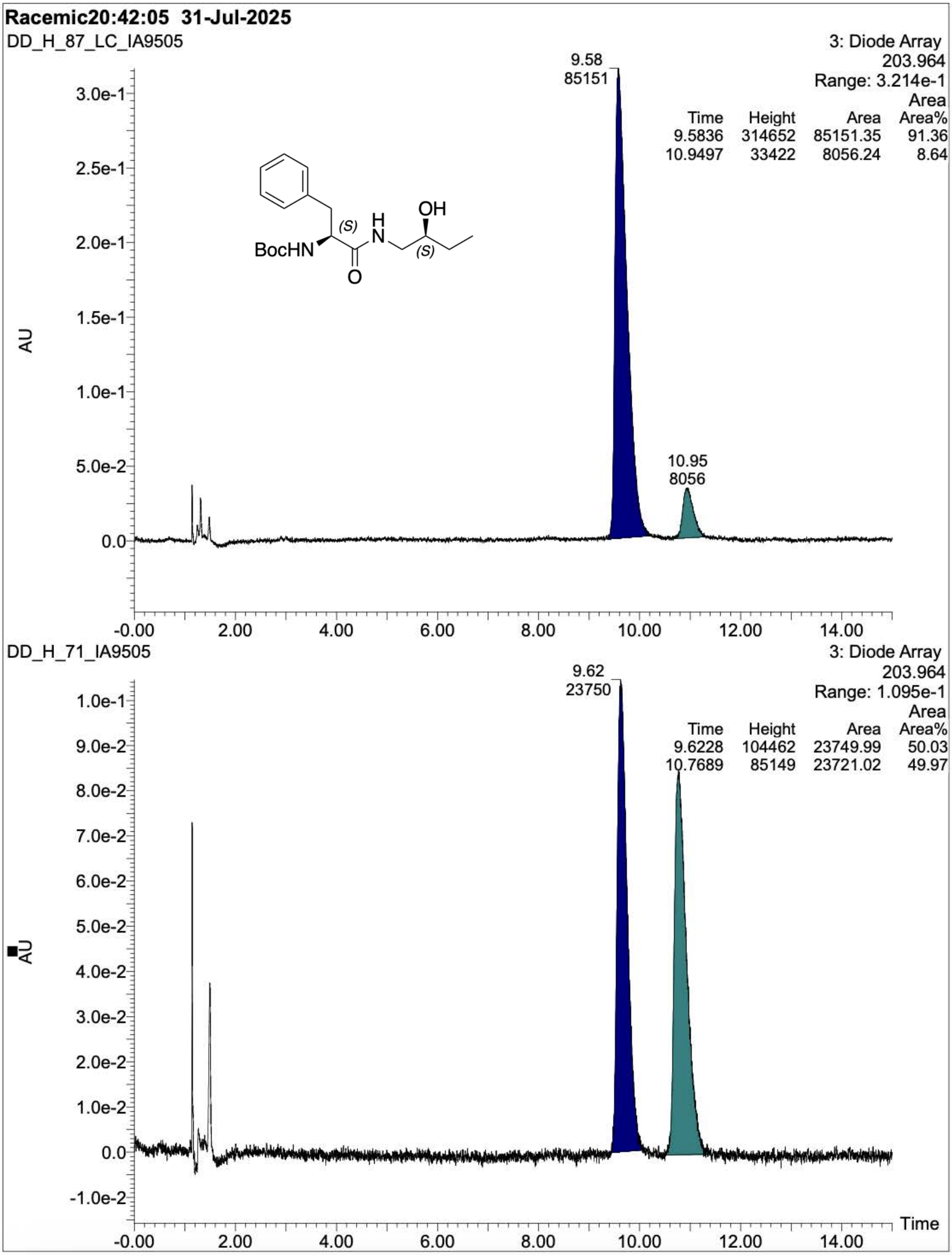

***tert*-butyl ((*R*)-1-(((*S*)-2-hydroxybutyl)amino)-1-oxo-3-phenylpropan-2-yl)carbamate (2b)**

Chiral SFC Analysis: CHIRALPAK IJ (CO_2:MeOH, 97:03, 2.5 mL min^{-1}, 40 °C, 216 nm) t_R = 4.6 (minor), 5.0 (major) minutes, 39.3 : 1.0 dr

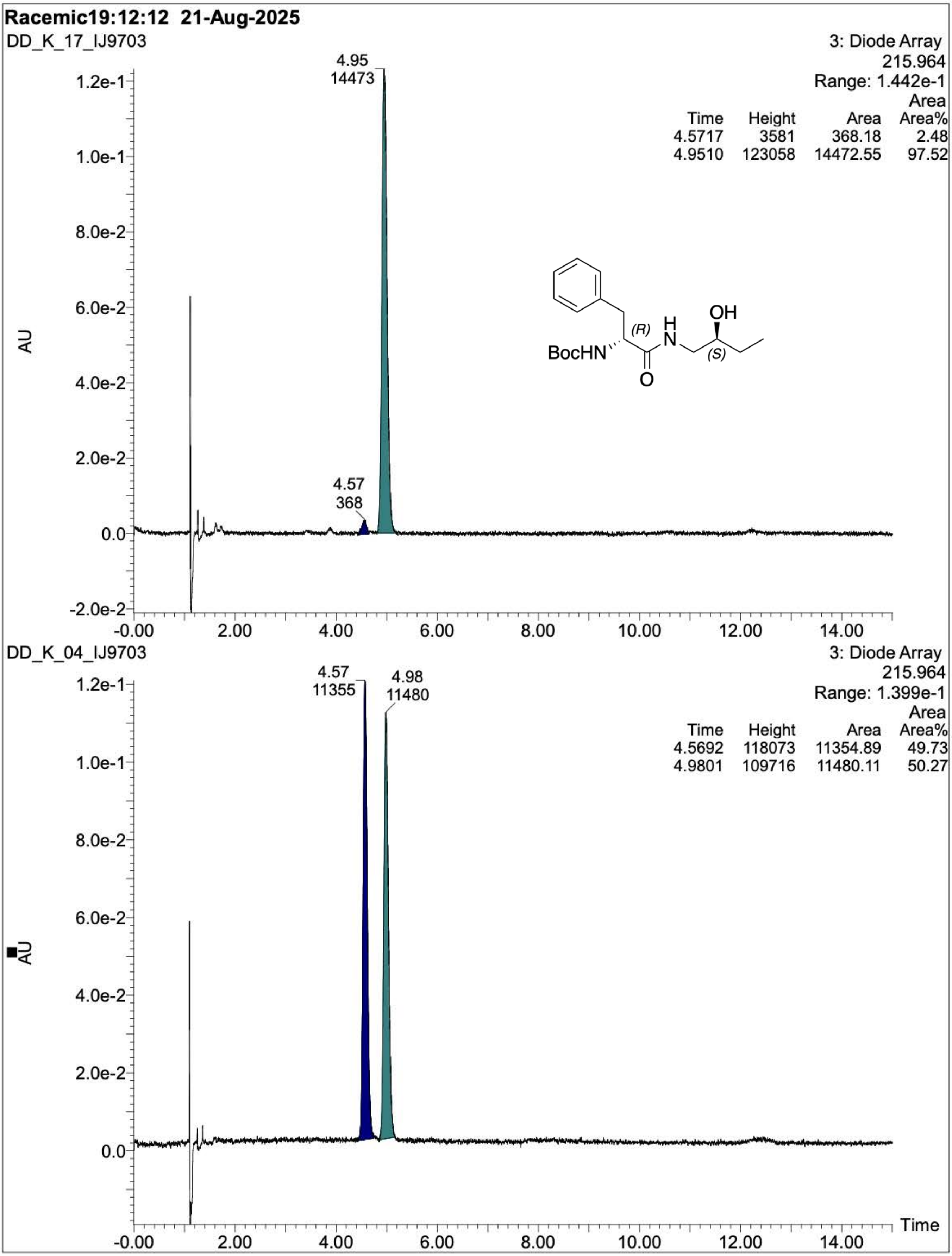

N-((S)-2-hydroxybutyl)-2-(4-isobutylphenyl)propenamide (2c)

Chiral HPLC Analysis: CHIRALPAK IH (Hexane:^{i}PrOH, 99:01, 1.25 mL min^{-1}, 40 °C, 220 nm) t_R = 68.5 (minor), 70.3 (minor), 74.9 (major), 82.3 (major) minutes, 97%, 97% ee

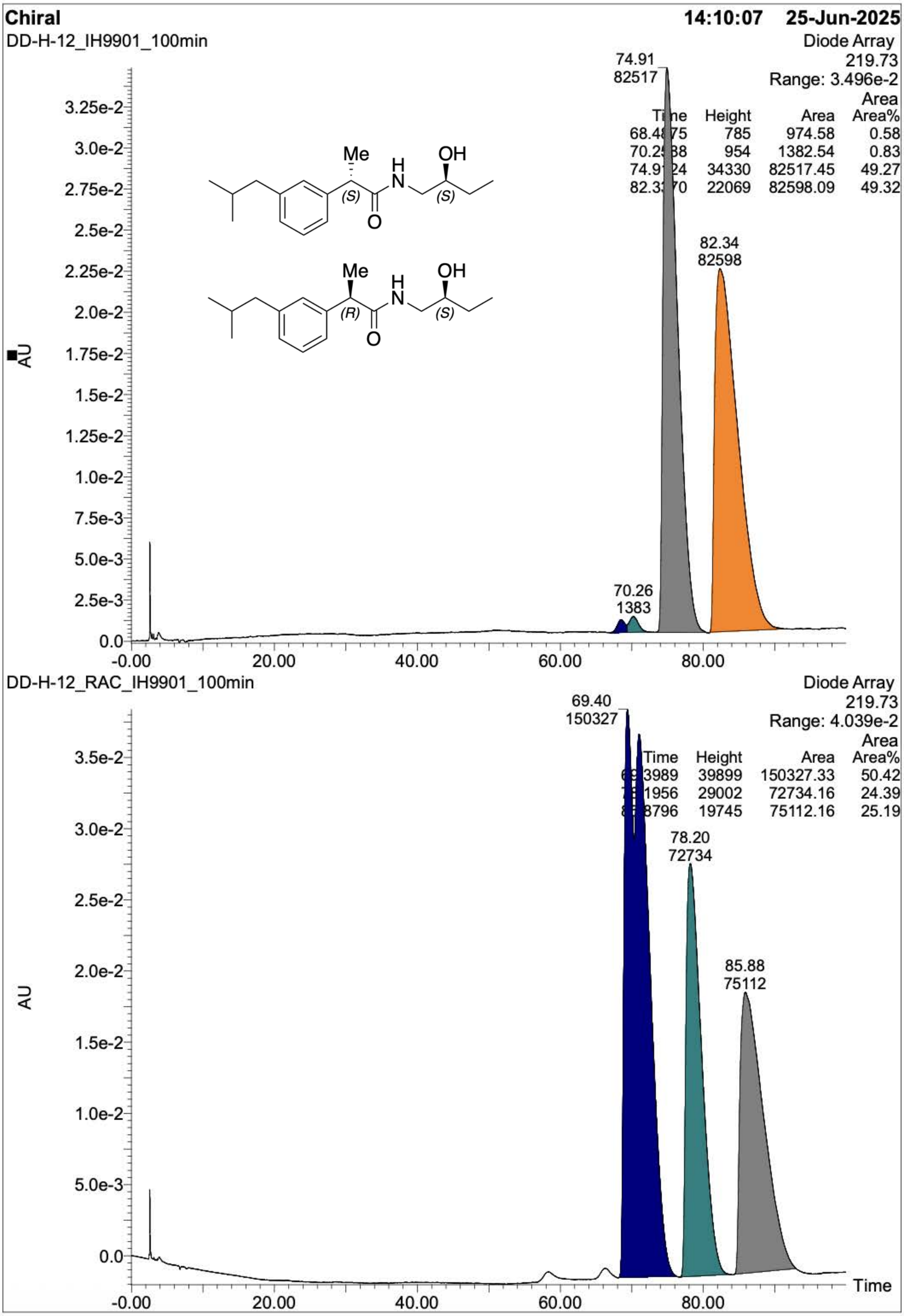

(*S*)-1-(*N*-acetylbenzamido)pentan-3-yl benzoate (6-Bz)

Chiral SFC Analysis: CHIRALPAK IE (CO_2:MeOH, 90:10, 2.5 mL min^{-1}, 40 °C, 227 nm) t_R = 7.2 (minor), 8.0 (major) minutes, 28% ee

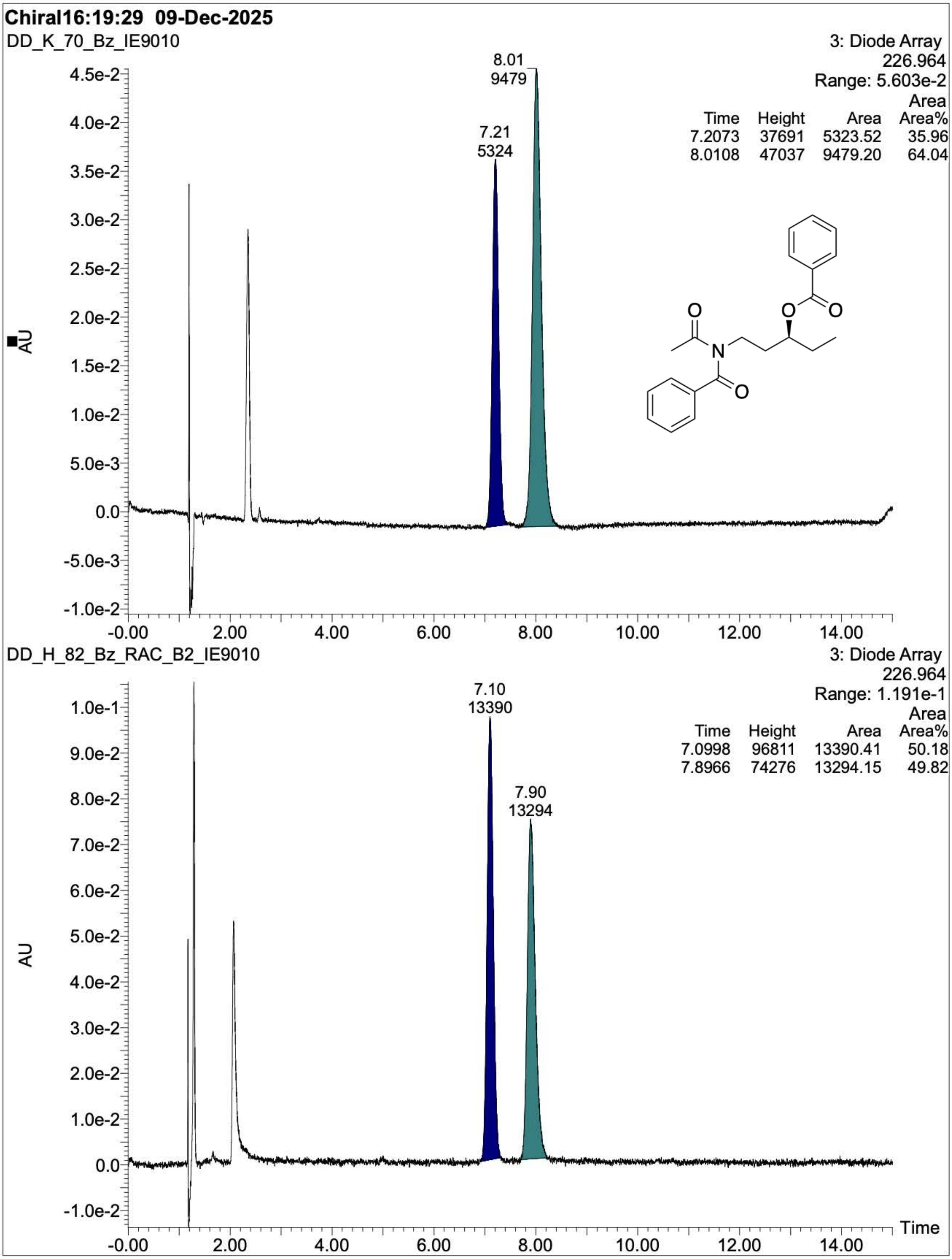

N-(2-hydroxybutyl)acetamide (1a-rac)

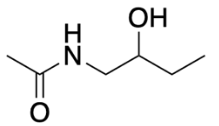

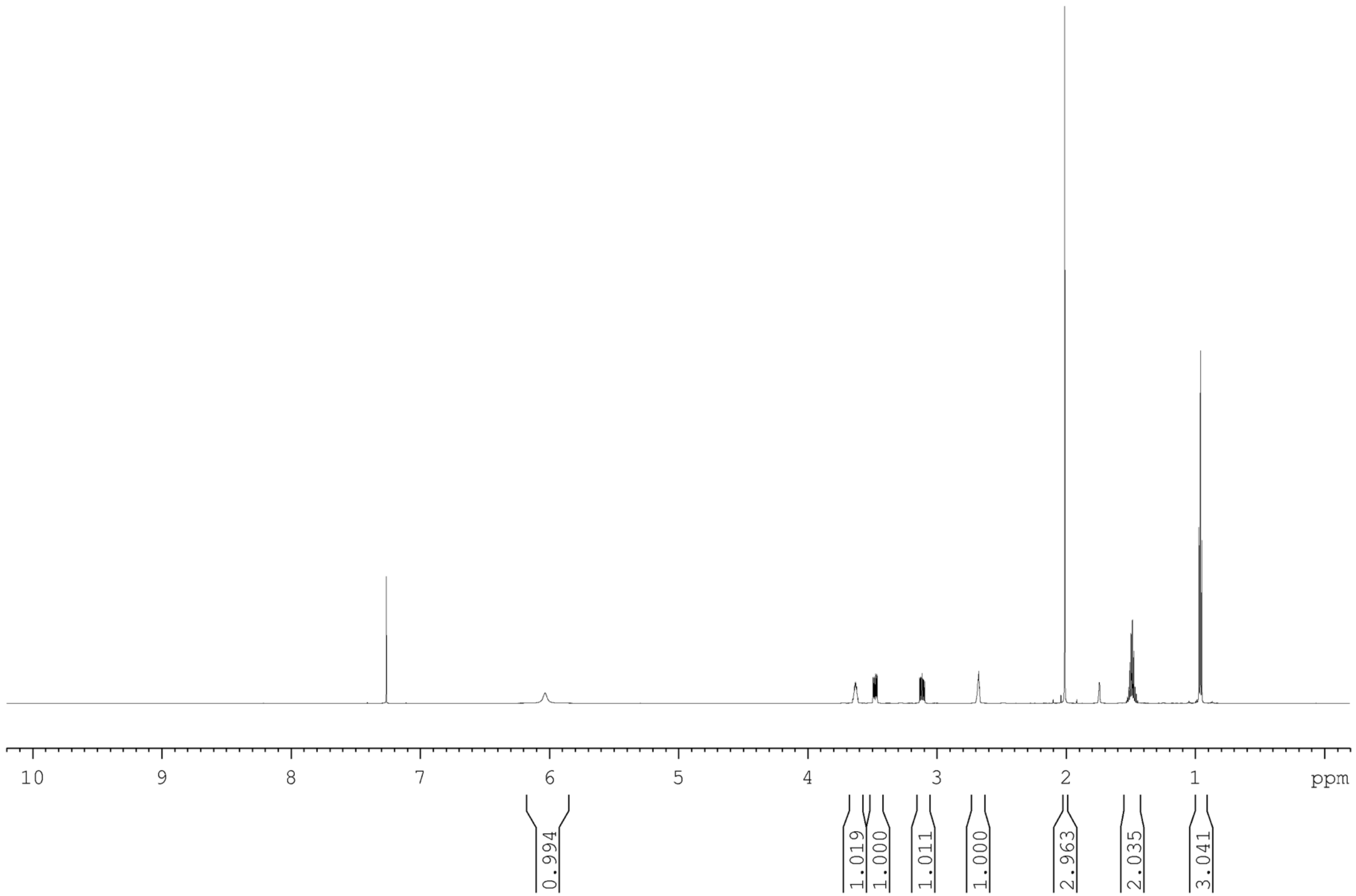

N-(2-hydroxybutyl)acetamide (1a-rac)

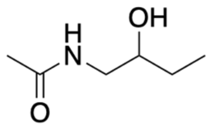

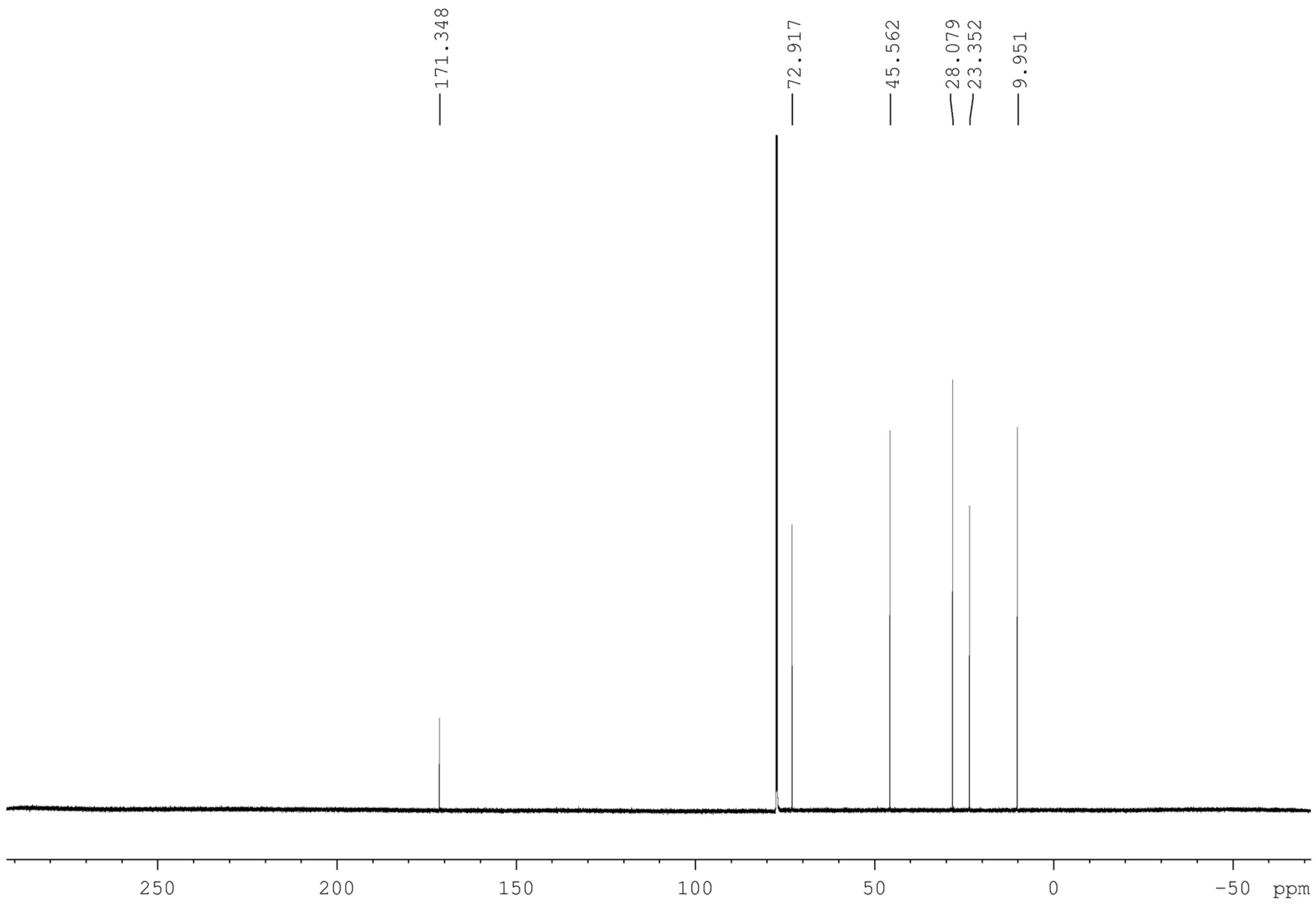

(*S*)-*N*-(2-hydroxybutyl)acetamide (1a)

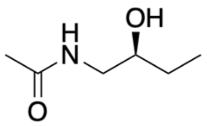

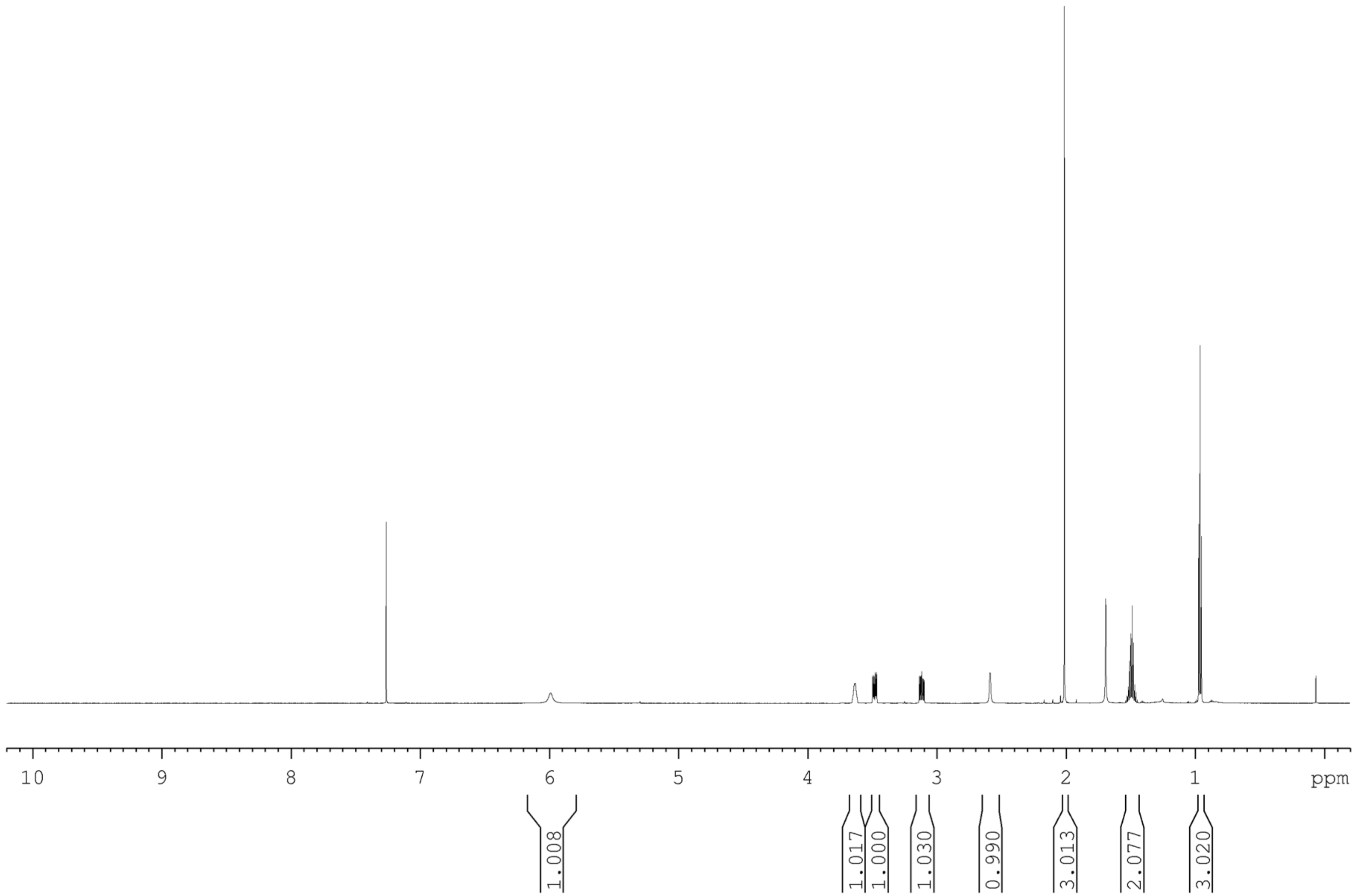

(*S*)-*N*-(2-hydroxybutyl)acetamide (1a)

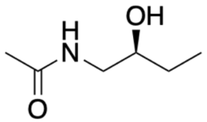

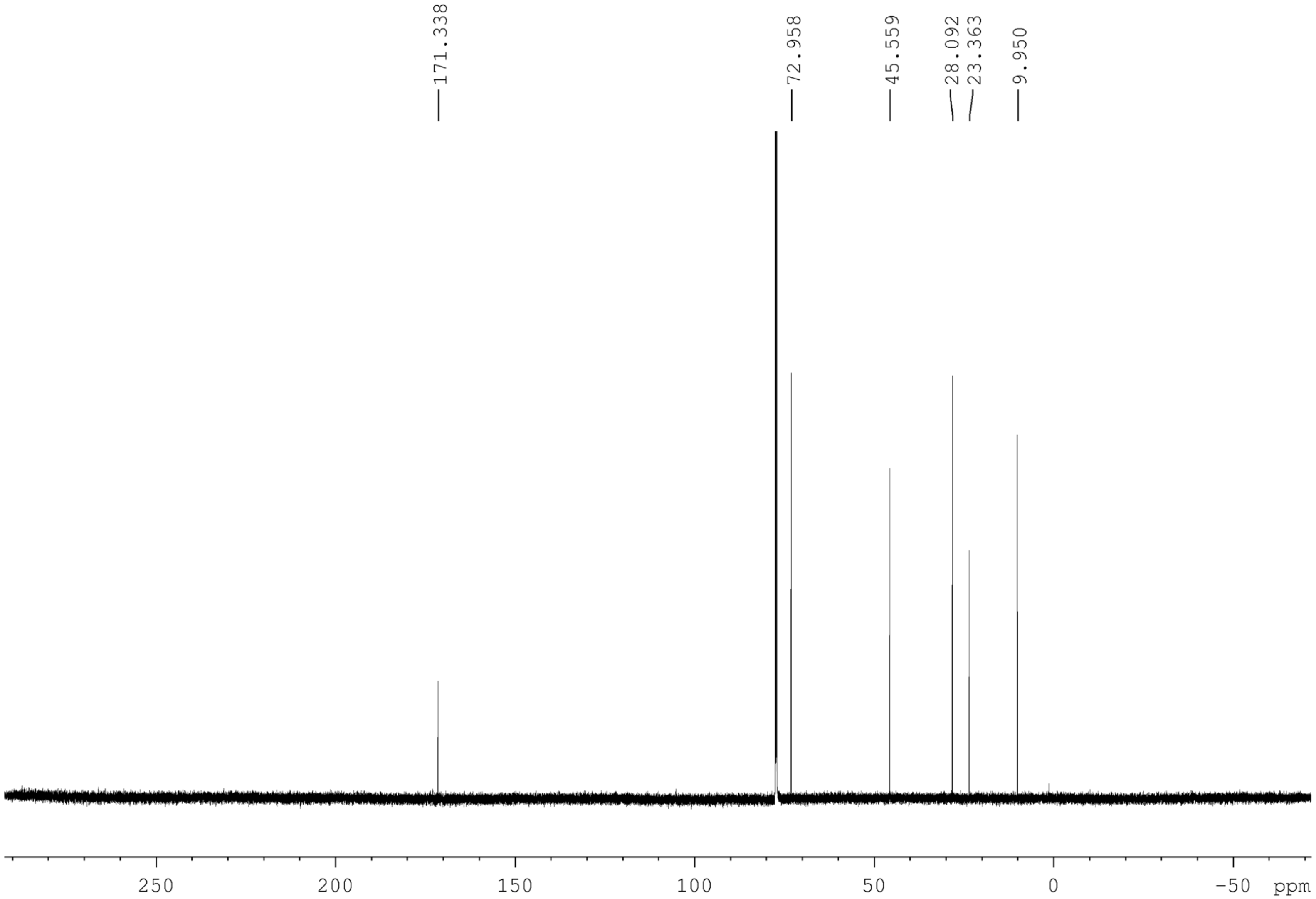

(*S*)-1-(*N*-acetylbenzamido)butan-2-yl benzoate (1a-Bz)

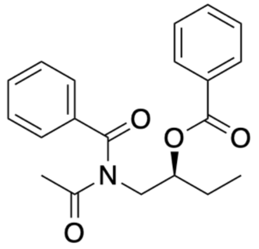

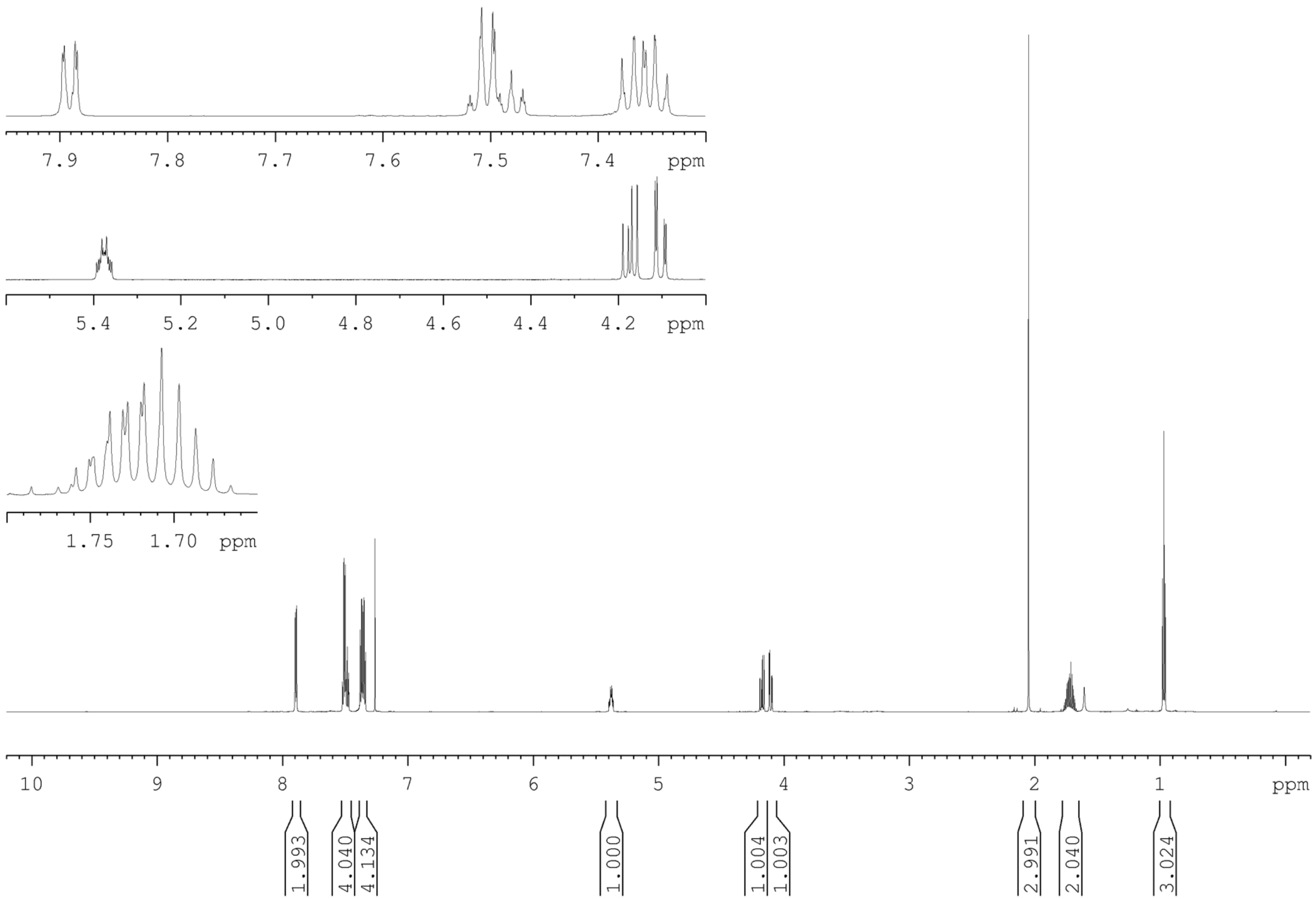

(*S*)-1-(*N*-acetylbenzamido)butan-2-yl benzoate (1a-Bz)

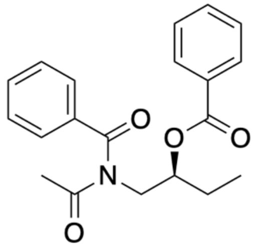

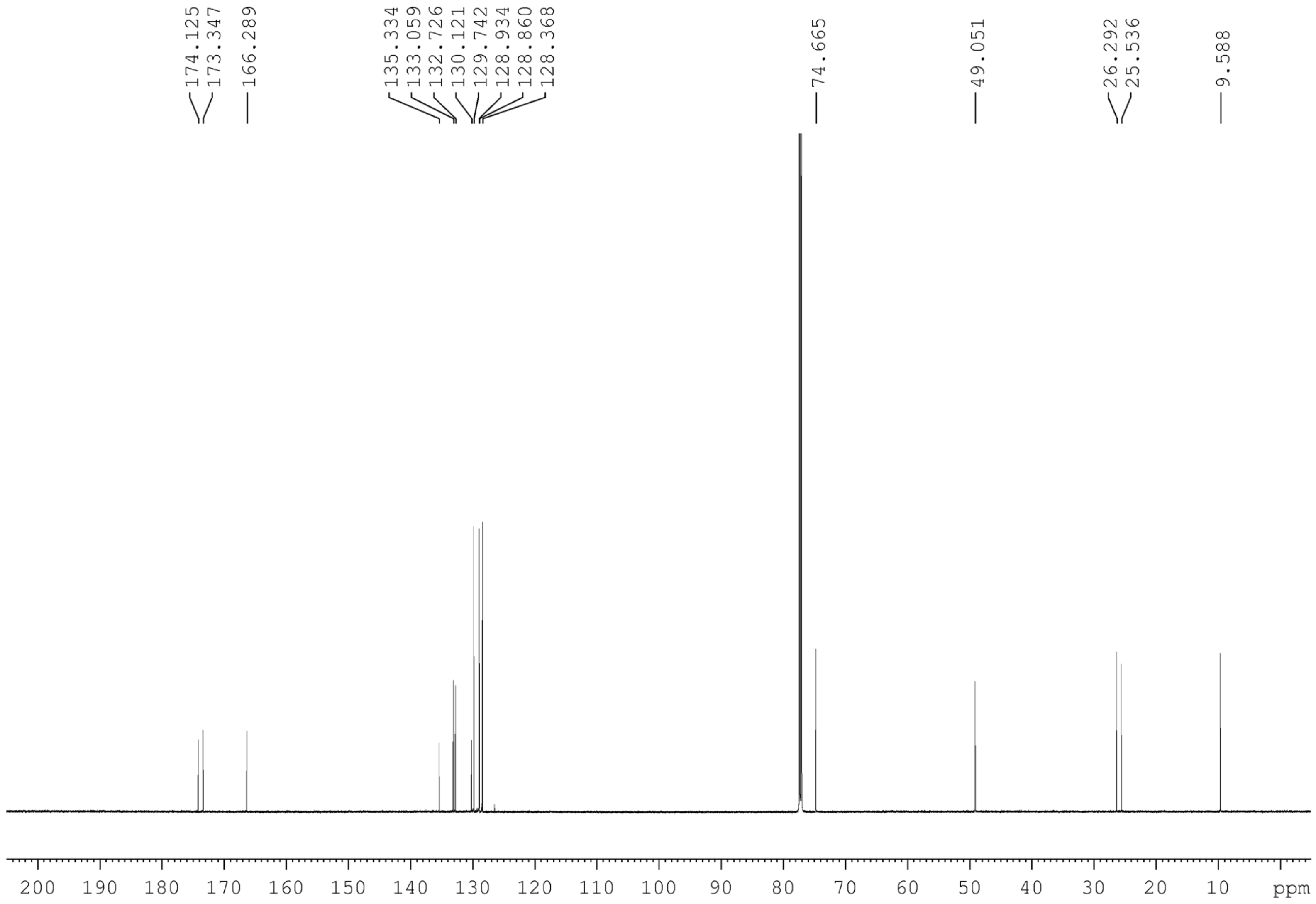

(*R*)-*N*-(2-hydroxybutyl)acetamide ((*R*)-1a)

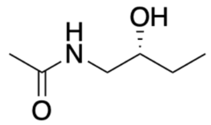

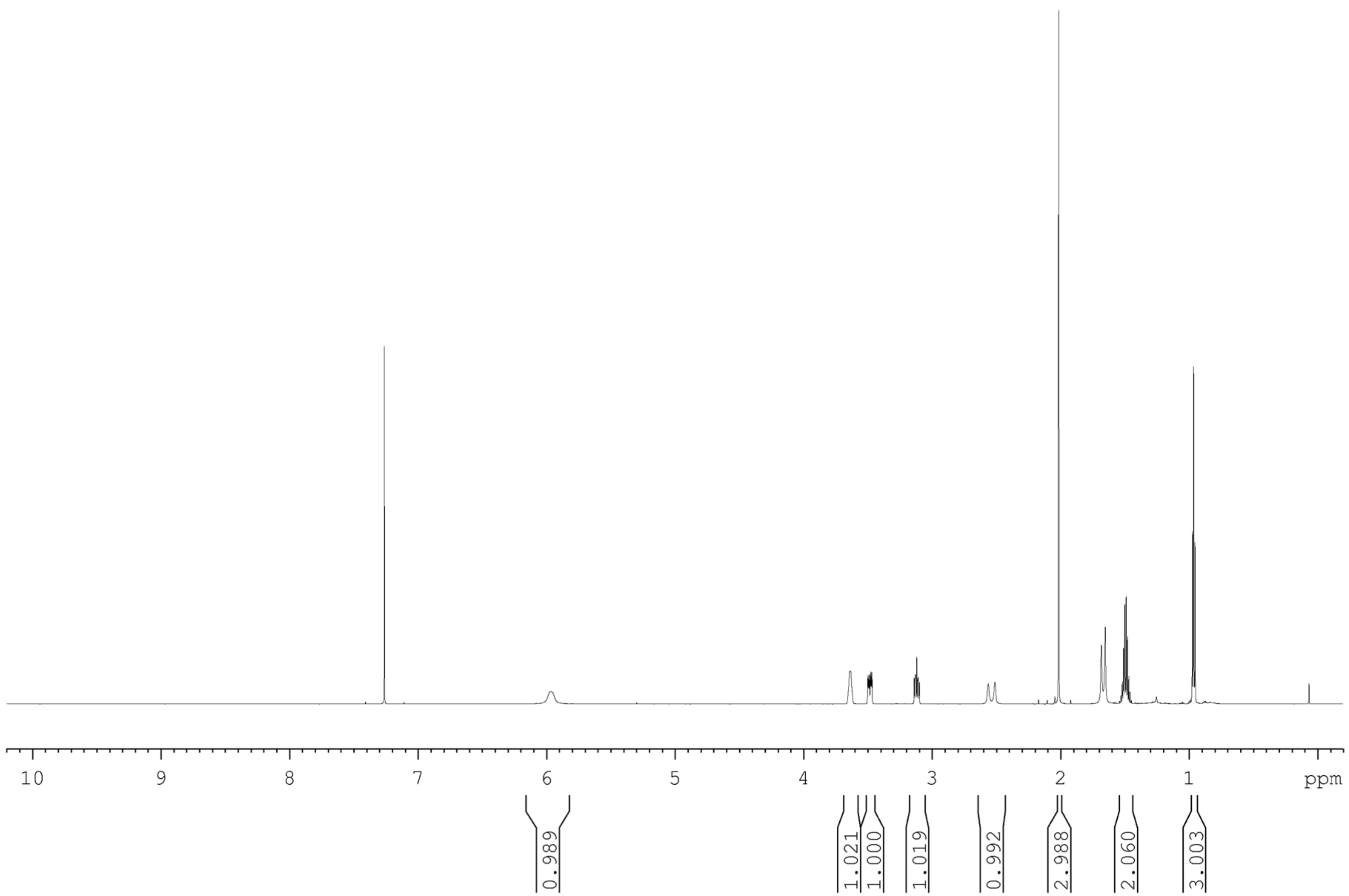

(*R*)-*N*-(2-hydroxybutyl)acetamide ((*R*)-1a)

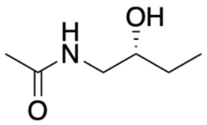

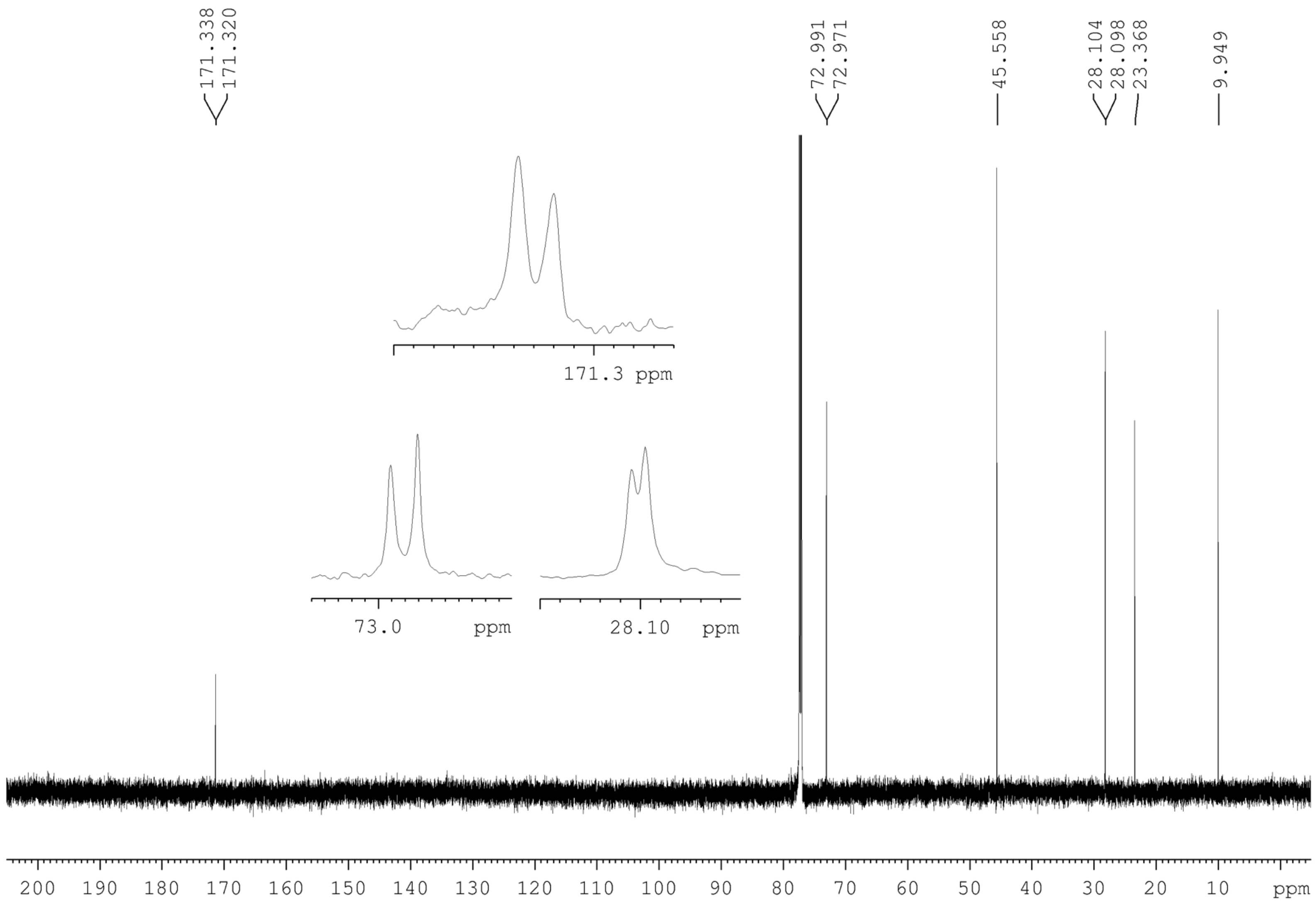

(*R*)-1-(*N*-acetylbenzamido)butan-2-yl benzoate ((*R*)-1a-Bz)

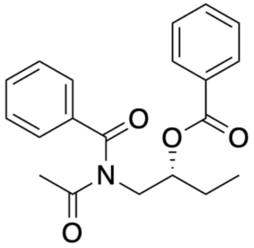

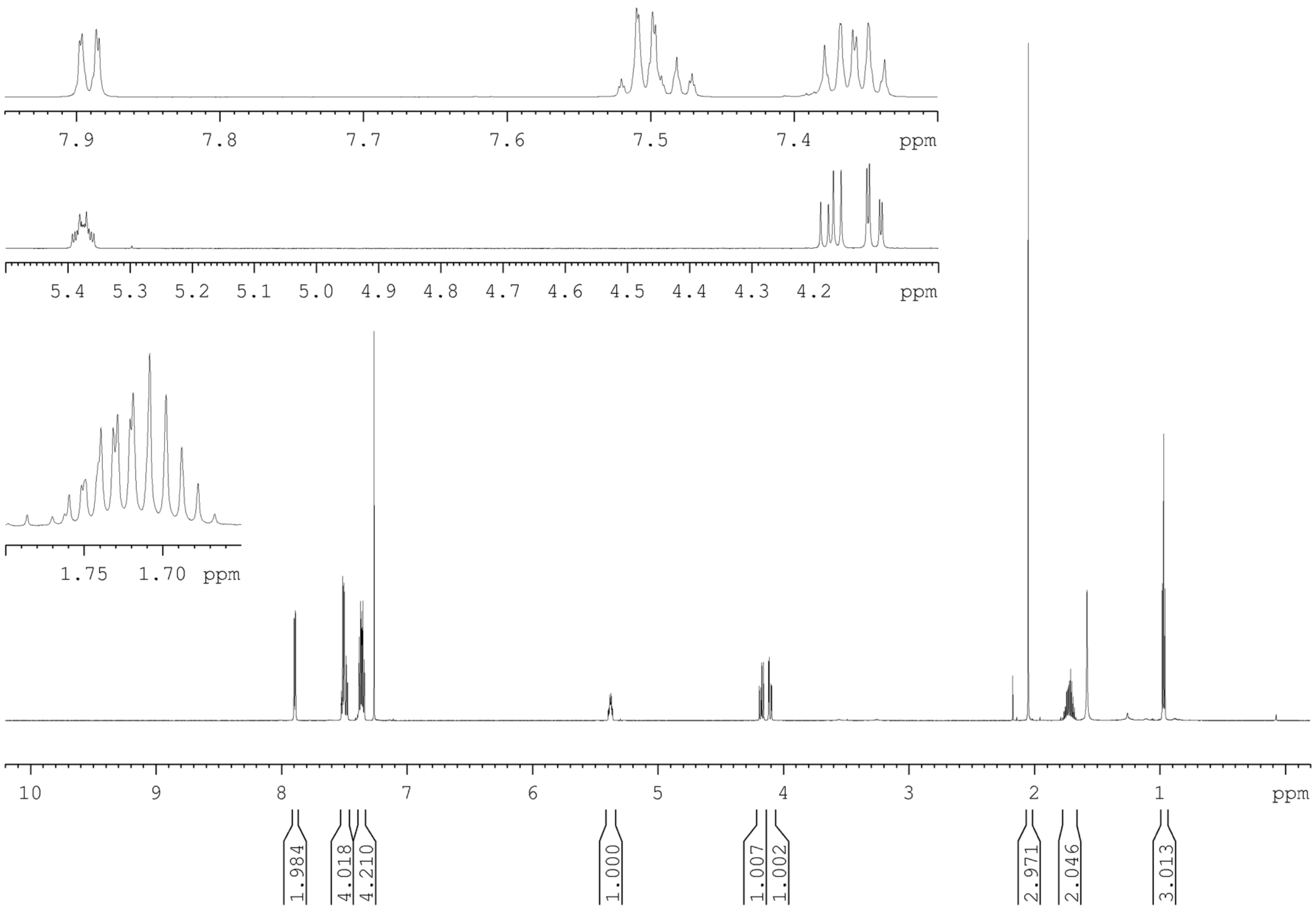

(*R*)-1-(*N*-acetylbenzamido)butan-2-yl benzoate ((*R*)-1a-Bz)

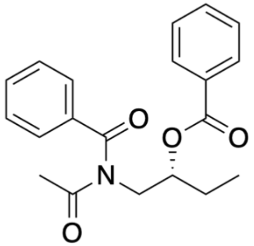

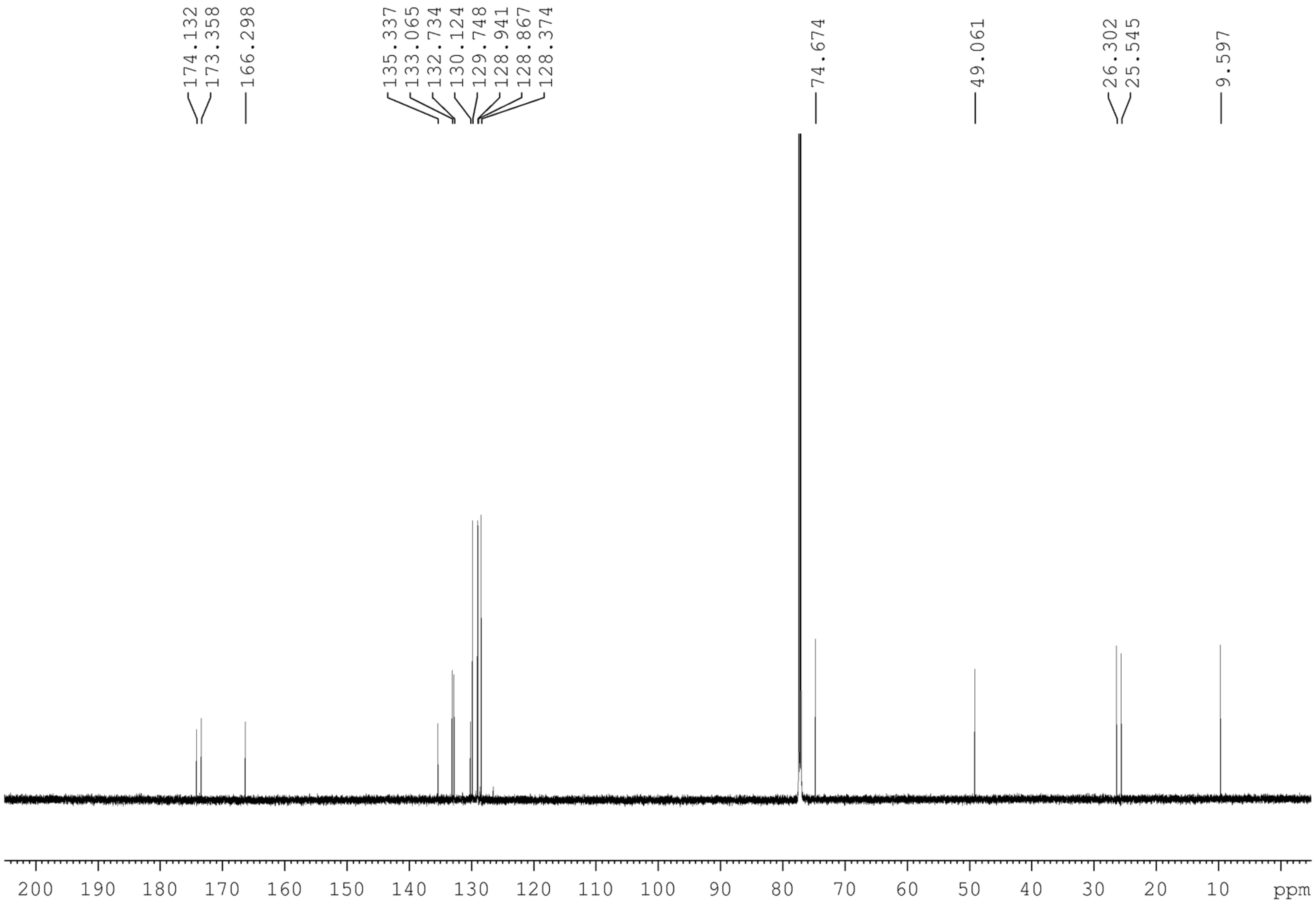

N-(2-hydroxybutyl)-*N*-methylacetamide (*N*-Me-1a-rac)

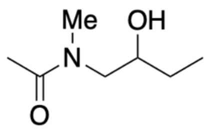

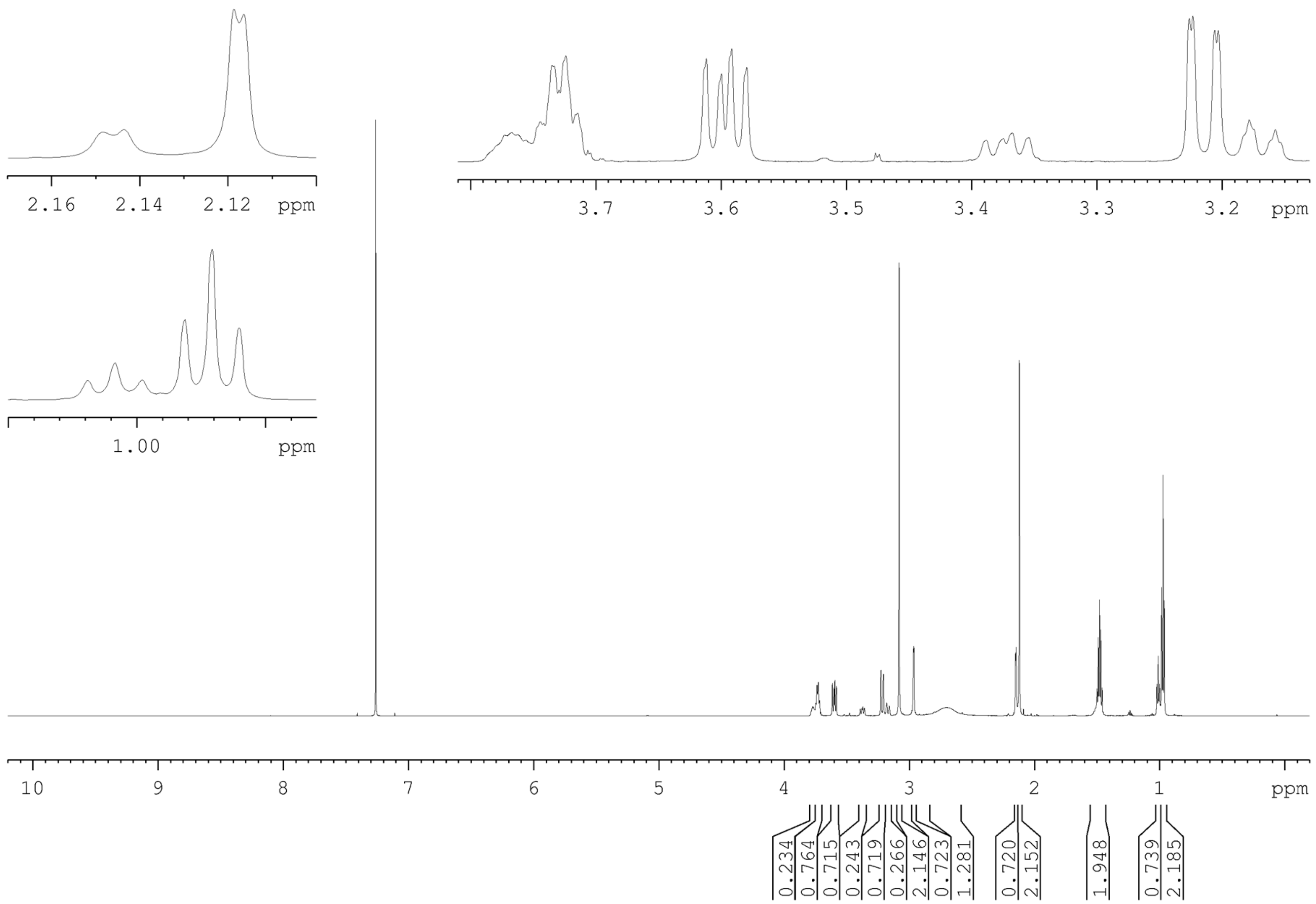

N-(2-hydroxybutyl)-*N*-methylacetamide (*N*-Me-1a-rac)

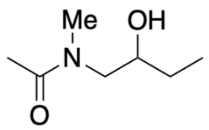

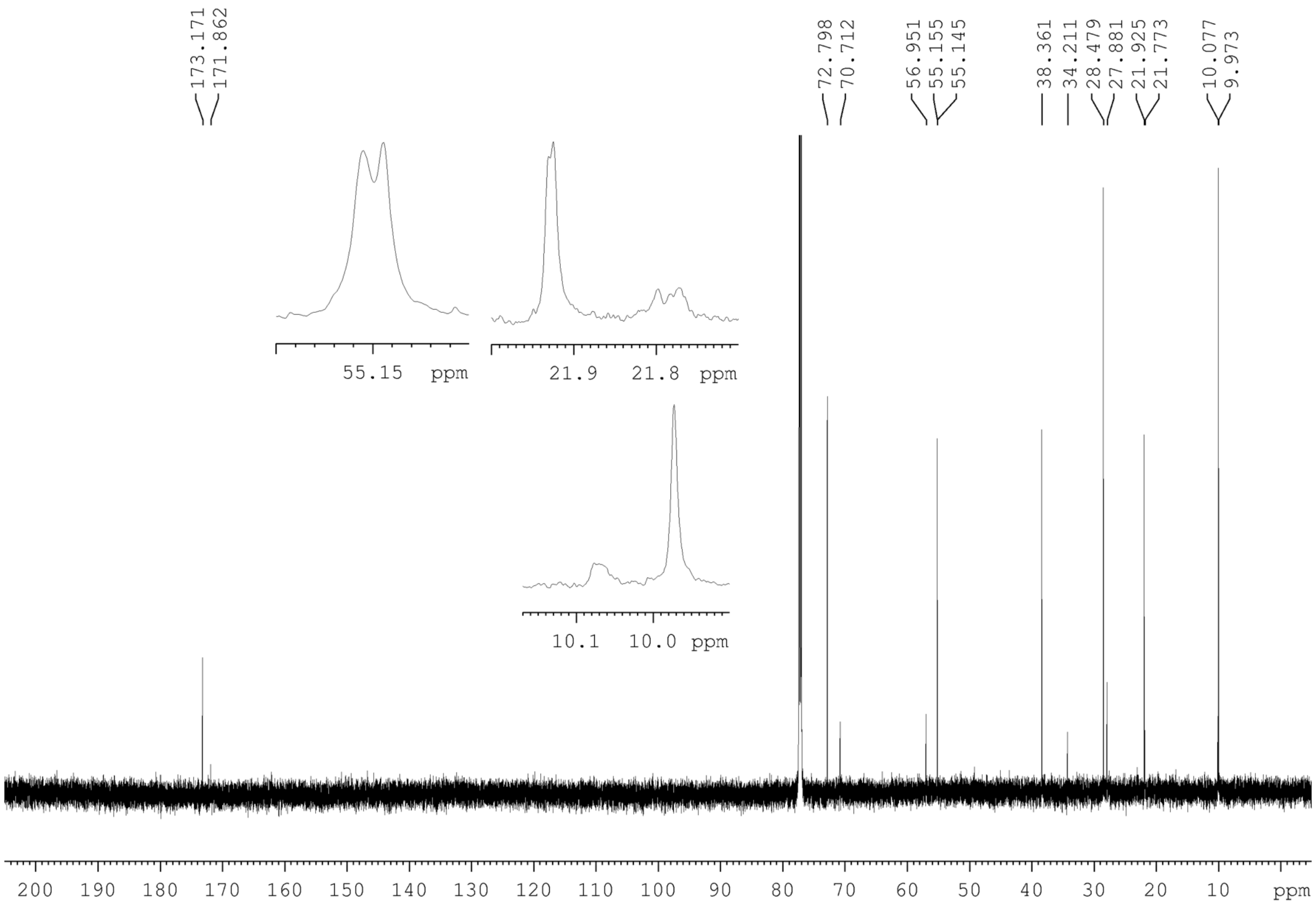

(*S*)-*N*-(2-hydroxybutyl)-*N*-methylacetamide (*N*-Me-1a)

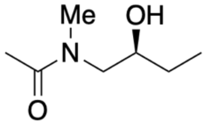

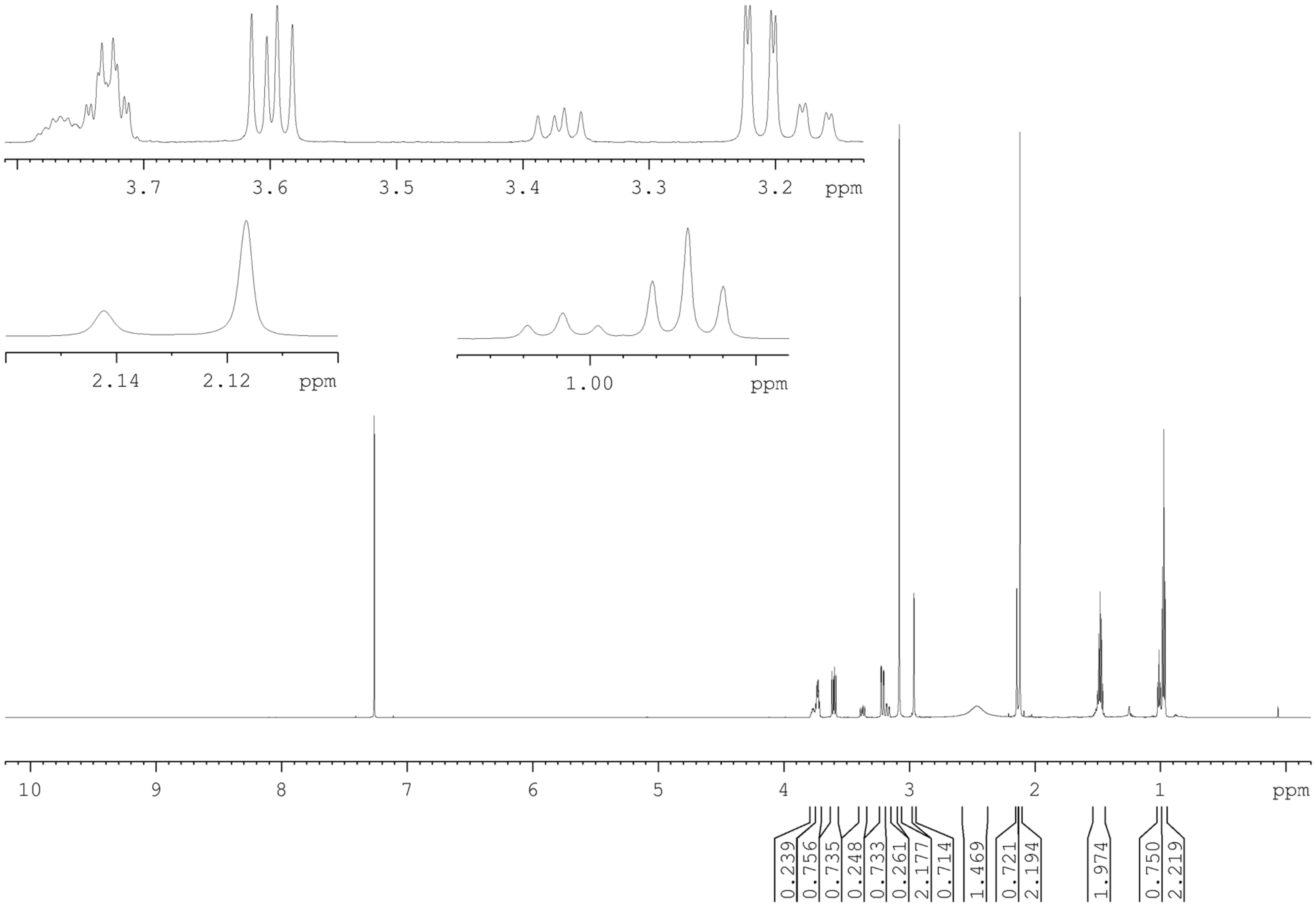

(*S*)-*N*-(2-hydroxybutyl)-*N*-methylacetamide (*N*-Me-1a)

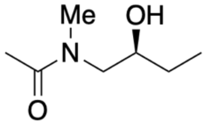

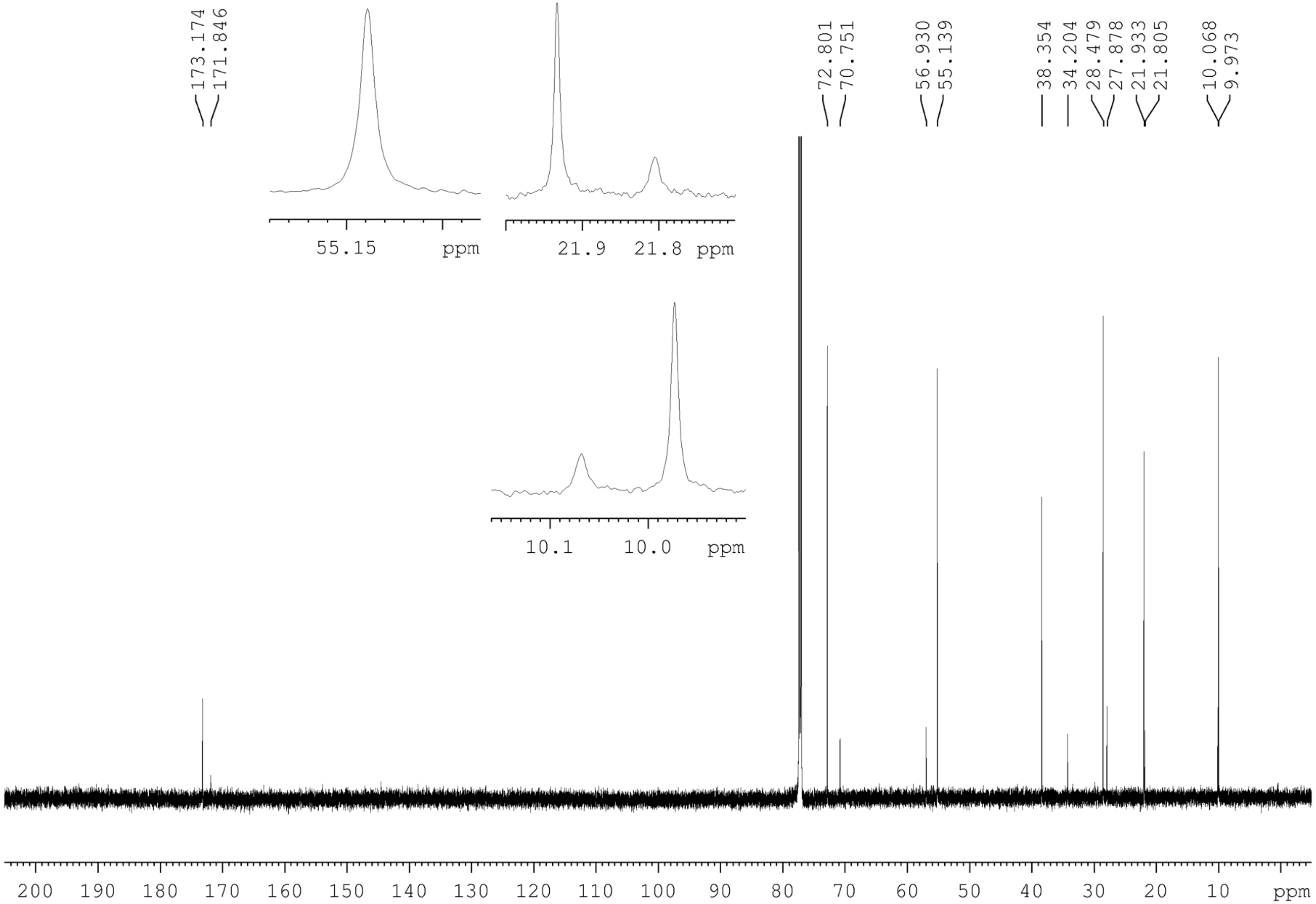

N-(2-hydroxypropyl)acetamide (1b-rac)

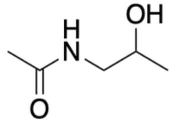

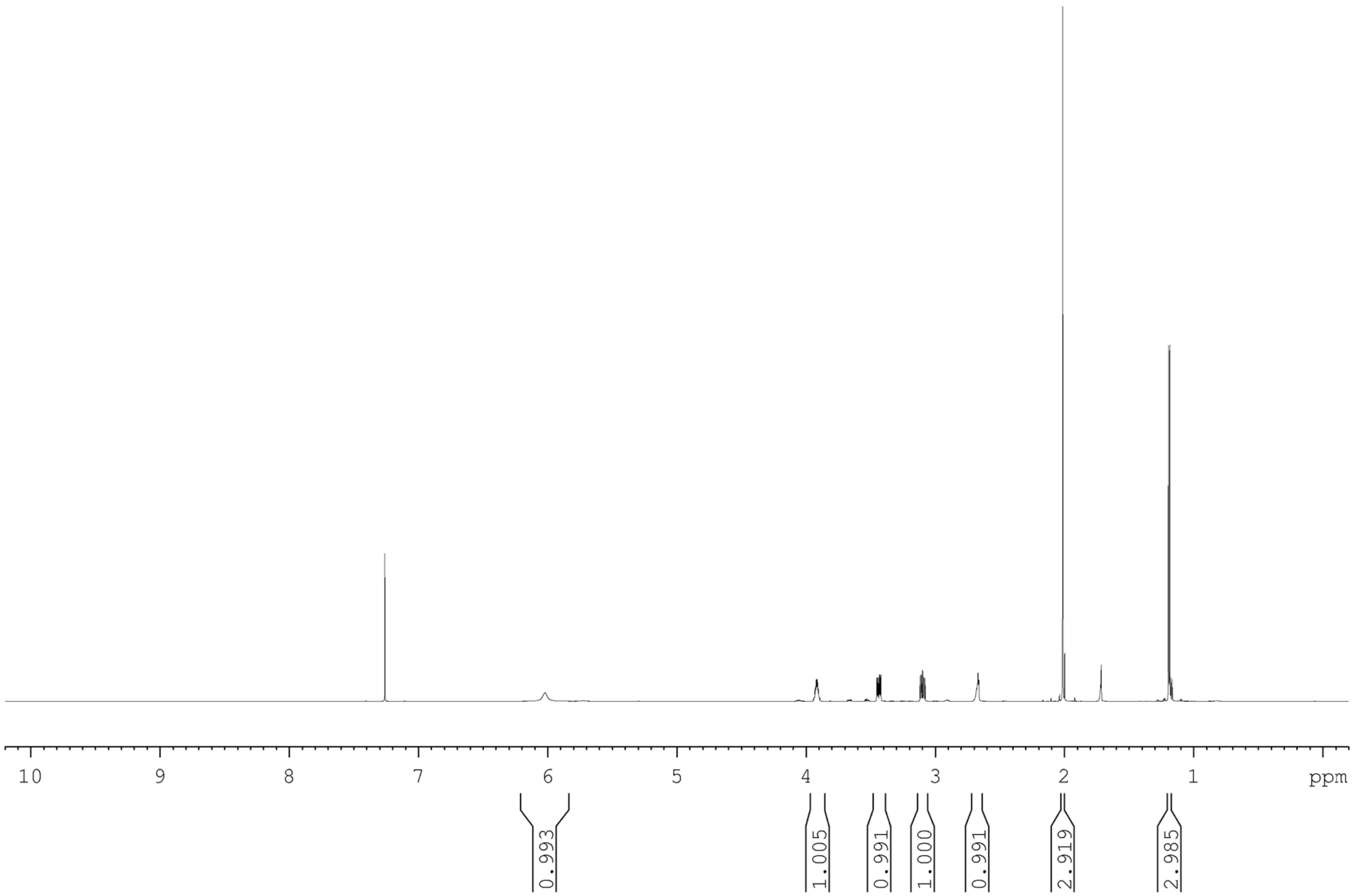

N-(2-hydroxypropyl)acetamide (1b-rac)

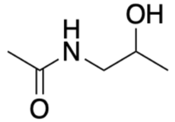

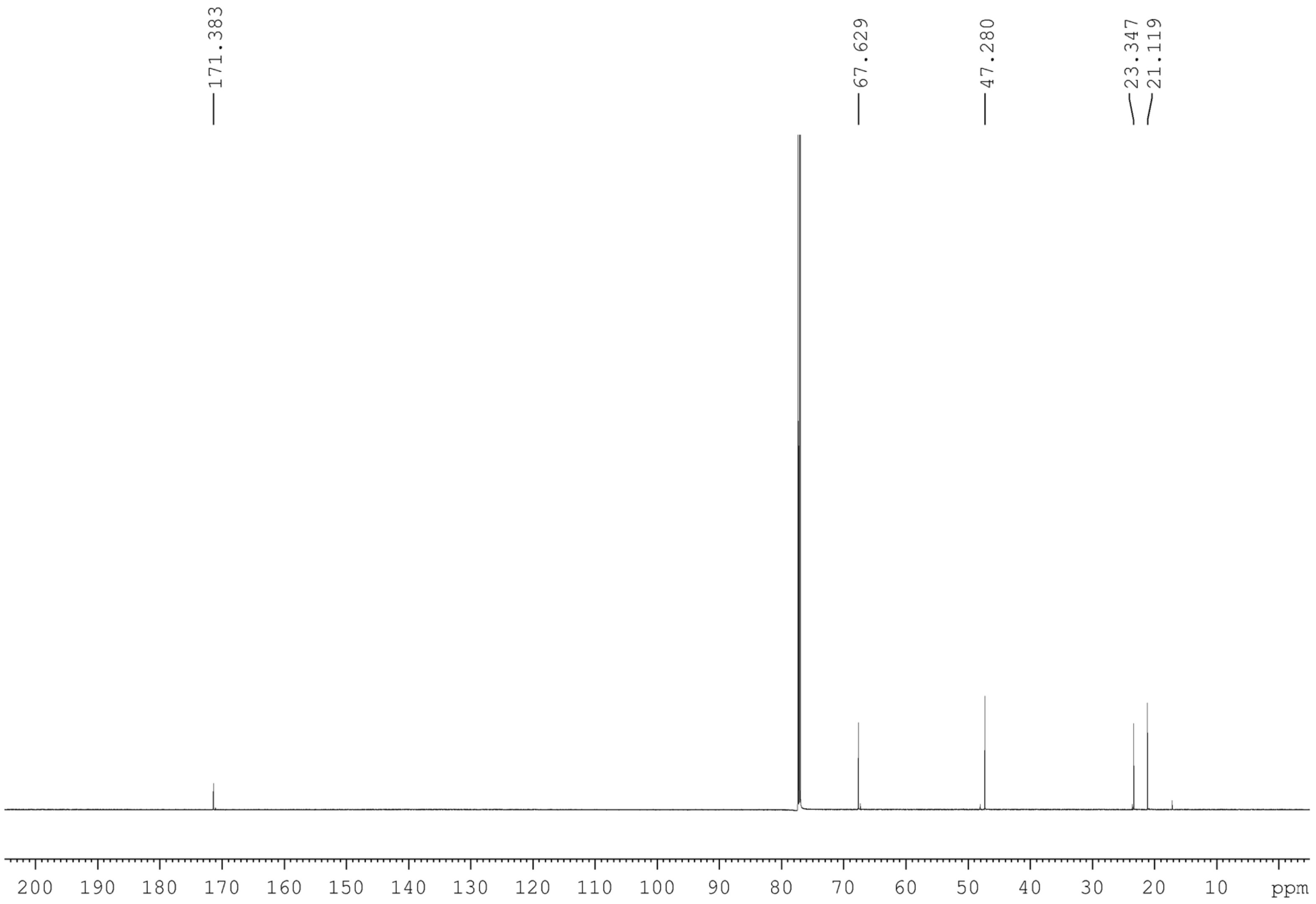

(*S*)-*N*-(2-hydroxypropyl)acetamide (1b)

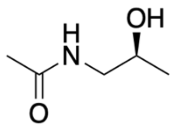

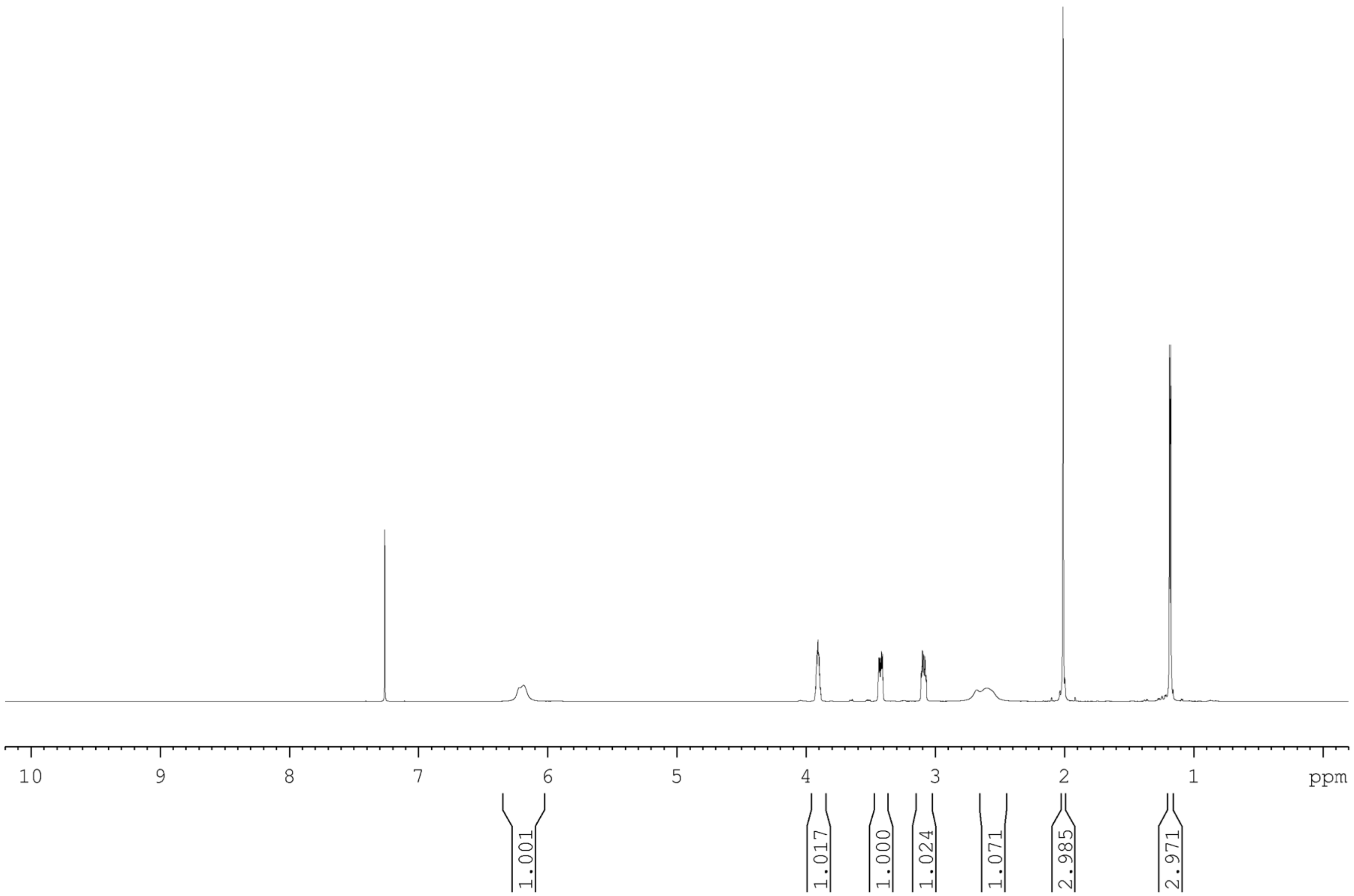

(*S*)-*N*-(2-hydroxypropyl)acetamide (1b)

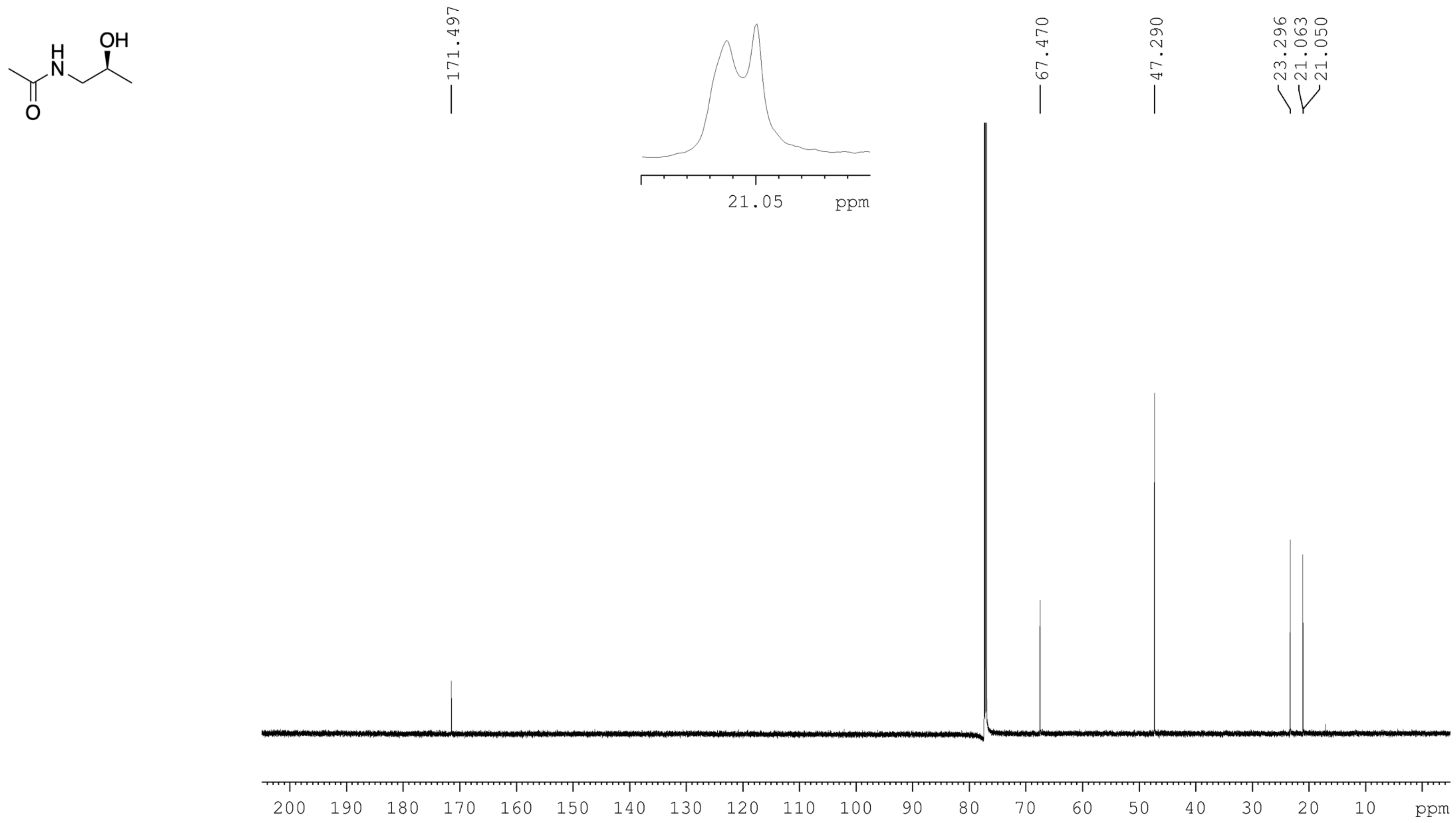

(*S*)-1-(*N*-acetylbenzamido)propan-2-yl benzoate (1b-Bz)

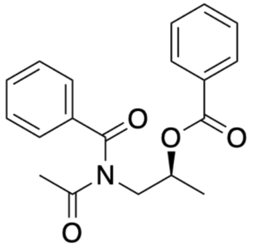

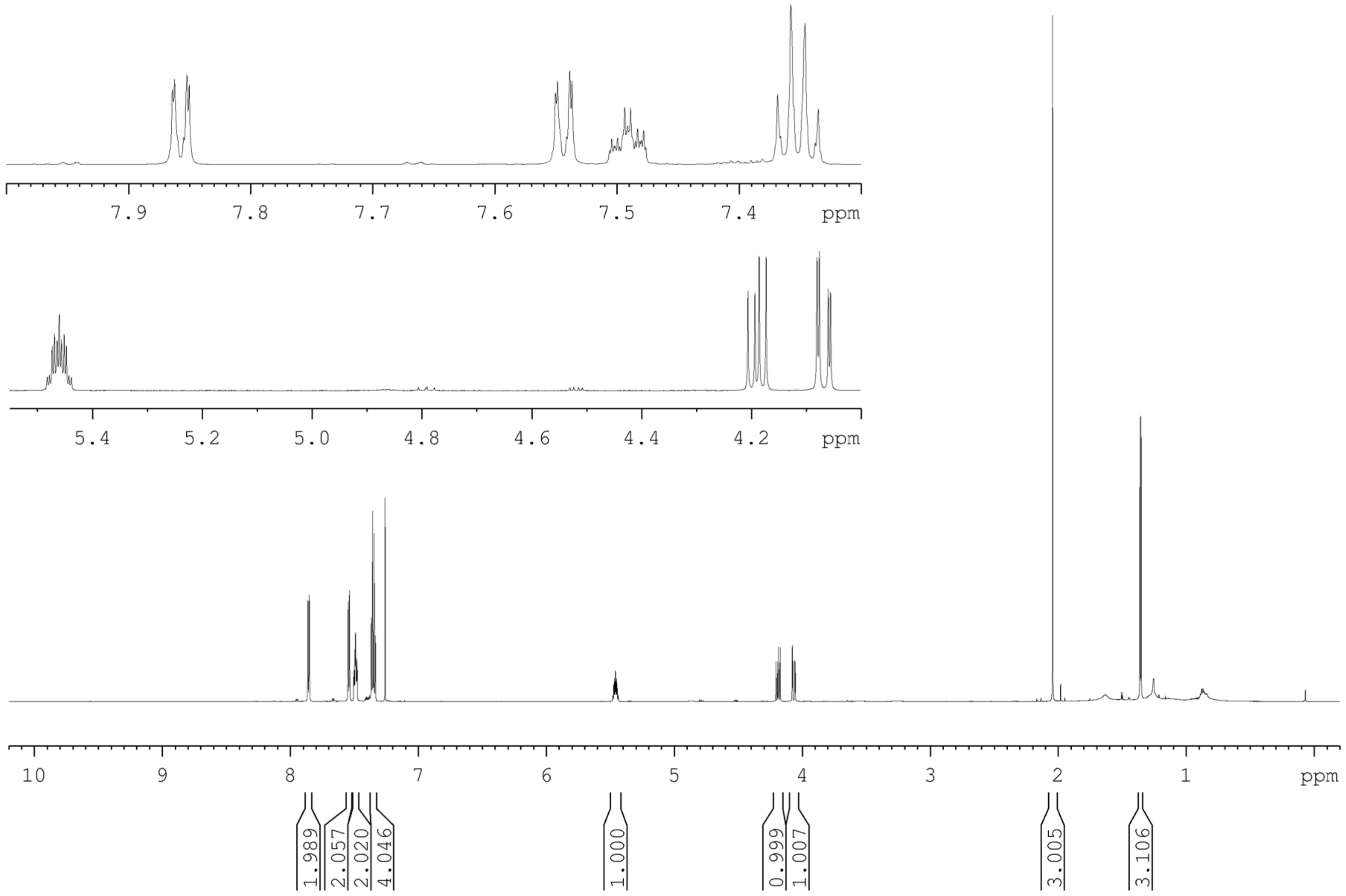

(*S*)-1-(*N*-acetylbenzamido)propan-2-yl benzoate (1b-Bz)

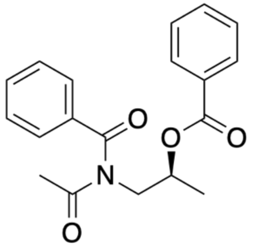

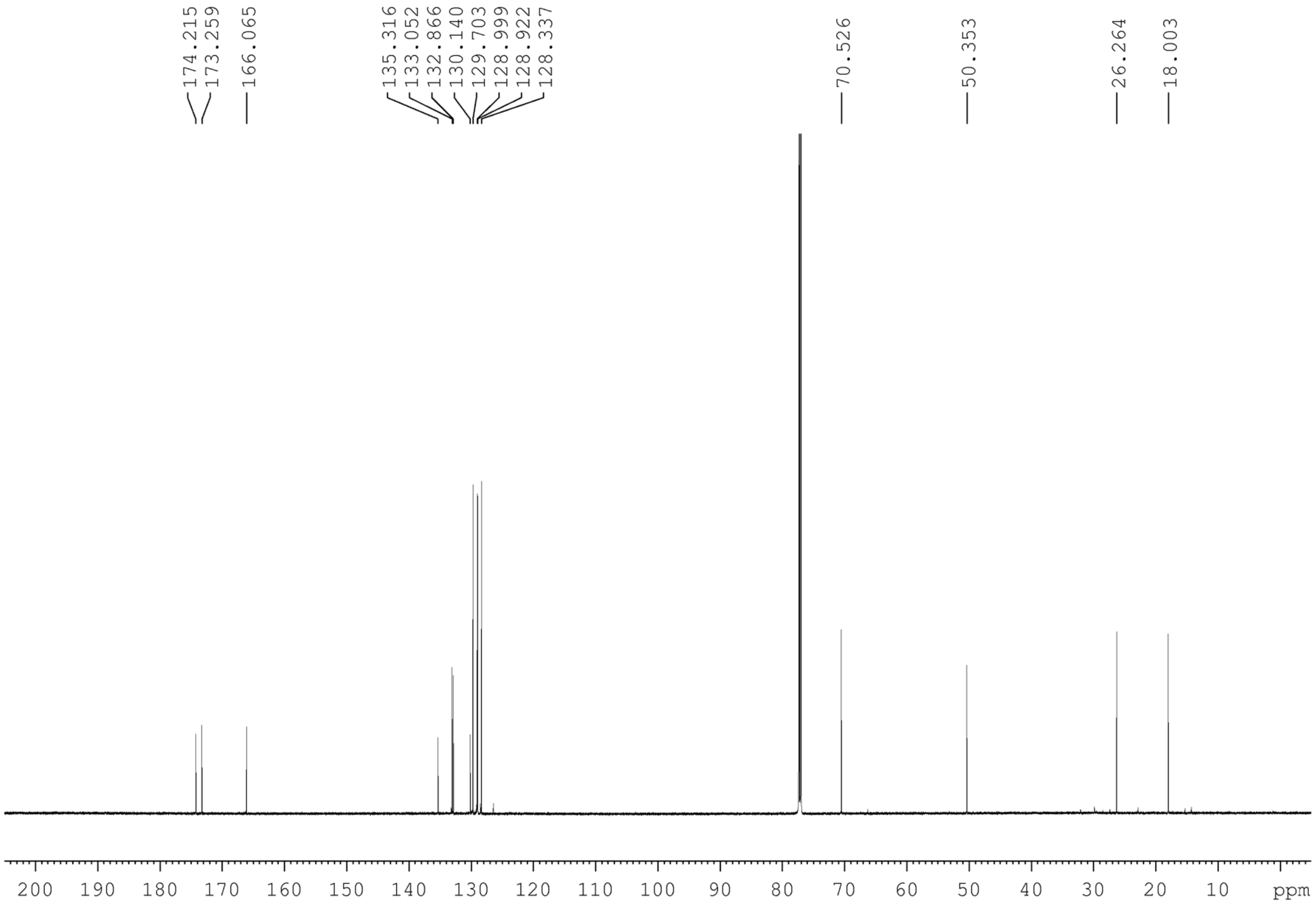

1-aminooctan-2-ol (1c-I_2)

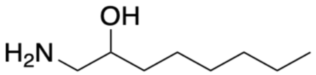

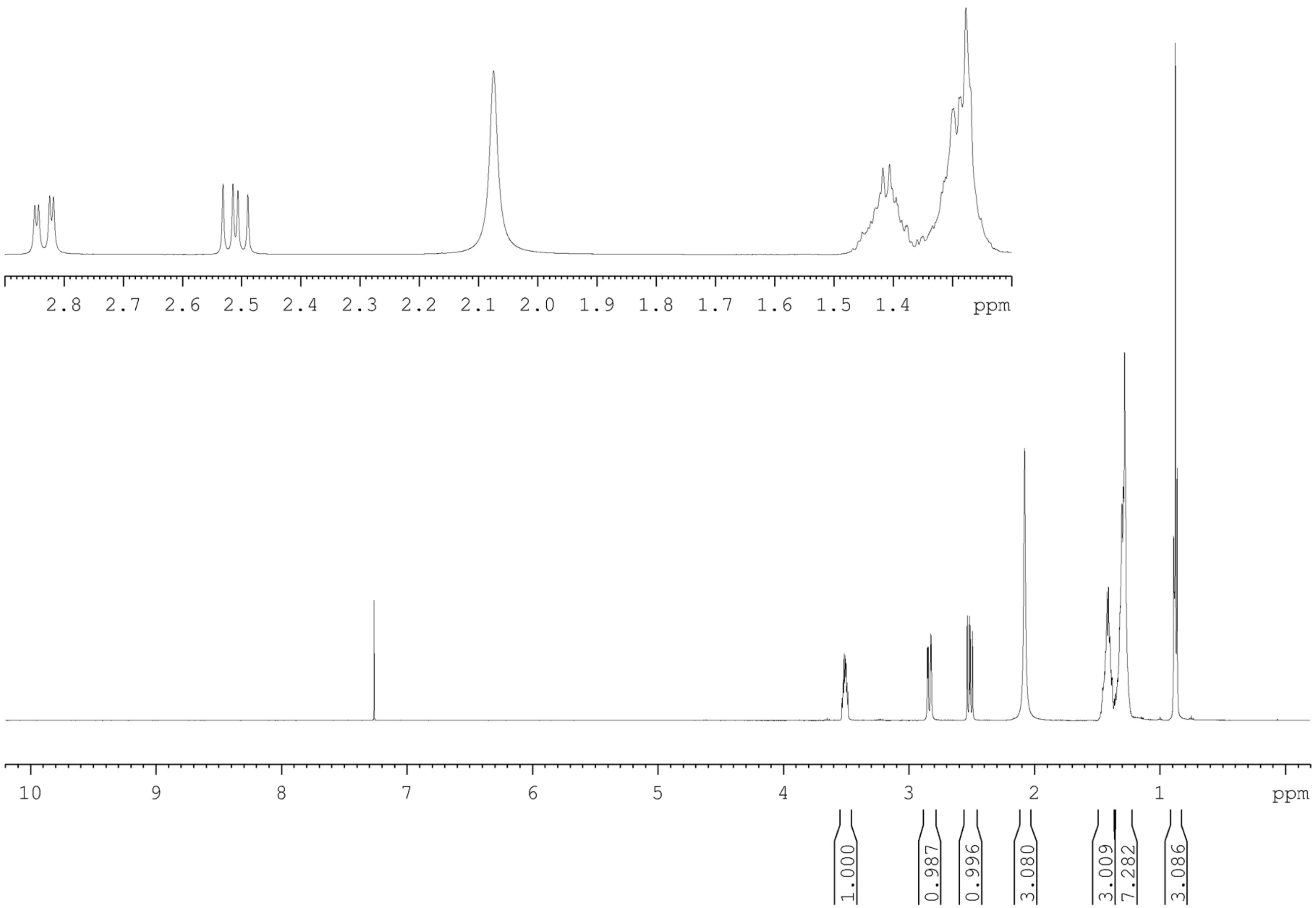

1-aminooctan-2-ol (1c-I_2)

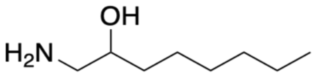

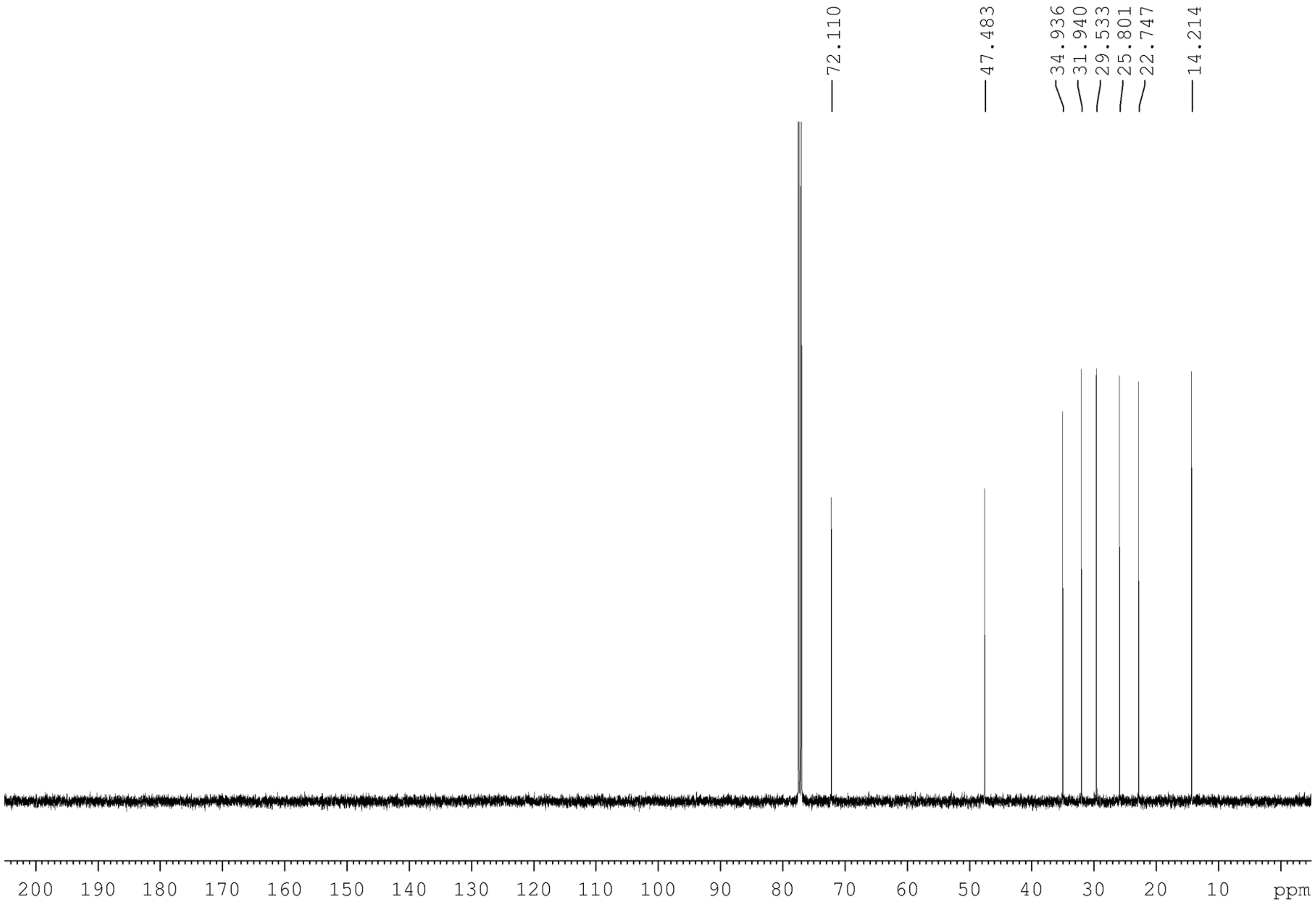

N-(2-hydroxyoctyl)acetamide (1c-rac)

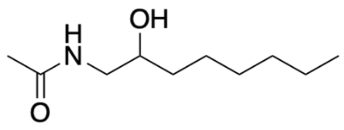

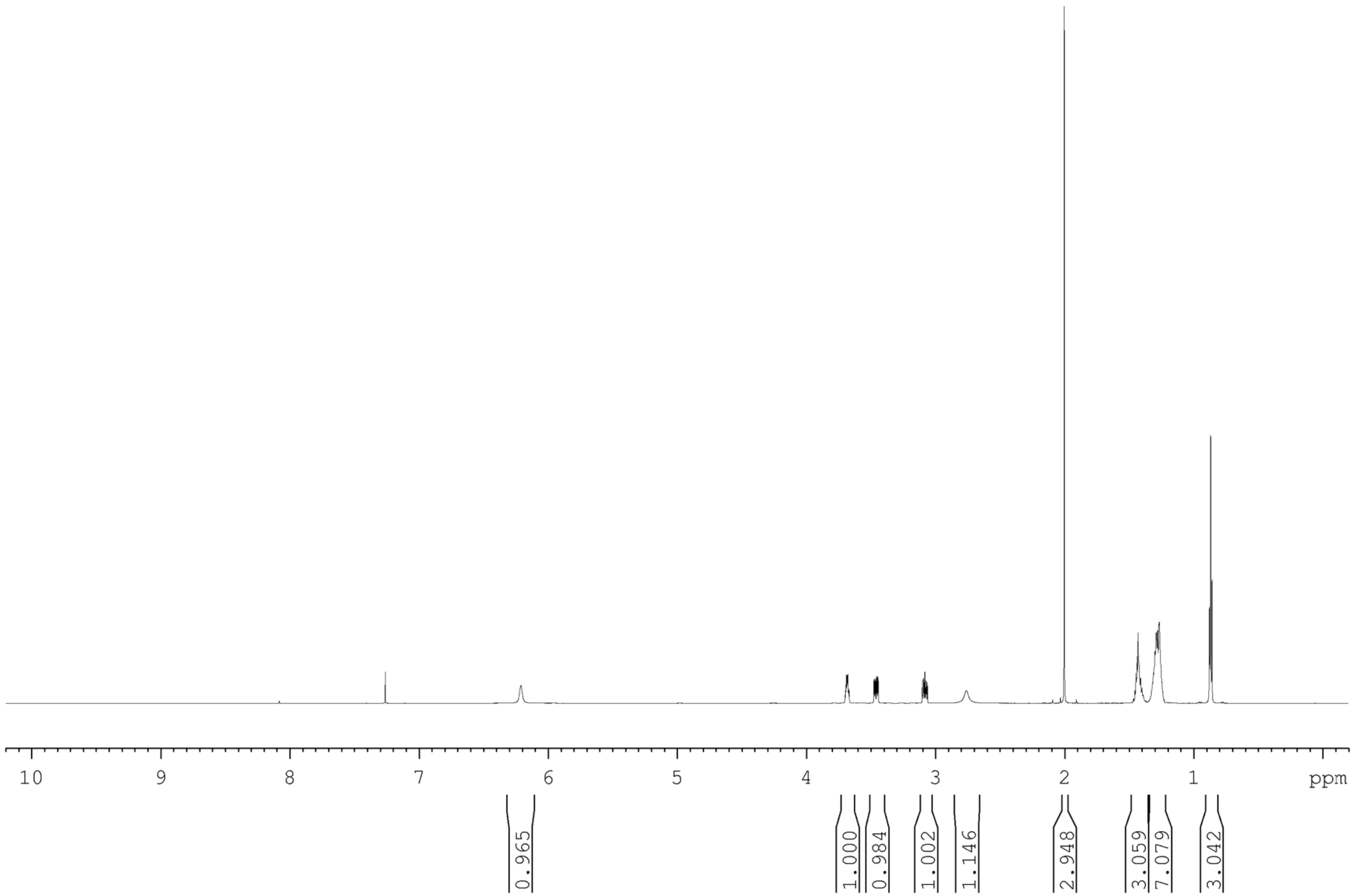

N-(2-hydroxyoctyl)acetamide (1c-rac)

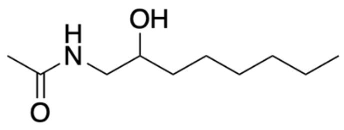

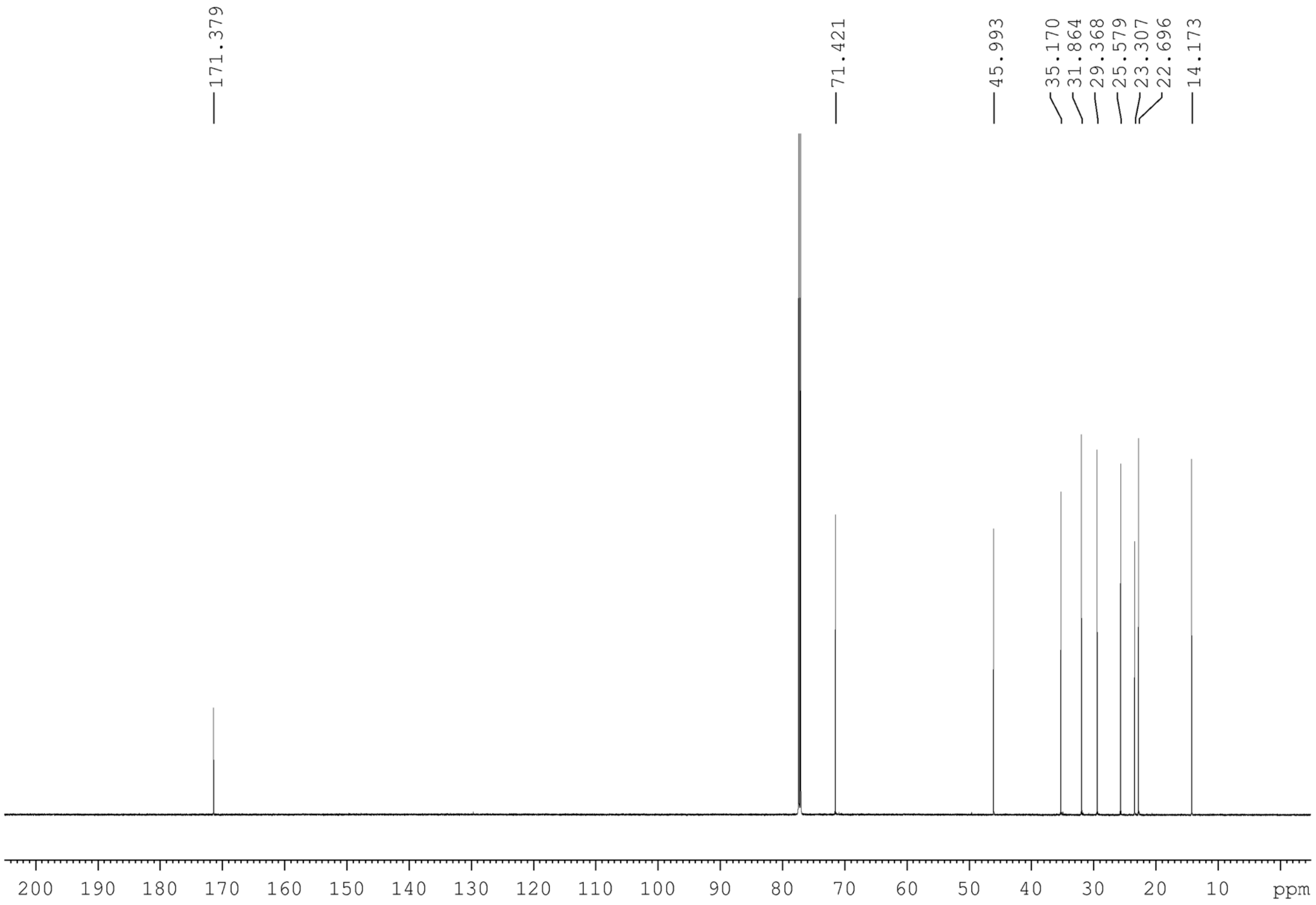

(*S*)-*N*-(2-hydroxyoctyl)acetamide (1c)

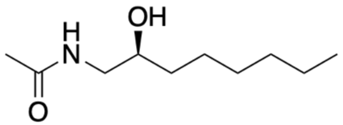

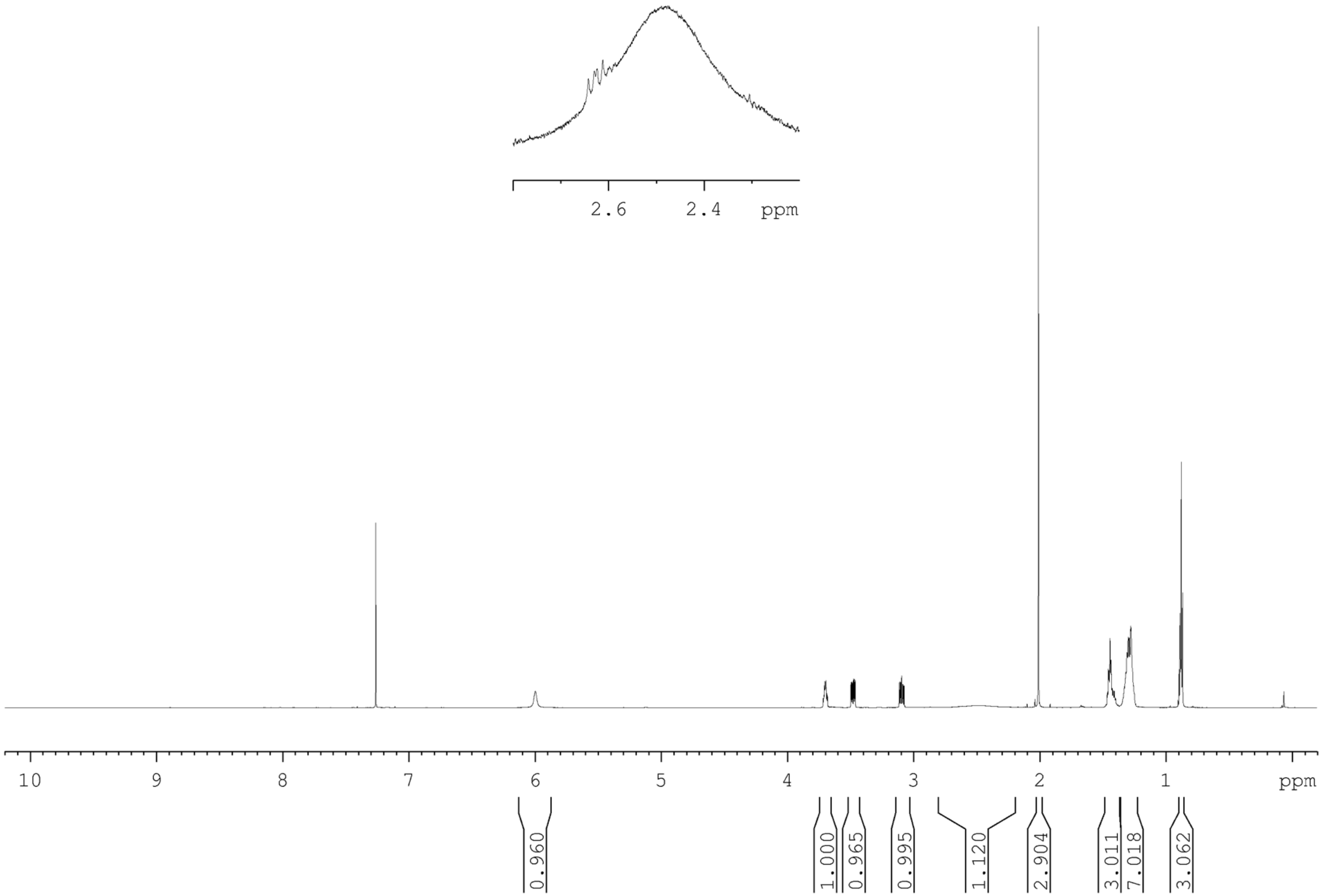

(*S*)-*N*-(2-hydroxyoctyl)acetamide (1c)

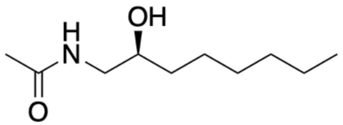

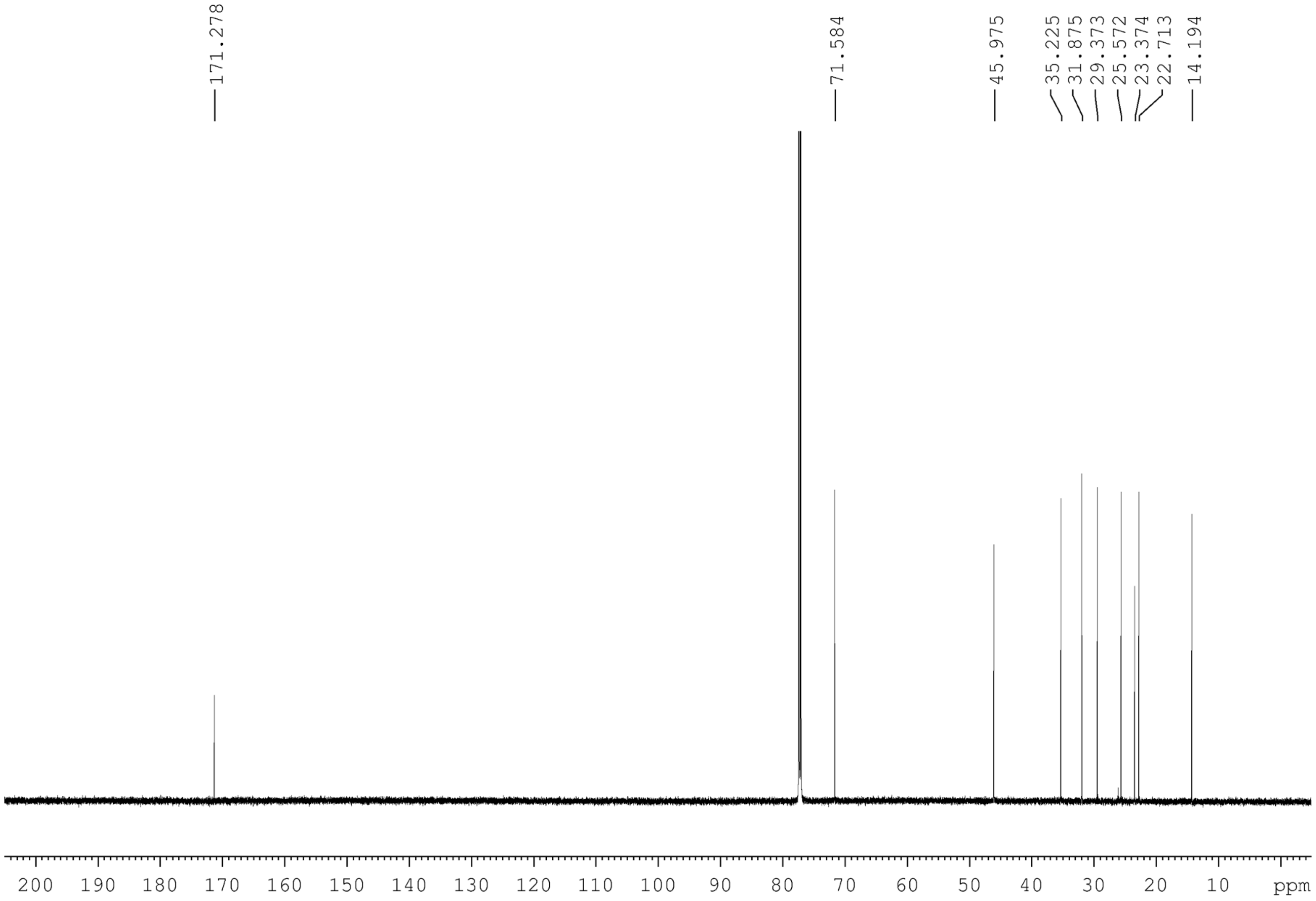

(*S*)-1-(*N*-acetylbenzamido)octan-2-yl benzoate (1c-Bz)

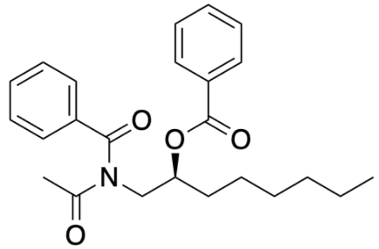

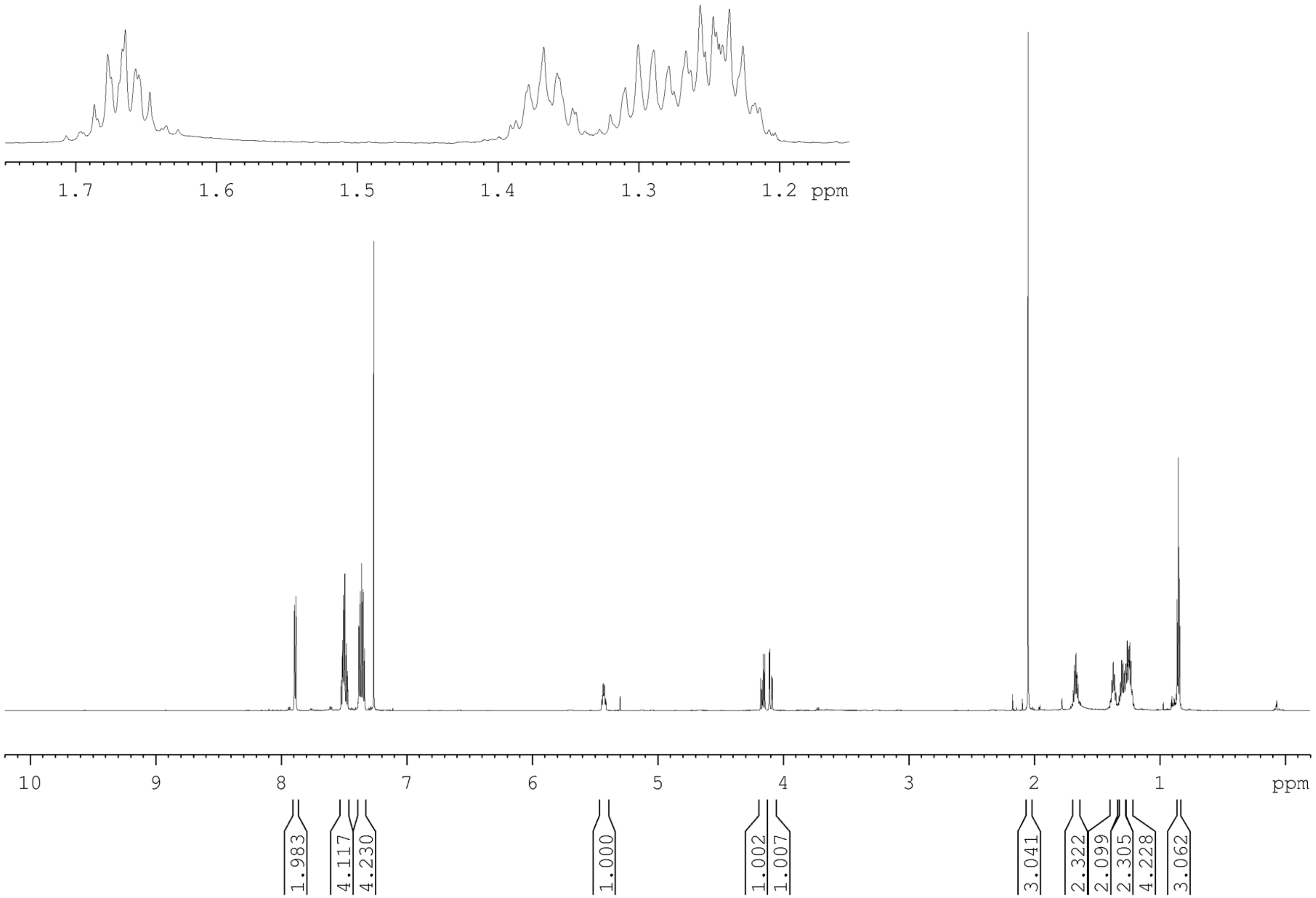

(*S*)-1-(*N*-acetylbenzamido)octan-2-yl benzoate (1c-Bz)

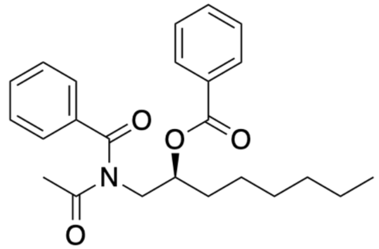

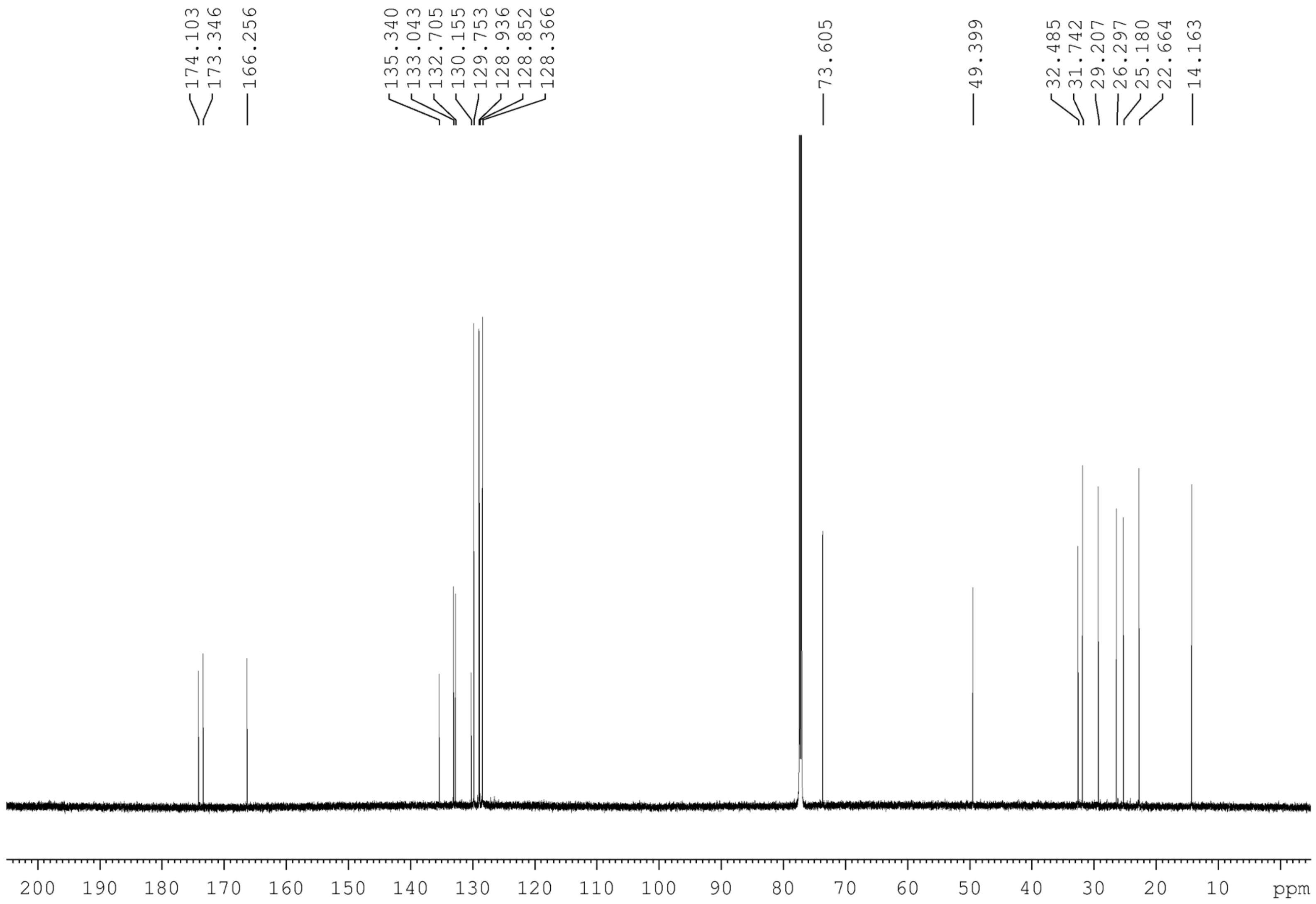

1-amino-7-methyloctan-2-ol (1d-I_3)

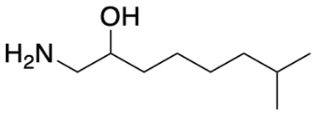

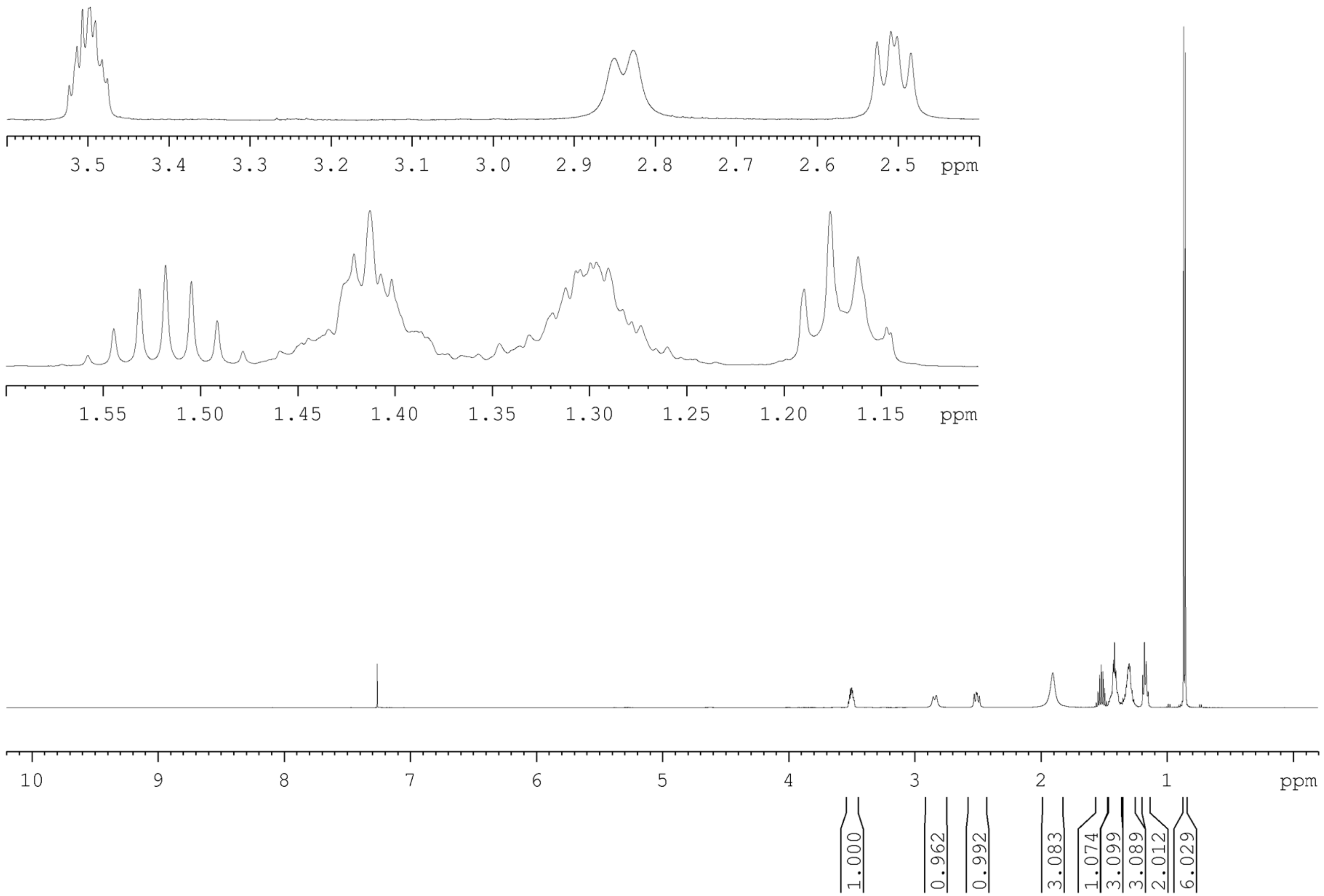

1-amino-7-methyloctan-2-ol (1d-I_3)

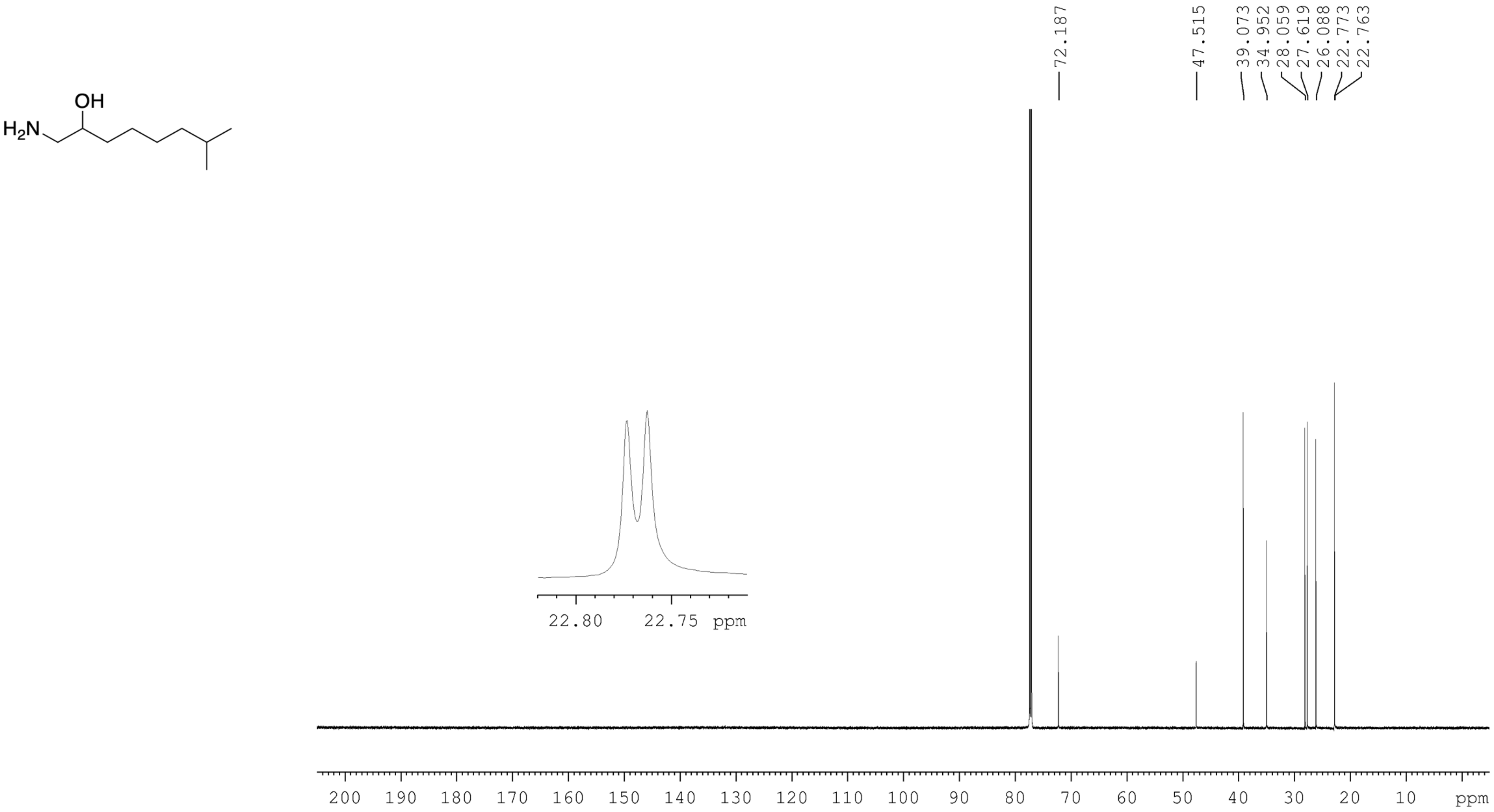

N-(2-hydroxy-7-methyloctyl)acetamide (1d-rac)

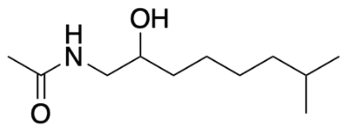

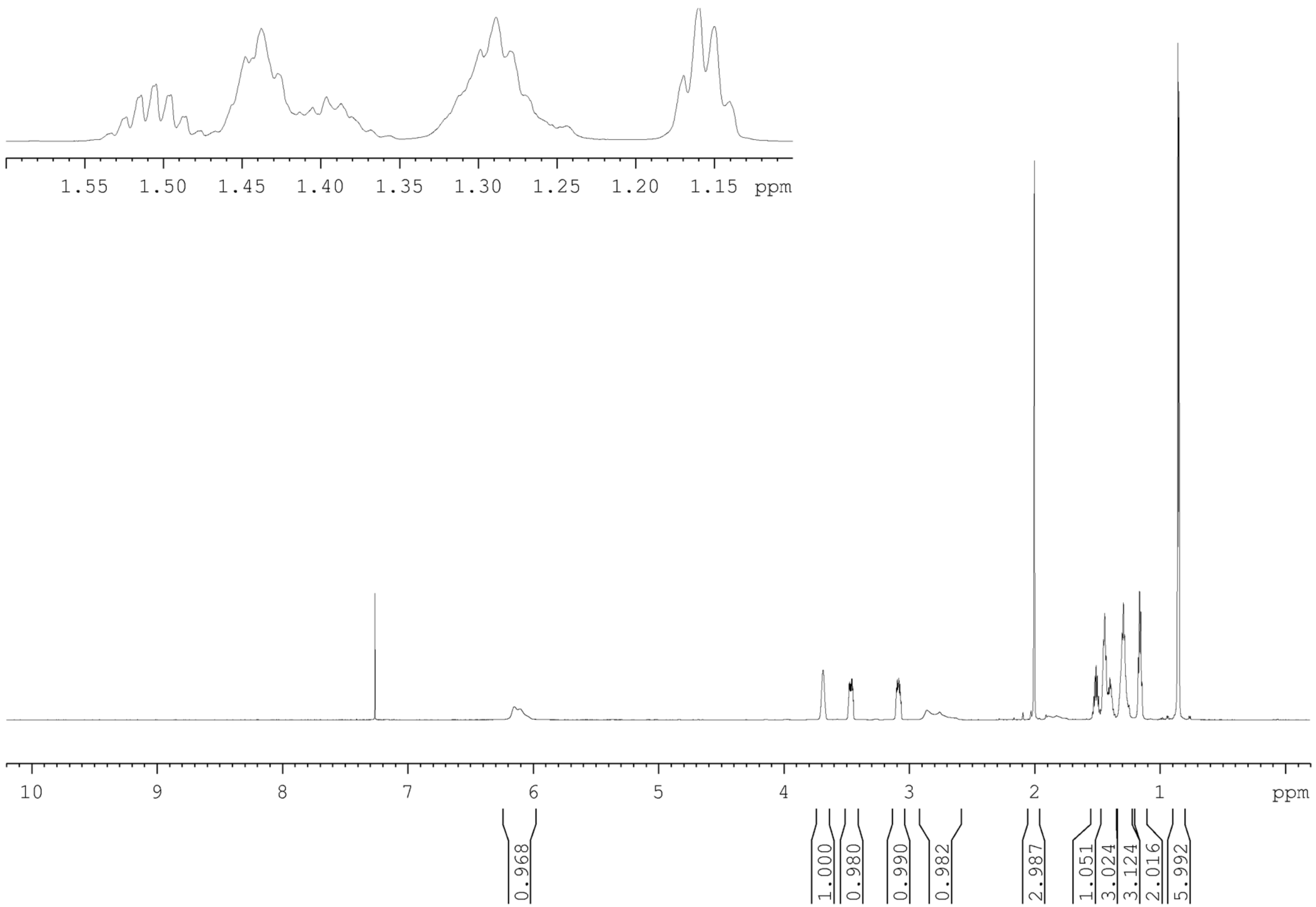

N-(2-hydroxy-7-methyloctyl)acetamide (1d-rac)

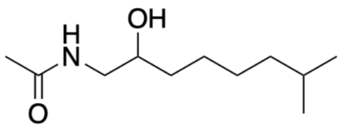

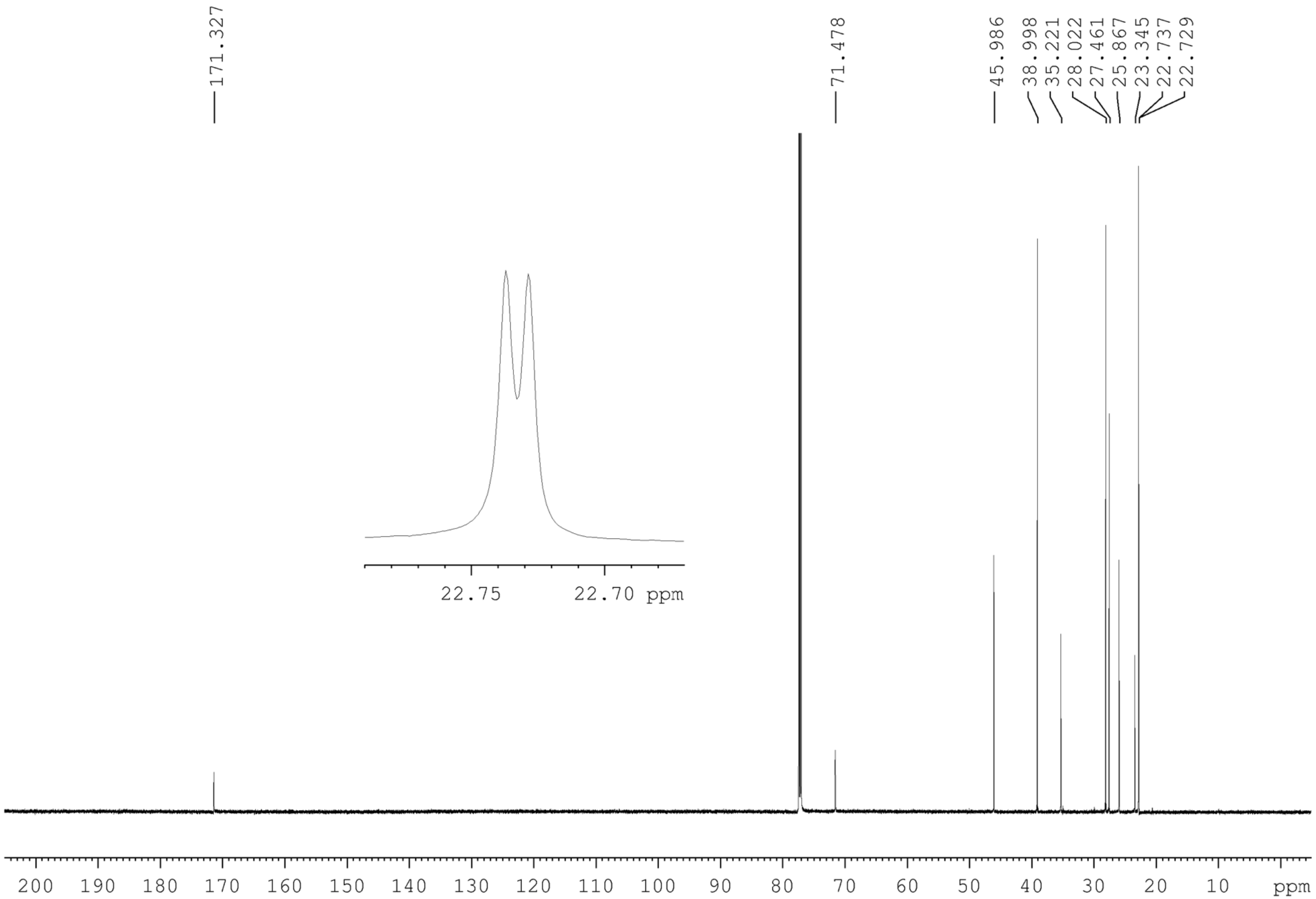

(*S*)-*N*-(2-hydroxy-7-methyloctyl)acetamide (1d)

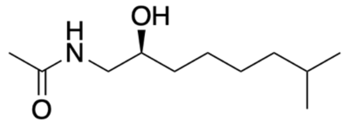

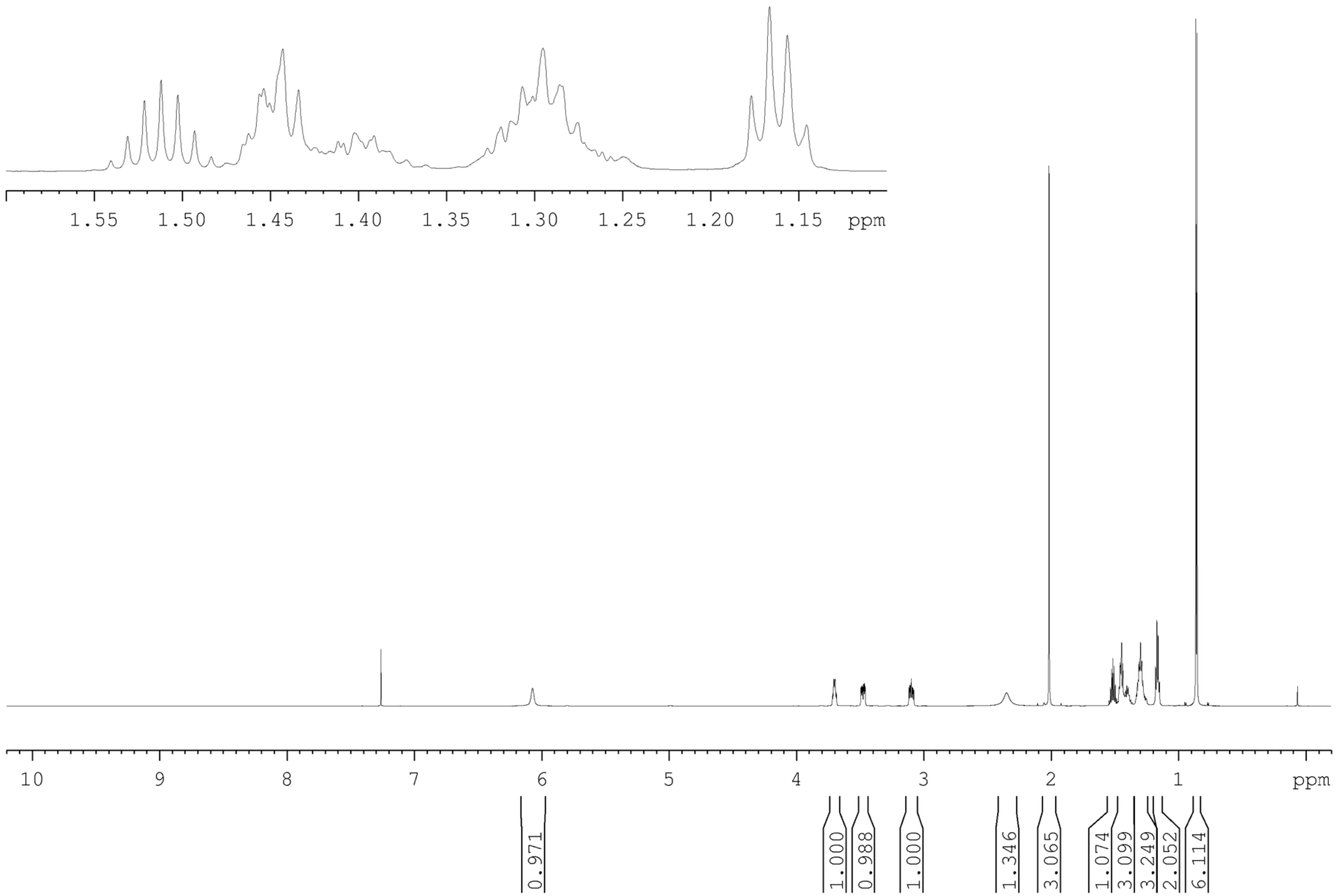

(*S*)-*N*-(2-hydroxy-7-methyloctyl)acetamide (1d)

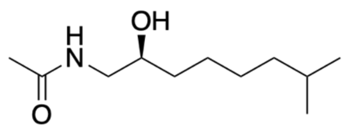

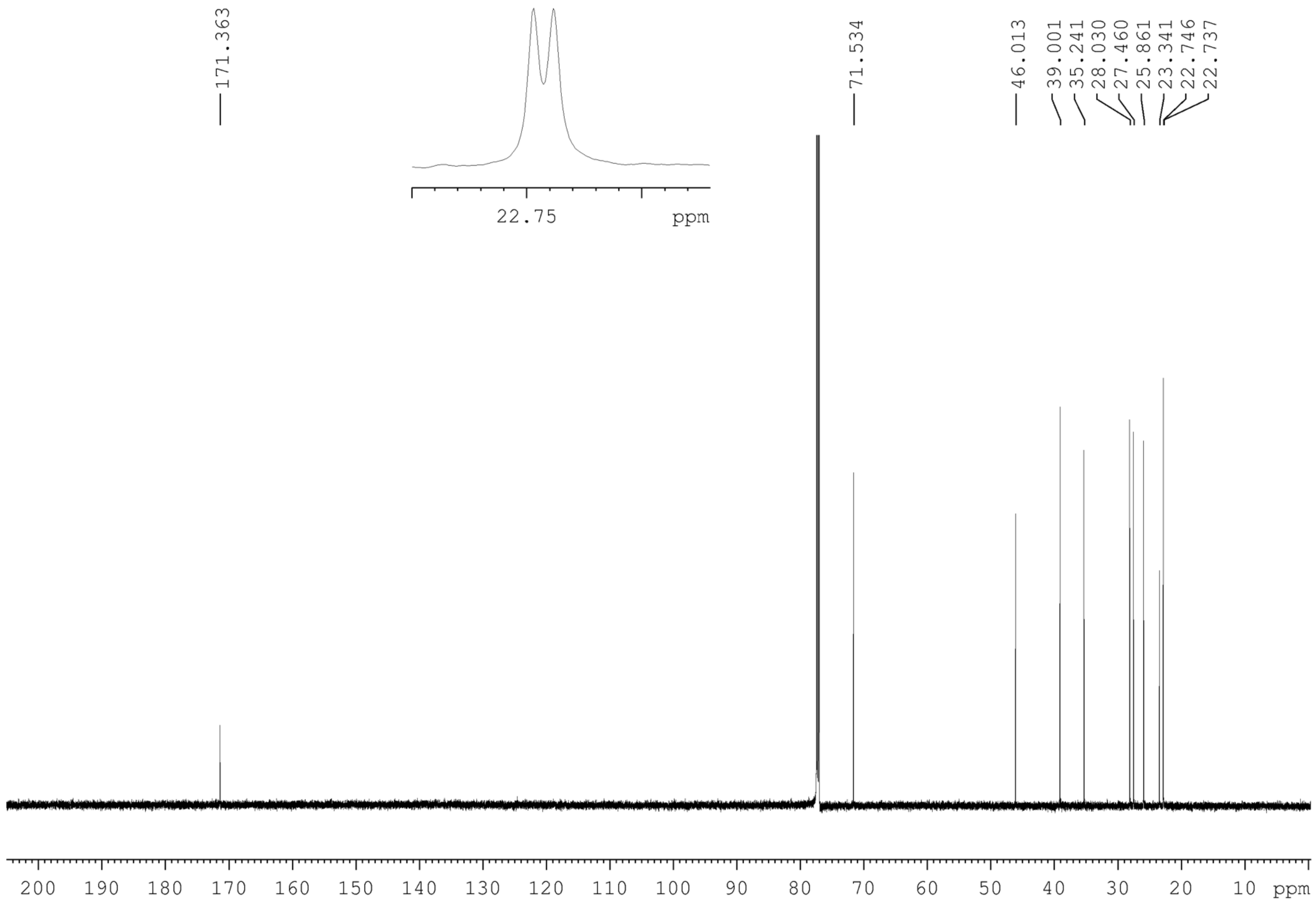

(*S*)-1-(*N*-acetylbenzamido)-7-methyloctan-2-yl benzoate (1d-Bz)

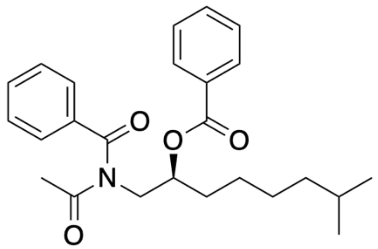

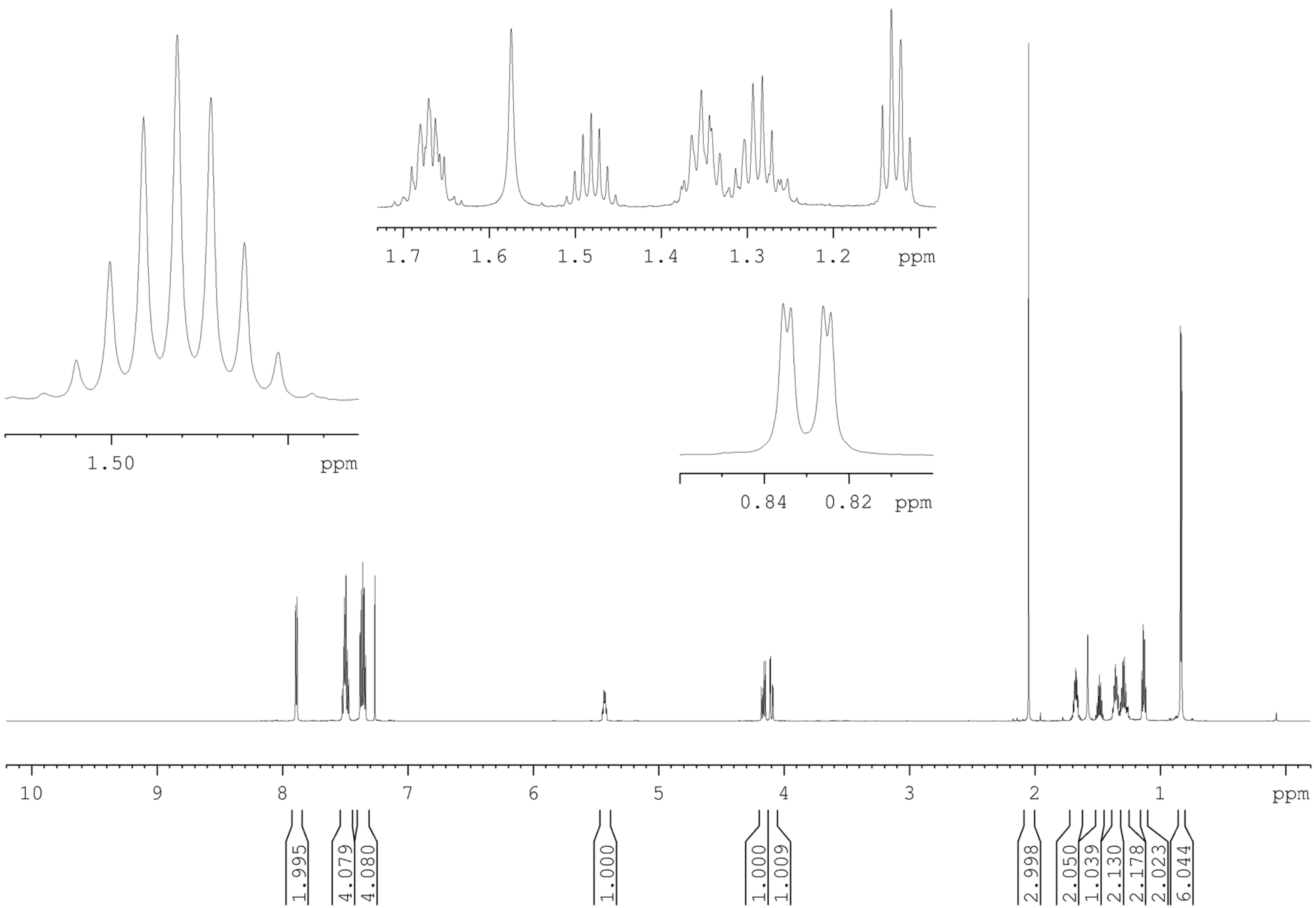

(*S*)-1-(*N*-acetylbenzamido)-7-methyloctan-2-yl benzoate (1d-Bz)

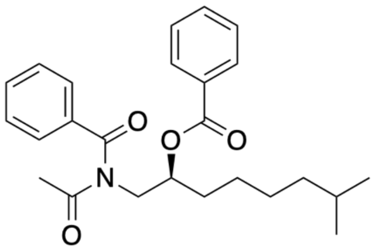

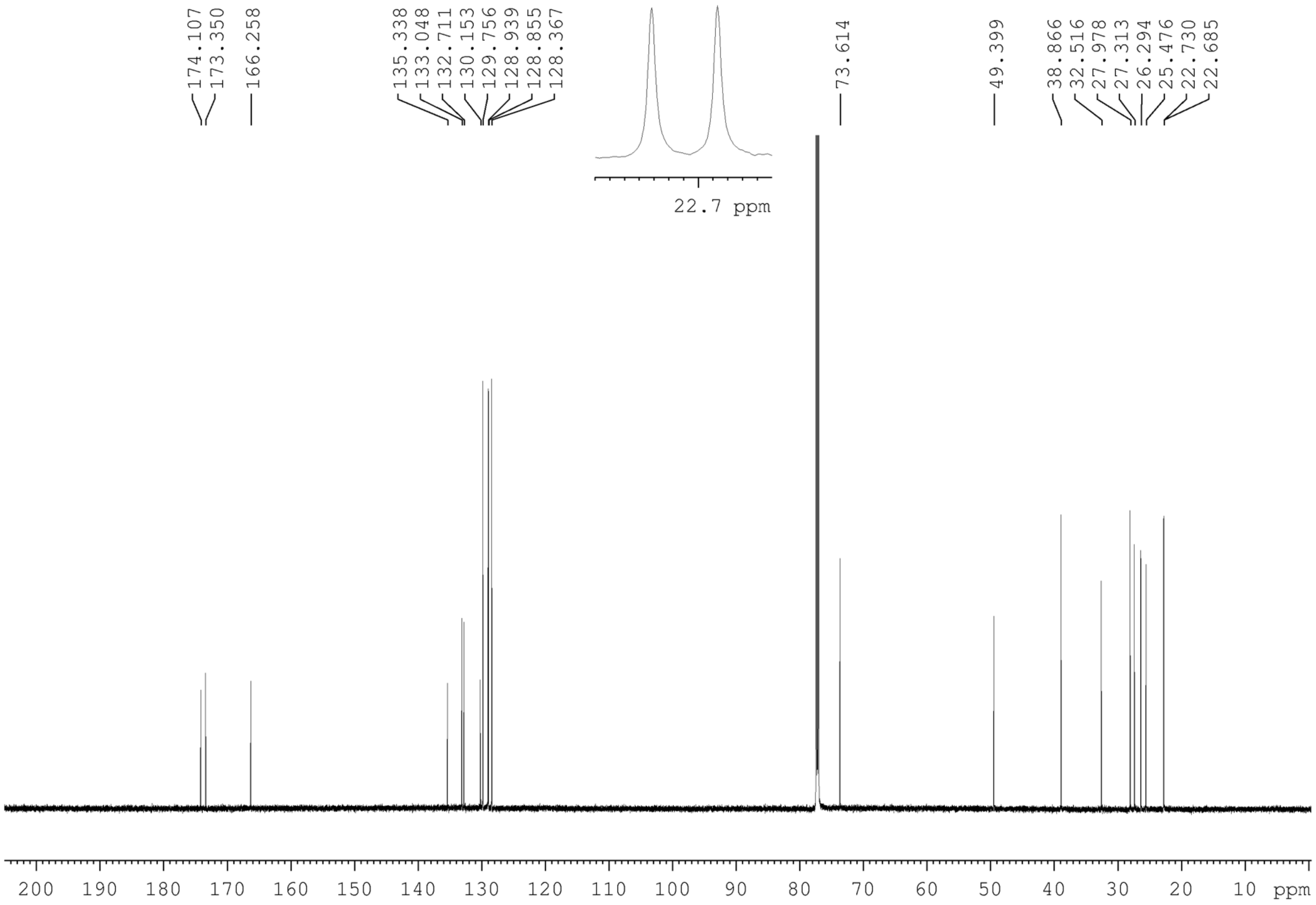

N-(4-cyclobutyl-2-hydroxybutyl)acetamide (1e-rac)

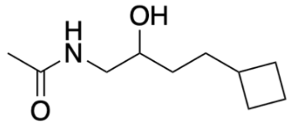

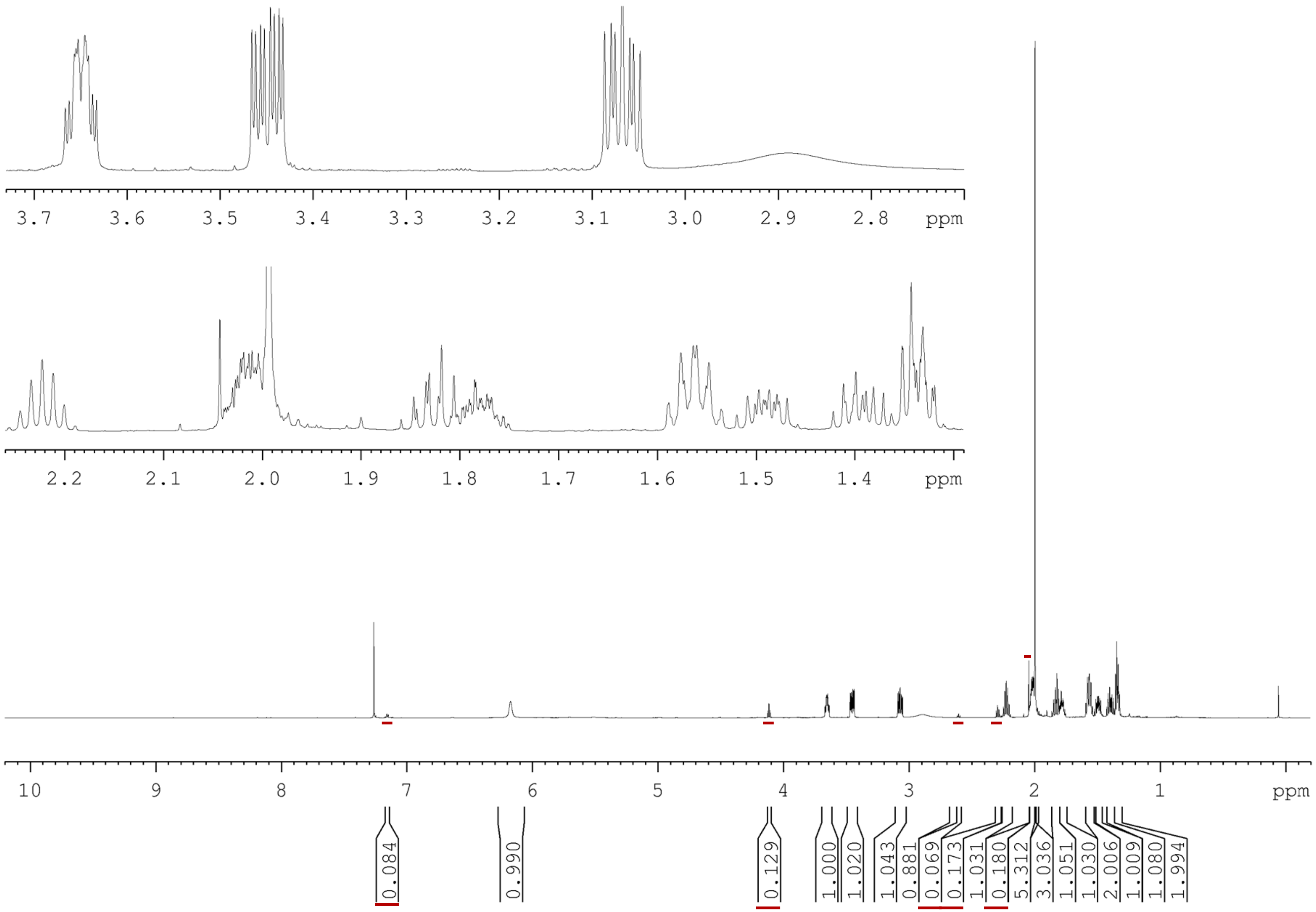

— *Indicates impurity peak.*

N-(4-cyclobutyl-2-hydroxybutyl)acetamide (1e-rac)

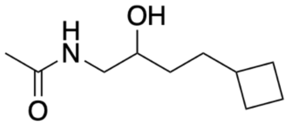

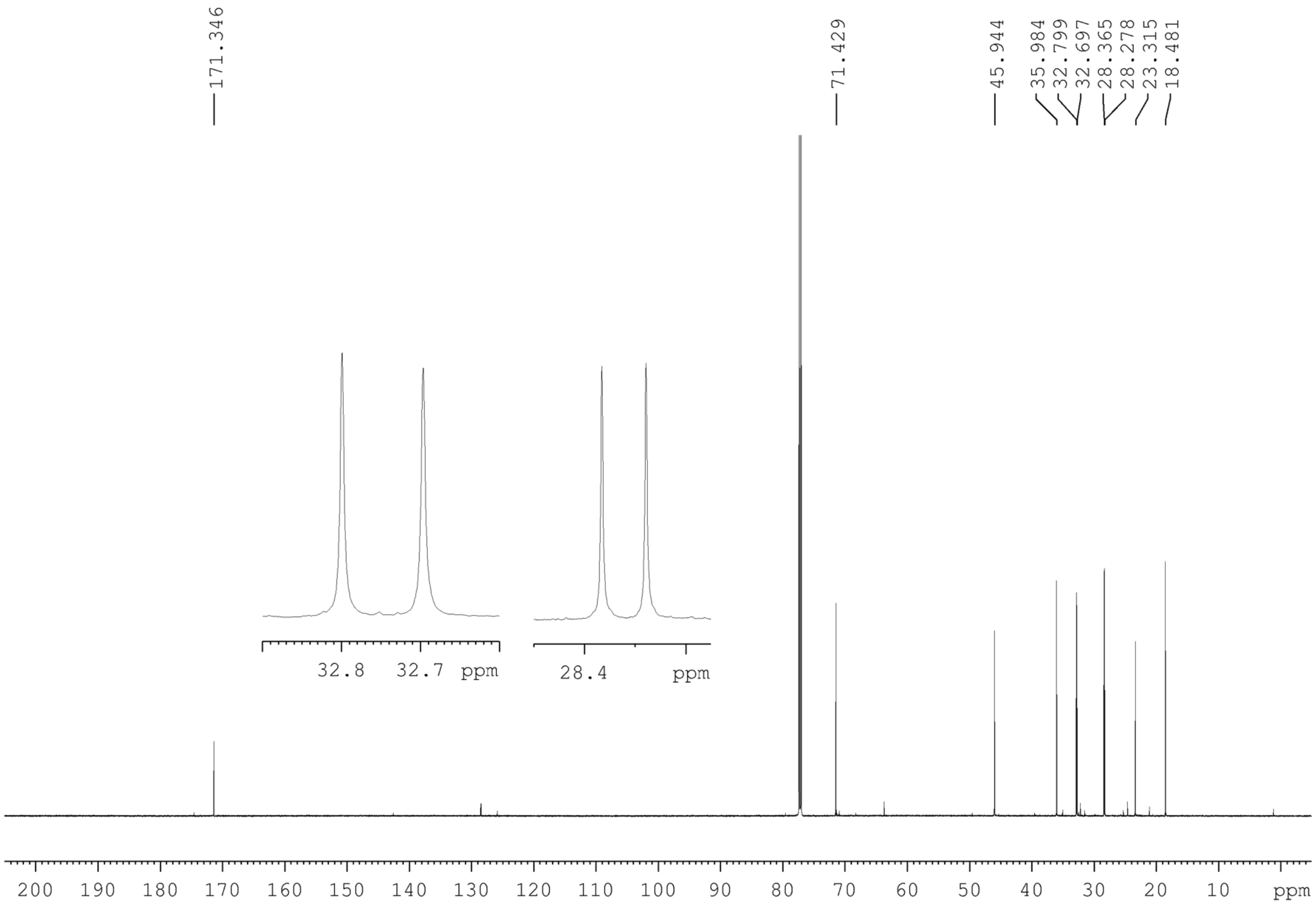

(*S*)-*N*-(4-cyclobutyl-2-hydroxybutyl)acetamide (1e)

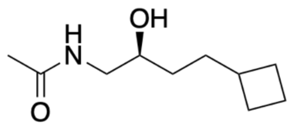

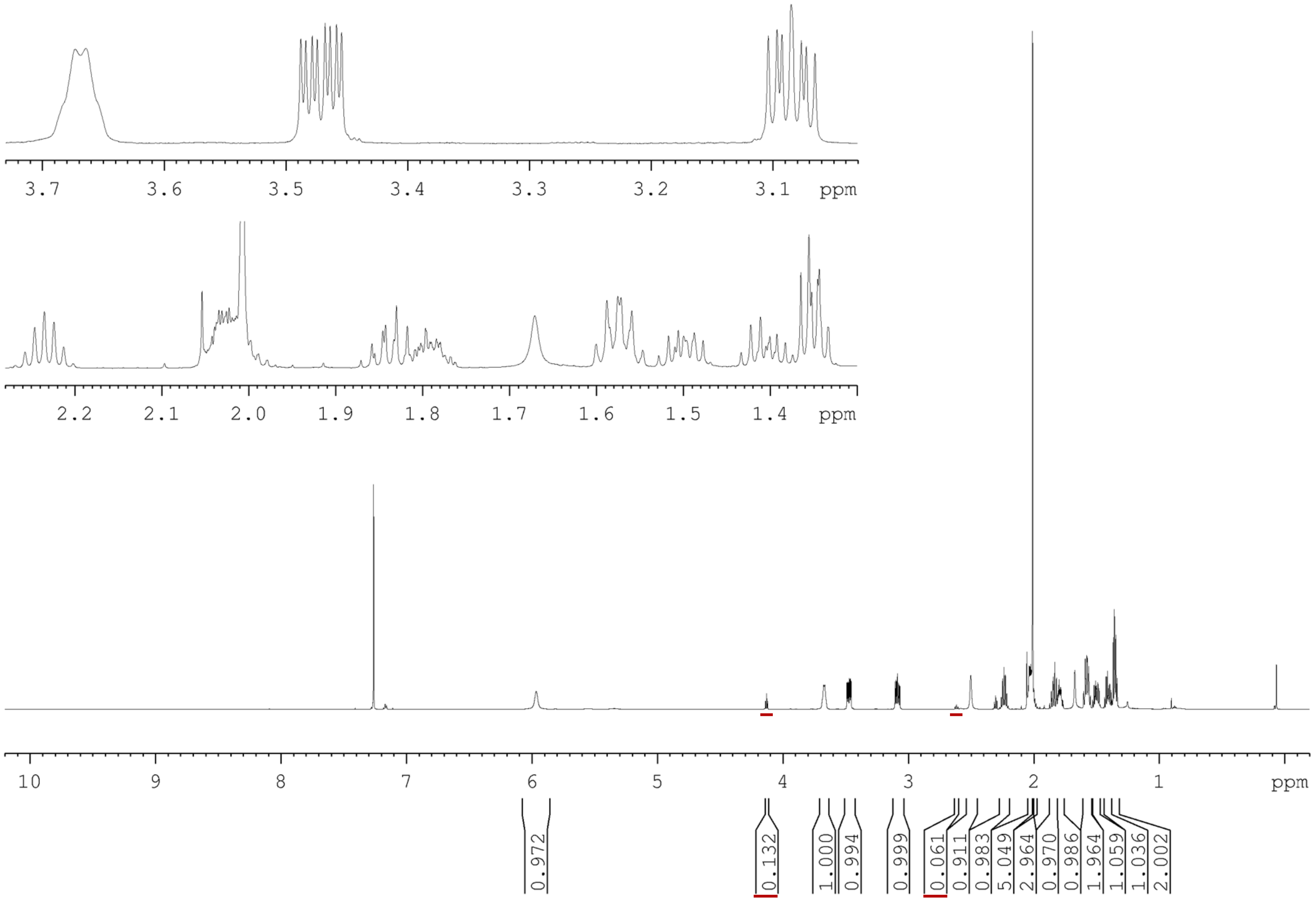

— *Indicates impurity peak. Some peaks mitigated for clarity – see **1e-rac** ¹H NMR spectrum for the indication and integration of each discernable impurity peak. Approximately equal amount of impurity in **1e-rac** and **1e**.*

(*S*)-*N*-(4-cyclobutyl-2-hydroxybutyl)acetamide (1e)

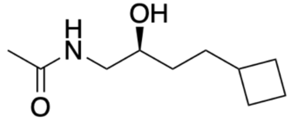

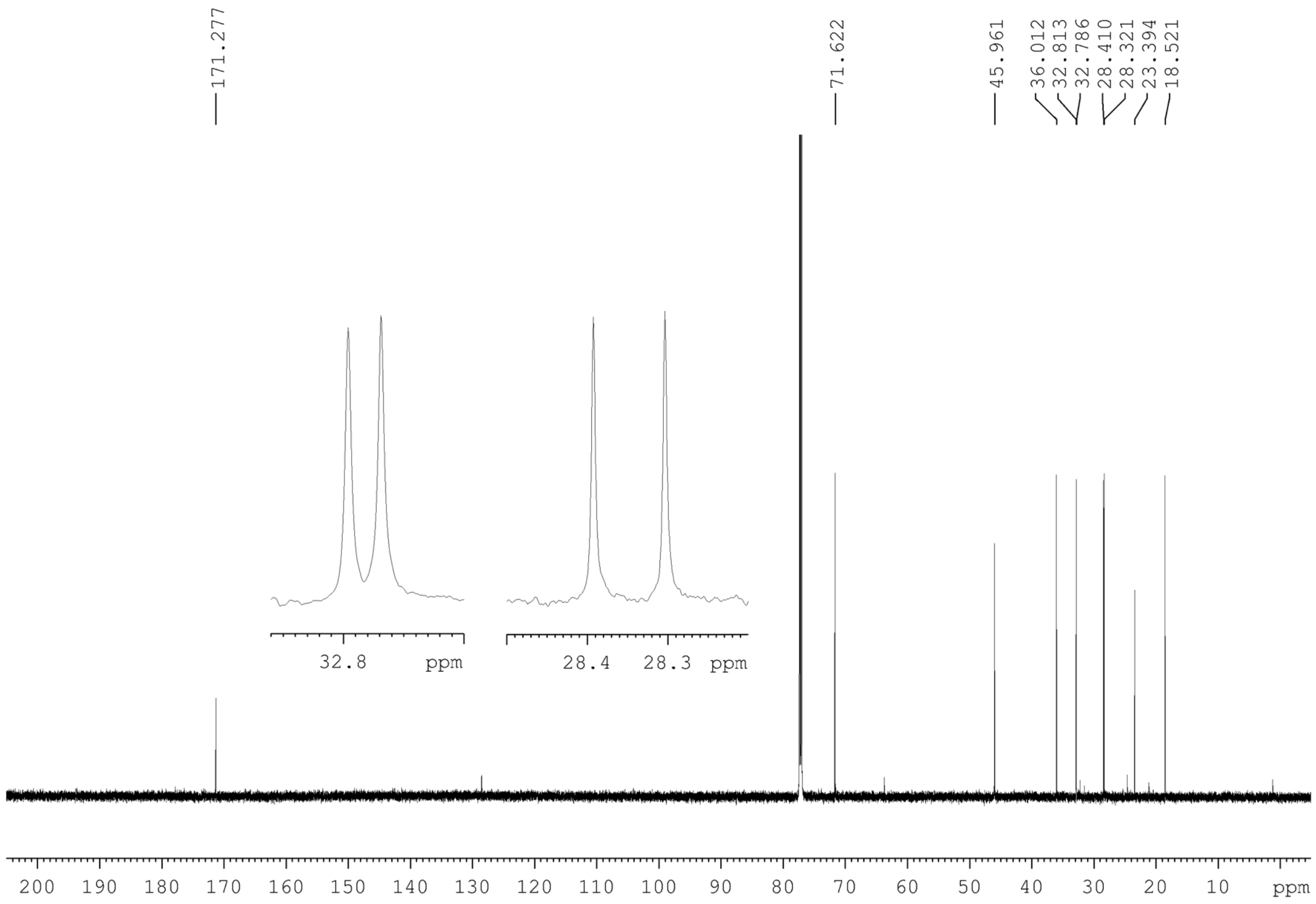

(*S*)-1-(*N*-acetylbenzamido)-4-cyclobutylbutan-2-yl benzoate (1e-Bz)

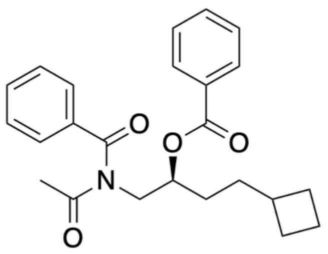

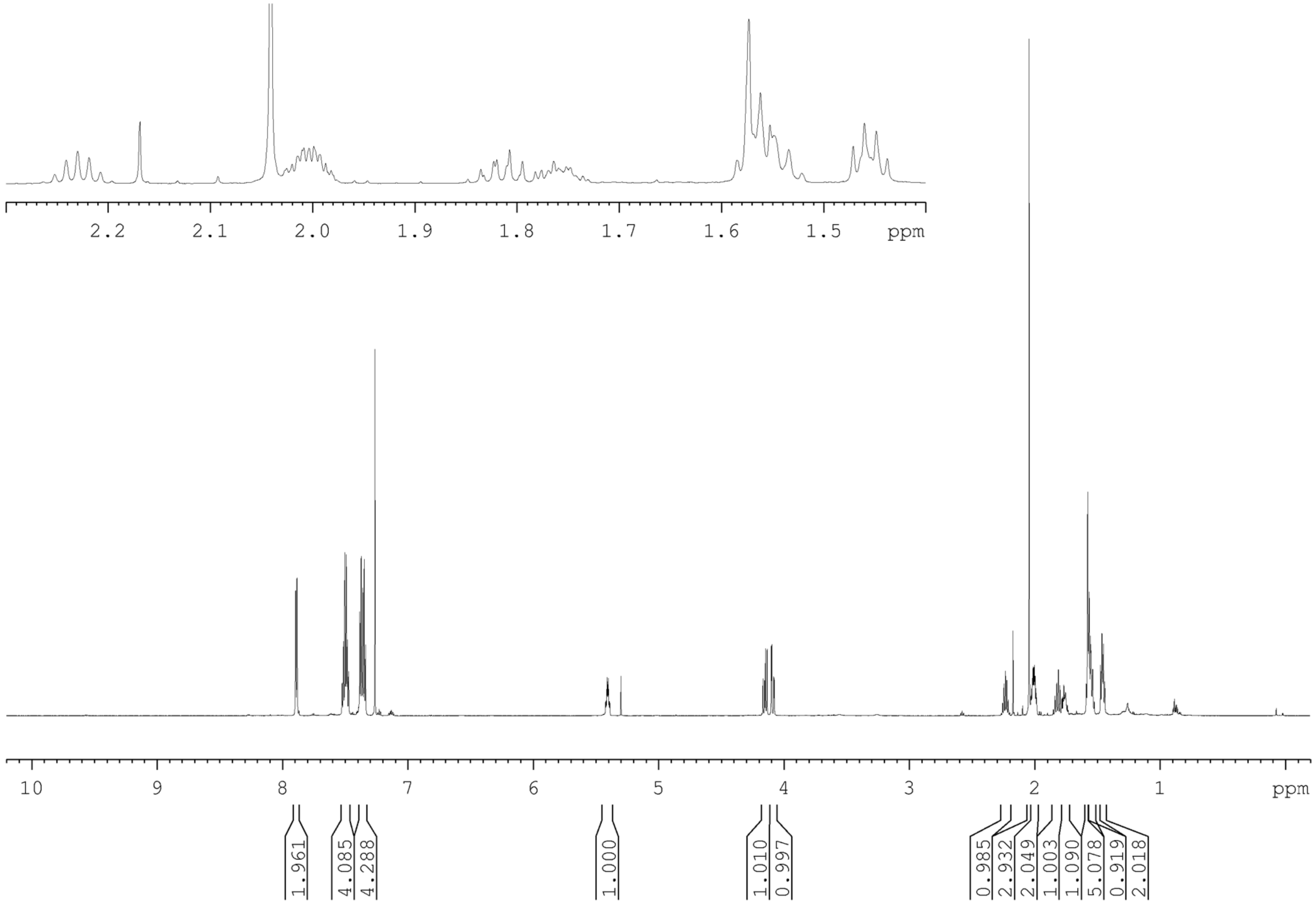

(*S*)-1-(*N*-acetylbenzamido)-4-cyclobutylbutan-2-yl benzoate (1e-Bz)

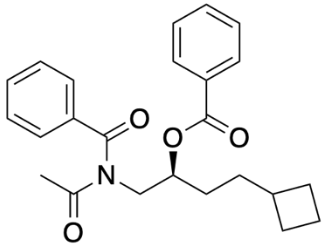

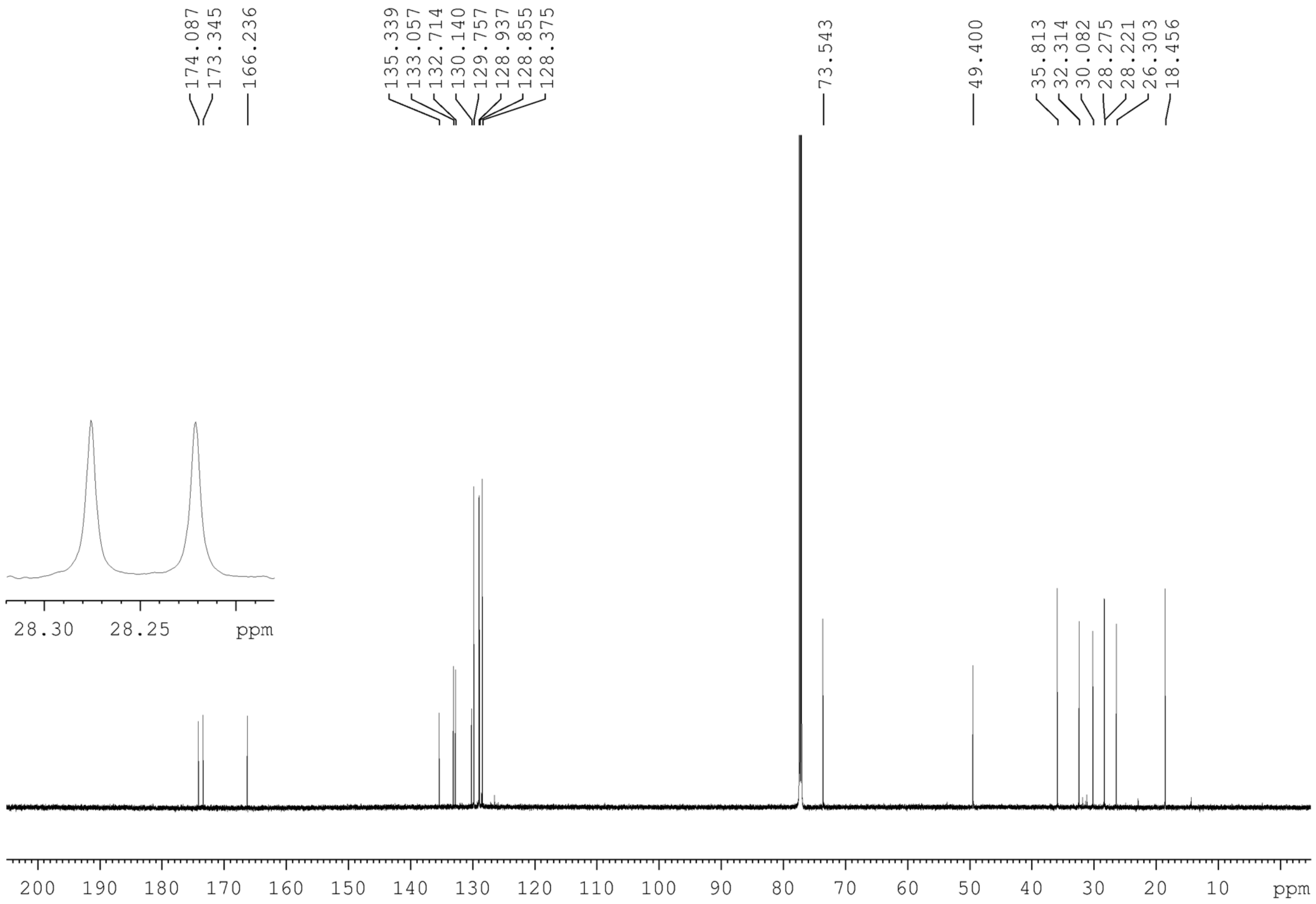

***N*-(5-cyclohexyl-2-hydroxypentyl)acetamide (1f-rac)**

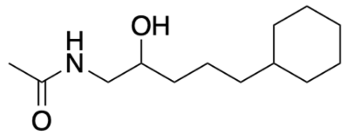

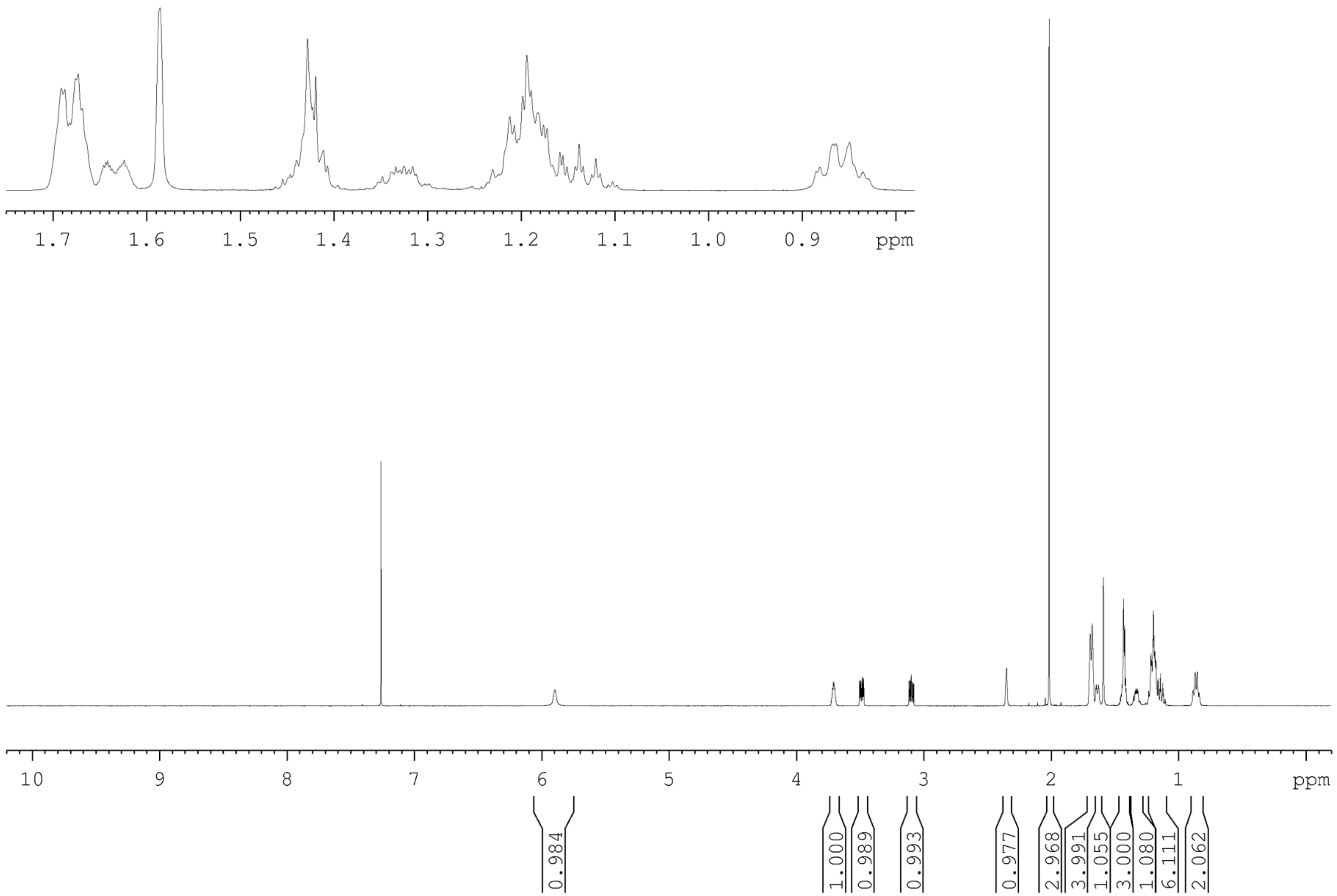

N-(5-cyclohexyl-2-hydroxypentyl)acetamide (1f-rac)

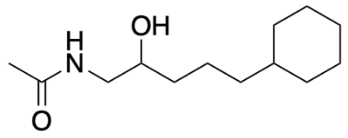

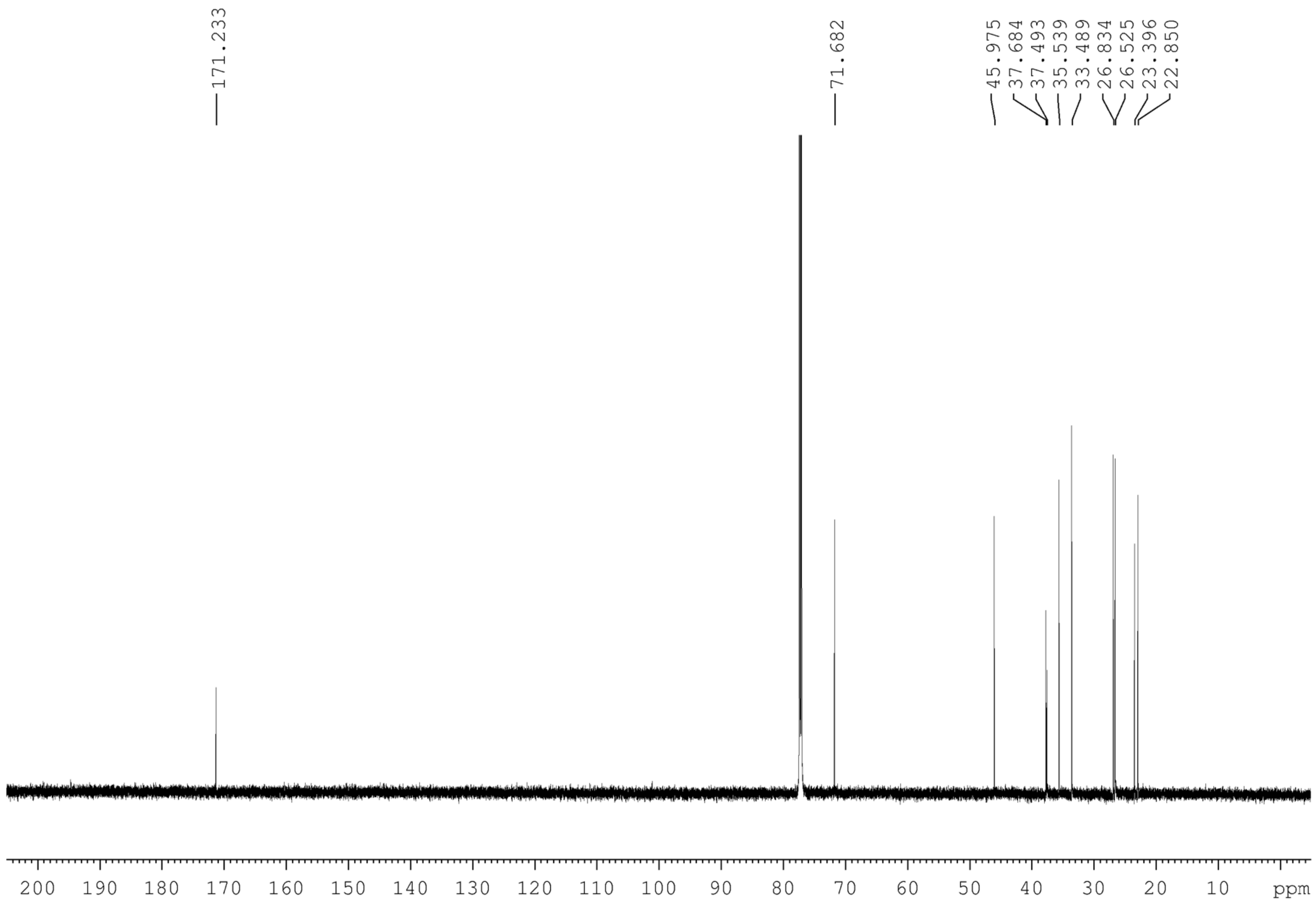

(*S*)-*N*-(5-cyclohexyl-2-hydroxypentyl)acetamide (1f)

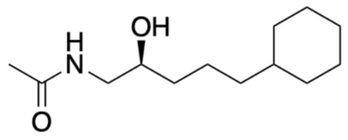

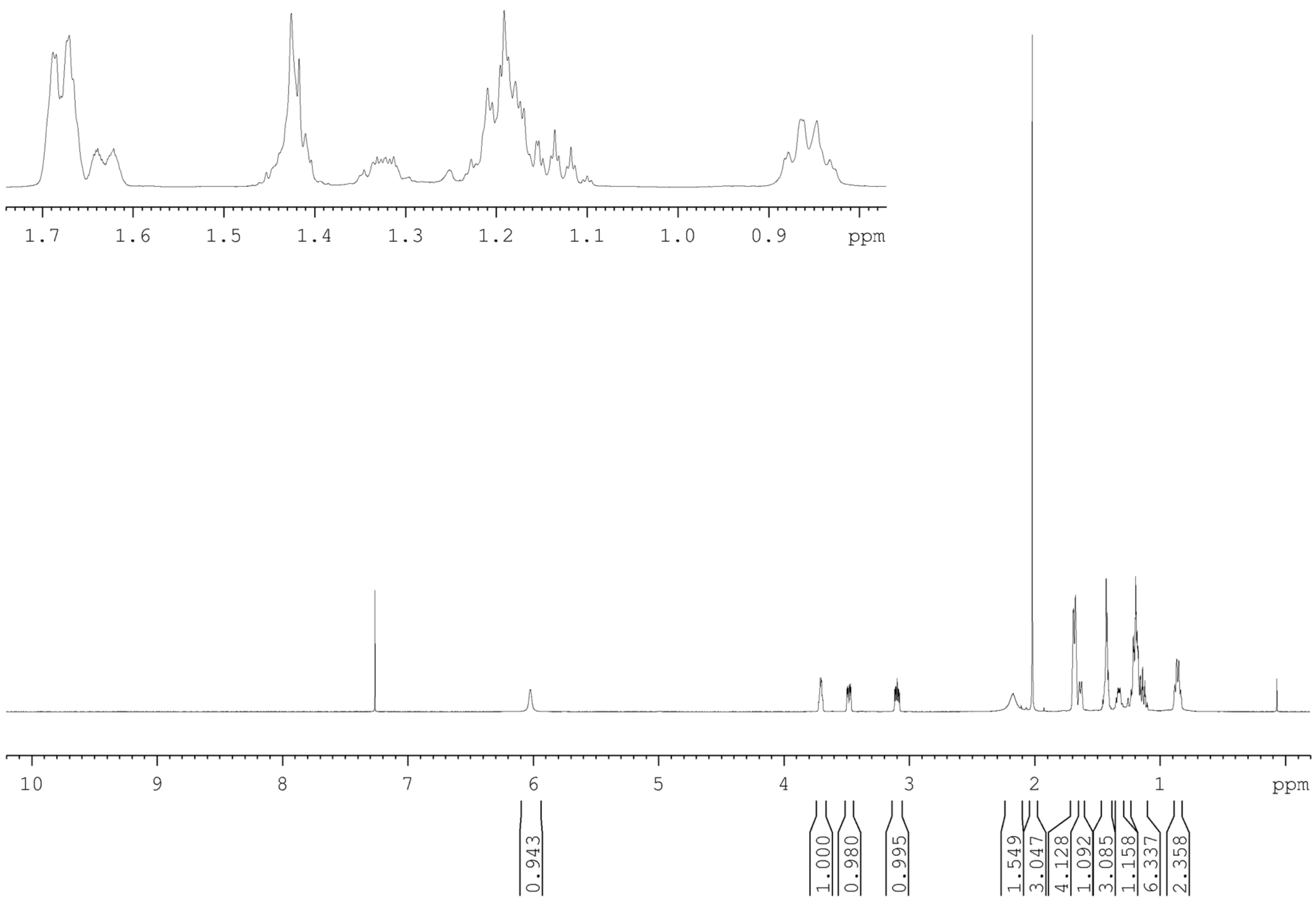

(*S*)-*N*-(5-cyclohexyl-2-hydroxypentyl)acetamide (1f)

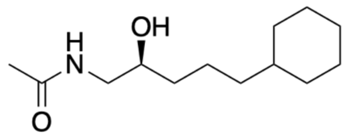

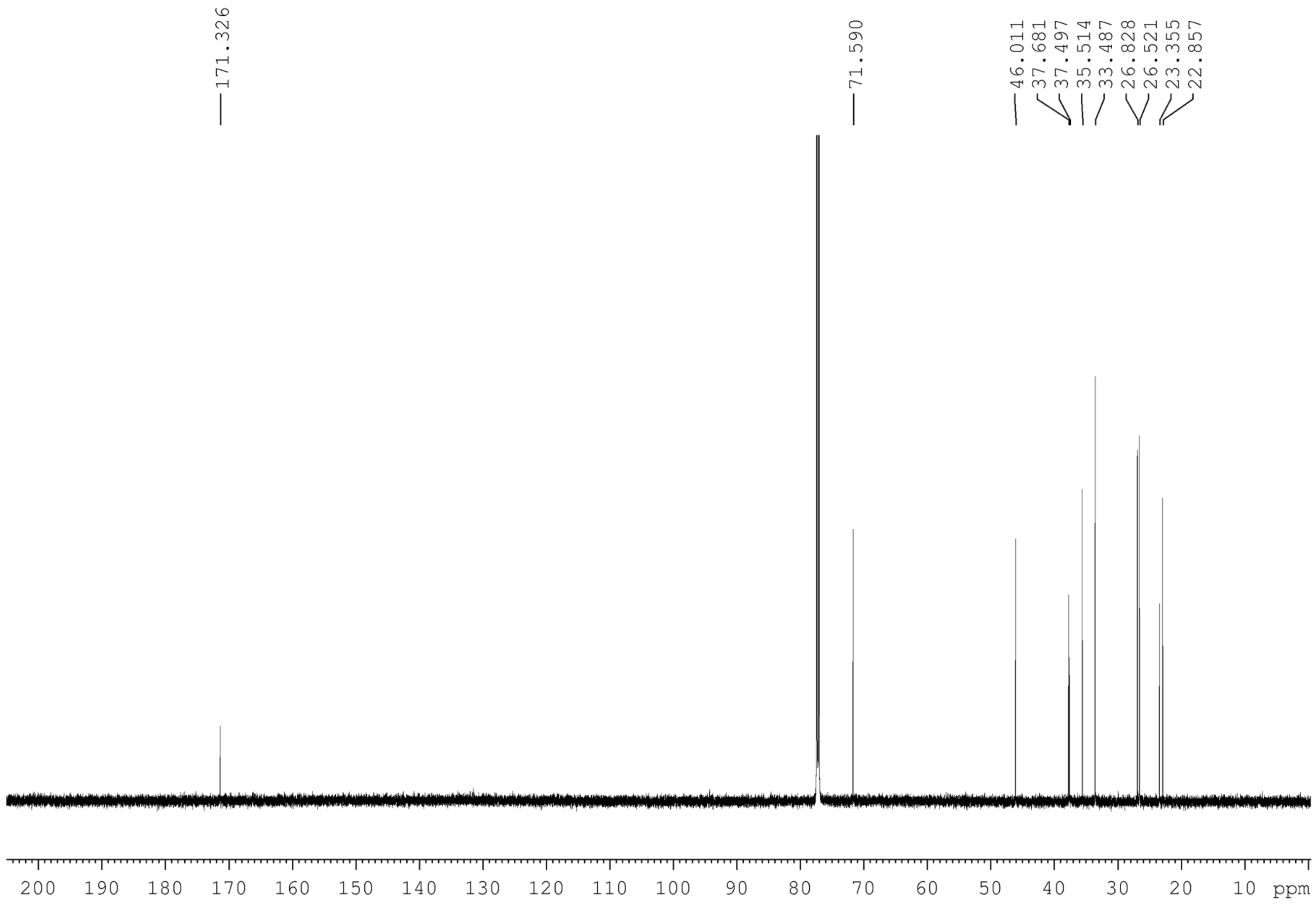

(*S*)-1-(*N*-acetylbenzamido)-5-cyclohexylpentan-2-yl benzoate (1f-Bz)

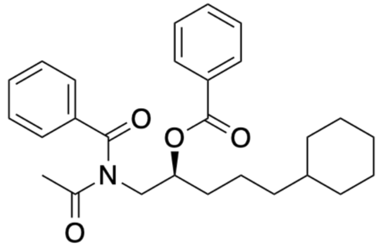

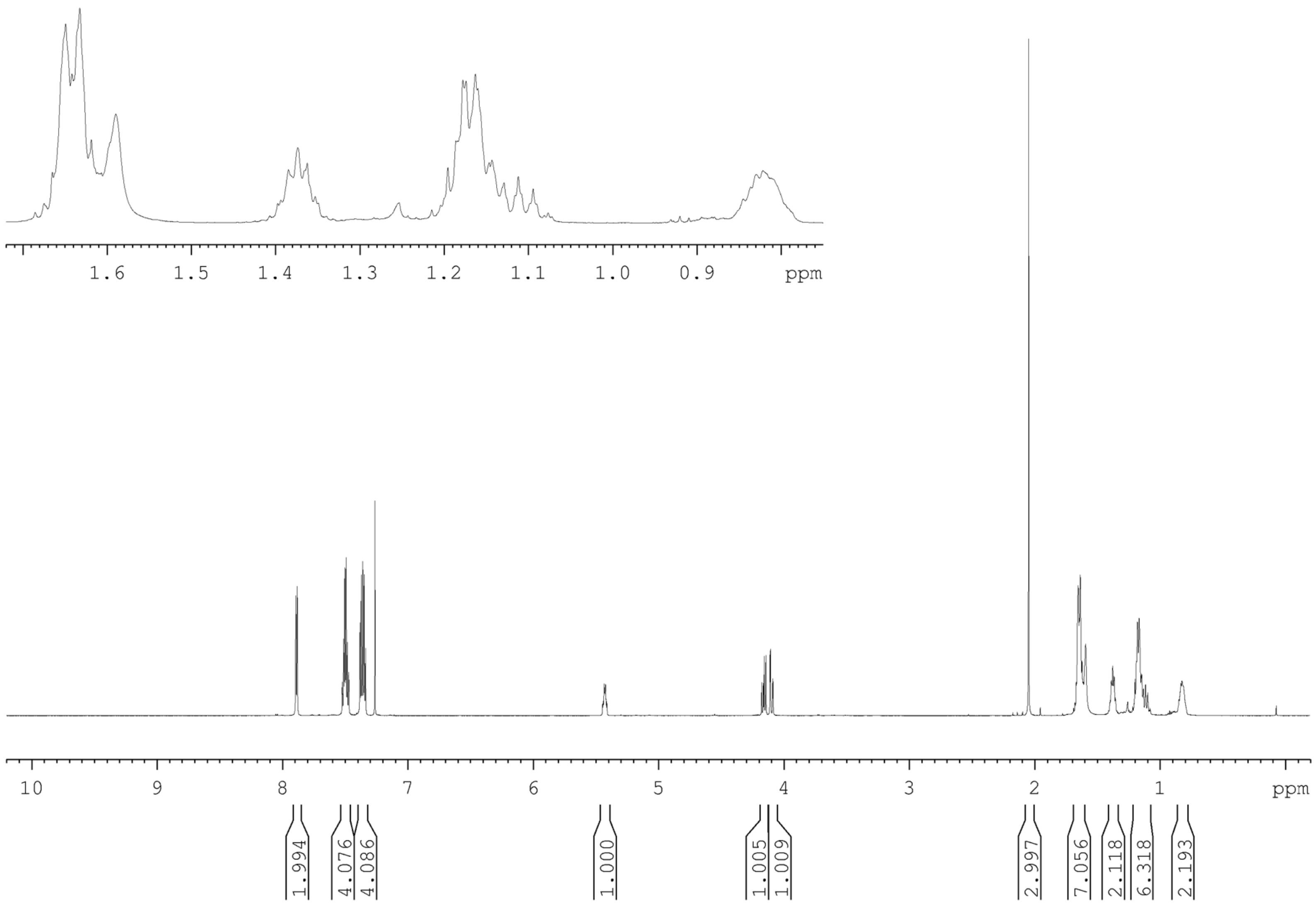

(*S*)-1-(*N*-acetylbenzamido)-5-cyclohexylpentan-2-yl benzoate (1f-Bz)

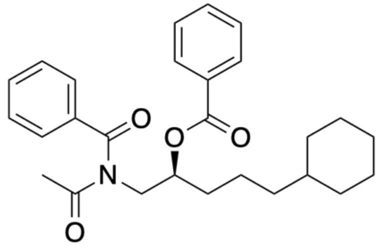

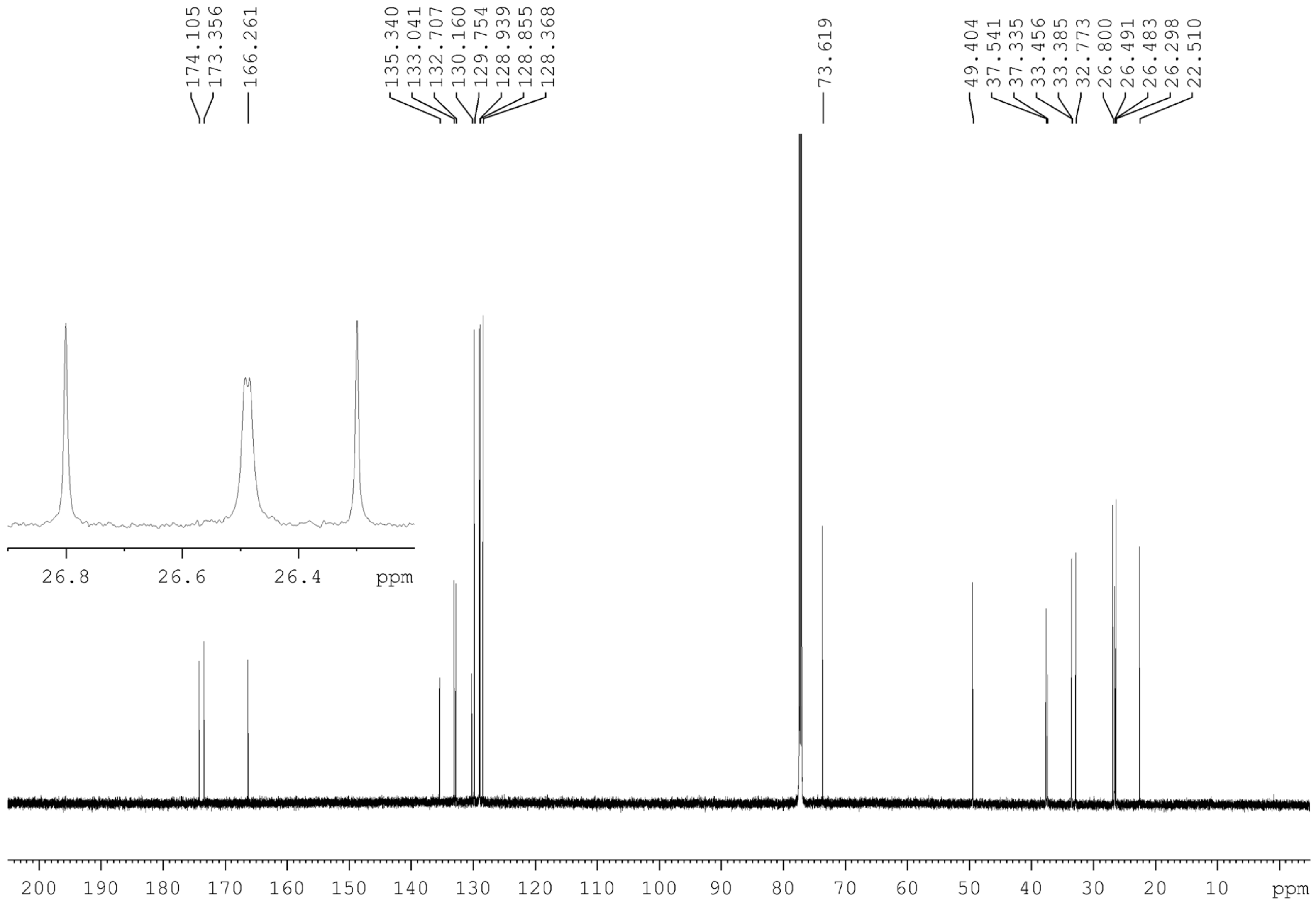

(*R*)-*N*-(5-cyclohexyl-2-hydroxypentyl)acetamide ((*R*)-1f)

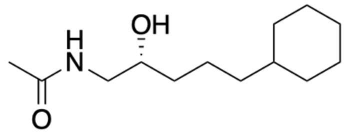

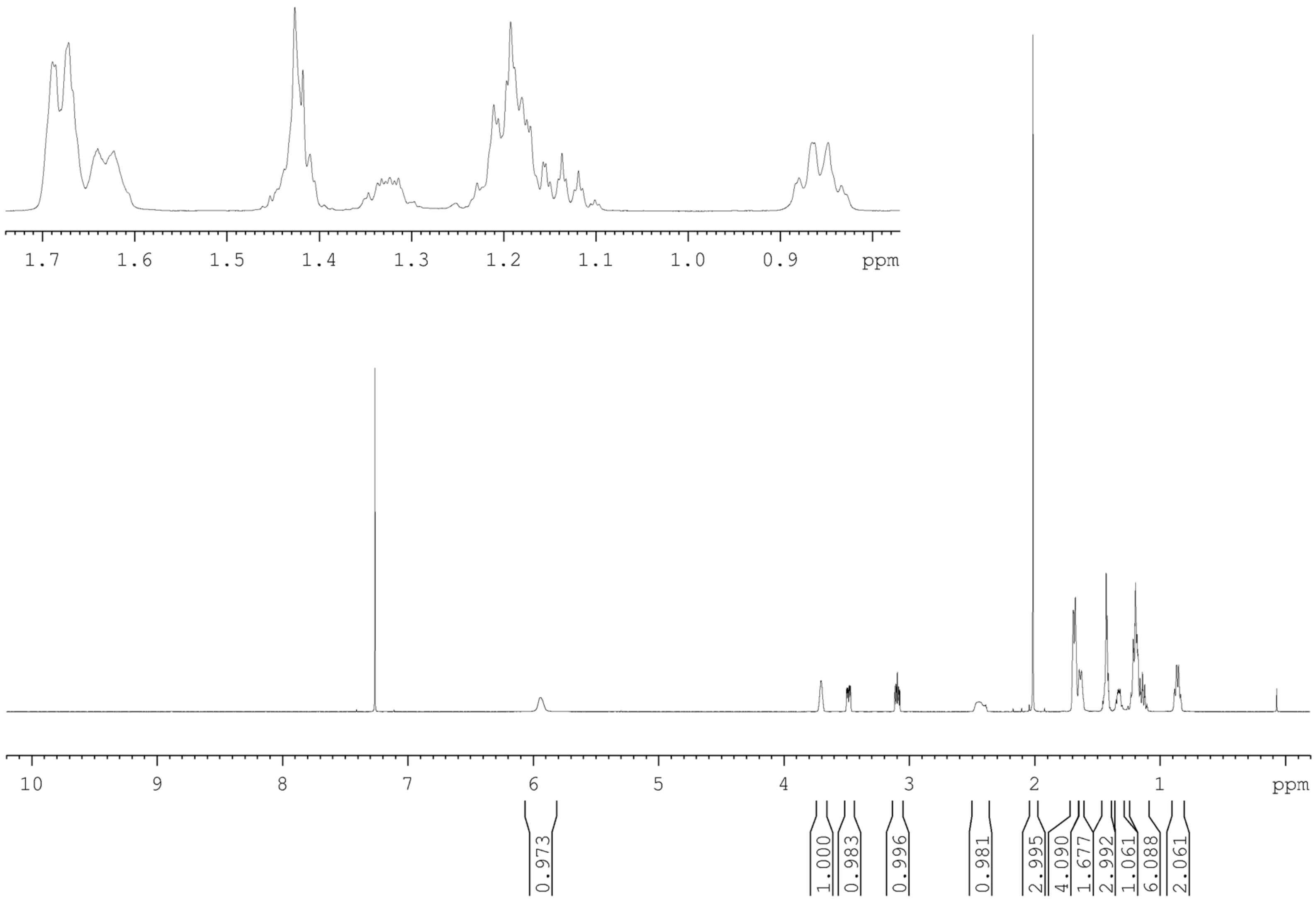

(*R*)-*N*-(5-cyclohexyl-2-hydroxypentyl)acetamide ((*R*)-1f)

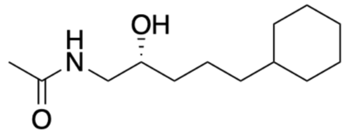

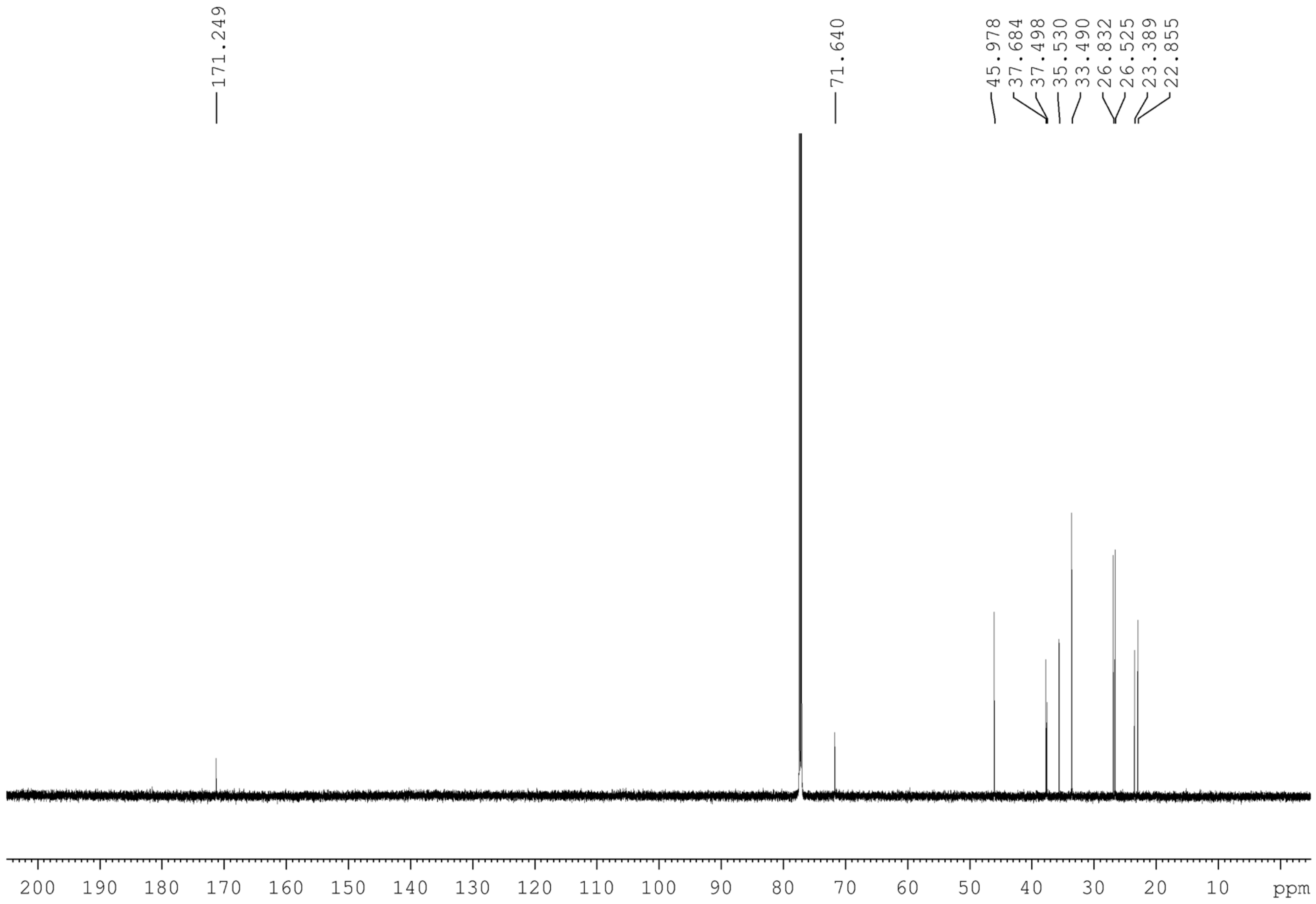

(*R*)-1-(*N*-acetylbenzamido)-5-cyclohexylpentan-2-yl benzoate ((*R*)-1f-Bz)

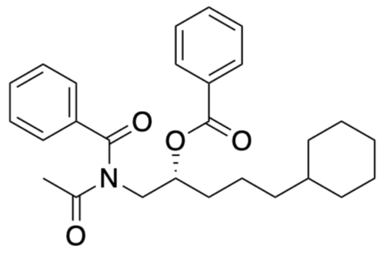

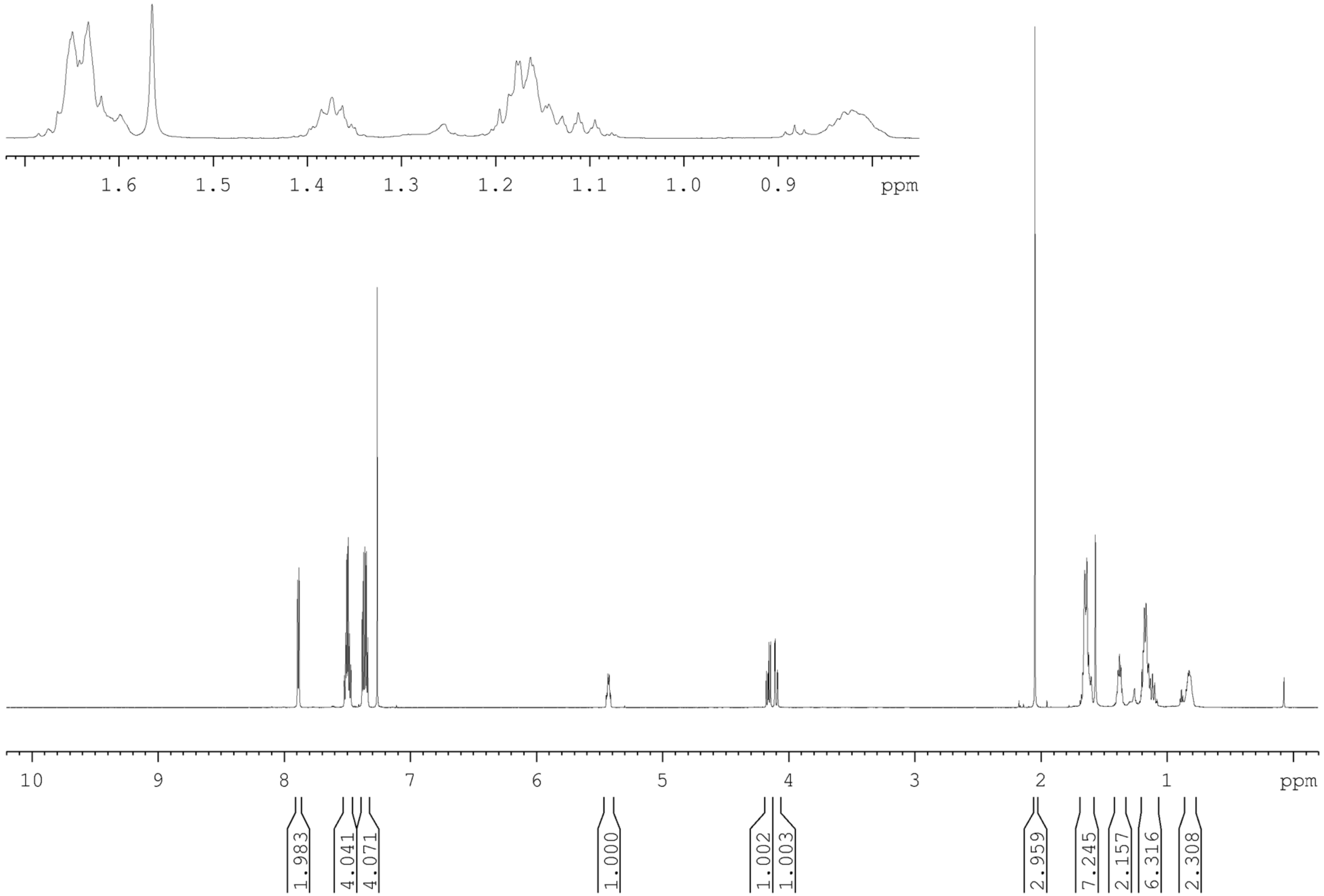

(*R*)-1-(*N*-acetylbenzamido)-5-cyclohexylpentan-2-yl benzoate ((*R*)-1f-Bz)

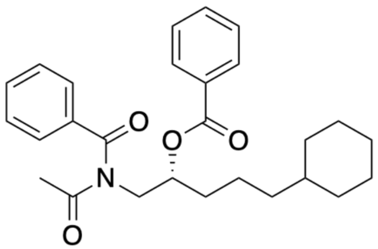

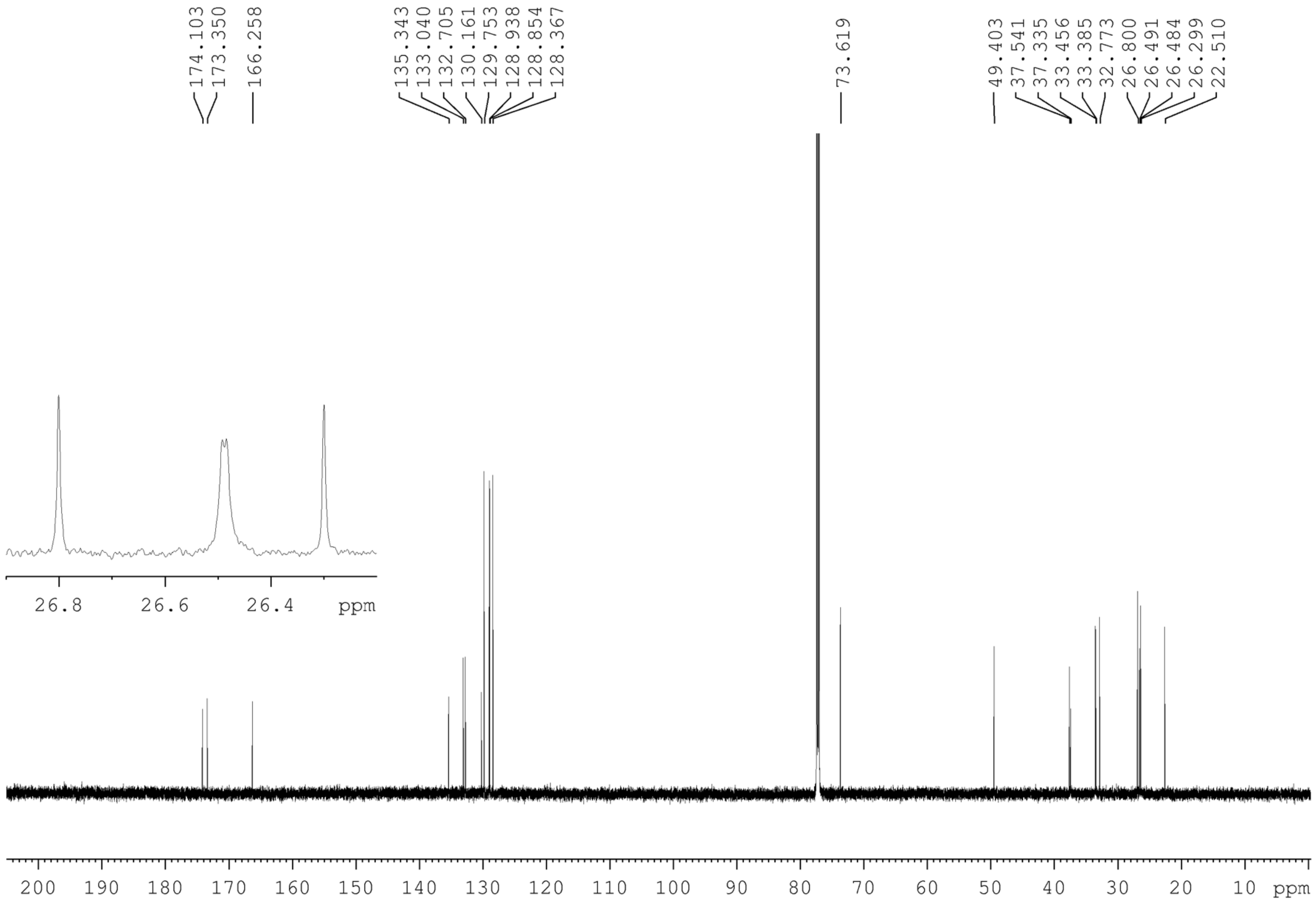

1-amino-3-phenylpropan-2-ol (1g-I_1)

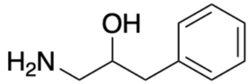

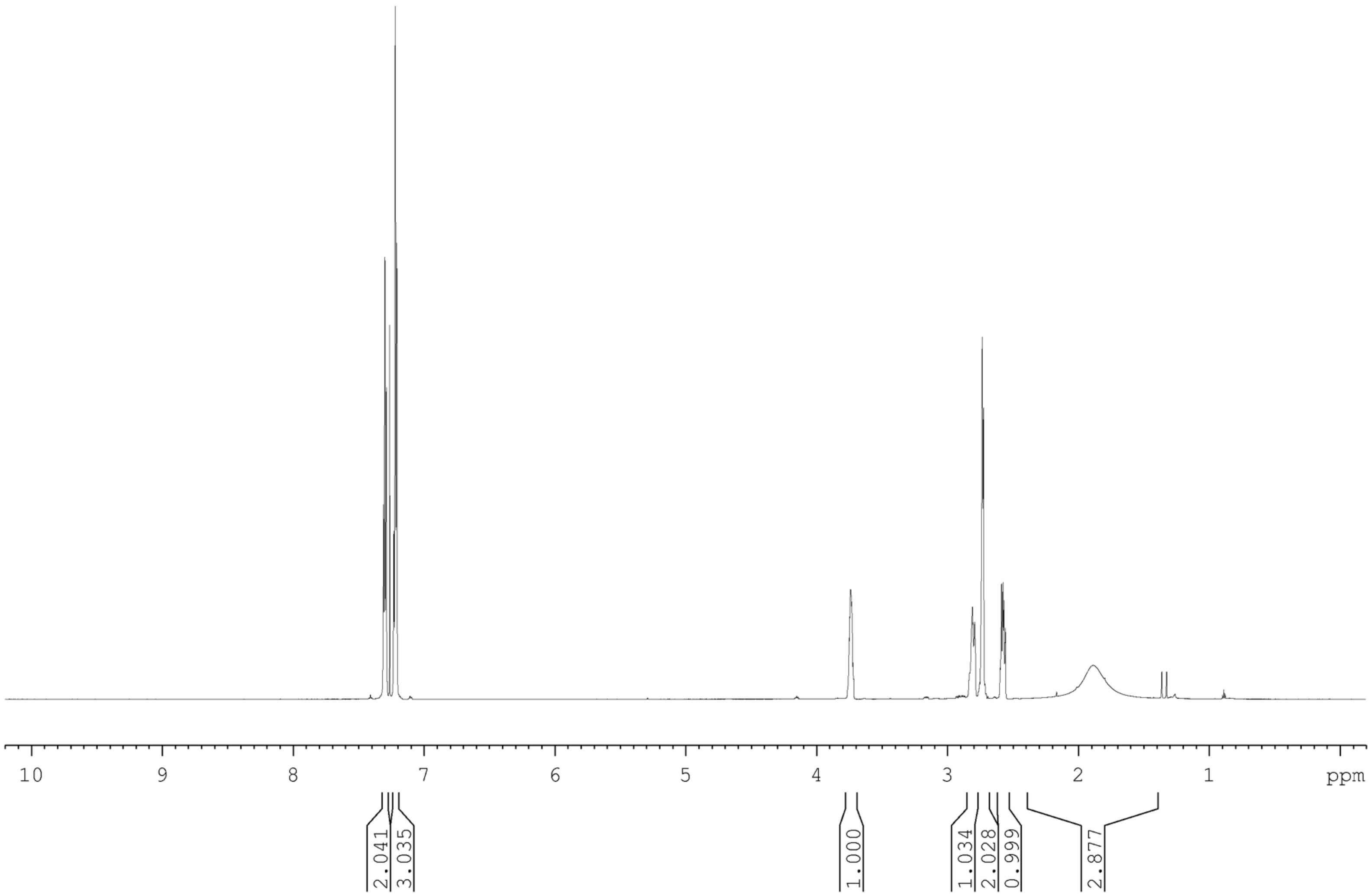

1-amino-3-phenylpropan-2-ol (1g-I_1)

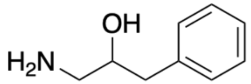

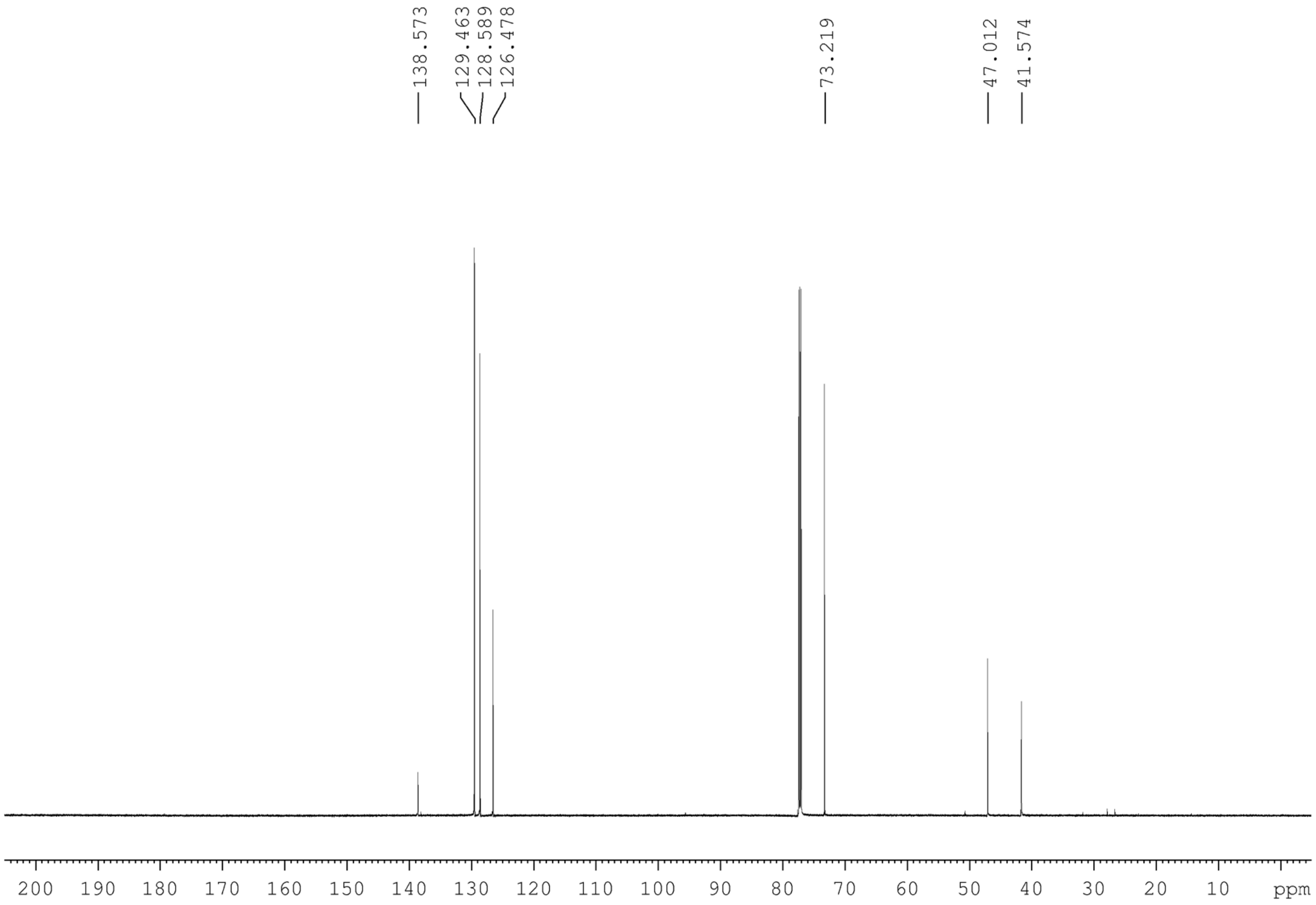

N-(2-hydroxy-3-phenylpropyl)acetamide (1g-rac)

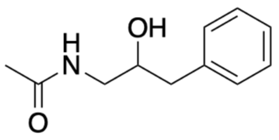

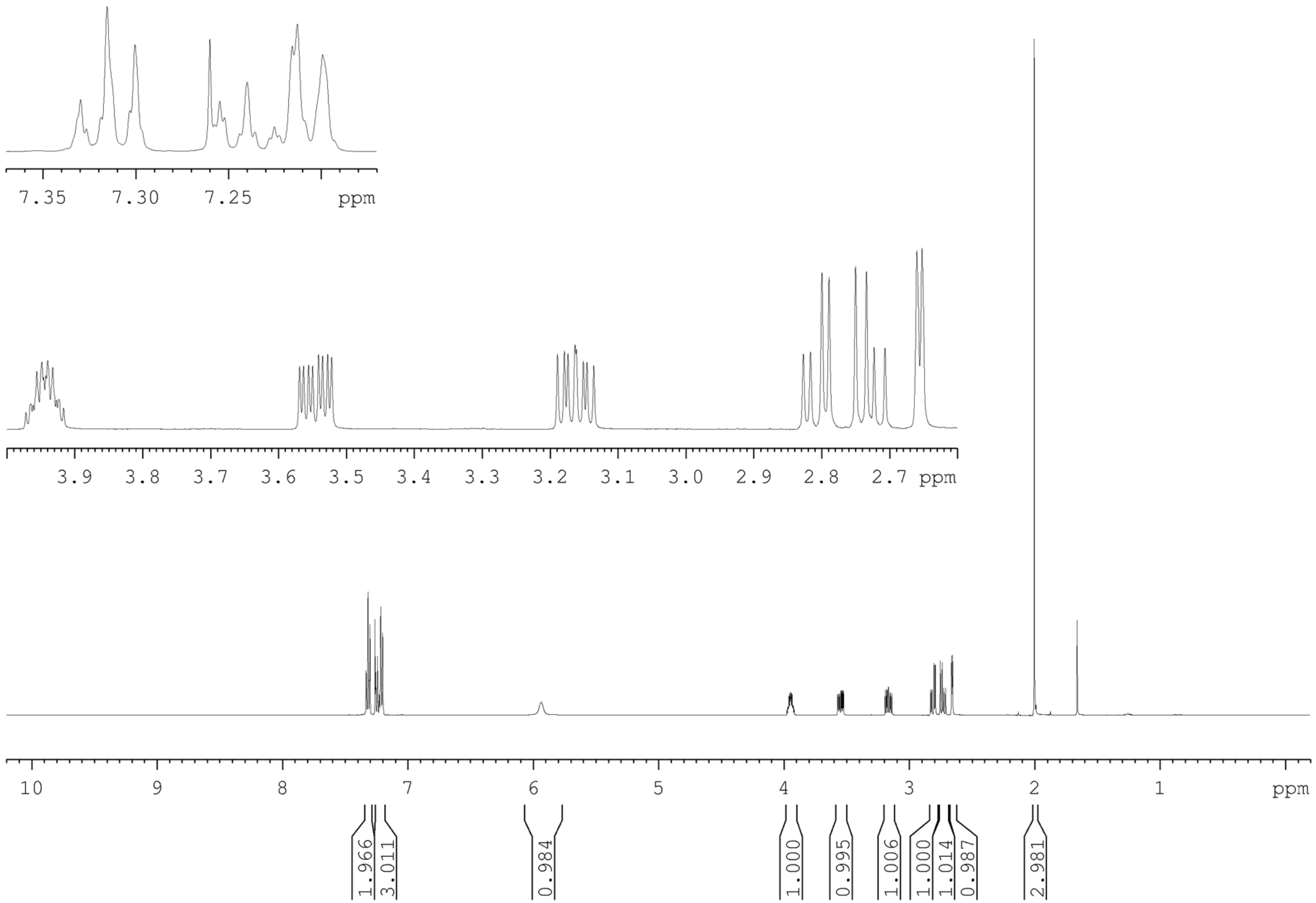

N-(2-hydroxy-3-phenylpropyl)acetamide (1g-rac)

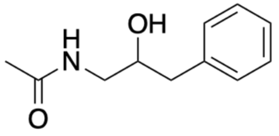

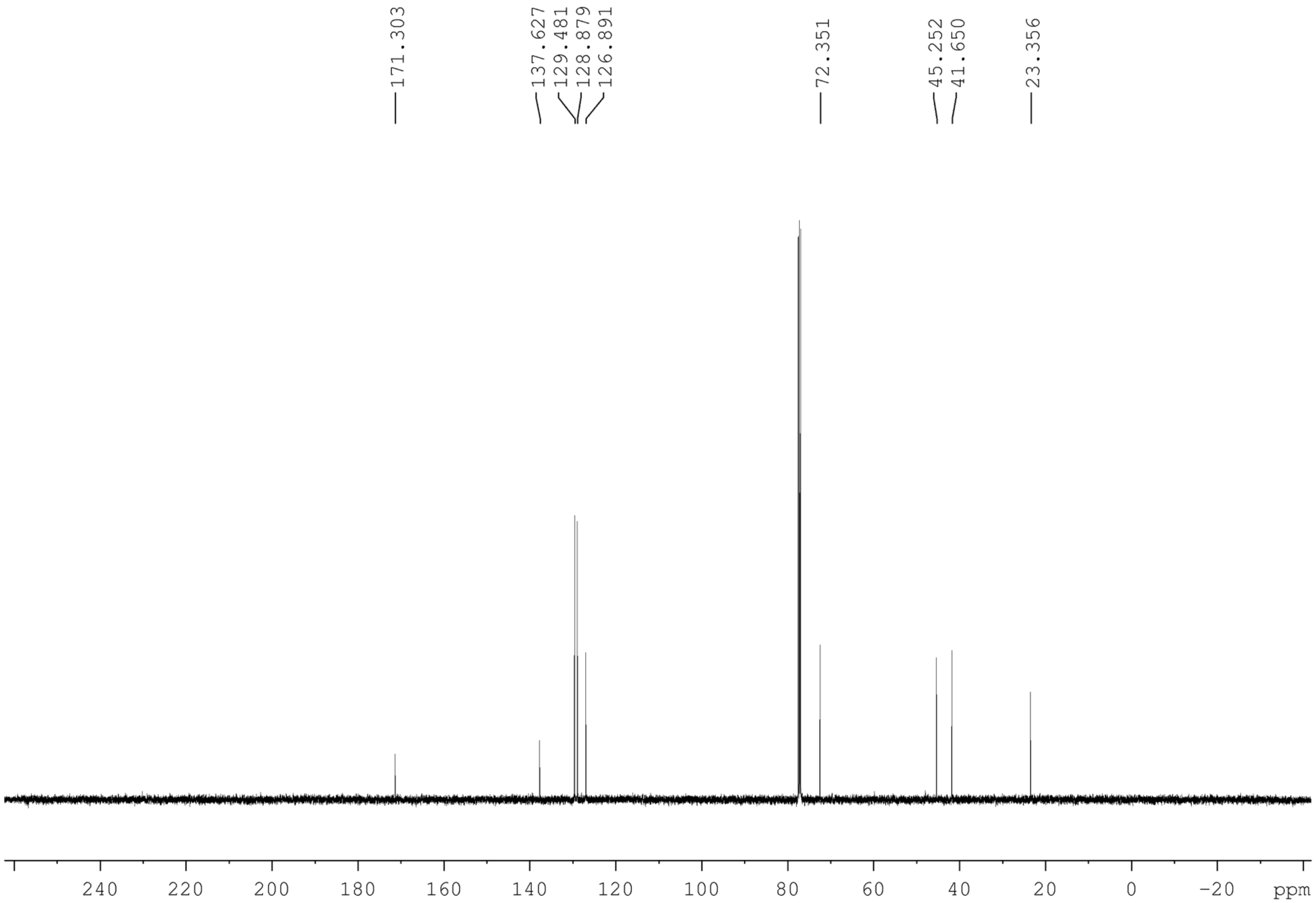

(*S*)-*N*-(2-hydroxy-3-phenylpropyl)acetamide (1g)

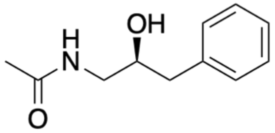

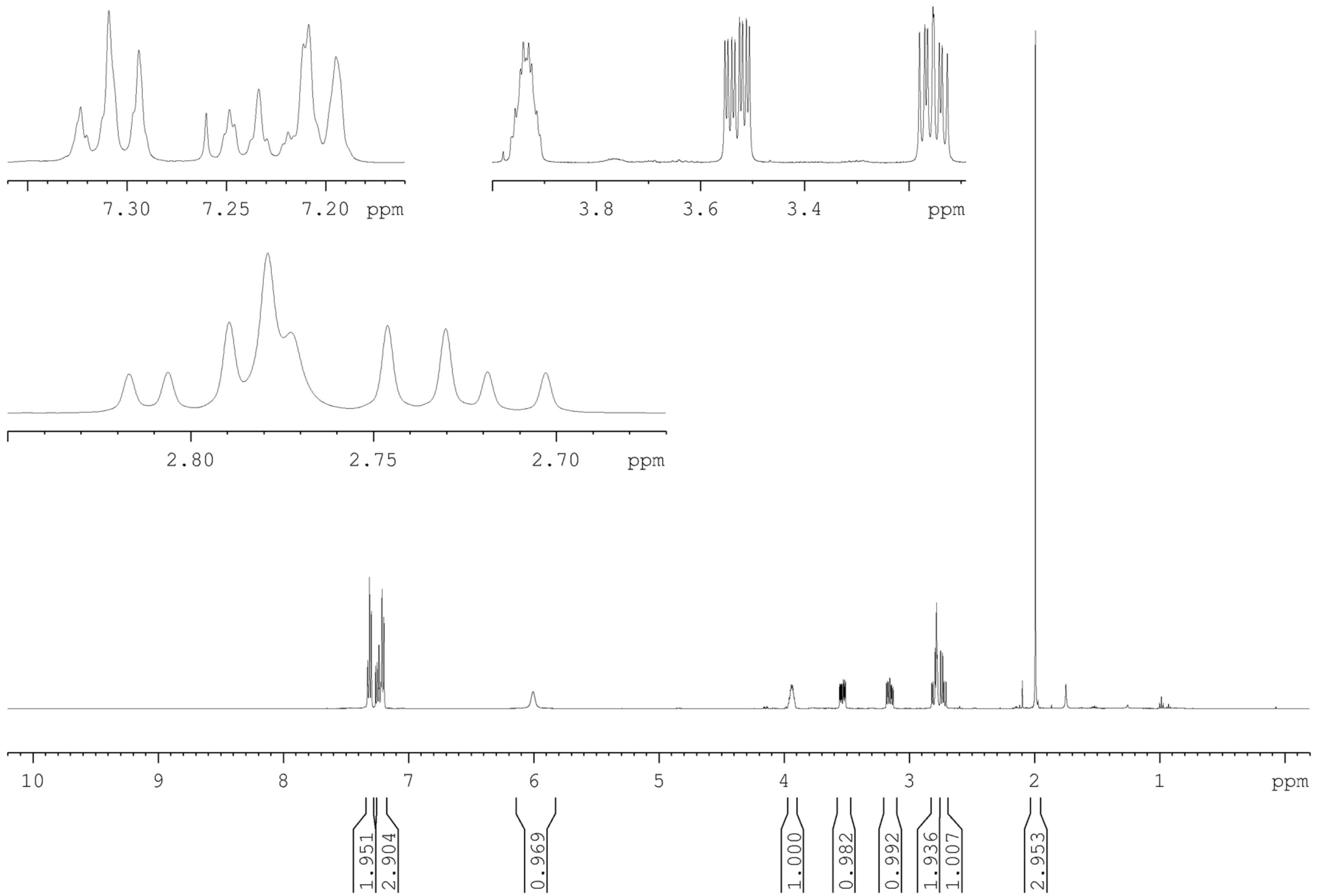

(*S*)-*N*-(2-hydroxy-3-phenylpropyl)acetamide (1g)

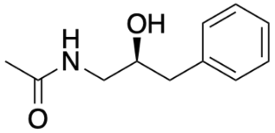

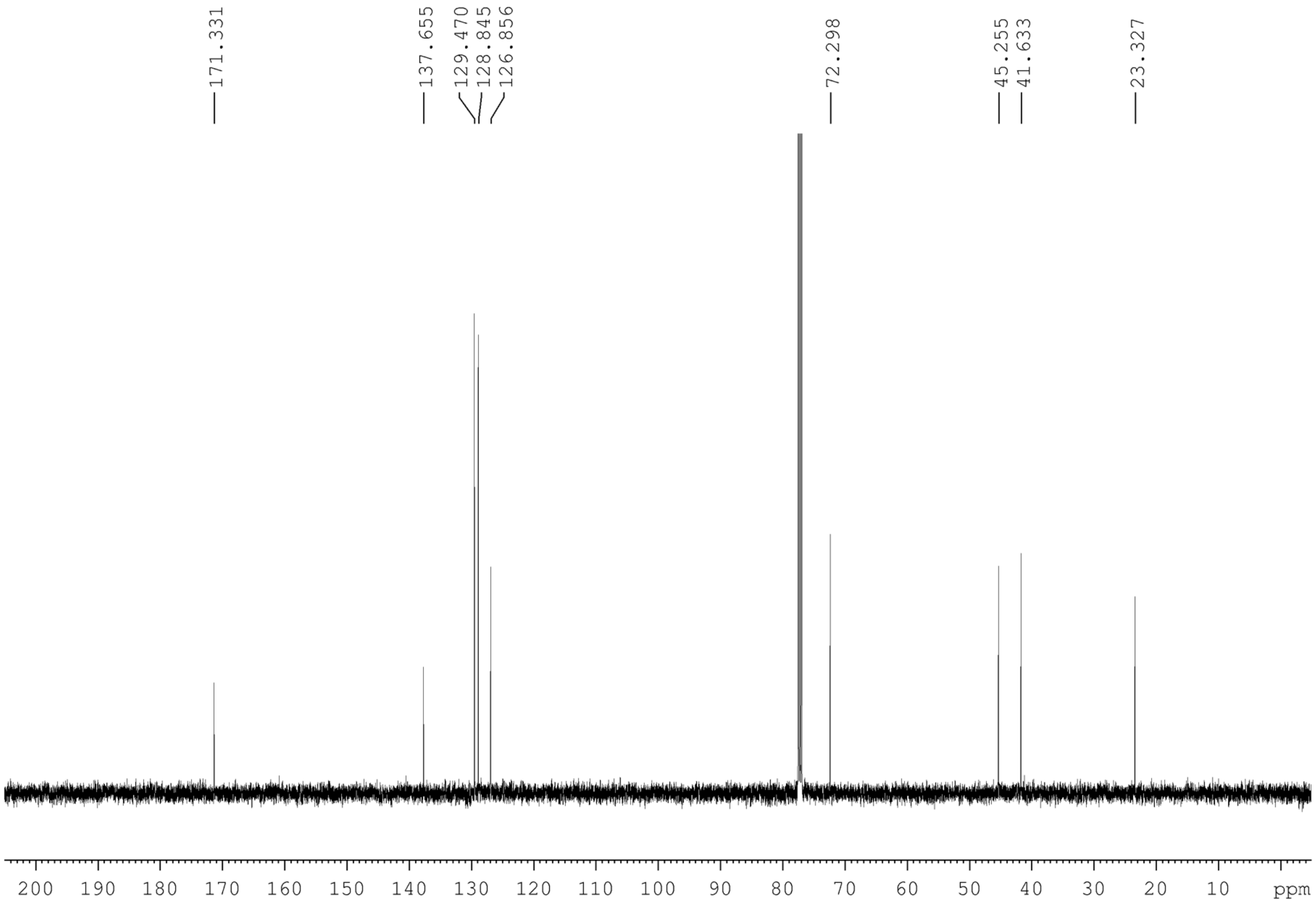

1-amino-4-phenylbutan-2-ol (1h-I_1)

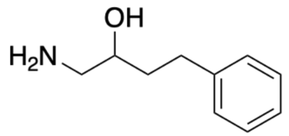

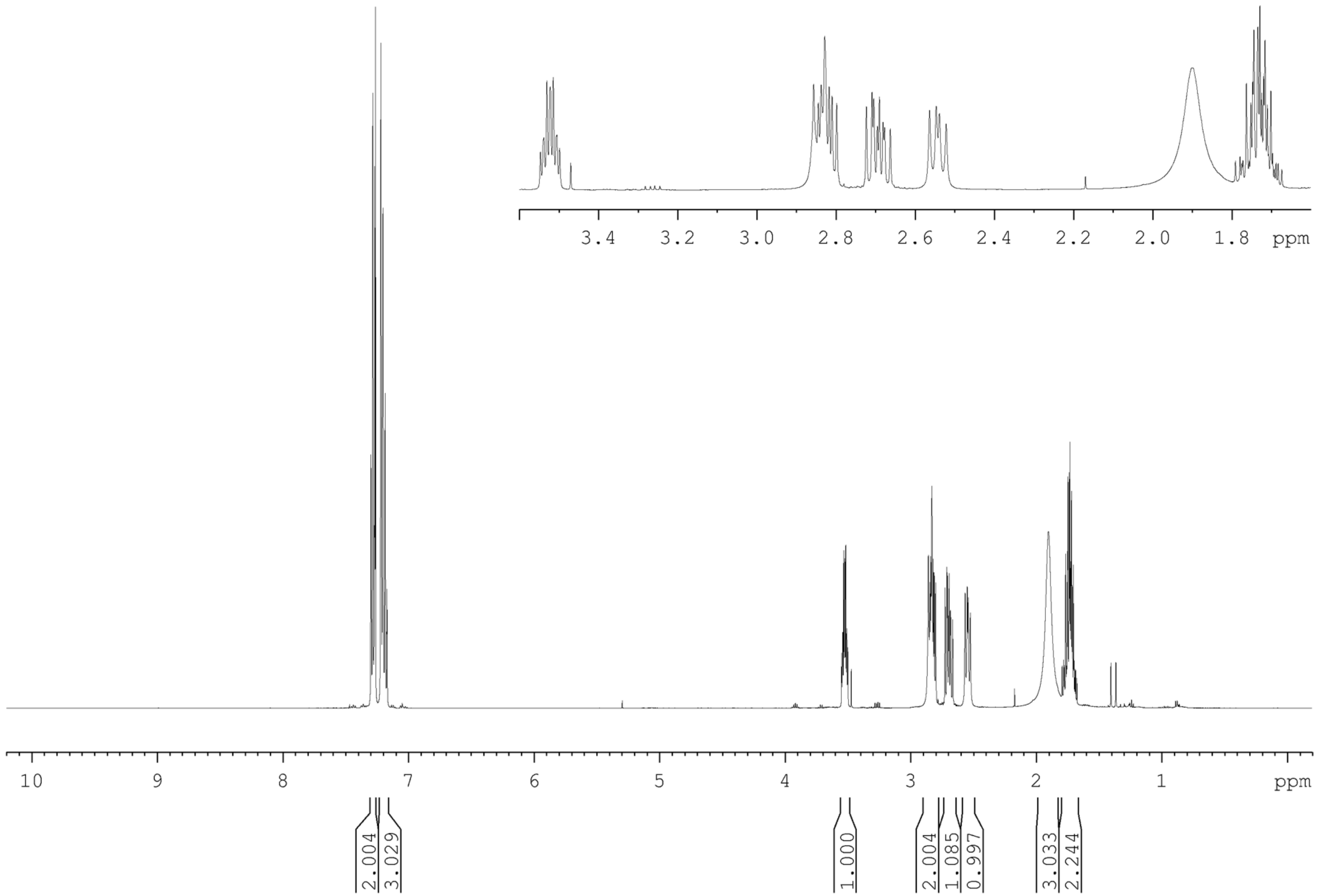

1-amino-4-phenylbutan-2-ol (1h-I_1)

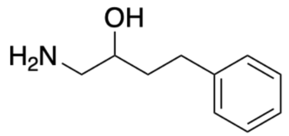

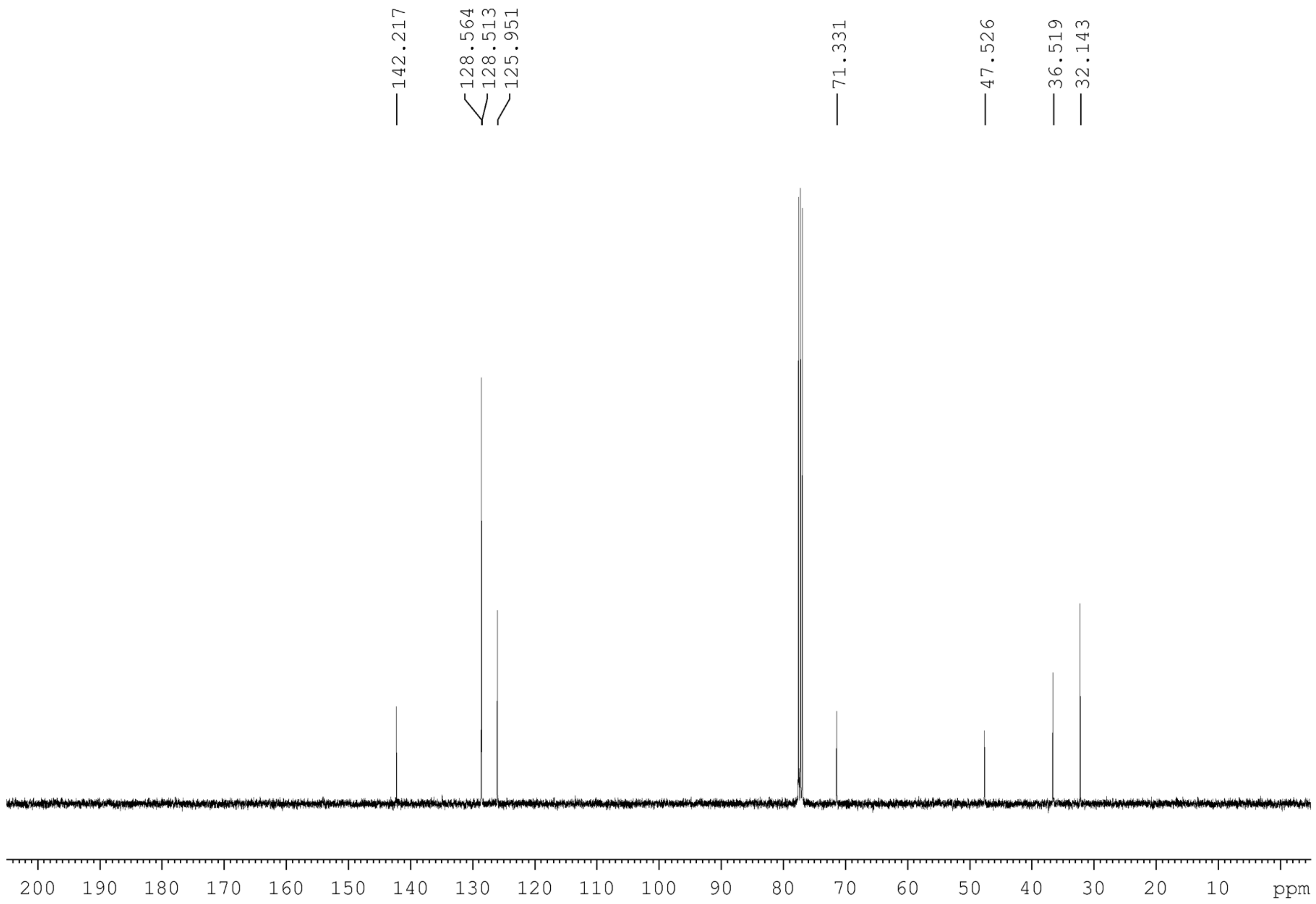

N-(2-hydroxy-4-phenylbutyl)acetamide (1h-rac)

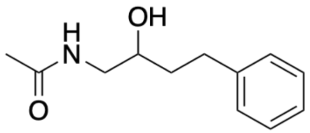

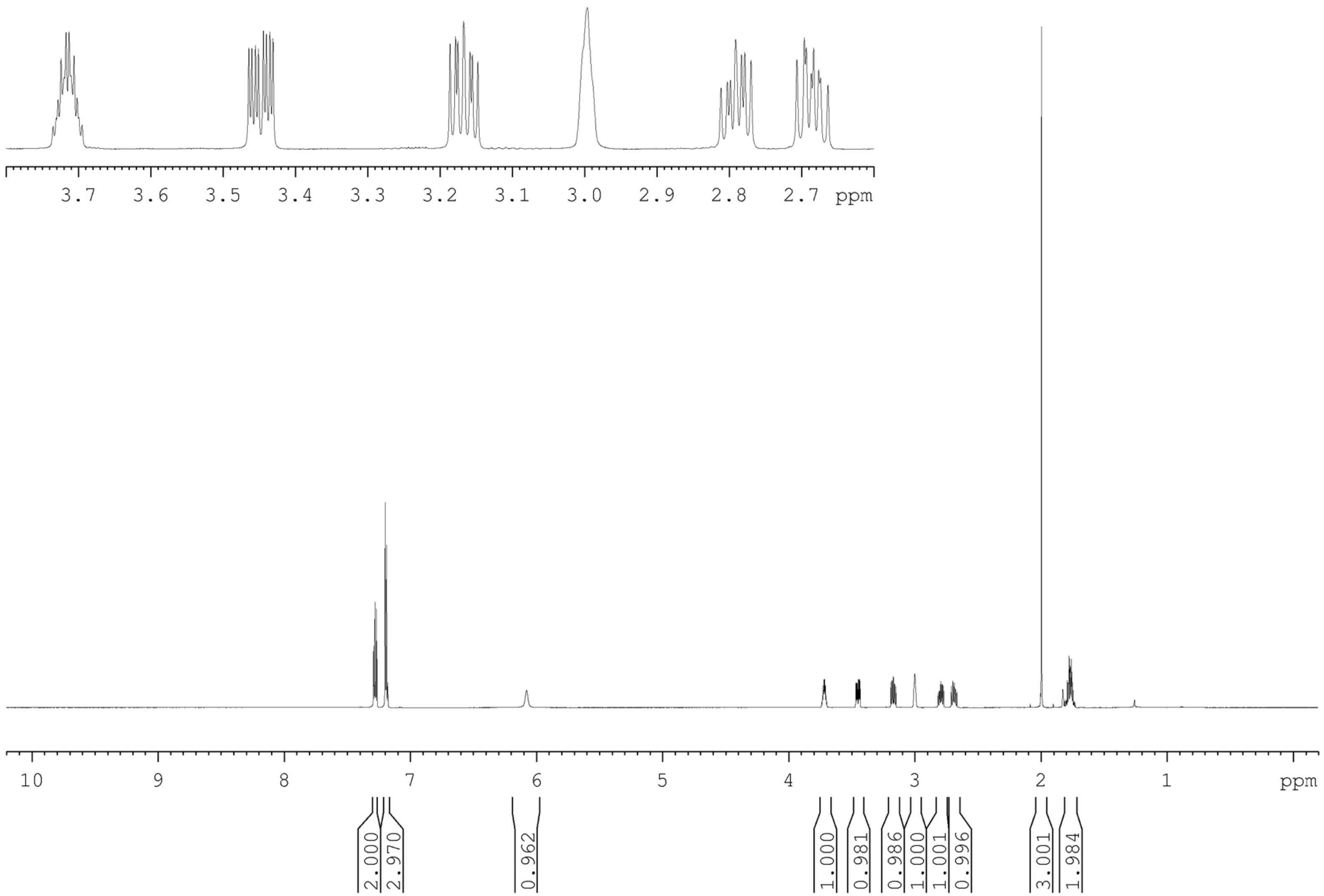

N-(2-hydroxy-4-phenylbutyl)acetamide (1h-rac)

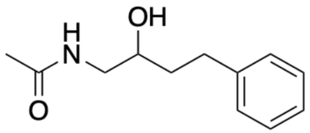

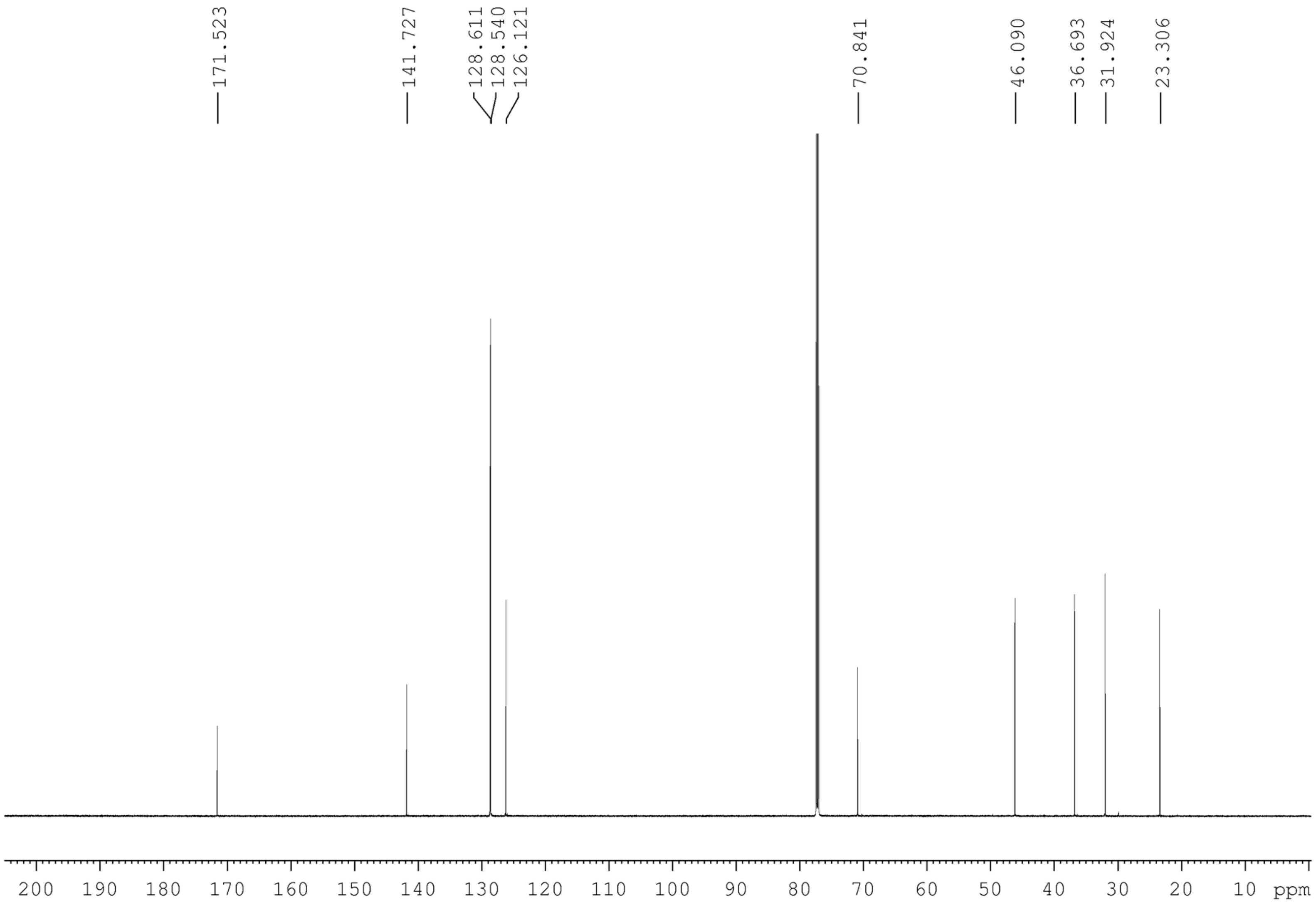

(*S*)-*N*-(2-hydroxy-4-phenylbutyl)acetamide (1h)

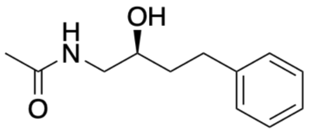

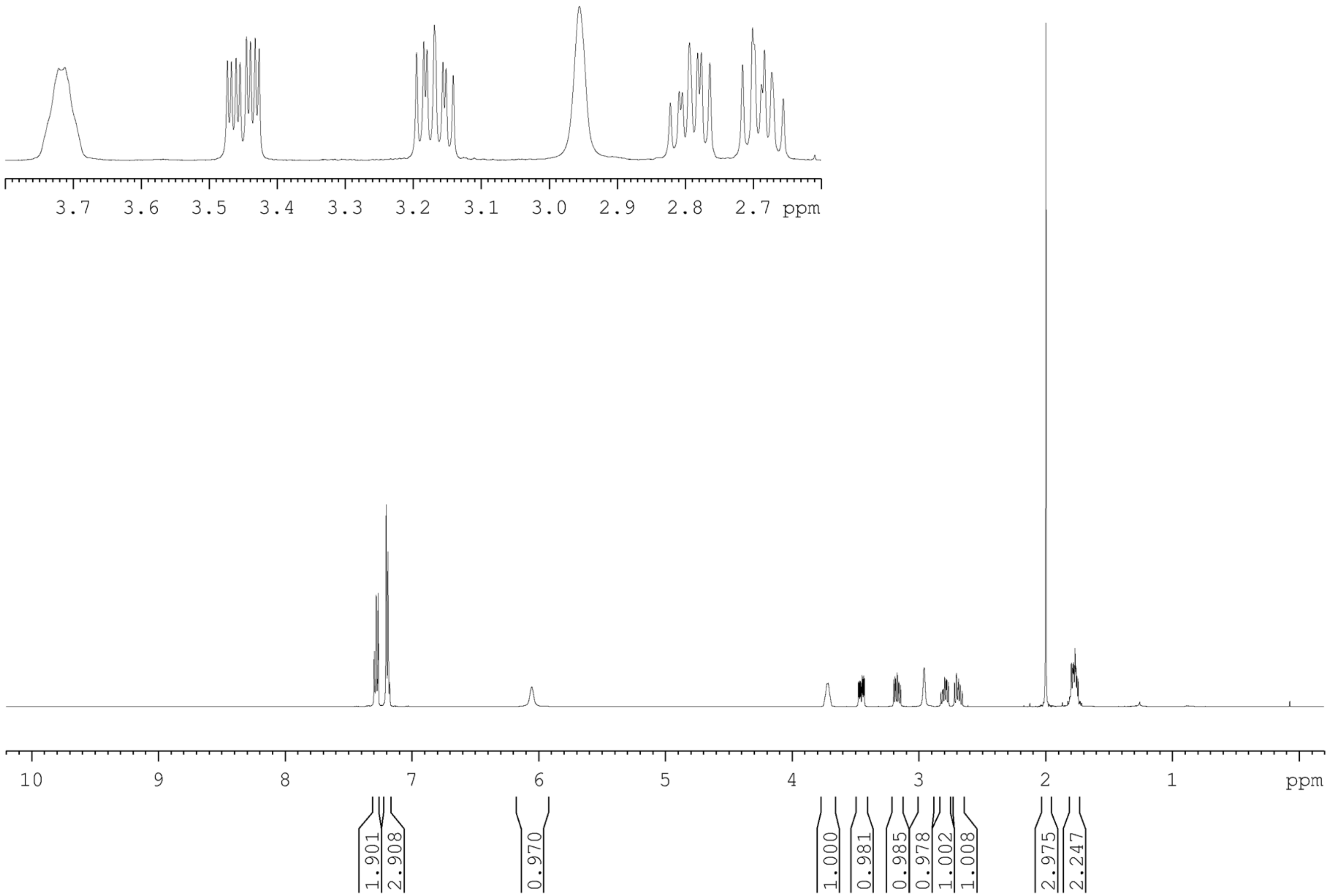

(*S*)-*N*-(2-hydroxy-4-phenylbutyl)acetamide (1h)

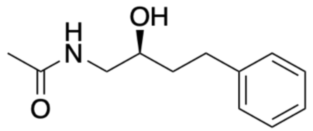

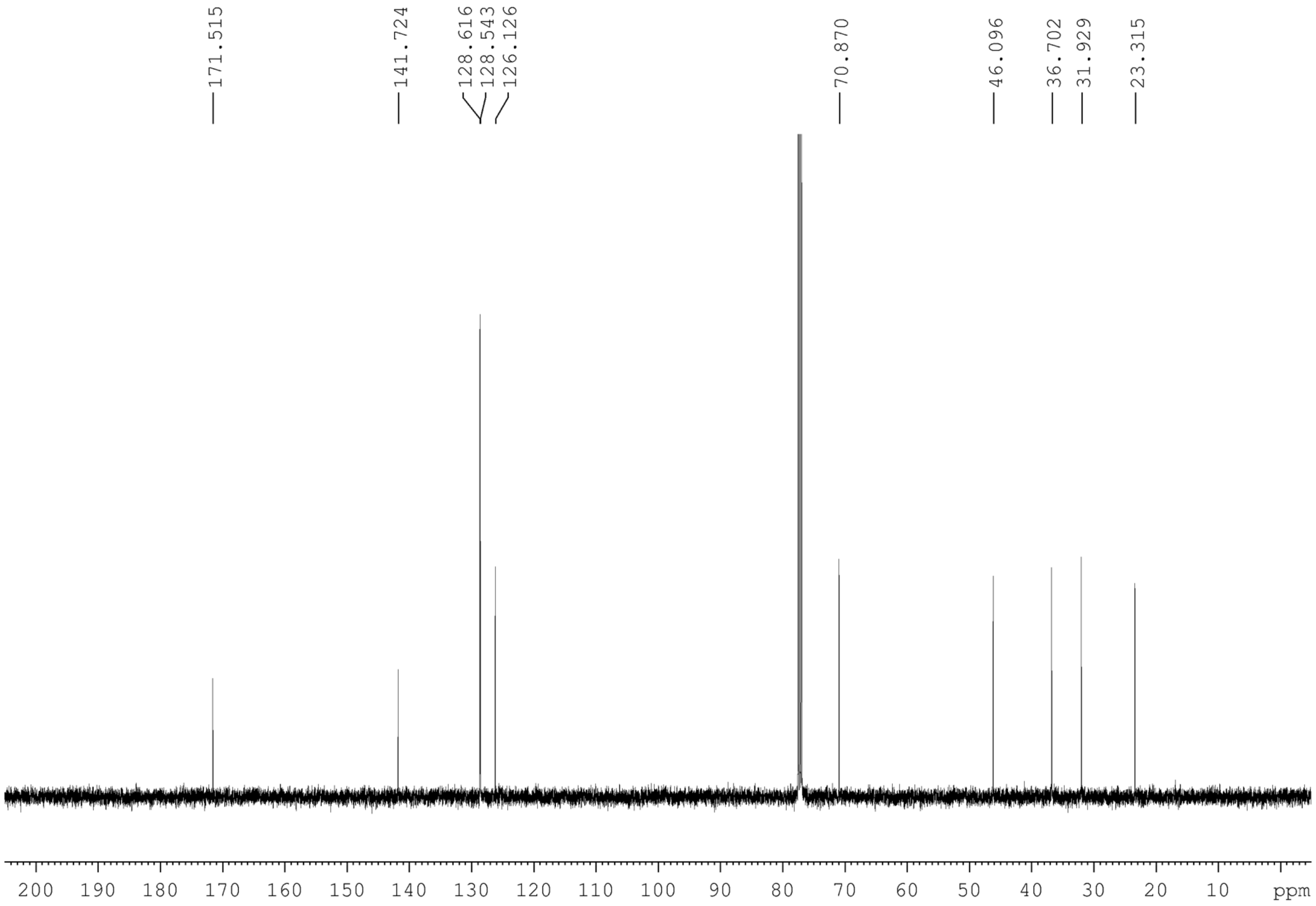

1-amino-5-phenylpentan-2-ol (1i-I_1)

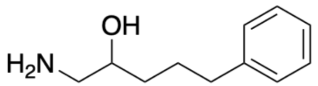

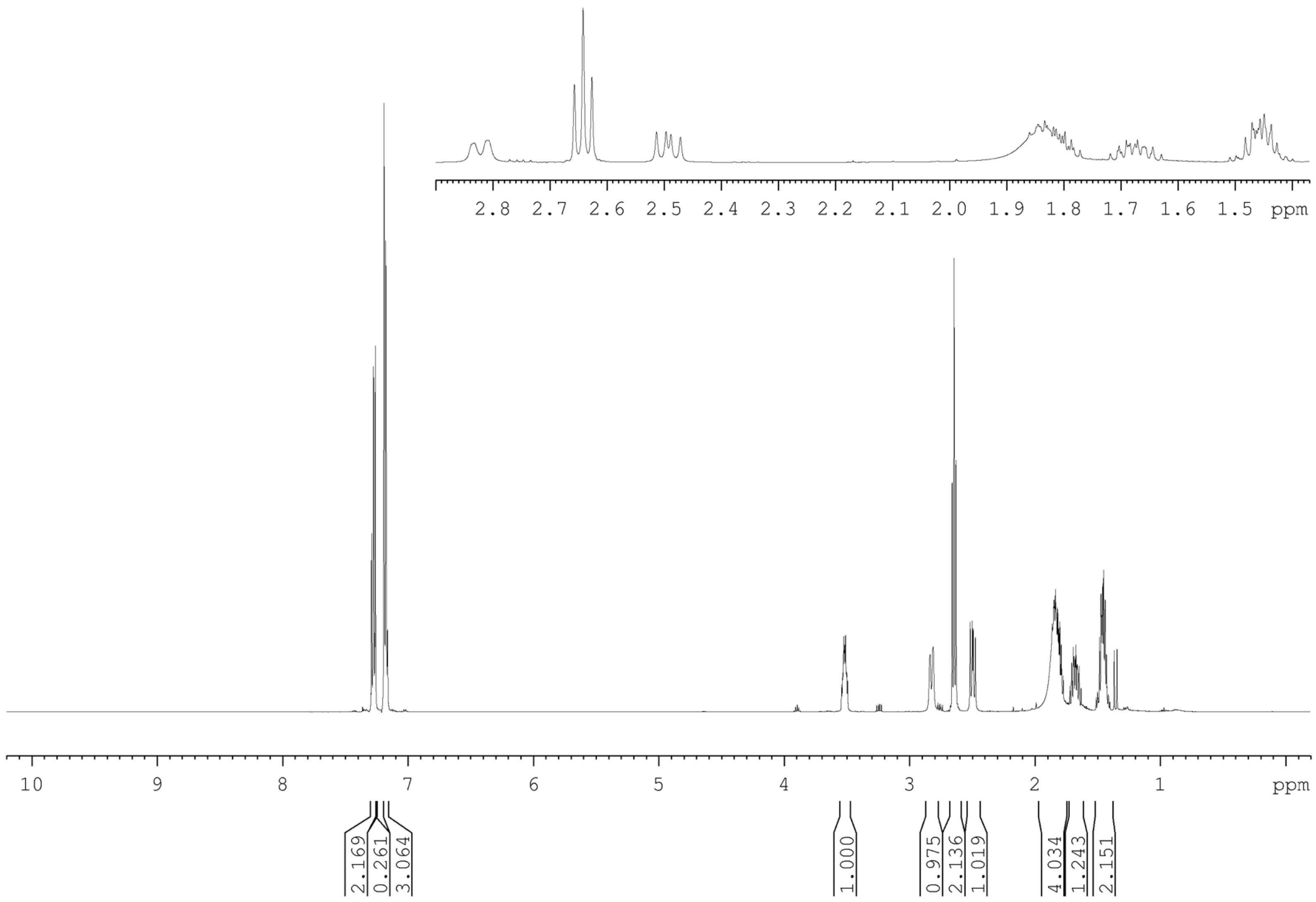

1-amino-5-phenylpentan-2-ol (1i-I_1)

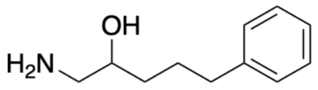

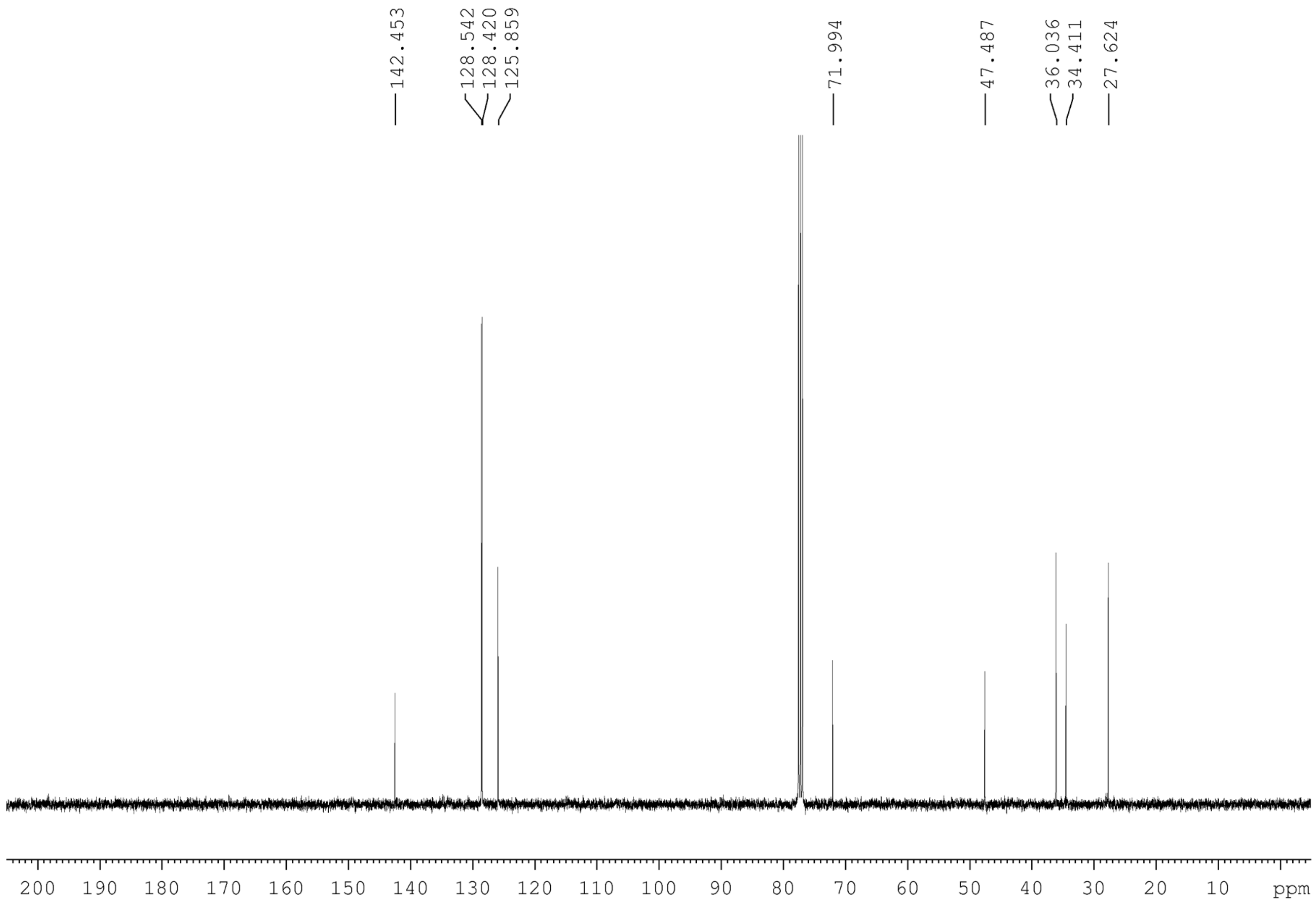

N-(2-hydroxy-5-phenylpentyl)acetamide (1i-rac)

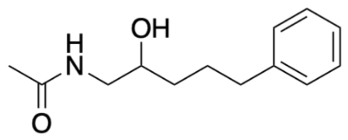

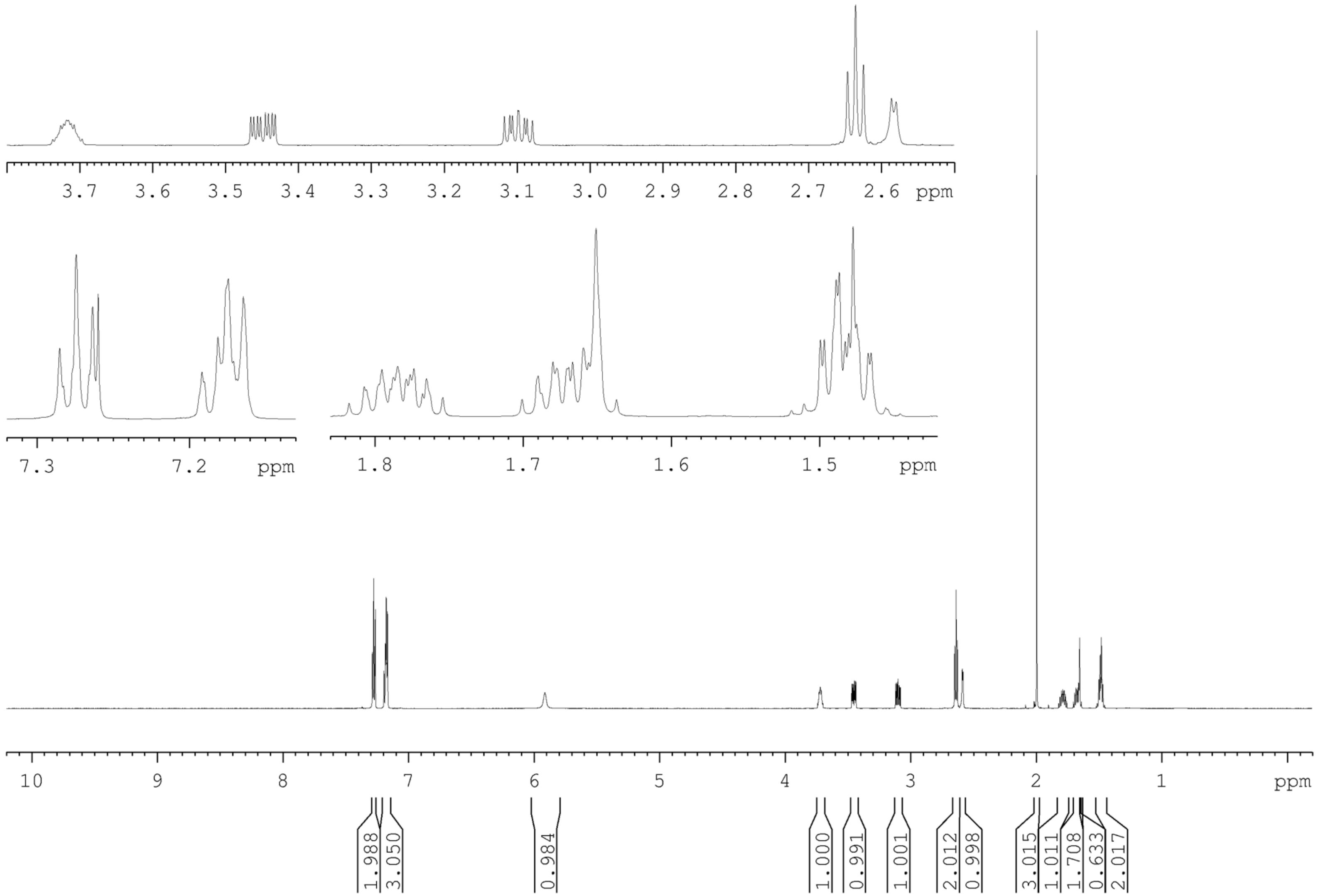

***N*-(2-hydroxy-5-phenylpentyl)acetamide (1i-rac)**

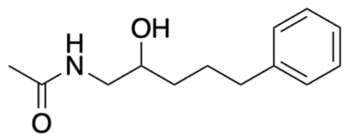

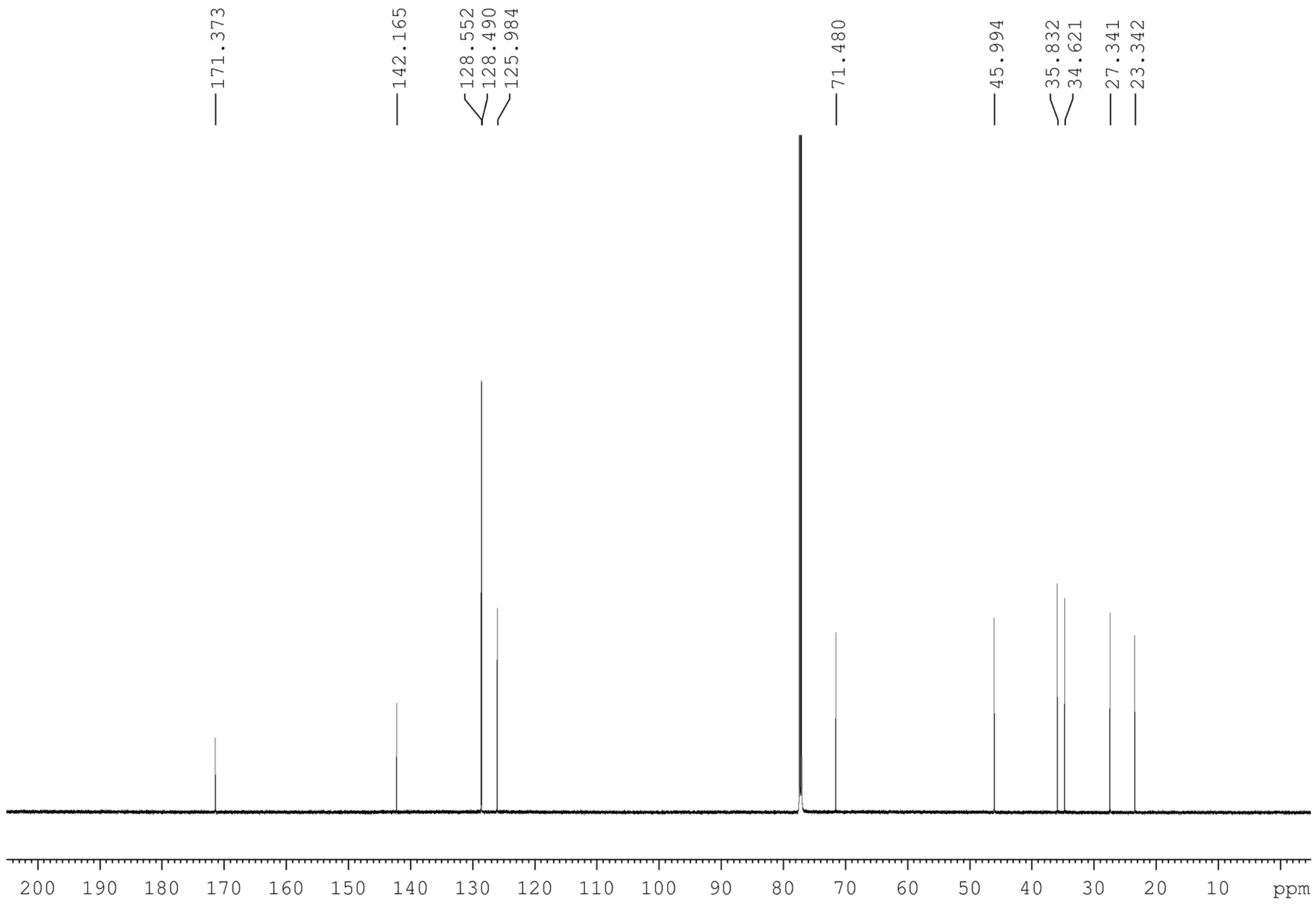

(*S*)-*N*-(2-hydroxy-5-phenylpentyl)acetamide (1i)

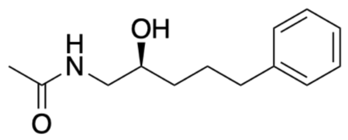

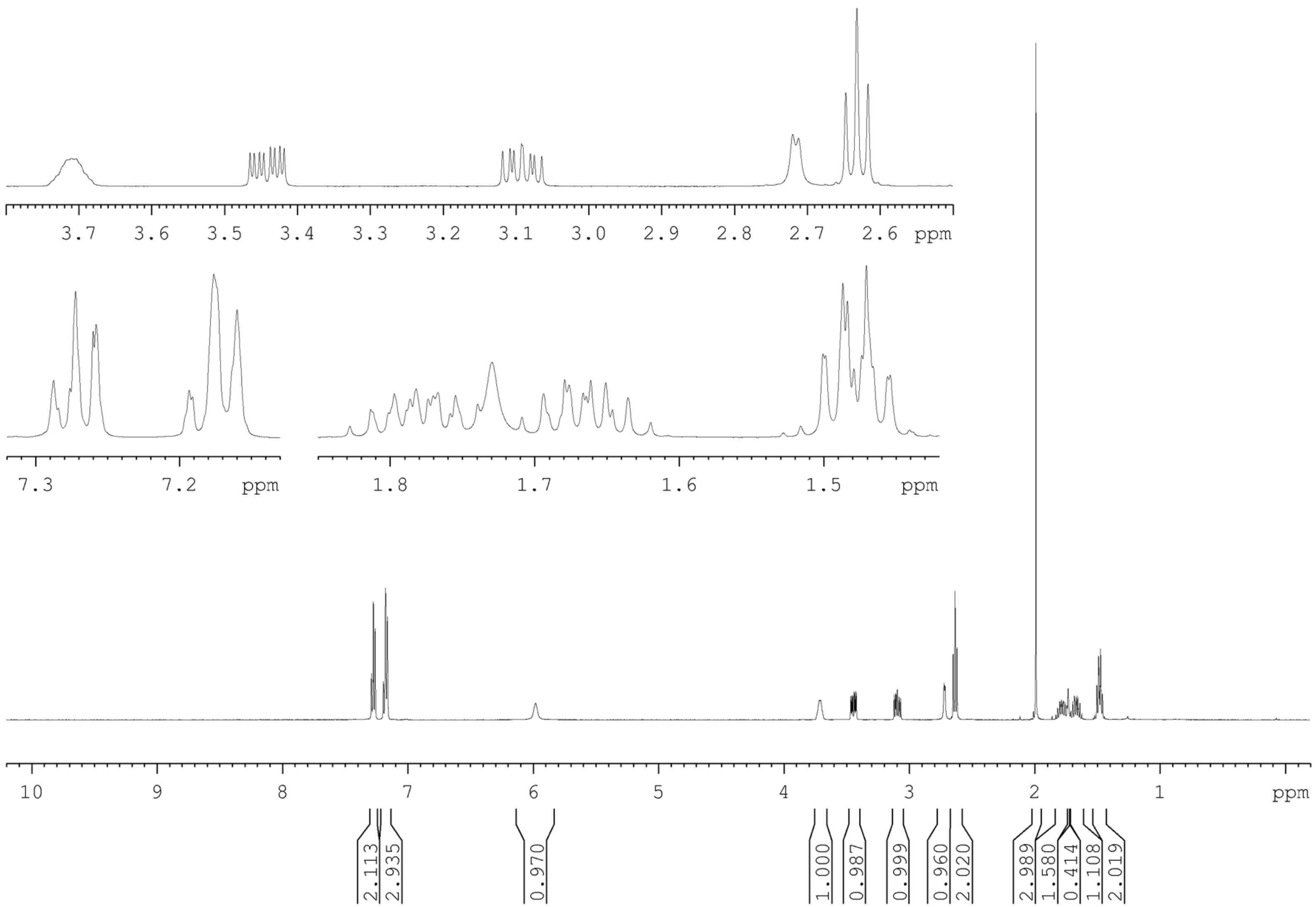

(*S*)-*N*-(2-hydroxy-5-phenylpentyl)acetamide (1i)

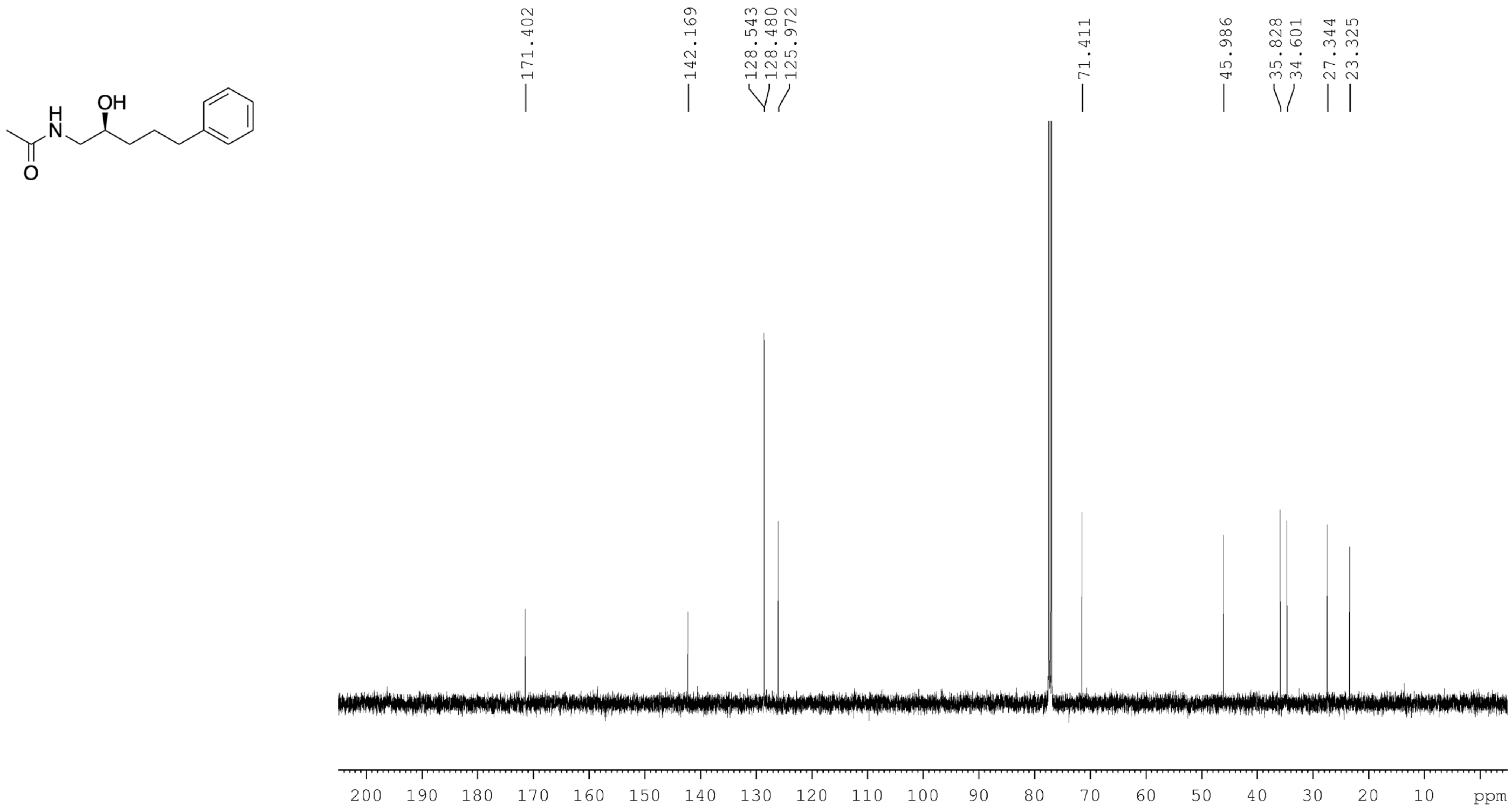

1-amino-6-phenylhexan-2-ol (1j-I_3)

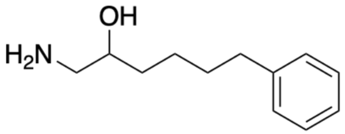

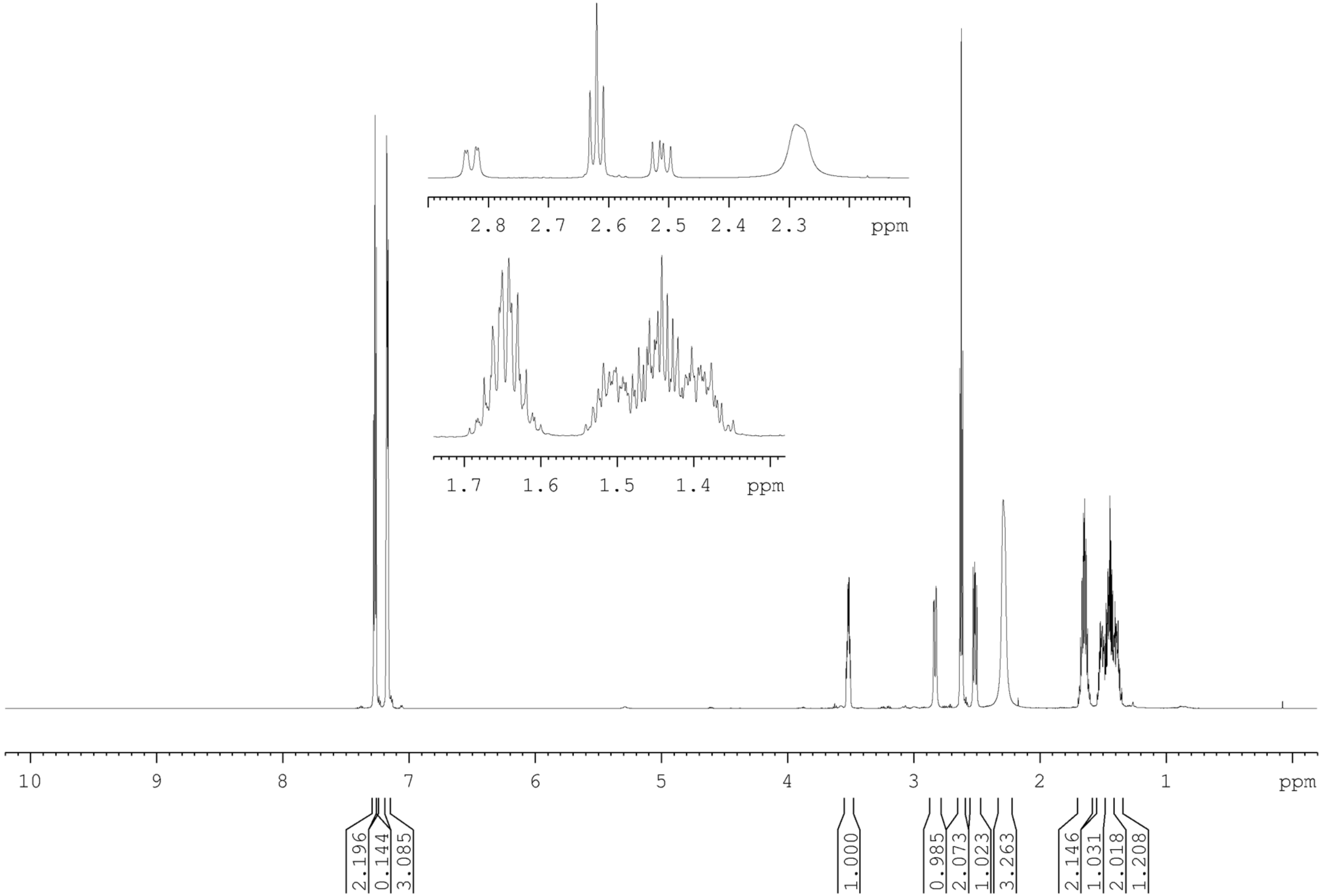

1-amino-6-phenylhexan-2-ol (1j-I_3)

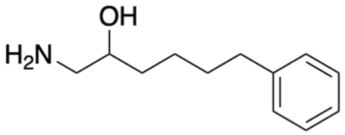

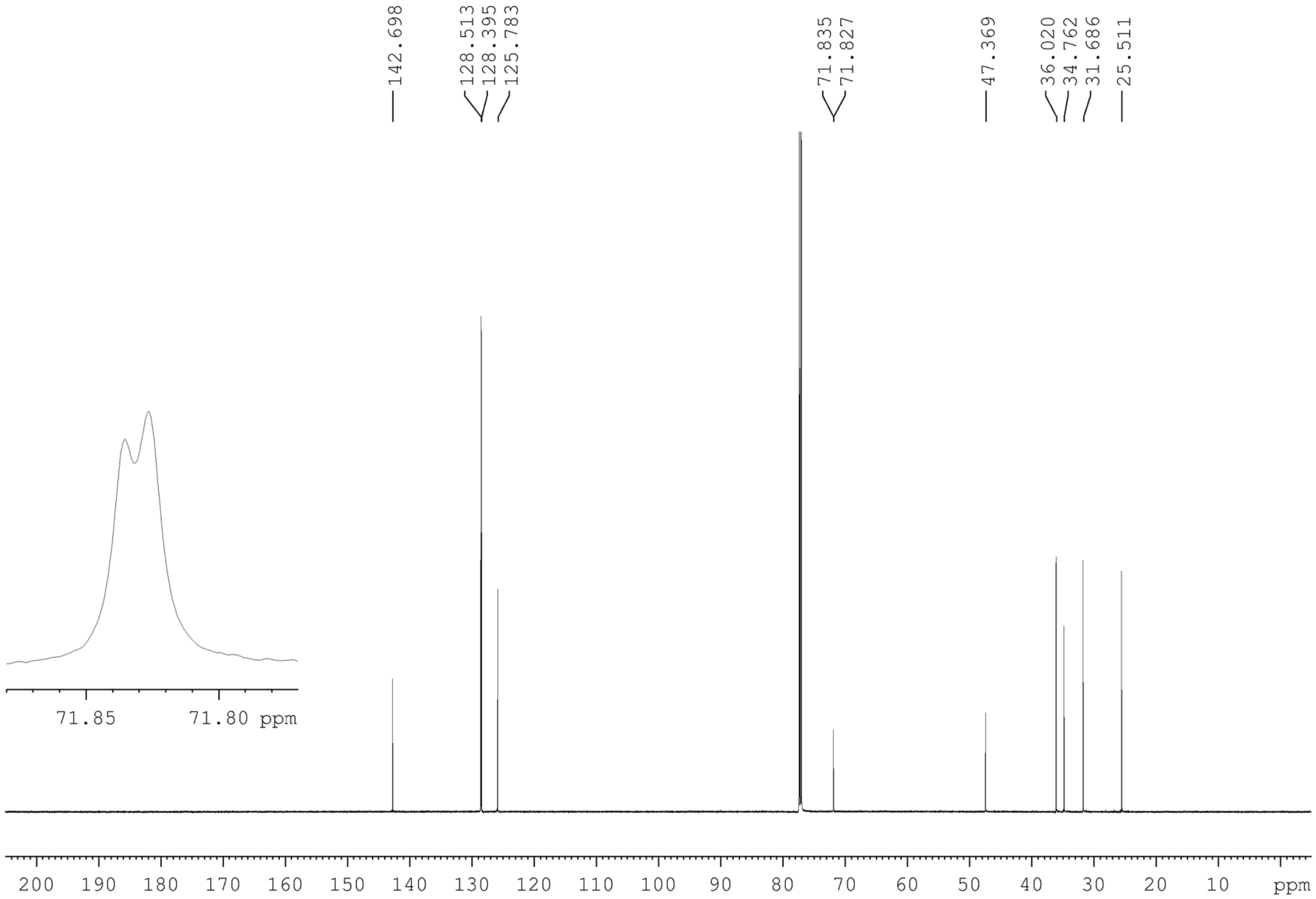

N-(2-hydroxy-6-phenylhexyl)acetamide (1j-rac)

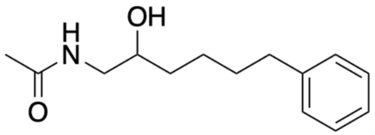

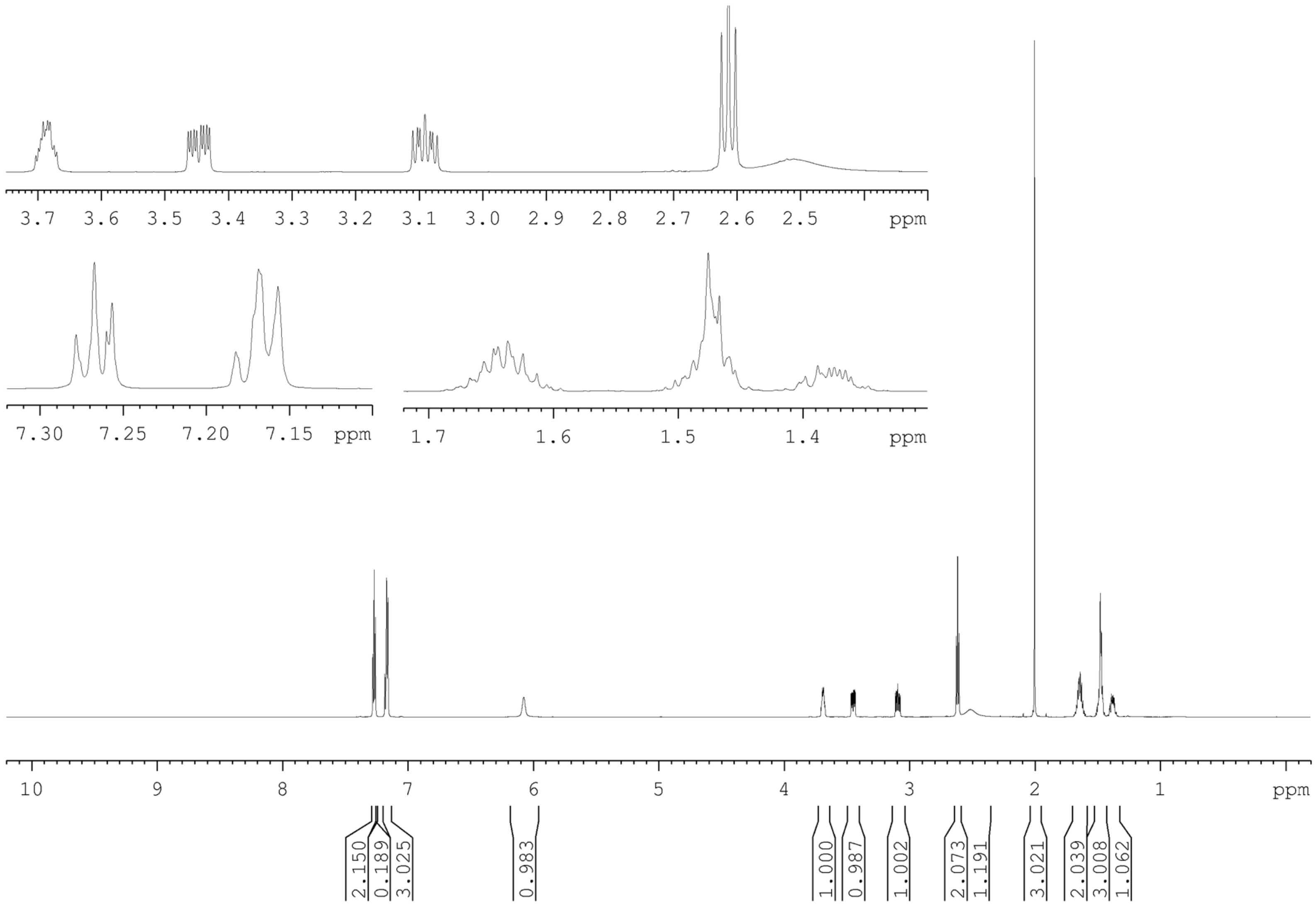

N-(2-hydroxy-6-phenylhexyl)acetamide (1j-rac)

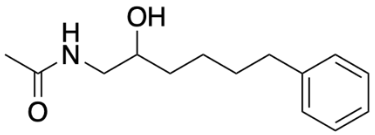

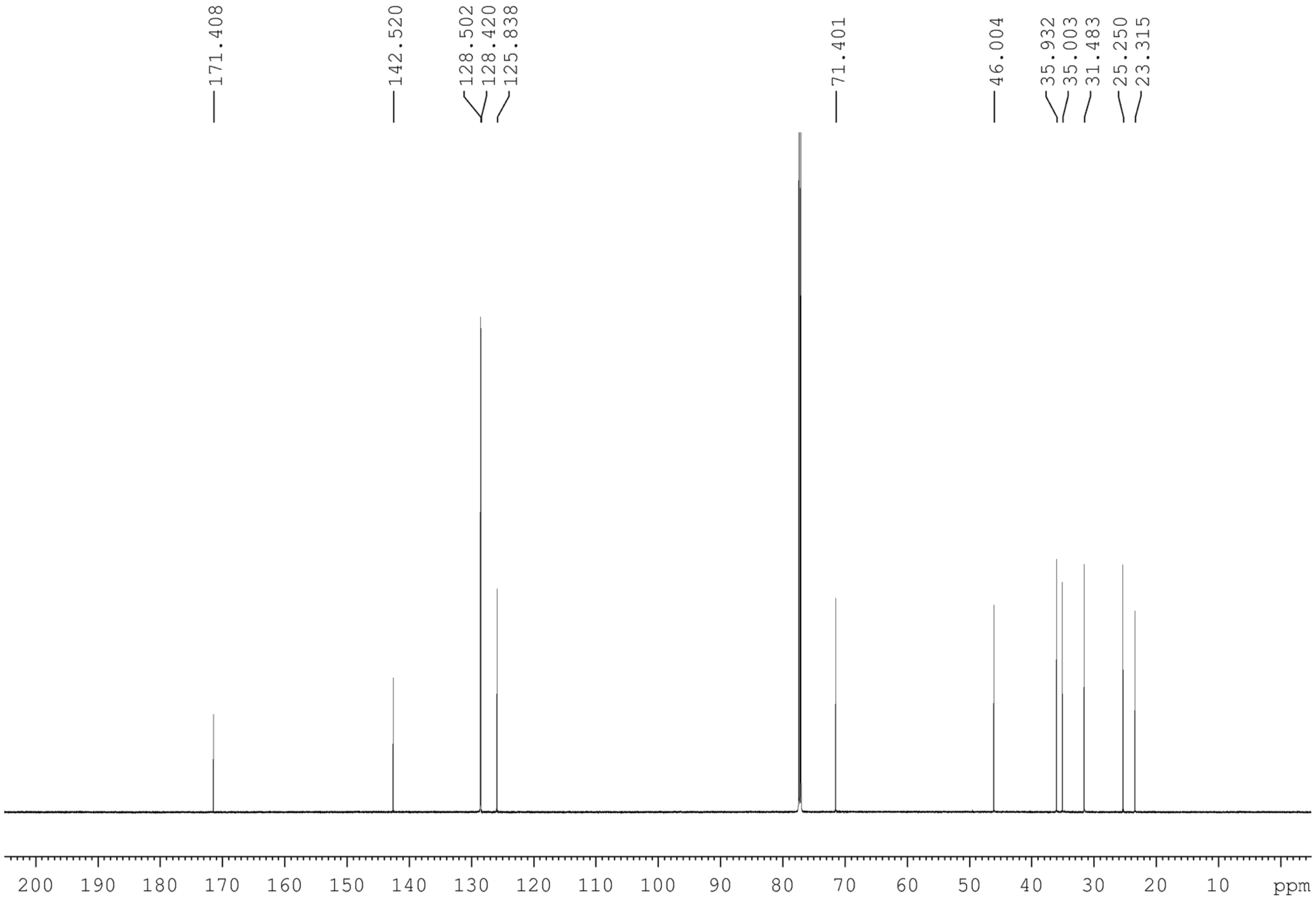

(*S*)-*N*-(2-hydroxy-6-phenylhexyl)acetamide (1j)

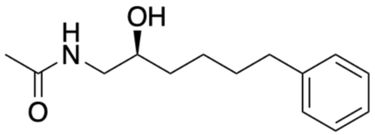

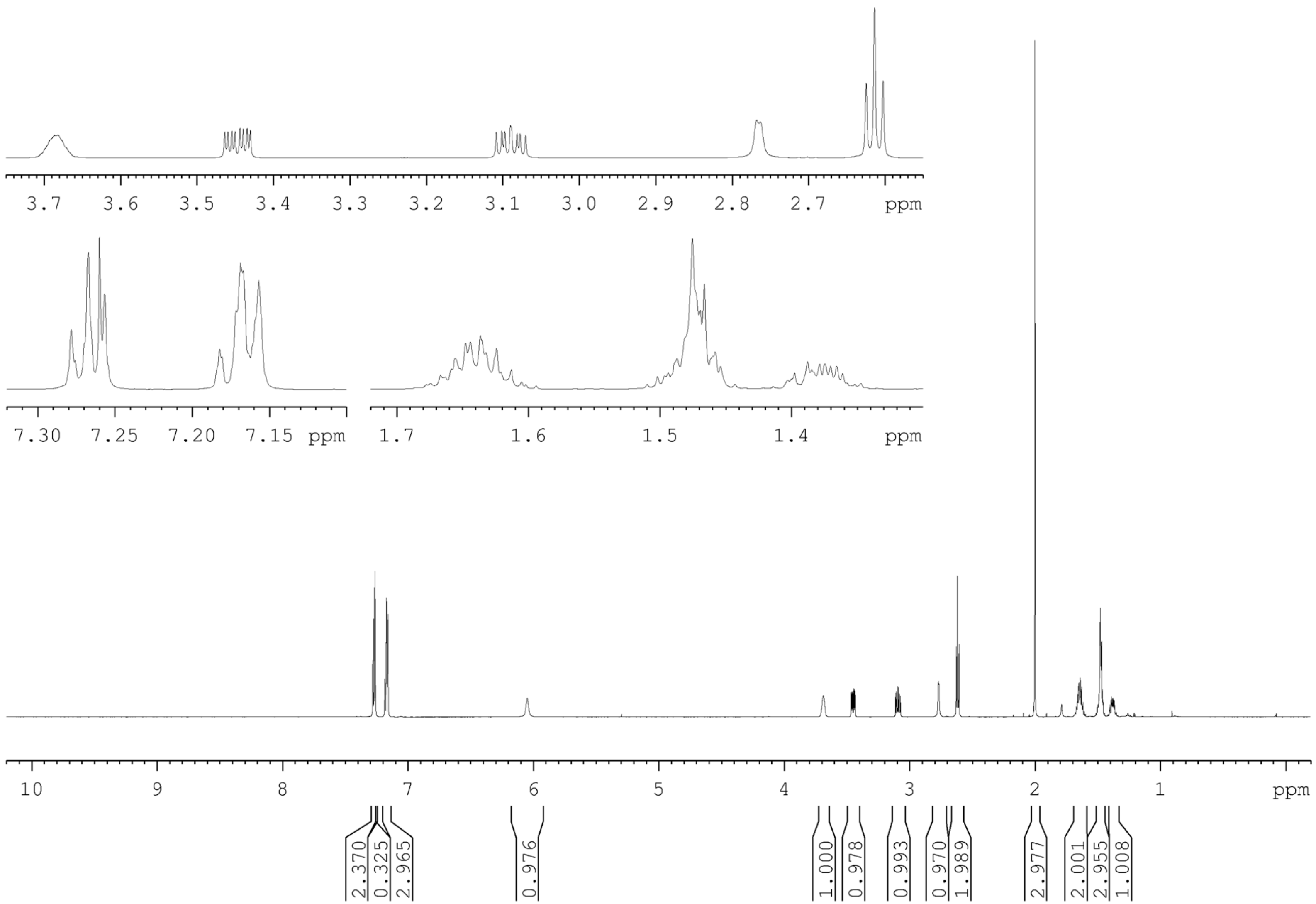

(*S*)-*N*-(2-hydroxy-6-phenylhexyl)acetamide (1j)

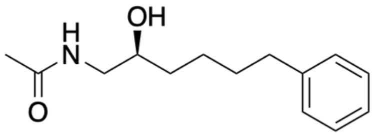

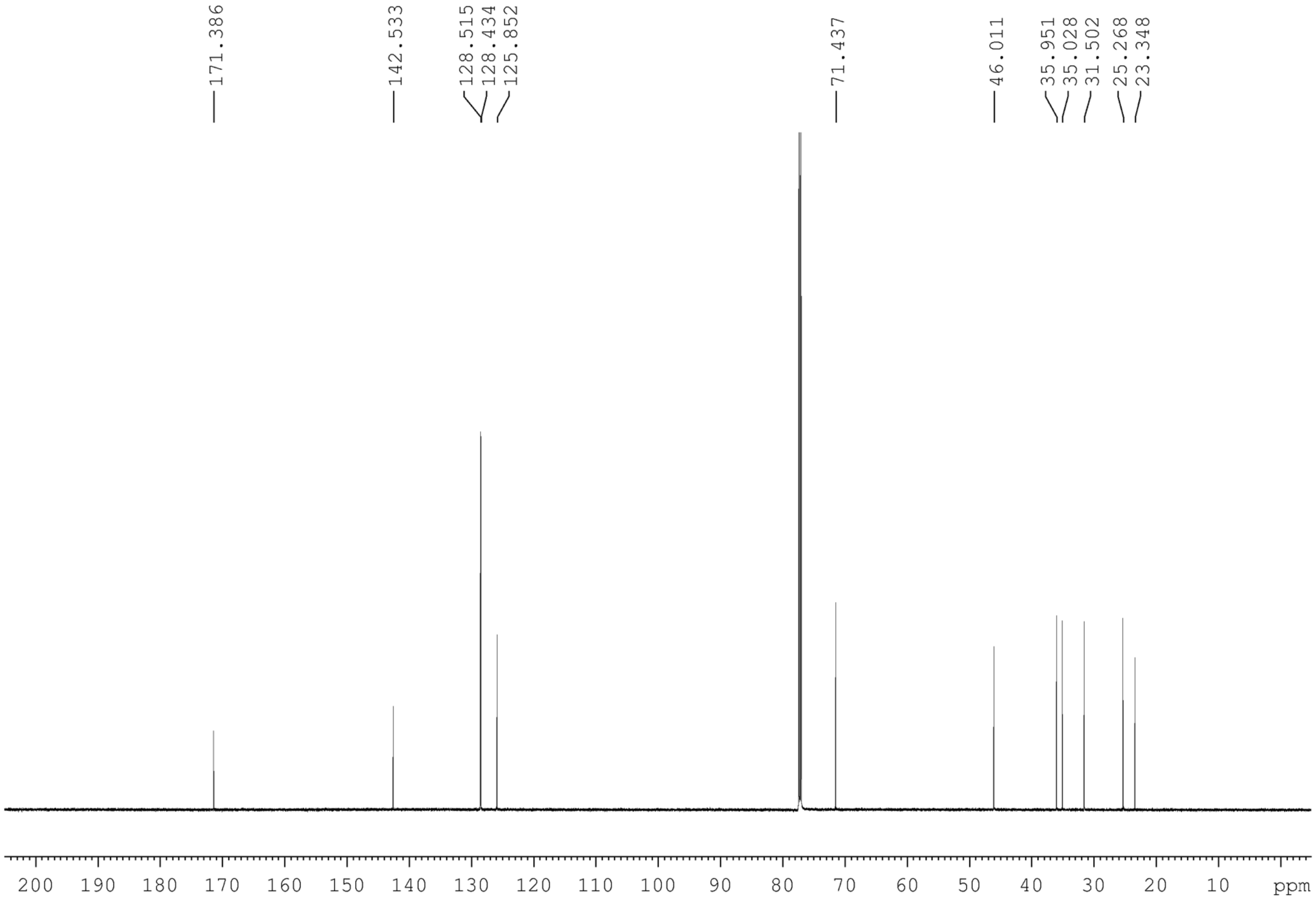

hept-6-en-1-ylbenzene (1k-I$_1$)

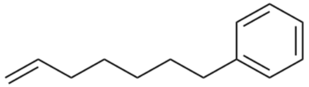

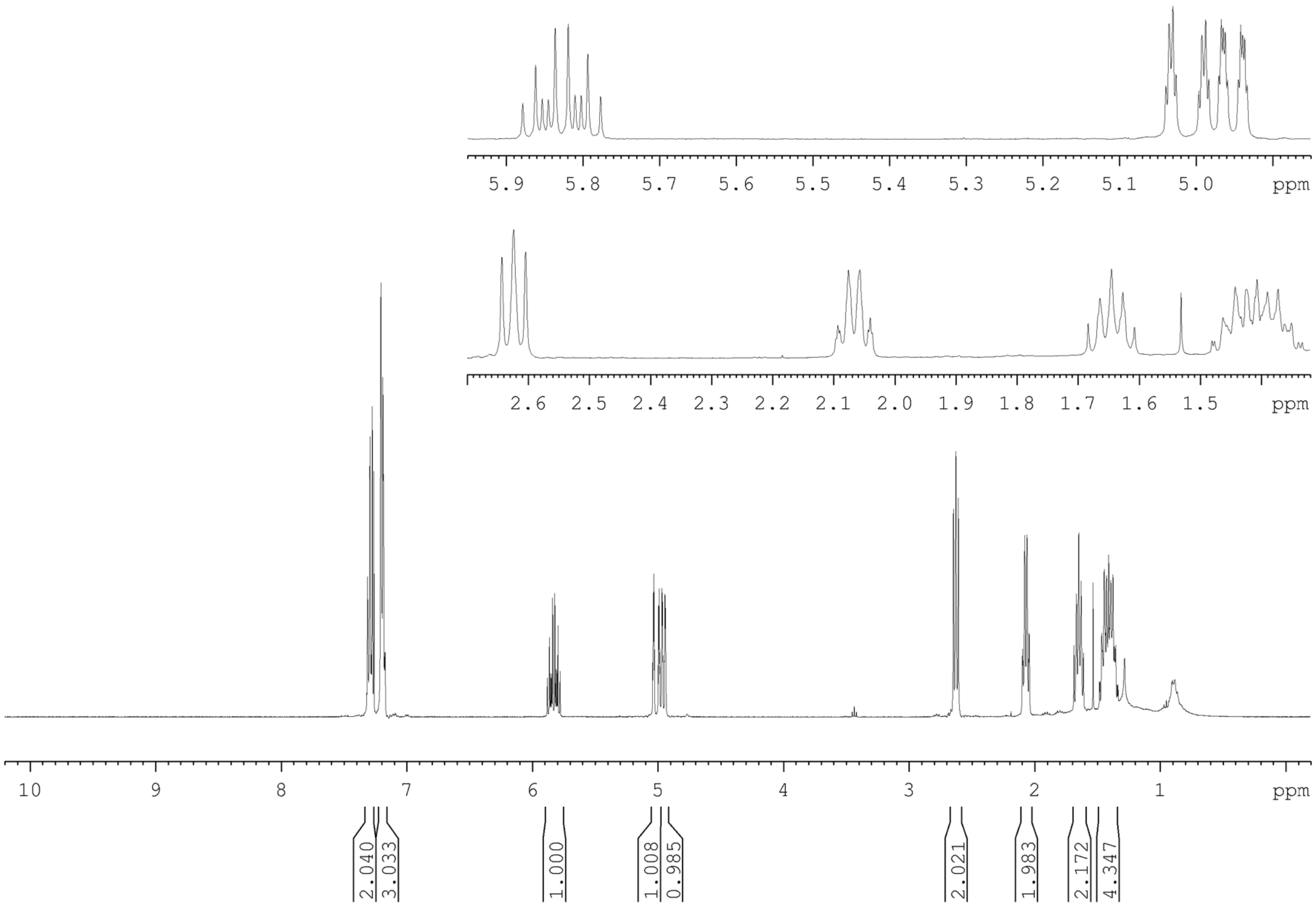

hept-6-en-1-ylbenzene (1k-I_1)

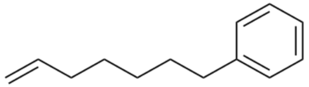

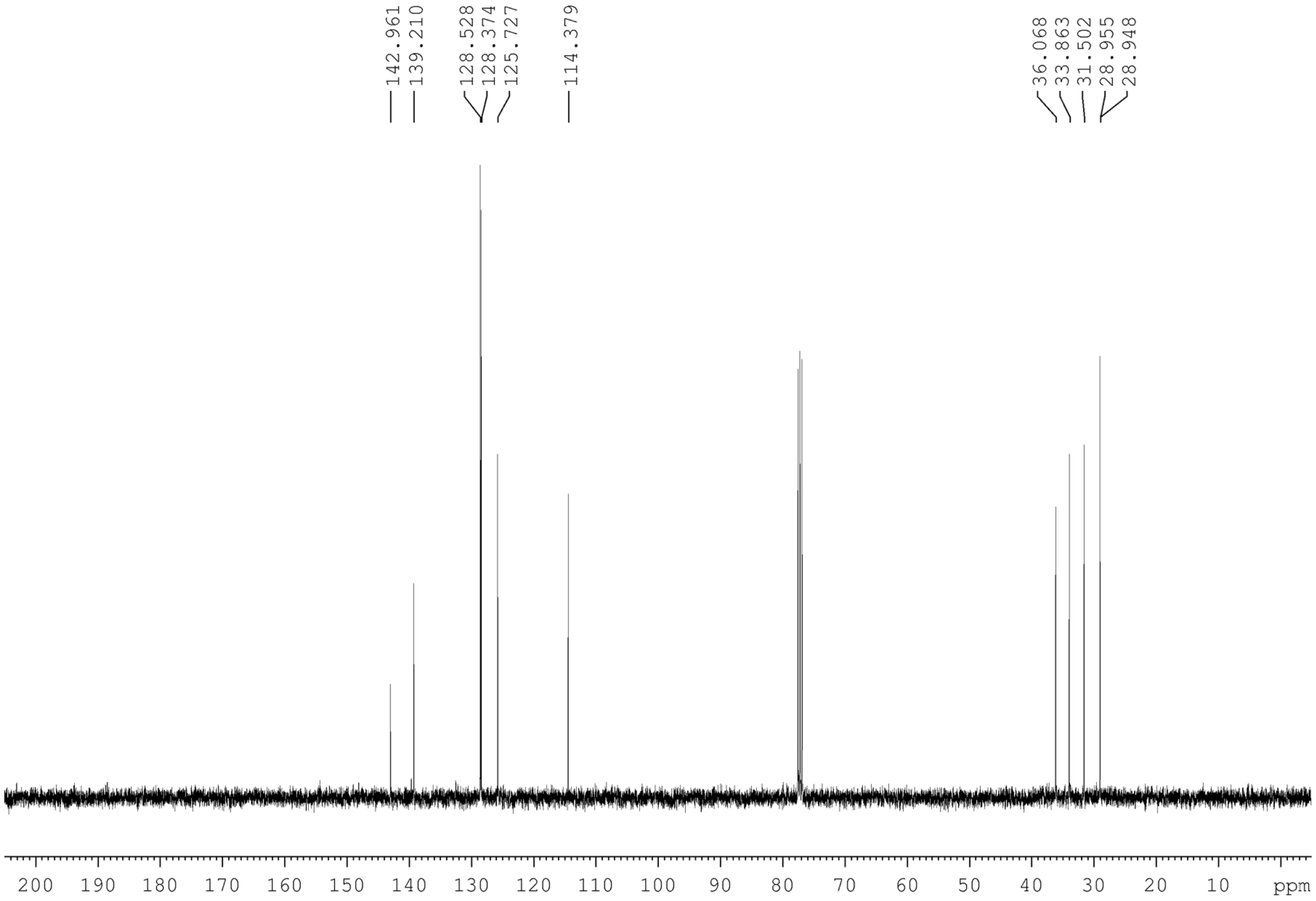

2-(5-phenylpentyl)oxirane (1k-I_2)

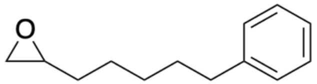

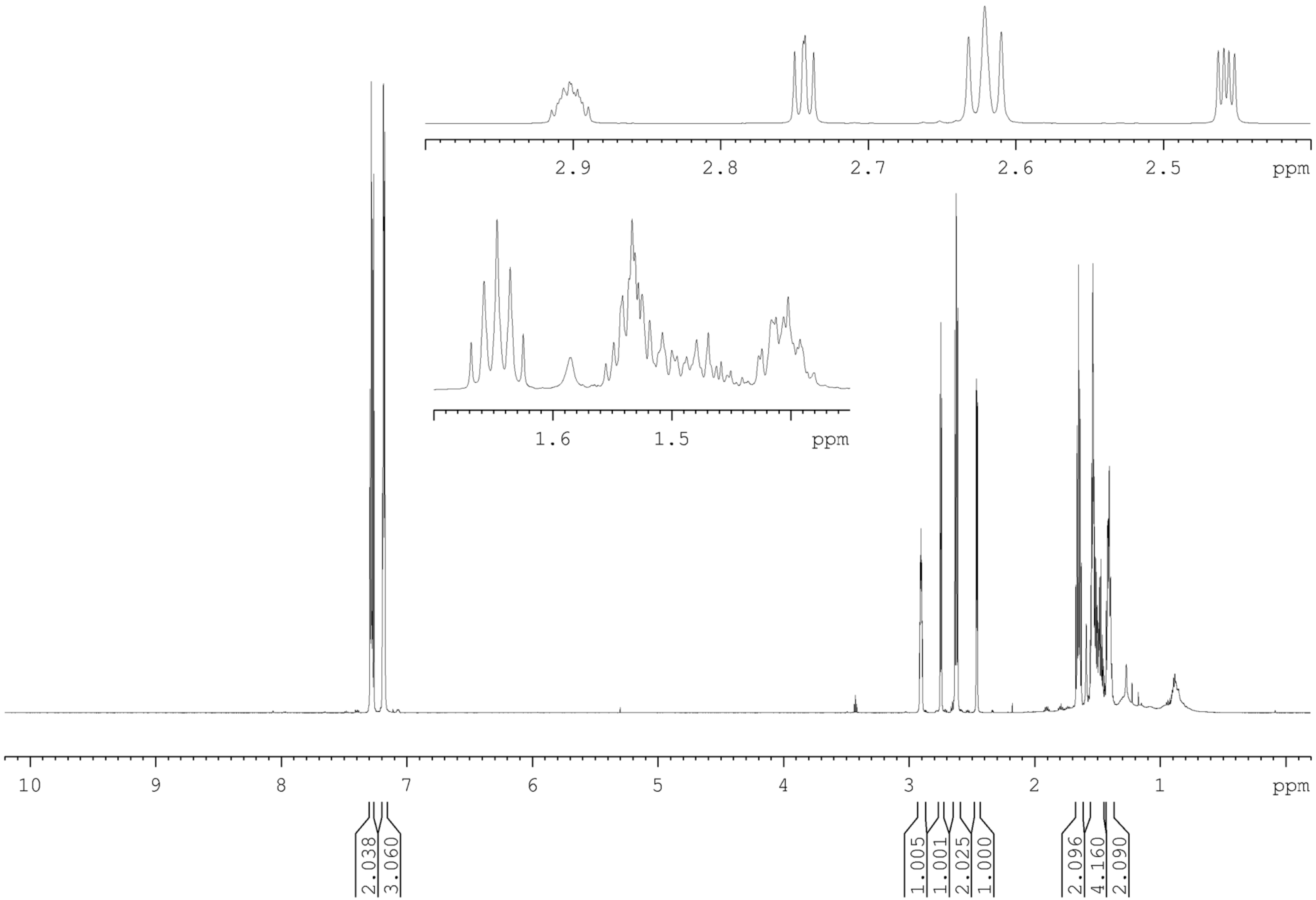

2-(5-phenylpentyl)oxirane (1k-I_2)

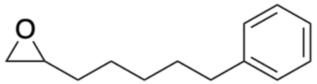

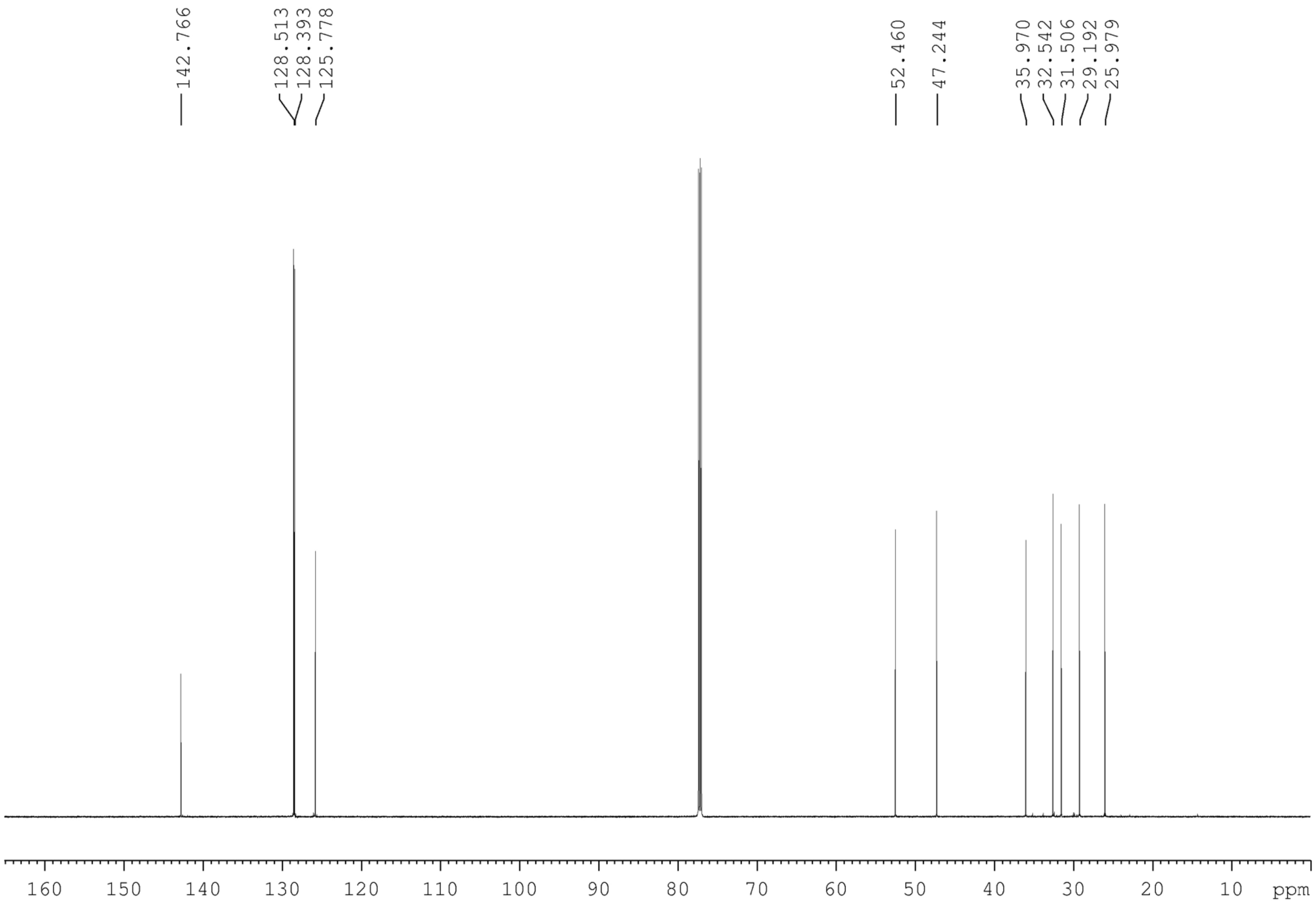

1-amino-7-phenylheptan-2-ol (1k-I_3)

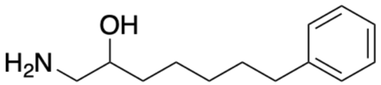

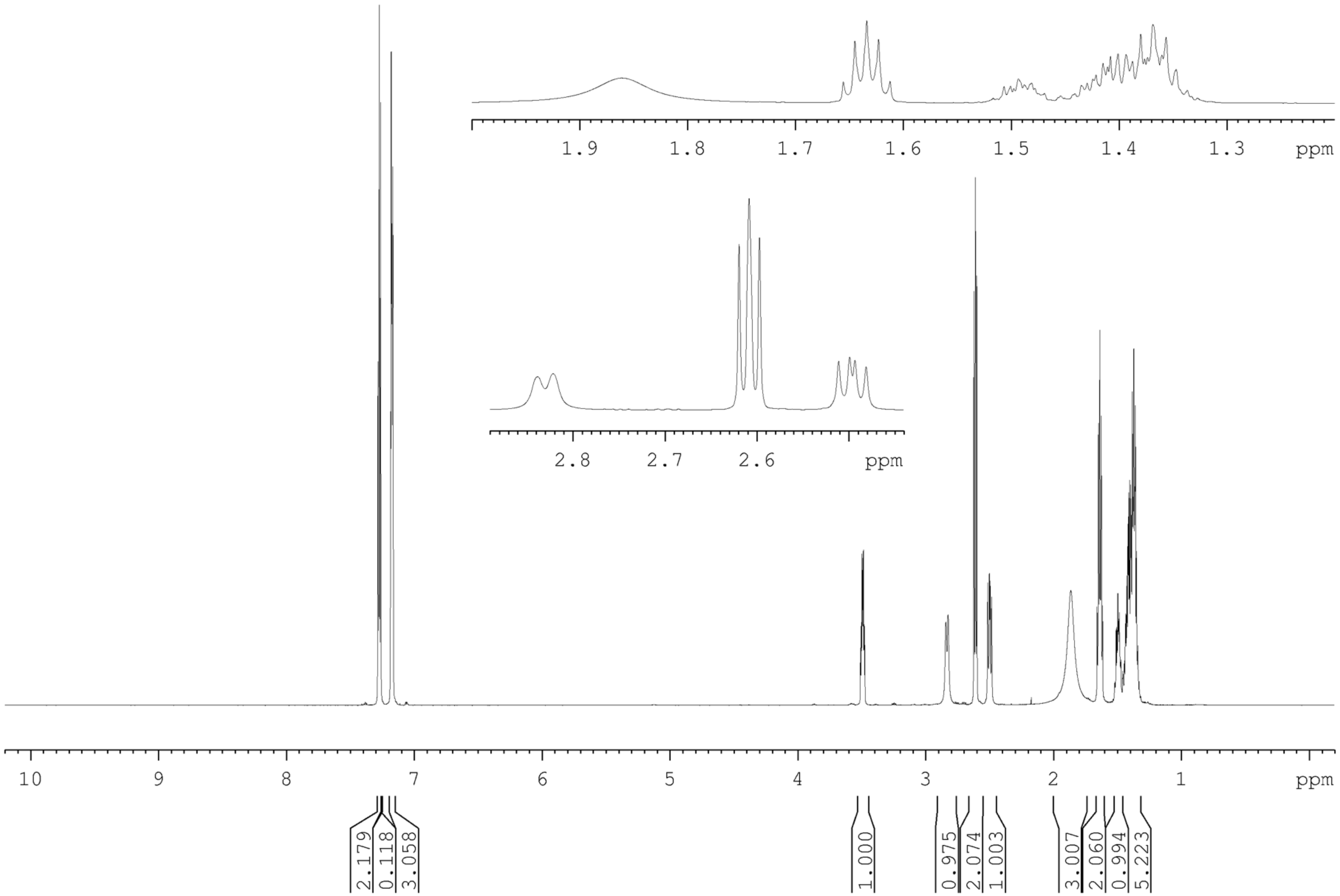

1-amino-7-phenylheptan-2-ol (1k-I_3)

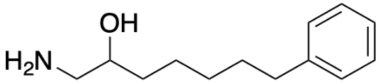

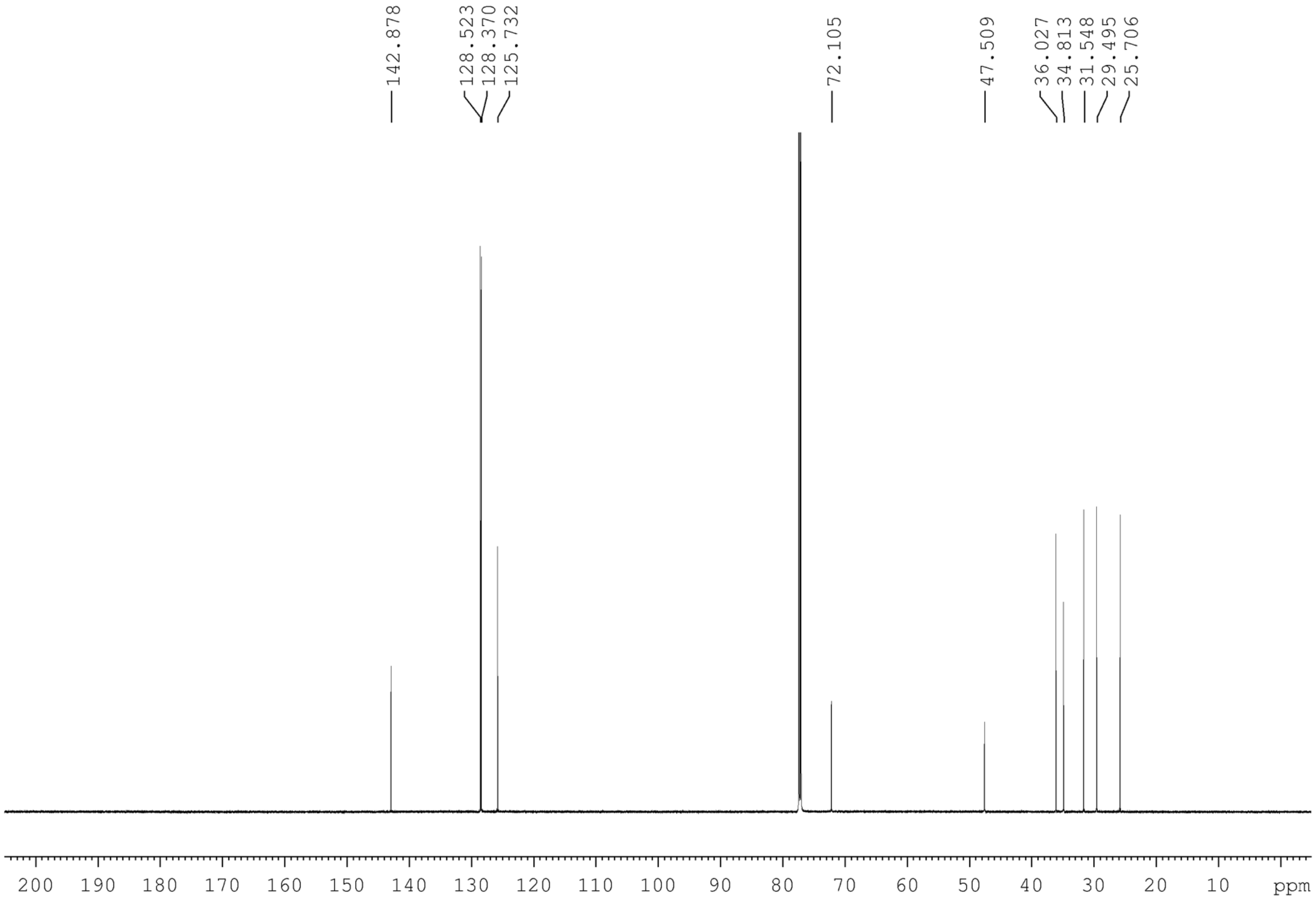

N-(2-hydroxy-7-phenylheptyl)acetamide (1k-rac)

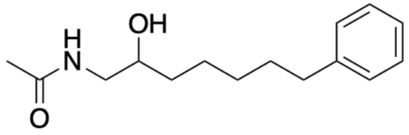

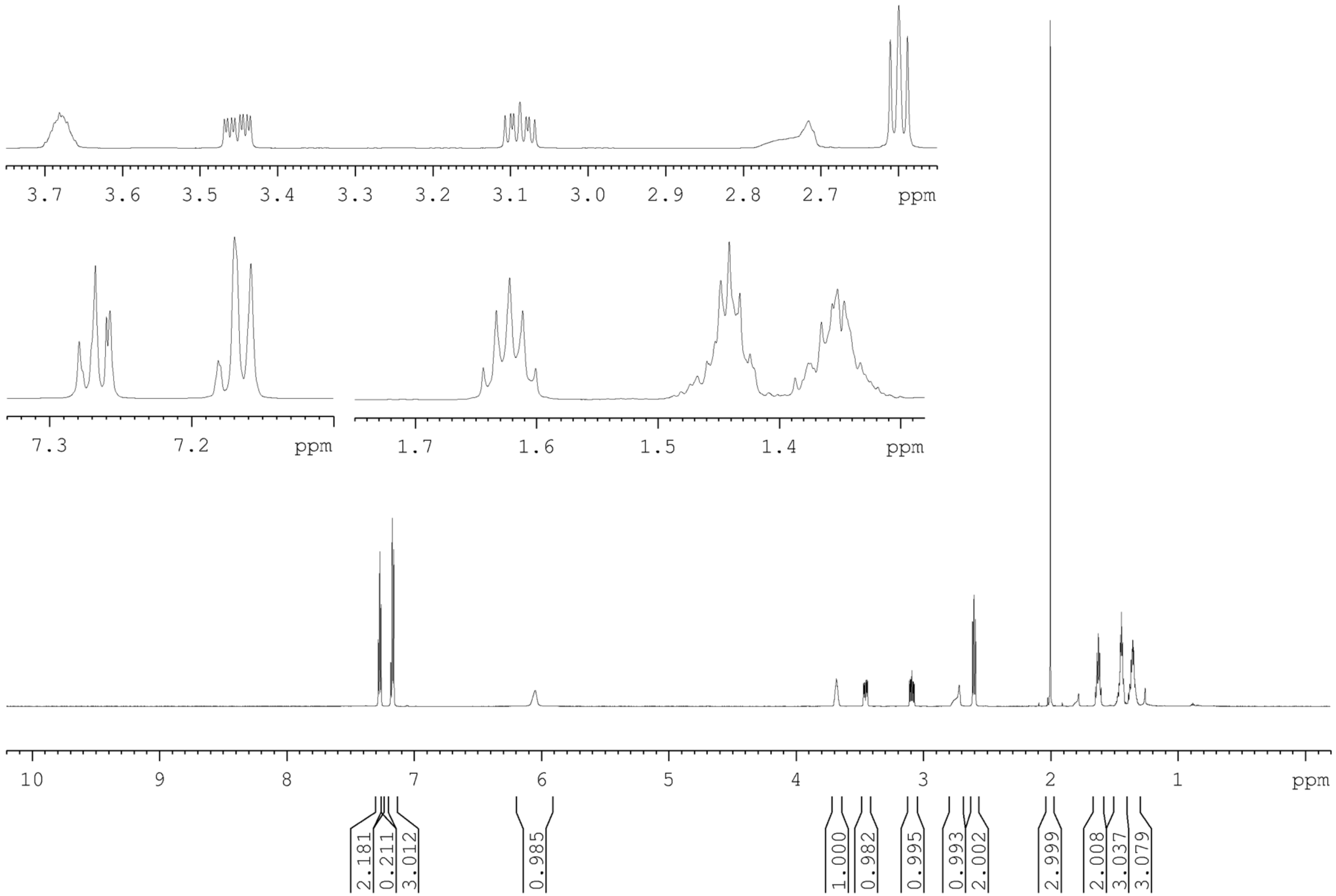

N-(2-hydroxy-7-phenylheptyl)acetamide (1k-rac)

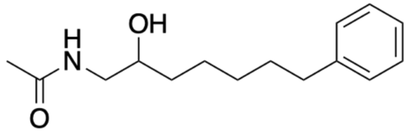

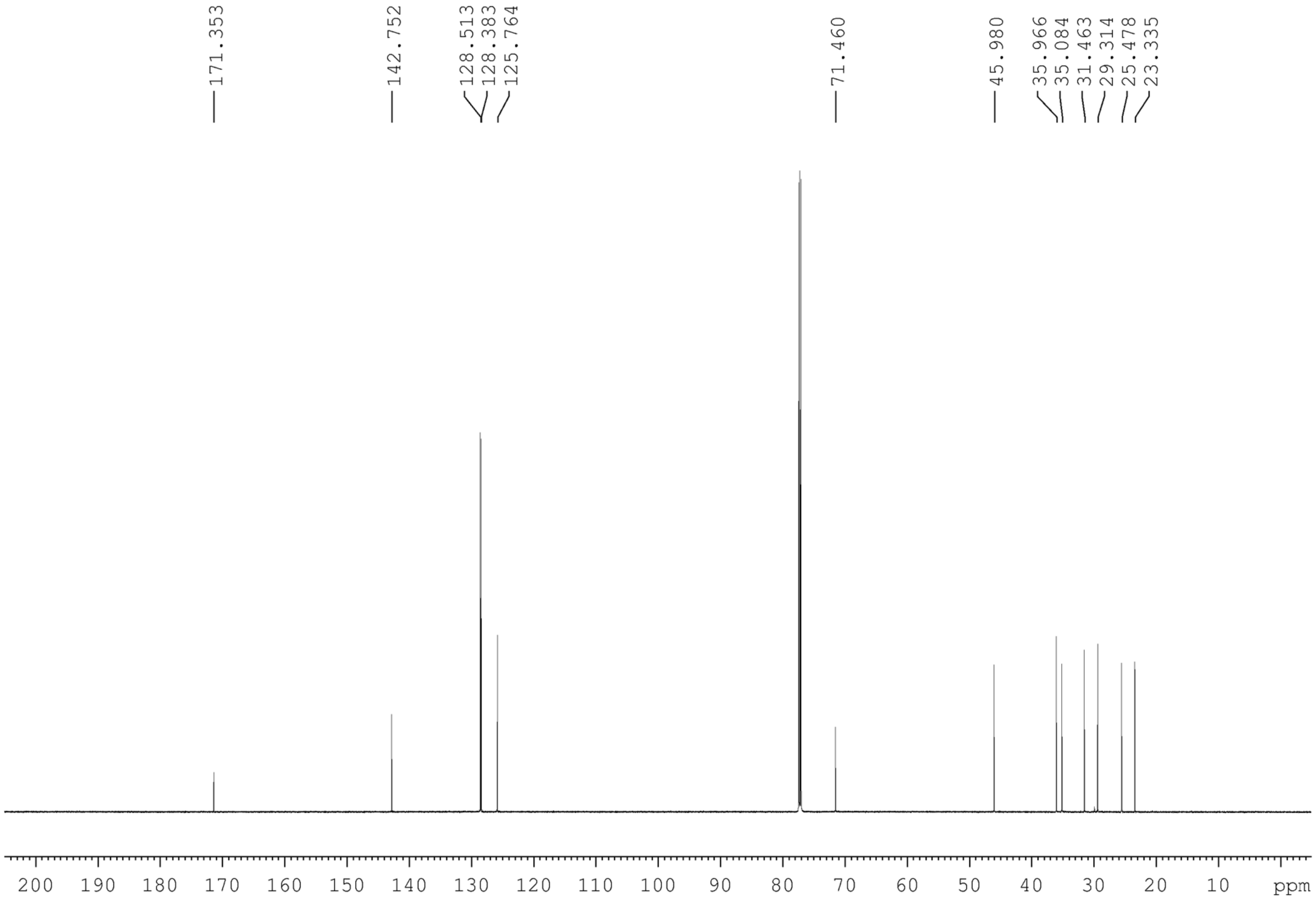

(*S*)-*N*-(2-hydroxy-7-phenylheptyl)acetamide (1k)

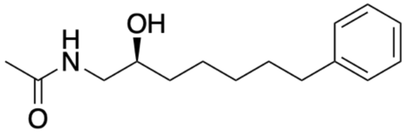

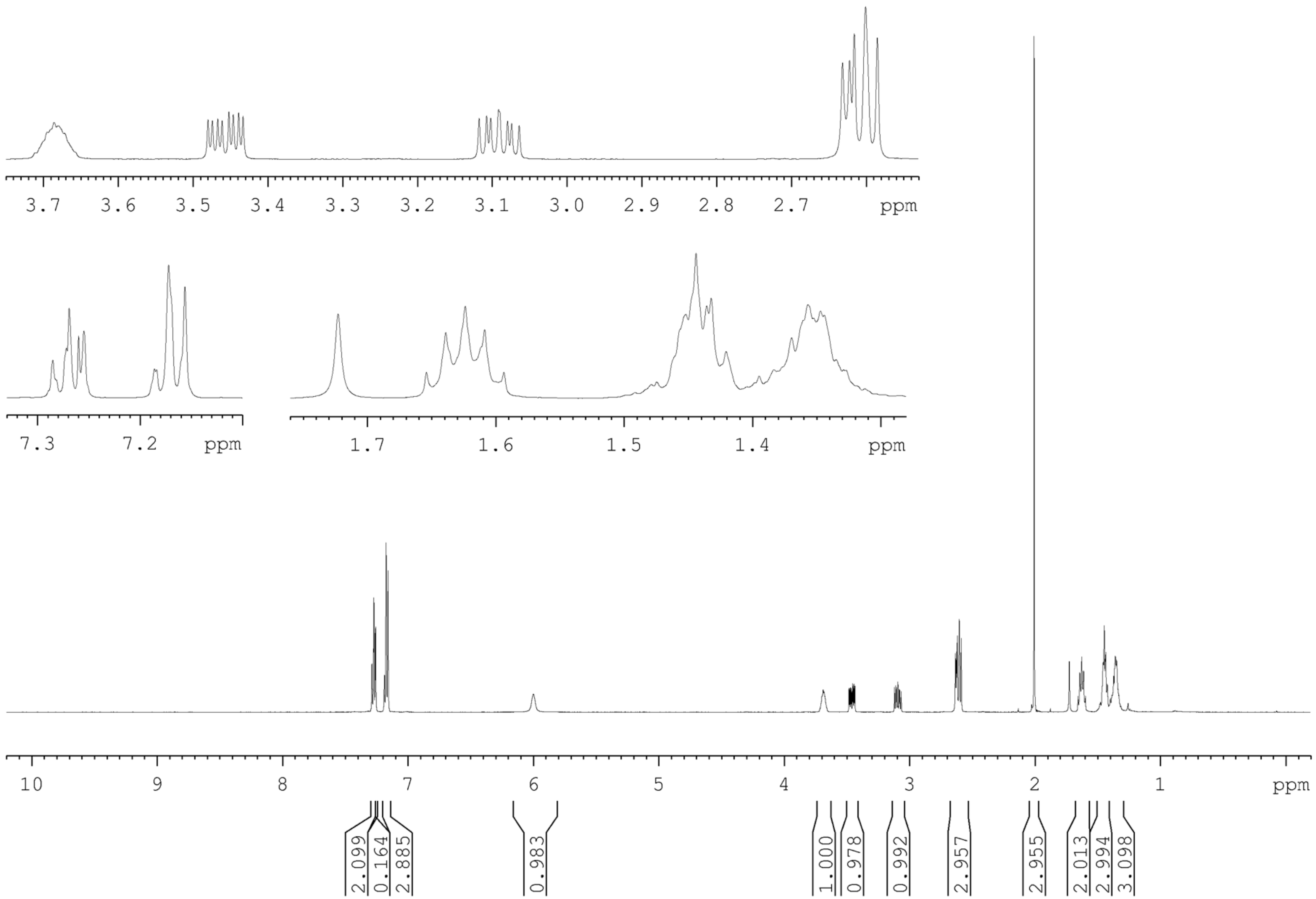

(*S*)-*N*-(2-hydroxy-7-phenylheptyl)acetamide (1k)

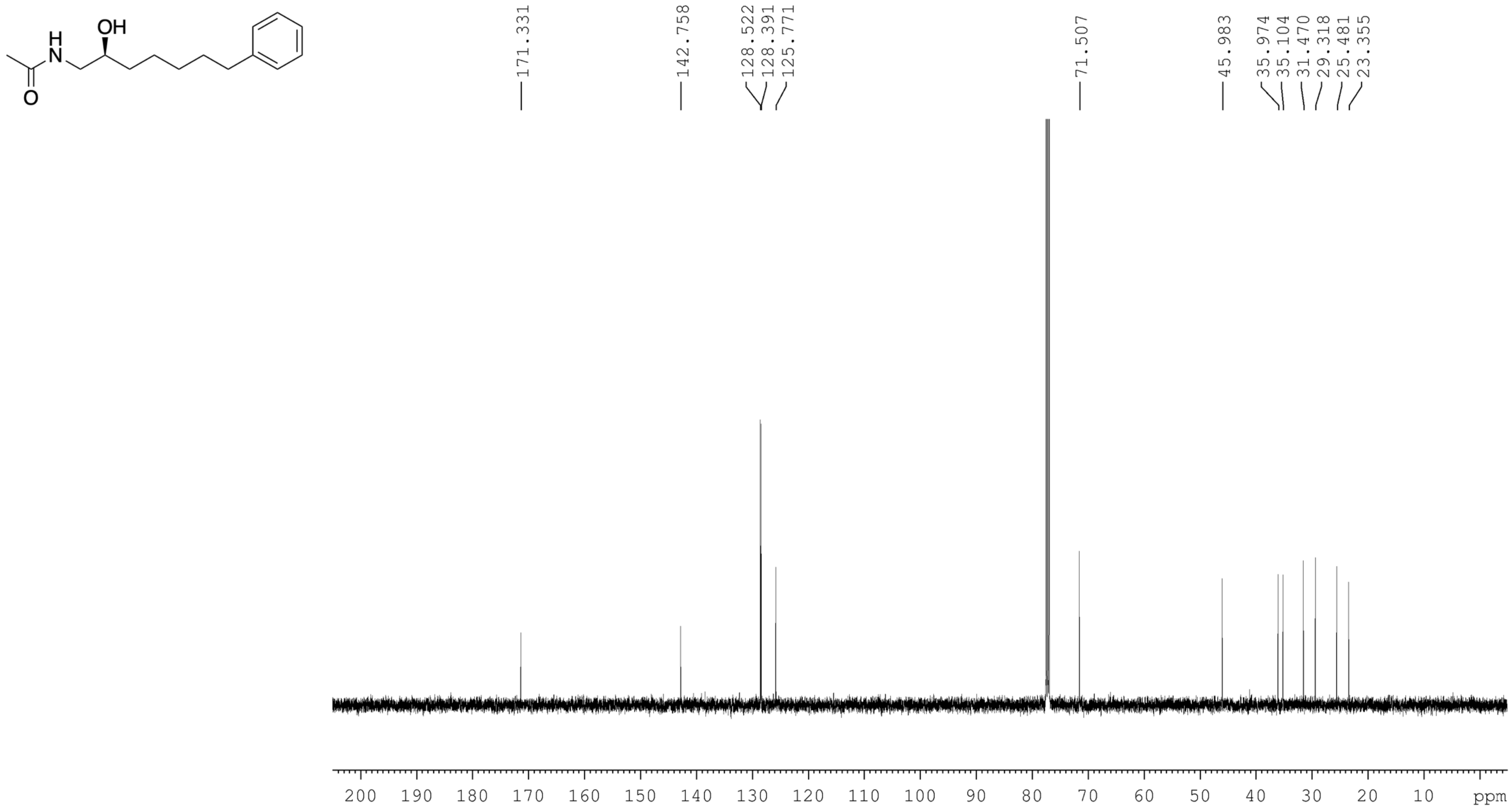

tert-butyl (5-acetamido-4-hydroxypentyl)carbamate (1l-rac)

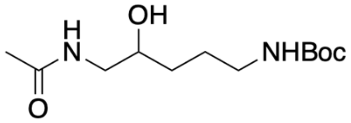

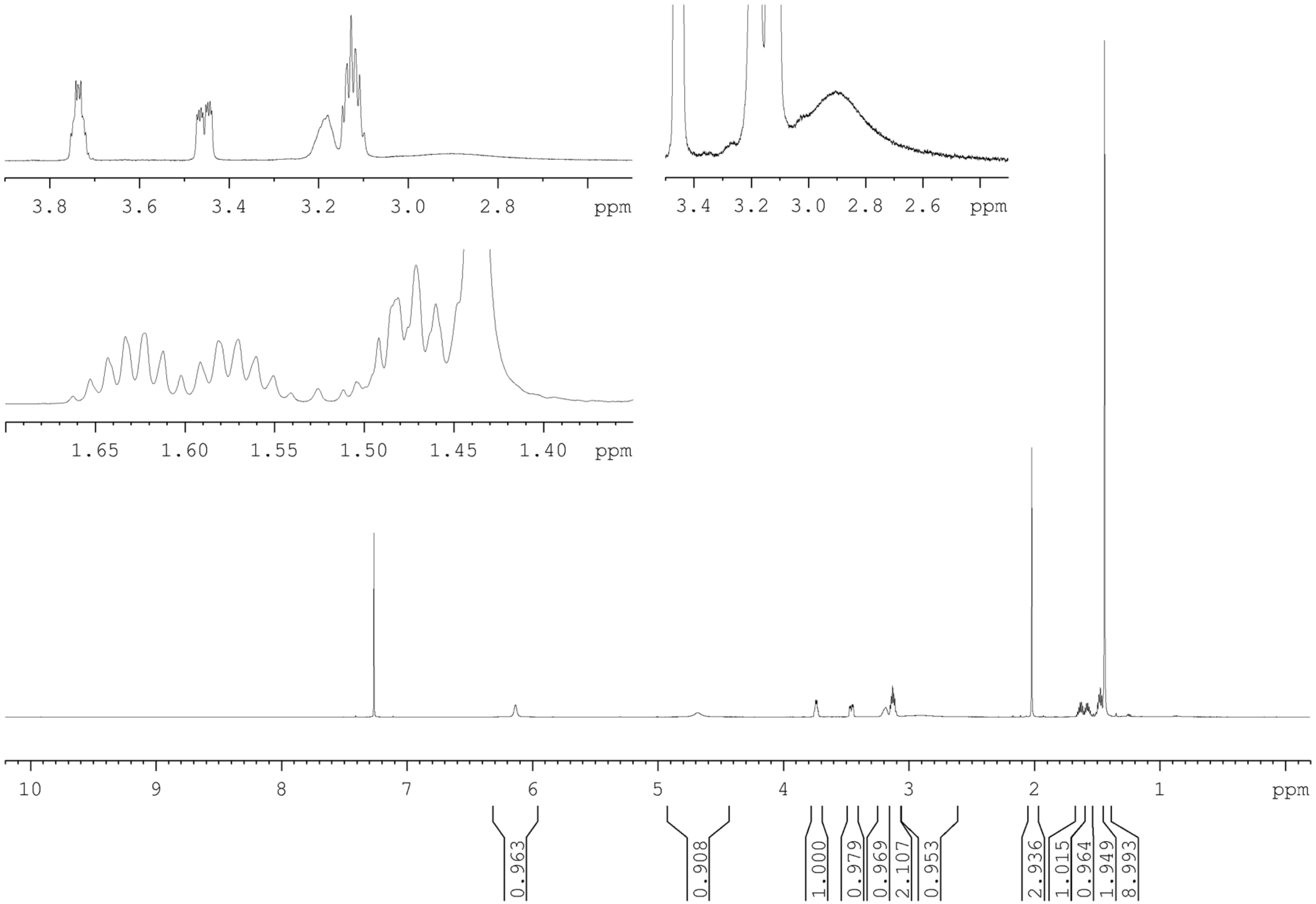

tert-butyl (5-acetamido-4-hydroxypentyl)carbamate (1l-rac)

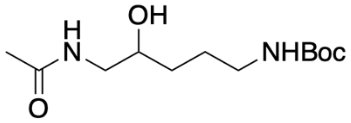

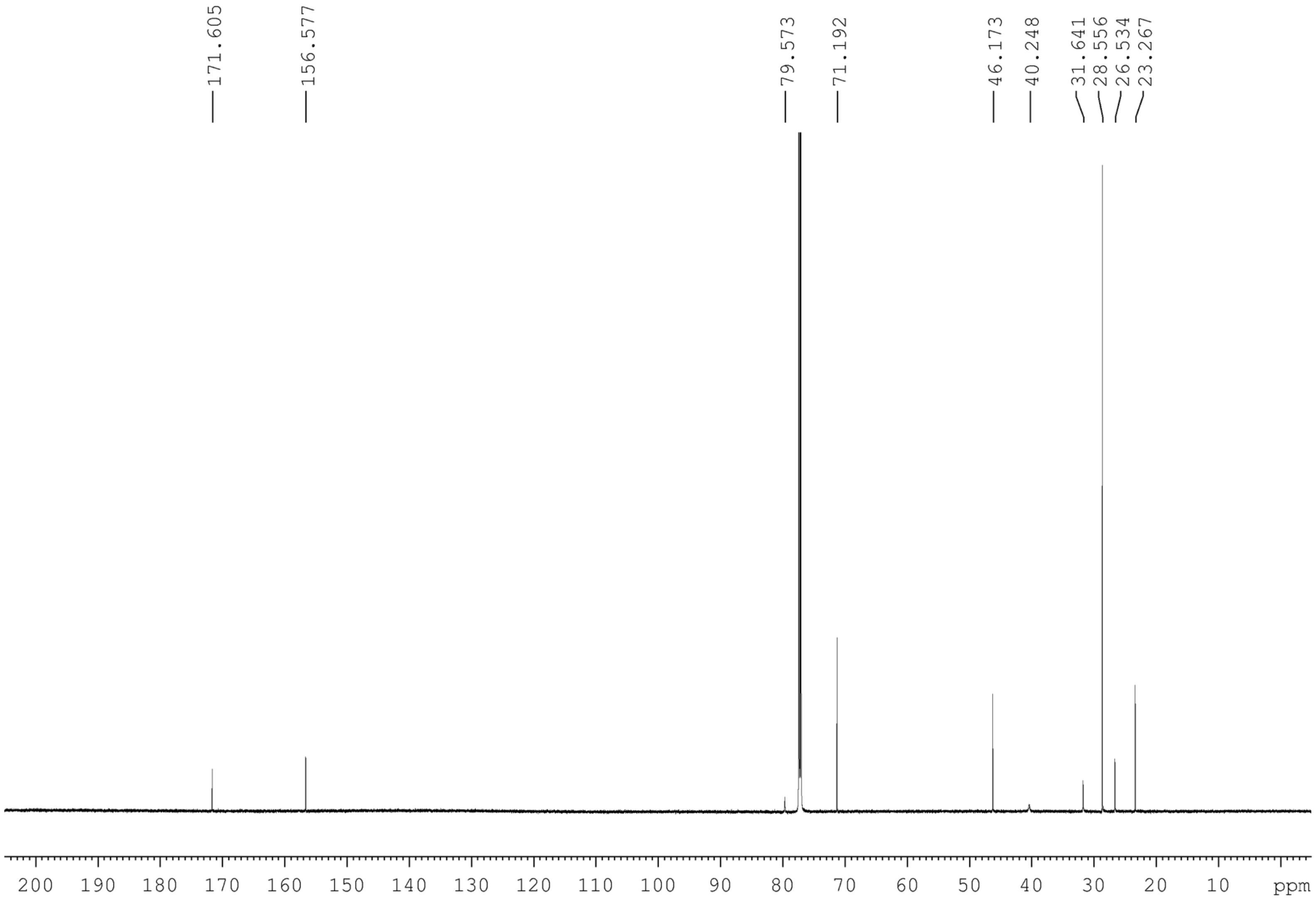

***tert*-butyl (*S*)-(5-acetamido-4-hydroxypentyl)carbamate (1l)**

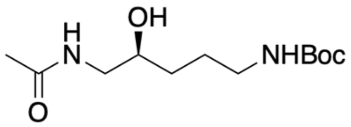

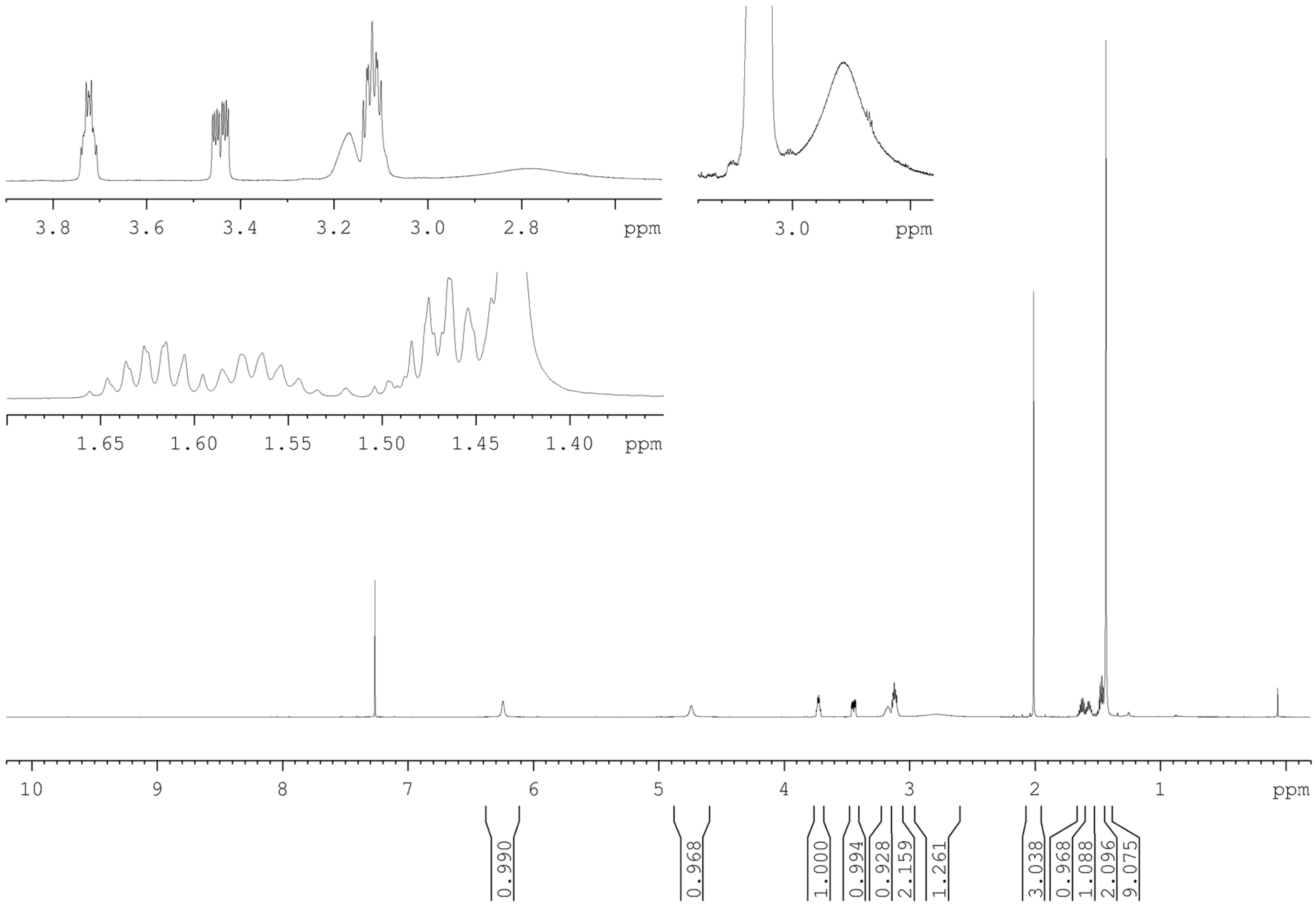

tert-butyl (_S_)-(5-acetamido-4-hydroxypentyl)carbamate (1l)

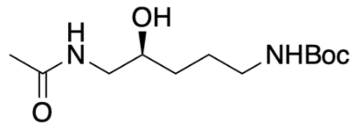

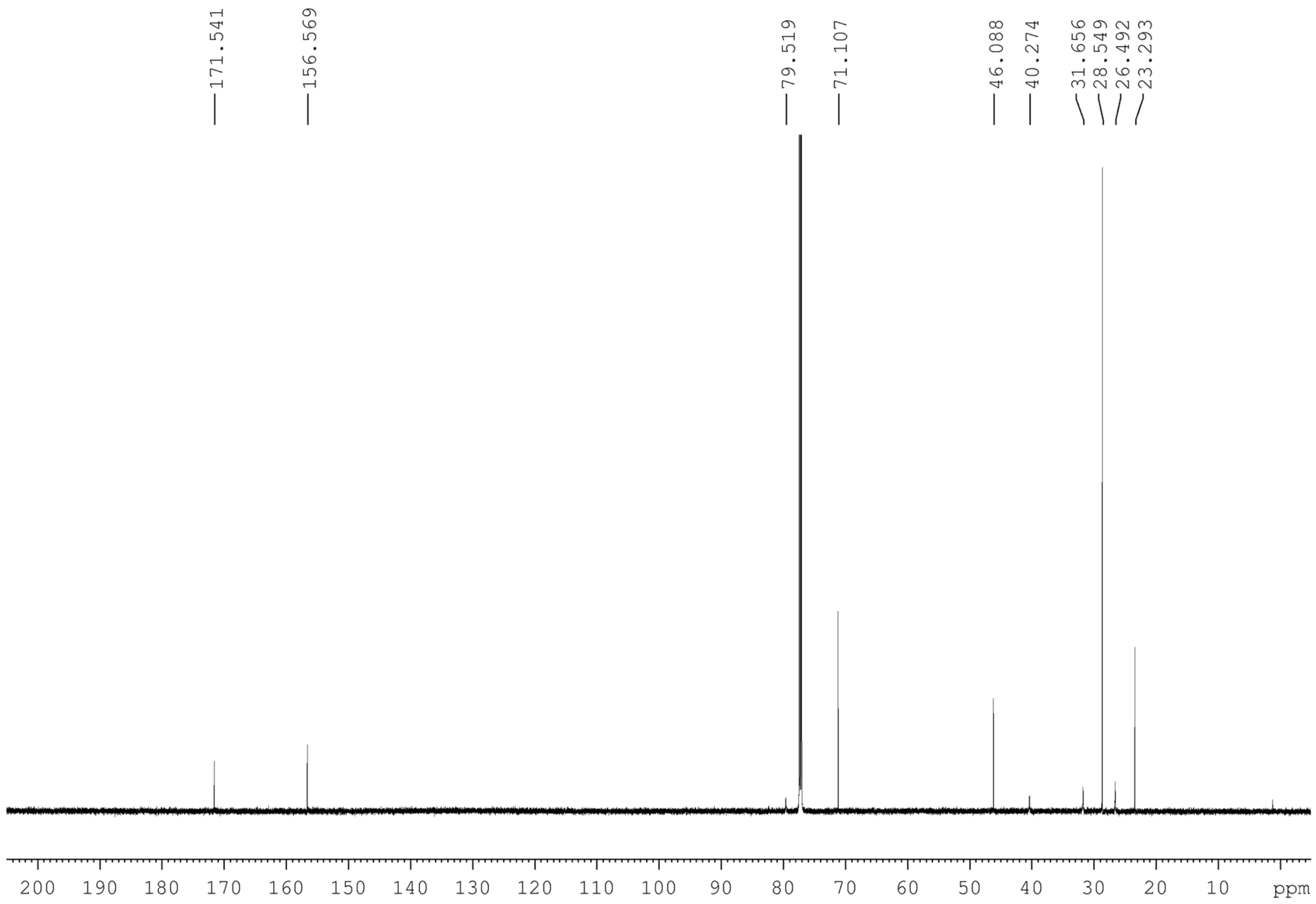

(*S*)-1-(*N*-acetylbenzamido)-5-((*tert*-butoxycarbonyl)amino)pentan-2-yl benzoate (1l-Bz)

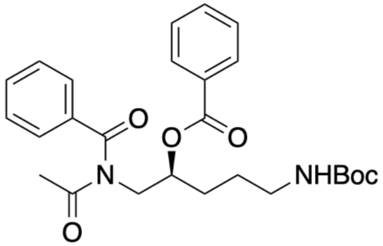

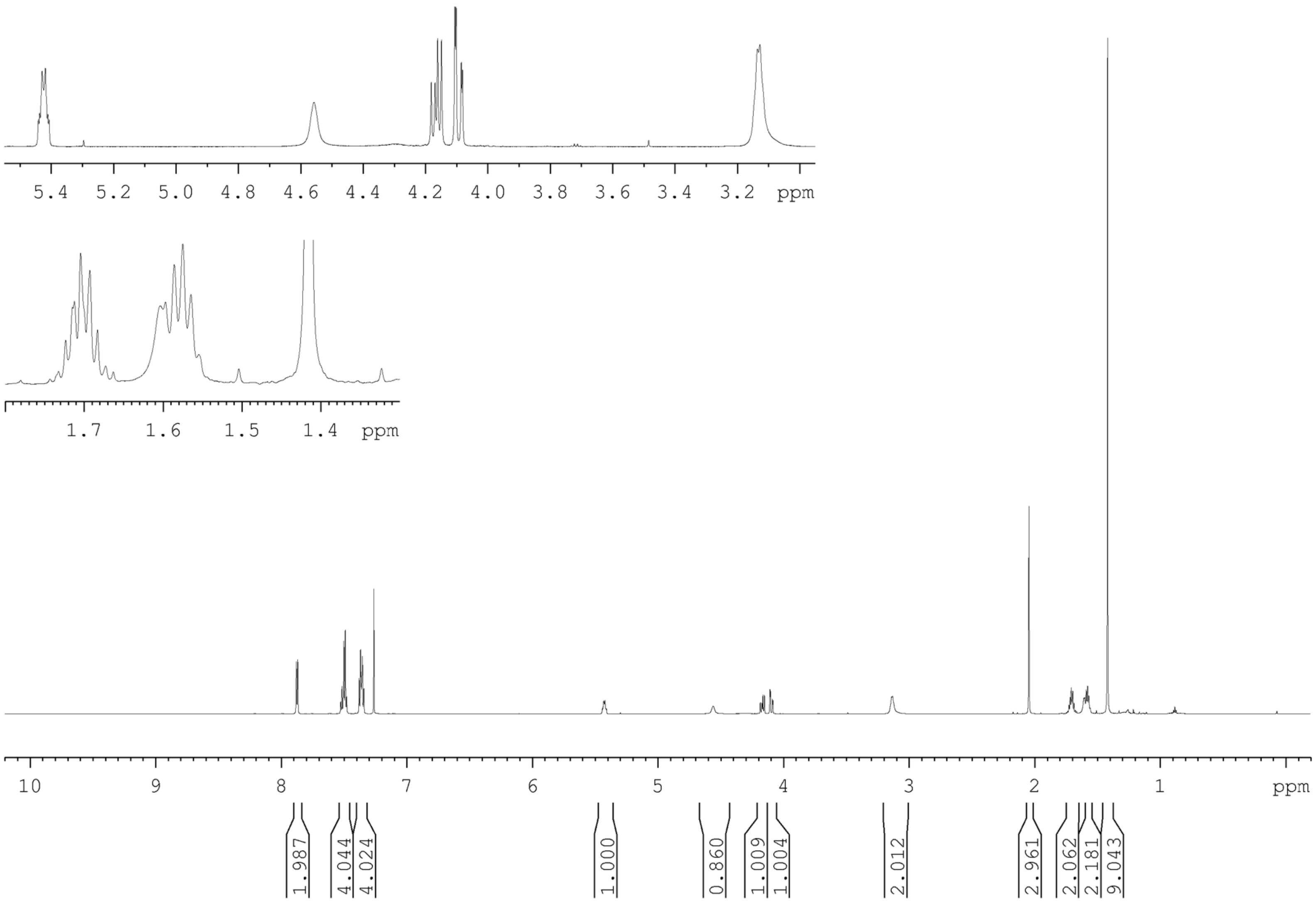

(*S*)-1-(*N*-acetylbenzamido)-5-((*tert*-butoxycarbonyl)amino)pentan-2-yl benzoate (1l-Bz)

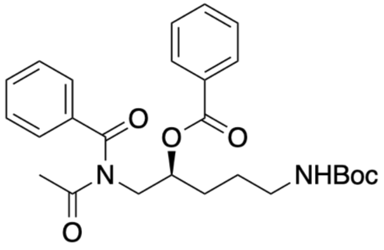

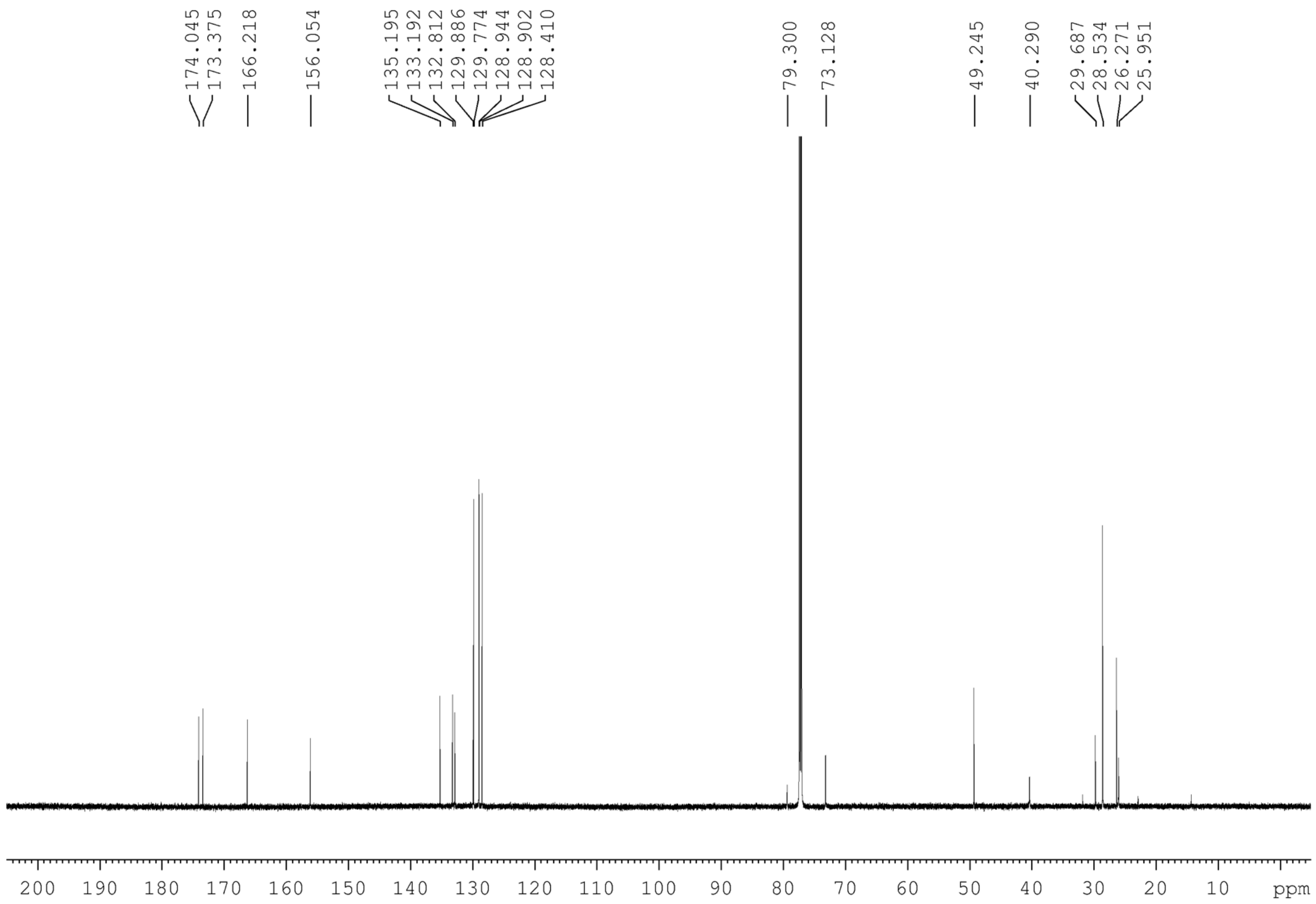

ethyl 7-acetamido-6-hydroxyheptanoate (1m-rac)

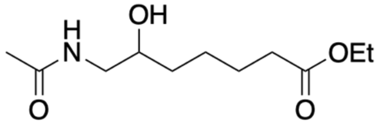

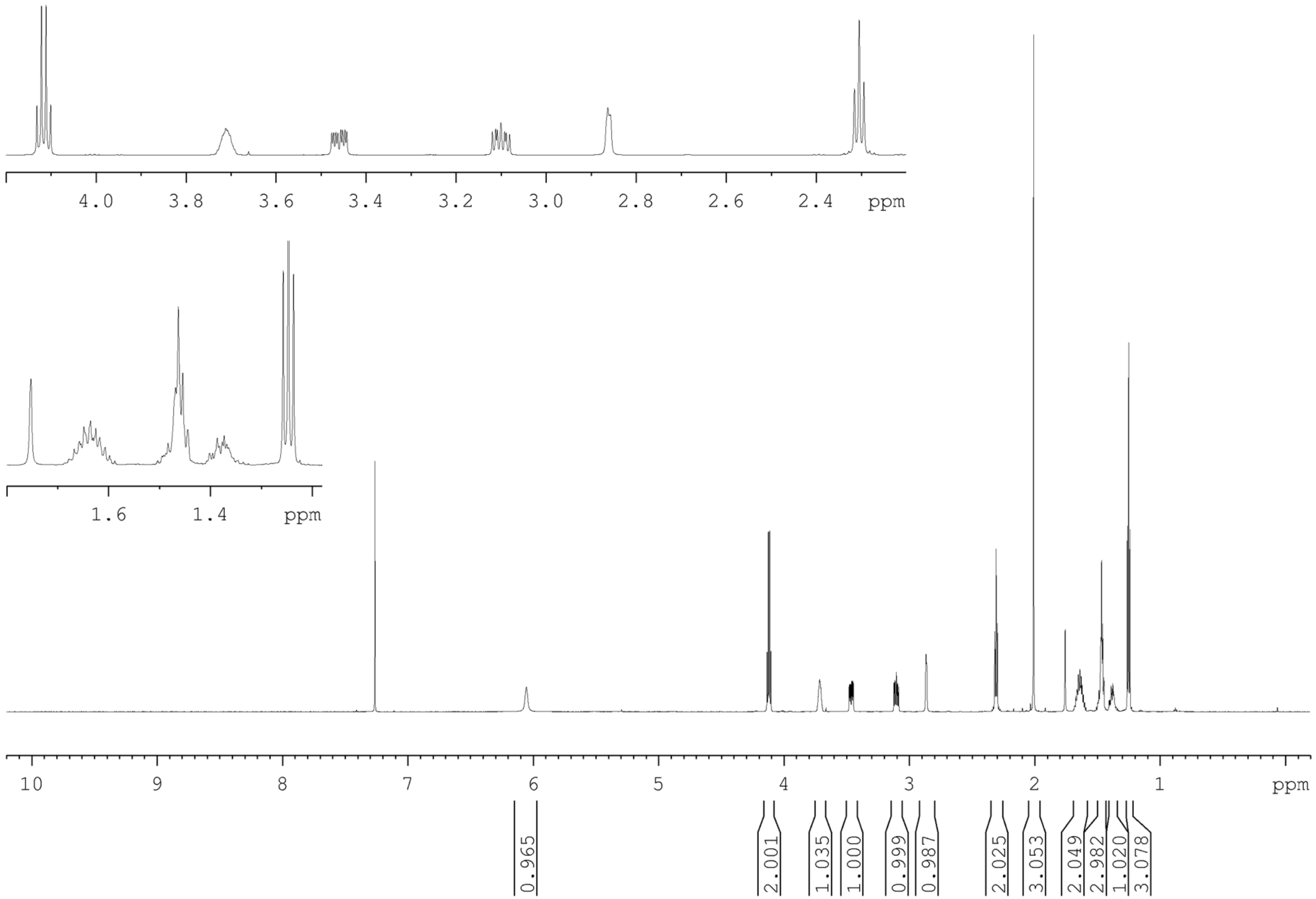

ethyl 7-acetamido-6-hydroxyheptanoate (1m-rac)

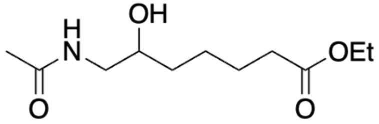

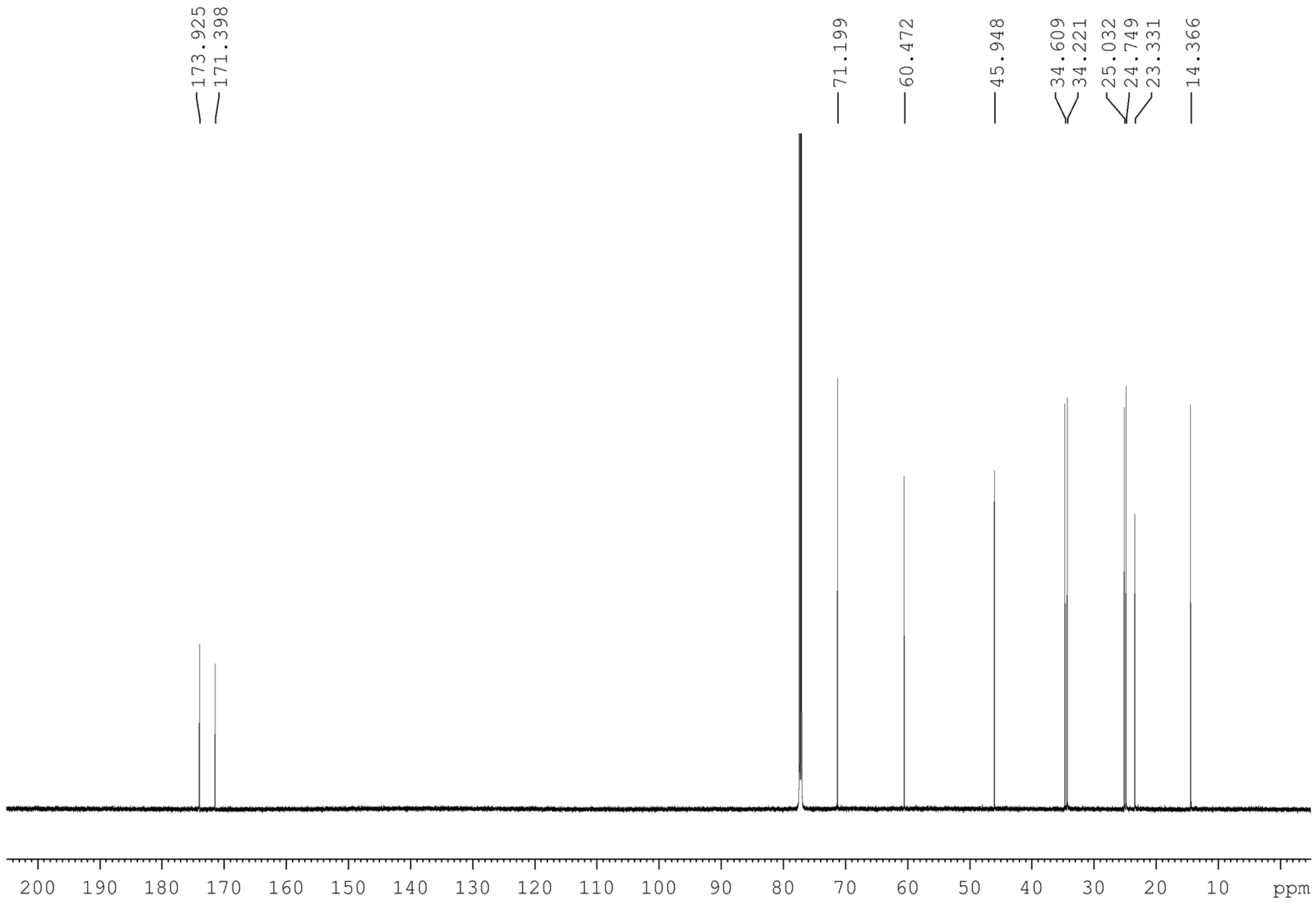

ethyl (*S*)-7-acetamido-6-hydroxyheptanoate (1m)

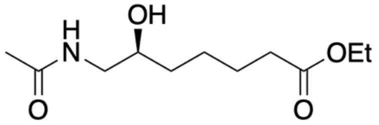

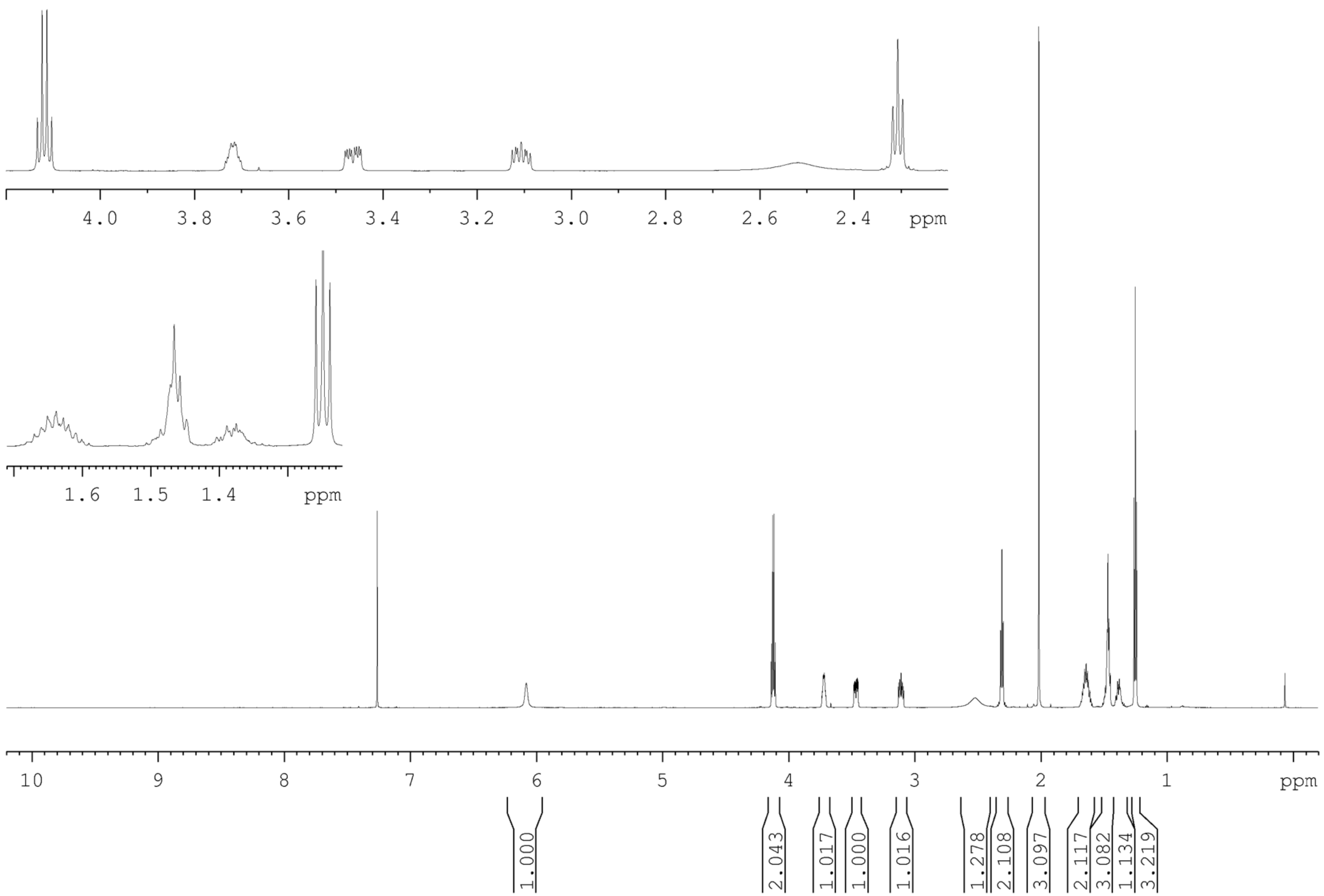

ethyl (*S*)-7-acetamido-6-hydroxyheptanoate (1m)

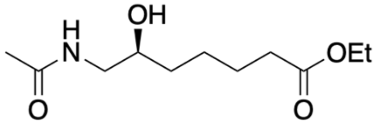

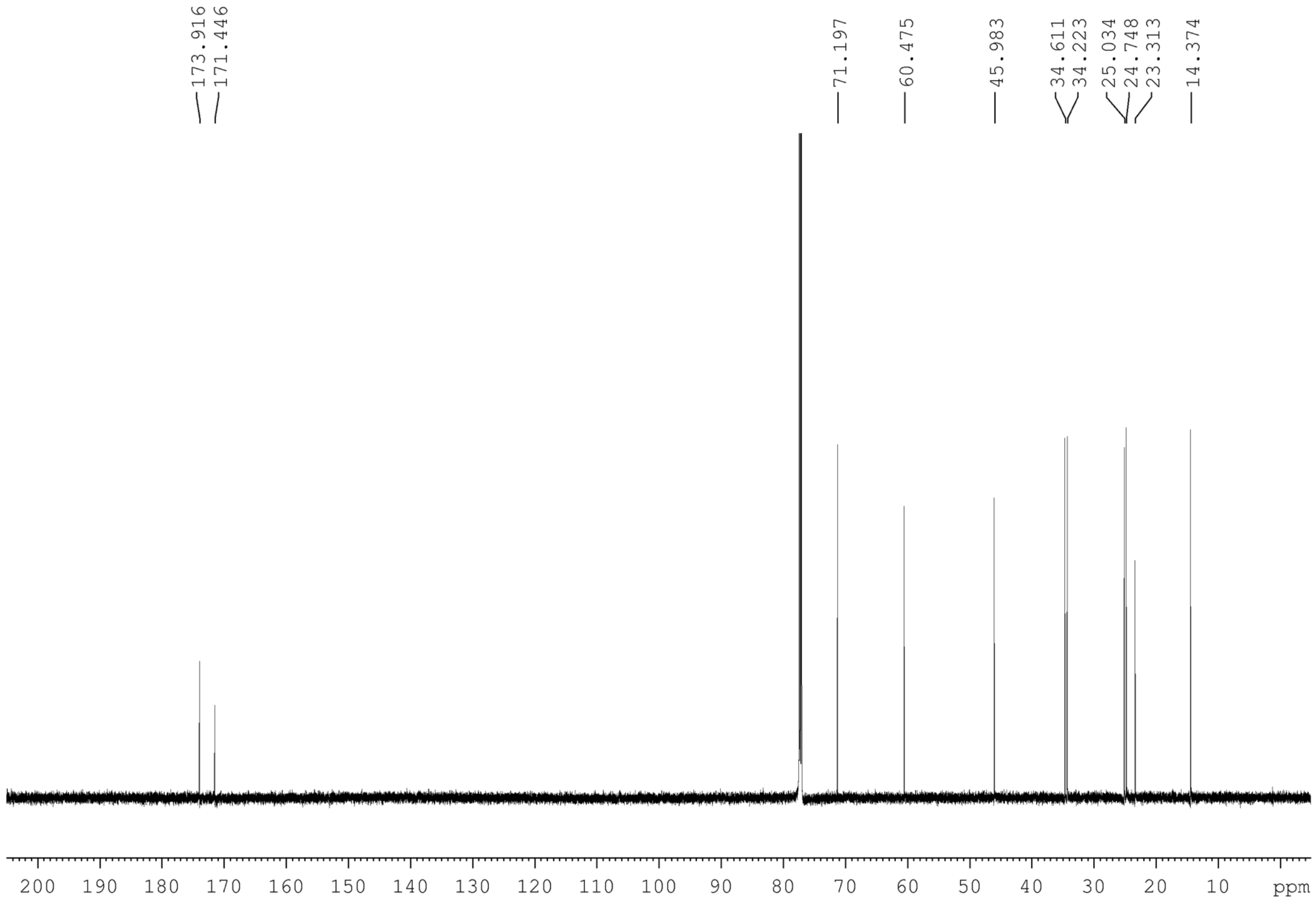

(*S*)-1-(*N*-acetylbenzamido)-7-ethoxy-7-oxoheptan-2-yl benzoate (1m-Bz)

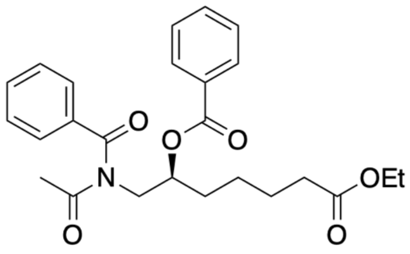

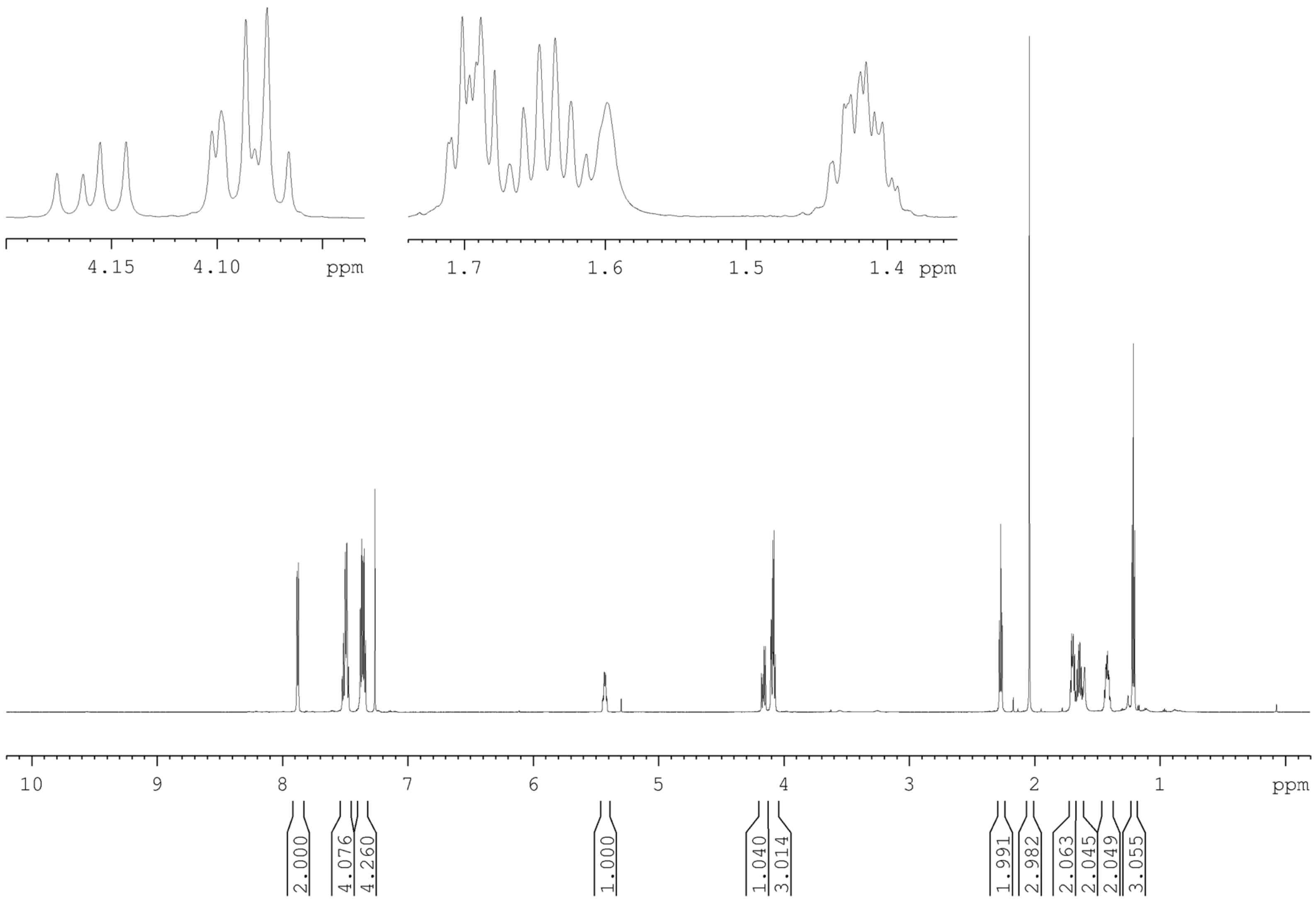

(*S*)-1-(*N*-acetylbenzamido)-7-ethoxy-7-oxoheptan-2-yl benzoate (1m-Bz)

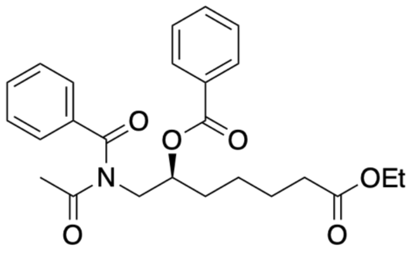

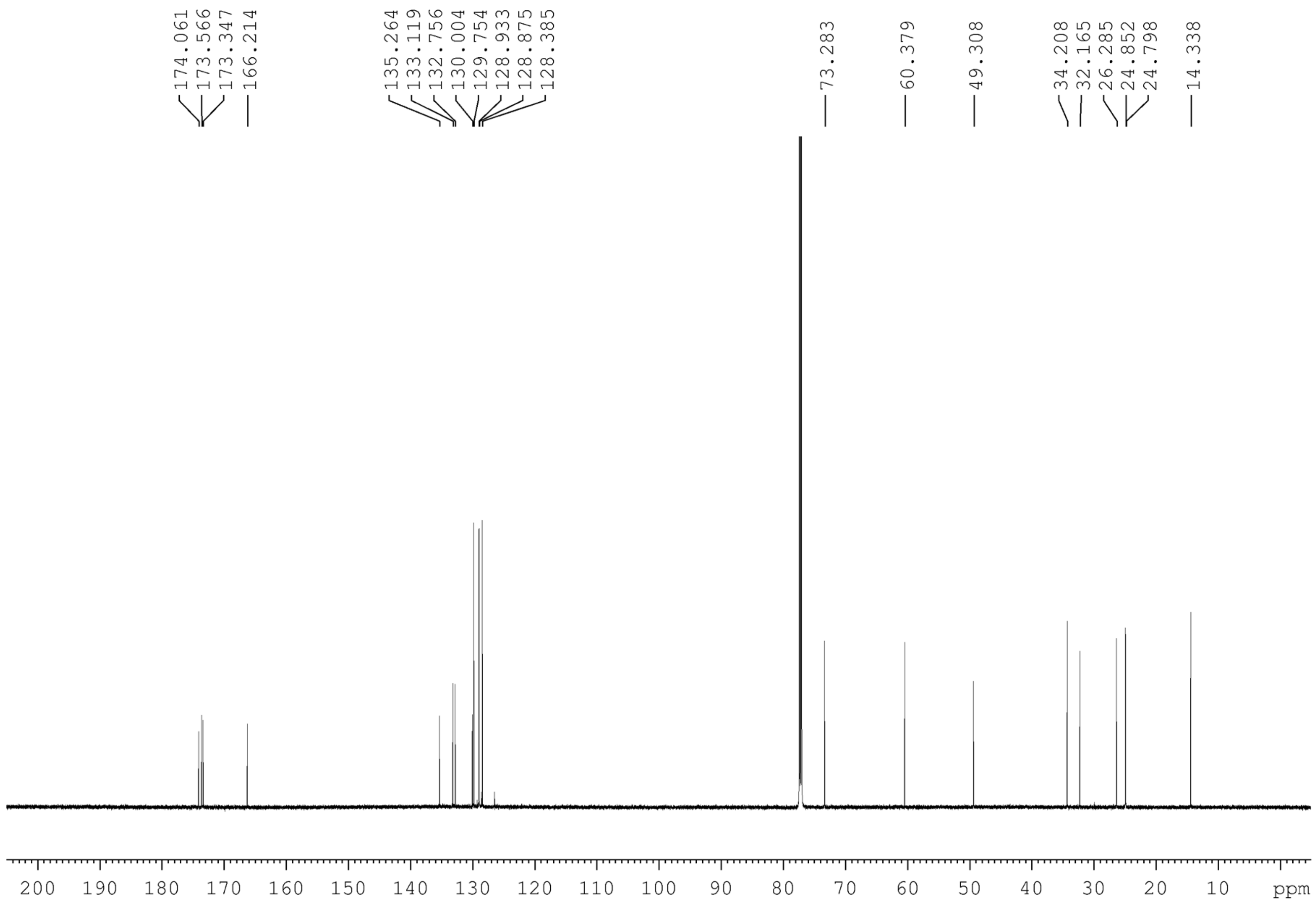

1-amino-5-((*tert*-butyldimethylsilyl)oxy)pentan-2-ol (1n-I_2)

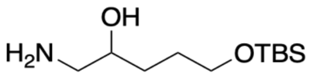

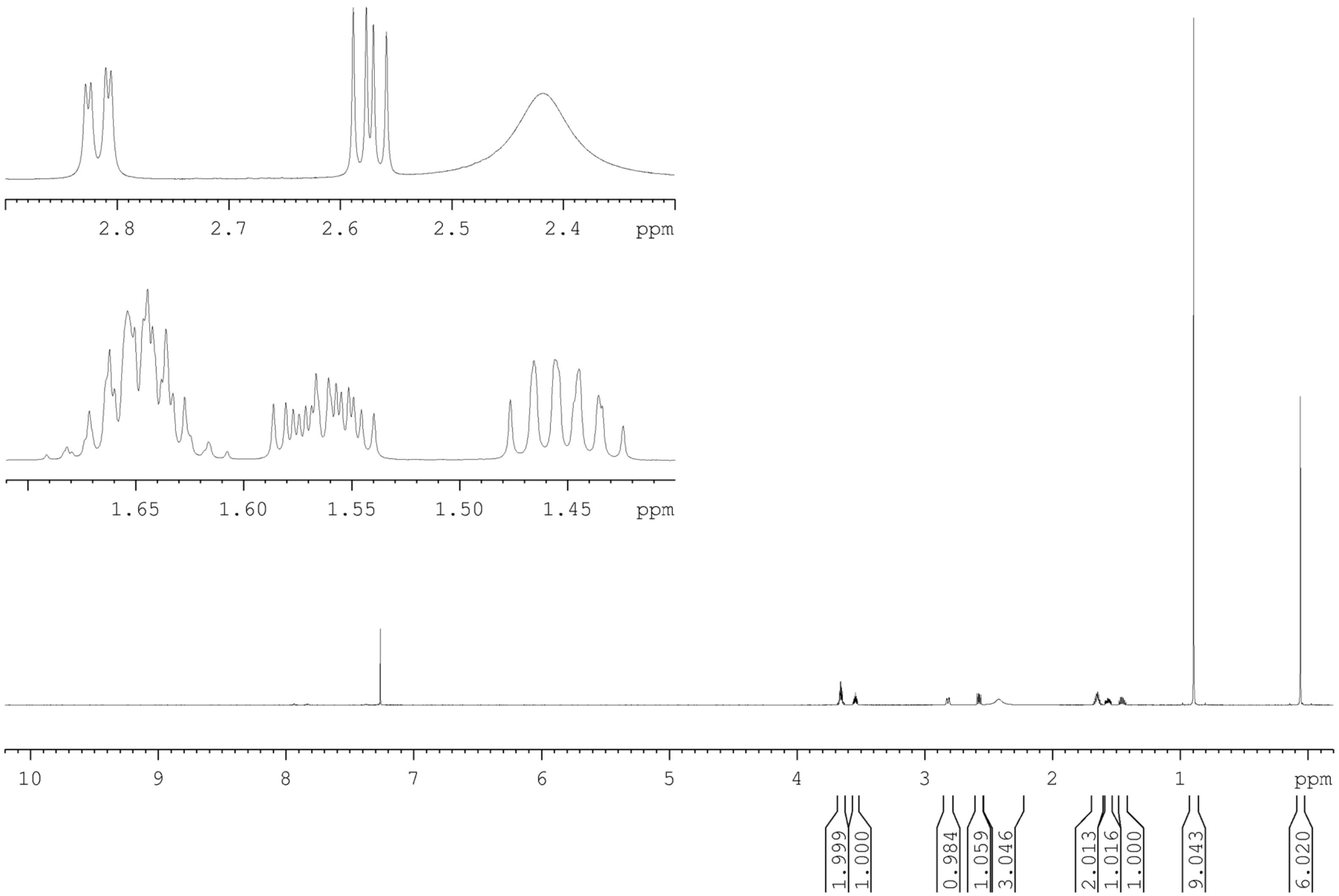

1-amino-5-((*tert*-butyldimethylsilyl)oxy)pentan-2-ol (1n-I_2)

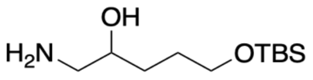

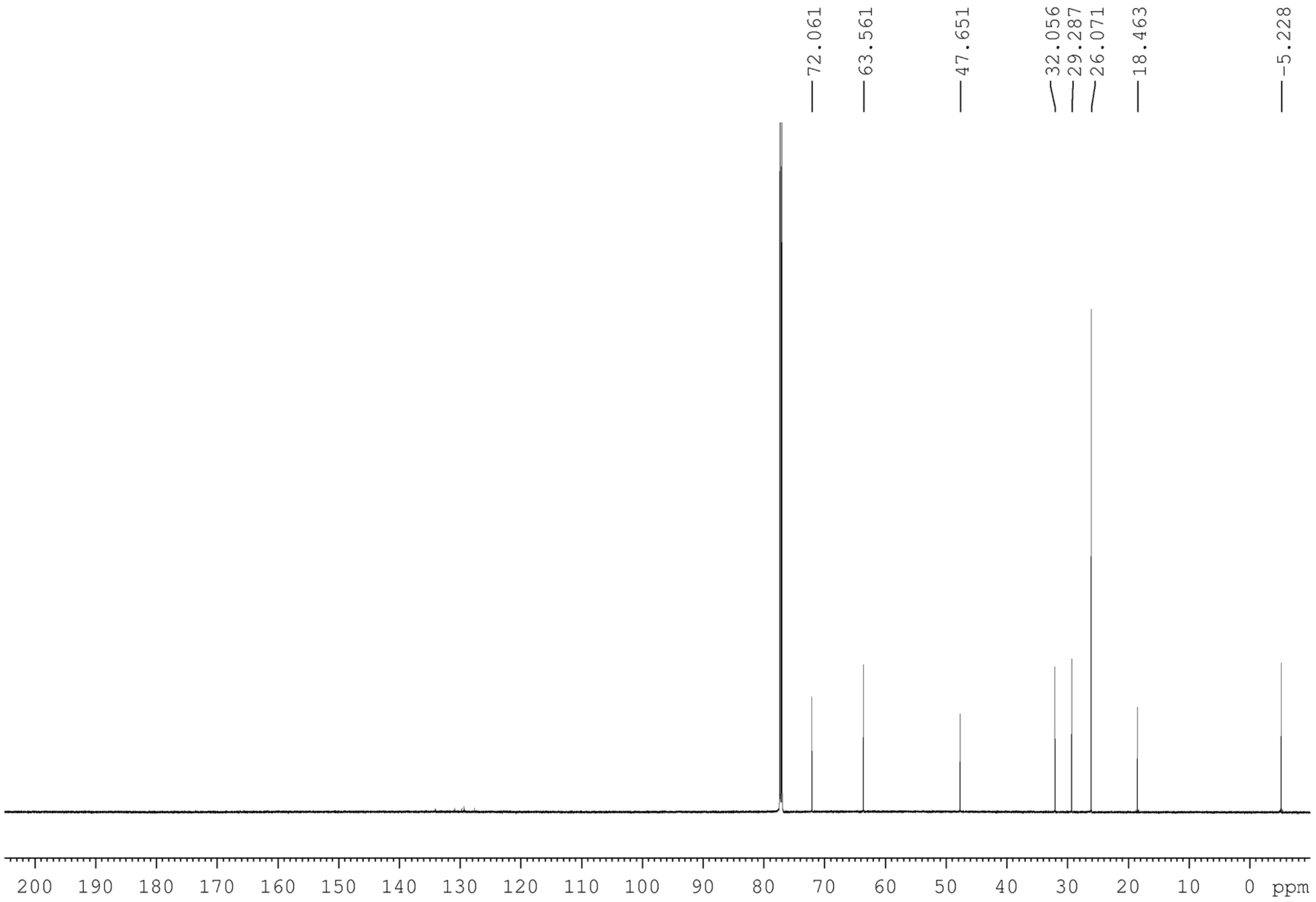

N-(5-((*tert*-butyldimethylsilyl)oxy)-2-hydroxypentyl)acetamide (1n-rac)

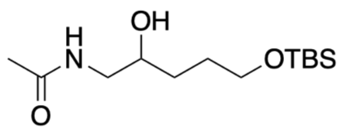

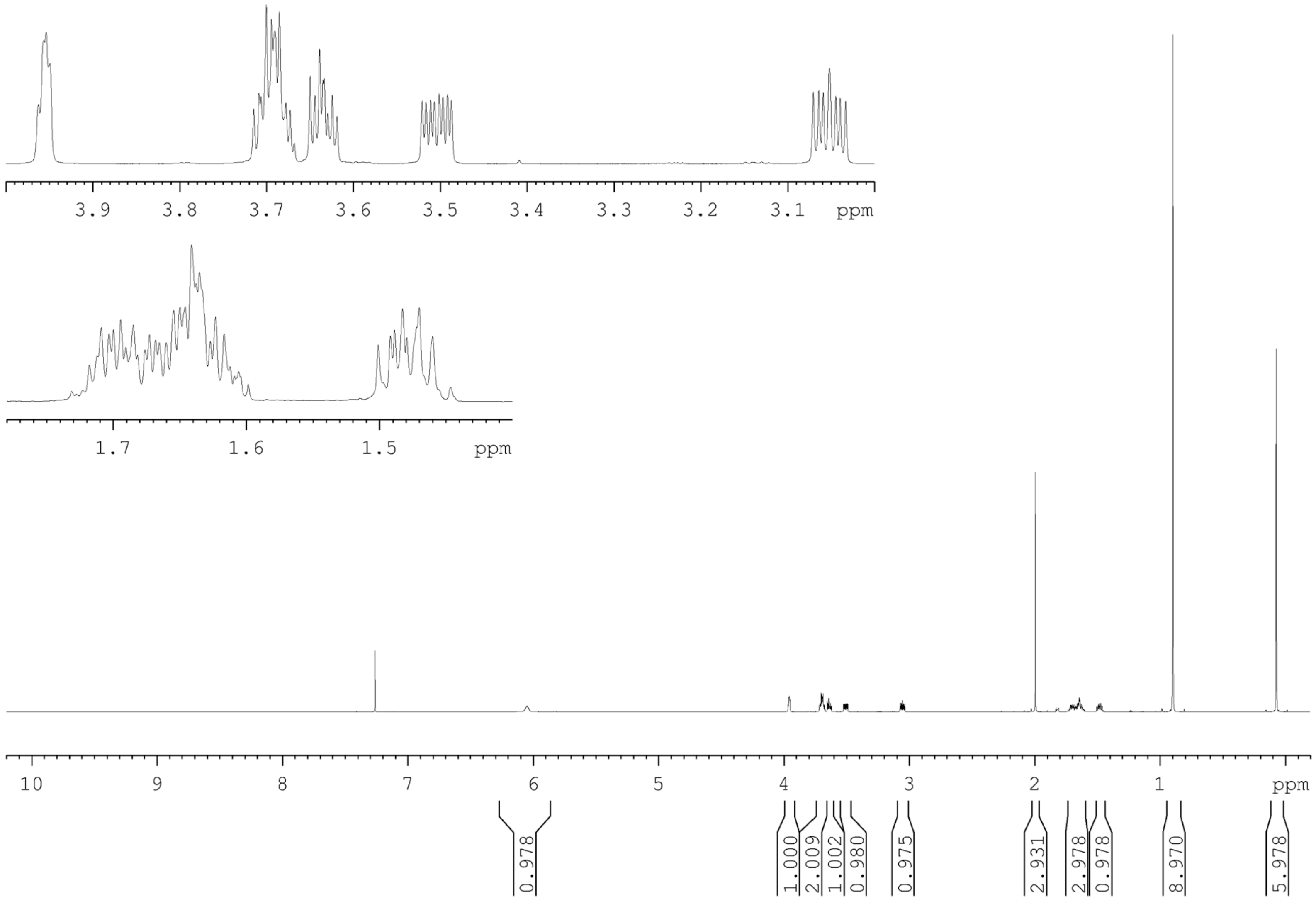

N-(5-((tert-butyldimethylsilyl)oxy)-2-hydroxypentyl)acetamide (1n-rac)

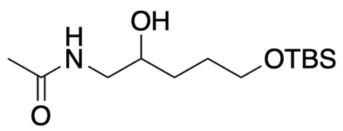

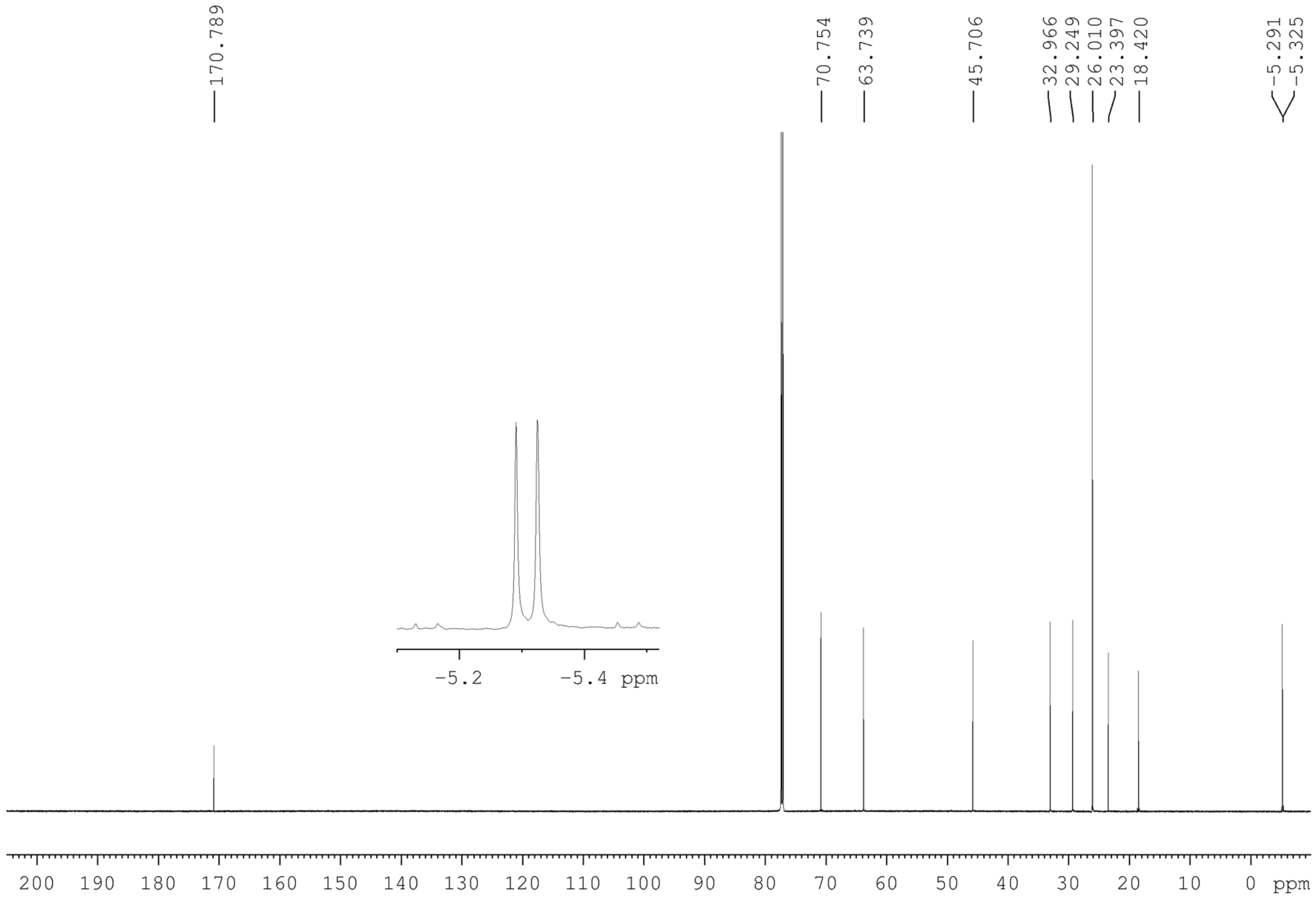

(*S*)-*N*-(5-((*tert*-butyldimethylsilyl)oxy)-2-hydroxypentyl)acetamide (1n)

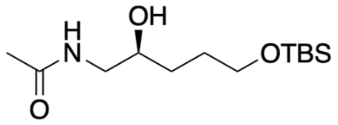

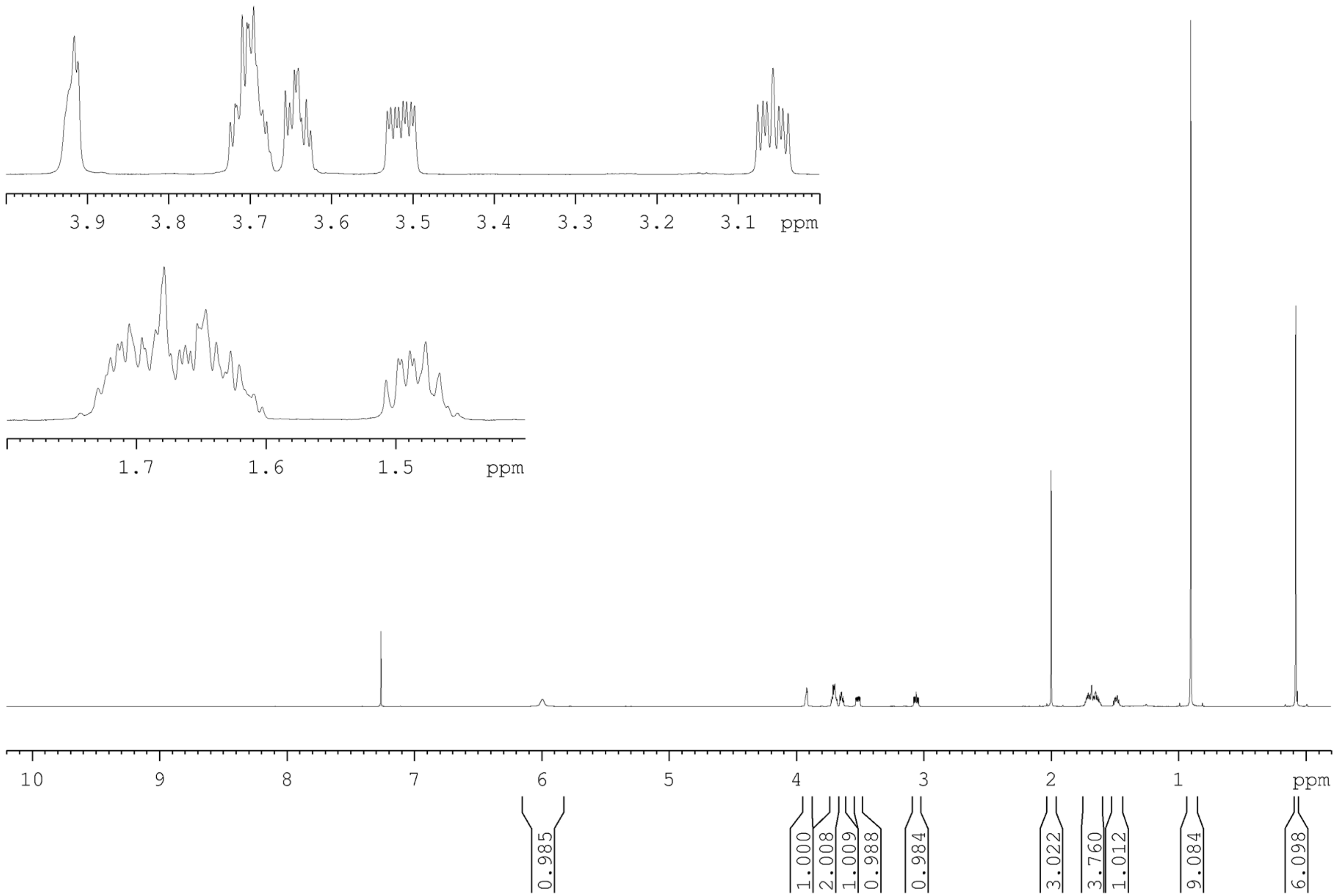

(*S*)-*N*-(5-((*tert*-butyldimethylsilyl)oxy)-2-hydroxypentyl)acetamide (1n)

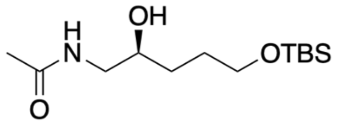

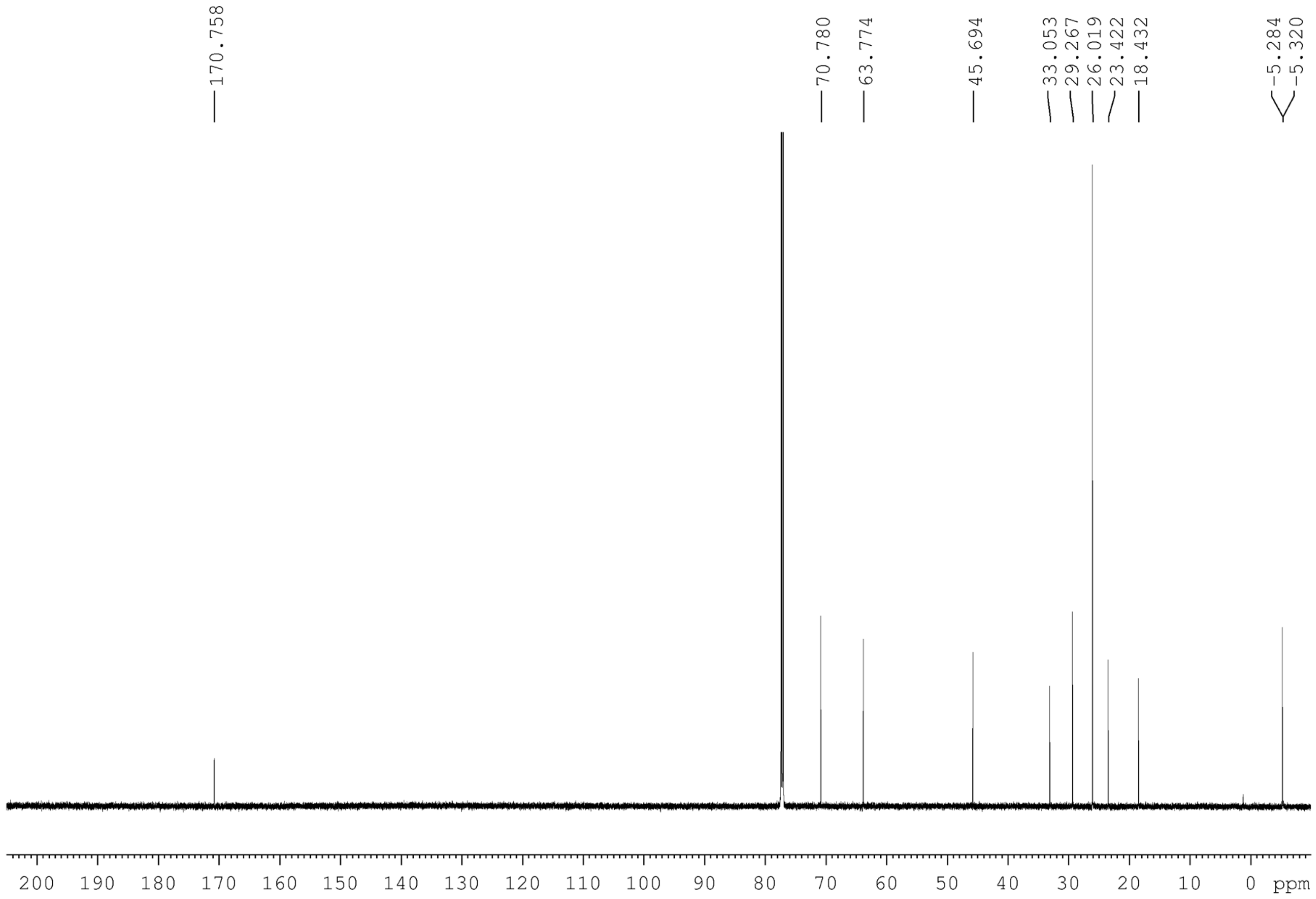

(*S*)-1-(*N*-acetylbenzamido)-5-((*tert*-butyldimethylsilyl)oxy)pentan-2-yl benzoate (1n-Bz)

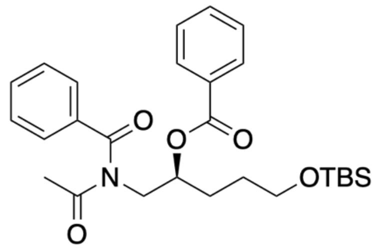

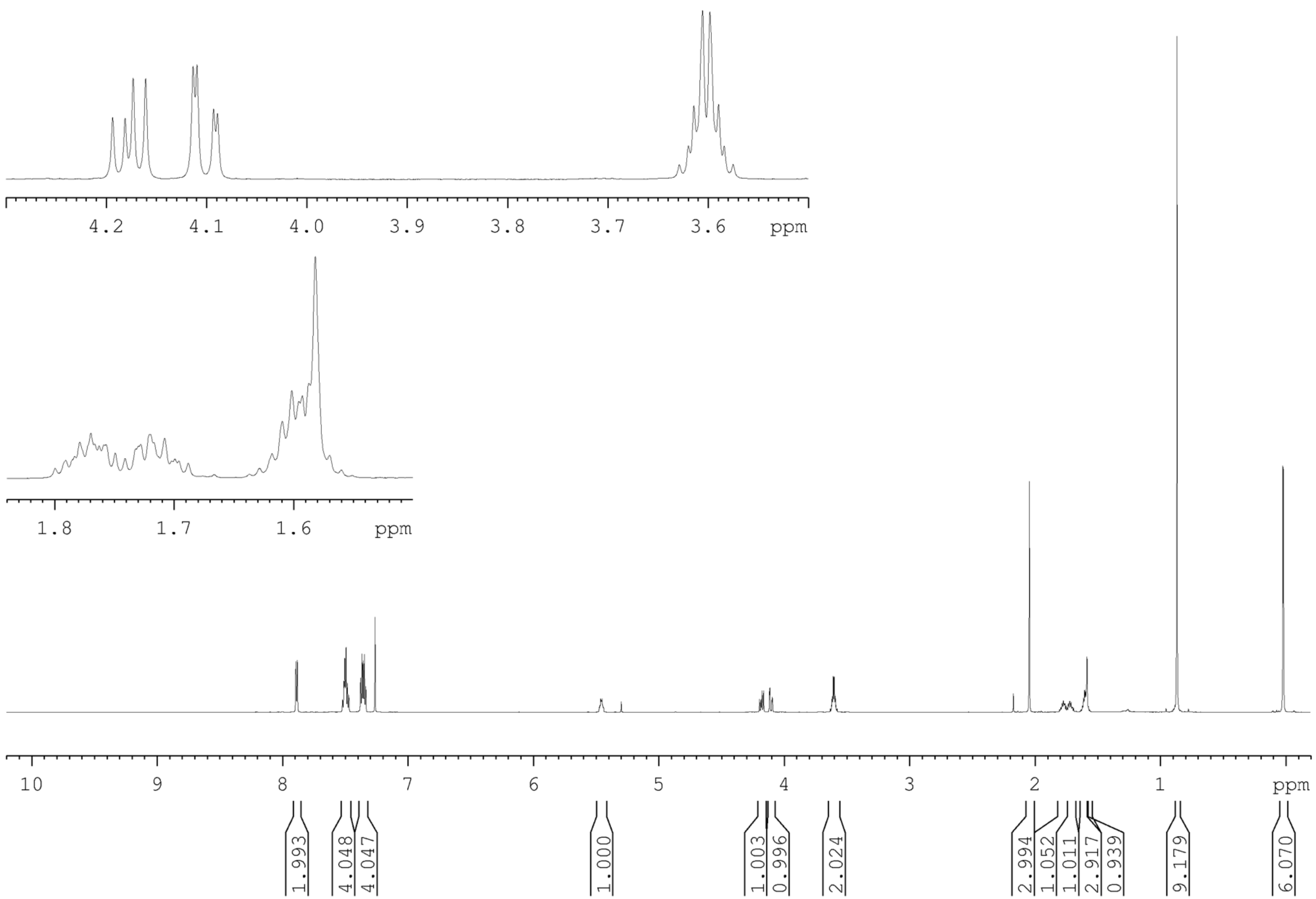

(*S*)-1-(*N*-acetylbenzamido)-5-((*tert*-butyldimethylsilyl)oxy)pentan-2-yl benzoate (1n-Bz)

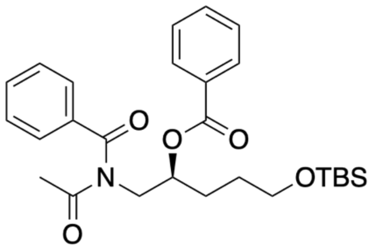

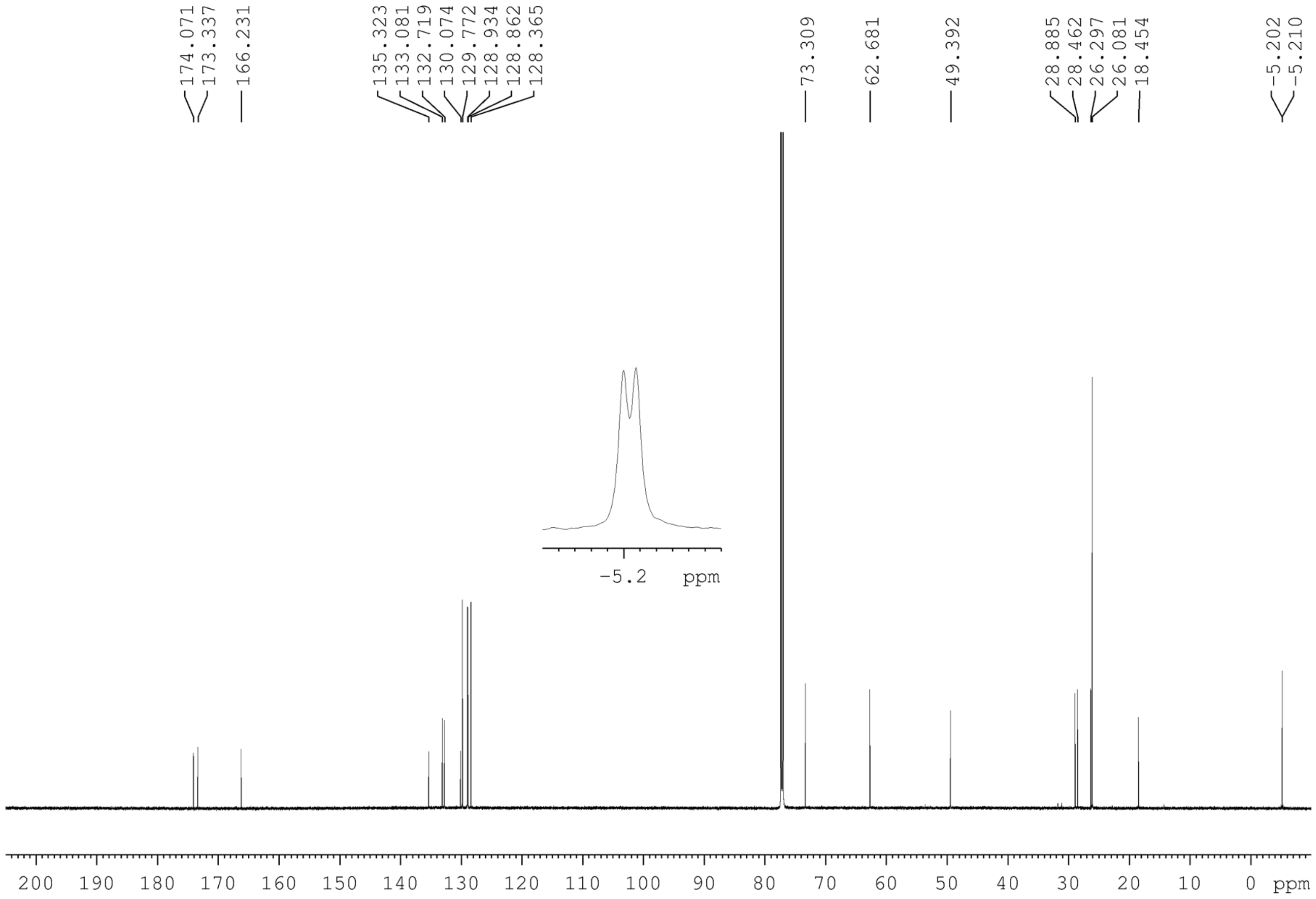

1-amino-5-ethoxypentan-2-ol (1o-I_3)

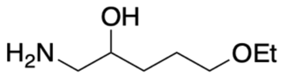

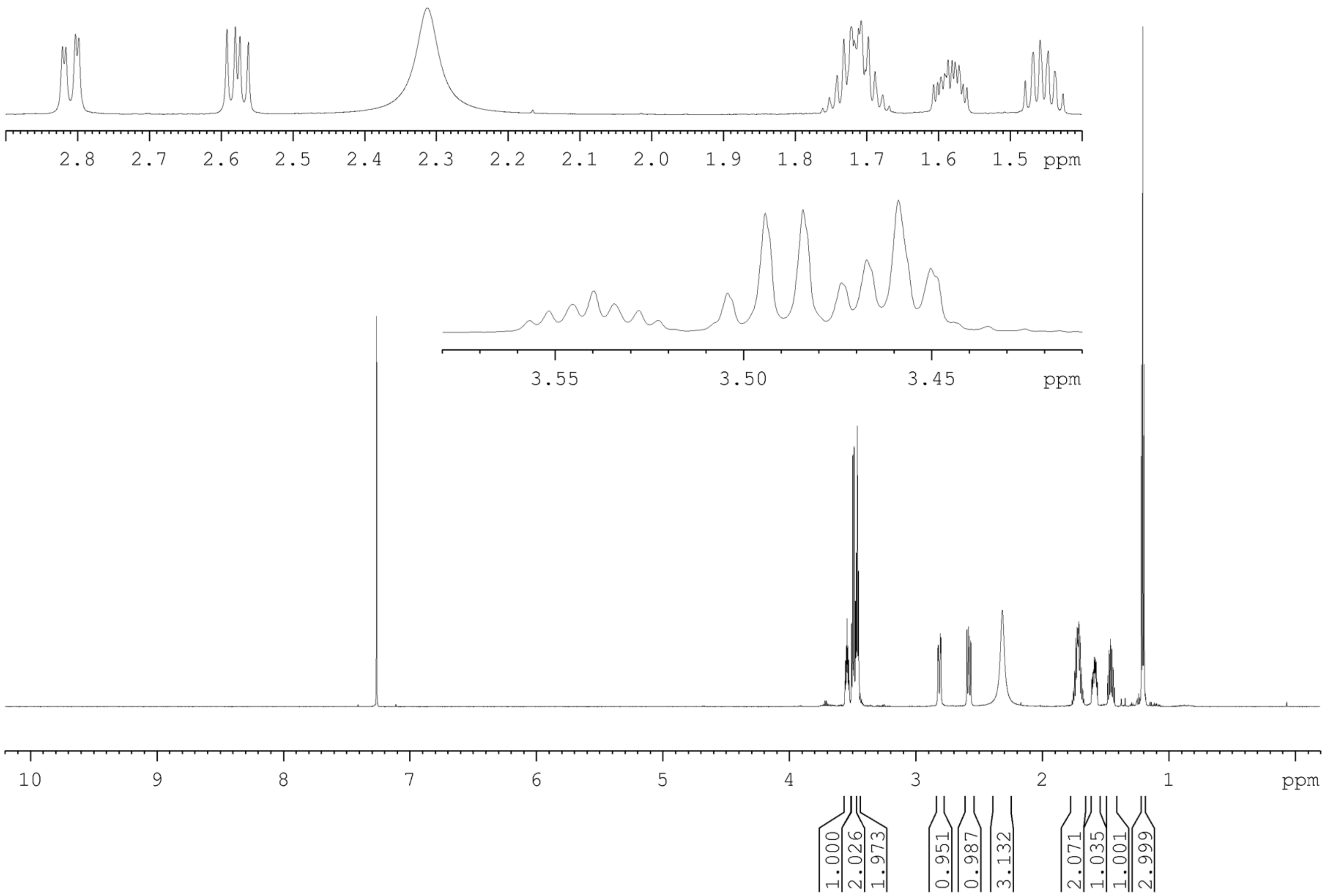

1-amino-5-ethoxypentan-2-ol (1o-I_3)

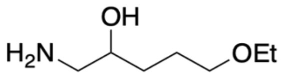

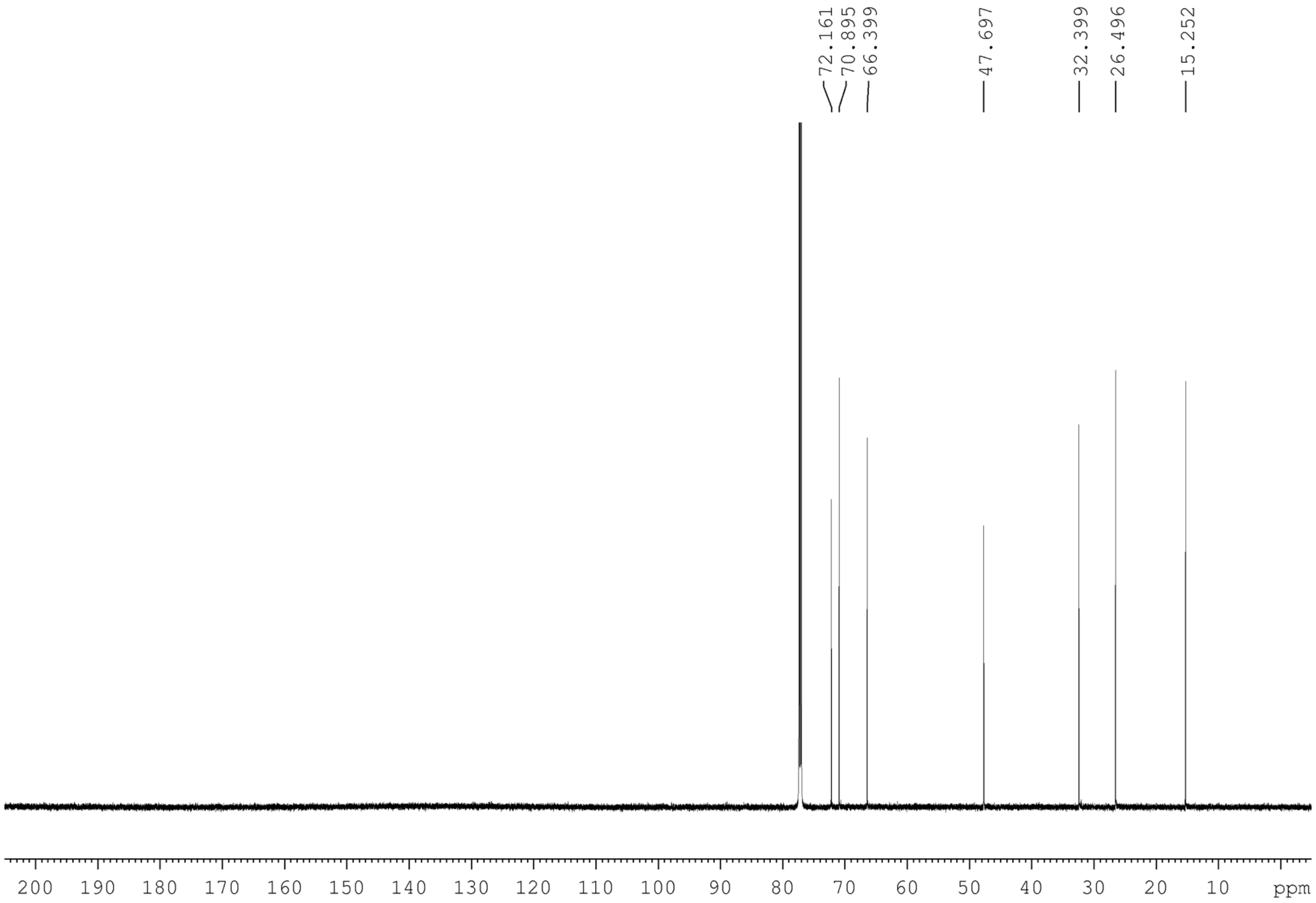

N-(5-ethoxy-2-hydroxypentyl)acetamide (1o-rac)

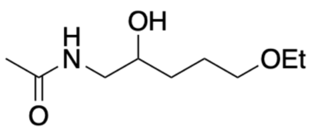

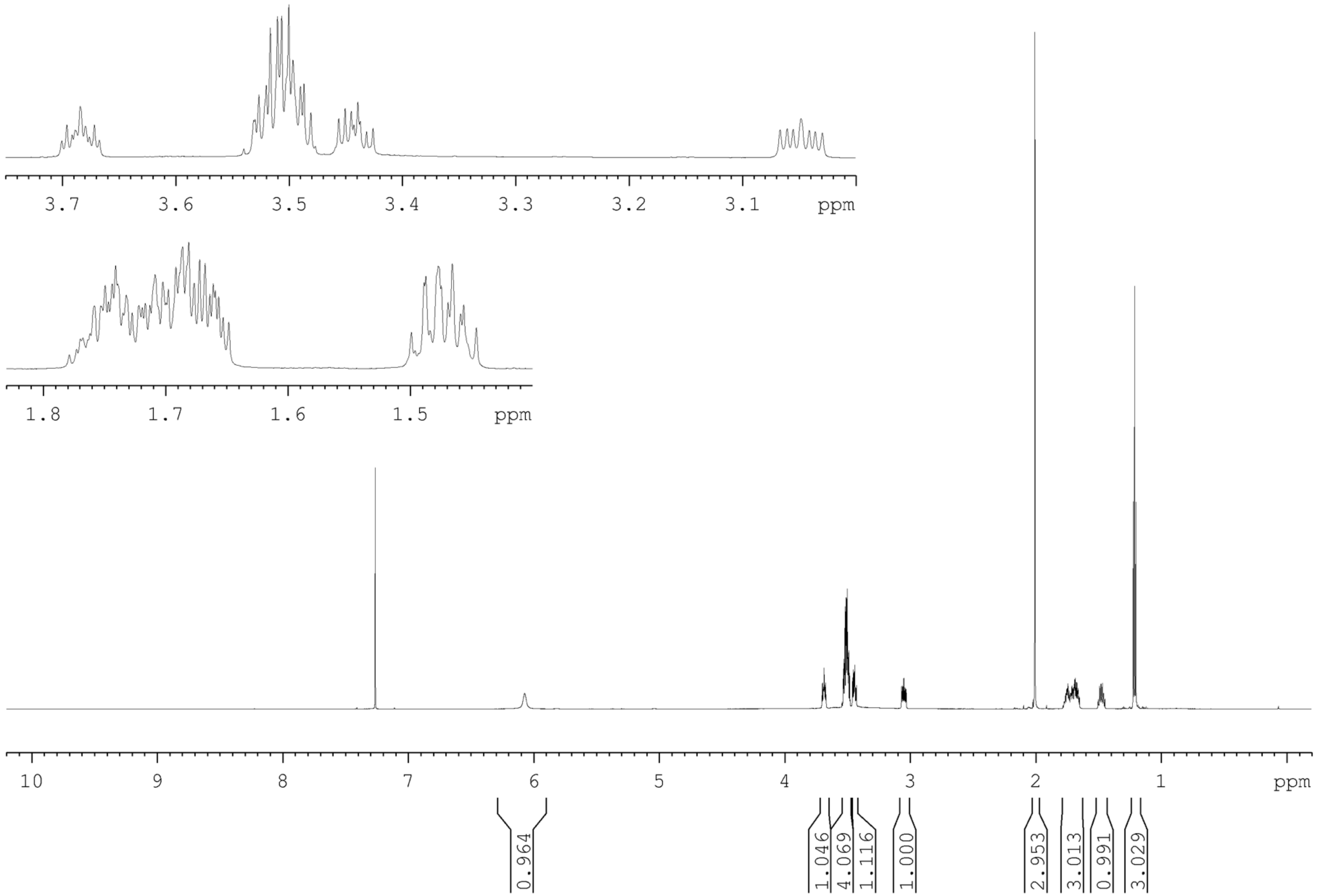

N-(5-ethoxy-2-hydroxypentyl)acetamide (1o-rac)

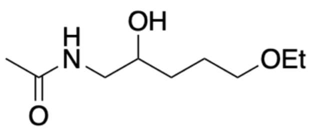

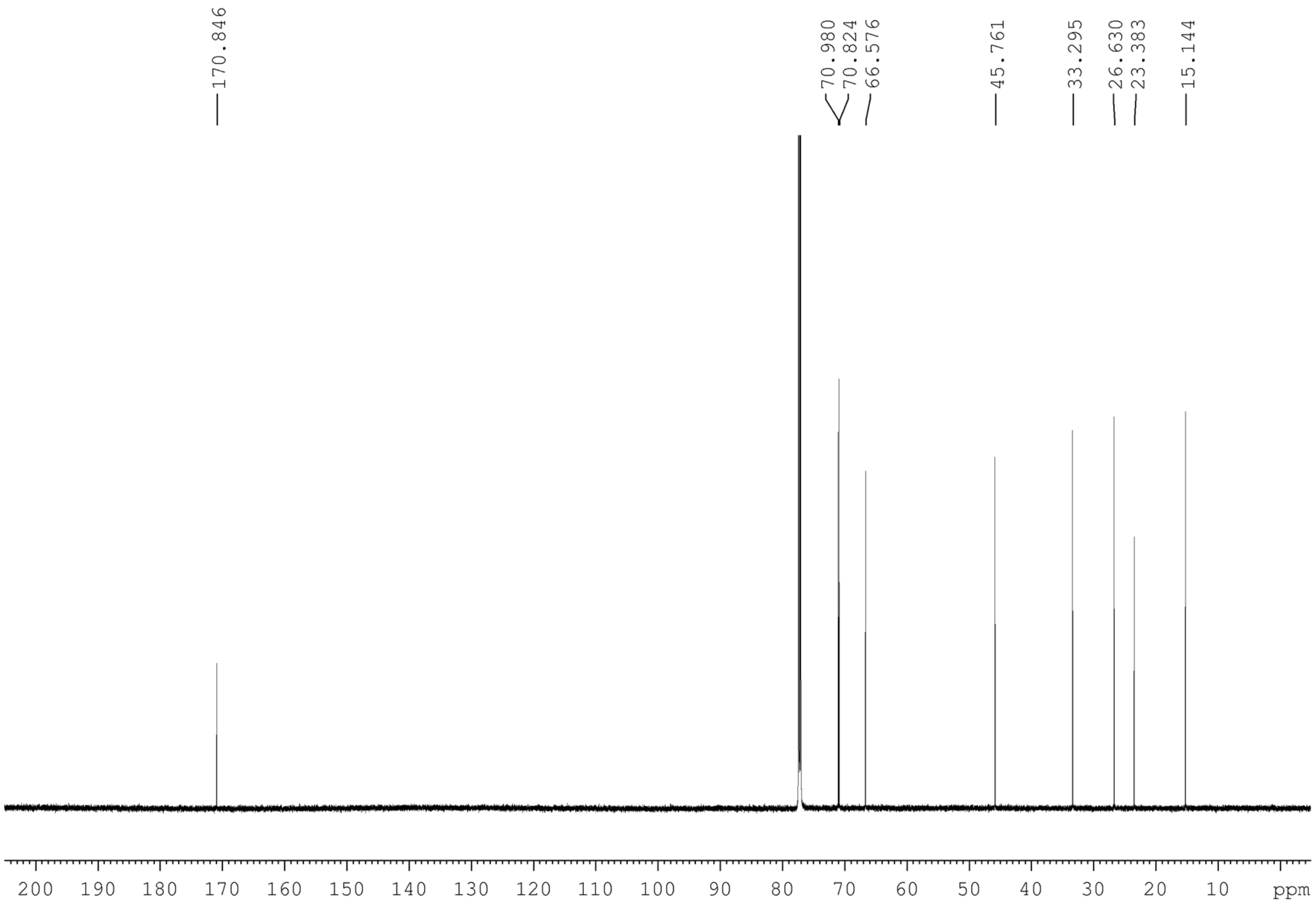

(*S*)-*N*-(5-ethoxy-2-hydroxypentyl)acetamide (1o)

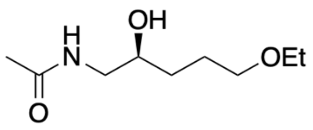

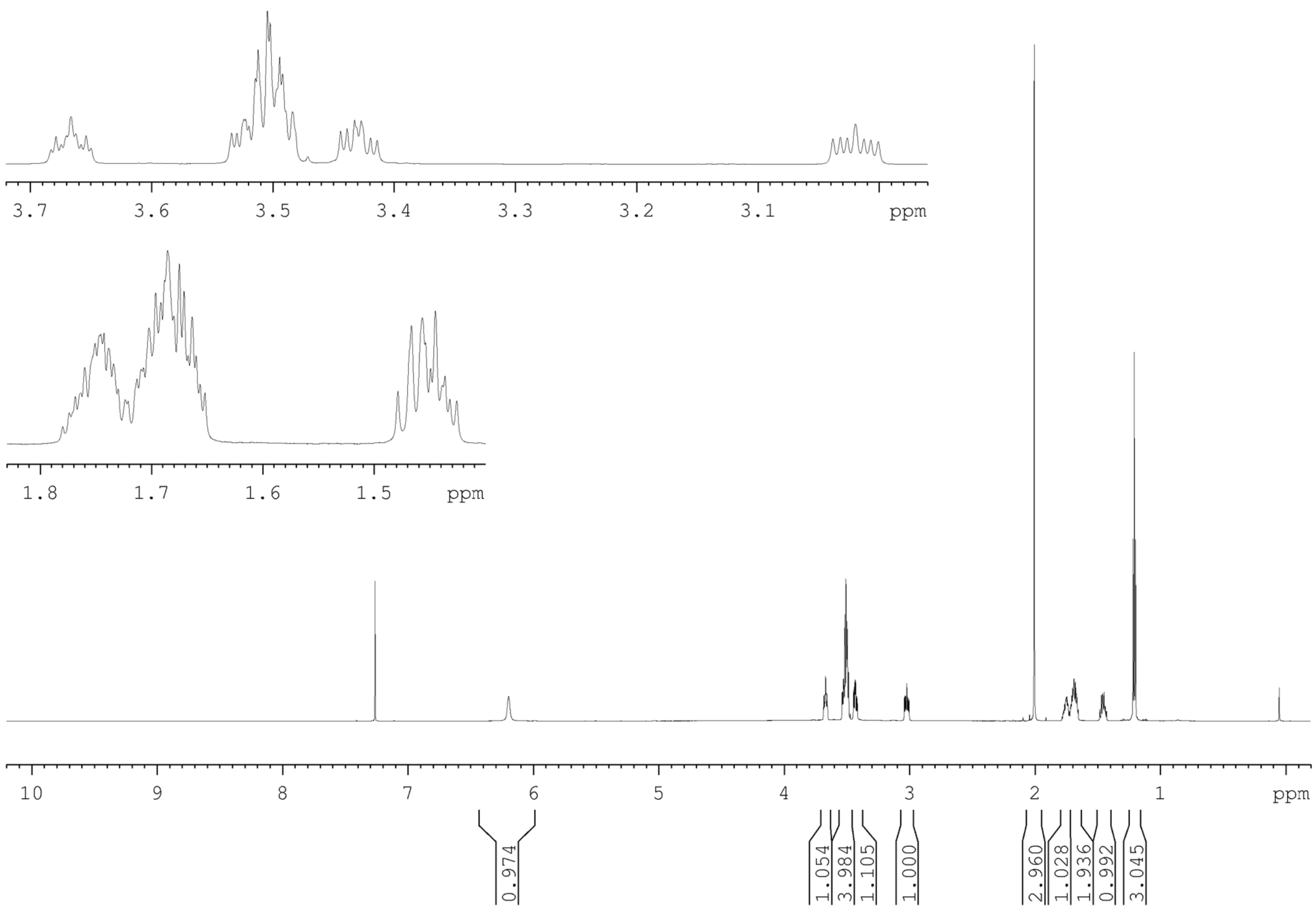

(*S*)-*N*-(5-ethoxy-2-hydroxypentyl)acetamide (1o)

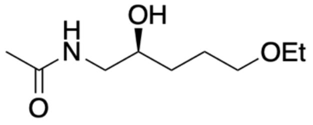

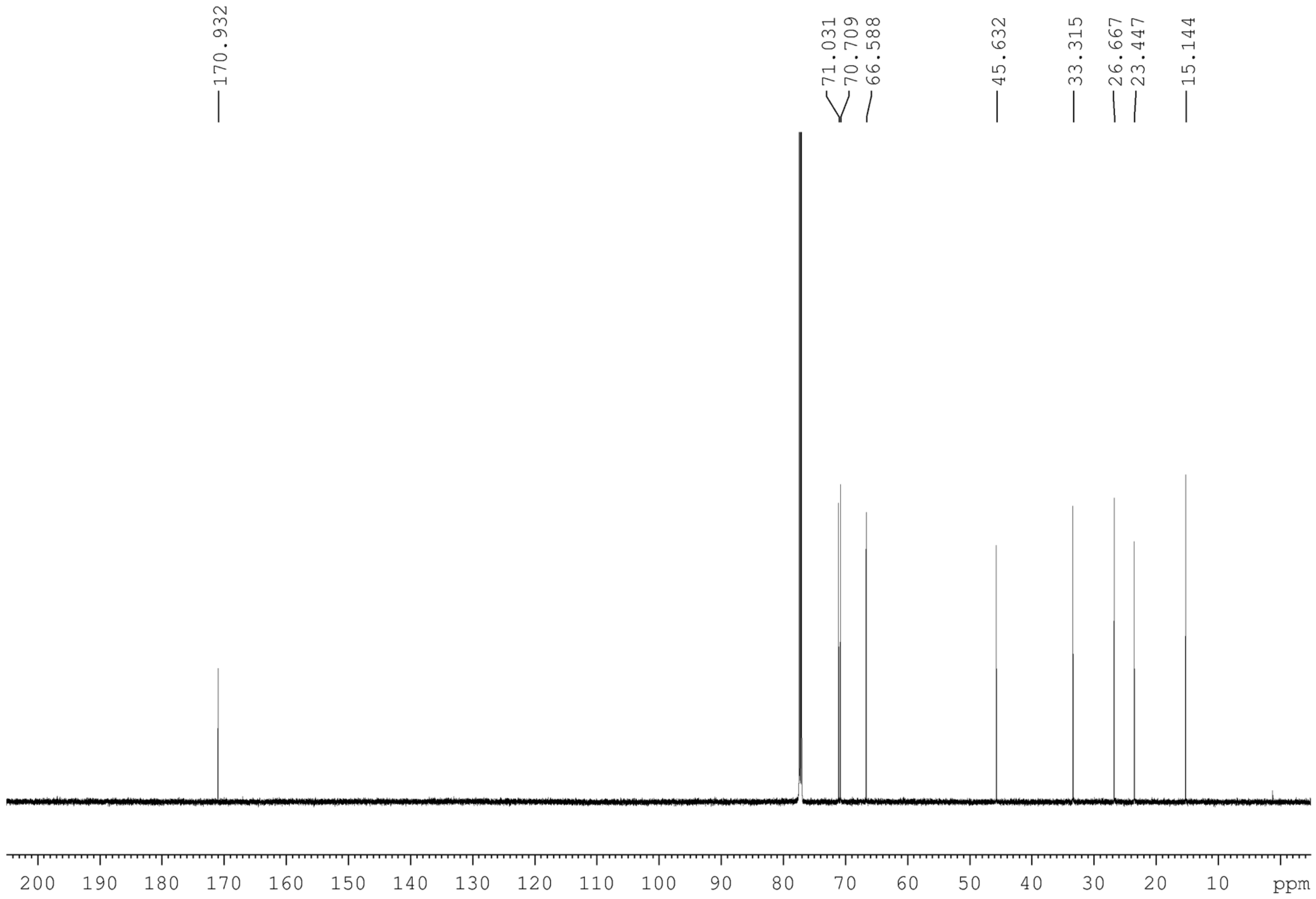

(*S*)-1-(*N*-acetylbenzamido)-5-ethoxypentan-2-yl benzoate (1o-Bz)

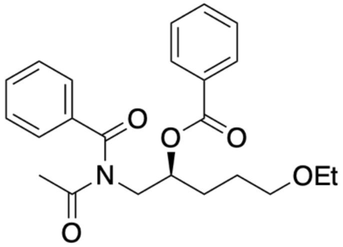

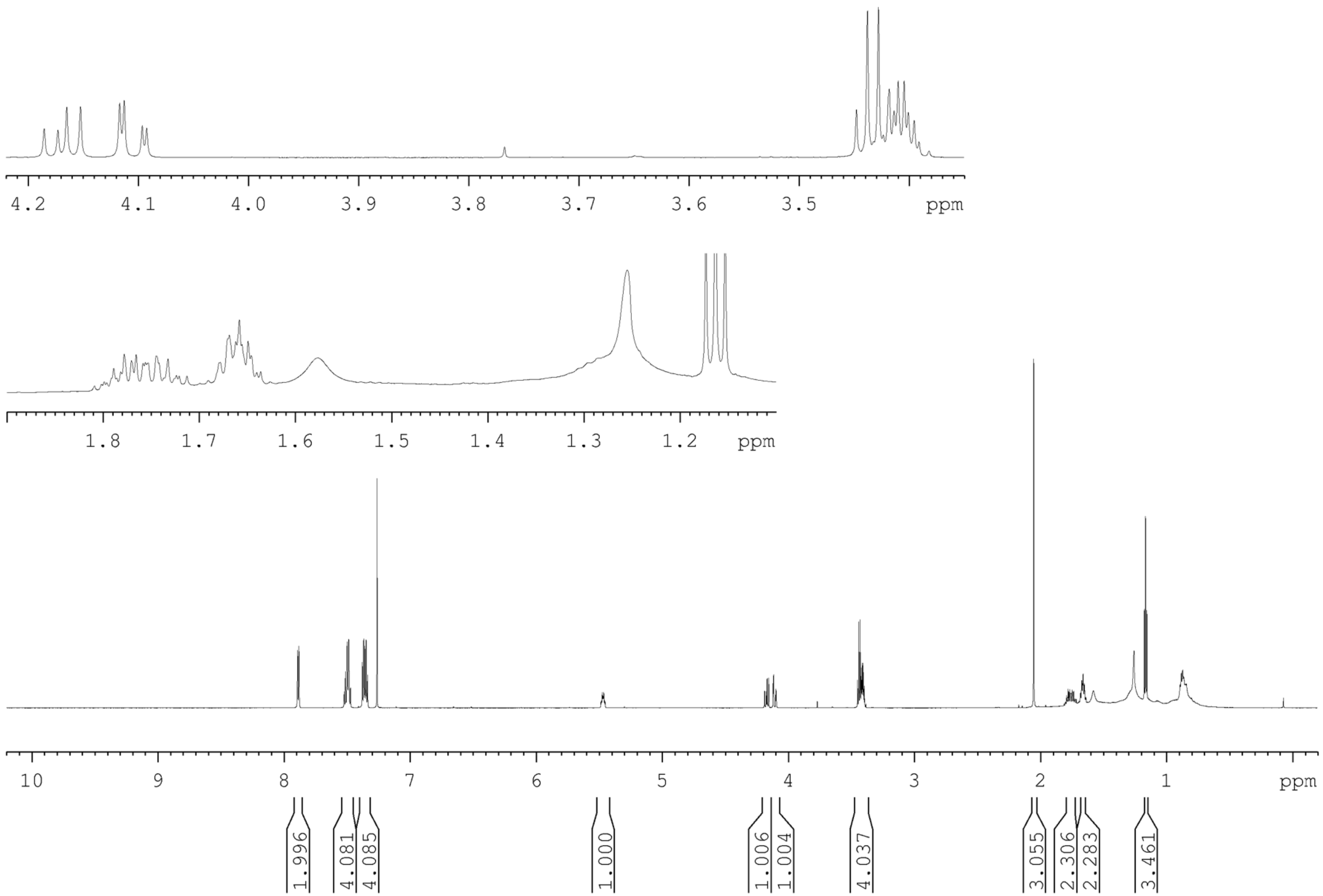

(*S*)-1-(*N*-acetylbenzamido)-5-ethoxypentan-2-yl benzoate (1o-Bz)

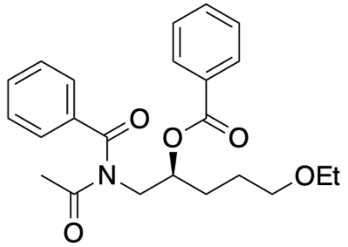

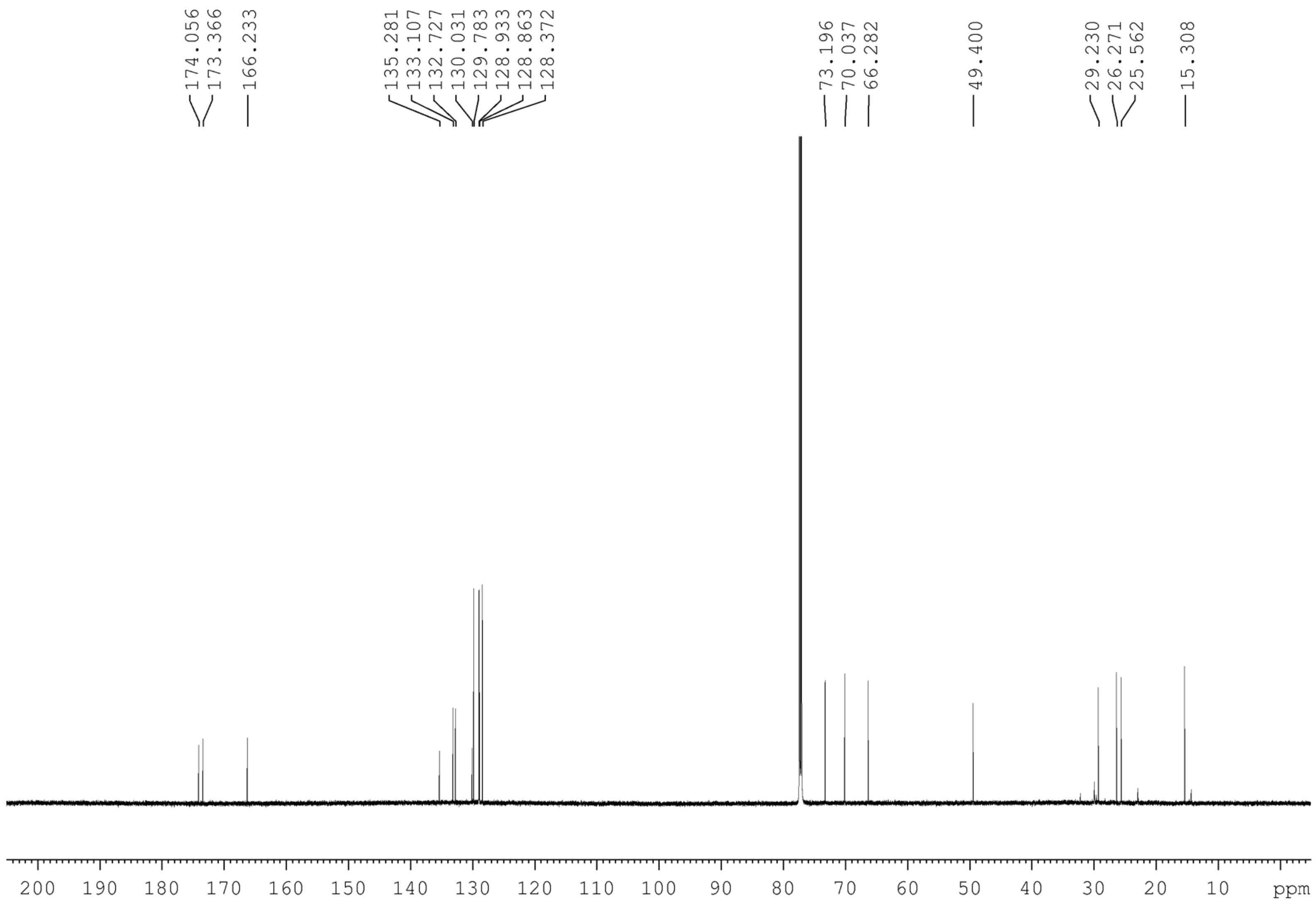

1-amino-4-(tetrahydro-2*H*-pyran-4-yl)butan-2-ol (1p-I_3)

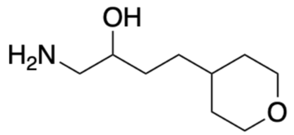

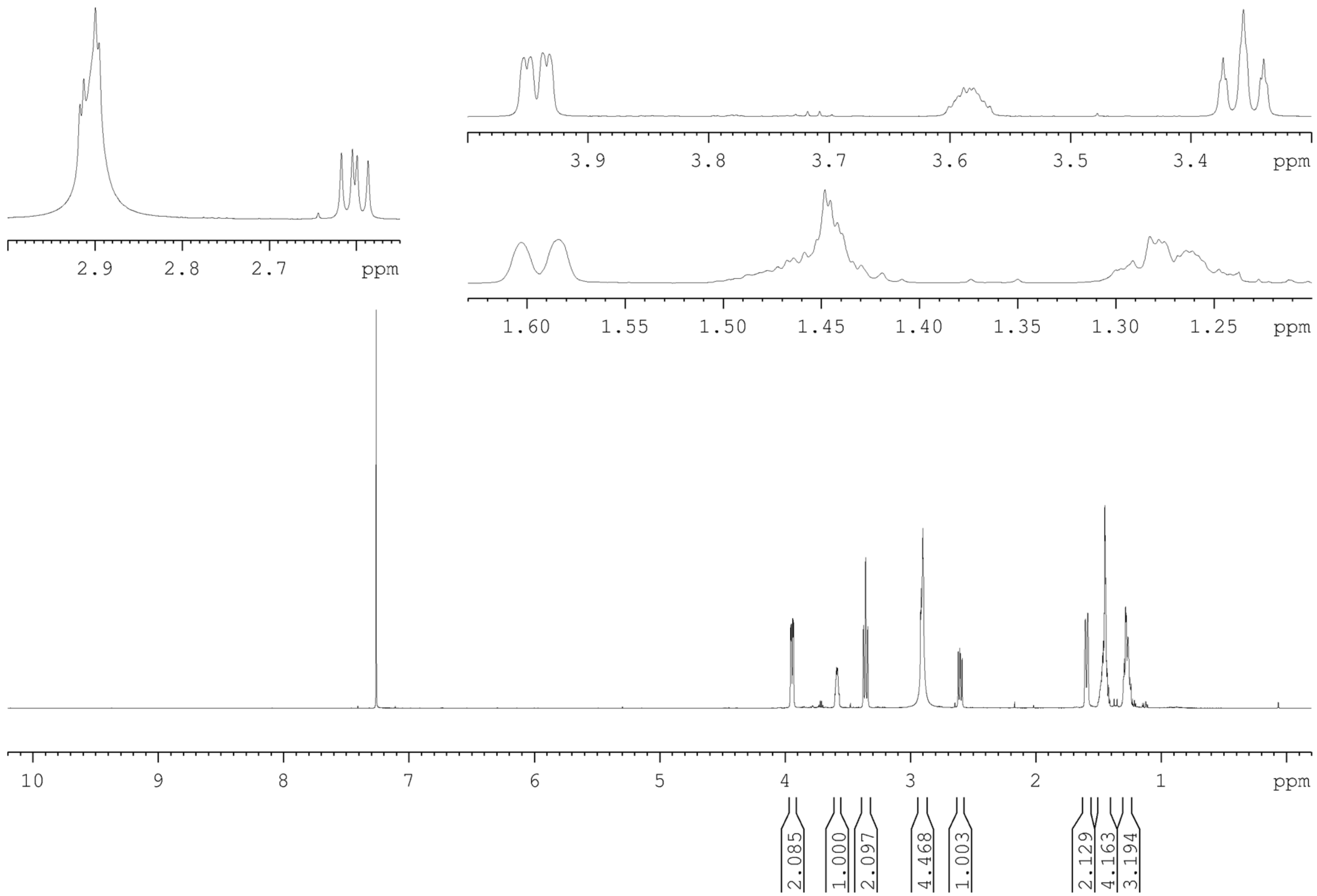

1-amino-4-(tetrahydro-2*H*-pyran-4-yl)butan-2-ol (1p-I_3)

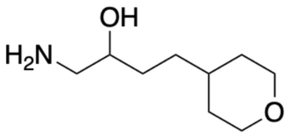

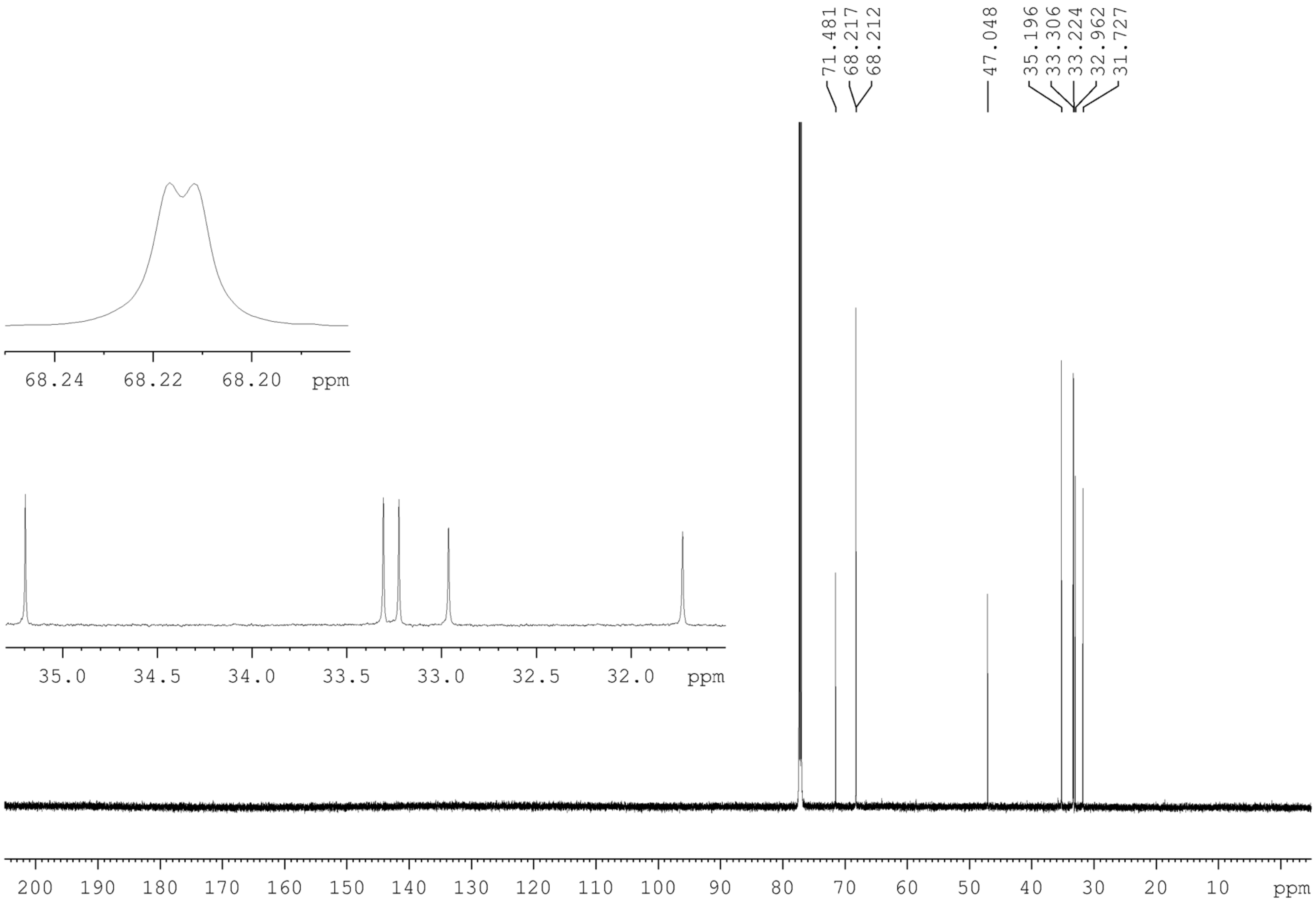

N-(2-hydroxy-4-(tetrahydro-2*H*-pyran-4-yl)butyl)acetamide (1p-rac)

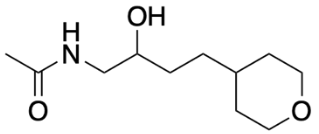

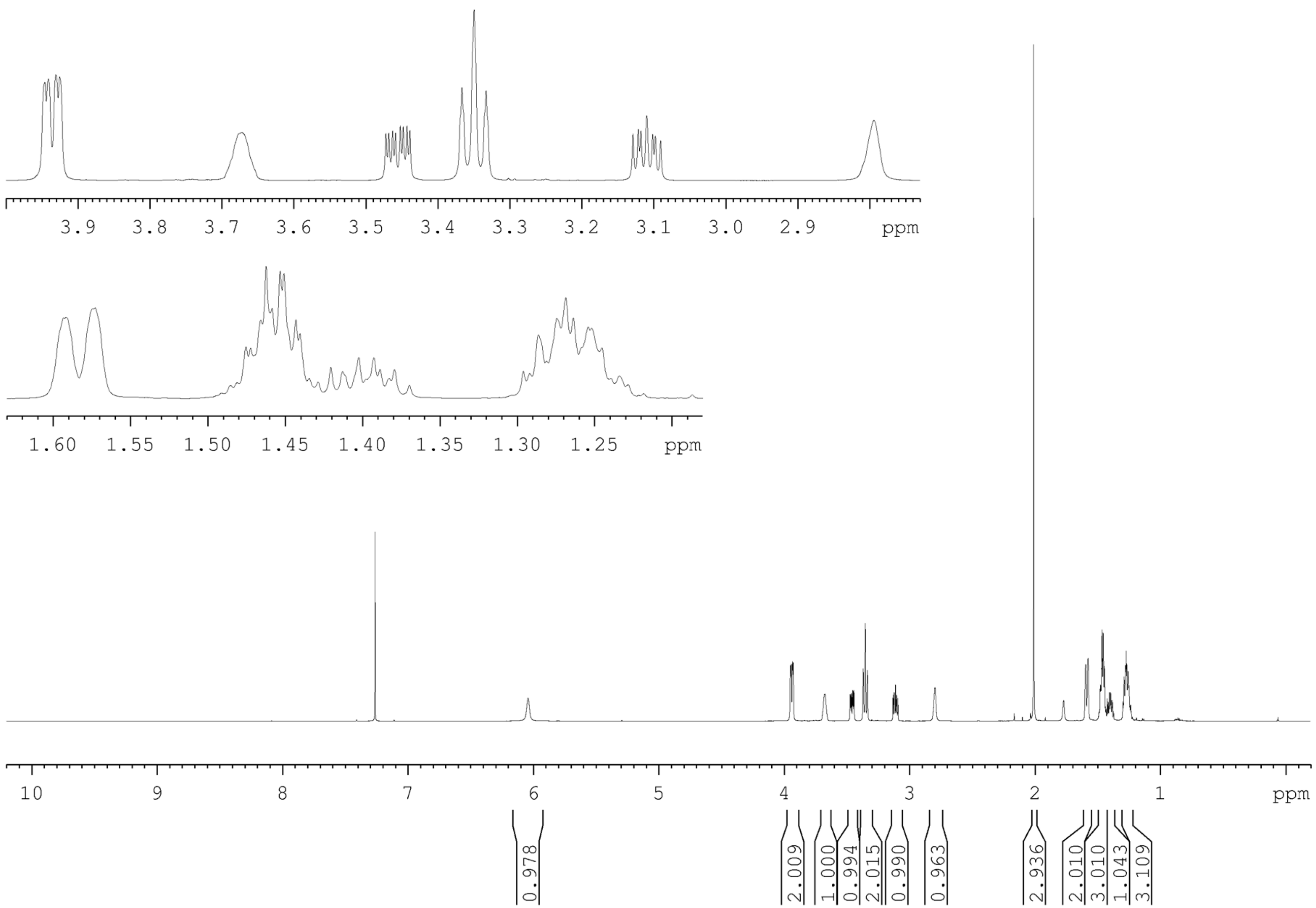

N-(2-hydroxy-4-(tetrahydro-2H-pyran-4-yl)butyl)acetamide (1p-rac)

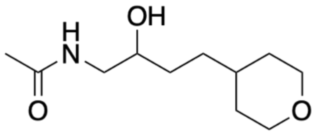

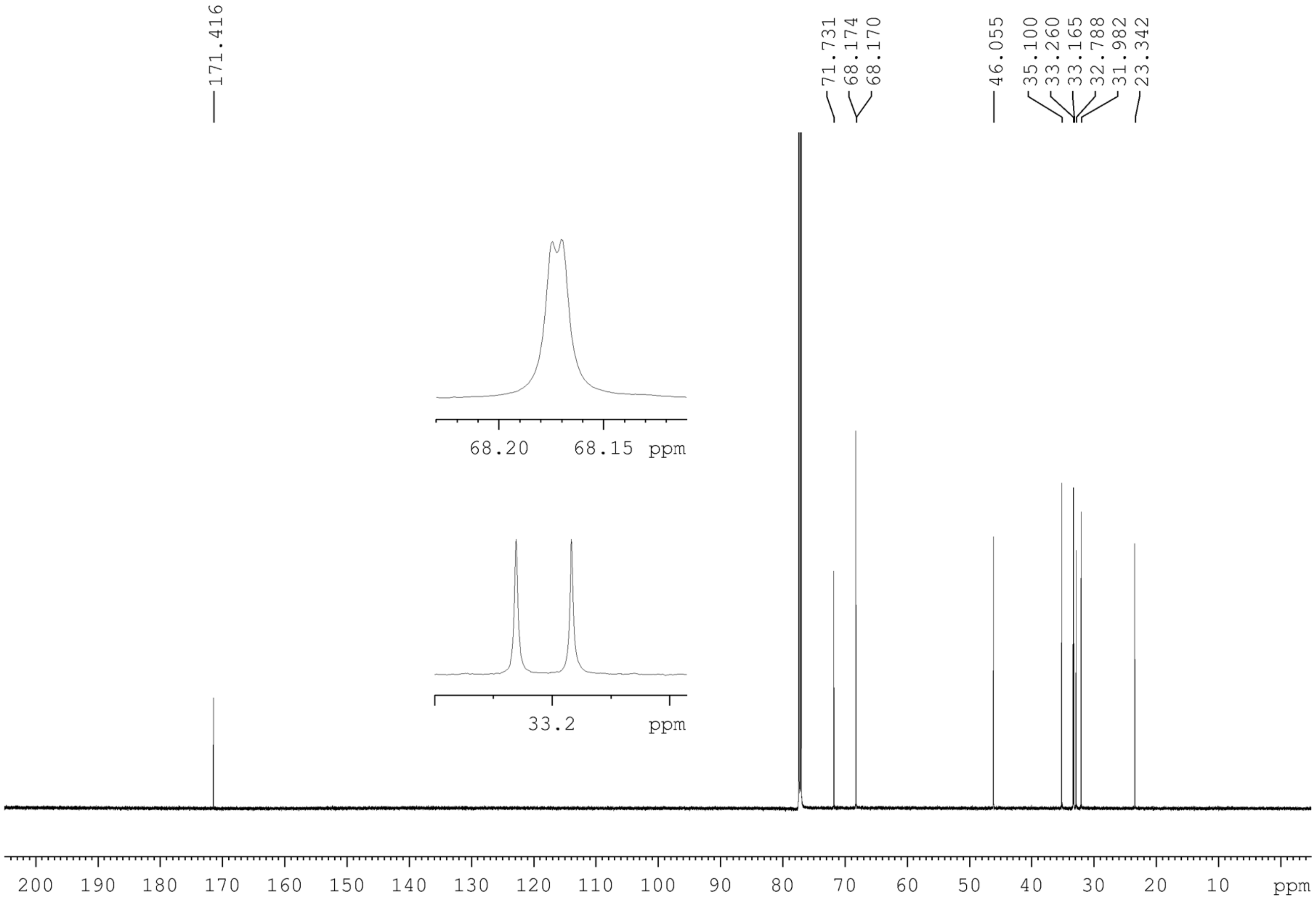

(*S*)-*N*-(2-hydroxy-4-(tetrahydro-2*H*-pyran-4-yl)butyl)acetamide (1p)

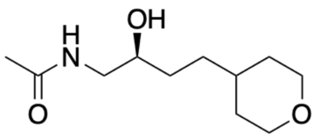

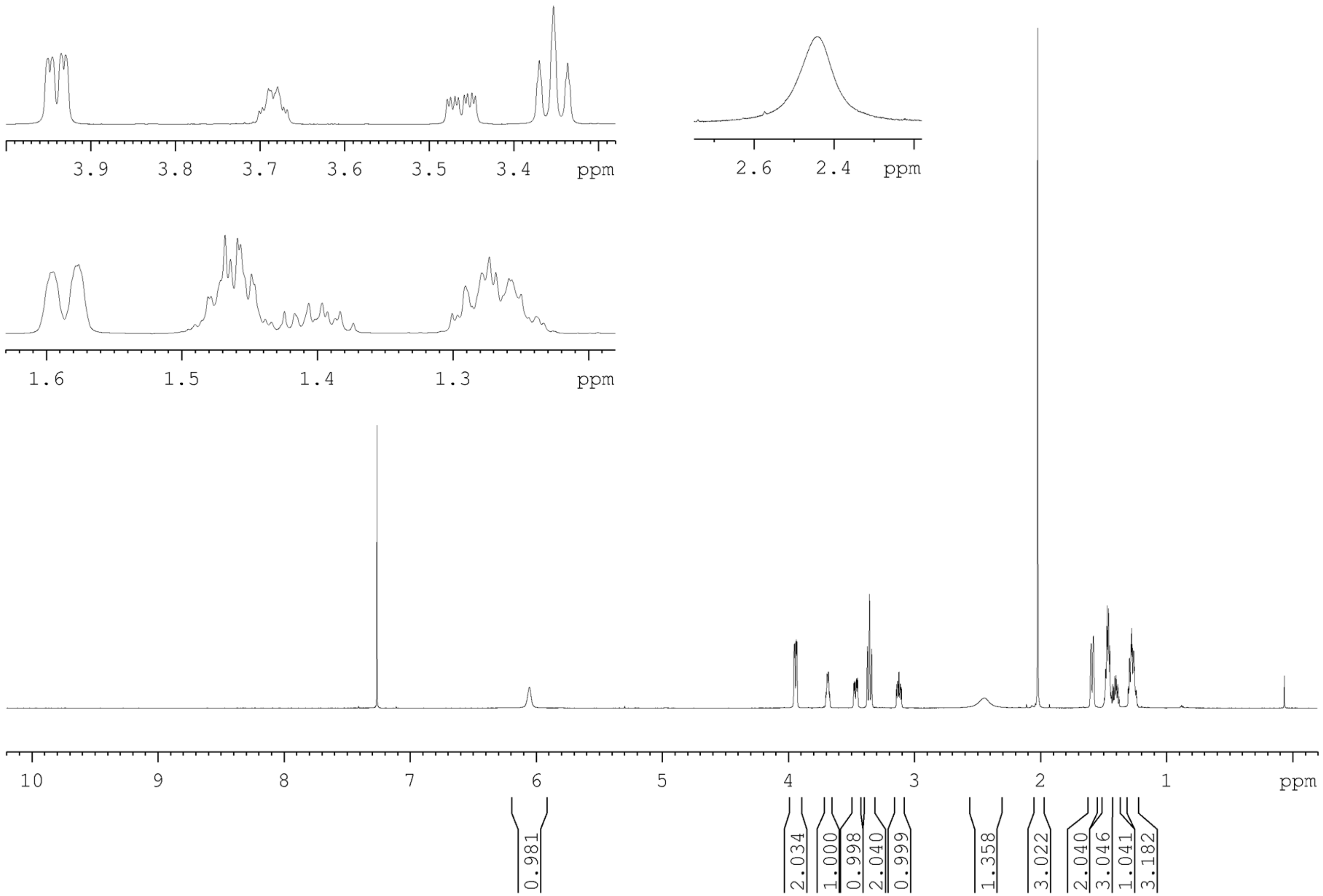

(*S*)-*N*-(2-hydroxy-4-(tetrahydro-2*H*-pyran-4-yl)butyl)acetamide (1p)

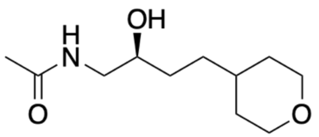

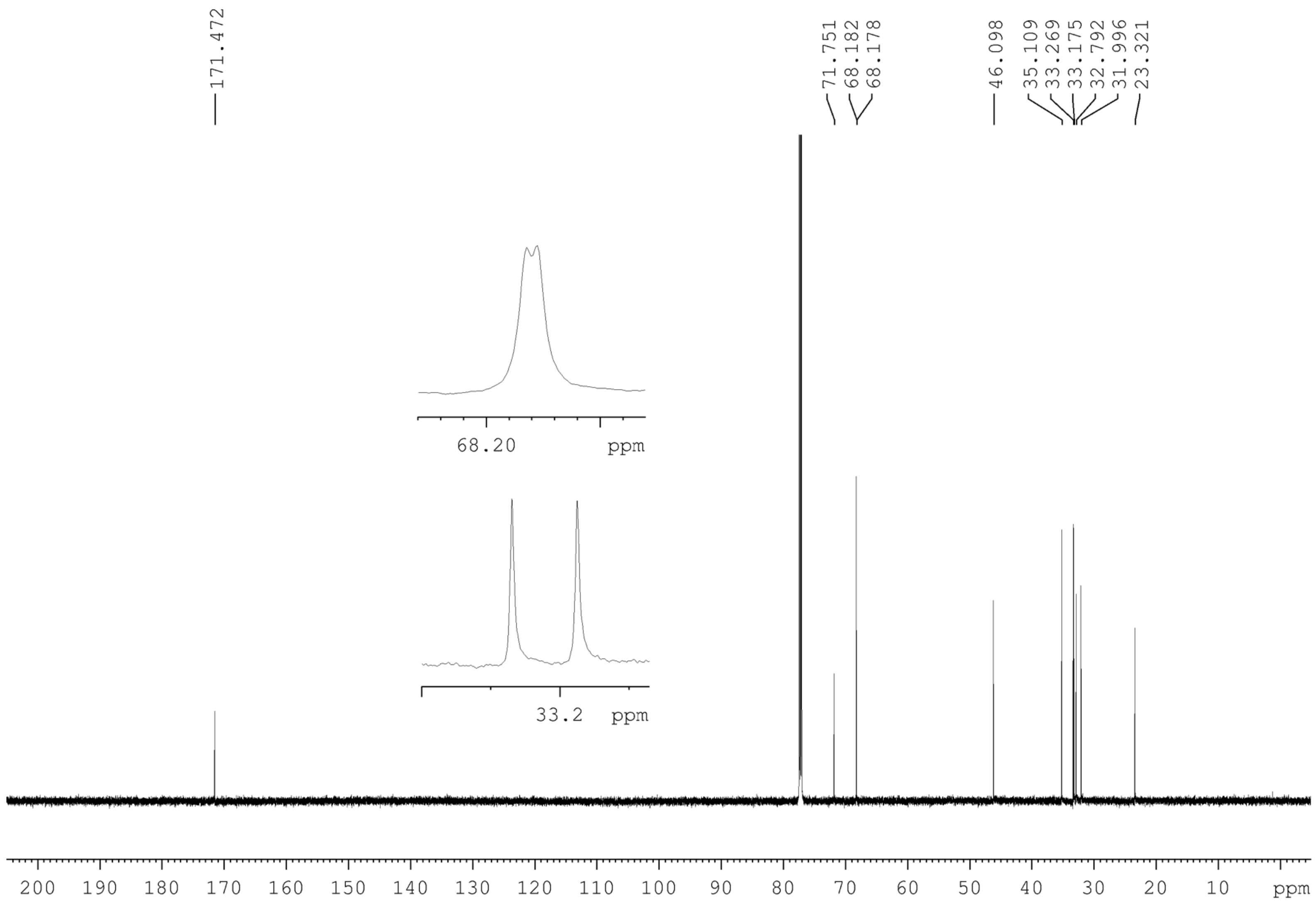

(*S*)-1-(*N*-acetylbenzamido)-4-(tetrahydro-2*H*-pyran-4-yl)butan-2-yl benzoate (1p-Bz)

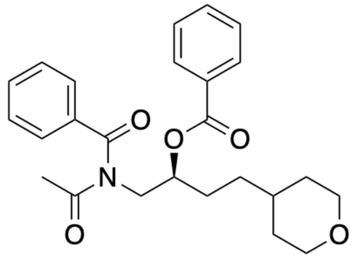

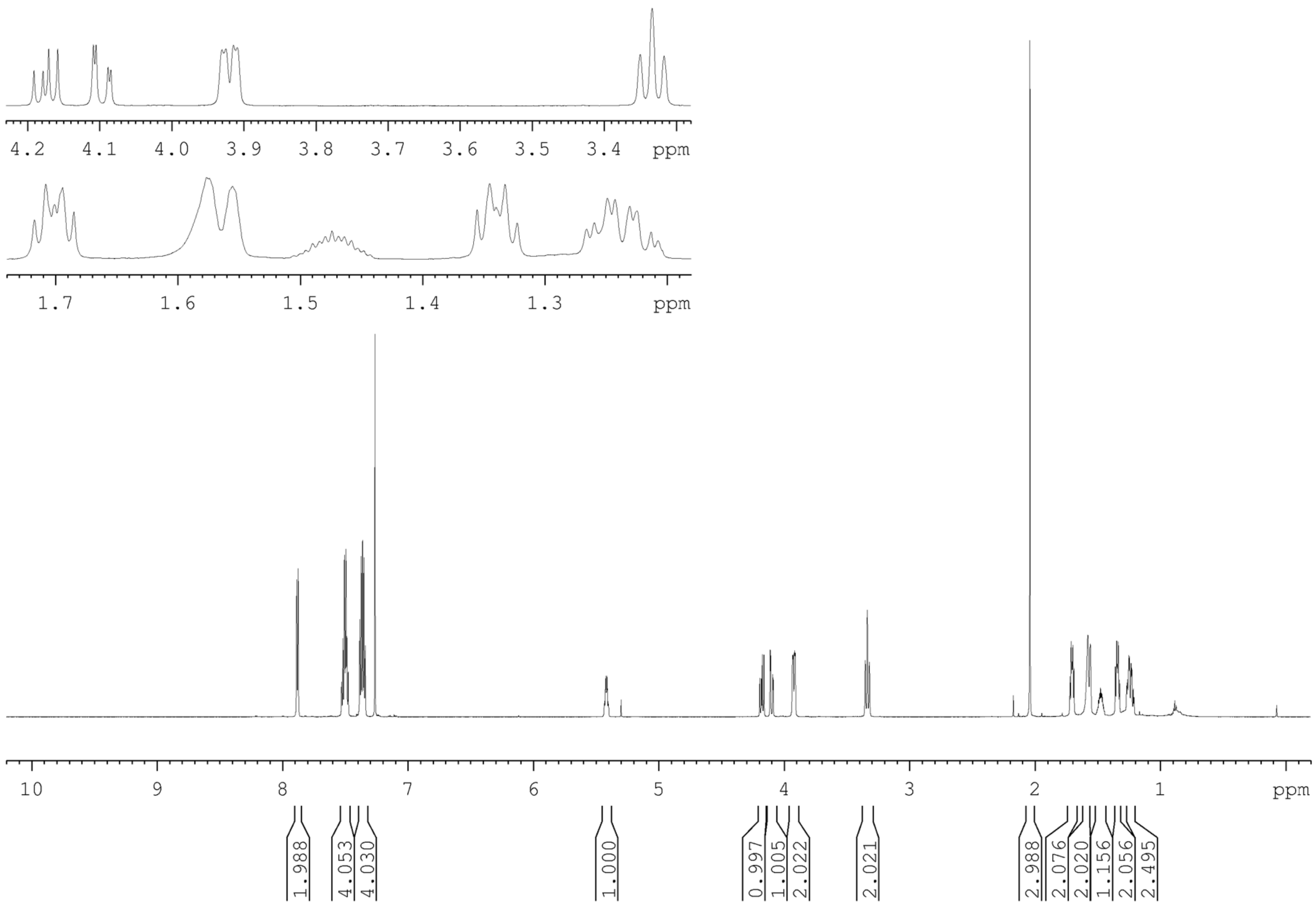

(*S*)-1-(*N*-acetylbenzamido)-4-(tetrahydro-2*H*-pyran-4-yl)butan-2-yl benzoate (1p-Bz)

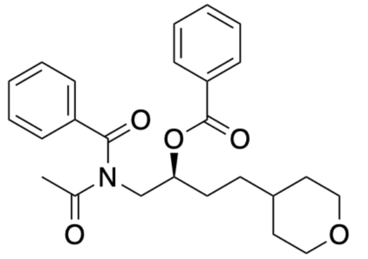

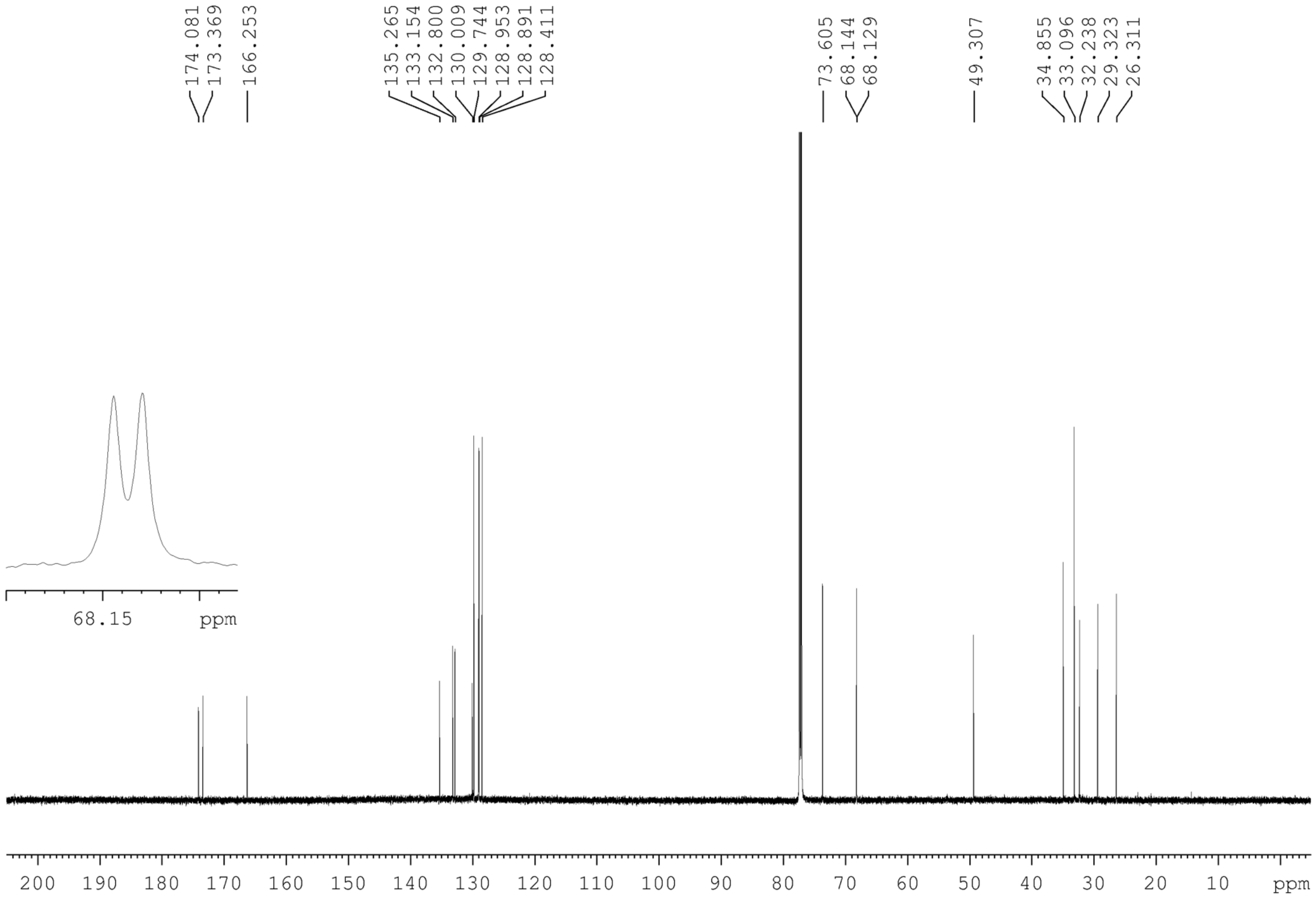

1-amino-4-phenoxybutan-2-ol (1q-I_2)

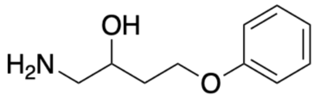

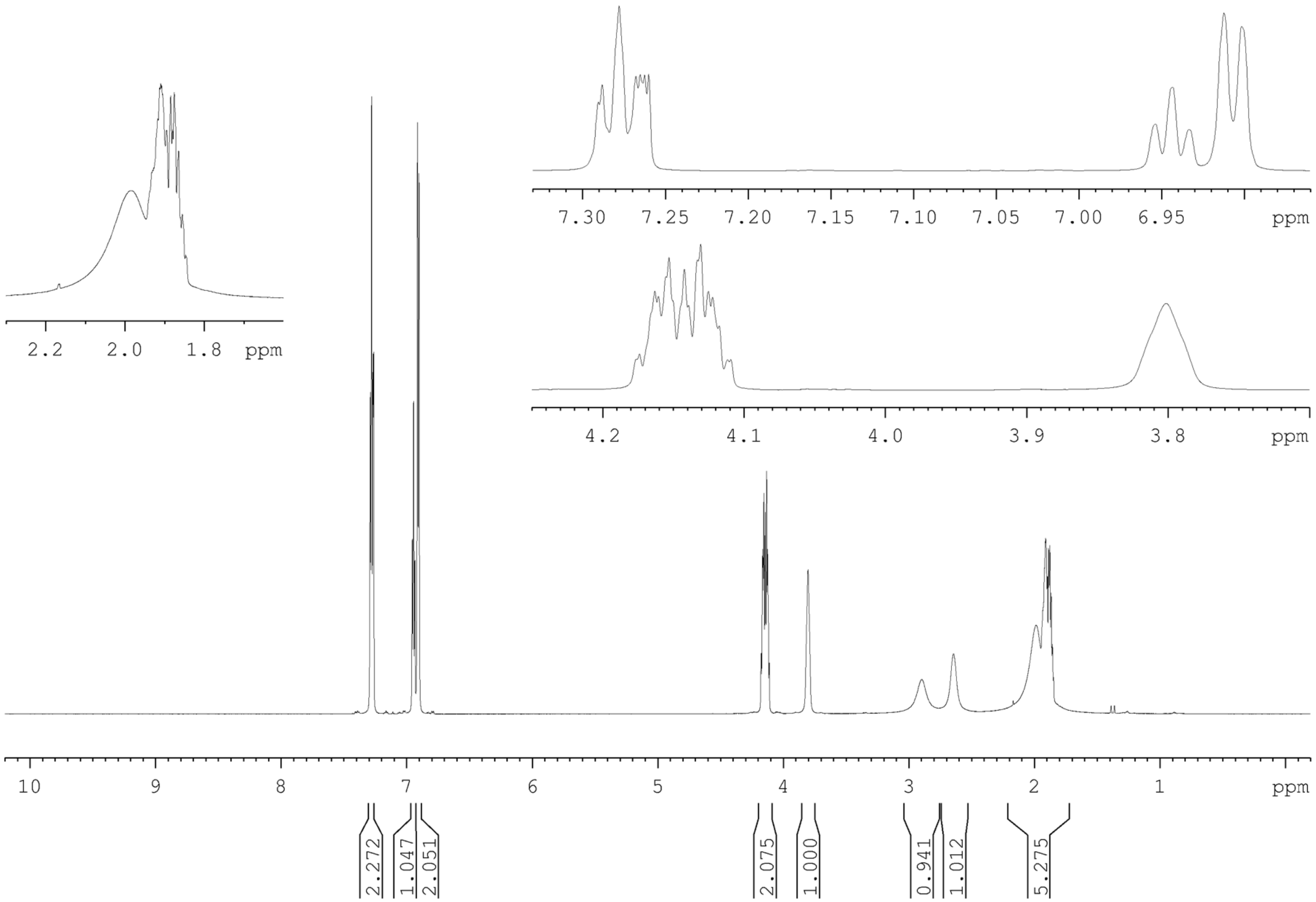

1-amino-4-phenoxybutan-2-ol (1q-I_2)

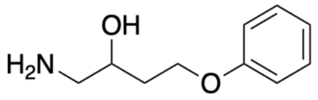

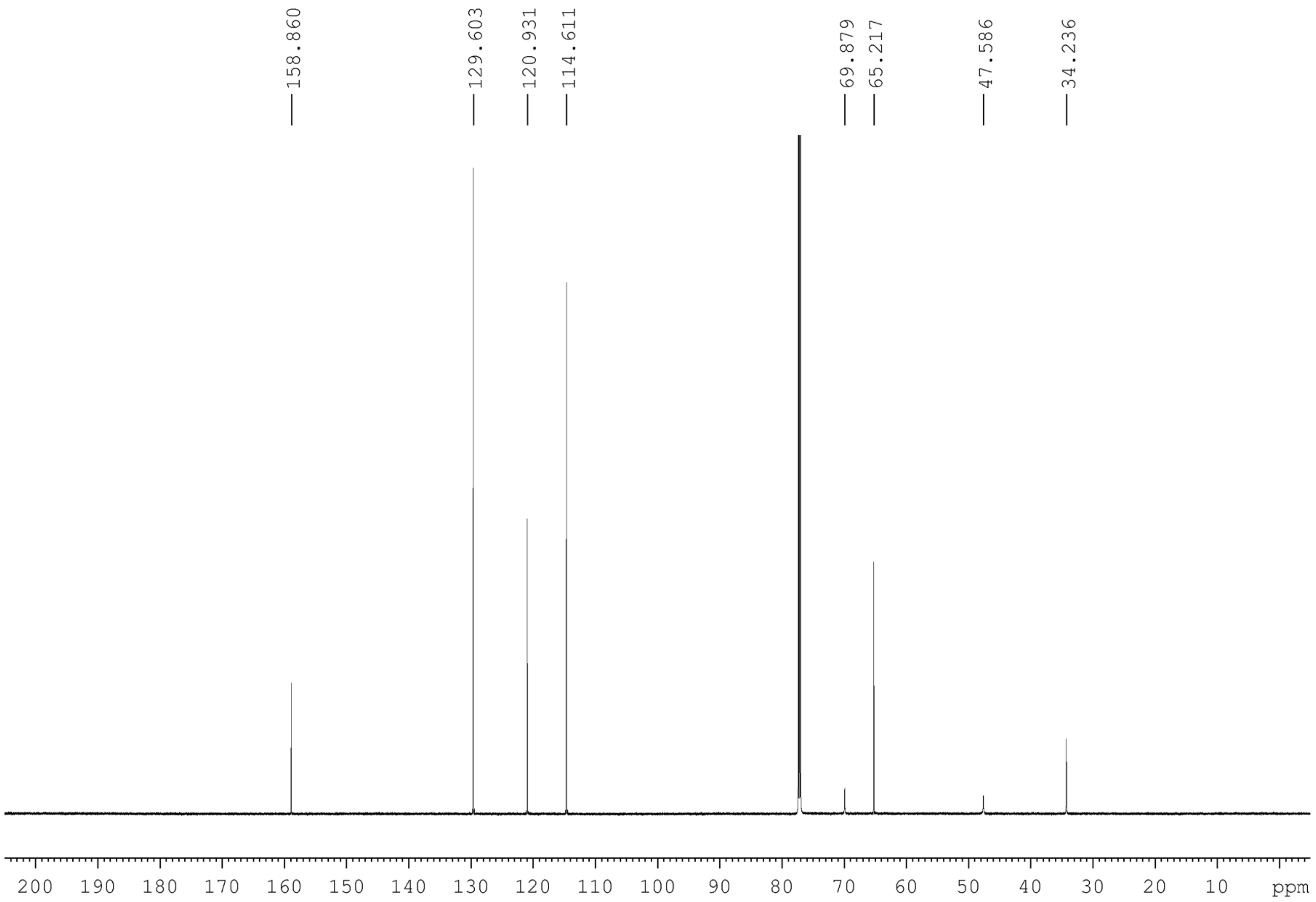

N-(2-hydroxy-4-phenoxybutyl)acetamide (1q-rac)

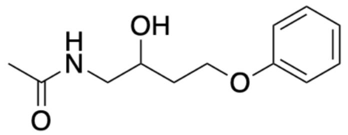

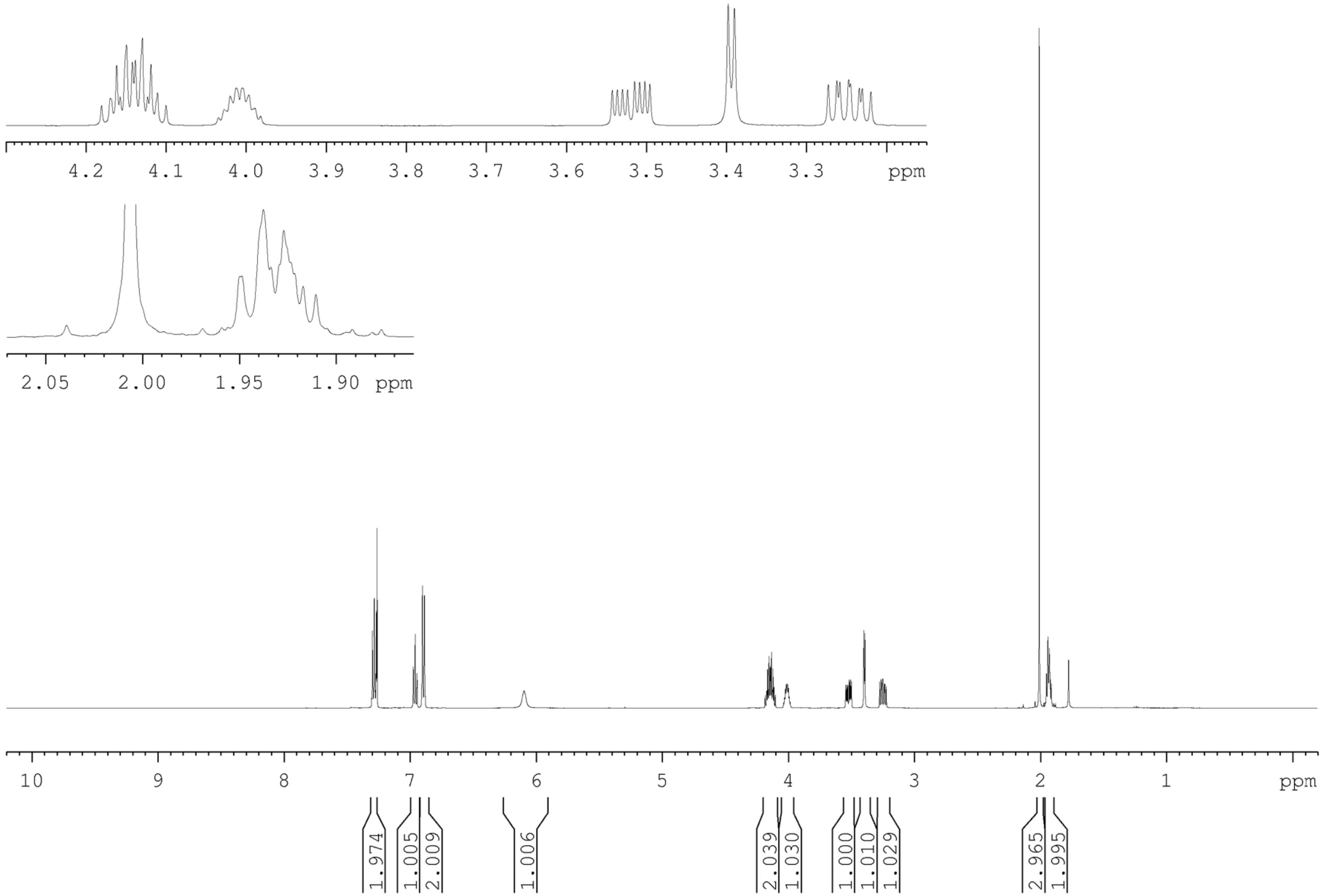

N-(2-hydroxy-4-phenoxybutyl)acetamide (1q-rac)

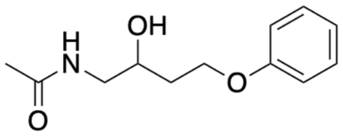

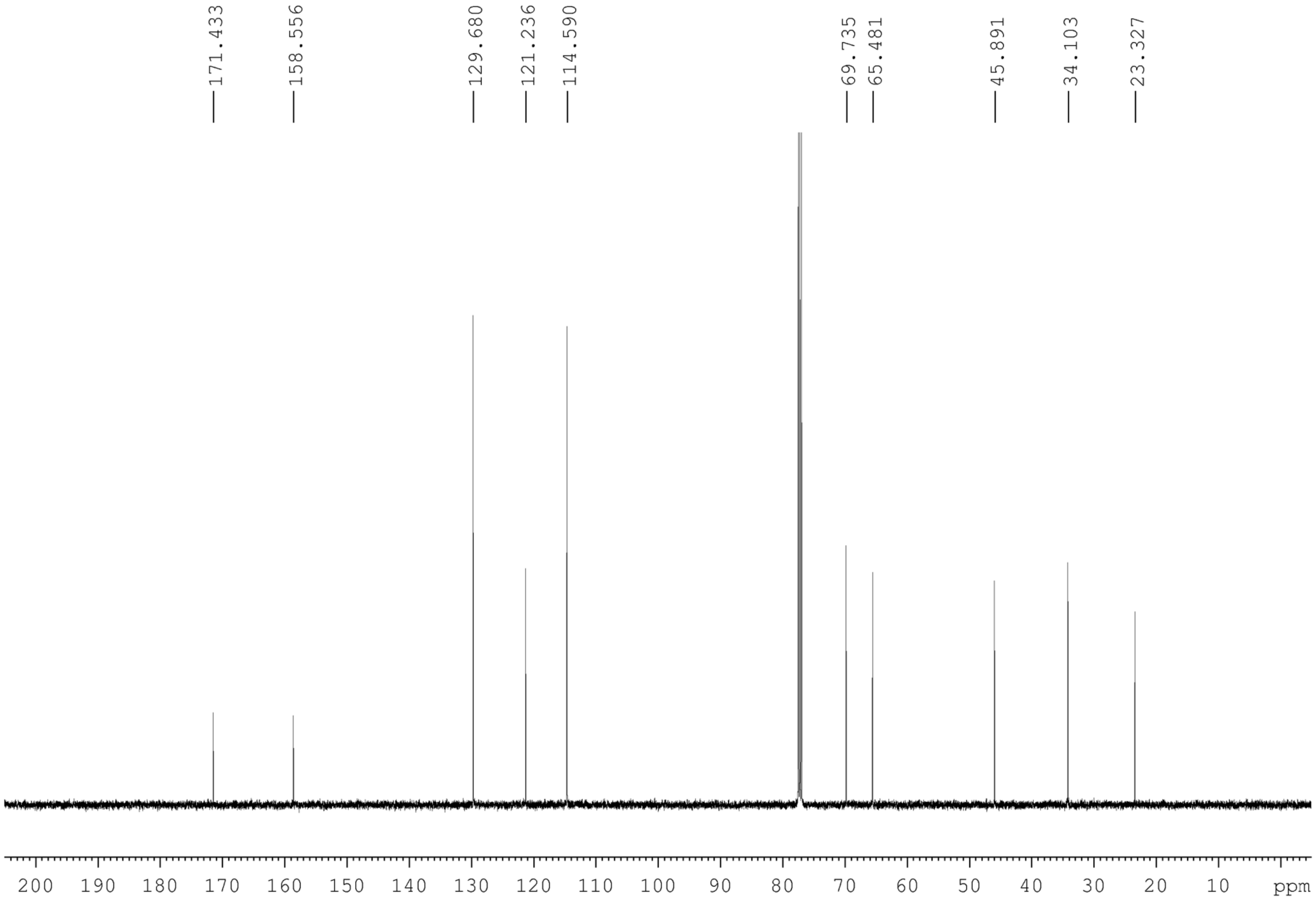

(*S*)-*N*-(2-hydroxy-4-phenoxybutyl)acetamide (1q)

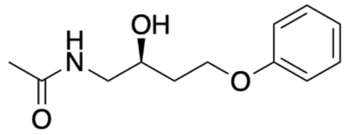

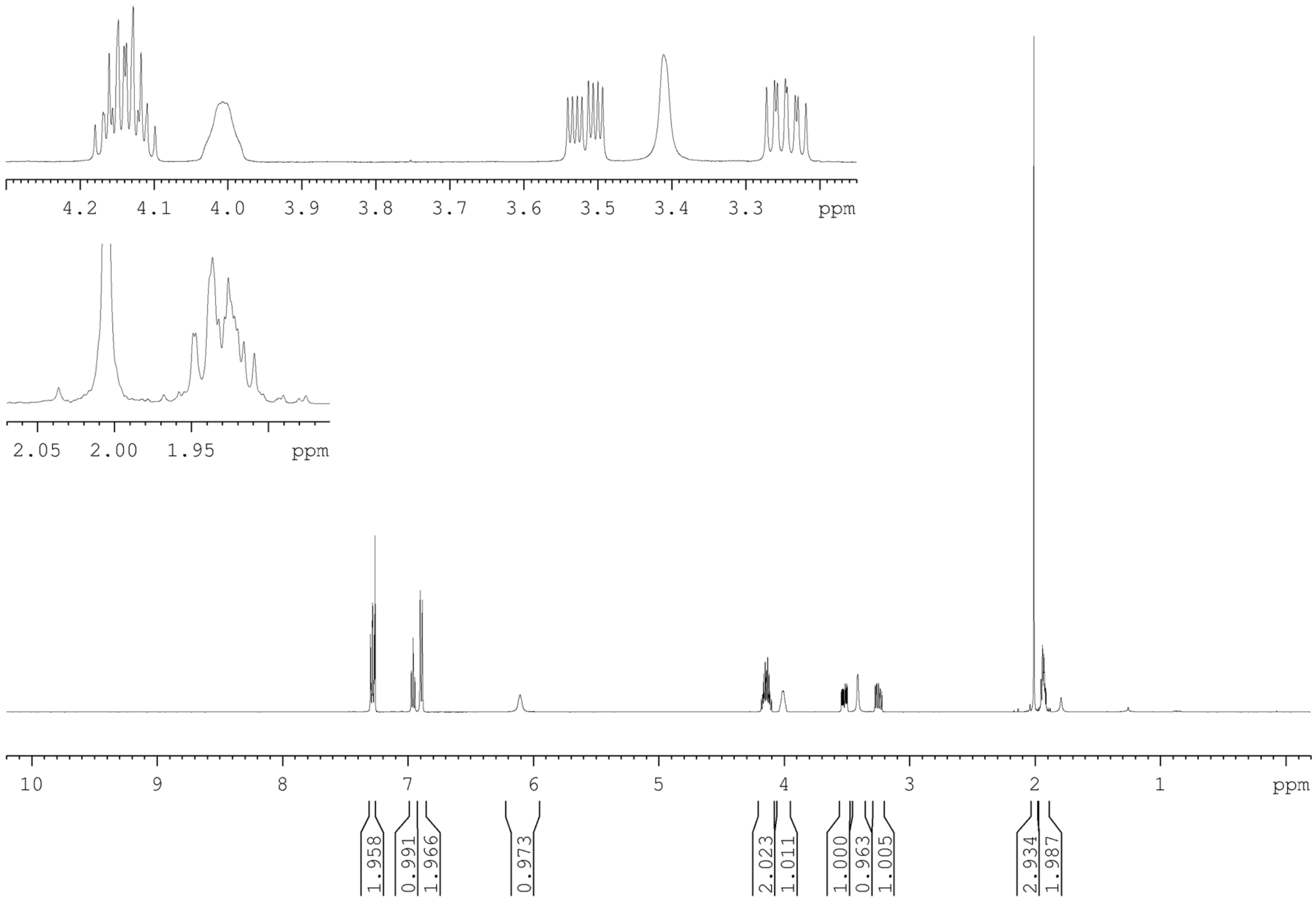

(*S*)-*N*-(2-hydroxy-4-phenoxybutyl)acetamide (1q)

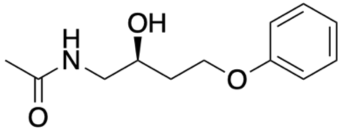

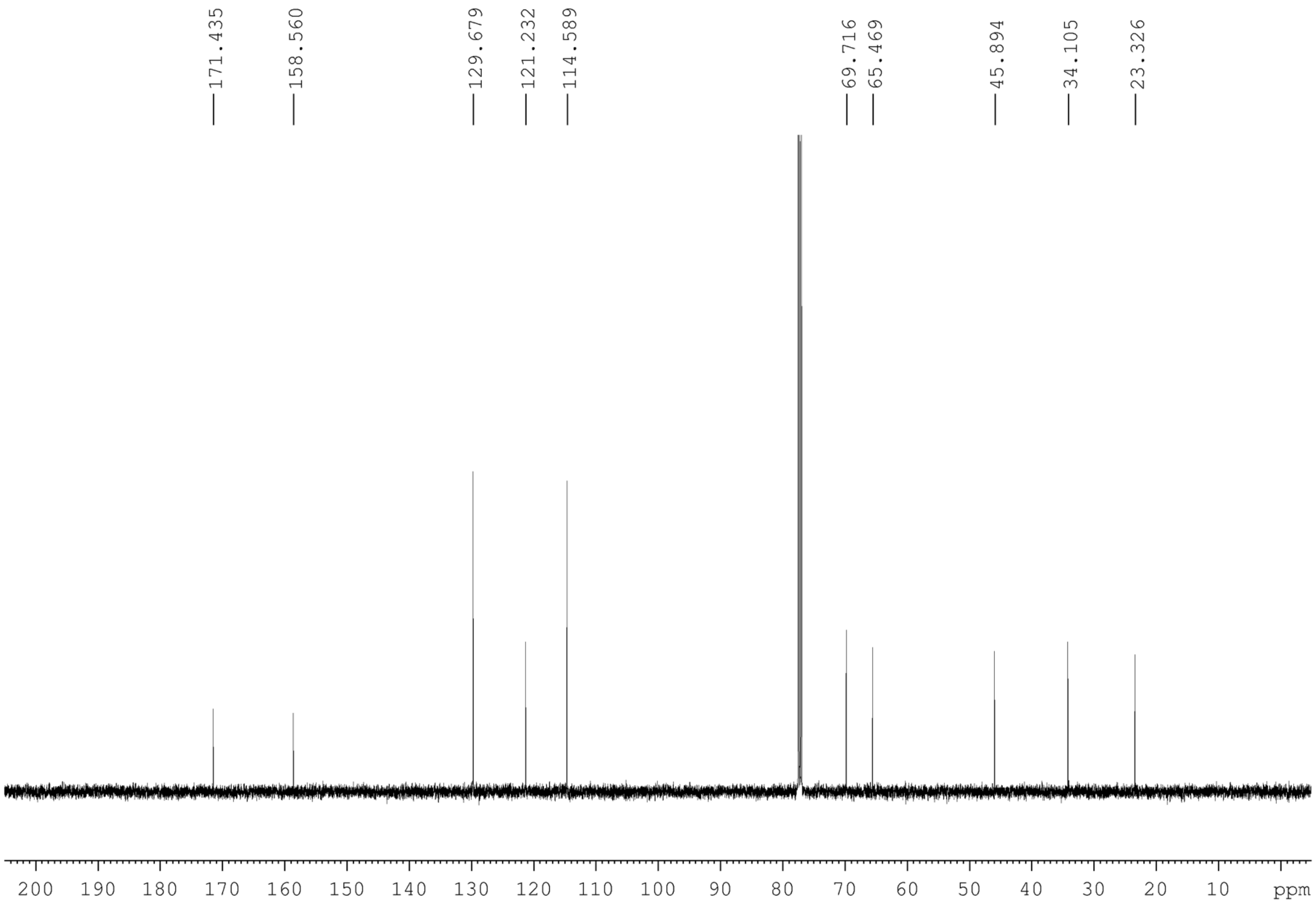

1-amino-3-(4-methoxyphenyl)propan-2-ol (1r-I_1)

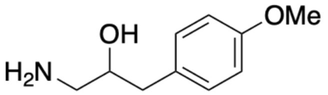

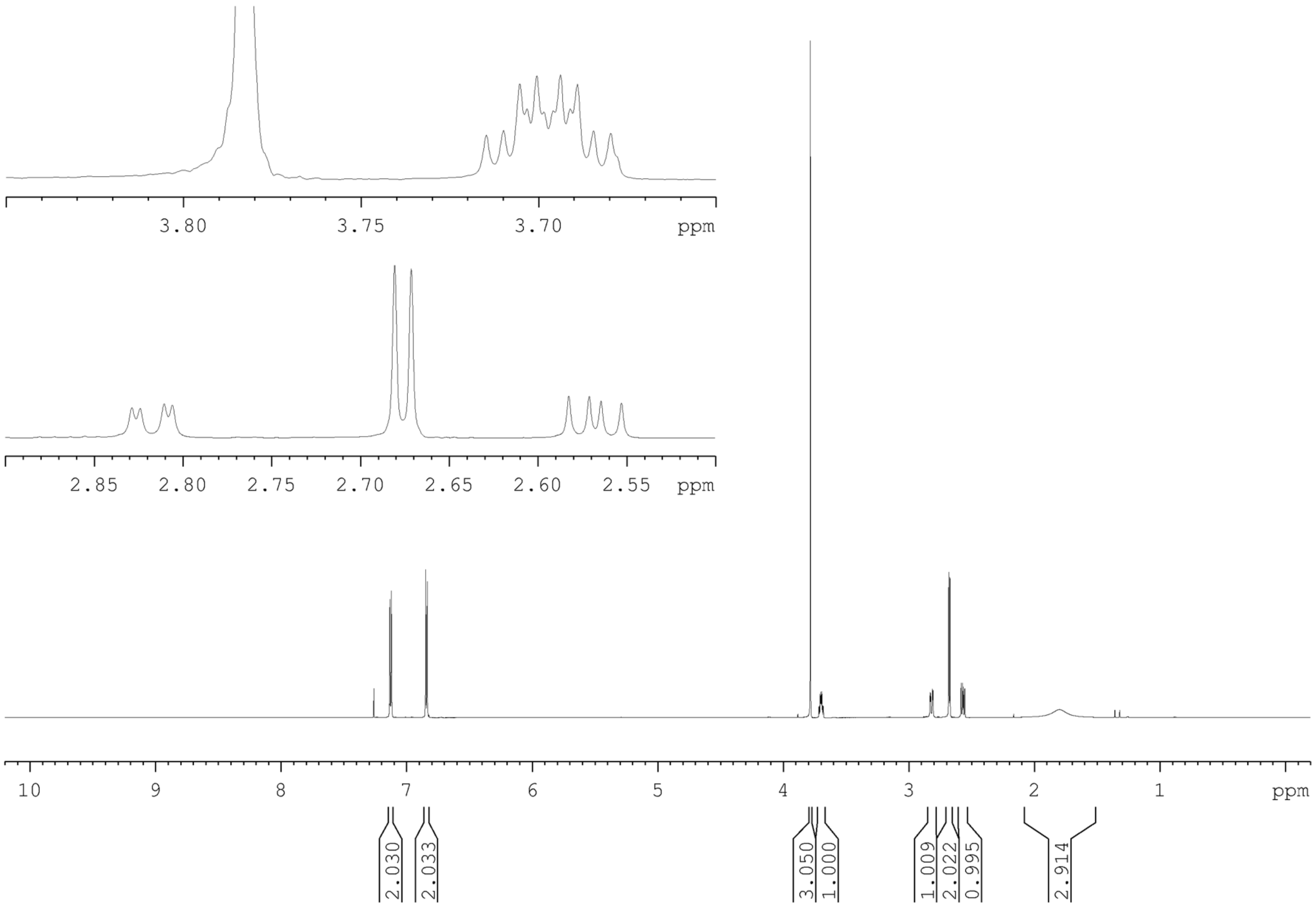

1-amino-3-(4-methoxyphenyl)propan-2-ol (1r-I_1)

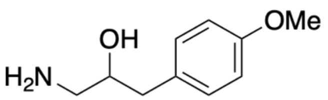

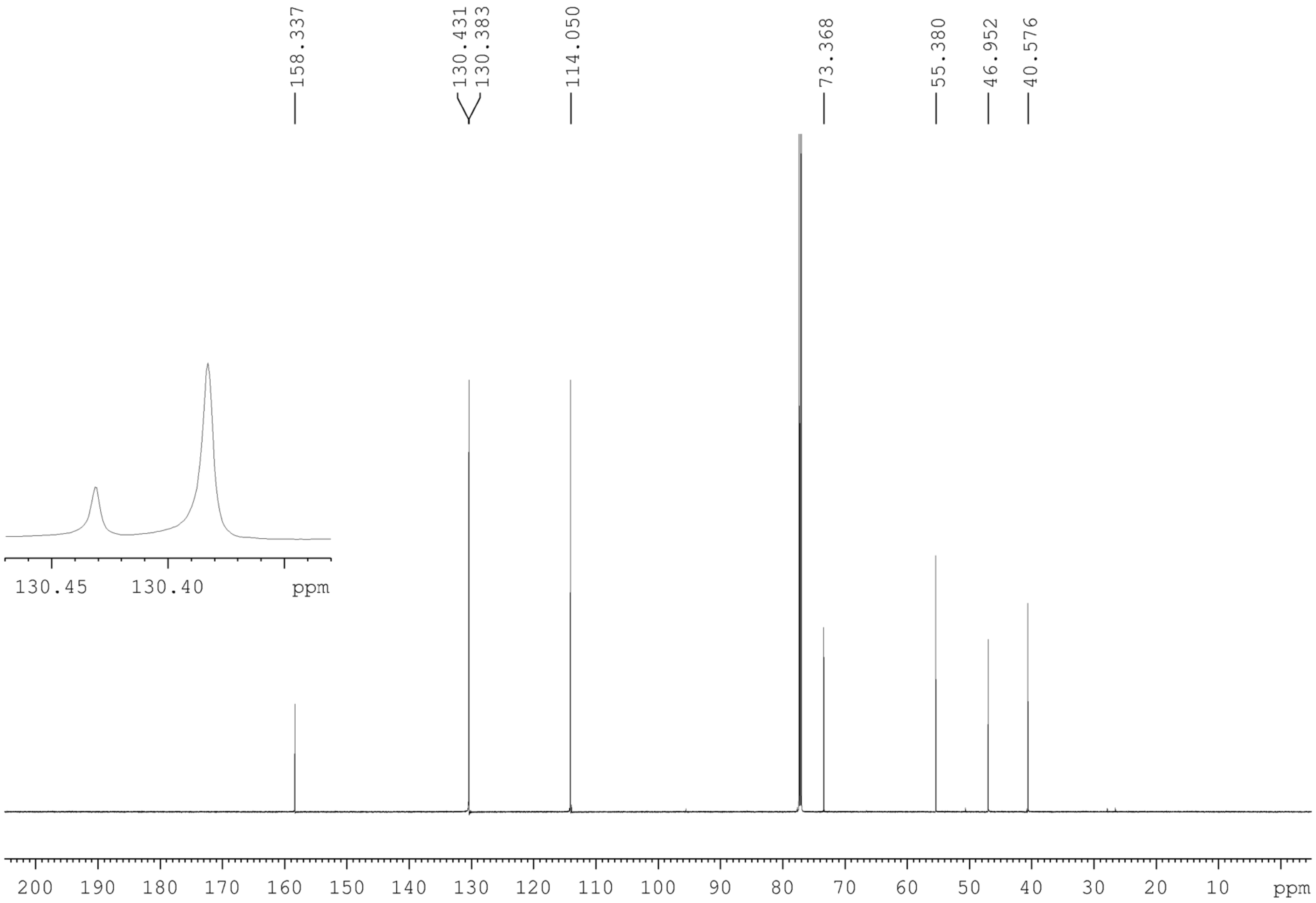

***N*-(2-hydroxy-3-(4-methoxyphenyl)propyl)acetamide (1r-rac)**

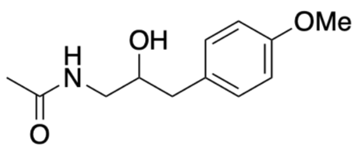

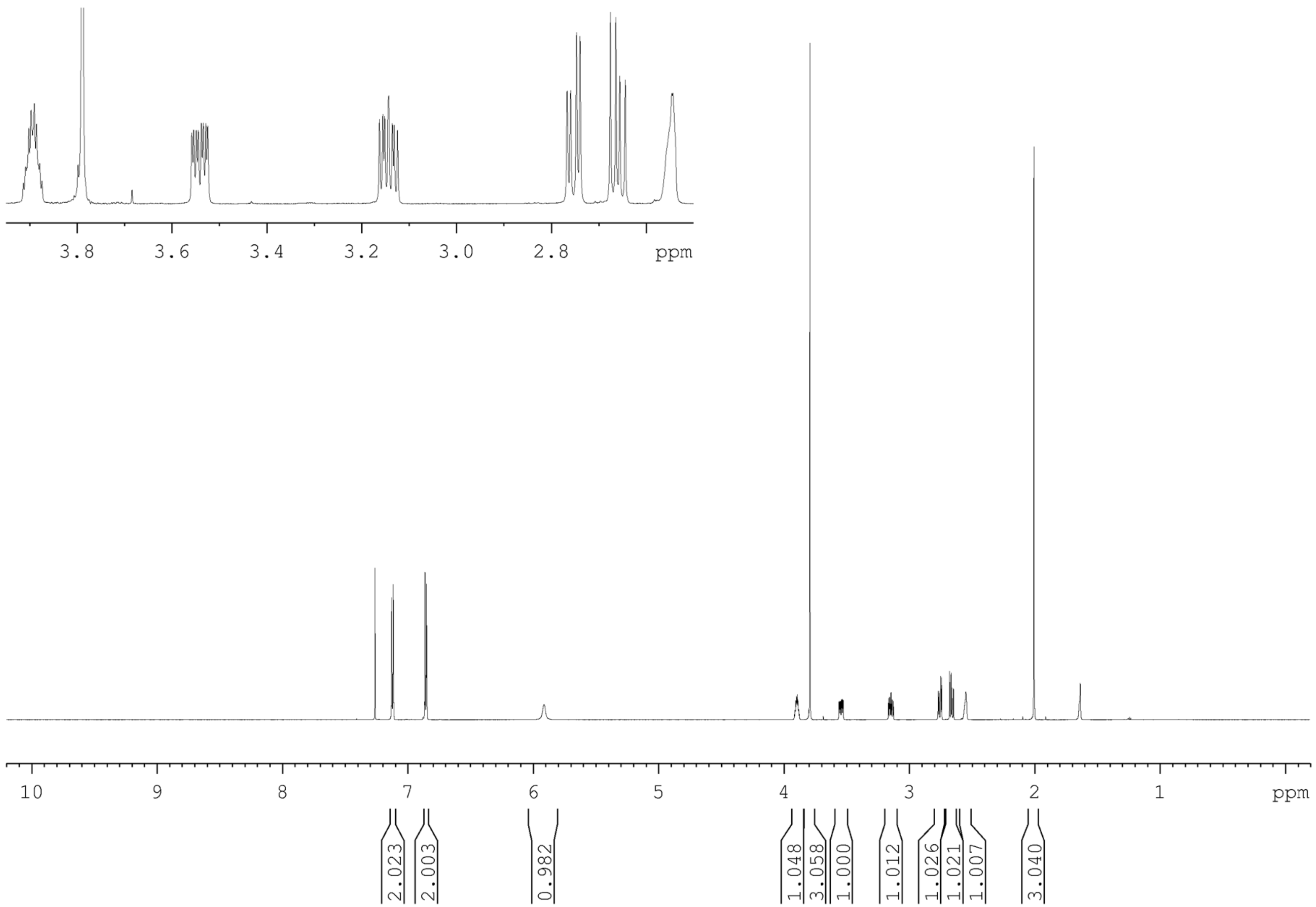

N-(2-hydroxy-3-(4-methoxyphenyl)propyl)acetamide (1r-rac)

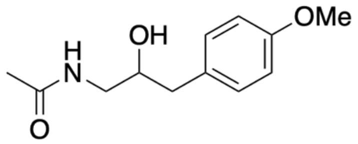

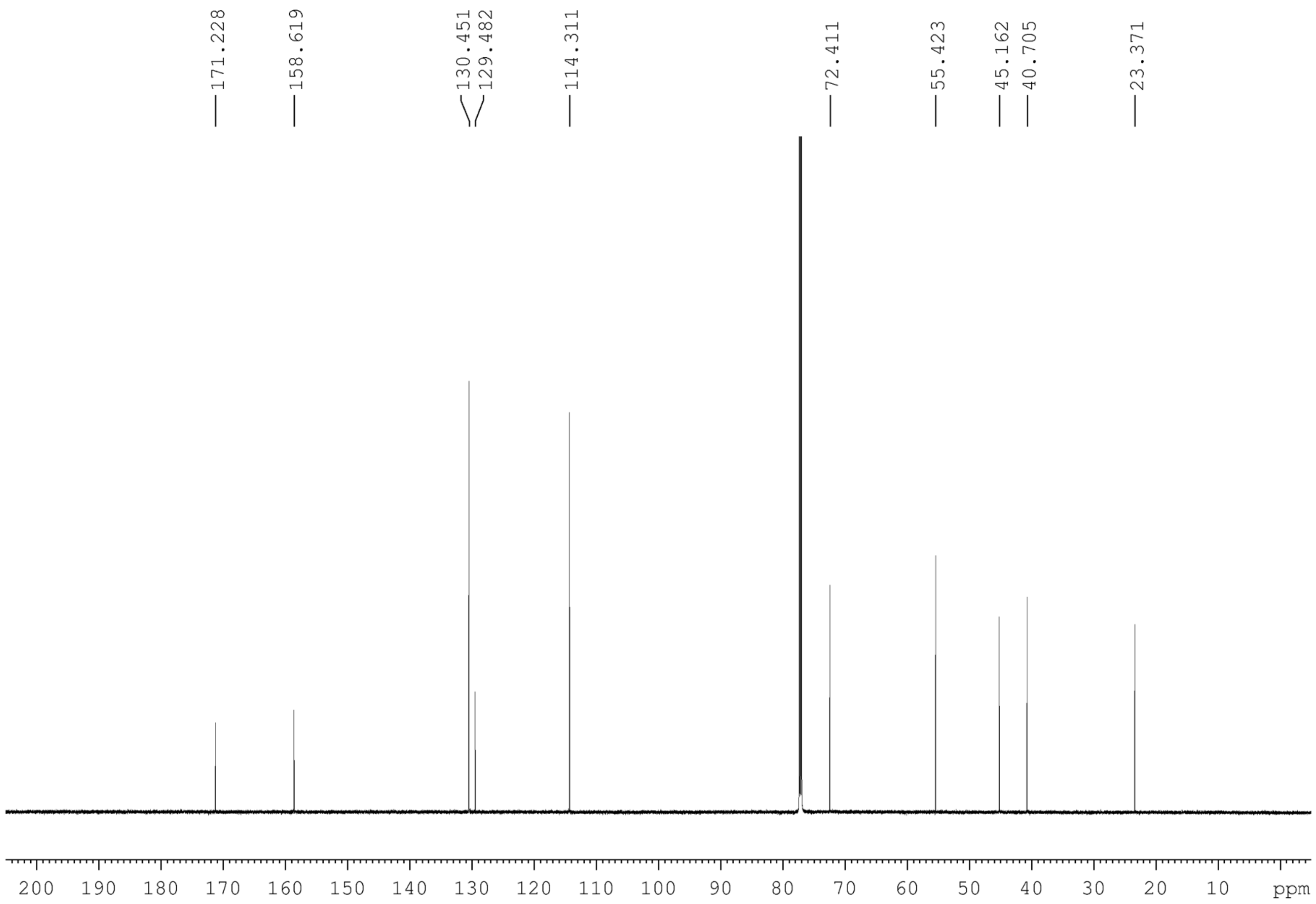

(*S*)-*N*-(2-hydroxy-3-(4-methoxyphenyl)propyl)acetamide (1r)

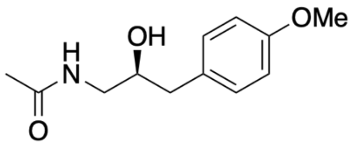

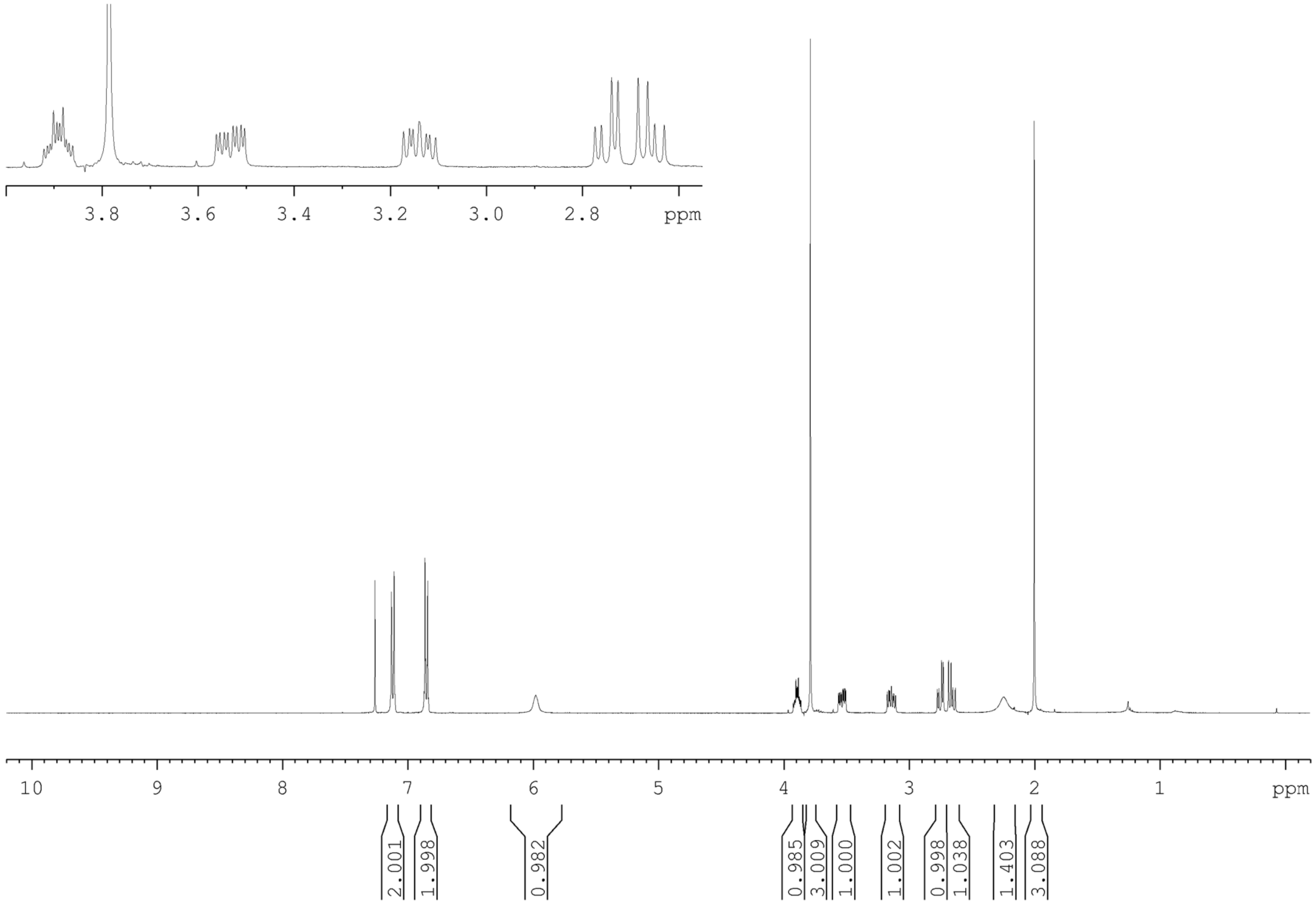

(*S*)-*N*-(2-hydroxy-3-(4-methoxyphenyl)propyl)acetamide (1r)

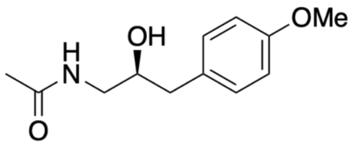

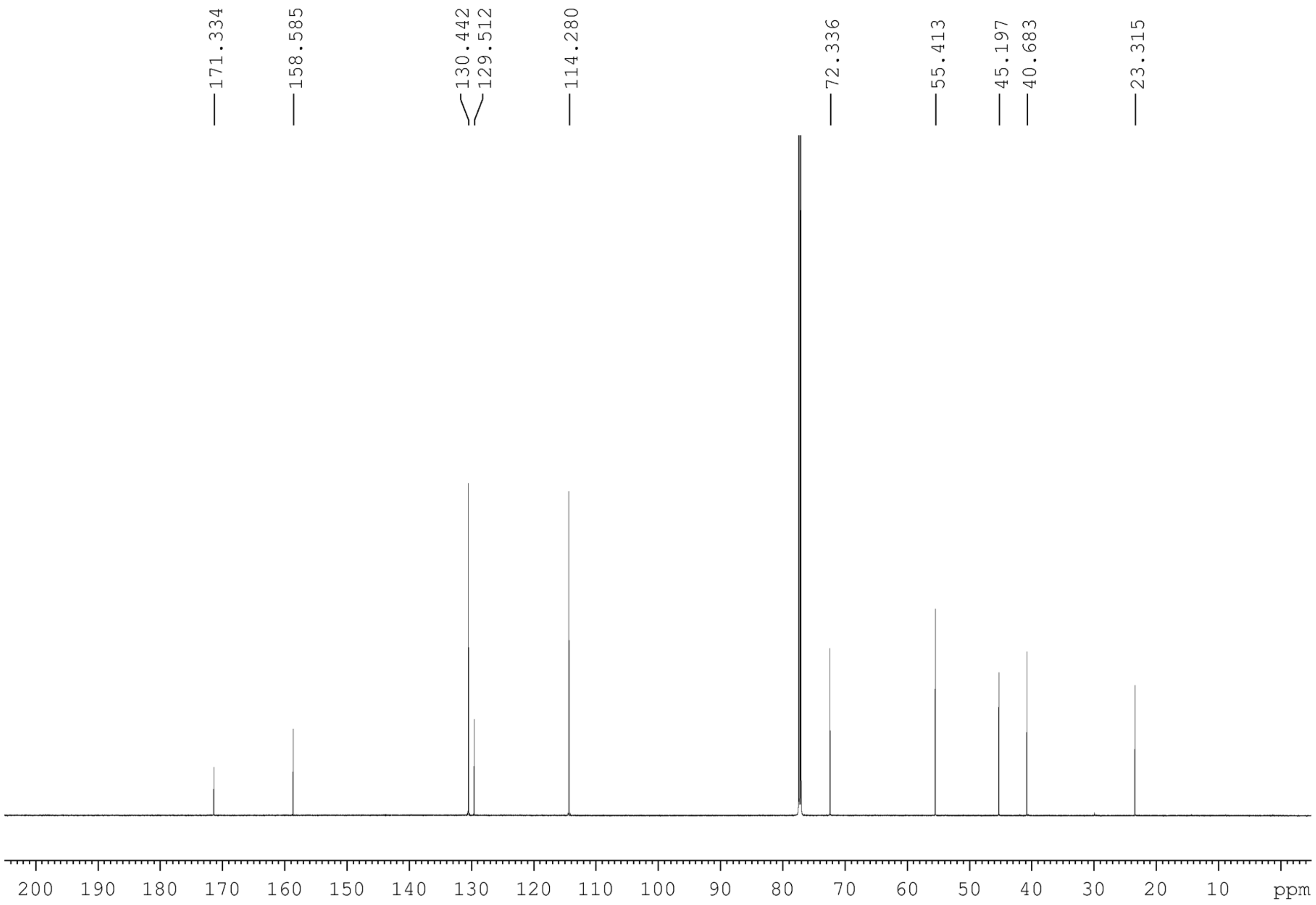

1-amino-3-(*p*-tolyl)propan-2-ol (1s-I_2)

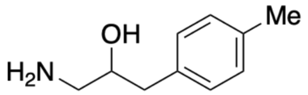

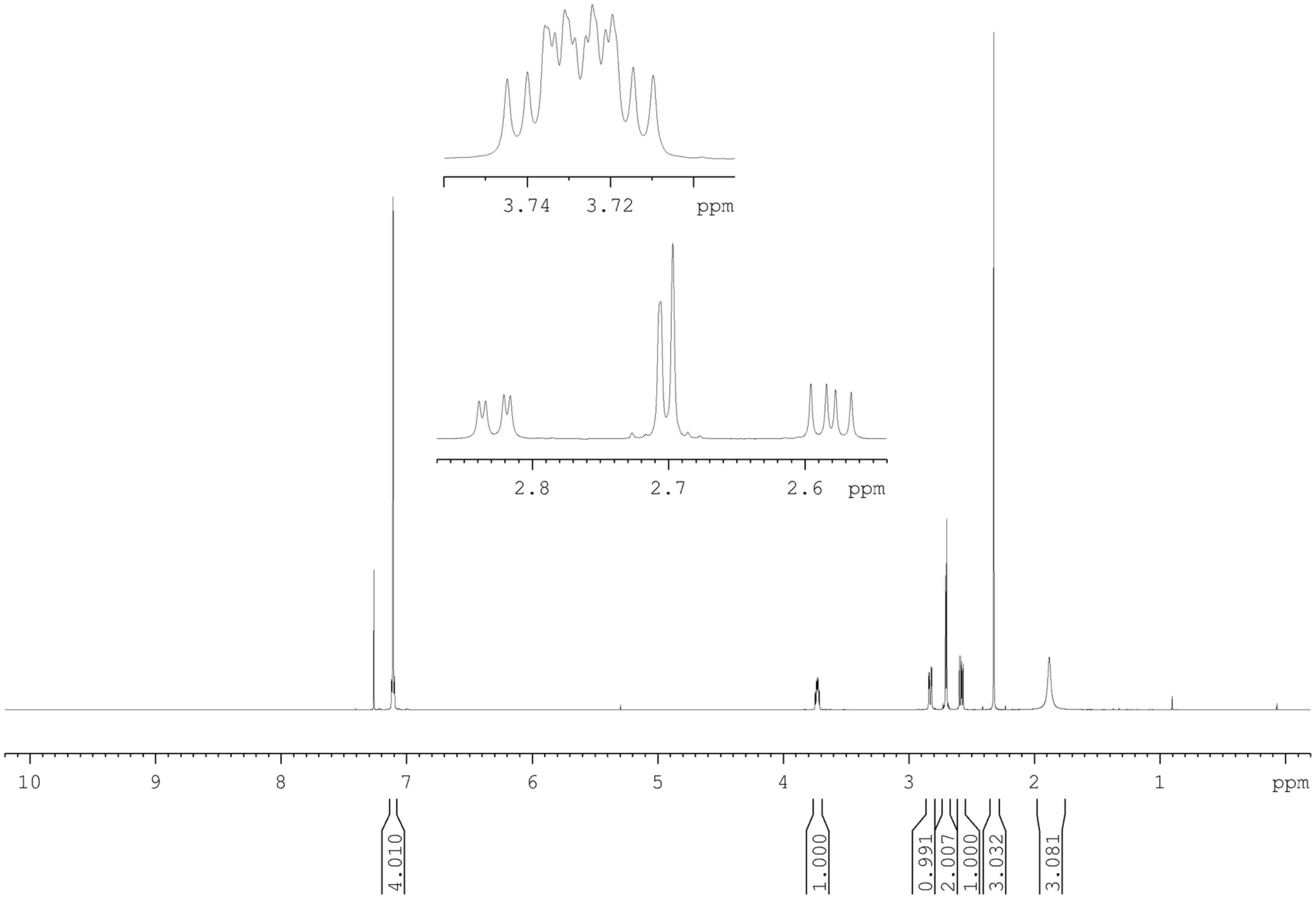

1-amino-3-(*p*-tolyl)propan-2-ol (1s-I_2)

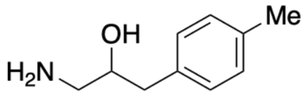

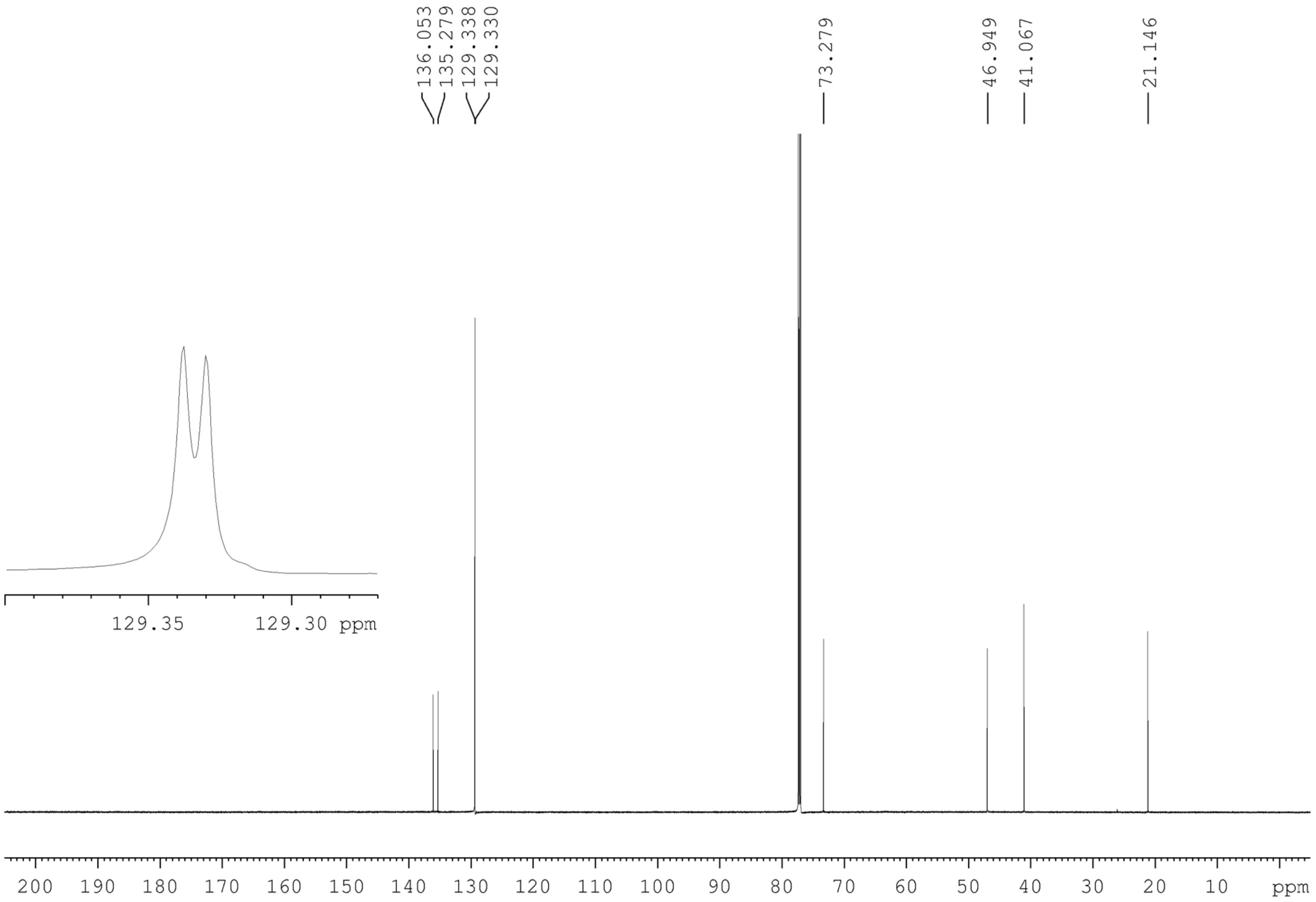

N-(2-hydroxy-3-(*p*-tolyl)propyl)acetamide (1s-rac)

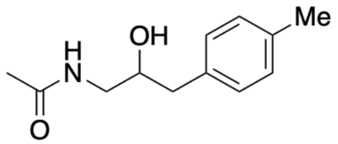

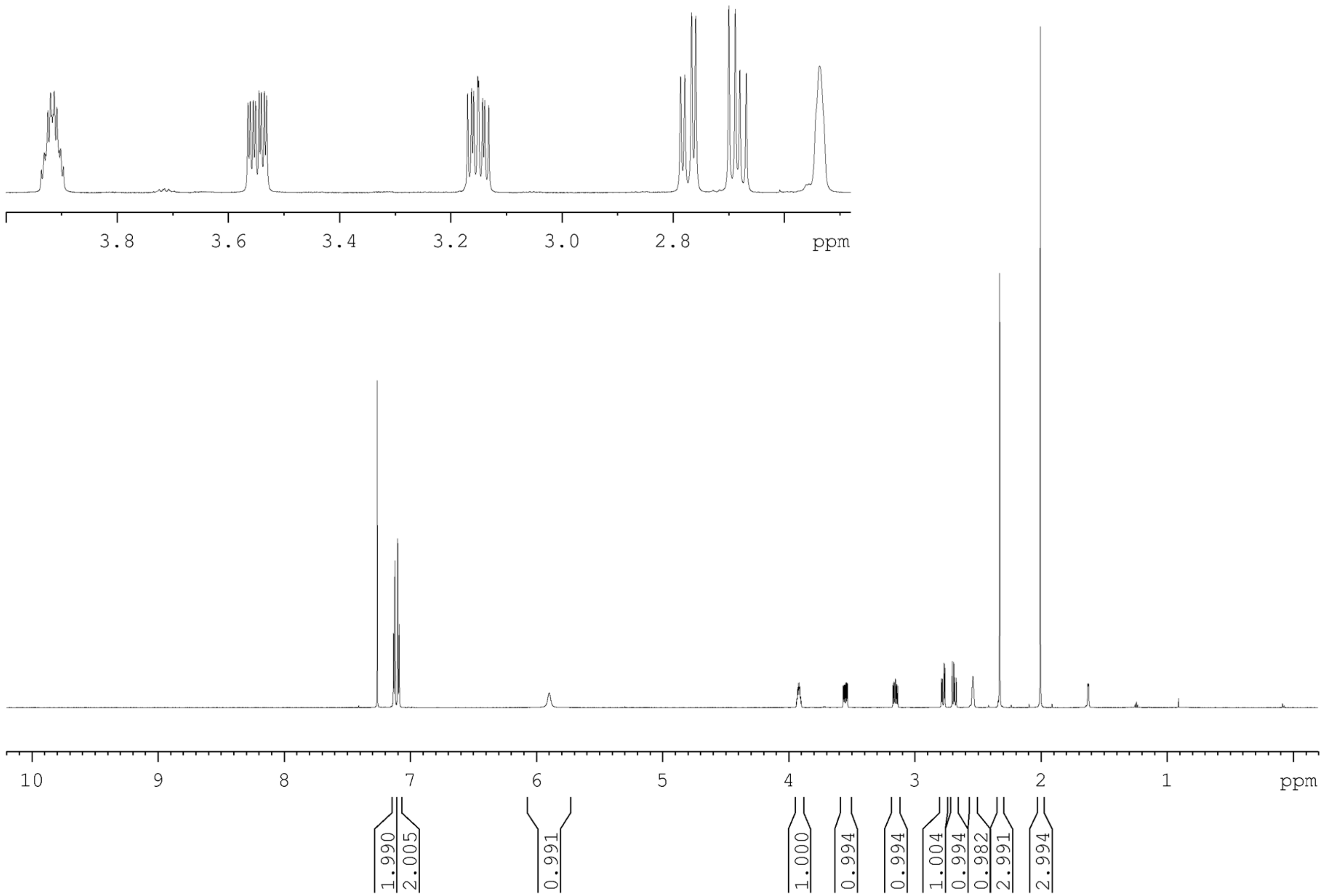

_N_-(2-hydroxy-3-(_p_-tolyl)propyl)acetamide (1s-rac)

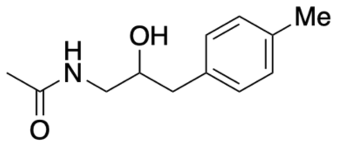

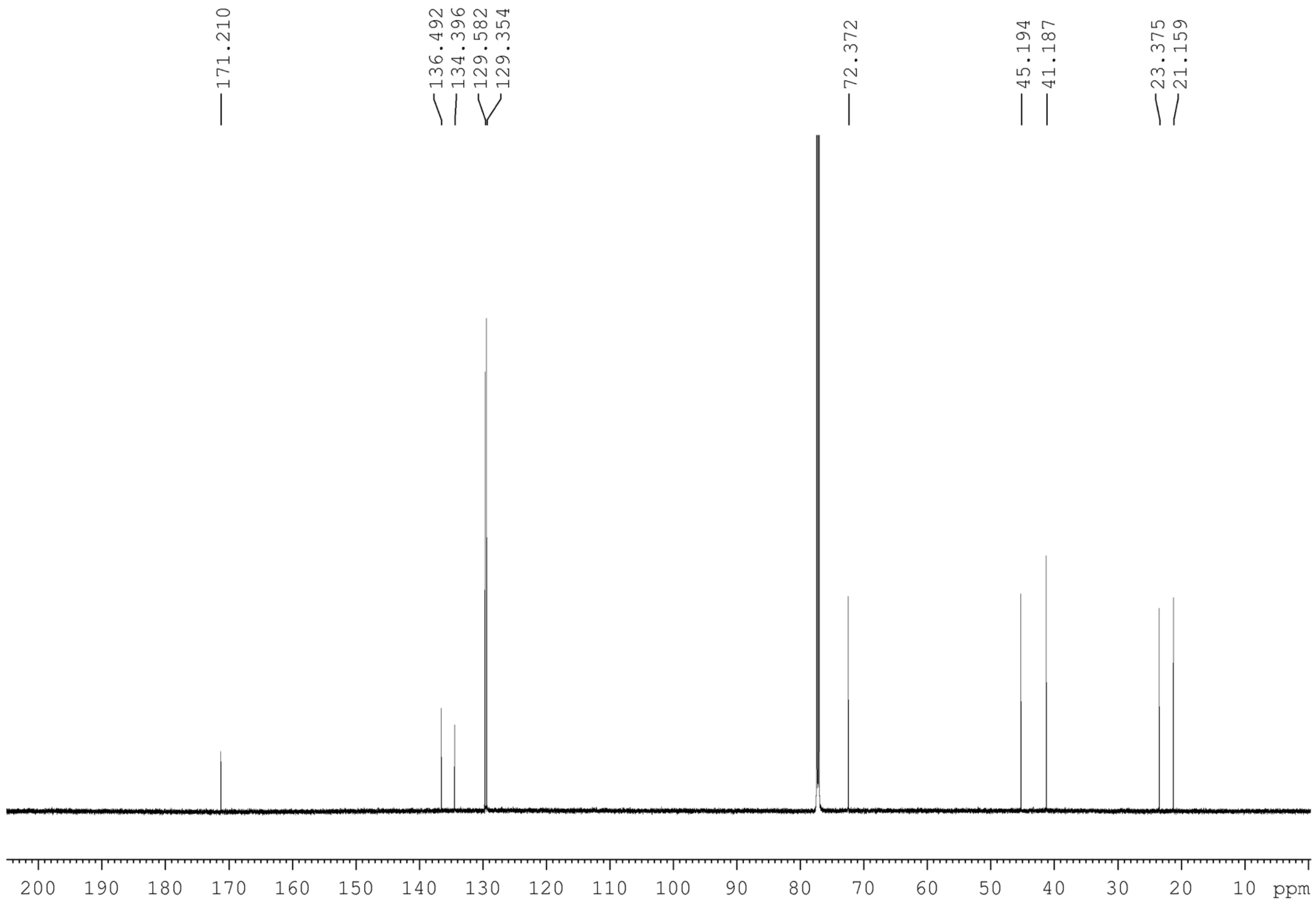

(*S*)-*N*-(2-hydroxy-3-(*p*-tolyl)propyl)acetamide (1s)

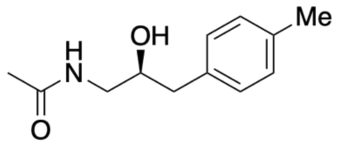

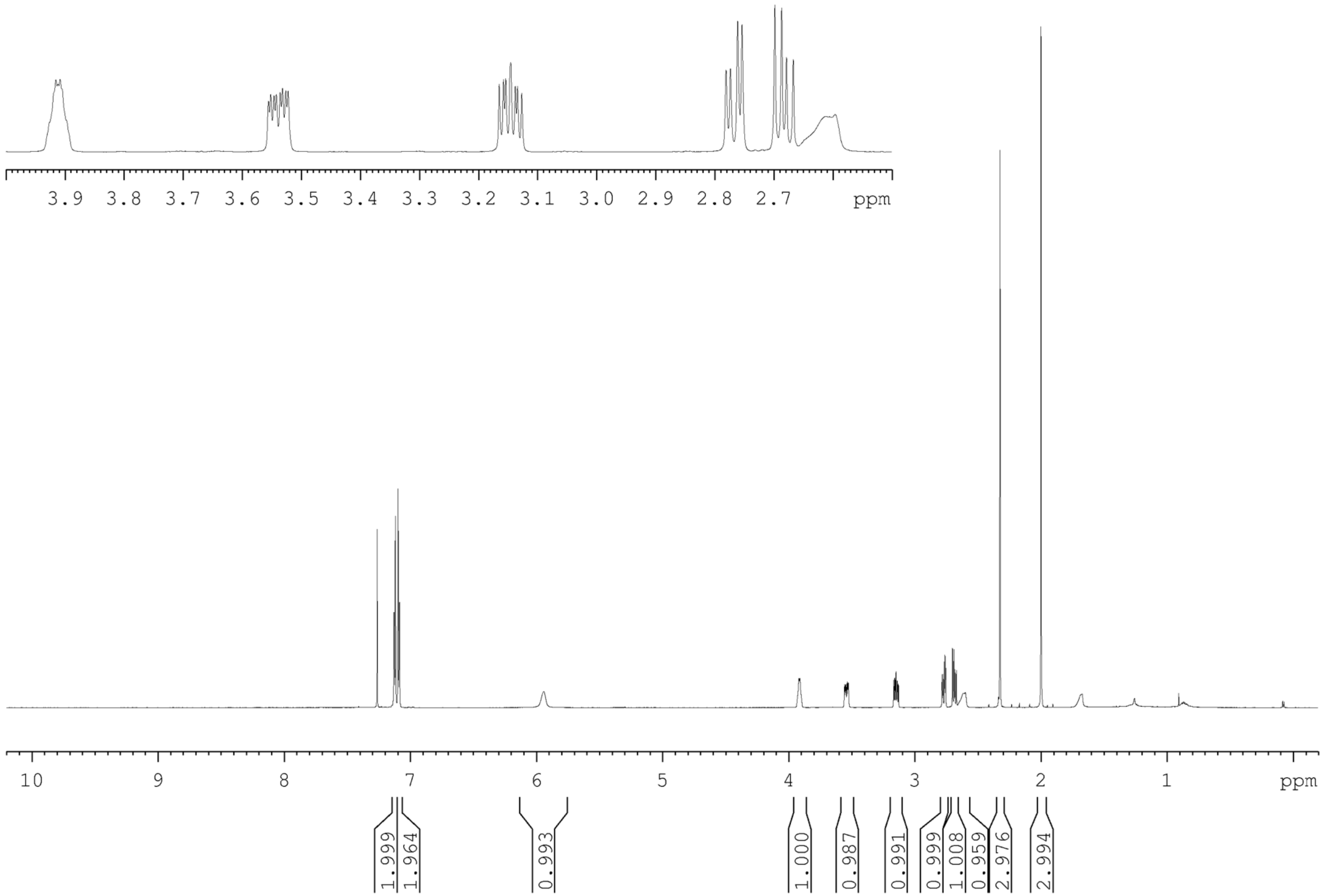

(*S*)-*N*-(2-hydroxy-3-(*p*-tolyl)propyl)acetamide (1s)

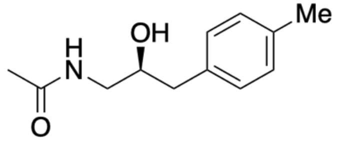

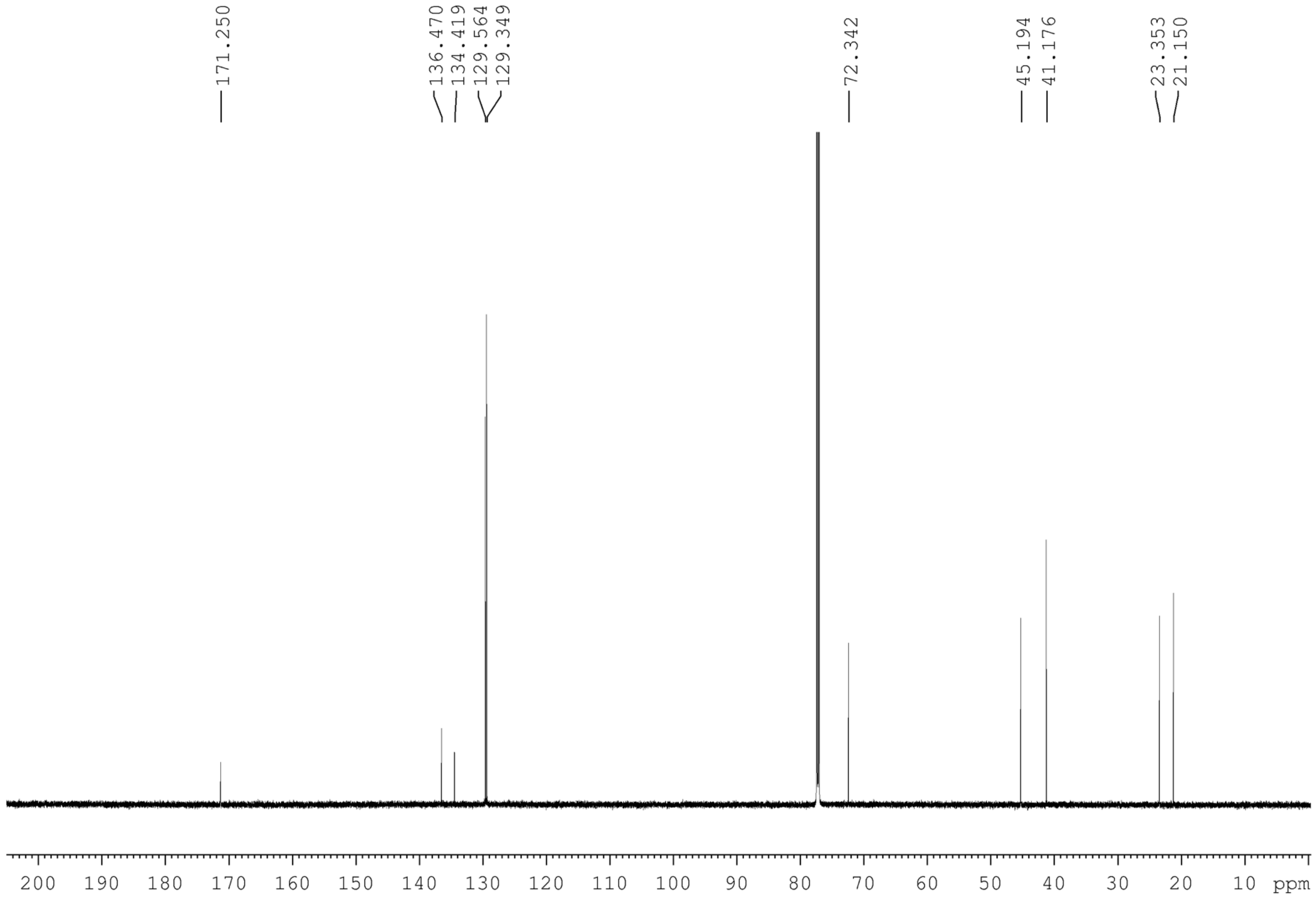

(*tert*-butyl (*S*)-(2-hydroxy-3-(*p*-tolyl)propyl)carbamate (Boc-1s)

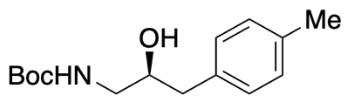

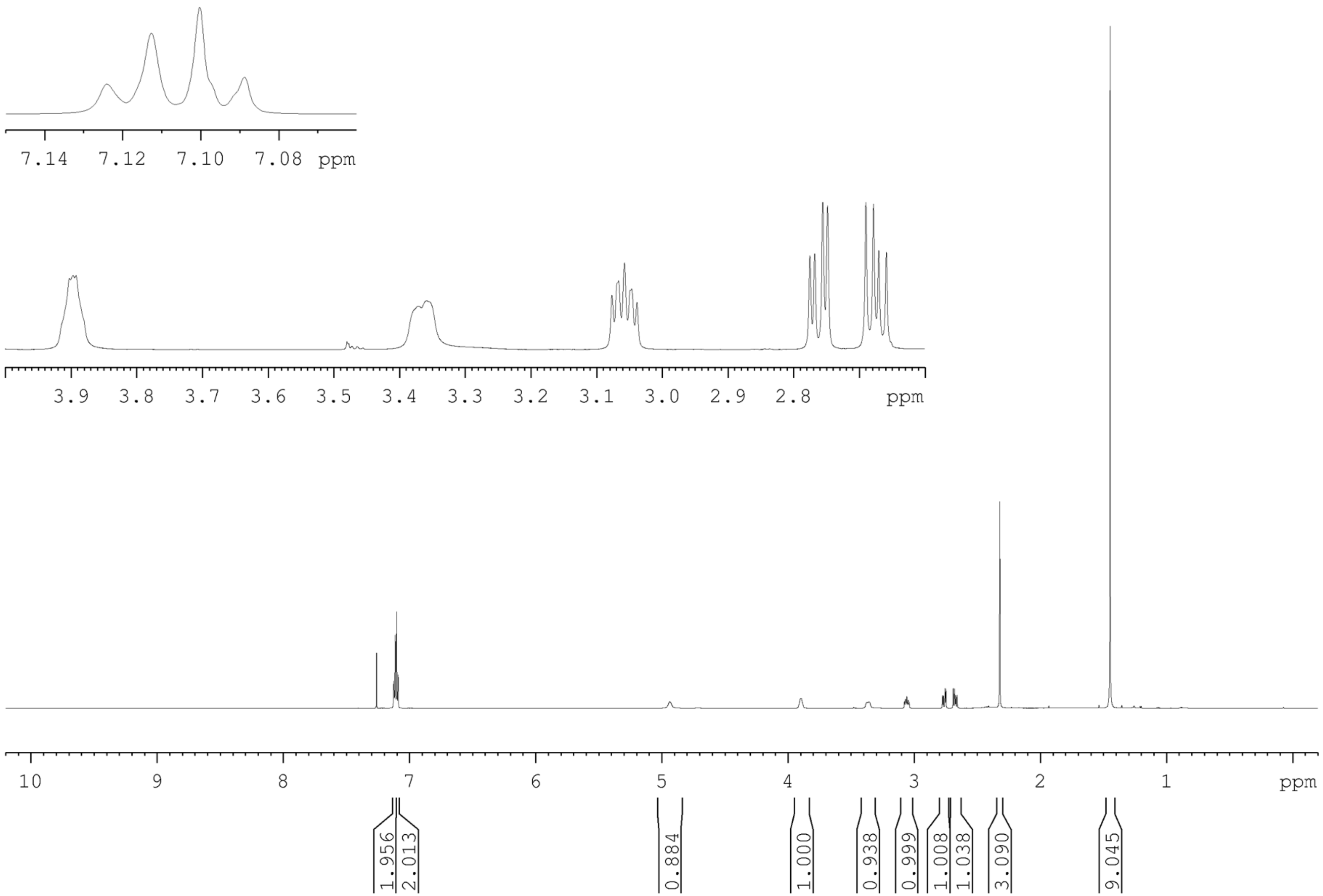

(*tert*-butyl (*S*)-(2-hydroxy-3-(*p*-tolyl)propyl)carbamate (Boc-1s)

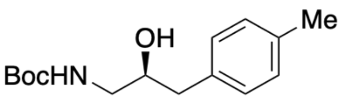

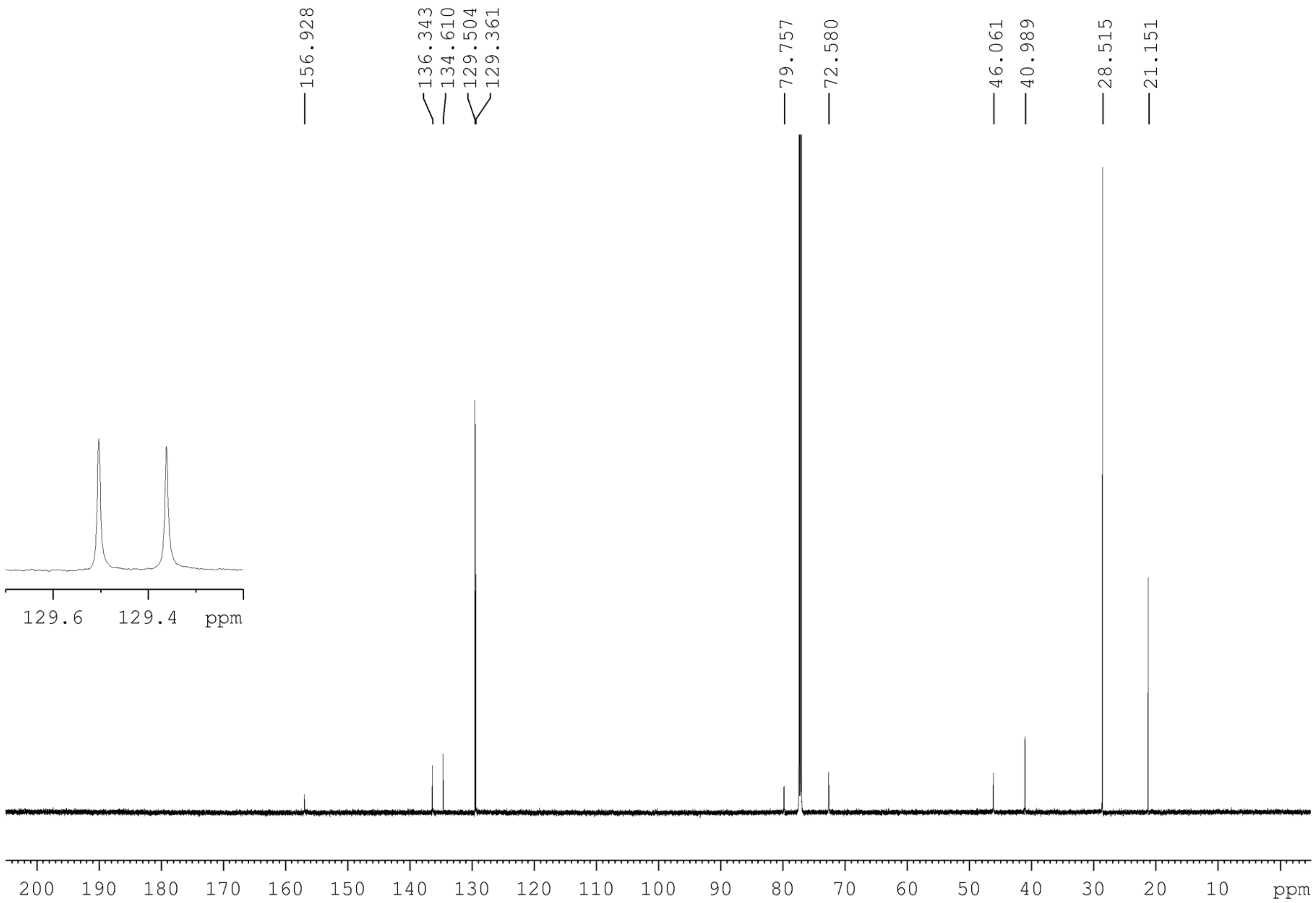

2-(4-fluorobenzyl)oxirane (1t-I_1)

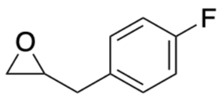

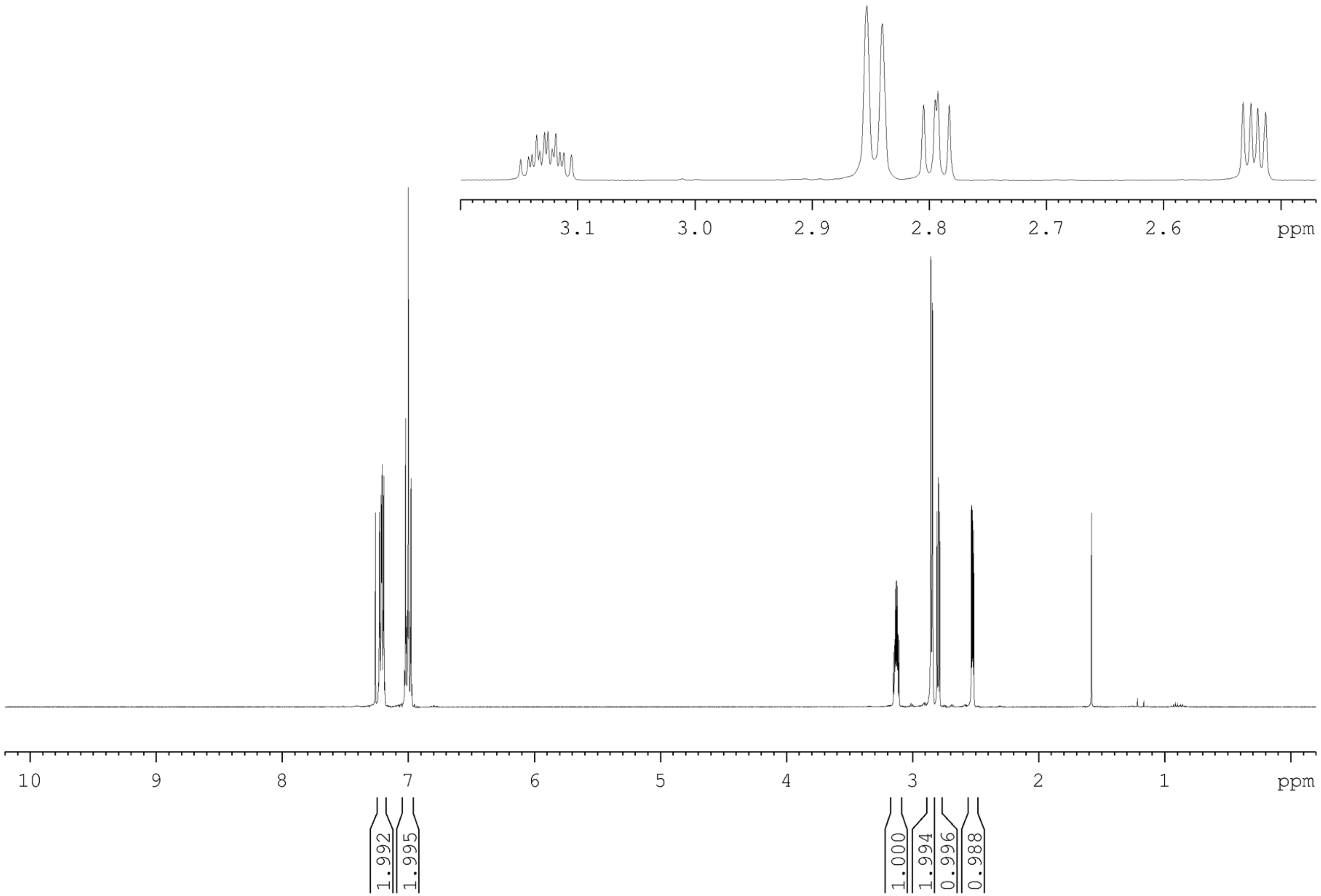

2-(4-fluorobenzyl)oxirane (1t-I$_1$)

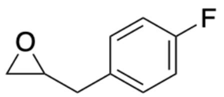

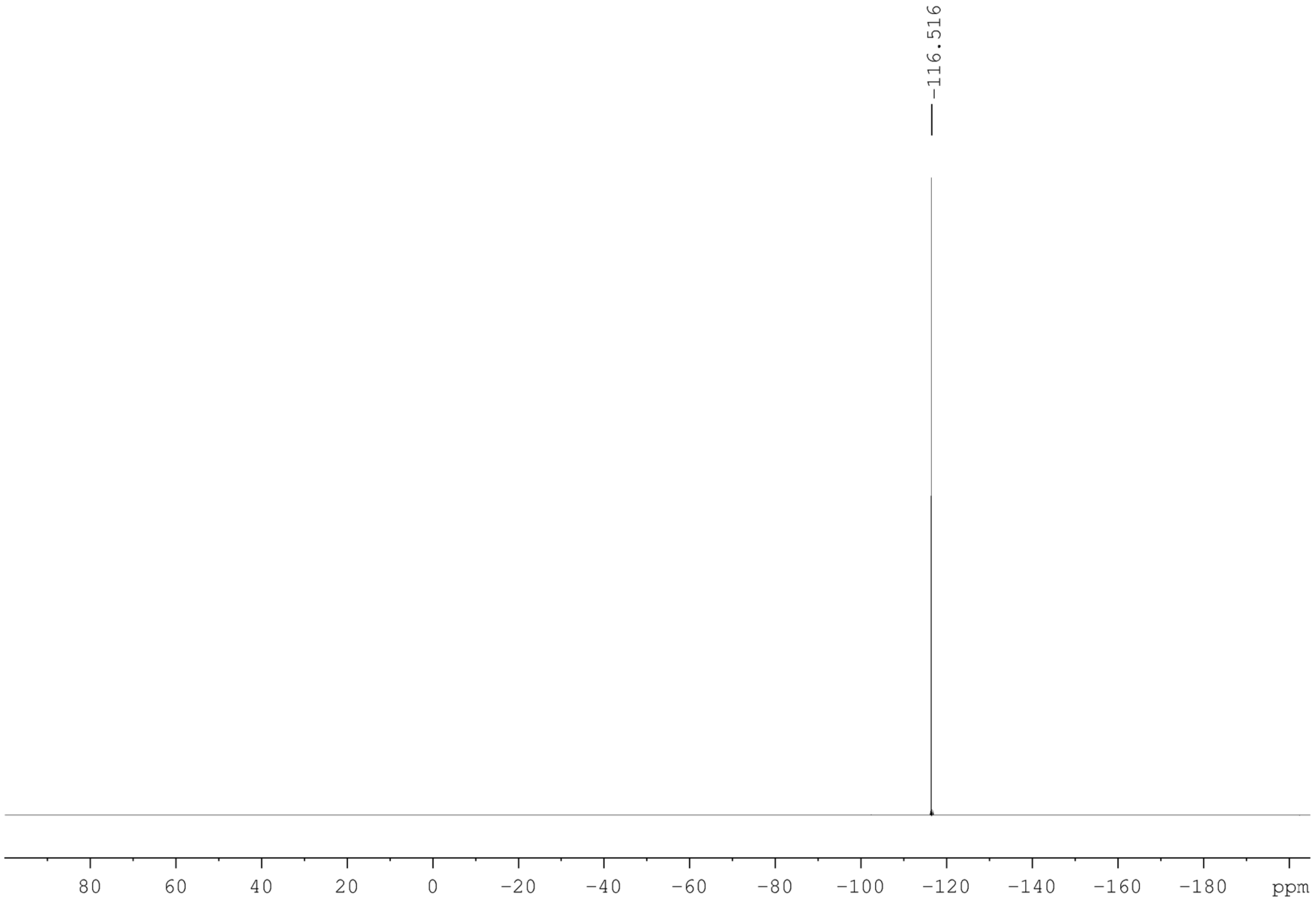

2-(4-fluorobenzyl)oxirane (1t-I_1)

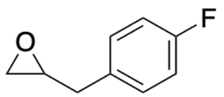

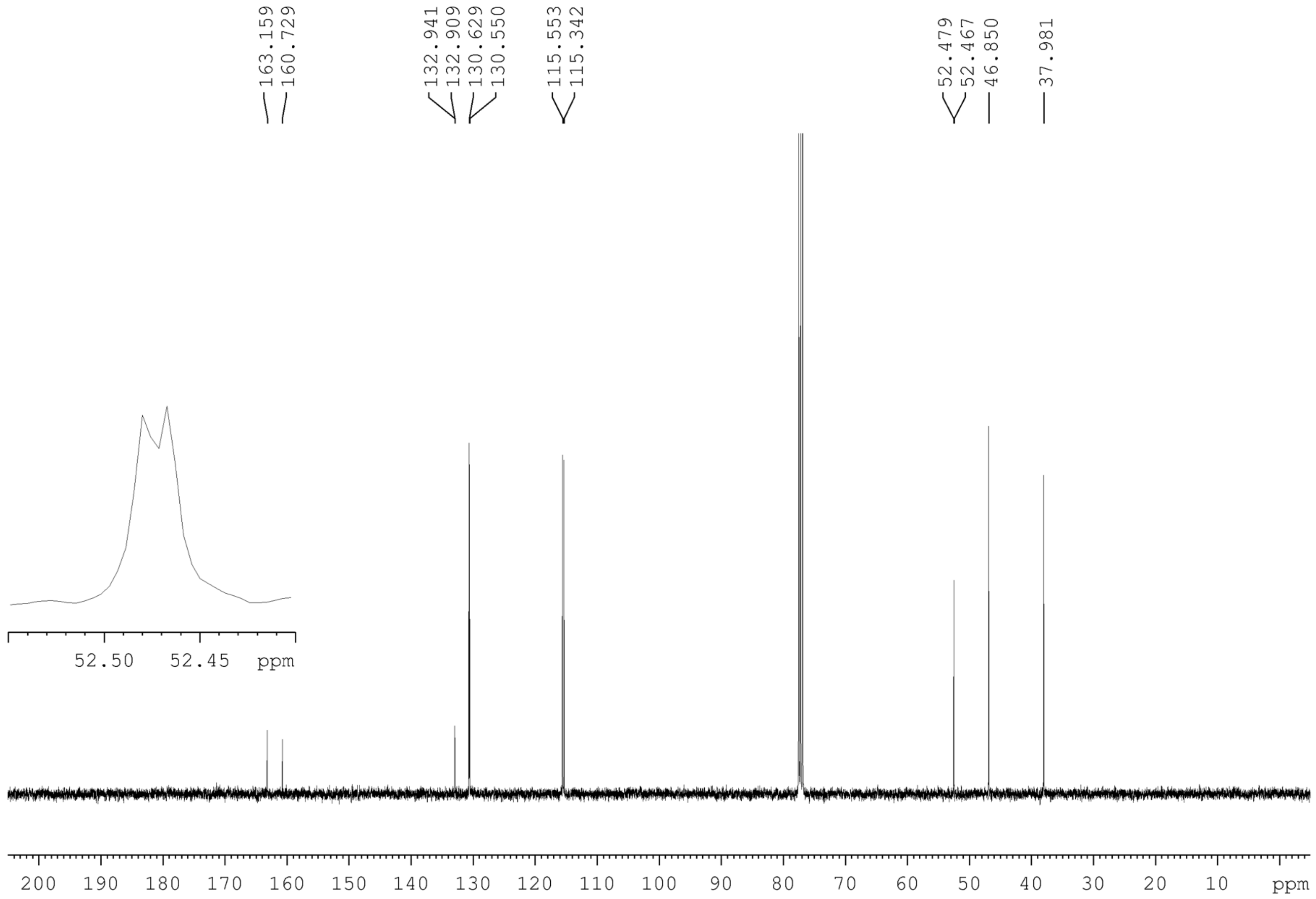

1-amino-3-(4-fluorophenyl)propan-2-ol (1t-I_2)

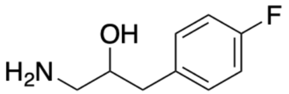

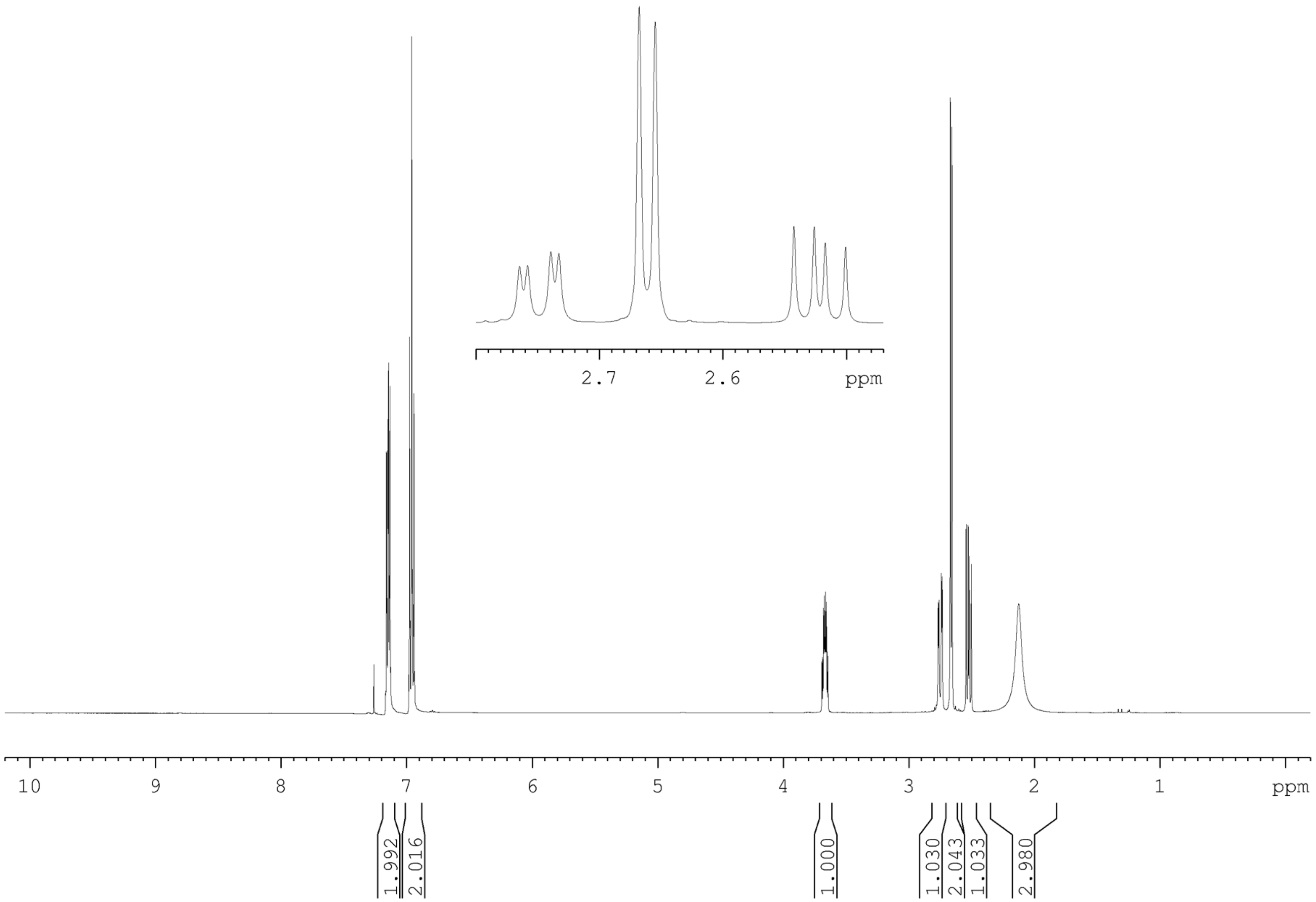

1-amino-3-(4-fluorophenyl)propan-2-ol (1t-I_2)

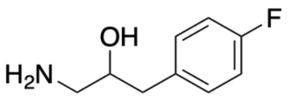

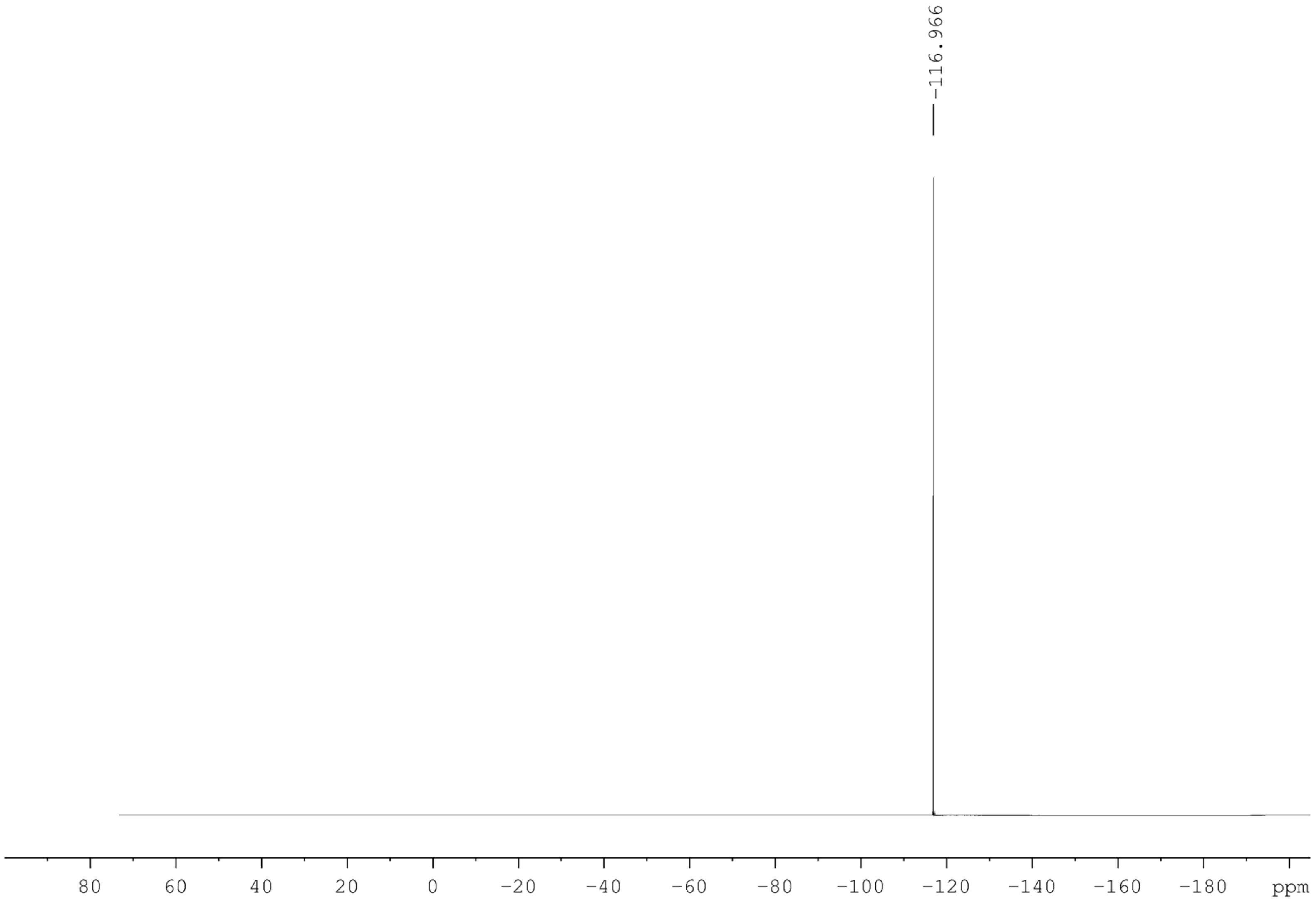

1-amino-3-(4-fluorophenyl)propan-2-ol (1t-I_2)

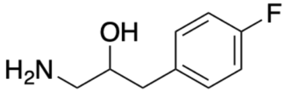

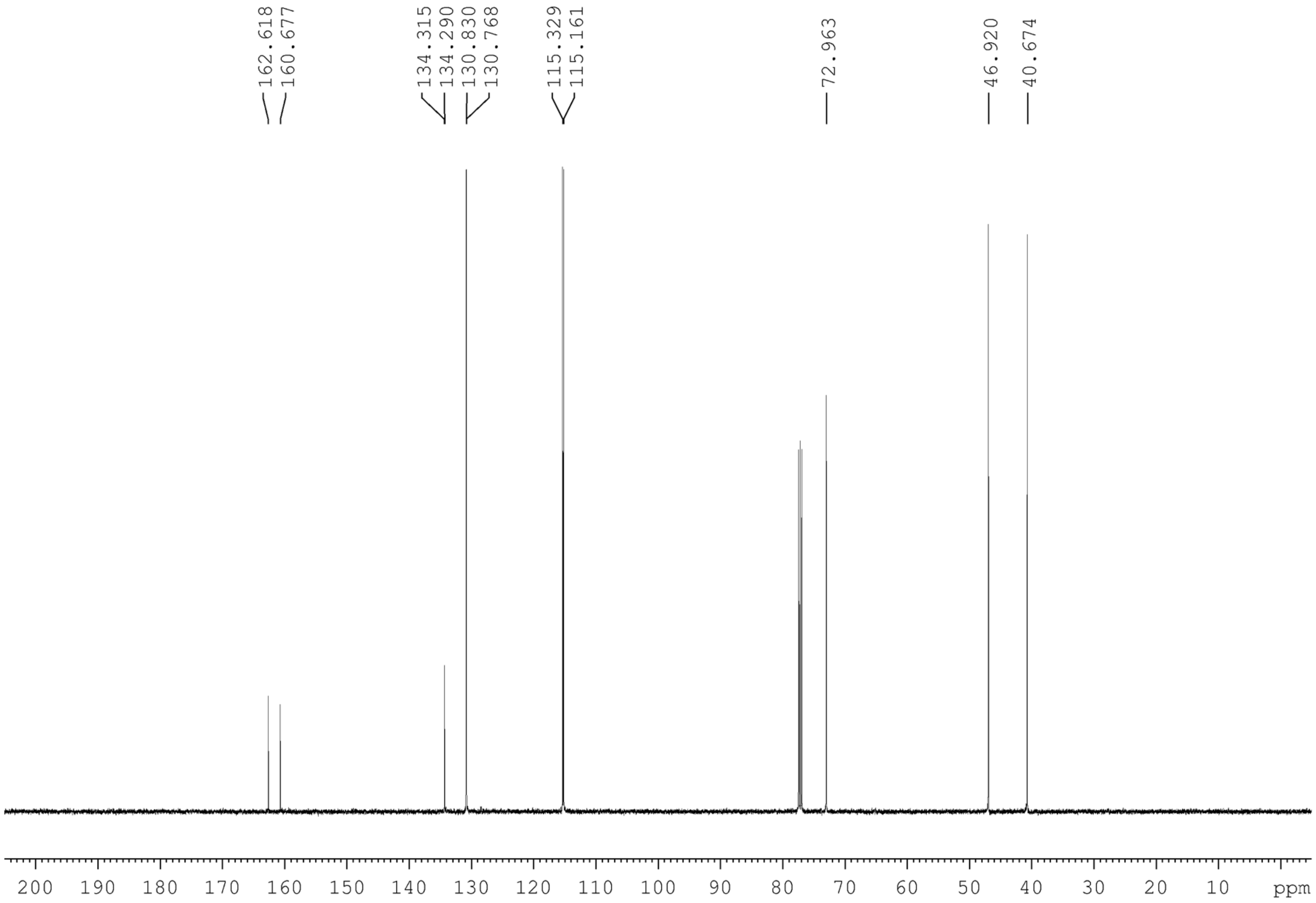

***N*-(3-(4-fluorophenyl)-2-hydroxypropyl)acetamide (1t-rac)**

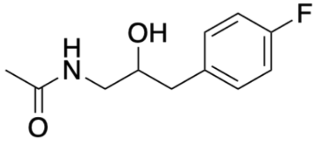

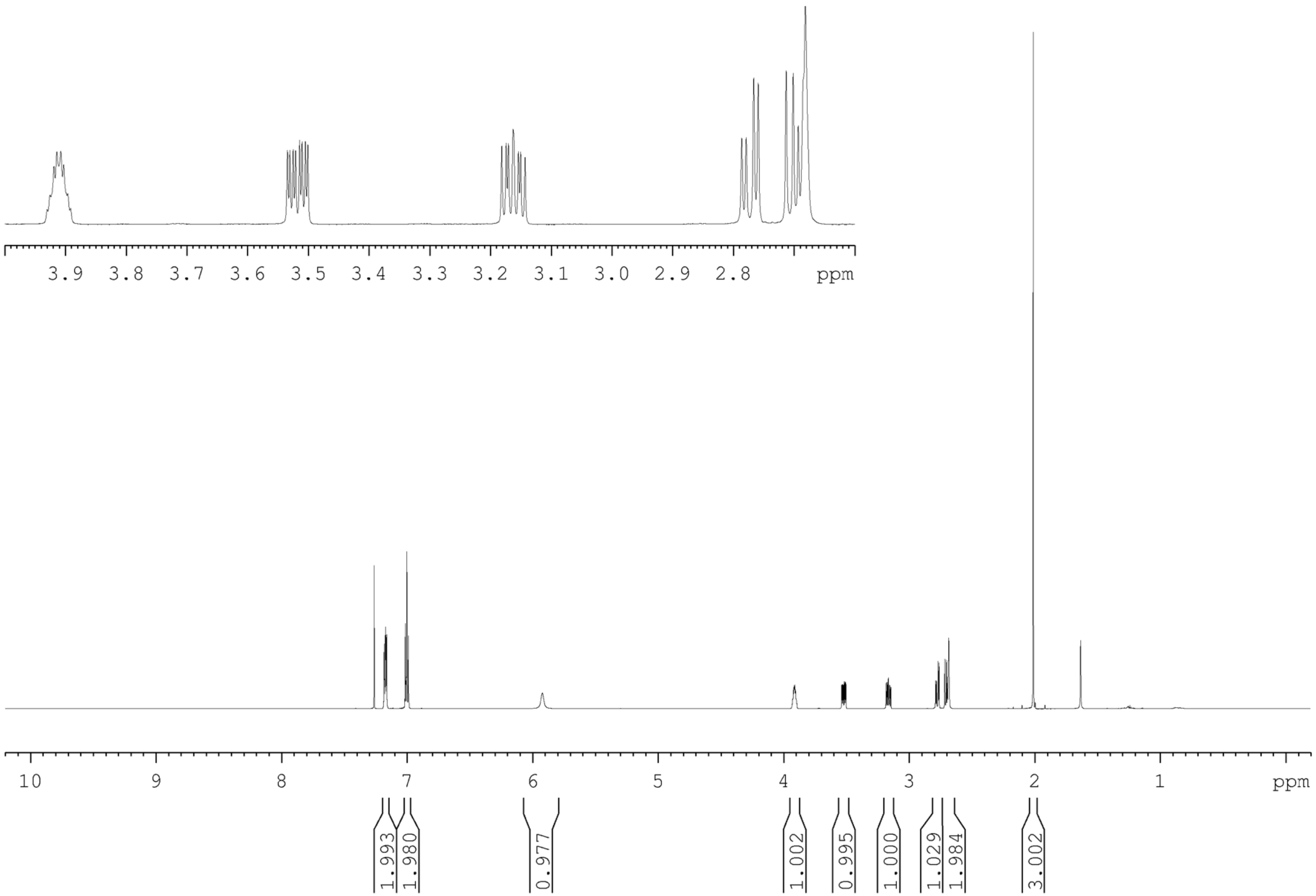

N-(3-(4-fluorophenyl)-2-hydroxypropyl)acetamide (1t-rac)

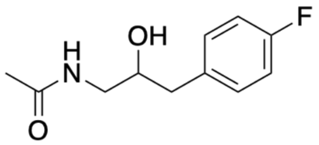

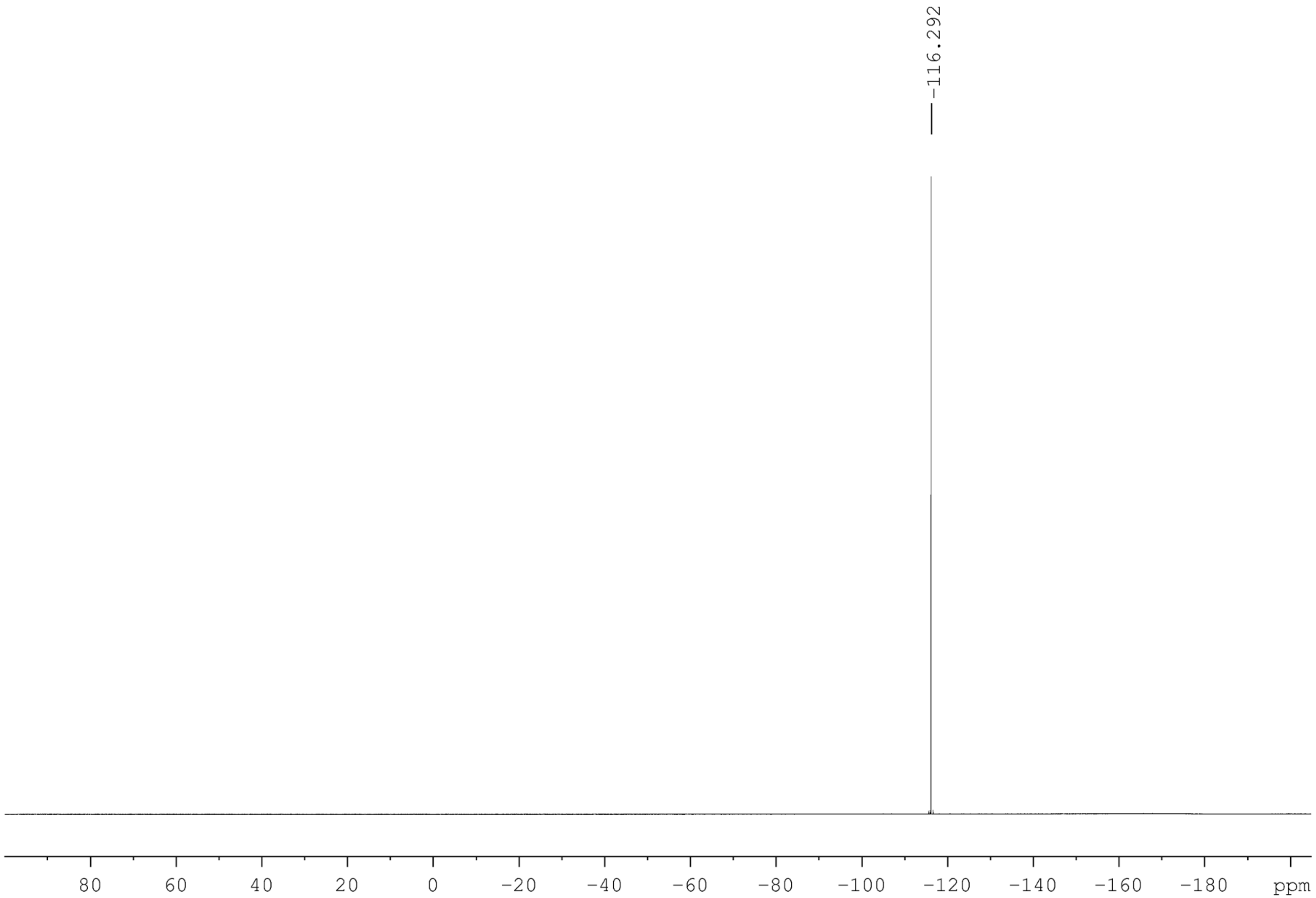

N-(3-(4-fluorophenyl)-2-hydroxypropyl)acetamide (1t-rac)

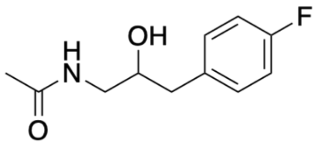

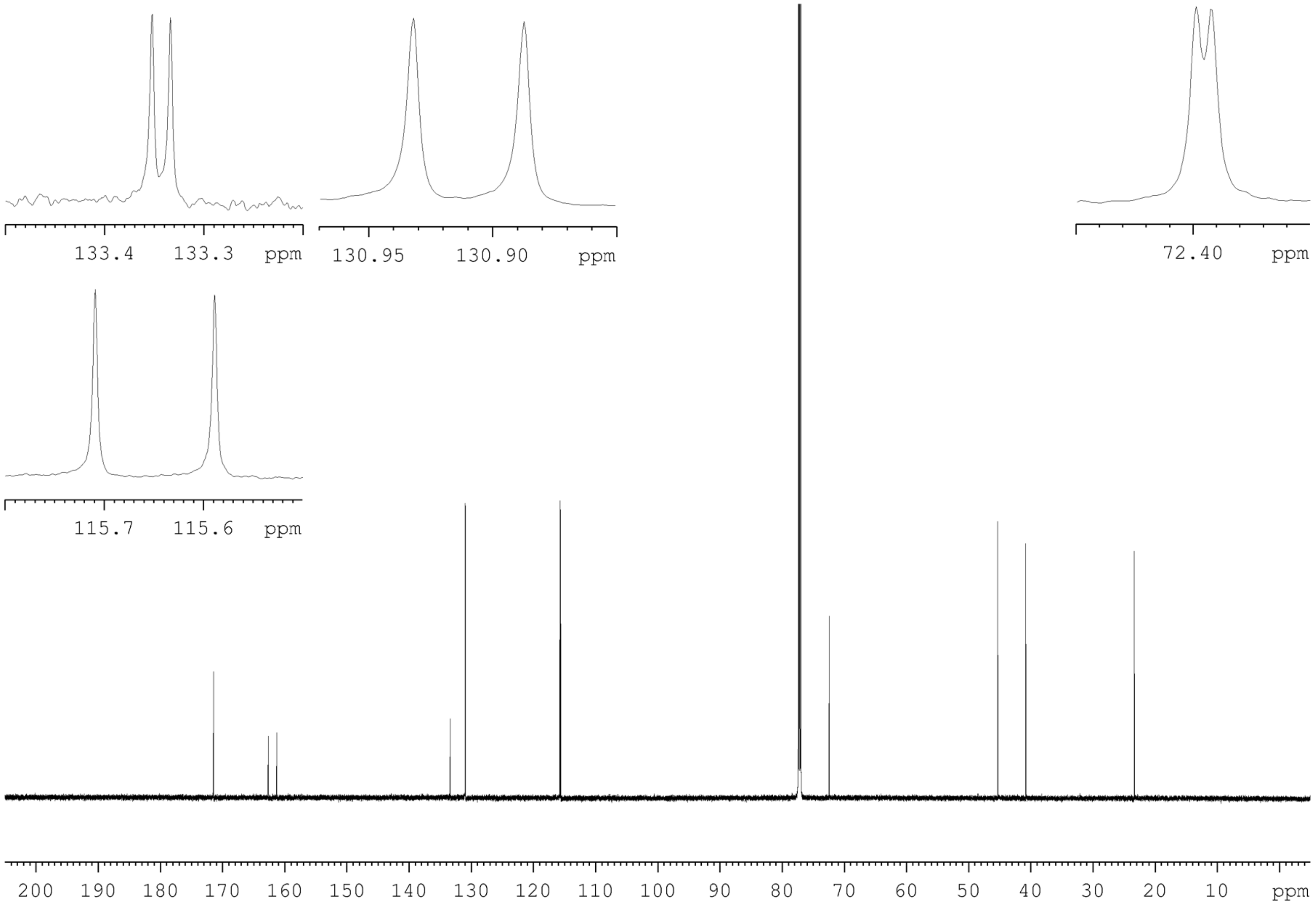

(S)-N-(3-(4-fluorophenyl)-2-hydroxypropyl)acetamide (1t)

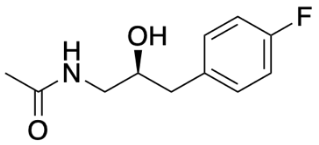

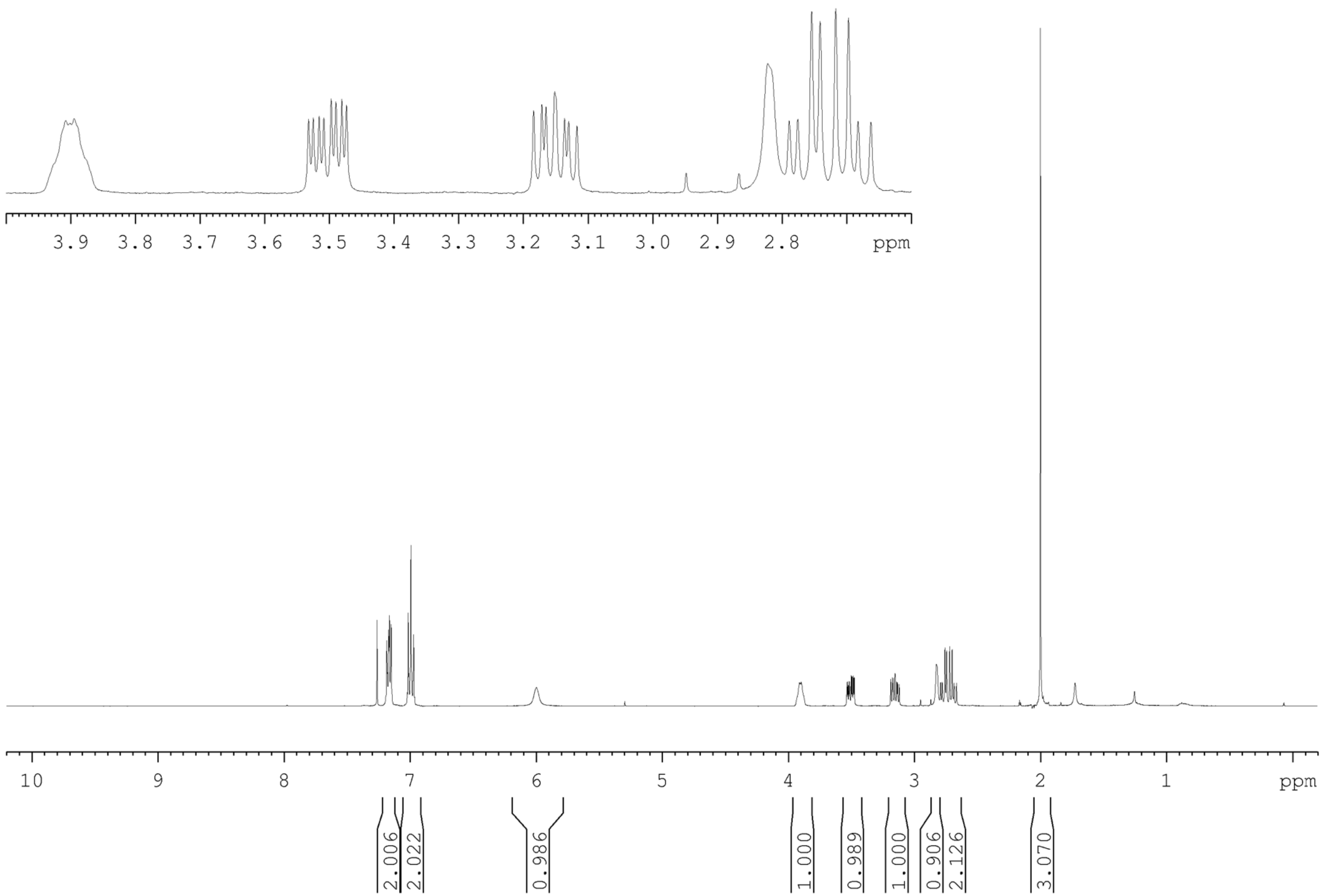

(*S*)-*N*-(3-(4-fluorophenyl)-2-hydroxypropyl)acetamide (1t)

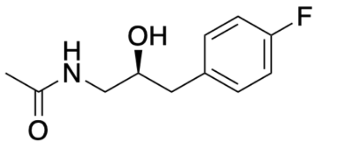

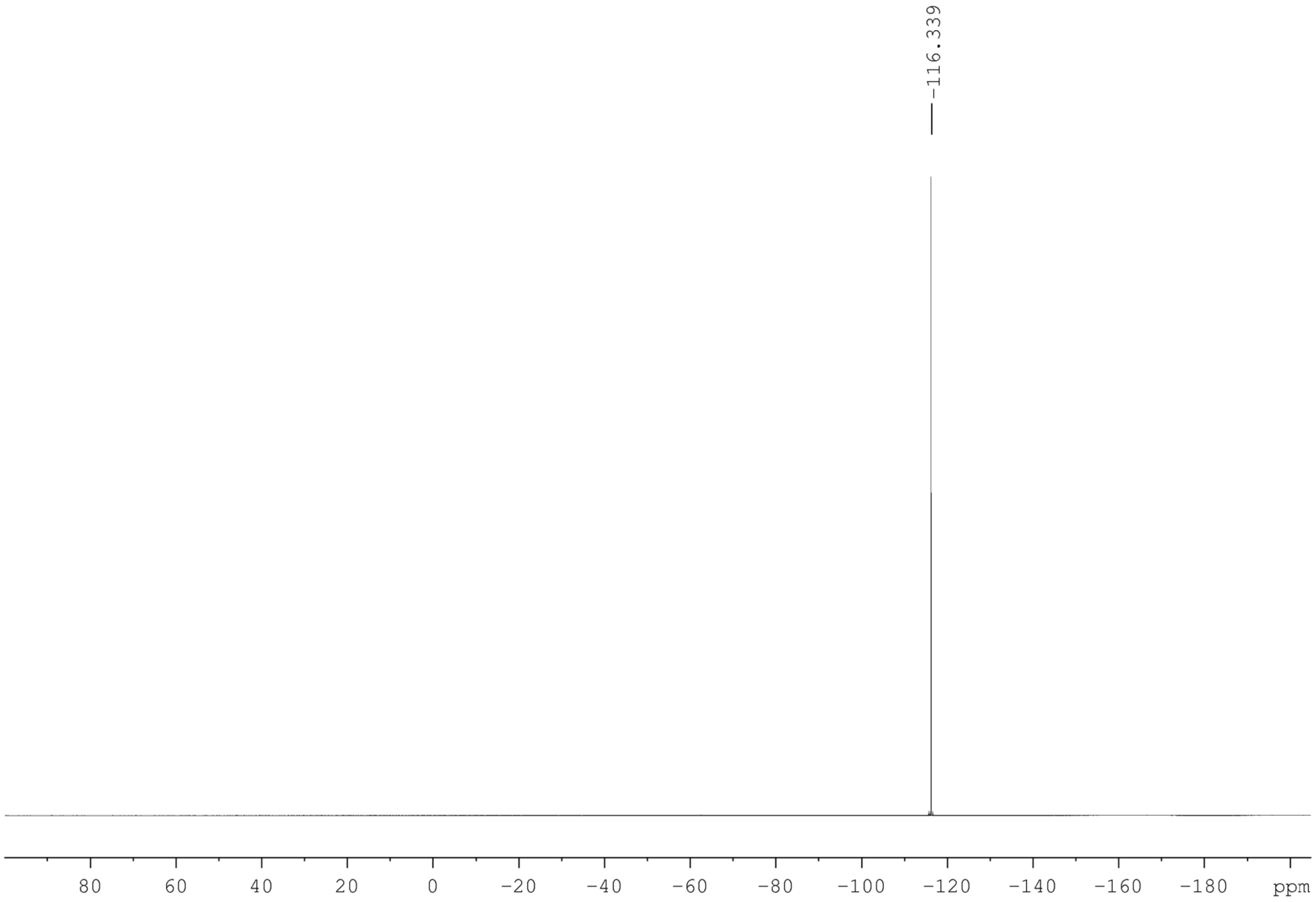

(*S*)-*N*-(3-(4-fluorophenyl)-2-hydroxypropyl)acetamide (1t)

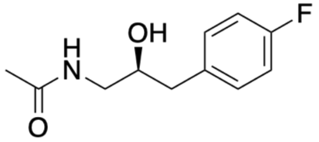

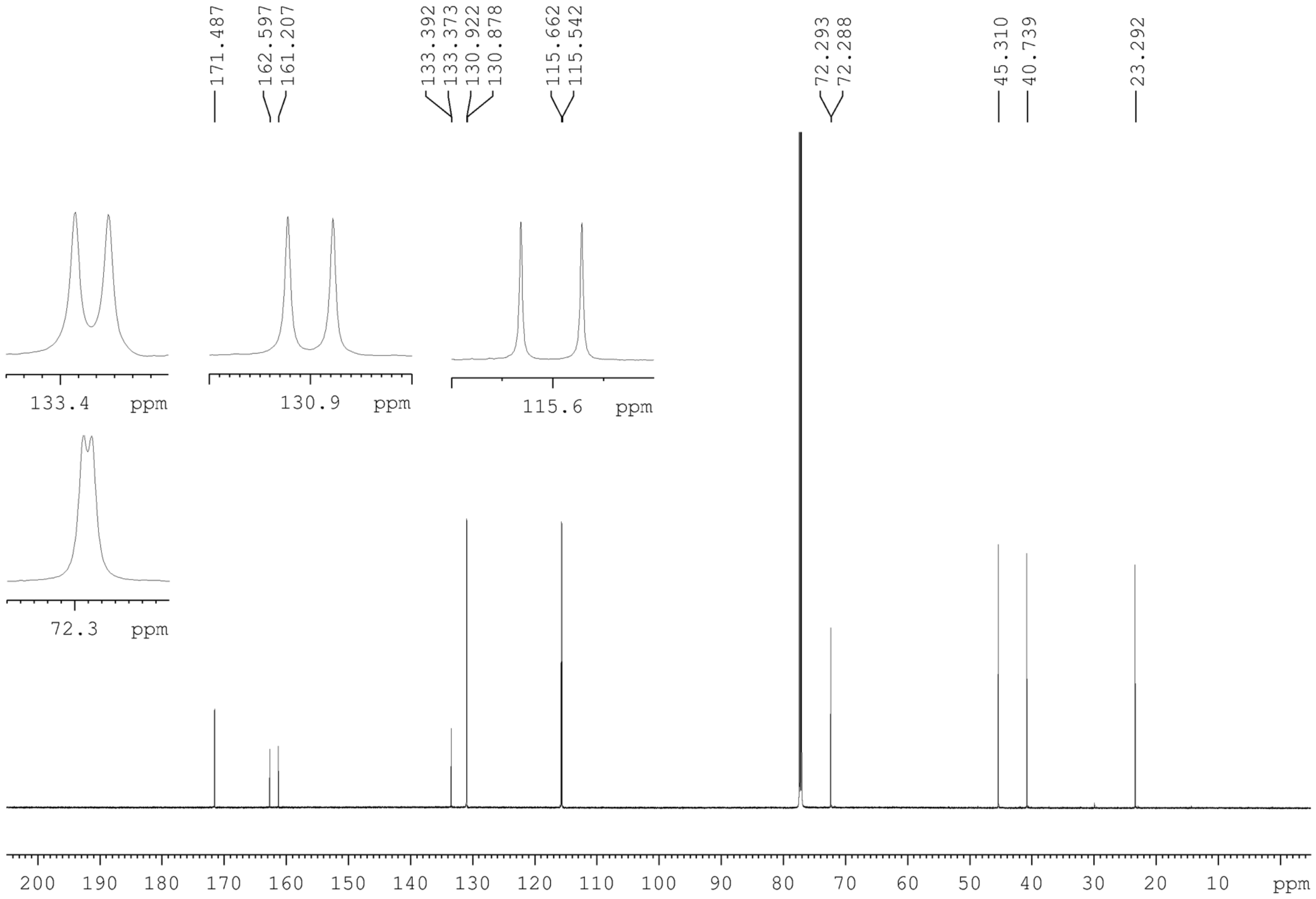

1-amino-3-(3-chlorophenyl)propan-2-ol (1u-I_2)

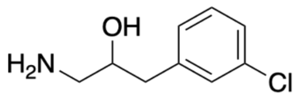

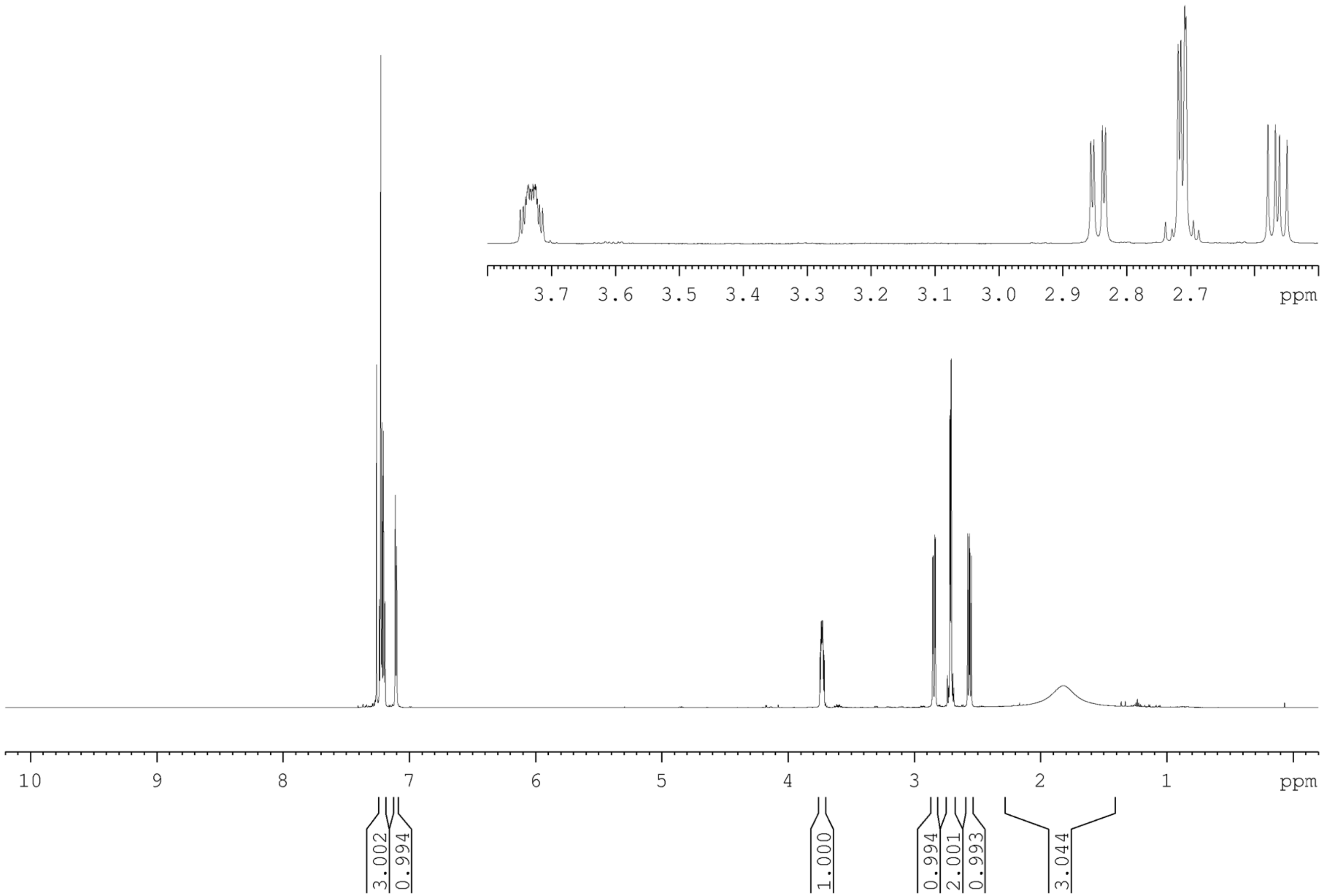

1-amino-3-(3-chlorophenyl)propan-2-ol (1u-I_2)

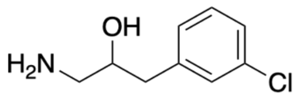

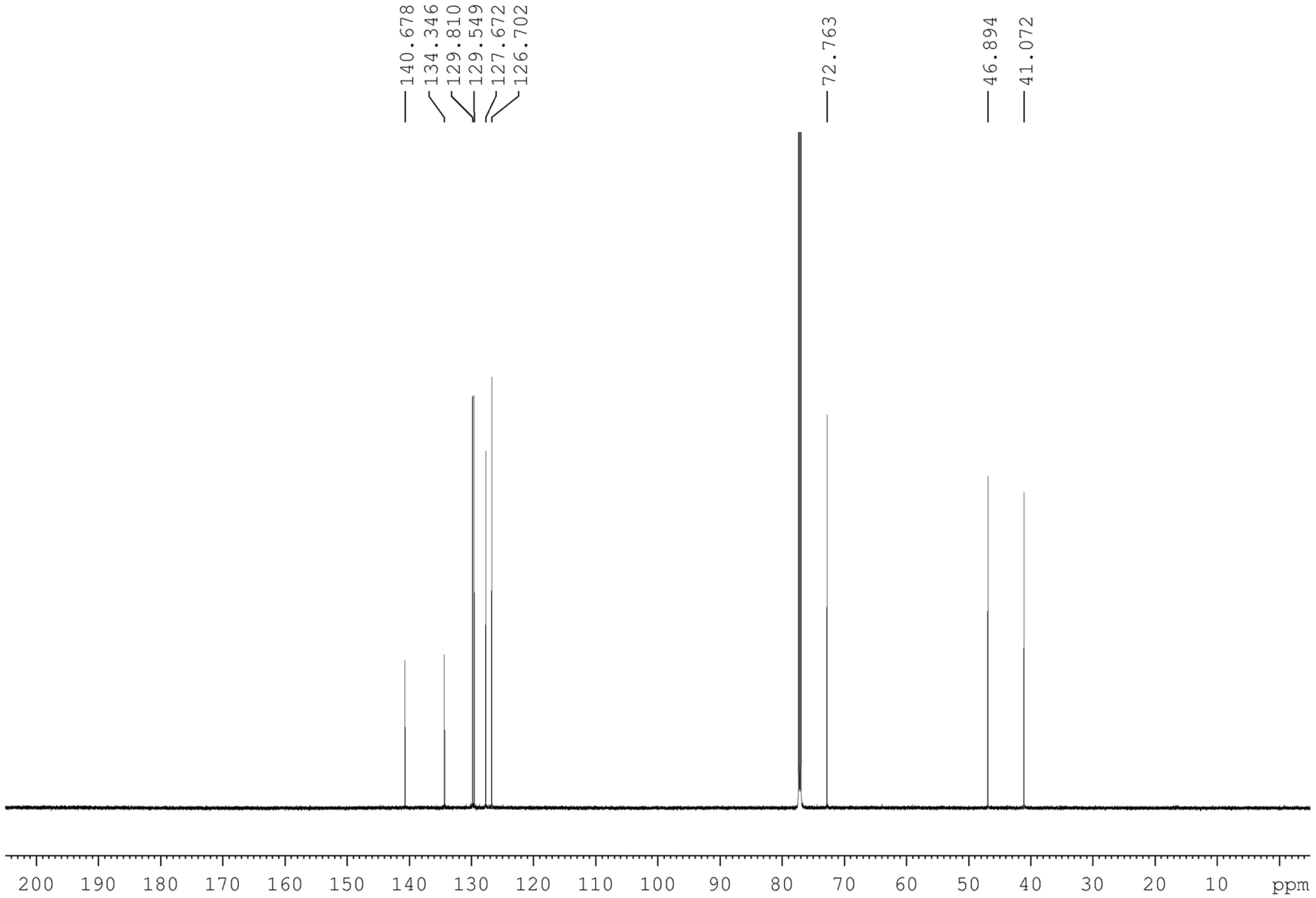

N-(3-(3-chlorophenyl)-2-hydroxypropyl)acetamide (1u-rac)

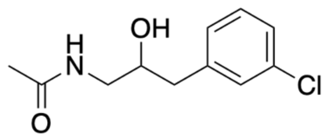

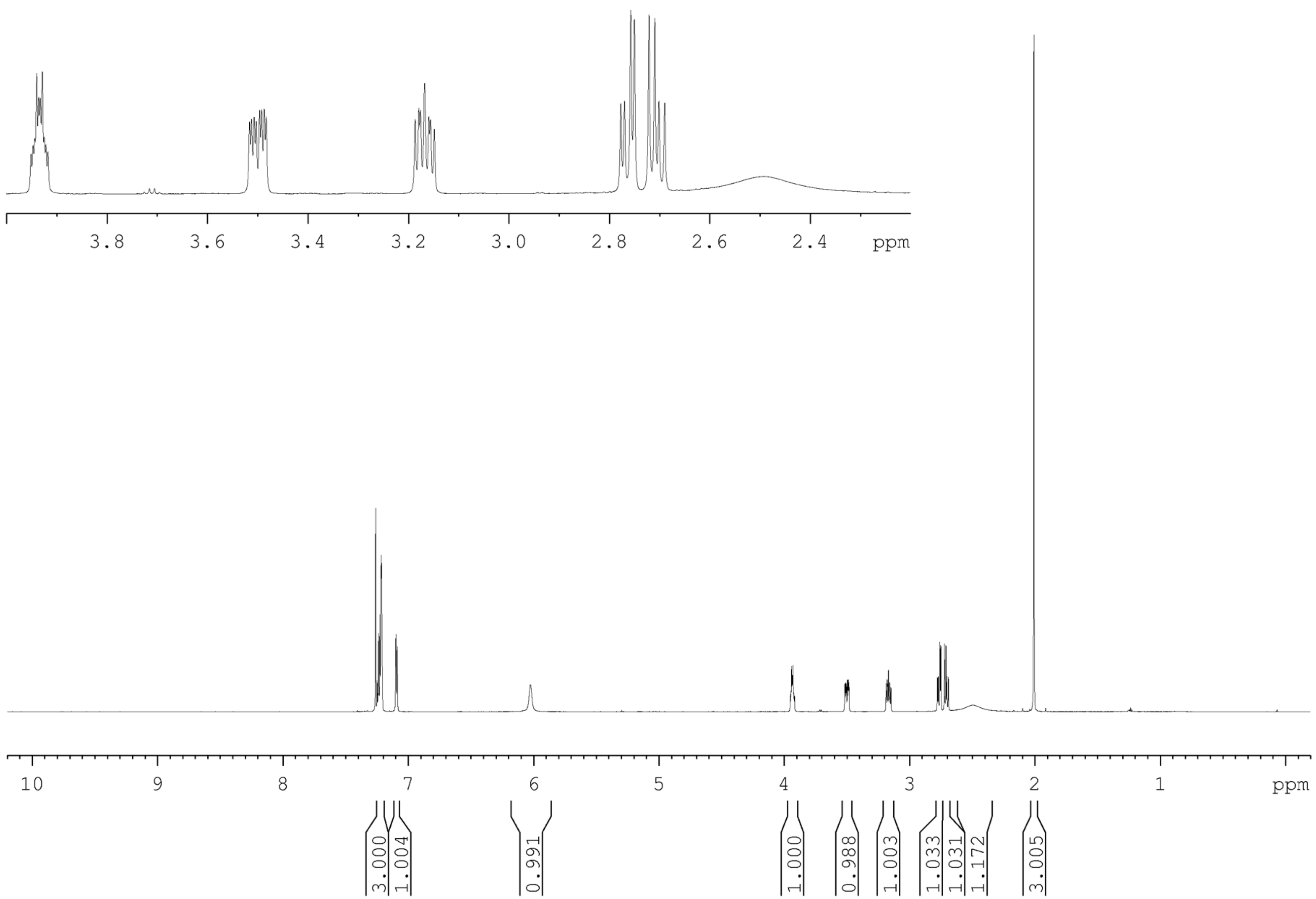

N-(3-(3-chlorophenyl)-2-hydroxypropyl)acetamide (1u-rac)

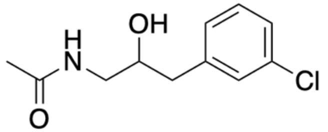

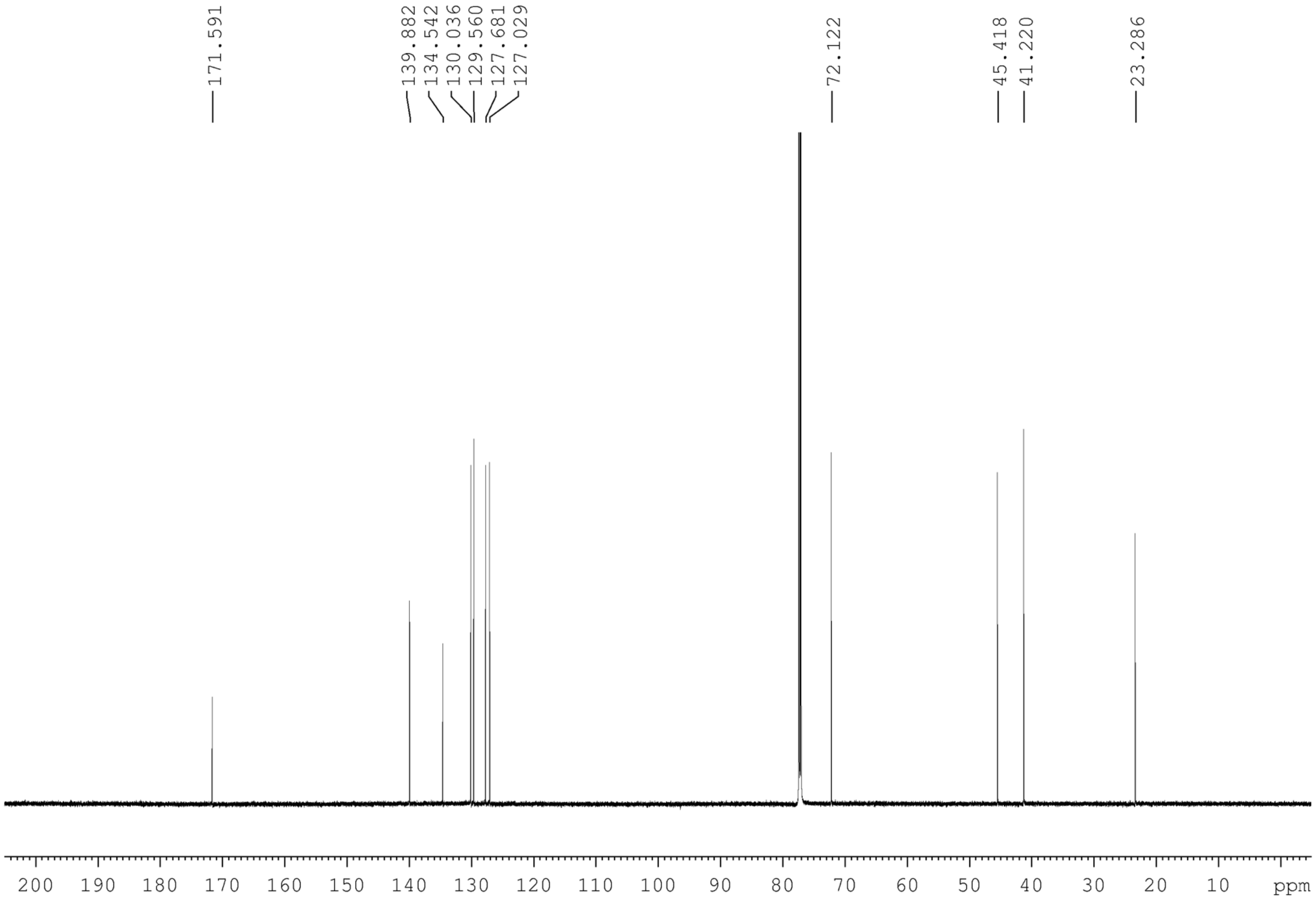

(*S*)-*N*-(3-(3-chlorophenyl)-2-hydroxypropyl)acetamide (1u)

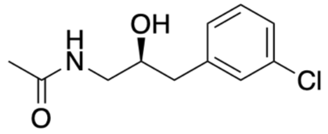

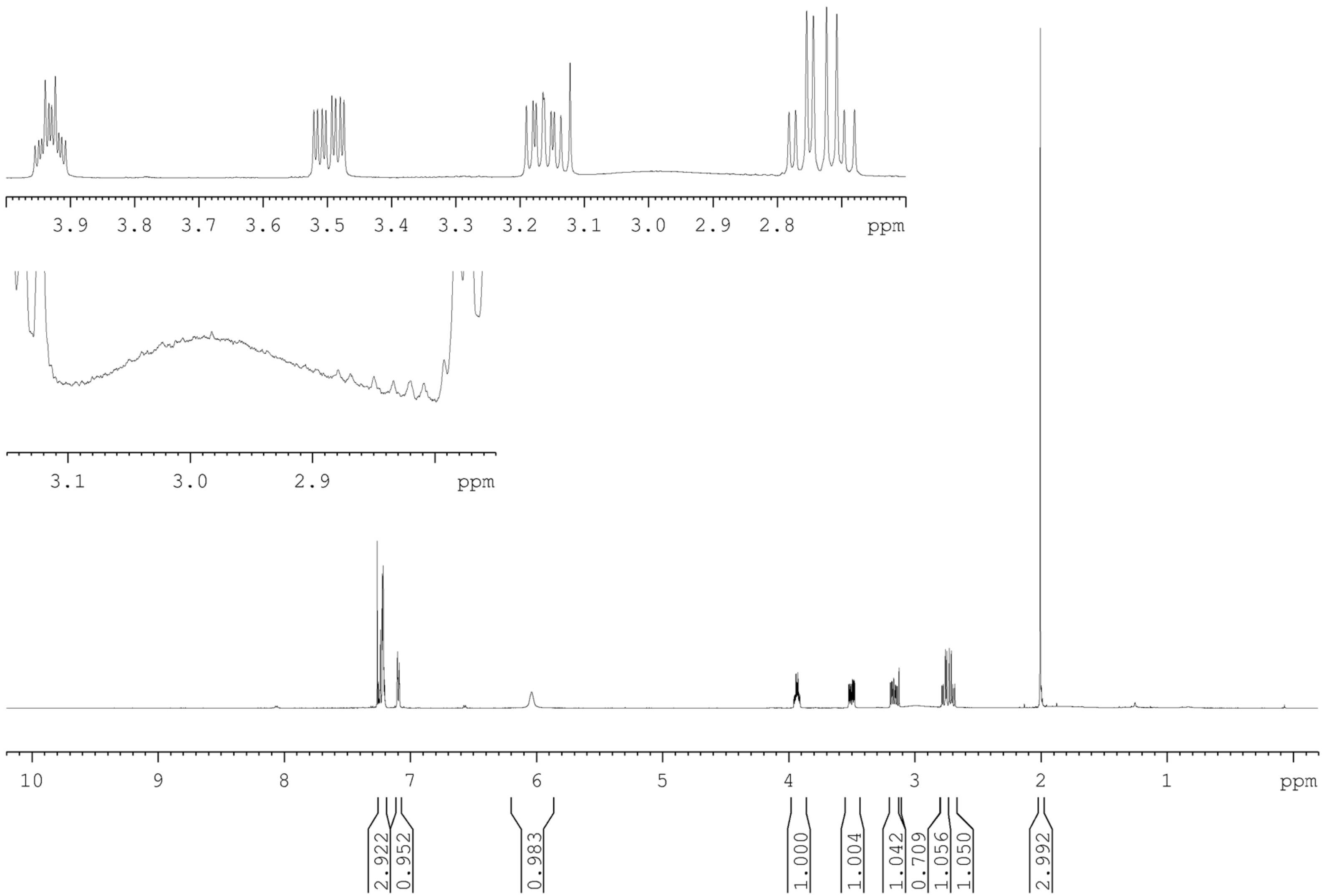

(*S*)-*N*-(3-(3-chlorophenyl)-2-hydroxypropyl)acetamide (1u)

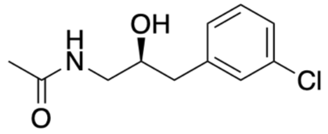

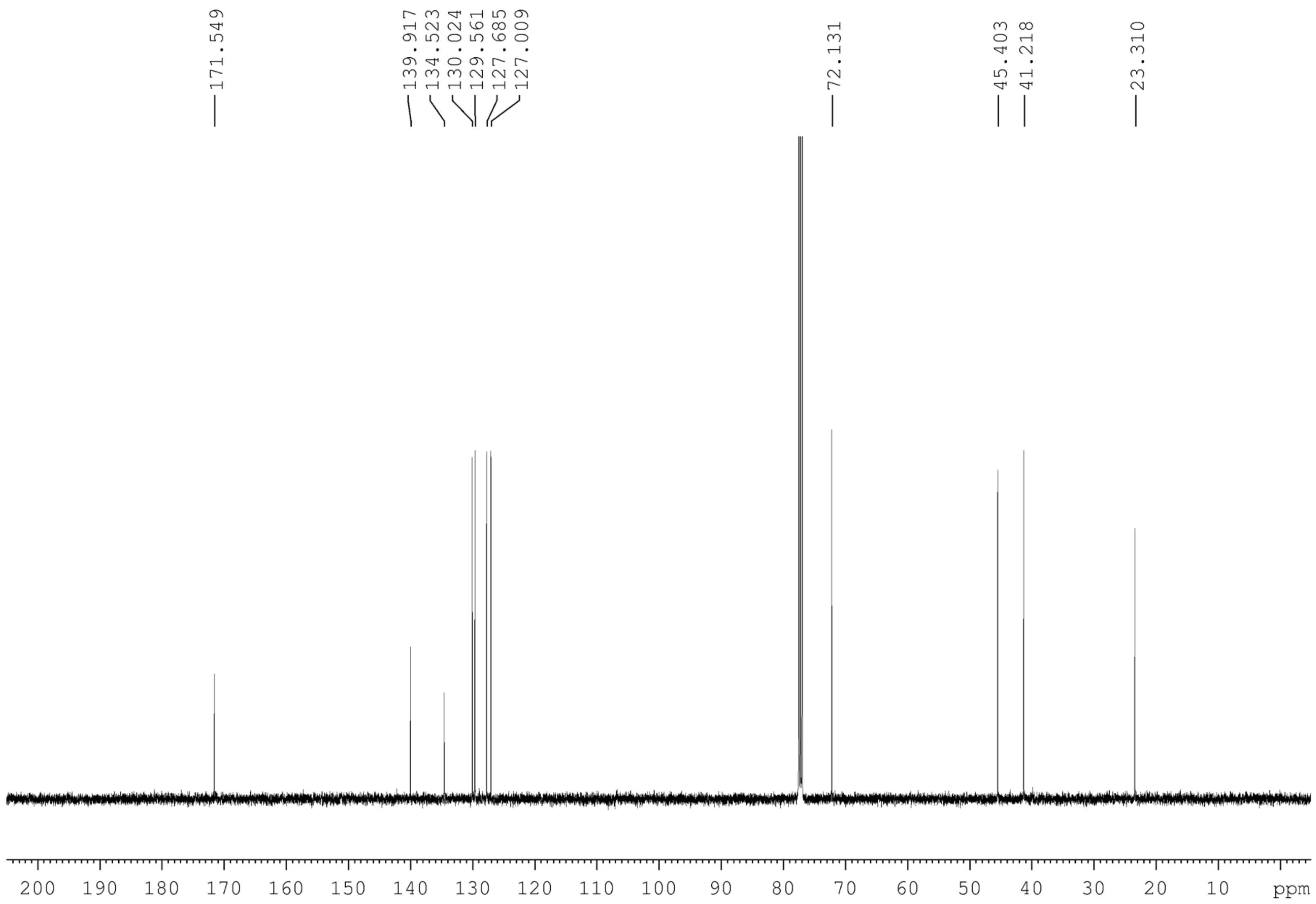

1-amino-3-(naphthalen-1-yl)propan-2-ol (1v-I_1)

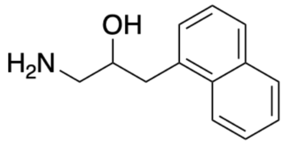

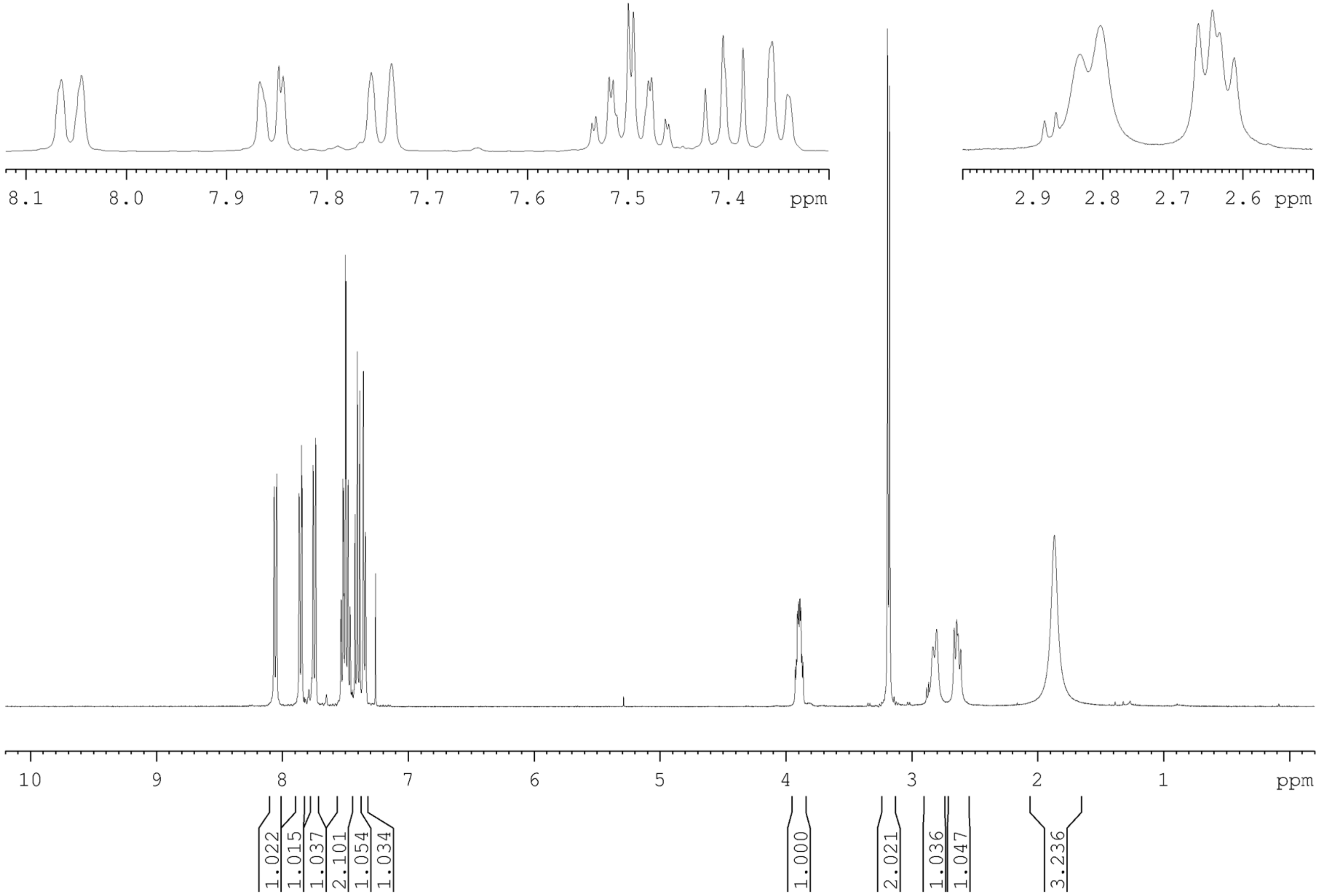

1-amino-3-(naphthalen-1-yl)propan-2-ol (1v-I_1)

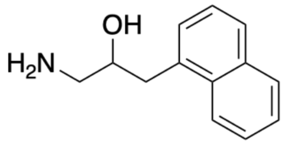

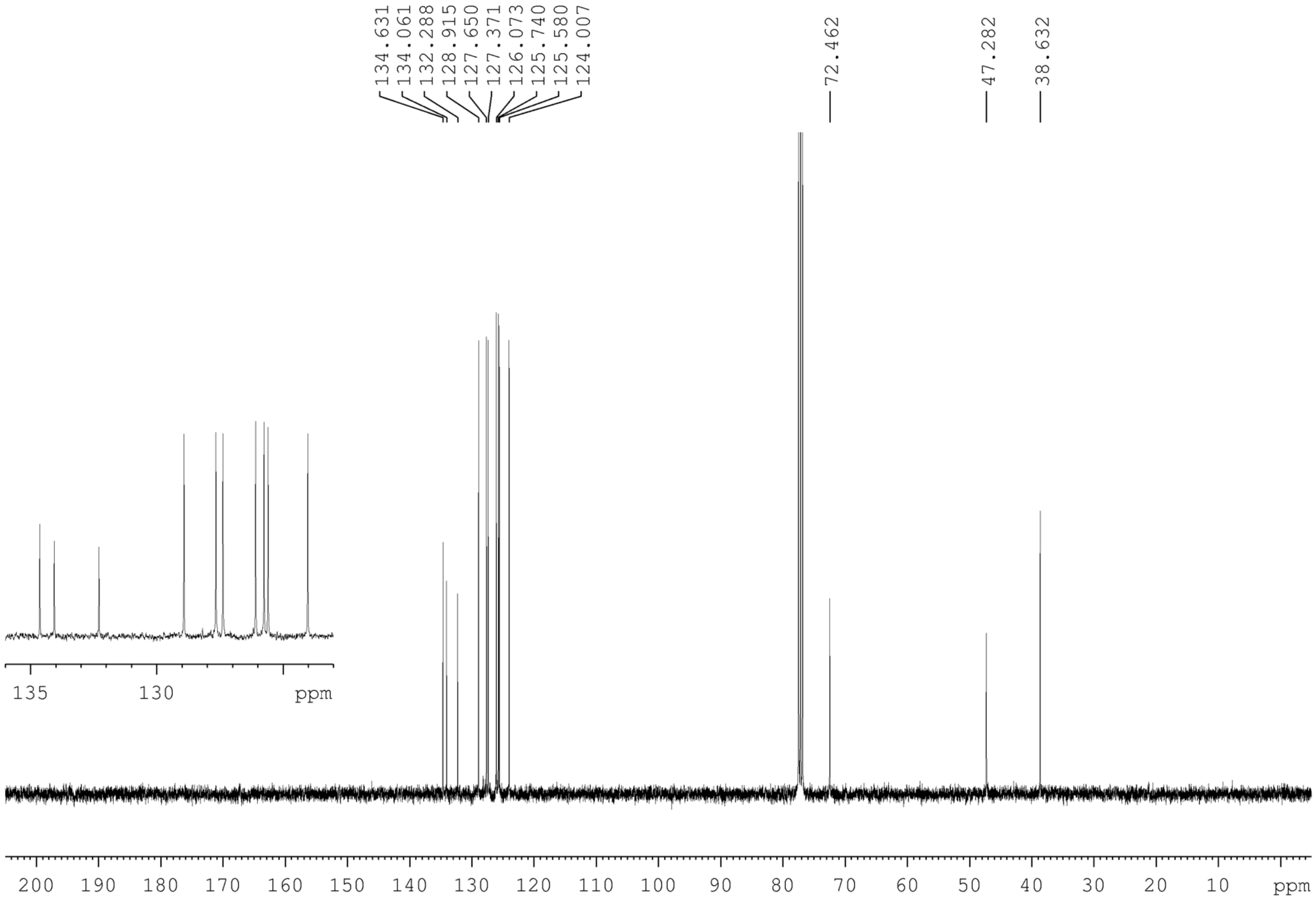

N-(2-hydroxy-3-(naphthalen-1-yl)propyl)acetamide (1v-rac)

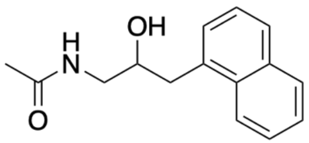

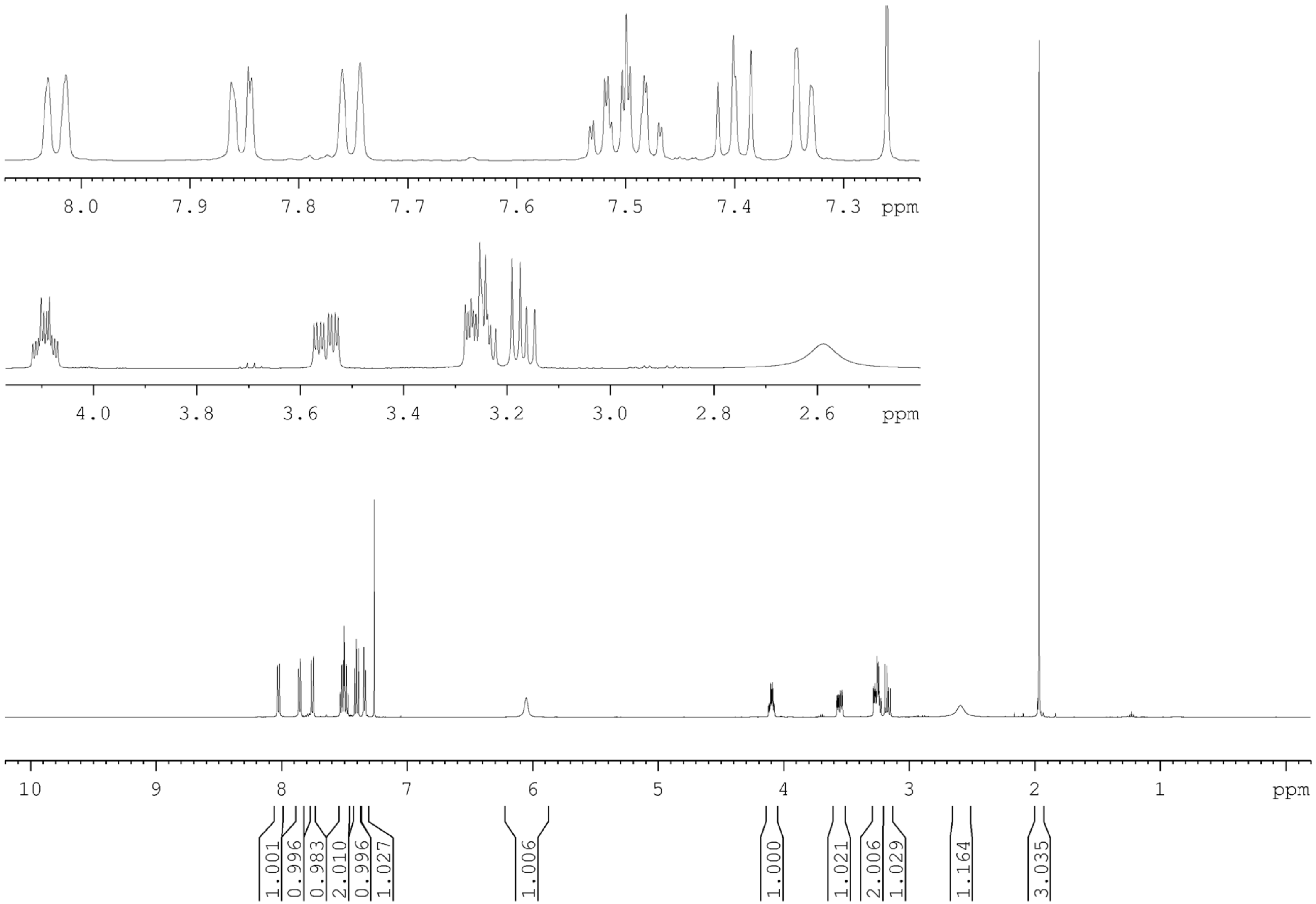

N-(2-hydroxy-3-(naphthalen-1-yl)propyl)acetamide (1v-rac)

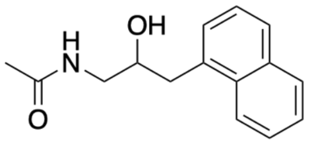

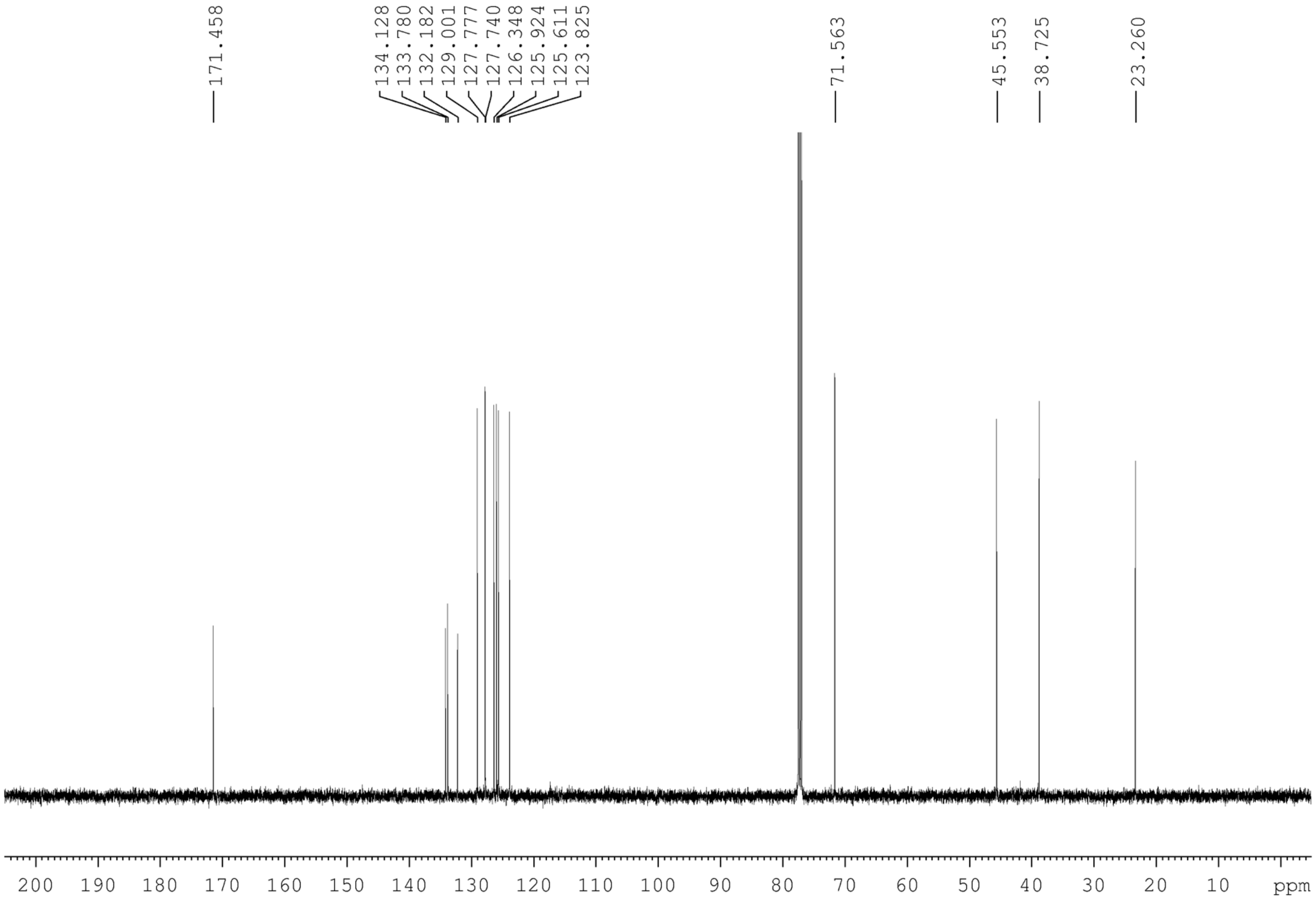

(*S*)-*N*-(2-hydroxy-3-(naphthalen-1-yl)propyl)acetamide (1v)

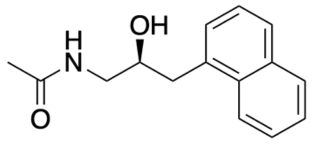

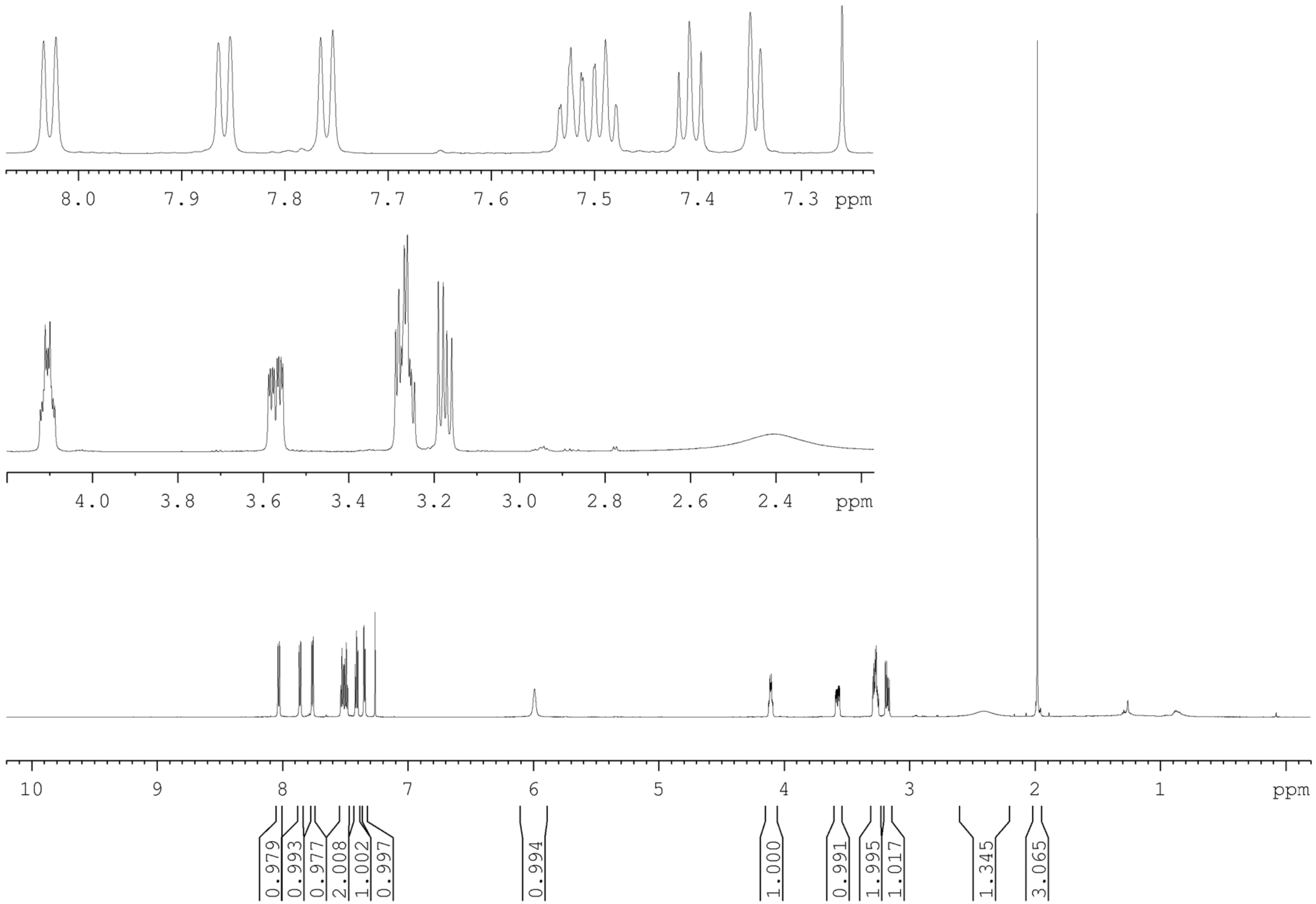

(*S*)-*N*-(2-hydroxy-3-(naphthalen-1-yl)propyl)acetamide (1v)

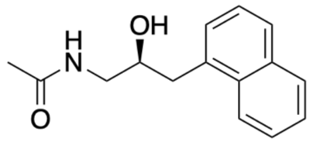

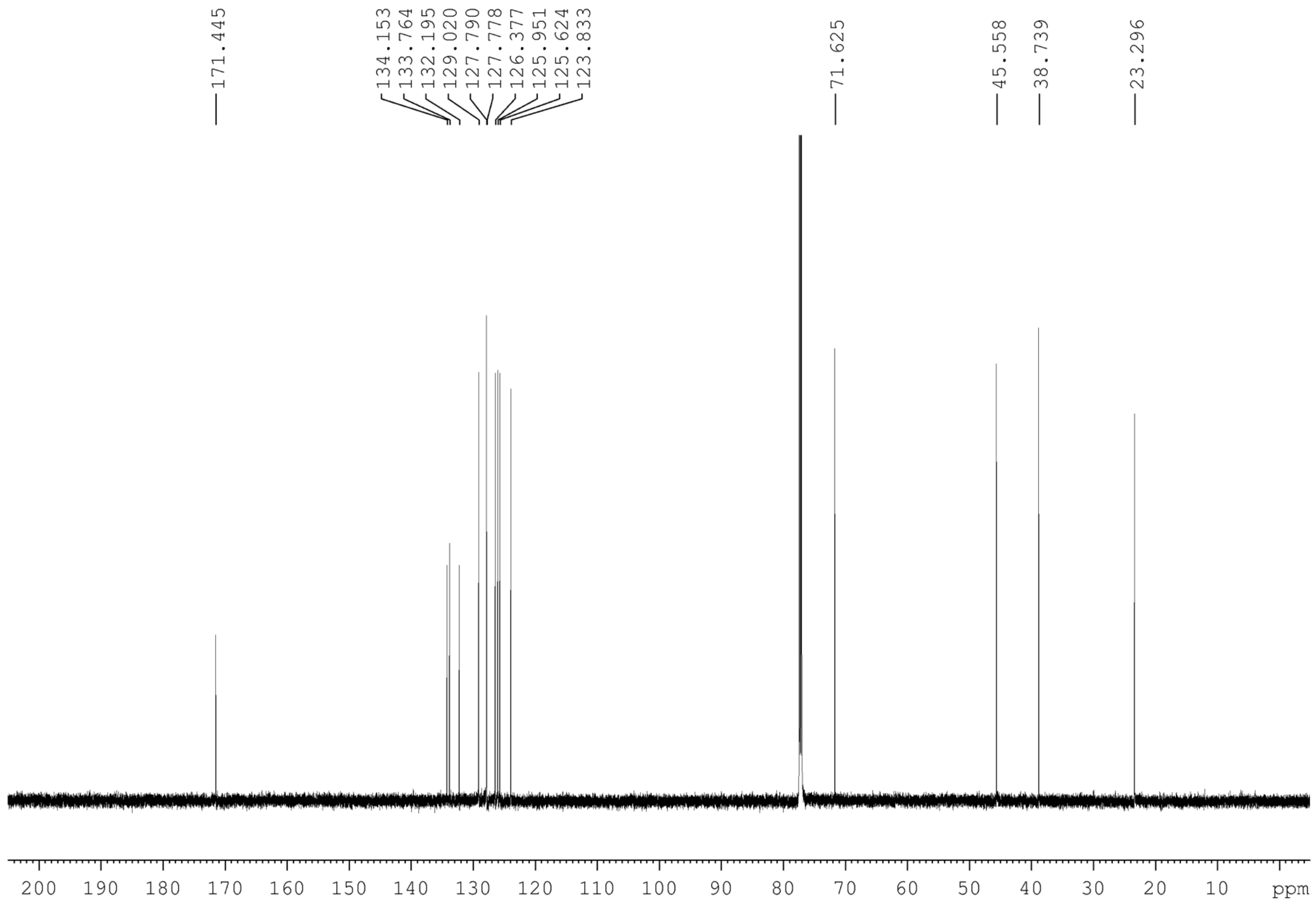

1-amino-3-(thiophen-3-yl)propan-2-ol (1w-I_2)

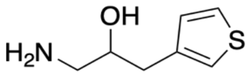

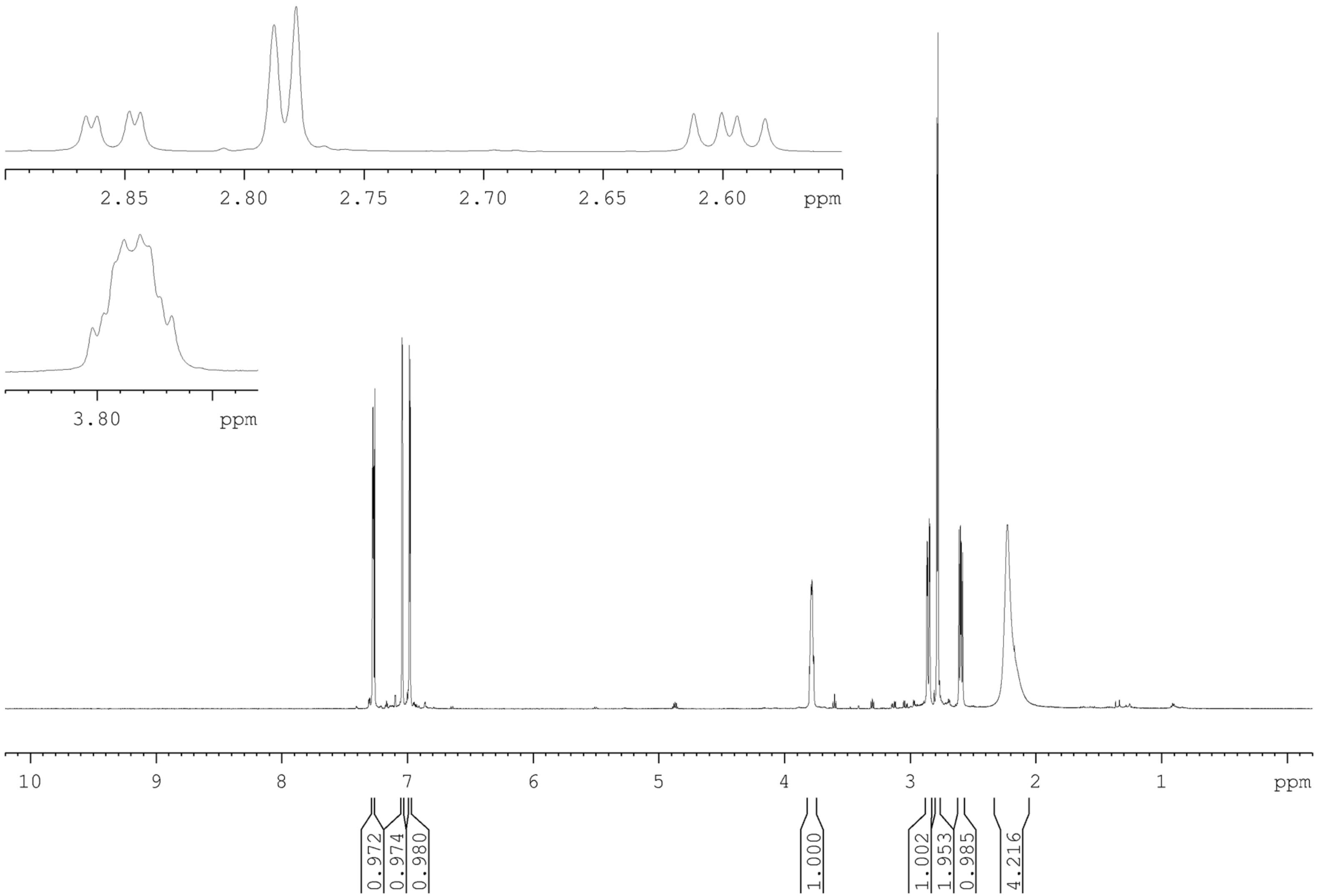

1-amino-3-(thiophen-3-yl)propan-2-ol (1w-I_2)

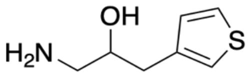

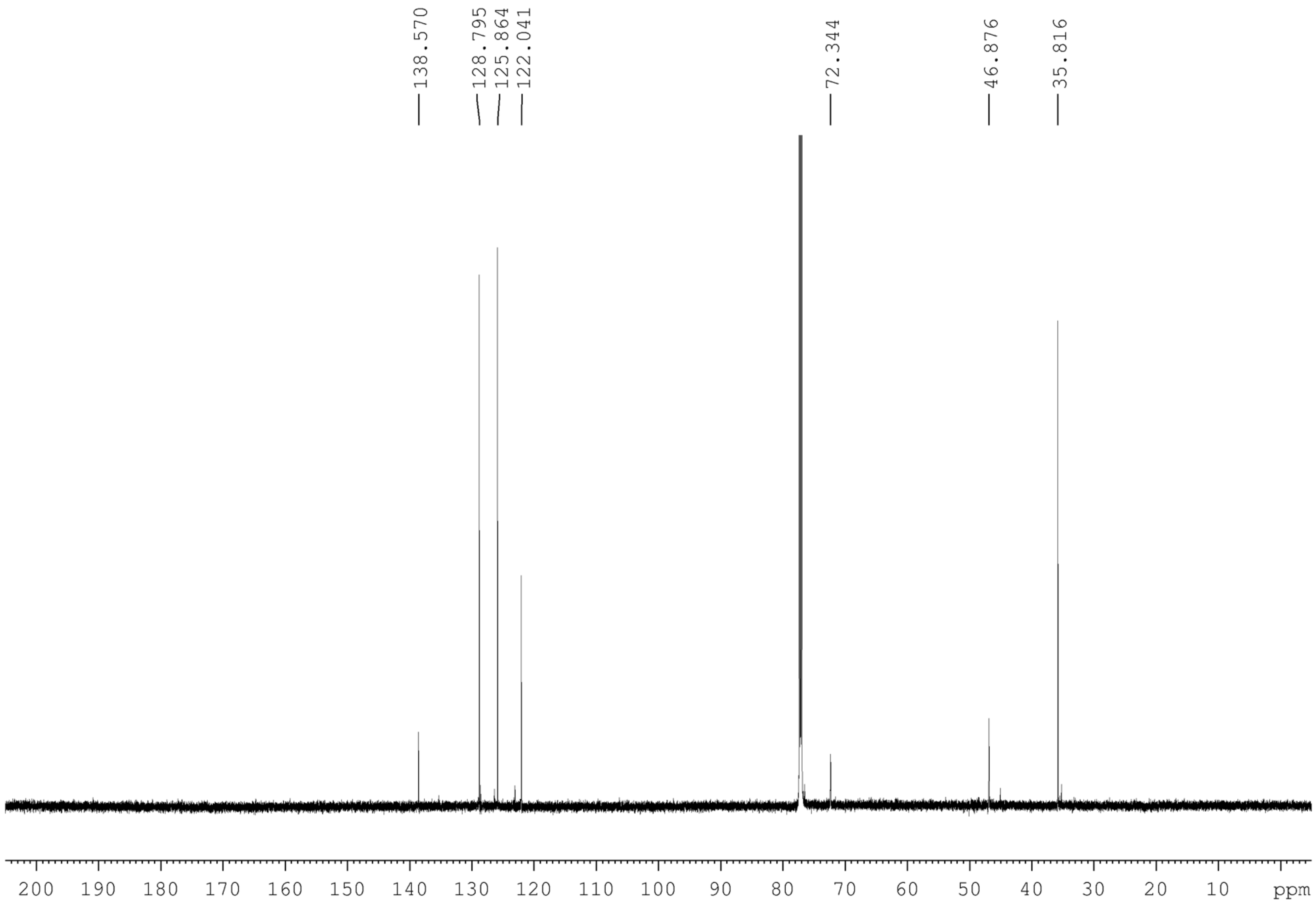

N-(2-hydroxy-3-(thiophen-3-yl)propyl)acetamide (1w-rac)

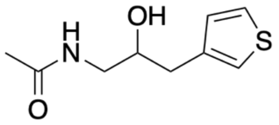

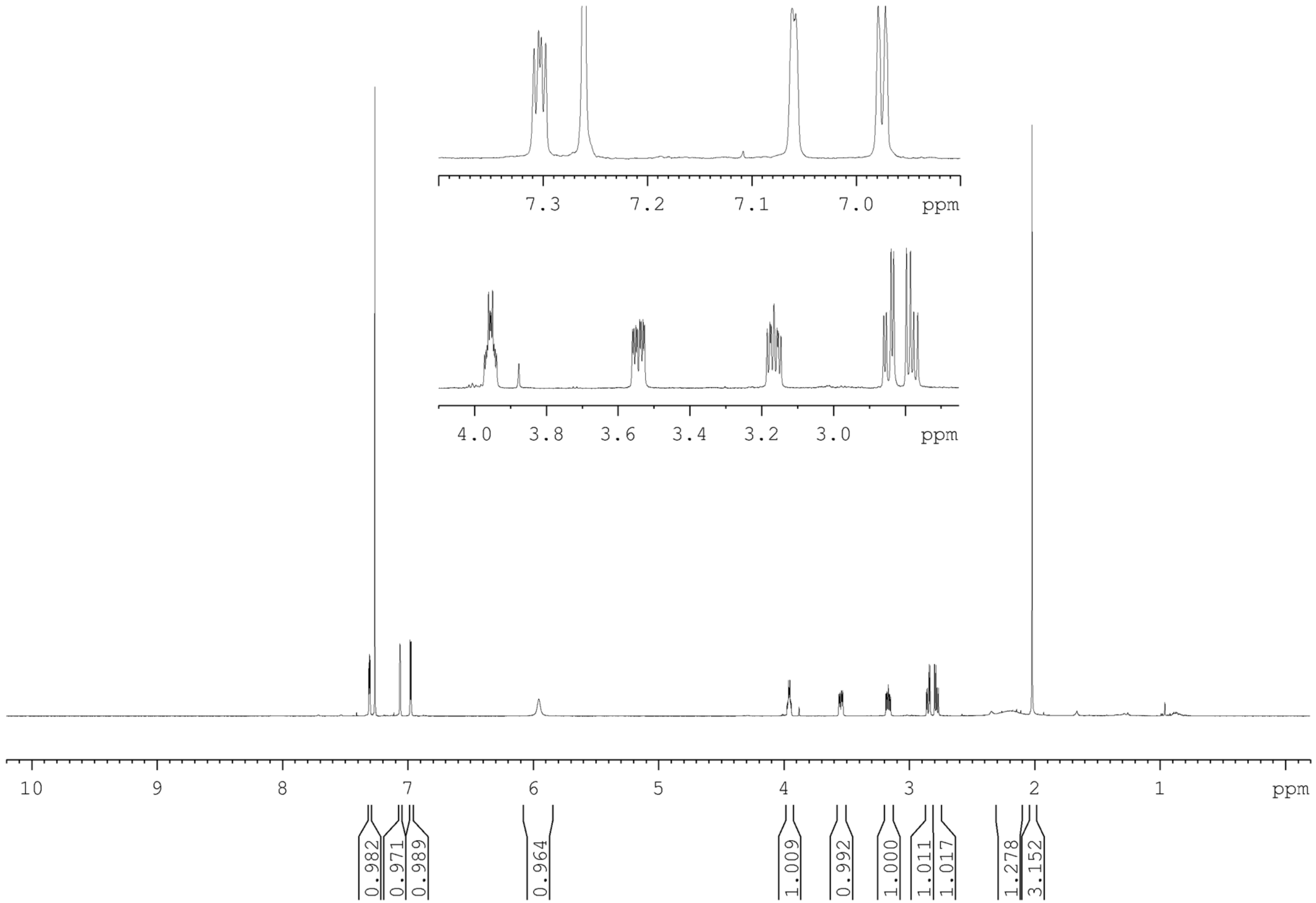

N-(2-hydroxy-3-(thiophen-3-yl)propyl)acetamide (1w-rac)

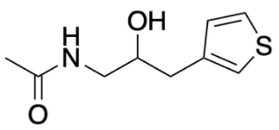

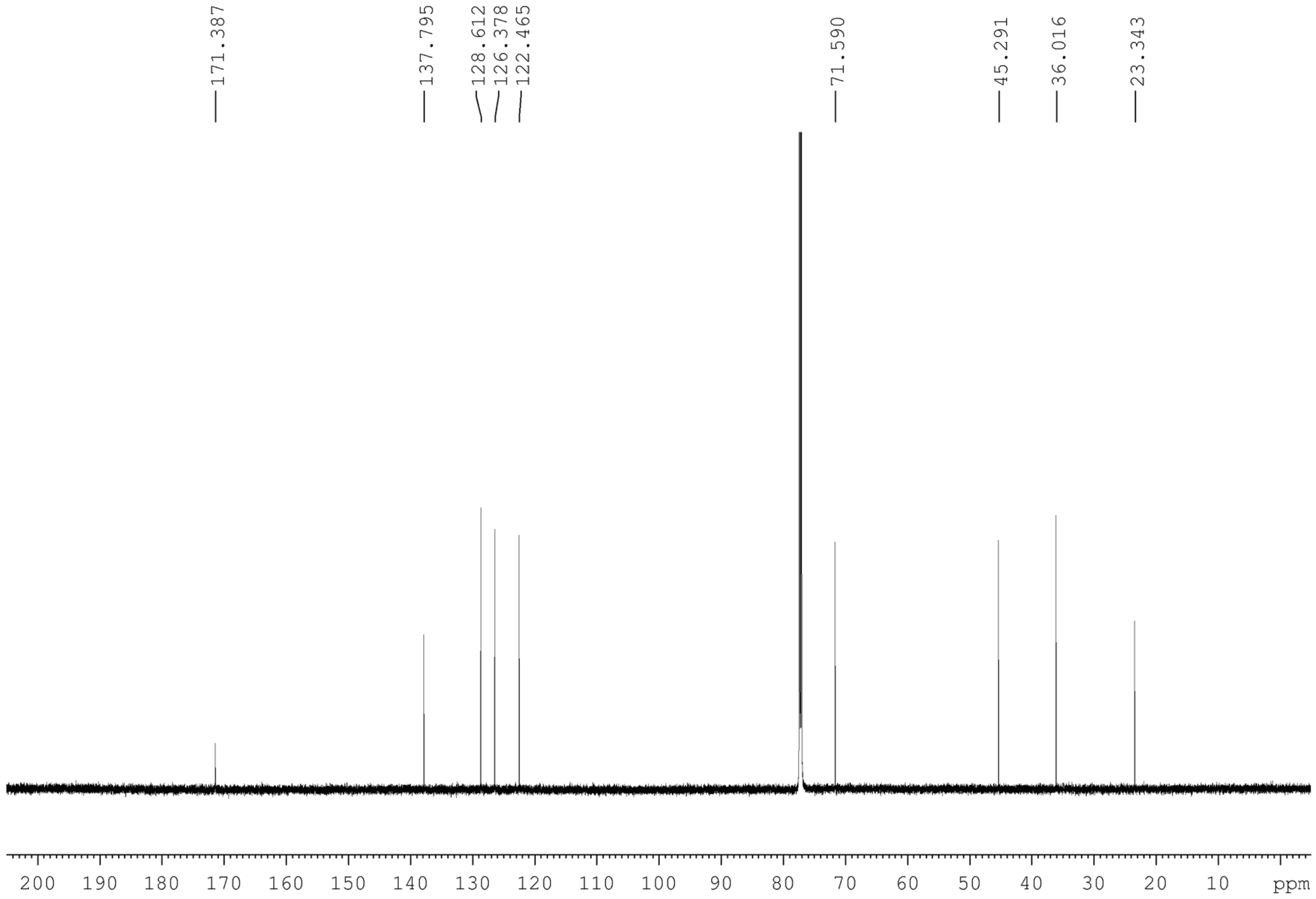

(*S*)-*N*-(2-hydroxy-3-(thiophen-3-yl)propyl)acetamide (1w)

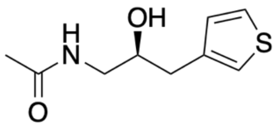

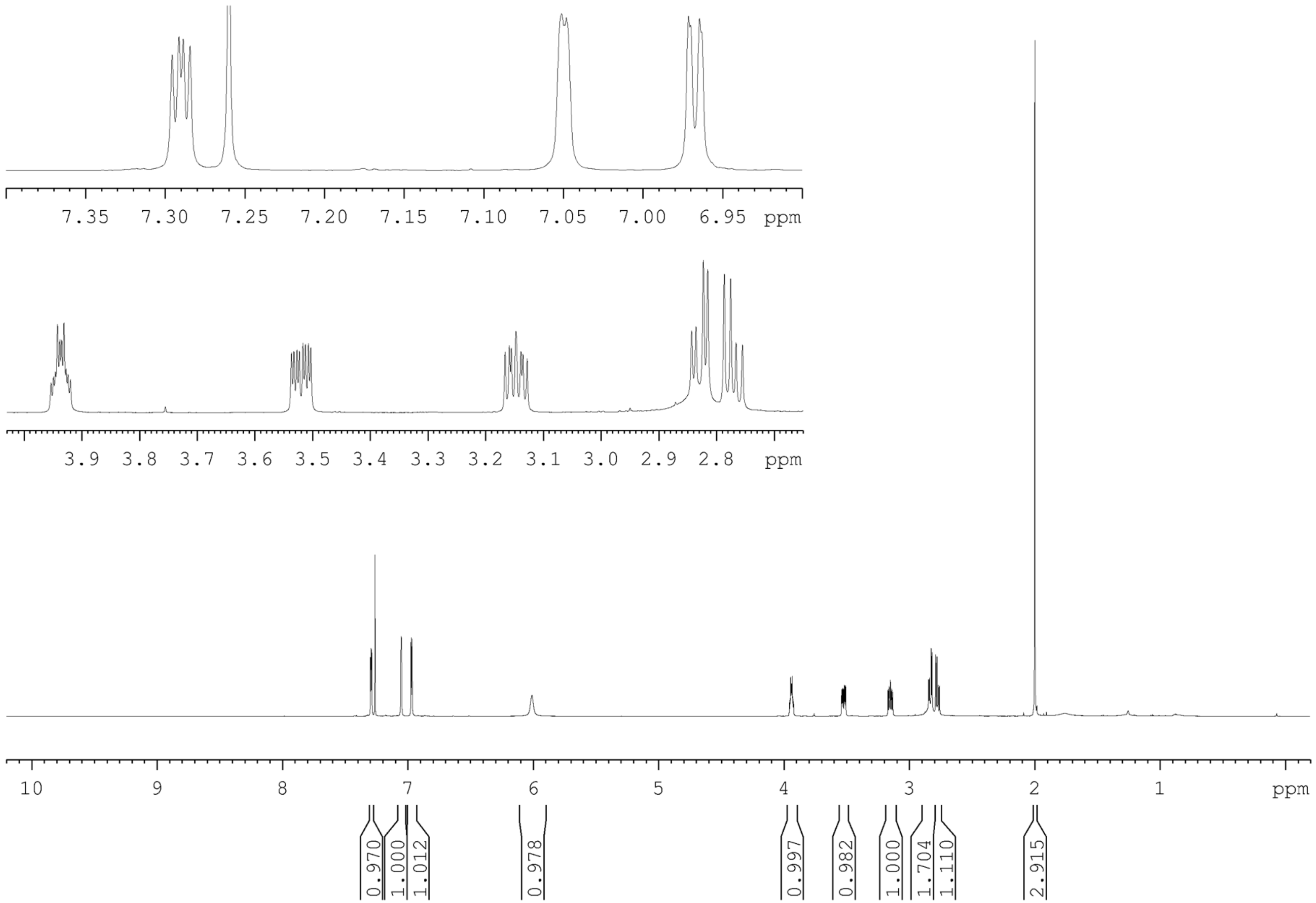

(*S*)-*N*-(2-hydroxy-3-(thiophen-3-yl)propyl)acetamide (1w)

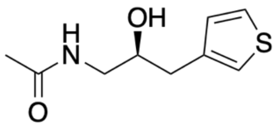

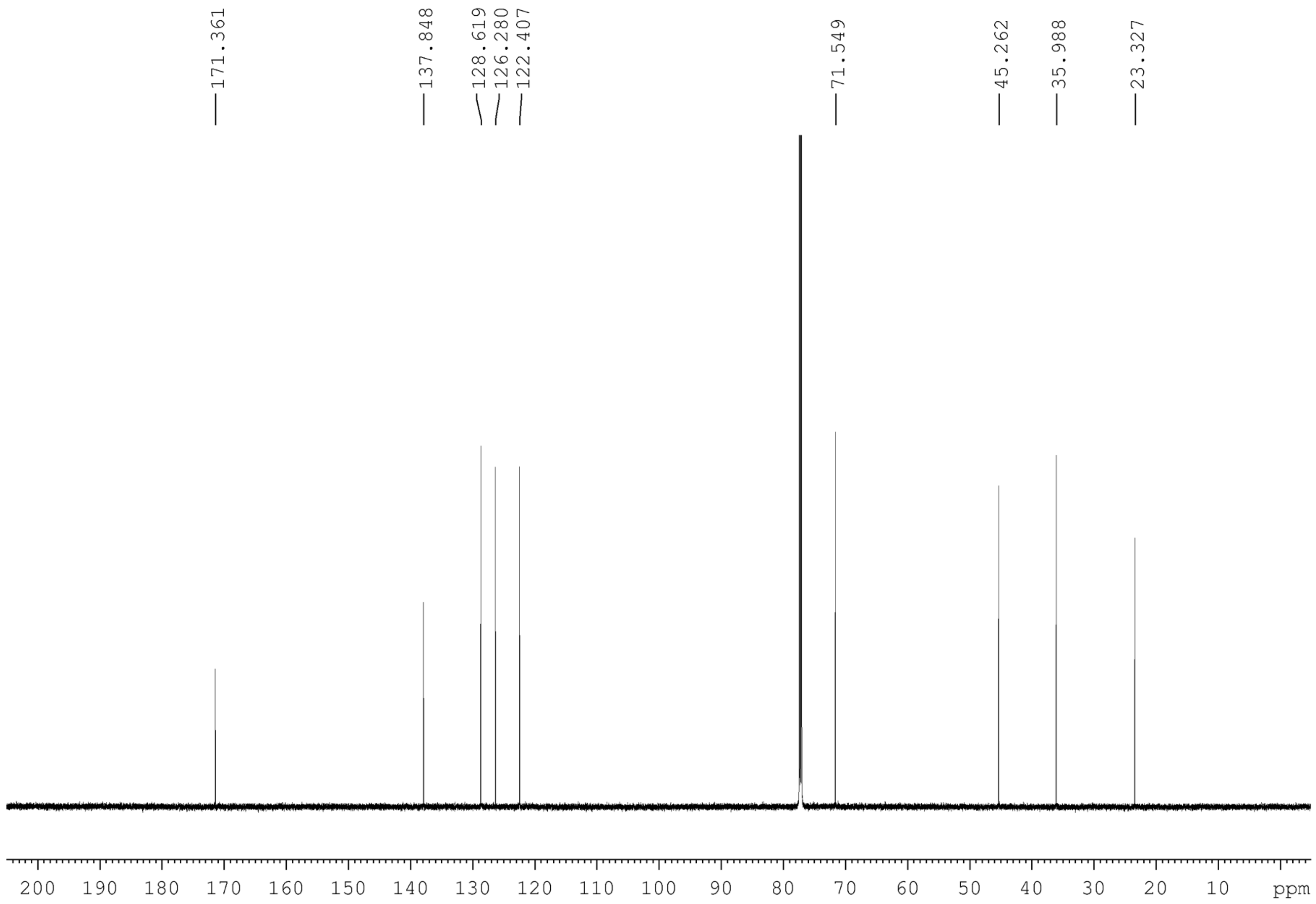

1-amino-4,4-dimethylpentan-2-ol (1x-I_2)

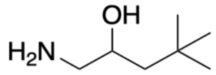

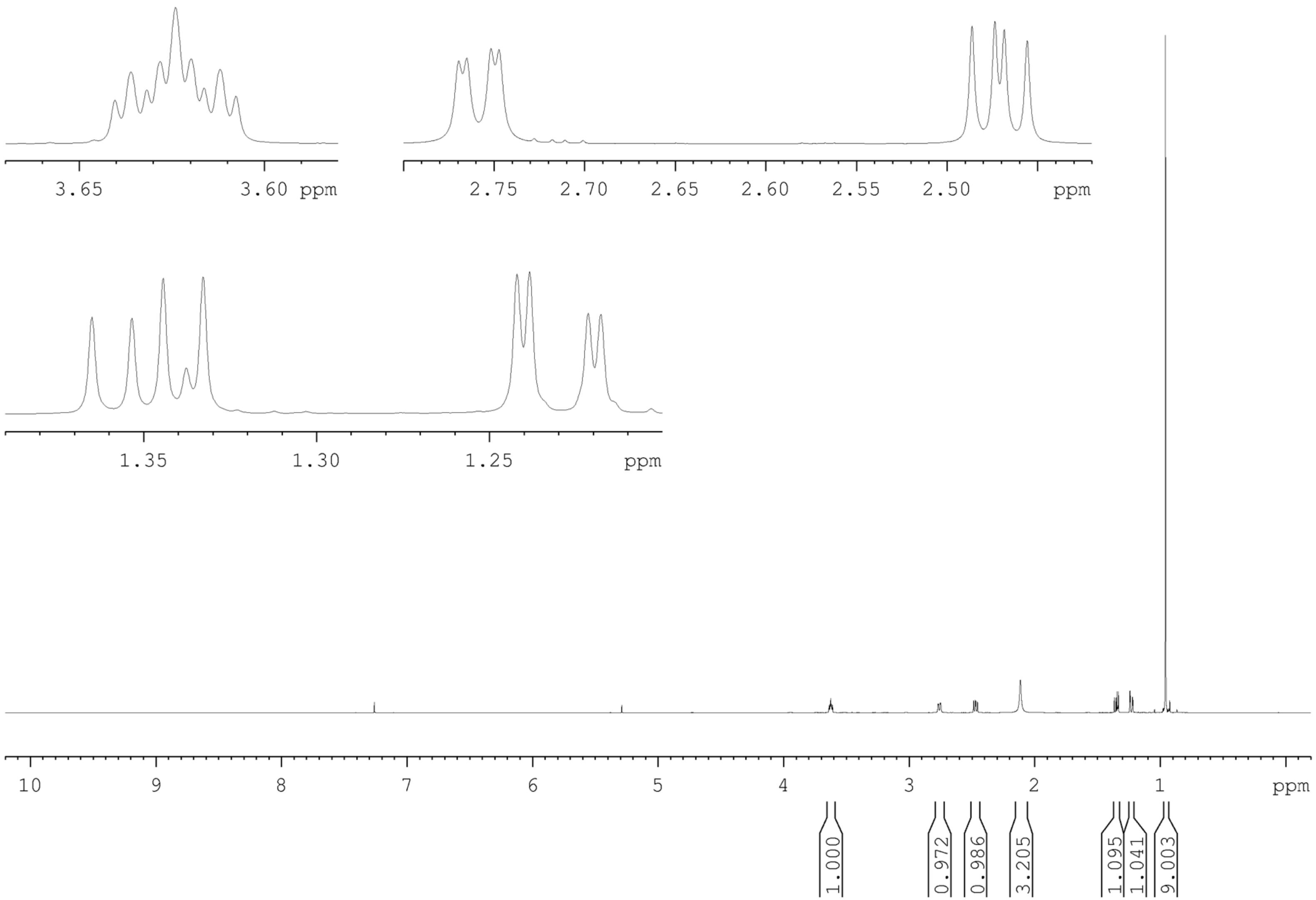

1-amino-4,4-dimethylpentan-2-ol (1x-I_2)

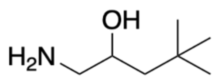

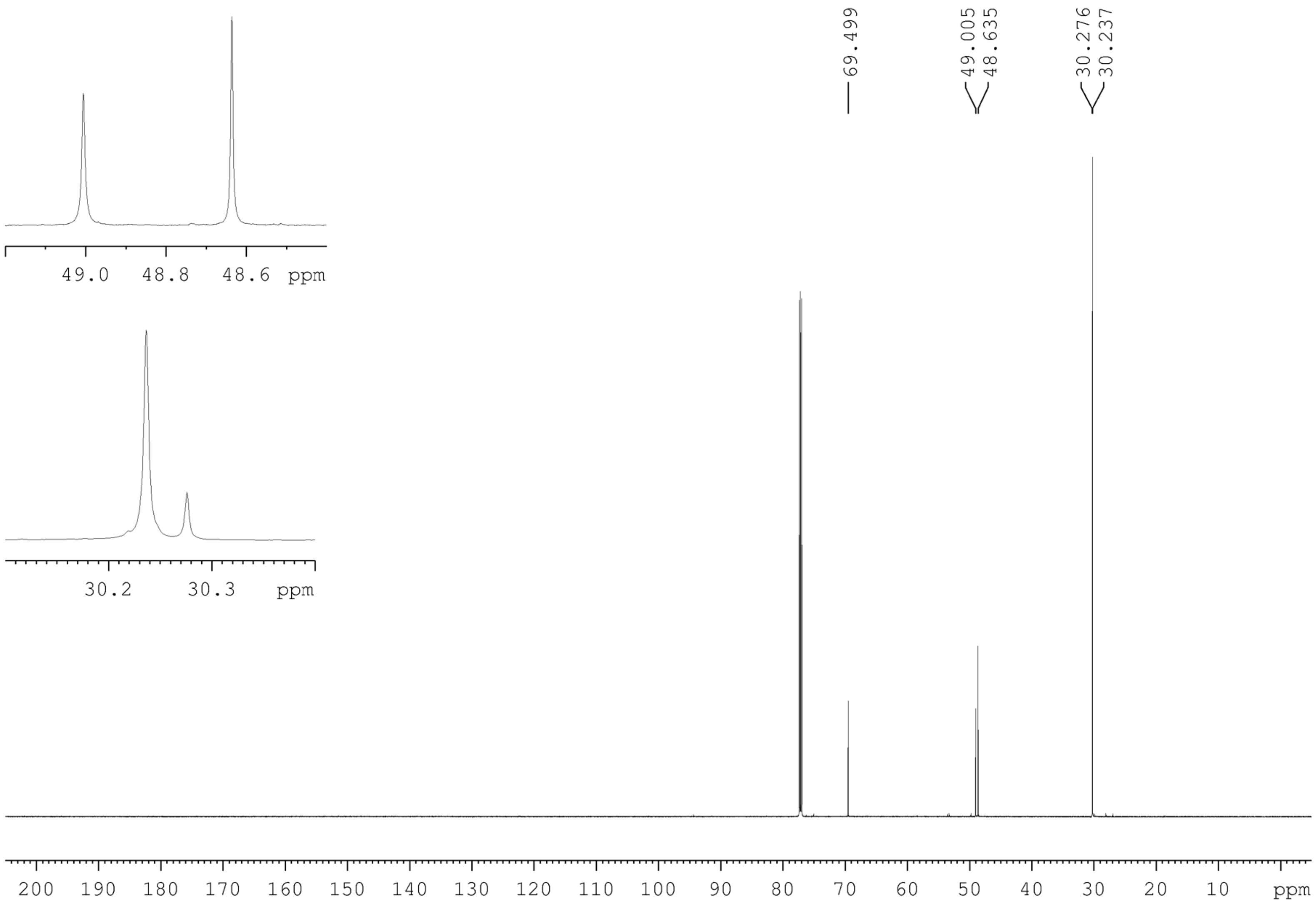

N-(2-hydroxy-4,4-dimethylpentyl)acetamide (1x-rac)

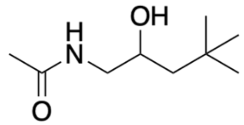

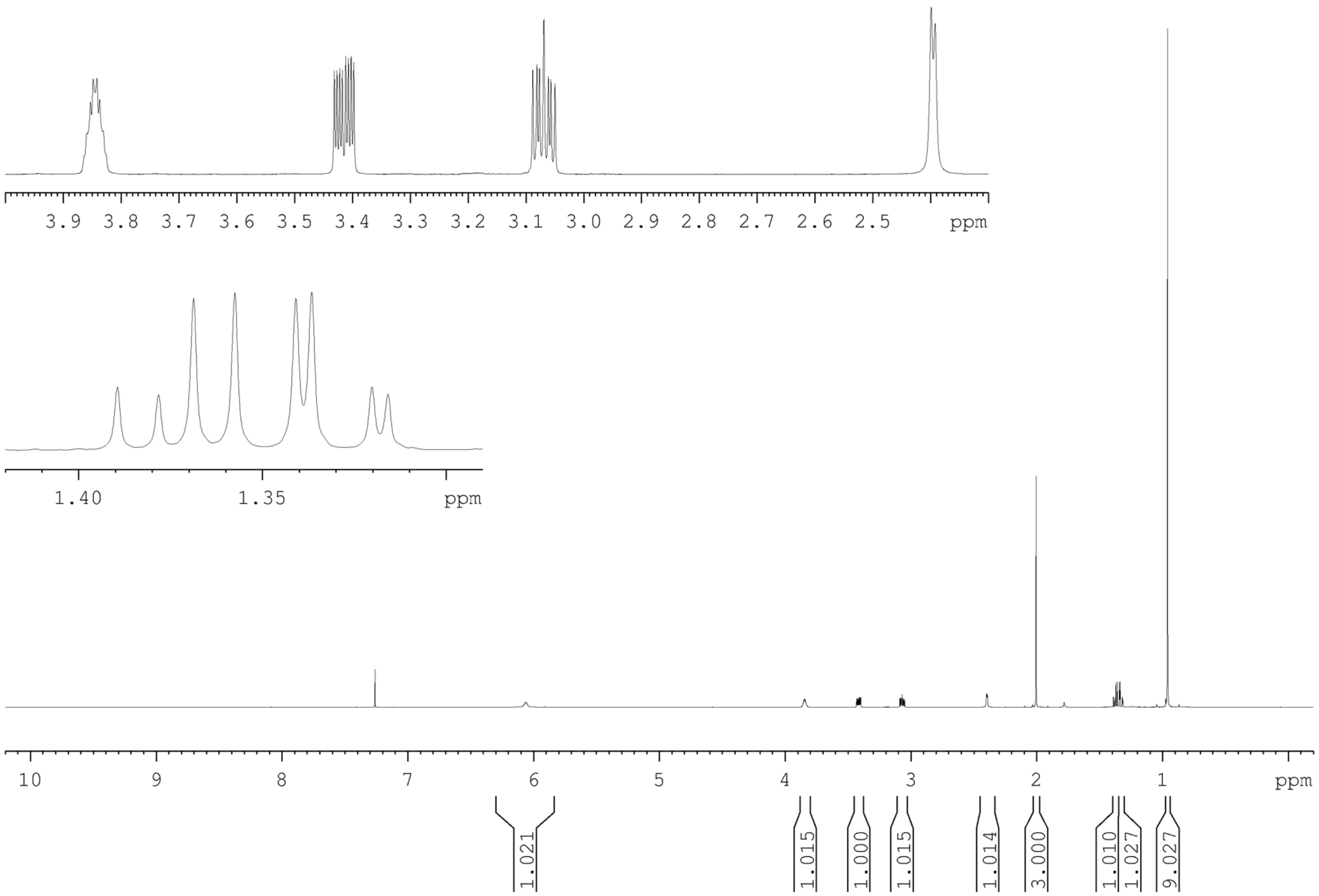

N-(2-hydroxy-4,4-dimethylpentyl)acetamide (1x-rac)

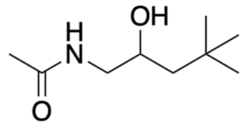

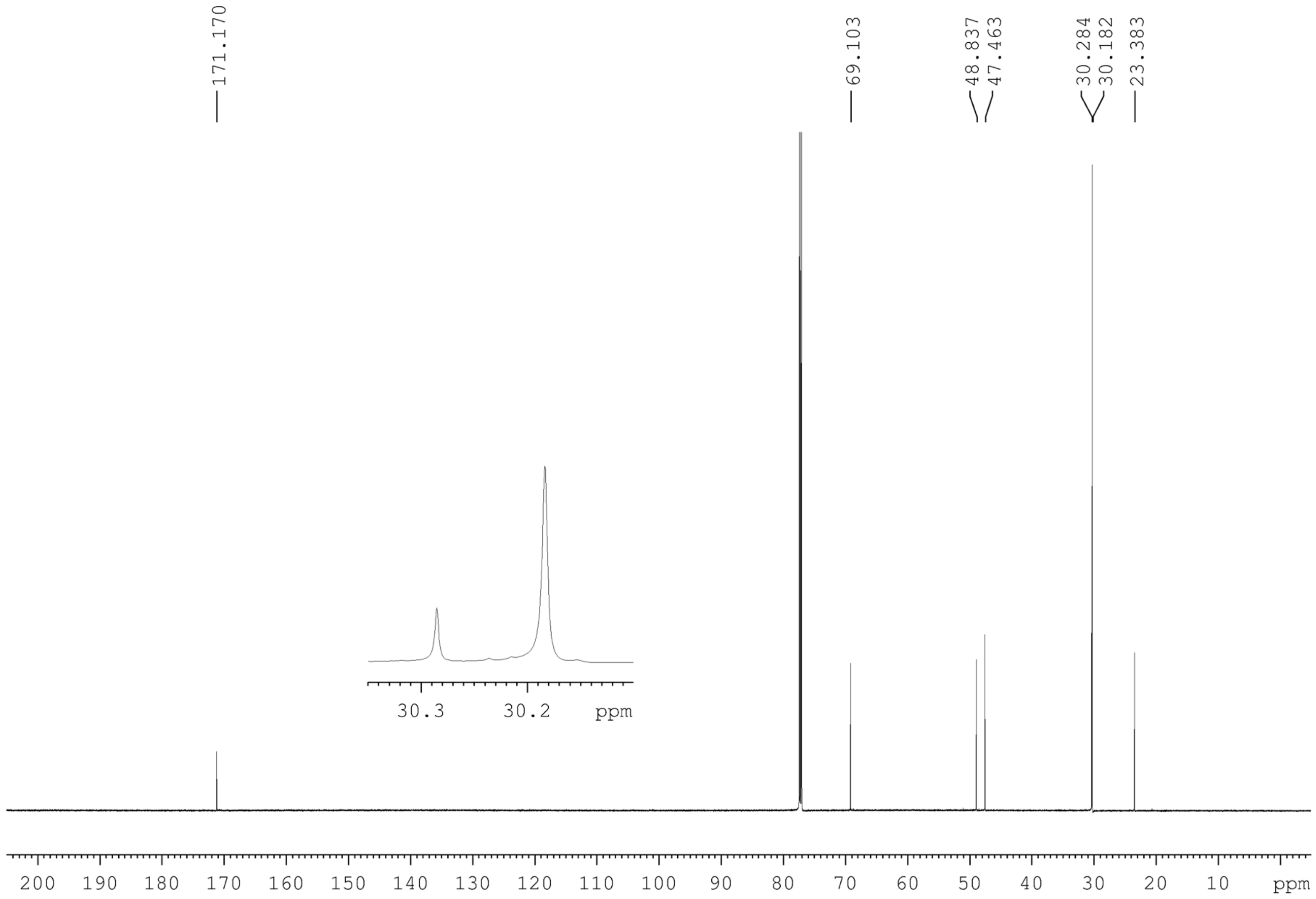

(*S*)-*N*-(2-hydroxy-4,4-dimethylpentyl)acetamide (1x)

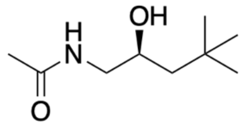

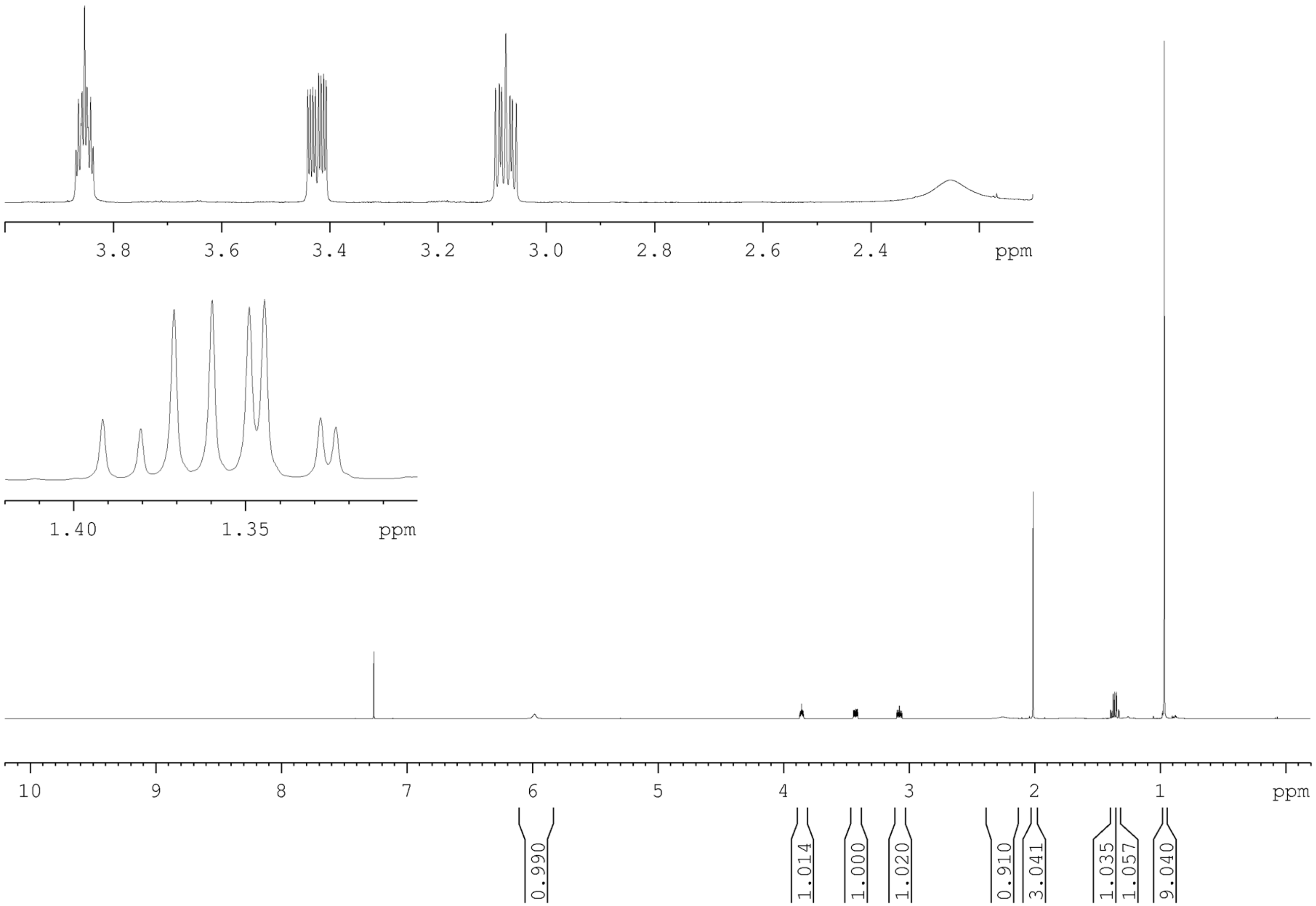

(*S*)-*N*-(2-hydroxy-4,4-dimethylpentyl)acetamide (1x)

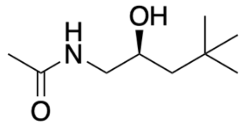

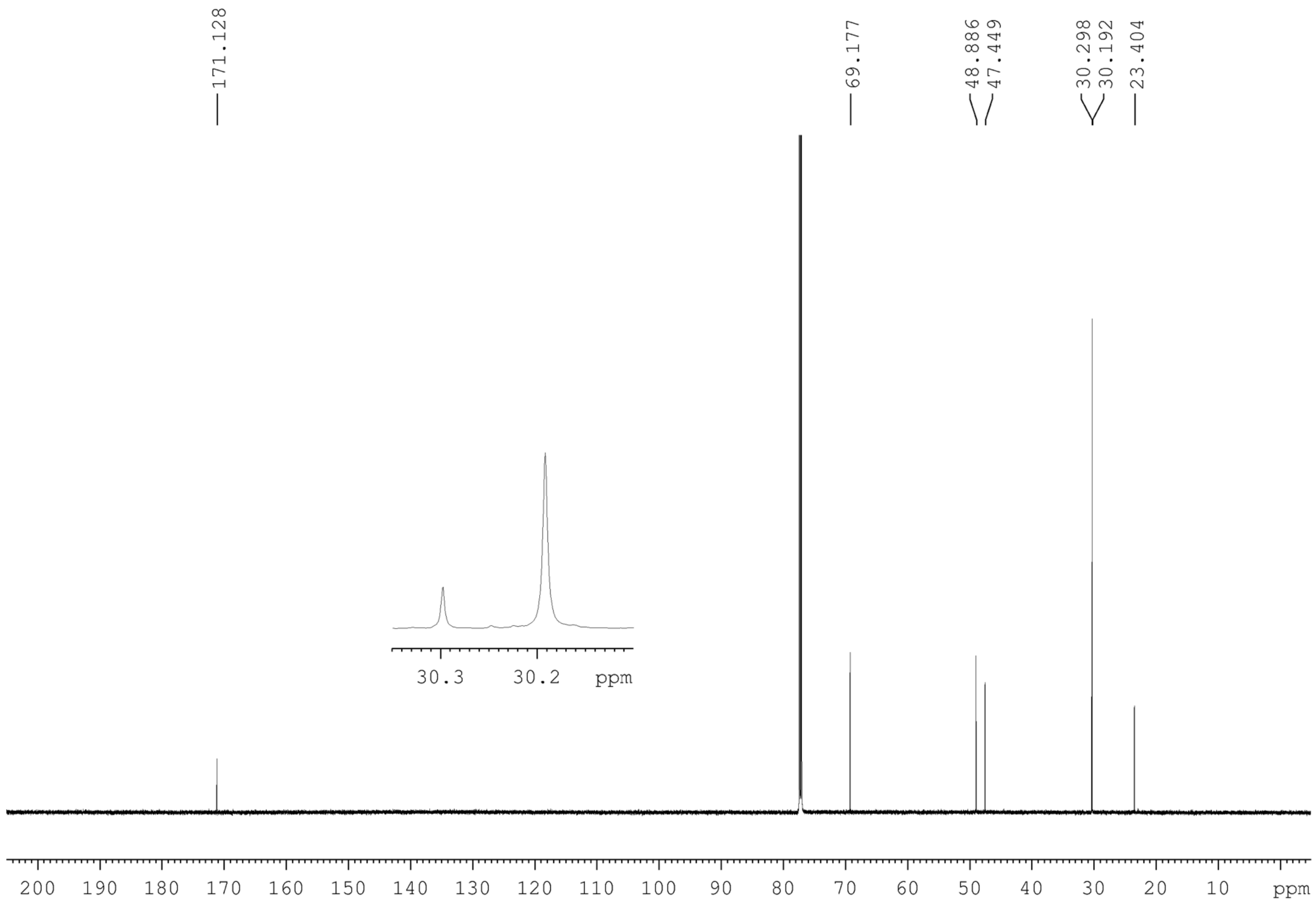

(*S*)-1-(*N*-acetylbenzamido)-4,4-dimethylpentan-2-yl benzoate (1x-Bz)

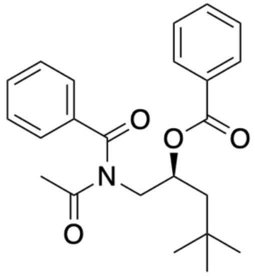

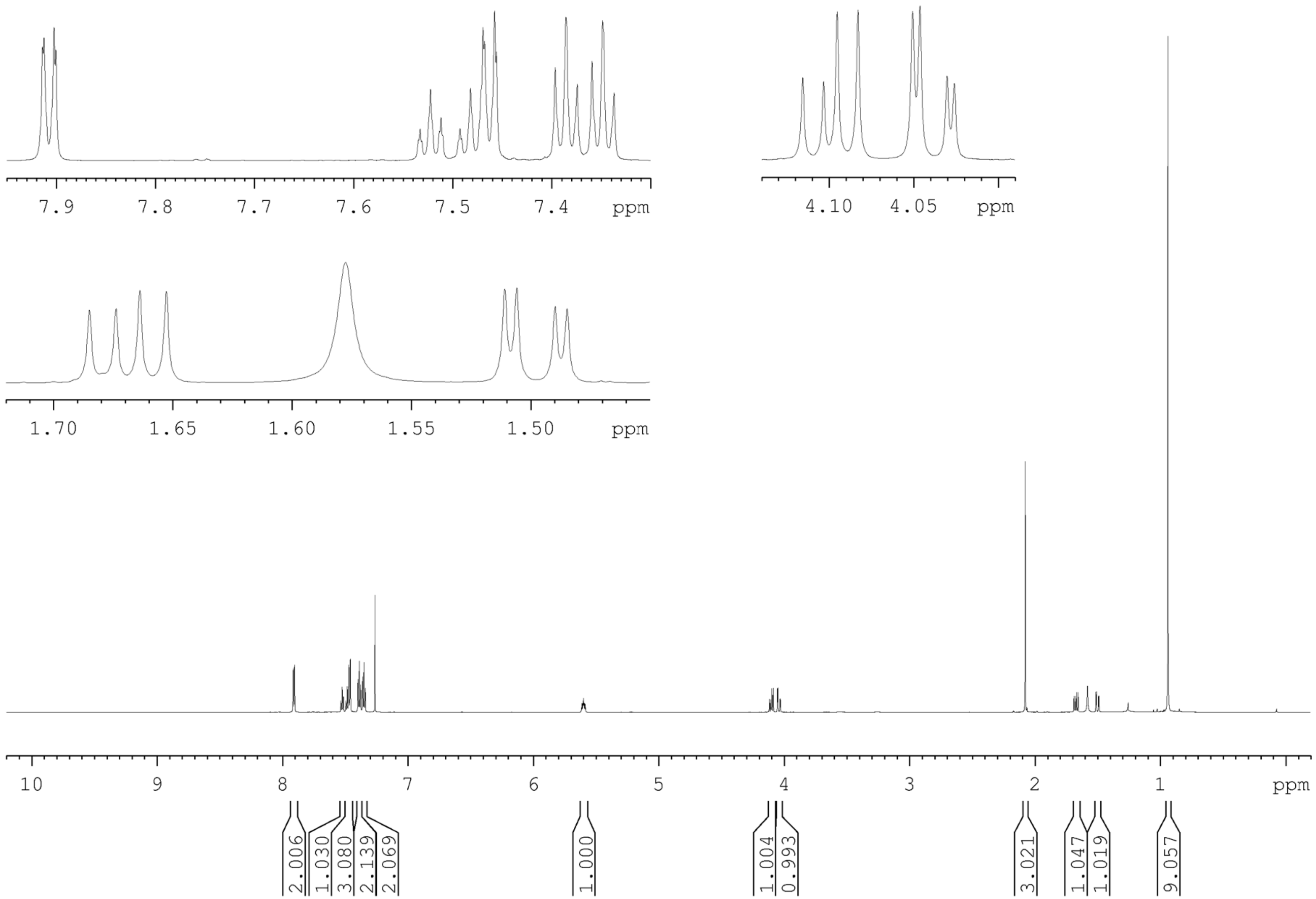

(*S*)-1-(*N*-acetylbenzamido)-4,4-dimethylpentan-2-yl benzoate (1x-Bz)

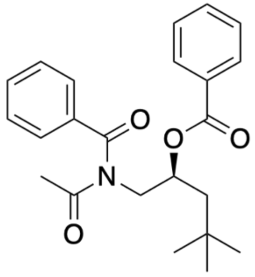

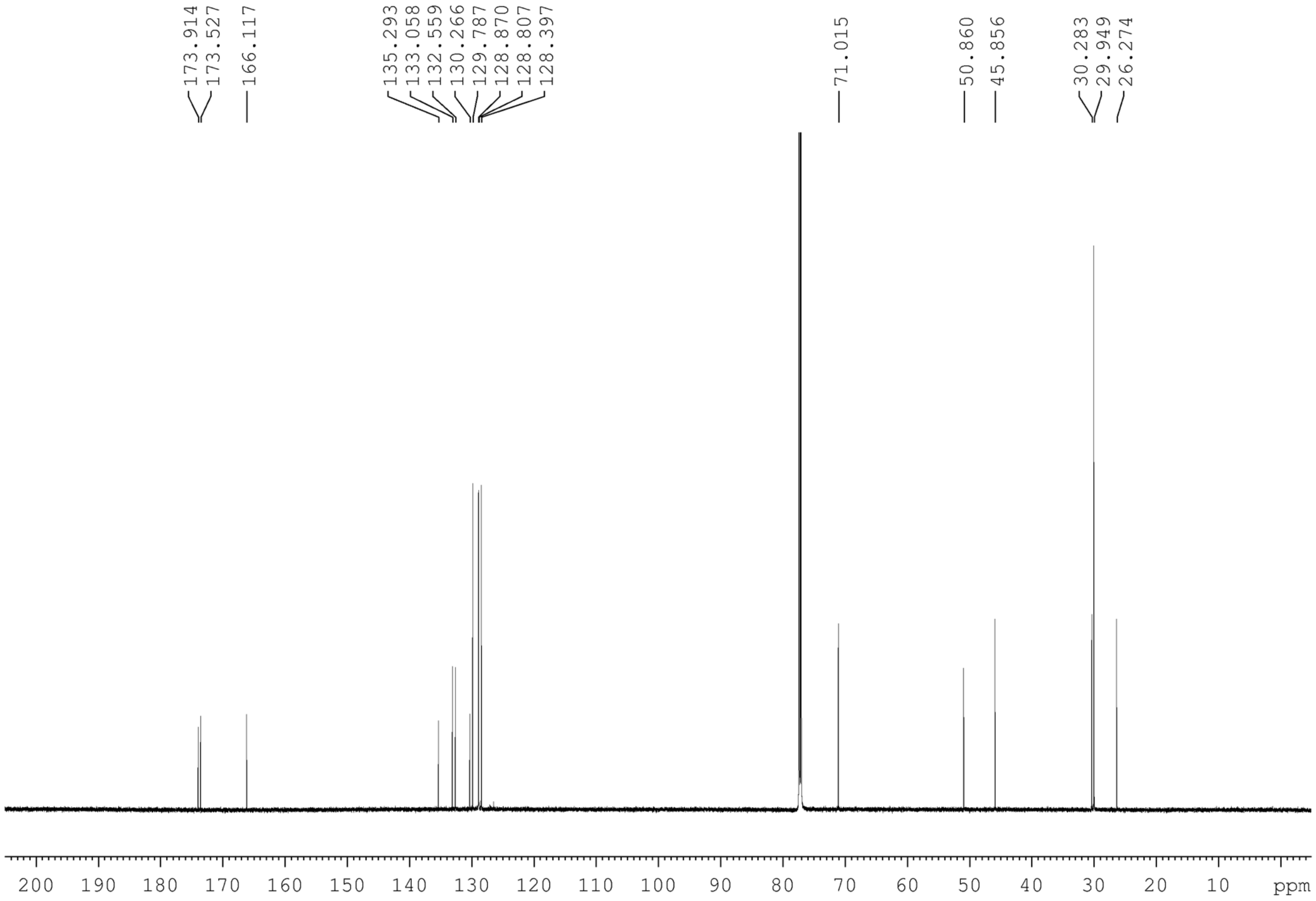

_N_-(2,5-dihydroxypentyl)acetamide (1y-rac)

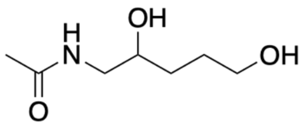

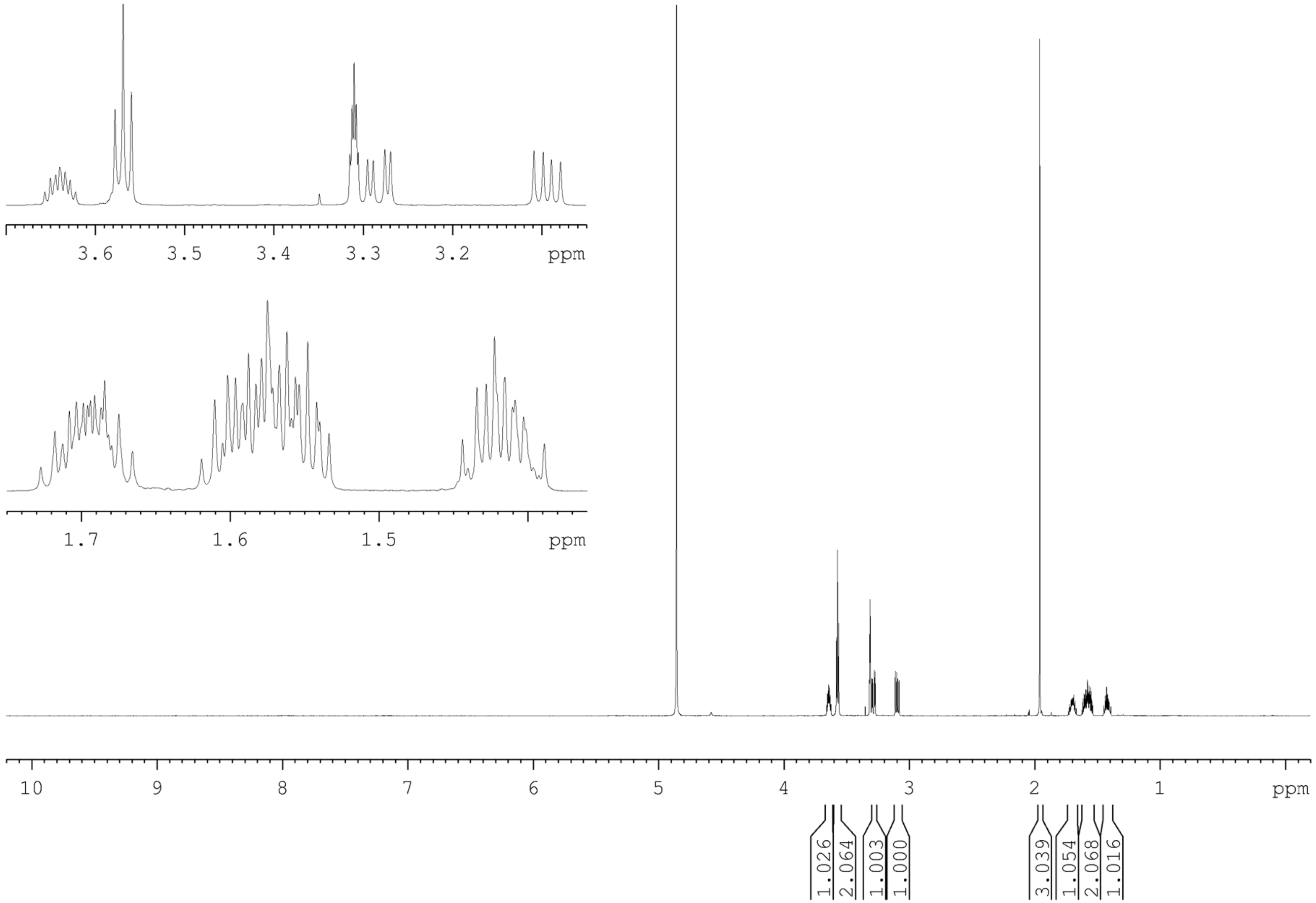

N-(2,5-dihydroxypentyl)acetamide (1y-rac)

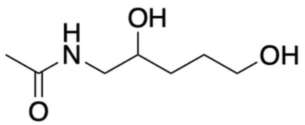

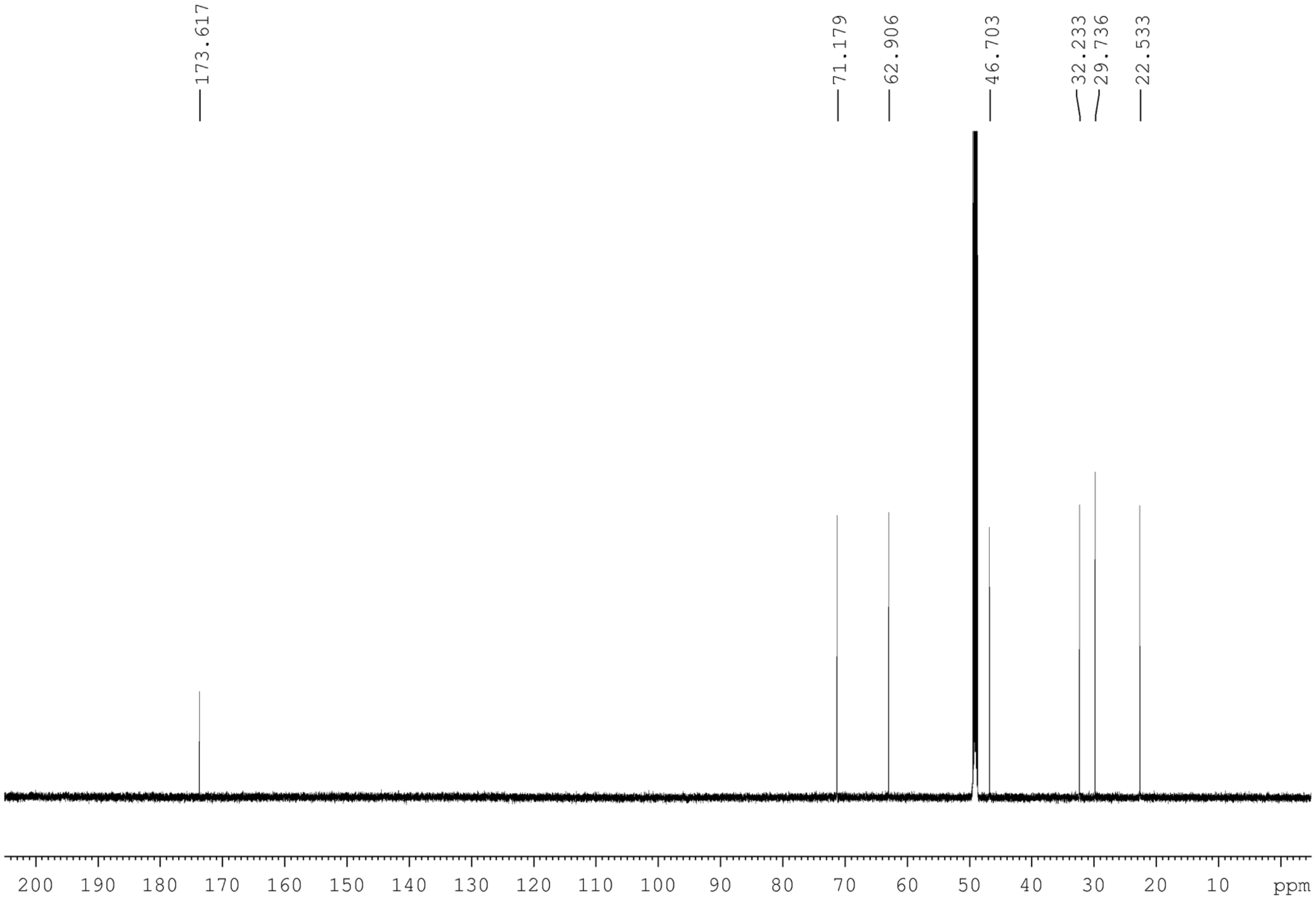

(*S*)-*N*-(2,5-dihydroxypentyl)acetamide (1y)

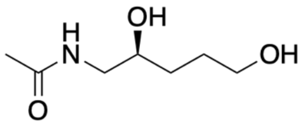

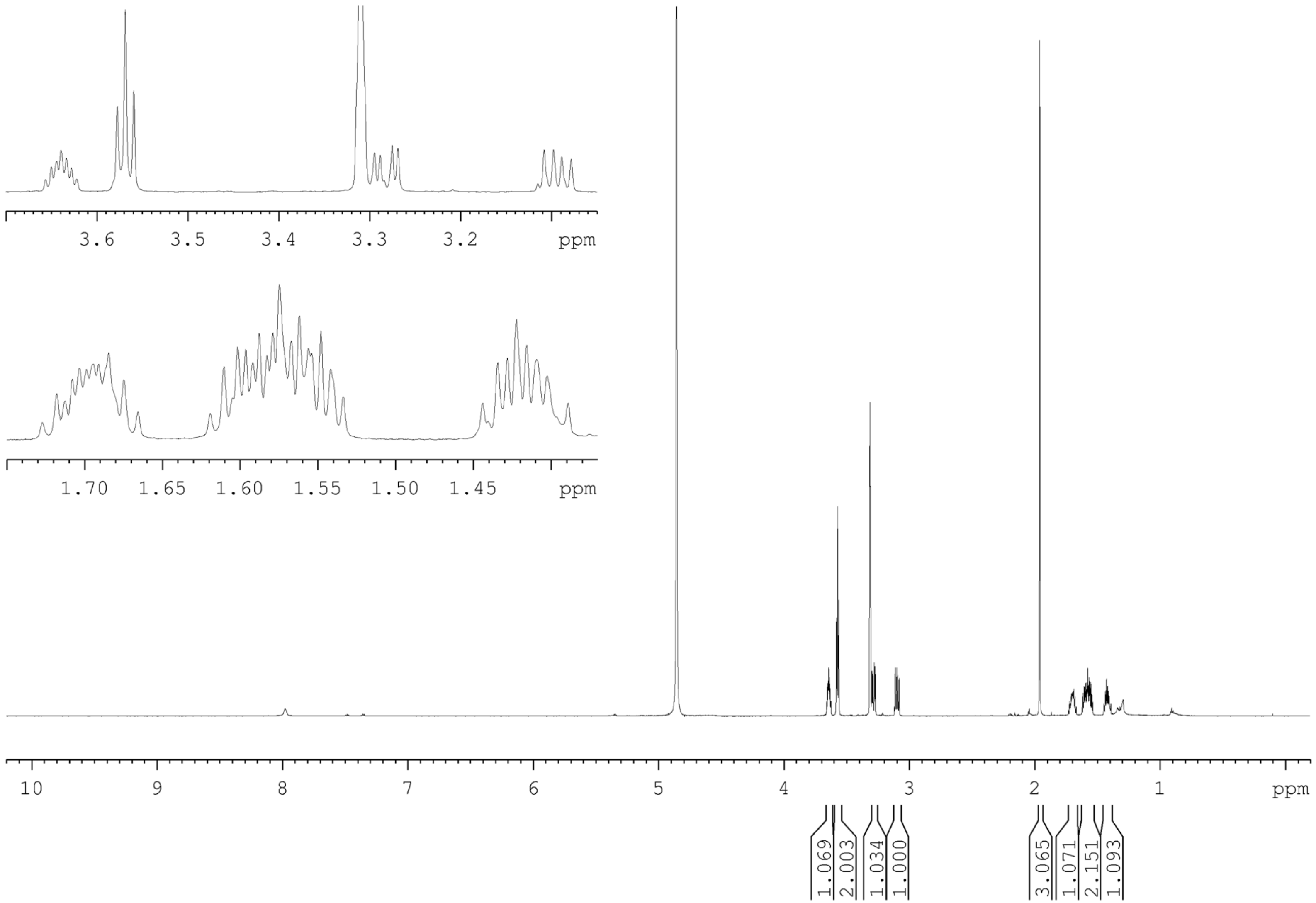

(*S*)-*N*-(2,5-dihydroxypentyl)acetamide (1y)

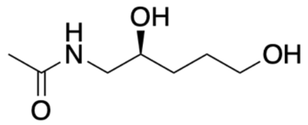

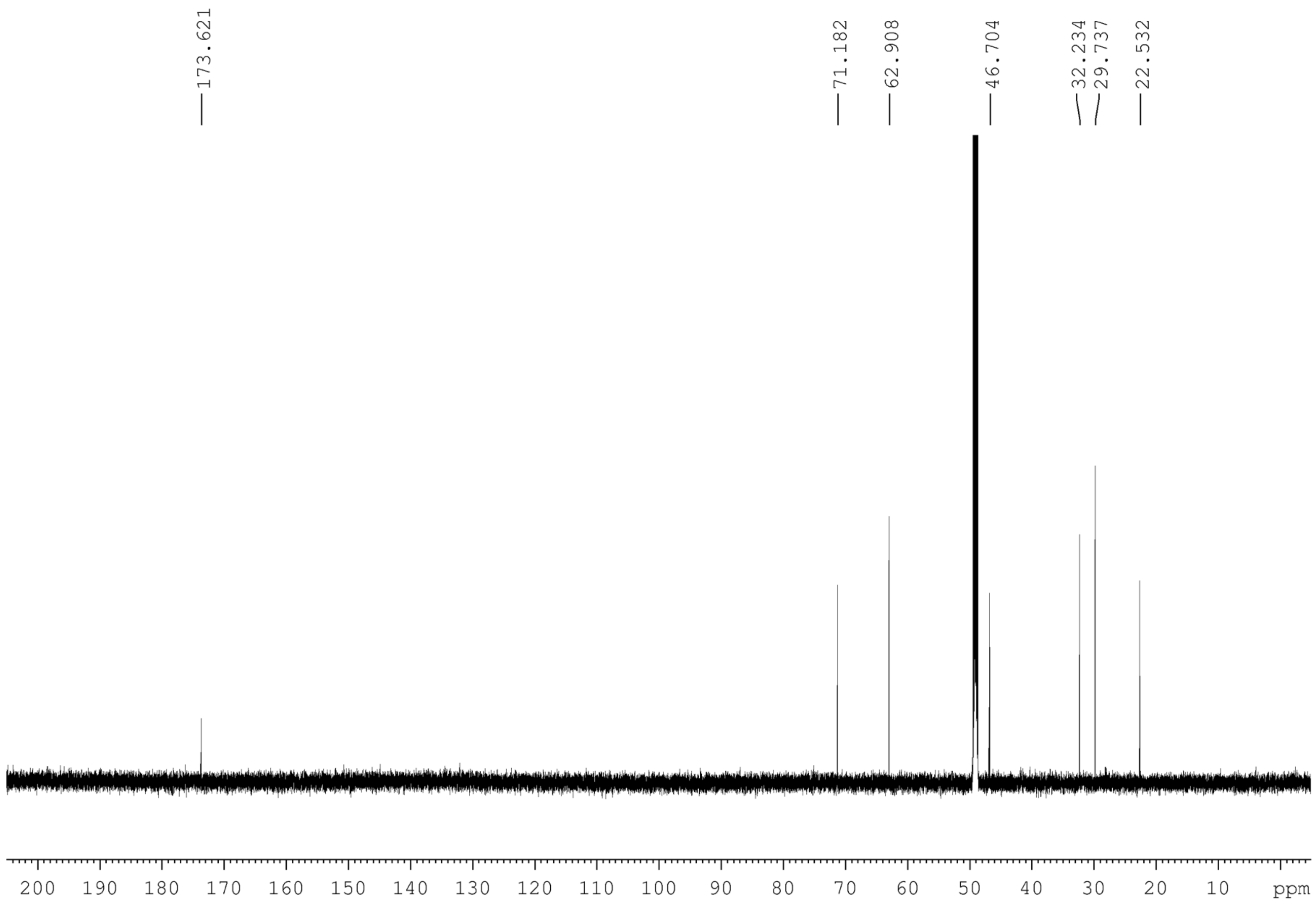

(*S*)-5-(*N*-acetylbenzamido)pentane-1,4-diyl dibenzoate (1y-Bz)

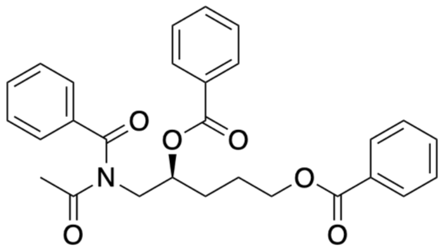

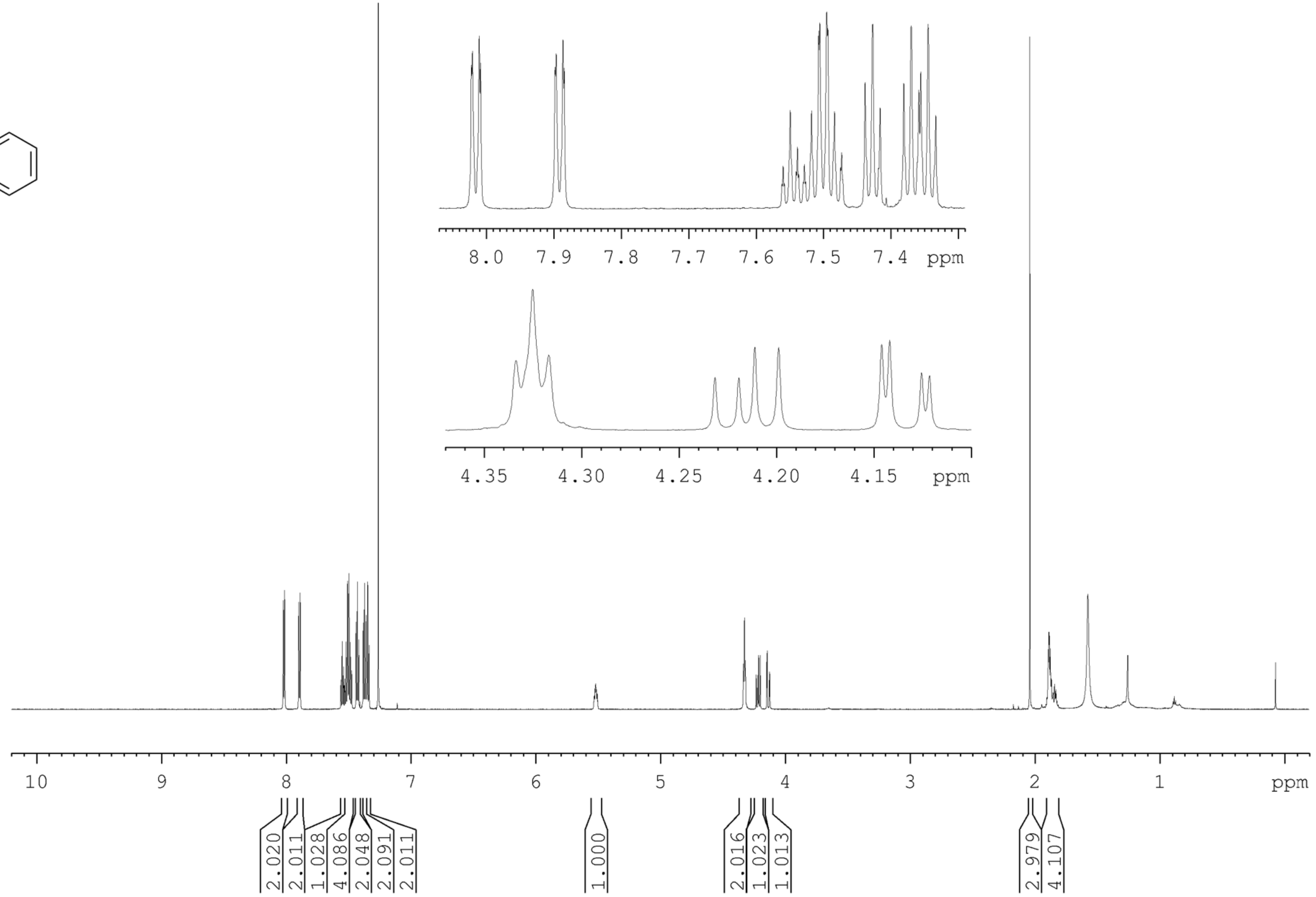

(*S*)-5-(*N*-acetylbenzamido)pentane-1,4-diyl dibenzoate (1y-Bz)

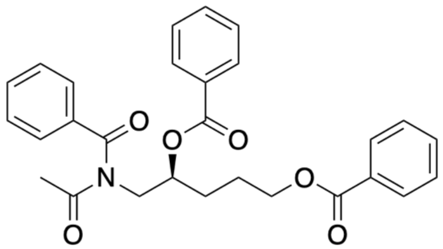

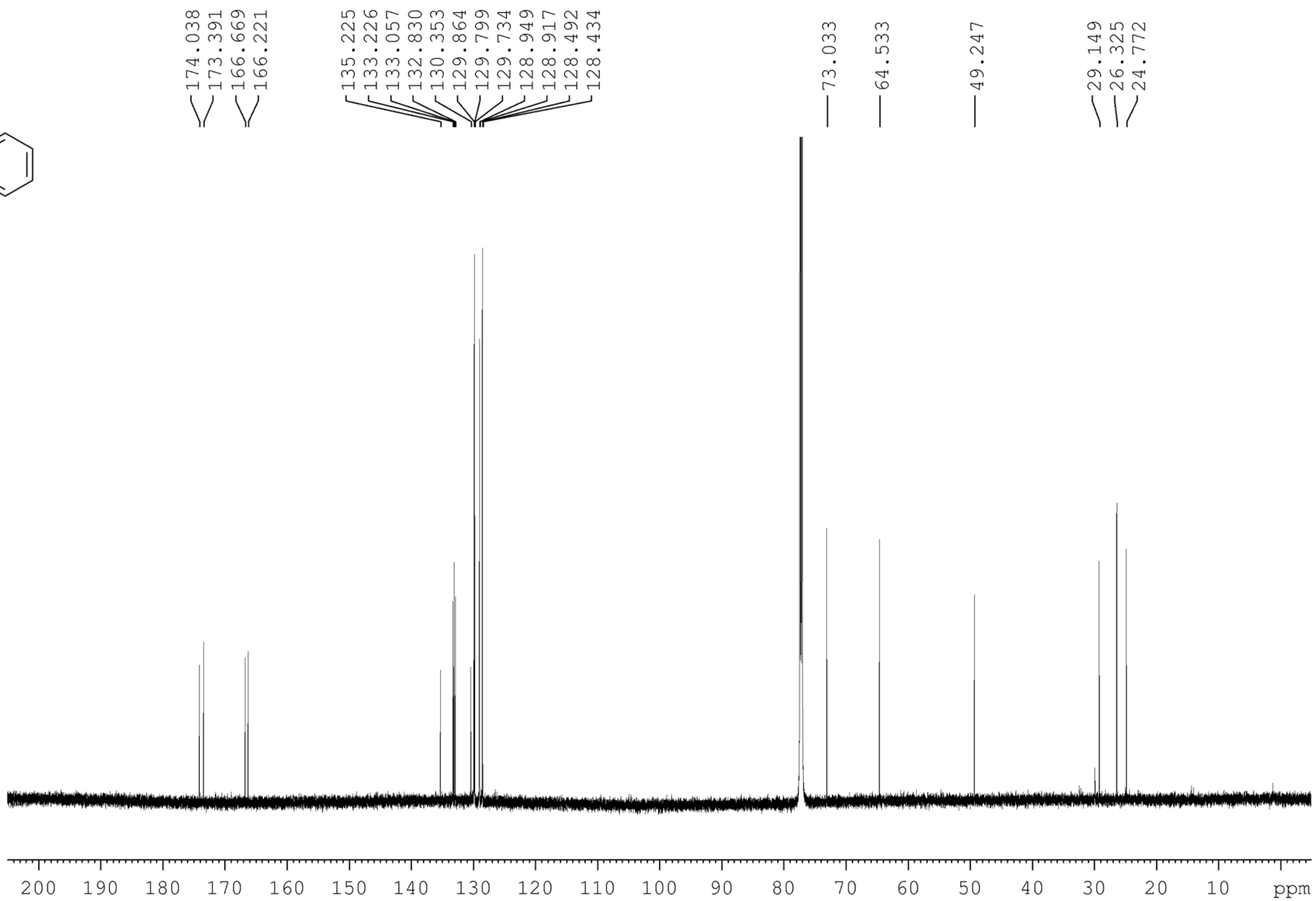

N-(2-hydroxybutyl)isobutyramide (1z-rac)

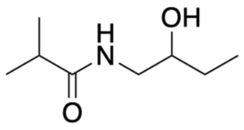

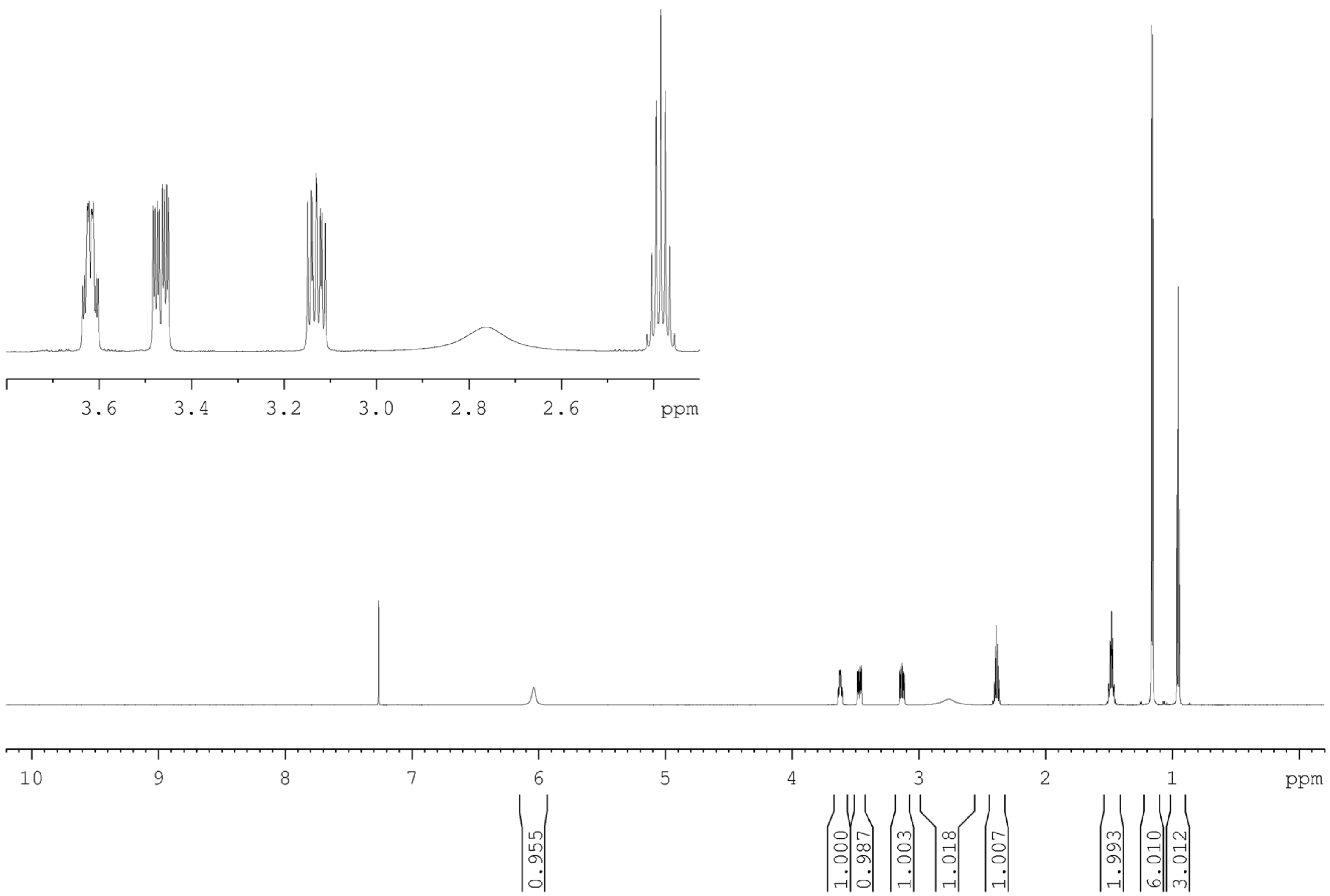

N-(2-hydroxybutyl)isobutyramide (1z-rac)

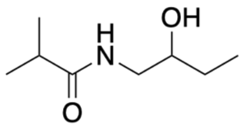

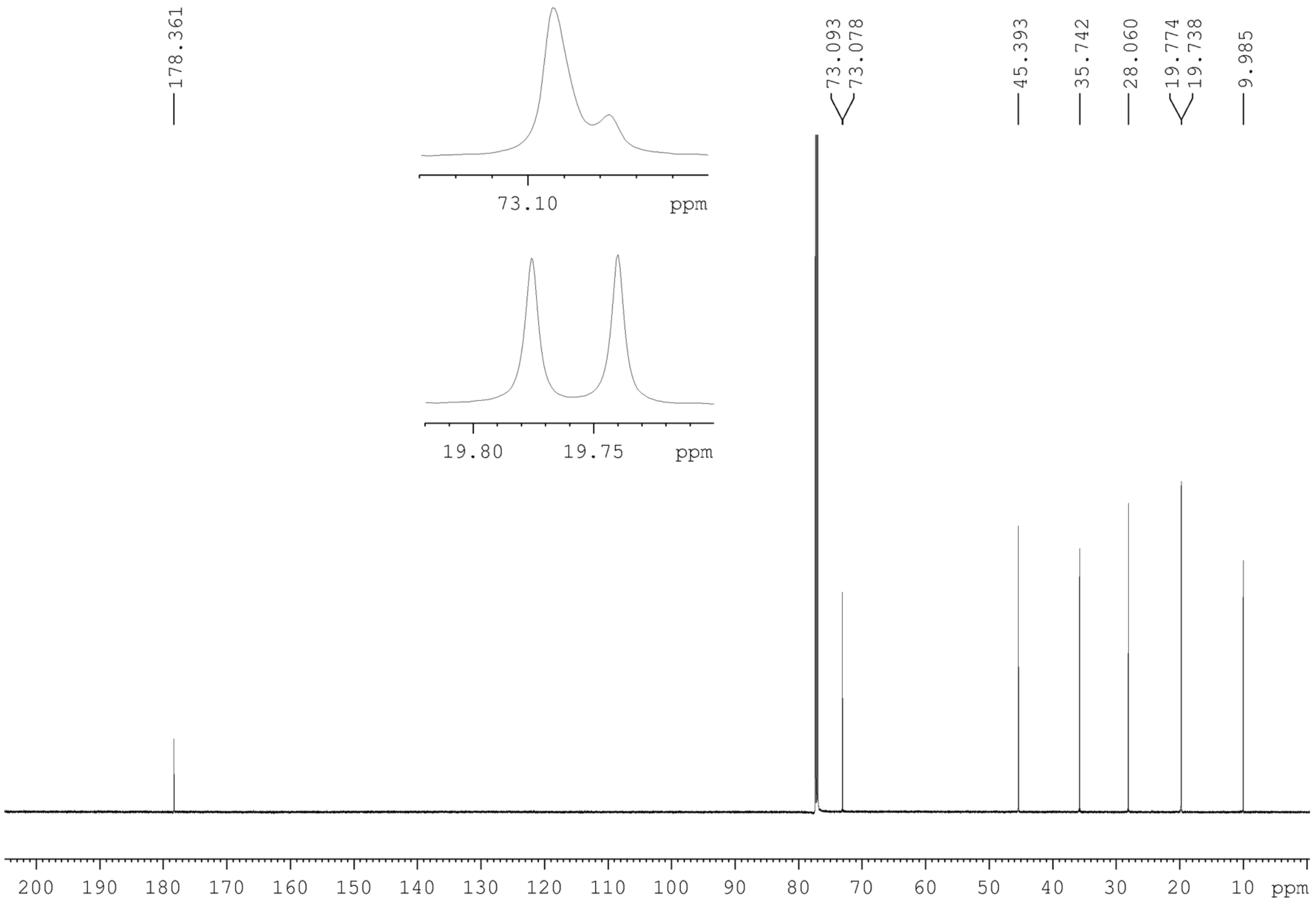

(*S*)-*N*-(2-hydroxybutyl)isobutyramide (1z)

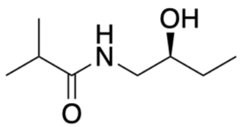

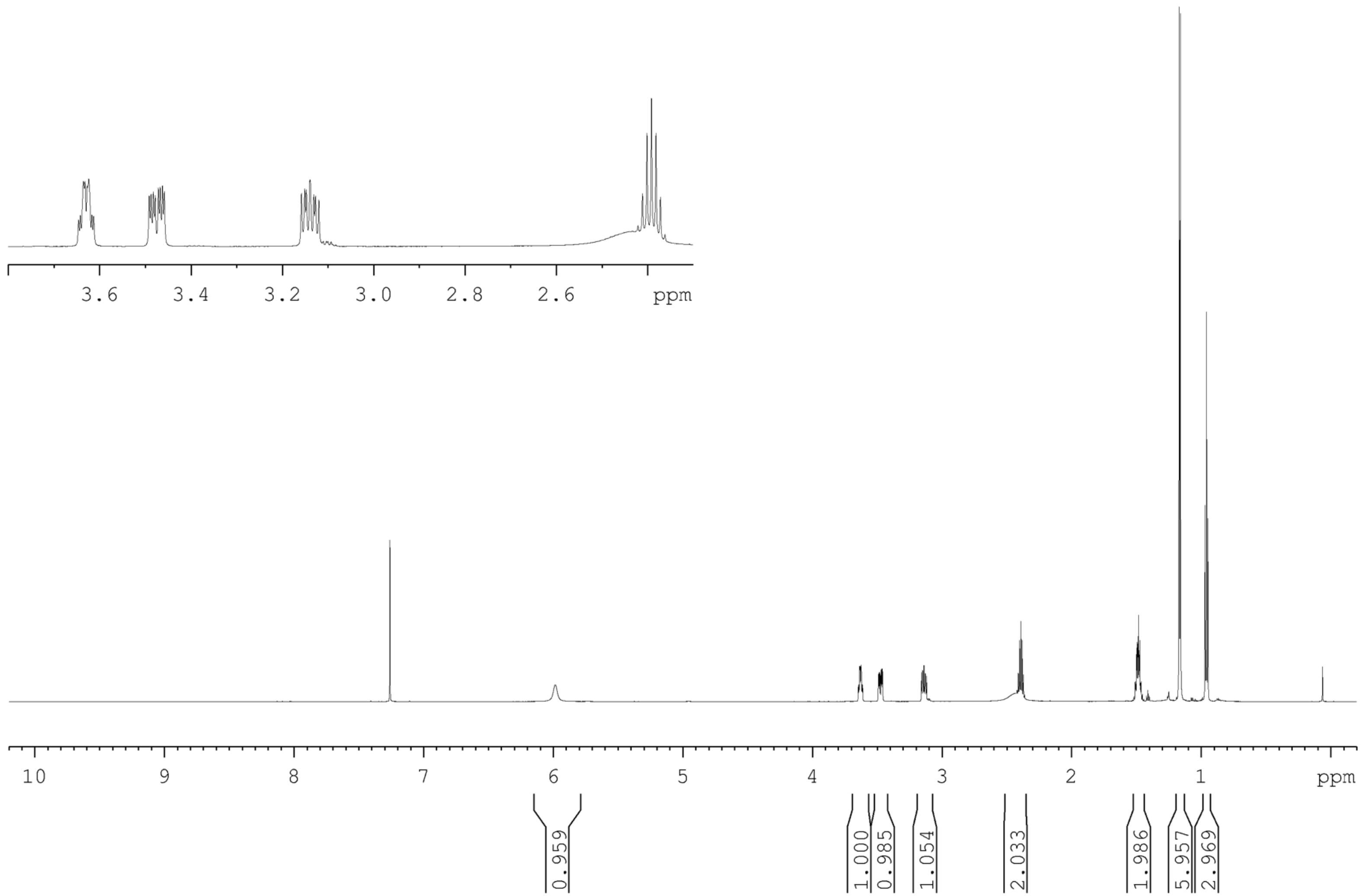

(*S*)-*N*-(2-hydroxybutyl)isobutyramide (1z)

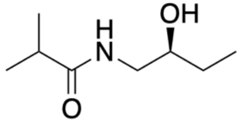

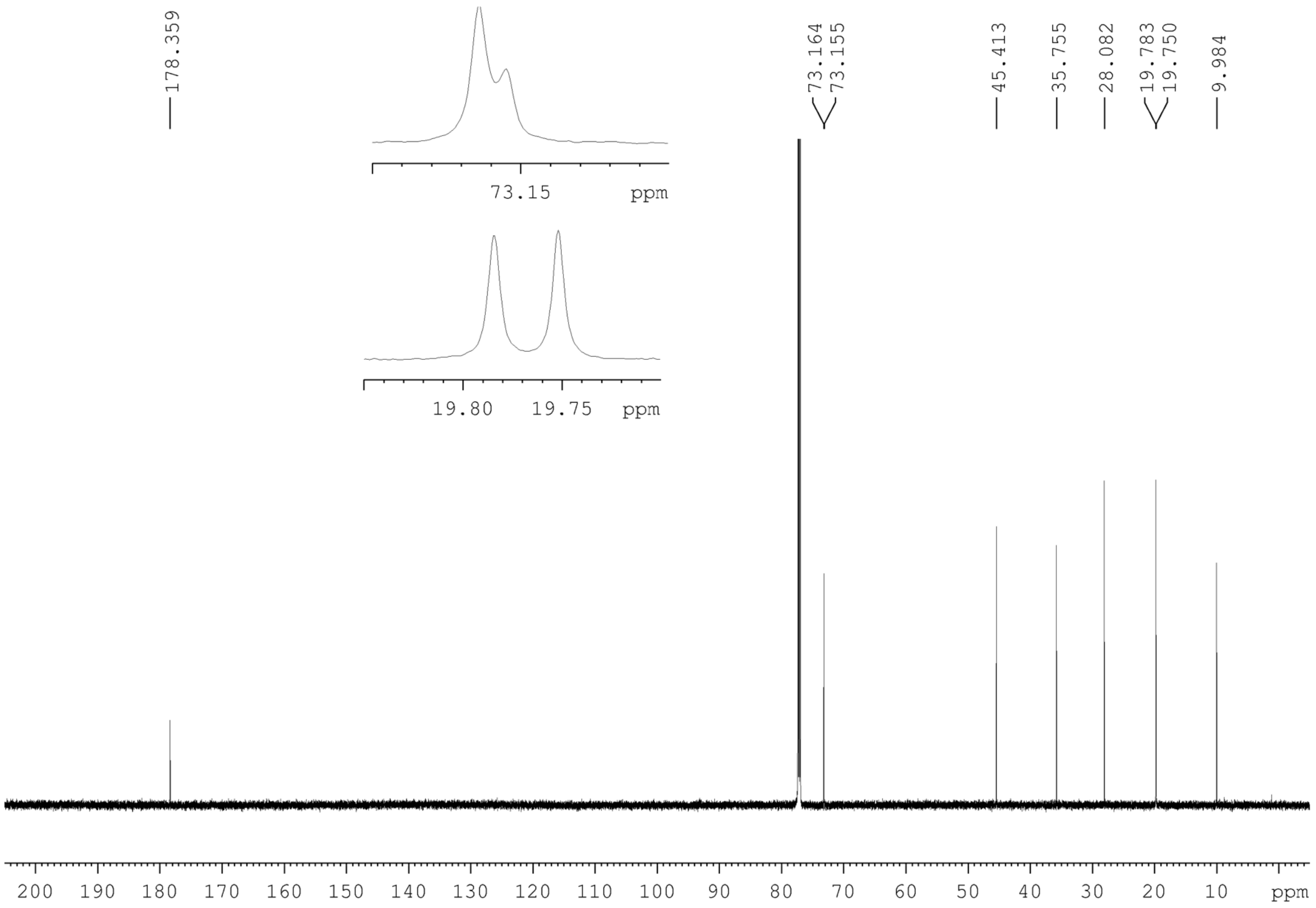

(*S*)-1-(*N*-isobutyrylbenzamido)butan-2-yl benzoate (1z-Bz)

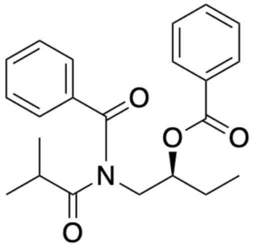

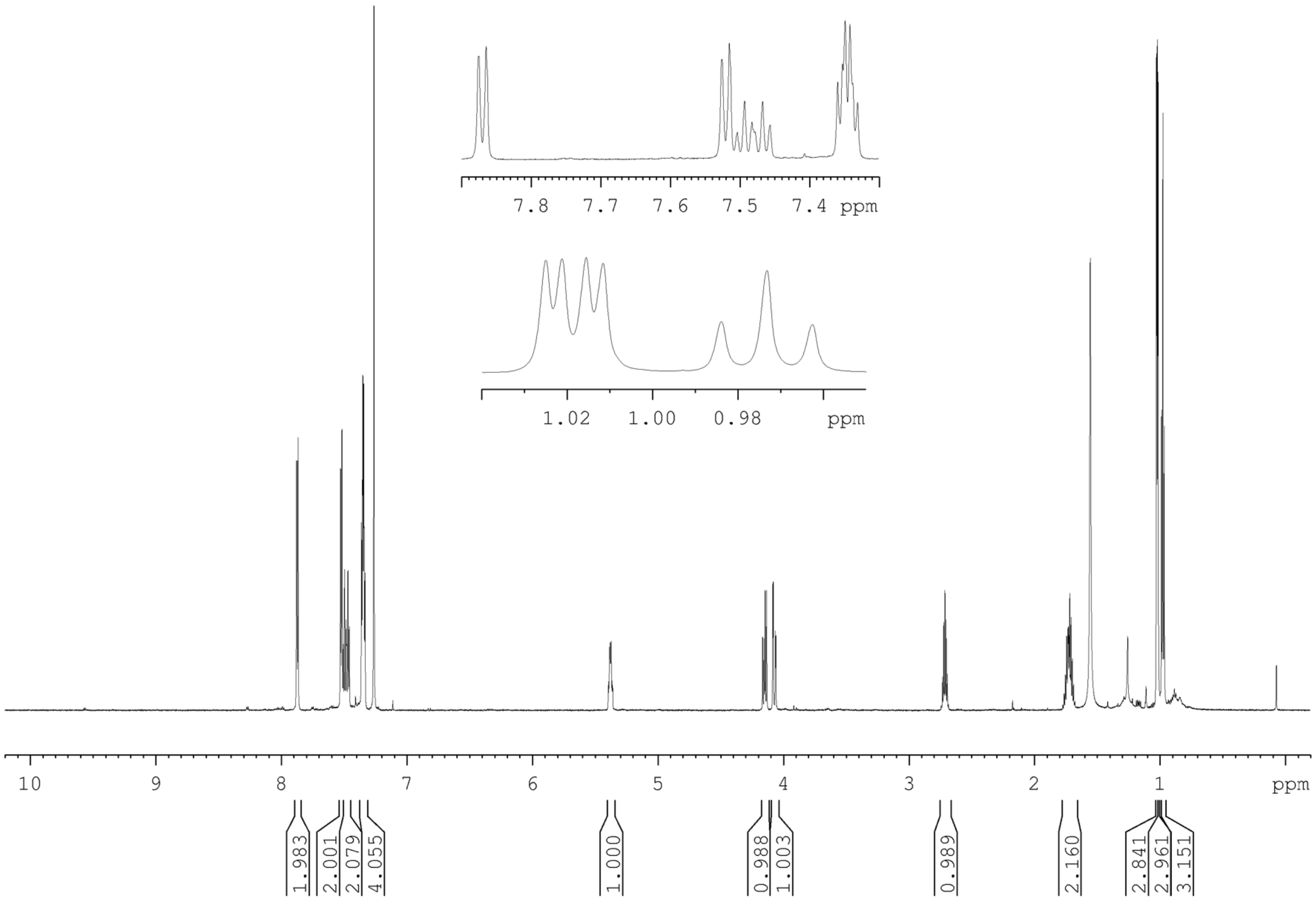

(*S*)-1-(*N*-isobutyrylbenzamido)butan-2-yl benzoate (1z-Bz)

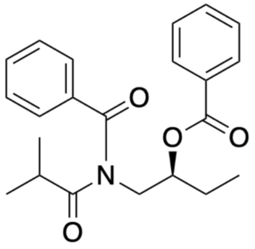

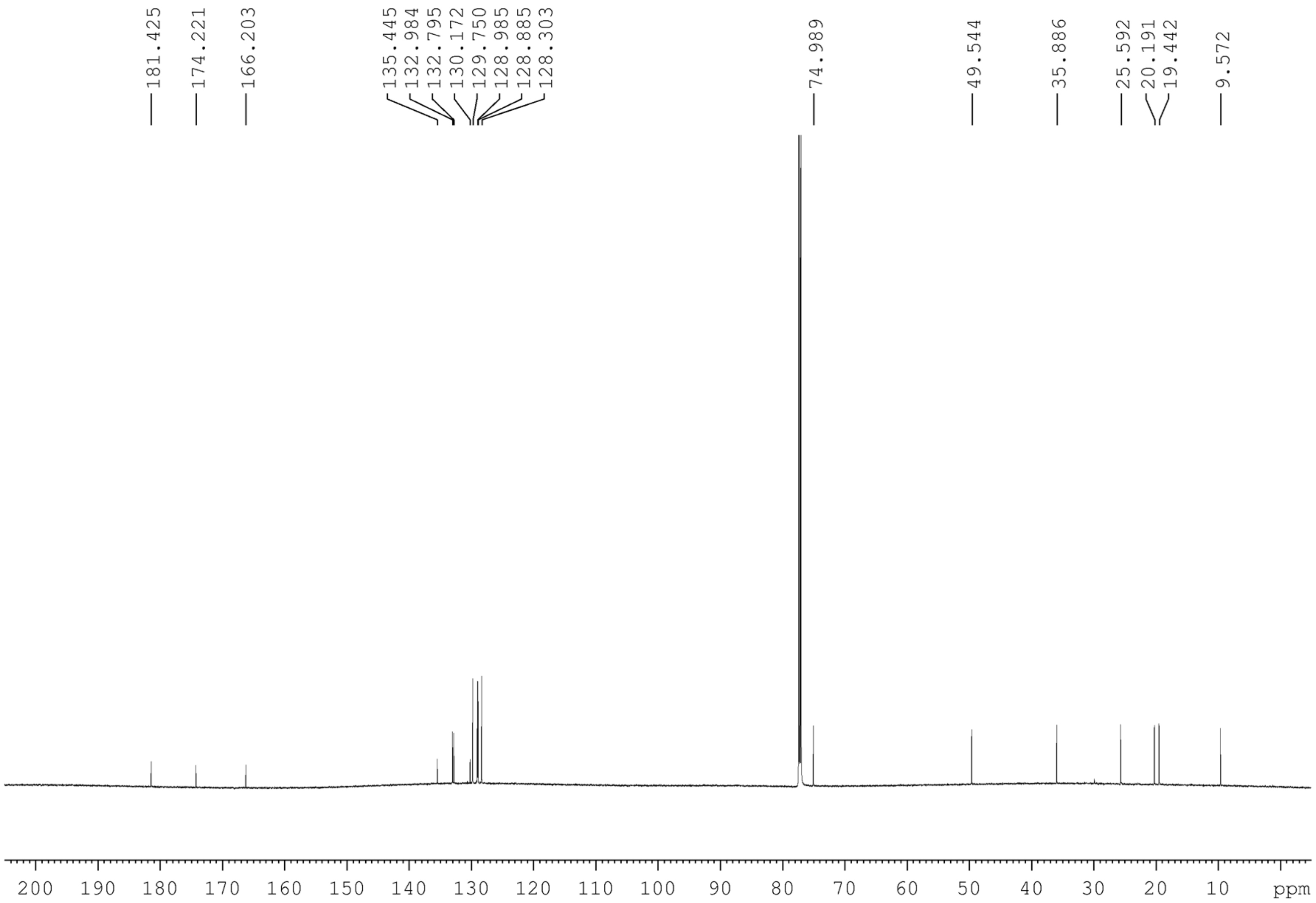

(3,5,7)-*N*-(2-hydroxybutyl)adamantane-1-carboxamide (1za-rac)

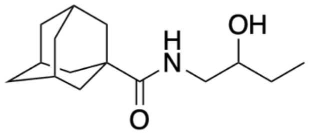

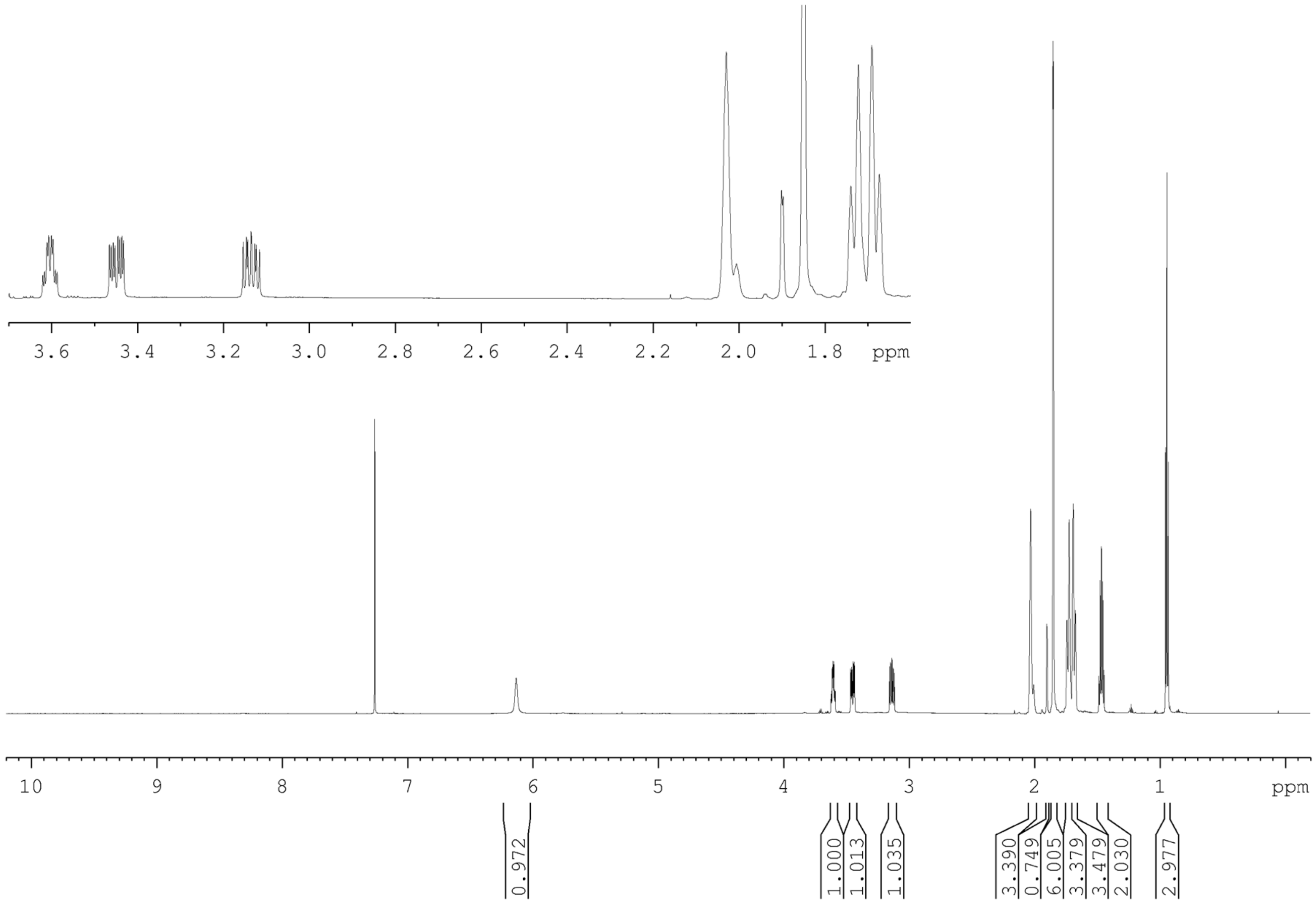

(3,5,7)-*N*-(2-hydroxybutyl)adamantane-1-carboxamide (1za-rac)

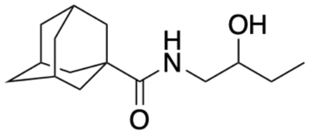

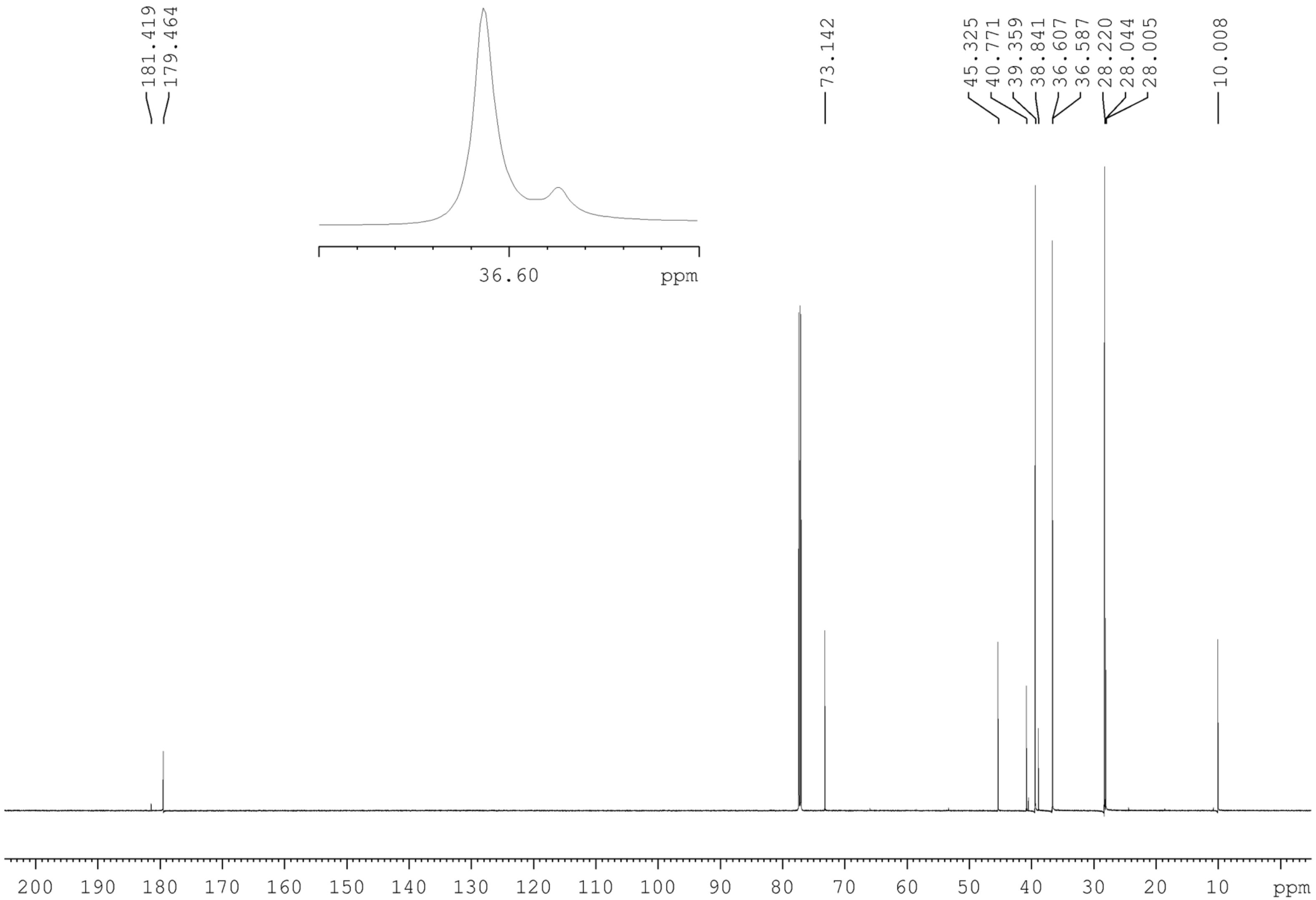

(3,5,7)-*N*-((*S*)-2-hydroxybutyl)adamantane-1-carboxamide (1za)

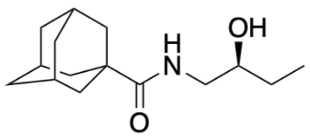

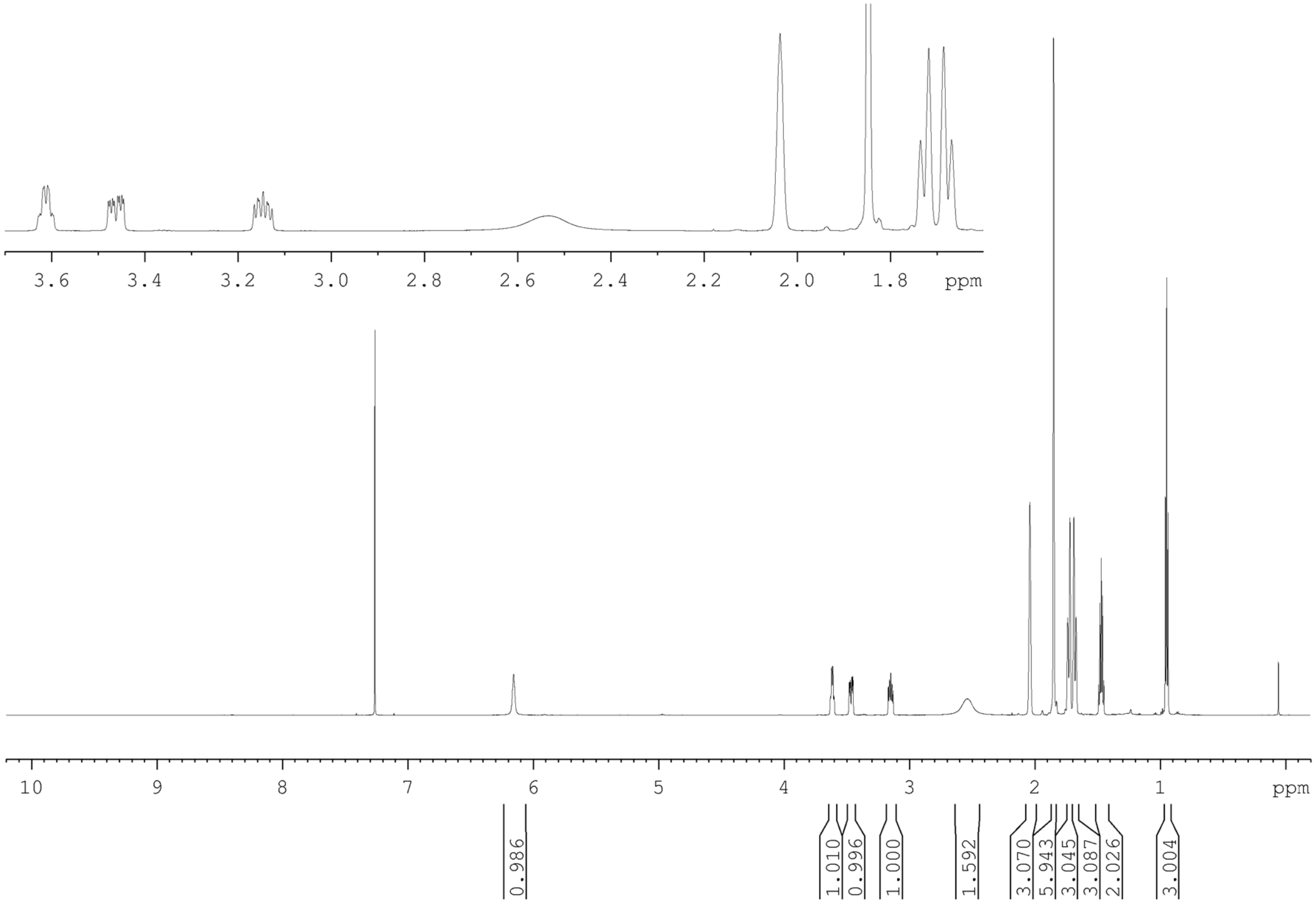

(3,5,7)-*N*-((*S*)-2-hydroxybutyl)adamantane-1-carboxamide (1za)

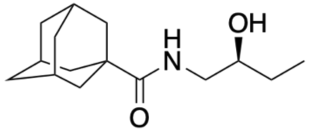

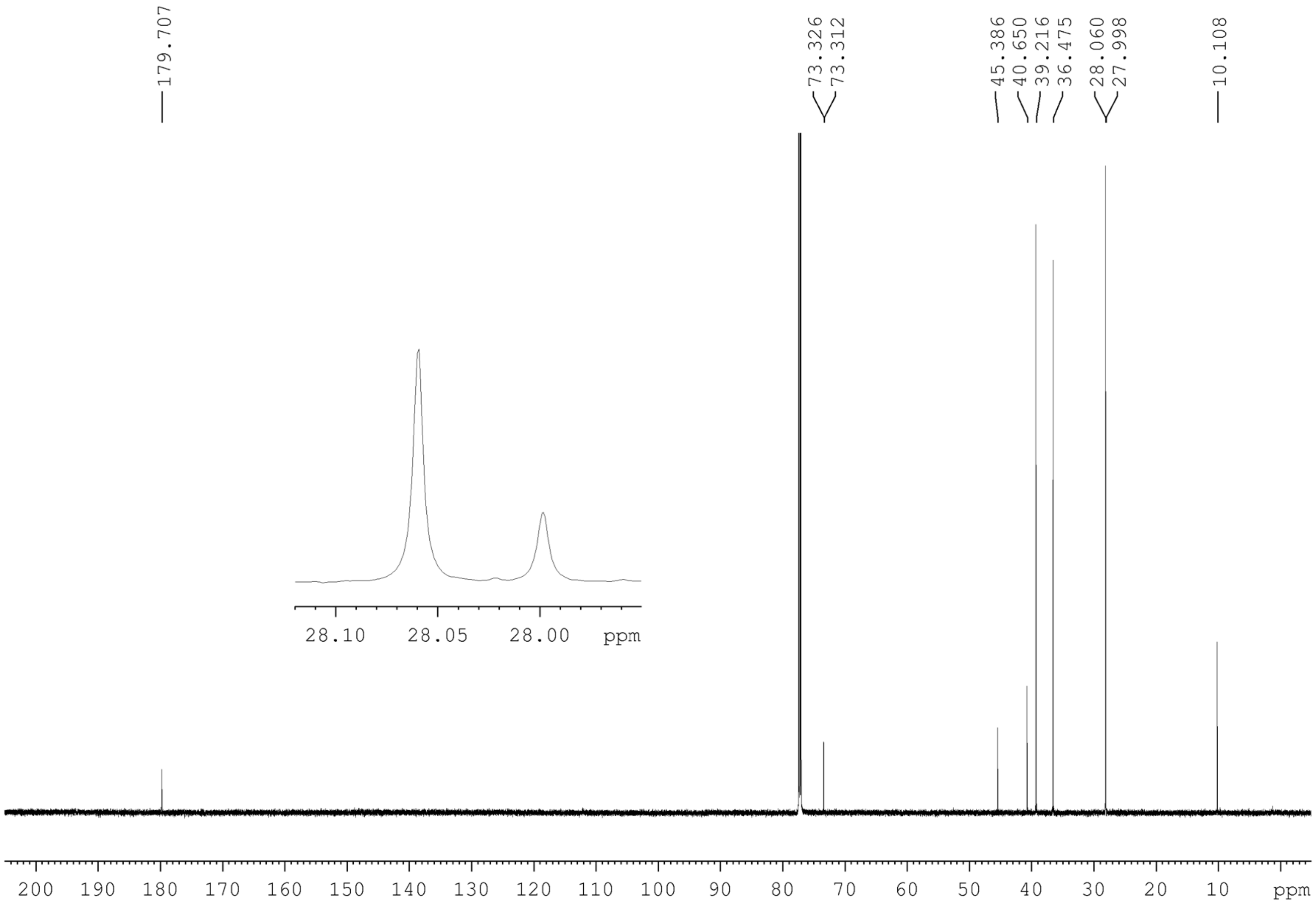

(*S*)-1-((3,5,7)-adamantane-1-carboxamido)butan-2-yl benzoate (1za-Bz)

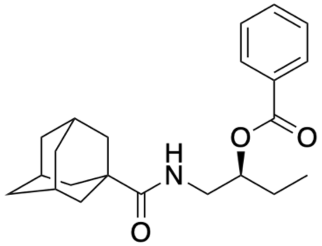

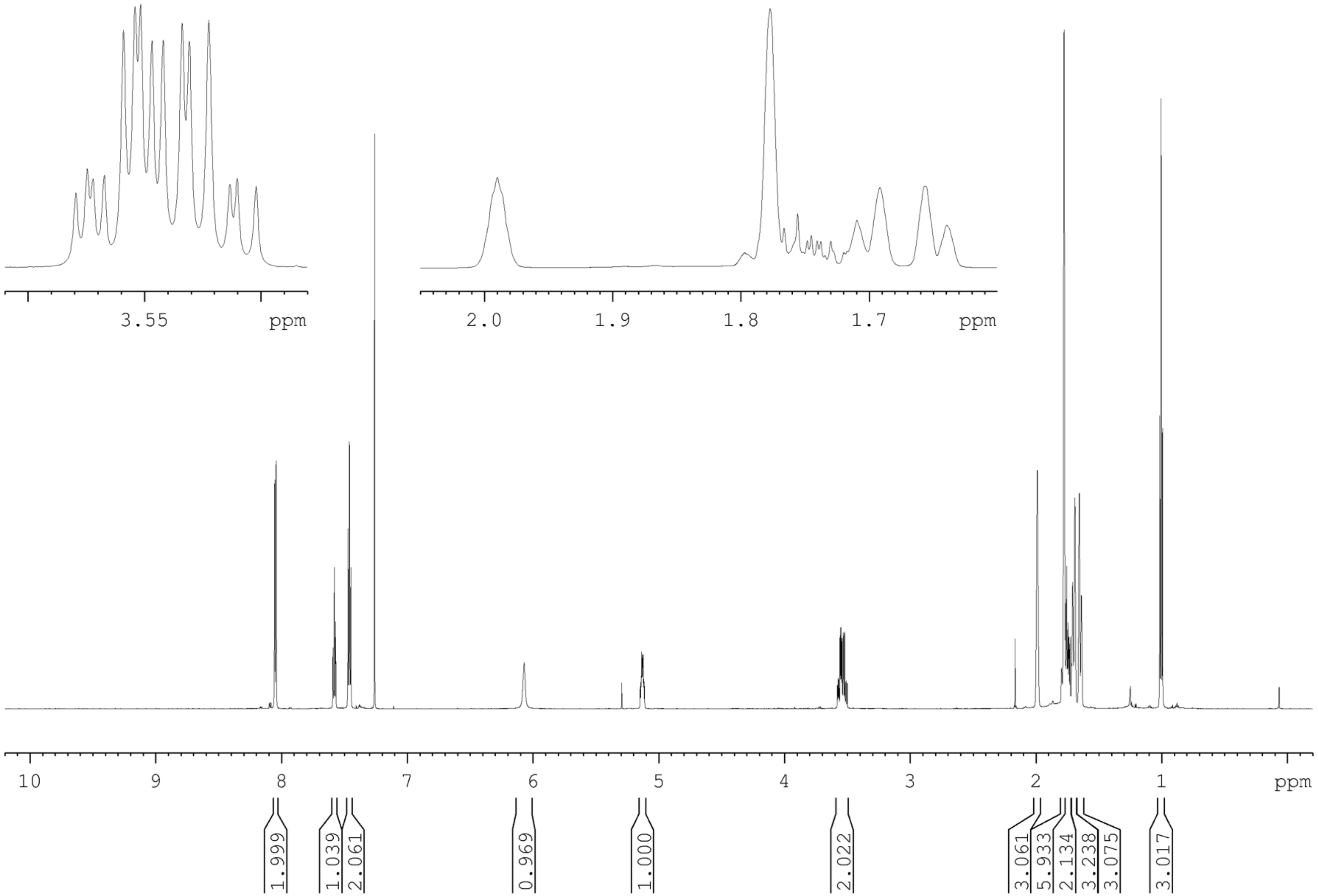

(*S*)-1-((3,5,7)-adamantane-1-carboxamido)butan-2-yl benzoate (1za-Bz)

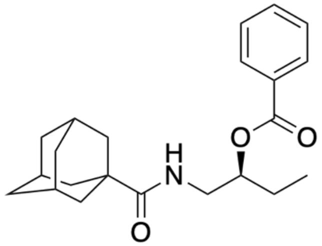

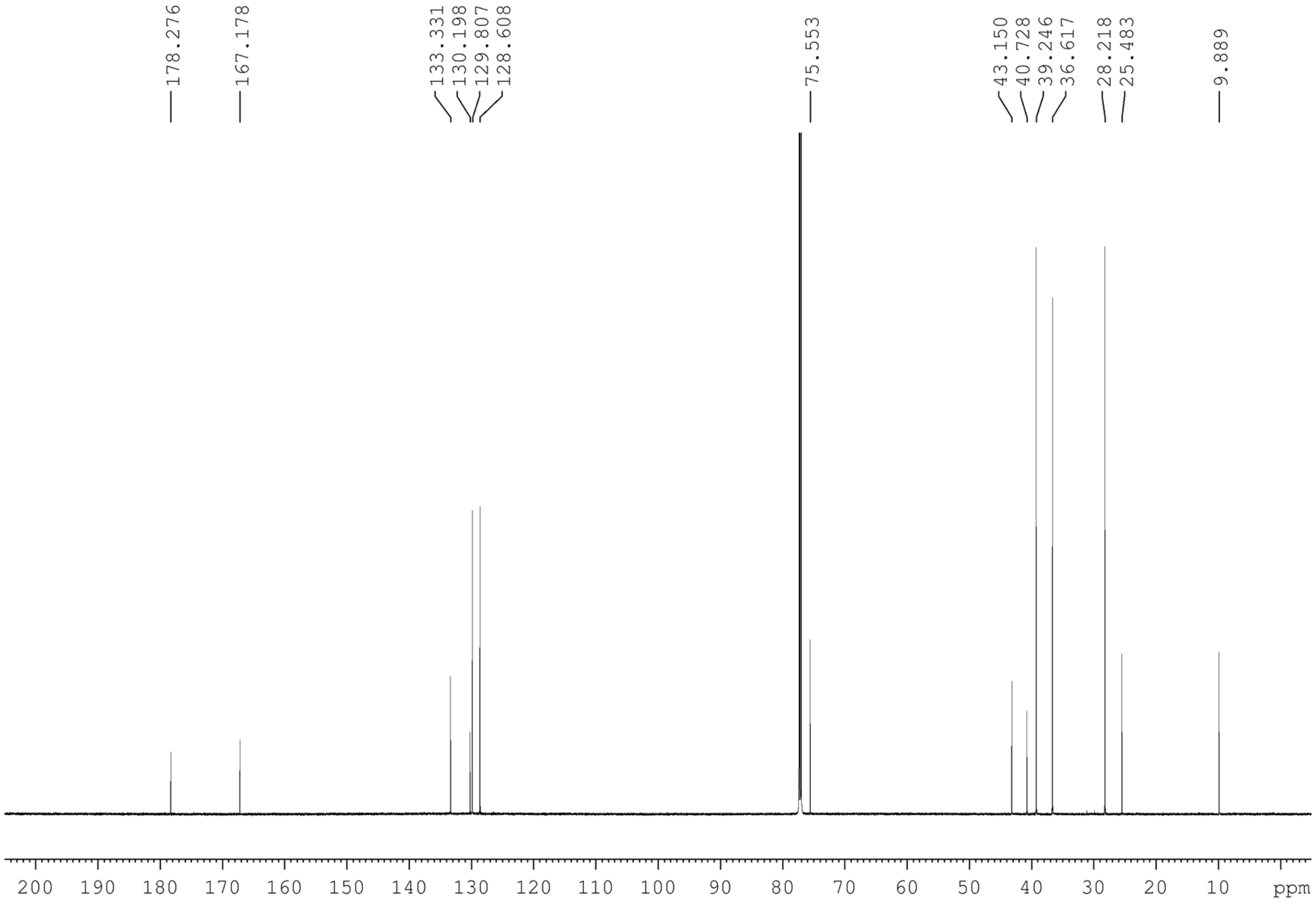

3-cyano-*N*-(2-hydroxybutyl)propanamide (1zb-rac)

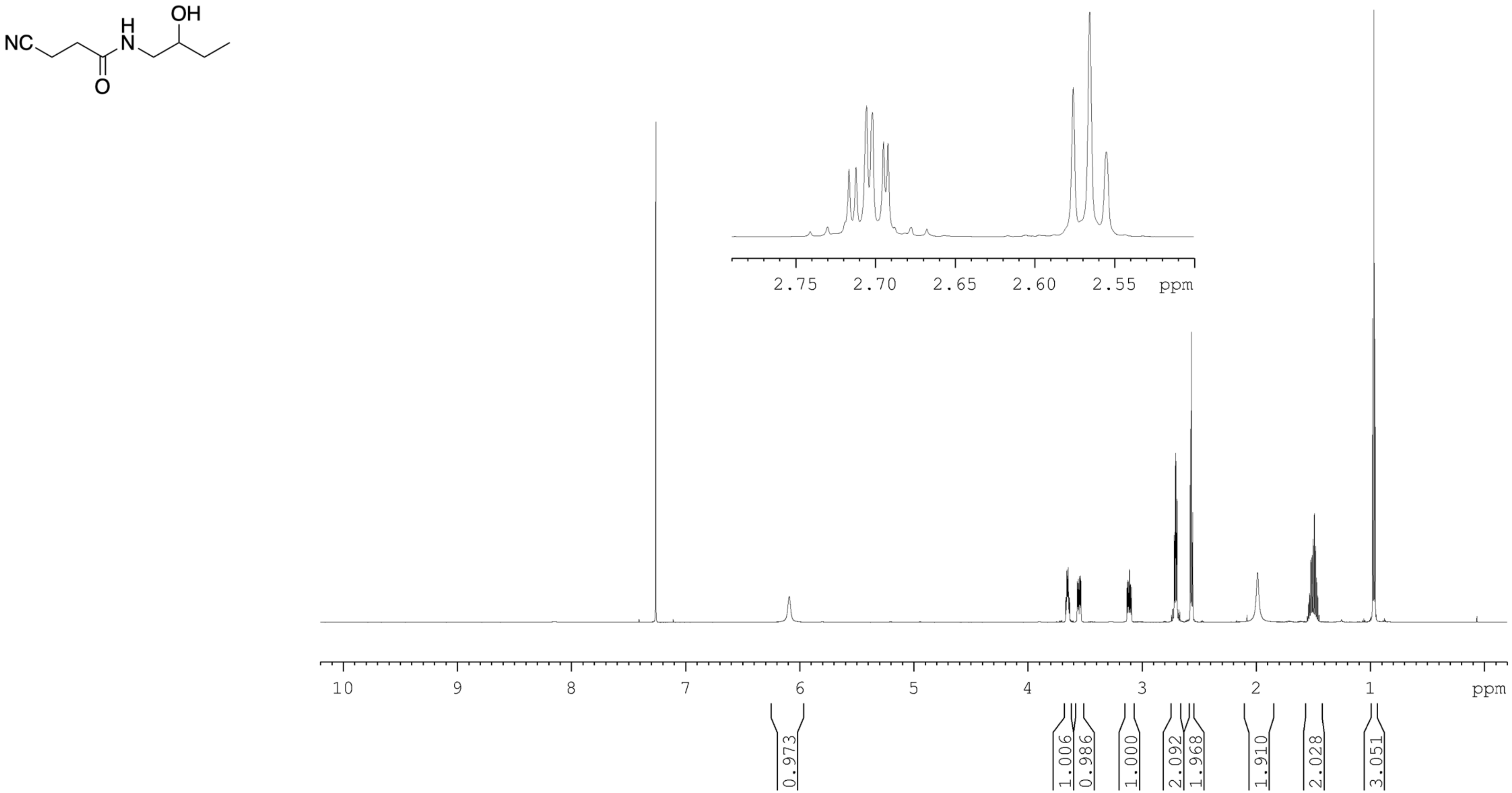

3-cyano-*N*-(2-hydroxybutyl)propanamide (1zb-rac)

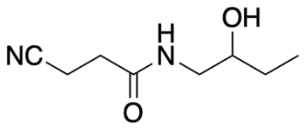

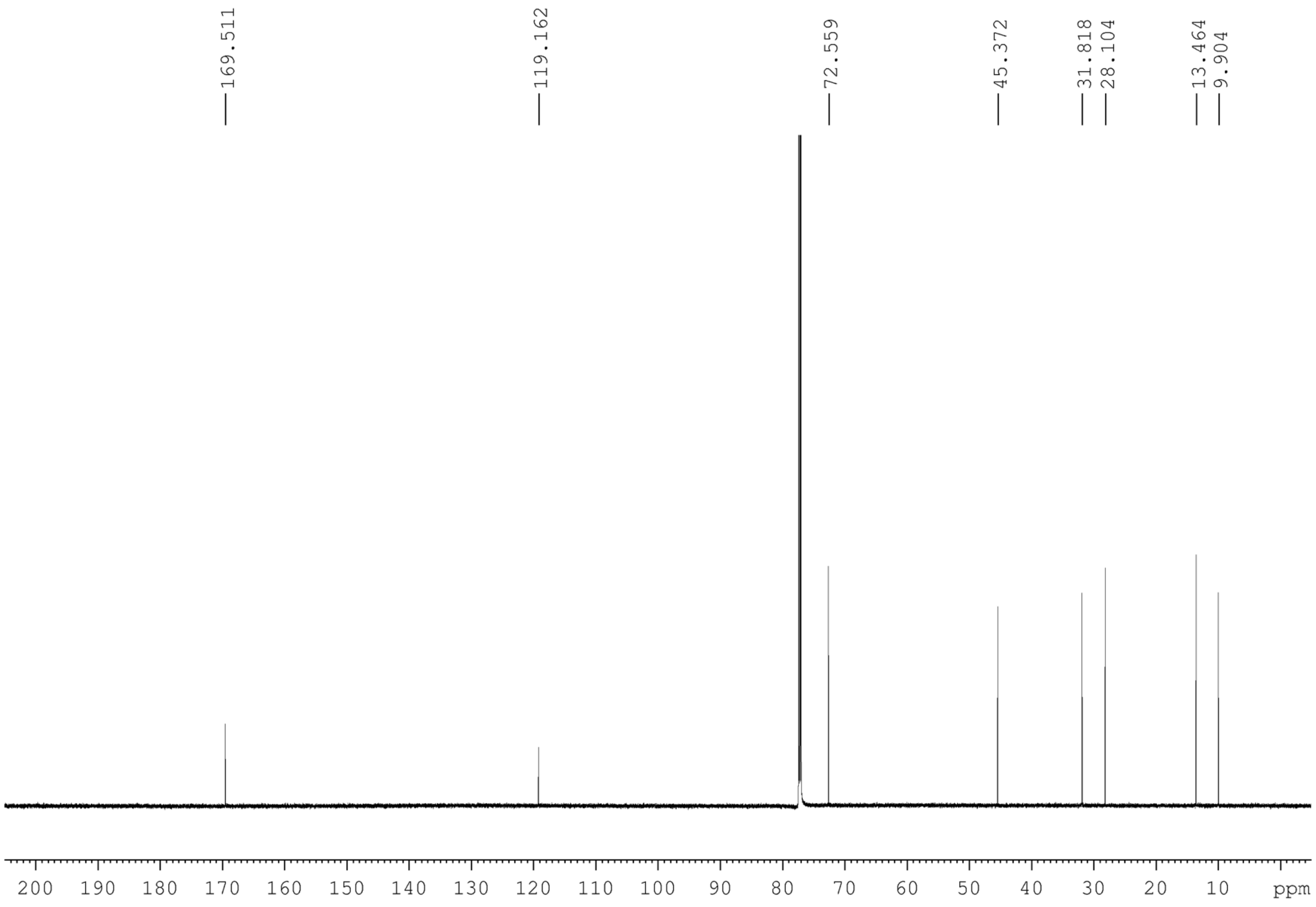

(*S*)-3-cyano-*N*-(2-hydroxybutyl)propanamide (1zb)

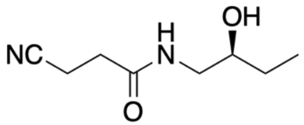

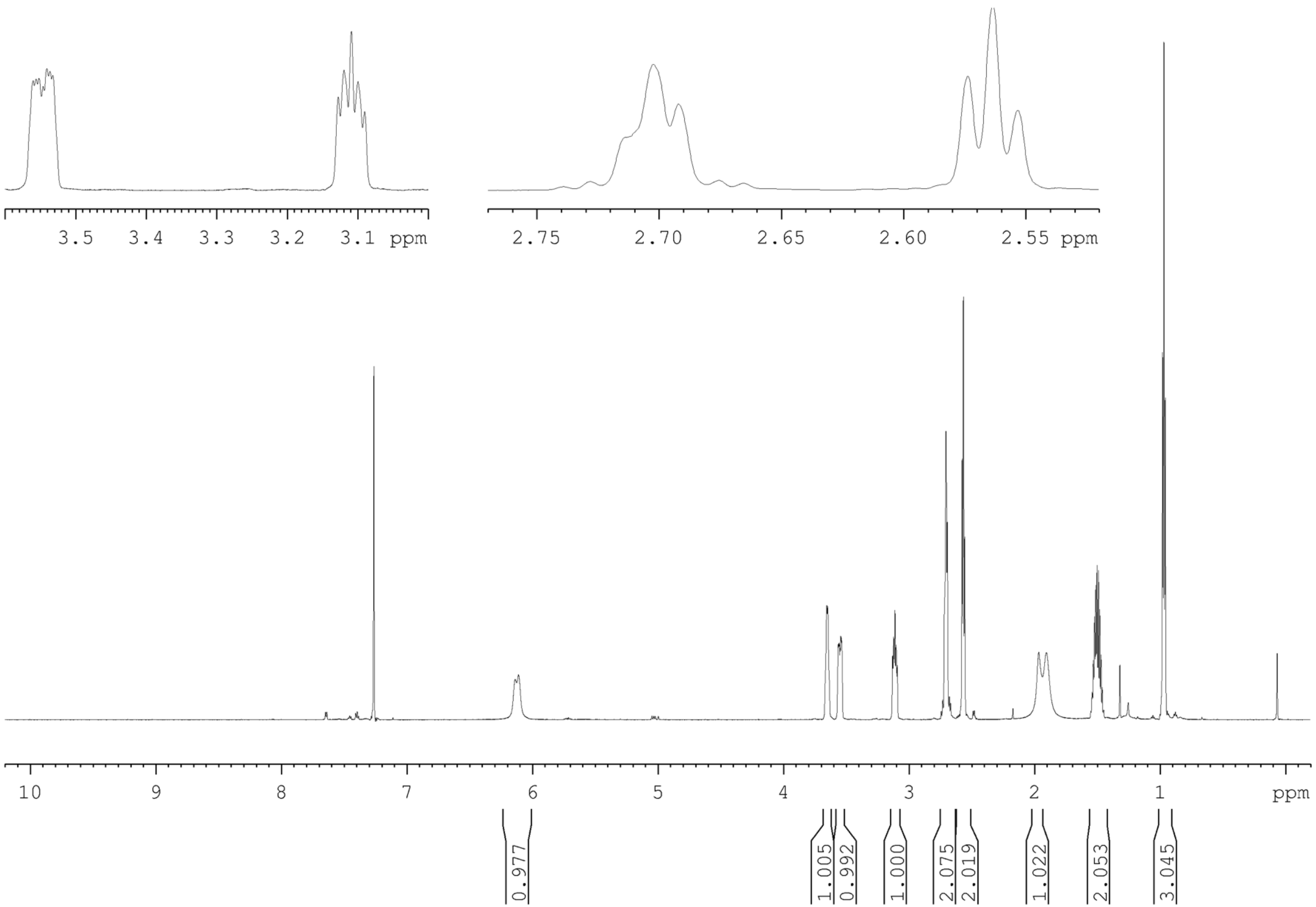

(*S*)-3-cyano-*N*-(2-hydroxybutyl)propanamide (1zb)

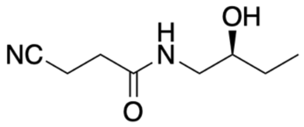

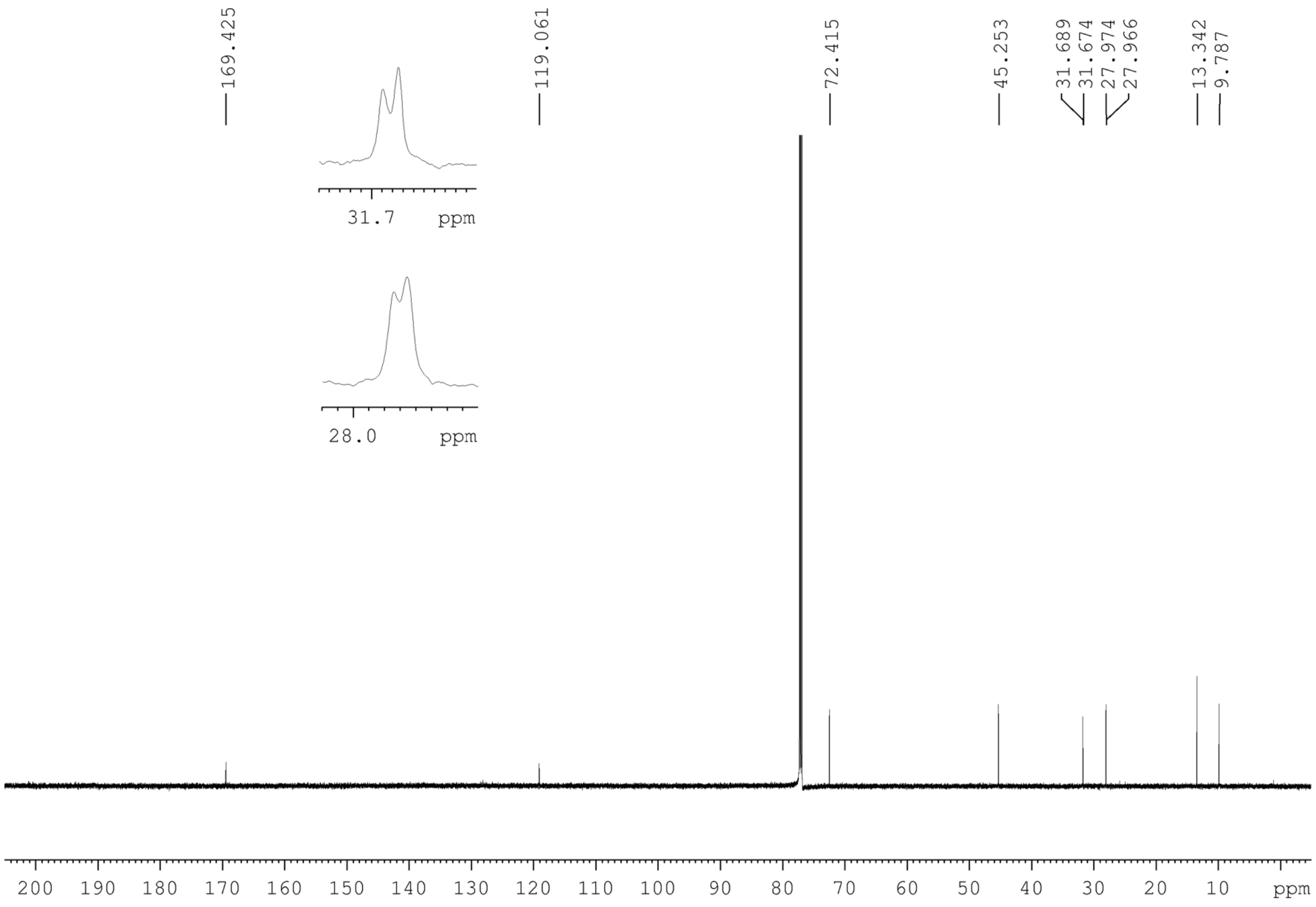

(*S*)-1-(*N*-(3-cyanopropanoyl)benzamido)butan-2-yl benzoate (1zb-Bz)

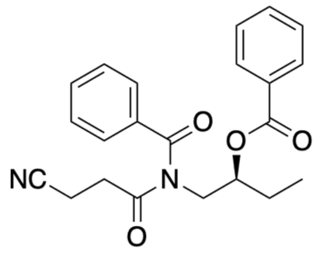

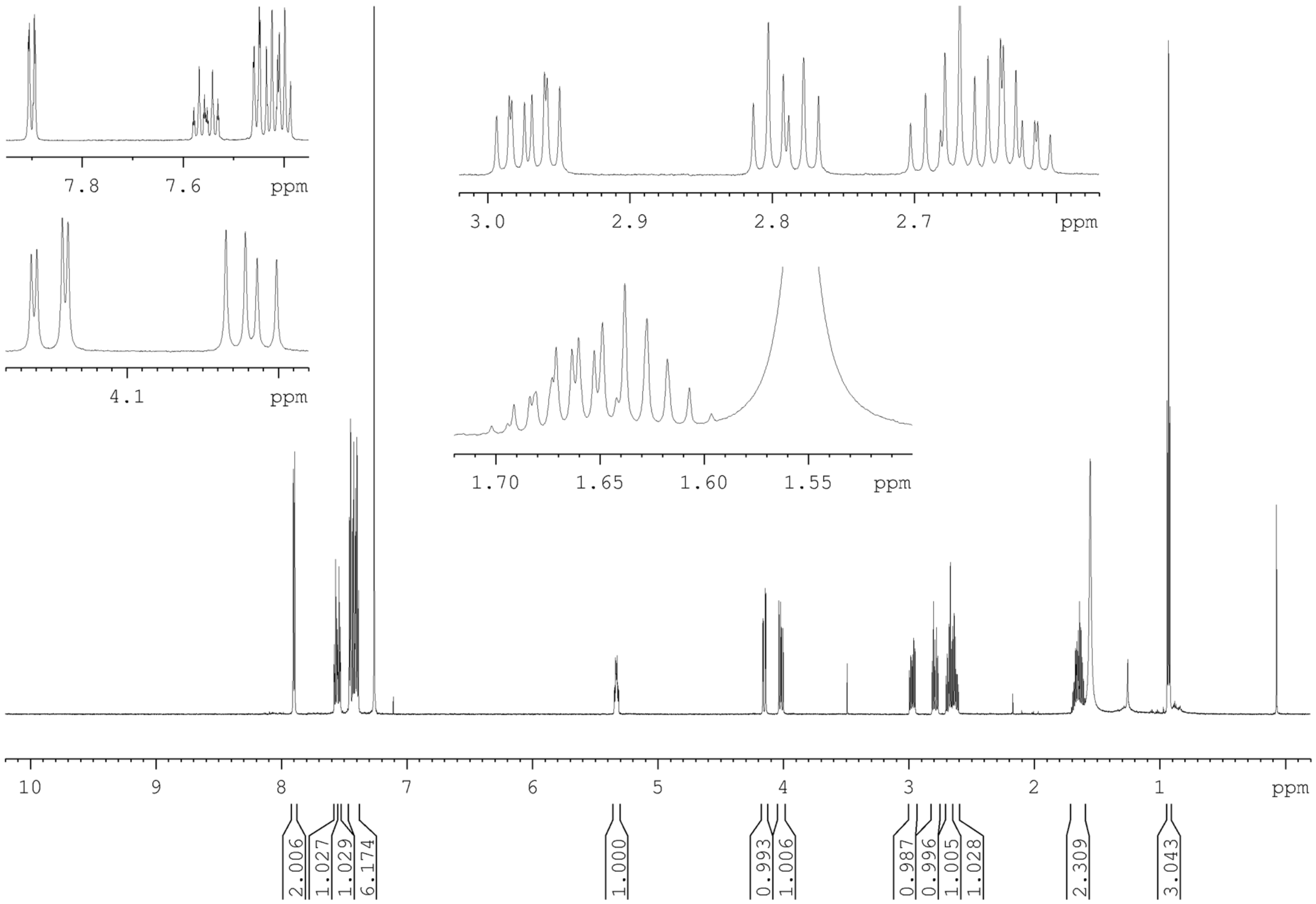

(*S*)-1-(*N*-(3-cyanopropanoyl)benzamido)butan-2-yl benzoate (1zb-Bz)

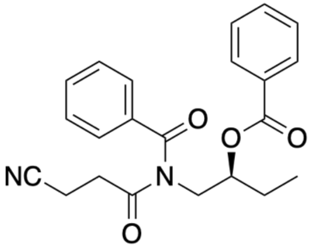

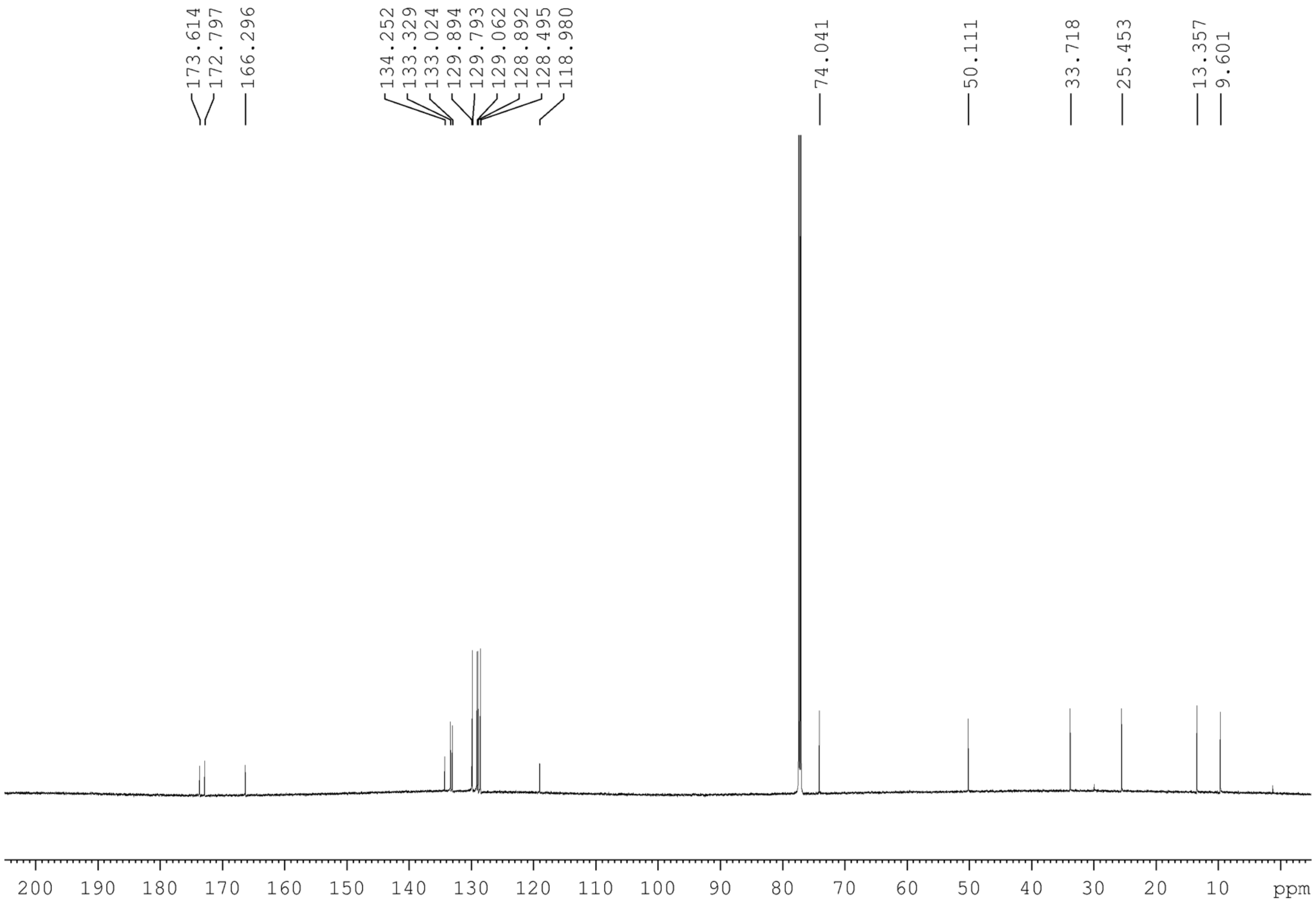

***N*-(2-hydroxybutyl)-2-(phenylsulfonyl)acetamide (1zc-rac)**

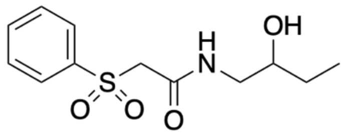

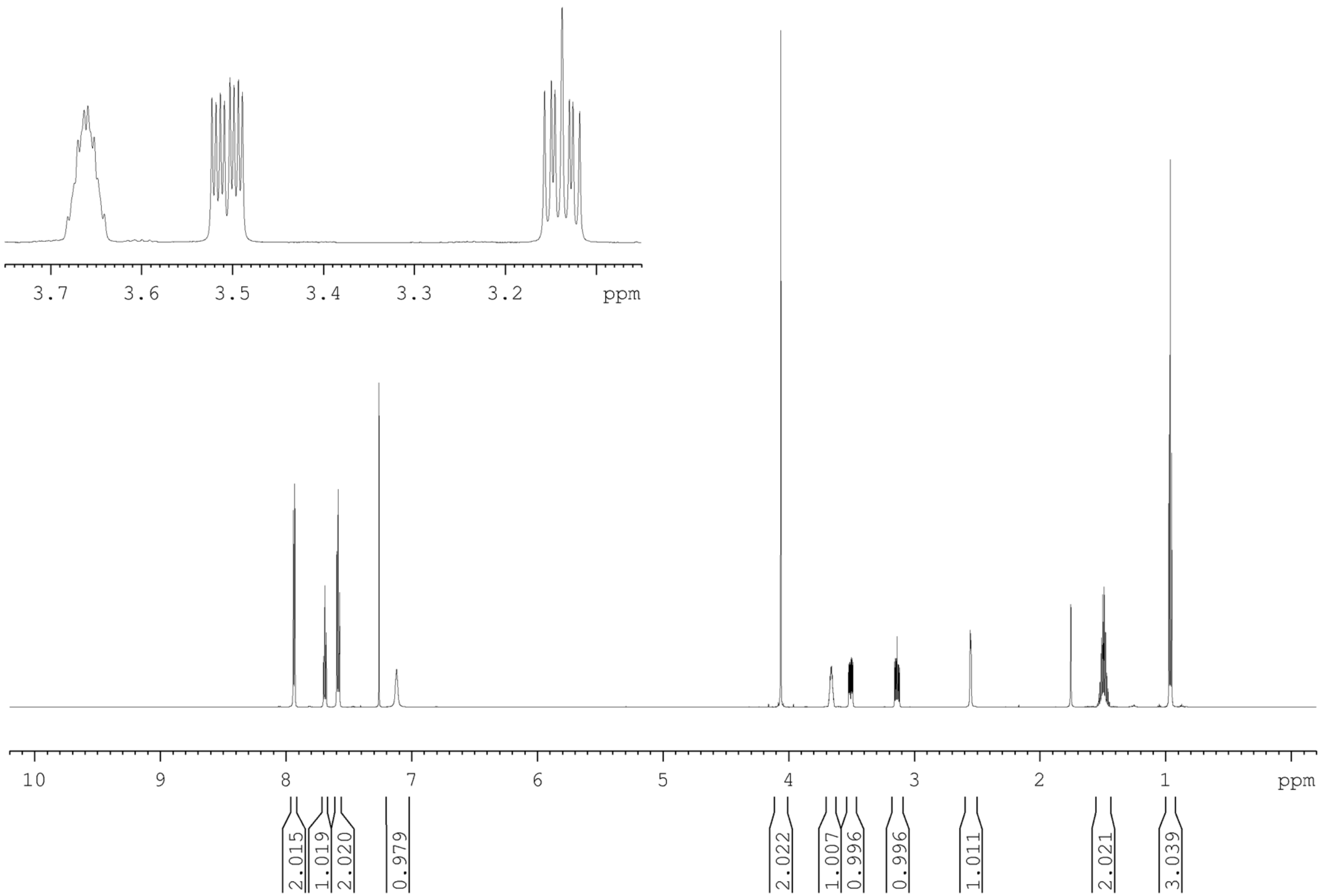

N-(2-hydroxybutyl)-2-(phenylsulfonyl)acetamide (1zc-rac)

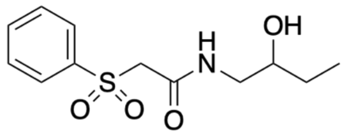

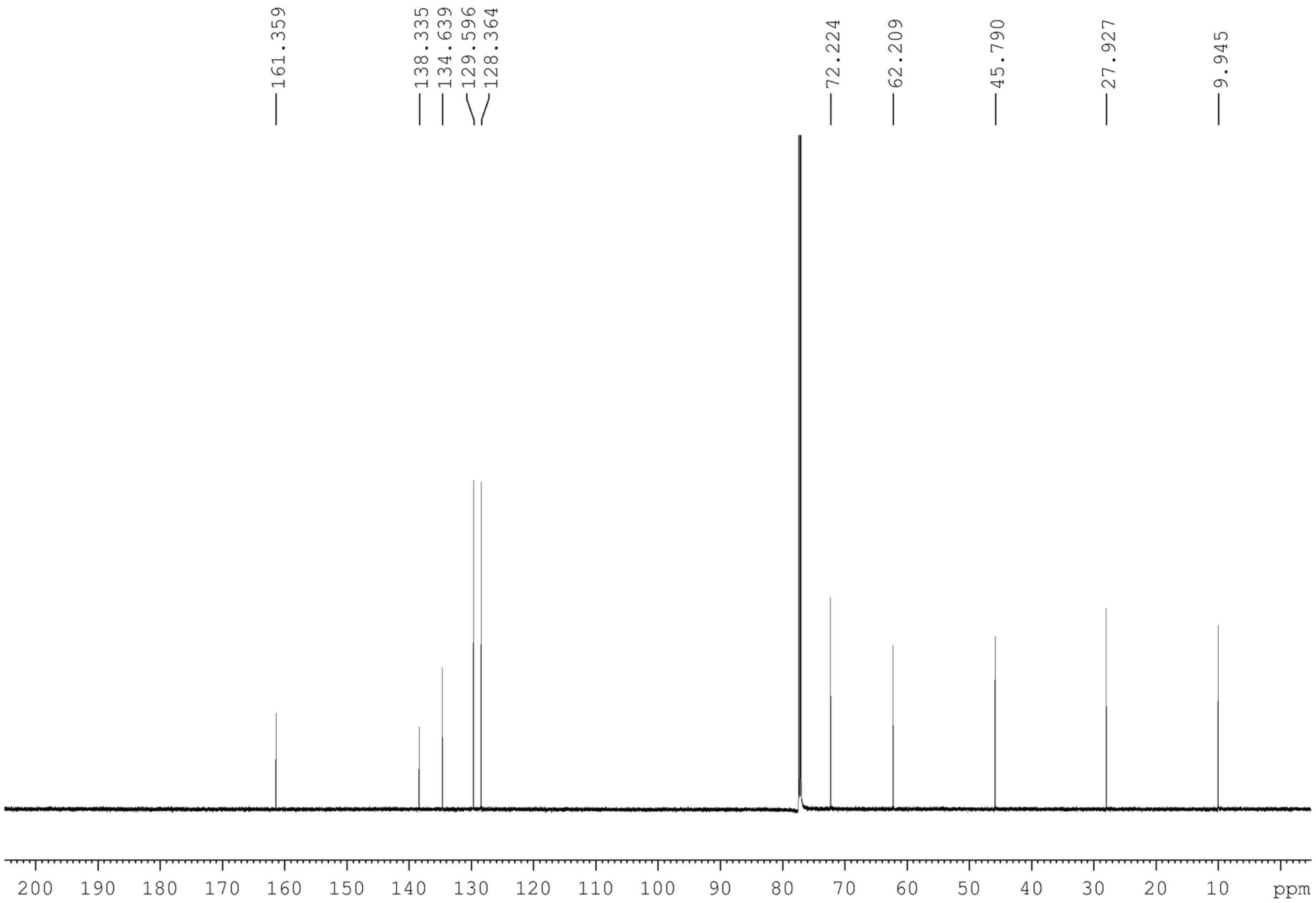

(*S*)-*N*-(2-hydroxybutyl)-2-(phenylsulfonyl)acetamide (1zc)

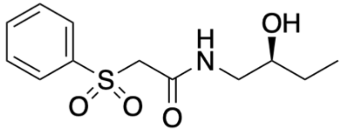

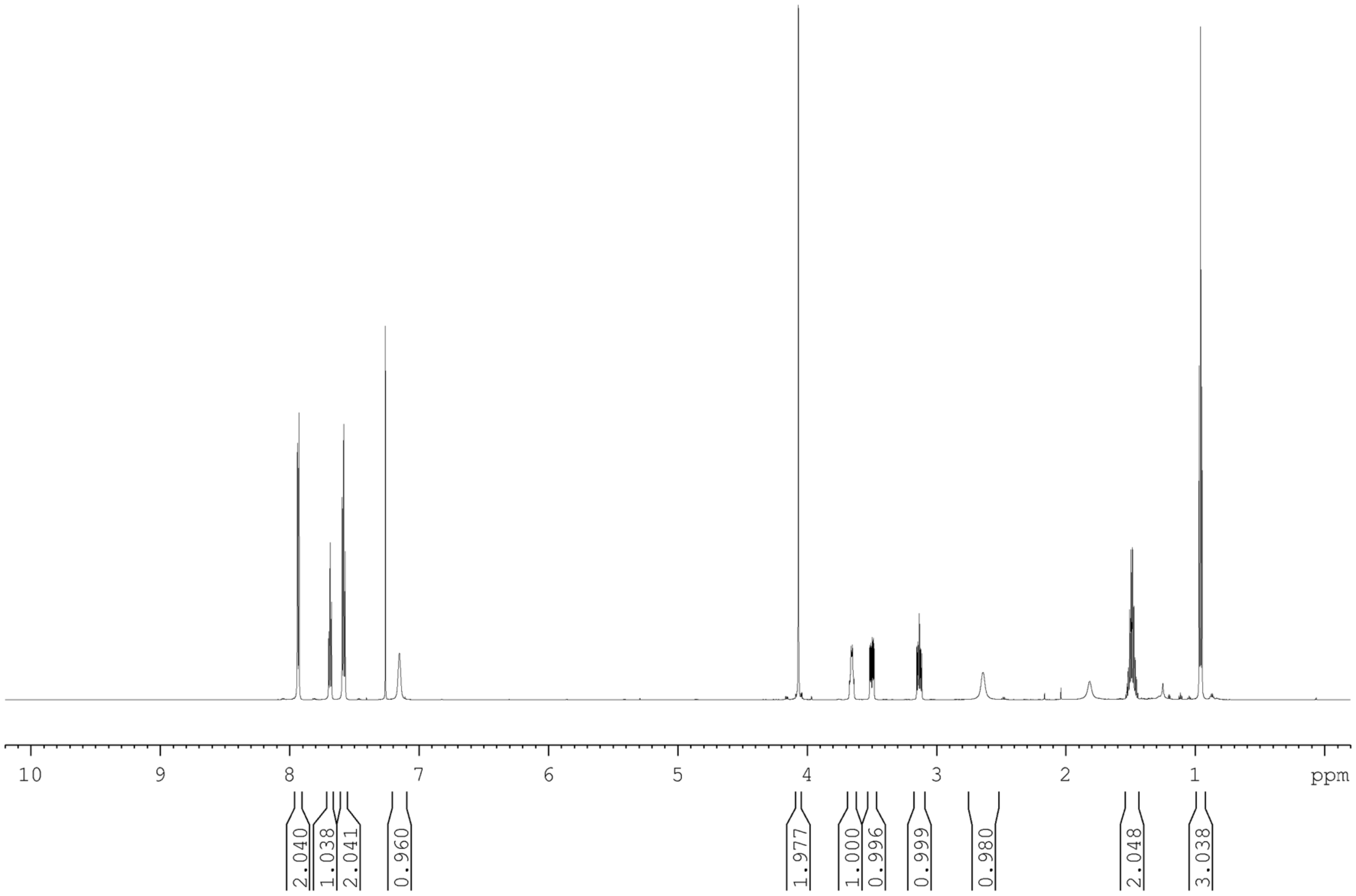

(*S*)-*N*-(2-hydroxybutyl)-2-(phenylsulfonyl)acetamide (1zc)

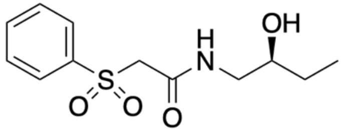

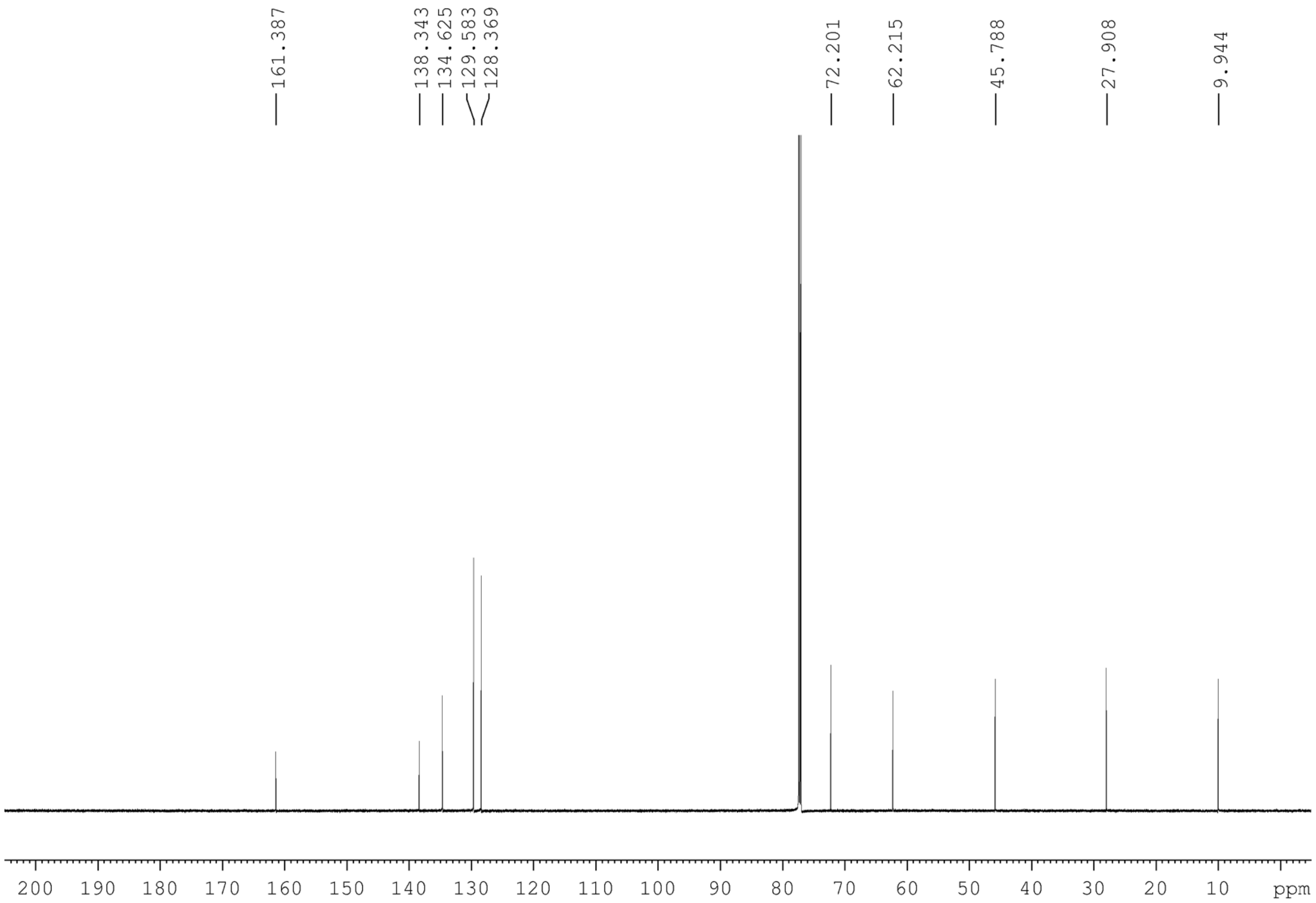

N-(2-hydroxybutyl)-3-phenylpropanamide (1zd-rac)

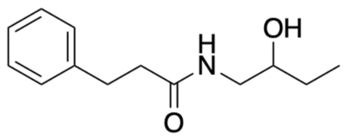

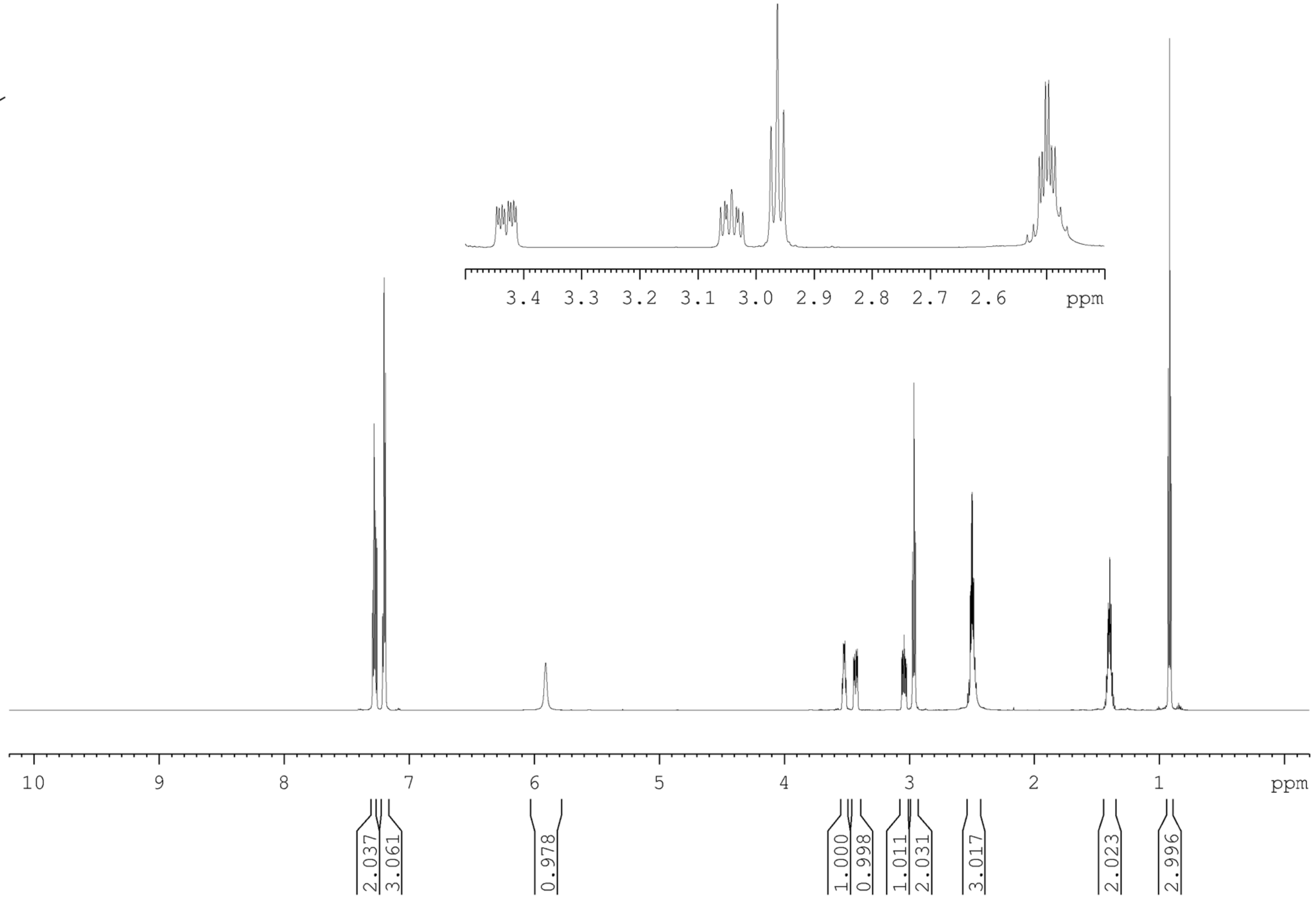

N-(2-hydroxybutyl)-3-phenylpropanamide (1zd-rac)

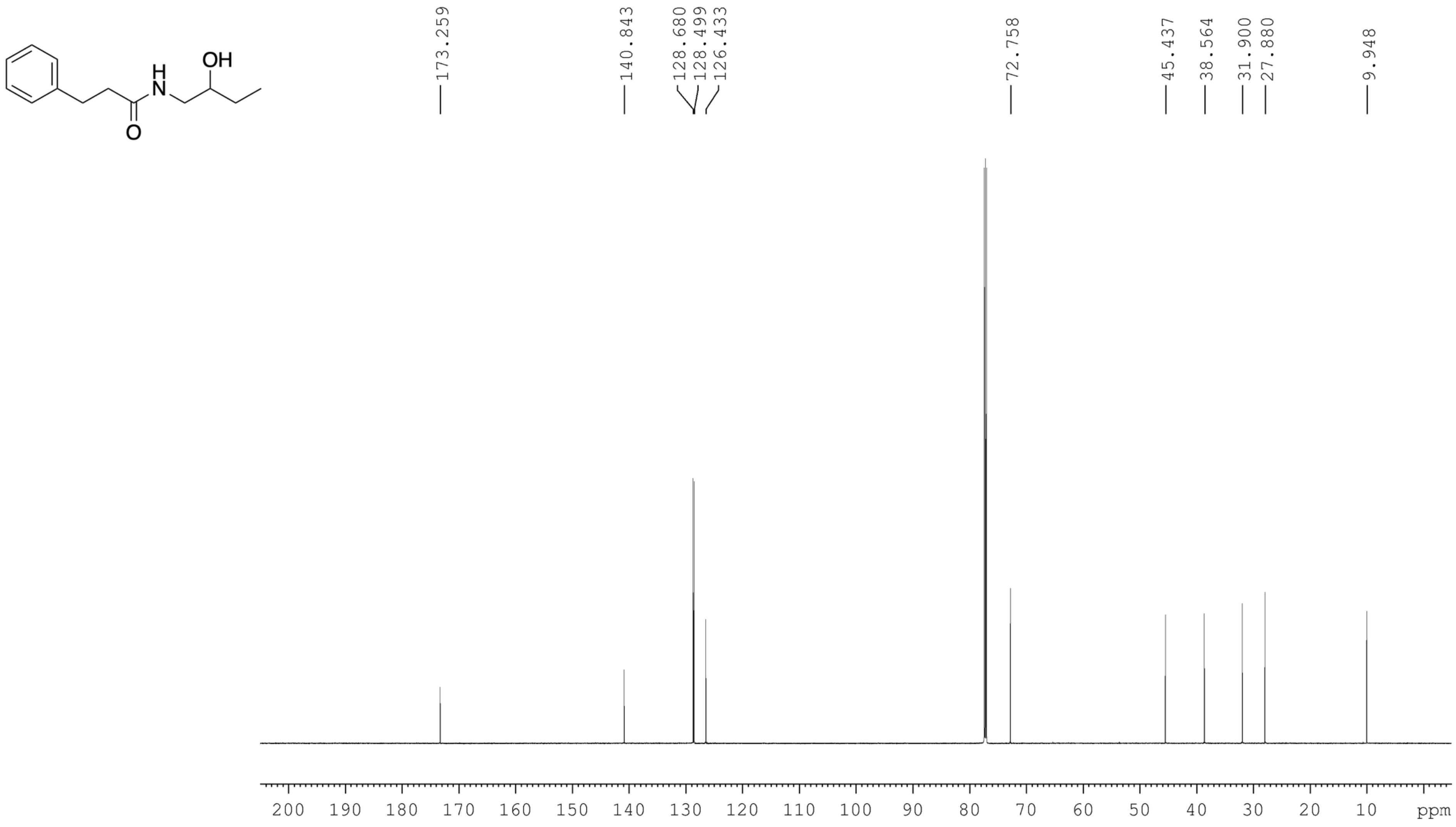

(*S*)-*N*-(2-hydroxybutyl)-3-phenylpropanamide (1zd)

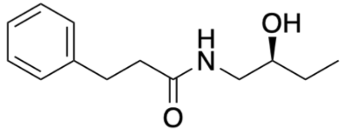

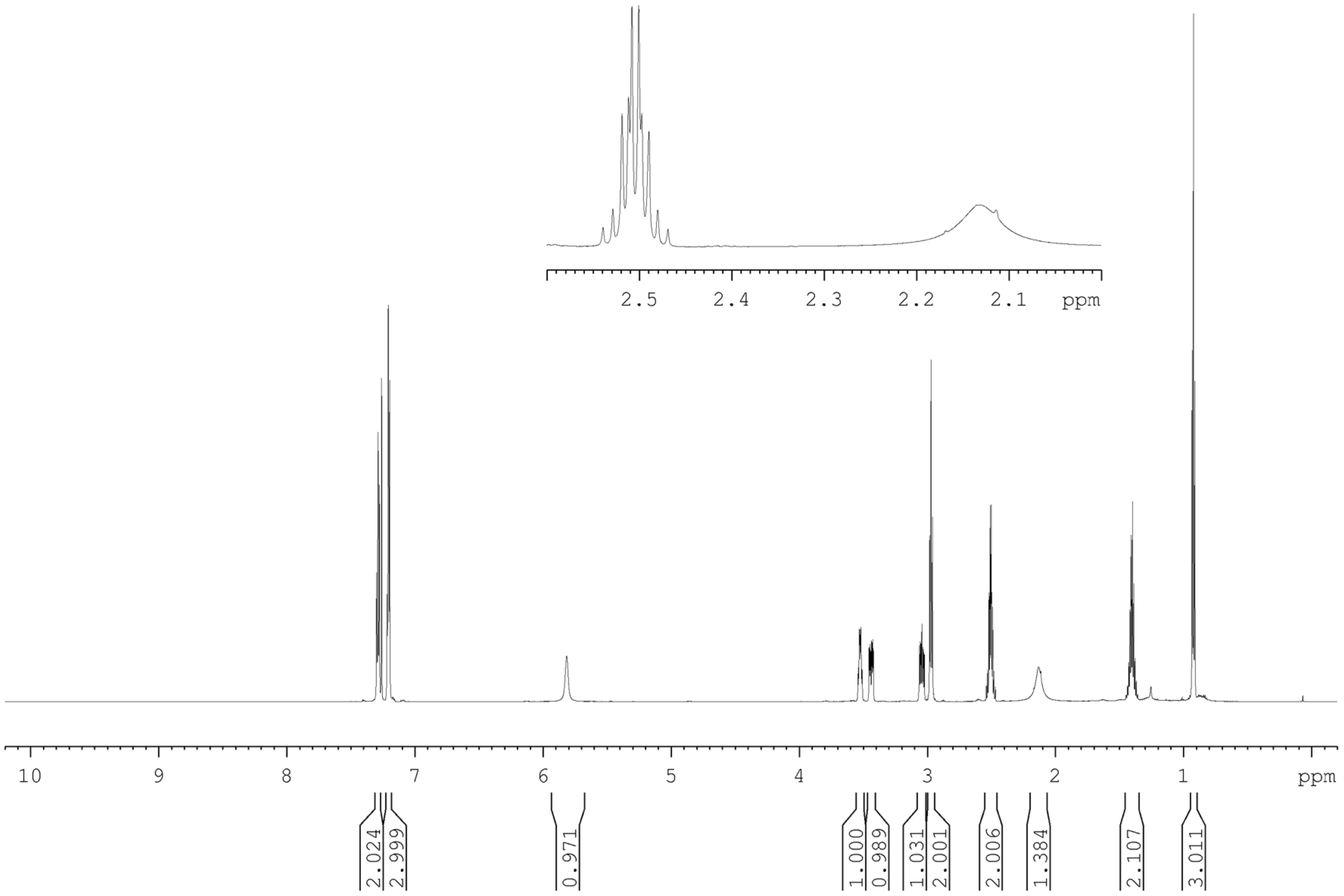

(*S*)-*N*-(2-hydroxybutyl)-3-phenylpropanamide (1zd)

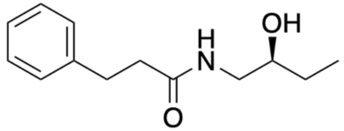

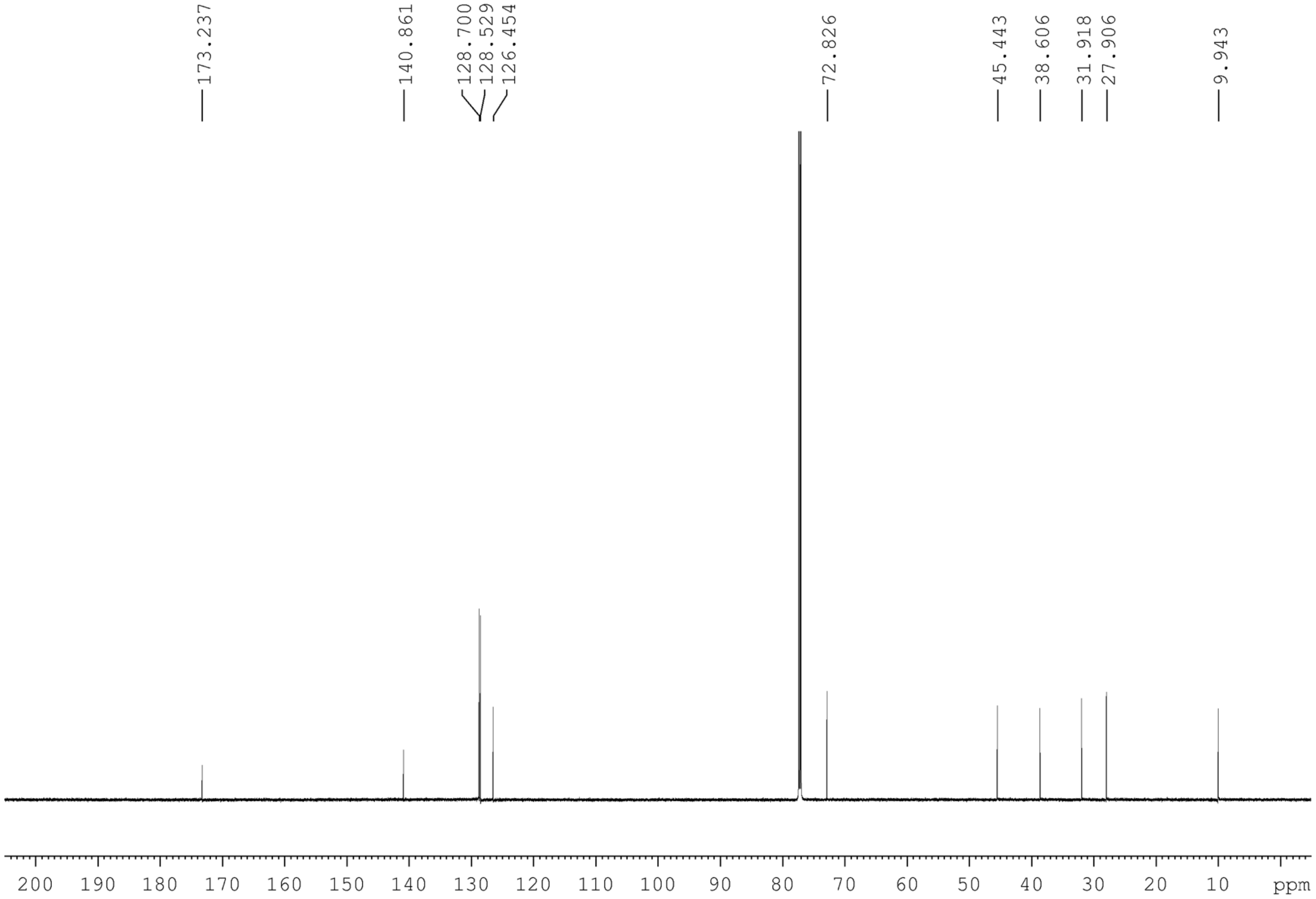

N-(2-hydroxybutyl)benzamide (1ze-rac)

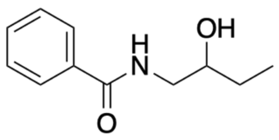

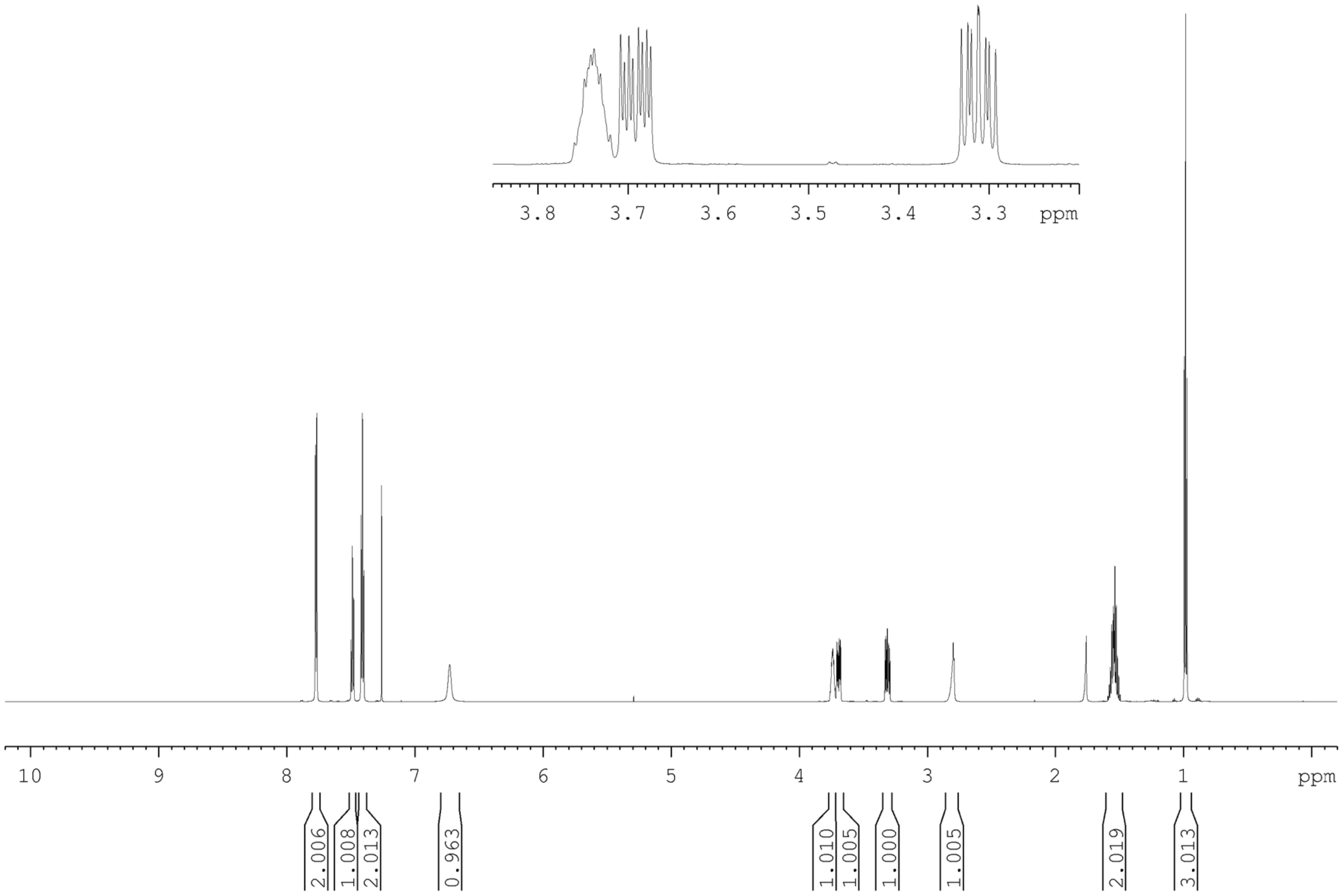

N-(2-hydroxybutyl)benzamide (1ze-rac)

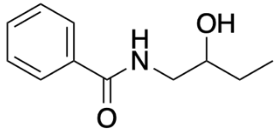

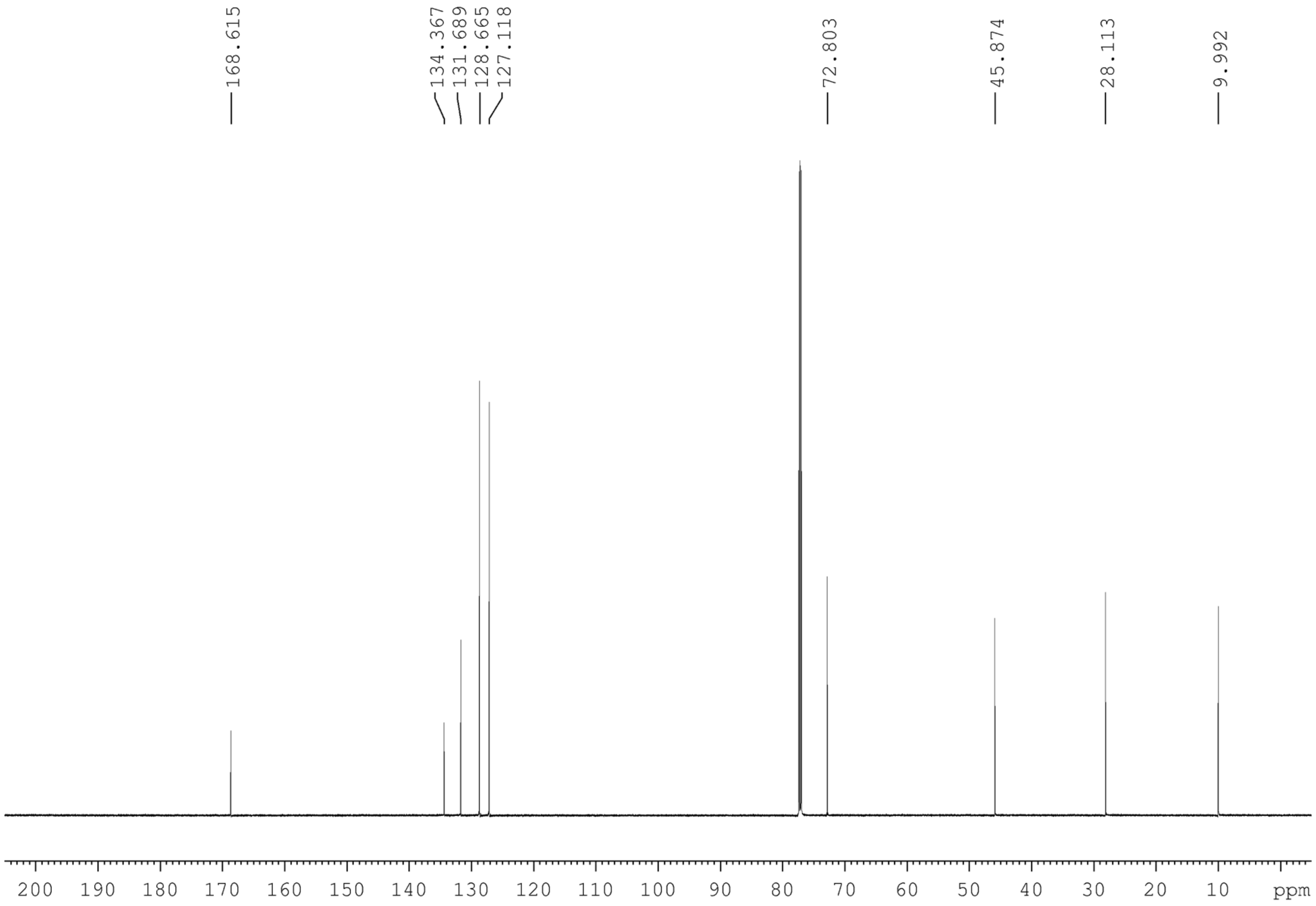

(*S*)-*N*-(2-hydroxybutyl)benzamide (1ze)

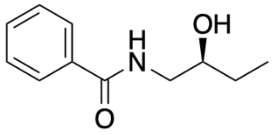

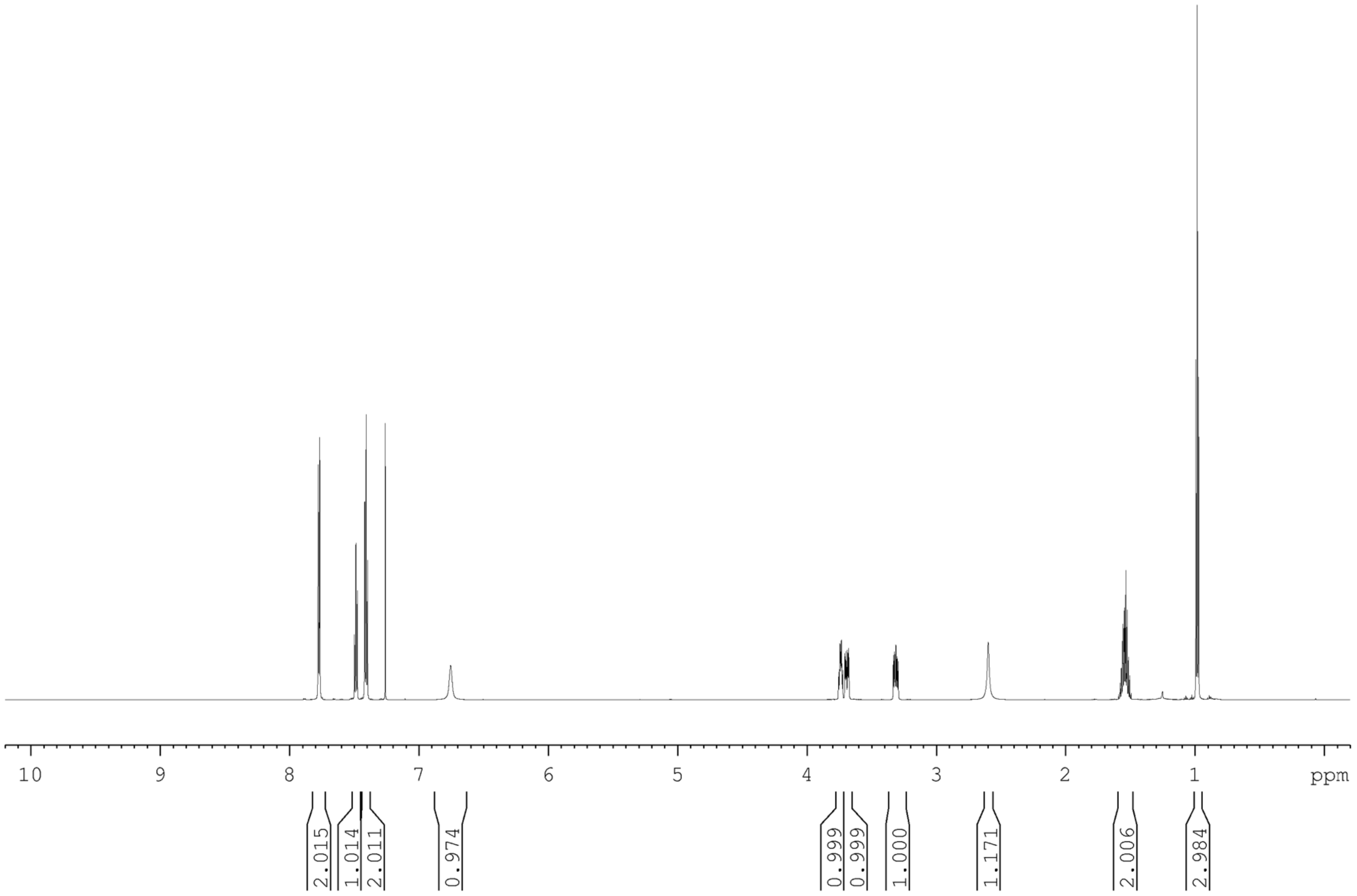

(*S*)-*N*-(2-hydroxybutyl)benzamide (1ze)

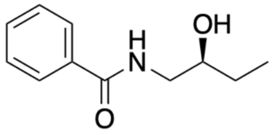

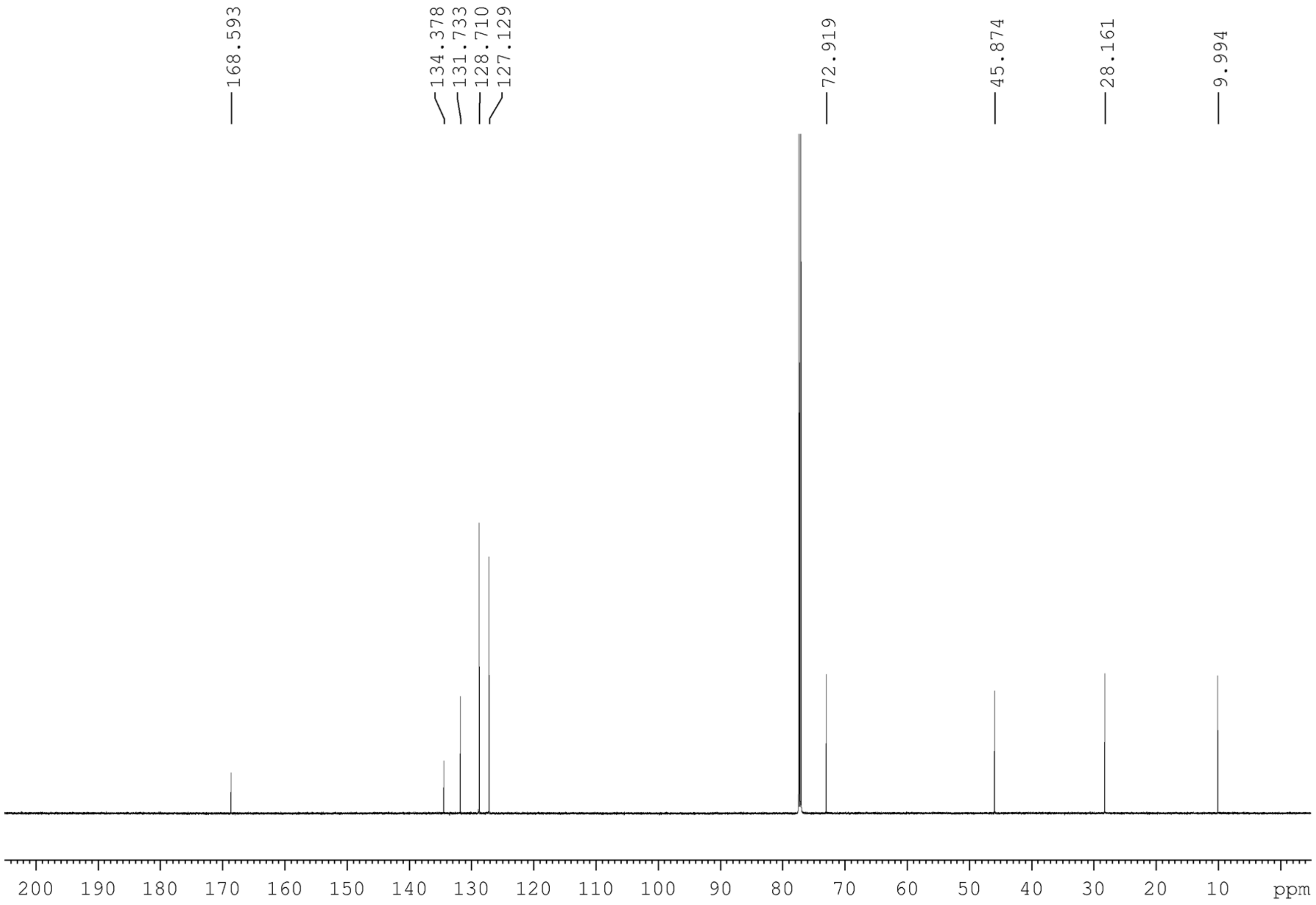

4-(*tert*-butyl)-*N*-(2-hydroxybutyl)benzamide (1zf-rac)

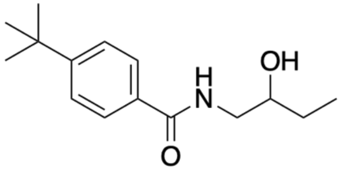

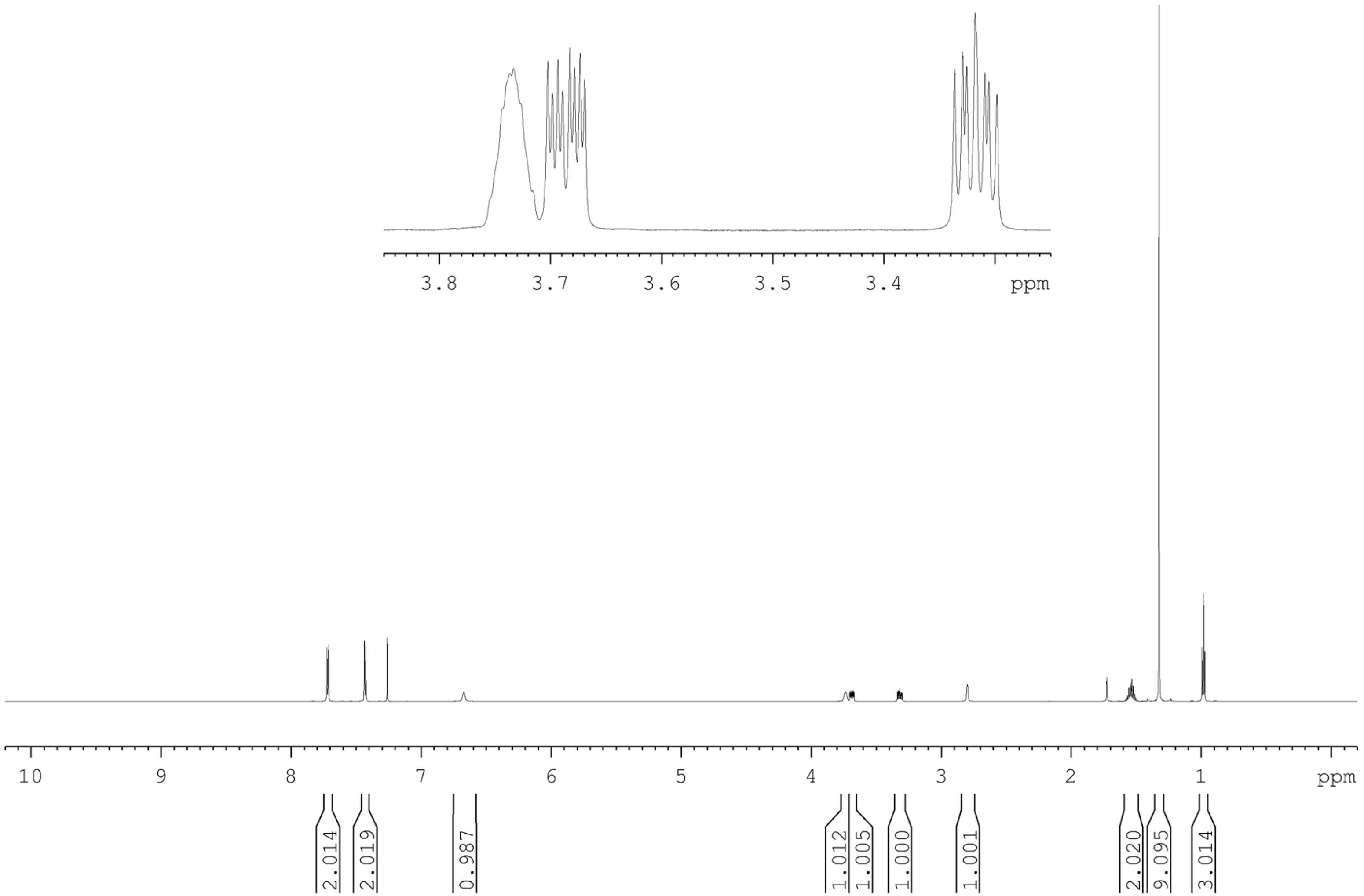

4-(*tert*-butyl)-*N*-(2-hydroxybutyl)benzamide (1zf-rac)

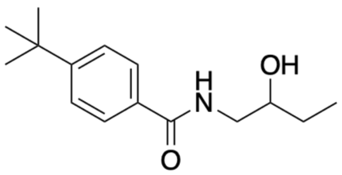

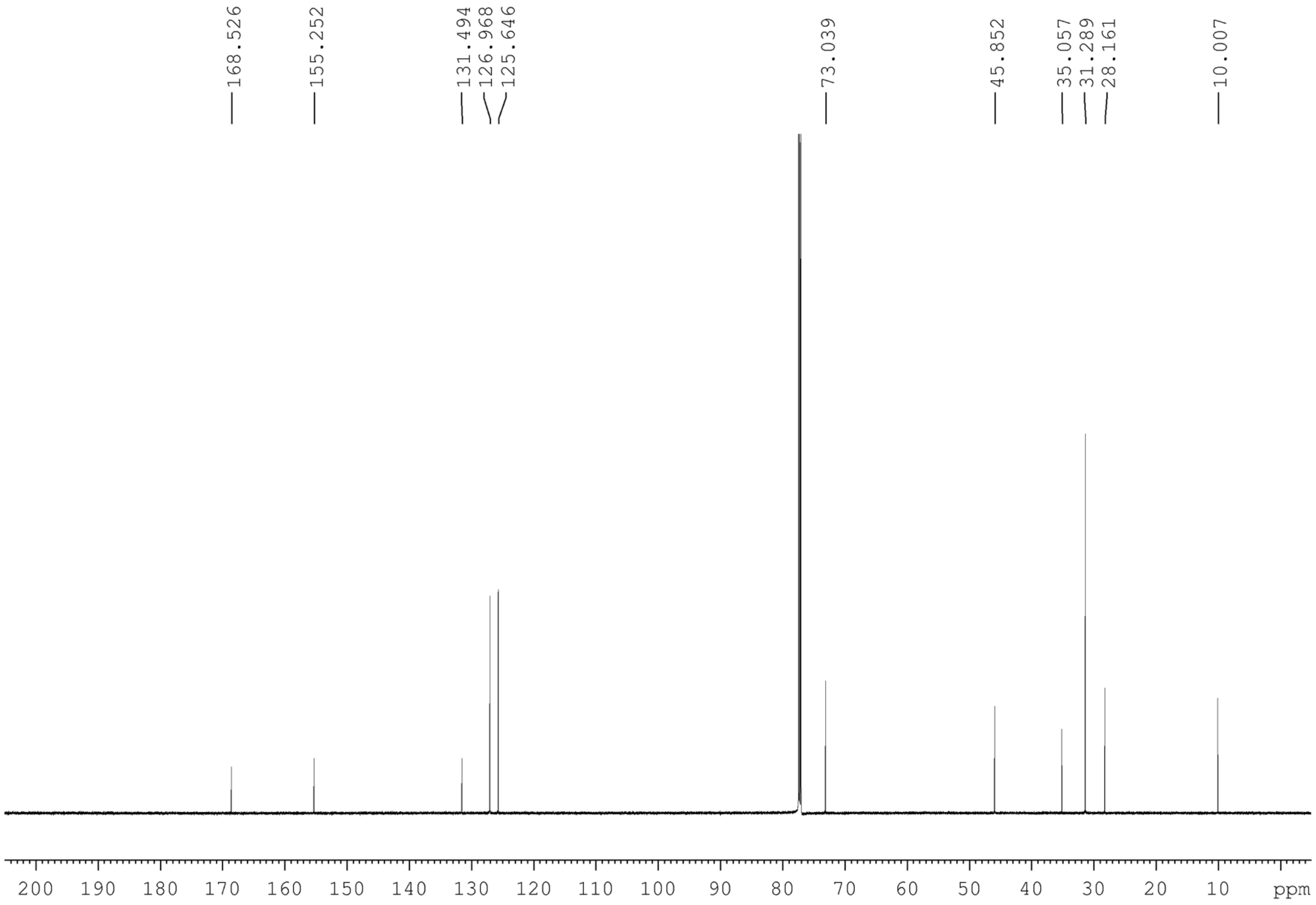

(*S*)-4-(*tert*-butyl)-*N*-(2-hydroxybutyl)benzamide (1zf)

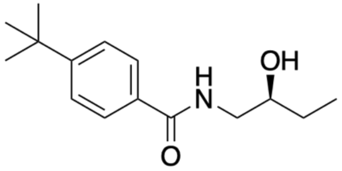

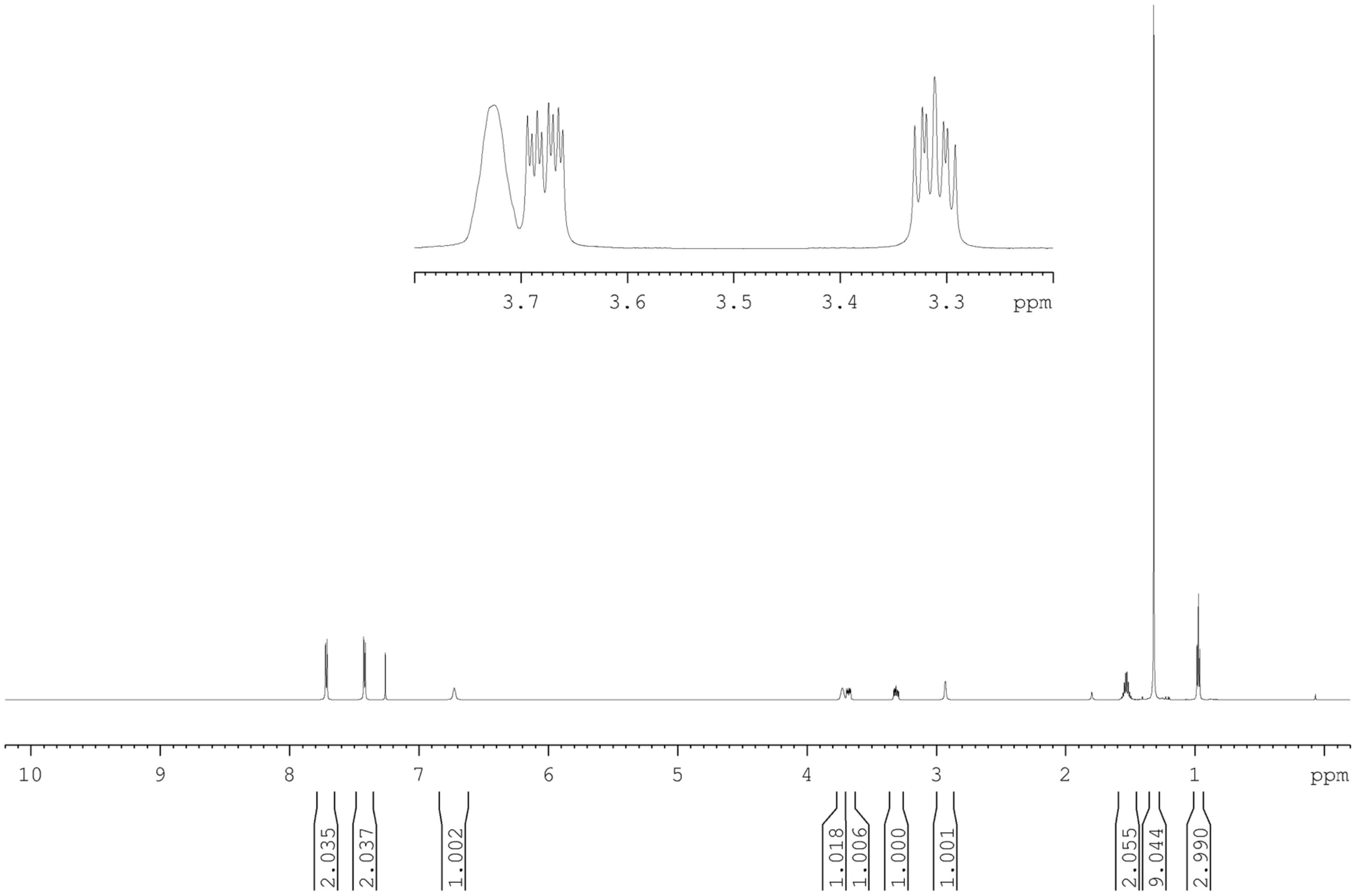

(*S*)-4-(*tert*-butyl)-*N*-(2-hydroxybutyl)benzamide (1zf)

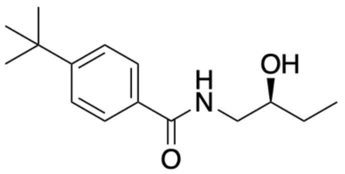

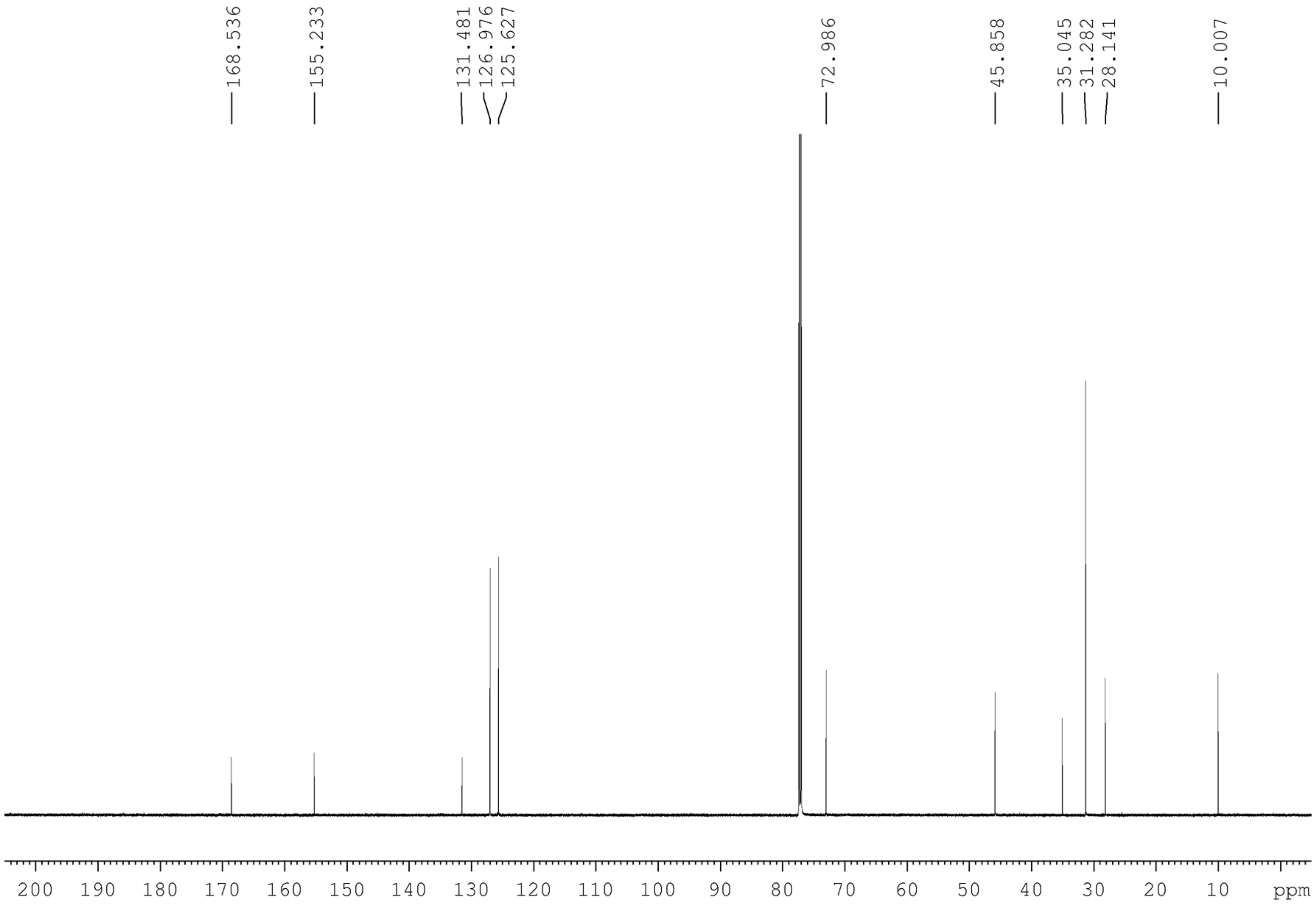

N-(2-hydroxybutyl)-4-(trifluoromethyl)benzamide (1zg-rac)

In $(CD_3)_2CO$

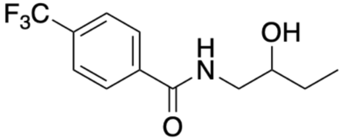

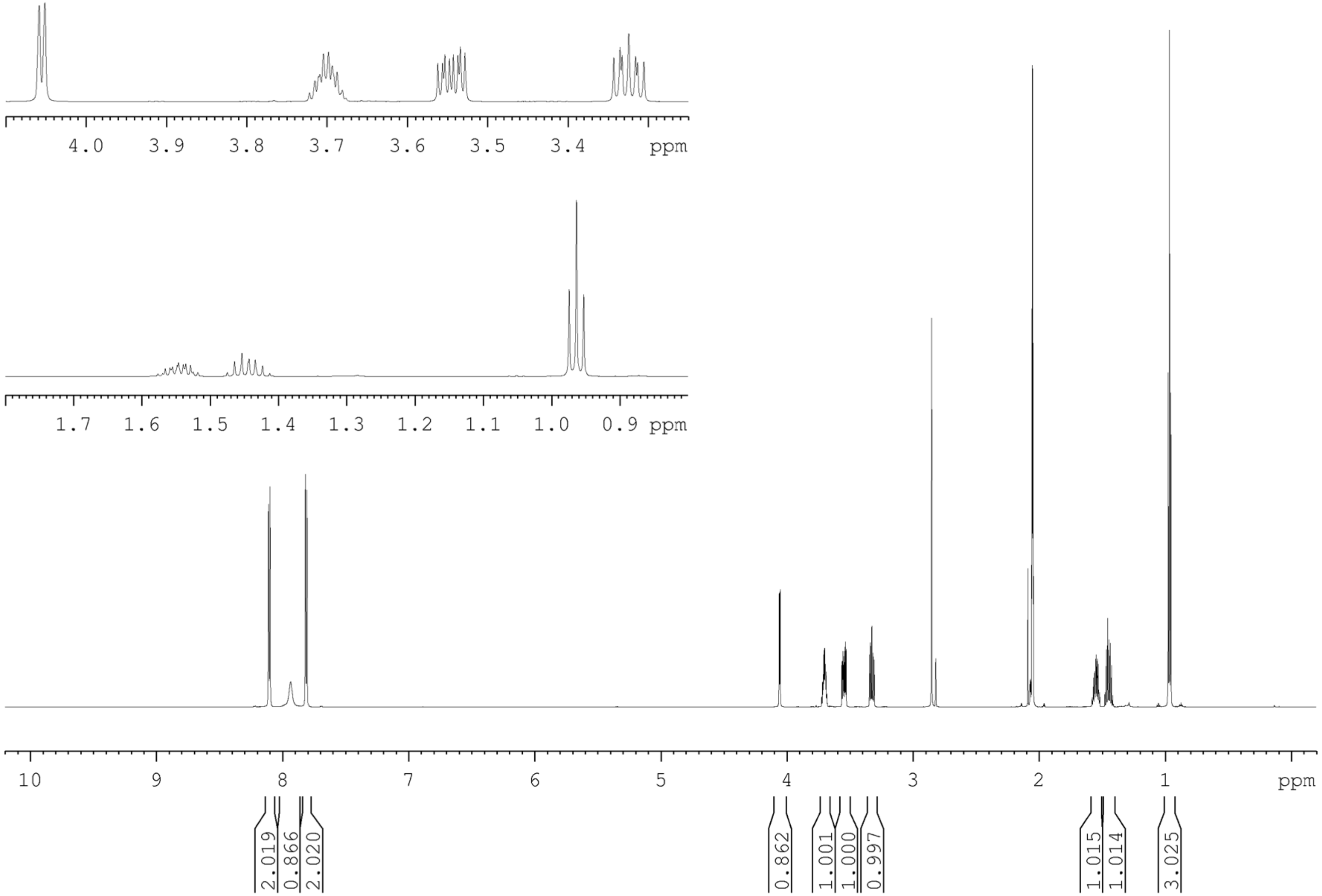

N-(2-hydroxybutyl)-4-(trifluoromethyl)benzamide (1zg-rac)

In $(CD_3)_2CO$

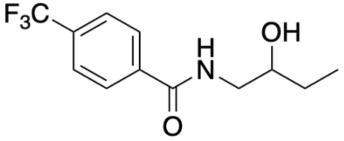

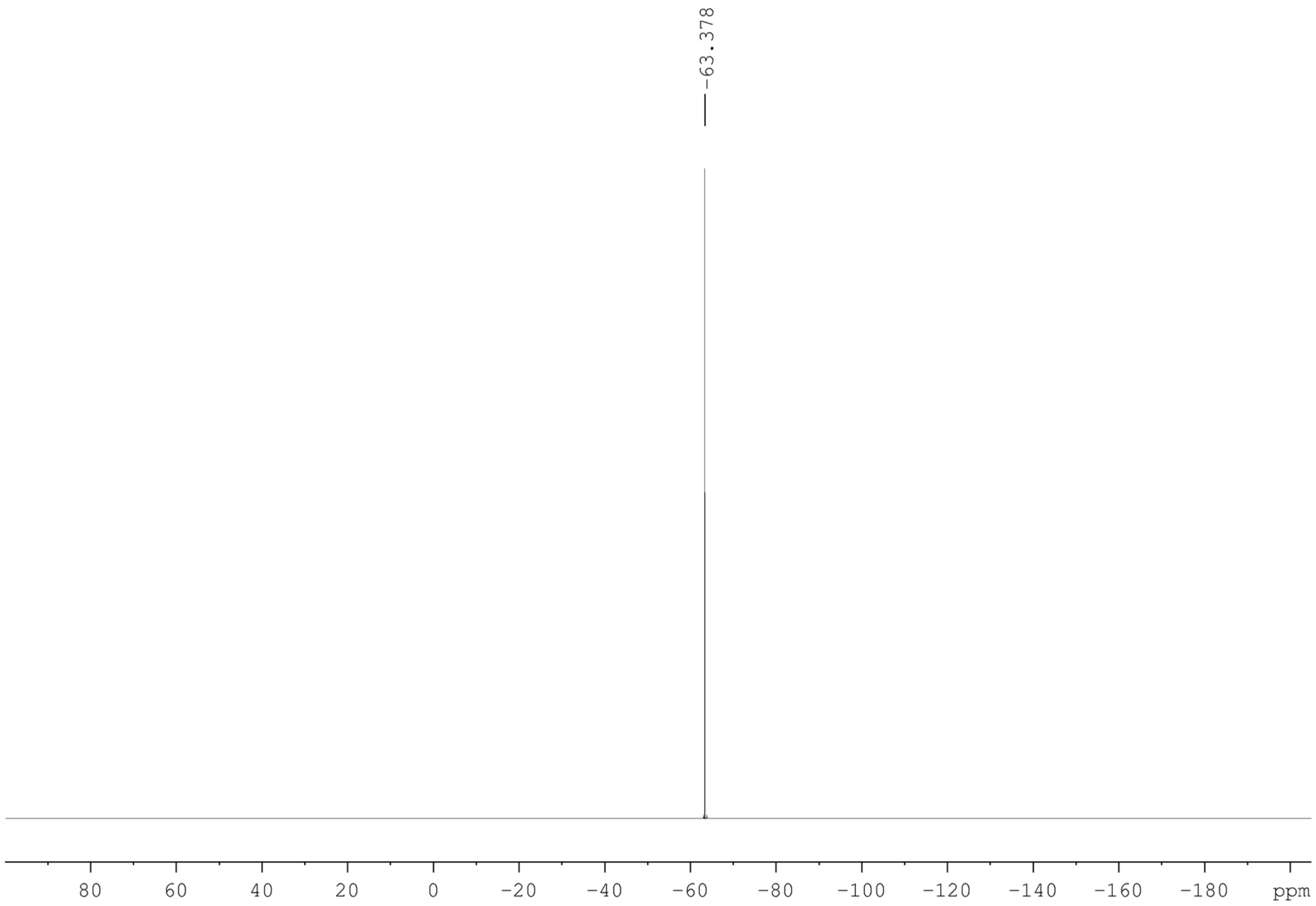

N-(2-hydroxybutyl)-4-(trifluoromethyl)benzamide (1zg-rac)

In $(CD_3)_2CO$

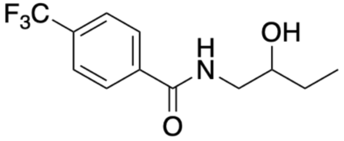

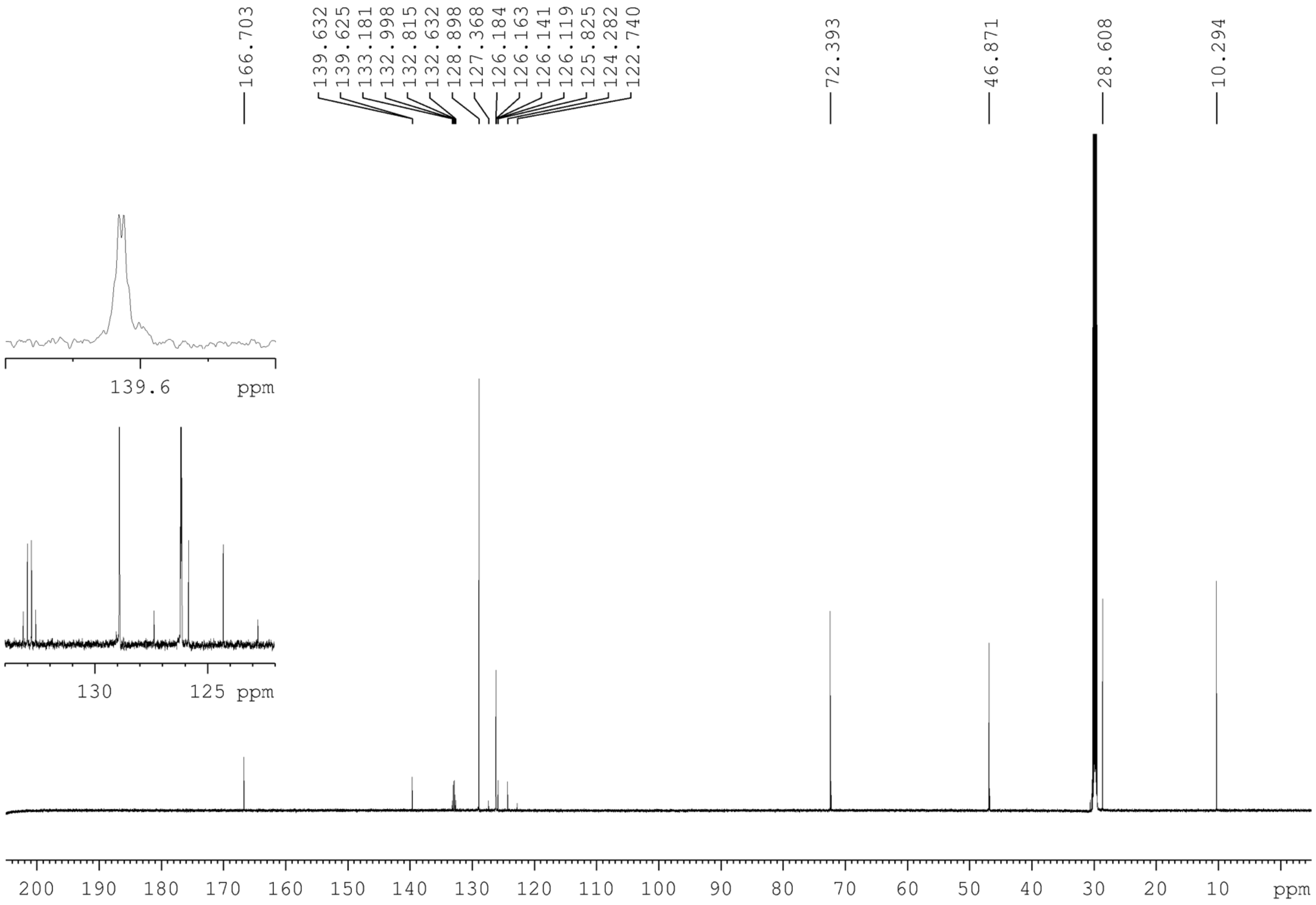

(*S*)-*N*-(2-hydroxybutyl)-4-(trifluoromethyl)benzamide (1zg)

In $(CD_3)_2CO$

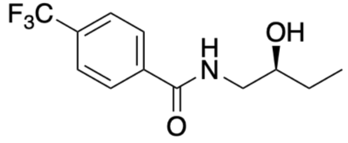

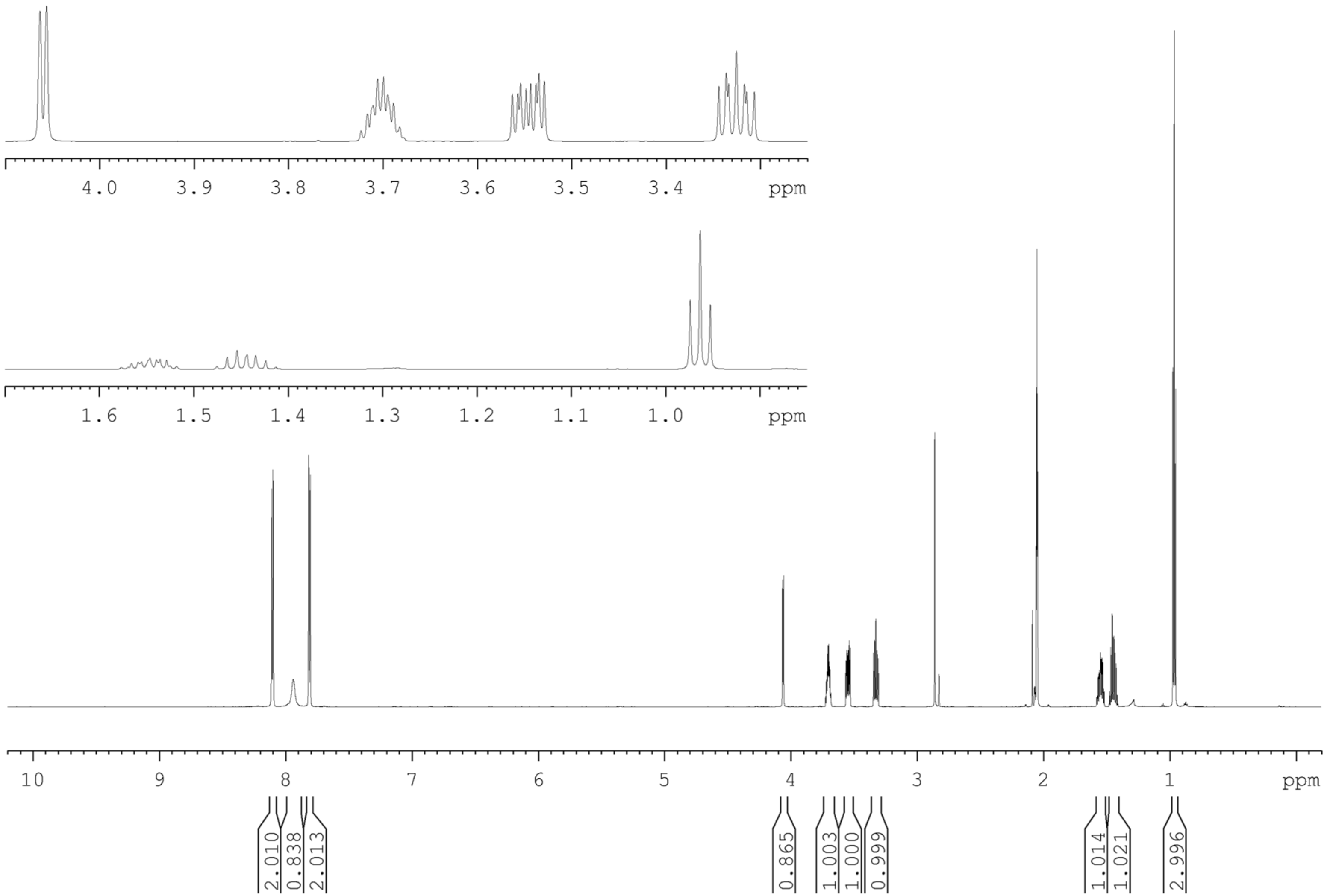

(*S*)-*N*-(2-hydroxybutyl)-4-(trifluoromethyl)benzamide (1zg)

In $(CD_3)_2CO$

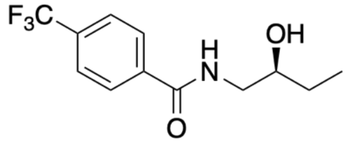

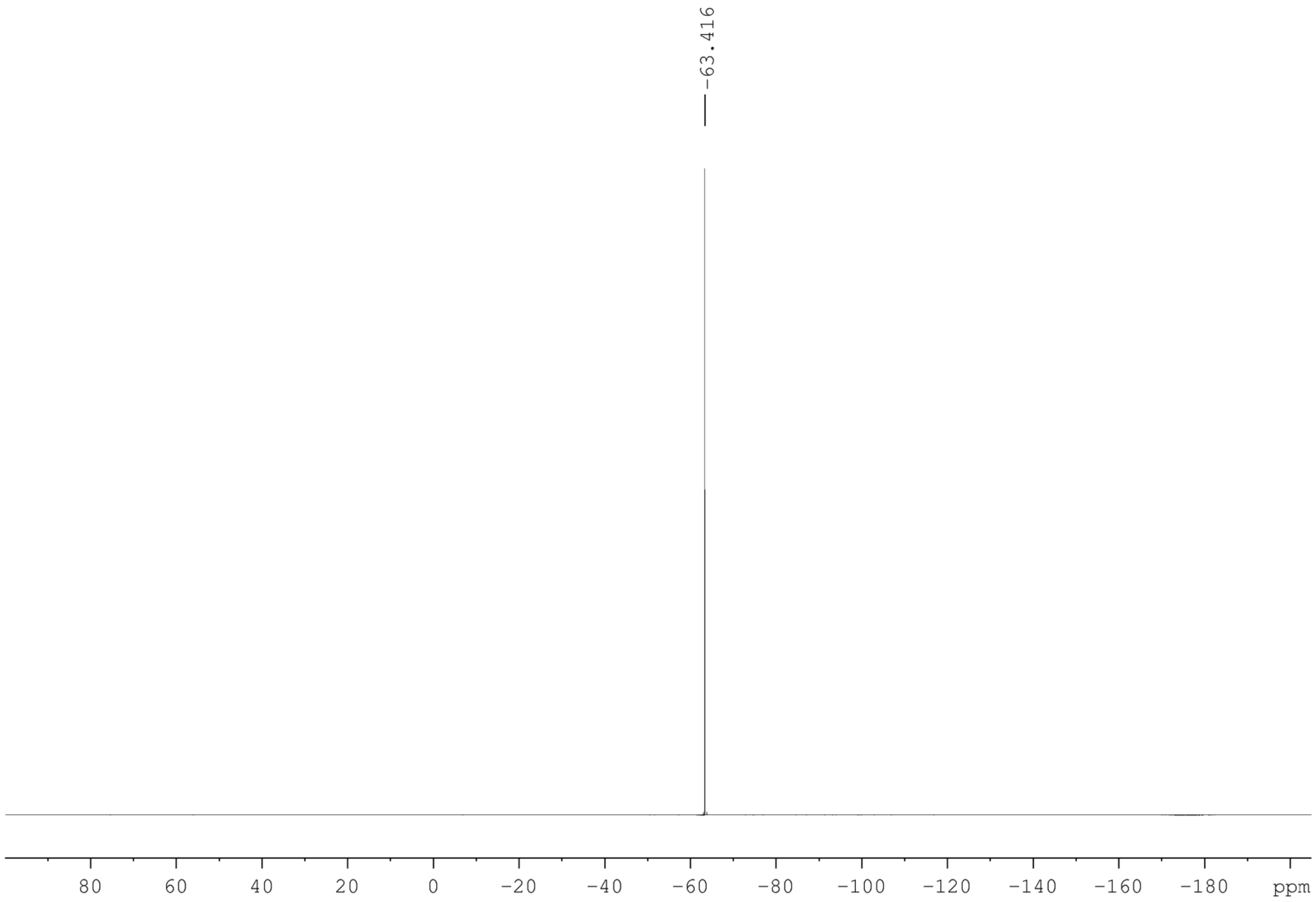

(*S*)-*N*-(2-hydroxybutyl)-4-(trifluoromethyl)benzamide (1zg)

In $(CD_3)_2CO$

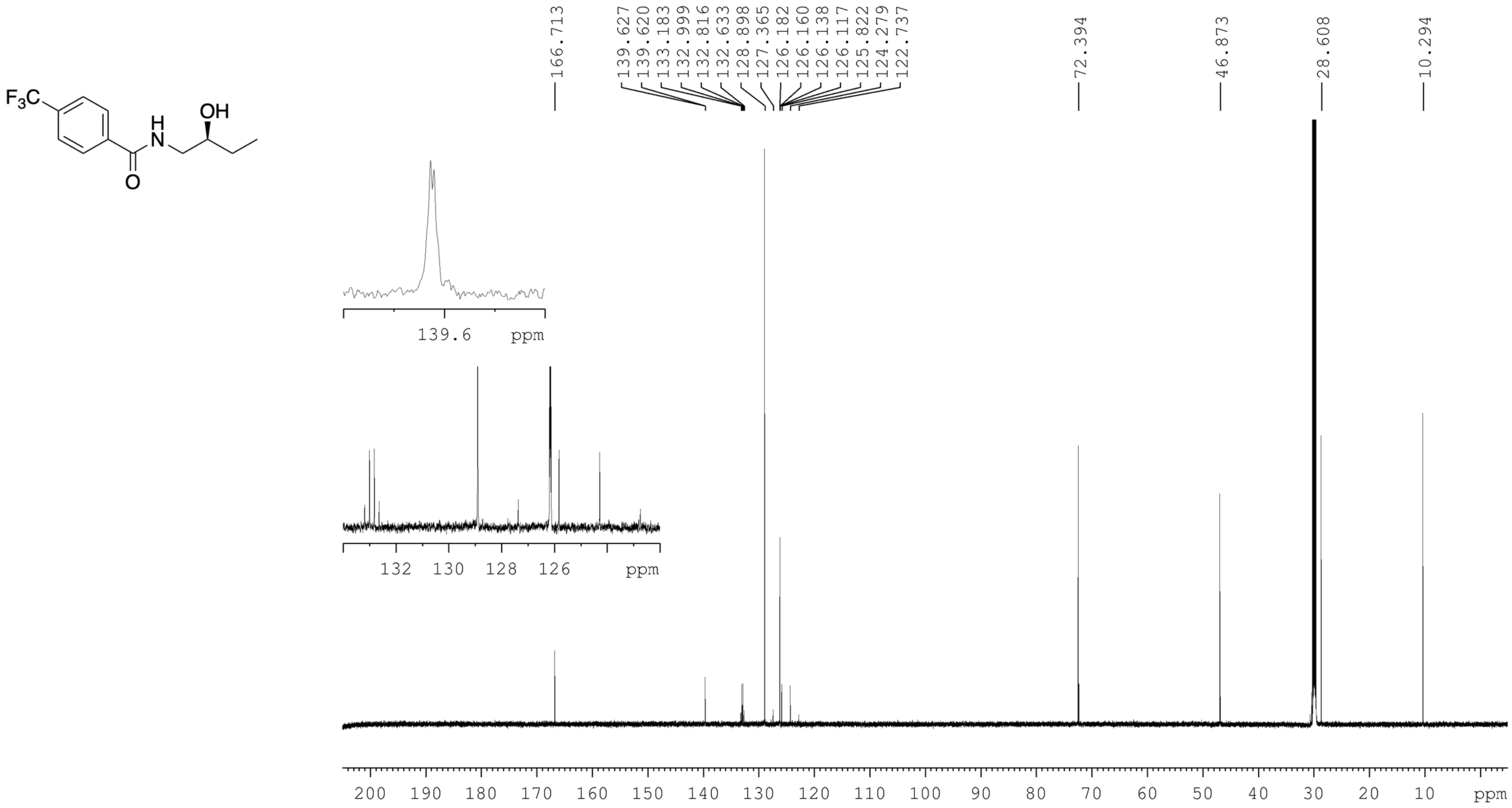

_N_-(2-hydroxybutyl)-4-methoxybenzamide (1zh-rac)

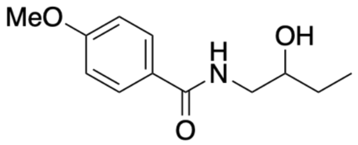

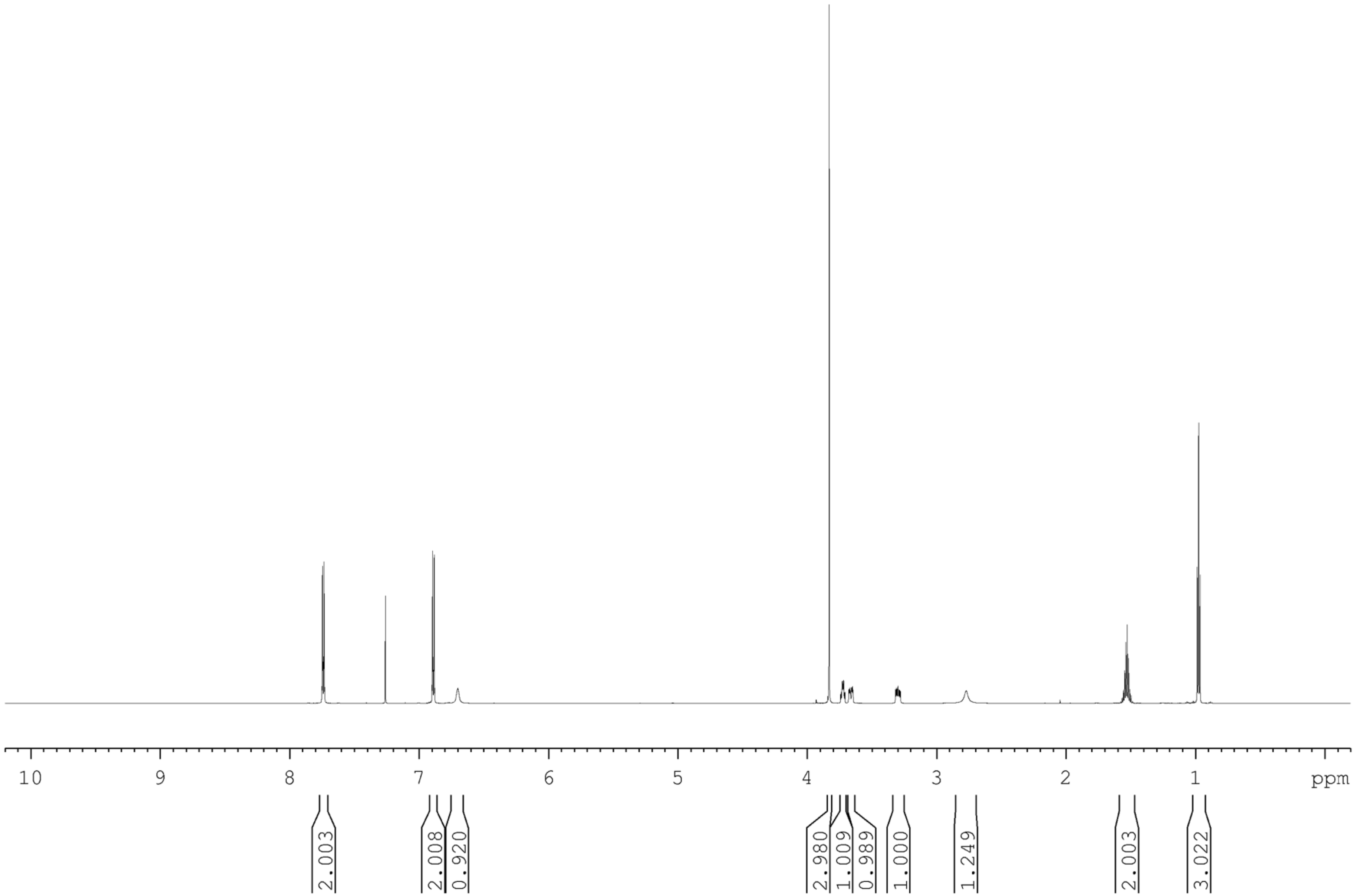

N-(2-hydroxybutyl)-4-methoxybenzamide (1zh-rac)

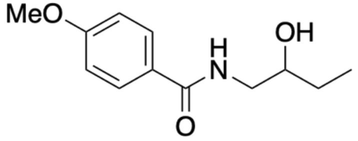

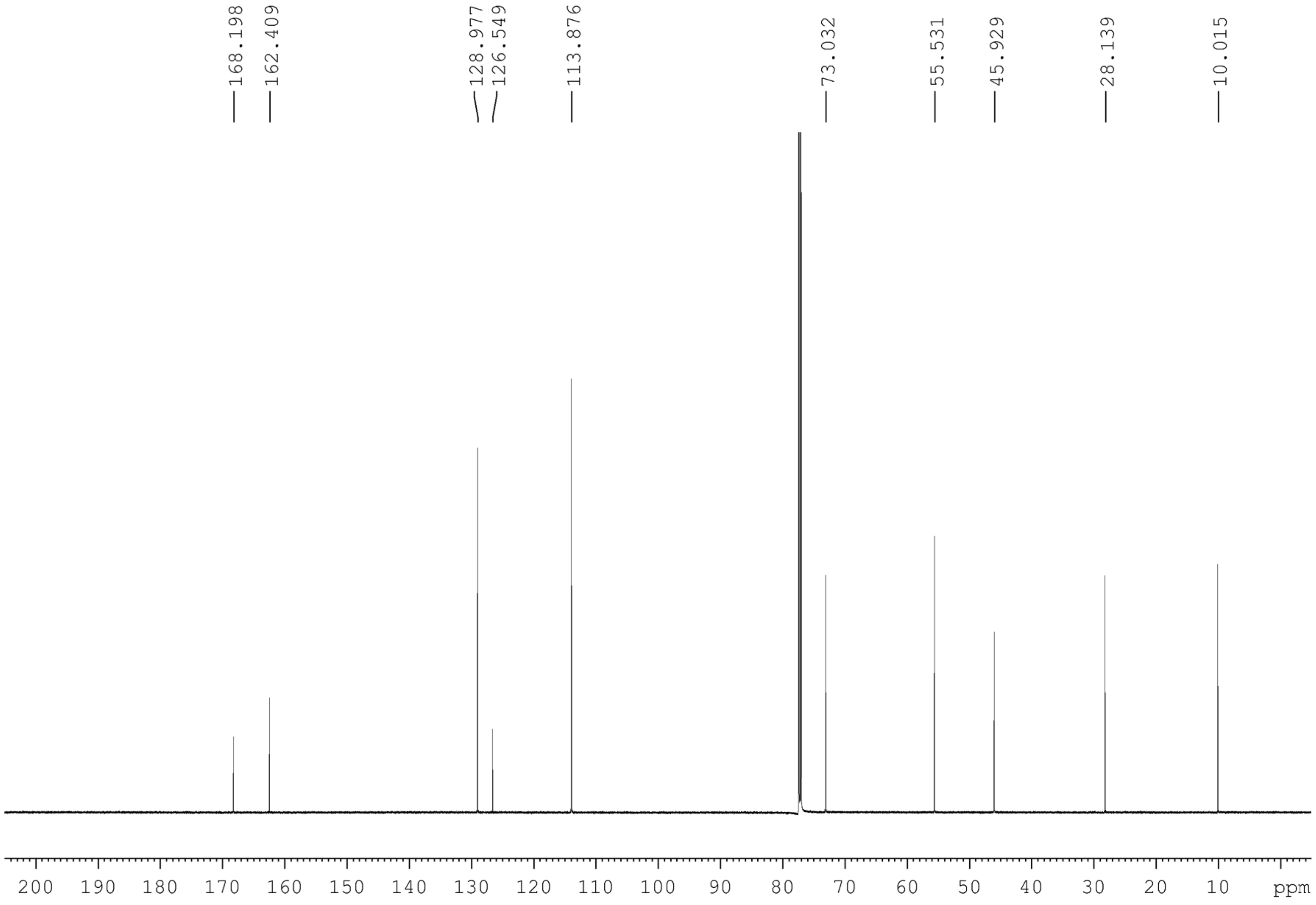

(*S*)-*N*-(2-hydroxybutyl)-4-methoxybenzamide (1zh)

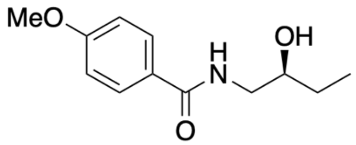

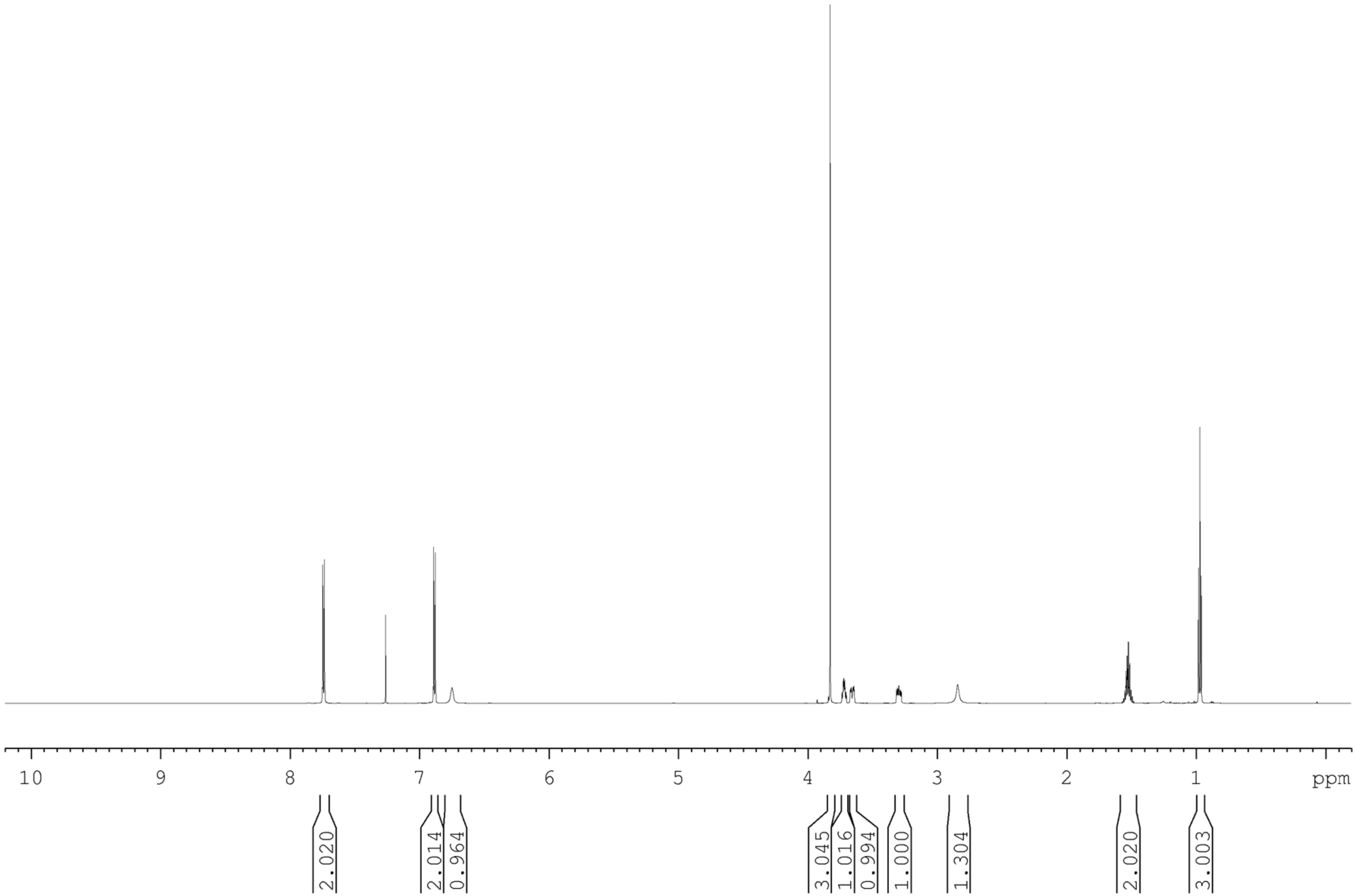

(*S*)-*N*-(2-hydroxybutyl)-4-methoxybenzamide (1zh)

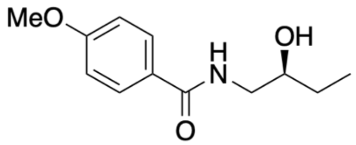

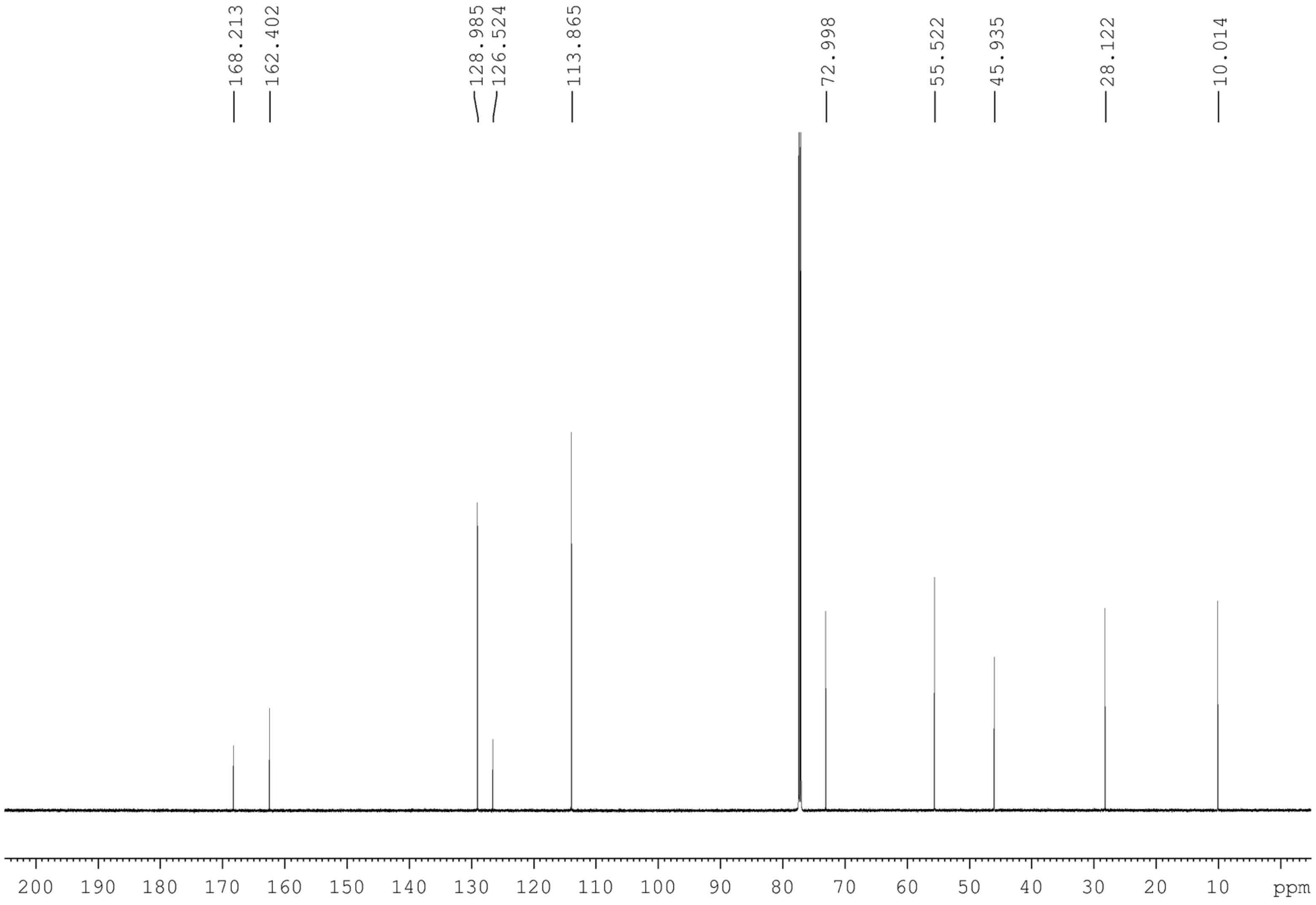

N-(2-hydroxybutyl)benzo[*d*][1,3]dioxole-5-carboxamide (1zi-rac)

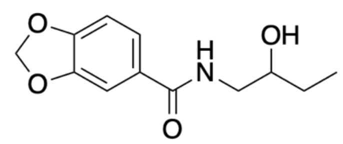

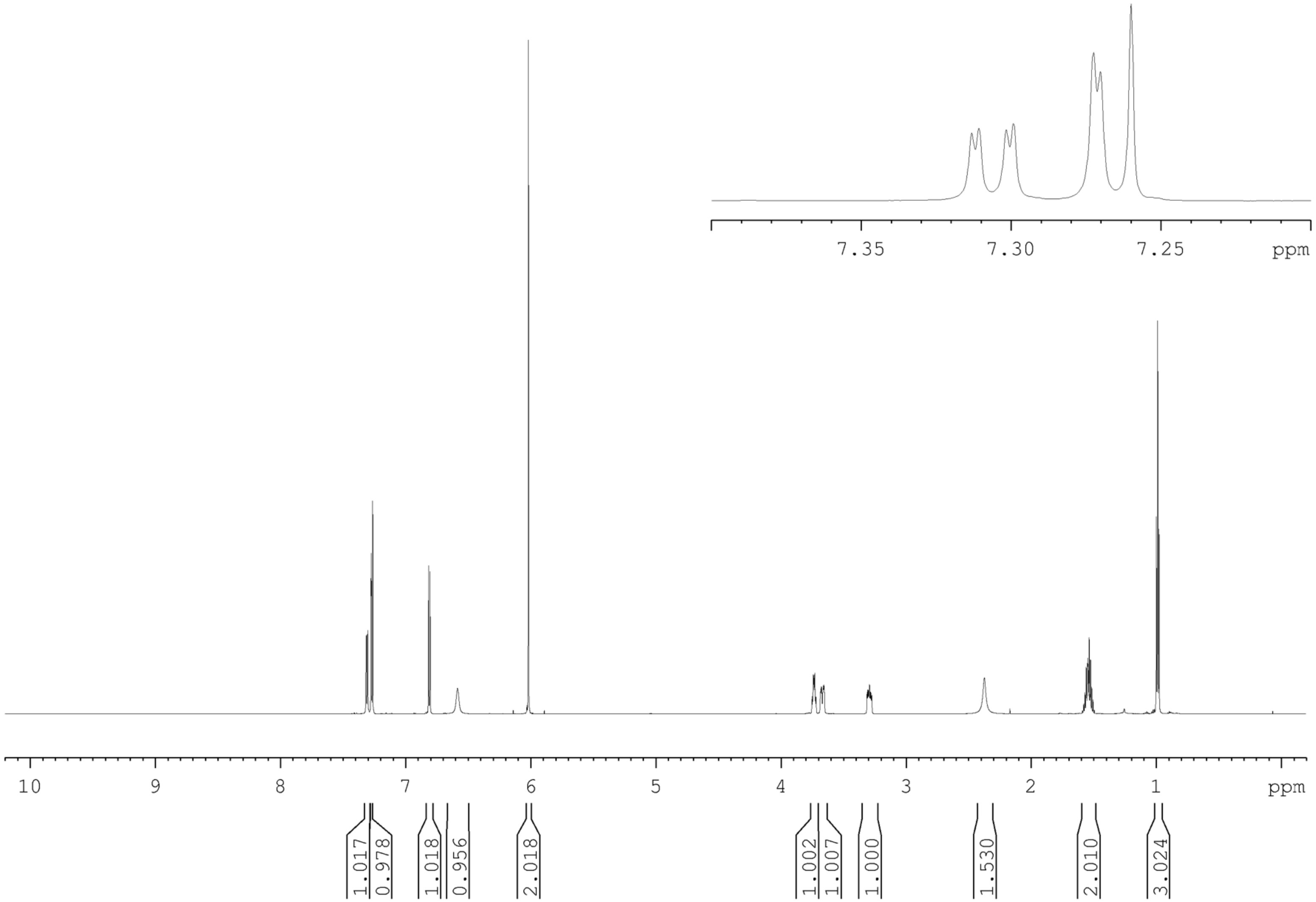

N-(2-hydroxybutyl)benzo[*d*][1,3]dioxole-5-carboxamide (1zi-rac)

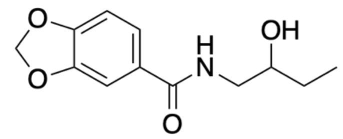

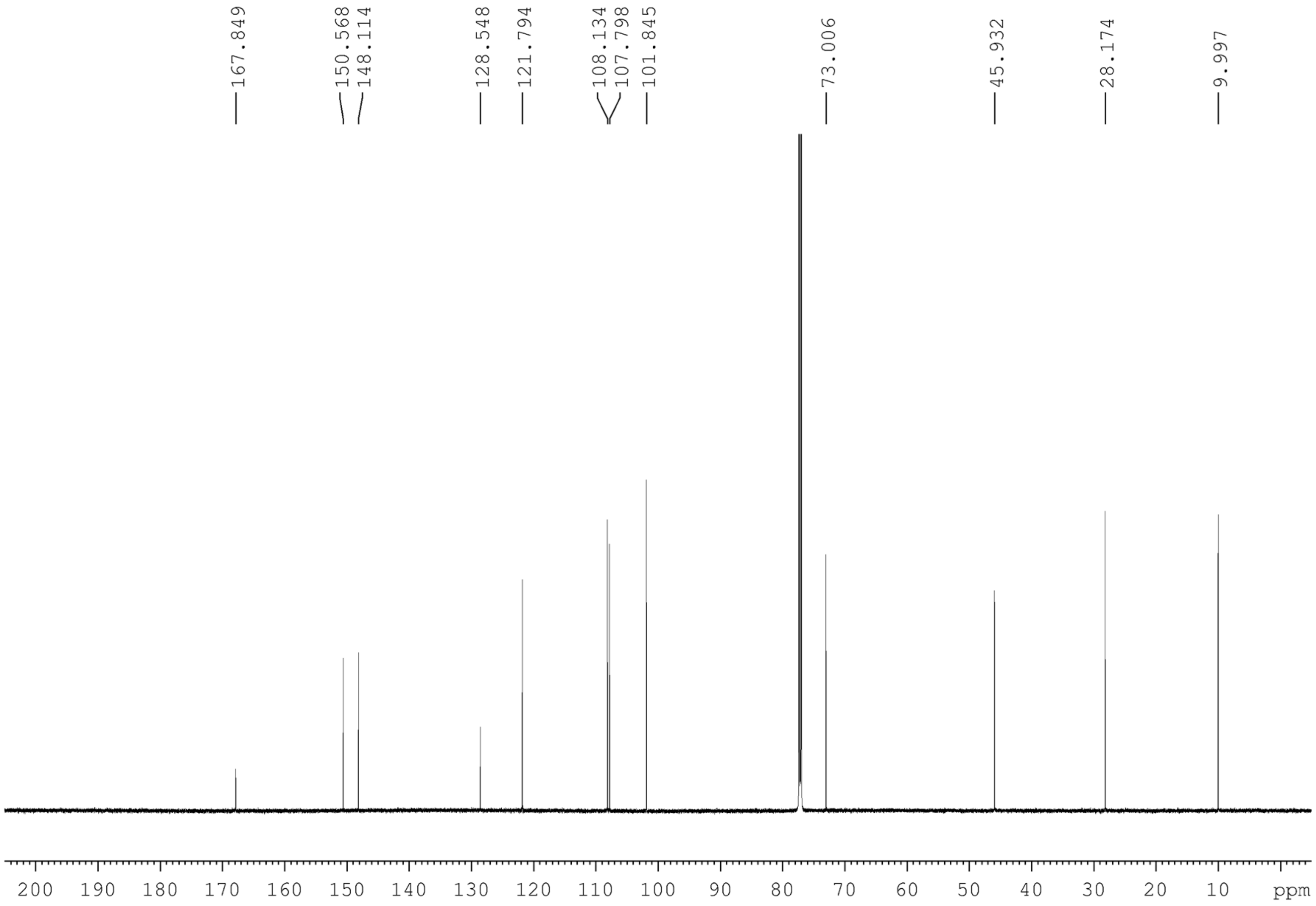

(*S*)-*N*-(2-hydroxybutyl)benzo[*d*][1,3]dioxole-5-carboxamide (1zi)

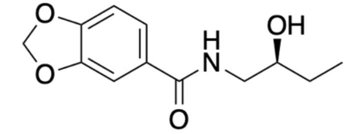

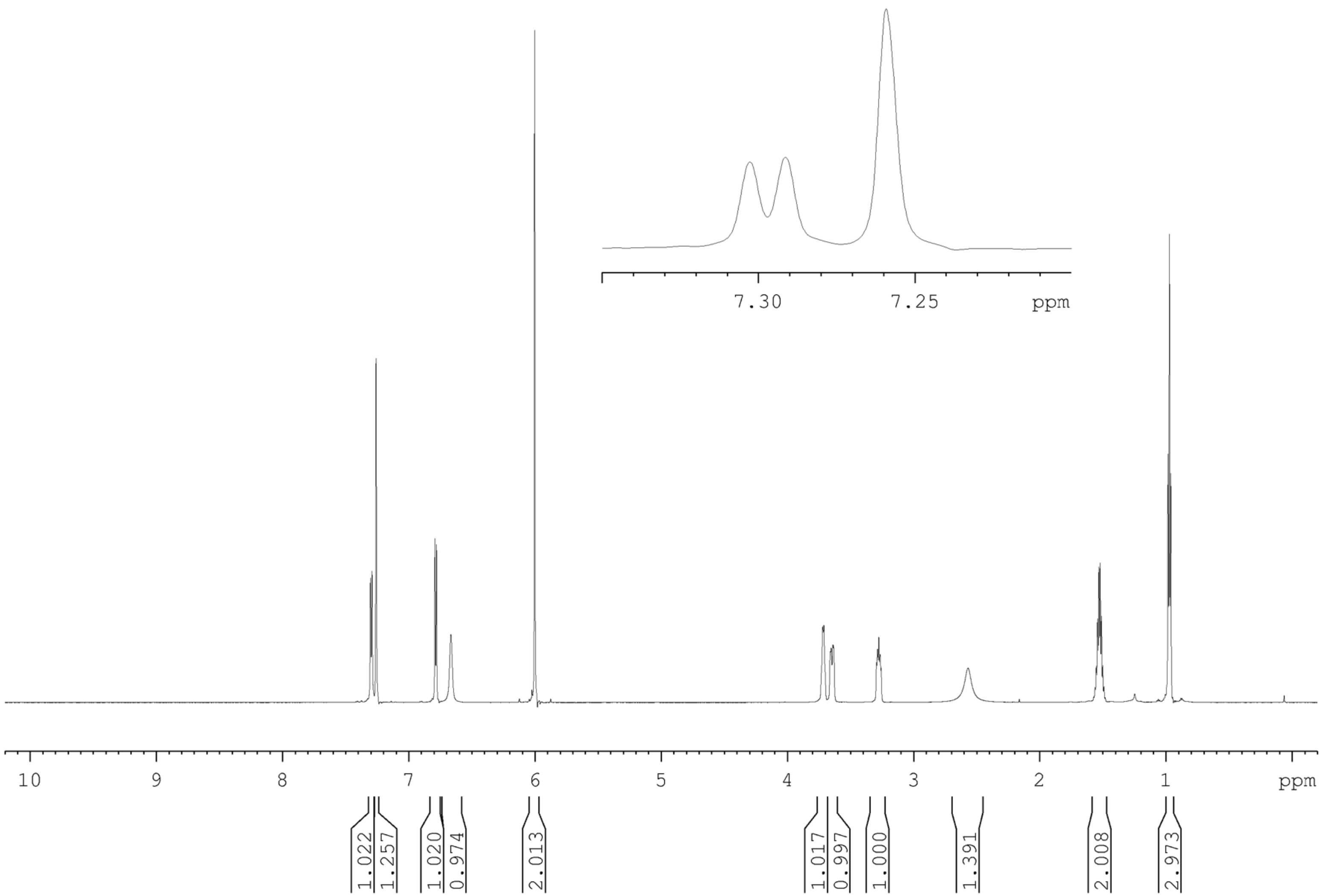

(*S*)-*N*-(2-hydroxybutyl)benzo[*d*][1,3]dioxole-5-carboxamide (1zi)

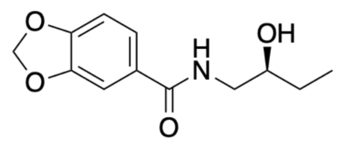

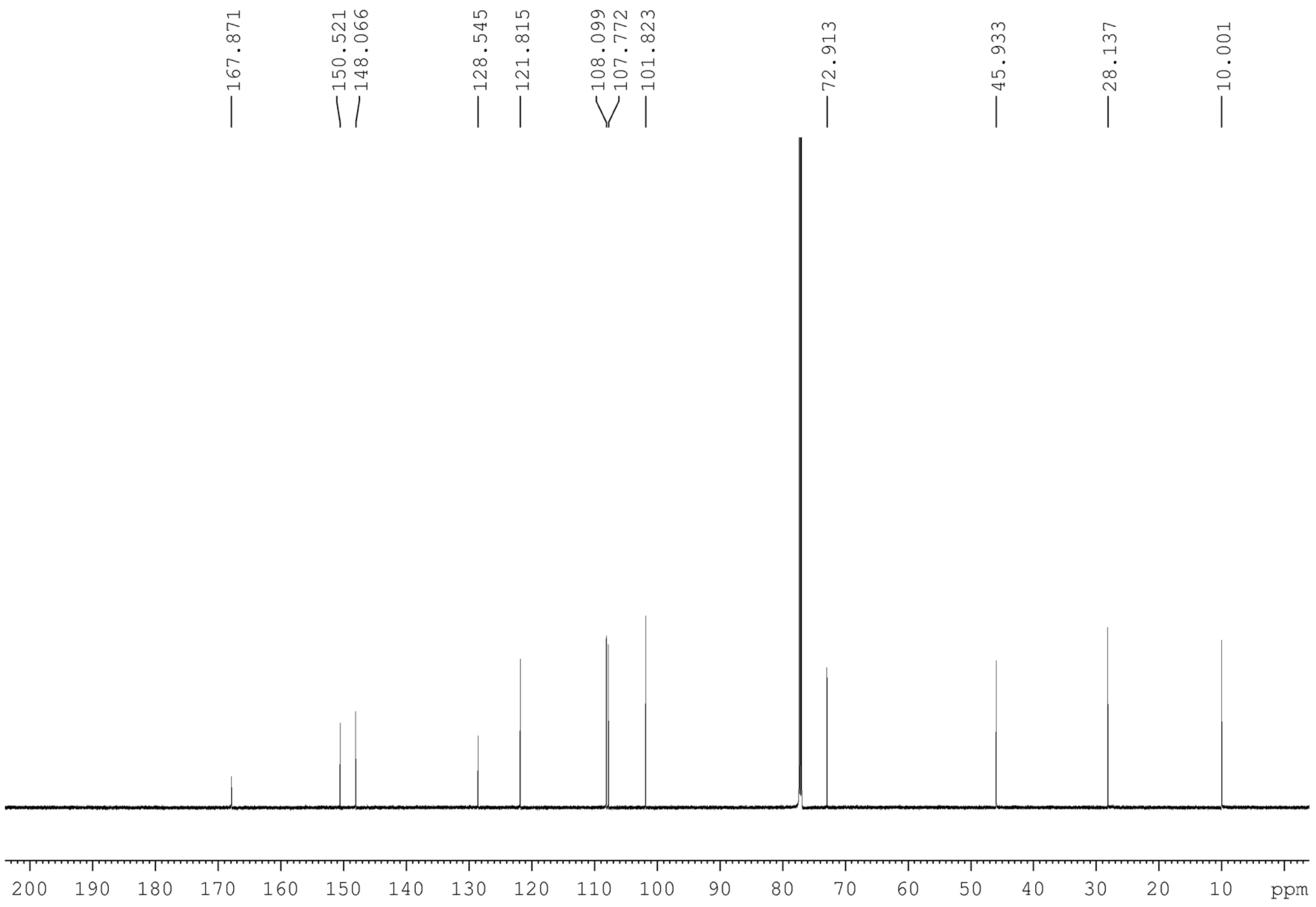

N-(2-hydroxybutyl)furan-3-carboxamide (1zj-rac)

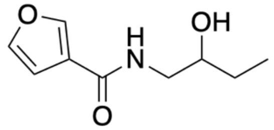

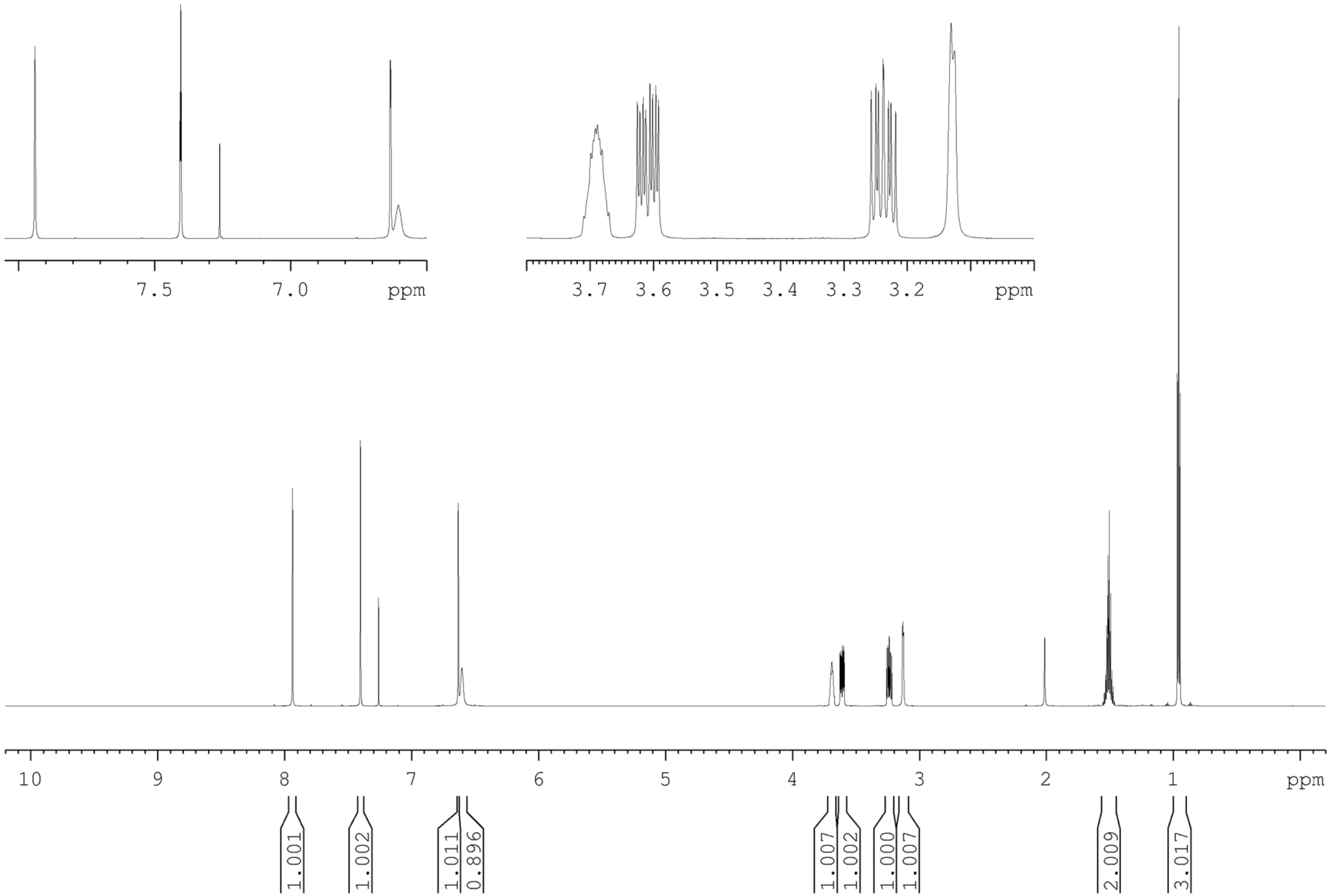

_N_-(2-hydroxybutyl)furan-3-carboxamide (1zj-rac)

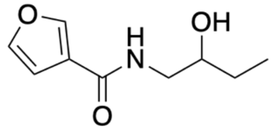

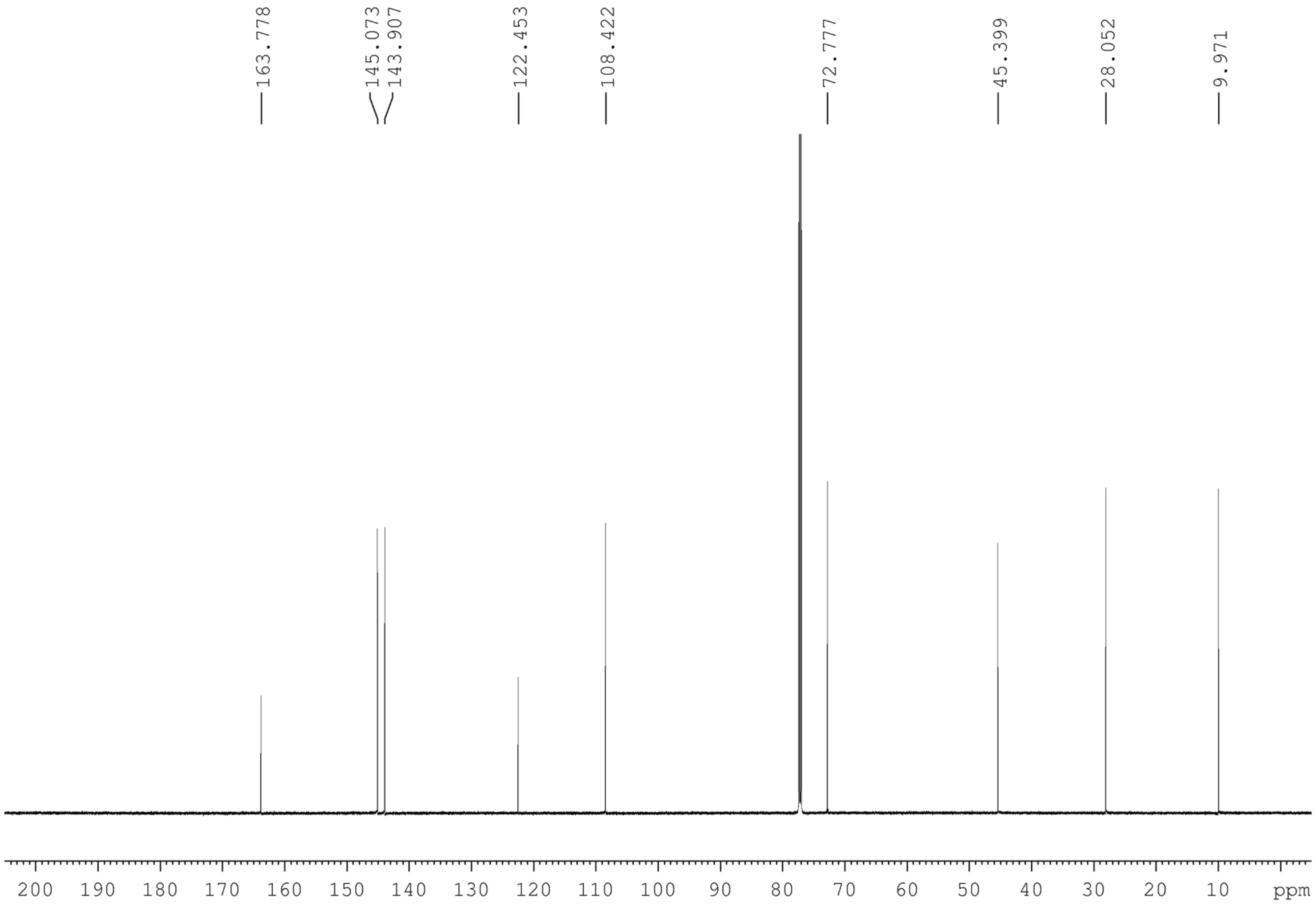

(*S*)-*N*-(2-hydroxybutyl)furan-3-carboxamide (1zj)

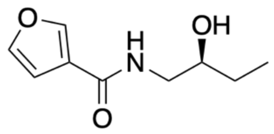

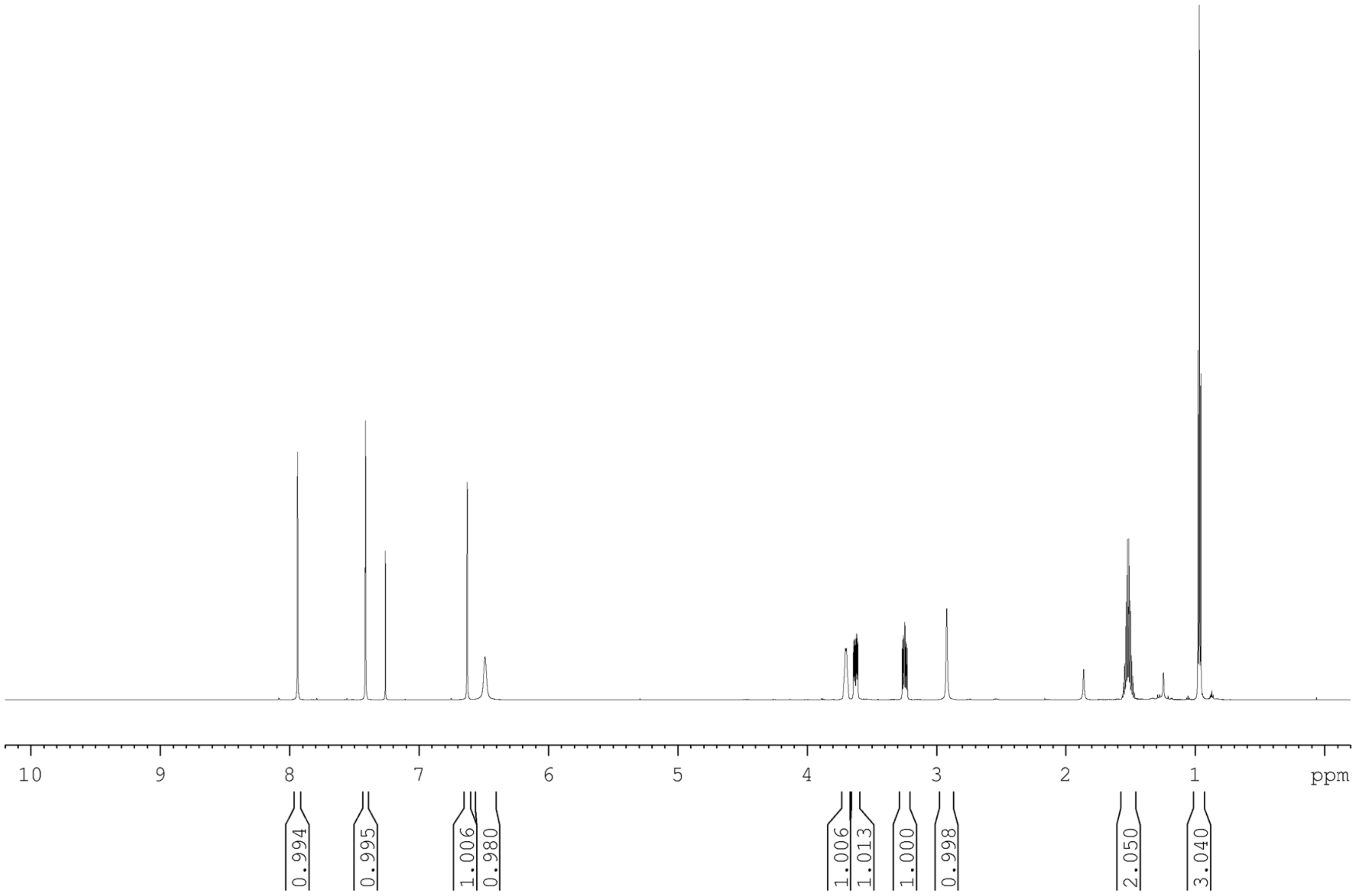

(*S*)-*N*-(2-hydroxybutyl)furan-3-carboxamide (1zj)

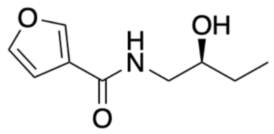

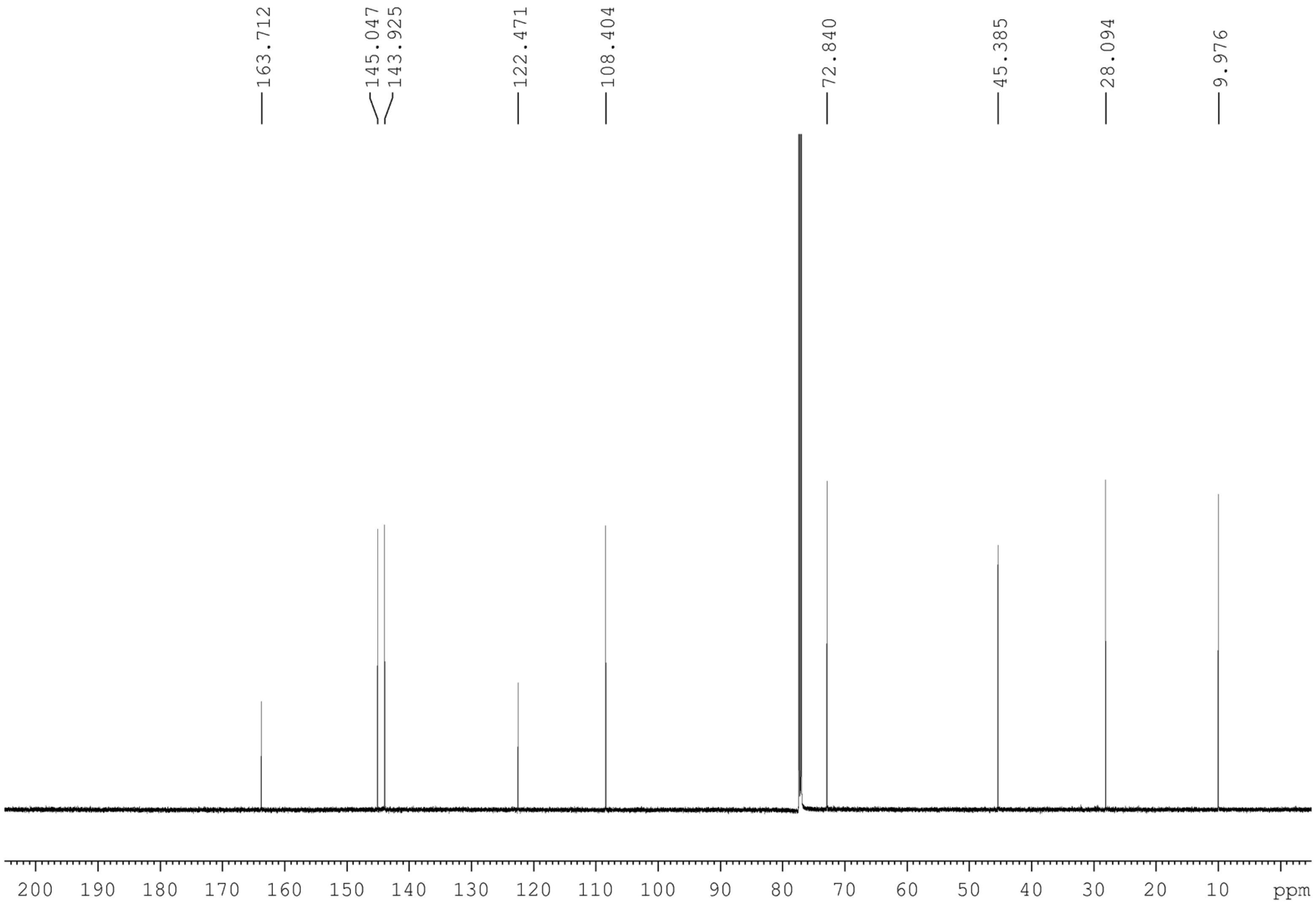

N-(2-hydroxybutyl)-1-methyl-1H-pyrazole-4-carboxamide (1zk-rac)

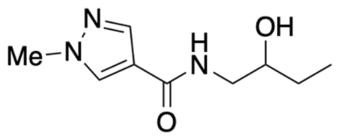

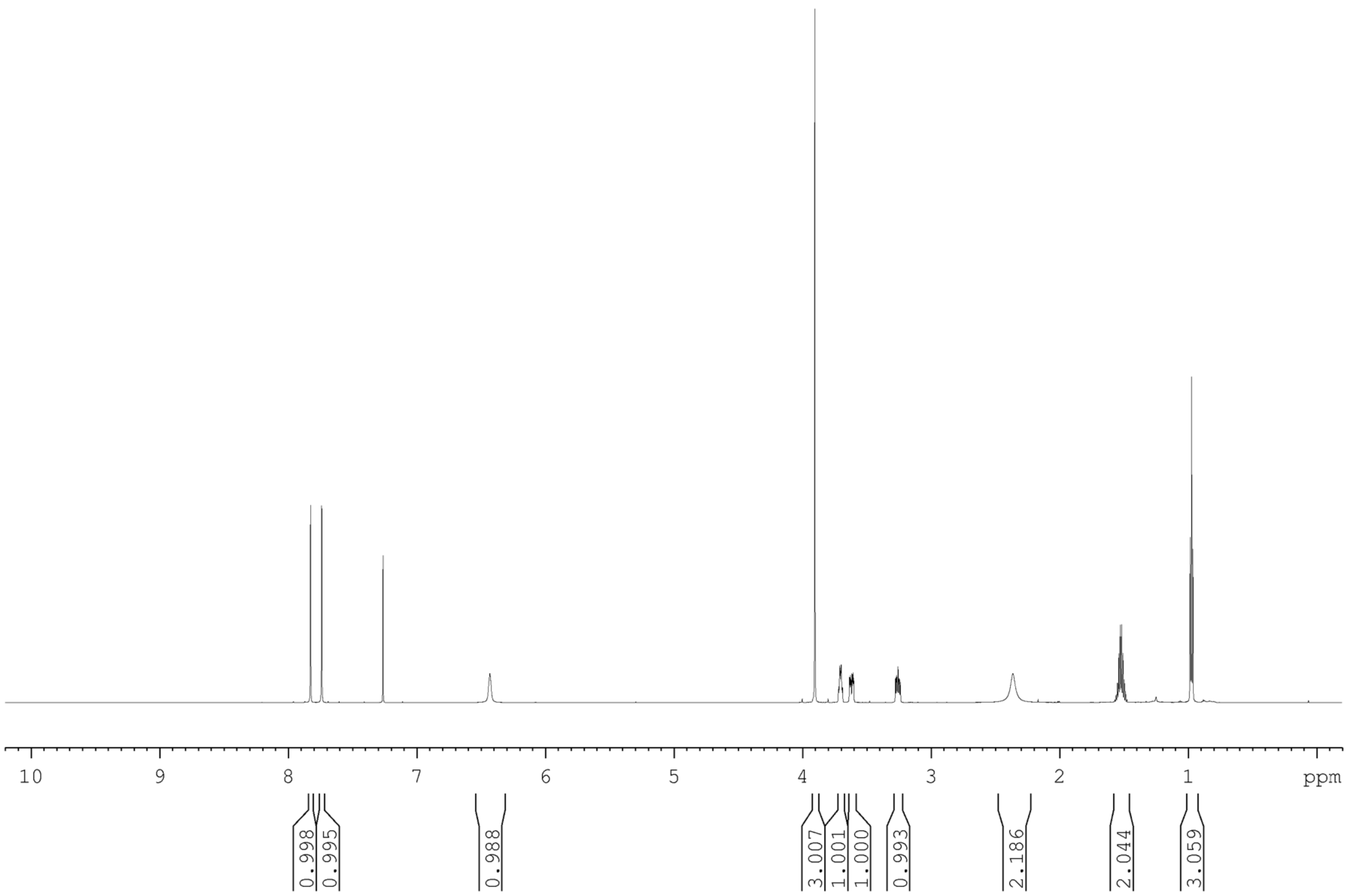

N-(2-hydroxybutyl)-1-methyl-1H-pyrazole-4-carboxamide (1zk-rac)

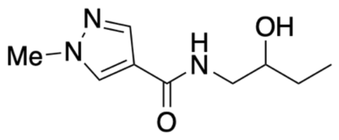

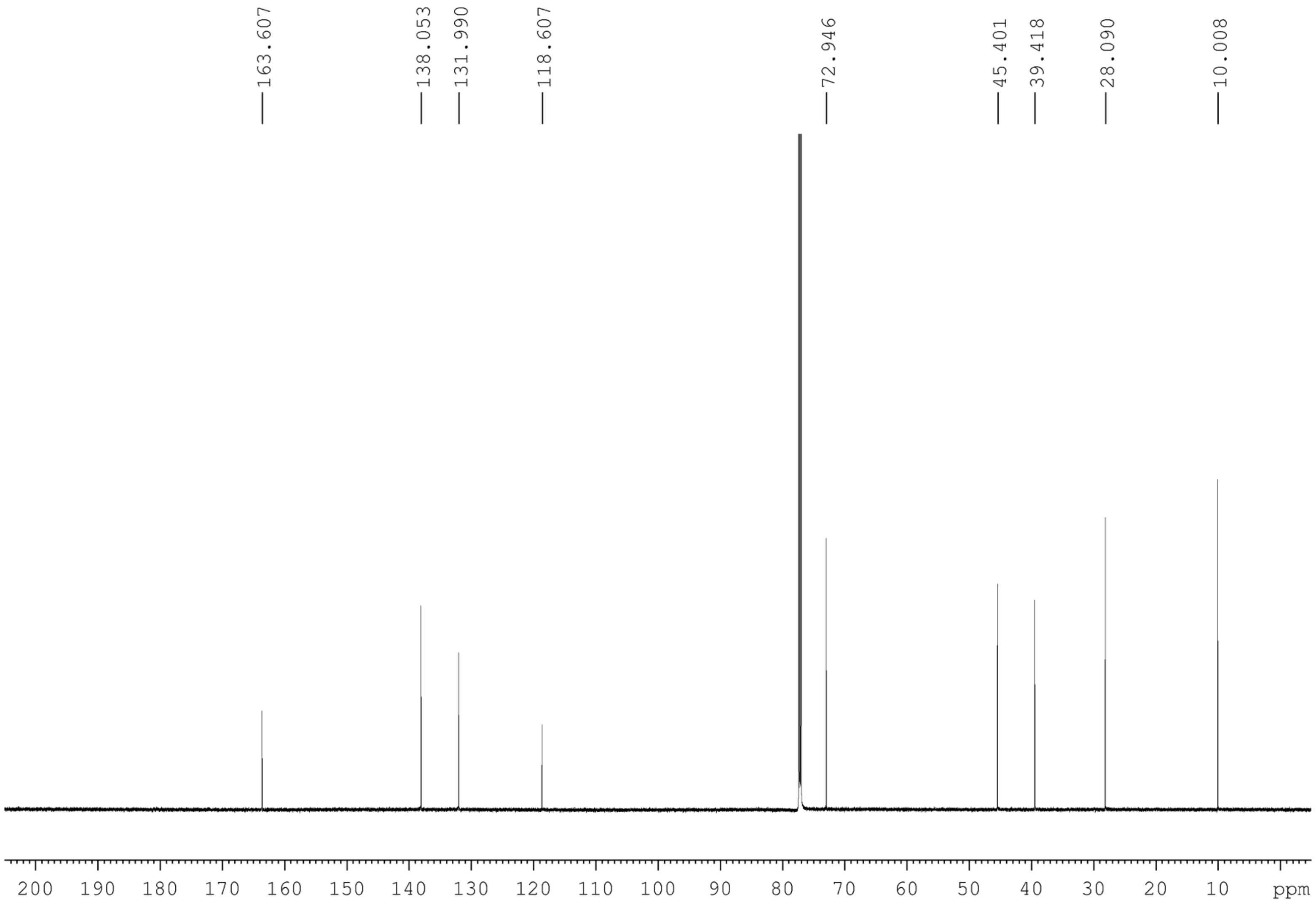

(*S*)-*N*-(2-hydroxybutyl)-1-methyl-1*H*-pyrazole-4-carboxamide (1zk)

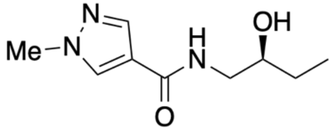

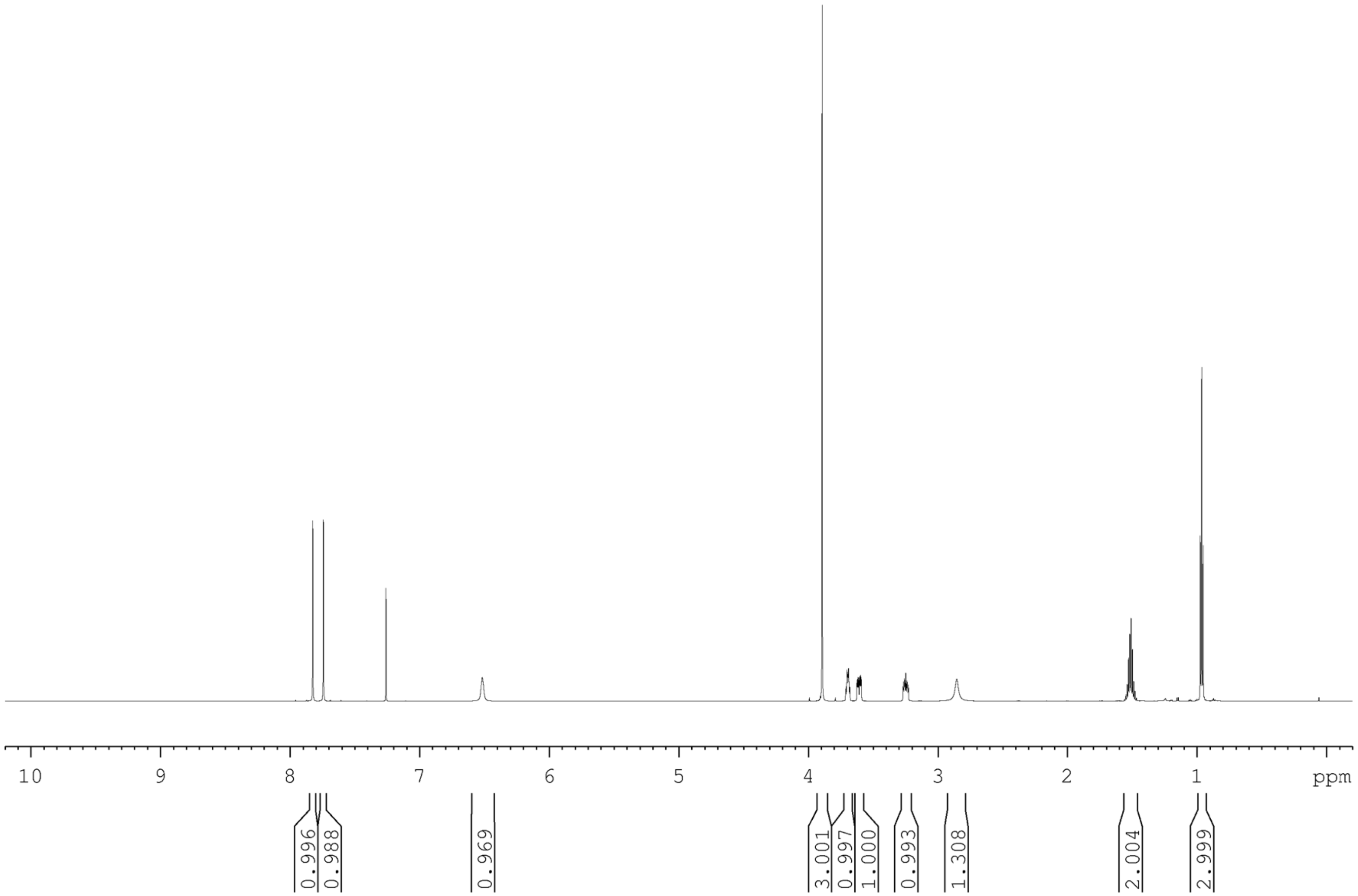

(*S*)-*N*-(2-hydroxybutyl)-1-methyl-1*H*-pyrazole-4-carboxamide (1zk)

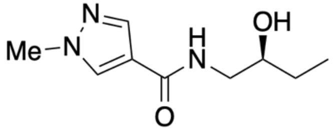

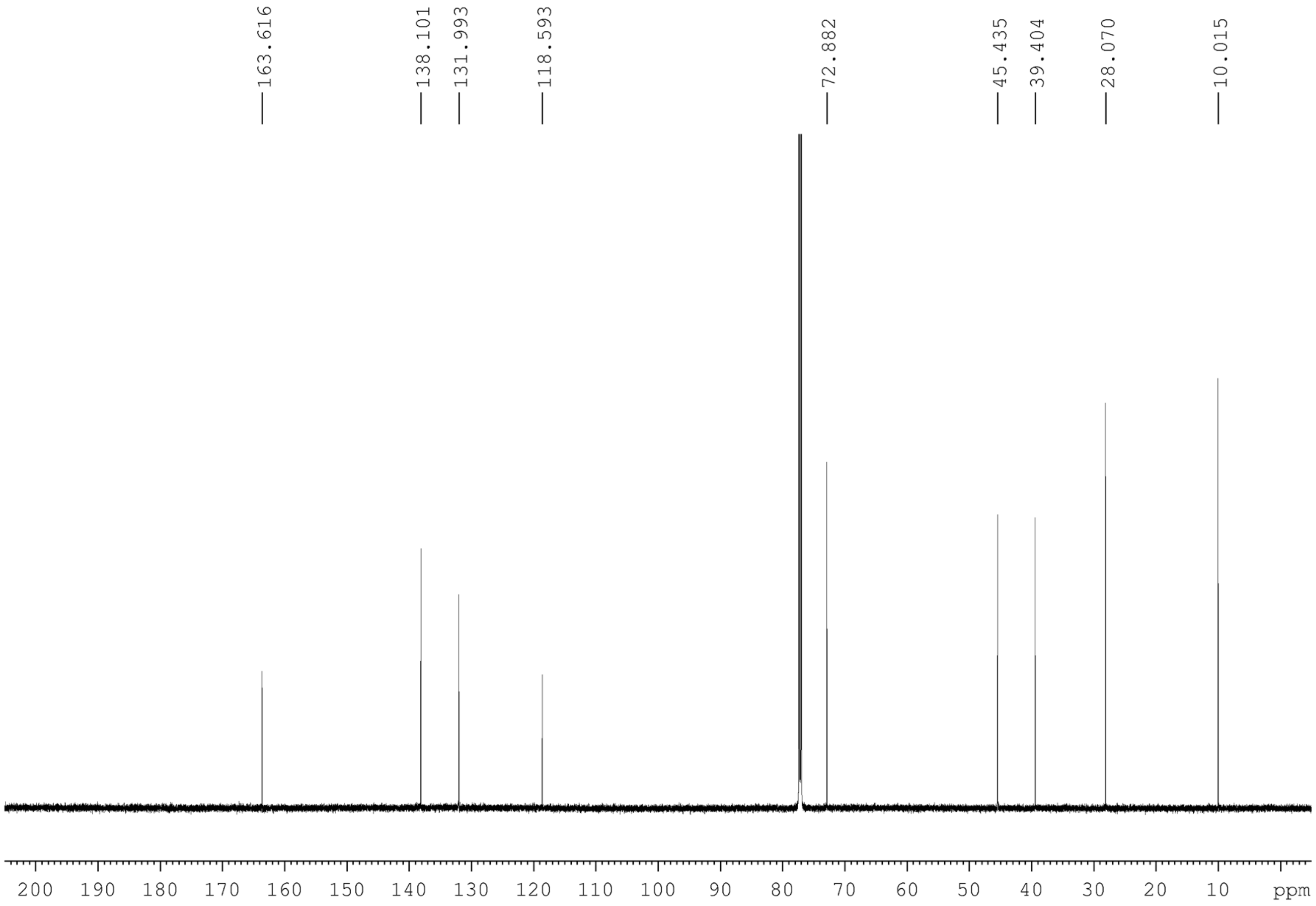

6-chloro-*N*-(2-hydroxybutyl)nicotinamide (1zl-rac)

In $(CD_3)_2CO$

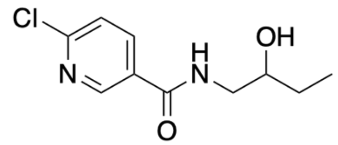

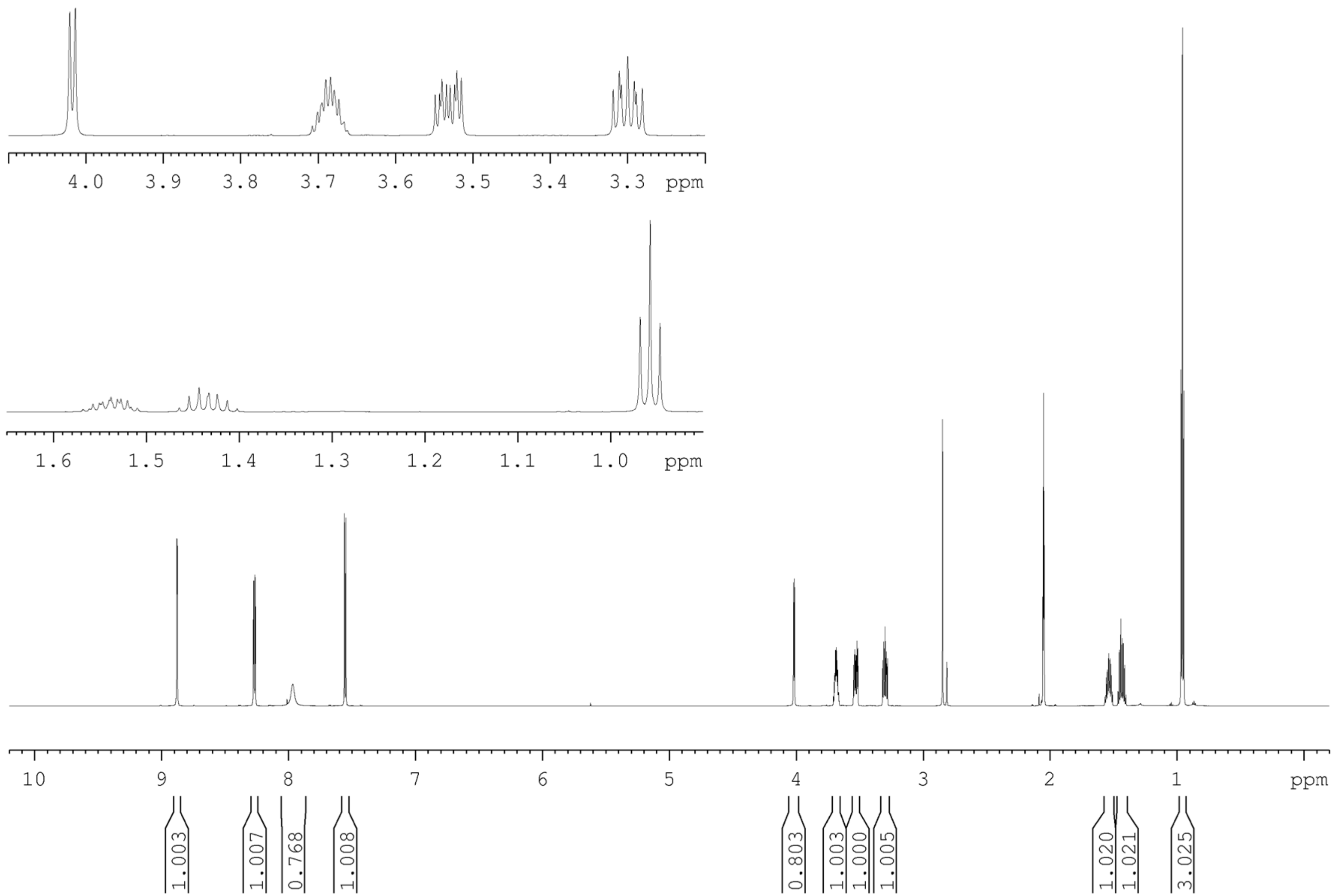

6-chloro-*N*-(2-hydroxybutyl)nicotinamide (1zl-rac)

In $(CD_3)_2CO$

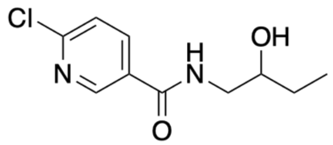

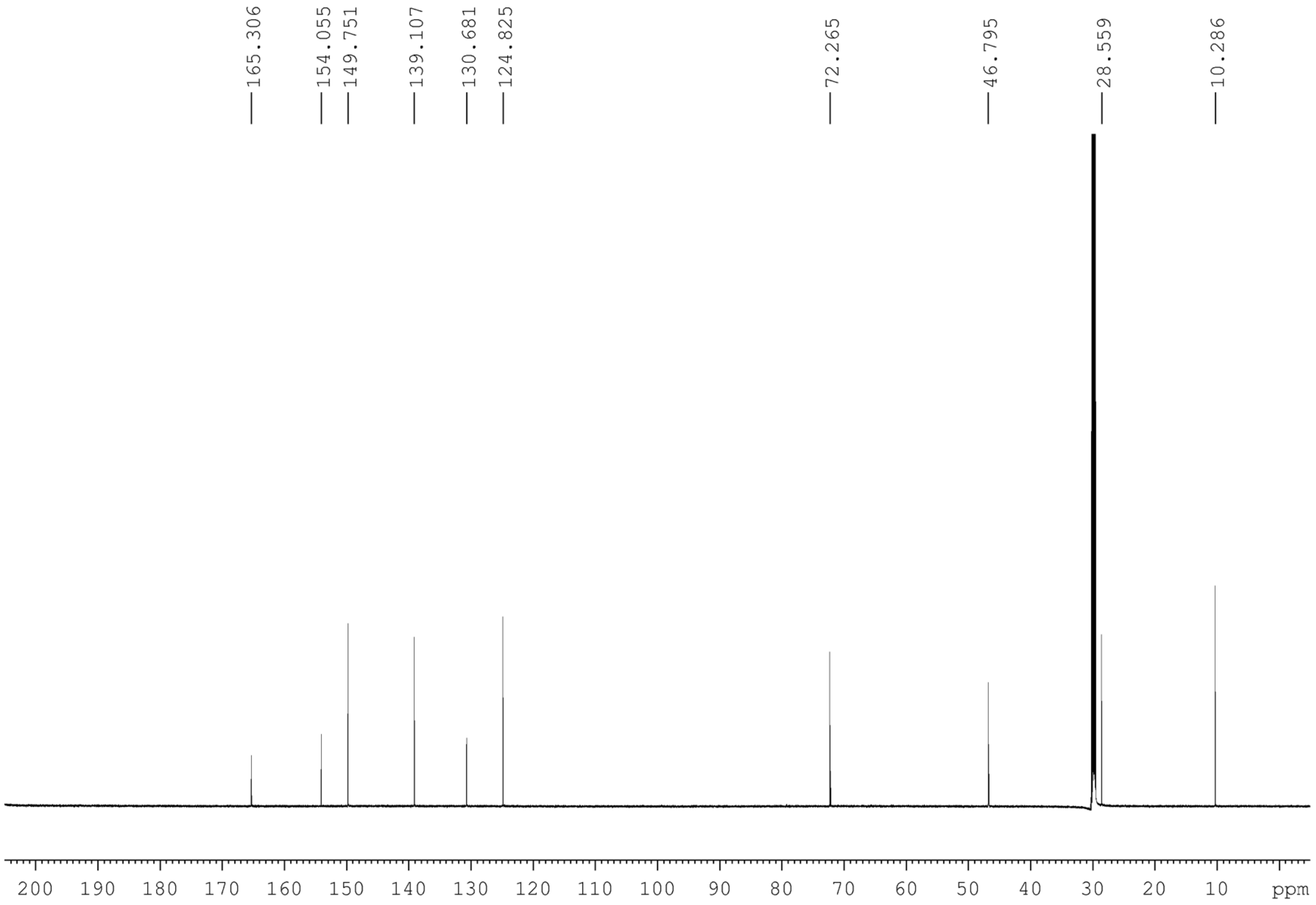

(*S*)-6-chloro-*N*-(2-hydroxybutyl)nicotinamide (1zl)

In $(CD_3)_2CO$

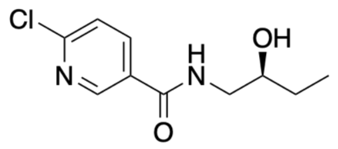

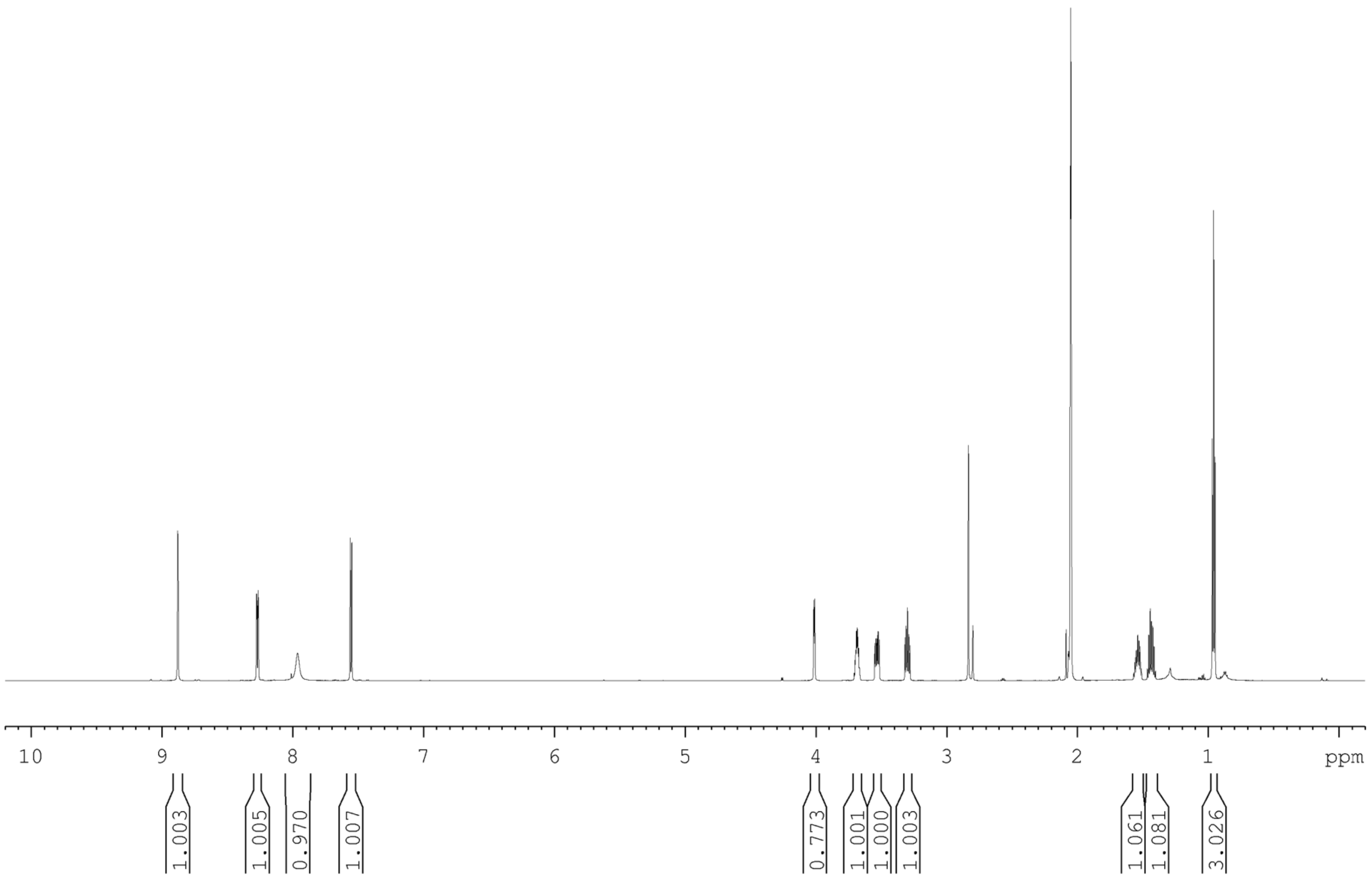

(*S*)-6-chloro-*N*-(2-hydroxybutyl)nicotinamide (1zl)

In $(CD_3)_2CO$

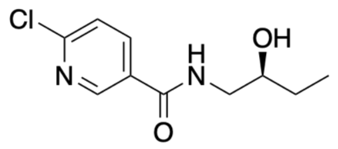

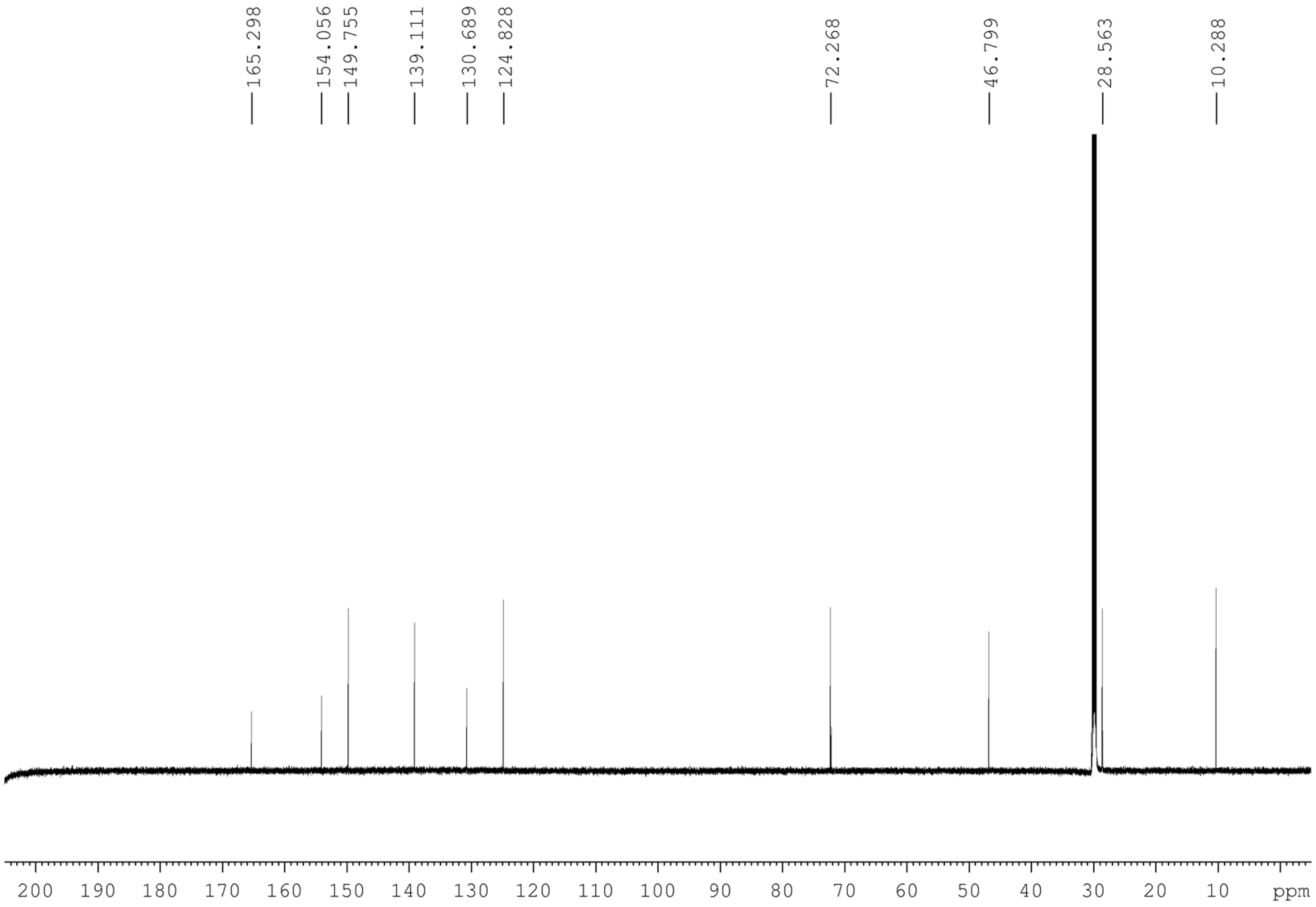

(*R*)-6-chloro-*N*-(2-hydroxybutyl)nicotinamide ((*R*)-1zl)

In $(CD_3)_2CO$

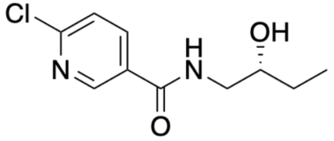

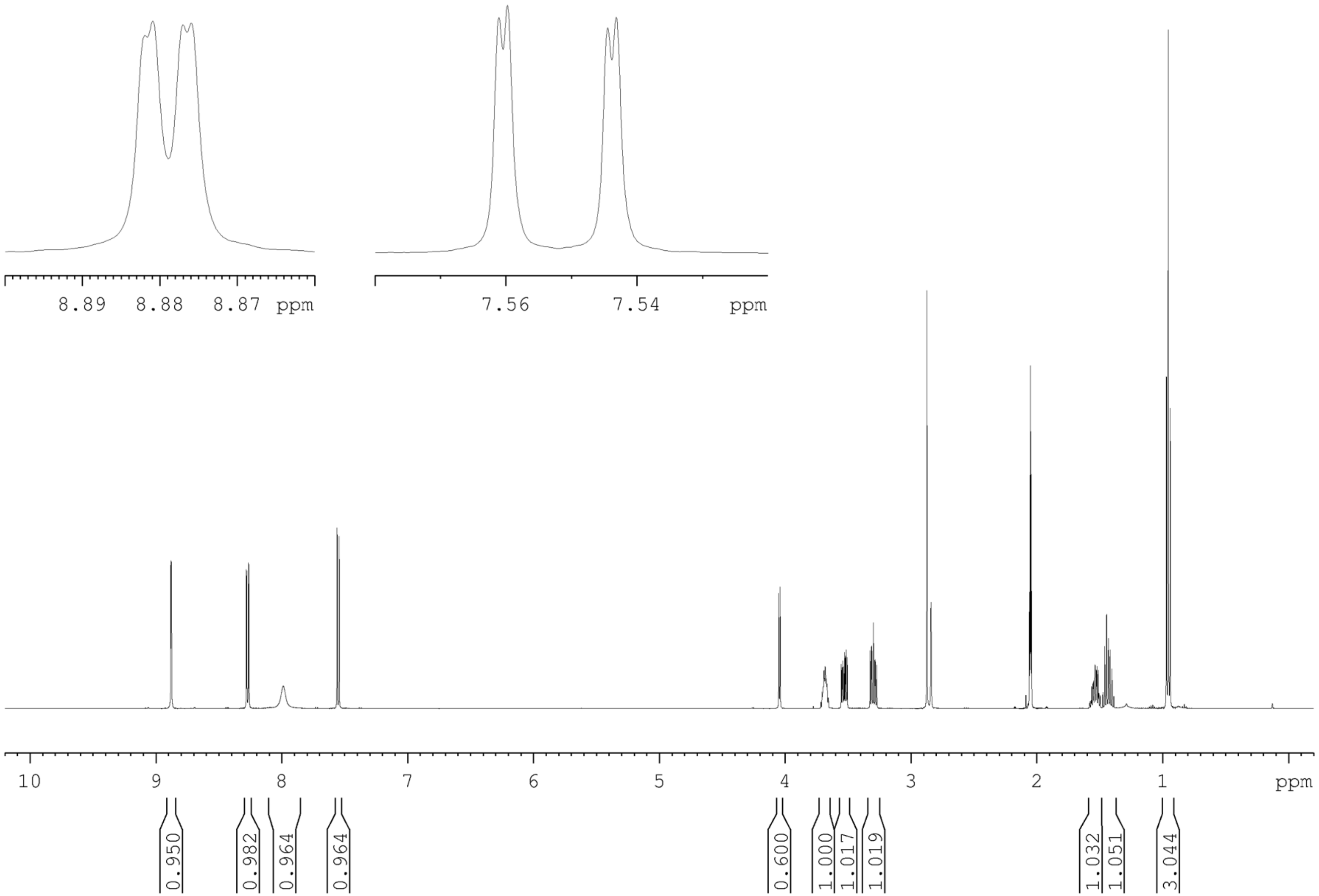

(*R*)-6-chloro-*N*-(2-hydroxybutyl)nicotinamide ((*R*)-1zl)

In $(CD_3)_2CO$

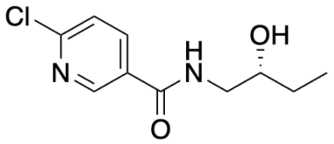

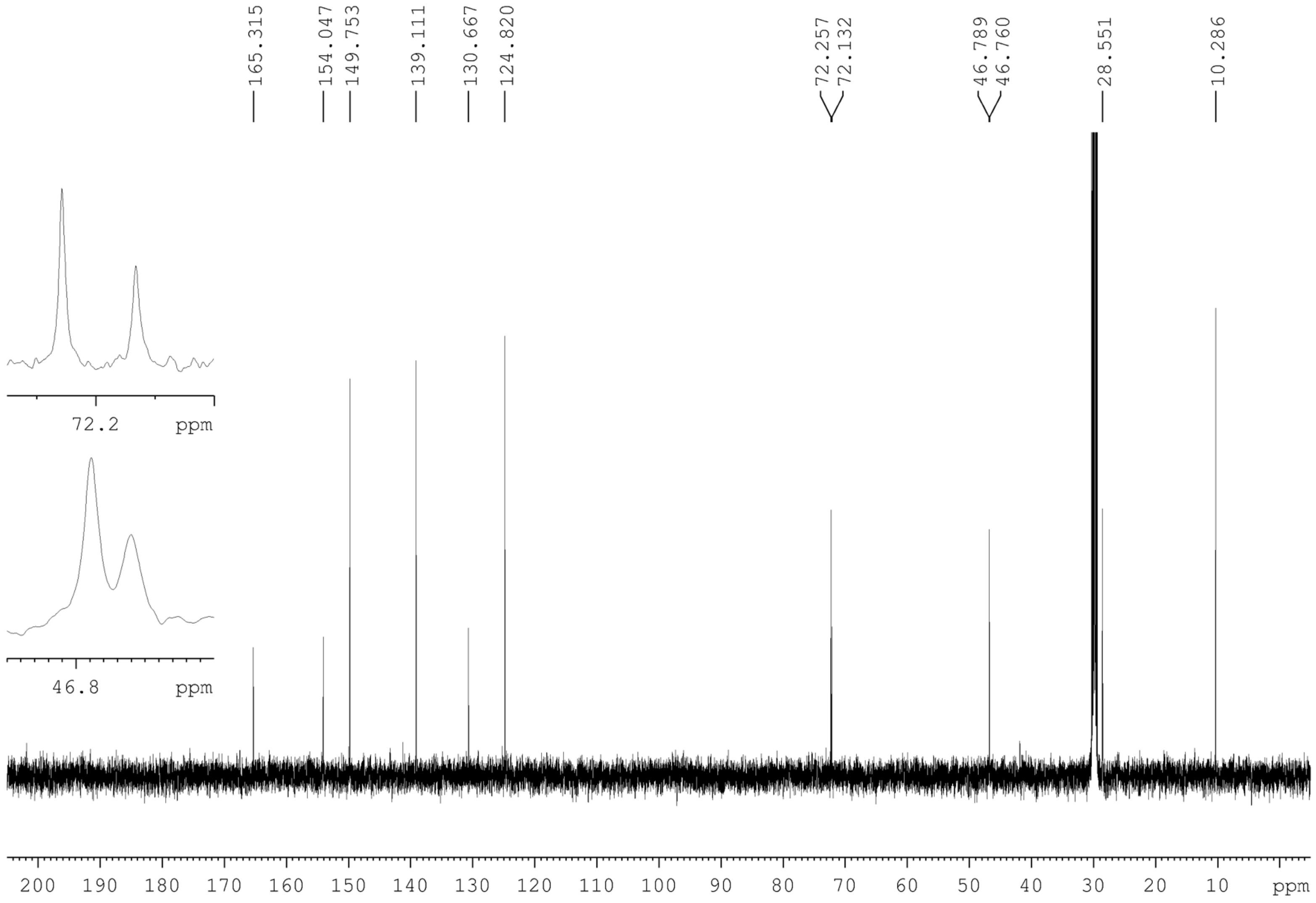

N-(2-hydroxybutyl)-2-(thiazol-4-yl)acetamide (1zm-rac)

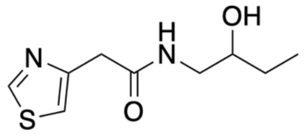

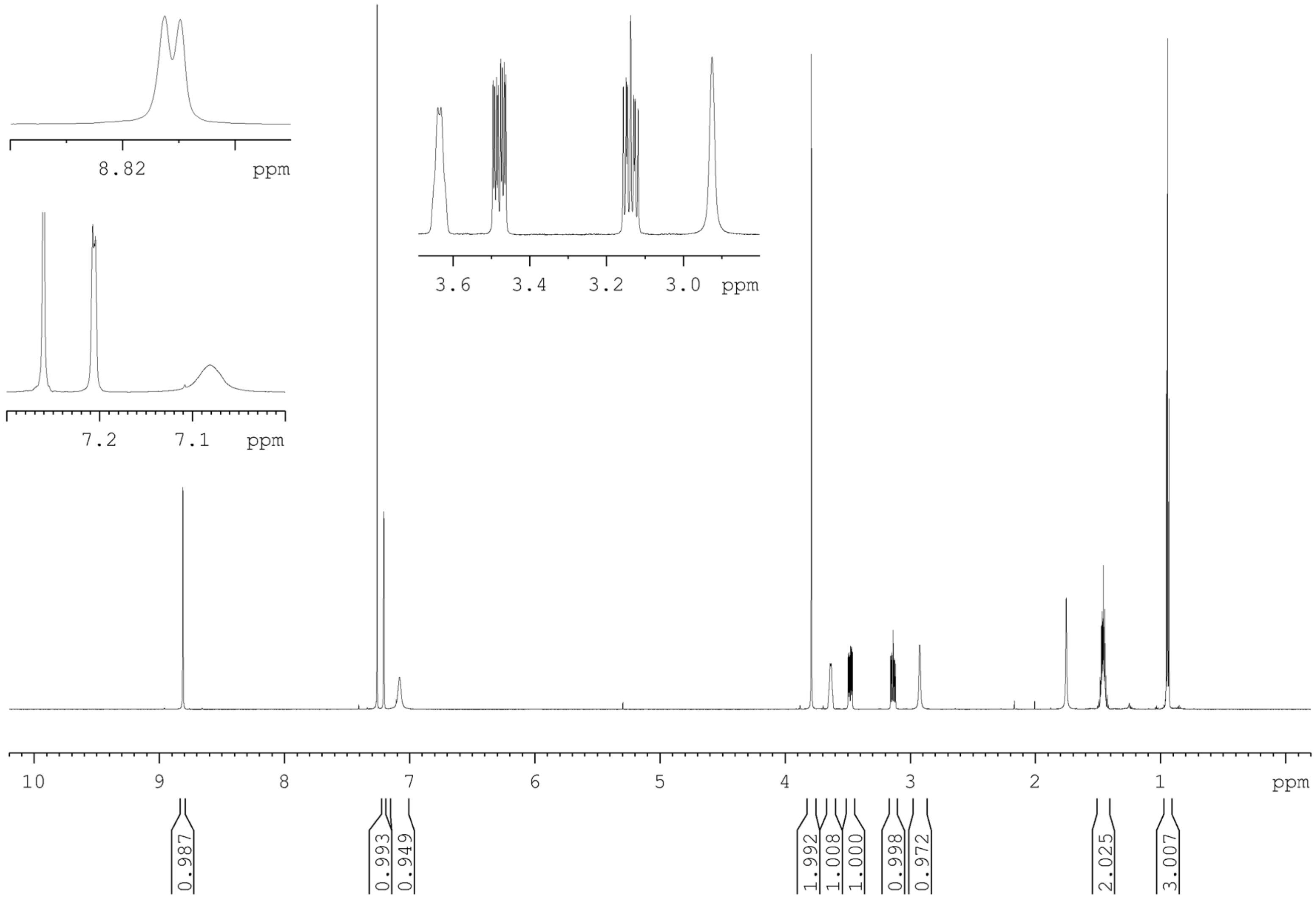

N-(2-hydroxybutyl)-2-(thiazol-4-yl)acetamide (1zm-rac)

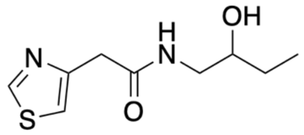

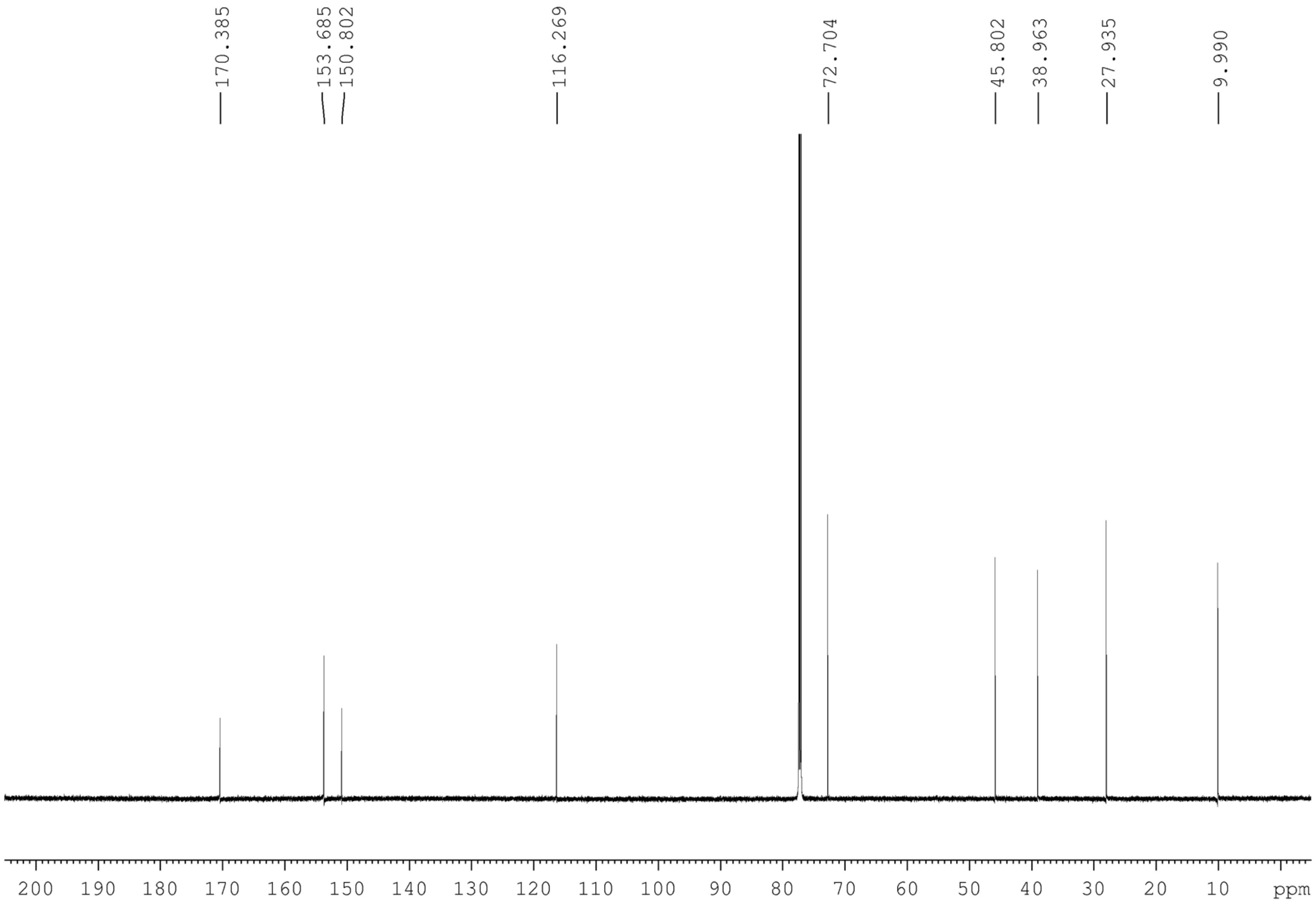

(*S*)-*N*-(2-hydroxybutyl)-2-(thiazol-4-yl)acetamide (1zm)

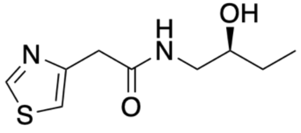

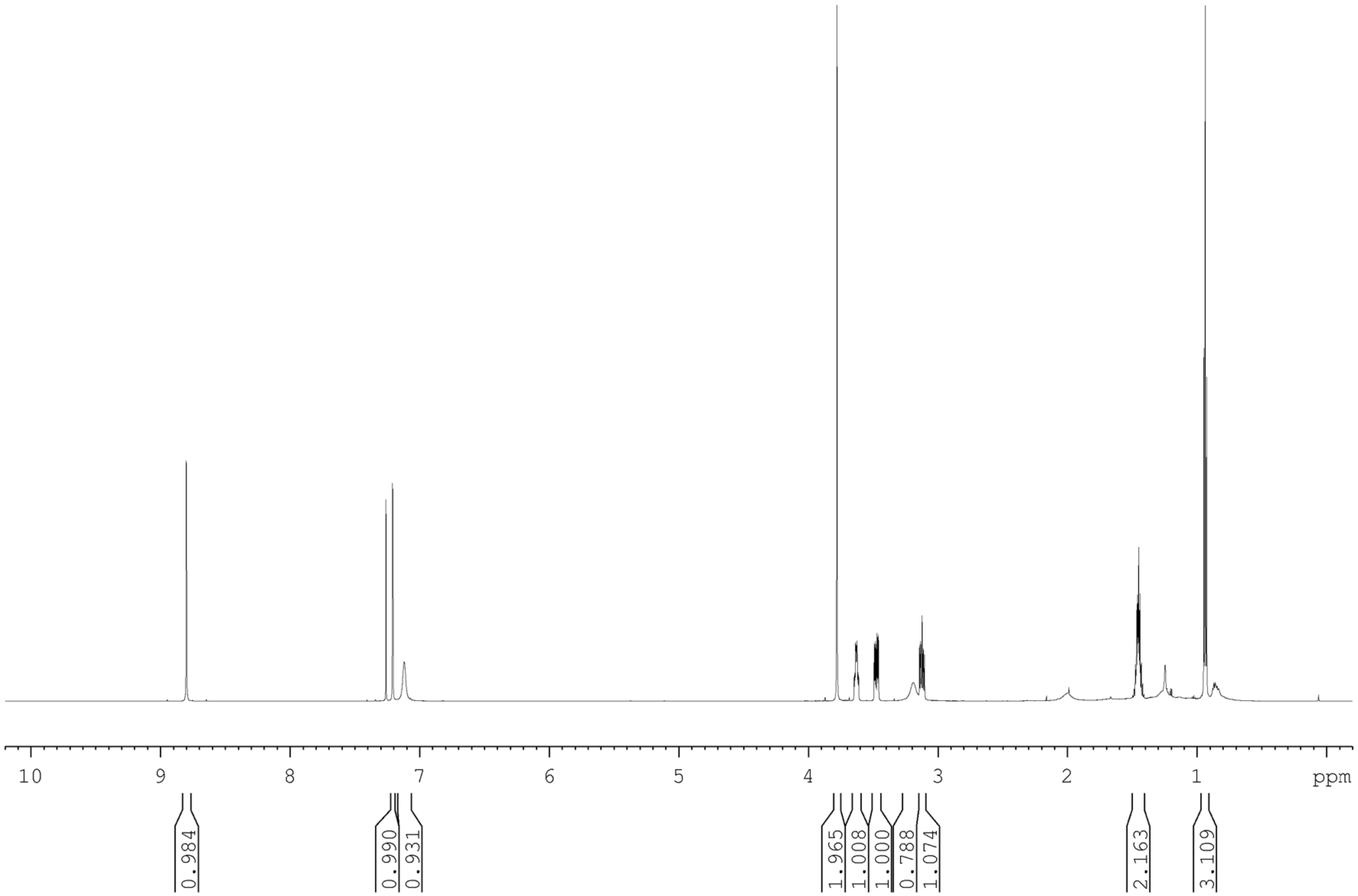

(*S*)-*N*-(2-hydroxybutyl)-2-(thiazol-4-yl)acetamide (1zm)

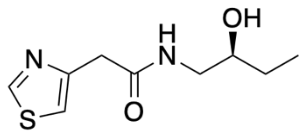

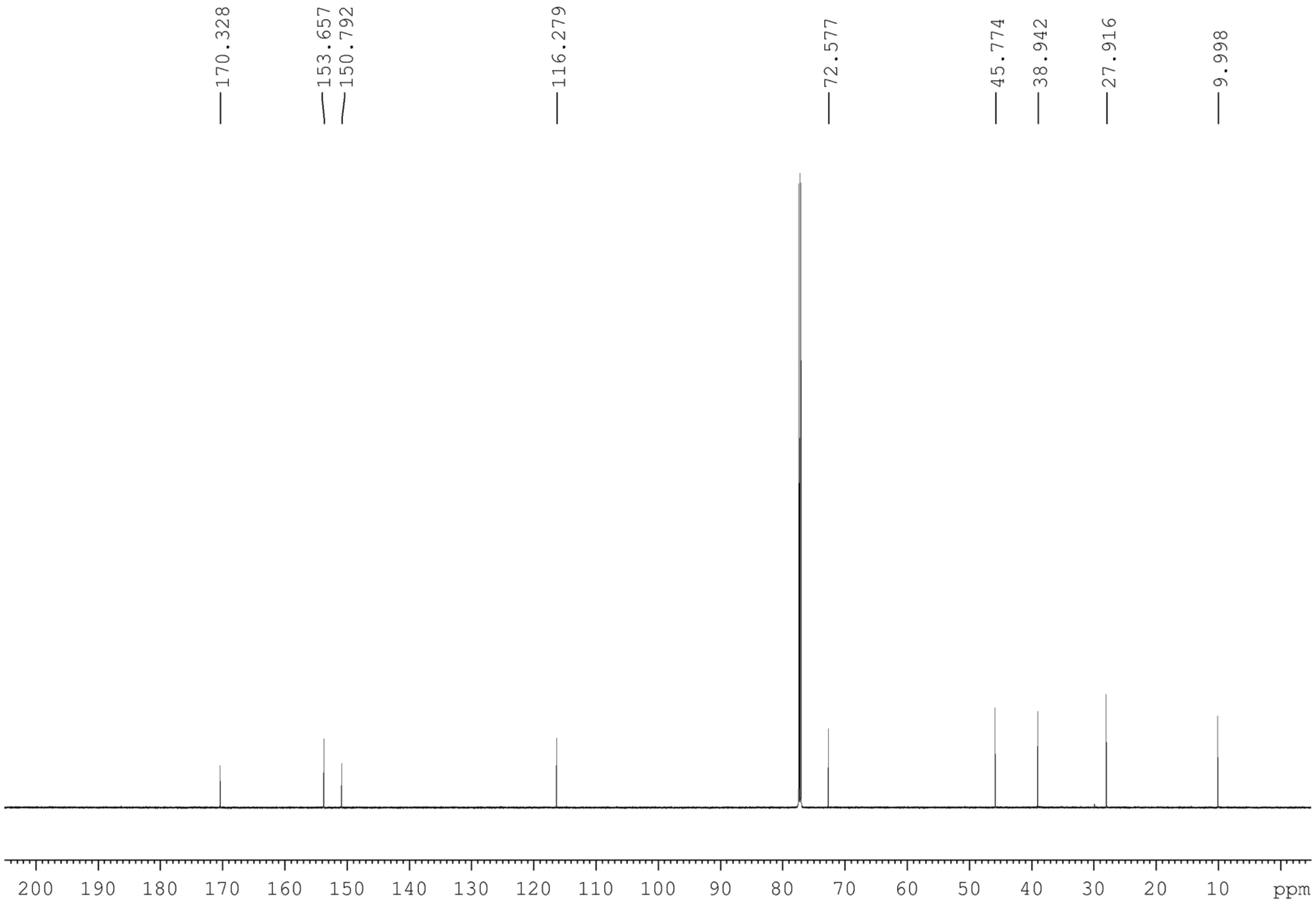

tert-butyl 4-((2-hydroxybutyl)carbamoyl)piperidine-1-carboxylate (1zn-rac)

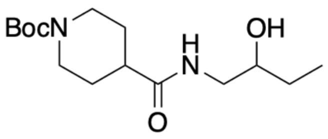

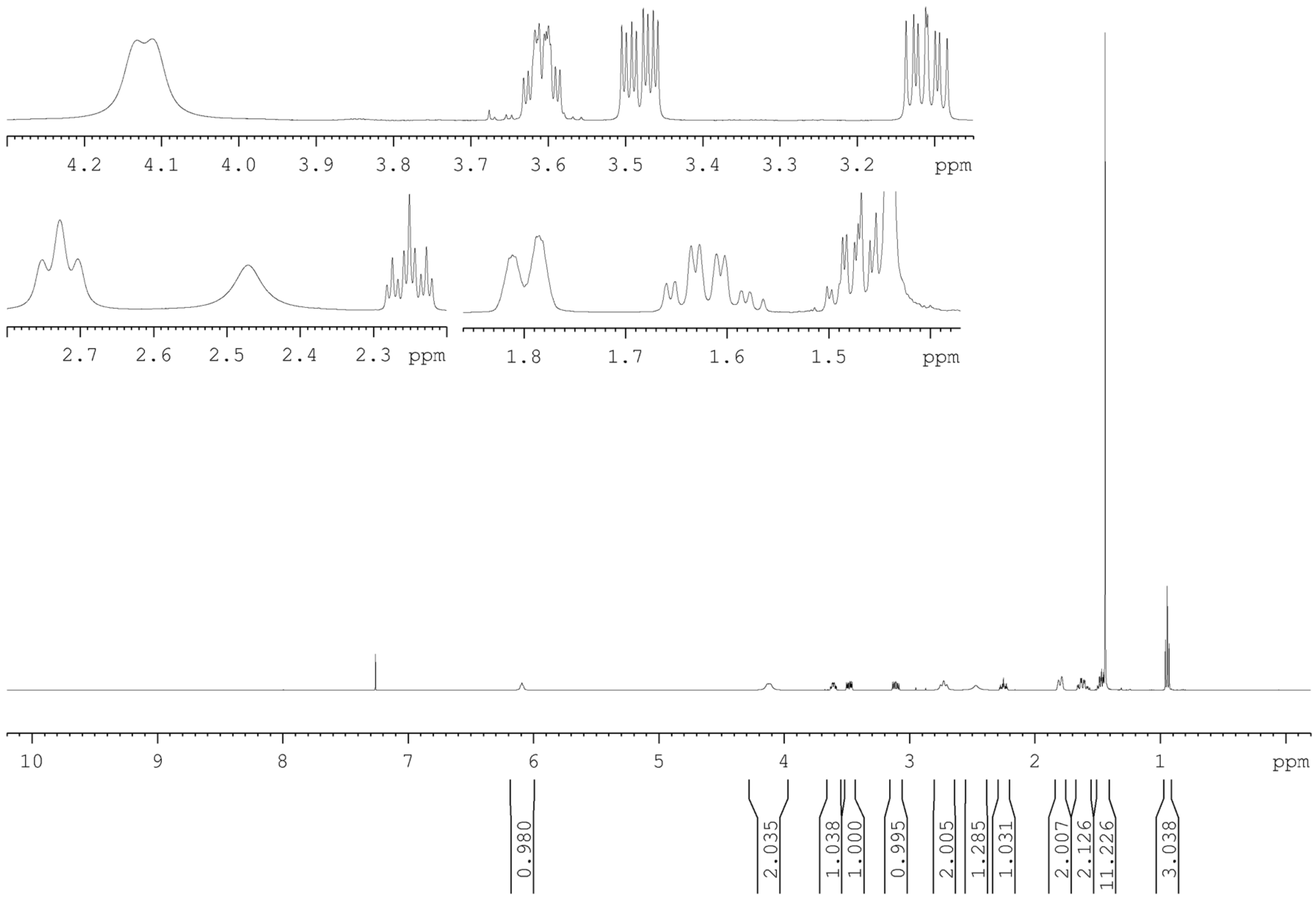

tert-butyl 4-((2-hydroxybutyl)carbamoyl)piperidine-1-carboxylate (1zn-rac)

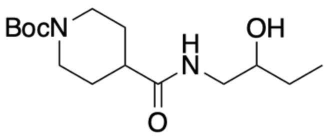

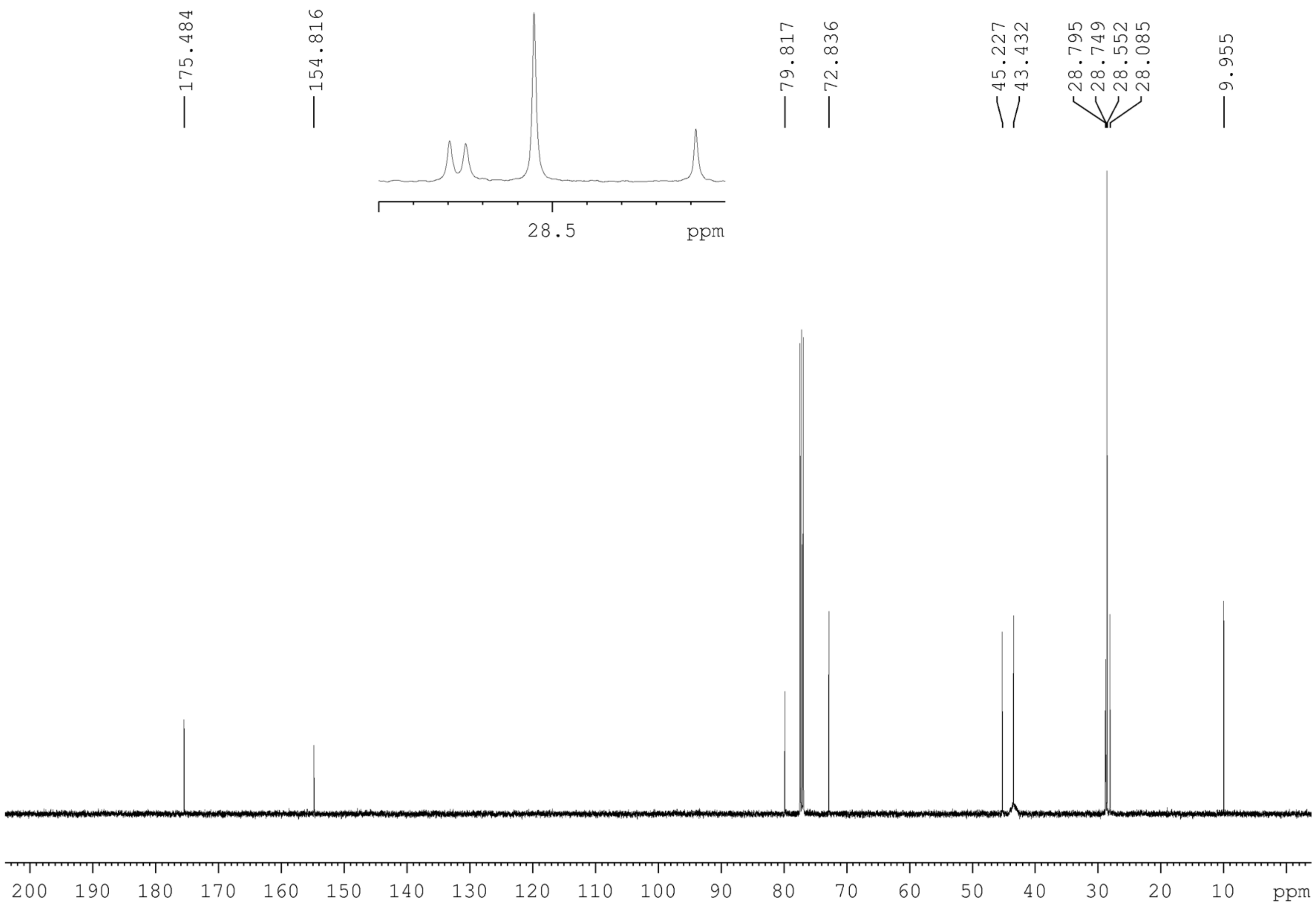

tert-butyl (S)-4-((2-hydroxybutyl)carbamoyl)piperidine-1-carboxylate (1zn)

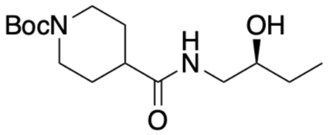

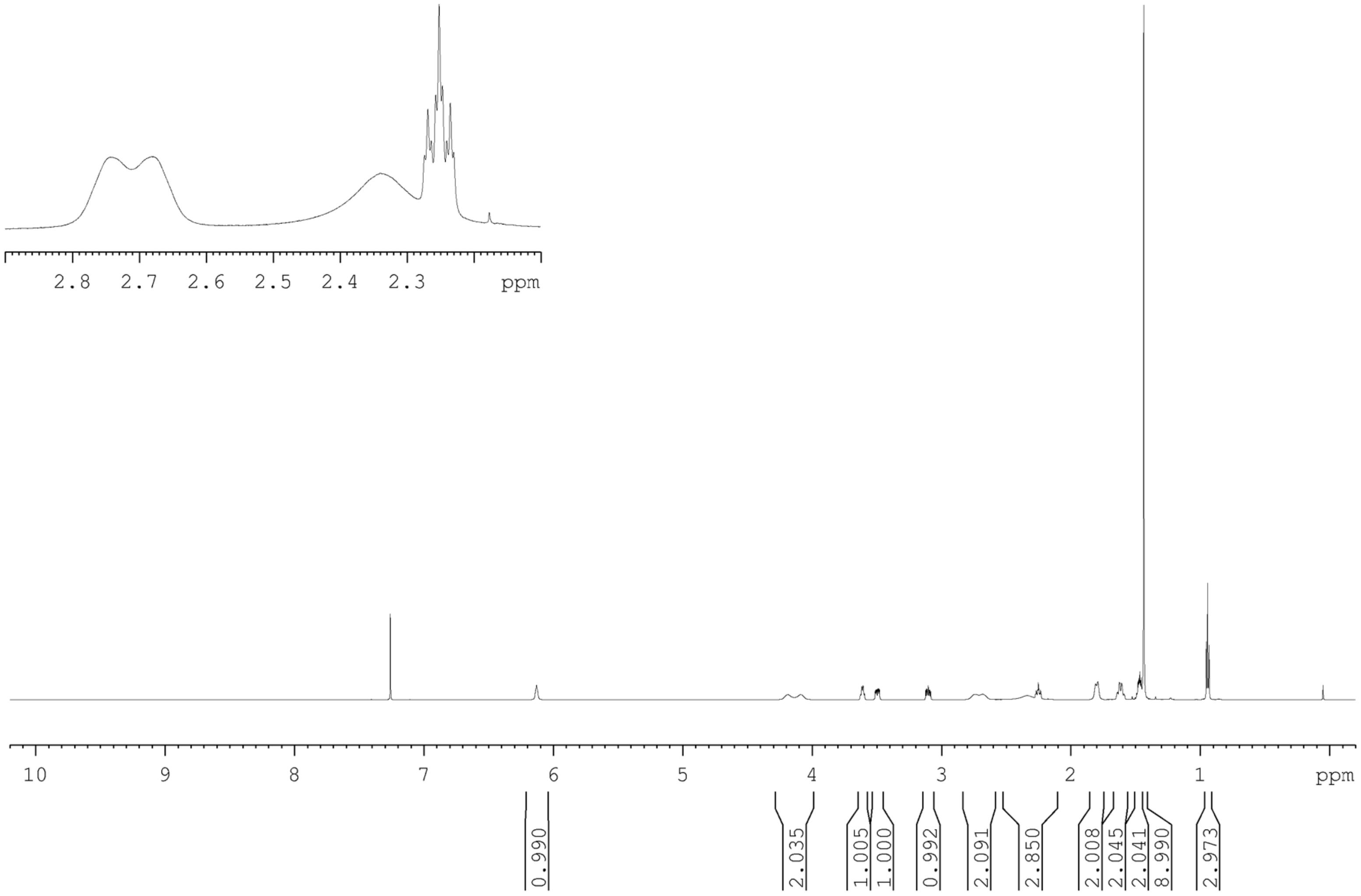

***tert*-butyl (*S*)-4-((2-hydroxybutyl)carbamoyl)piperidine-1-carboxylate (1zn)**

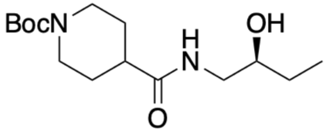

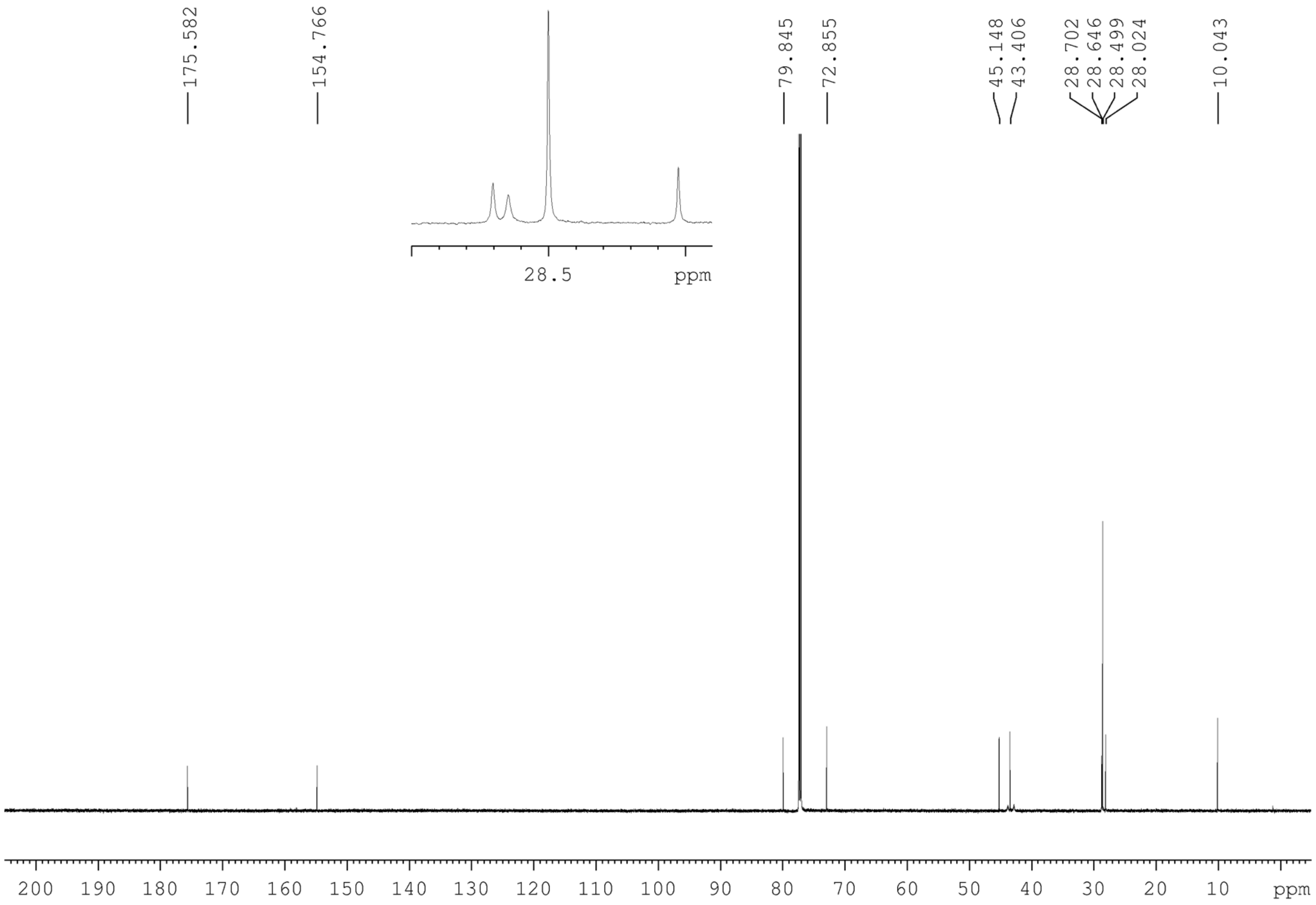

tert-butyl (_S_)-4-((2-(benzoyloxy)butyl)carbamoyl)piperidine-1-carboxylate (1zn-Bz)

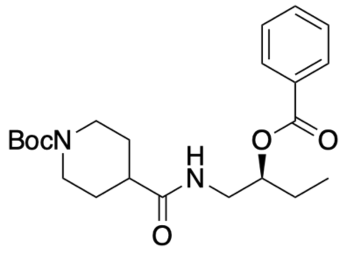

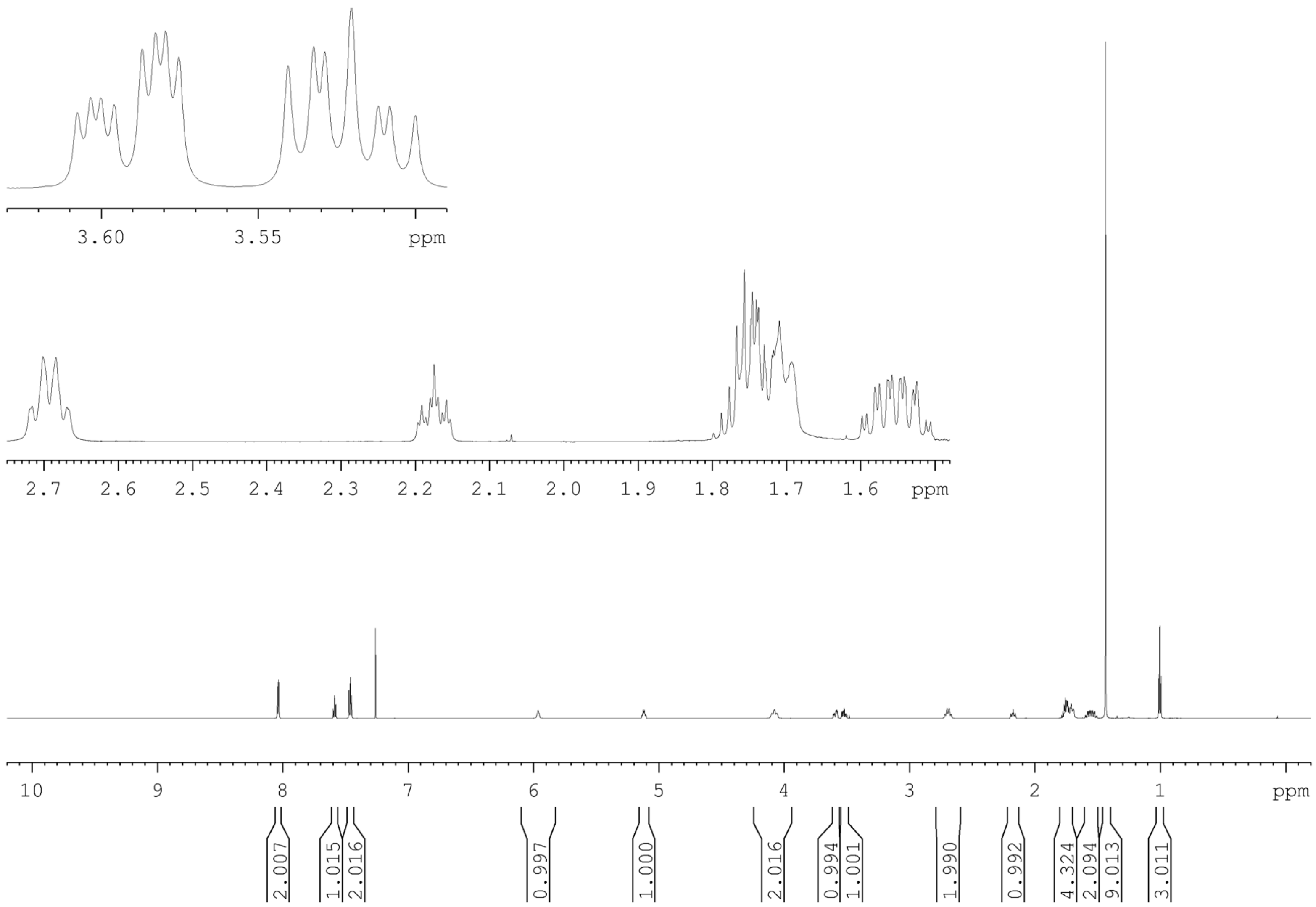

tert-butyl (_S_)-4-((2-(benzoyloxy)butyl)carbamoyl)piperidine-1-carboxylate (1zn-Bz)

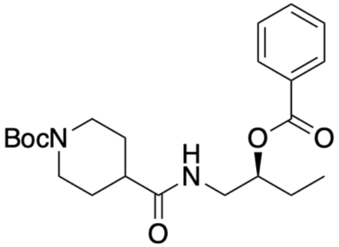

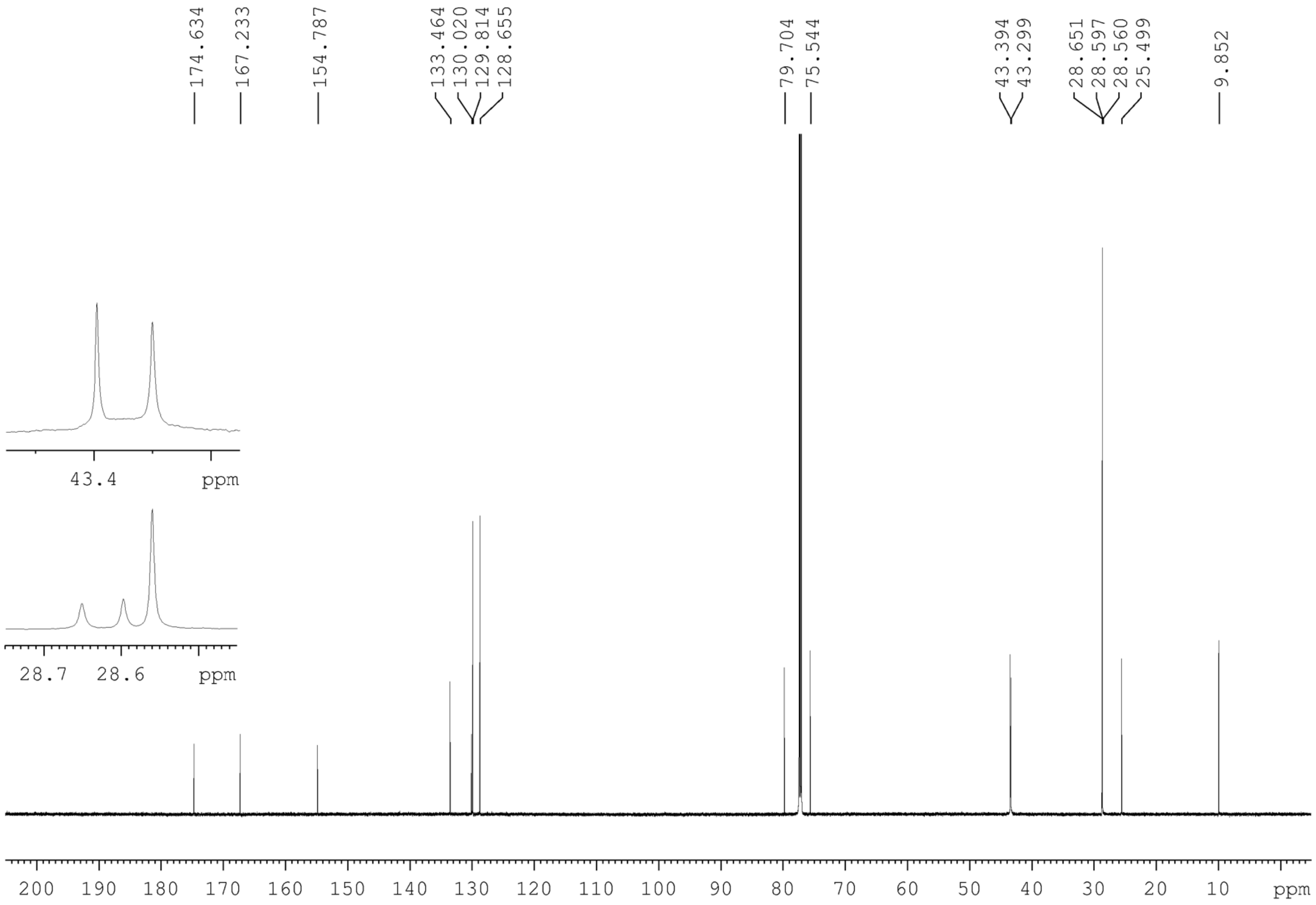

N-(2-hydroxybutyl)morpholine-4-carboxamide (1zo-rac)

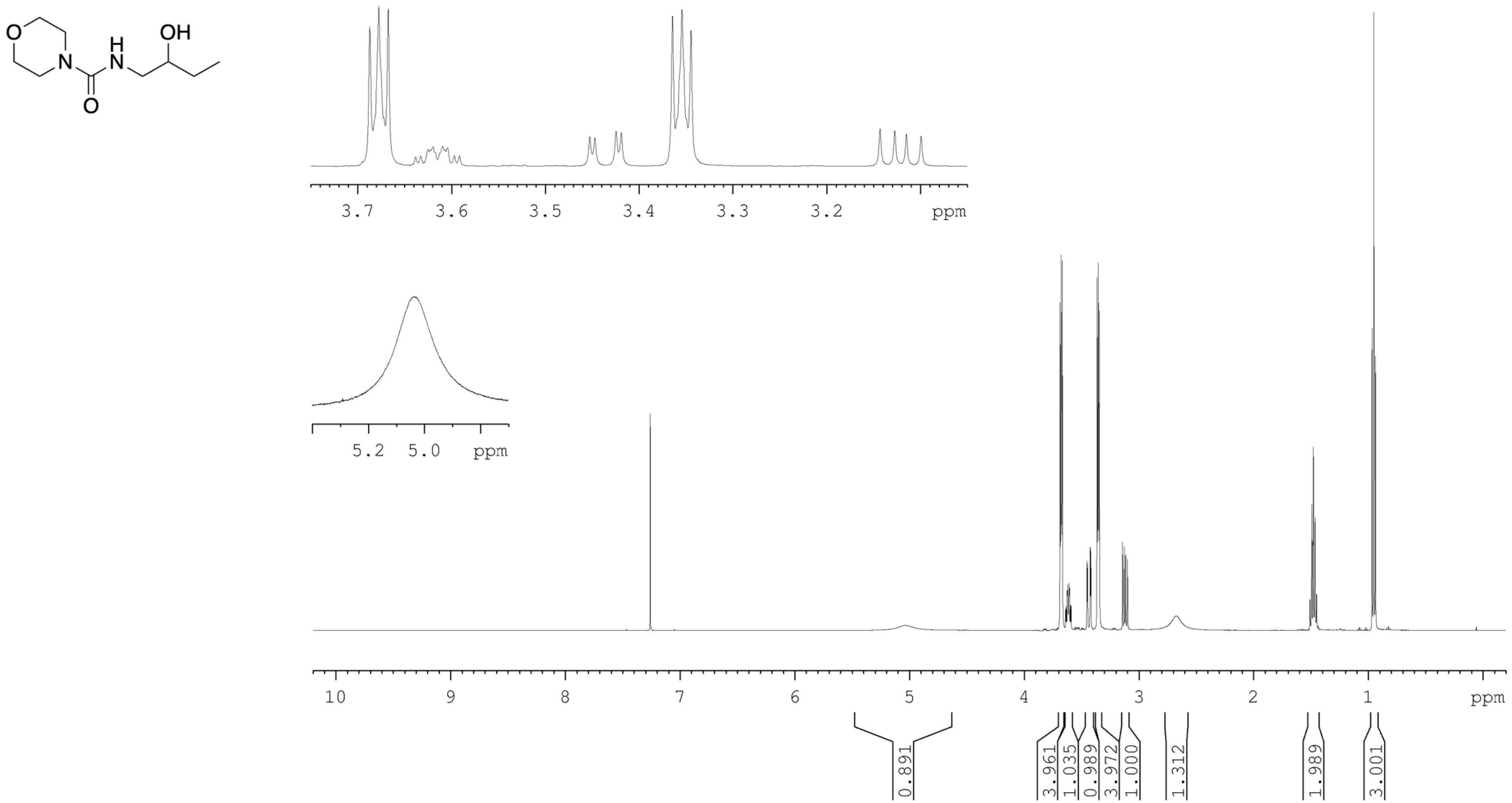

N-(2-hydroxybutyl)morpholine-4-carboxamide (1zo-rac)

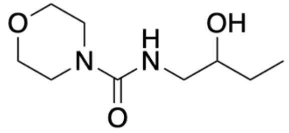

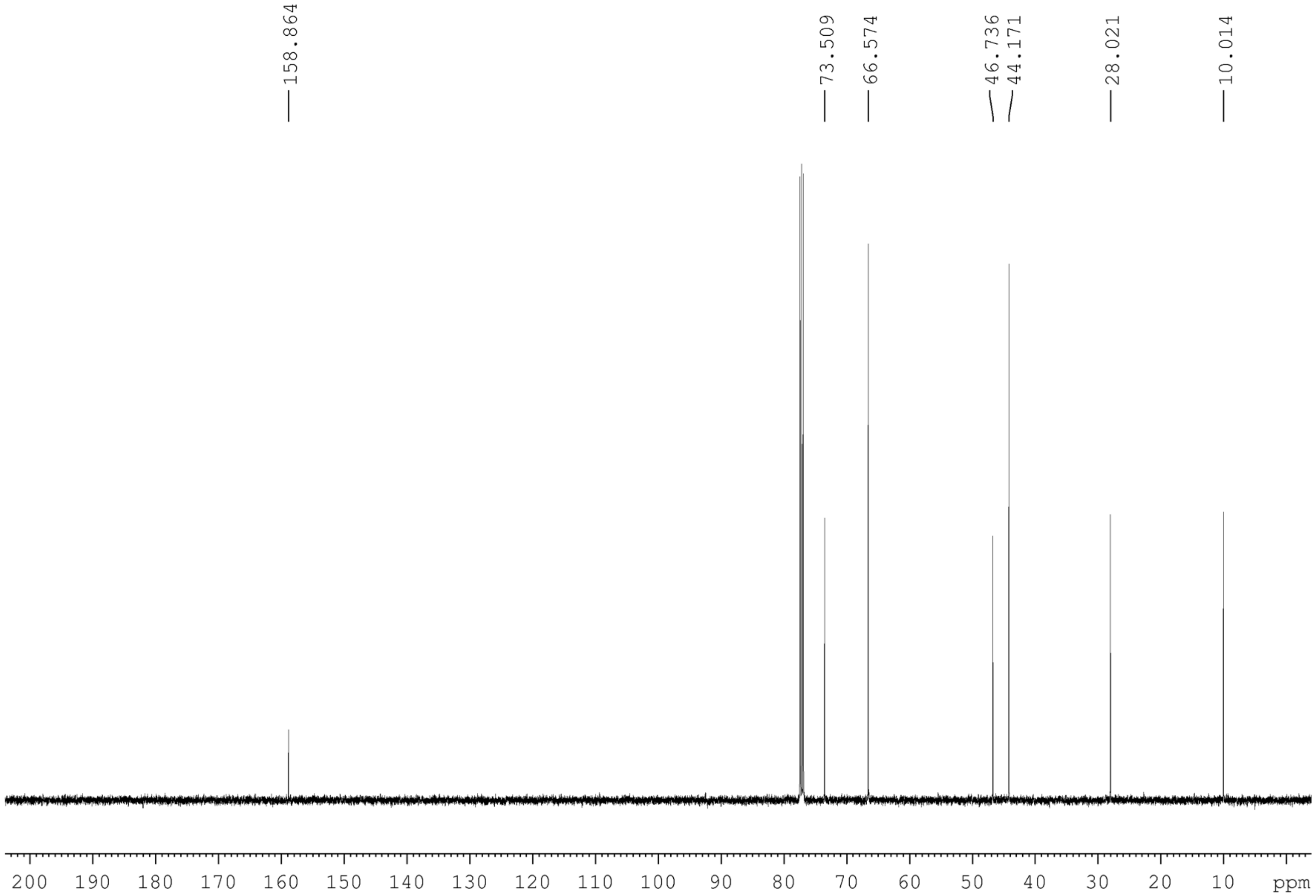

(*S*)-*N*-(2-hydroxybutyl)morpholine-4-carboxamide (1zo)

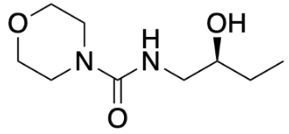

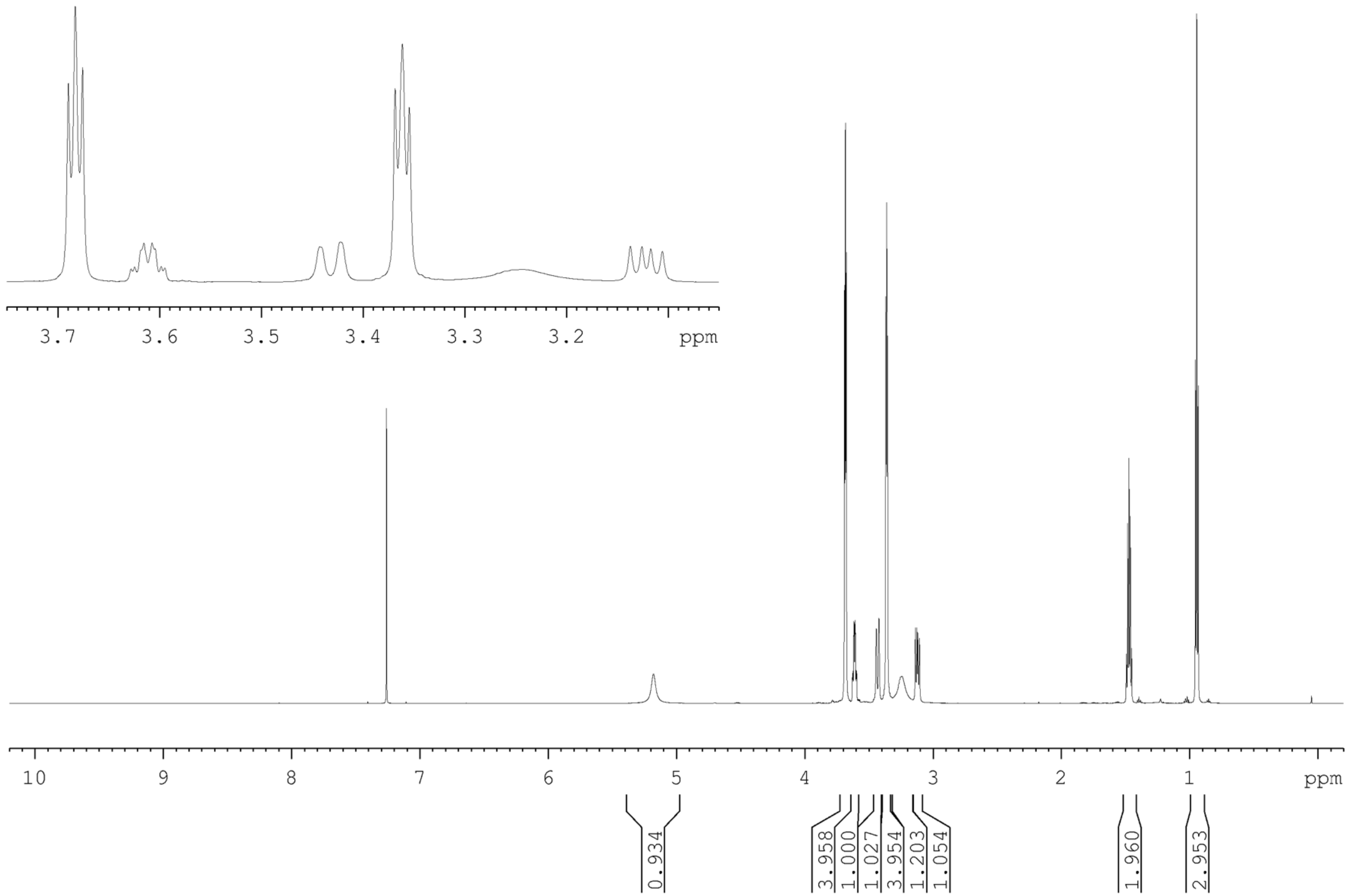

(*S*)-*N*-(2-hydroxybutyl)morpholine-4-carboxamide (1zo)

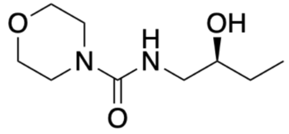

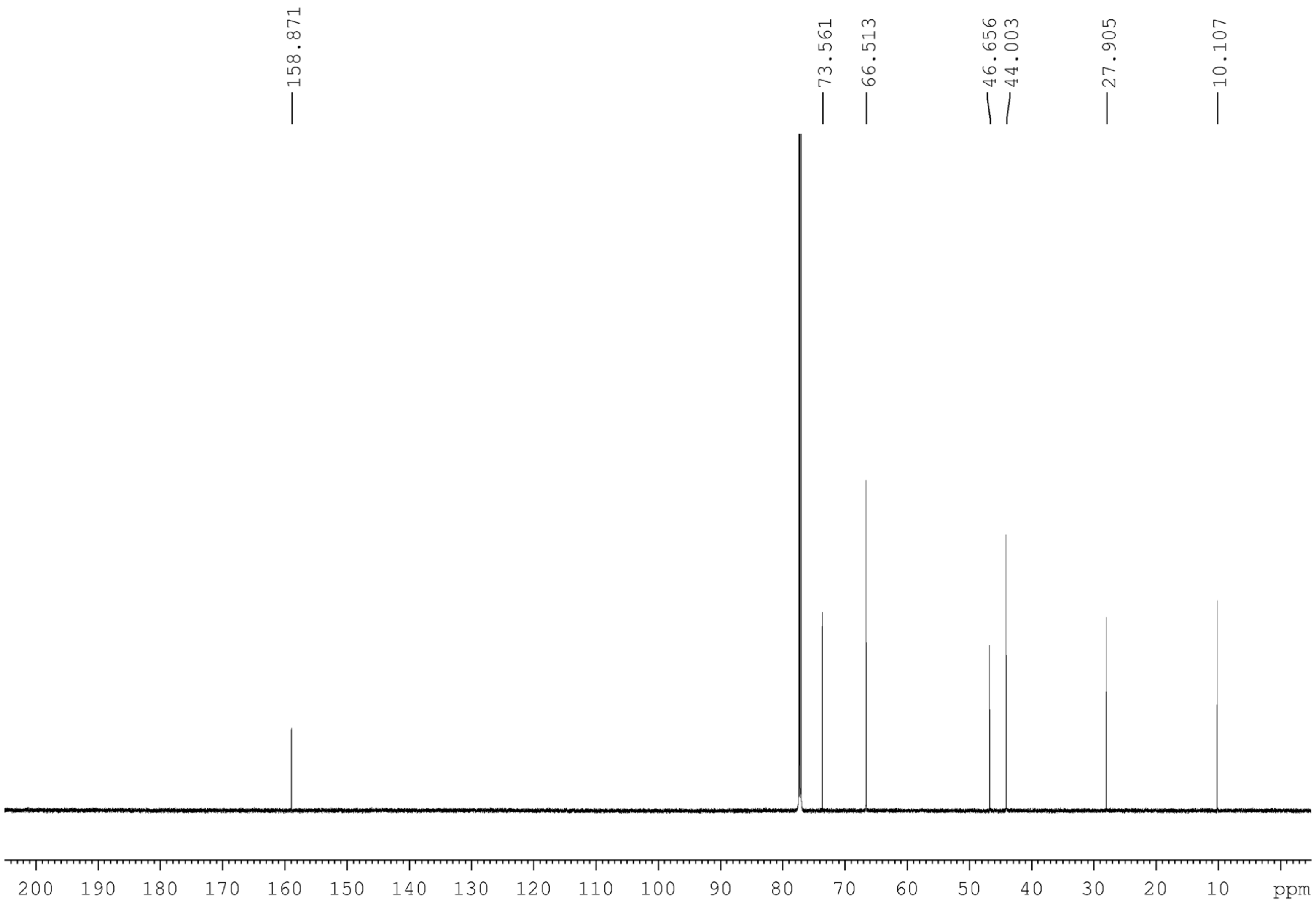

(*S*)-1-(*N*-benzoylmorpholine-4-carboxamido)butan-2-yl benzoate (1zo-Bz)

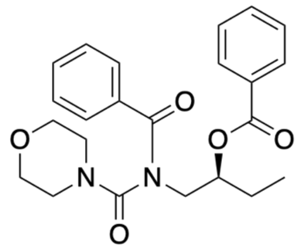

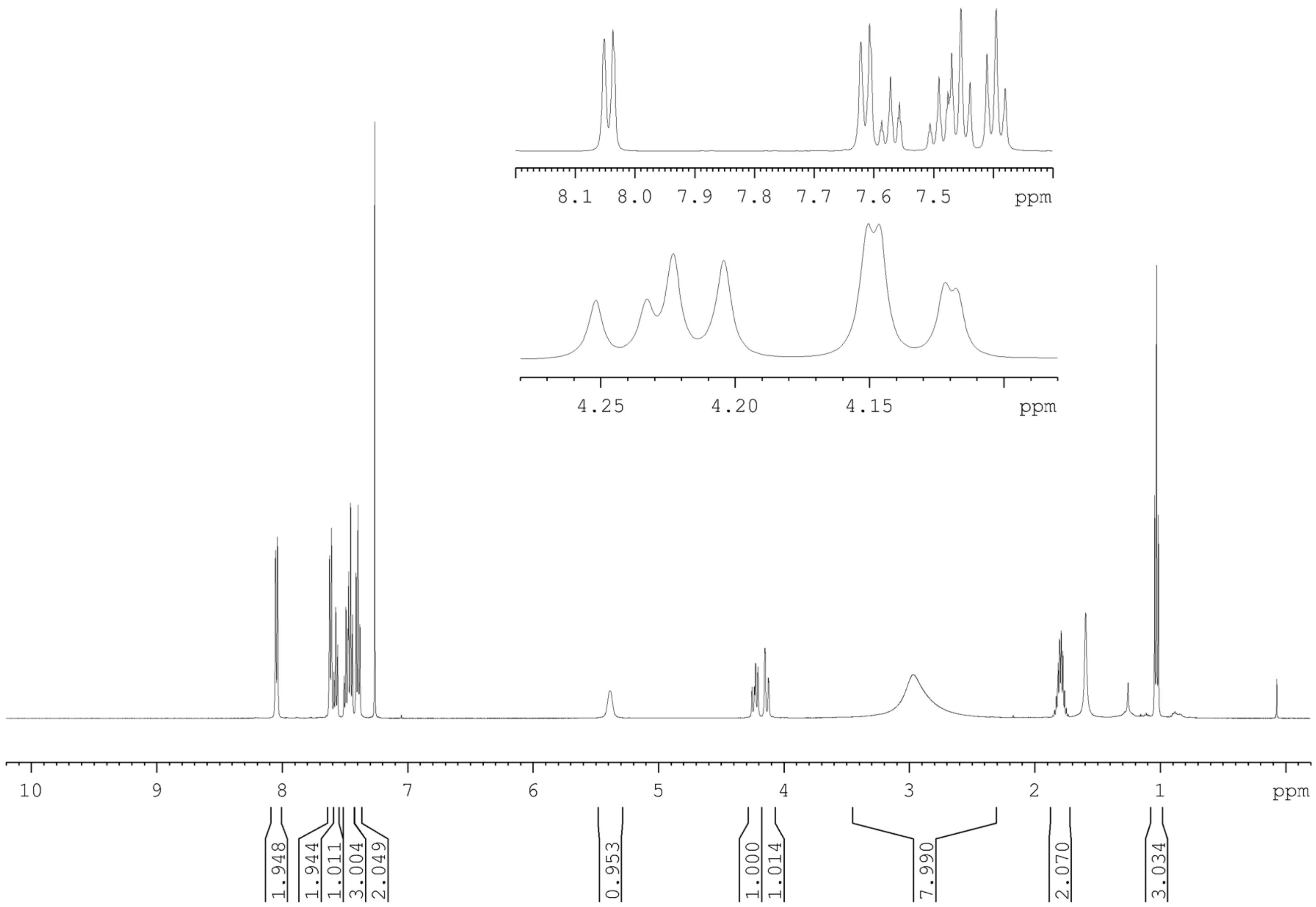

(*S*)-1-(*N*-benzoylmorpholine-4-carboxamido)butan-2-yl benzoate (1zo-Bz)

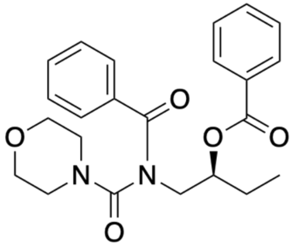

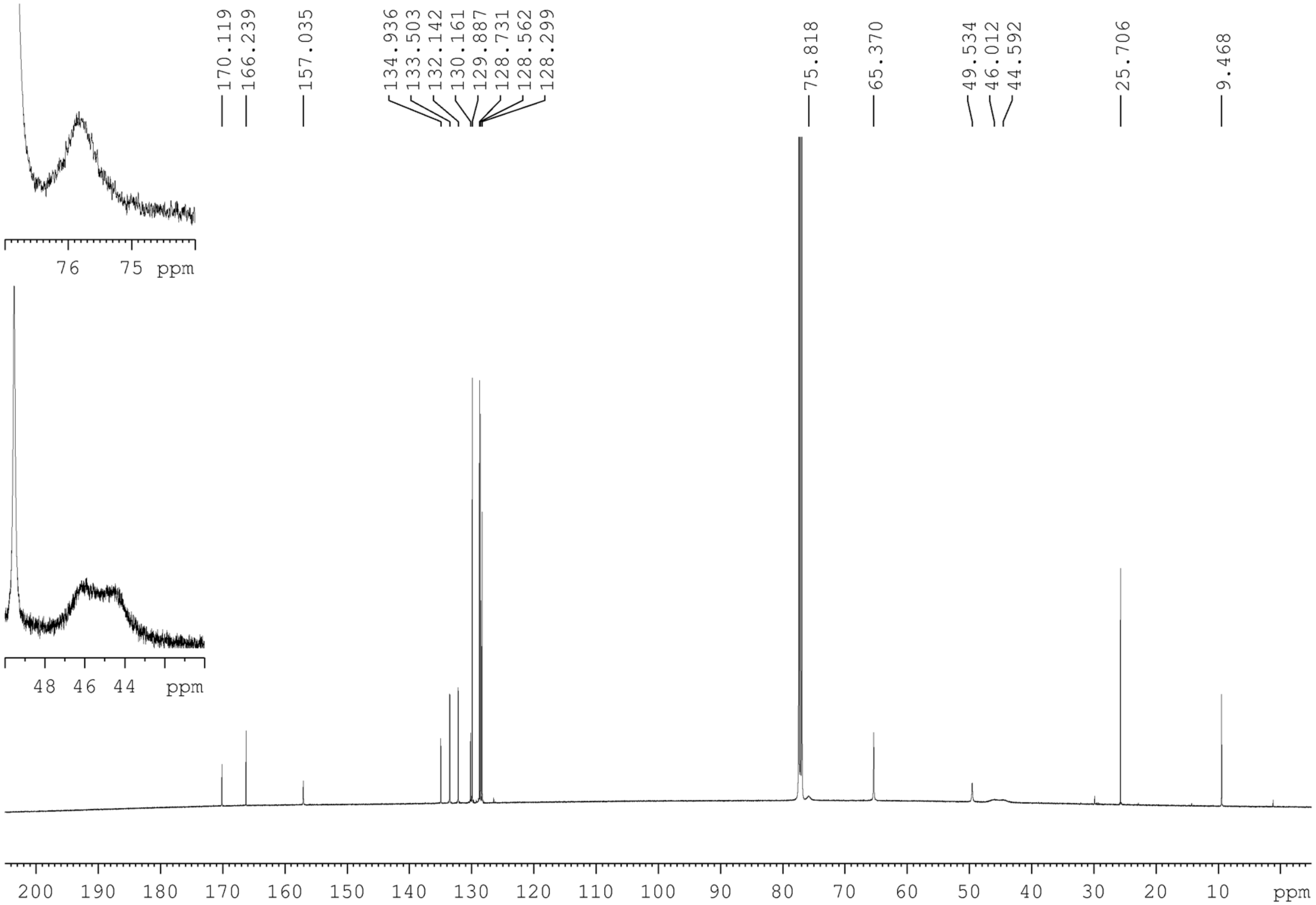

(*R*)-*N*-(2-hydroxybutyl)morpholine-4-carboxamide ((*R*)-1zo)

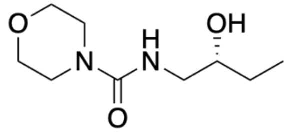

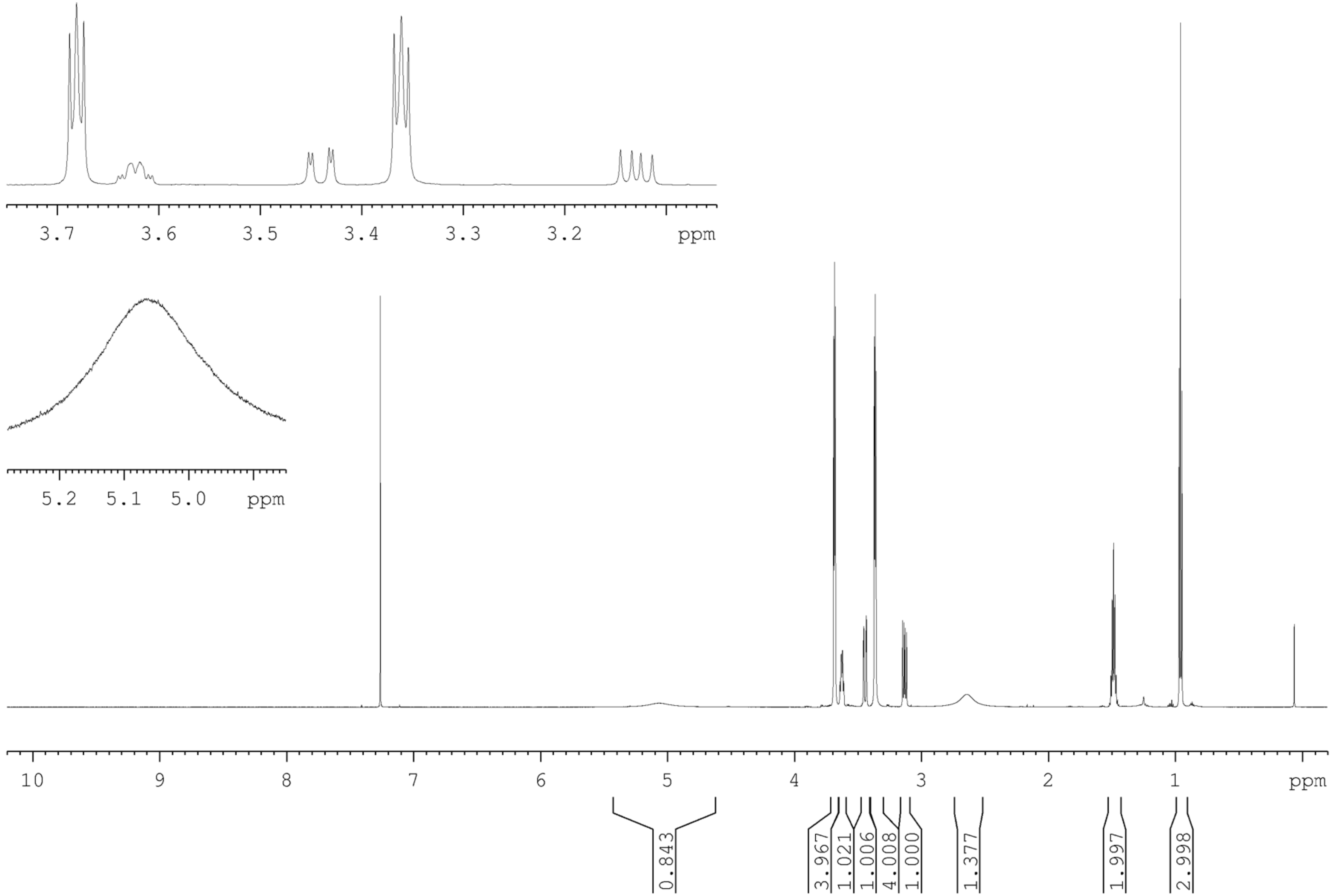

(*R*)-*N*-(2-hydroxybutyl)morpholine-4-carboxamide ((*R*)-1zo)

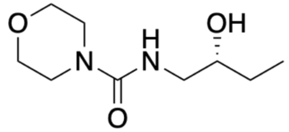

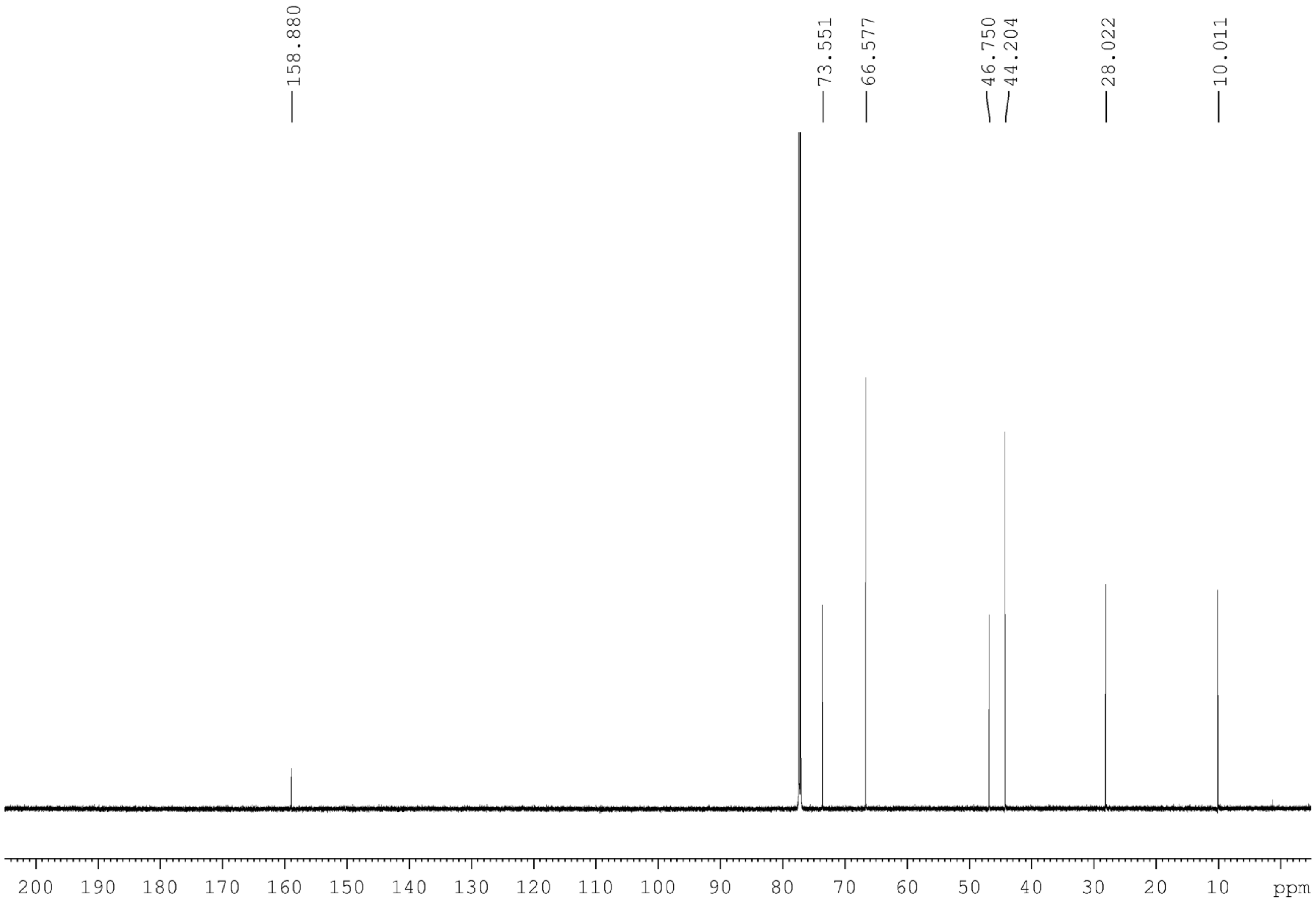

(*R*)-1-(*N*-benzoylmorpholine-4-carboxamido)butan-2-yl benzoate ((*R*)-1zo-Bz)

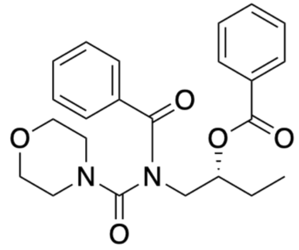

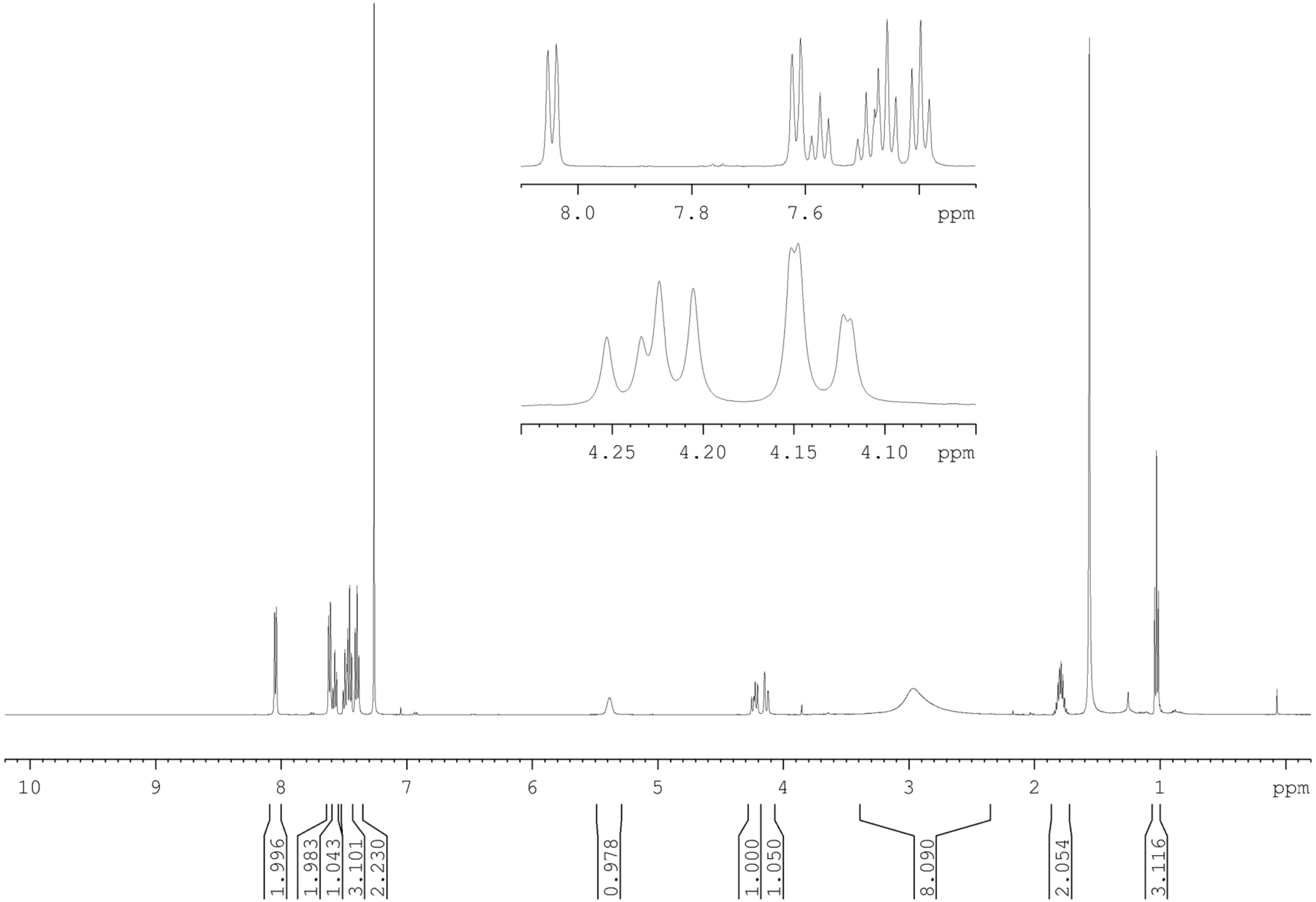

(*R*)-1-(*N*-benzoylmorpholine-4-carboxamido)butan-2-yl benzoate ((*R*)-1zo-Bz)

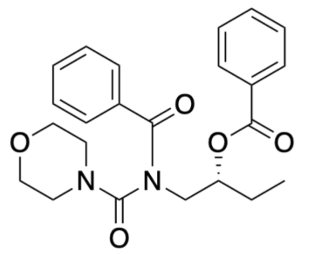

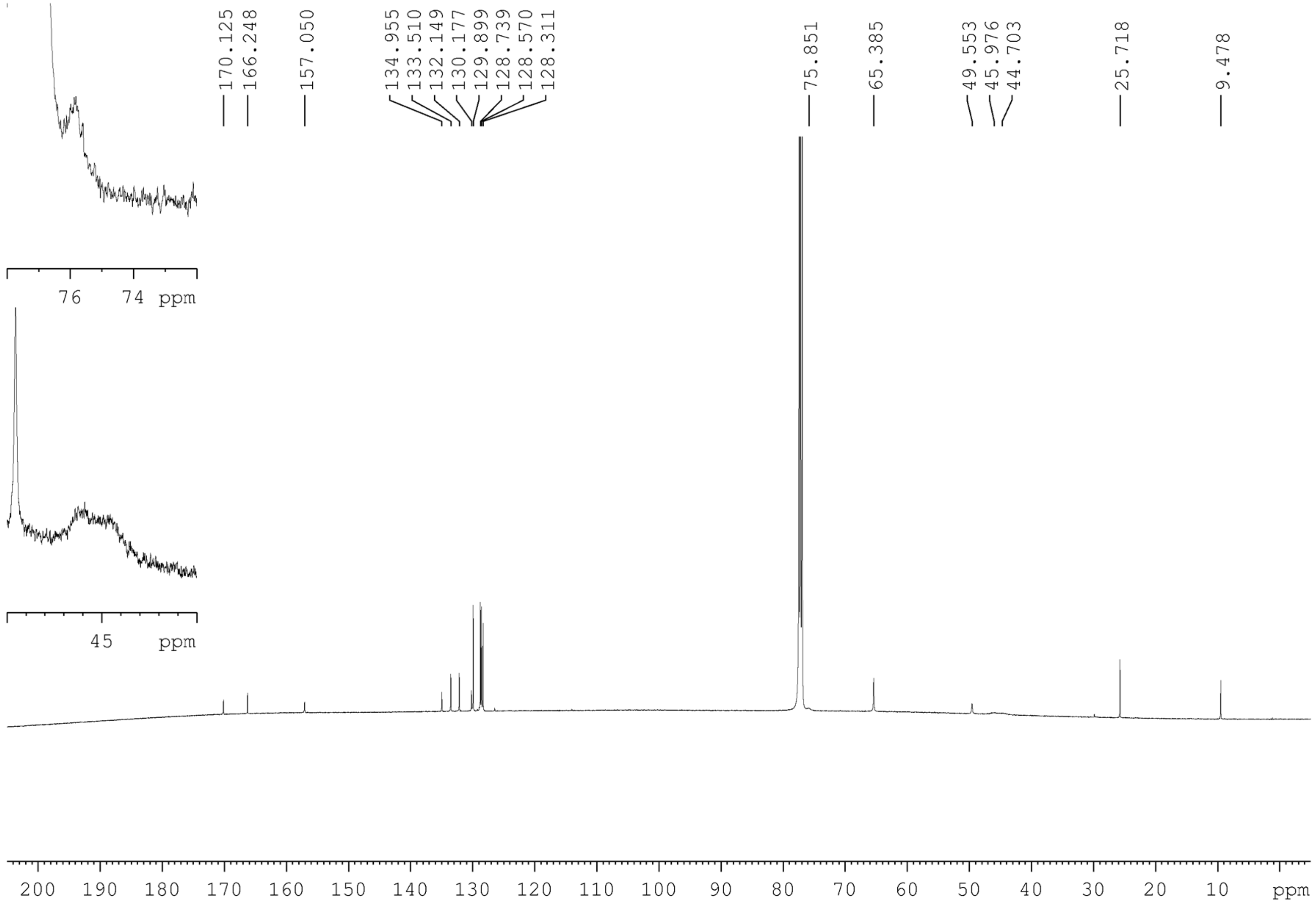

1-(*tert*-butyl)-3-(2-hydroxybutyl)urea (1zp-rac)

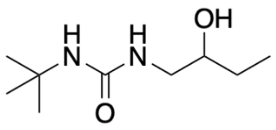

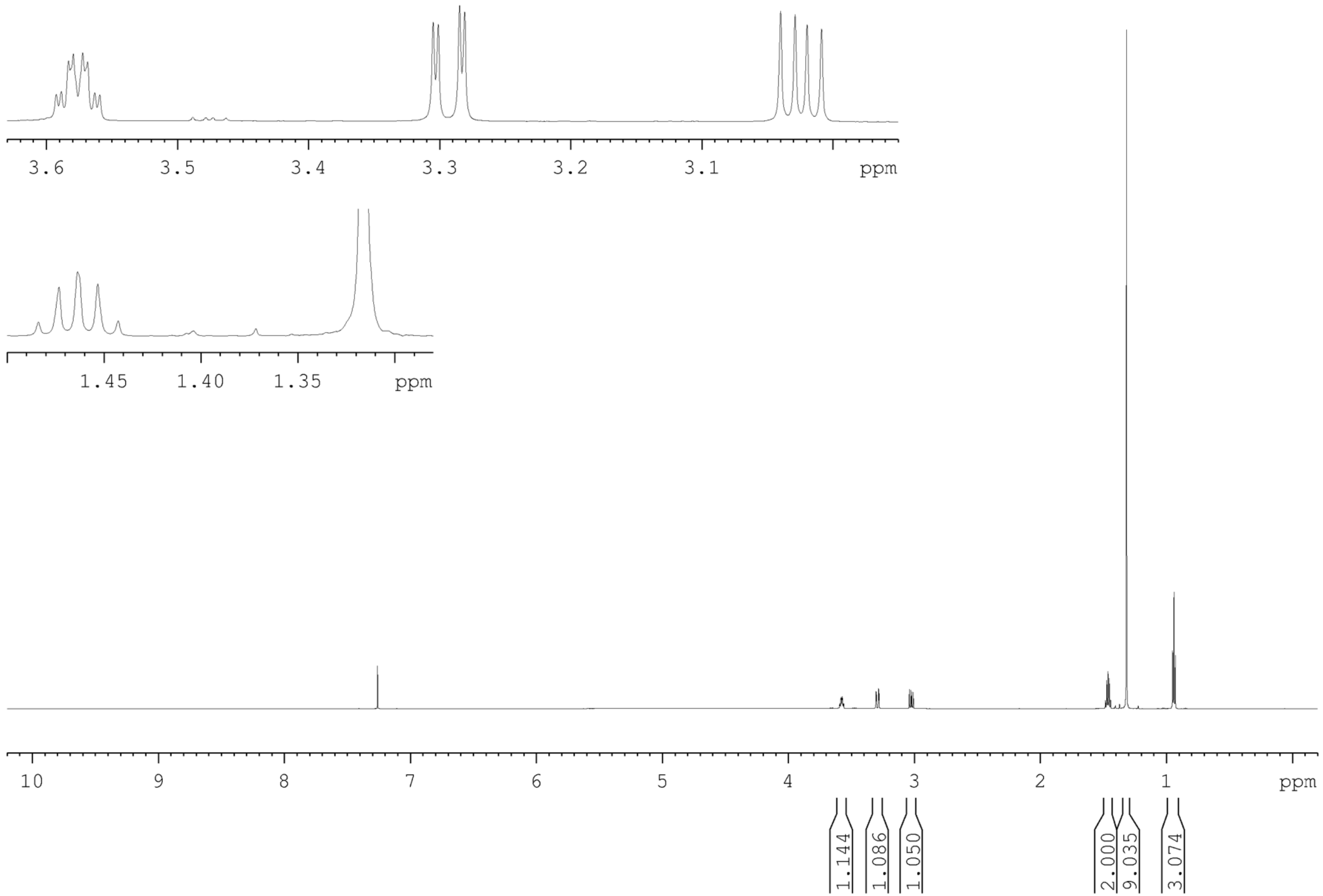

1-(*tert*-butyl)-3-(2-hydroxybutyl)urea (1zp-rac)

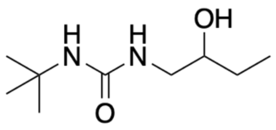

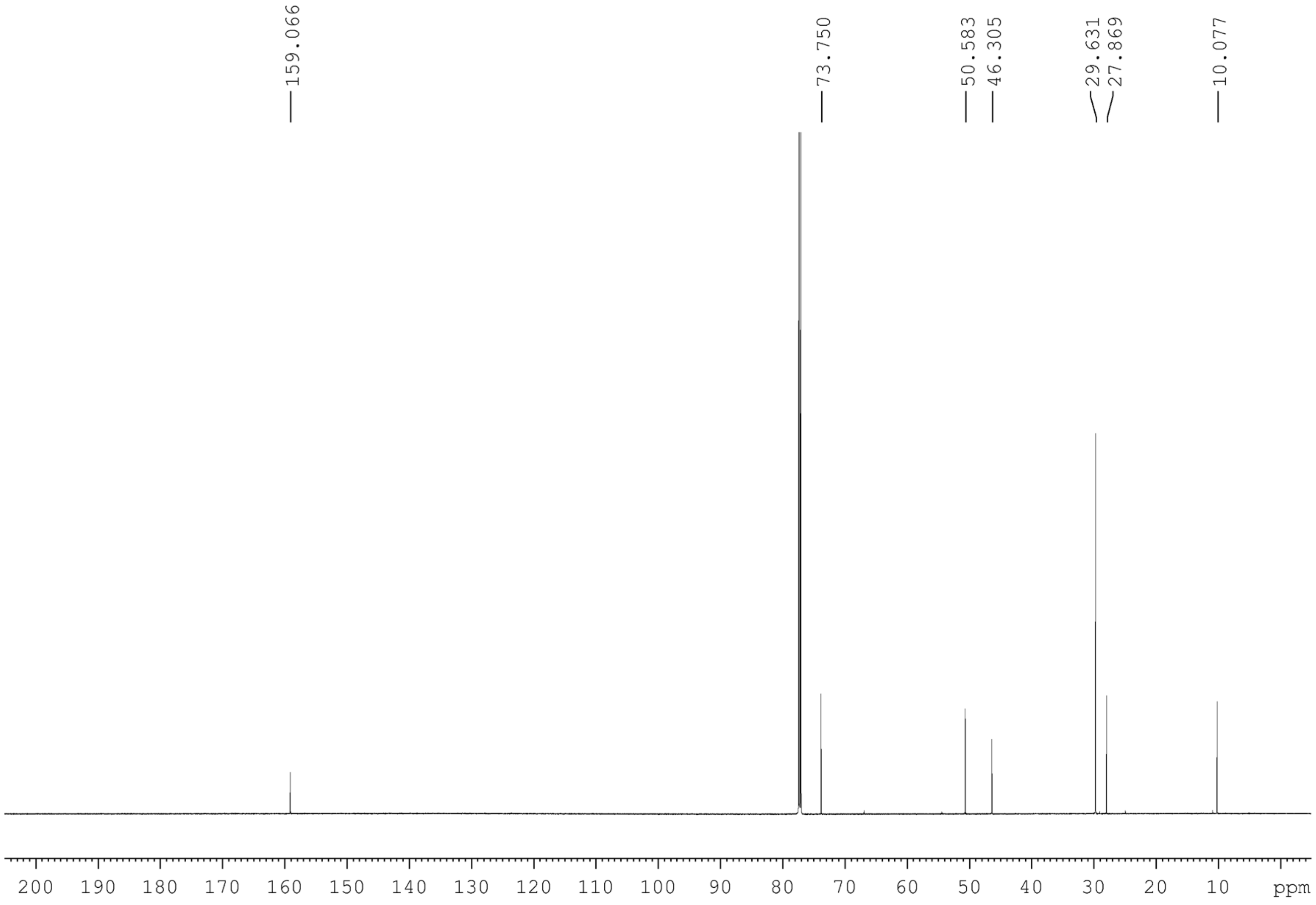

(*S*)-1-(*tert*-butyl)-3-(2-hydroxybutyl)urea (1zp)

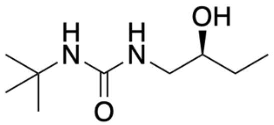

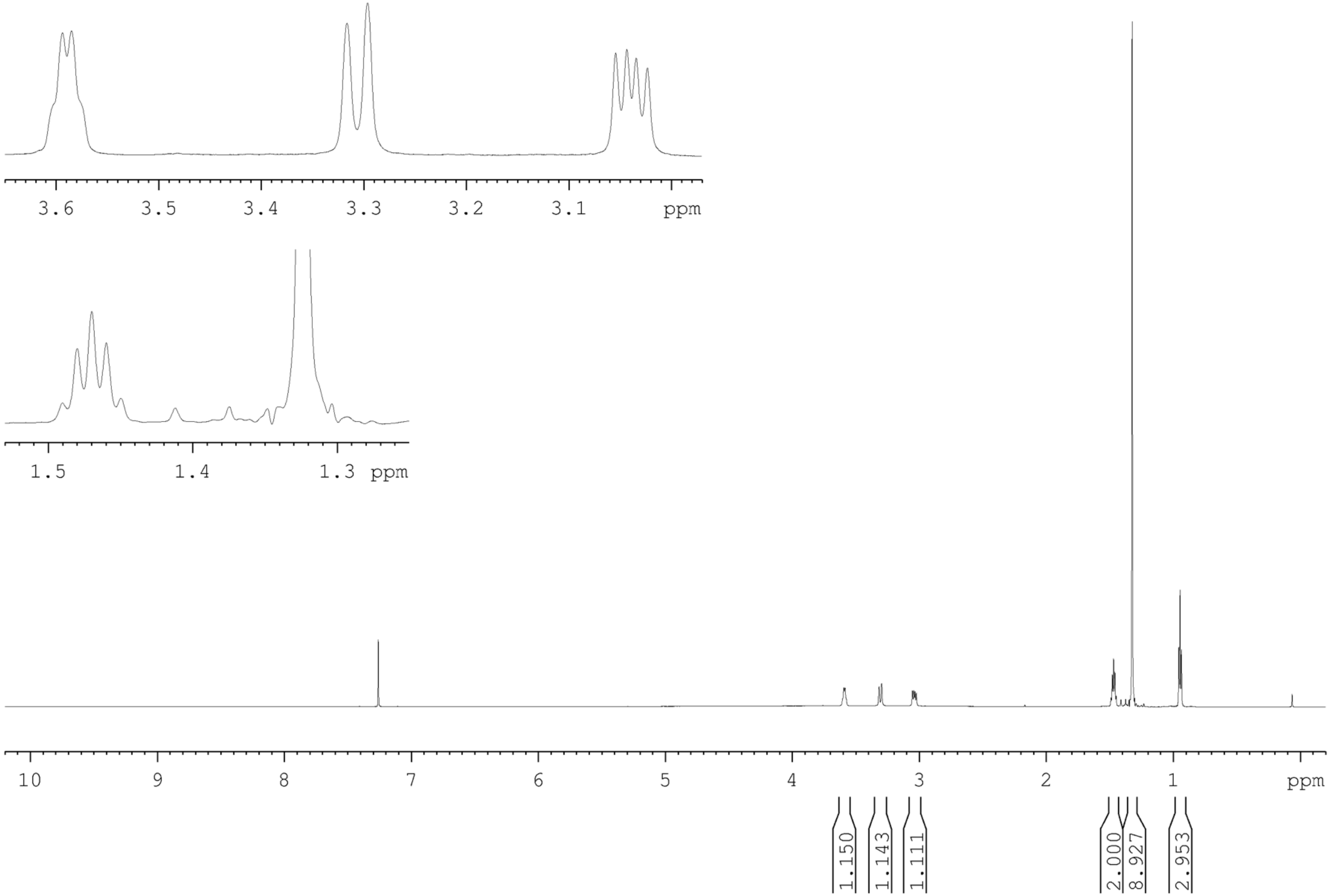

(*S*)-1-(*tert*-butyl)-3-(2-hydroxybutyl)urea (1zp)

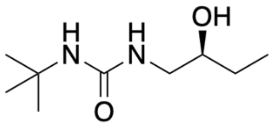

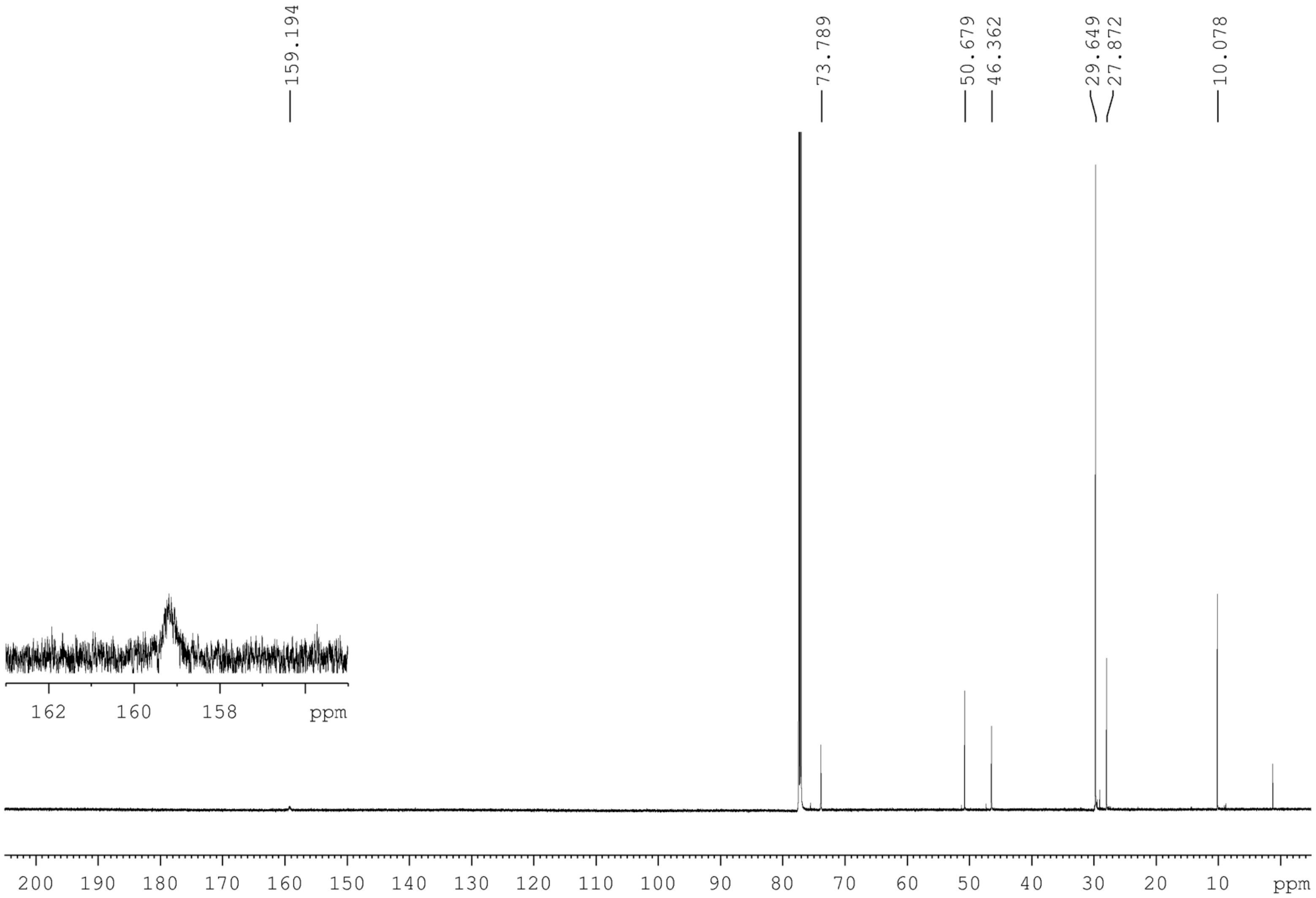

(S)-1-(N-(*tert*-butylcarbamoyl)benzamido)butan-2-yl benzoate (1zp-Bz)

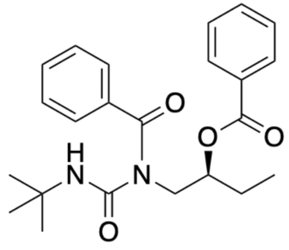

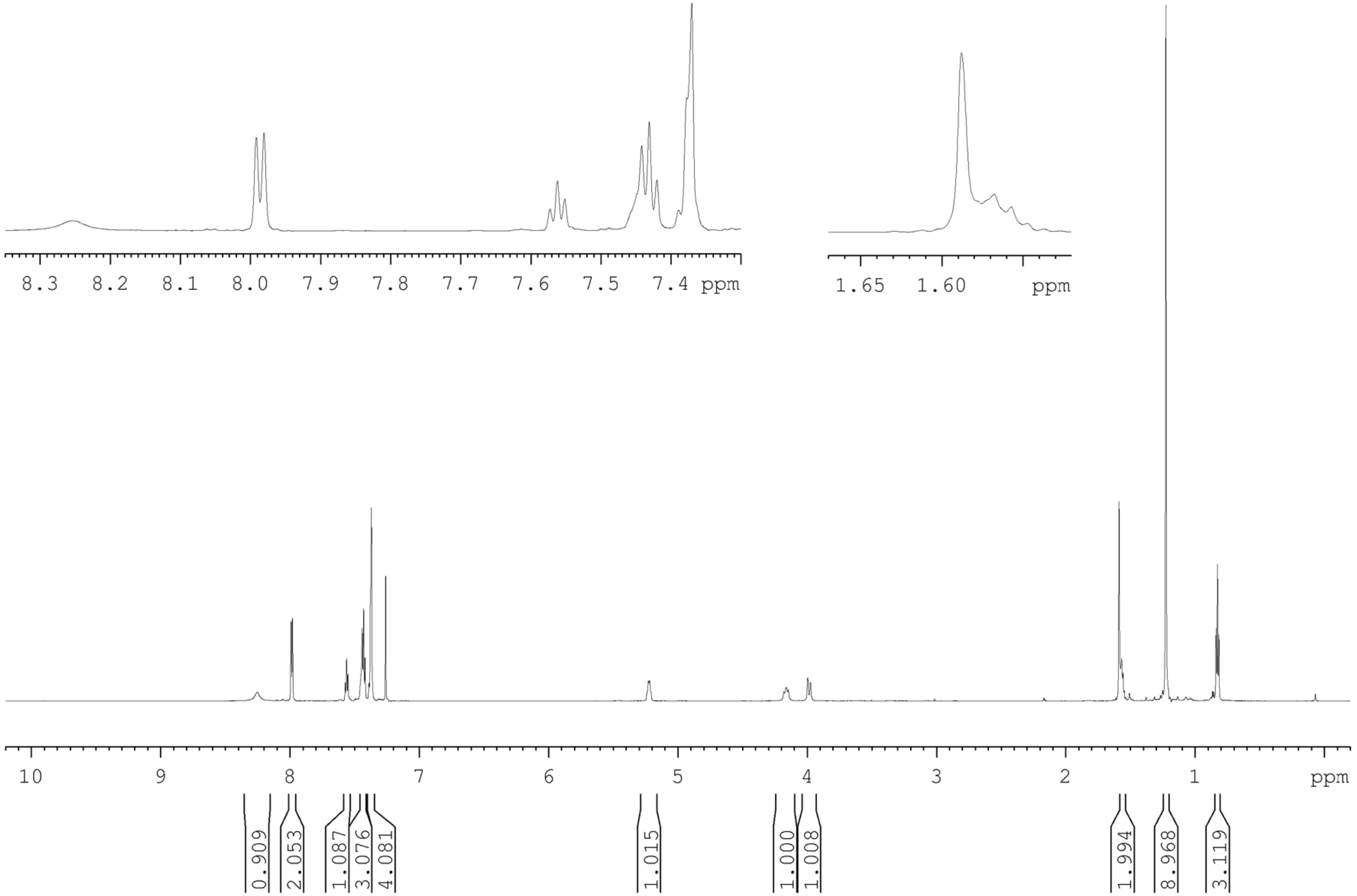

(*S*)-1-(*N*-(*tert*-butylcarbamoyl)benzamido)butan-2-yl benzoate (1zp-Bz)

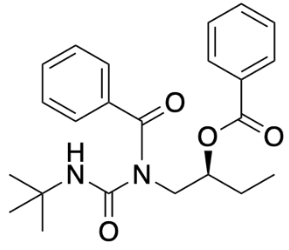

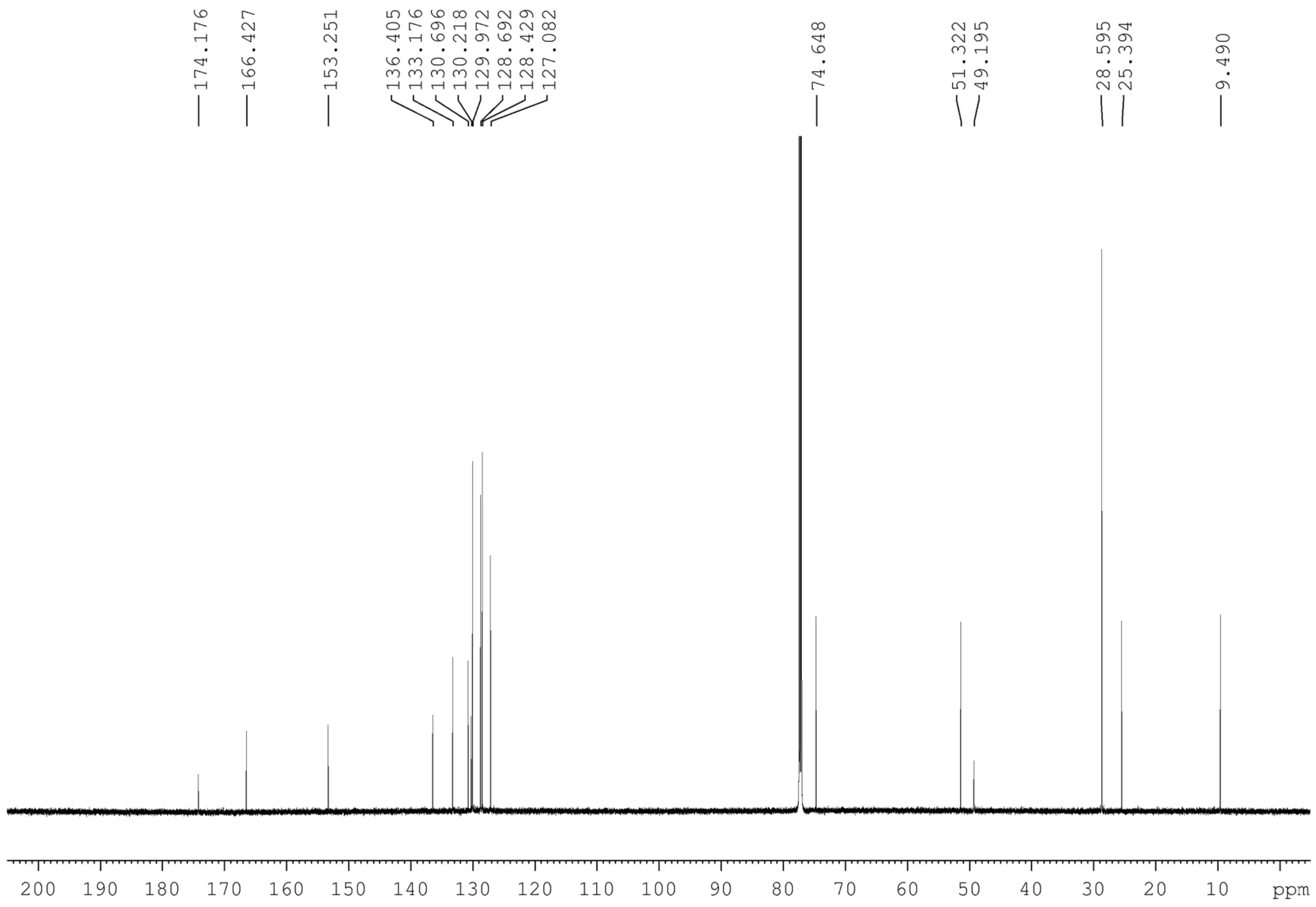

1-(3,5-bis(trifluoromethyl)phenyl)-3-(2-hydroxybutyl)urea (1zq-rac)

In $(CD_3)_2CO$

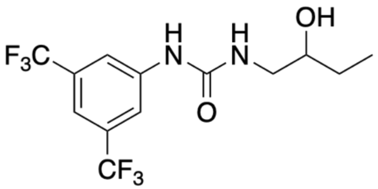

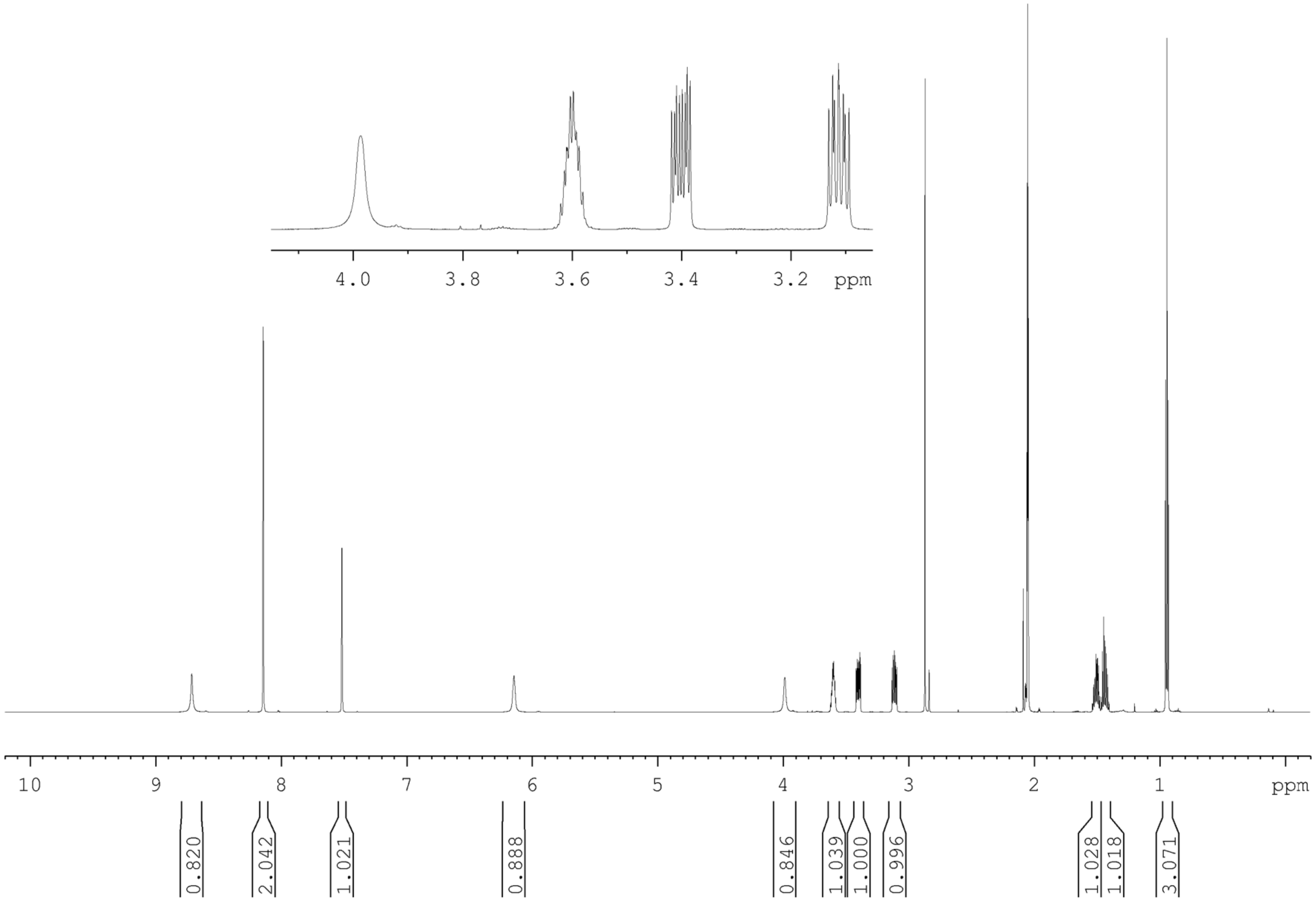

1-(3,5-bis(trifluoromethyl)phenyl)-3-(2-hydroxybutyl)urea (1zq-rac)

In $(CD_3)_2CO$

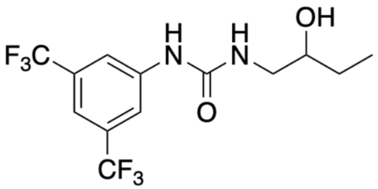

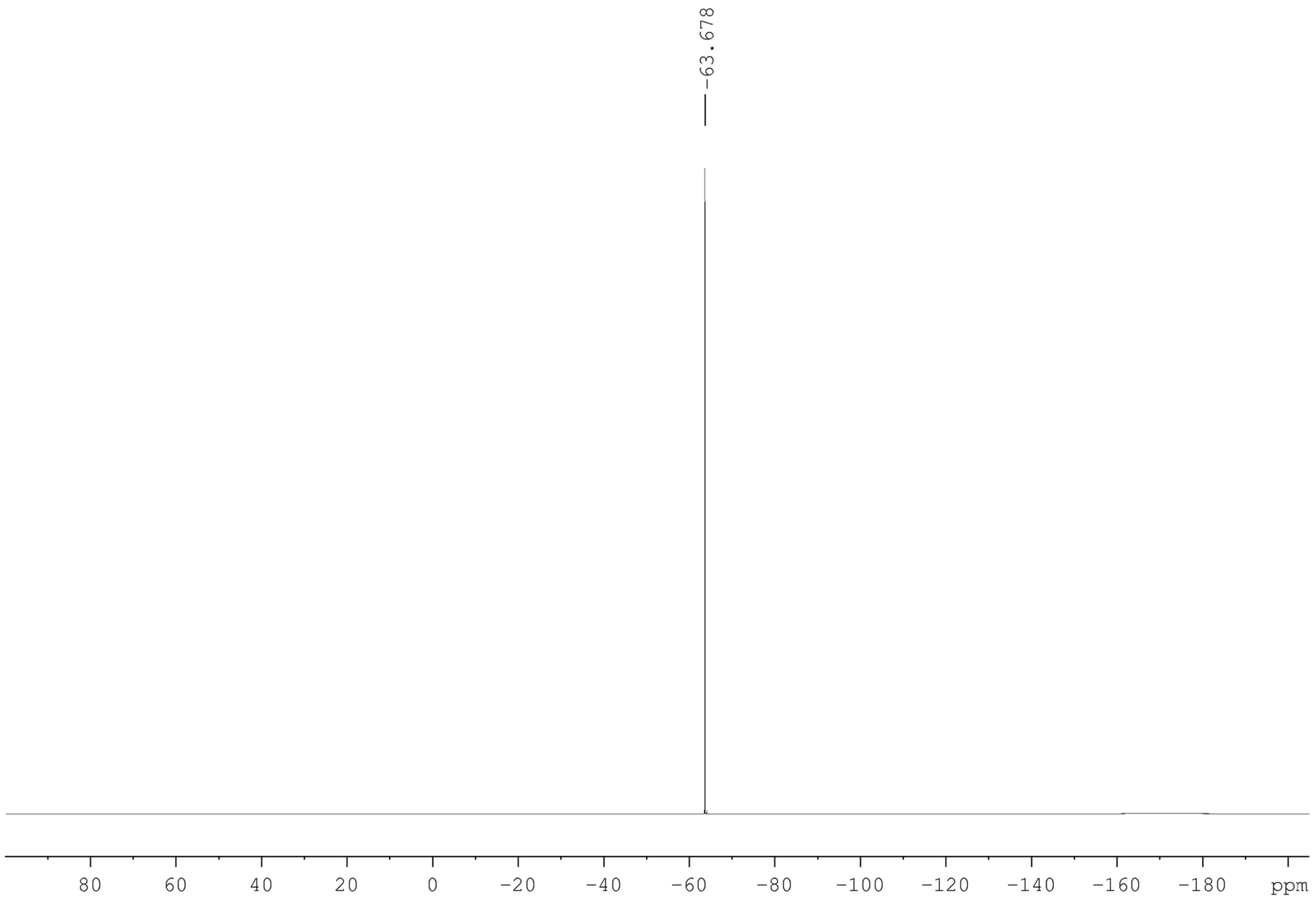

1-(3,5-bis(trifluoromethyl)phenyl)-3-(2-hydroxybutyl)urea (1zq-rac)

In $(CD_3)_2CO$

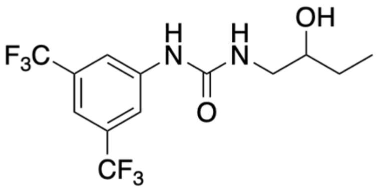

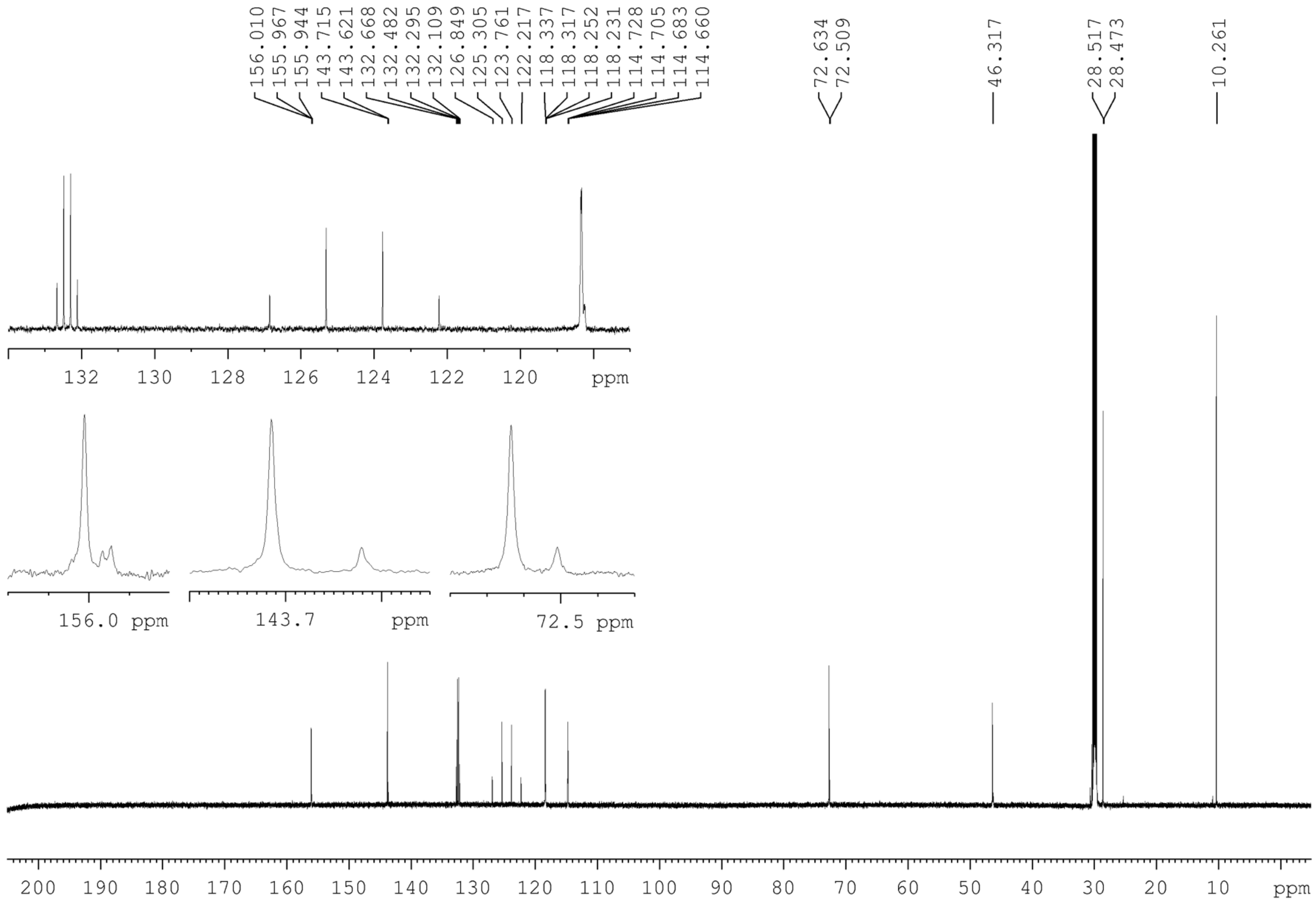

(*S*)-1-(3,5-bis(trifluoromethyl)phenyl)-3-(2-hydroxybutyl)urea (1zq)

In $(CD_3)_2CO$

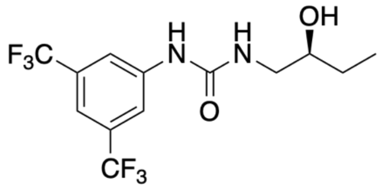

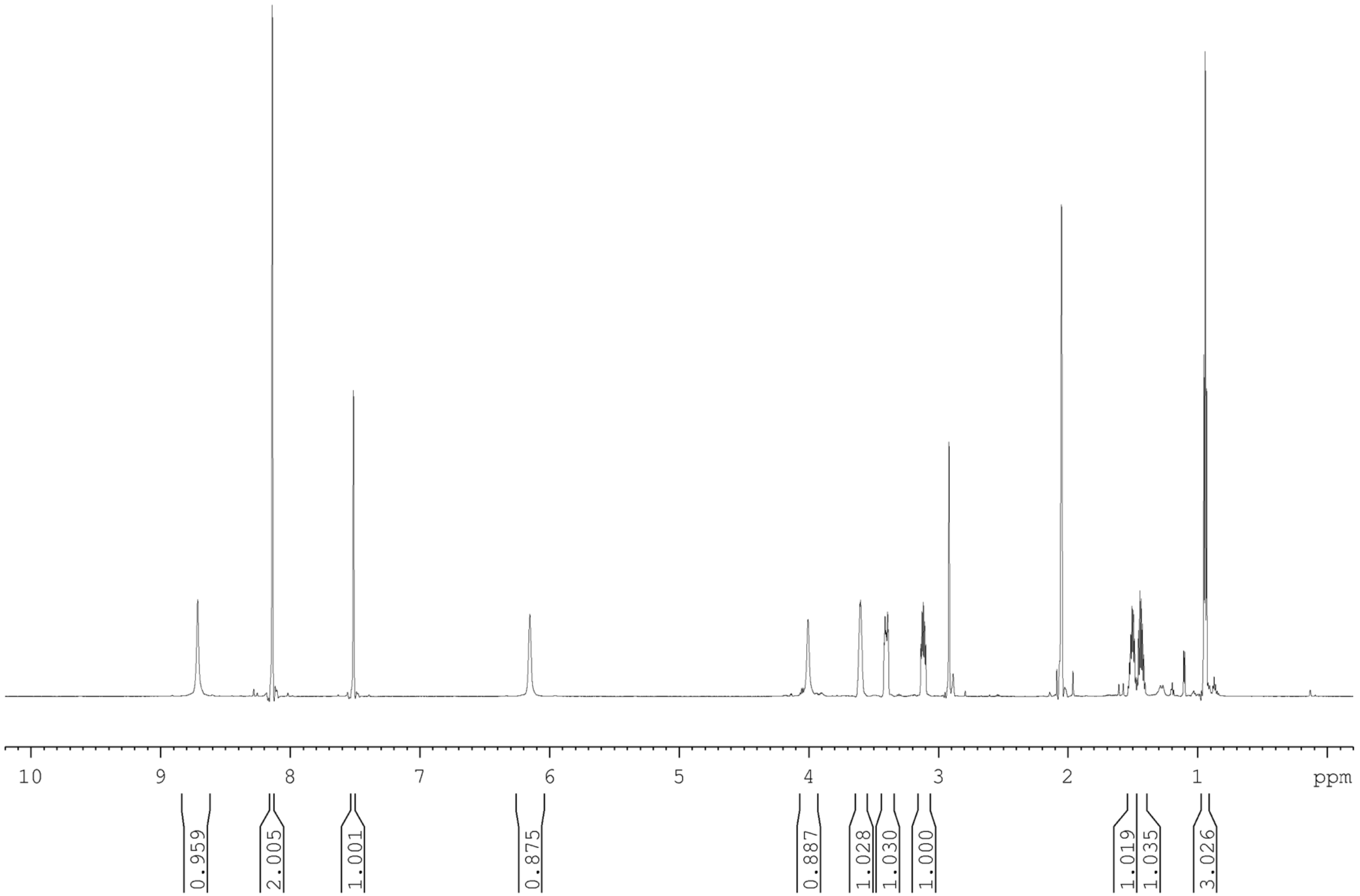

(*S*)-1-(3,5-bis(trifluoromethyl)phenyl)-3-(2-hydroxybutyl)urea (1zq)

In $(CD_3)_2CO$

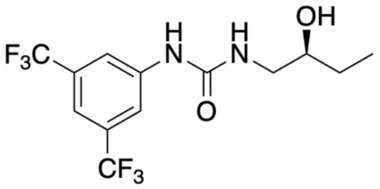

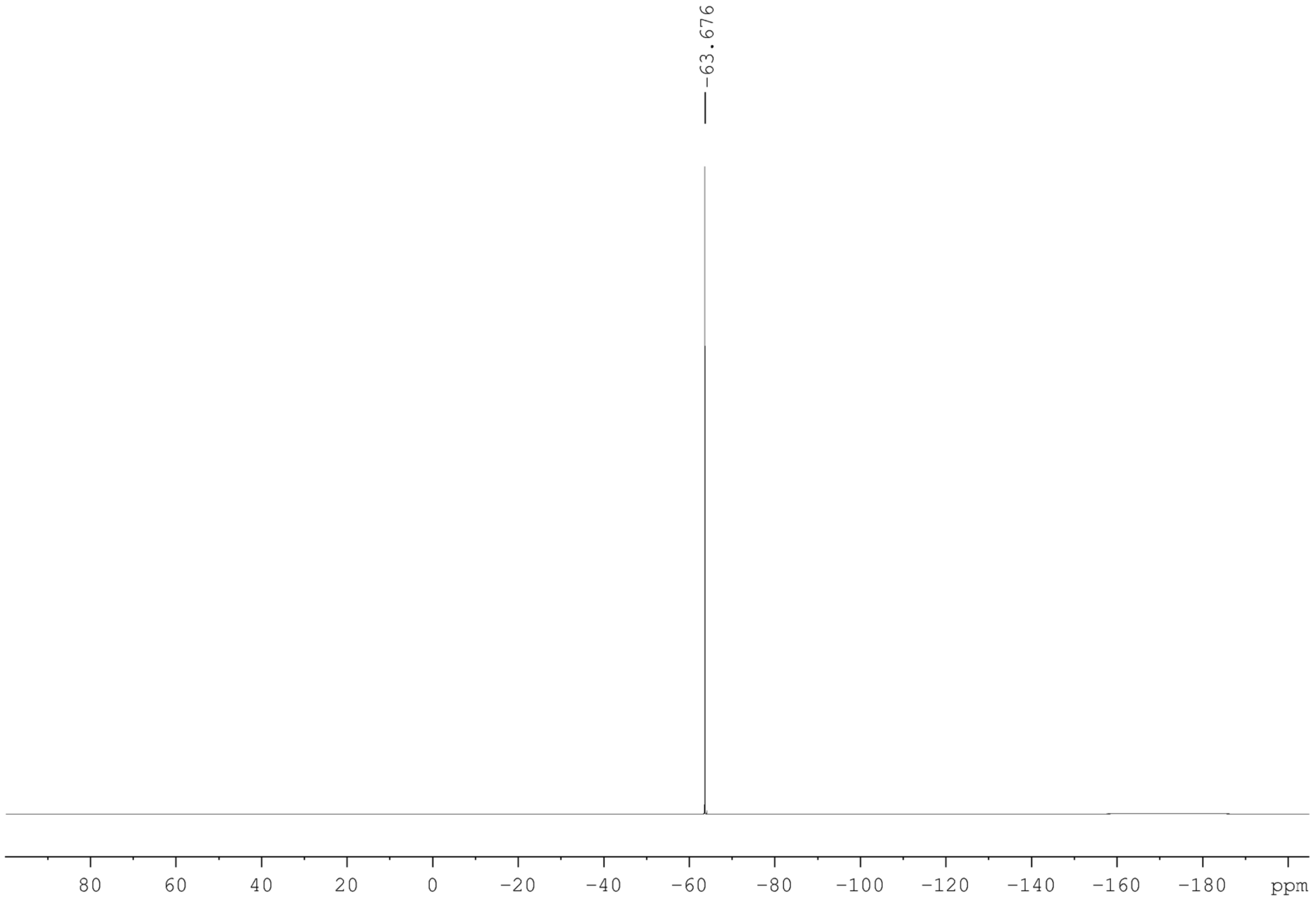

(*S*)-1-(3,5-bis(trifluoromethyl)phenyl)-3-(2-hydroxybutyl)urea (1zq)

In $(CD_3)_2CO$

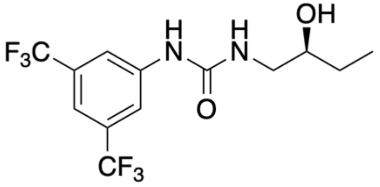

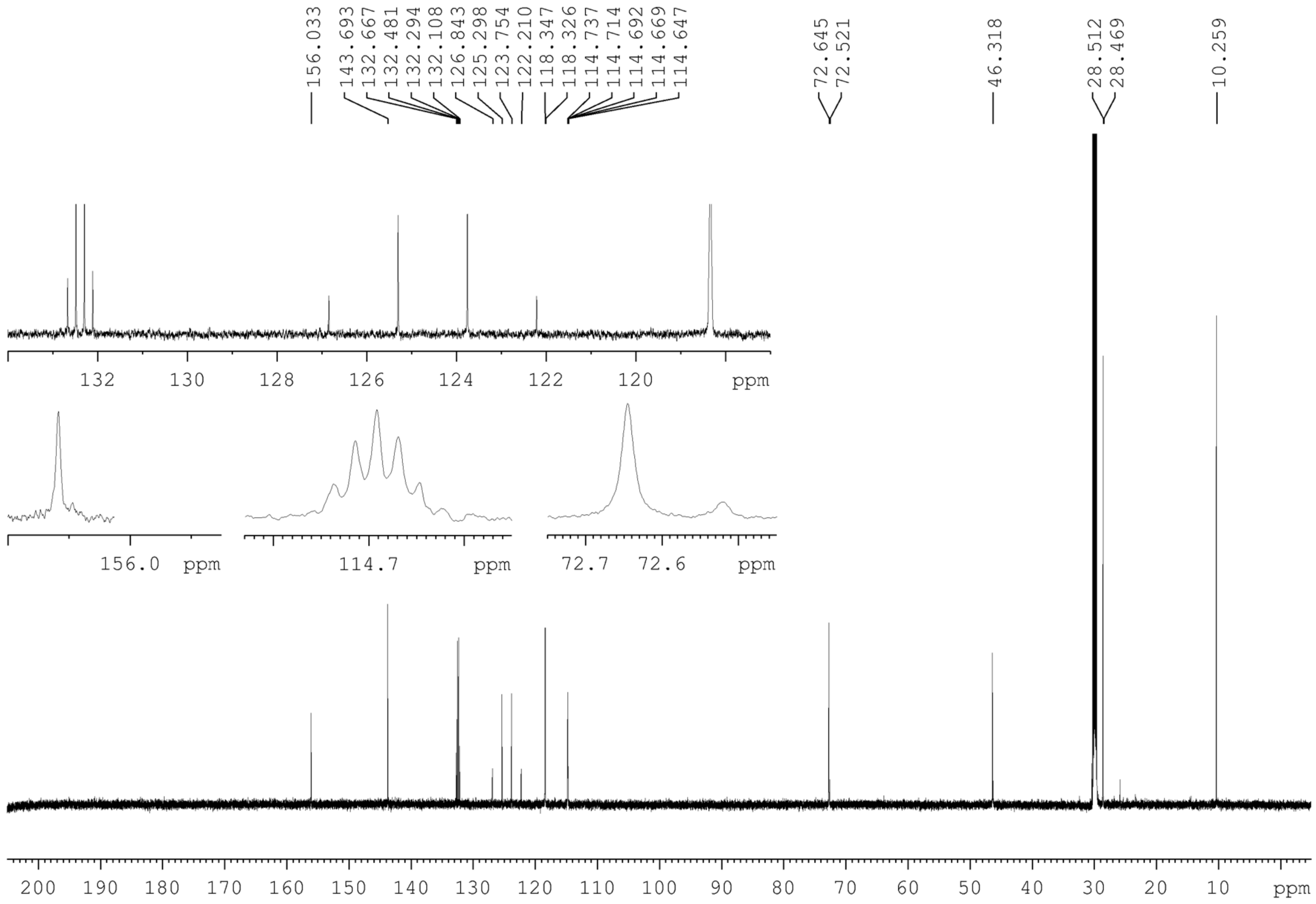

***tert*-butyl (2-hydroxybutyl)carbamate (1zr-rac)**

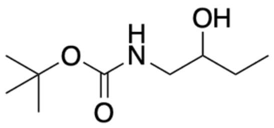

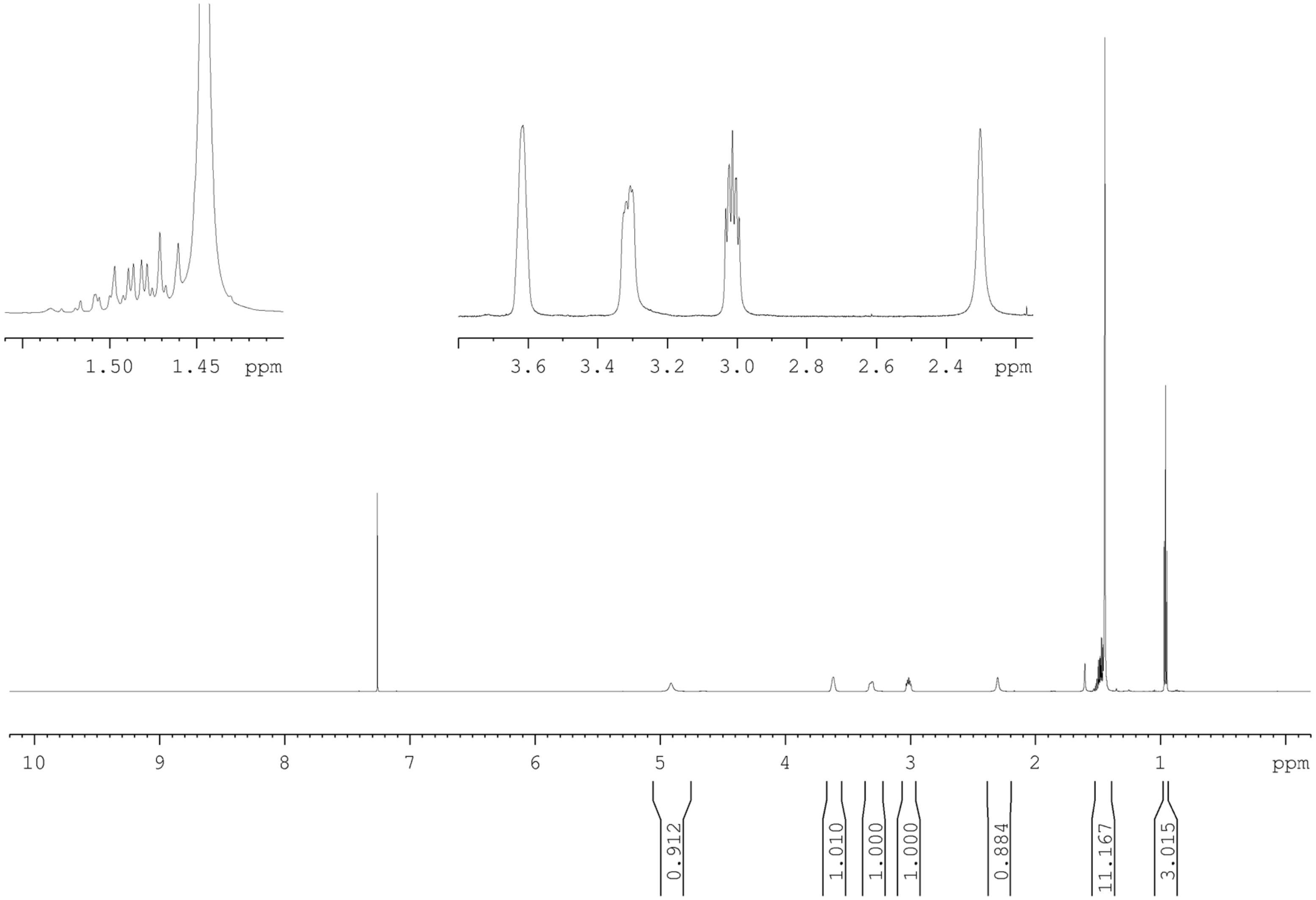

tert-butyl (2-hydroxybutyl)carbamate (1zr-rac)

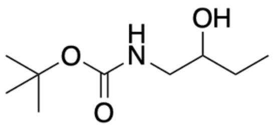

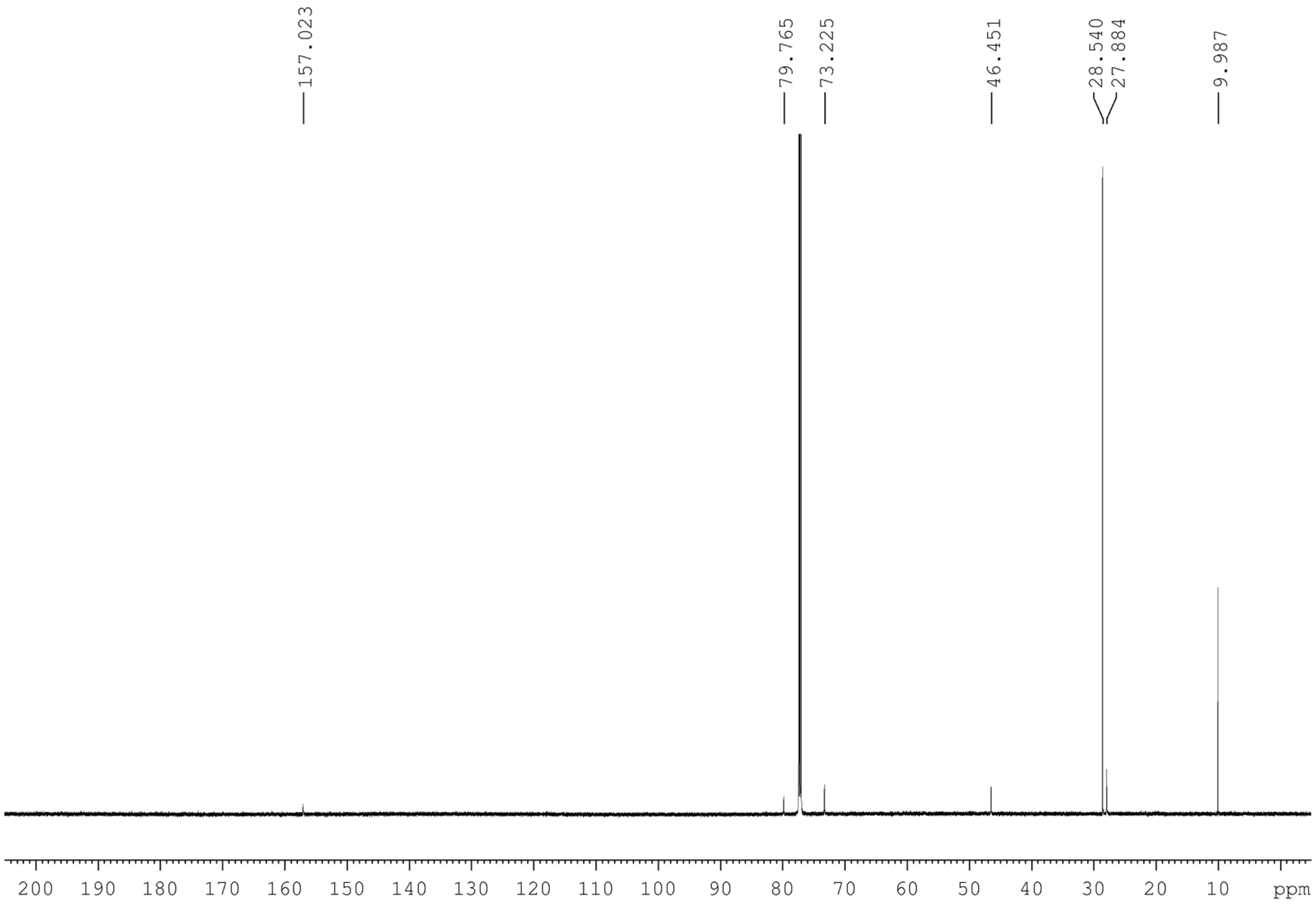

tert-butyl (_S_)-(2-hydroxybutyl)carbamate (1zr)

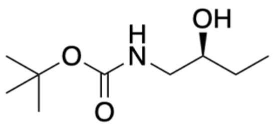

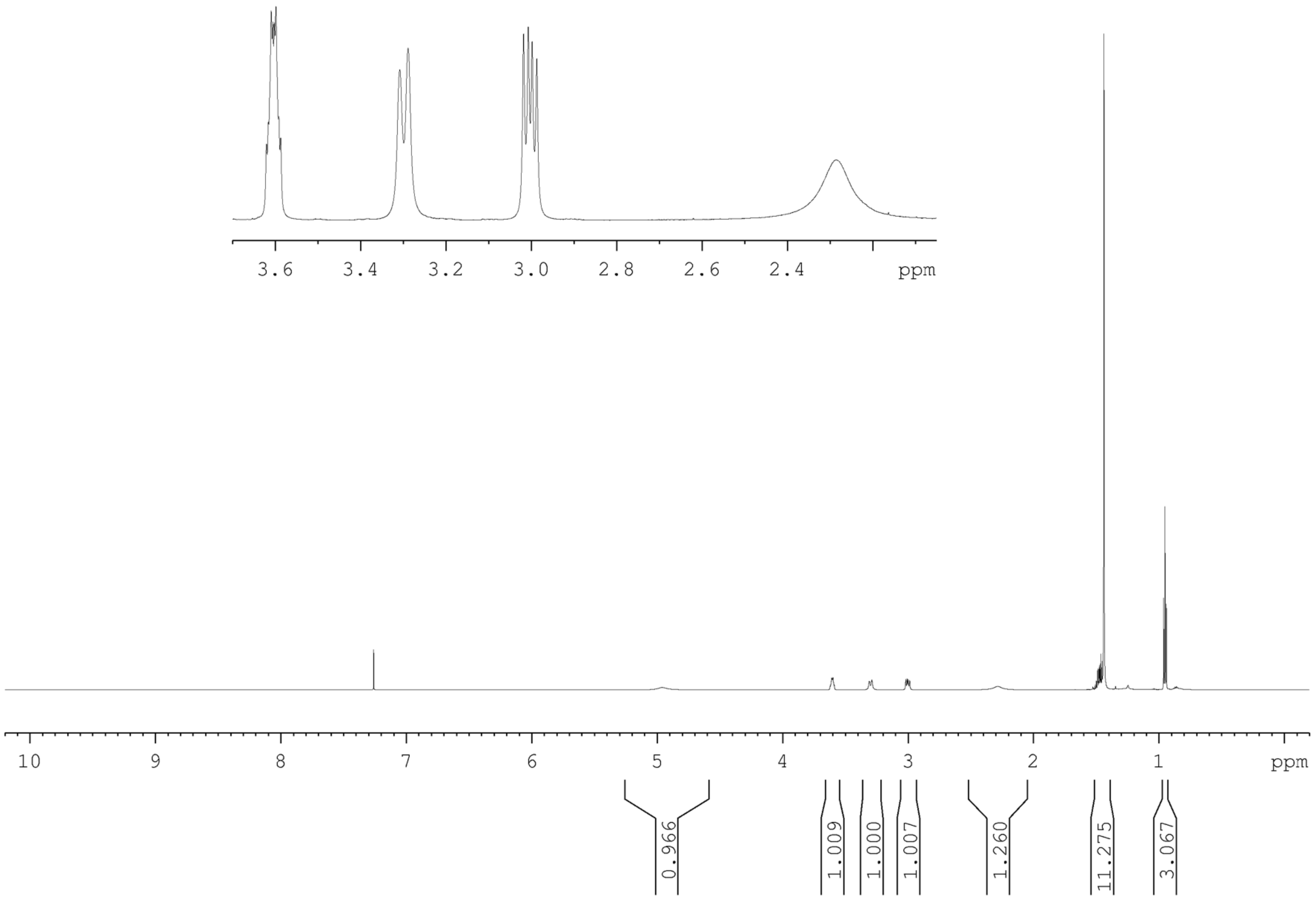

tert-butyl (_S_)-(2-hydroxybutyl)carbamate (1zr)

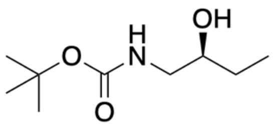

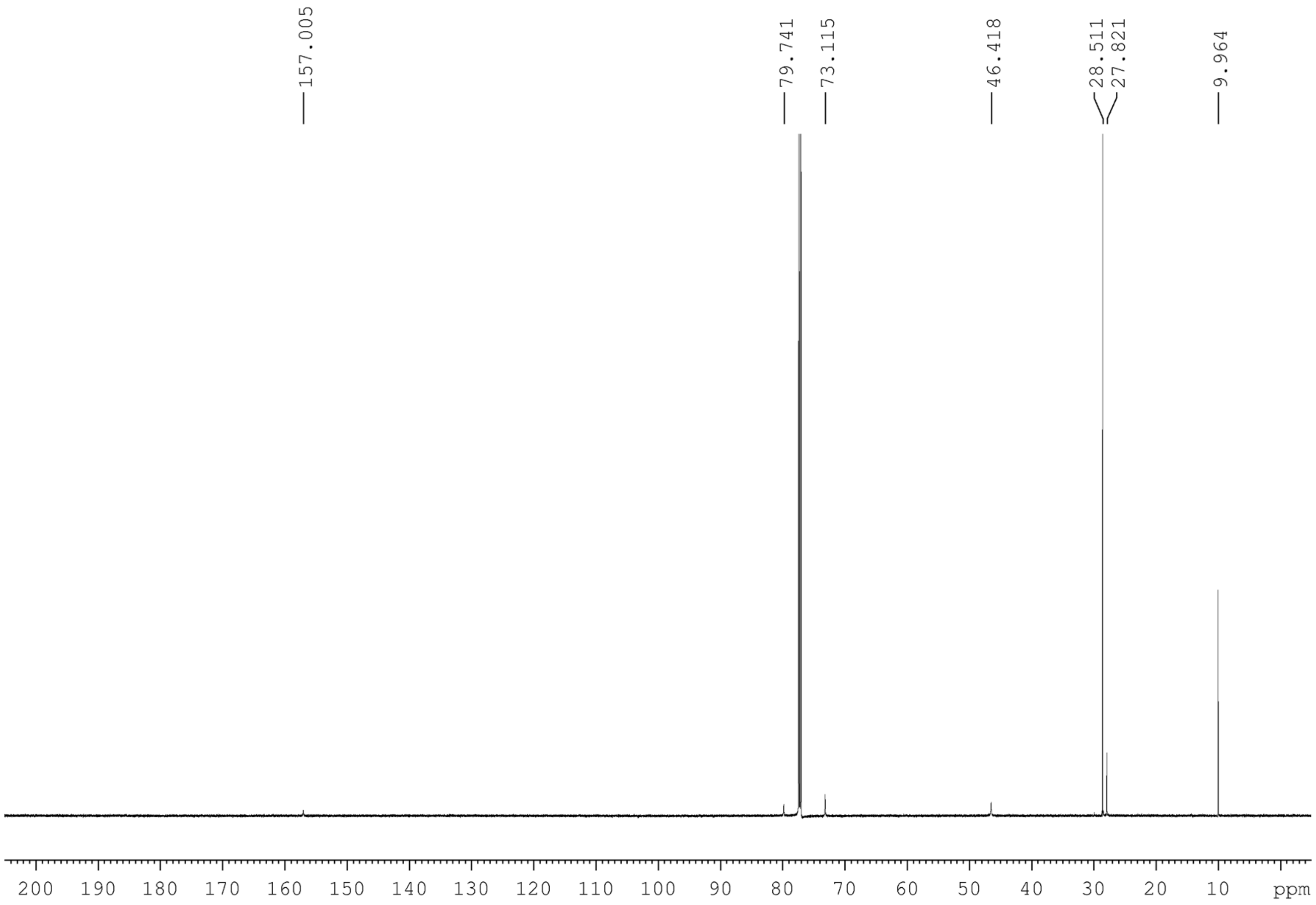

(*S*)-1-(*N*-(*tert*-butoxycarbonyl)benzamido)butan-2-yl benzoate (1zr-Bz)

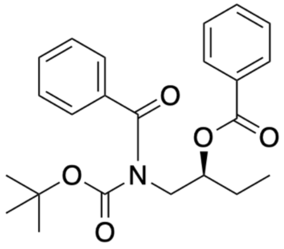

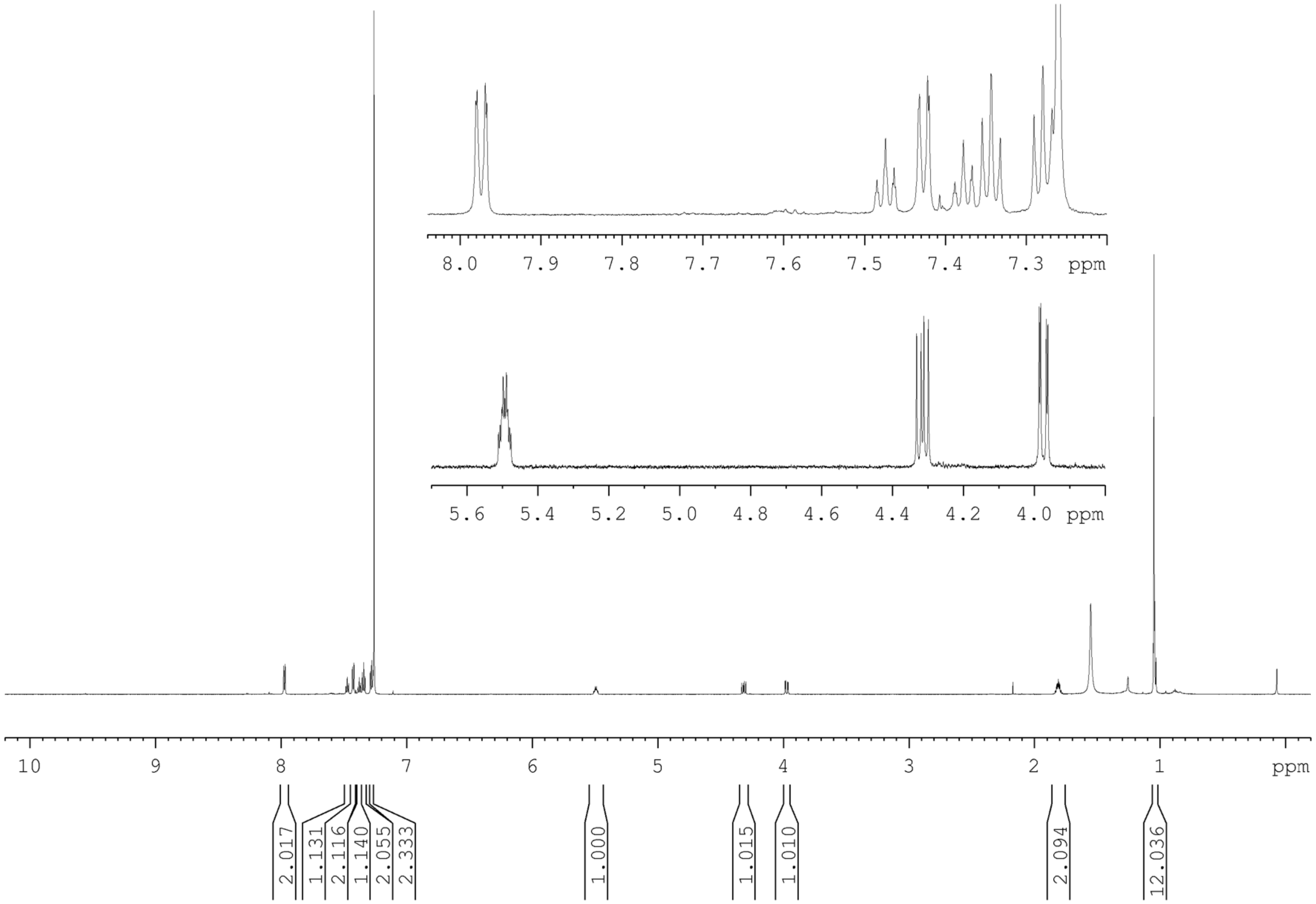

(*S*)-1-(*N*-(*tert*-butoxycarbonyl)benzamido)butan-2-yl benzoate (1zr-Bz)

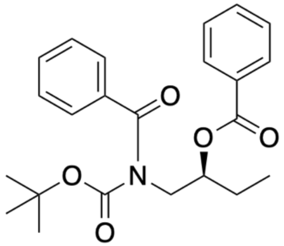

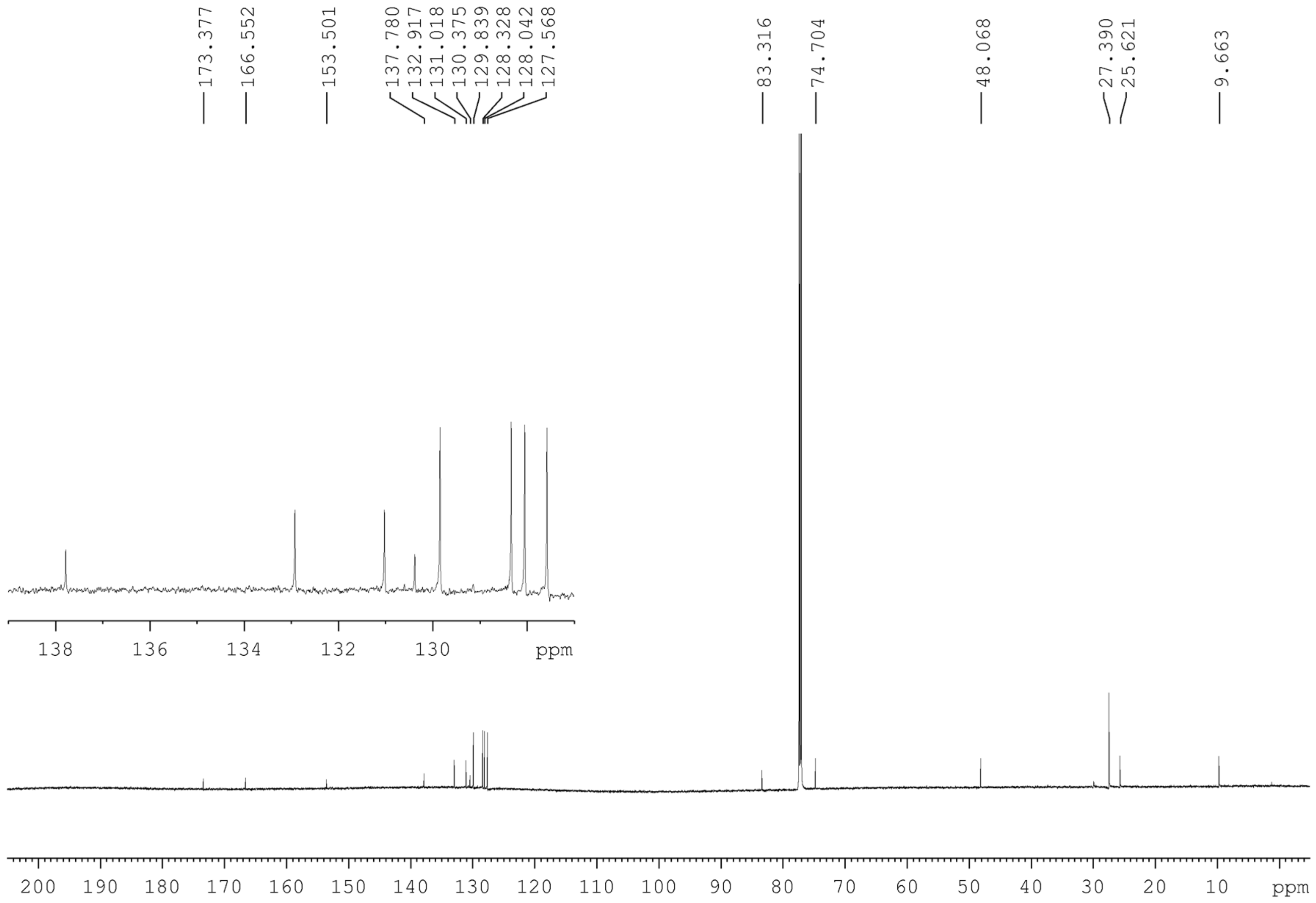

2-(benzyloxy)-*N*-(2-hydroxybutyl)acetamide (1zs-rac)

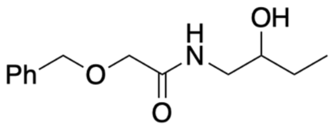

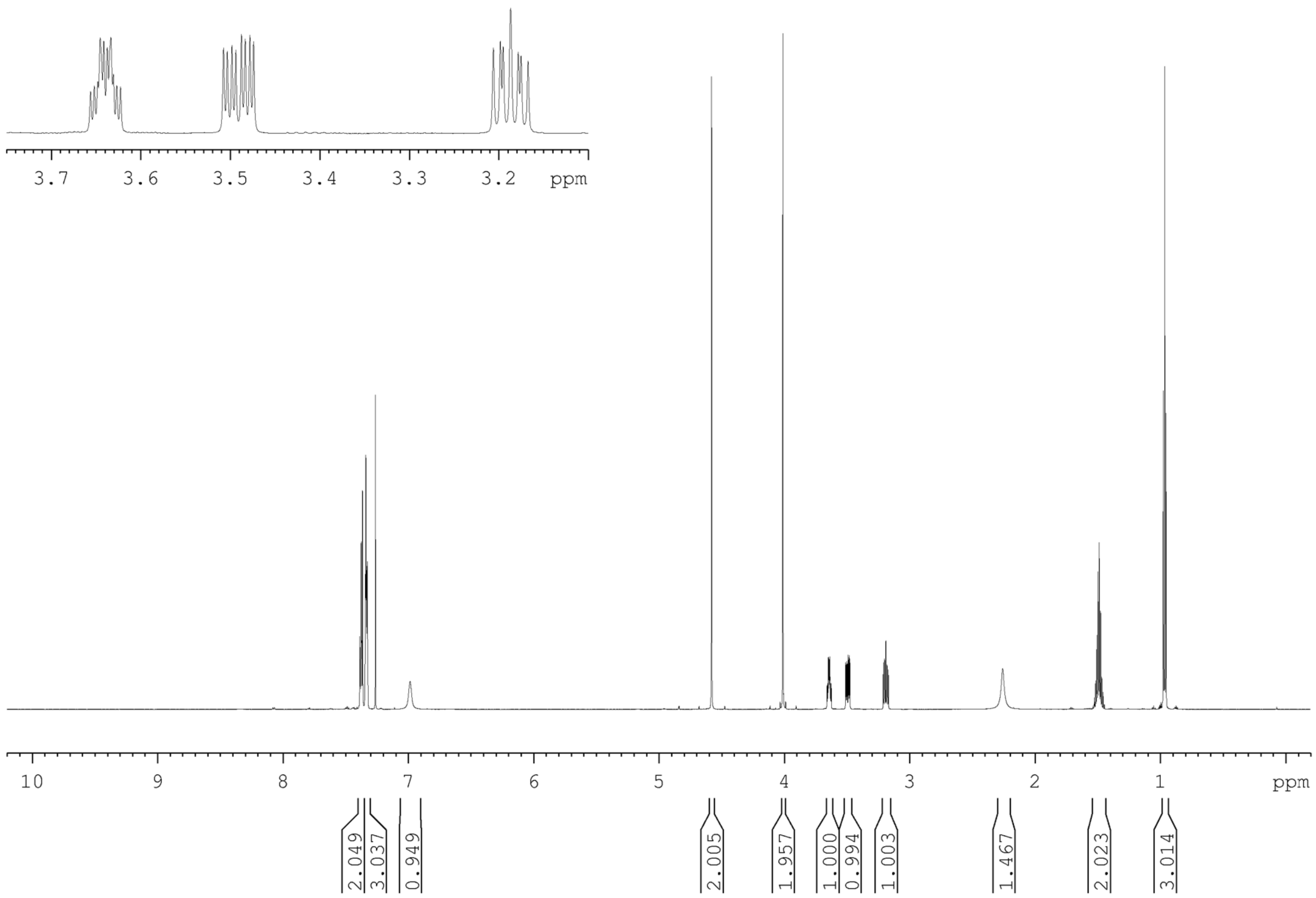

2-(benzyloxy)-*N*-(2-hydroxybutyl)acetamide (1zs-rac)

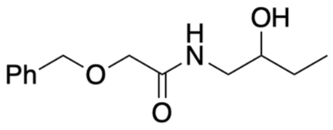

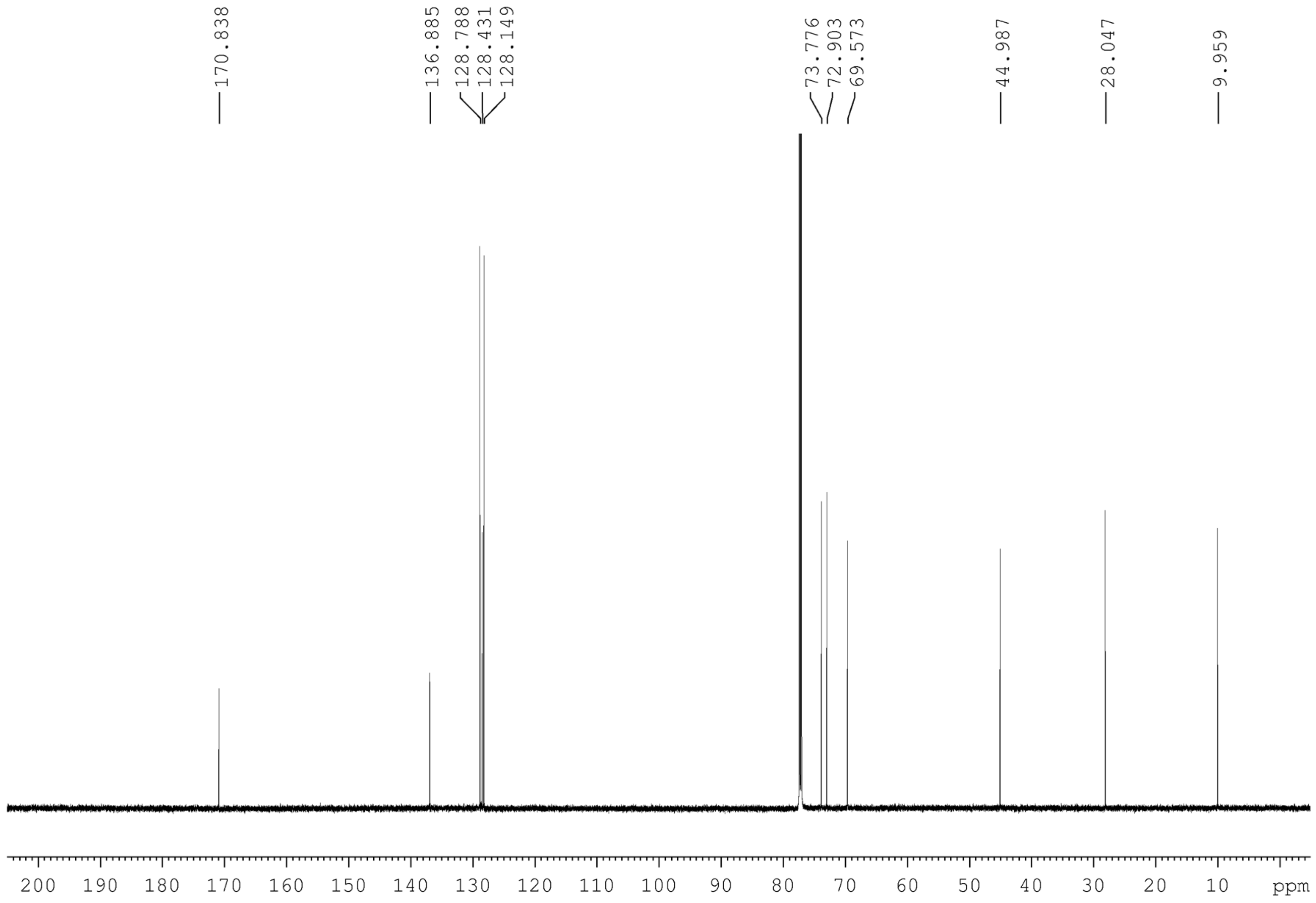

(*S*)-2-(benzyloxy)-*N*-(2-hydroxybutyl)acetamide (1zs)

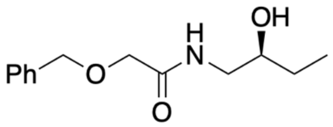

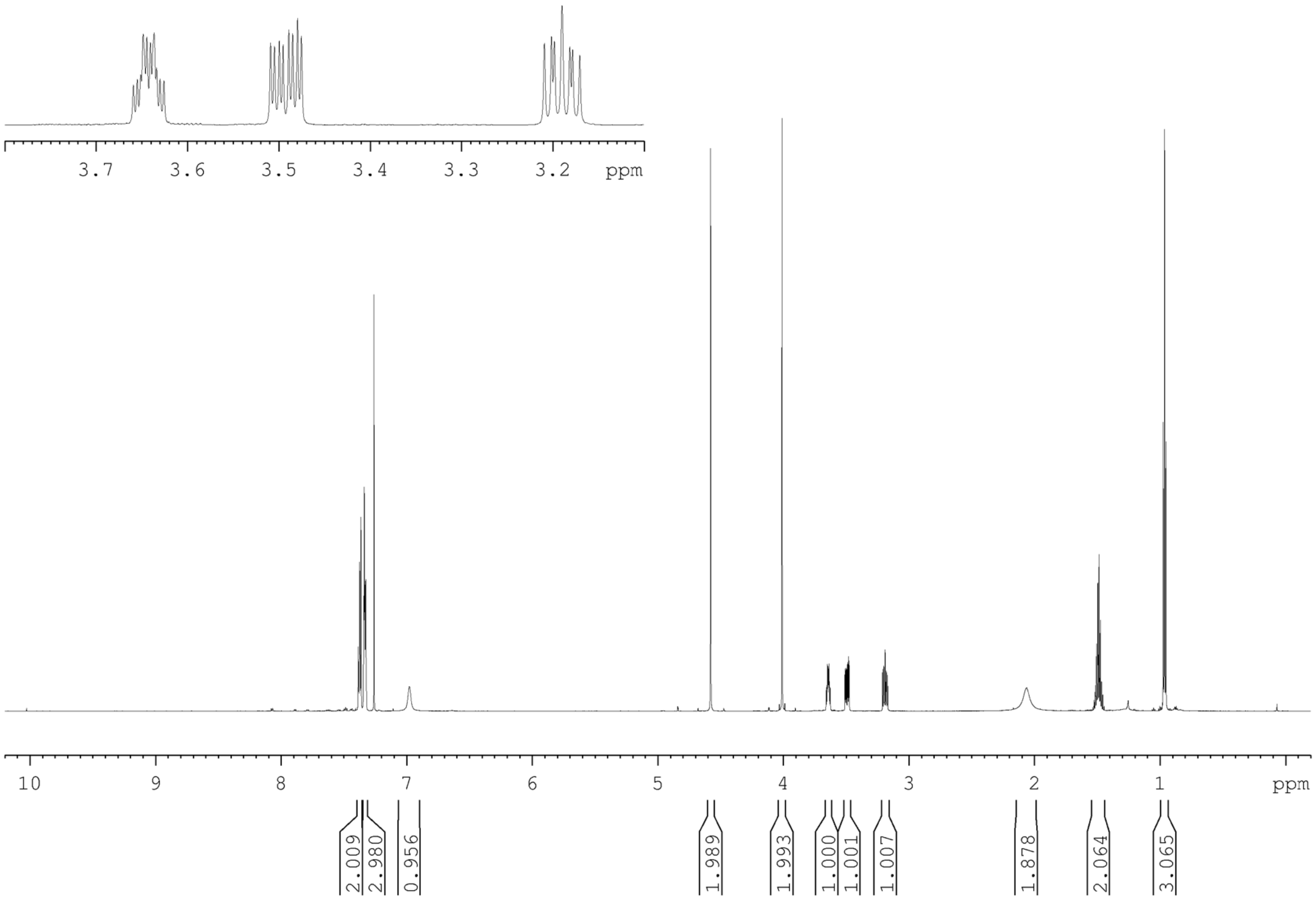

(*S*)-2-(benzyloxy)-*N*-(2-hydroxybutyl)acetamide (1zs)

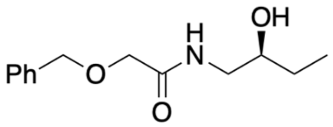

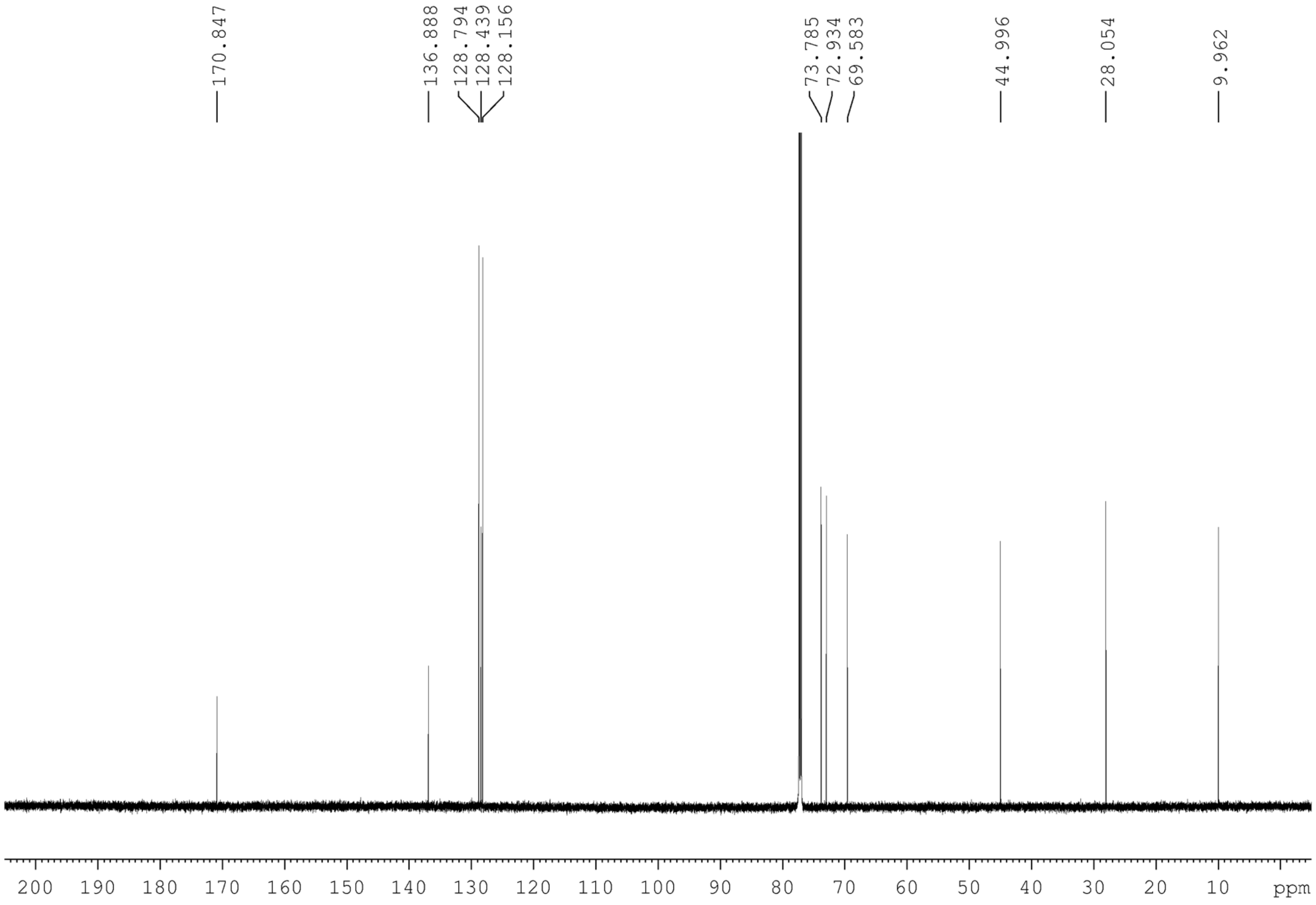

N-(2-hydroxybutyl)-2-(methylthio)acetamide (1zt-rac)

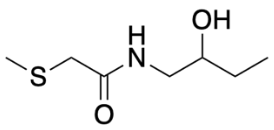

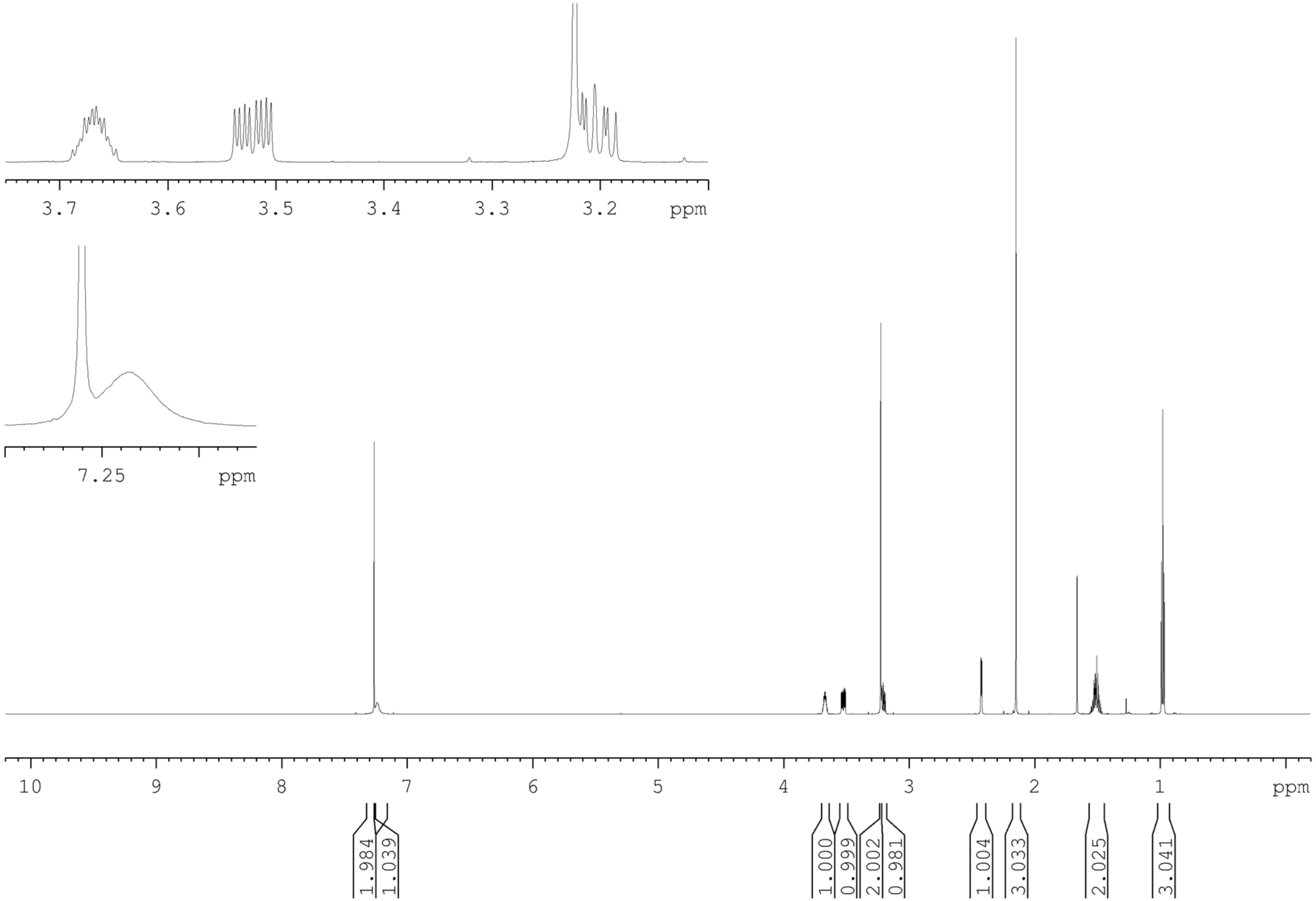

N-(2-hydroxybutyl)-2-(methylthio)acetamide (1zt-rac)

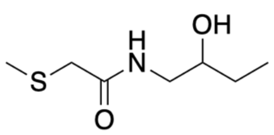

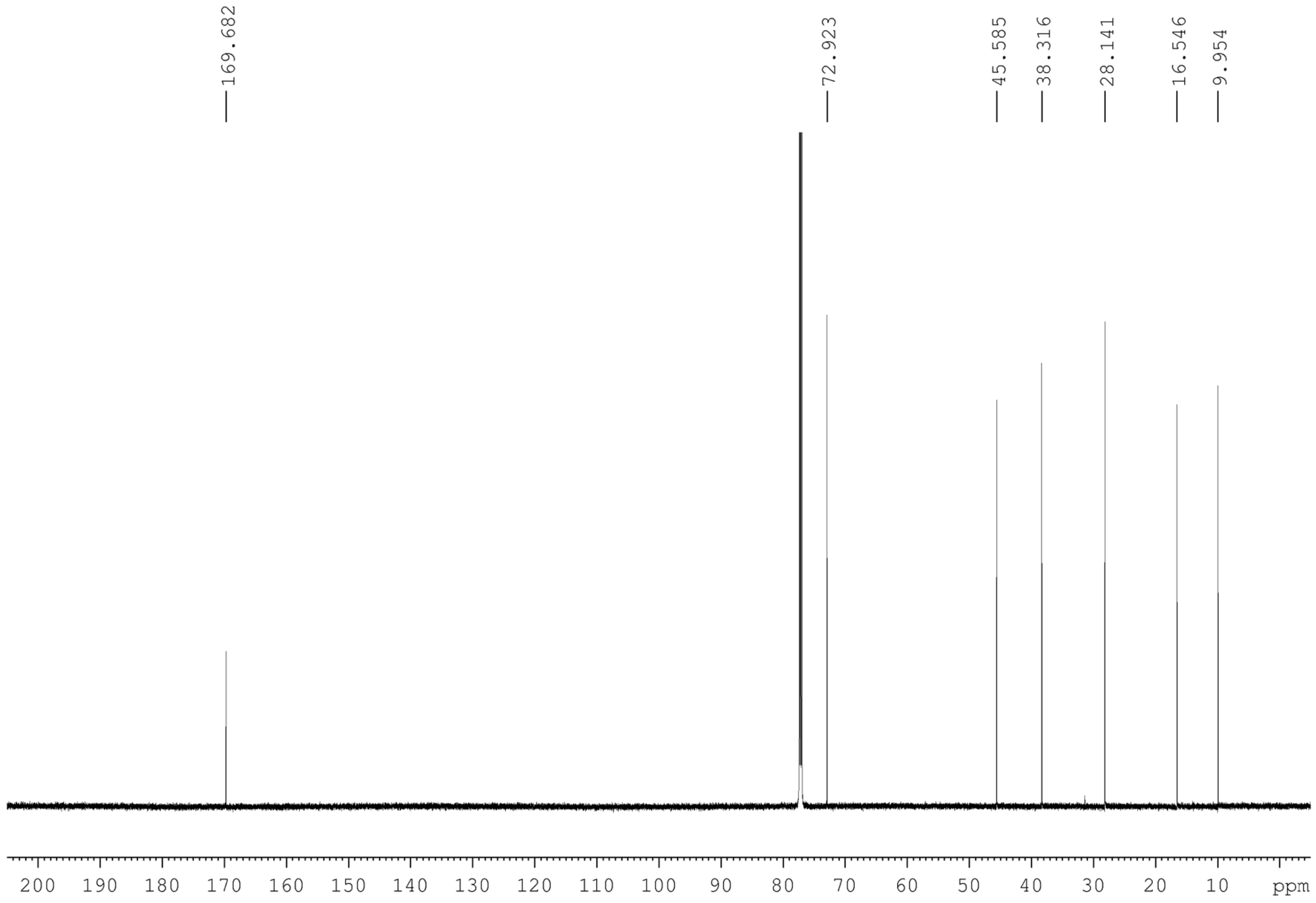

(*S*)-*N*-(2-hydroxybutyl)-2-(methylthio)acetamide (1zt)

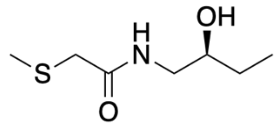

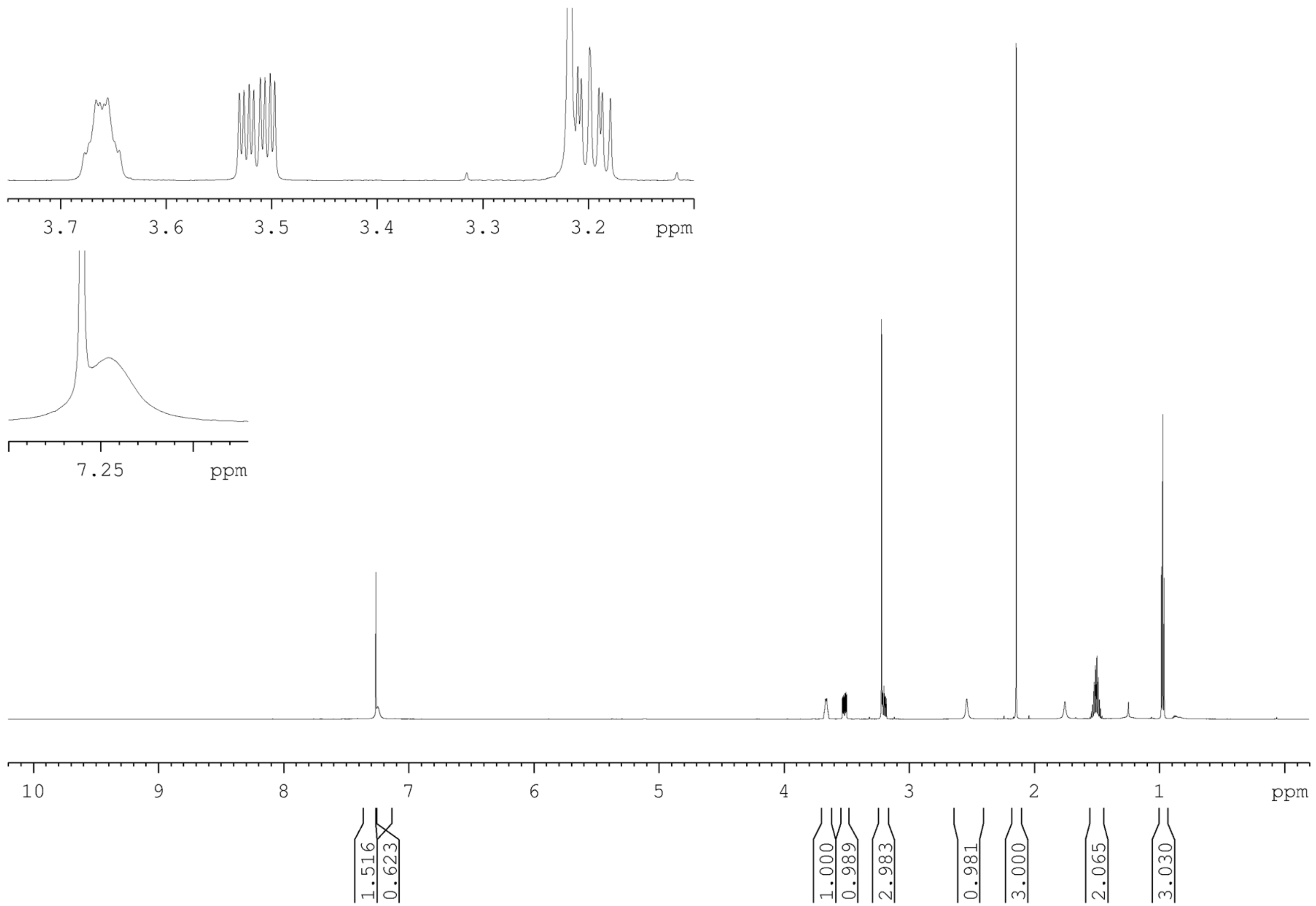

(*S*)-*N*-(2-hydroxybutyl)-2-(methylthio)acetamide (1zt)

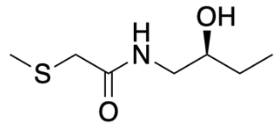

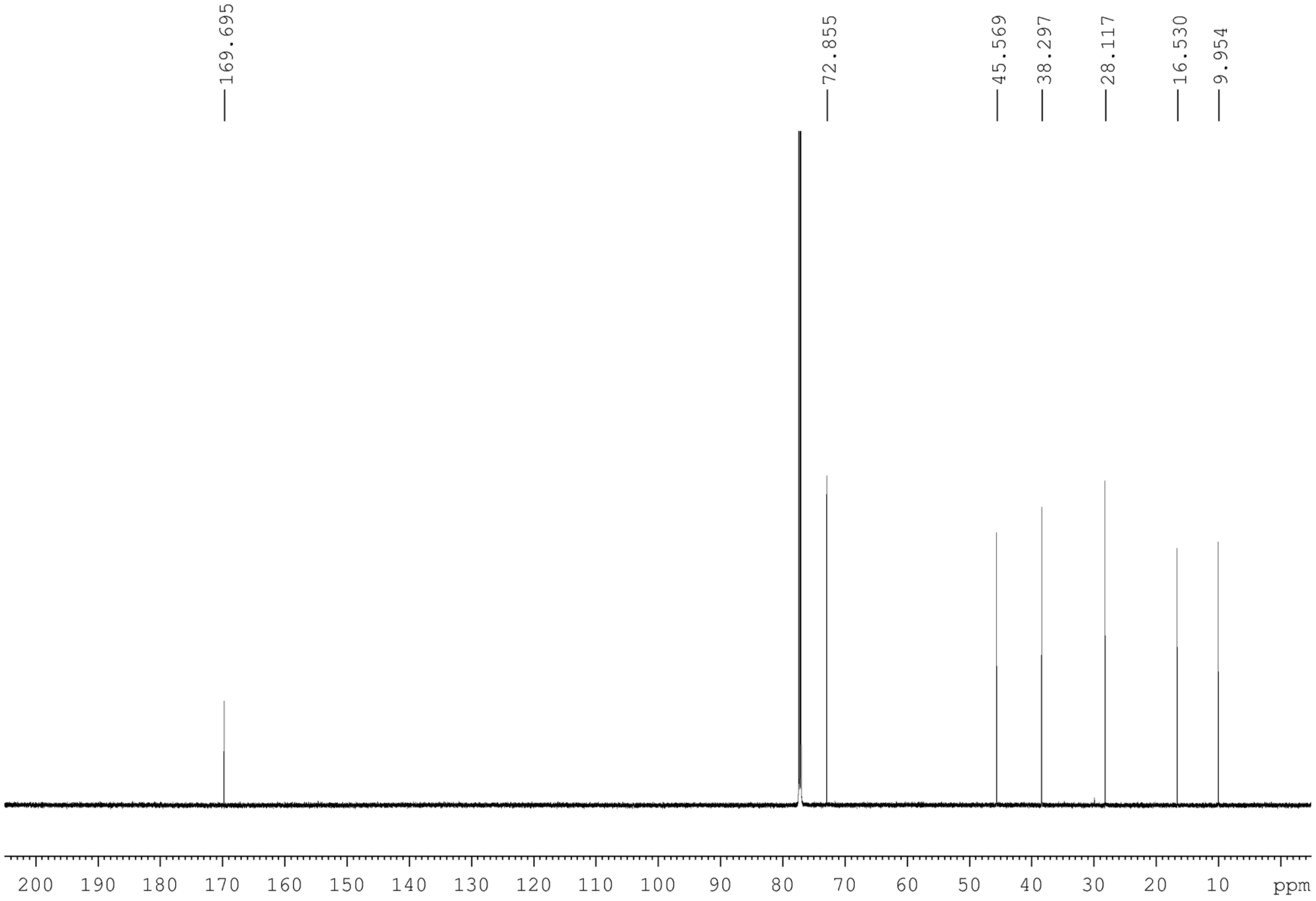

N-(5-ethoxy-2-hydroxypentyl)-2-(thiazol-4-yl)acetamide (1zu-rac)

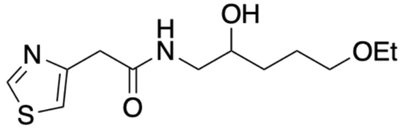

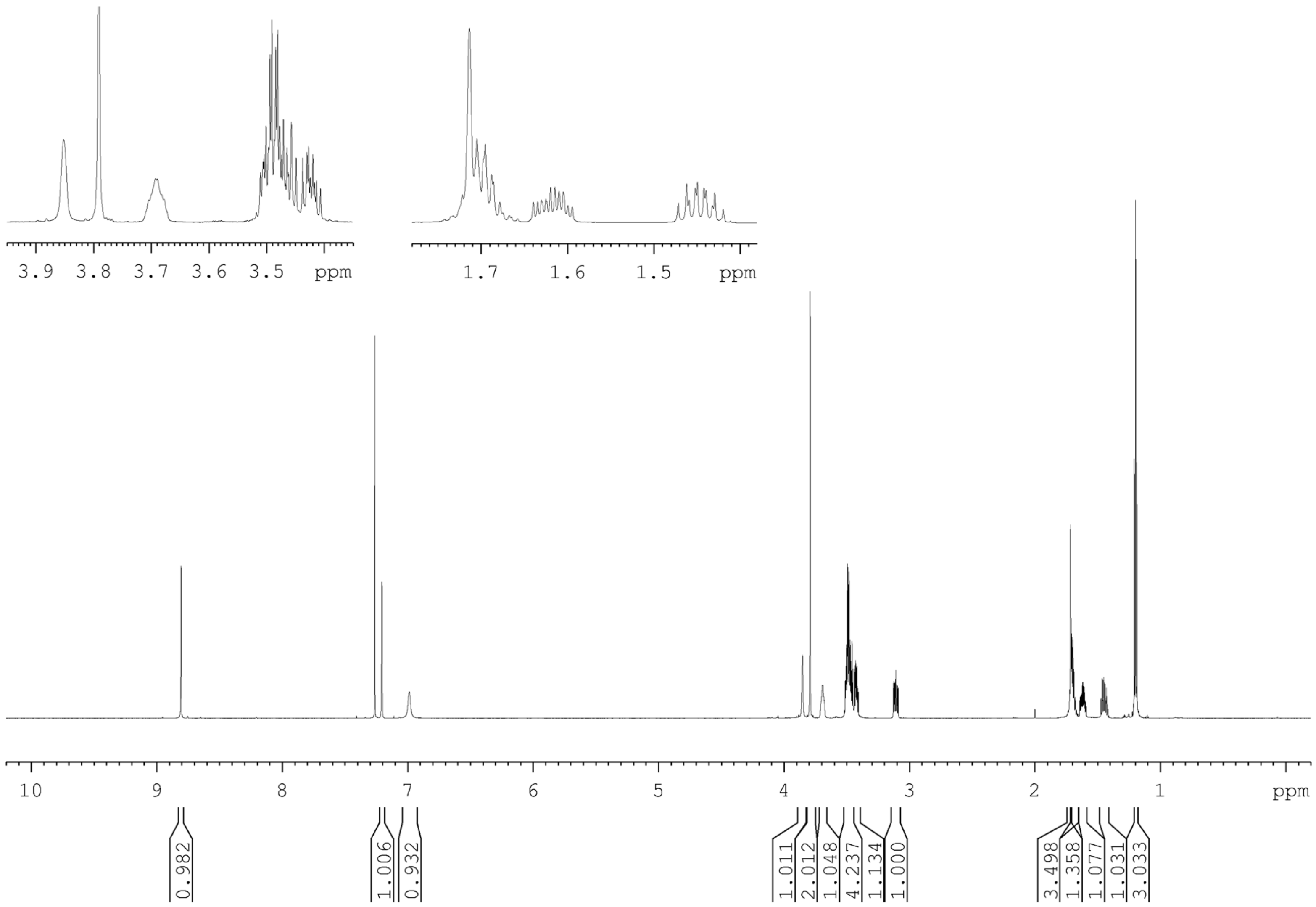

N-(5-ethoxy-2-hydroxypentyl)-2-(thiazol-4-yl)acetamide (1zu-rac)

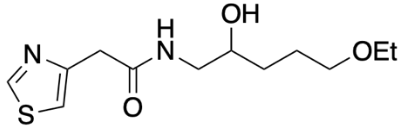

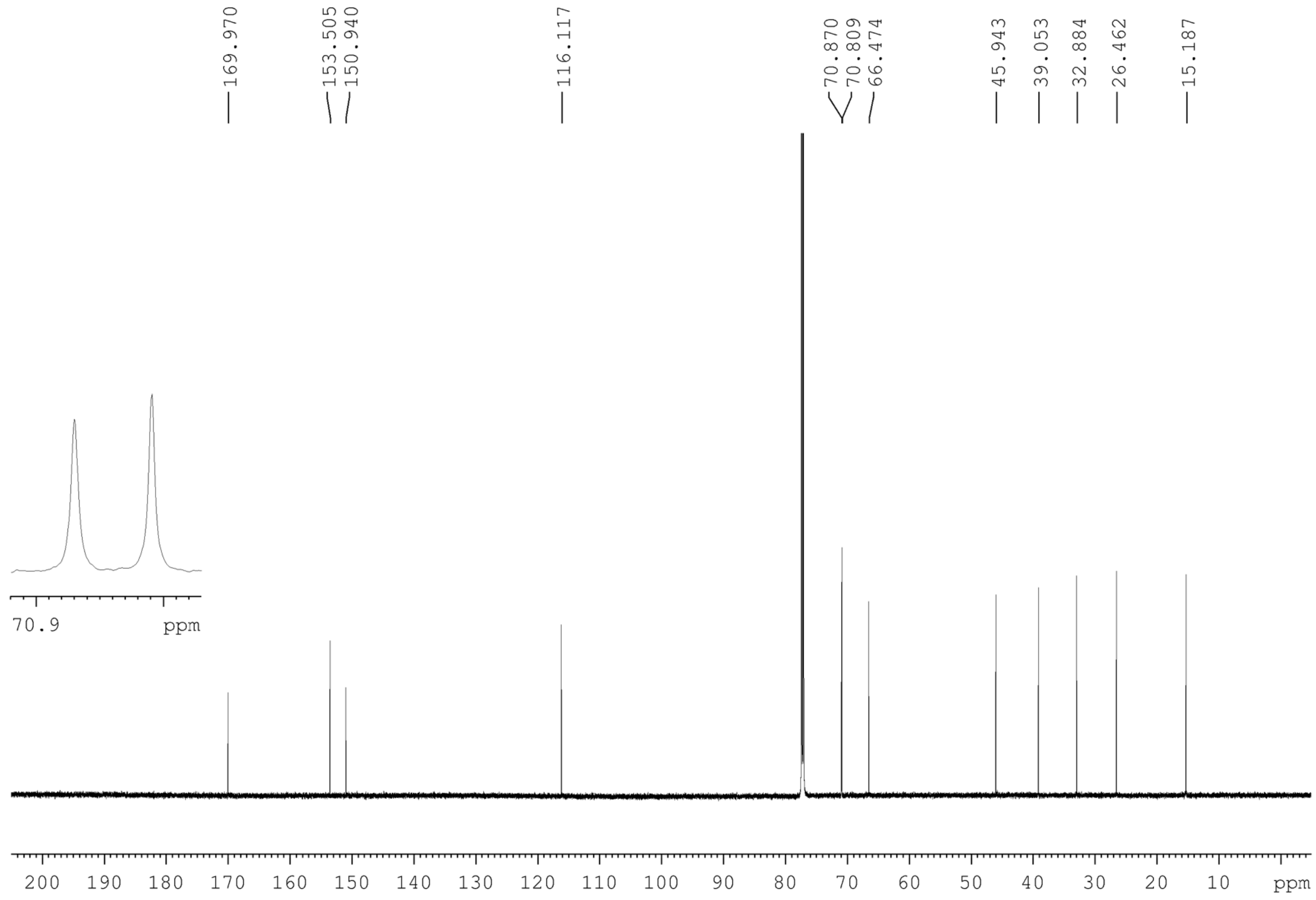

(*S*)-*N*-(5-ethoxy-2-hydroxypentyl)-2-(thiazol-4-yl)acetamide (1zu)

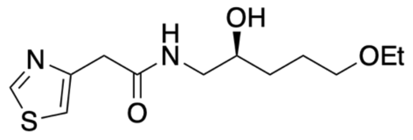

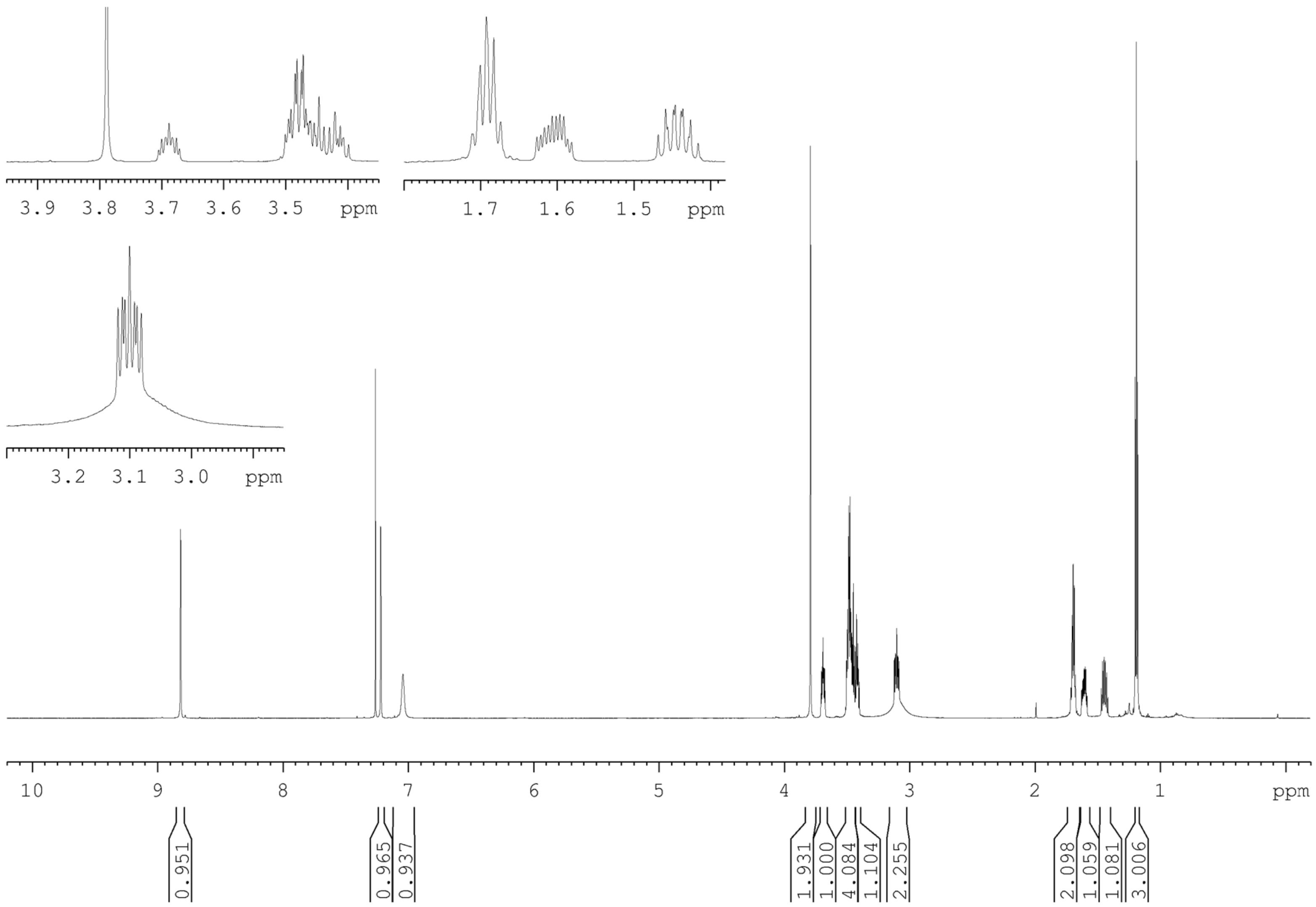

(*S*)-*N*-(5-ethoxy-2-hydroxypentyl)-2-(thiazol-4-yl)acetamide (1zu)

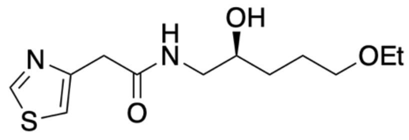

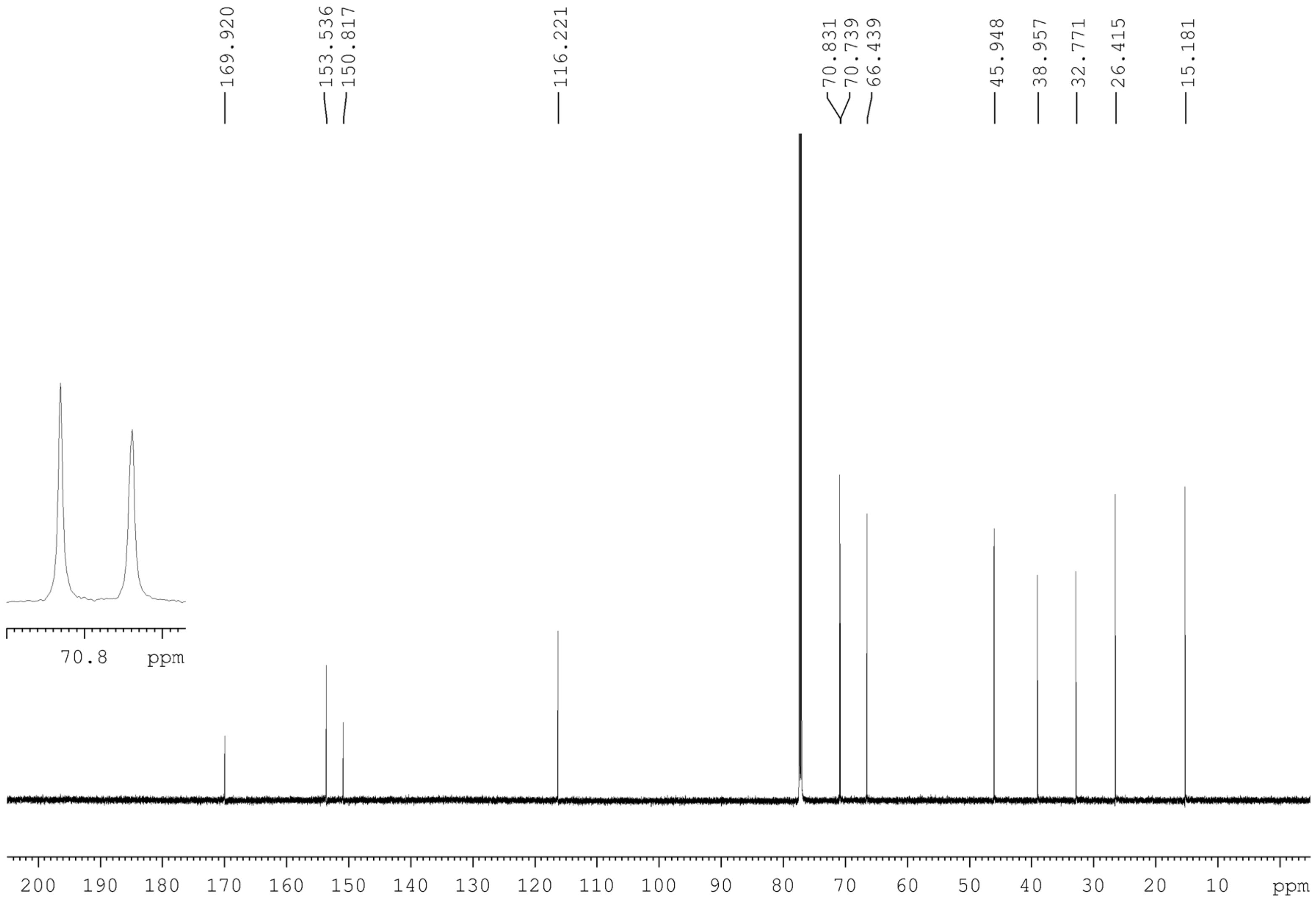

N-(2-hydroxy-3-phenylpropyl)morpholine-4-carboxamide (1zv-rac)

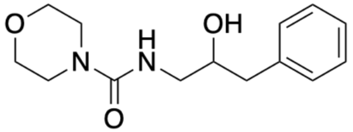

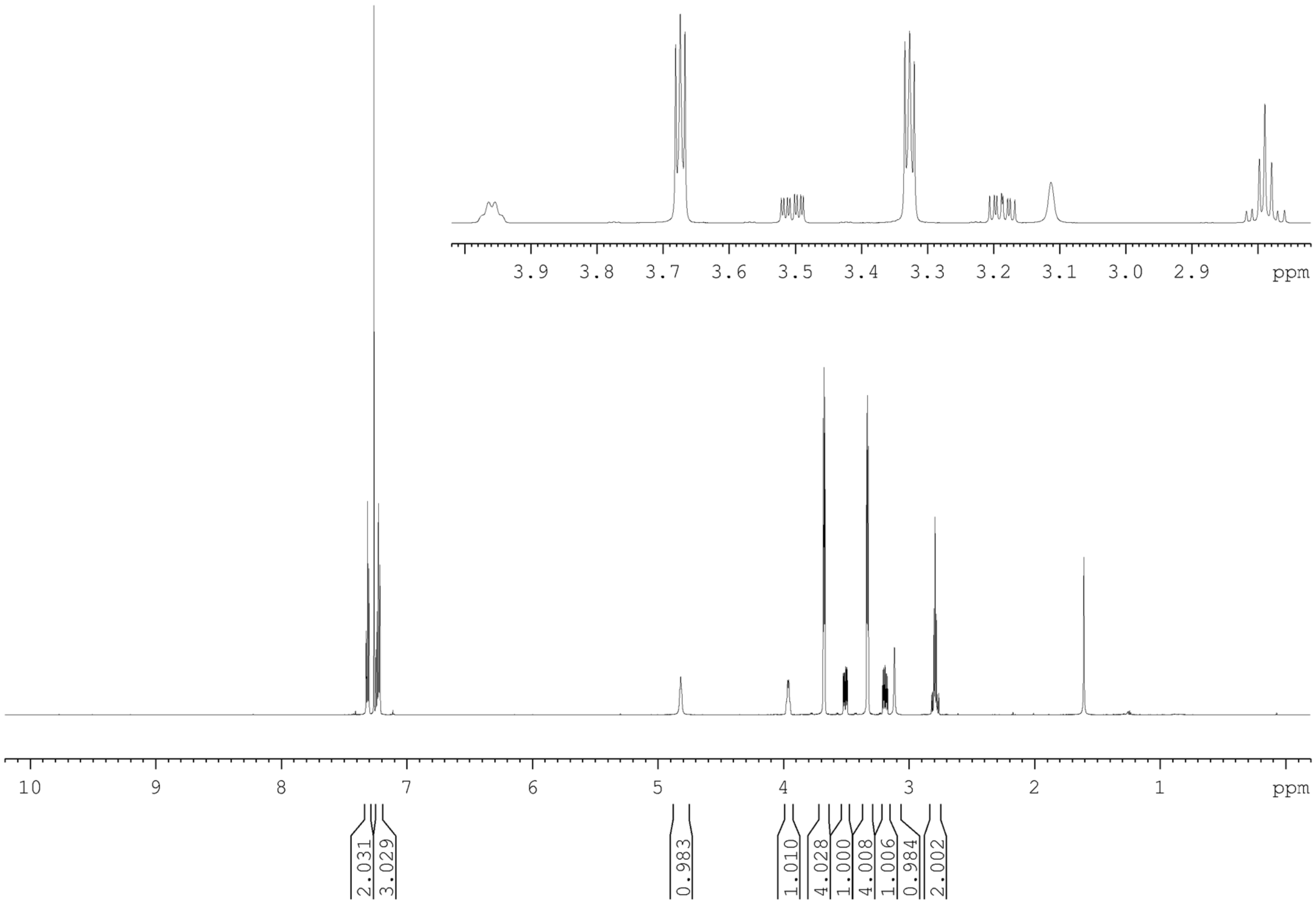

N-(2-hydroxy-3-phenylpropyl)morpholine-4-carboxamide (1zv-rac)

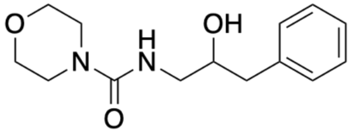

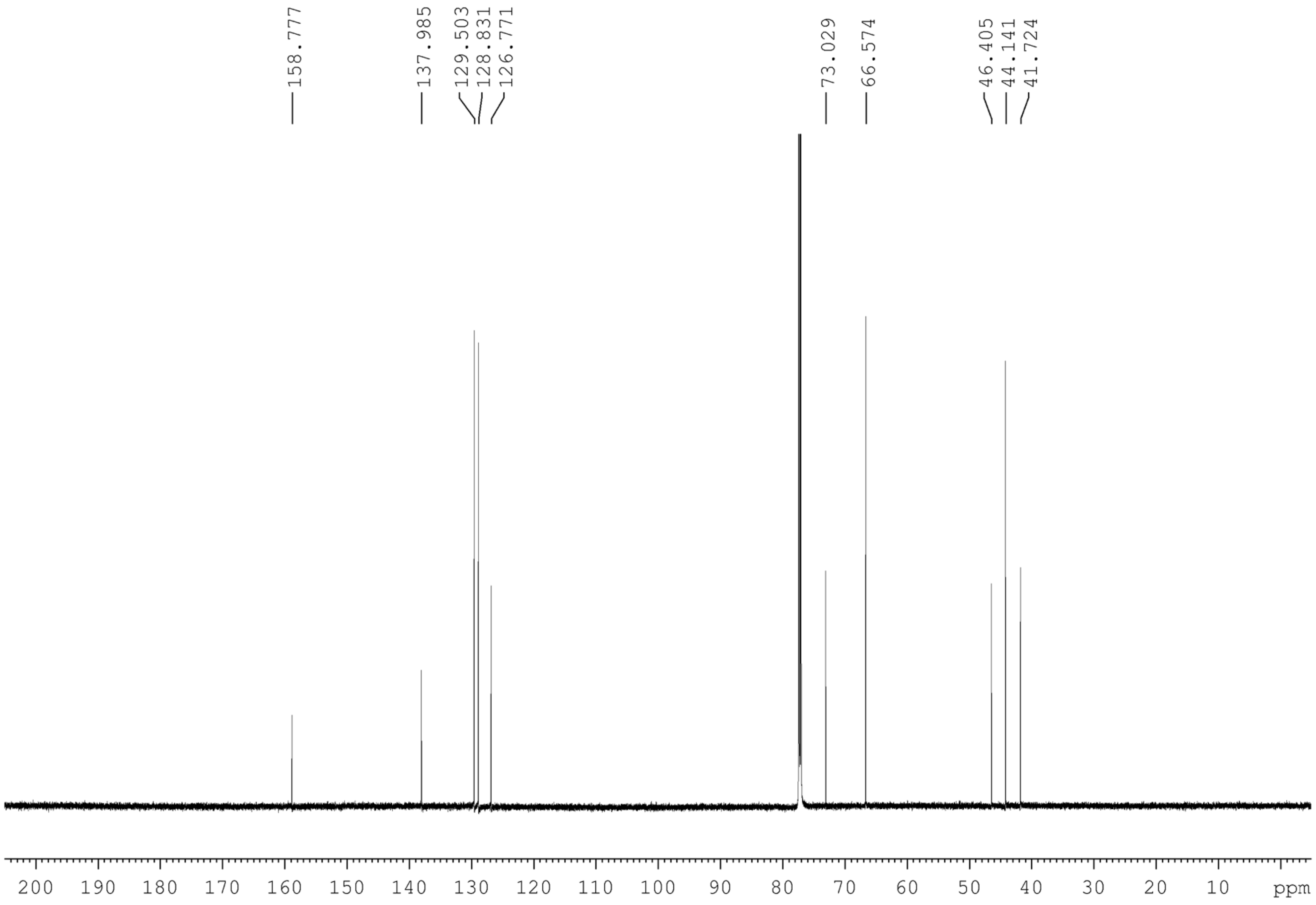

(*S*)-*N*-(2-hydroxy-3-phenylpropyl)morpholine-4-carboxamide (1zv)

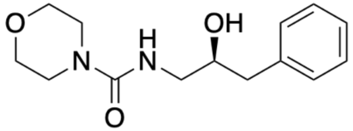

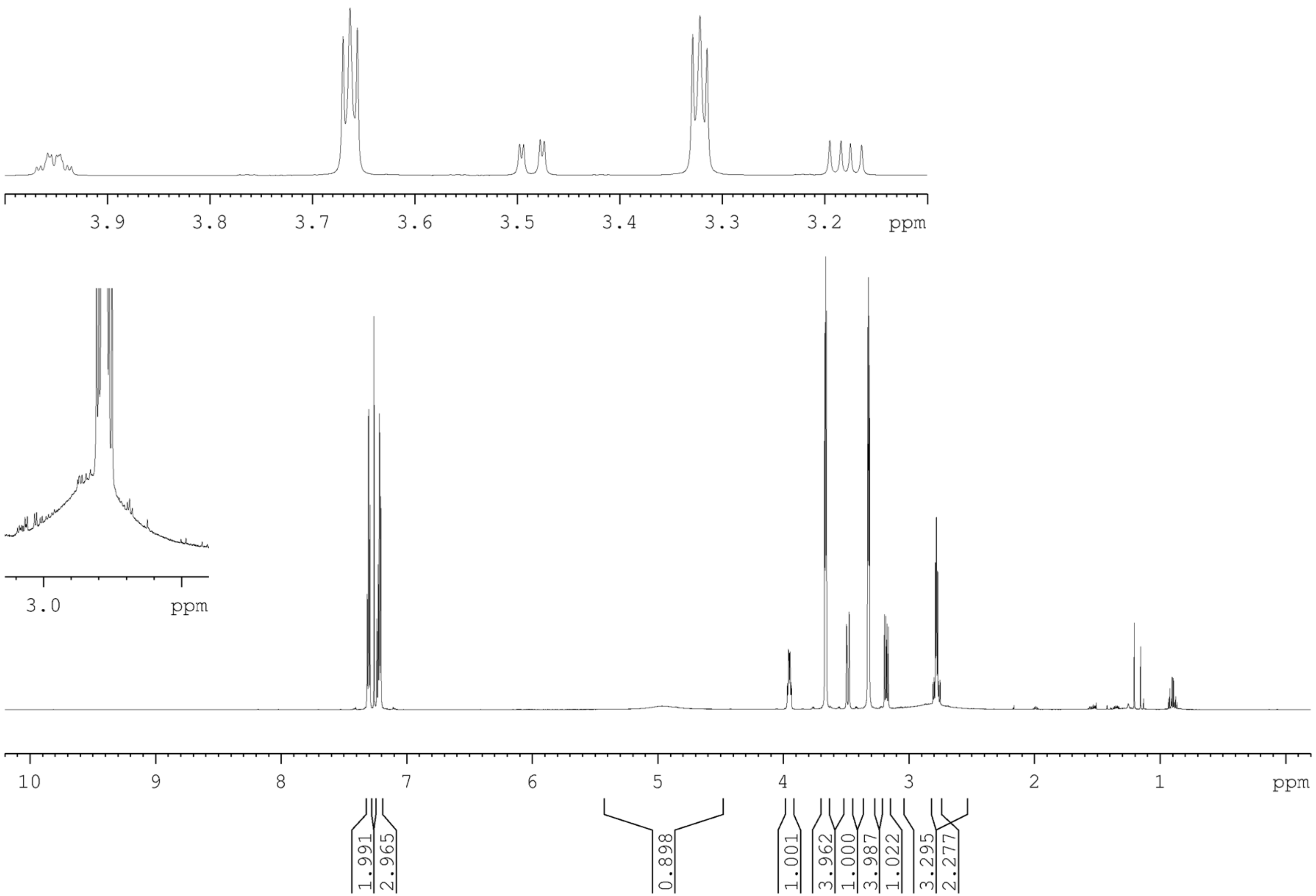

(*S*)-*N*-(2-hydroxy-3-phenylpropyl)morpholine-4-carboxamide (1zv)

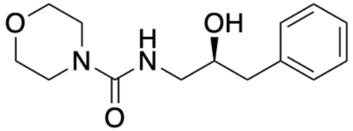

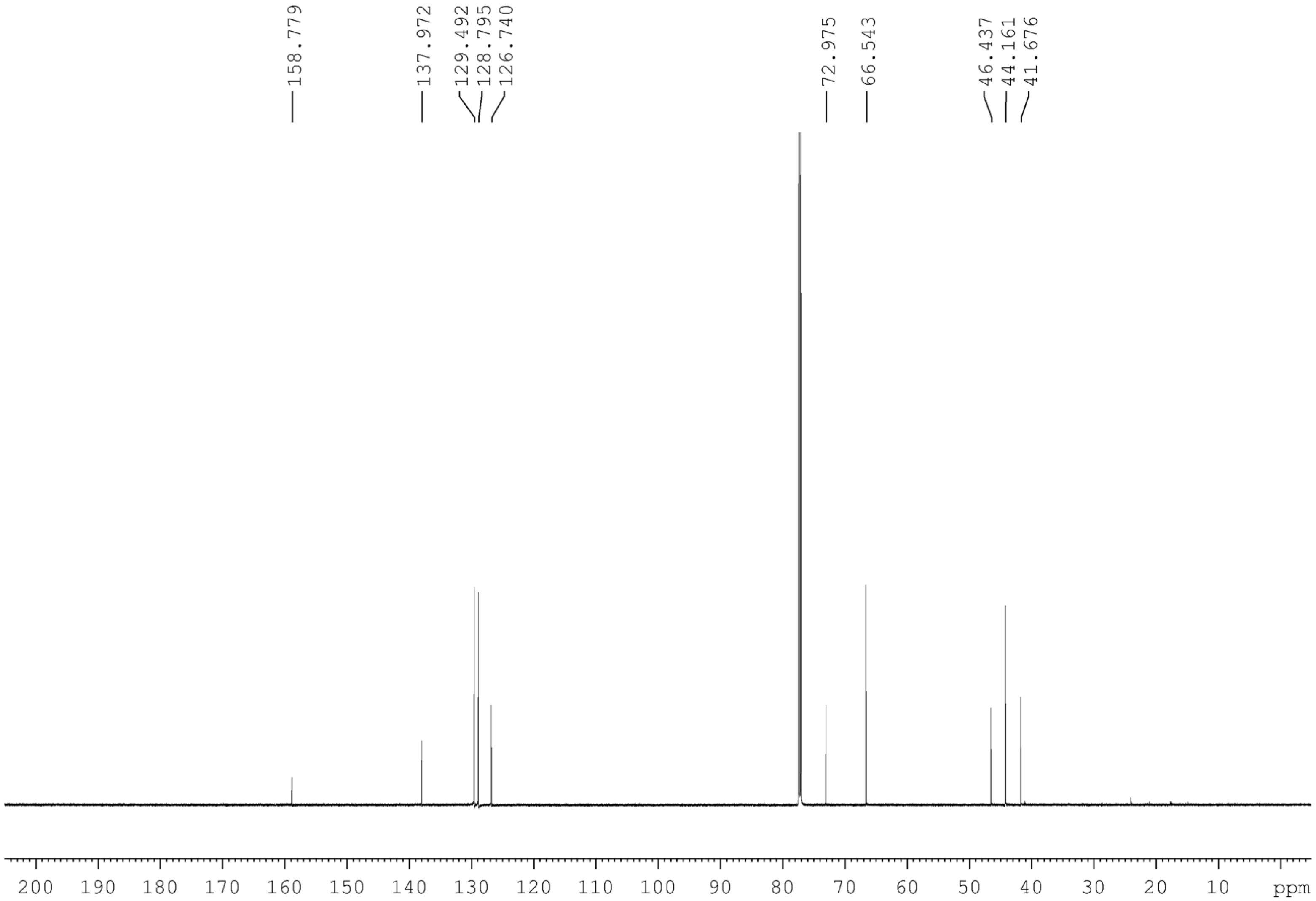

tert-butyl ((2S)-1-((2-hydroxybutyl)amino)-1-oxo-3-phenylpropan-2-yl)carbamate (2a-rac)

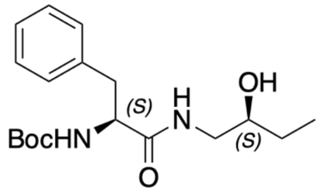

d1

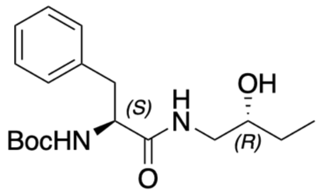

d2

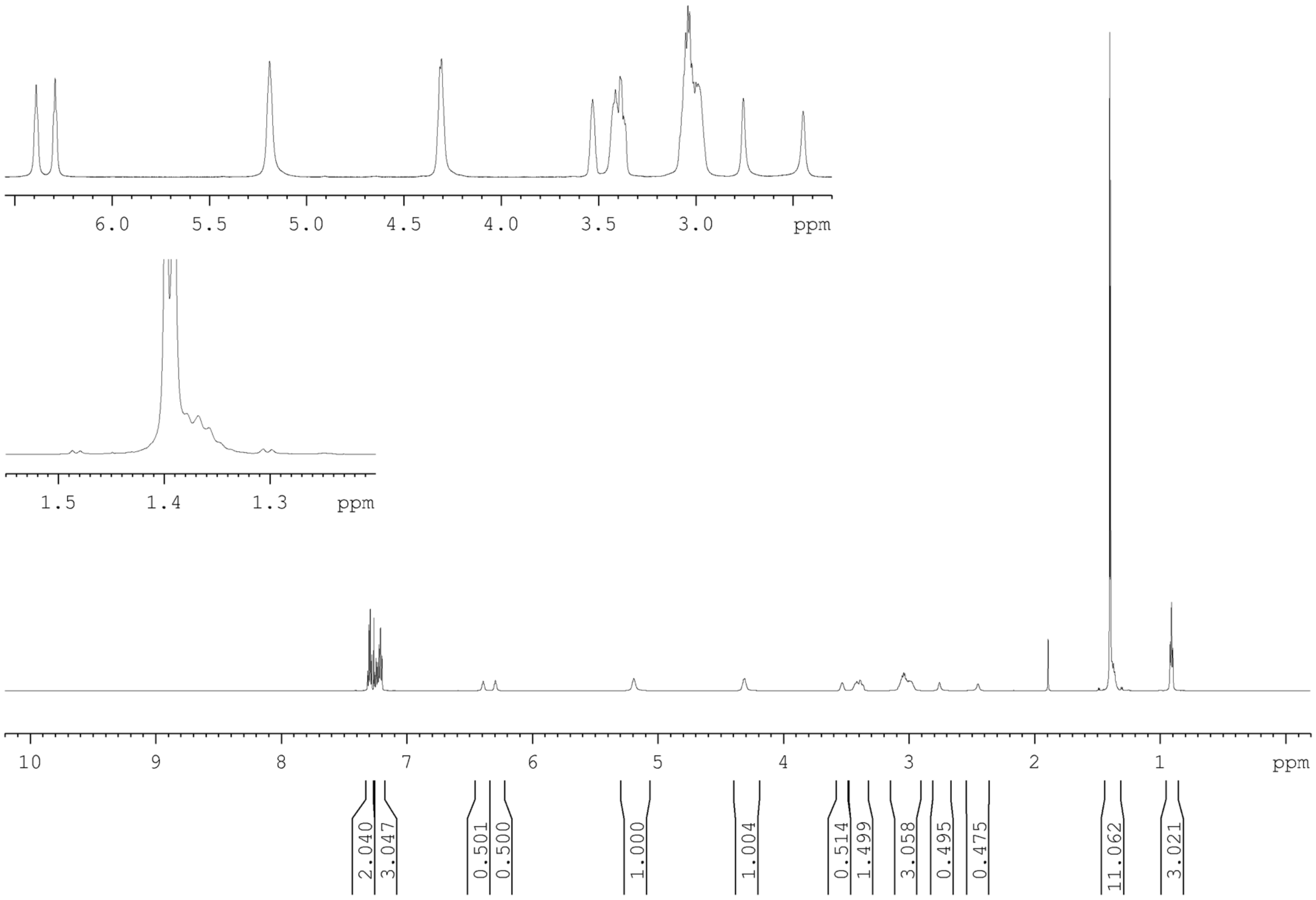

tert-butyl ((2S)-1-((2-hydroxybutyl)amino)-1-oxo-3-phenylpropan-2-yl)carbamate (2a-rac)

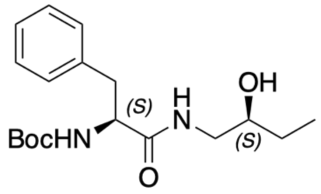

d1

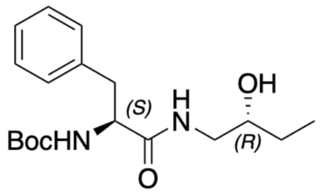

d2

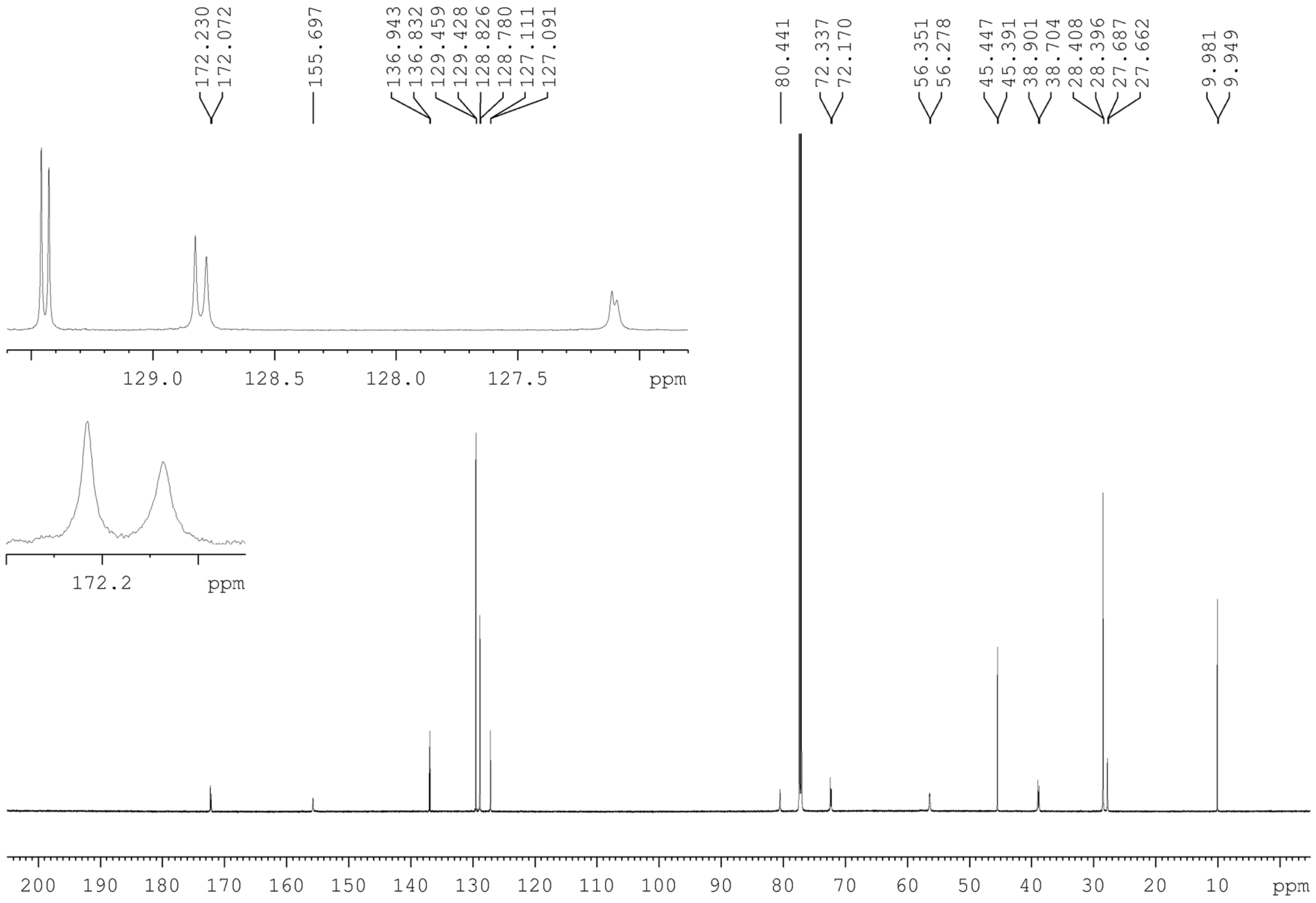

tert-butyl ((_S_)-1-(((_S_)-2-hydroxybutyl)amino)-1-oxo-3-phenylpropan-2-yl)carbamate (2a)

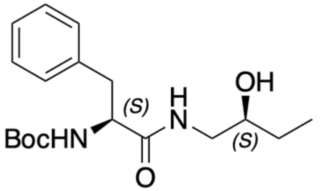

d1 (major)

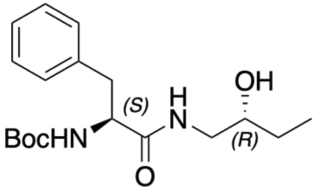

d2 (minor)

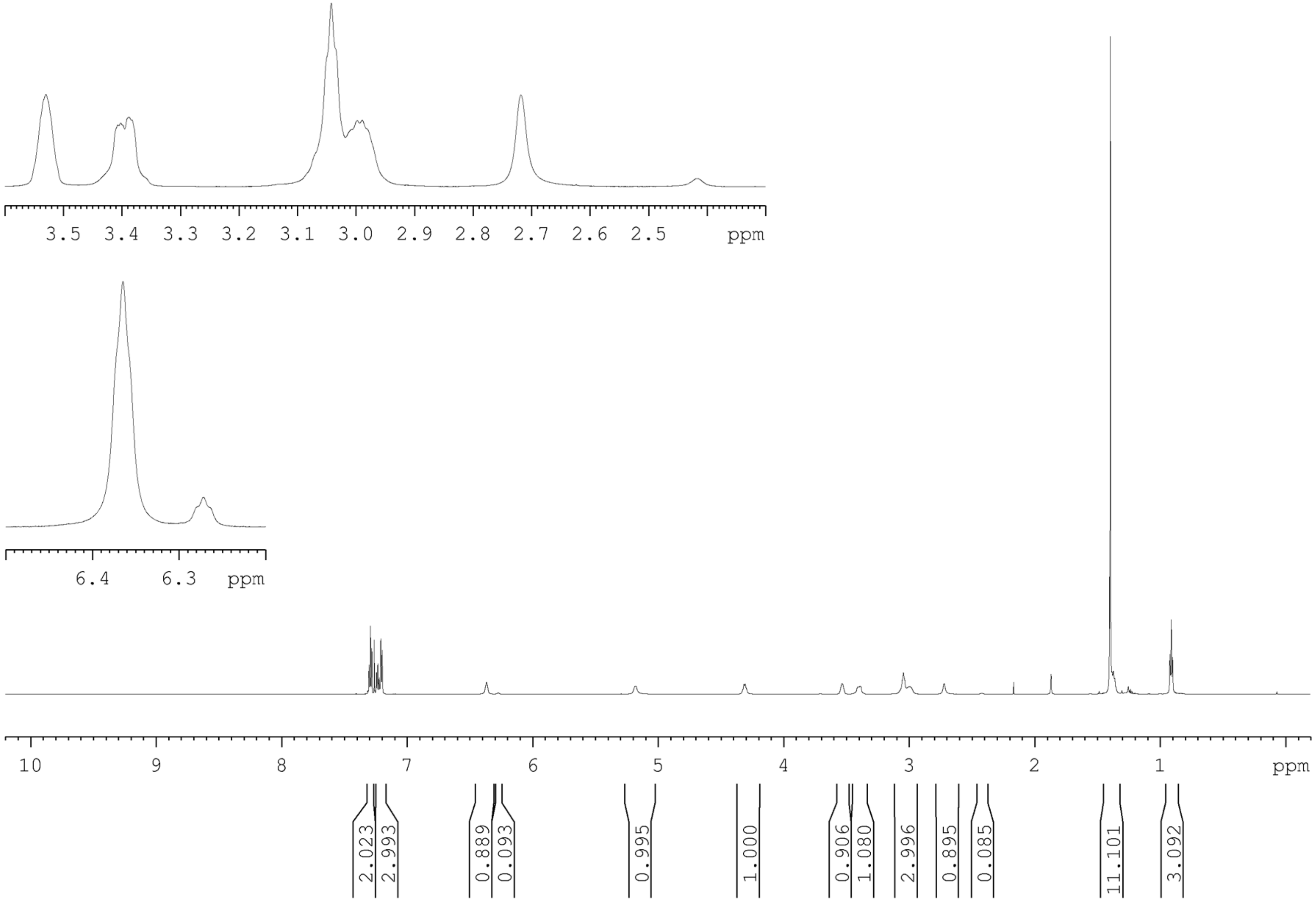

tert-butyl ((_S_)-1-(((_S_)-2-hydroxybutyl)amino)-1-oxo-3-phenylpropan-2-yl)carbamate (2a)

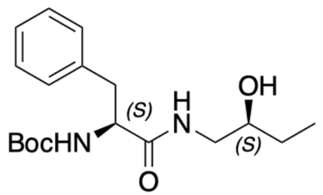

d1 (major)

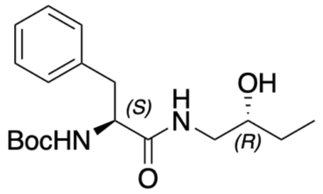

d2 (minor)

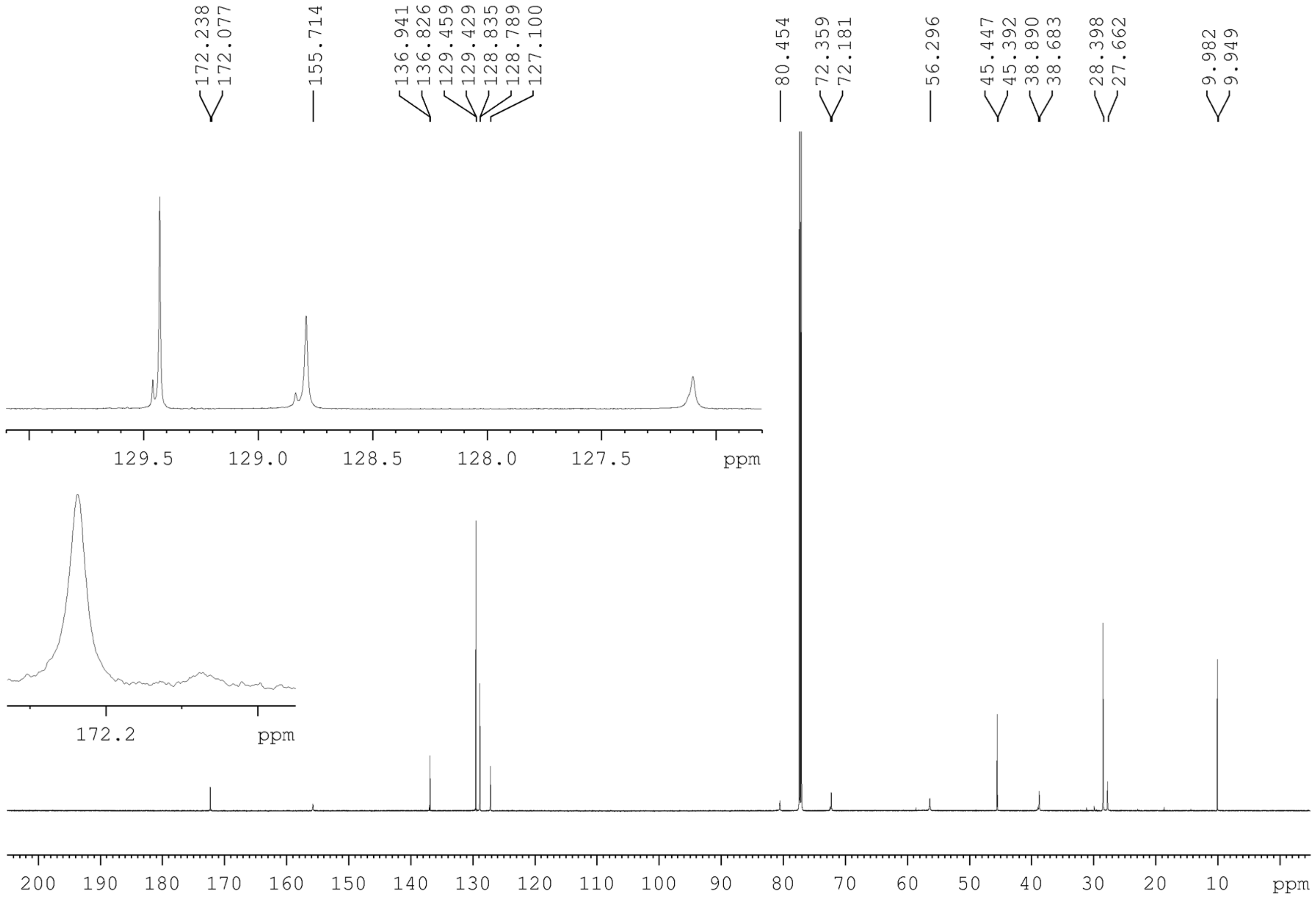

tert-butyl ((2_R_)-1-((2-hydroxybutyl)amino)-1-oxo-3-phenylpropan-2-yl)carbamate (2b-rac)

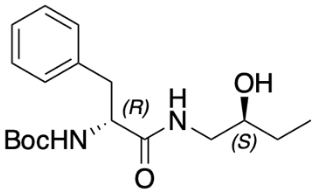

d1

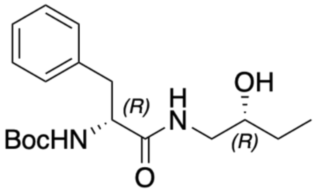

d2

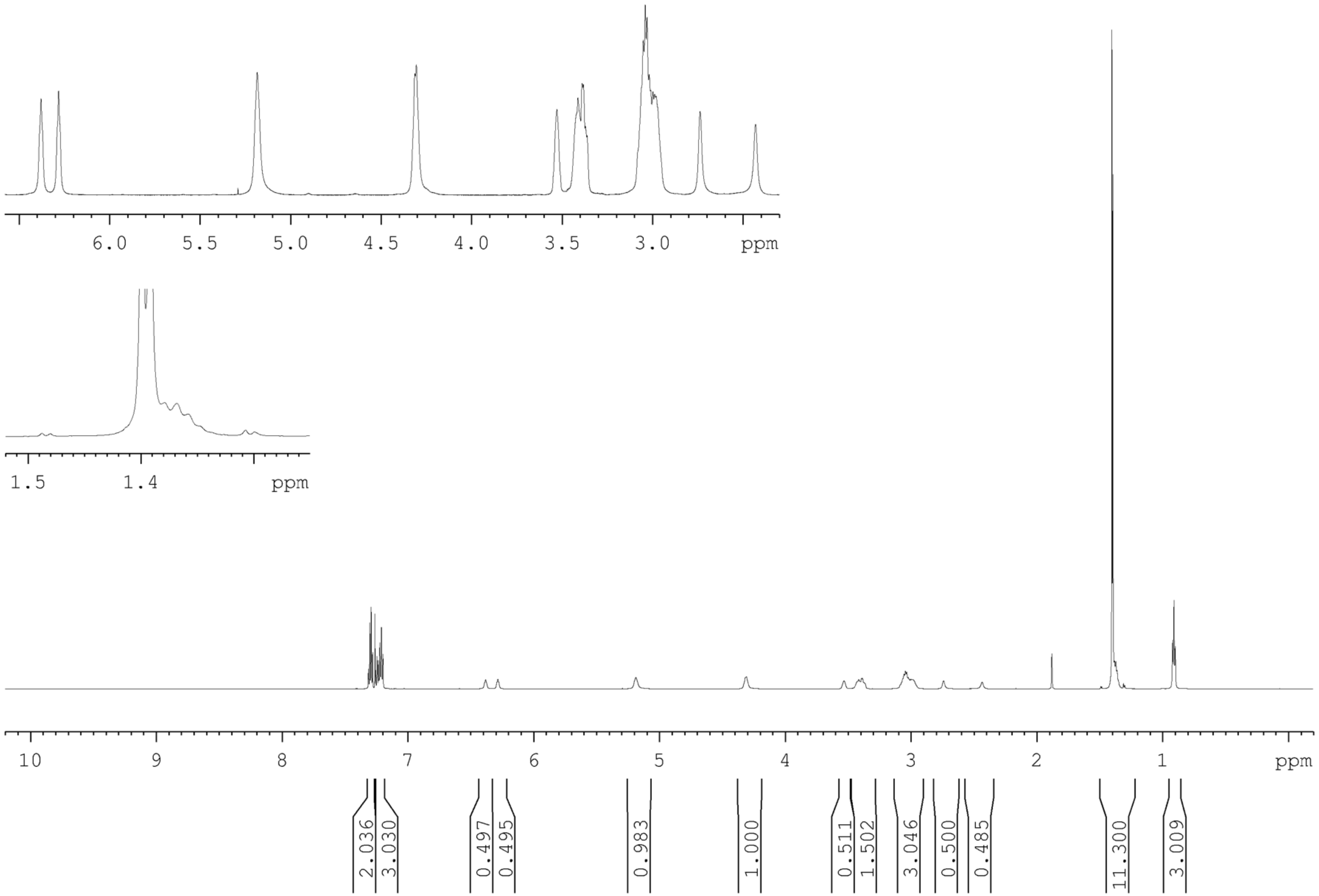

***tert*-butyl ((2*R*)-1-((2-hydroxybutyl)amino)-1-oxo-3-phenylpropan-2-yl)carbamate (2b-rac)**

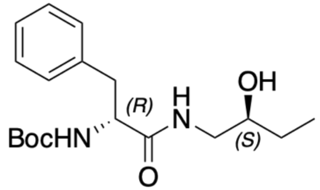

d1

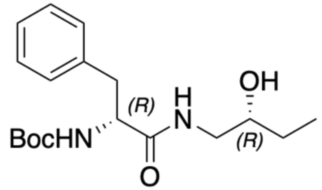

d2

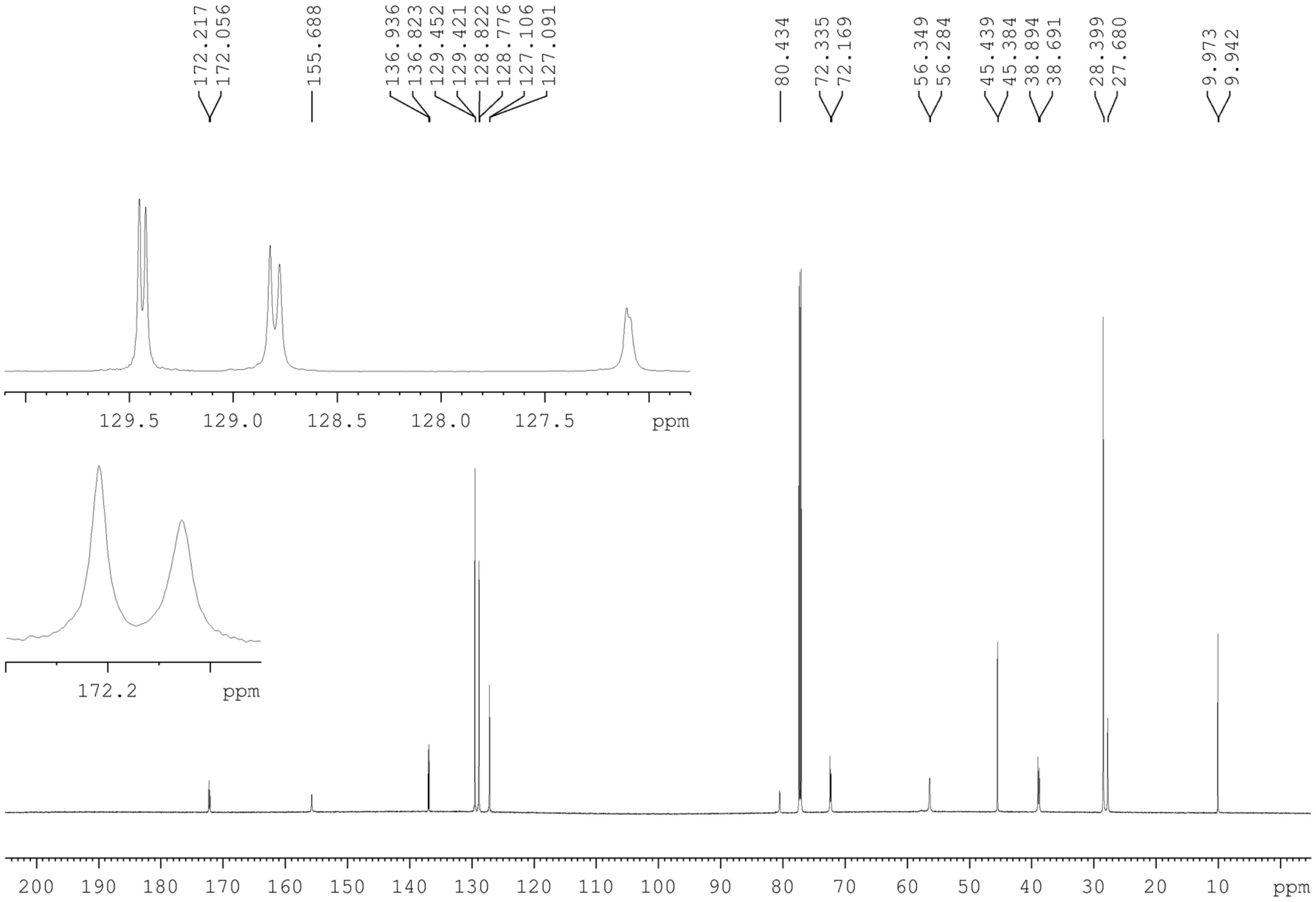

***tert*-butyl ((*R*)-1-(((*S*)-2-hydroxybutyl)amino)-1-oxo-3-phenylpropan-2-yl)carbamate (2b)**

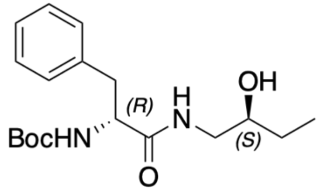

d1 (major)

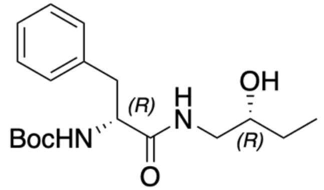

d2 (minor)

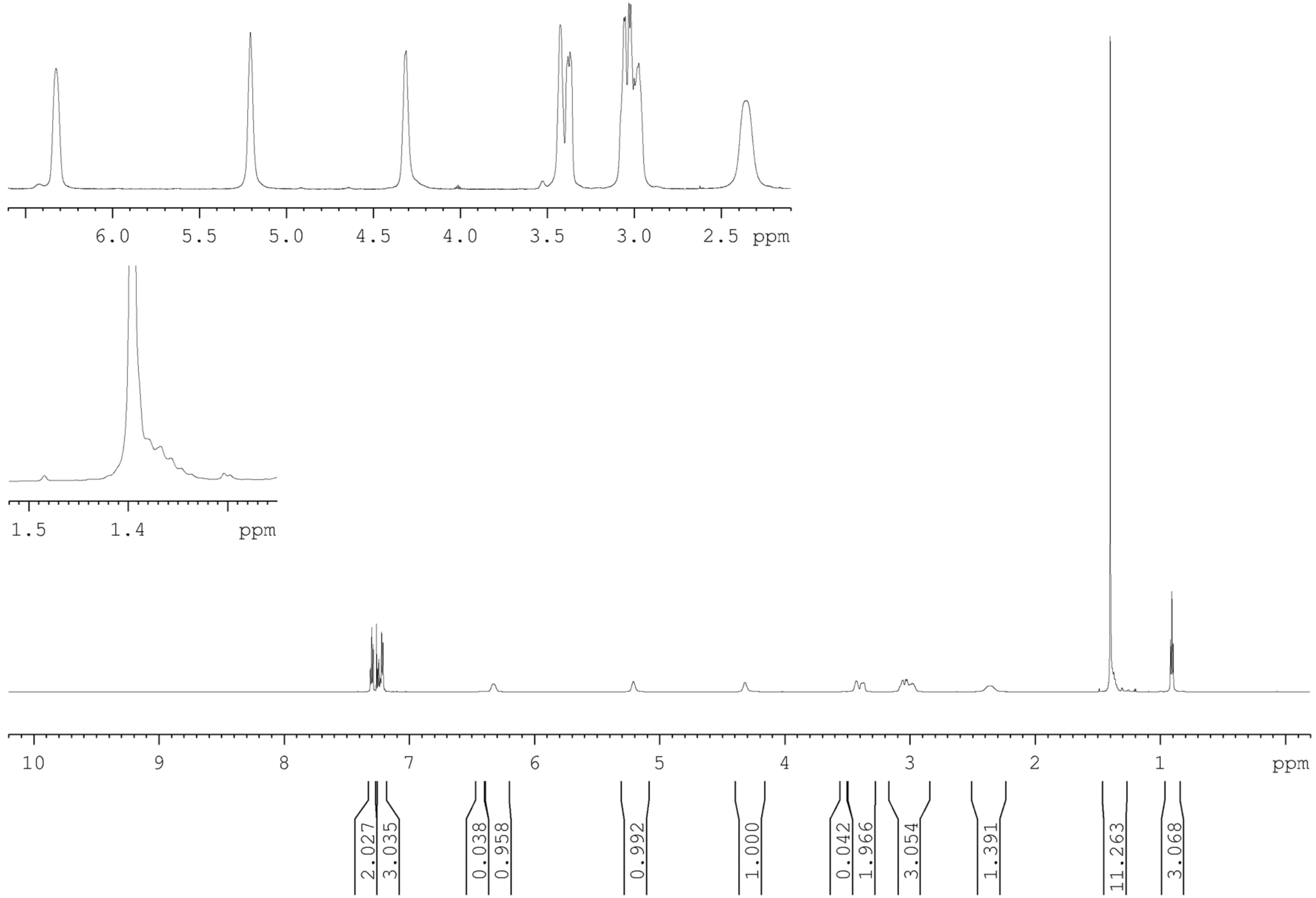

tert-butyl ((_R_)-1-(((_S_)-2-hydroxybutyl)amino)-1-oxo-3-phenylpropan-2-yl)carbamate (2b)

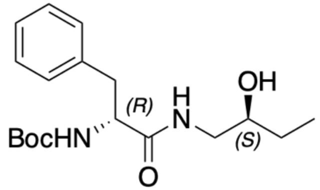

d1 (major)

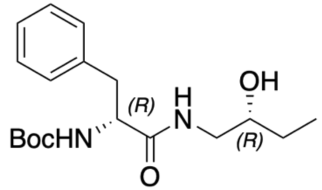

d2 (minor)

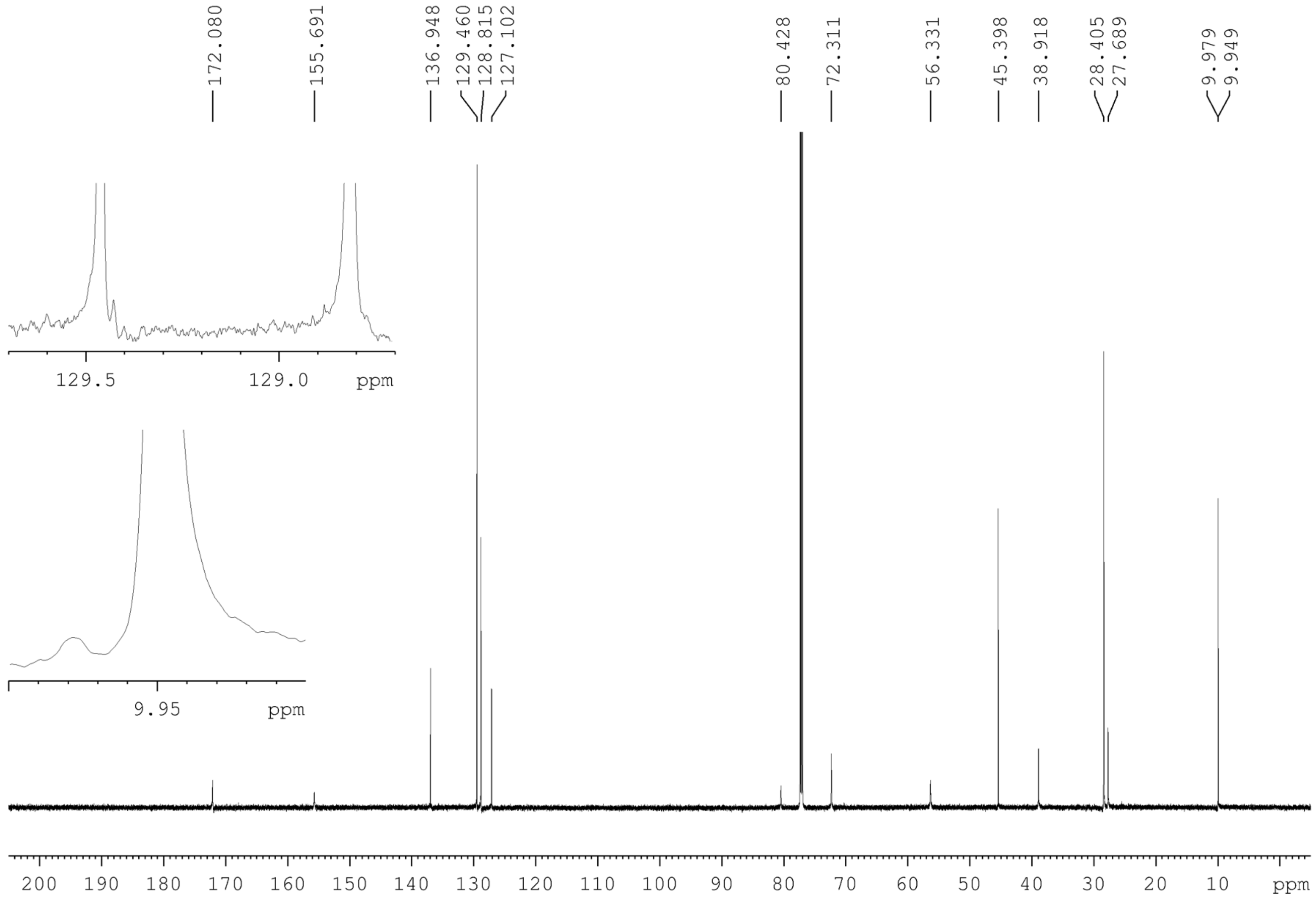

N-(2-hydroxybutyl)-2-(4-isobutylphenyl)propanamide (2c-rac)

In CDCl$_3$

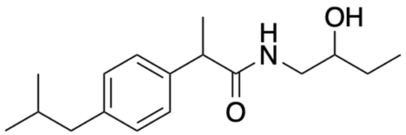

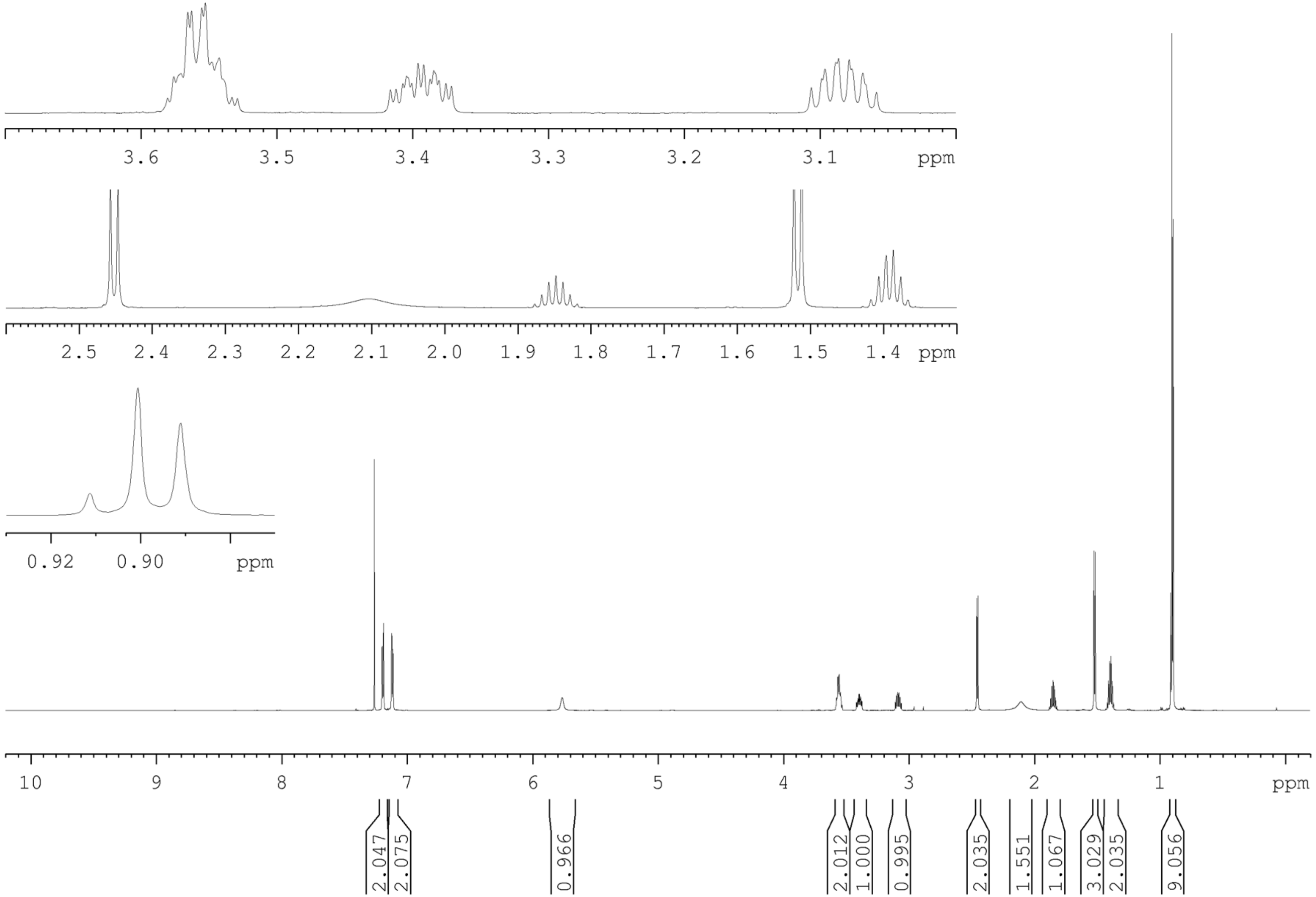

<u>*Diastereomer 1*</u>

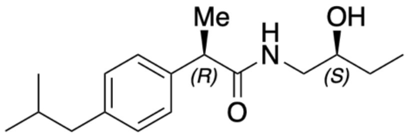

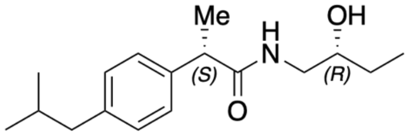

<u>*Diastereomer 2*</u>

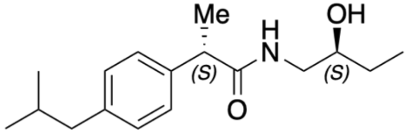

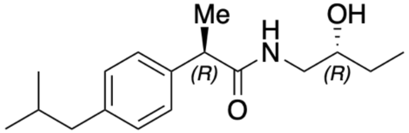

N-(2-hydroxybutyl)-2-(4-isobutylphenyl)propanamide (2c-rac)

In $CDCl_3$

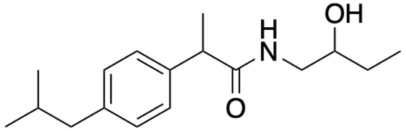

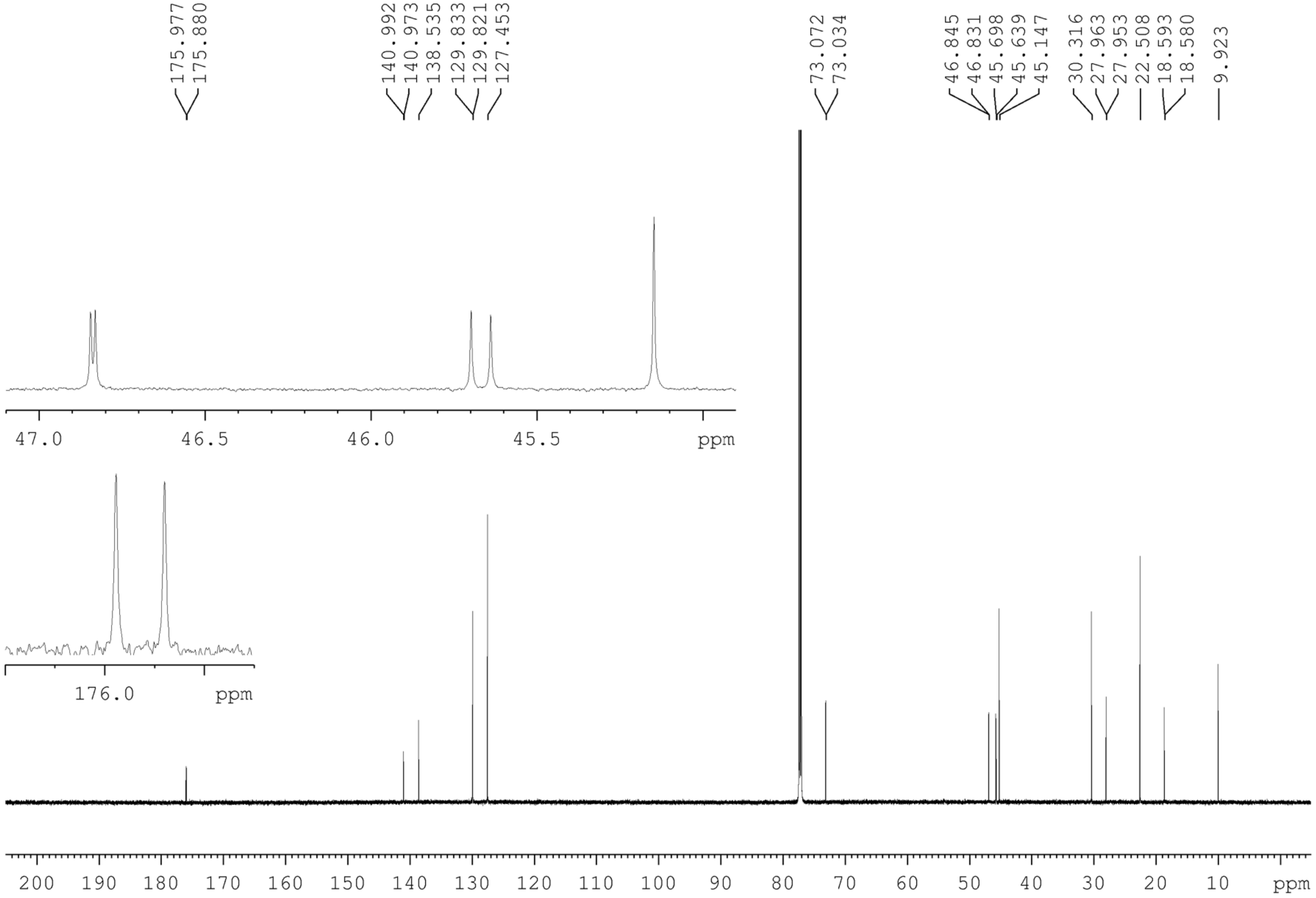

Diastereomer 1

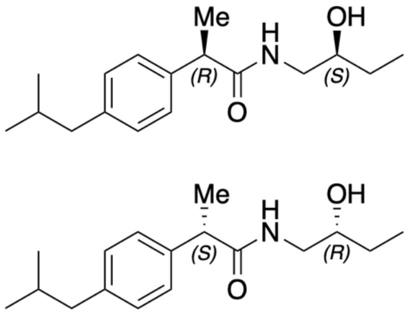

Diastereomer 2

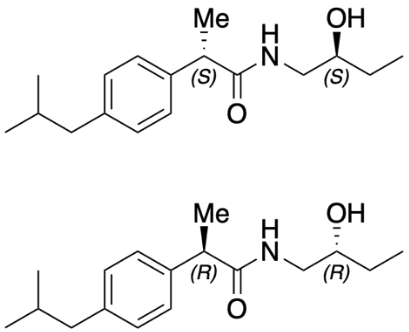

N-(2-hydroxybutyl)-2-(4-isobutylphenyl)propanamide (2c-rac)

In $(CD_3)_2SO$, RT

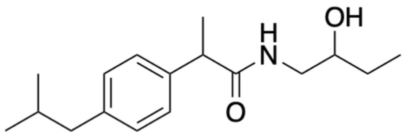

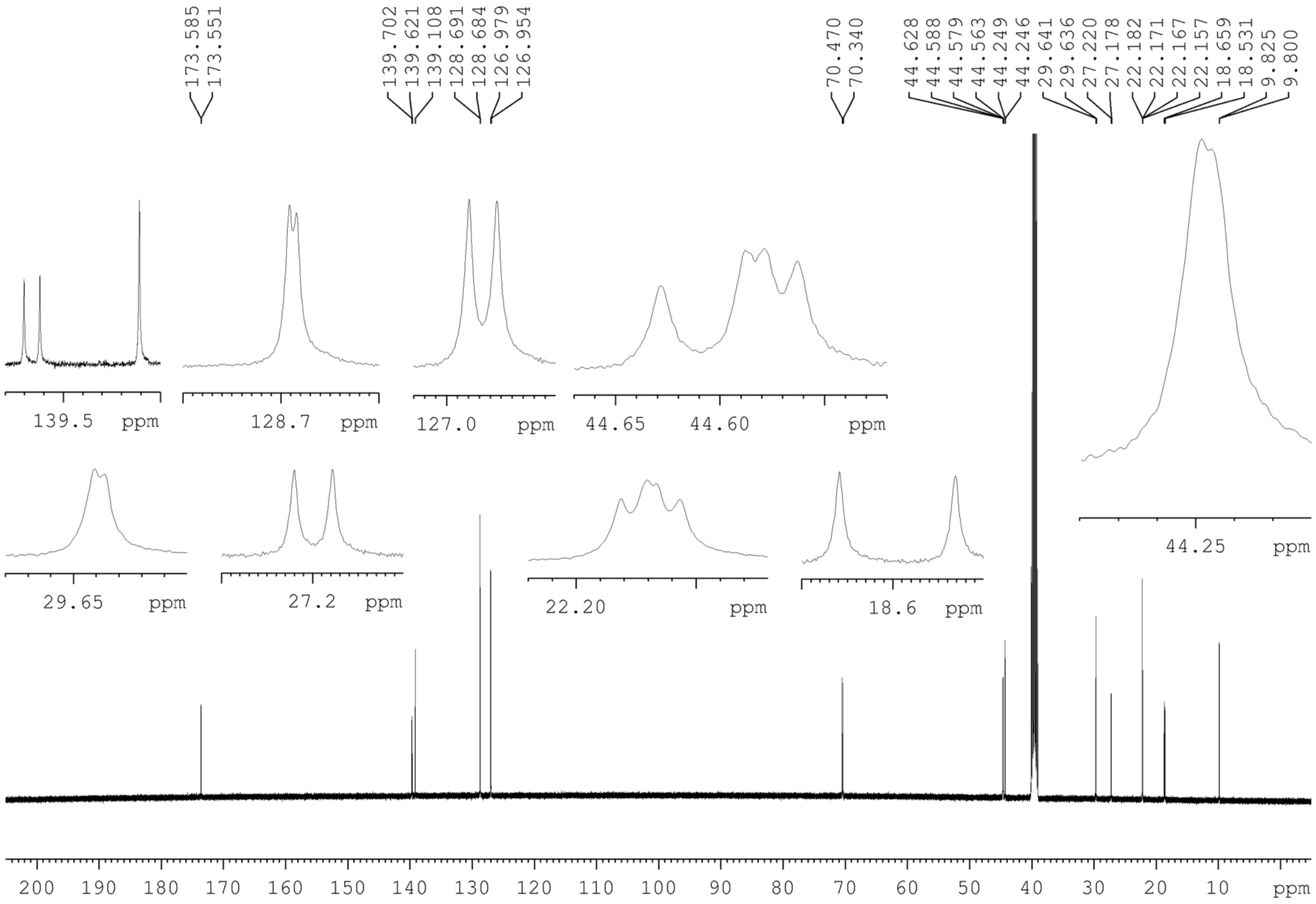

Diastereomer 1

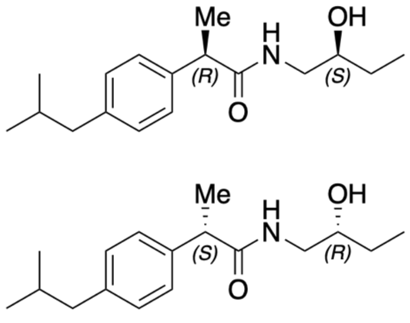

Diastereomer 2

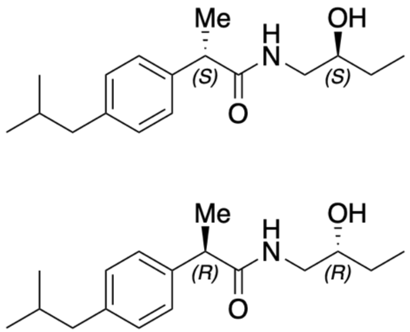

N-(2-hydroxybutyl)-2-(4-isobutylphenyl)propanamide (2c-rac)

In $(CD_3)_2SO$, RT

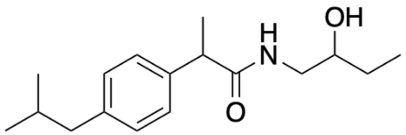

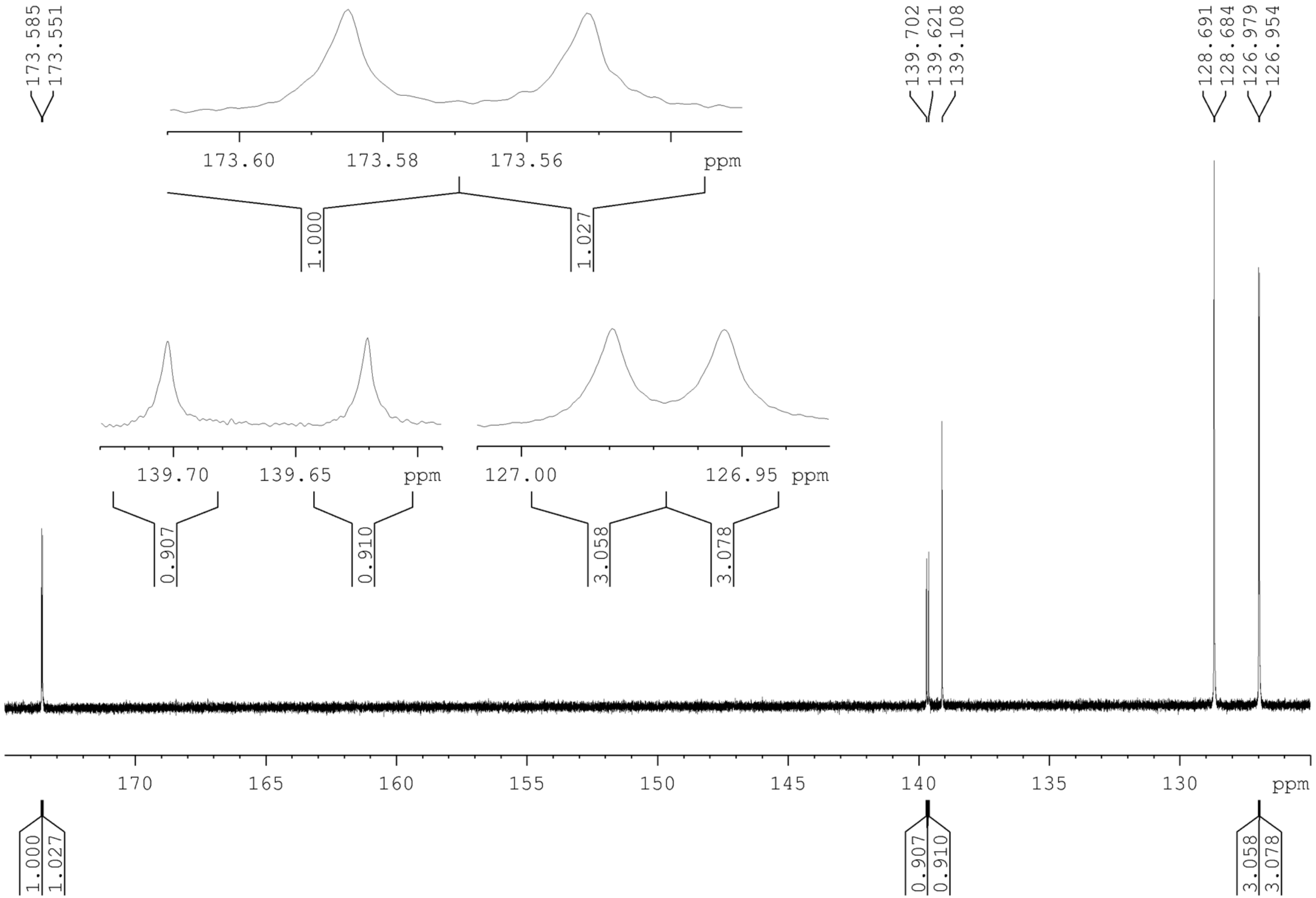

<u>*Diastereomer 1*</u>

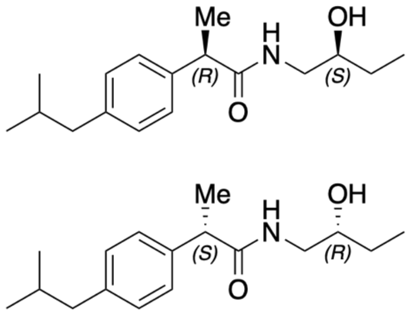

<u>*Diastereomer 2*</u>

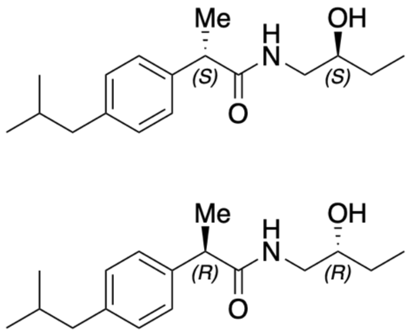

N-(2-hydroxybutyl)-2-(4-isobutylphenyl)propanamide (2c-rac)

In $(CD_3)_2SO$, 80 °C

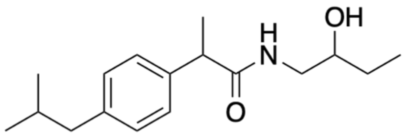

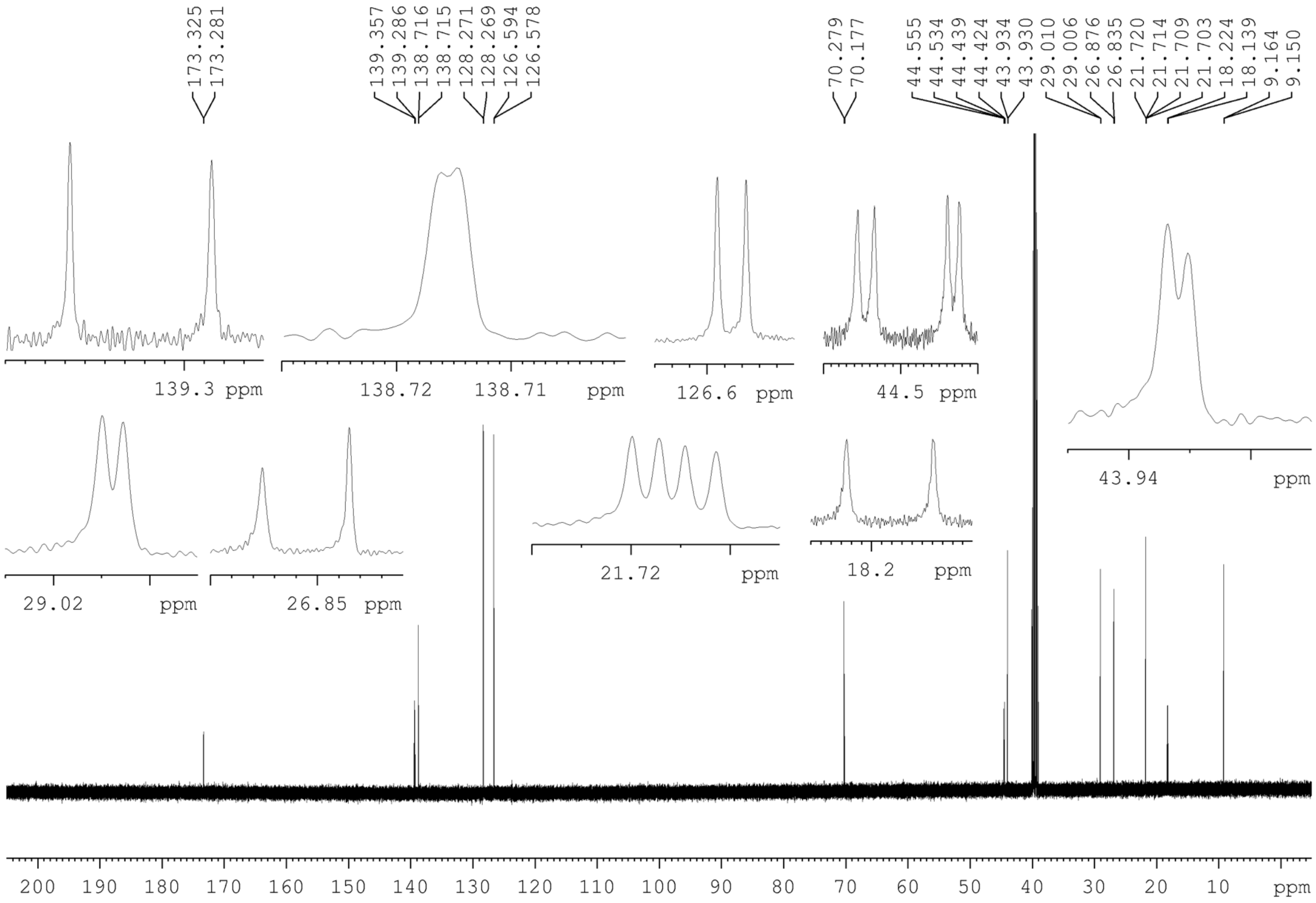

Diastereomer 1

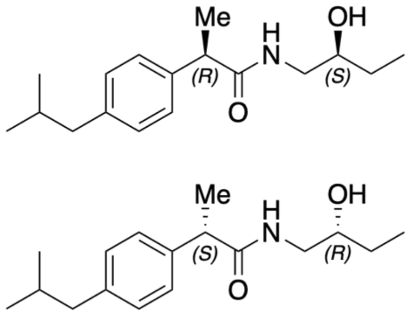

Diastereomer 2

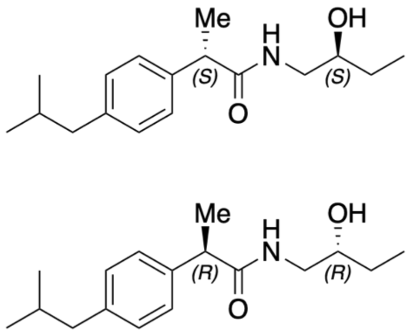

N-(2-hydroxybutyl)-2-(4-isobutylphenyl)propanamide (2c-rac)

In $(CD_3)_2SO$, 80 °C

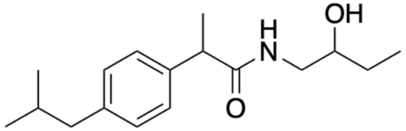

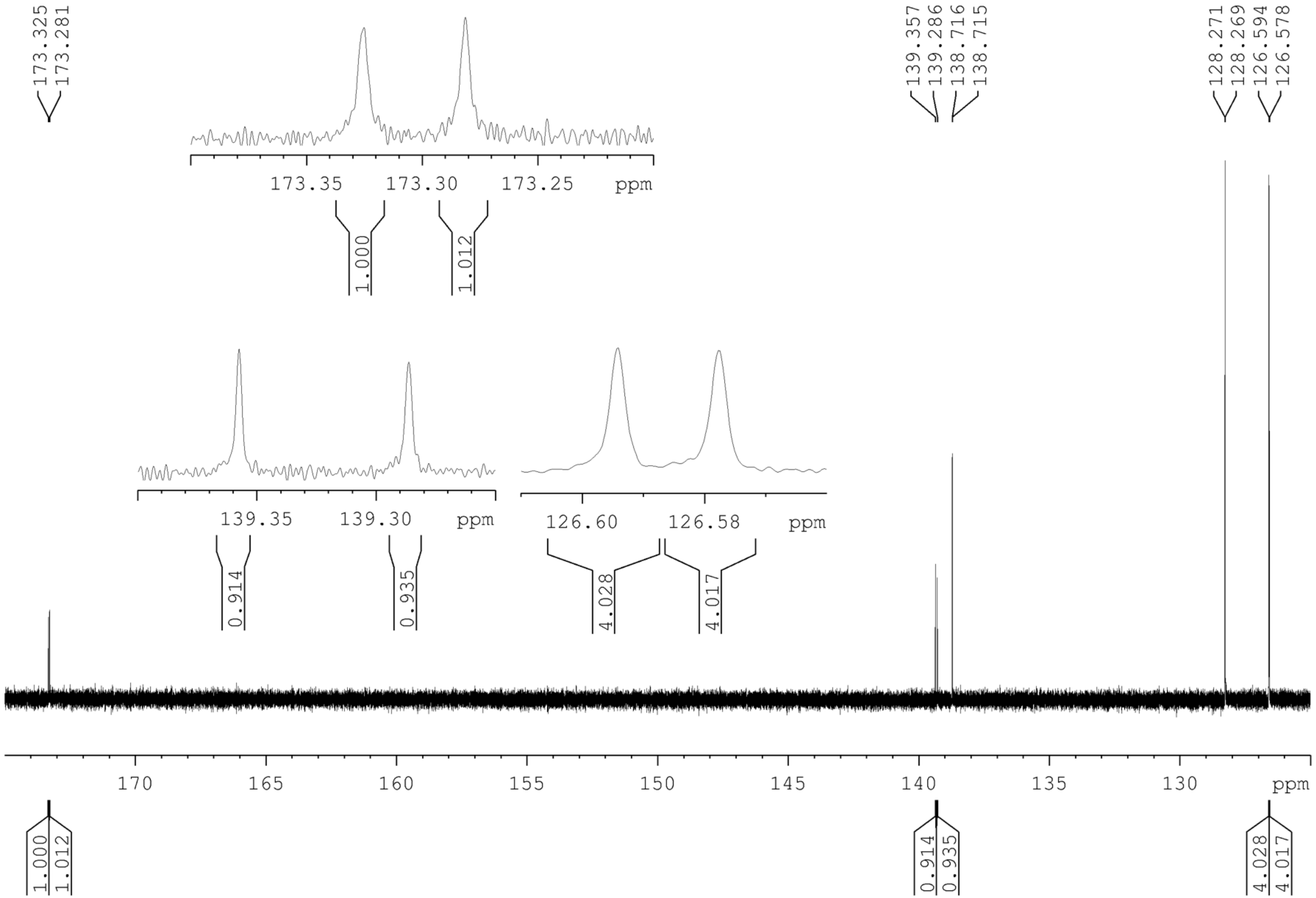

<u>*Diastereomer 1*</u>

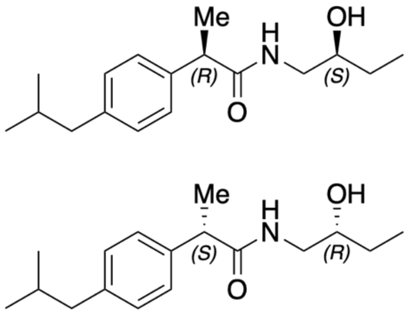

<u>*Diastereomer 2*</u>

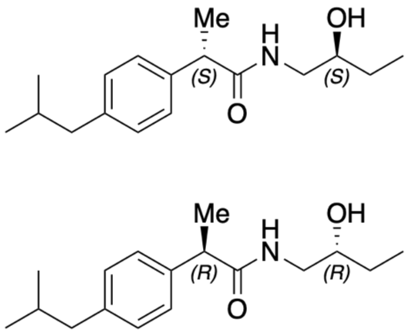

N-((*S*)-2-hydroxybutyl)-2-(4-isobutylphenyl)propanamide (2c)

In $CDCl_3$

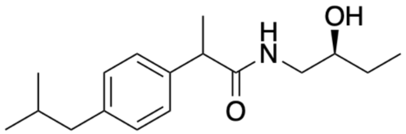

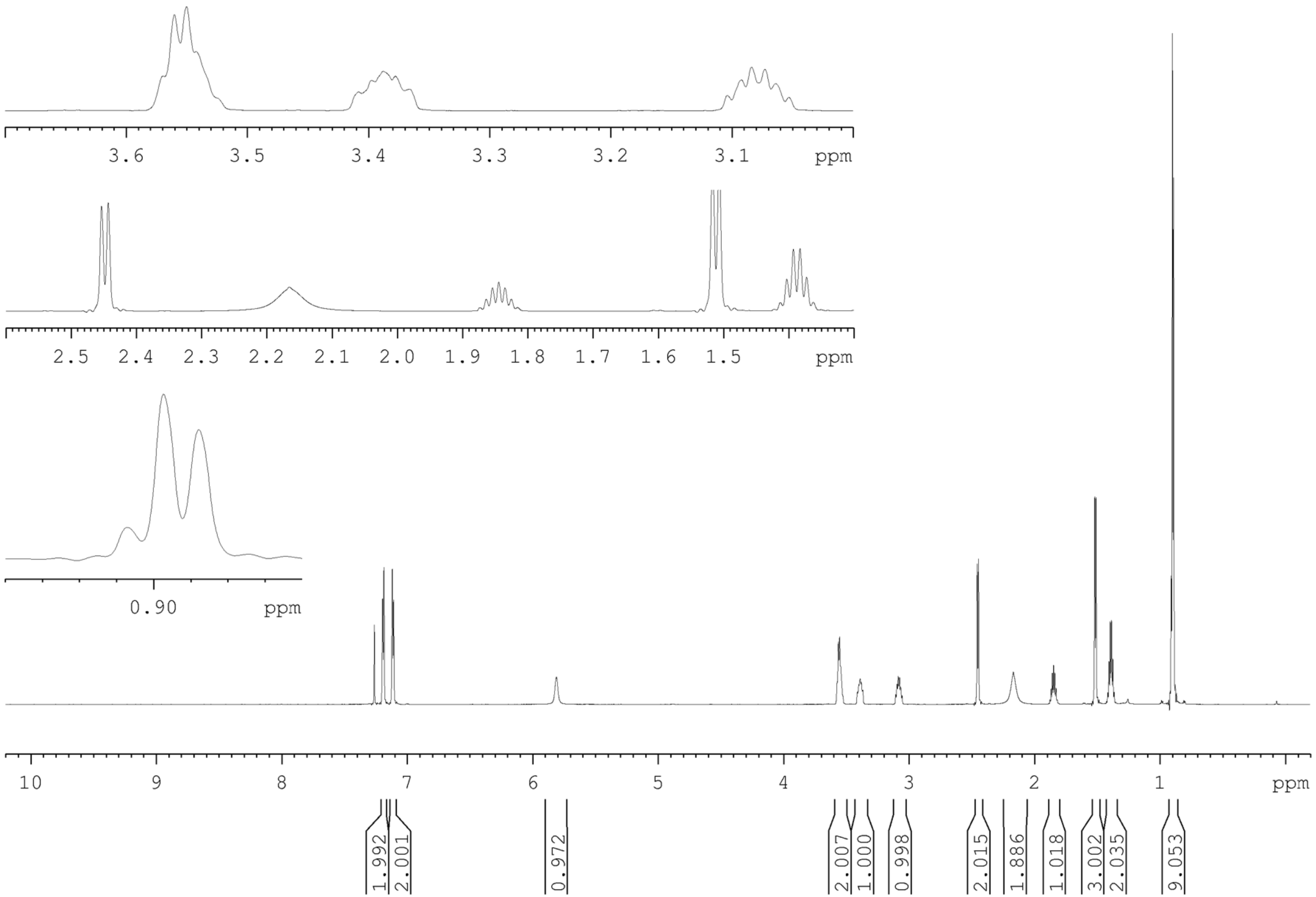

<u>*Diastereomer 1*</u>

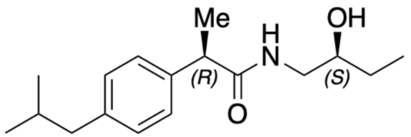

Major enantiomer

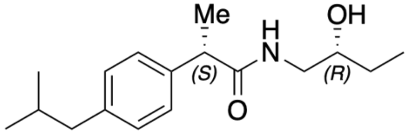

Minor enantiomer

<u>*Diastereomer 2*</u>

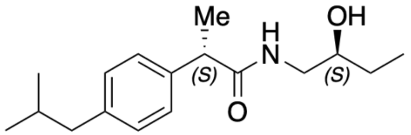

Major enantiomer

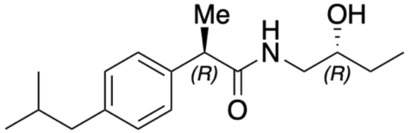

Minor enantiomer

N-((*S*)-2-hydroxybutyl)-2-(4-isobutylphenyl)propanamide (2c)

In $CDCl_3$

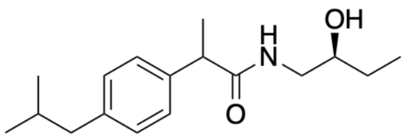

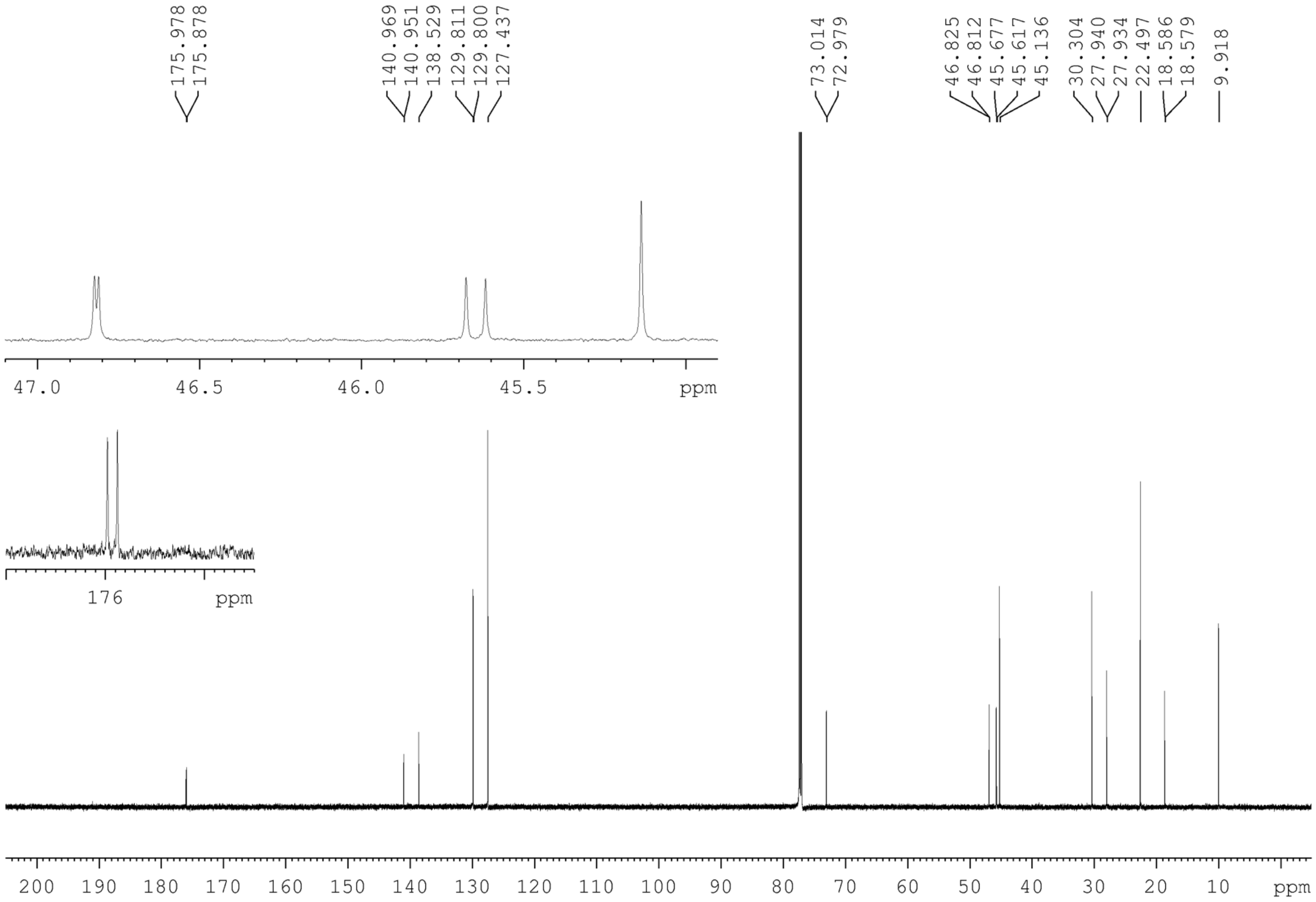

<u>*Diastereomer 1*</u>

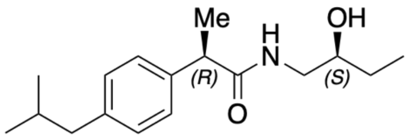

Major enantiomer

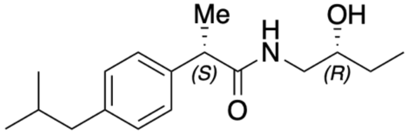

Minor enantiomer

<u>*Diastereomer 2*</u>

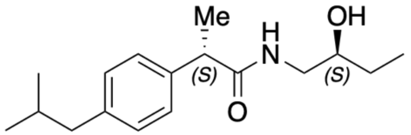

Major enantiomer

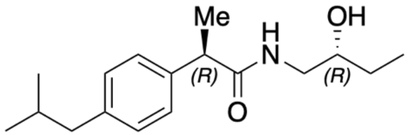

Minor enantiomer

N-((*S*)-2-hydroxybutyl)-2-(4-isobutylphenyl)propanamide (2c)

In $(CD_3)_2SO$, RT

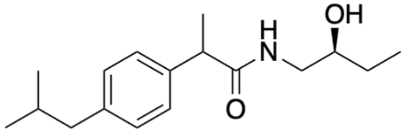

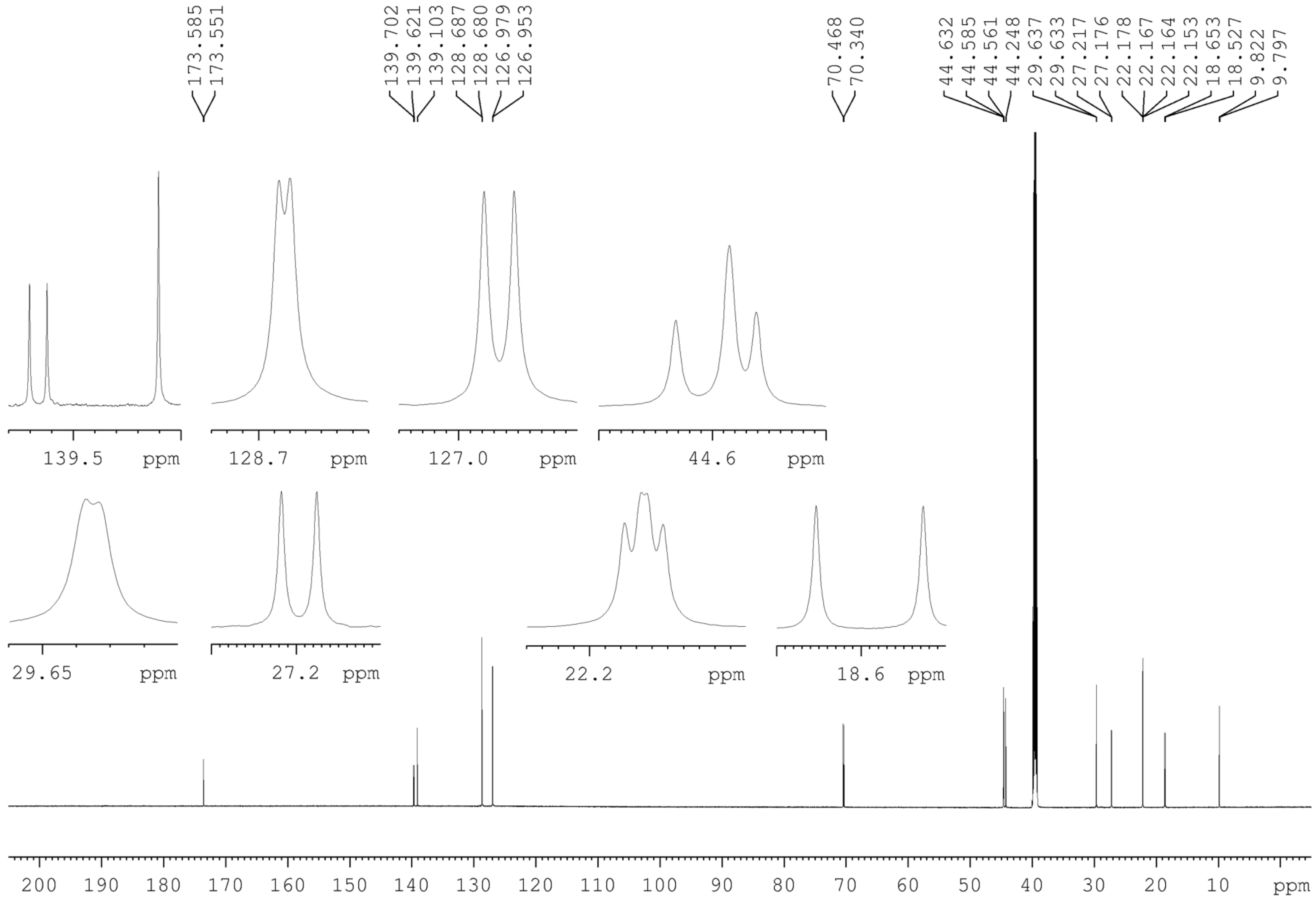

<u>*Diastereomer 1*</u>

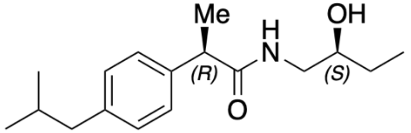

Major enantiomer

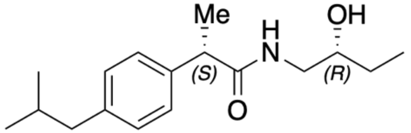

Minor enantiomer

<u>*Diastereomer 2*</u>

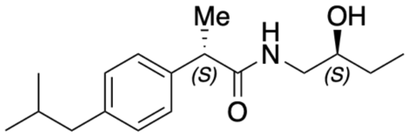

Major enantiomer

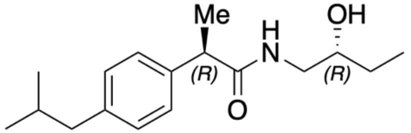

Minor enantiomer

N-((*S*)-2-hydroxybutyl)-2-(4-isobutylphenyl)propanamide (2c)

In $(CD_3)_2SO$, RT

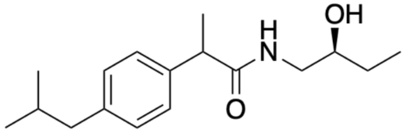

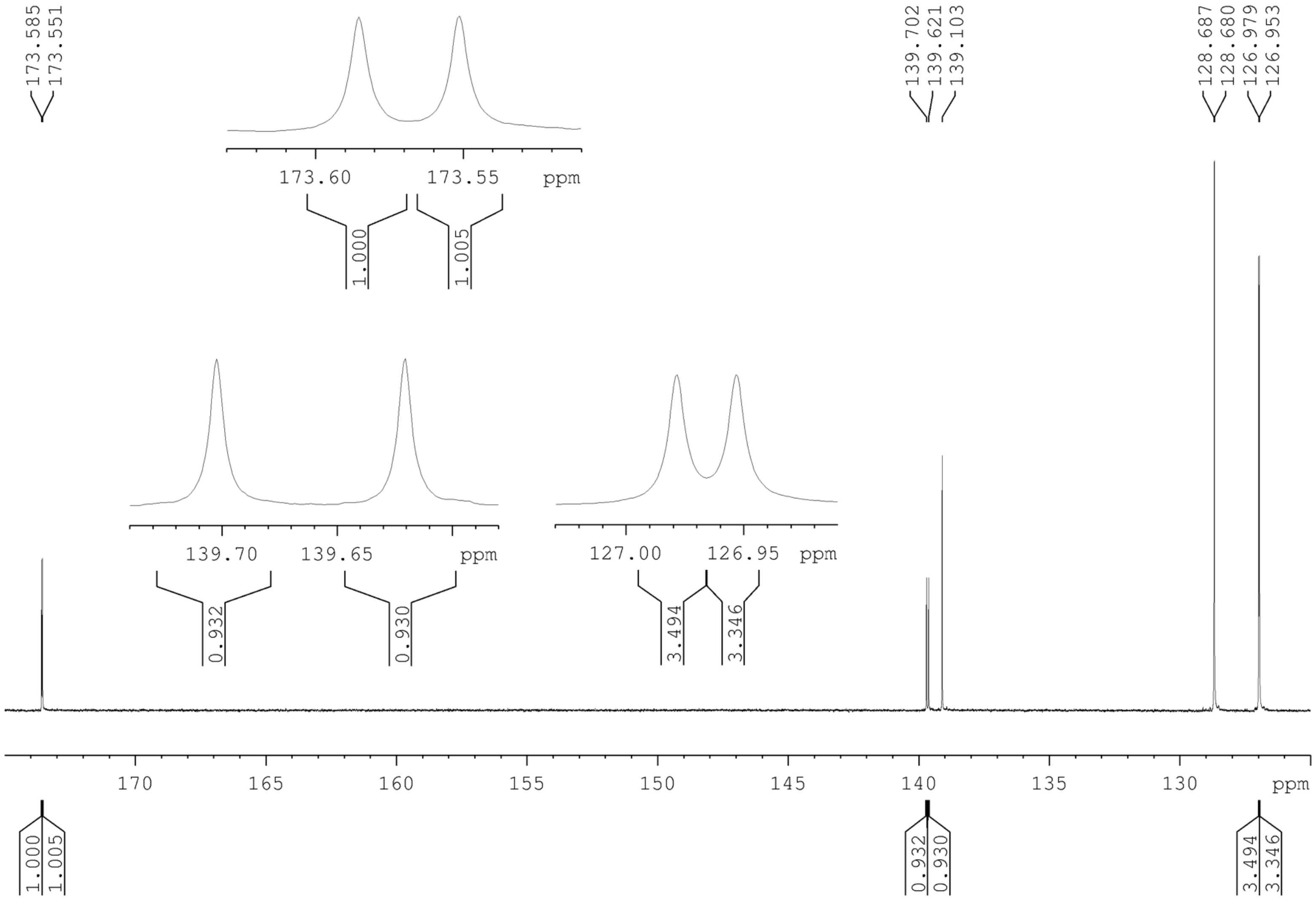

<u>*Diastereomer 1*</u>

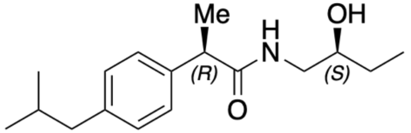

Major enantiomer

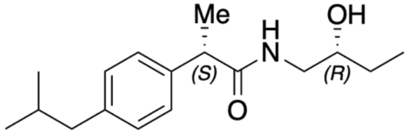

Minor enantiomer

<u>*Diastereomer 2*</u>

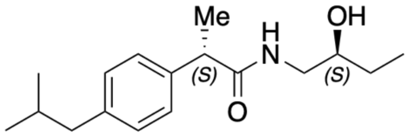

Major enantiomer

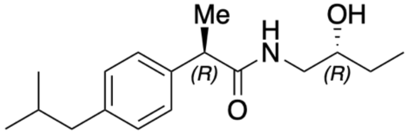

Minor enantiomer

N-(3-hydroxypentyl)acetamide (6-rac)

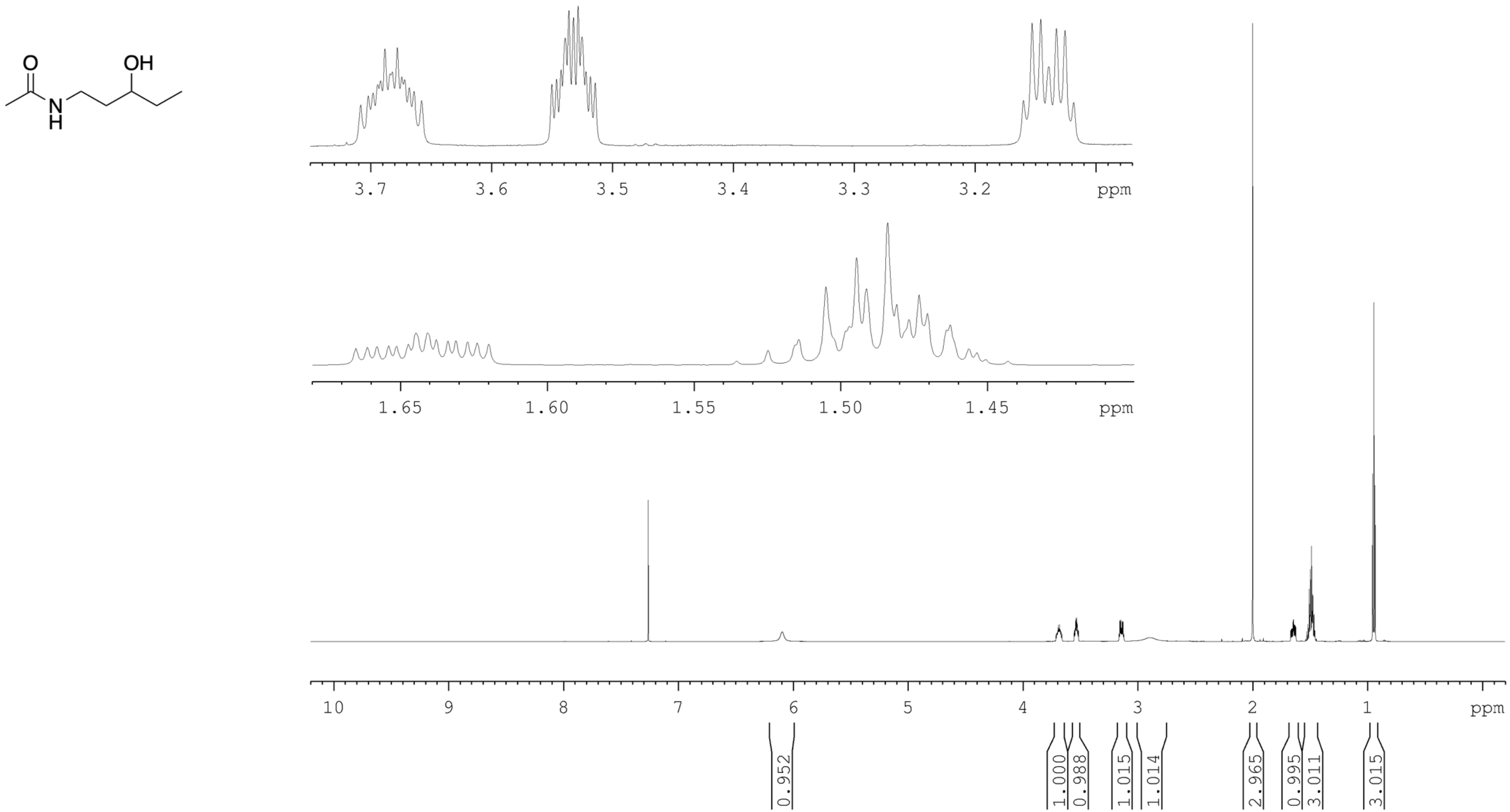

N-(3-hydroxypentyl)acetamide (6-rac)

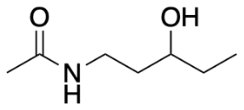

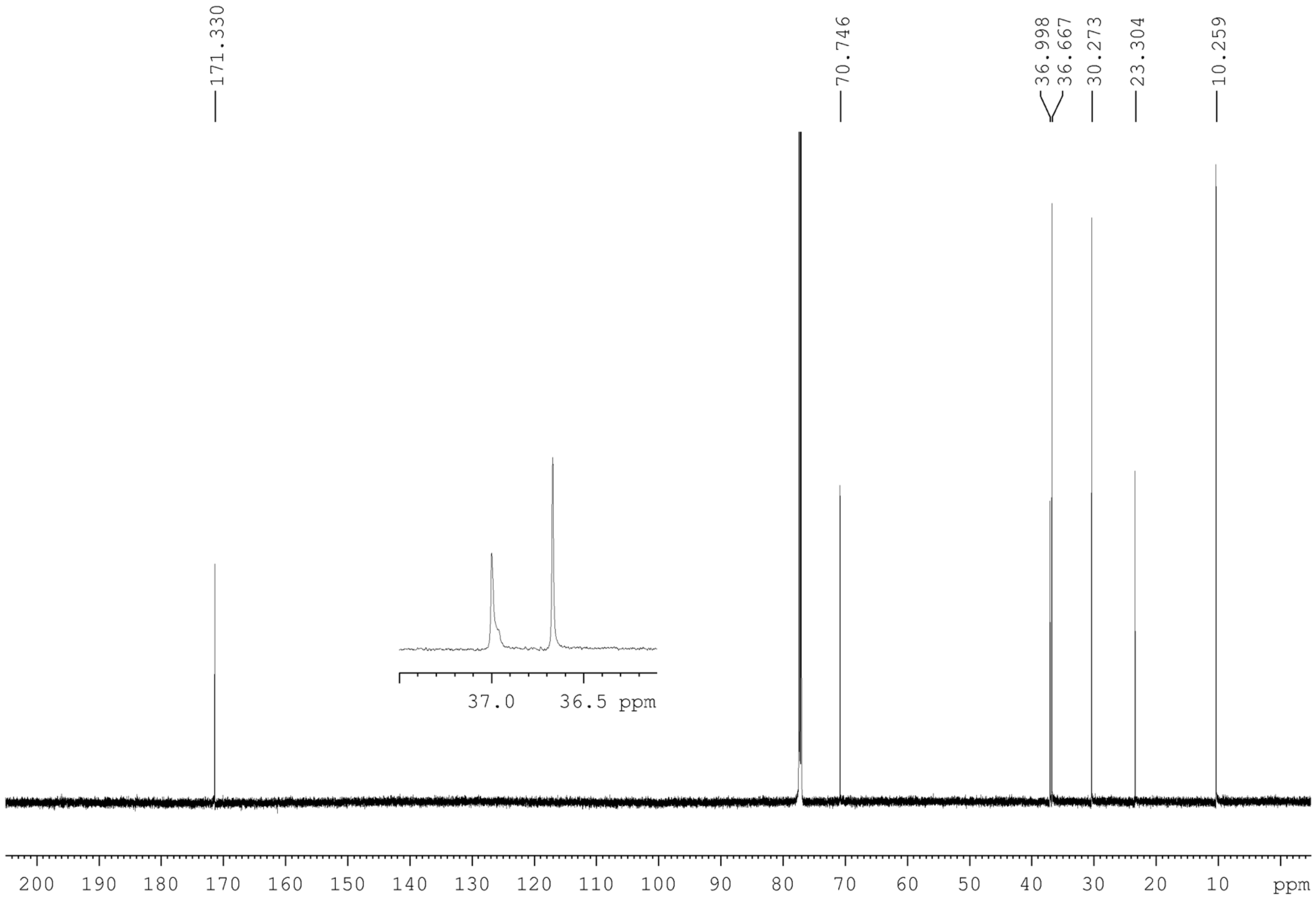

(*S*)-*N*-(3-hydroxypentyl)acetamide (6)

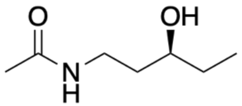

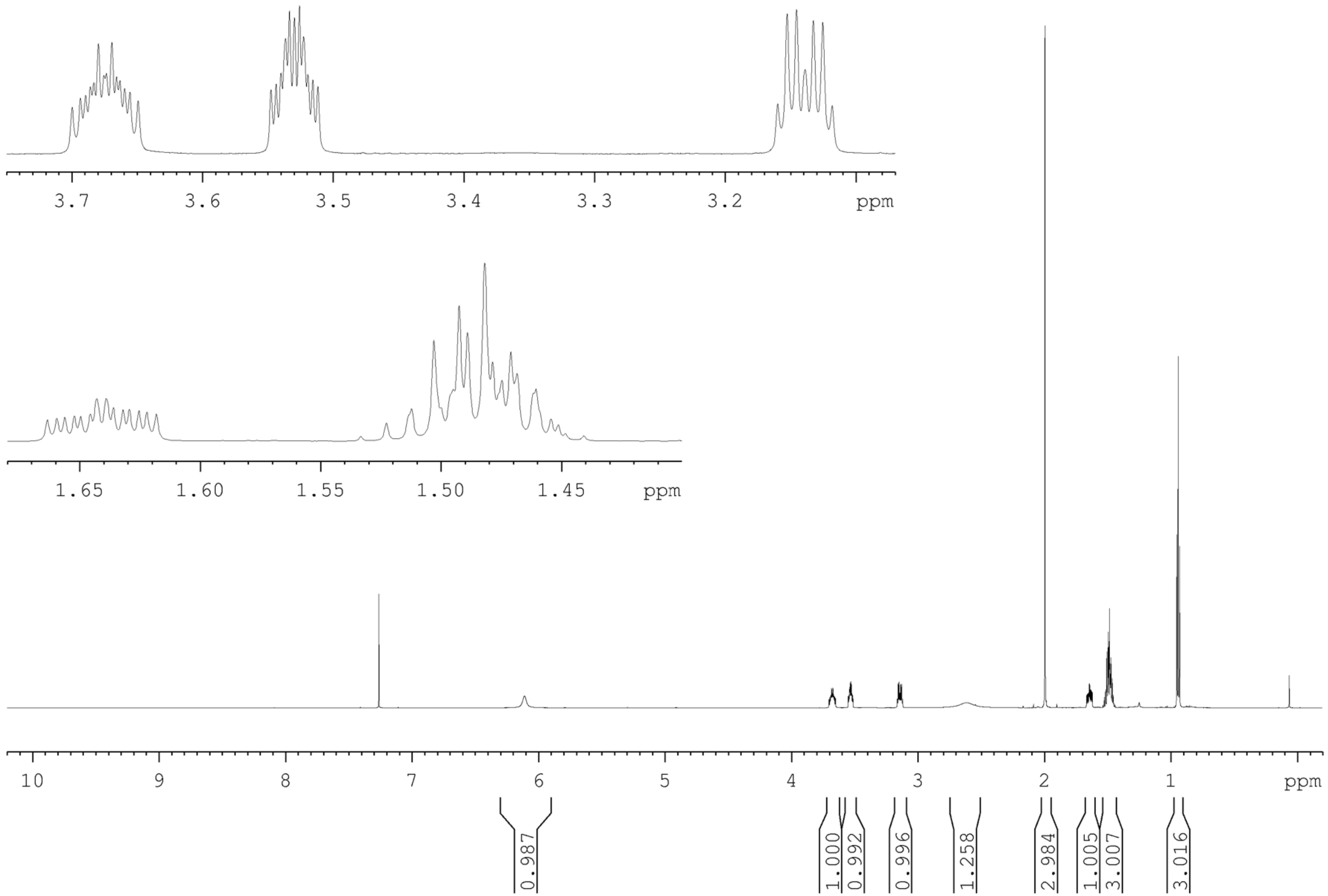

(*S*)-*N*-(3-hydroxypentyl)acetamide (6)

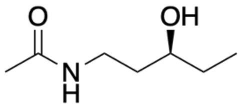

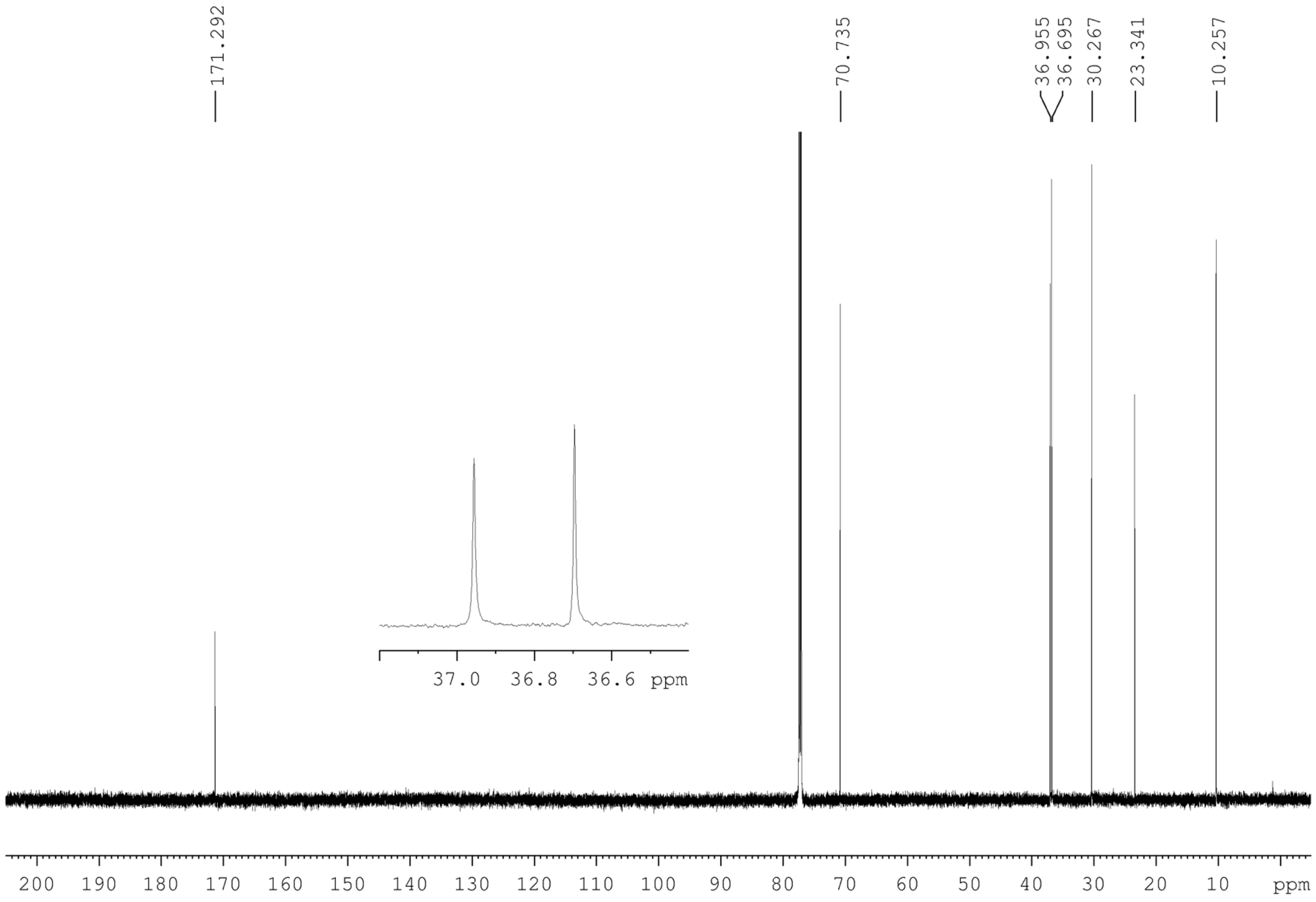

(*S*)-1-(*N*-acetylbenzamido)pentan-3-yl benzoate (6-Bz)

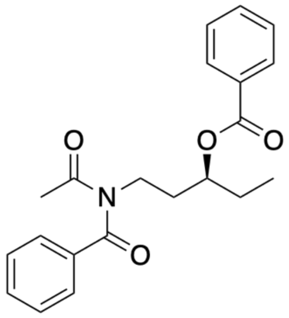

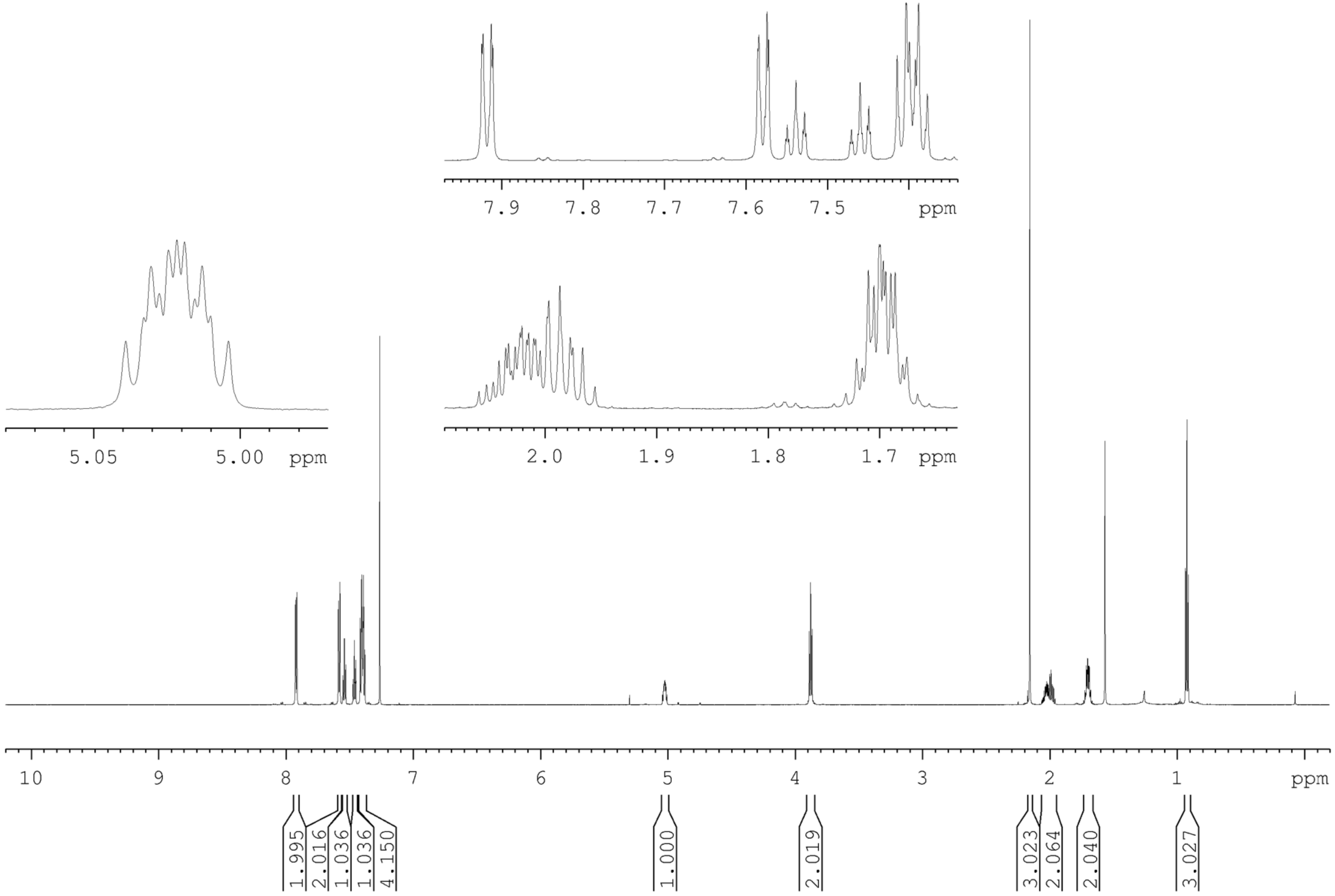

(*S*)-1-(*N*-acetylbenzamido)pentan-3-yl benzoate (6-Bz)

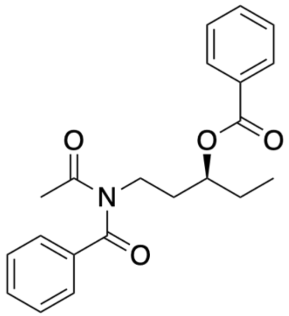

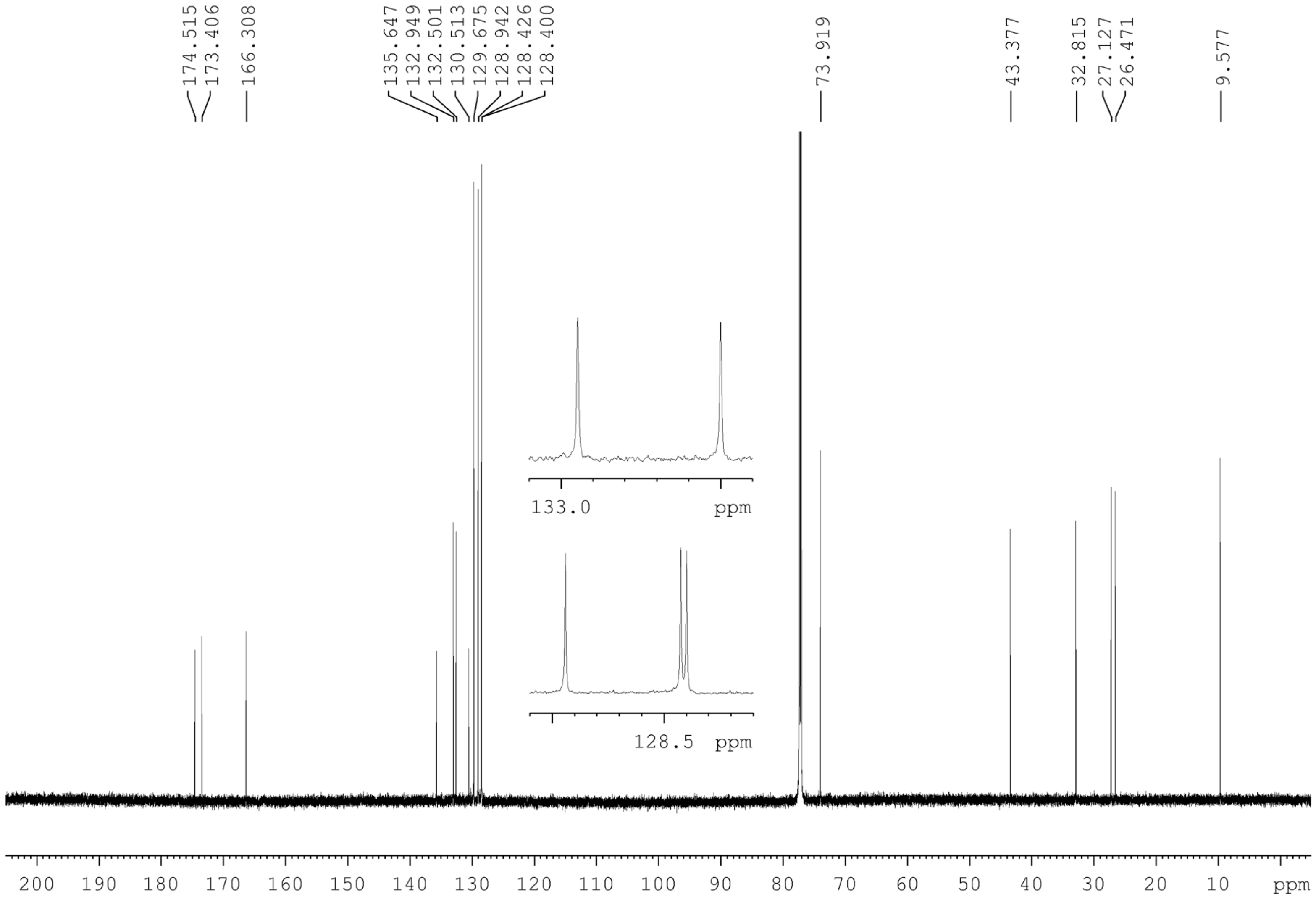

Supplement: Supplementary file 1 [file ja5c20160_si_001.pdf]
